# Multi‐omics analyses reveal spatial heterogeneity in primary and metastatic oesophageal squamous cell carcinoma

**DOI:** 10.1002/ctm2.1493

**Published:** 2023-11-27

**Authors:** Haitao Huang, Na Li, Yingkuan Liang, Rutao Li, Xing Tong, Jinyuan Xiao, Hongzhen Tang, Dong Jiang, Kai Xie, Chen Fang, Shaomu Chen, Guangbin Li, Bin Wang, Jiaqian Wang, Haitao Luo, Lingchuan Guo, Haitao Ma, Wei Jiang, Yu Feng

**Affiliations:** ^1^ Department of Thoracic Surgery the First Affiliated Hospital of Soochow University Suzhou China; ^2^ Institute of Thoracic Surgery the First Affiliated Hospital of Soochow University Suzhou China; ^3^ Shenzhen Engineering Center for Translational Medicine of Precision Cancer Immunodiagnosis and Therapy YuceBio Technology Co., Ltd Shenzhen China; ^4^ Department of Thoracic Surgery Nanjing Medical University Affiliated Cancer Hospital Nanjing China; ^5^ Department of Thoracic Surgery Dushu Lake Hospital Affiliated to Soochow University Suzhou China; ^6^ Department of Pathology the First Affiliated Hospital of Soochow University Suzhou Jiangsu China

**Keywords:** digital spatial profiling, genomic, immunological, oesophageal squamous cell carcinoma, proteomic, spatial intra‐tumoral heterogeneity, transcriptomic

## Abstract

**Background:**

Biopsies obtained from primary oesophageal squamous cell carcinoma (ESCC) guide diagnosis and treatment. However, spatial intra‐tumoral heterogeneity (ITH) influences biopsy‐derived information and patient responsiveness to therapy. Here, we aimed to elucidate the spatial ITH of ESCC and matched lymph node metastasis (LN_met_).

**Methods:**

Primary tumour superficial (PT_sup_), deep (PT_deep_) and LN_met_ subregions of patients with locally advanced resectable ESCC were evaluated using whole‐exome sequencing (WES), whole‐transcriptome sequencing and spatially resolved digital spatial profiling (DSP). To validate the findings, immunohistochemistry was conducted and a single‐cell transcriptomic dataset was analysed.

**Results:**

WES revealed 15.72%, 5.02% and 32.00% unique mutations in PT_sup_, PT_deep_ and LN_met_, respectively. Copy number alterations and phylogenetic trees showed spatial ITH among subregions both within and among patients. Driver mutations had a mixed intra‐tumoral clonal status among subregions. Transcriptome data showed distinct differentially expressed genes among subregions. LN_met_ exhibited elevated expression of immunomodulatory genes and enriched immune cells, particularly when compared with PT_sup_ (all *P* < .05). DSP revealed orthogonal support of bulk transcriptome results, with differences in protein and immune cell abundance between subregions in a spatial context. The integrative analysis of multi‐omics data revealed complex heterogeneity in mRNA/protein levels and immune cell abundance within each subregion.

**Conclusions:**

This study comprehensively characterised spatial ITH in ESCC, and the findings highlight the clinical significance of unbiased molecular classification based on multi‐omics data and their potential to improve the understanding and management of ESCC. The current practices for tissue sampling are insufficient for guiding precision medicine for ESCC, and routine profiling of PT_deep_ and/or LN_met_ should be systematically performed to obtain a more comprehensive understanding of ESCC and better inform treatment decisions.

## Graphical abstract

1. Our study comprehensively characterised spatial intra‐tumoral heterogeneity (ITH) in ESCC by multi‐omics analyses of the superficial and deep subregions of the primary tumour and matched lymph node metastasis (LN_met_).
2. Spatial segmentation through digital spatial profiling was used to explore ITH.
3. Differential molecular profiles were observed across the subregions. Routine profiling of deeper subregions of primary tumour and/or LN_met_ should be systematically performed to guide diagnosis, biomarker testing and treatment.

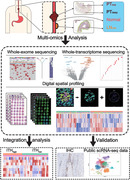

## 1 INTRODUCTION

1

Oesophageal cancer (EC) is one of the most aggressive malignancies with a 5‐year survival rate below 20%.^1^, ^2^ Oesophageal squamous cell carcinoma (ESCC) is the predominant histological subtype of EC in Asian populations^3^ and has a poor outcome largely attributed to tumour metastasis.^4^, ^5^ Lymph node metastasis (LN_met_) is the leading cause of mortality and a major determinant of clinical staging and prognosis in ESCC.^6^ The emergence of targeting specific cancer‐associated driver genes has significantly improved the outcomes for patients with lung, breast and colorectal cancer.^7^, ^8^, ^9^ Nevertheless, advancements in targeted therapy in the context of ESCC have only seen limited strides.^10^ Currently, surgical intervention, chemotherapy and radiotherapy are the primary modalities for ESCC treatment.^11^ Despite the recent approval of VEGFR‐2 antagonists and PD‐1/PD‐L1 blockade therapies for ESCC treatment,^12^, ^13^ responses to these therapies are highly heterogenous. Intra‐tumoral heterogeneity (ITH) is a major obstacle in the development of novel therapeutics and serves as a pivotal determinant of therapeutic responses and patient outcomes.^14^, ^15^, ^16^, ^17^ ITH is characterised by diverse genetic and molecular profiles of tumour cells, which admix with diverse compositions of surrounding immune and stromal cells in the tumour microenvironment (TME).

Clinically, cancer diagnosis and treatment choices typically rely on the evaluation of biopsies obtained from the primary tumour superficial (PT_sup_) subregion. However, samples from primary tumour deep subregion (PT_deep_) or metastatic sites, such as regional LN_met_ or distant metastasis, are often challenging to access and are therefore not typically investigated at the time of diagnosis. Thus, to determine the appropriate sampling depth and make precise treatment choices in ESCC with LN_met_, the spatial heterogeneity of primary tumours in the superficial and deep subregions and matched LN_met_ must be explored.

Currently, several institutions have conducted large‐scale whole‐exome sequencing (WES) on ESCC, thereby unveiling novel actionable targets and genomic ITH.^18^, ^19^, ^20^ However, spatial genomic ITH has not been explored in PT_sup_, PT_deep_ and matched LN_met_ subregions. Furthermore, spatial heterogeneity at the RNA/protein level within these regions remains unexplored. Additionally, the integration of molecular alterations across multi‐omics within the same samples is urgently needed. Consequently, comprehensive molecular characterisation of PT_sup_, PT_deep_ and matched LN_met_ subregions in ESCC is crucial for precise diagnosis, optimisation of treatment strategies and prediction of prognosis.

Spatial heterogeneity of the TME, which remains unknown in the distinct subregions of ESCC, poses a considerable challenge for cancer immunotherapy and has drawn research attention in various cancers.^21^, ^22^, ^23^, ^24^ Conventional bulk sequencing has masked intratumor complexity, hindering the complete assessment of ITH. GeoMx digital spatial profiling (DSP) by Nanostring (now commercially available as GeoMx^®^) has been introduced to quantitatively and spatially assess transcript and protein levels within biospecimens, enabling the examination of multiple user‐defined regions of interest to define heterogeneity and TME interactions.^25^, ^26^ Thus, spatial characterisation of the state of immunologically relevant proteins and immune cells in the TME of primary tumours and metastases using DSP analysis may highlight the potential for identifying the mechanisms underlying tumour immune escape and response to immunotherapy and other potential therapeutic targets in ESCC.

In this study, by analysing the molecular profiles of PT_sup_ and PT_deep_, as well as matched LN_met_ subregions in ESCC, using WES, whole‐transcriptome sequencing and DSP, we investigated the spatial heterogeneity at genomic, transcriptomic, proteomic and immunological levels. We further integrated multi‐omics data to better understand the complex heterogeneity of ESCC. Our findings provide a deeper understanding of the spatial ITH in ESCC.

## 2 METHODS

### 2.1 Patient samples

Twenty‐one patients with ESCC with lymph node metastases who underwent complete surgical resection were recruited from the First Affiliated Hospital of Soochow University between June 2020 and August 2021. The eligibility criteria were as follows: (a) no history of preoperative treatment, (b) complete tumour resection via either an Ivor Lewis or McKeown esophagectomy and routine lymph node dissection, (c) postoperative pathology confirmed as ESCC with LN_met_ and (d) no history of other or complicated malignant tumours. All patients underwent pathological staging according to the 8th Edition of tumour‐node‐metastasis staging system by the American Joint Committee on Cancer. PT_sup_ extended to an average depth of 2.5 mm in the mucosa/superficial submucosa, approximately equivalent to the depth achieved using an endoscopic biopsy. PT_deep_ extended from a depth of 2.5 mm to the deepest point of the tumour invasion. PT_sup_, PT_deep_ and LN_met_ were analysed. All participants provided written informed consent to participate in this study.

### 2.2 Whole‐exome sequencing

Genomic DNA was extracted from surgically removed formalin‐fixed paraffin‐embedded (FFPE) tumour tissues and their corresponding adjacent normal tissues using the GeneRead DNA FFPE Tissue Kit (QIAGEN, Germany), following the manufacturer's instructions. DNA quantification was performed using the dsDNA HS Assay Kit (ThermoFisher Scientific, USA). Subsequently, sequencing libraries were constructed using the SureSelect XT Human All Exon V6 Kit (Agilent Technologies, USA), as previously described.^27^ The libraries were subjected to sequencing on the DNBSEQ T7 platform, generating paired‐end reads of 100 bp. The average effective sequencing depths were ≥ 300 × and 150 × for tumours and adjacent normal tissues, respectively. The insert size of DNA fragments was ≥ 120 bp. Tumour purity was computationally determined using two distinct approaches: All‐FIT and FACETs.^28^, ^29^

### 2.3 Alignment of sequencing reads and somatic variant detection

To filter adaptor sequences and remove low‐quality reads, SOAPnuke (v1.5.6) was used. Low‐quality reads were determined as those with an N rate > 10% and/or with > 10% bases with a quality score < 20. The Burrows‐Wheeler Aligner (BWA‐MEM, v0.7.12) was used to align the clean reads to the reference human genome hg19,^30^ followed by the removal of duplicate reads using Sambamba (v0.5.4) to generate BAM files, which were used for downstream analysis. The identification of single nucleotide variants (SNVs) and insertions and deletions (indels) was performed using VarDict (v1.7.0), whereas the annotation was carried out utilising the Ensembl Variant Effect Predictor (v97). SNVs and indels with a variant allelic fraction (VAF) < .02 or a total read coverage < 10 reads for both tumour and germline samples were excluded from the analysis. In addition, the identified mutations were annotated using snpEff (v4.3) with NCBIrefseq. Somatic copy number alterations (CNAs) were assessed using the software package CNVkit (v0.9.2), which is a purely depth‐based method and incorporates a pipeline for detecting CNA. It leverages both on‐ and off‐target sequencing reads and utilises a series of corrections to enhance the accuracy of copy number calling.^31^

### 2.4 Phylogenetic tree construction and putative driver mutation identification

The phylogenetic tree for each patient was constructed using the Wagner parsimony method with PHYLIP, as previously described.^18^ Briefly, phylogenetic trees were generated using the Discrete Character Parsimony, implemented in the PHYLogeny Inference Package (URL) based on the binary tables converted from the VAFs of tumour samples obtained from each patient. An outgroup was artificially introduced as an additional input, which was considered the virtual root of the rooted tree and the starting point of the tree. The branch/trunk lengths were calculated from mutation counts and the final trees were manually constructed. Putative oncogenic driver genes were initially identified using recent large‐scale sequencing data from ESCC^18^, ^19^, ^32^, ^33^, ^34^ and the COSMIC v96 and DriverDBv3 databases. Non‐silent variants within these genes were assessed, and putative driver mutations were identified if they fulfilled either of the following criteria: (a) the exact mutation, identical mutation site or a minimum of three mutations located within 15 bp around the variant were discovered in COSMIC; and (b) the candidate gene was annotated as recessive in COSMIC, the variant was predicted to be detrimental, encompassing stop‐gain, frameshift and splicing mutations and exhibited either an SIFT score < .05^35^ or Polyphen score > .995.^36^

### 2.5 Cancer cell fraction (CCF) analysis and genomic biomarker calculation

PyClone (v0.13.1) was used to detect subclones and infer the CCF of mutations in tumours based on the corrected VAF.^37^ Mutations with a CCF > .8 were categorised as clonal mutations, whereas the others were classified as subclonal mutations. The ITH level for tumour tissue can be evaluated by calculating an ITH index based on genetic alterations. This index is defined as the proportion of subclonal mutations to the total number of mutations (the sum of the clonal and subclonal mutation numbers). To determine the number of clonal (*n_main_*) and subclonal mutations (*n_sub_*) in each tumour sample, *C_main_* (the cluster with the largest CCF comprising more than one mutation) and subclone clusters (*C_sub_*) were identified. Specifically, the ITH index is calculated by dividing the number of mutations in the subclone mutation cluster by the total number of mutations: ITH = *n_sub_* /(*n_main_* + *n_sub_*).^38^ Microsatellite instability (MSI) status was determined using MSIsensor (v0.2), a C++ program for automated detection of somatic microsatellite changes.^39^ The proportion of unstable microsatellite loci was denoted as the MSI score. MSI scores ≥ 20 were categorised as MSI‐High (MSI‐H). Tumour mutational burden (TMB) was determined based on the number of all non‐synonymous mutations, including coding base substitutions and indels, per megabase. A TMB > 10, 2.5 ≤ TMB ≤ 10 and TMB < 2.5 mutations/Mb was defined as TMB‐H, TMB‐M and TMB‐L, respectively. Tumour neoantigen burden (TNB) was evaluated by calculating the number of mutations capable of generating neoantigens per megabase. A TNB > 4.5, .5 ≤ TNB ≤ 4.5 and TNB < .5 neoantigens/Mb was defined as TNB‐H, TNB‐M and TNB‐L, respectively. Human lymphocyte antigen I (HLA‐I) typing of tumours and matched normal samples was performed according to previously described protocols.^40^ Briefly, HLA typing was performed using the patched OptiType software.^41^ Subsequently, the loss of heterozygosity in HLA (LOHHLA) algorithm was employed to detect HLA loss of heterozygosity (HLA LOH) in tumour samples.^42^ LOH was classified when two conditions were met: (1) a copy number < .5 and (2) allelic imbalance with a *P*‐value < .01 determined using the paired Student's t‐test between the two distributions. The LOH status was further categorised into two grades: non‐LOH (no LOH detected in any HLA alleles) and LOH (LOH detected in at least one HLA allele). Fisher's exact test was performed to determine whether the classes of HLA LOH results have a non‐random association.

### 2.6 Mutational signature analysis

The R package deconstructSigs (v1.6.0), which uses an iterative approach to calculate the combination of COSMIC signatures that best approximate a tumour's mutational spectrum,^43^ was utilised to explore the mutational signatures based on the trinucleotide contexts of SNVs. The 96 single‐base substitution (SBS) mutational signatures were downloaded from COSMIC. For each individual sample, the dominant signature was determined as the one exhibiting the highest proportion of contribution.

### 2.7 Whole‐transcriptome sequencing

Total RNA was extracted from the surgically resected FFPE tumour samples using the QIAGEN AllPrep DNA/RNA FFPE isolation kit. The concentration of RNA was determined using the Qubit™ RNA HS Assay Kit (ThermoFisher Scientific, USA). RNA purity and integrity were analysed using a Take3 microvolume plate (BioTek, USA) and the RNA Cartridge kit of the Qseq100 Bio‐Fragment Analyzer (Bioptic, China), respectively. RNA‐seq libraries were prepared using the NadPrep^®^ DNA Universal Library Preparation Kit (for MGI) (Nanodigmbio, China). Libraries were subjected to sequencing on the DNBSEQ T7 platform, yielding sequencing data comprising 100 bp paired‐end reads in the FASTQ format. Raw sequencing data underwent processing to eliminate low‐quality reads and adapters using Trim Galore (v0.6.7). Clean reads were acquired from each sample and utilised in the subsequent analysis. Clean reads were aligned to the hg19 reference using the STAR software (v2.7.8a) and annotated according to the Gencode (v37) database. Based on the aligned reads, raw counts and transcripts per million values were computed in Rsem (v1.3.0).

### 2.8 RNA‐sequencing data analysis

The read count matrix was employed for the identification of differentially expressed genes (DEGs) using the DESeq2 package (v1.32.1). *DEGs with a false discovery rate (FDR) < .05, and an absolute value of log2 (fold change) > 1* were considered significantly DEGs. To generate volcano and heatmap plots, the ggpubr and Complexheatmap R packages were used, respectively. Enrichment analysis of Kyoto Encyclopedia of Genes and Genomes (KEGG) pathways was conducted using the KOBAS‐i web tool, with a significance threshold of an adjusted *P‐*value corrected using the Benjamini & Hochberg method, set at < .05. xCell was utilised to infer the abundance of 64 immune and stromal cell types.^44^ In addition, quanTIseq methods were adopted to further validate the enrichment level of 10 major infiltrating lymphocytes, including B cells, M1 and M2 macrophages, monocytes and neutrophils, as well as natural killer (NK), CD4+ T, CD8+ T, regulatory T (Tregs) and dendritic cells (DCs) in tumour samples.^45^

### 2.9 Single‐cell RNA‐sequencing (scRNA‐seq) analysis

We downloaded the publicly available scRNA‐seq ESCC dataset of Jiang et al.^6^ from the article's Supporting Information. This dataset consists of four ESCC primary tumour samples and three metastatic lymph nodes. We classified the primary tumour tissues therein as the PT group and the metastatic lymph nodes as the LN_met_ group. Subsequently, we conducted a statistical analysis to compare the difference in the abundance and proportions of six major cell types, namely endothelial, fibroblast, B, epithelial, T and DCs, between the two groups. Additionally, the abundance and proportions of various B cell subsets were analysed. The statistical method used was the independent Student's t‐test.

### 2.10 Tissue microarray (TMA) construction and DSP

The DSP (NanoString) study design comprised specimens (PT_sup_, PT_deep_ and matched LN_met_) collected from 21 patients at the time of resection. The FFPE tissues were stored as tumour blocks. TMAs were constructed from three 2‐mm‐diameter cores punched from each FFPE tissue block. DSP analysis of the NanoString Human Immune Cell Profiling Panel, including immune checkpoints, proteins that mark distinct immune cell types (T cells, B cells, macrophages, DCs and NK cells), and other immune modulatory proteins, plus three positive controls and three negative isotype controls was performed, as previously described.^46^ Briefly, FFPE TMA sections were incubated with a cocktail of barcode‐conjugated antibodies and then stained with the following immunofluorescent antibodies to facilitate the identification of tissue morphology: PanCK (NBP2‐33200, Novus Biologicals; green) for epithelial cells, CD45 (NBP2‐34528, Novus Biologicals; red) for T cells and Syto13 (S7575, Invitrogen; blue) for DNA stain. Stained slides were loaded onto the GeoMx instrument and scanned for the region of interest (ROI) selection based on the immunofluorescence images. One or two ROIs were selected per tissue core. Compartment‐specific areas of interest (AOIs) were assigned from the sequential masks as stroma‐enriched (CD45‐positive staining and stroma‐enriched segment) and tumour‐enriched (PanCK‐positive staining and tumour‐enriched segment) compartments. Ultraviolet radiation was applied to release the oligonucleotide barcodes that were then collected and deposited into designated wells on a microtiter plate, enabling well indexing of each AOI and quantitation using the nCounter instrumentation (NanoString Technologies).

### 2.11 DSP data processing and analysis

The raw counts from barcodes corresponding to antibody targets underwent External RNA Control Consortium normalisation to address technical variation (based on the geometric mean of the three positive control markers: S6, histone H3 and GAPDH). The quality control (QC) section includes four parameters: field of view (FOV) detection percentage, binding density, nuclei count and surface area. FOV detection was employed to eliminate AOIs when the FOV detection percentage was ≤ 75%, and AOIs with a binding density ranging from .1 to 2.25 were retained. To meet the QC standards, an AOI must have a nucleus count of >20, or surface area of >1600 μm^2^. The raw digital counts corresponding to specific probes were normalised. To avoid variations, AOIs were adjusted by area normalisation and cell numbers. Data that met the QC criteria underwent sample‐wise normalisation based on the background of three immunoglobulin G antibodies (Ms IgG1, Ms IgG2a and Rb IgG). The normalised counts were utilised to assess disparities between groups. Heatmaps were drawn using the Complex Heatmap package (v2.8.0) of R (v4.1.0). The Benjamini–Hochberg method was employed for multiple comparison testing to control the FDR. Proteins with an FDR ≤ .05 and |FC| > 1.5 were reported as significantly differentially expressed. To generate the simulated bulk sequencing data for the respective proteins in each sample, the following equation was employed to calculate the protein expression levels of all stromal, and tumour AOIs within each sample: average value = (*X*1+*X*2…+*Xn*)/*N* (where *N* represents the total number of corresponding AOIs in each sample). The average value obtained represents the simulated bulk sequencing expression value of the specific protein within the target region. Uniform manifold approximation and projection (UMAP) (v0.2.8.0) was performed for dimension reduction analysis. The abundance of immune and stromal cells in the TME was calculated using the following equation: (log2 (*X_1_*)+log2 (*X_i_*)+…+log2 (*X_n_*))/*n*, where *Xi* represents the normalised expression value of each protein marker, and *n* denotes the number of protein markers considered for the particular cell type. Wilcoxon's rank sum test was conducted to assess the differences between groups, with statistical significance set at *P* < .05.

### 2.12 Immunohistochemistry

FFPE tissue sections of 4 μm thickness underwent deparaffinisation and rehydration. Subsequently, heat‐mediated antigen retrieval was performed using Tris‐EDTA. To prevent non‐specific binding, slides were blocked using PBS blocking buffer for 30 min at room temperature. The following primary antibodies were used: anti‐PD‐L1 (22C3, 1:50 dilution, DAKO, Denmark), anti‐CD20 (GA604, 1:100 dilution, DAKO, Denmark), anti‐CD8 (SP16, 1:200 dilution, ZSGB‐BIO, China) and anti‐CD11C (EP1347Y, 1:500 dilution, Abcam, USA). Antibodies were diluted in PBS buffer. The tissue sections were incubated with the primary antibody overnight at 4°C. Next, slides underwent incubation with poly‐HRP (Cat#21140, Thermo Scientific, USA) for 1 h followed by development with DAB chromogen (Cat#K3468, DAKO, 1:50 dilution, Denmark). Haematoxylins were used for counterstaining. Slides were dehydrated, mounted with Micromount (Cat#3801731, Leica, Germany) and evaluated by two pathologists. The proportion of positive cells was analysed using Image‐Pro Plus. Student's t‐test was performed to compare differences between two groups.

### 2.13 Statistical analyses

All statistical tests conducted were two‐sided, and statistical significance was set at *P* < .05. The Student's t‐test and Wilcoxon's rank sum tests were used to compare differences between two groups.

## 3 RESULTS

### 3.1 Overview of included patients with ESCC and samples

**FIGURE 1:**
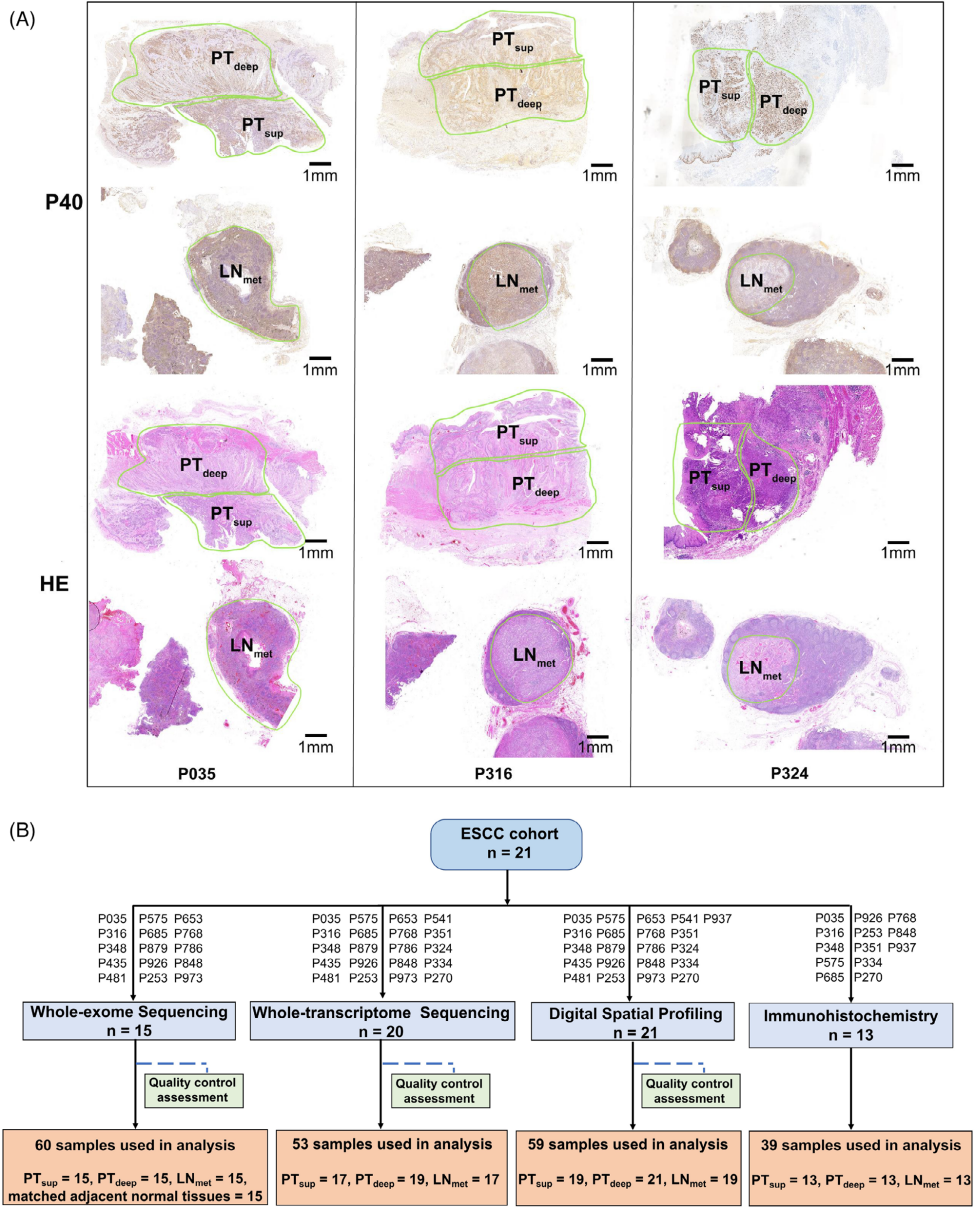
Pathological selection of tumour subregions and schematic representation of methods and samples used in this study. (A) Blocks with the highest tumour content per area were selected from the resection specimen to perform P40 IHC and HE staining. Subregions were manually delineated in the primary tumour by pathologists. Pathological selection of tumour subregions from three representative ESCC patients was shown. (B) Overview of the methods used, and flowchart of patients and samples included. The final inclusion of samples used for analysis in each method is depicted in sandy brown. ESCC, oesophageal squamous cell carcinoma HE, haematoxylin‐eosin; IHC, immunohistochemistry; LN_met_, lymph node metastasis; PT_sup_, primary tumour superficial; PT_deep_, primary tumour deep.

The clinicopathological characteristics of the 21 ESCC patients with lymph node metastases are summarised in Table 1 and detailed in Table S1. The median age was 68 years (range: 41−80), and 76% were male. Each patient had three tumour subregions analysed, including PT_sup_, PT_deep_ and LN_met_. Representative stained immunohistochemistry (IHC) and haematoxylin‐eosin sections from the three subregions were reviewed and manually delineated (Figure 1A). Samples from 15 and 20 randomly selected patients were subjected to WES and whole‐transcriptome sequencing, respectively. Moreover, samples from all 21 patients were processed using DSP. Based on the rigorous QC of each sample (Table S2), the availability of samples for the different assays varied (Figure 1B and Table S3). Additionally, IHC was conducted on the remaining samples from 13 patients to further validate the findings (Figure 1B).

**TABLE 1:** Clinicopathological features of 21 patients with ESCC.

Characteristics	Number	Frequency (%)
**Gender**		
Male	16	76.2
Female	5	23.8
**Median age at diagnosis (range), years**	68 (41–80)	
**Tumour location**		
Upper thoracic	2	9.5
Middle thoracic	14	66.7
Lower thoracic	5	23.8
**TNM staging**		
T3N1M0	6	28.6
T3N2M0	5	23.8
T3N3M0	1	4.8
T4aN1M0	3	14.2
T4aN2M0	5	23.8
T4aN3M0	1	4.8
**P40 IHC positive**	21	100
**Treatment**		
Complete tumour resection and routine lymph node dissection	21	100
**Follow‐up status**		
Alive	10	47.6
Dead	10	47.6
Lost	1	4.8

ESCC, oesophageal squamous cell carcinoma; IHC, immunohistochemistry; TNM, Tumor‐Node‐Metastasis.

### 3.2 Landscape of spatial genomic heterogeneity in ESCC

**FIGURE 2:**
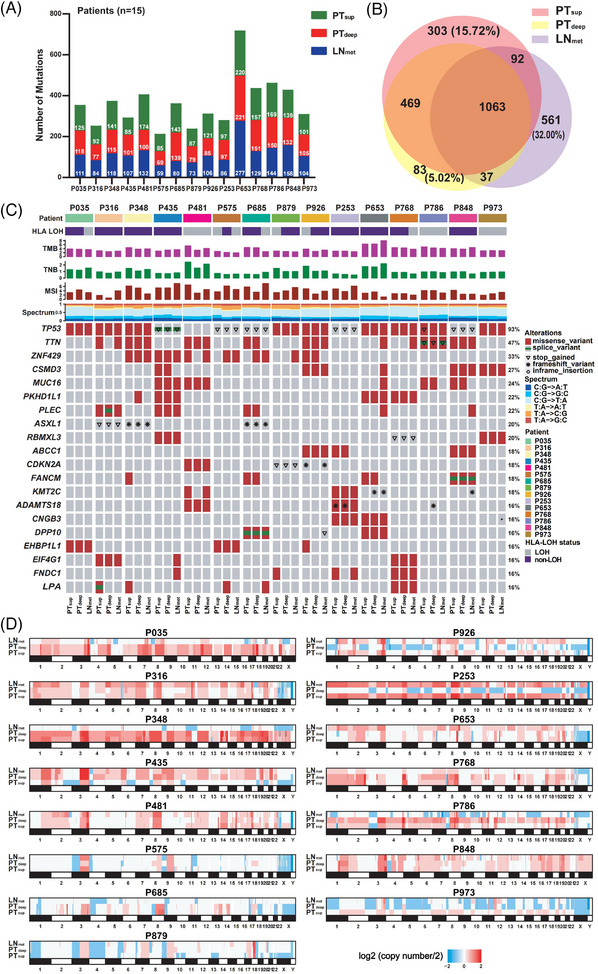
WES analysis of spatial heterogeneity in ESCC. (A) Number of mutations detected using WES across three tumour subregions (PT_sup_, PT_deep_ and LN_met_) in each ESCC individual (*n* = 15). (B) A Venn diagram showing the overlapping and unique mutated genes among the three subregions of ESCC. (C) The mutational landscapes of the three tumour subregions in each ESCC patient. The right bar represents the mutation frequency of the top 20 genes in each subregion of the 15 ESCCs. HLA LOH status, TMB, TNB, MSI scores and mutation spectrum in each subregion are shown in the upper panel. The mutational types were annotated by colours and shapes. (D) The spectrum diagrams of the CNA patterns among the three subregions in each case. The copy number loss and gain are shown in blue and red, respectively. The colour intensity within the scale at the right bottom is corresponding to the distribution of copy numbers. The colour gradient ranges from −2 to 2, indicating the logarithmic measure of log2 (copy number/2). Blue colours correspond to copy numbers lower than 2 (loss), while red colours indicate copy numbers higher than 2 (gain). CNA, copy number alteration; ESCC, oesophageal squamous cell carcinoma; HLA LOH, human leukocyte antigen loss of heterozygosity; MSI, microsatellite instability; TMB, tumour mutational burden; TNB, tumour neoantigen burden; WES, whole‐exome sequencing.

A total of 45 tumour samples (three subregions per sample: PT_sup_, PT_deep_ and LN_met_) and 15 matched adjacent normal tissues from 15 patients with ESCC were collected to perform WES (Figure 1B and Table S3). Tumour purity was highly consistent when estimated computationally using All‐FIT and FACETs algorithms (Table S4). We detected 4280 missense, 335 nonsense and 363 frameshift mutations in the protein‐coding sequences (Table S5). The number of mutations and mutational categories varied among the three subregions within each patient (Figures 2A and S1A). PT_sup_, PT_deep_ and LN_met_ harboured 15.72%, 5.02% and 32.00% unique mutations, respectively (Figure 2B). The detected mutations and mutational categories among the three subregions across patients showed no significant difference (Figures S1B and C). The various variant types in each gene with a frequency above 16% (top 20) in the 45 ESCC samples are displayed in Figure 2C. Among the identified mutated genes, *TP53* was the most frequently mutated gene (93%) within the three subregions, followed by *TTN* (47%) and *ZNF429* (33%).

All subregions harboured generally low MSI scores, and most subregions had moderate TMB and TNB levels (Figure 2C and Table S6). No significant differences were observed in MSI, TMB and TNB among the three subregions (Figures S1D–F). The genomic ITH level for the three subregions per patient was subsequently calculated, which varied among different patients and subregions (Table S6). PT_sup_ had a higher ITH value than PT_deep_ and LN_met_, though insignificant (Figure S1G). HLA LOH may facilitate immune evasion.^42^ HLA LOH was detected in 80% (12/15) of patients (Figure S1H). Although the HLA statuses among PT_sup_, PT_deep_ and LN_met_ showed no significant difference (Figure S1I), the presence of HLA LOH was inconsistent among the three subregions in 7 of 15 cases (Figure S1H).

Next, we investigated the ITH of CNAs within the three subregions (Figure 2D and Table S7). In the majority of cases, PT_sup_ and PT_deep_ from the same patient shared most of the CNAs, yet a few copy number events exhibited distinguishable variations between them. Notably, large‐scale diversity in CNAs was observed between PT_sup_ and PT_deep_ in five patients (P435, P879, P926, P253 and P973). Additionally, the pattern of CNAs observed in LN_met_ differed markedly from that observed in both PT_sup_ and PT_deep_ subregions in most cases, suggesting that the primary tumour and LN_met_ may originate from distinct tumour cell subpopulations, which exhibit differences in CNAs in their genomes. The CNA patterns displayed distinct characteristics across different patients. Thus, it is evident that spatial heterogeneity in terms of CNAs exists not only among LN_met_ and the subregions of the primary tumour within patients but also among patients.

**FIGURE 3:**
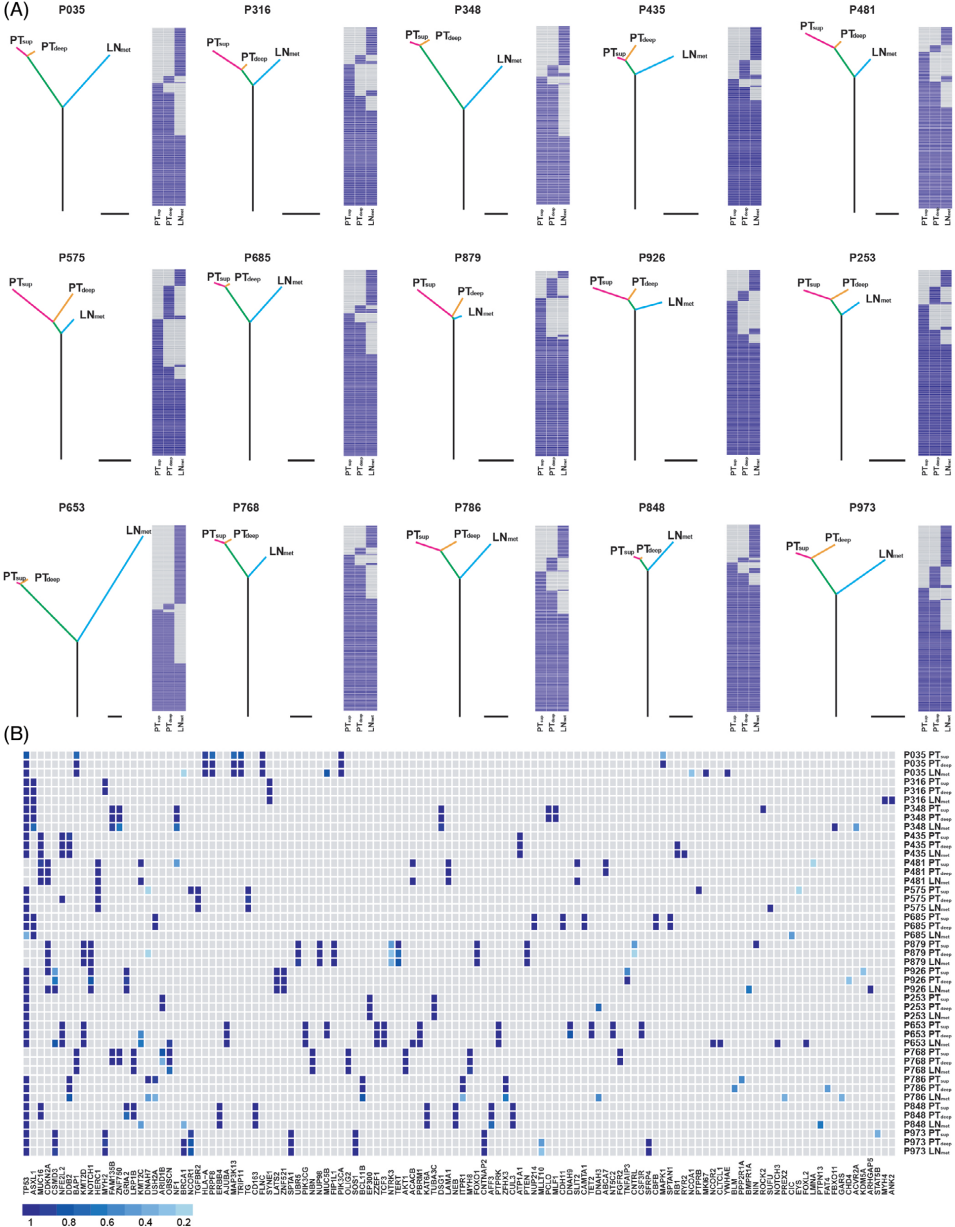
Spatial ITH of somatic and driver mutations in ESCC tumours. (A) Phylogenetic trees were constructed from all somatic mutations by the Wagner parsimony method using PHYLIP. Lengths of trunks and branches are proportional to the number of mutations acquired. The trunk, shared branch and private branch of the tree represented mutations in all tumour regions, some but not all regions, and only one region, respectively. The trunk and shared branch were indicated as black and green, respectively. Heatmaps showed the presence (blue) or absence (grey) of a somatic mutation in each tumour region. Scale bar: 25 mutations. (B) The heatmap displays the clonal status of putative driver mutations in each subregion of the ESCC tumours. ITH, intra‐tumoral heterogeneity.

To explore the genomic evolution among PT_sup_, PT_deep_ and LN_met_, phylogenetic trees were constructed using somatic mutations (both silent and non‐silent) detected in individual tumour regions. The phylogenetic trees displayed divergence across different cases, and various extents of private mutations were observed for each subregion (Figure 3A), suggesting spatial ITH in ESCC. Based on recent large‐scale sequencing data of ESCC^18^, ^19^, ^32^, ^33^, ^34^ and COSMIC v96 and DriverDBv3 databases, the potential driver mutations that occur relatively early in the tumour's evolutionary process were identified. The driver mutations exhibited a significant enrichment within the trunks compared with those of other mutations (58.56% vs. 51.58%, *P* = .00928) (Figure S2A and Table S8), which is in accordance with a previous finding.^18^ To investigate the clonal status of driver mutations within distinct regions, the CCF in each tumour subregion was assessed. Notably, certain driver variants identified as clonal in certain tumour regions within an individual were either absent or present as subclonal in other regions of the same individual (Figure 3B). For example, the PT_sup_ subregion of P035 harboured *MAPK1* as a subclonal mutation yet was clonal in PT_deep_. Mutations in *MKI67* and *YWHAE* were undetectable in primary tumour regions in P035 but were clonally dominant in LN_met_. Likewise, *TP53* in P685 and *ASXL1, NF1* and *ZNF750* in P348 were mutated in a clonal manner in the primary tumour but in a subclonal manner in LN_met_. Collectively, our findings indicate that driver mutations in ESCC can exhibit diverse and intricate clonal statuses within the tumour.

The mutations in an individual cancer genome may have been generated by external and endogenous mutational processes.^47^, ^48^ Thus, we assessed the contribution of the previously described SBS mutational signatures (COSMIC v2) in trunks and branches. The overall mutational spectra exhibited remarkable similarity between trunk and branch mutations, with robust enrichment of SBS1 (related to aging) and subtle but enriched APOBEC‐associated SBS13 (C > T and C > G in the TpCpW context) (Figure S2B), consistent with previous results for ESCC.^18^ SBS1 (age), SBS2 and SBS13 (APOBEC), SBS6 (DNA mismatch repair) and SBS10 (polymerase epsilon exonuclease domain mutations) were significant contributors to both trunk and branch mutations (Figure S2C). The relative contribution of each SBS exhibited variations among patients with ESCC, both in the trunk and branch (Figure S2D). These results suggest that diverse mutational processes potentially contribute significantly to the subclonal diversification during the progression of ESCC, as reported in previous studies.^18^, ^19^

### 3.3 Spatial heterogeneity of RNA profiles and TME in ESCC

**FIGURE 4:**
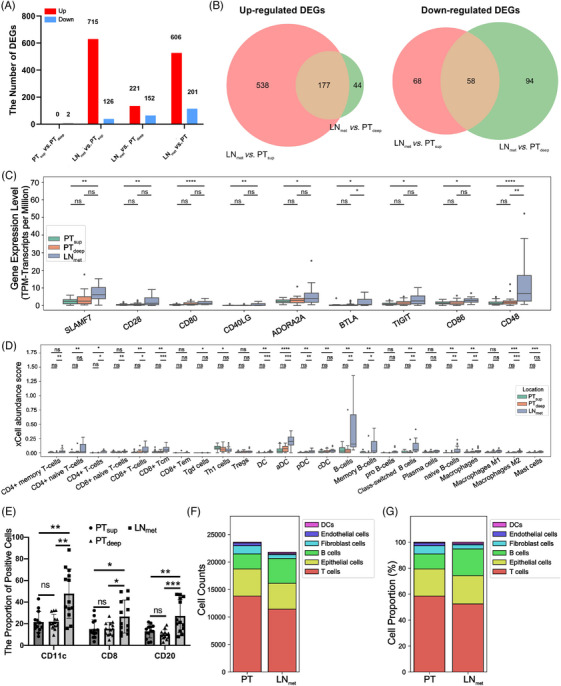
Differential expression of genes and distinct immune‐related cell types among subregions. (A) Number of genes in the whole transcriptome with significant differences in analyses of tumour subregions. Expression of genes with an FDR value < .05, and absolute value of log_2_ (fold change) > 1 was considered significant difference. (B) Venn diagrams showing the distinct and overlapping DEGs between LN_met_ versus PT_sup_ and LN_met_ versus PT_deep_. (C) Comparisons of the expression levels of immunomodulatory genes in the three subregions. These comparisons were performed as part of the RNAseq analysis pipeline using DeSeq2. Adjusted *P* value (corrected *P* value for multiple hypothesis testing) was adopted. For the boxplot, the centerline represents the median, and box limits represent the upper and lower quartiles. Each dot represents a sample. (D) Boxplots showing the abundance of 24 immune cells in the three distinct subregions estimated using xCell. The Student's *t*‐test was used to compare differences. For the boxplot, the centerline represents the median, and box limits represent the upper and lower quartiles. Each dot represents a sample. (E) The proportion of CD11c, CD8 and CD20 staining positive cells. The Student's *t*‐test was used to compare differences. (F–G) Stacked bar charts displaying cell abundance (F) and proportion (G) of six major cell types in PT and LN_met_ from the scRNA‐seq data set by Jia et al., 2023. ns, no significance, *P < .05, **P < .01, ***P < .001, ****P < .0001. aDC, activated DCs; cDC, conventional DCs; CD8+ Tem, CD8+ effector memory T; DCs, dendritic cells; DEGs, differentially expressed genes; FDR, false discovery rate; pDC, plasmacytoid DC; sscRNA‐seq, single‐cell RNA‐sequencing; Tgd cells, T gamma delta cells; Th1 cells, T helper type 1 cells; Tregs, regulatory T cells.

Molecular distinctions were further revealed between the subregions of the primary tumour and metastatic lymph nodes at the transcriptional level in 20 patients with ESCC. Following QC, transcriptome data were available from PT_sup_ (17, 85%), PT_deep_ (19, 95%) and LN_met_ (17, 85%) (Figure 1B). Two DEGs were identified between PT_sup_ and PT_deep_ (Figure 4A and Tables S9 and S10). A total of 841 DEGs between LN_met_ and PT_sup_ were identified, including 715 upregulated and 126 downregulated genes in LN_met_ (Figure 4A and Tables S9 and S10). KEGG analysis revealed that the upregulated DEGs in LN_met_ were observed in the T/B cell receptor signalling pathway, Th1/Th2/Th17 cell differentiation, cytokine‐cytokine receptor interaction, NF‐κB signalling pathway and chemokine signalling pathway (*P* < 1e^−6^) (Figure S3A and Table S11). These pathways were primarily enriched in functions such as immune response, inflammatory response and chemotaxis. The upregulated DEGs in PT_sup_ were enriched in proteoglycans in cancer and PI3K‐AKT signalling pathways, but the difference was insignificant according to the adjusted *P*‐value (Figure S3B and Table S11). Moreover, 373 DEGs between LN_met_ and PT_deep_ were observed. Of these, 221 genes exhibited overexpression in LN_met_ compared with 152 overexpressed genes in PT_deep_ (Figure 4A and Tables S9 and S10). Similarly, the upregulated DEGs in LN_met_ were significantly enhanced in pathways associated with the control of immune and inflammatory responses (Figure S3C and Table S11). The DEGs upregulated in PT_deep_ were enriched in focal adhesion and cGMP‐PKG signalling pathways (*P* < .0025) (Figure S3D and Table S11). PT_sup_ vs. LN_met_ and PT_deep_ vs. LN_met_ harboured 606 (538 upregulated and 68 downregulated) and 138 (44 upregulated and 94 downregulated) unique DEGs, respectively, with 235 (177 upregulated and 58 downregulated) overlapping DEGs (Figure 4B). The low number of DEGs between LN_met_ and PT_deep_ indicate that the RNA expression profile of the tumour in LN_met_ bears a greater resemblance to the deep subregion of the primary tumour than to the superficial subregions, which was substantiated by the proximity relationship analysis (Figure S3G).

Next, PT_sup_ and PT_deep_ were combined as PT, which was compared with the LN_met_ subregion. LN_met_ had 606 upregulated and 201 downregulated genes compared with PT (Figure 4A and Tables S9 and S10). Consistent with the analysis of LN_met_ vs. PT_sup_ and LN_met_ vs. PT_deep_, these upregulated DEGs in LN_met_ were primarily enriched in functions such as immune response, inflammatory response and chemotaxis (Figure S3E and Table S11). The upregulated DEGs in PT were enriched in protein digestion and absorption, focal adhesion and proteoglycans in cancer (*P* < .021) (Figure S3F and Table S11).

Unprecedented advances have been made in ESCC treatment with the application of immune checkpoint blockade (ICB).^49^, ^50^ However, immunomodulators and the immune cell repertoire have not been well characterised in ESCC, particularly in the subregions of the primary tumour (PT_sup_ and PT_deep_) and matched LN_met_. First, we focused on the expression of the 33 immunomodulatory genes associated with cancer immunity (Table S12). There was no significant distinction observed in the expression of these immunomodulators between PT_sup_ and PT_deep_ groups (Figure 4C). In contrast with those in PT_deep_, the immunomodulators *BTLA* and *CD48* demonstrated increased expression in LN_met_. As revealed in Figures 4C and S4A, the LN_met_ samples showed significantly higher expression levels of eight immunomodulatory genes, namely *SLAMF7, CD28, CD80, CD40LG, BTLA, TIGIT, CD86* and *CD48*, than the PT_sup_ and PT samples (*P* < .05). Thus, the heterogeneous expression of the immunomodulatory genes holds the potential to unveil novel therapeutic targets that could effectively enhance immunotherapy efficacy or impede tumour growth and metastasis in ESCC.

Next, the relative abundance and proportion of specific cell subsets in the four regions were estimated using xCell and quanTIseq, respectively (Figures 4D, S4B, S4C and S5). No significant differences between PT_sup_ and PT_deep_ were observed. Both algorithms validated that LN_met_ had significantly enriched B cells, CD8^+^ T cells and DCs compared with PT_sup_, PT_deep_ and PT (*P* < .05). The IHC results further verified that the LN_met_ subregion was characterised by a higher proportion of CD20, CD8 and CD11c positive cells (*P* < .05; Figures 4E and S6 and Table S13). Additionally, PT_sup_ had a higher abundance of T gamma delta (Tgd) cells and T helper type 1 (Th1) cells than LN_met_ (Figure 4D). Regarding the stromal cell classes, PT_sup_ samples were enriched with epithelial cells, keratinocytes and sebocytes, whereas PT_deep_ had more myocytes than LN_met_ (Figure S4C). Overall, these findings demonstrate the presence of spatial heterogeneity in the TME between metastatic lymph nodes and primary tumours of ESCC.

To further substantiate the heterogeneity of TME in ESCC, the publicly available scRNA‐seq dataset from PT and LN_met_ of ESCC by Jia et al. was investigated.^6^ This dataset includes single cells derived from four PT tissues (27,979 cells) and three LN_met_ samples (24,751 cells). Comparative analysis of the fraction of six predominant cell types between PT and LN_met_ revealed notable differences in both the abundance and proportion of cellular compositions (Figures 4F and G), suggesting the heterogeneity of TME in ESCC. Consistent with our observations of RNA‐seq data, the abundance and proportion of DCs, B cells and associated subtypes were enriched in LN_met_ (Figures 4F, 4G, S4D andS4E).

### 3.4 Spatial heterogeneities of the immune contexture in ESCC by DSP

**FIGURE 5:**
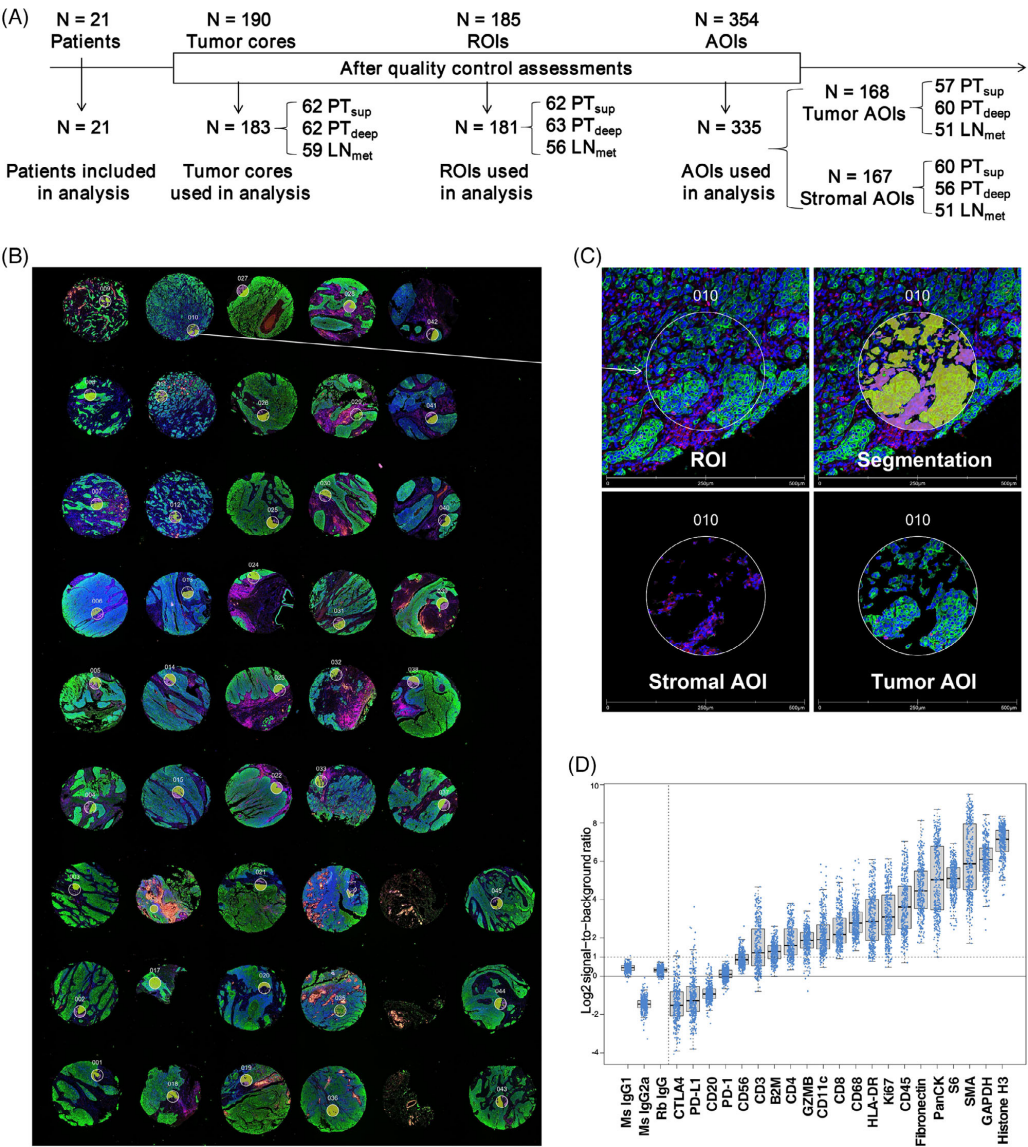
DSP of primary ESCC and metastasised LN tumour tissues. (**A**) Schematic of the study design and workflow. ESCC patients with LN metastases (n = 21) are represented in four TMA blocks for DSP of the Immune Cell Profiling panel (24 proteins). Of the total 185 ROIs, 181 were utilised for segmentation. A total of 335 AOIs were analysed. The reason for exclusion was quality control failure. (B) Representative image of TMA acquired using the GeoMx DSP system. The numerical annotations corresponding to the respective ROI numbers. (C) Representative ROI (ROI no. 10) and AOIs annotated based on histology by pathologists and immunofluorescence staining with the morphological markers and the compartmentalised image created by fluorescence colocalisation. One 300 μM ROI per core was selected for DSP. panCK (green), CD45 (red), SYTO13 (blue). The scale bar is 500 μm. (D) The quality control of the expression of each target protein relative to the negative control. Data are graphed as log2 SNR. Each dot represents an AOI. AOIs, areas of interest; DSP, digital spatial profiling; LN, lymph node; ROIs, regions of interest, SNR, signal‐to‐noise ratio; TMA, tissue microarray.

Bulk RNA‐sequencing data do not provide information on spatial relationships. Thus, spatially resolved multiplexed DSP was performed to identify the differences between the subregions by characterising immunologically relevant proteins and the immune cell repertoire (Table S14). A schematic illustration of the DSP experimental design is shown in Figure 5A. Four TMAs were constructed with 183 tumour cores punched from 62 PT_sup_, 62 PT_deep_ and 59 LN_met_ regions of 21 patients with ESCC. To select ROIs, TMA sections were stained simultaneously with fluorescently labelled antibodies for the leukocyte marker CD45 to demarcate the stroma, epithelial cell marker PanCK to mark the tumour and nuclear stain SYTO13 to mark the cell nuclei (Figure 5B). In total, 181 ROIs (62 PT_sup_, 63 PT_deep_ and 56 LN_met_), identified based on immunostaining patterns for morphological markers and histology by pathologists, were selected. Next, each ROI was segmented into two AOIs as stroma‐enriched (CD45‐positive staining and stroma‐enriched segment) and tumour‐enriched (PanCK‐positive staining and tumour‐enriched segment) compartments using a masking and segmentation strategy (Figure 5C). In total, 335 AOIs met the QC criterion, comprising 168 tumour AOIs (57 PT_sup_, 60 PT_deep_ and 51 LN_met_) and 167 stromal AOIs (60 PT_sup_, 56 PT_deep_ and 51 LN_met_) (Figure 5A). The probe QC analysis revealed that most targets exhibited high signal in comparison to the non‐specific counts across all samples, except PD‐L1, PD‐1, CTLA4 and CD20 (Figure 5D). We observed that the transcript levels of genes encoding PD‐L1, PD‐1, CTLA4 and CD20 were remarkably low, thereby confirming a high concordance between the transcript‐based measurements and antibody‐based assessments for these proteins (Figure S7A). ESCC tissues generally have a low PD‐L1 expression,^51^, ^52^, ^53^ and Yan et al. revealed the heterogeneity of PD‐L1 expression within distinct spatial regions in ESCC.^19^ We determined that the PD‐L1 protein level was low in many AOIs and distinct among the AOIs (Figures 5D, S7B andS7C), which verified these findings.

Distinct patterns of tumour and stromal AOIs were observed using dimensionality reduction analysis (Figure S7D). Tumour AOIs enriched protein markers PanCK and Ki‐67, closely linked with the tumour phenotype, whereas proteins that mark immune cells were enriched in stromal AOIs (Figure S8A). These results established that the segmentation of ROIs worked well. Notably, the stromal AOIs could be divided into two clusters (clusters 2 and 3) (Figure S8A and Table S15). Specifically, most stromal AOIs from LN_met_ belonged to cluster 3, which was enriched with proteins that mark distinct immune cell types and antigen‐presenting proteins (Figure S8A), indicating that the LN_met_ region had a distinct TME compared with the primary tumour.

**FIGURE 6:**
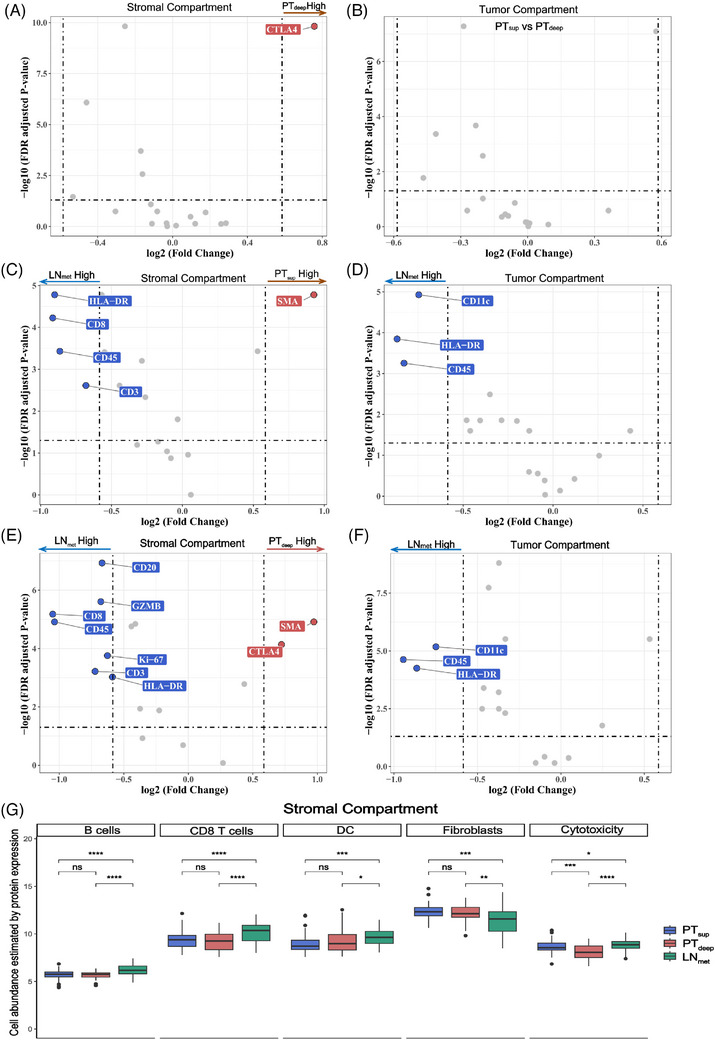
Differential protein expression analysis and cell abundance of spatially defined AOIs identified by DSP in the three subregions. (A and B) Volcano plots showing differential expression of proteins based on comparison of PT_sup_ versus PT_deep_ in stromal (A) and tumour‐enriched (B) compartments. (C and D) Volcano plots showing differential expression of proteins based on comparison of LN_met_ versus PT_sup_ in stromal (C) and tumour‐enriched (D) compartments. (E and F) Volcano plots showing differential expression of proteins based on comparison of LN_met_ versus PT_deep_ in stromal (E) and tumour‐enriched (F) compartments. −log10 (FDR‐adjusted *P*‐value), which increases with statistical significance, is indicated along the *y*‐axis. The dotted horizontal line represents the adjusted *P*‐value cutoff (FDR < .05). The dotted vertical lines represent the log2 (Fold change) cutoffs (|FC| > 1.5). Points in red and blue are those that achieved statistical significance. (G) Boxplots showing the cell abundance estimated by protein expression in the three distinct subregions in the stromal compartment (ns, no significance, *P < .05, **P < .01, ***P < .001, ****P < .0001). Pairwise comparison was conducted using the Wilcoxon rank‐sum test. For the boxplot, the centerline represents the median, and box limits represent the upper and lower quartiles. AOIs, areas of interest; FDR, false discovery rate.

Next, we compared the differential expression of proteins among the three subregions (PT_sup_, PT_deep_ and LN_met_) in all, tumour and stromal AOIs. The average of all AOIs within each tumour sample was computed to obtain a composite value representing simulated bulk sequencing data. We first visualised the significance (FDR‐adjusted *P* < .05) of all markers. Most markers exhibited differential expression in LN_met_ compared with that in PT_sup_ or PT_deep_ in all, tumour and stromal AOIs (Table S16). To identify significantly differentially expressed proteins in each comparison group, FDR < .05 and |FC| > 1.5 were applied (Table S17). We noted significantly higher expression of CTLA4 in the stromal and all but not tumour AOIs of PT_deep_ than in those of PT_sup_ and LN_met_ (Figures 6A vs. B, 6E vs. F, S8B, S8D and S9). Using simulated bulk data, significantly higher expression of CD45, CD3, CD8 and HLA‐DR was observed in LN_met_ than in PT_sup_ and PT_deep_ (Figures S8C andD). In contrast with those in PT_sup_ and PT_deep_, the expression of CD45, CD3, CD8 and HLA‐DR in the stromal compartment and that of CD45, CD11c and HLA‐DR in the tumour‐enriched compartment exhibited the most significant increase in LN_met_ (Figures 6C–F). Moreover, elevated expression levels of CD20 and granzyme B (GZMB) (indicating cytotoxicity) were observed in the stromal AOIs of LN_met_ compared with those of PT_deep_ (Figure 6E). In contrast, SMA was enriched in stromal AOIs in primary tumours (Figures 6C and E). Taken together, spatial proteomics revealed certain differences between PT_sup_ and PT_deep_ and a clear distinction between the LN_met_ and primary tumour in both ESCC stromal and tumour regions.

Next, we evaluated and compared the heterogeneity of immune cell infiltration in the three subregions. Consistent with the UMAP patterns, the cell abundance profiles exhibited a distinct spatial demarcation between the tumour and stroma (Figure S8E). The primary and lymph node metastasis tumour cores exhibited predominant enrichment of epithelial cells, while their stromal compartments were enriched in immune cells and fibroblasts (Figure S8E). Then, we compared the immune cell abundance among the three subregions in the stromal compartment (Figure 6G and Table S18). The LN_met_ subregion had a significantly higher abundance of four immune‐related cell types (B, CD8^+^ T, DCs and cytotoxic cells) in the stromal AOIs than PT_sup_ and PT_deep_, which was in accordance with the bulk RNA‐seq data (Figures 4D and E). Fibroblasts were enriched in primary tumours compared with that in LN_met_, in accordance with the scRNA‐seq data (Figures 4F and G). Furthermore, PT_sup_ contained more cytotoxic cells in the stromal region than PT_deep_. Collectively, these data unveil the heterogeneity of the ESCC immune landscape among the three subregions in the stromal region through spatial segmentation using DSP.

To gain a deeper understanding of spatial ITH, the expression patterns of proteins and abundance of immune cells among spatially distinct AOIs for each patient in every subregion were analysed. Variations in protein levels were observed among AOIs within the tumour and stromal compartments of the same subregion and patient (Figures S10A–C). Moreover, we observed some degree of heterogeneity in terms of immune cell abundance within the stromal AOIs for each patient in every subregion (Figures S11A–C). Collectively, the utilisation of DSP allows for a clear observation of the intra‐AOI heterogeneity present in patients with ESCC.

### 3.5 Multi‐omics integrative analysis reveals spatial heterogeneity

**FIGURE 7:**
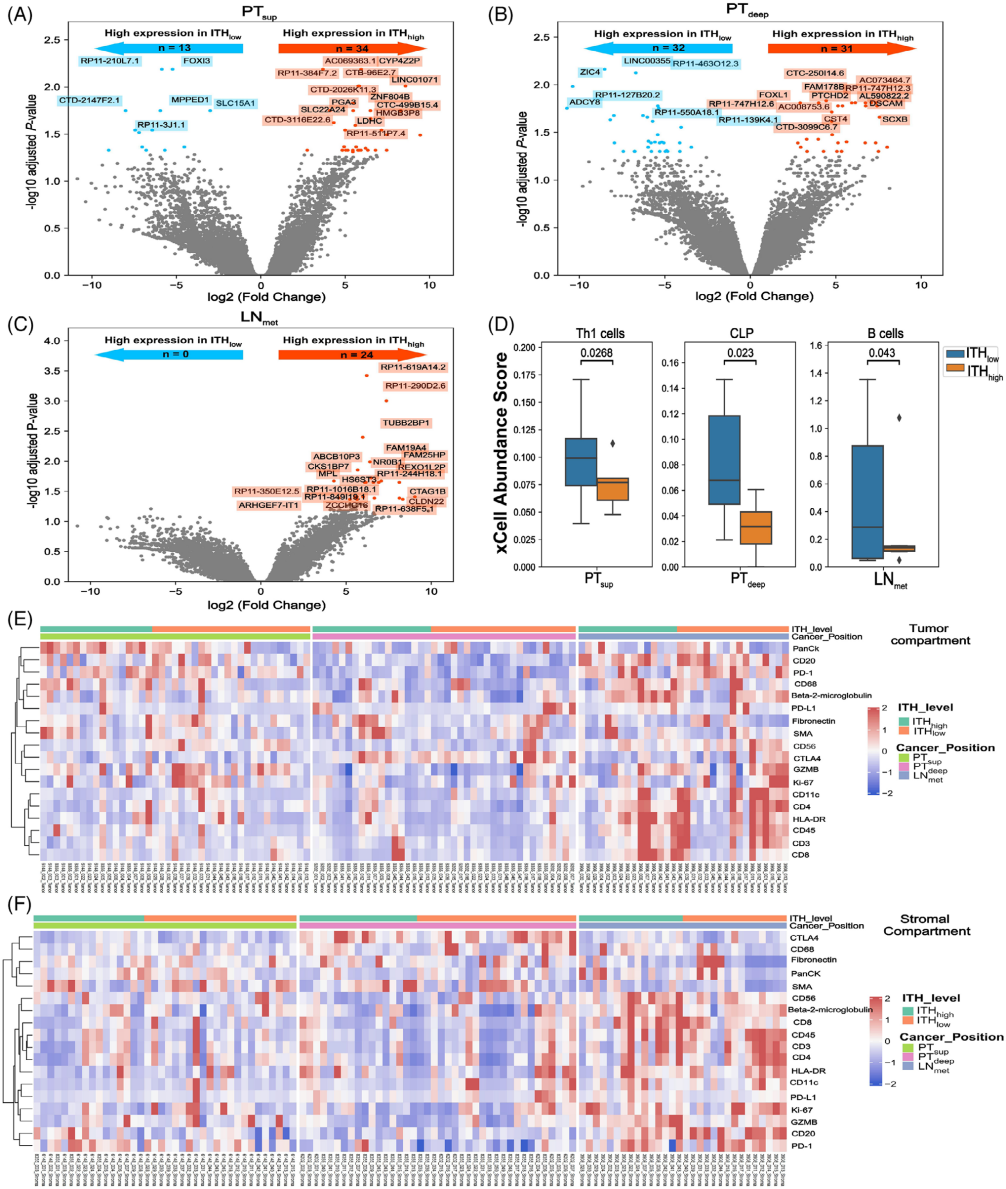
The association of genomic ITH levels with RNA/protein expression and immune cell abundance. (A–C) Volcano plots of DEGs between genomic ITH_high_ and ITH_low_ groups in PT_sup_ (A), PT_deep_ (B) and LN_met_ (C), respectively. FDR is calculated using the Benjamini–Hochberg method. (D) Boxplots showing the association between genomic ITH levels and immune cell abundance in the three distinct subregions estimated using xCell. The Student's *t*‐test was used to compare differences. For the boxplot, the centerline represents the median, and box limits represent the upper and lower quartiles. Each dot represents a sample. (E–F) Heatmaps showing the association between genomic ITH levels and relative expression of proteins per AOI in tumour (E) or stromal compartment (F) of each subregion. Colour key: Colour gradient blue to red indicates relative expression levels from low to high.

To gain a deeper comprehension of the intricate spatial heterogeneity in ESCC, we integrated multi‐omics data, including genomic ITH level, mRNA/protein expression and immune cell abundance. Each subregion was classified as either ITH_high_ or ITH_low_ based on the median ITH value (Table S6), to further unveil the heterogeneity within ESCC subregions. As shown in Figures 7A–C and Table S19, DEGs were observed between the ITH_high_ and ITH_low_ groups in PT_sup_, PT_deep_ and LN_met_, indicating transcriptional heterogeneity in each subregion of ESCC. Additionally, we identified a significant association between genomic ITH level and immune cell abundance (Figure 7D). Briefly, the abundance of Th1 cells in PT_sup_, cytotoxic T lymphocyte precursor cells in PT_deep_ and B cells in LN_met_ were significantly higher in the ITH_low_ group than in the ITH_high_ group (*P* < .05), underscoring the heterogeneity of TME in subregions with distinct genomic ITH levels. When focusing on tumour or stromal compartments, divergent protein and immune cell profiles were presented in each group within each subregion (Figures 7E, F and S11D), further highlighting the spatial heterogeneity in ESCC.

## 4 DISCUSSION

ESCC is recognised as a heterogeneous group of cancers. This study comprehensively analysed the multi‐omics features, including genomics, transcriptomics and spatial proteomics, of PT_sup_, PT_deep_ and LN_met_ subregions in ESCC and revealed spatial ITH. Genomic ITH is characterised by an uneven distribution, either spatially or temporally, of genomic diversity within an individual tumour^54^ and has posed a formidable challenge in the realm of precision oncology.^16^ Fang et al. indicated that genomic ITH is a potential biomarker that enables the prediction of the efficacy of tumours treated with ICB, including in EC.^38^ In our study, PT_sup_, PT_deep_ and LN_met_ subregions were identified to harbour unique somatic mutations, suggesting that the genomic alterations occurring within the deep subregions and/or metastatic sites, which propel tumour oncogenesis, hold the potential for being effectively targeted but have been overlooked based on the present clinical sampling depth of ESCC. Additionally, differences in CNA patterns among the three subregions were observed. A recent study on ESCC likewise unveiled a significant heterogeneity in CNA patterns between the primary tumour cells and lymph node metastatic malignant epithelial cells.^6^ Thus, comprehensive genomic profiling of PT_sup_, PT_deep_ and LN_met_ subregions in the same patient may be beneficial for accurate estimation of genomic ITH. The presence of HLA LOH, signifying impairment in the presentation of neoantigens, has been demonstrated to exhibit a clinical correlation with unfavourable prognosis and mediates resistance to ICB.^55^ To our knowledge, this study is the first to investigate the frequency of the somatic tumour HLA‐I status. Furthermore, we discovered that the occurrence of HLA LOH is common in ESCC, and that the HLA status is inconsistent among subregions. Elucidating the clonal status of driver mutations assists in discerning between early and late events, and targeting the clonally dominant driver mutations (early events) represents a refined approach to therapeutic intervention.^56^, ^57^ Driver mutations exhibit diverse and intricate clonal statuses within different subregions of ESCC tumours, aligning with the outcomes of a previous study.^18^ Our findings indicate that the prevalence of these driver events and the overall rate of subclonality are prone to be underestimated when relying solely on a single biopsy to represent an individual patient.

Distinct RNA profiles between LN_met_ and the primary tumour in ESCC were observed. Consistent with previous findings in gastric cancers,^58^ the comparison of DEGs between the deep tumour subregion and LN_met_ revealed fewer discrepancies, implying a higher likelihood that tumour cells in LN_met_ originate from the deep subregion rather than the superficial subregion. Based on these findings, biomarker analyses performed on superficial biopsies alone may overlook important information regarding the deep subregions and/or metastatic sites of ESCC. Therefore, multiregional sampling may be necessary to minimise the effects of ITH.

The presence of metastatic LNs can exert a considerable influence on the immune response towards tumours. Remarkably, targeting LN_met_ can substantially augment the therapeutic efficacy on primary tumours.^59^ Therefore, it is vital to characterise and discriminate between the microenvironments of LN_met_ and PT. Our findings demonstrate that there is spatial heterogeneity in the TME between metastatic LNs and ESCC primary tumours, with more immune cells in the TME of LN_met_. Jia et al. revealed that the TME of metastatic LNs is more complex than that of ESCC primary tumours and first identified interferon‐induced B and T cell subsets that uniquely existed within the LN_met_ microenvironment of ESCC.^6^ Quah et al. revealed differences in composition between primary and metastatic sites, with higher proportions of B, plasma and DCs in LN_met_.^60^ Wang et al. investigated the heterogeneity of TME between metastatic and primary sites in oral squamous cell carcinoma and revealed a remarkable increase in cytotoxic T cells and M1 macrophages in LN_met_, whereas the abundance of M2 macrophages decreased.^61^ Qian et al. revealed the heterogeneities of the TME in primary tumours and metastatic LNs of gastric cancer.^62^ Liu et al. generated single‐cell maps of PT and paired LN_met_ in eight patients with breast cancer and reported that B and CD4 T cells were enriched in the microenvironment of LN_met_; however, CD8 T cells, cancer‐associated fibroblasts and mast cells were abundant in PT.^63^ TME heterogeneity between the primary tumour and locoregional metastases in the same patient may account for differences in response to immunotherapy.^64^ Collectively, the insights derived from these findings possess the potential to shape the TME and guide diverse therapeutic strategies aimed at impeding tumour progression and overcoming metastasis.

Although ICB has revolutionised ESCC therapeutics, not all patients gain clinical benefits from these treatments,^50^ which raises the question of whether the spatial ITH is responsible for these differences. This study utilised emerging DSP technology to address the AOIs of specific tumours and stroma and to assess the fundamental molecular nature of our ESCC cohort. In addition, this technology has the capacity to integrate multiplexed molecular assays with a spatial context, allowing for the identification of heterogeneity. This has the potential to greatly bolster the accuracy of tumour diagnoses and shed light on pioneering treatment strategies.^26^ In our study, the expression of immunologically relevant proteins exhibited differences among subregions in both the stromal and tumour compartments. Therefore, a better understanding of immune‐related protein expression profiling of ESCC will enable the tailored treatment of patients to further improve outcomes. Advances in immunotherapy have uncovered PD‐L1 as a potential therapeutic target and biomarker in patients with ESCC.^53^, ^65^ Hwang et al. revealed that the ITH of PD‐L1 expression may result in misclassification of the PD‐L1 status.^66^ Yan et al. reported that the expression of PD‐L1 in ESCC exhibits significant heterogeneity across various spatial regions.^19^ In this study, we demonstrated the heterogeneity of PD‐L1 expression within the AOIs of ESCC. Thus, by employing spatial segmentation, certain traditional biomarkers can provide more insightful information, emphasising the need for further investigation to ascertain the impact of spatial ITH on the accuracy of biomarkers. Moreover, CTLA4 was identified to have a higher level in the stromal AOIs of PT_deep_ than in those of PT_sup_ and LN_met_, indicating that the assignment of CTLA4 expression may be influenced by spatial heterogeneity. Consistent with whole‐transcriptomic data, the stromal compartment of the LN_met_ subregion had a significantly increased abundance of immune‐related cell types. We also found that PT_sup_ contained more cytotoxic cells in the stromal region than PT_deep_. These results indicate that the TME exhibits spatial heterogeneity in ESCC. To our knowledge, this study is the first to explore the spatial ITH in ESCC using the DSP platform. The ability of DSP to isolate proteomics data from defined tissue areas without destroying or cutting the tissue is extremely valuable, thus providing a foundation for additional investigations.

This study has some limitations. Firstly, our study solely focused on primary tumours and metastatic lymph nodes, excluding distant metastatic sites from analysis. Secondly, we only included locally advanced stage ESCC patients with metastatic lymph nodes; thus, whether the observed ITH is exclusive to tumours with LN_met_ or if it can also be observed in primary ESCC without LN_met_ requires further investigation. Finally, although we innovatively used the DSP method to quantitate proteins in the spatially defined subregions of ESCC, we only focused on the Immune Cell Profiling Panel. Therefore, future studies should analyse other DSP protein panels to further explore the differential proteomic landscapes in the stromal and tumour AOIs of the three subregions.

## 5 CONCLUSIONS

Our study comprehensively characterised the genomic, transcriptomic and spatial proteomic heterogeneity in ESCC defined by spatial subregions. Our findings underscore the clinical significance of unbiased molecular classification relying on multi‐omics data, thereby advancing the current comprehension and management of ESCC. Consideration of the deeper parts of the primary tumour and/or LN_met_ must guide the diagnosis, biomarker testing and treatment of ESCC. Accordingly, future studies and clinical trials in ESCC should consider this spatial heterogeneity.

## AUTHOR CONTRIBUTIONS

Yu Feng, Haitao Ma and Wei Jiang conceived the study. Na Li, Haitao Huang and Yingkuan Liang wrote the manuscript. Jinyuan Xiao and Hongzhen Tang assisted in bioinformatic analysis. Na Li, Haitao Huang, Yingkuan Liang, Xing Tong and Rutao Li analysed the data. Dong Jiang, Kai Xie and Chen Fang performed the IHC experiment. Guangbin Li, Bin Wang and Shaomu Chen collected the tumour samples. Haitao Luo, Jiaqian Wang and Lingchuan Guo provided critical discussions. Yu Feng, Na Li and Haitao Huang edited the manuscript. Yu Feng, Haitao Ma, Wei Jiang and Na Li supervised this study.

## FUNDING INFORMATION

This study was supported by the National Natural Science Foundation of China (Grant No. 81972800) and Suzhou Medical Science and Technology Innovation Project (SKY2022142).

## CONFLICTS OF INTEREST STATEMENT

The authors declare that they have no competing interests.

## ETHICS APPROVAL

This study was approved by the Ethics Committee of the First Affiliated Hospital of Soochow University (approval number [2022]427) and was performed in accordance with the provisions of the Ethics Committee of Soochow University and Declaration of Helsinki.

## Supporting information

Figure S1. Comparison of the number of mutations, mutational categories and genomic biomarkers among the three subregions of ESCC.
(A) The stacked bar chart displaying the number of mutational categories in each subregion per case. (B) The boxplot showing no significant difference in the number of mutations among PT_sup_, PT_deep_ and LN_met_ in 15 ESCCs. (C) The boxplot indicating no difference in the mutational categories among PT_sup_, PT_deep_ and LN_met_. (D‐G) The boxplots showing no significant difference in MSI (D), TMB (E), TNB (F) and ITH levels (G) among the three subregions. (H) The LOH statuses of *HLA‐A*, *HLA‐B* and *HLA‐C* in the three subregions of each case. (I) The proportion of LOH for *HLA‐A*, *HLA‐B* and *HLA‐C* in each subregion. The *P* values overlaid onto the bar plot were calculated using Fisher's exact test. ESCC, oesophageal squamous cell carcinoma; HLA, human leukocyte antigen; LOH, loss of heterozygosity; LN_met_, lymph node metastasis; MSI, microsatellite instability; PT_sup_, primary tumour superficial; PT_deep_, primary tumour deep; TMB, tumour mutational burden; TNB, tumour neoantigen burden.

Figure S2. The proportion of driver mutations and mutational signatures in ESCC tumours.
(**A**) Bar plots showing the proportions of putative driver mutations versus other mutations on the trunks and branches. The number of mutations on the trunks and branches was shown. Statistical differences of truncal and branched proportions between driver and other mutations across all cases were analysed using a Fisher's exact test, and a significant *P* value is shown. (**B**) The 96‐trinucleotide mutational spectrum of truncal (Top panel) and branched (Bottom panel) mutations across all regions, as inferred by deconstructSigs. Current SBS signatures have been identified using 96 different contexts constituted by the six base substitutions C > A, C > G, C > T, T > A, T > C and T > G (in which the mutated base is represented by the pyrimidine of the base pair), considering not only the mutated base, but also the bases immediately 5′ and 3′. (**C**) Boxplots displaying the contributions of individual mutational signatures to individual cases, with each dot representing one case. Signatures 1−30 were based on the Wellcome Trust Sanger Institute COSMIC Mutational Signature Framework. For the boxplot, the centerline represents the median, and box limits represent upper and lower quartiles. (D) Pie charts displaying the distinct contribution of the truncal and branch mutational signatures in the 15 ESCC cases. SBS, single‐base substitution.

Figure S3. Functional pathways analyse of the differentially expressed genes and proximity relationship analysis.
(A–F) Enriched KEGG pathways of the up‐regulated genes in LN_met_ versus PT_sup_ (A), PT_sup_ versus LN_met_ (B), LN_met_ versus PT_deep_ (C), PT_deep_ versus LN_met_ (D), LN_met_ versus PT (E) and PT versus LN_met_ (F) are shown. The top 20 pathways, sorted by statistical significance (log base 10 of *P*‐values), are shown. *P*‐value was corrected using Benjamini & Hochberg method. KEGG, Kyoto Encyclopedia of Genes and Genomes. (**G**) The UMAP dimensionality reduction analysis demonstrating that the RNA expression characteristics of PT_deep_ are closer to LN_met_ than PT_sup_.

Figure S4. Differential expression of immunomodulatory genes and distinct immune‐related cell types between the primary tumour and LN_met_.
(**A**) Comparisons of the expression level of immunomodulatory genes in LN_met_ and PT. These comparisons were performed as part of the RNAseq analysis pipeline using DeSeq2. The adjusted *P* value (corrected *P* value for multiple hypothesis testing) was adopted. (**B**) Boxplots showing the abundance of immune and non‐immune cells estimated using xCell in LN_met_ and PT. The Student's *t*‐test was used to compare differences. (**C**) Boxplots showing the abundance of non‐immune cells estimated using xCell in LN_met_ and PT subregions. The Student's *t*‐test was used to compare differences. (D and E) Barcharts displaying cell abundance (D) and proportions (E) of B cell subtypes in PT and LN_met_ from the scRNA‐seq data set by Jia et al, 2023. ns, no significance, *P < .05, **P < .01, ***P < .001, ****P < .0001. Tcm, central memory T cells; Tem, effector memory T cells; DCs, dendritic cells; cDC, conventional DCs; iDC, immature DCs; aDC, activated DCs; pDC, plasmacytoid DCs; Tgd cells, T gamma delta cells; Th1 cells, T helper type 1 cells; Tregs, regulatory T cells; CLP, cytotoxic T lymphocyte precursor cells; CMP, common myeloid progenitor ***cells;*** GMP, granulocyte‐macrophage progenitor cells; NK, natural killer cells; NKT, natural killer T cells; DN B cells, double‐negative B cells; GC B cells, germinal center B cells; scRNA‐seq, single‐cell RNA‐sequencing.

Figure S5. The proportion of distinct immune‐related cell types estimated using quanTIseq.
(**A**) Boxplots showing the proportion of 10 major immune‐related cells in LN_met_ and PT subregions. (**B**) Boxplots showing the proportion of 10 major immune‐related cells in LN_met_ and PT. For the boxplot, the centerline represents the median, and box limits represent upper and lower quartiles. Each dot represents a sample. Pairwise comparison was conducted using the Wilcox rank sum tests.

Figure S6. Representative immunohistochemical images of CD11c, CD8 and CD20 expression in PT_sup_, PT_deep_ and LN_met_ subregions.

Figure S7. Quality control of AOIs by DSP.
(**A**) Expression is consistent across RNA and protein DSP in fibronectin, HLA‐DR, PD‐1, CD20, PD‐L1 and CTLA4. Data are presented as log2 normalised counts. (**B and C**) The quality control of the expression of each target protein in tumour (**B**) or stromal (**C**) compartment relative to negative control. Data are graphed as log2 SNR. Each dot represents an AOI. (**D**) UMAP dimensionality reduction analysis of AOIs at protein level. AOIs, areas of interest; DSP, digital spatial profiling; SNR, signal‐to‐noise ratio; UMAP, uniform manifold approximation and projection.

Figure S8. The distinct protein expression patterns and abundance of immune cells in spatial AOIs identified by DSP.
(A) Clustered heatmap showing the relative expression of proteins per AOI in stromal or tumour compartment of each subregion. Colour key: Colour gradient blue to red indicates relative expression levels from low to high. (**B–D**) Volcano plots showing differential expression of proteins based on comparison of PT_sup_ versus PT_deep_ (**B**), LN_met_ versus PT_sup_ (**C**) and LN_met_ versus PT_deep_ (**D**) through mimic bulk sequencing in the whole area. (**E**) Heatmap of the lymphocyte infiltration abundance estimated by protein expression in stromal or tumour compartment of each subregion. Colour key: Colour gradient blue to red indicates relative levels from low to high. AOIs, areas of interest; DSP, digital spatial profiling.

Figure S9. The CTLA4 expression level in LN_met_ and PT subregions of each patient. Boxplots showing the CTLA4 expression level in all AOIs (A) and stromal compartment (B).

Figure S10. Analysis of the intra‐AOI heterogeneity in protein levels. (A–C) Heatmaps of protein levels among AOIs in PT_sup_ (A), PT_deep_ (B) and LN_met_ (C).

Figure S11. Analysis of the intra‐AOI heterogeneity in immune cell abundance.
**(A–C)** Heatmaps of immune cell abundance among AOIs in PT_sup_ (**A**), PT_deep_ (**B**) and LN_met_ (**C**); (**D**) heatmap showing the association between genomic ITH levels and the lymphocyte infiltration abundance estimated by protein expression in stromal compartment of each subregion.

**Table S1**. Detailed clinicopathological features of 21 patients with ESCC.

**Table S2**. The quality control of WES, RNA‐seq and DSP data.

**Table S3**. Sample list and various analyses performed per section.

**Table S4**. Tumour purity estimated by All‐FIT and FACETs.

**Table S5**. Detailed information of all somatic mutations in 45 tumour subregions from 15 ESCC patients.

**Table S6**. MSI, TMB, TNB and ITH levels in the three subregions of each patient.

**Table S7**. Information of copy number alterations (CNAs).

**Table S8**. Detailed information of driver mutations in tumour subregions of each patient.

**Table S9**. Number of genes in the whole transcriptome with significant differences in analyses of tumour subregions.

**Table S10**. Genes in the whole transcriptome with significant differences in analyses of tumour subregions.

**Table S11**. Enriched pathways of DEGs in analyses of tumour subregions by KEGG analysis.

**Table S12**. Expression of immunomodulators across distinct regions.

**Table S13**. The proportion of CD11c, CD8 and CD20 staining positive cells in each subregion.

**Table 14**. Probe details in the NanoString Human Immune Cell Profiling panel.

**Table S15**. Information of all AOIs.

**Table S16**. Differential proteins with FDR < .05 between subregions in the whole area, stromal and tumour compartments, respectively.

Table S17. Differential proteins with both FDR < 0.05 and |FC| > 1.5 between subregions in the whole area, stromal and tumour compartments, respectively.

**Table S18**. Protein markers for cell types.

**Table S19**. Differentially expressed genes between genomic ITH_high_ and ITH_low_ groups.

## Data Availability

This study did not generate any unique reagents or materials. All the raw data have been deposited to the public database ‘ Genome Sequence Archive (GSA) ’ with the dataset identifier PRJCA017168. Other data that support the findings of this study are available from the corresponding author upon reasonable request.
