# Supplementary figures and images for: Multi‐omics analyses reveal spatial heterogeneity in primary and metastatic oesophageal squamous cell carcinoma
Source: Clin Transl Med. 2023 Nov 27;13(11):e1493. doi: 10.1002/ctm2.1493 (PMC10679972; doi:10.1002/ctm2.1493)

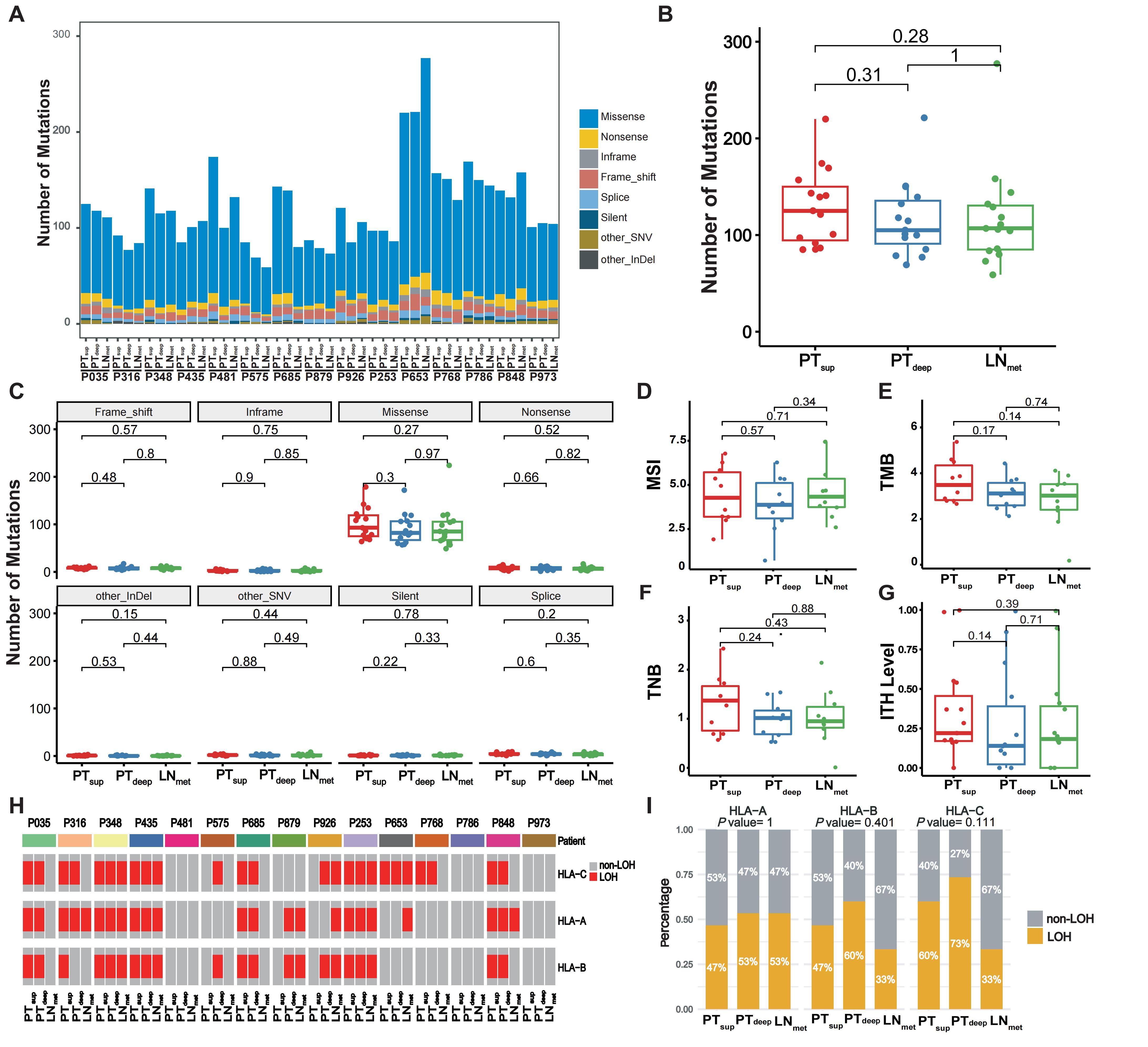

Supplement: Supplementary file 1 — Figure S1. Comparison of the number of mutations, mutational categories and genomic biomarkers among the three subregions of ESCC. (A) The stacked bar chart displaying the number of mutational categories in each subregion per case. (B) The boxplot showing no significant difference in the number of mutations among PTsup, PTdeep and LNmet in 15 ESCCs. (C) The boxplot indicating no difference in the mutational categories among PTsup, PTdeep and LNmet. (D‐G) The boxplots showing no significant difference in MSI (D), TMB (E), TNB (F) and ITH levels (G) among the three subregions. (H) The LOH statuses of HLA‐A, HLA‐B and HLA‐C in the three subregions of each case. (I) The proportion of LOH for HLA‐A, HLA‐B and HLA‐C in each subregion. The P values overlaid onto the bar plot were calculated using Fisher's exact test. ESCC, oesophageal squamous cell carcinoma; HLA, human leukocyte antigen; LOH, loss of heterozygosity; LNmet, lymph node metastasis; MSI, microsatellite instability; PTsup, primary tumour superficial; PTdeep, primary tumour deep; TMB, tumour mutational burden; TNB, tumour neoantigen burden. [file CTM2-13-e1493-s020.jpg]

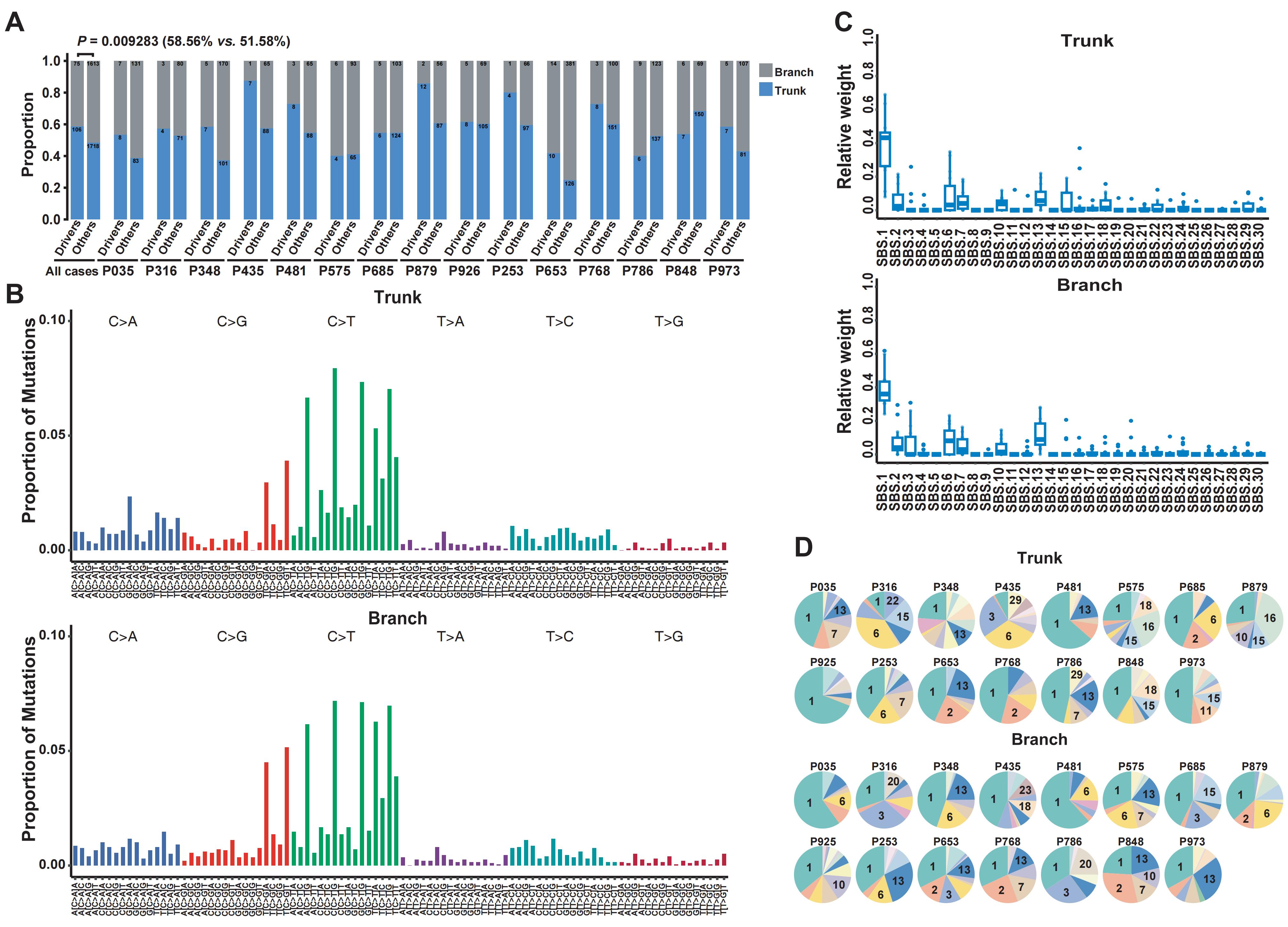

Supplement: Supplementary file 2 — Figure S2. The proportion of driver mutations and mutational signatures in ESCC tumours. (A) Bar plots showing the proportions of putative driver mutations versus other mutations on the trunks and branches. The number of mutations on the trunks and branches was shown. Statistical differences of truncal and branched proportions between driver and other mutations across all cases were analysed using a Fisher's exact test, and a significant P value is shown. (B) The 96‐trinucleotide mutational spectrum of truncal (Top panel) and branched (Bottom panel) mutations across all regions, as inferred by deconstructSigs. Current SBS signatures have been identified using 96 different contexts constituted by the six base substitutions C > A, C > G, C > T, T > A, T > C and T > G (in which the mutated base is represented by the pyrimidine of the base pair), considering not only the mutated base, but also the bases immediately 5′ and 3′. (C) Boxplots displaying the contributions of individual mutational signatures to individual cases, with each dot representing one case. Signatures 1−30 were based on the Wellcome Trust Sanger Institute COSMIC Mutational Signature Framework. For the boxplot, the centerline represents the median, and box limits represent upper and lower quartiles. (D) Pie charts displaying the distinct contribution of the truncal and branch mutational signatures in the 15 ESCC cases. SBS, single‐base substitution. [file CTM2-13-e1493-s021.jpg]

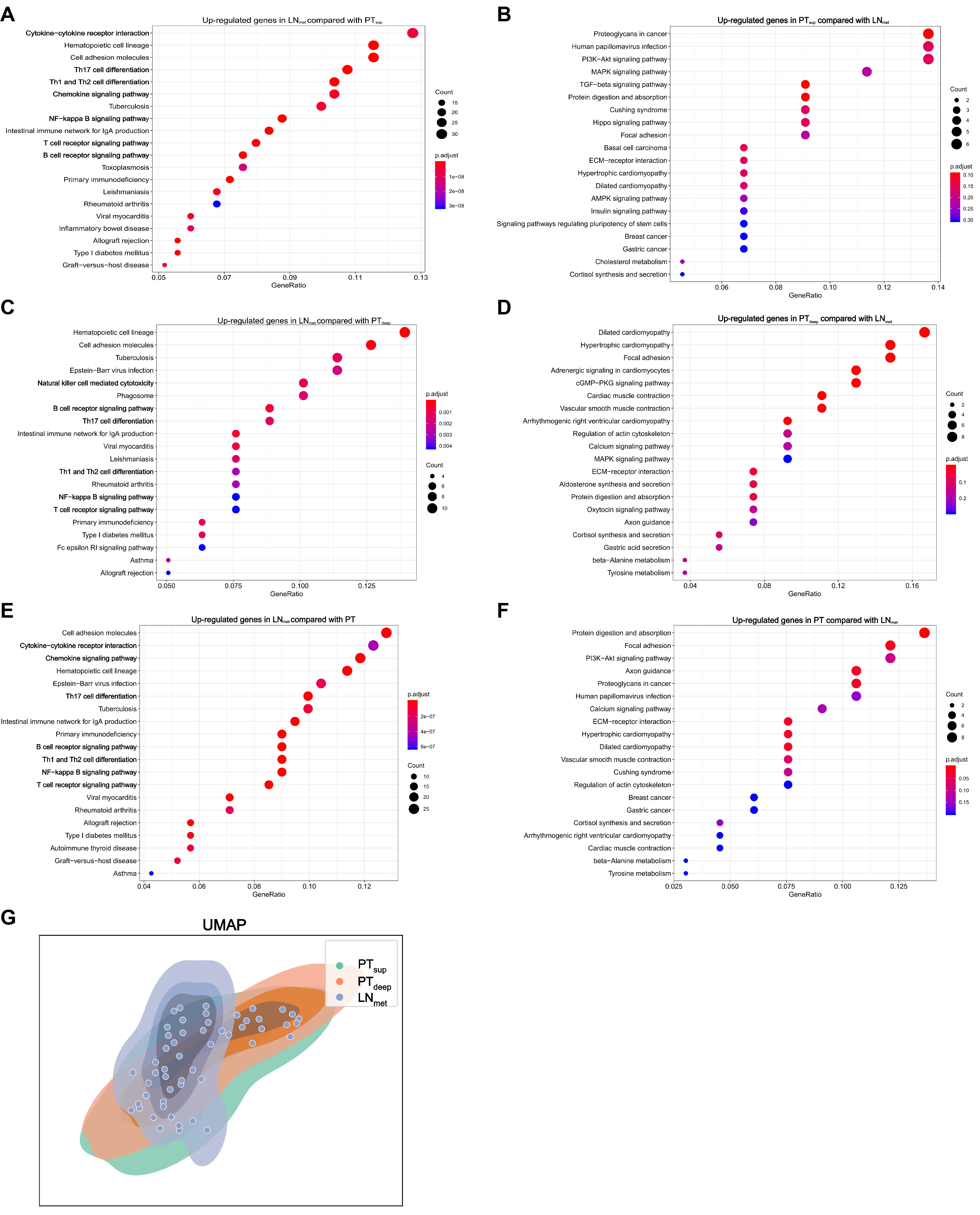

Supplement: Supplementary file 3 — Figure S3. Functional pathways analyse of the differentially expressed genes and proximity relationship analysis. (A–F) Enriched KEGG pathways of the up‐regulated genes in LNmet versus PTsup (A), PTsup versus LNmet (B), LNmet versus PTdeep (C), PTdeep versus LNmet (D), LNmet versus PT (E) and PT versus LNmet (F) are shown. The top 20 pathways, sorted by statistical significance (log base 10 of P‐values), are shown. P‐value was corrected using Benjamini & Hochberg method. KEGG, Kyoto Encyclopedia of Genes and Genomes. (G) The UMAP dimensionality reduction analysis demonstrating that the RNA expression characteristics of PTdeep are closer to LNmet than PTsup. [file CTM2-13-e1493-s009.jpg]

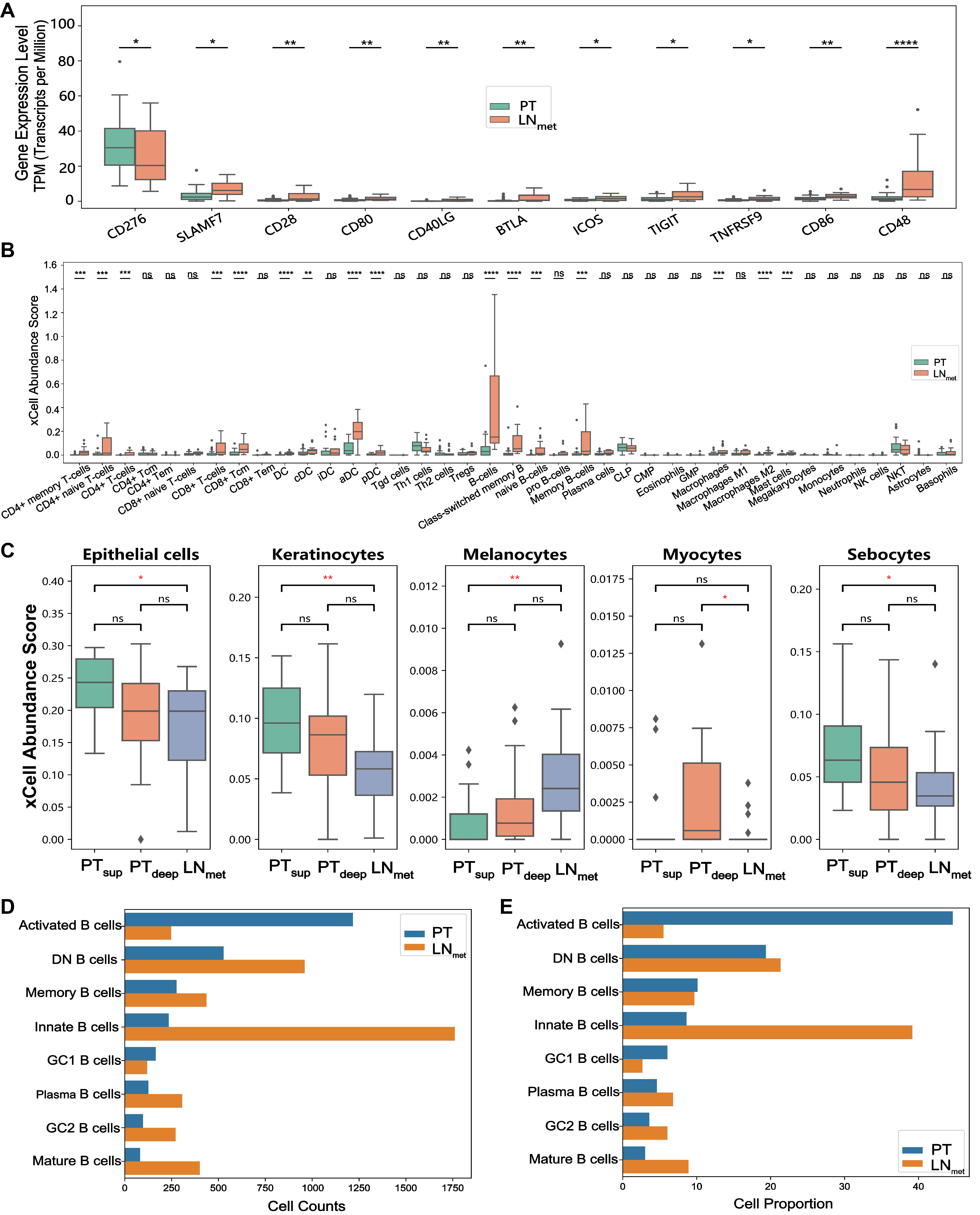

Supplement: Supplementary file 4 — Figure S4. Differential expression of immunomodulatory genes and distinct immune‐related cell types between the primary tumour and LNmet. (A) Comparisons of the expression level of immunomodulatory genes in LNmet and PT. These comparisons were performed as part of the RNAseq analysis pipeline using DeSeq2. The adjusted P value (corrected P value for multiple hypothesis testing) was adopted. (B) Boxplots showing the abundance of immune and non‐immune cells estimated using xCell in LNmet and PT. The Student's t‐test was used to compare differences. (C) Boxplots showing the abundance of non‐immune cells estimated using xCell in LNmet and PT subregions. The Student's t‐test was used to compare differences. (D and E) Barcharts displaying cell abundance (D) and proportions (E) of B cell subtypes in PT and LNmet from the scRNA‐seq data set by Jia et al, 2023. ns, no significance, *P < .05, **P < .01, ***P < .001, ****P < .0001. Tcm, central memory T cells; Tem, effector memory T cells; DCs, dendritic cells; cDC, conventional DCs; iDC, immature DCs; aDC, activated DCs; pDC, plasmacytoid DCs; Tgd cells, T gamma delta cells; Th1 cells, T helper type 1 cells; Tregs, regulatory T cells; CLP, cytotoxic T lymphocyte precursor cells; CMP, common myeloid progenitor cells; GMP, granulocyte‐macrophage progenitor cells; NK, natural killer cells; NKT, natural killer T cells; DN B cells, double‐negative B cells; GC B cells, germinal center B cells; scRNA‐seq, single‐cell RNA‐sequencing. [file CTM2-13-e1493-s003.jpg]

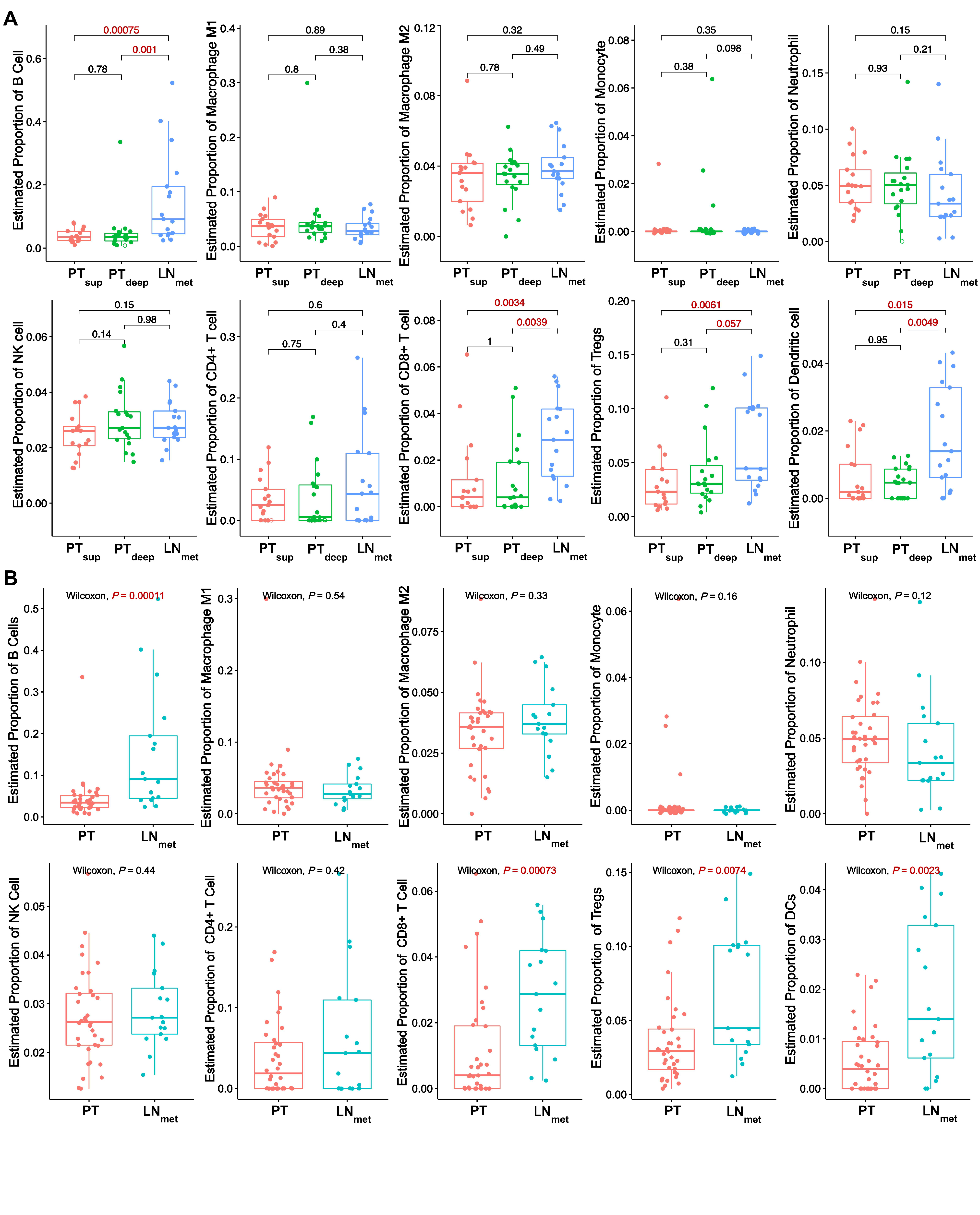

Supplement: Supplementary file 5 — Figure S5. The proportion of distinct immune‐related cell types estimated using quanTIseq. (A) Boxplots showing the proportion of 10 major immune‐related cells in LNmet and PT subregions. (B) Boxplots showing the proportion of 10 major immune‐related cells in LNmet and PT. For the boxplot, the centerline represents the median, and box limits represent upper and lower quartiles. Each dot represents a sample. Pairwise comparison was conducted using the Wilcox rank sum tests. [file CTM2-13-e1493-s010.jpg]

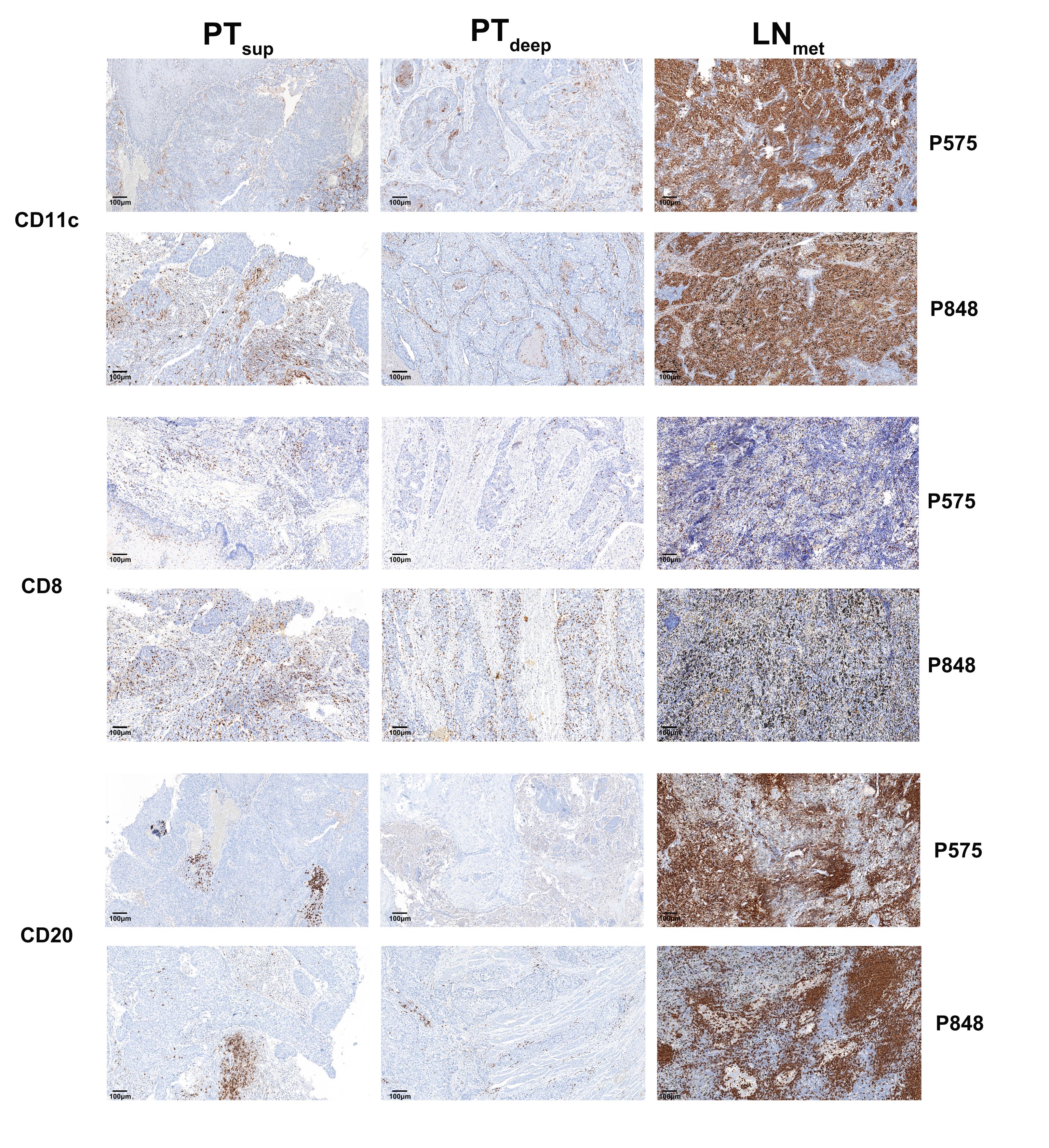

Supplement: Supplementary file 6 — Figure S6. Representative immunohistochemical images of CD11c, CD8 and CD20 expression in PTsup, PTdeep and LNmet subregions. [file CTM2-13-e1493-s022.jpg]

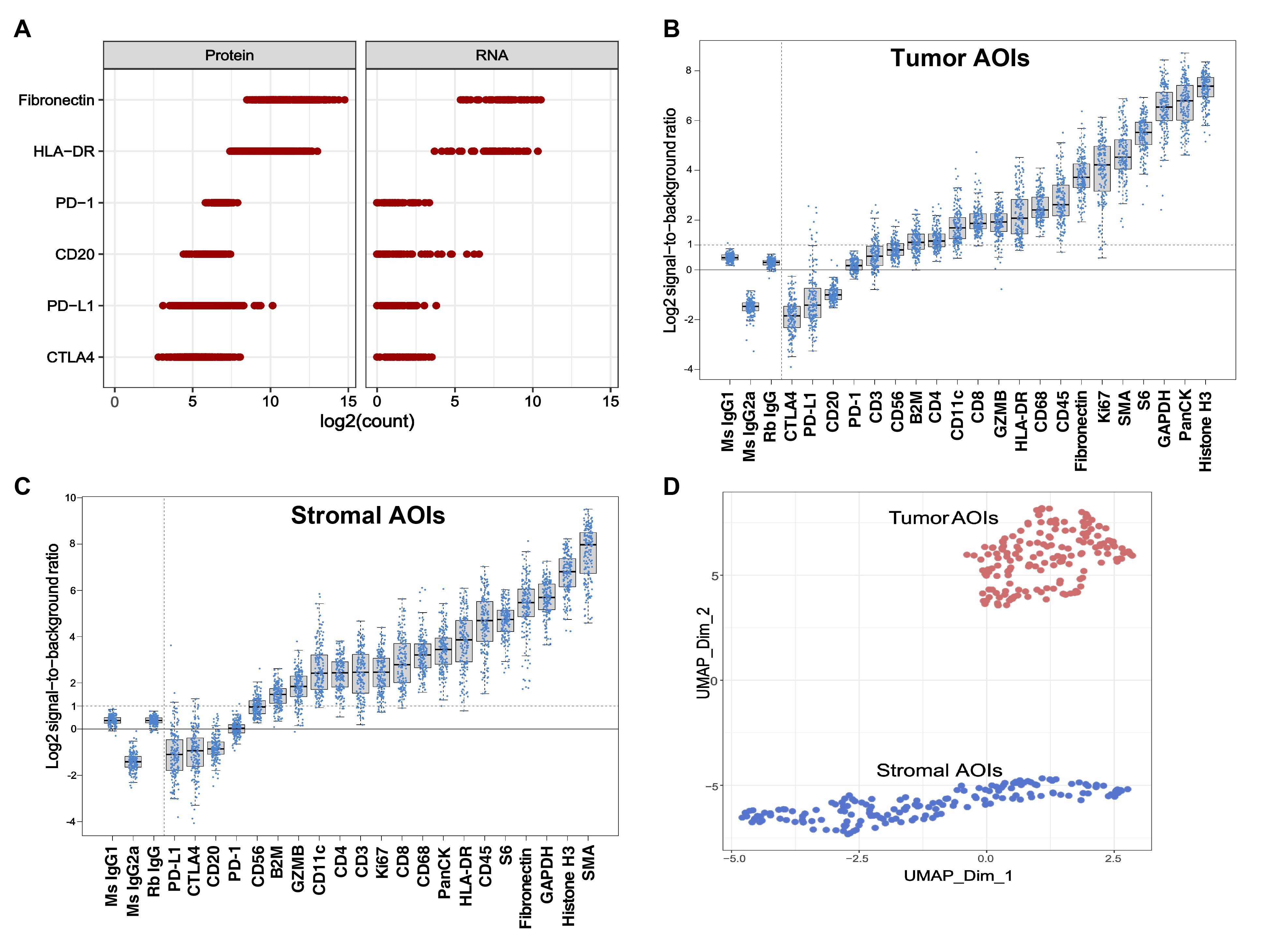

Supplement: Supplementary file 7 — Figure S7. Quality control of AOIs by DSP. (A) Expression is consistent across RNA and protein DSP in fibronectin, HLA‐DR, PD‐1, CD20, PD‐L1 and CTLA4. Data are presented as log2 normalised counts. (B and C) The quality control of the expression of each target protein in tumour (B) or stromal (C) compartment relative to negative control. Data are graphed as log2 SNR. Each dot represents an AOI. (D) UMAP dimensionality reduction analysis of AOIs at protein level. AOIs, areas of interest; DSP, digital spatial profiling; SNR, signal‐to‐noise ratio; UMAP, uniform manifold approximation and projection. [file CTM2-13-e1493-s001.jpg]

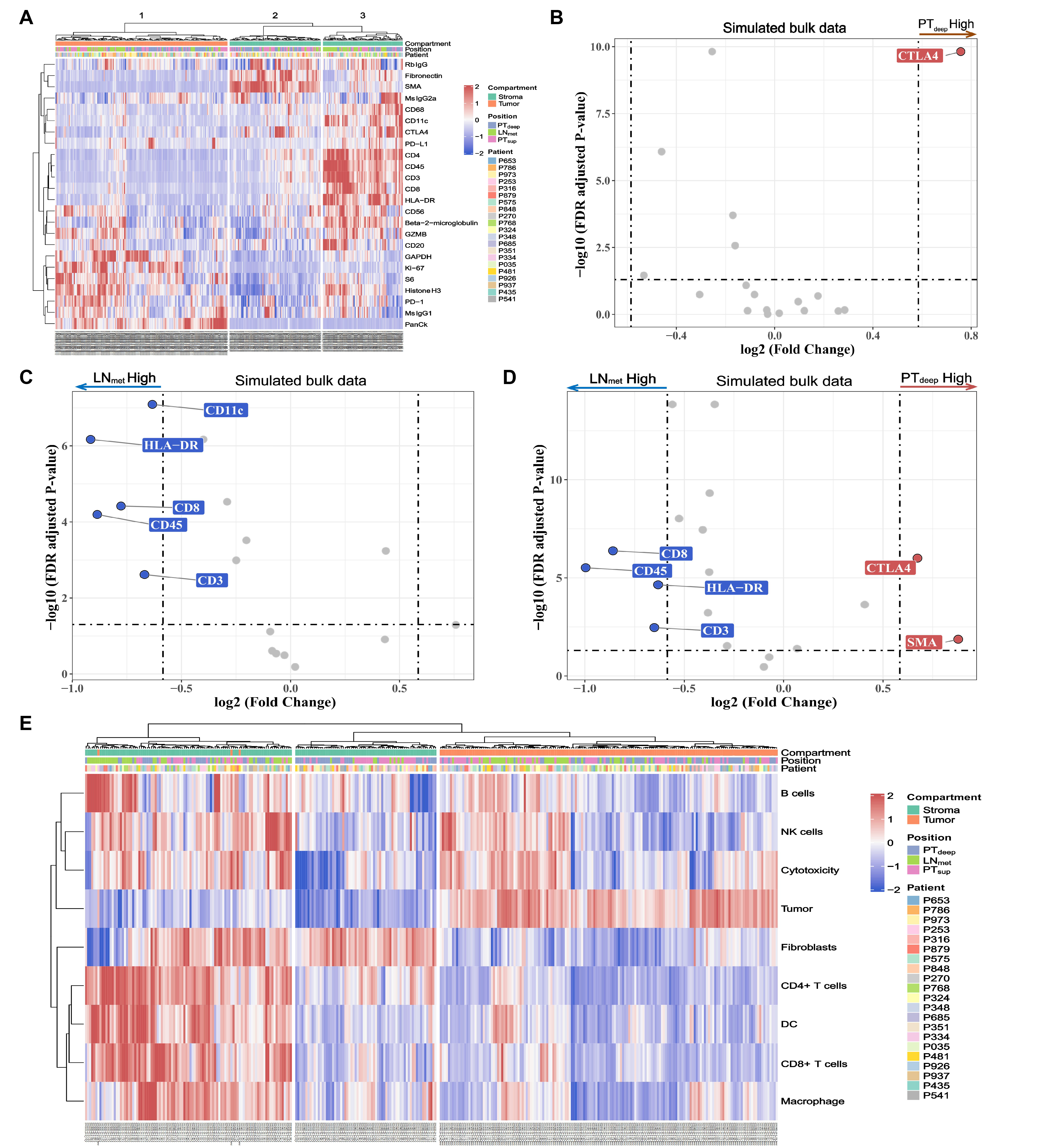

Supplement: Supplementary file 8 — Figure S8. The distinct protein expression patterns and abundance of immune cells in spatial AOIs identified by DSP. (A) Clustered heatmap showing the relative expression of proteins per AOI in stromal or tumour compartment of each subregion. Colour key: Colour gradient blue to red indicates relative expression levels from low to high. (B–D) Volcano plots showing differential expression of proteins based on comparison of PTsup versus PTdeep (B), LNmet versus PTsup (C) and LNmet versus PTdeep (D) through mimic bulk sequencing in the whole area. (E) Heatmap of the lymphocyte infiltration abundance estimated by protein expression in stromal or tumour compartment of each subregion. Colour key: Colour gradient blue to red indicates relative levels from low to high. AOIs, areas of interest; DSP, digital spatial profiling. [file CTM2-13-e1493-s028.jpg]

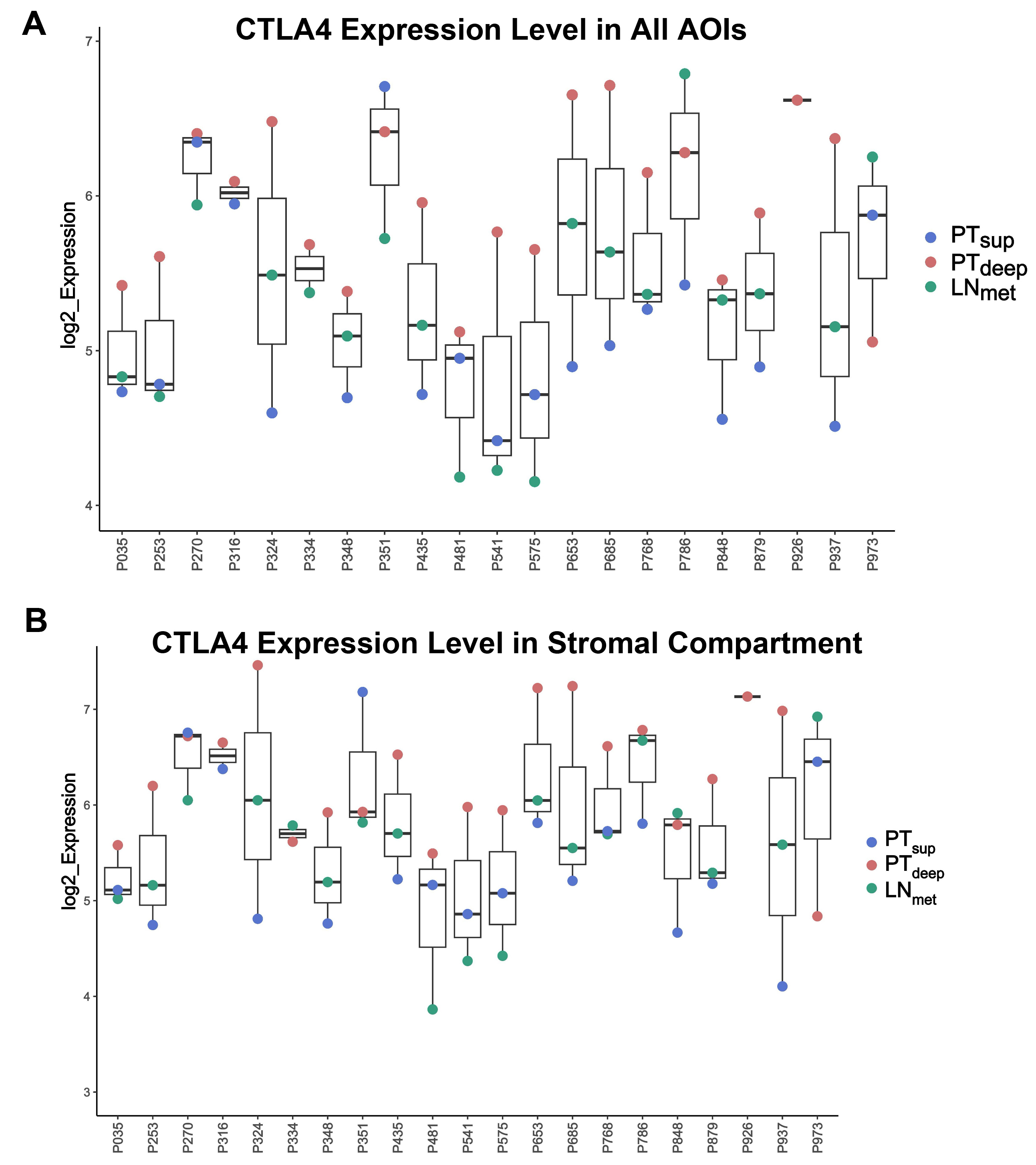

Supplement: Supplementary file 9 — Figure S9. The CTLA4 expression level in LNmet and PT subregions of each patient. Boxplots showing the CTLA4 expression level in all AOIs (A) and stromal compartment (B). [file CTM2-13-e1493-s019.jpg]

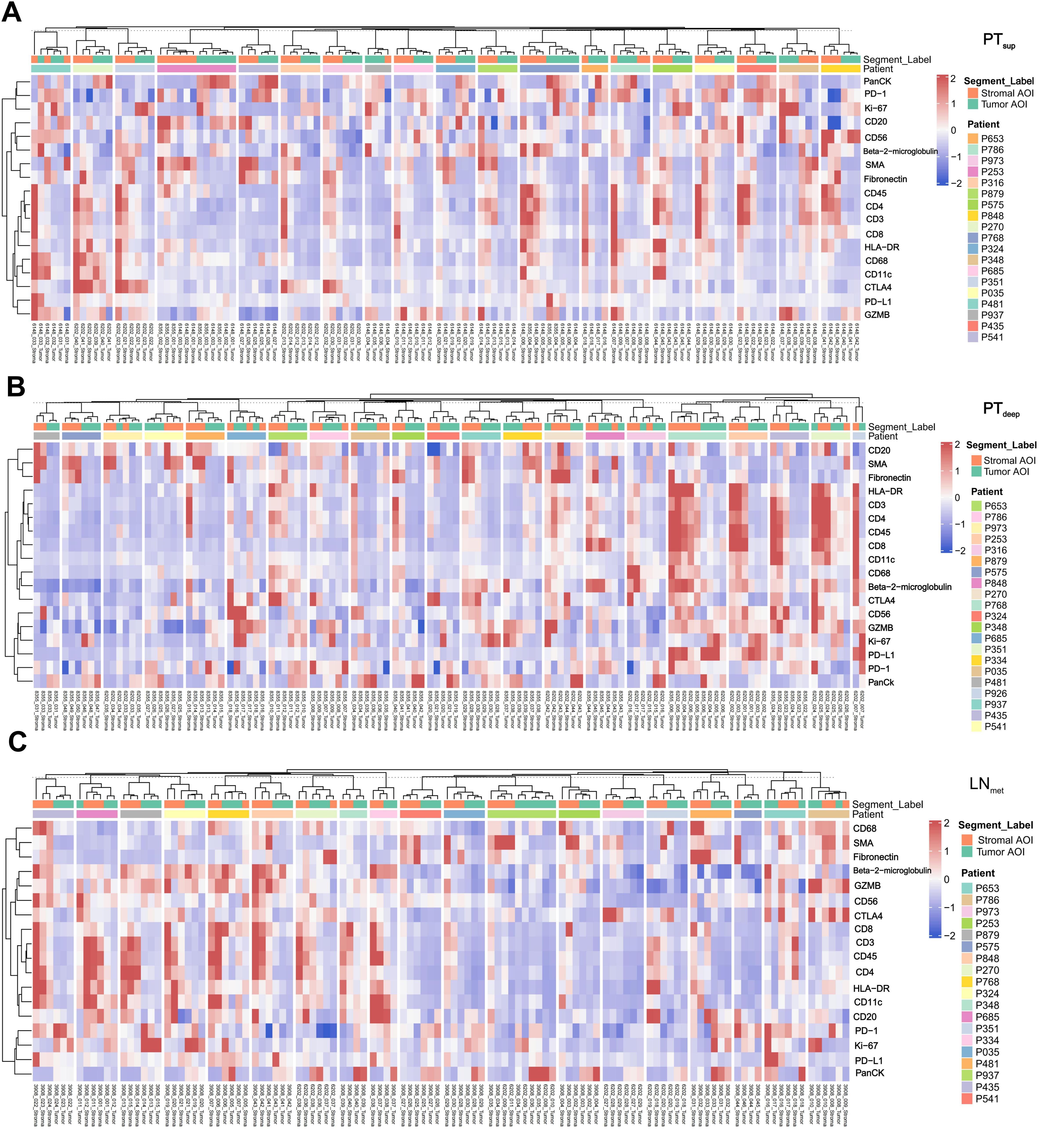

Supplement: Supplementary file 10 — Figure S10. Analysis of the intra‐AOI heterogeneity in protein levels. (A–C) Heatmaps of protein levels among AOIs in PTsup (A), PTdeep (B) and LNmet (C). [file CTM2-13-e1493-s018.jpg]

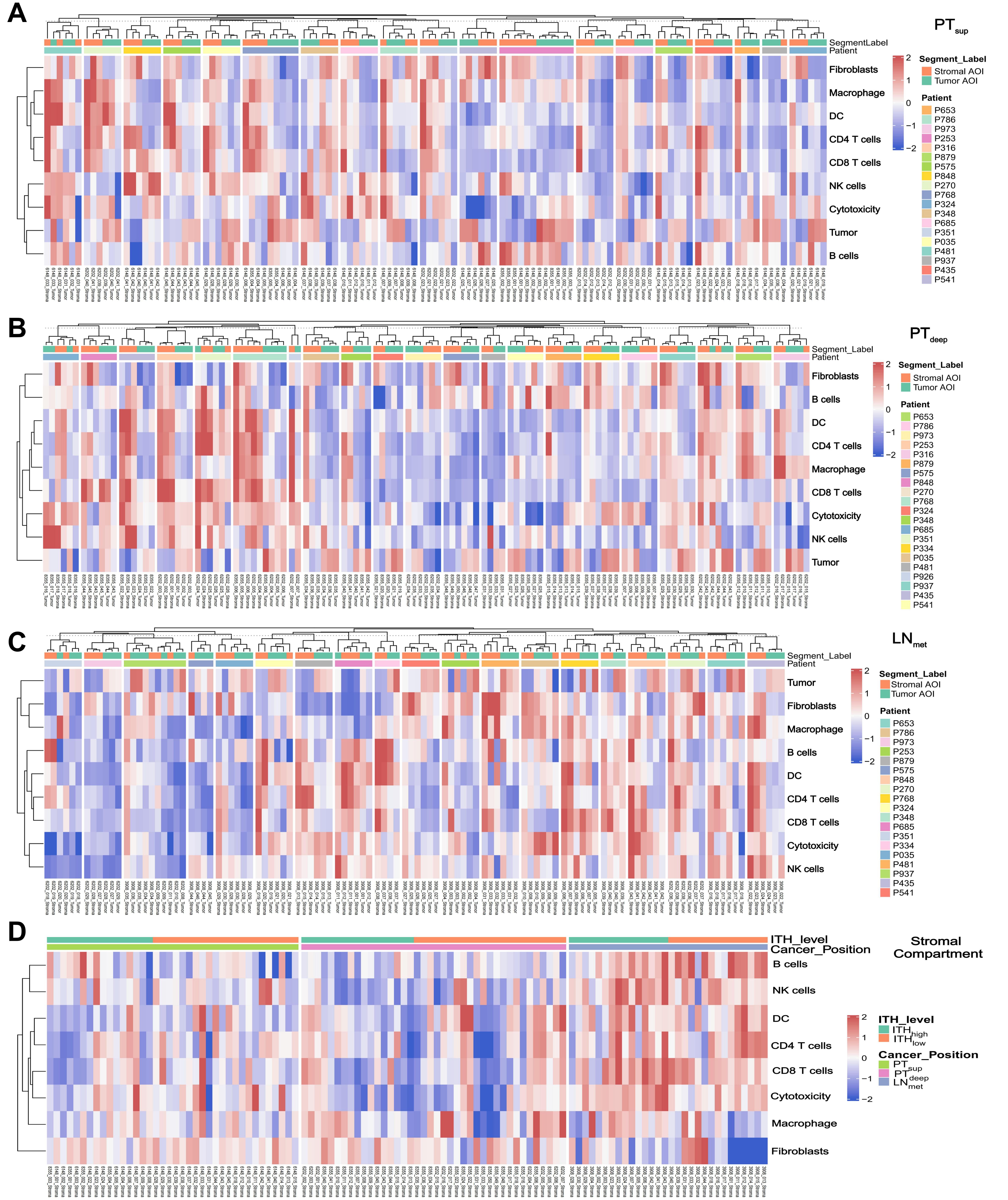

Supplement: Supplementary file 11 — Figure S11. Analysis of the intra‐AOI heterogeneity in immune cell abundance. (A–C) Heatmaps of immune cell abundance among AOIs in PTsup (A), PTdeep (B) and LNmet (C); (D) heatmap showing the association between genomic ITH levels and the lymphocyte infiltration abundance estimated by protein expression in stromal compartment of each subregion. [file CTM2-13-e1493-s008.jpg]
