# Supplementary material for: Multi‐omics analyses reveal spatial heterogeneity in primary and metastatic oesophageal squamous cell carcinoma
Source: Clin Transl Med. 2023 Nov 27;13(11):e1493. doi: 10.1002/ctm2.1493 (PMC10679972; doi:10.1002/ctm2.1493)
Supplement: Supplementary file 12 — Table S1. Detailed clinicopathological features of 21 patients with ESCC. [file CTM2-13-e1493-s011.docx]

**Supplementary Table 1. Detailed clinicopathological features of 21 patients with ESCC.**

| **Patient ID** | **Gender** | **Age (yr)** | **Tumor Location** | **TNM stage** | **IHC** | **Treatment** | **OS_Month** | **OS_Status** |
| --- | --- | --- | --- | --- | --- | --- | --- | --- |
| P035 | Female | 74 | Middle thoracic | T3N1M0 | P40(+), Ki-67(80%+), | Complete tumor resection and routine lymph node dissection | 29 | Alive |
| P316 | Female | 54 | Middle thoracic | T4aN1M0 | P40(+), Ki-67(60%+) | Complete tumor resection and routine lymph node dissection | 27 | Alive |
| P348 | Male | 65 | Middle thoracic | T4aN2M0 | P40(+), Ki-67(40%+) | Complete tumor resection and routine lymph node dissection | 33 | Alive |
| P435 | Male | 63 | Middle thoracic | T4aN2M0 | P40(+) | Complete tumor resection and routine lymph node dissection | 23 | Alive |
| P481 | Male | 68 | Upper thoracic | T4aN2M0 | P40(+) | Complete tumor resection and routine lymph node dissection | 1 | Dead |
| P575 | Male | 62 | Middle thoracic | T4aN3M0 | P40(+), Ki-67(70%+) | Complete tumor resection and routine lymph node dissection | 12 | Dead |
| P685 | Female | 70 | Middle thoracic | T4aN1M0 | P40(+), Ki-67(80%+) | Complete tumor resection and routine lymph node dissection | 8 | Dead |
| P879 | Male | 67 | Lower thoracic | T4aN2M0 | P40(+), Ki-67(60%+) | Complete tumor resection and routine lymph node dissection | 35 | Alive |
| P926 | Male | 73 | Middle thoracic | T2N1M0 | P40(+), Ki-67(70%+) | Complete tumor resection and routine lymph node dissection | 23 | Dead |
| P253 | Male | 71 | Middle thoracic | T3N2M0 | P40(+), Ki-67(50%+) | Complete tumor resection and routine lymph node dissection | 17 | Dead |
| P653 | Male | 64 | Middle thoracic | T3N3M0 | P40(+) | Complete tumor resection and routine lymph node dissection | NA | Lost |
| P768 | Male | 73 | Middle thoracic | T3N2M0 | P40(+), Ki-67(50%+) | Complete tumor resection and routine lymph node dissection | 26 | Dead |
| P786 | Male | 72 | Lower thoracic | T4aN2M0 | P40(+), Ki-67(70%+) | Complete tumor resection and routine lymph node dissection | 22 | Dead |
| P848 | Female | 73 | Upper thoracic | T3N2M0 | P40(+), Ki-67(80%+) | Complete tumor resection and routine lymph node dissection | 34 | Alive |
| P973 | Male | 68 | Middle thoracic | T3N2M0 | P40(+) | Complete tumor resection and routine lymph node dissection | 34 | Alive |
| P541 | Female | 41 | Middle thoracic | T3N2M0 | P40(+) | Complete tumor resection and routine lymph node dissection | 8 | Dead |
| P351 | Male | 53 | Lower thoracic | T3N1M0 | P40(+), Ki-67(30%+) | Complete tumor resection and routine lymph node dissection | 31 | Alive |
| P324 | Male | 80 | Middle thoracic | T3N1M0 | P40(+) | Complete tumor resection and routine lymph node dissection | 1 | Dead |
| P334 | Male | 57 | Lower thoracic | T3N1M0 | P40(+), Ki-67(40%+) | Complete tumor resection and routine lymph node dissection | 29 | Alive |
| P270 | Male | 72 | Middle thoracic | T3N1M0 | P40(+), Ki67(50%+) | Complete tumor resection and routine lymph node dissection | 12 | Dead |
| P937 | Male | 48 | Lower thoracic | T4aN1M0 | P40(+), Ki-67(70%+) | Complete tumor resection and routine lymph node dissection | 25 | Alive |
