# Supplementary material for: Multi‐omics analyses reveal spatial heterogeneity in primary and metastatic oesophageal squamous cell carcinoma
Source: Clin Transl Med. 2023 Nov 27;13(11):e1493. doi: 10.1002/ctm2.1493 (PMC10679972; doi:10.1002/ctm2.1493)
Supplement: Supplementary file 13 — Table S2. The quality control of WES, RNA‐seq and DSP data. [file CTM2-13-e1493-s027.docx]

**Supplementary Table 2. The quality control of WES, RNA-seq, and DSP data.**

| **The quality control of WES data** | | | | | | | | | | | | | | | | |
| --- | --- | --- | --- | --- | --- | --- | --- | --- | --- | --- | --- | --- | --- | --- | --- | --- |
| **Sample** | **Clean_Base** | **Q20** | **Q30** | **GC** | **GC-AT_Seperation** | **N_Rate** | **Average_read_length** | **Read_length_stddev** | **Average_base_quality** | **Mapping_rate** | **Mapping_quality** | **Insert_size** | **Duplication_rate** | **Capture_rate** | **Depth_in_target** | **Target_coverage** |
| P035-PTsup | 64269759124 | 98.70% | 95.46% | 53.59% | 0.339 | 0.001 | 99 | 2.689 | 36.539 | 99.99% | 53.512 | 162 | 29.18% | 84.24% | 620.114 | 99.78% |
| P035-PTdeep | 48967101462 | 98.74% | 95.58% | 55.15% | 0.363 | 0.001 | 99 | 2.855 | 36.566 | 99.99% | 53.423 | 158 | 25.30% | 84.91% | 505.354 | 99.77% |
| P035-LNmet | 68153182736 | 98.43% | 94.56% | 53.58% | 0.383 | 0.001 | 99 | 2.92 | 36.316 | 99.99% | 53.177 | 157 | 31.05% | 82.34% | 624.989 | 99.80% |
| P035-N | 19821728252 | 98.33% | 94.23% | 54.20% | 0.385 | 0.001 | 99 | 2.12 | 36.216 | 99.99% | 53.923 | 176 | 9.43% | 85.95% | 248.194 | 99.75% |
| P316-PTsup | 56488547938 | 99.11% | 96.82% | 54.02% | 0.271 | 0.001 | 99 | 4.254 | 36.928 | 100.00% | 53.809 | 138 | 38.65% | 88.59% | 499.448 | 99.95% |
| P316-PTdeep | 24306416352 | 96.86% | 90.11% | 54.61% | 0.413 | 0.006 | 99 | 2.866 | 34.981 | 99.88% | 53.493 | 147 | 21.11% | 87.56% | 270.2 | 99.94% |
| P316-LNmet | 22645466008 | 96.76% | 89.84% | 53.78% | 0.441 | 0.006 | 99 | 3.473 | 34.934 | 99.88% | 53.748 | 145 | 20.21% | 88.02% | 255.499 | 99.93% |
| P316-N | 29015033202 | 98.87% | 95.94% | 53.55% | 0.406 | 0.001 | 99 | 3.17 | 36.669 | 99.99% | 53.755 | 149 | 15.81% | 87.70% | 351.323 | 99.94% |
| P348-PTsup | 22826384710 | 96.47% | 89.13% | 56.71% | 0.476 | 0.006 | 99 | 2.483 | 34.774 | 99.83% | 52.533 | 157 | 16.76% | 83.51% | 254.373 | 99.93% |
| P348-PTdeep | 21017557654 | 96.21% | 88.95% | 55.34% | 2.76 | 0.017 | 99 | 2.932 | 34.709 | 99.76% | 51.4 | 148 | 18.70% | 80.50% | 220.405 | 99.93% |
| P348-LNmet | 1.04806E+11 | 98.69% | 95.65% | 56.43% | 0.236 | 0.001 | 99 | 4.053 | 36.641 | 99.99% | 52.095 | 143 | 51.51% | 84.06% | 670.299 | 99.95% |
| P348-N | 20888823944 | 98.70% | 95.51% | 53.33% | 0.386 | 0.001 | 99 | 2.719 | 36.579 | 99.99% | 53.7 | 161 | 10.07% | 86.67% | 264.648 | 99.92% |
| P435-PTsup | 70529513516 | 98.49% | 94.71% | 54.42% | 0.387 | 0.001 | 99 | 2.614 | 36.333 | 99.99% | 53.115 | 162 | 30.04% | 84.30% | 671.297 | 99.95% |
| P435-PTdeep | 74023318508 | 98.55% | 94.92% | 53.83% | 0.434 | 0.001 | 99 | 2.59 | 36.38 | 99.99% | 53.287 | 165 | 31.12% | 84.64% | 693.185 | 99.95% |
| P435-LNmet | 47648665992 | 98.87% | 96.13% | 56.35% | 0.354 | 0.001 | 99 | 3.344 | 36.754 | 100.00% | 52.766 | 148 | 31.71% | 86.51% | 458.685 | 99.94% |
| P435-N | 14677614356 | 98.55% | 94.88% | 54.11% | 0.434 | 0.001 | 99 | 2.112 | 36.368 | 99.99% | 53.758 | 172 | 6.94% | 86.31% | 190.528 | 99.92% |
| P481-PTsup | 33409791540 | 95.54% | 87.33% | 56.98% | 2.357 | 0.017 | 99 | 2.678 | 34.384 | 99.75% | 52.603 | 156 | 20.38% | 83.63% | 351.524 | 99.94% |
| P481-PTdeep | 19477580206 | 96.72% | 89.78% | 56.71% | 0.418 | 0.005 | 99 | 2.3 | 34.9 | 99.83% | 52.41 | 148 | 15.48% | 83.64% | 224.055 | 99.93% |
| P481-LNmet | 29905452696 | 96.25% | 88.64% | 57.06% | 0.426 | 0.006 | 99 | 2.363 | 34.672 | 99.84% | 53.194 | 154 | 17.82% | 85.50% | 338.039 | 99.94% |
| P481-N | 19573790664 | 98.38% | 94.29% | 53.50% | 0.444 | 0.001 | 99 | 1.892 | 36.207 | 99.99% | 53.779 | 185 | 9.49% | 84.12% | 238.609 | 99.93% |
| P575-PTsup | 59142438812 | 98.67% | 95.32% | 53.38% | 0.317 | 0.001 | 99 | 3.187 | 36.501 | 99.99% | 53.465 | 150 | 31.22% | 84.45% | 559.066 | 99.96% |
| P575-PTdeep | 29012741082 | 95.73% | 88.03% | 57.05% | 2.283 | 0.018 | 99 | 3.67 | 34.532 | 99.76% | 52.114 | 138 | 26.86% | 83.91% | 284.994 | 99.95% |
| P575-LNmet | 19014800212 | 96.77% | 90.08% | 55.05% | 0.346 | 0.006 | 99 | 3.172 | 34.971 | 99.82% | 52.539 | 144 | 22.04% | 84.00% | 200.966 | 99.94% |
| P575-N | 39147711564 | 98.27% | 94.02% | 56.25% | 0.43 | 0.001 | 99 | 2.322 | 36.168 | 99.99% | 53.507 | 168 | 13.64% | 85.32% | 466.566 | 99.95% |
| P685-PTsup | 46637765148 | 98.95% | 96.33% | 53.42% | 0.373 | 0.001 | 99 | 3.51 | 36.783 | 99.99% | 53.465 | 145 | 29.05% | 83.95% | 449.474 | 99.79% |
| P685-PTdeep | 36269964244 | 96.49% | 89.64% | 55.64% | 1.867 | 0.017 | 99 | 3.295 | 34.857 | 99.82% | 52.889 | 144 | 25.61% | 85.56% | 368.494 | 99.77% |
| P685-LNmet | 49200597316 | 95.69% | 87.73% | 55.17% | 1.909 | 0.017 | 98 | 4.92 | 34.466 | 99.79% | 50.687 | 122 | 45.17% | 80.66% | 335.578 | 99.78% |
| P685-N | 22617637324 | 98.23% | 94.04% | 53.60% | 0.418 | 0.001 | 99 | 2.574 | 36.187 | 99.99% | 53.196 | 159 | 14.87% | 85.66% | 268.479 | 99.76% |
| P879-PTsup | 26927334446 | 96.57% | 89.38% | 53.47% | 0.482 | 0.006 | 99 | 3.024 | 34.835 | 99.84% | 52.721 | 148 | 21.05% | 85.46% | 291.014 | 99.93% |
| P879-PTdeep | 51169854524 | 98.88% | 96.06% | 53.55% | 0.278 | 0.001 | 99 | 3.224 | 36.71 | 100.00% | 52.884 | 148 | 30.97% | 86.36% | 498.419 | 99.94% |
| P879-LNmet | 61763589940 | 99.01% | 96.58% | 53.97% | 0.39 | 0.001 | 99 | 4.376 | 36.874 | 100.00% | 52.655 | 137 | 41.79% | 85.99% | 499.89 | 99.94% |
| P879-N | 13035642850 | 98.70% | 95.54% | 54.67% | 0.379 | 0.001 | 99 | 2.865 | 36.607 | 99.99% | 53.463 | 152 | 8.26% | 87.31% | 171.849 | 99.91% |
| P926-PTsup | 64181151716 | 98.50% | 94.85% | 54.34% | 0.3 | 0.001 | 99 | 3.921 | 36.405 | 99.99% | 52.686 | 141 | 45.64% | 86.23% | 486.212 | 99.94% |
| P926-PTdeep | 50596739872 | 99.01% | 96.50% | 52.75% | 0.344 | 0.001 | 99 | 3.384 | 36.83 | 99.99% | 52.277 | 140 | 28.07% | 79.08% | 477.831 | 99.95% |
| P926-LNmet | 44906630358 | 98.96% | 96.45% | 54.08% | 0.233 | 0.001 | 99 | 4.152 | 36.878 | 100.00% | 53.129 | 140 | 29.80% | 85.86% | 446.387 | 99.94% |
| P926-N | 24831406818 | 98.75% | 95.64% | 52.10% | 0.411 | 0.001 | 99 | 3.133 | 36.616 | 99.99% | 53.886 | 152 | 13.63% | 87.63% | 307.499 | 99.93% |
| P253-LNmet | 60429968726 | 99.37% | 97.41% | 53.09% | 0.718 | 0.003 | 98 | 4.858 | 37.069 | 99.92% | 53.576 | 141 | 26.33% | 82.96% | 736.456 | 99.97% |
| P253-PTsup | 62328880084 | 99.29% | 97.09% | 52.63% | 0.845 | 0.003 | 99 | 4.178 | 36.956 | 99.94% | 53.3 | 147 | 27.96% | 82.62% | 738.25 | 99.98% |
| P253-PTdeep | 55970136433 | 99.04% | 96.26% | 52.47% | 0.822 | 0 | 98 | 4.445 | 36.715 | 99.89% | 53.632 | 148 | 21.85% | 81.98% | 710.957 | 99.97% |
| P253-N | 31487874167 | 99.18% | 96.71% | 51.87% | 0.719 | 0 | 99 | 3.97 | 36.834 | 99.89% | 53.904 | 150 | 15.12% | 82.82% | 441.357 | 99.97% |
| P653-PTsup | 58808425565 | 99.13% | 96.55% | 52.43% | 0.646 | 0.001 | 99 | 3.617 | 36.789 | 99.91% | 54.062 | 162 | 20.87% | 81.46% | 748.988 | 99.97% |
| P653-PTdeep | 52371841246 | 99.20% | 96.81% | 51.86% | 0.74 | 0.001 | 99 | 3.533 | 36.865 | 99.92% | 54.056 | 163 | 18.64% | 82.36% | 693.639 | 99.96% |
| P653-LNmet | 47249528393 | 99.17% | 96.70% | 50.23% | 0.874 | 0.001 | 99 | 3.583 | 36.816 | 99.95% | 54.369 | 161 | 19.01% | 83.09% | 625.048 | 99.96% |
| P653-N | 29626416228 | 99.13% | 96.52% | 51.83% | 0.814 | 0 | 99 | 3.548 | 36.775 | 99.92% | 53.915 | 161 | 12.10% | 83.15% | 429.697 | 99.96% |
| P768-PTsup | 44915733831 | 99.16% | 96.67% | 51.97% | 0.71 | 0.001 | 99 | 3.534 | 36.822 | 99.93% | 53.962 | 163 | 16.61% | 83.09% | 614.783 | 99.95% |
| P768-PTdeep | 62450106517 | 99.03% | 96.08% | 50.95% | 0.868 | 0 | 99 | 3.704 | 36.611 | 99.93% | 53.858 | 157 | 23.23% | 83.22% | 782.559 | 99.96% |
| P768-LNmet | 29265229036 | 99.15% | 96.59% | 51.14% | 0.882 | 0 | 99 | 3.662 | 36.78 | 99.95% | 53.895 | 158 | 12.79% | 83.77% | 423.055 | 99.94% |
| P768-N | 18069199425 | 99.22% | 96.89% | 51.52% | 0.821 | 0 | 99 | 3.899 | 36.898 | 99.94% | 53.793 | 152 | 10.31% | 84.34% | 273.669 | 99.94% |
| P786-PTsup | 68669608349 | 99.31% | 97.14% | 50.53% | 0.721 | 0.003 | 99 | 4.246 | 36.956 | 99.94% | 53.646 | 153 | 24.57% | 78.93% | 798.445 | 99.97% |
| P786-PTdeep | 32979997950 | 99.23% | 96.86% | 50.83% | 0.735 | 0 | 99 | 3.557 | 36.857 | 99.95% | 54.061 | 160 | 13.76% | 83.44% | 469.051 | 99.95% |
| P786-LNmet | 40946552665 | 99.19% | 96.79% | 52.77% | 0.817 | 0 | 99 | 3.83 | 36.875 | 99.94% | 54.051 | 151 | 18.59% | 85.00% | 564.366 | 99.97% |
| P786-N | 27935197738 | 99.24% | 96.94% | 50.91% | 0.674 | 0 | 99 | 3.924 | 36.901 | 99.94% | 54.132 | 154 | 12.14% | 84.20% | 410.102 | 99.97% |
| P848-PTsup | 87190041316 | 99.15% | 96.61% | 51.38% | 0.788 | 0 | 99 | 4.143 | 36.813 | 99.95% | 53.692 | 149 | 33.55% | 83.04% | 941.429 | 99.87% |
| P848-PTdeep | 58715385738 | 99.20% | 96.79% | 51.82% | 0.714 | 0 | 98 | 4.432 | 36.875 | 99.95% | 54.067 | 148 | 29.83% | 84.80% | 687.854 | 99.88% |
| P848-LNmet | 55794238496 | 99.17% | 96.67% | 52.70% | 0.736 | 0 | 98 | 4.416 | 36.825 | 99.95% | 54.122 | 143 | 29.57% | 85.72% | 666.086 | 99.83% |
| P848-N | 26411820228 | 99.28% | 97.10% | 51.55% | 0.539 | 0 | 98 | 4.496 | 36.969 | 99.92% | 54.744 | 147 | 23.49% | 85.82% | 342.49 | 99.84% |
| P973-LNmet | 72824601175 | 99.25% | 97.03% | 54.12% | 0.886 | 0 | 98 | 5.282 | 36.966 | 99.96% | 53.173 | 134 | 34.38% | 85.14% | 821.597 | 99.97% |
| P973-PTsup | 78384759220 | 99.28% | 97.04% | 51.56% | 0.678 | 0.003 | 99 | 4.034 | 36.918 | 99.94% | 53.299 | 154 | 25.95% | 78.63% | 894.694 | 99.98% |
| P973-PTdeep | 56132577167 | 99.24% | 96.92% | 52.98% | 0.78 | 0 | 98 | 4.43 | 36.883 | 99.92% | 53.726 | 146 | 26.76% | 84.49% | 690.129 | 99.98% |
| P973-N | 22118989680 | 99.36% | 97.39% | 52.14% | 0.615 | 0.003 | 98 | 4.383 | 37.029 | 99.94% | 53.183 | 143 | 16.01% | 82.46% | 309.379 | 99.97% |

| **The quality control of RNA-seq data** | | | | | | | | | | | | | | | | | | |
| --- | --- | --- | --- | --- | --- | --- | --- | --- | --- | --- | --- | --- | --- | --- | --- | --- | --- | --- |
| **Sample** | **Clean_Base** | **Q20** | **Q30** | **GC** | **GC-AT_**  **Seperation** | **Average_read_**  **length** | **Average_base_**  **quality** | **Mapping_rate** | **Duplication_rate** | **Insert_size** | **Mapping_quality** | **Aligned_gene** | **Exon_rate** | **Intron_rate** | **Intergenic_rate** | **FPKM(>0)** | **FPKM(>1)** | **MT_rate** |
| P035-PTsup | 30959273316 | 99.14% | 97.04% | 54.03% | 0.406 | 98 | 37.038 | 95.99% | 58.74% | 146 | 26.991 | 95,295,907 | 37.23% | 50.26% | 12.51% | 33050 | 18087 | 1.08 |
| P035-PTdeep | 18540277428 | 98.92% | 96.41% | 51.65% | 0.236 | 99 | 36.86 | 93.06% | 39.15% | 143 | 37.58 | 44,006,394 | 29.67% | 56.86% | 13.47% | 31646 | 18695 | 0.75 |
| P035-LNmet | 17291974022 | 98.57% | 95.31% | 46.70% | 0.569 | 99 | 36.601 | 91.20% | 51.92% | 154 | 39.969 | 29,344,302 | 21.27% | 62.75% | 15.98% | 30991 | 18731 | 0.63 |
| P316-PTdeep | 14103178200 | 98.04% | 93.81% | 53.38% | 0.338 | 100 | 36.07 | 87.06% | 78.47% | 134 | 20.539 | 25,077,776 | 26.75% | 53.18% | 20.07% | 18543 | 15697 | 0.14 |
| P316-LNmet | 13052588200 | 97.83% | 93.13% | 55.11% | 0.27 | 100 | 35.905 | 76.12% | 71.74% | 128 | 26.381 | 13,523,194 | 20.14% | 52.88% | 26.98% | 15168 | 13606 | 0.13 |
| P348-PTsup | 42052066432 | 96.56% | 89.39% | 53.92% | 0.448 | 99 | 34.923 | 92.55% | 62.81% | 158 | 30.436 | 112,199,838 | 33.30% | 52.63% | 14.07% | 32912 | 18289 | 0.29 |
| P348-PTdeep | 38197923410 | 97.75% | 92.92% | 53.33% | 0.303 | 99 | 35.833 | 93.87% | 85.60% | 149 | 25.036 | 86,013,438 | 28.98% | 57.93% | 13.09% | 30249 | 19406 | 0.23 |
| P348-LNmet | 17131949798 | 98.40% | 95.18% | 56.10% | 0.548 | 98 | 36.627 | 92.51% | 83.96% | 128 | 21.328 | 27,453,571 | 23.12% | 60.47% | 16.40% | 29004 | 21259 | 0.6 |
| P435-PTsup | 18416640766 | 98.71% | 95.88% | 49.99% | 0.281 | 98 | 36.749 | 88.54% | 44.83% | 138 | 37.661 | 36,489,179 | 26.05% | 59.26% | 14.69% | 31821 | 19285 | 1.06 |
| P435-PTdeep | 22445249176 | 99.00% | 96.50% | 46.56% | 0.83 | 99 | 36.827 | 96.55% | 31.35% | 156 | 43.012 | 47,863,254 | 23.98% | 63.49% | 12.53% | 33942 | 20626 | 1.83 |
| P435-LNmet | 13529721394 | 98.63% | 95.70% | 48.84% | 0.211 | 98 | 36.72 | 91.05% | 86.52% | 138 | 24.139 | 23,254,872 | 22.84% | 57.79% | 19.38% | 23219 | 17076 | 0.44 |
| P481-PTsup | 59793745250 | 98.75% | 95.95% | 51.67% | 0.61 | 99 | 36.75 | 95.73% | 54.63% | 152 | 28.203 | 196,298,806 | 38.55% | 48.24% | 13.21% | 35802 | 18382 | 2.36 |
| P481-PTdeep | 15995574558 | 98.91% | 96.37% | 51.71% | 0.657 | 99 | 36.841 | 95.16% | 33.82% | 154 | 33.488 | 46,035,148 | 35.02% | 51.18% | 13.79% | 31352 | 17756 | 1.09 |
| P481-LNmet | 25183610040 | 98.23% | 94.52% | 54.32% | 0.752 | 98 | 36.357 | 90.63% | 58.72% | 150 | 31.601 | 58,545,610 | 31.61% | 53.91% | 14.48% | 32094 | 19273 | 1.44 |
| P575-PTsup | 60570311108 | 96.38% | 89.01% | 48.68% | 1.546 | 99 | 34.804 | 90.29% | 77.14% | 136 | 23.887 | 146,415,439 | 31.04% | 54.10% | 14.86% | 31385 | 17965 | 0.81 |
| P575-PTdeep | 12878206468 | 98.67% | 95.76% | 48.64% | 0.2 | 99 | 36.669 | 95.40% | 85.72% | 143 | 26.325 | 27,093,785 | 25.08% | 59.28% | 15.64% | 26253 | 19535 | 0.43 |
| P575-LNmet | 17805129870 | 99.02% | 96.71% | 51.79% | 0.32 | 99 | 36.905 | 90.02% | 88.28% | 148 | 23.727 | 49,602,674 | 36.20% | 49.42% | 14.38% | 26398 | 17678 | 0.22 |
| P685-PTsup | 18299967900 | 98.78% | 96.00% | 45.91% | 0.335 | 98 | 36.76 | 92.13% | 86.50% | 145 | 25.238 | 43,392,837 | 28.07% | 58.86% | 13.07% | 26151 | 18784 | 0.71 |
| P685-PTdeep | 21148196994 | 98.50% | 95.18% | 52.51% | 0.509 | 98 | 36.548 | 94.73% | 87.87% | 138 | 20.837 | 52,397,580 | 31.13% | 55.43% | 13.44% | 26763 | 19012 | 0.32 |
| P685-LNmet | 46231494700 | 98.87% | 96.29% | 48.42% | 0.053 | 98 | 36.823 | 87.86% | 82.92% | 134 | 24.461 | 69,390,513 | 19.13% | 65.15% | 15.72% | 24931 | 20402 | 0.32 |
| P879-PTsup | 12194426262 | 98.79% | 96.09% | 55.45% | 0.25 | 98 | 36.789 | 86% | 85.48% | 130 | 21.84 | 18,505,604 | 24.42% | 55.50% | 20.08% | 20352 | 16534 | 0.28 |
| P879-PTdeep | 22712960400 | 97.36% | 91.85% | 49.04% | 0.182 | 100 | 35.592 | 89.93% | 81.30% | 132 | 21.515 | 37,105,018 | 21.48% | 62.55% | 15.97% | 23764 | 19576 | 0.35 |
| P879-LNmet | 11964278148 | 98.48% | 95.16% | 52.07% | 0.112 | 98 | 36.572 | 82.83% | 83.65% | 128 | 21.669 | 20,508,552 | 26.57% | 55.54% | 17.89% | 21502 | 17927 | 5.3 |
| P926-PTsup | 14127872432 | 98.19% | 94.43% | 49.31% | 0.489 | 99 | 36.362 | 89.49% | 83.52% | 142 | 25.285 | 26,178,662 | 25.20% | 56.94% | 17.86% | 24257 | 18584 | 0.59 |
| P926-PTdeep | 31299596626 | 98.76% | 95.85% | 46.75% | 0.753 | 99 | 36.731 | 96.83% | 33% | 152 | 38.932 | 76,629,555 | 27.62% | 57.32% | 15.06% | 36213 | 20763 | 2.77 |
| P926-LNmet | 17543645616 | 98.49% | 95.17% | 47.71% | 0.425 | 99 | 36.568 | 85.24% | 62.03% | 138 | 36.402 | 28,041,773 | 21.42% | 59.94% | 18.64% | 31001 | 19769 | 0.71 |
| P253-PTsup | 14299876676 | 98.82% | 96.17% | 51.20% | 0.352 | 98 | 36.822 | 93.52% | 84.87% | 146 | 23.139 | 36,740,959 | 33.24% | 52.56% | 14.20% | 25514 | 17310 | 0.86 |
| P253-PTdeep | 24366744884 | 98.50% | 95.27% | 55.87% | 0.216 | 98 | 36.54 | 92.95% | 78.89% | 141 | 23.384 | 60,493,431 | 35.55% | 48.76% | 15.69% | 28939 | 16862 | 0.54 |
| P253-LNmet | 33340166294 | 98.60% | 95.66% | 49.55% | 1.775 | 99 | 36.702 | 85.69% | 82.95% | 141 | 23.93 | 72,961,704 | 32.15% | 50.19% | 17.65% | 31365 | 18050 | 0.81 |
| P653-PTsup | 18756581640 | 98.53% | 95.36% | 58.14% | 0.216 | 98 | 36.582 | 95.75% | 35.46% | 139 | 36.1 | 43,672,125 | 31.37% | 54.01% | 14.62% | 33251 | 19980 | 1.02 |
| P653-PTdeep | 19567859770 | 98.15% | 94.21% | 58.82% | 0.237 | 99 | 36.226 | 94.89% | 40.42% | 150 | 33.61 | 49,452,468 | 35.46% | 50.58% | 13.96% | 31825 | 18319 | 0.69 |
| P653-LNmet | 18930105276 | 98.47% | 94.99% | 45.42% | 0.201 | 98 | 36.486 | 93.52% | 76.56% | 143 | 29.045 | 36,245,782 | 22.87% | 62.22% | 14.90% | 29853 | 20405 | 2 |
| P768-PTsup | 16326878180 | 98.49% | 95.30% | 56.34% | 0.256 | 98 | 36.547 | 95.67% | 34.68% | 142 | 35.663 | 43,134,156 | 34.04% | 50.55% | 15.41% | 31828 | 18646 | 0.97 |
| P768-PTdeep | 17503838840 | 98.93% | 96.29% | 49.97% | 1.035 | 99 | 36.812 | 96.37% | 66.56% | 158 | 35.182 | 36,717,024 | 25.39% | 60.89% | 13.72% | 29679 | 18979 | 0.71 |
| P768-LNmet | 18926828746 | 99.05% | 96.80% | 52.05% | 0.702 | 98 | 36.985 | 94.37% | 66.23% | 134 | 32.863 | 41,607,304 | 27.58% | 58.10% | 14.32% | 30846 | 19773 | 0.46 |
| P786-PTsup | 15433962534 | 99.04% | 96.71% | 50.01% | 0.78 | 99 | 36.952 | 95.85% | 77.54% | 145 | 27.739 | 37,465,918 | 29.34% | 55.51% | 15.14% | 28350 | 18823 | 0.68 |
| P786-PTdeep | 16233311206 | 98.52% | 95.23% | 53.05% | 0.186 | 99 | 36.502 | 95.01% | 66.02% | 154 | 30.894 | 31,961,784 | 25.89% | 55.85% | 18.27% | 29258 | 18874 | 0.63 |
| P848-PTsup | 13931883042 | 99.07% | 96.95% | 55.29% | 0.19 | 98 | 37.028 | 88.93% | 36.40% | 132 | 34.071 | 27,250,676 | 29.31% | 51.81% | 18.87% | 31668 | 18140 | 1.34 |
| P848-PTdeep | 14603001346 | 98.93% | 96.64% | 56.12% | 0.925 | 98 | 36.974 | 95.15% | 73.51% | 129 | 25.577 | 31,995,671 | 30.51% | 54.55% | 14.94% | 29810 | 19811 | 1.17 |
| P848-LNmet | 36371510876 | 98.48% | 95.39% | 55.57% | 2.168 | 99 | 36.623 | 80.50% | 72.96% | 132 | 22.175 | 59,509,151 | 28.53% | 54.86% | 16.61% | 32971 | 20075 | 0.99 |
| P541-PTsup | 11666232306 | 97.19% | 91.96% | 59.21% | 0.323 | 99 | 35.68 | 92.19% | 30.44% | 149 | 33.248 | 28,678,289 | 39.72% | 42.48% | 17.79% | 29062 | 16248 | 1.35 |
| P541-PTdeep | 21829075612 | 98.95% | 96.63% | 56.62% | 0.54 | 97 | 36.953 | 85.23% | 37.38% | 129 | 30.881 | 48,701,599 | 34.97% | 47.03% | 18% | 31568 | 16983 | 1.58 |
| P541-LNmet | 17659870046 | 99.01% | 96.67% | 50.88% | 0.407 | 98 | 36.914 | 95.76% | 32.73% | 140 | 37.657 | 45,529,383 | 31.08% | 49.16% | 19.77% | 32741 | 18610 | 2.03 |
| P351-PTsup | 17024061424 | 98.74% | 96.01% | 54.47% | 0.334 | 99 | 36.791 | 93.68% | 77.12% | 145 | 27.553 | 31,719,553 | 27.19% | 57.10% | 15.71% | 29084 | 18680 | 0.51 |
| P351-PTdeep | 13229832416 | 98.64% | 95.53% | 50.27% | 0.518 | 99 | 36.568 | 93.48% | 87.43% | 148 | 24.264 | 22,173,346 | 21.82% | 62.11% | 16.07% | 24927 | 18814 | 0.42 |
| P351-LNmet | 13397502674 | 98.70% | 95.64% | 48.89% | 0.358 | 99 | 36.617 | 88.41% | 85.66% | 146 | 26.245 | 18,864,592 | 19.16% | 64.74% | 16.11% | 22667 | 18364 | 0.15 |
| P324-PTsup | 38313299152 | 96.91% | 90.29% | 49.28% | 1.228 | 99 | 35.074 | 73.03% | 77.91% | 129 | 17.472 | 40,757,129 | 18.80% | 60.82% | 20.38% | 24040 | 17123 | 1.16 |
| P324-PTdeep | 17625707818 | 98.37% | 94.73% | 49.61% | 0.218 | 99 | 36.351 | 83.72% | 70.77% | 129 | 28.706 | 26,248,451 | 23.06% | 54.64% | 22.30% | 16037 | 14499 | 0.37 |
| P334-PTsup | 32015124942 | 96.68% | 89.99% | 50.01% | 1.746 | 99 | 35.056 | 81.88% | 64.59% | 124 | 21.777 | 39,204,969 | 18.82% | 63.40% | 17.78% | 21209 | 18674 | 0.16 |
| P334-PTdeep | 12366623942 | 98.58% | 95.48% | 51.27% | 0.229 | 97 | 36.652 | 81.41% | 75.39% | 125 | 29.719 | 17,527,552 | 23.83% | 55.51% | 20.66% | 15804 | 13887 | 0.19 |
| P334-LNmet | 19474385530 | 99.02% | 96.69% | 47.98% | 0.823 | 98 | 36.951 | 93.89% | 84.81% | 128 | 26.054 | 34,813,176 | 22.31% | 62.77% | 14.91% | 29075 | 20429 | 0.55 |
| P270-PTdeep | 12762230622 | 98.79% | 95.97% | 52.91% | 1.132 | 99 | 36.744 | 96.61% | 58.47% | 158 | 34.069 | 24,194,586 | 24.51% | 60.66% | 14.82% | 29350 | 19533 | 0.74 |
| P270-LNmet | 20588283956 | 98.75% | 95.83% | 53.07% | 0.321 | 98 | 36.662 | 95.87% | 32.66% | 150 | 36.531 | 55,713,566 | 33.11% | 53.04% | 13.85% | 34235 | 20052 | 0.91 |

| **The quality control of DSP data** | | | | | | | | |
| --- | --- | --- | --- | --- | --- | --- | --- | --- |
| **Patient_No.** | **Cancer_Position** | **ROI_Name** | **Segment_Name** | **QC_Status** | **Binding_Density** | **FOV_Detection_Percentage** | **Surface_Area** | **Nuclei_Count** |
| P879 | PTdeep | 13 | Stroma | PASSED | 0.52 | 0.939285714 | 8111.177153 | 68 |
| P324 | PTdeep | 19 | Stroma | PASSED | 0.52 | 0.939285714 | 23935.43198 | 326 |
| P541 | PTdeep | 25 | Stroma | PASSED | 0.52 | 0.939285714 | 33584.75702 | 522 |
| P481 | PTdeep | 31 | Stroma | PASSED | 0.52 | 0.939285714 | 33777.66374 | 209 |
| P334 | PTdeep | 37 | Stroma | PASSED | 0.52 | 0.939285714 | 18684.18872 | 153 |
| P848 | PTdeep | 43 | Stroma | PASSED | 0.52 | 0.939285714 | 37280.30091 | 186 |
| P879 | PTdeep | 13 | Tumor | PASSED | 0.44 | 0.942857143 | 25324.07317 | 124 |
| P324 | LNmet | 19 | Tumor | PASSED | 0.44 | 0.942857143 | 36700.6234 | 486 |
| P541 | PTdeep | 25 | Tumor | PASSED | 0.44 | 0.942857143 | 20284.56458 | 124 |
| P481 | PTdeep | 31 | Tumor | PASSED | 0.44 | 0.942857143 | 21365.73575 | 126 |
| P334 | PTdeep | 37 | Tumor | PASSED | 0.44 | 0.942857143 | 24573.18894 | 156 |
| P848 | PTdeep | 43 | Tumor | PASSED | 0.44 | 0.942857143 | 27253.29994 | 106 |
| P768 | PTdeep | 6 | Tumor | PASSED | 0.63 | 0.953571429 | 29930.53888 | 226 |
| P653 | PTdeep | 10 | Stroma | PASSED | 0.63 | 0.953571429 | 6055.579724 | 50 |
| P685 | PTdeep | 16 | Stroma | PASSED | 0.63 | 0.953571429 | 15339.83398 | 87 |
| P435 | PTdeep | 23 | Tumor | PASSED | 0.63 | 0.953571429 | 19410.97955 | 148 |
| P937 | PTdeep | 29 | Tumor | PASSED | 0.63 | 0.953571429 | 24752.21404 | 177 |
| P035 | PTdeep | 35 | Stroma | PASSED | 0.63 | 0.953571429 | 16693.05309 | 105 |
| P348 | PTdeep | 41 | Stroma | PASSED | 0.63 | 0.953571429 | 12589.51706 | 94 |
| P575 | PTdeep | 49 | Stroma | PASSED | 0.63 | 0.953571429 | 17526.11015 | 72 |
| P768 | PTdeep | 5 | Stroma | PASSED | 0.74 | 0.960714286 | 27023.85423 | 179 |
| P786 | PTdeep | 7 | Stroma | PASSED | 0.47 | 0.960714286 | 7589.100401 | 67 |
| P653 | PTdeep | 10 | Tumor | PASSED | 0.74 | 0.960714286 | 39408.65721 | 154 |
| P879 | PTdeep | 15 | Tumor | PASSED | 0.47 | 0.960714286 | 32195.63715 | 57 |
| P685 | PTdeep | 16 | Tumor | PASSED | 0.74 | 0.960714286 | 13966.67001 | 111 |
| P324 | PTdeep | 21 | Tumor | PASSED | 0.47 | 0.960714286 | 25479.16443 | 165 |
| P435 | PTdeep | 22 | Stroma | PASSED | 0.74 | 0.960714286 | 7494.801086 | 69 |
| P541 | PTdeep | 27 | Tumor | PASSED | 0.47 | 0.960714286 | 54392.67482 | 400 |
| P937 | PTdeep | 28 | Stroma | PASSED | 0.74 | 0.960714286 | 8002.517535 | 39 |
| P481 | PTdeep | 33 | Tumor | PASSED | 0.47 | 0.960714286 | 18247.95466 | 95 |
| P035 | PTdeep | 35 | Tumor | PASSED | 0.74 | 0.960714286 | 43546.65796 | 214 |
| P334 | PTdeep | 39 | Tumor | PASSED | 0.47 | 0.960714286 | 42128.33881 | 95 |
| P348 | PTdeep | 41 | Tumor | PASSED | 0.74 | 0.960714286 | 33572.94966 | 175 |
| P575 | PTdeep | 48 | Stroma | PASSED | 0.74 | 0.960714286 | 3979.558756 | 44 |
| P768 | PTsup | 4 | Stroma | PASSED | 0.65 | 0.964285714 | 9506.998491 | 116 |
| P768 | PTsup | 5 | Tumor | PASSED | 0.84 | 0.964285714 | 34903.83052 | 97 |
| P768 | PTdeep | 6 | Tumor | PASSED | 0.65 | 0.964285714 | 29009.40529 | 238 |
| P786 | PTdeep | 8 | Tumor | PASSED | 0.43 | 0.964285714 | 70490.0945 | 263 |
| P786 | PTdeep | 9 | Stroma | PASSED | 0.84 | 0.964285714 | 15350.20531 | 117 |
| P653 | PTdeep | 12 | Tumor | PASSED | 0.65 | 0.964285714 | 31402.46982 | 195 |
| P879 | PTdeep | 15 | Stroma | PASSED | 0.43 | 0.964285714 | 11757.89602 | 87 |
| P879 | LNmet | 15 | Stroma | PASSED | 0.84 | 0.964285714 | 13072.66144 | 134 |
| P324 | LNmet | 19 | Tumor | PASSED | 0.65 | 0.964285714 | 50134.20709 | 192 |
| P324 | PTdeep | 21 | Stroma | PASSED | 0.43 | 0.964285714 | 33305.05025 | 189 |
| P435 | PTdeep | 22 | Tumor | PASSED | 0.84 | 0.964285714 | 32615.27708 | 262 |
| P541 | LNmet | 25 | Tumor | PASSED | 0.65 | 0.964285714 | 44526.03054 | 168 |
| P541 | PTdeep | 27 | Stroma | PASSED | 0.43 | 0.964285714 | 5232.415312 | 76 |
| P937 | PTdeep | 28 | Tumor | PASSED | 0.84 | 0.964285714 | 37868.91373 | 287 |
| P481 | LNmet | 31 | Tumor | PASSED | 0.65 | 0.964285714 | 37197.80896 | 327 |
| P481 | PTdeep | 33 | Stroma | PASSED | 0.43 | 0.964285714 | 38392.26695 | 284 |
| P035 | PTdeep | 34 | Stroma | PASSED | 0.84 | 0.964285714 | 25131.00689 | 224 |
| P334 | PTdeep | 38 | Tumor | PASSED | 0.65 | 0.964285714 | 31539.2118 | 263 |
| P334 | PTdeep | 39 | Stroma | PASSED | 0.43 | 0.964285714 | 24518.61979 | 169 |
| P348 | PTdeep | 40 | Stroma | PASSED | 0.84 | 0.964285714 | 9932.86122 | 98 |
| P848 | PTdeep | 44 | Tumor | PASSED | 0.65 | 0.964285714 | 38313.28529 | 256 |
| P575 | PTdeep | 48 | Tumor | PASSED | 0.84 | 0.964285714 | 36369.69822 | 323 |
| P768 | LNmet | 6 | Stroma | PASSED | 0.85 | 0.971428571 | 37369.49435 | 329 |
| P653 | PTdeep | 11 | Tumor | PASSED | 0.85 | 0.971428571 | 34390.84863 | 114 |
| P685 | PTdeep | 17 | Tumor | PASSED | 0.85 | 0.971428571 | 16969.56868 | 148 |
| P435 | PTdeep | 23 | Stroma | PASSED | 0.85 | 0.971428571 | 37979.80717 | 218 |
| P937 | PTdeep | 29 | Stroma | PASSED | 0.85 | 0.971428571 | 7603.141585 | 41 |
| P035 | PTdeep | 36 | Tumor | PASSED | 0.85 | 0.971428571 | 36820.45214 | 171 |
| P348 | PTdeep | 42 | Tumor | PASSED | 0.85 | 0.971428571 | 38373.75812 | 58 |
| P575 | PTdeep | 50 | Stroma | PASSED | 0.85 | 0.971428571 | 17526.11015 | 64 |
| P253 | PTsup | 3 | Stroma | PASSED | 0.8 | 0.975 | 40169.11497 | 325 |
| P786 | PTdeep | 8 | Tumor | PASSED | 0.8 | 0.975 | 22071.14568 | 96 |
| P653 | PTdeep | 12 | Stroma | PASSED | 0.5 | 0.975 | 25277.32241 | 123 |
| P879 | PTdeep | 14 | Tumor | PASSED | 0.8 | 0.975 | 26186.96775 | 172 |
| P685 | PTdeep | 18 | Stroma | PASSED | 0.5 | 0.975 | 33871.64394 | 542 |
| P324 | PTdeep | 20 | Stroma | PASSED | 0.8 | 0.975 | 5047.486536 | 52 |
| P435 | PTdeep | 24 | Stroma | PASSED | 0.5 | 0.975 | 25728.39544 | 294 |
| P541 | PTdeep | 26 | Stroma | PASSED | 0.8 | 0.975 | 10739.43151 | 64 |
| P937 | PTdeep | 30 | Stroma | PASSED | 0.5 | 0.975 | 17417.13142 | 167 |
| P481 | LNmet | 33 | Tumor | PASSED | 0.8 | 0.975 | 32857.64707 | 265 |
| P035 | PTdeep | 36 | Stroma | PASSED | 0.5 | 0.975 | 24546.38304 | 291 |
| P348 | LNmet | 39 | Tumor | PASSED | 0.8 | 0.975 | 35088.12106 | 230 |
| P848 | LNmet | 42 | Stroma | PASSED | 0.5 | 0.975 | 26772.86806 | 126 |
| P848 | PTdeep | 45 | Stroma | PASSED | 0.8 | 0.975 | 21240.482 | 81 |
| P253 | PTsup | 3 | Tumor | PASSED | 0.85 | 0.982142857 | 27469.3427 | 192 |
| P768 | PTsup | 4 | Tumor | PASSED | 0.44 | 0.982142857 | 33548.05847 | 217 |
| P768 | LNmet | 5 | Stroma | PASSED | 0.44 | 0.982142857 | 20289.67046 | 175 |
| P786 | PTdeep | 7 | Stroma | PASSED | 0.85 | 0.982142857 | 13114.30631 | 78 |
| P653 | PTdeep | 11 | Stroma | PASSED | 0.92 | 0.982142857 | 4956.378399 | 31 |
| P685 | LNmet | 11 | Stroma | PASSED | 0.44 | 0.982142857 | 22512.80474 | 108 |
| P879 | LNmet | 13 | Stroma | PASSED | 0.85 | 0.982142857 | 29884.90503 | 139 |
| P685 | PTdeep | 17 | Stroma | PASSED | 0.92 | 0.982142857 | 20123.88875 | 158 |
| P685 | PTdeep | 18 | Stroma | PASSED | 0.44 | 0.982142857 | 6716.313168 | 54 |
| P324 | PTdeep | 20 | Tumor | PASSED | 0.85 | 0.982142857 | 27631.45455 | 172 |
| P435 | LNmet | 23 | Stroma | PASSED | 0.92 | 0.982142857 | 5436.172039 | 121 |
| P435 | PTdeep | 24 | Stroma | PASSED | 0.44 | 0.982142857 | 8509.117072 | 99 |
| P541 | PTdeep | 26 | Tumor | PASSED | 0.85 | 0.982142857 | 30998.78577 | 192 |
| P035 | LNmet | 29 | Stroma | PASSED | 0.92 | 0.982142857 | 26313.49796 | 239 |
| P937 | PTdeep | 30 | Stroma | PASSED | 0.44 | 0.982142857 | 9924.404597 | 112 |
| P481 | LNmet | 32 | Tumor | PASSED | 0.85 | 0.982142857 | 31551.17872 | 92 |
| P937 | LNmet | 35 | Stroma | PASSED | 0.92 | 0.982142857 | 37671.85848 | 137 |
| P334 | LNmet | 37 | Stroma | PASSED | 0.44 | 0.982142857 | 27284.25437 | 103 |
| P334 | PTdeep | 38 | Stroma | PASSED | 0.85 | 0.982142857 | 34060.08301 | 70 |
| P848 | LNmet | 41 | Stroma | PASSED | 0.92 | 0.982142857 | 23891.55328 | 158 |
| P848 | LNmet | 43 | Stroma | PASSED | 0.44 | 0.982142857 | 14206.6466 | 124 |
| P848 | PTdeep | 45 | Tumor | PASSED | 0.85 | 0.982142857 | 36924.16543 | 341 |
| P786 | LNmet | 10 | Tumor | PASSED | 0.59 | 0.985714286 | 16836.01787 | 75 |
| P879 | PTdeep | 14 | Tumor | PASSED | 0.57 | 0.985714286 | 26808.29014 | 181 |
| P653 | LNmet | 17 | Tumor | PASSED | 0.59 | 0.985714286 | 43066.22608 | 172 |
| P324 | LNmet | 20 | Tumor | PASSED | 0.57 | 0.985714286 | 9848.454557 | 70 |
| P435 | LNmet | 23 | Tumor | PASSED | 0.59 | 0.985714286 | 20050.49166 | 161 |
| P541 | LNmet | 26 | Tumor | PASSED | 0.57 | 0.985714286 | 14470.7166 | 115 |
| P035 | LNmet | 29 | Tumor | PASSED | 0.59 | 0.985714286 | 26737.60554 | 189 |
| P481 | LNmet | 32 | Tumor | PASSED | 0.57 | 0.985714286 | 34209.74927 | 215 |
| P937 | LNmet | 35 | Tumor | PASSED | 0.59 | 0.985714286 | 21907.27868 | 82 |
| P334 | LNmet | 38 | Tumor | PASSED | 0.57 | 0.985714286 | 36377.35705 | 276 |
| P848 | LNmet | 41 | Tumor | PASSED | 0.59 | 0.985714286 | 41028.97793 | 189 |
| P848 | PTdeep | 44 | Tumor | PASSED | 0.57 | 0.985714286 | 23140.19038 | 93 |
| P253 | PTsup | 2 | Stroma | PASSED | 0.69 | 0.989285714 | 10762.72711 | 83 |
| P253 | LNmet | 4 | Tumor | PASSED | 0.69 | 0.989285714 | 23042.22121 | 211 |
| P786 | PTdeep | 9 | Tumor | PASSED | 0.51 | 0.989285714 | 21723.30726 | 203 |
| P685 | PTsup | 10 | Tumor | PASSED | 0.69 | 0.989285714 | 33663.89824 | 299 |
| P653 | LNmet | 16 | Tumor | PASSED | 0.69 | 0.989285714 | 31224.72119 | 207 |
| P653 | LNmet | 16 | Tumor | PASSED | 0.51 | 0.989285714 | 32566.2925 | 110 |
| P435 | LNmet | 22 | Tumor | PASSED | 0.51 | 0.989285714 | 35152.4233 | 249 |
| P435 | PTsup | 23 | Tumor | PASSED | 0.69 | 0.989285714 | 36590.68731 | 229 |
| P035 | LNmet | 28 | Tumor | PASSED | 0.51 | 0.989285714 | 28483.02045 | 175 |
| P035 | PTsup | 29 | Tumor | PASSED | 0.69 | 0.989285714 | 14670.80347 | 124 |
| P035 | PTdeep | 34 | Tumor | PASSED | 0.51 | 0.989285714 | 21236.33347 | 148 |
| P937 | PTsup | 35 | Tumor | PASSED | 0.69 | 0.989285714 | 30004.25509 | 313 |
| P348 | PTdeep | 40 | Tumor | PASSED | 0.51 | 0.989285714 | 38236.05878 | 118 |
| P848 | LNmet | 42 | Tumor | PASSED | 0.69 | 0.989285714 | 37194.29866 | 278 |
| P253 | PTsup | 1 | Tumor | PASSED | 0.53 | 0.992857143 | 19210.414 | 162 |
| P768 | PTsup | 4 | Tumor | PASSED | 0.75 | 0.992857143 | 42854.81053 | 354 |
| P786 | LNmet | 8 | Stroma | PASSED | 0.75 | 0.992857143 | 2884.665521 | 25 |
| P786 | LNmet | 9 | Stroma | PASSED | 1.01 | 0.992857143 | 29059.66635 | 189 |
| P685 | LNmet | 12 | Tumor | PASSED | 0.48 | 0.992857143 | 29751.0351 | 186 |
| P879 | LNmet | 13 | Tumor | PASSED | 0.53 | 0.992857143 | 38684.57888 | 389 |
| P879 | LNmet | 14 | Stroma | PASSED | 0.75 | 0.992857143 | 15437.80315 | 130 |
| P653 | PTsup | 16 | Stroma | PASSED | 1.01 | 0.992857143 | 8781.48413 | 51 |
| P653 | LNmet | 18 | Tumor | PASSED | 0.48 | 0.992857143 | 30995.27548 | 440 |
| P324 | PTsup | 19 | Tumor | PASSED | 0.53 | 0.992857143 | 37378.90832 | 221 |
| P324 | LNmet | 21 | Tumor | PASSED | 0.75 | 0.992857143 | 41874.32103 | 106 |
| P435 | LNmet | 22 | Stroma | PASSED | 1.01 | 0.992857143 | 24528.35289 | 63 |
| P435 | LNmet | 24 | Tumor | PASSED | 0.48 | 0.992857143 | 9118.951223 | 70 |
| P541 | LNmet | 27 | Tumor | PASSED | 0.75 | 0.992857143 | 27803.45906 | 139 |
| P035 | PTsup | 28 | Stroma | PASSED | 1.01 | 0.992857143 | 9648.686802 | 103 |
| P035 | LNmet | 30 | Tumor | PASSED | 0.48 | 0.992857143 | 31649.62657 | 142 |
| P481 | LNmet | 31 | Tumor | PASSED | 0.53 | 0.992857143 | 42011.54169 | 264 |
| P481 | LNmet | 33 | Stroma | PASSED | 0.75 | 0.992857143 | 16273.89183 | 56 |
| P937 | LNmet | 34 | Stroma | PASSED | 1.01 | 0.992857143 | 30795.34817 | 174 |
| P937 | LNmet | 36 | Tumor | PASSED | 0.48 | 0.992857143 | 23371.23168 | 98 |
| P334 | LNmet | 37 | Stroma | PASSED | 0.53 | 0.992857143 | 25414.22395 | 286 |
| P348 | LNmet | 39 | Stroma | PASSED | 0.75 | 0.992857143 | 28627.10215 | 130 |
| P348 | LNmet | 40 | Stroma | PASSED | 1.01 | 0.992857143 | 14672.07994 | 96 |
| P848 | PTsup | 42 | Tumor | PASSED | 0.48 | 0.992857143 | 22522.85696 | 148 |
| P848 | LNmet | 43 | Stroma | PASSED | 0.53 | 0.992857143 | 5777.947222 | 77 |
| P575 | PTdeep | 46 | Tumor | PASSED | 0.75 | 0.992857143 | 7615.427621 | 59 |
| P253 | PTsup | 1 | Stroma | PASSED | 0.59 | 0.996428571 | 6645.628571 | 36 |
| P685 | LNmet | 1 | Stroma | PASSED | 0.65 | 0.996428571 | 13845.40523 | 93 |
| P253 | PTsup | 2 | Tumor | PASSED | 0.46 | 0.996428571 | 31682.97438 | 106 |
| P253 | LNmet | 2 | Stroma | PASSED | 0.8 | 0.996428571 | 37904.65493 | 350 |
| P253 | LNmet | 2 | Tumor | PASSED | 0.52 | 0.996428571 | 59466.48857 | 152 |
| P253 | LNmet | 3 | Stroma | PASSED | 0.46 | 0.996428571 | 8073.52125 | 66 |
| P253 | LNmet | 3 | Stroma | PASSED | 0.6 | 0.996428571 | 10541.09978 | 122 |
| P253 | PTsup | 3 | Tumor | PASSED | 0.65 | 0.996428571 | 22874.84391 | 46 |
| P253 | PTsup | 3 | Tumor | PASSED | 0.45 | 0.996428571 | 36109.77676 | 115 |
| P253 | PTdeep | 3 | Tumor | PASSED | 0.62 | 0.996428571 | 36954.32206 | 147 |
| P768 | PTsup | 4 | Stroma | PASSED | 0.62 | 0.996428571 | 20132.18582 | 159 |
| P768 | PTdeep | 4 | Tumor | PASSED | 0.49 | 0.996428571 | 163268.6539 | 426 |
| P768 | LNmet | 5 | Tumor | PASSED | 0.6 | 0.996428571 | 36934.53676 | 291 |
| P768 | LNmet | 6 | Stroma | PASSED | 0.56 | 0.996428571 | 17423.67333 | 217 |
| P768 | LNmet | 7 | Stroma | PASSED | 0.59 | 0.996428571 | 17564.40429 | 128 |
| P786 | PTsup | 7 | Tumor | PASSED | 0.8 | 0.996428571 | 24532.34186 | 115 |
| P786 | PTsup | 7 | Tumor | PASSED | 0.52 | 0.996428571 | 29104.02373 | 254 |
| P786 | LNmet | 8 | Tumor | PASSED | 0.52 | 0.996428571 | 27951.21061 | 191 |
| P786 | PTsup | 9 | Tumor | PASSED | 0.45 | 0.996428571 | 12868.10692 | 83 |
| P786 | PTsup | 9 | Tumor | PASSED | 0.65 | 0.996428571 | 17766.88455 | 89 |
| P937 | LNmet | 9 | Stroma | PASSED | 0.46 | 0.996428571 | 28450.78955 | 138 |
| P685 | PTsup | 10 | Stroma | PASSED | 0.62 | 0.996428571 | 12274.0691 | 123 |
| P685 | PTsup | 11 | Tumor | PASSED | 0.6 | 0.996428571 | 28683.42644 | 230 |
| P685 | LNmet | 12 | Stroma | PASSED | 0.56 | 0.996428571 | 28982.5994 | 483 |
| P879 | PTsup | 13 | Stroma | PASSED | 0.59 | 0.996428571 | 4551.258328 | 35 |
| P879 | PTsup | 13 | Tumor | PASSED | 0.8 | 0.996428571 | 34002.00357 | 211 |
| P879 | LNmet | 14 | Stroma | PASSED | 0.52 | 0.996428571 | 20376.31095 | 186 |
| P879 | PTsup | 14 | Tumor | PASSED | 0.52 | 0.996428571 | 29579.82839 | 332 |
| P879 | LNmet | 15 | Stroma | PASSED | 0.46 | 0.996428571 | 19237.06034 | 164 |
| P879 | PTsup | 15 | Tumor | PASSED | 0.45 | 0.996428571 | 29106.57667 | 294 |
| P879 | PTsup | 15 | Tumor | PASSED | 0.65 | 0.996428571 | 43326.78578 | 286 |
| P316 | PTdeep | 16 | Tumor | PASSED | 0.49 | 0.996428571 | 19072.71466 | 87 |
| P653 | LNmet | 17 | Tumor | PASSED | 0.62 | 0.996428571 | 70053.2222 | 280 |
| P653 | LNmet | 18 | Stroma | PASSED | 0.56 | 0.996428571 | 21048.69219 | 300 |
| P653 | PTsup | 18 | Tumor | PASSED | 0.6 | 0.996428571 | 34829.95475 | 121 |
| P324 | PTsup | 19 | Tumor | PASSED | 0.8 | 0.996428571 | 68407.69117 | 167 |
| P324 | LNmet | 20 | Stroma | PASSED | 0.59 | 0.996428571 | 5024.829171 | 48 |
| P324 | PTsup | 20 | Stroma | PASSED | 0.52 | 0.996428571 | 16817.50904 | 169 |
| P324 | LNmet | 21 | Tumor | PASSED | 0.45 | 0.996428571 | 36477.87916 | 189 |
| P435 | PTsup | 22 | Stroma | PASSED | 0.46 | 0.996428571 | 18907.41163 | 154 |
| P435 | PTsup | 22 | Tumor | PASSED | 0.49 | 0.996428571 | 35259.64689 | 234 |
| P351 | PTsup | 22 | Tumor | PASSED | 0.65 | 0.996428571 | 49781.74146 | 318 |
| P435 | PTsup | 23 | Stroma | PASSED | 0.62 | 0.996428571 | 9209.261566 | 105 |
| P435 | LNmet | 24 | Stroma | PASSED | 0.56 | 0.996428571 | 22641.8879 | 159 |
| P435 | PTsup | 24 | Tumor | PASSED | 0.6 | 0.996428571 | 41699.28491 | 274 |
| P541 | LNmet | 25 | Stroma | PASSED | 0.8 | 0.996428571 | 13557.72052 | 99 |
| P541 | LNmet | 26 | Stroma | PASSED | 0.59 | 0.996428571 | 6036.273096 | 32 |
| P541 | PTsup | 26 | Stroma | PASSED | 0.52 | 0.996428571 | 36627.06674 | 205 |
| P541 | LNmet | 27 | Tumor | PASSED | 0.45 | 0.996428571 | 36995.48826 | 113 |
| P035 | PTsup | 28 | Tumor | PASSED | 0.65 | 0.996428571 | 31731.32073 | 200 |
| P973 | LNmet | 28 | Stroma | PASSED | 0.46 | 0.996428571 | 36814.86758 | 283 |
| P035 | PTsup | 29 | Stroma | PASSED | 0.62 | 0.996428571 | 7034.633192 | 109 |
| P035 | LNmet | 30 | Stroma | PASSED | 0.56 | 0.996428571 | 10797.83007 | 209 |
| P035 | PTsup | 30 | Tumor | PASSED | 0.6 | 0.996428571 | 32322.32693 | 176 |
| P481 | PTsup | 31 | Stroma | PASSED | 0.8 | 0.996428571 | 21764.15434 | 38 |
| P481 | PTsup | 32 | Stroma | PASSED | 0.59 | 0.996428571 | 10341.17247 | 70 |
| P481 | PTsup | 32 | Stroma | PASSED | 0.52 | 0.996428571 | 15929.40415 | 100 |
| P481 | PTsup | 33 | Tumor | PASSED | 0.45 | 0.996428571 | 23383.99639 | 105 |
| P937 | LNmet | 34 | Stroma | PASSED | 0.46 | 0.996428571 | 6689.985948 | 42 |
| P937 | PTsup | 34 | Tumor | PASSED | 0.65 | 0.996428571 | 14240.15398 | 100 |
| P937 | PTsup | 34 | Tumor | PASSED | 0.49 | 0.996428571 | 32210.47613 | 240 |
| P937 | PTsup | 36 | Tumor | PASSED | 0.62 | 0.996428571 | 69016.40841 | 370 |
| P348 | PTsup | 37 | Tumor | PASSED | 0.6 | 0.996428571 | 29442.92685 | 253 |
| P334 | LNmet | 38 | Tumor | PASSED | 0.8 | 0.996428571 | 22662.15188 | 176 |
| P348 | PTsup | 38 | Stroma | PASSED | 0.52 | 0.996428571 | 28562.16167 | 337 |
| P348 | PTsup | 38 | Stroma | PASSED | 0.52 | 0.996428571 | 30376.18691 | 442 |
| P348 | PTsup | 39 | Stroma | PASSED | 0.45 | 0.996428571 | 6841.72647 | 109 |
| P348 | PTsup | 39 | Stroma | PASSED | 0.59 | 0.996428571 | 27061.82925 | 207 |
| P348 | LNmet | 40 | Stroma | PASSED | 0.49 | 0.996428571 | 14052.99138 | 206 |
| P848 | PTsup | 41 | Stroma | PASSED | 0.46 | 0.996428571 | 10913.66984 | 99 |
| P848 | PTsup | 41 | Tumor | PASSED | 0.65 | 0.996428571 | 38110.9646 | 237 |
| P848 | PTsup | 42 | Stroma | PASSED | 0.62 | 0.996428571 | 10018.3848 | 74 |
| P575 | PTsup | 43 | Tumor | PASSED | 0.56 | 0.996428571 | 23332.93754 | 142 |
| P575 | PTsup | 43 | Tumor | PASSED | 0.6 | 0.996428571 | 32512.84026 | 278 |
| P575 | LNmet | 44 | Stroma | PASSED | 0.52 | 0.996428571 | 7620.373948 | 32 |
| P575 | LNmet | 44 | Stroma | PASSED | 0.52 | 0.996428571 | 17268.26296 | 120 |
| P575 | PTsup | 44 | Stroma | PASSED | 0.8 | 0.996428571 | 17721.72938 | 116 |
| P575 | LNmet | 45 | Stroma | PASSED | 0.59 | 0.996428571 | 8054.693299 | 66 |
| P575 | LNmet | 46 | Tumor | PASSED | 0.45 | 0.996428571 | 26759.94379 | 327 |
| P685 | LNmet | 1 | Stroma | PASSED | 0.64 | 1 | 20583.73753 | 169 |
| P253 | PTsup | 1 | Tumor | PASSED | 0.53 | 1 | 29668.06447 | 108 |
| P253 | PTsup | 1 | Tumor | PASSED | 0.9 | 1 | 29771.93732 | 261 |
| P253 | PTdeep | 1 | Tumor | PASSED | 0.44 | 1 | 30454.21122 | 115 |
| P253 | PTdeep | 1 | Stroma | PASSED | 0.49 | 1 | 35504.41025 | 752 |
| P253 | PTsup | 2 | Stroma | PASSED | 0.44 | 1 | 25202.8084 | 137 |
| P253 | PTsup | 2 | Tumor | PASSED | 0.63 | 1 | 26642.82755 | 46 |
| P253 | PTdeep | 2 | Tumor | PASSED | 0.59 | 1 | 31586.91991 | 325 |
| P253 | PTdeep | 2 | Stroma | PASSED | 0.51 | 1 | 52614.23121 | 444 |
| P253 | PTdeep | 3 | Stroma | PASSED | 0.52 | 1 | 65289.11229 | 662 |
| P768 | PTdeep | 4 | Stroma | PASSED | 0.61 | 1 | 24147.16665 | 234 |
| P768 | PTsup | 5 | Stroma | PASSED | 0.62 | 1 | 4027.107311 | 52 |
| P768 | PTsup | 5 | Stroma | PASSED | 0.9 | 1 | 7657.710732 | 84 |
| P768 | PTdeep | 5 | Tumor | PASSED | 0.69 | 1 | 25357.26142 | 85 |
| P768 | PTdeep | 5 | Tumor | PASSED | 0.46 | 1 | 44089.15825 | 200 |
| P768 | PTsup | 6 | Stroma | PASSED | 0.69 | 1 | 7972.839578 | 116 |
| P768 | PTsup | 6 | Stroma | PASSED | 0.59 | 1 | 12173.8661 | 111 |
| P768 | PTdeep | 6 | Tumor | PASSED | 0.63 | 1 | 26533.21058 | 159 |
| P768 | PTdeep | 6 | Tumor | PASSED | 0.64 | 1 | 33743.99681 | 129 |
| P926 | PTdeep | 7 | Stroma | PASSED | 0.49 | 1 | 14386.78861 | 209 |
| P926 | PTdeep | 7 | Tumor | PASSED | 0.53 | 1 | 41464.09507 | 135 |
| P786 | PTsup | 8 | Stroma | PASSED | 0.44 | 1 | 7348.645125 | 69 |
| P786 | PTsup | 8 | Stroma | PASSED | 0.51 | 1 | 8855.679023 | 80 |
| P937 | LNmet | 8 | Tumor | PASSED | 0.63 | 1 | 46599.65813 | 130 |
| P937 | LNmet | 9 | Tumor | PASSED | 0.61 | 1 | 28363.82995 | 159 |
| P937 | LNmet | 10 | Stroma | PASSED | 0.38 | 1 | 6900.125031 | 66 |
| P937 | LNmet | 11 | Stroma | PASSED | 0.9 | 1 | 9329.888102 | 67 |
| P685 | PTsup | 12 | Stroma | PASSED | 0.69 | 1 | 17989.9479 | 114 |
| P685 | PTsup | 12 | Tumor | PASSED | 0.64 | 1 | 21082.8378 | 79 |
| P316 | PTsup | 12 | Stroma | PASSED | 0.59 | 1 | 21109.96281 | 145 |
| P316 | PTsup | 12 | Tumor | PASSED | 0.63 | 1 | 28147.94674 | 205 |
| P316 | PTsup | 13 | Stroma | PASSED | 0.49 | 1 | 17725.39923 | 286 |
| P316 | PTsup | 13 | Tumor | PASSED | 0.53 | 1 | 41772.68201 | 278 |
| P879 | PTsup | 14 | Stroma | PASSED | 0.51 | 1 | 20234.46308 | 428 |
| P316 | PTsup | 14 | Stroma | PASSED | 0.44 | 1 | 25823.81167 | 177 |
| P316 | PTsup | 14 | Tumor | PASSED | 0.63 | 1 | 38044.90721 | 224 |
| P316 | PTdeep | 15 | Stroma | PASSED | 0.52 | 1 | 28591.83963 | 509 |
| P316 | PTdeep | 15 | Tumor | PASSED | 0.61 | 1 | 45570.50316 | 307 |
| P316 | PTdeep | 16 | Stroma | PASSED | 0.38 | 1 | 13634.3088 | 139 |
| P653 | PTsup | 17 | Stroma | PASSED | 0.9 | 1 | 8633.73258 | 87 |
| P316 | PTdeep | 17 | Tumor | PASSED | 0.46 | 1 | 17183.37762 | 104 |
| P316 | PTdeep | 17 | Stroma | PASSED | 0.62 | 1 | 25370.98349 | 193 |
| P653 | PTsup | 18 | Stroma | PASSED | 0.59 | 1 | 5163.485863 | 34 |
| P351 | LNmet | 18 | Tumor | PASSED | 0.64 | 1 | 37072.2361 | 312 |
| P351 | LNmet | 18 | Tumor | PASSED | 0.63 | 1 | 59652.3747 | 41 |
| P351 | LNmet | 19 | Stroma | PASSED | 0.69 | 1 | 10686.45795 | 54 |
| P351 | LNmet | 19 | Stroma | PASSED | 0.49 | 1 | 16979.46133 | 214 |
| P324 | PTsup | 20 | Stroma | PASSED | 0.51 | 1 | 20438.21981 | 236 |
| P351 | LNmet | 20 | Tumor | PASSED | 0.53 | 1 | 31398.79996 | 139 |
| P324 | PTsup | 21 | Stroma | PASSED | 0.61 | 1 | 3197.081865 | 36 |
| P324 | PTsup | 21 | Stroma | PASSED | 0.44 | 1 | 12358.63532 | 66 |
| P351 | PTsup | 21 | Stroma | PASSED | 0.52 | 1 | 18864.17117 | 150 |
| P351 | PTsup | 21 | Tumor | PASSED | 0.63 | 1 | 37622.39522 | 140 |
| P351 | PTsup | 22 | Stroma | PASSED | 0.38 | 1 | 18347.04074 | 191 |
| P351 | PTsup | 23 | Stroma | PASSED | 0.62 | 1 | 8148.035261 | 94 |
| P351 | PTsup | 23 | Tumor | PASSED | 0.46 | 1 | 17061.15549 | 129 |
| P435 | PTsup | 24 | Stroma | PASSED | 0.64 | 1 | 6577.177799 | 103 |
| P351 | PTdeep | 24 | Tumor | PASSED | 0.9 | 1 | 40647.15346 | 348 |
| P541 | PTsup | 25 | Stroma | PASSED | 0.69 | 1 | 5458.191168 | 45 |
| P541 | PTsup | 25 | Stroma | PASSED | 0.49 | 1 | 18260.08114 | 107 |
| P351 | PTdeep | 25 | Tumor | PASSED | 0.59 | 1 | 26041.29046 | 46 |
| P541 | PTsup | 26 | Stroma | PASSED | 0.51 | 1 | 4998.661509 | 49 |
| P351 | PTdeep | 26 | Tumor | PASSED | 0.53 | 1 | 40556.20488 | 135 |
| P541 | PTsup | 27 | Stroma | PASSED | 0.61 | 1 | 4287.188333 | 32 |
| P541 | PTsup | 27 | Stroma | PASSED | 0.52 | 1 | 8758.507647 | 79 |
| P973 | LNmet | 27 | Stroma | PASSED | 0.44 | 1 | 29201.83334 | 56 |
| P973 | LNmet | 27 | Tumor | PASSED | 0.63 | 1 | 30440.96783 | 108 |
| P973 | LNmet | 28 | Stroma | PASSED | 0.38 | 1 | 6200.618773 | 64 |
| P973 | LNmet | 29 | Stroma | PASSED | 0.62 | 1 | 13194.88356 | 99 |
| P973 | LNmet | 29 | Tumor | PASSED | 0.46 | 1 | 51566.2483 | 342 |
| P035 | PTsup | 30 | Tumor | PASSED | 0.63 | 1 | 6333.212227 | 49 |
| P973 | PTsup | 30 | Stroma | PASSED | 0.64 | 1 | 8811.321646 | 51 |
| P973 | PTsup | 30 | Tumor | PASSED | 0.9 | 1 | 50310.51968 | 396 |
| P481 | PTsup | 31 | Stroma | PASSED | 0.69 | 1 | 11544.24664 | 50 |
| P973 | PTsup | 31 | Stroma | PASSED | 0.49 | 1 | 13311.36156 | 82 |
| P973 | PTsup | 31 | Tumor | PASSED | 0.59 | 1 | 30600.04806 | 187 |
| P973 | PTsup | 32 | Stroma | PASSED | 0.51 | 1 | 13323.6476 | 39 |
| P973 | PTsup | 32 | Tumor | PASSED | 0.53 | 1 | 27246.27935 | 224 |
| P481 | PTsup | 33 | Stroma | PASSED | 0.52 | 1 | 6017.764263 | 57 |
| P973 | PTdeep | 33 | Stroma | PASSED | 0.44 | 1 | 7865.456432 | 96 |
| P973 | PTdeep | 33 | Tumor | PASSED | 0.63 | 1 | 33335.20688 | 236 |
| P973 | PTdeep | 34 | Stroma | PASSED | 0.38 | 1 | 28077.10259 | 197 |
| P973 | PTdeep | 34 | Tumor | PASSED | 0.61 | 1 | 29462.23348 | 187 |
| P973 | PTdeep | 35 | Stroma | PASSED | 0.62 | 1 | 5396.760988 | 60 |
| P973 | PTdeep | 35 | Tumor | PASSED | 0.46 | 1 | 47623.06897 | 227 |
| P270 | LNmet | 36 | Stroma | PASSED | 0.9 | 1 | 9772.344957 | 93 |
| P348 | PTsup | 37 | Stroma | PASSED | 0.59 | 1 | 15937.54165 | 78 |
| P270 | LNmet | 37 | Tumor | PASSED | 0.64 | 1 | 25714.1947 | 210 |
| P270 | LNmet | 38 | Stroma | PASSED | 0.69 | 1 | 26268.66191 | 154 |
| P270 | PTsup | 39 | Tumor | PASSED | 0.53 | 1 | 36659.77632 | 210 |
| P848 | PTsup | 40 | Stroma | PASSED | 0.44 | 1 | 10061.62526 | 75 |
| P848 | PTsup | 40 | Tumor | PASSED | 0.61 | 1 | 32974.92287 | 119 |
| P270 | PTsup | 40 | Tumor | PASSED | 0.63 | 1 | 34540.35533 | 256 |
| P270 | PTsup | 41 | Stroma | PASSED | 0.46 | 1 | 11718.16586 | 90 |
| P270 | PTsup | 41 | Tumor | PASSED | 0.38 | 1 | 32425.7211 | 274 |
| P270 | PTdeep | 42 | Stroma | PASSED | 0.63 | 1 | 8250.791198 | 97 |
| P270 | PTdeep | 43 | Stroma | PASSED | 0.64 | 1 | 19972.46735 | 107 |
| P270 | PTdeep | 43 | Tumor | PASSED | 0.9 | 1 | 42218.33004 | 352 |
| P575 | PTsup | 44 | Stroma | PASSED | 0.69 | 1 | 12802.84732 | 115 |
| P270 | PTdeep | 44 | Tumor | PASSED | 0.59 | 1 | 33139.58766 | 275 |
| P270 | PTdeep | 44 | Tumor | PASSED | 0.49 | 1 | 38657.93254 | 427 |
| P575 | PTsup | 45 | Tumor | PASSED | 0.51 | 1 | 19773.01871 | 150 |
| P575 | PTsup | 45 | Tumor | PASSED | 0.53 | 1 | 37094.7339 | 297 |
| P575 | PTdeep | 47 | Tumor | PASSED | 0.61 | 1 | 17497.38955 | 83 |
