# Supplementary material for: Multi‐omics analyses reveal spatial heterogeneity in primary and metastatic oesophageal squamous cell carcinoma
Source: Clin Transl Med. 2023 Nov 27;13(11):e1493. doi: 10.1002/ctm2.1493 (PMC10679972; doi:10.1002/ctm2.1493)
Supplement: Supplementary file 14 — Table S3. Sample list and various analyses performed per section. [file CTM2-13-e1493-s004.docx]

**Supplementary Table 3. Sample list and various analyses performed per section.**

| **Patient ID** | **Whole Exome Sequencing** | | | | **Whole Transcriptome Sequencing** | | | **Digital Spatial Profiling** | | | **Immunohistochemistry** | | | **Histopath** | **Clinical** |
| --- | --- | --- | --- | --- | --- | --- | --- | --- | --- | --- | --- | --- | --- | --- | --- |
|  | PT**_sup_** | PT**_deep_** | LN**_met_** | **Adjacent normal tissues** | PT**_sup_** | PT**_deep_** | LN**_met_** | PT**_sup_** | PT**_deep_** | LN**_met_** | PT**_sup_** | PT**_deep_** | LN**_met_** |  |  |
| P035 | √ | √ | √ | √ | √ | √ | √ | √ | √ | √ | √ | √ | √ | √ | √ |
| P316 | √ | √ | √ | √ |  | √ | √ | √ | √ |  | √ | √ | √ | √ | √ |
| P348 | √ | √ | √ | √ | √ | √ | √ | √ | √ | √ | √ | √ | √ | √ | √ |
| P435 | √ | √ | √ | √ | √ | √ | √ | √ | √ | √ |  |  |  | √ | √ |
| P481 | √ | √ | √ | √ | √ | √ | √ | √ | √ | √ |  |  |  | √ | √ |
| P575 | √ | √ | √ | √ | √ | √ | √ | √ | √ | √ | √ | √ | √ | √ | √ |
| P685 | √ | √ | √ | √ | √ | √ | √ | √ | √ | √ | √ | √ | √ | √ | √ |
| P879 | √ | √ | √ | √ | √ | √ | √ | √ | √ | √ |  |  |  | √ | √ |
| P926 | √ | √ | √ | √ | √ | √ | √ |  | √ |  | √ | √ | √ | √ | √ |
| P253 | √ | √ | √ | √ | √ | √ | √ | √ | √ | √ | √ | √ | √ | √ | √ |
| P653 | √ | √ | √ | √ | √ | √ | √ | √ | √ | √ |  |  |  | √ | √ |
| P768 | √ | √ | √ | √ | √ | √ | √ | √ | √ | √ | √ | √ | √ | √ | √ |
| P786 | √ | √ | √ | √ | √ | √ |  | √ | √ | √ |  |  |  | √ | √ |
| P848 | √ | √ | √ | √ | √ | √ | √ | √ | √ | √ | √ | √ | √ | √ | √ |
| P973 | √ | √ | √ | √ |  |  |  | √ | √ | √ |  |  |  | √ | √ |
| P541 |  |  |  |  | √ | √ | √ | √ | √ | √ |  |  |  | √ | √ |
| P351 |  |  |  |  | √ | √ | √ | √ | √ | √ | √ | √ | √ | √ | √ |
| P324 |  |  |  |  | √ | √ |  | √ | √ | √ |  |  |  | √ | √ |
| P334 |  |  |  |  | √ | √ | √ |  | √ | √ | √ | √ | √ | √ | √ |
| P270 |  |  |  |  |  | √ | √ | √ | √ | √ | √ | √ | √ | √ | √ |
| P937 |  |  |  |  |  |  |  | √ | √ | √ | √ | √ | √ | √ | √ |
