# Supplementary material for: Multi‐omics analyses reveal spatial heterogeneity in primary and metastatic oesophageal squamous cell carcinoma
Source: Clin Transl Med. 2023 Nov 27;13(11):e1493. doi: 10.1002/ctm2.1493 (PMC10679972; doi:10.1002/ctm2.1493)
Supplement: Supplementary file 15 — Table S4. Tumour purity estimated by All‐FIT and FACETs. [file CTM2-13-e1493-s007.docx]

**Supplementary Table 4. Tumor purity estimated by All-FIT and FACETs.**

| **Sample** | **Purity_All-FIT** | **Purity_FACETs** | **Sample** | **Purity_All-FIT** | **Purity_FACETs** |
| --- | --- | --- | --- | --- | --- |
| 035-LN_met_ | 0.39 | 0.49 | 685-LN_met_ | 0.19 | 0.3 |
| 035-PT_deep_ | 0.37 | 0.32 | 685-PT_deep_ | 0.34 | 0.37 |
| 035-PT_sup_ | 0.37 | 0.39 | 685-PT_sup_ | 0.45 | 0.5 |
| 253-LN_met_ | 0.44 | 0.42 | 768-LN_met_ | 0.22 | 0.27 |
| 253-PT_deep_ | 0.16 | 0.2 | 768-PT_deep_ | 0.31 | 0.42 |
| 253-PT_sup_ | 0.36 | 0.4 | 768-PT_sup_ | 0.42 | 0.45 |
| 316-LN_met_ | 0.39 | 0.38 | 786-LN_met_ | 0.19 | 0.38 |
| 316-PT_deep_ | 0.35 | 0.36 | 786-PT_deep_ | 0.31 | 0.44 |
| 316-PT_sup_ | 0.45 | 0.65 | 786-PT_sup_ | 0.28 | 0.36 |
| 348-LN_met_ | 0.15 | 0.14 | 848-LN_met_ | 0.21 | 0.32 |
| 348-PT_deep_ | 0.38 | 0.45 | 848-PT_deep_ | 0.4 | 0.47 |
| 348-PT_sup_ | 0.47 | 0.54 | 848-PT_sup_ | 0.39 | 0.46 |
| 435-LN_met_ | 0.42 | 0.67 | 879-LN_met_ | 0.33 | 0.36 |
| 435-PT_deep_ | 0.35 | 0.47 | 879-PT_deep_ | 0.56 | 0.8 |
| 435-PT_sup_ | 0.2 | 0.21 | 879-PT_sup_ | 0.45 | 0.55 |
| 481-LN_met_ | 0.4 | 0.44 | 926-LN_met_ | 0.29 | 0.3 |
| 481-PT_deep_ | 0.31 | 0.28 | 926-PT_deep_ | 0.2 | 0.22 |
| 481-PT_sup_ | 0.46 | 0.66 | 926-PT_sup_ | 0.38 | 0.53 |
| 575-LN_met_ | 0.37 | 0.49 | 973-LN_met_ | 0.42 | 0.31 |
| 575-PT_deep_ | 0.46 | 0.61 | 973-PT_deep_ | 0.4 | 0.29 |
| 575-PT_sup_ | 0.49 | 0.73 | 973-PT_sup_ | 0.25 | 0.31 |
| 653-LN_met_ | 0.23 | 0.29 |  |  |  |
| 653-PT_deep_ | 0.27 | 0.28 |  |  |  |
| 653-PT_sup_ | 0.4 | 0.44 |  |  |  |
