# Supplementary material for: Multi‐omics analyses reveal spatial heterogeneity in primary and metastatic oesophageal squamous cell carcinoma
Source: Clin Transl Med. 2023 Nov 27;13(11):e1493. doi: 10.1002/ctm2.1493 (PMC10679972; doi:10.1002/ctm2.1493)
Supplement: Supplementary file 17 — Table S6. MSI, TMB, TNB and ITH levels in the three subregions of each patient. [file CTM2-13-e1493-s012.docx]

**Supplementary Table 6. MSI, TMB, TNB, and ITH levels in the three subregions of each patient.**

| **Sample** | **MSI** | **MSI Type** | **TMB** | **TMB Type** | **TNB** | **TNB Type** | **Clonal Count** | **Subclonal Count** | **ITH** |
| --- | --- | --- | --- | --- | --- | --- | --- | --- | --- |
| P035-LNmet | 3.12 | MSI-L | 3.53 | TMB-M | 1.53 | TNB-M | 262 | 67 | 0.2 |
| P035-PTdeep | 4.4 | MSI-L | 3.67 | TMB-M | 1.2 | TNB-M | 301 | 39 | 0.11 |
| P035-PTsup | 3.12 | MSI-L | 3.87 | TMB-M | 1.27 | TNB-M | 290 | 55 | 0.16 |
| P253-LNmet | 6.13 | MSI-L | 2.46 | TMB-L | 0.46 | TNB-L | 173 | 49 | 0.2207 |
| P253-PTdeep | 3.64 | MSI-L | 2.78 | TMB-M | 0.54 | TNB-M | 79 | 157 | 0.6653 |
| P253-PTsup | 4.82 | MSI-L | 2.78 | TMB-M | 0.63 | TNB-M | 158 | 93 | 0.3705 |
| P316-LNmet | 3.95 | MSI-L | 2.77 | TMB-M | 0.9 | TNB-M | 205 | 39 | 0.16 |
| P316-PTdeep | 0.65 | MSI-L | 2.47 | TMB-L | 0.73 | TNB-M | 243 | 0 | 0 |
| P316-PTsup | 1.85 | MSI-L | 2.9 | TMB-M | 0.93 | TNB-M | 146 | 180 | 0.55 |
| P348-LNmet | 2.53 | MSI-L | 3.9 | TMB-M | 1.3 | TNB-M | 320 | 0 | 0 |
| P348-PTdeep | 5.26 | MSI-L | 3.73 | TMB-M | 1.07 | TNB-M | 47 | 291 | 0.86 |
| P348-PTsup | 6.21 | MSI-L | 4.5 | TMB-M | 1.47 | TNB-M | 361 | 0 | 0 |
| P435-LNmet | 7.38 | MSI-L | 3.5 | TMB-M | 0.8 | TNB-M | 143 | 101 | 0.41 |
| P435-PTdeep | 5.3 | MSI-L | 3.3 | TMB-M | 0.67 | TNB-M | 203 | 20 | 0.09 |
| P435-PTsup | 5.3 | MSI-L | 2.8 | TMB-M | 0.7 | TNB-M | 120 | 70 | 0.37 |
| P481-LNmet | 4.58 | MSI-L | 4.1 | TMB-M | 2.13 | TNB-M | 374 | 0 | 0 |
| P481-PTdeep | 2.92 | MSI-L | 3.2 | TMB-M | 1.53 | TNB-M | 256 | 0 | 0 |
| P481-PTsup | 6.71 | MSI-L | 5.37 | TMB-M | 2.43 | TNB-M | 342 | 95 | 0.22 |
| P575-LNmet | 4.61 | MSI-L | 1.87 | TMB-L | 0.6 | TNB-M | 193 | 0 | 0 |
| P575-PTdeep | 6.21 | MSI-L | 2.13 | TMB-L | 0.53 | TNB-M | 245 | 0 | 0 |
| P575-PTsup | 3.53 | MSI-L | 2.67 | TMB-M | 0.7 | TNB-M | 296 | 0 | 0 |
| P653-LNmet | 1.15 | MSI-L | 7.94 | TMB-M | 2.18 | TNB-M | 447 | 100 | 0.1828 |
| P653-PTdeep | 3.45 | MSI-L | 6.34 | TMB-M | 1.69 | TNB-M | 397 | 105 | 0.2092 |
| P653-PTsup | 2.87 | MSI-L | 6.31 | TMB-M | 1.75 | TNB-M | 430 | 91 | 0.1747 |
| P685-LNmet | 7.38 | MSI-L | 2.5 | TMB-M | 0.87 | TNB-M | 248 | 0 | 0 |
| P685-PTdeep | 3.38 | MSI-L | 4.43 | TMB-M | 0.53 | TNB-M | 383 | 0 | 0 |
| P685-PTsup | 5.77 | MSI-L | 4.6 | TMB-M | 0.57 | TNB-M | 320 | 72 | 0.18 |
| P768-LNmet | 1.97 | MSI-L | 3.7 | TMB-M | 0.63 | TNB-M | 2 | 309 | 0.9936 |
| P768-PTdeep | 2.63 | MSI-L | 4.33 | TMB-M | 0.86 | TNB-M | 335 | 53 | 0.1366 |
| P768-PTsup | 2.61 | MSI-L | 4.5 | TMB-M | 0.86 | TNB-M | 306 | 121 | 0.2834 |
| P786-LNmet | 2.96 | MSI-L | 4.13 | TMB-M | 0.83 | TNB-M | 33 | 253 | 0.8846 |
| P786-PTdeep | 4.09 | MSI-L | 4.31 | TMB-M | 0.95 | TNB-M | 265 | 44 | 0.1424 |
| P786-PTsup | 4.09 | MSI-L | 4.84 | TMB-M | 0.92 | TNB-M | 1 | 369 | 0.9973 |
| P848-LNmet | 2.42 | MSI-L | 4.54 | TMB-M | 0.63 | TNB-M | 217 | 128 | 0.371 |
| P848-PTdeep | 2.41 | MSI-L | 3.79 | TMB-M | 0.95 | TNB-M | 271 | 47 | 0.1478 |
| P848-PTsup | 4.19 | MSI-L | 3.99 | TMB-M | 0.92 | TNB-M | 278 | 55 | 0.1652 |
| P879-LNmet | 5.51 | MSI-L | 2.37 | TMB-L | 1 | TNB-M | 182 | 36 | 0.17 |
| P879-PTdeep | 3.91 | MSI-L | 2.57 | TMB-M | 1 | TNB-M | 138 | 115 | 0.45 |
| P879-PTsup | 3.15 | MSI-L | 2.8 | TMB-M | 1.47 | TNB-M | 203 | 58 | 0.22 |
| P926-LNmet | 3.75 | MSI-L | 3.27 | TMB-M | 1.07 | TNB-M | 296 | 0 | 0 |
| P926-PTdeep | 2.47 | MSI-L | 2.67 | TMB-M | 1.03 | TNB-M | 234 | 0 | 0 |
| P926-PTsup | 4.88 | MSI-L | 3.8 | TMB-M | 1.8 | TNB-M | 146 | 173 | 0.54 |
| P973-LNmet | 3.27 | MSI-L | 2.98 | TMB-M | 0.92 | TNB-M | 130 | 114 | 0.4672 |
| P973-PTdeep | 2.58 | MSI-L | 3.01 | TMB-M | 0.89 | TNB-M | 2 | 239 | 0.9917 |
| P973-PTsup | 1.92 | MSI-L | 2.89 | TMB-M | 0.86 | TNB-M | 3 | 220 | 0.9865 |
