# Supplementary material for: Multi‐omics analyses reveal spatial heterogeneity in primary and metastatic oesophageal squamous cell carcinoma
Source: Clin Transl Med. 2023 Nov 27;13(11):e1493. doi: 10.1002/ctm2.1493 (PMC10679972; doi:10.1002/ctm2.1493)
Supplement: Supplementary file 19 — Table S8. Detailed information of driver mutations in tumour subregions of each patient. [file CTM2-13-e1493-s016.docx]

**Supplementary Table 8. Detailed information of driver mutations in tumor subregions of each patient.**

| **Sample** | **Status** | **Hugo_Symbol** | **Chromosome** | **Strand** | **Variant_Classification** | **Variant_Type** | **Reference_Allele** | **Tumor_Seq_Allele1** | **Tumor_Seq_Allele2** | **dbSNP_RS** | **HGVSc** | **HGVSp** | **HGVSp_Short** | **Transcript_ID** | **t_depth** | **t_ref_count** | **t_alt_count** | **n_depth** | **n_ref_count** | **n_alt_count** |
| --- | --- | --- | --- | --- | --- | --- | --- | --- | --- | --- | --- | --- | --- | --- | --- | --- | --- | --- | --- | --- |
| P035-LNmet | Branch | YWHAE | 17 | + | Missense_Mutation | SNP | C | C | A | rs754551486 | c.49G>T | p.Ala17Ser | p.A17S | NM_006761.5 | 989 | 761 | 220 | 402 | 402 | 0 |
| P035-LNmet | Trunk | TRIP11 | 14 | + | Missense_Mutation | SNP | C | C | T | novel | c.2731G>A | p.Glu911Lys | p.E911K | NM_004239.4 | 605 | 468 | 137 | 221 | 219 | 0 |
| P035-LNmet | Trunk | TP53 | 17 | + | Missense_Mutation | SNP | A | A | T | rs730882027 | c.752T>A | p.Ile251Asn | p.I251N | NM_000546.5 | 375 | 284 | 91 | 102 | 102 | 0 |
| P035-LNmet | Branch | PRPF8 | 17 | + | In_Frame_Del | DEL | GTA | GTA | - | novel | c.695_697del | p.Leu232del | p.L232del | NM_006445.4 | 469 | 390 | 78 | 183 | 183 | 0 |
| P035-LNmet | Trunk | PRPF8 | 17 | + | Missense_Mutation | SNP | G | G | A |  | c.5090C>T | p.Ser1697Leu | p.S1697L | NM_006445.4 | 679 | 550 | 129 | 279 | 278 | 1 |
| P035-LNmet | Branch | NCOA1 | 2 | + | In_Frame_Del | DEL | TGTGGC | TGTGGC | - | novel | c.1198_1203del | p.Val400_Ala401del | p.V400_A401del | NM_003743.5 | 723 | 703 | 19 | 239 | 239 | 0 |
| P035-LNmet | Trunk | PIK3CA | 3 | + | Missense_Mutation | SNP | G | G | A | rs104886003 | c.1633G>A | p.Glu545Lys | p.E545K | NM_006218.4 | 633 | 166 | 467 | 74 | 73 | 0 |
| P035-LNmet | Branch | MUC5B | 11 | + | Missense_Mutation | SNP | G | G | C | novel | c.5119G>C | p.Gly1707Arg | p.G1707R | NM_002458.3 | 1423 | 1200 | 222 | 813 | 812 | 0 |
| P035-LNmet | Branch | MKI67 | 10 | + | Missense_Mutation | SNP | T | T | C | novel | c.264A>G | p.Ile88Met | p.I88M | NM_002417.5 | 238 | 188 | 50 | 111 | 111 | 0 |
| P035-LNmet | Trunk | MAP3K13 | 3 | + | Missense_Mutation | SNP | C | C | T | rs746379057 | c.566C>T | p.Ala189Val | p.A189V | NM_004721.5 | 617 | 519 | 94 | 205 | 200 | 0 |
| P035-LNmet | Trunk | HLA-A | 6 | + | Missense_Mutation | SNP | G | G | C |  | c.296G>C | p.Arg99Pro | p.R99P | NM_002116.8 | 2629 | 1883 | 742 | 1521 | 1517 | 4 |
| P035-LNmet | Trunk | FLNC | 7 | + | Nonsense_Mutation | SNP | G | G | T |  | c.5086G>T | p.Glu1696Ter | p.E1696* | NM_001458.4 | 803 | 623 | 178 | 365 | 365 | 0 |
| P035-LNmet | Branch | BRCA1 | 17 | + | Missense_Mutation | SNP | C | C | A |  | c.5258G>T | p.Arg1753Ile | p.R1753I | NM_007294.4 | 954 | 929 | 21 | 401 | 401 | 0 |
| P035-LNmet | Trunk | BAP1 | 3 | + | Nonsense_Mutation | SNP | G | G | T | novel | c.1454C>A | p.Ser485Ter | p.S485* | NM_004656.4 | 1760 | 1339 | 421 | 682 | 681 | 1 |
| P035-PTdeep | Trunk | TRIP11 | 14 | + | Missense_Mutation | SNP | C | C | T | novel | c.2731G>A | p.Glu911Lys | p.E911K | NM_004239.4 | 702 | 570 | 131 | 221 | 219 | 0 |
| P035-PTdeep | Trunk | TP53 | 17 | + | Missense_Mutation | SNP | A | A | T | rs730882027 | c.752T>A | p.Ile251Asn | p.I251N | NM_000546.5 | 342 | 259 | 81 | 102 | 102 | 0 |
| P035-PTdeep | Trunk | PRPF8 | 17 | + | Missense_Mutation | SNP | G | G | A |  | c.5090C>T | p.Ser1697Leu | p.S1697L | NM_006445.4 | 617 | 483 | 134 | 279 | 278 | 1 |
| P035-PTdeep | Trunk | PIK3CA | 3 | + | Missense_Mutation | SNP | G | G | A | rs104886003 | c.1633G>A | p.Glu545Lys | p.E545K | NM_006218.4 | 402 | 183 | 218 | 74 | 73 | 0 |
| P035-PTdeep | Branch | MAPK1 | 22 | + | Missense_Mutation | SNP | G | G | T | novel | c.171C>A | p.Ser57Arg | p.S57R | NM_138957.3 | 622 | 585 | 36 | 188 | 188 | 0 |
| P035-PTdeep | Trunk | MAP3K13 | 3 | + | Missense_Mutation | SNP | C | C | T | rs746379057 | c.566C>T | p.Ala189Val | p.A189V | NM_004721.5 | 1005 | 900 | 97 | 205 | 200 | 0 |
| P035-PTdeep | Trunk | HLA-A | 6 | + | Missense_Mutation | SNP | G | G | C |  | c.296G>C | p.Arg99Pro | p.R99P | NM_002116.8 | 2203 | 1526 | 675 | 1521 | 1517 | 4 |
| P035-PTdeep | Trunk | FLNC | 7 | + | Nonsense_Mutation | SNP | G | G | T |  | c.5086G>T | p.Glu1696Ter | p.E1696* | NM_001458.4 | 923 | 623 | 297 | 365 | 365 | 0 |
| P035-PTdeep | Trunk | BAP1 | 3 | + | Nonsense_Mutation | SNP | G | G | T | novel | c.1454C>A | p.Ser485Ter | p.S485* | NM_004656.4 | 1441 | 1077 | 363 | 682 | 681 | 1 |
| P035-PTsup | Trunk | TRIP11 | 14 | + | Missense_Mutation | SNP | C | C | T | novel | c.2731G>A | p.Glu911Lys | p.E911K | NM_004239.4 | 440 | 326 | 114 | 221 | 219 | 0 |
| P035-PTsup | Trunk | TP53 | 17 | + | Missense_Mutation | SNP | A | A | T | rs730882027 | c.752T>A | p.Ile251Asn | p.I251N | NM_000546.5 | 309 | 241 | 68 | 102 | 102 | 0 |
| P035-PTsup | Trunk | PRPF8 | 17 | + | Missense_Mutation | SNP | G | G | A |  | c.5090C>T | p.Ser1697Leu | p.S1697L | NM_006445.4 | 468 | 350 | 117 | 279 | 278 | 1 |
| P035-PTsup | Trunk | PIK3CA | 3 | + | Missense_Mutation | SNP | G | G | A | rs104886003 | c.1633G>A | p.Glu545Lys | p.E545K | NM_006218.4 | 273 | 100 | 173 | 74 | 73 | 0 |
| P035-PTsup | Branch | MAPK1 | 22 | + | Missense_Mutation | SNP | G | G | T | novel | c.171C>A | p.Ser57Arg | p.S57R | NM_138957.3 | 371 | 345 | 26 | 188 | 188 | 0 |
| P035-PTsup | Trunk | MAP3K13 | 3 | + | Missense_Mutation | SNP | C | C | T | rs746379057 | c.566C>T | p.Ala189Val | p.A189V | NM_004721.5 | 780 | 735 | 41 | 205 | 200 | 0 |
| P035-PTsup | Trunk | HLA-A | 6 | + | Missense_Mutation | SNP | G | G | C |  | c.296G>C | p.Arg99Pro | p.R99P | NM_002116.8 | 2306 | 1600 | 701 | 1521 | 1517 | 4 |
| P035-PTsup | Trunk | FLNC | 7 | + | Nonsense_Mutation | SNP | G | G | T |  | c.5086G>T | p.Glu1696Ter | p.E1696* | NM_001458.4 | 724 | 501 | 222 | 365 | 365 | 0 |
| P035-PTsup | Trunk | BAP1 | 3 | + | Nonsense_Mutation | SNP | G | G | T | novel | c.1454C>A | p.Ser485Ter | p.S485* | NM_004656.4 | 1246 | 893 | 353 | 682 | 681 | 1 |
| P316-LNmet | Trunk | TP53 | 17 | + | Missense_Mutation | SNP | G | G | T | rs876660254 | c.568C>A | p.Pro190Thr | p.P190T | NM_000546.5 | 186 | 117 | 69 | 285 | 283 | 0 |
| P316-LNmet | Trunk | SYNE1 | 6 | + | Missense_Mutation | SNP | C | C | G |  | c.23337G>C | p.Leu7779Phe | p.L7779F | NM_182961.4 | 206 | 146 | 60 | 267 | 267 | 0 |
| P316-LNmet | Trunk | SYNE1 | 6 | + | Splice_Site | SNP | T | T | C | novel | c.20068-2A>G |  | p.X6690_splice | NM_182961.4 | 260 | 187 | 73 | 363 | 363 | 0 |
| P316-LNmet | Branch | MYH4 | 17 | + | Missense_Mutation | SNP | C | C | G | novel | c.1240G>C | p.Val414Leu | p.V414L | NM_017533.2 | 188 | 157 | 31 | 321 | 320 | 0 |
| P316-LNmet | Branch | ANK2 | 4 | + | Missense_Mutation | SNP | G | G | C | novel | c.5482G>C | p.Gly1828Arg | p.G1828R | NM_001127493.2 | 171 | 129 | 42 | 250 | 248 | 0 |
| P316-LNmet | Trunk | ASXL1 | 20 | + | Nonsense_Mutation | SNP | A | A | T | rs749831576 | c.2734A>T | p.Lys912Ter | p.K912* | NM_015338.6 | 314 | 200 | 114 | 395 | 395 | 0 |
| P316-PTdeep | Trunk | TP53 | 17 | + | Missense_Mutation | SNP | G | G | T | rs876660254 | c.568C>A | p.Pro190Thr | p.P190T | NM_000546.5 | 196 | 142 | 54 | 285 | 283 | 0 |
| P316-PTdeep | Trunk | SYNE1 | 6 | + | Missense_Mutation | SNP | C | C | G |  | c.23337G>C | p.Leu7779Phe | p.L7779F | NM_182961.4 | 247 | 201 | 46 | 267 | 267 | 0 |
| P316-PTdeep | Trunk | SYNE1 | 6 | + | Splice_Site | SNP | T | T | C | novel | c.20068-2A>G |  | p.X6690_splice | NM_182961.4 | 244 | 190 | 53 | 363 | 363 | 0 |
| P316-PTdeep | Branch | MYH2 | 17 | + | Missense_Mutation | SNP | C | C | G | novel | c.418G>C | p.Val140Leu | p.V140L | NM_017534.6 | 322 | 295 | 27 | 543 | 542 | 0 |
| P316-PTdeep | Trunk | ASXL1 | 20 | + | Nonsense_Mutation | SNP | A | A | T | rs749831576 | c.2734A>T | p.Lys912Ter | p.K912* | NM_015338.6 | 285 | 211 | 74 | 395 | 395 | 0 |
| P316-PTsup | Trunk | TP53 | 17 | + | Missense_Mutation | SNP | G | G | T | rs876660254 | c.568C>A | p.Pro190Thr | p.P190T | NM_000546.5 | 279 | 89 | 189 | 285 | 283 | 0 |
| P316-PTsup | Trunk | SYNE1 | 6 | + | Missense_Mutation | SNP | C | C | G |  | c.23337G>C | p.Leu7779Phe | p.L7779F | NM_182961.4 | 423 | 230 | 191 | 267 | 267 | 0 |
| P316-PTsup | Trunk | SYNE1 | 6 | + | Splice_Site | SNP | T | T | C | novel | c.20068-2A>G |  | p.X6690_splice | NM_182961.4 | 413 | 210 | 202 | 363 | 363 | 0 |
| P316-PTsup | Branch | MYH2 | 17 | + | Missense_Mutation | SNP | C | C | G | novel | c.418G>C | p.Val140Leu | p.V140L | NM_017534.6 | 591 | 470 | 120 | 543 | 542 | 0 |
| P316-PTsup | Trunk | ASXL1 | 20 | + | Nonsense_Mutation | SNP | A | A | T | rs749831576 | c.2734A>T | p.Lys912Ter | p.K912* | NM_015338.6 | 574 | 247 | 327 | 395 | 395 | 0 |
| P348-LNmet | Trunk | ZNF750 | 17 | + | Missense_Mutation | SNP | C | C | T | rs1040401049 | c.20G>A | p.Arg7Gln | p.R7Q | NM_024702.3 | 104 | 71 | 33 | 203 | 201 | 0 |
| P348-LNmet | Trunk | TP53 | 17 | + | Missense_Mutation | SNP | C | C | A | rs121912655 | c.725G>T | p.Cys242Phe | p.C242F | NM_000546.5 | 103 | 34 | 69 | 146 | 145 | 1 |
| P348-LNmet | Branch | ROCK2 | 2 | + | Missense_Mutation | SNP | C | C | G | novel | c.3079G>C | p.Glu1027Gln | p.E1027Q | NM_004850.5 | 78 | 63 | 15 | 159 | 158 | 0 |
| P348-LNmet | Trunk | PCLO | 7 | + | Missense_Mutation | SNP | A | A | G | novel | c.13721T>C | p.Ile4574Thr | p.I4574T | NM_033026.6 | 63 | 41 | 22 | 102 | 102 | 0 |
| P348-LNmet | Branch | MLF1 | 3 | + | Missense_Mutation | SNP | A | A | C |  | c.897A>C | p.Lys299Asn | p.K299N | NM_001195432.2 | 173 | 145 | 27 | 169 | 169 | 0 |
| P348-LNmet | Trunk | NF1 | 17 | + | Nonsense_Mutation | SNP | C | C | T | rs137854559 | c.4021C>T | p.Gln1341Ter | p.Q1341* | NM_001042492.3 | 161 | 108 | 53 | 212 | 212 | 0 |
| P348-LNmet | Trunk | FAM135B | 8 | + | Missense_Mutation | SNP | G | G | C | novel | c.1677C>G | p.Ser559Arg | p.S559R | NM_015912.4 | 185 | 129 | 56 | 288 | 287 | 0 |
| P348-LNmet | Trunk | DSG1 | 18 | + | Nonsense_Mutation | SNP | C | C | G | novel | c.899C>G | p.Ser300Ter | p.S300* | NM_001942.4 | 123 | 94 | 29 | 127 | 126 | 0 |
| P348-LNmet | Branch | ASXL1 | 20 | + | Frame_Shift_Del | DEL | GA | GA | - | novel | c.1926_1927del | p.Gly645TrpfsTer12 | p.G645Wfs*12 | NM_015338.6 | 1696 | 1593 | 78 | 730 | 730 | 0 |
| P348-LNmet | Trunk | ASXL1 | 20 | + | Frame_Shift_Del | DEL | A | A | - |  | c.1926del | p.Gly645ValfsTer58 | p.G645Vfs*58 | NM_015338.6 | 1685 | 1012 | 380 | 729 | 722 | 1 |
| P348-PTsup | Trunk | ZNF750 | 17 | + | Missense_Mutation | SNP | C | C | T | rs1040401049 | c.20G>A | p.Arg7Gln | p.R7Q | NM_024702.3 | 317 | 305 | 12 | 203 | 201 | 0 |
| P348-PTsup | Trunk | ZNF750 | 17 | + | Missense_Mutation | SNP | C | C | T | rs1040401049 | c.20G>A | p.Arg7Gln | p.R7Q | NM_024702.3 | 135 | 106 | 29 | 203 | 201 | 0 |
| P348-PTsup | Trunk | TP53 | 17 | + | Missense_Mutation | SNP | C | C | A | rs121912655 | c.725G>T | p.Cys242Phe | p.C242F | NM_000546.5 | 507 | 438 | 67 | 146 | 145 | 1 |
| P348-PTsup | Trunk | TP53 | 17 | + | Missense_Mutation | SNP | C | C | A | rs121912655 | c.725G>T | p.Cys242Phe | p.C242F | NM_000546.5 | 154 | 83 | 70 | 146 | 145 | 1 |
| P348-PTsup | Trunk | PCLO | 7 | + | Missense_Mutation | SNP | A | A | G | novel | c.13721T>C | p.Ile4574Thr | p.I4574T | NM_033026.6 | 59 | 43 | 16 | 102 | 102 | 0 |
| P348-PTsup | Branch | MLF1 | 3 | + | Missense_Mutation | SNP | A | A | C |  | c.897A>C | p.Lys299Asn | p.K299N | NM_001195432.2 | 209 | 190 | 19 | 169 | 169 | 0 |
| P348-PTsup | Trunk | NF1 | 17 | + | Nonsense_Mutation | SNP | C | C | T | rs137854559 | c.4021C>T | p.Gln1341Ter | p.Q1341* | NM_001042492.3 | 508 | 483 | 25 | 212 | 212 | 0 |
| P348-PTsup | Trunk | NF1 | 17 | + | Nonsense_Mutation | SNP | C | C | T | rs137854559 | c.4021C>T | p.Gln1341Ter | p.Q1341* | NM_001042492.3 | 127 | 86 | 41 | 212 | 212 | 0 |
| P348-PTsup | Branch | FBXO11 | 2 | + | Missense_Mutation | SNP | T | T | A | novel | c.2418A>T | p.Leu806Phe | p.L806F | NM_001190274.1 | 348 | 324 | 24 | 148 | 148 | 0 |
| P348-PTsup | Trunk | FAM135B | 8 | + | Missense_Mutation | SNP | G | G | C | novel | c.1677C>G | p.Ser559Arg | p.S559R | NM_015912.4 | 545 | 505 | 37 | 288 | 287 | 0 |
| P348-PTsup | Trunk | FAM135B | 8 | + | Missense_Mutation | SNP | G | G | C | novel | c.1677C>G | p.Ser559Arg | p.S559R | NM_015912.4 | 181 | 150 | 31 | 288 | 287 | 0 |
| P348-PTsup | Trunk | DSG1 | 18 | + | Nonsense_Mutation | SNP | C | C | G | novel | c.899C>G | p.Ser300Ter | p.S300* | NM_001942.4 | 326 | 305 | 20 | 127 | 126 | 0 |
| P348-PTsup | Trunk | DSG1 | 18 | + | Nonsense_Mutation | SNP | C | C | G | novel | c.899C>G | p.Ser300Ter | p.S300* | NM_001942.4 | 87 | 73 | 14 | 127 | 126 | 0 |
| P348-PTsup | Branch | ASXL1 | 20 | + | Frame_Shift_Del | DEL | GA | GA | - | novel | c.1926_1927del | p.Gly645TrpfsTer12 | p.G645Wfs*12 | NM_015338.6 | 1540 | 1465 | 58 | 730 | 730 | 0 |
| P348-PTsup | Branch | ACVR2A | 2 | + | Frame_Shift_Ins | INS | - | - | A | novel | c.1310dup | p.Arg438GlufsTer19 | p.R438Efs*19 | NM_001616.5 | 460 | 448 | 12 | 229 | 229 | 0 |
| P348-PTsup | Trunk | ASXL1 | 20 | + | Frame_Shift_Del | DEL | A | A | - |  | c.1926del | p.Gly645ValfsTer58 | p.G645Vfs*58 | NM_015338.6 | 1885 | 1713 | 108 | 729 | 722 | 1 |
| P348-PTsup | Trunk | ASXL1 | 20 | + | Frame_Shift_Del | DEL | A | A | - |  | c.1926del | p.Gly645ValfsTer58 | p.G645Vfs*58 | NM_015338.6 | 1536 | 985 | 306 | 729 | 722 | 1 |
| P435-LNmet | Trunk | TP53 | 17 | + | Nonsense_Mutation | SNP | G | G | A | rs730882029 | c.1024C>T | p.Arg342Ter | p.R342* | NM_000546.5 | 406 | 288 | 118 | 92 | 92 | 0 |
| P435-LNmet | Trunk | TP53 | 17 | + | Splice_Site | SNP | A | A | G |  | c.993+2T>C |  | p.X331_splice | NM_000546.5 | 400 | 257 | 140 | 108 | 108 | 0 |
| P435-LNmet | Trunk | RB1 | 13 | + | Splice_Site | SNP | G | G | T |  | c.1390-1G>T |  | p.X464_splice | NM_000321.2 | 116 | 67 | 48 | 62 | 62 | 0 |
| P435-LNmet | Trunk | NFE2L2 | 2 | + | Missense_Mutation | SNP | T | T | A |  | c.230A>T | p.Asp77Val | p.D77V | NM_006164.5 | 427 | 204 | 223 | 162 | 162 | 0 |
| P435-LNmet | Trunk | MUC16 | 19 | + | Missense_Mutation | SNP | C | C | T | rs75665802 | c.8064G>A | p.Met2688Ile | p.M2688I | NM_024690.2 | 156 | 93 | 63 | 50 | 50 | 0 |
| P435-LNmet | Trunk | DDB2 | 11 | + | Nonsense_Mutation | SNP | C | C | T | novel | c.274C>T | p.Gln92Ter | p.Q92* | NM_000107.2 | 390 | 325 | 65 | 93 | 92 | 0 |
| P435-LNmet | Trunk | ATP1A1 | 1 | + | Missense_Mutation | SNP | T | T | G | novel | c.1006T>G | p.Leu336Val | p.L336V | NM_001160233.1 | 610 | 399 | 211 | 181 | 181 | 0 |
| P435-PTdeep | Trunk | TP53 | 17 | + | Nonsense_Mutation | SNP | G | G | A | rs730882029 | c.1024C>T | p.Arg342Ter | p.R342* | NM_000546.5 | 355 | 324 | 30 | 92 | 92 | 0 |
| P435-PTdeep | Trunk | TP53 | 17 | + | Splice_Site | SNP | A | A | G |  | c.993+2T>C |  | p.X331_splice | NM_000546.5 | 335 | 300 | 33 | 108 | 108 | 0 |
| P435-PTdeep | Trunk | NFE2L2 | 2 | + | Missense_Mutation | SNP | T | T | A |  | c.230A>T | p.Asp77Val | p.D77V | NM_006164.5 | 512 | 450 | 62 | 162 | 162 | 0 |
| P435-PTdeep | Trunk | MUC16 | 19 | + | Missense_Mutation | SNP | C | C | T | rs75665802 | c.8064G>A | p.Met2688Ile | p.M2688I | NM_024690.2 | 178 | 148 | 30 | 50 | 50 | 0 |
| P435-PTdeep | Trunk | DDB2 | 11 | + | Nonsense_Mutation | SNP | C | C | T | novel | c.274C>T | p.Gln92Ter | p.Q92* | NM_000107.2 | 365 | 339 | 26 | 93 | 92 | 0 |
| P435-PTdeep | Trunk | ATP1A1 | 1 | + | Missense_Mutation | SNP | T | T | G | novel | c.1006T>G | p.Leu336Val | p.L336V | NM_001160233.1 | 666 | 585 | 79 | 181 | 181 | 0 |
| P435-PTsup | Branch | RYR2 | 1 | + | Frame_Shift_Del | DEL | GG | GG | - | novel | c.3962_3963del | p.Gly1321AlafsTer12 | p.G1321Afs*12 | NM_001035.3 | 541 | 508 | 32 | 193 | 192 | 0 |
| P435-PTsup | Trunk | TP53 | 17 | + | Nonsense_Mutation | SNP | G | G | A | rs730882029 | c.1024C>T | p.Arg342Ter | p.R342* | NM_000546.5 | 227 | 144 | 83 | 92 | 92 | 0 |
| P435-PTsup | Trunk | TP53 | 17 | + | Splice_Site | SNP | A | A | G |  | c.993+2T>C |  | p.X331_splice | NM_000546.5 | 230 | 152 | 78 | 108 | 108 | 0 |
| P435-PTsup | Trunk | RB1 | 13 | + | Splice_Site | SNP | G | G | T |  | c.1390-1G>T |  | p.X464_splice | NM_000321.2 | 41 | 17 | 24 | 62 | 62 | 0 |
| P435-PTsup | Trunk | NFE2L2 | 2 | + | Missense_Mutation | SNP | T | T | A |  | c.230A>T | p.Asp77Val | p.D77V | NM_006164.5 | 212 | 67 | 144 | 162 | 162 | 0 |
| P435-PTsup | Trunk | MUC16 | 19 | + | Missense_Mutation | SNP | C | C | T | rs75665802 | c.8064G>A | p.Met2688Ile | p.M2688I | NM_024690.2 | 49 | 19 | 30 | 50 | 50 | 0 |
| P435-PTsup | Trunk | DDB2 | 11 | + | Nonsense_Mutation | SNP | C | C | T | novel | c.274C>T | p.Gln92Ter | p.Q92* | NM_000107.2 | 174 | 116 | 58 | 93 | 92 | 0 |
| P435-PTsup | Trunk | ATP1A1 | 1 | + | Missense_Mutation | SNP | T | T | G | novel | c.1006T>G | p.Leu336Val | p.L336V | NM_001160233.1 | 306 | 155 | 151 | 181 | 181 | 0 |
| P481-LNmet | Trunk | SLIT2 | 4 | + | Missense_Mutation | SNP | C | C | T | novel | c.2555C>T | p.Ser852Leu | p.S852L | NM_004787.4 | 79 | 60 | 19 | 72 | 72 | 0 |
| P481-LNmet | Branch | NF1 | 17 | + | Nonsense_Mutation | SNP | C | C | T | rs137854562 | c.3721C>T | p.Arg1241Ter | p.R1241* | NM_001042492.3 | 200 | 193 | 7 | 189 | 188 | 0 |
| P481-LNmet | Branch | LMNA | 1 | + | Missense_Mutation | SNP | C | C | T | rs58133342 | c.1162C>T | p.Arg388Cys | p.R388C | NM_170708.3 | 874 | 853 | 19 | 306 | 306 | 0 |
| P481-LNmet | Trunk | MUC16 | 19 | + | Missense_Mutation | SNP | G | G | A | novel | c.2233C>T | p.His745Tyr | p.H745Y | NM_024690.2 | 242 | 193 | 49 | 212 | 212 | 0 |
| P481-LNmet | Trunk | LAMA1 | 18 | + | Missense_Mutation | SNP | C | C | T | novel | c.7663G>A | p.Glu2555Lys | p.E2555K | NM_005559.4 | 309 | 272 | 36 | 199 | 199 | 0 |
| P481-LNmet | Trunk | KMT2C | 7 | + | Missense_Mutation | SNP | C | C | G | novel | c.13981G>C | p.Glu4661Gln | p.E4661Q | NM_170606.3 | 136 | 109 | 26 | 164 | 163 | 0 |
| P481-LNmet | Trunk | HERC1 | 15 | + | Missense_Mutation | SNP | G | G | A | rs865926938 | c.4342C>T | p.Arg1448Trp | p.R1448W | NM_003922.4 | 120 | 86 | 34 | 183 | 183 | 0 |
| P481-LNmet | Trunk | CDKN2A | 9 | + | Missense_Mutation | SNP | C | C | T | rs121913381 | c.322G>A | p.Asp108Asn | p.D108N | NM_000077.4 | 1043 | 316 | 725 | 812 | 811 | 1 |
| P481-LNmet | Branch | ABCA7 | 19 | + | Missense_Mutation | SNP | G | G | A | rs202238511 | c.5339G>A | p.Arg1780Gln | p.R1780Q | NM_019112.4 | 208 | 156 | 49 | 328 | 326 | 0 |
| P481-LNmet | Trunk | ACACB | 12 | + | Missense_Mutation | SNP | A | A | G | novel | c.5869A>G | p.Ile1957Val | p.I1957V | NM_001093.4 | 160 | 124 | 35 | 152 | 152 | 0 |
| P481-PTsup | Trunk | SLIT2 | 4 | + | Missense_Mutation | SNP | C | C | T | novel | c.2555C>T | p.Ser852Leu | p.S852L | NM_004787.4 | 99 | 80 | 19 | 72 | 72 | 0 |
| P481-PTsup | Trunk | MUC16 | 19 | + | Missense_Mutation | SNP | G | G | A | rs1446280776 | c.16898C>T | p.Ser5633Phe | p.S5633F | NM_024690.2 | 85 | 71 | 13 | 145 | 145 | 0 |
| P481-PTsup | Trunk | MUC16 | 19 | + | Missense_Mutation | SNP | G | G | A | rs1446280776 | c.16898C>T | p.Ser5633Phe | p.S5633F | NM_024690.2 | 91 | 76 | 15 | 145 | 145 | 0 |
| P481-PTsup | Trunk | MUC16 | 19 | + | Missense_Mutation | SNP | G | G | A | novel | c.2233C>T | p.His745Tyr | p.H745Y | NM_024690.2 | 232 | 187 | 45 | 212 | 212 | 0 |
| P481-PTsup | Trunk | LAMA1 | 18 | + | Missense_Mutation | SNP | C | C | T | novel | c.7663G>A | p.Glu2555Lys | p.E2555K | NM_005559.4 | 146 | 132 | 13 | 199 | 199 | 0 |
| P481-PTsup | Trunk | LAMA1 | 18 | + | Missense_Mutation | SNP | C | C | T | novel | c.7663G>A | p.Glu2555Lys | p.E2555K | NM_005559.4 | 240 | 217 | 23 | 199 | 199 | 0 |
| P481-PTsup | Trunk | KMT2C | 7 | + | Missense_Mutation | SNP | C | C | G | novel | c.13981G>C | p.Glu4661Gln | p.E4661Q | NM_170606.3 | 138 | 109 | 28 | 164 | 163 | 0 |
| P481-PTsup | Trunk | HERC1 | 15 | + | Missense_Mutation | SNP | G | G | A | rs865926938 | c.4342C>T | p.Arg1448Trp | p.R1448W | NM_003922.4 | 86 | 71 | 15 | 183 | 183 | 0 |
| P481-PTsup | Trunk | HERC1 | 15 | + | Missense_Mutation | SNP | G | G | A | rs865926938 | c.4342C>T | p.Arg1448Trp | p.R1448W | NM_003922.4 | 127 | 98 | 28 | 183 | 183 | 0 |
| P481-PTsup | Trunk | CDKN2A | 9 | + | Missense_Mutation | SNP | C | C | T | rs121913381 | c.322G>A | p.Asp108Asn | p.D108N | NM_000077.4 | 1053 | 779 | 272 | 812 | 811 | 1 |
| P481-PTsup | Trunk | CDKN2A | 9 | + | Missense_Mutation | SNP | C | C | T | rs121913381 | c.322G>A | p.Asp108Asn | p.D108N | NM_000077.4 | 1463 | 798 | 663 | 812 | 811 | 1 |
| P481-PTsup | Trunk | ACACB | 12 | + | Missense_Mutation | SNP | A | A | G | novel | c.5869A>G | p.Ile1957Val | p.I1957V | NM_001093.4 | 169 | 150 | 19 | 152 | 152 | 0 |
| P481-PTsup | Branch | ABCA7 | 19 | + | Missense_Mutation | SNP | G | G | A | rs202238511 | c.5339G>A | p.Arg1780Gln | p.R1780Q | NM_019112.4 | 157 | 147 | 10 | 328 | 326 | 0 |
| P575-LNmet | Trunk | TP53 | 17 | + | Nonsense_Mutation | SNP | C | C | A | rs201744589 | c.892G>T | p.Glu298Ter | p.E298* | NM_000546.5 | 118 | 91 | 27 | 353 | 353 | 0 |
| P575-LNmet | Branch | SUFU | 10 | + | Missense_Mutation | SNP | C | C | T | rs751728820 | c.1027C>T | p.Arg343Cys | p.R343C | NM_016169.3 | 143 | 116 | 27 | 336 | 336 | 0 |
| P575-LNmet | Trunk | TGFBR2 | 3 | + | Missense_Mutation | SNP | G | G | A |  | c.1393G>A | p.Glu465Lys | p.E465K | NM_001024847.2 | 101 | 71 | 30 | 288 | 288 | 0 |
| P575-LNmet | Trunk | TG | 8 | + | Missense_Mutation | SNP | G | G | T | novel | c.2693G>T | p.Cys898Phe | p.C898F | NM_003235.5 | 257 | 226 | 31 | 545 | 544 | 0 |
| P575-LNmet | Trunk | HERC1 | 15 | + | Frame_Shift_Del | DEL | T | T | - | novel | c.10607del | p.Glu3536GlyfsTer26 | p.E3536Gfs*26 | NM_003922.4 | 112 | 86 | 25 | 197 | 197 | 0 |
| P575-PTdeep | Trunk | TP53 | 17 | + | Nonsense_Mutation | SNP | C | C | A | rs201744589 | c.892G>T | p.Glu298Ter | p.E298* | NM_000546.5 | 333 | 151 | 182 | 353 | 353 | 0 |
| P575-PTdeep | Trunk | TGFBR2 | 3 | + | Missense_Mutation | SNP | G | G | A |  | c.1393G>A | p.Glu465Lys | p.E465K | NM_001024847.2 | 265 | 139 | 126 | 288 | 288 | 0 |
| P575-PTdeep | Trunk | TG | 8 | + | Missense_Mutation | SNP | G | G | T | novel | c.2693G>T | p.Cys898Phe | p.C898F | NM_003235.5 | 874 | 648 | 225 | 545 | 544 | 0 |
| P575-PTdeep | Branch | PTPRB | 12 | + | Missense_Mutation | SNP | G | G | T | novel | c.3638C>A | p.Pro1213Gln | p.P1213Q | NM_001109754.4 | 379 | 237 | 137 | 260 | 260 | 0 |
| P575-PTdeep | Branch | NCOR1 | 17 | + | Missense_Mutation | SNP | G | G | C |  | c.4535C>G | p.Ser1512Cys | p.S1512C | NM_006311.4 | 219 | 102 | 116 | 227 | 226 | 0 |
| P575-PTdeep | Branch | EYS | 6 | + | Frame_Shift_Del | DEL | A | A | - | rs786205652 | c.179del | p.Leu60TrpfsTer3 | p.L60Wfs*3 | NM_001292009.1 | 376 | 366 | 10 | 248 | 248 | 0 |
| P575-PTdeep | Trunk | HERC1 | 15 | + | Frame_Shift_Del | DEL | T | T | - | novel | c.10607del | p.Glu3536GlyfsTer26 | p.E3536Gfs*26 | NM_003922.4 | 354 | 230 | 119 | 197 | 197 | 0 |
| P575-PTdeep | Branch | DNAH7 | 2 | + | Frame_Shift_Ins | INS | - | - | T | rs34468832 | c.3770dup | p.Asn1257LysfsTer3 | p.N1257Kfs*3 | NM_018897.3 | 494 | 482 | 12 | 298 | 298 | 0 |
| P575-PTsup | Trunk | TP53 | 17 | + | Nonsense_Mutation | SNP | C | C | A | rs201744589 | c.892G>T | p.Glu298Ter | p.E298* | NM_000546.5 | 151 | 111 | 40 | 353 | 353 | 0 |
| P575-PTsup | Trunk | TGFBR2 | 3 | + | Missense_Mutation | SNP | G | G | A |  | c.1393G>A | p.Glu465Lys | p.E465K | NM_001024847.2 | 117 | 71 | 46 | 288 | 288 | 0 |
| P575-PTsup | Trunk | TG | 8 | + | Missense_Mutation | SNP | G | G | T | novel | c.2693G>T | p.Cys898Phe | p.C898F | NM_003235.5 | 471 | 383 | 84 | 545 | 544 | 0 |
| P575-PTsup | Branch | NFE2L2 | 2 | + | Missense_Mutation | SNP | T | T | G |  | c.77A>C | p.Gln26Pro | p.Q26P | NM_006164.5 | 251 | 201 | 49 | 262 | 262 | 0 |
| P575-PTsup | Trunk | HERC1 | 15 | + | Frame_Shift_Del | DEL | T | T | - | novel | c.10607del | p.Glu3536GlyfsTer26 | p.E3536Gfs*26 | NM_003922.4 | 98 | 82 | 16 | 197 | 197 | 0 |
| P685-LNmet | Trunk | USH2A | 1 | + | Missense_Mutation | SNP | C | C | A | novel | c.2717G>T | p.Gly906Val | p.G906V | NM_206933.3 | 410 | 297 | 112 | 294 | 294 | 0 |
| P685-LNmet | Branch | SPTAN1 | 9 | + | Missense_Mutation | SNP | C | C | G | novel | c.5821C>G | p.Leu1941Val | p.L1941V | NM_001363759.2 | 221 | 177 | 44 | 179 | 179 | 0 |
| P685-LNmet | Trunk | TP53 | 17 | + | Nonsense_Mutation | SNP | G | G | A | rs764735889 | c.949C>T | p.Gln317Ter | p.Q317* | NM_000546.5 | 286 | 209 | 76 | 268 | 266 | 0 |
| P685-LNmet | Trunk | NUP214 | 9 | + | Missense_Mutation | SNP | G | G | A | rs770633748 | c.5983G>A | p.Gly1995Ser | p.G1995S | NM_005085.4 | 374 | 271 | 103 | 331 | 329 | 1 |
| P685-LNmet | Branch | CDH11 | 16 | + | Missense_Mutation | SNP | G | G | T | novel | c.2039C>A | p.Thr680Asn | p.T680N | NM_001797.4 | 229 | 167 | 62 | 156 | 156 | 0 |
| P685-LNmet | Trunk | CBFB | 16 | + | Nonsense_Mutation | SNP | G | G | T | novel | c.265G>T | p.Glu89Ter | p.E89* | NM_022845.3 | 204 | 100 | 104 | 151 | 151 | 0 |
| P685-LNmet | Trunk | CAMTA1 | 1 | + | Missense_Mutation | SNP | C | C | T | rs750058404 | c.1127C>T | p.Pro376Leu | p.P376L | NM_015215.4 | 718 | 550 | 166 | 345 | 345 | 0 |
| P685-LNmet | Trunk | ASXL1 | 20 | + | Frame_Shift_Ins | INS | - | - | TAGA | novel | c.2468_2471dup | p.Glu824AspfsTer10 | p.E824Dfs*10 | NM_015338.6 | 409 | 222 | 183 | 280 | 280 | 0 |
| P685-PTdeep | Trunk | TP53 | 17 | + | Nonsense_Mutation | SNP | G | G | A | rs764735889 | c.949C>T | p.Gln317Ter | p.Q317* | NM_000546.5 | 485 | 474 | 10 | 268 | 266 | 0 |
| P685-PTdeep | Branch | F8 | X | + | Frame_Shift_Ins | INS | - | - | T | rs387906450 | c.3637dup | p.Ile1213AsnfsTer28 | p.I1213Nfs*28 | NM_000132.3 | 244 | 230 | 10 | 181 | 181 | 0 |
| P685-PTdeep | Branch | CIC | 19 | + | Missense_Mutation | SNP | T | T | C | novel | c.7204T>C | p.Phe2402Leu | p.F2402L | NM_001304815.1 | 533 | 518 | 11 | 444 | 444 | 0 |
| P685-PTdeep | Branch | CIC | 19 | + | Missense_Mutation | SNP | G | G | A | rs200799936 | c.7214G>A | p.Arg2405His | p.R2405H | NM_001304815.1 | 529 | 507 | 13 | 446 | 442 | 1 |
| P685-PTdeep | Trunk | ASXL1 | 20 | + | Frame_Shift_Ins | INS | - | - | TAGA | novel | c.2468_2471dup | p.Glu824AspfsTer10 | p.E824Dfs*10 | NM_015338.6 | 286 | 270 | 16 | 280 | 280 | 0 |
| P685-PTsup | Trunk | USH2A | 1 | + | Missense_Mutation | SNP | C | C | A | novel | c.2717G>T | p.Gly906Val | p.G906V | NM_206933.3 | 376 | 327 | 48 | 294 | 294 | 0 |
| P685-PTsup | Branch | SPTAN1 | 9 | + | Missense_Mutation | SNP | C | C | G | novel | c.5821C>G | p.Leu1941Val | p.L1941V | NM_001363759.2 | 124 | 105 | 19 | 179 | 179 | 0 |
| P685-PTsup | Trunk | TP53 | 17 | + | Nonsense_Mutation | SNP | G | G | A | rs764735889 | c.949C>T | p.Gln317Ter | p.Q317* | NM_000546.5 | 285 | 232 | 53 | 268 | 266 | 0 |
| P685-PTsup | Trunk | NUP214 | 9 | + | Missense_Mutation | SNP | G | G | A | rs770633748 | c.5983G>A | p.Gly1995Ser | p.G1995S | NM_005085.4 | 275 | 229 | 44 | 331 | 329 | 1 |
| P685-PTsup | Branch | CDH11 | 16 | + | Missense_Mutation | SNP | G | G | T | novel | c.2039C>A | p.Thr680Asn | p.T680N | NM_001797.4 | 146 | 126 | 20 | 156 | 156 | 0 |
| P685-PTsup | Trunk | CBFB | 16 | + | Nonsense_Mutation | SNP | G | G | T | novel | c.265G>T | p.Glu89Ter | p.E89* | NM_022845.3 | 141 | 95 | 45 | 151 | 151 | 0 |
| P685-PTsup | Trunk | CAMTA1 | 1 | + | Missense_Mutation | SNP | C | C | T | rs750058404 | c.1127C>T | p.Pro376Leu | p.P376L | NM_015215.4 | 494 | 418 | 75 | 345 | 345 | 0 |
| P685-PTsup | Trunk | ASXL1 | 20 | + | Frame_Shift_Ins | INS | - | - | TAGA | novel | c.2468_2471dup | p.Glu824AspfsTer10 | p.E824Dfs*10 | NM_015338.6 | 327 | 216 | 107 | 280 | 280 | 0 |
| P879-LNmet | Trunk | UBR5 | 8 | + | Missense_Mutation | SNP | A | A | C | novel | c.6709T>G | p.Ser2237Ala | p.S2237A | NM_015902.6 | 233 | 148 | 83 | 107 | 106 | 0 |
| P879-LNmet | Trunk | TERT | 5 | + | Frame_Shift_Del | DEL | ATCAGCCA | ATCAGCCA | - | novel | c.1639_1646del | p.Trp547GlufsTer79 | p.W547Efs*79 | NM_198253.3 | 285 | 214 | 69 | 209 | 209 | 0 |
| P879-LNmet | Trunk | PTEN | 10 | + | Frame_Shift_Del | DEL | CTTTGAGTTC | CTTTGAGTTC | - | novel | c.721_730del | p.Phe241LeufsTer12 | p.F241Lfs*12 | NM_000314.8 | 105 | 74 | 31 | 135 | 135 | 0 |
| P879-LNmet | Branch | NIN | 14 | + | Missense_Mutation | SNP | G | G | A | rs757331038 | c.3457C>T | p.Arg1153Trp | p.R1153W | NM_020921.3 | 227 | 195 | 32 | 166 | 165 | 0 |
| P879-LNmet | Trunk | NUP98 | 11 | + | Missense_Mutation | SNP | C | C | T | rs987383683 | c.3380G>A | p.Arg1127His | p.R1127H | NM_016320.5 | 206 | 165 | 41 | 150 | 150 | 0 |
| P879-LNmet | Trunk | NTRK3 | 15 | + | Splice_Site | SNP | C | C | A |  | c.1586-1G>T |  | p.X529_splice | NM_001012338.2 | 469 | 450 | 18 | 364 | 364 | 0 |
| P879-LNmet | Trunk | NOTCH1 | 9 | + | Missense_Mutation | SNP | G | G | A |  | c.1172C>T | p.Pro391Leu | p.P391L | NM_017617.5 | 911 | 672 | 238 | 359 | 359 | 0 |
| P879-LNmet | Trunk | NOTCH1 | 9 | + | Missense_Mutation | SNP | G | G | A |  | c.1154C>T | p.Ser385Phe | p.S385F | NM_017617.5 | 849 | 400 | 445 | 359 | 359 | 0 |
| P879-LNmet | Trunk | KMT2D | 12 | + | Frame_Shift_Del | DEL | T | T | - |  | c.5485del | p.Ile1829LeufsTer23 | p.I1829Lfs*23 | NM_003482.3 | 156 | 113 | 43 | 88 | 88 | 0 |
| P879-LNmet | Trunk | FOXO1 | 13 | + | Missense_Mutation | SNP | G | G | A |  | c.71C>T | p.Thr24Ile | p.T24I | NM_002015.4 | 451 | 323 | 128 | 398 | 398 | 0 |
| P879-LNmet | Trunk | FIP1L1 | 4 | + | Missense_Mutation | SNP | A | A | T | novel | c.541A>T | p.Asn181Tyr | p.N181Y | NM_030917.4 | 124 | 56 | 67 | 79 | 79 | 0 |
| P879-LNmet | Trunk | CNTRL | 9 | + | Frame_Shift_Ins | INS | - | - | A | rs747099268 | c.1336dup | p.Ile446AsnfsTer13 | p.I446Nfs*13 | NM_007018.5 | 448 | 431 | 13 | 188 | 185 | 0 |
| P879-LNmet | Trunk | CDKN2A | 9 | + | Nonsense_Mutation | SNP | G | G | A | rs121913387 | c.172C>T | p.Arg58Ter | p.R58* | NM_000077.4 | 562 | 225 | 332 | 461 | 461 | 0 |
| P879-PTdeep | Trunk | UBR5 | 8 | + | Missense_Mutation | SNP | A | A | C | novel | c.6709T>G | p.Ser2237Ala | p.S2237A | NM_015902.6 | 417 | 366 | 51 | 107 | 106 | 0 |
| P879-PTdeep | Trunk | TERT | 5 | + | Frame_Shift_Del | DEL | ATCAGCCA | ATCAGCCA | - | novel | c.1639_1646del | p.Trp547GlufsTer79 | p.W547Efs*79 | NM_198253.3 | 478 | 422 | 53 | 209 | 209 | 0 |
| P879-PTdeep | Trunk | PTEN | 10 | + | Frame_Shift_Del | DEL | CTTTGAGTTC | CTTTGAGTTC | - | novel | c.721_730del | p.Phe241LeufsTer12 | p.F241Lfs*12 | NM_000314.8 | 416 | 296 | 120 | 135 | 135 | 0 |
| P879-PTdeep | Trunk | NUP98 | 11 | + | Missense_Mutation | SNP | C | C | T | rs987383683 | c.3380G>A | p.Arg1127His | p.R1127H | NM_016320.5 | 418 | 350 | 67 | 150 | 150 | 0 |
| P879-PTdeep | Trunk | NTRK3 | 15 | + | Splice_Site | SNP | C | C | A |  | c.1586-1G>T |  | p.X529_splice | NM_001012338.2 | 787 | 760 | 27 | 364 | 364 | 0 |
| P879-PTdeep | Trunk | NOTCH1 | 9 | + | Missense_Mutation | SNP | G | G | A |  | c.1172C>T | p.Pro391Leu | p.P391L | NM_017617.5 | 1293 | 1078 | 214 | 359 | 359 | 0 |
| P879-PTdeep | Trunk | NOTCH1 | 9 | + | Missense_Mutation | SNP | G | G | A |  | c.1154C>T | p.Ser385Phe | p.S385F | NM_017617.5 | 1276 | 886 | 390 | 359 | 359 | 0 |
| P879-PTdeep | Trunk | KMT2D | 12 | + | Frame_Shift_Del | DEL | T | T | - |  | c.5485del | p.Ile1829LeufsTer23 | p.I1829Lfs*23 | NM_003482.3 | 287 | 236 | 51 | 88 | 88 | 0 |
| P879-PTdeep | Trunk | FOXO1 | 13 | + | Missense_Mutation | SNP | G | G | A |  | c.71C>T | p.Thr24Ile | p.T24I | NM_002015.4 | 1135 | 899 | 235 | 398 | 398 | 0 |
| P879-PTdeep | Trunk | FIP1L1 | 4 | + | Missense_Mutation | SNP | A | A | T | novel | c.541A>T | p.Asn181Tyr | p.N181Y | NM_030917.4 | 266 | 219 | 47 | 79 | 79 | 0 |
| P879-PTdeep | Trunk | CDKN2A | 9 | + | Nonsense_Mutation | SNP | G | G | A | rs121913387 | c.172C>T | p.Arg58Ter | p.R58* | NM_000077.4 | 1216 | 864 | 352 | 461 | 461 | 0 |
| P879-PTsup | Trunk | UBR5 | 8 | + | Missense_Mutation | SNP | A | A | C | novel | c.6709T>G | p.Ser2237Ala | p.S2237A | NM_015902.6 | 349 | 224 | 125 | 107 | 106 | 0 |
| P879-PTsup | Trunk | TERT | 5 | + | Frame_Shift_Del | DEL | ATCAGCCA | ATCAGCCA | - | novel | c.1639_1646del | p.Trp547GlufsTer79 | p.W547Efs*79 | NM_198253.3 | 612 | 474 | 135 | 209 | 209 | 0 |
| P879-PTsup | Trunk | PTEN | 10 | + | Frame_Shift_Del | DEL | CTTTGAGTTC | CTTTGAGTTC | - | novel | c.721_730del | p.Phe241LeufsTer12 | p.F241Lfs*12 | NM_000314.8 | 400 | 144 | 254 | 135 | 135 | 0 |
| P879-PTsup | Trunk | NUP98 | 11 | + | Missense_Mutation | SNP | C | C | T | rs987383683 | c.3380G>A | p.Arg1127His | p.R1127H | NM_016320.5 | 406 | 258 | 147 | 150 | 150 | 0 |
| P879-PTsup | Trunk | NTRK3 | 15 | + | Splice_Site | SNP | C | C | A |  | c.1586-1G>T |  | p.X529_splice | NM_001012338.2 | 934 | 861 | 70 | 364 | 364 | 0 |
| P879-PTsup | Trunk | NOTCH1 | 9 | + | Missense_Mutation | SNP | G | G | A |  | c.1172C>T | p.Pro391Leu | p.P391L | NM_017617.5 | 1227 | 880 | 347 | 359 | 359 | 0 |
| P879-PTsup | Trunk | NOTCH1 | 9 | + | Missense_Mutation | SNP | G | G | A |  | c.1154C>T | p.Ser385Phe | p.S385F | NM_017617.5 | 1213 | 507 | 706 | 359 | 359 | 0 |
| P879-PTsup | Trunk | KMT2D | 12 | + | Frame_Shift_Del | DEL | T | T | - |  | c.5485del | p.Ile1829LeufsTer23 | p.I1829Lfs*23 | NM_003482.3 | 258 | 170 | 87 | 88 | 88 | 0 |
| P879-PTsup | Trunk | FOXO1 | 13 | + | Missense_Mutation | SNP | G | G | A |  | c.71C>T | p.Thr24Ile | p.T24I | NM_002015.4 | 822 | 480 | 339 | 398 | 398 | 0 |
| P879-PTsup | Branch | DNAH7 | 2 | + | Frame_Shift_Ins | INS | - | - | T | rs34468832 | c.3770dup | p.Asn1257LysfsTer3 | p.N1257Kfs*3 | NM_018897.3 | 437 | 419 | 18 | 143 | 143 | 0 |
| P879-PTsup | Trunk | FIP1L1 | 4 | + | Missense_Mutation | SNP | A | A | T | novel | c.541A>T | p.Asn181Tyr | p.N181Y | NM_030917.4 | 150 | 53 | 97 | 79 | 79 | 0 |
| P879-PTsup | Trunk | CNTRL | 9 | + | Frame_Shift_Ins | INS | - | - | A | rs747099268 | c.1336dup | p.Ile446AsnfsTer13 | p.I446Nfs*13 | NM_007018.5 | 739 | 703 | 22 | 188 | 185 | 0 |
| P879-PTsup | Trunk | CDKN2A | 9 | + | Nonsense_Mutation | SNP | G | G | A | rs121913387 | c.172C>T | p.Arg58Ter | p.R58* | NM_000077.4 | 793 | 186 | 606 | 461 | 461 | 0 |
| P926-LNmet | Trunk | ZNF521 | 18 | + | Missense_Mutation | SNP | T | T | A | novel | c.3461A>T | p.Gln1154Leu | p.Q1154L | NM_015461.3 | 338 | 289 | 49 | 372 | 371 | 0 |
| P926-LNmet | Trunk | TP53 | 17 | + | Missense_Mutation | SNP | C | C | T | rs587781288 | c.422G>A | p.Cys141Tyr | p.C141Y | NM_000546.5 | 640 | 404 | 235 | 402 | 401 | 1 |
| P926-LNmet | Trunk | NOTCH1 | 9 | + | Missense_Mutation | SNP | C | C | A |  | c.4636G>T | p.Asp1546Tyr | p.D1546Y | NM_017617.5 | 1282 | 1122 | 156 | 695 | 694 | 0 |
| P926-LNmet | Trunk | LATS2 | 13 | + | In_Frame_Ins | INS | - | - | GGGGCG | rs550642106 | c.1431_1436dup | p.Pro479_Ala480dup | p.P479_A480dup | NM_014572.3 | 218 | 131 | 72 | 74 | 69 | 3 |
| P926-LNmet | Trunk | GRIA2 | 4 | + | Missense_Mutation | SNP | C | C | T |  | c.1132C>T | p.Leu378Phe | p.L378F | NM_001083619.1 | 149 | 136 | 13 | 93 | 93 | 0 |
| P926-LNmet | Branch | BMPR1A | 10 | + | Missense_Mutation | SNP | G | G | A | rs535109719 | c.1439G>A | p.Arg480Gln | p.R480Q | NM_004329.2 | 427 | 392 | 35 | 452 | 451 | 0 |
| P926-LNmet | Trunk | CSMD3 | 8 | + | Missense_Mutation | SNP | A | A | T | novel | c.8713T>A | p.Tyr2905Asn | p.Y2905N | NM_198123.2 | 288 | 258 | 30 | 196 | 196 | 0 |
| P926-LNmet | Trunk | CDKN2A | 9 | + | Frame_Shift_Del | DEL | ACCTCCTCTACCCGACCCCGGGCCGCGGCCGT | ACCTCCTCTACCCGACCCCGGGCCGCGGCCGT | - |  | c.52_83del | p.Thr18AlafsTer15 | p.T18Afs*15 | NM_000077.4 | 626 | 551 | 69 | 523 | 516 | 1 |
| P926-LNmet | Trunk | ARHGAP5 | 14 | + | Missense_Mutation | SNP | G | G | A | rs78337553 | c.1465G>A | p.Glu489Lys | p.E489K | NM_001030055.2 | 590 | 312 | 278 | 301 | 288 | 13 |
| P926-PTdeep | Trunk | ZNF521 | 18 | + | Missense_Mutation | SNP | T | T | A | novel | c.3461A>T | p.Gln1154Leu | p.Q1154L | NM_015461.3 | 337 | 217 | 120 | 372 | 371 | 0 |
| P926-PTdeep | Branch | TNFAIP3 | 6 | + | Splice_Site | SNP | G | G | C | novel | c.635-1G>C |  | p.X212_splice | NM_001270508.2 | 219 | 193 | 25 | 200 | 200 | 0 |
| P926-PTdeep | Trunk | TP53 | 17 | + | Missense_Mutation | SNP | C | C | T | rs587781288 | c.422G>A | p.Cys141Tyr | p.C141Y | NM_000546.5 | 493 | 200 | 293 | 402 | 401 | 1 |
| P926-PTdeep | Trunk | NOTCH1 | 9 | + | Missense_Mutation | SNP | C | C | A |  | c.4636G>T | p.Asp1546Tyr | p.D1546Y | NM_017617.5 | 2087 | 1656 | 429 | 695 | 694 | 0 |
| P926-PTdeep | Branch | KDM5A | 12 | + | Frame_Shift_Ins | INS | - | - | T | rs771545848 | c.3597dup | p.Gly1200ArgfsTer7 | p.G1200Rfs*7 | NM_001042603.3 | 447 | 436 | 11 | 233 | 233 | 0 |
| P926-PTdeep | Trunk | LATS2 | 13 | + | In_Frame_Ins | INS | - | - | GGGGCG | rs550642106 | c.1431_1436dup | p.Pro479_Ala480dup | p.P479_A480dup | NM_014572.3 | 377 | 236 | 116 | 74 | 69 | 3 |
| P926-PTdeep | Trunk | GRIA2 | 4 | + | Missense_Mutation | SNP | C | C | T |  | c.1132C>T | p.Leu378Phe | p.L378F | NM_001083619.1 | 124 | 82 | 41 | 93 | 93 | 0 |
| P926-PTdeep | Branch | CSMD3 | 8 | + | Missense_Mutation | SNP | T | T | G | novel | c.6449A>C | p.His2150Pro | p.H2150P | NM_198123.2 | 322 | 310 | 12 | 150 | 150 | 0 |
| P926-PTdeep | Trunk | CDKN2A | 9 | + | Frame_Shift_Del | DEL | ACCTCCTCTACCCGACCCCGGGCCGCGGCCGT | ACCTCCTCTACCCGACCCCGGGCCGCGGCCGT | - |  | c.52_83del | p.Thr18AlafsTer15 | p.T18Afs*15 | NM_000077.4 | 731 | 497 | 223 | 523 | 516 | 1 |
| P926-PTsup | Trunk | ZNF521 | 18 | + | Missense_Mutation | SNP | T | T | A | novel | c.3461A>T | p.Gln1154Leu | p.Q1154L | NM_015461.3 | 608 | 516 | 92 | 372 | 371 | 0 |
| P926-PTsup | Branch | TNFAIP3 | 6 | + | Splice_Site | SNP | G | G | C | novel | c.635-1G>C |  | p.X212_splice | NM_001270508.2 | 228 | 210 | 18 | 200 | 200 | 0 |
| P926-PTsup | Trunk | TP53 | 17 | + | Missense_Mutation | SNP | C | C | T | rs587781288 | c.422G>A | p.Cys141Tyr | p.C141Y | NM_000546.5 | 719 | 558 | 159 | 402 | 401 | 1 |
| P926-PTsup | Trunk | NOTCH1 | 9 | + | Missense_Mutation | SNP | C | C | A |  | c.4636G>T | p.Asp1546Tyr | p.D1546Y | NM_017617.5 | 1256 | 1160 | 91 | 695 | 694 | 0 |
| P926-PTsup | Trunk | LATS2 | 13 | + | In_Frame_Ins | INS | - | - | GGGGCG | rs550642106 | c.1431_1436dup | p.Pro479_Ala480dup | p.P479_A480dup | NM_014572.3 | 206 | 126 | 66 | 74 | 69 | 3 |
| P926-PTsup | Trunk | GRIA2 | 4 | + | Missense_Mutation | SNP | C | C | T |  | c.1132C>T | p.Leu378Phe | p.L378F | NM_001083619.1 | 155 | 145 | 10 | 93 | 93 | 0 |
| P926-PTsup | Branch | CHD4 | 12 | + | Frame_Shift_Del | DEL | T | T | - | rs1322050057 | c.218del | p.Lys73ArgfsTer129 | p.K73Rfs*129 | NM_001273.5 | 768 | 752 | 16 | 380 | 379 | 0 |
| P926-PTsup | Trunk | CSMD3 | 8 | + | Missense_Mutation | SNP | A | A | T | novel | c.8713T>A | p.Tyr2905Asn | p.Y2905N | NM_198123.2 | 341 | 328 | 13 | 196 | 196 | 0 |
| P653-PTsup | Branch | CSF3R | 1 | + | Nonsense_Mutation | SNP | G | G | A | rs756679232 | c.1318C>T | p.Arg440Ter | p.R440* | NM_156039.3 | 775 | 633 | 142 | 488 | 487 | 0 |
| P653-PTsup | Trunk | NFE2L2 | 2 | + | Missense_Mutation | SNP | C | C | G | rs1057519920 | c.85G>C | p.Asp29His | p.D29H | NM_006164.5 | 671 | 493 | 177 | 337 | 333 | 4 |
| P653-PTsup | Trunk | PBRM1 | 3 | + | Frame_Shift_Del | DEL | T | T | - | rs771618422 | c.835del | p.Ile279TyrfsTer4 | p.I279Yfs*4 | NM_018313.5 | 539 | 348 | 190 | 434 | 429 | 5 |
| P653-PTsup | Branch | TET2 | 4 | + | Missense_Mutation | SNP | G | G | A | novel | c.1432G>A | p.Glu478Lys | p.E478K | NM_001127208.2 | 566 | 430 | 134 | 412 | 409 | 1 |
| P653-PTsup | Trunk | PTPRK | 6 | + | Missense_Mutation | SNP | G | G | A | novel | c.842C>T | p.Ser281Phe | p.S281F | NM_001291981.2 | 481 | 393 | 87 | 328 | 326 | 0 |
| P653-PTsup | Trunk | PIK3CG | 7 | + | Missense_Mutation | SNP | C | C | G | novel | c.2792C>G | p.Ser931Cys | p.S931C | NM_002649.3 | 589 | 452 | 137 | 429 | 424 | 4 |
| P653-PTsup | Branch | NT5C2 | 10 | + | Missense_Mutation | SNP | C | C | A | novel | c.1468G>T | p.Val490Phe | p.V490F | NM_001351173.1 | 383 | 300 | 83 | 243 | 243 | 0 |
| P653-PTsup | Branch | MUC5B | 11 | + | Missense_Mutation | SNP | C | C | T | rs778789276 | c.4423C>T | p.Arg1475Trp | p.R1475W | NM_002458.3 | 1393 | 1092 | 300 | 702 | 702 | 0 |
| P653-PTsup | Trunk | KMT2D | 12 | + | Missense_Mutation | SNP | C | C | T | rs587778486 | c.15317G>A | p.Arg5106His | p.R5106H | NM_003482.3 | 1014 | 802 | 212 | 608 | 602 | 6 |
| P653-PTsup | Trunk | AJUBA | 14 | + | Frame_Shift_Del | DEL | TG | TG | - | novel | c.1434_1435del | p.Ile479ArgfsTer13 | p.I479Rfs*13 | NM_032876.6 | 424 | 359 | 65 | 254 | 252 | 2 |
| P653-PTsup | Trunk | ZZEF1 | 17 | + | Missense_Mutation | SNP | C | C | A | novel | c.233G>T | p.Gly78Val | p.G78V | NM_015113.4 | 928 | 798 | 129 | 530 | 518 | 11 |
| P653-PTsup | Trunk | TP53 | 17 | + | Missense_Mutation | SNP | C | C | A | rs876660754 | c.517G>T | p.Val173Leu | p.V173L | NM_000546.5 | 1499 | 712 | 787 | 784 | 760 | 24 |
| P653-PTsup | Branch | DNAH9 | 17 | + | Splice_Site | SNP | G | G | A | novel | c.4614+1G>A |  | p.X1538_splice | NM_001372.4 | 565 | 452 | 113 | 291 | 291 | 0 |
| P653-PTsup | Trunk | TCF3 | 19 | + | Missense_Mutation | SNP | C | C | T | rs375296938 | c.1936G>A | p.Glu646Lys | p.E646K | NM_003200.5 | 1382 | 1132 | 249 | 720 | 713 | 7 |
| P653-PTdeep | Branch | CSF3R | 1 | + | Nonsense_Mutation | SNP | G | G | A | rs756679232 | c.1318C>T | p.Arg440Ter | p.R440* | NM_156039.3 | 706 | 620 | 84 | 488 | 487 | 0 |
| P653-PTdeep | Trunk | NFE2L2 | 2 | + | Missense_Mutation | SNP | C | C | G | rs1057519920 | c.85G>C | p.Asp29His | p.D29H | NM_006164.5 | 653 | 503 | 150 | 337 | 333 | 4 |
| P653-PTdeep | Trunk | PBRM1 | 3 | + | Frame_Shift_Del | DEL | T | T | - | rs771618422 | c.835del | p.Ile279TyrfsTer4 | p.I279Yfs*4 | NM_018313.5 | 625 | 467 | 157 | 434 | 429 | 5 |
| P653-PTdeep | Branch | TET2 | 4 | + | Missense_Mutation | SNP | G | G | A | novel | c.1432G>A | p.Glu478Lys | p.E478K | NM_001127208.2 | 534 | 458 | 75 | 412 | 409 | 1 |
| P653-PTdeep | Trunk | PTPRK | 6 | + | Missense_Mutation | SNP | G | G | A | novel | c.842C>T | p.Ser281Phe | p.S281F | NM_001291981.2 | 475 | 411 | 63 | 328 | 326 | 0 |
| P653-PTdeep | Trunk | PIK3CG | 7 | + | Missense_Mutation | SNP | C | C | G | novel | c.2792C>G | p.Ser931Cys | p.S931C | NM_002649.3 | 618 | 527 | 91 | 429 | 424 | 4 |
| P653-PTdeep | Trunk | KMT2C | 7 | + | Frame_Shift_Del | DEL | T | T | - | rs747256476 | c.8390del | p.Lys2797ArgfsTer26 | p.K2797Rfs*26 | NM_170606.3 | 412 | 392 | 17 | 256 | 253 | 2 |
| P653-PTdeep | Branch | NT5C2 | 10 | + | Missense_Mutation | SNP | C | C | A | novel | c.1468G>T | p.Val490Phe | p.V490F | NM_001351173.1 | 385 | 328 | 57 | 243 | 243 | 0 |
| P653-PTdeep | Branch | MUC5B | 11 | + | Missense_Mutation | SNP | C | C | T | rs778789276 | c.4423C>T | p.Arg1475Trp | p.R1475W | NM_002458.3 | 1169 | 1043 | 125 | 702 | 702 | 0 |
| P653-PTdeep | Trunk | KMT2D | 12 | + | Missense_Mutation | SNP | C | C | T | rs587778486 | c.15317G>A | p.Arg5106His | p.R5106H | NM_003482.3 | 951 | 825 | 125 | 608 | 602 | 6 |
| P653-PTdeep | Trunk | AJUBA | 14 | + | Frame_Shift_Del | DEL | TG | TG | - | novel | c.1434_1435del | p.Ile479ArgfsTer13 | p.I479Rfs*13 | NM_032876.6 | 396 | 339 | 57 | 254 | 252 | 2 |
| P653-PTdeep | Trunk | ZZEF1 | 17 | + | Missense_Mutation | SNP | C | C | A | novel | c.233G>T | p.Gly78Val | p.G78V | NM_015113.4 | 695 | 613 | 81 | 530 | 518 | 11 |
| P653-PTdeep | Trunk | TP53 | 17 | + | Missense_Mutation | SNP | C | C | A | rs876660754 | c.517G>T | p.Val173Leu | p.V173L | NM_000546.5 | 1228 | 744 | 483 | 784 | 760 | 24 |
| P653-PTdeep | Branch | DNAH9 | 17 | + | Splice_Site | SNP | G | G | A | novel | c.4614+1G>A |  | p.X1538_splice | NM_001372.4 | 465 | 434 | 30 | 291 | 291 | 0 |
| P653-PTdeep | Trunk | TCF3 | 19 | + | Missense_Mutation | SNP | C | C | T | rs375296938 | c.1936G>A | p.Glu646Lys | p.E646K | NM_003200.5 | 1212 | 1047 | 164 | 720 | 713 | 7 |
| P653-LNmet | Branch | OBSCN | 1 | + | Missense_Mutation | SNP | G | G | C | rs377234188 | c.12759G>C | p.Glu4253Asp | p.E4253D | NM_001271223.2 | 1109 | 1066 | 42 | 913 | 913 | 0 |
| P653-LNmet | Branch | OBSCN | 1 | + | Missense_Mutation | SNP | G | G | C | novel | c.18622G>C | p.Glu6208Gln | p.E6208Q | NM_001271223.2 | 267 | 247 | 20 | 303 | 303 | 0 |
| P653-LNmet | Trunk | NFE2L2 | 2 | + | Missense_Mutation | SNP | C | C | G | rs1057519920 | c.85G>C | p.Asp29His | p.D29H | NM_006164.5 | 639 | 499 | 140 | 337 | 333 | 4 |
| P653-LNmet | Trunk | PBRM1 | 3 | + | Frame_Shift_Del | DEL | T | T | - | rs771618422 | c.835del | p.Ile279TyrfsTer4 | p.I279Yfs*4 | NM_018313.5 | 660 | 488 | 171 | 434 | 429 | 5 |
| P653-LNmet | Branch | FOXL2 | 3 | + | Nonsense_Mutation | SNP | G | G | A | rs104893741 | c.655C>T | p.Gln219Ter | p.Q219* | NM_023067.4 | 367 | 318 | 48 | 263 | 263 | 0 |
| P653-LNmet | Trunk | PTPRK | 6 | + | Missense_Mutation | SNP | G | G | A | novel | c.842C>T | p.Ser281Phe | p.S281F | NM_001291981.2 | 564 | 456 | 107 | 328 | 326 | 0 |
| P653-LNmet | Trunk | PIK3CG | 7 | + | Missense_Mutation | SNP | C | C | G | novel | c.2792C>G | p.Ser931Cys | p.S931C | NM_002649.3 | 805 | 557 | 246 | 429 | 424 | 4 |
| P653-LNmet | Trunk | KMT2C | 7 | + | Frame_Shift_Del | DEL | T | T | - | rs747256476 | c.8390del | p.Lys2797ArgfsTer26 | p.K2797Rfs*26 | NM_170606.3 | 422 | 399 | 18 | 256 | 253 | 2 |
| P653-LNmet | Branch | CSMD3 | 8 | + | Missense_Mutation | SNP | C | C | T | novel | c.5311G>A | p.Gly1771Ser | p.G1771S | NM_198123.2 | 395 | 372 | 22 | 303 | 300 | 2 |
| P653-LNmet | Trunk | KMT2D | 12 | + | Missense_Mutation | SNP | C | C | T | rs587778486 | c.15317G>A | p.Arg5106His | p.R5106H | NM_003482.3 | 805 | 692 | 112 | 608 | 602 | 6 |
| P653-LNmet | Branch | ACACB | 12 | + | Missense_Mutation | SNP | G | G | A | rs556775153 | c.1672G>A | p.Val558Met | p.V558M | NM_001093.4 | 685 | 609 | 75 | 409 | 407 | 2 |
| P653-LNmet | Branch | NCOR2 | 12 | + | In_Frame_Del | DEL | CAGGCTCTCCTCGTA | CAGGCTCTCCTCGTA | - | novel | c.4510_4524del | p.Tyr1504_Leu1508del | p.Y1504_L1508del | NM_006312.6 | 411 | 372 | 39 | 533 | 533 | 0 |
| P653-LNmet | Trunk | AJUBA | 14 | + | Frame_Shift_Del | DEL | TG | TG | - | novel | c.1434_1435del | p.Ile479ArgfsTer13 | p.I479Rfs*13 | NM_032876.6 | 581 | 535 | 45 | 254 | 252 | 2 |
| P653-LNmet | Trunk | ZZEF1 | 17 | + | Missense_Mutation | SNP | C | C | A | novel | c.233G>T | p.Gly78Val | p.G78V | NM_015113.4 | 640 | 566 | 74 | 530 | 518 | 11 |
| P653-LNmet | Trunk | TP53 | 17 | + | Missense_Mutation | SNP | C | C | A | rs876660754 | c.517G>T | p.Val173Leu | p.V173L | NM_000546.5 | 808 | 472 | 336 | 784 | 760 | 24 |
| P653-LNmet | Trunk | TCF3 | 19 | + | Missense_Mutation | SNP | C | C | T | rs375296938 | c.1936G>A | p.Glu646Lys | p.E646K | NM_003200.5 | 695 | 622 | 73 | 720 | 713 | 7 |
| P653-LNmet | Branch | NOTCH3 | 19 | + | Missense_Mutation | SNP | C | C | T | rs777488182 | c.4703G>A | p.Arg1568Gln | p.R1568Q | NM_000435.3 | 554 | 491 | 61 | 551 | 550 | 1 |
| P653-LNmet | Branch | CLTCL1 | 22 | + | Missense_Mutation | SNP | C | C | T | novel | c.4882G>A | p.Glu1628Lys | p.E1628K | NM_007098.4 | 575 | 517 | 58 | 399 | 398 | 0 |
| P653-LNmet | Branch | AR | X | + | Missense_Mutation | SNP | T | T | A | novel | c.2465T>A | p.Leu822Gln | p.L822Q | NM_000044.6 | 227 | 197 | 30 | 133 | 133 | 0 |
| P786-PTsup | Branch | USH2A | 1 | + | Missense_Mutation | SNP | C | C | A | novel | c.8402G>T | p.Gly2801Val | p.G2801V | NM_206933.3 | 546 | 513 | 33 | 321 | 320 | 0 |
| P786-PTsup | Trunk | DNAH7 | 2 | + | Missense_Mutation | SNP | G | G | T |  | c.6819C>A | p.Phe2273Leu | p.F2273L | NM_018897.3 | 835 | 771 | 64 | 343 | 343 | 0 |
| P786-PTsup | Trunk | ITPR1 | 3 | + | Missense_Mutation | SNP | C | C | A | novel | c.1459C>A | p.Pro487Thr | p.P487T | NM_001168272.1 | 814 | 735 | 79 | 654 | 654 | 0 |
| P786-PTsup | Trunk | DDB2 | 11 | + | Missense_Mutation | SNP | G | G | A | rs567082210 | c.1117G>A | p.Asp373Asn | p.D373N | NM_000107.2 | 903 | 737 | 166 | 545 | 544 | 1 |
| P786-PTsup | Trunk | BCL11B | 14 | + | In_Frame_Del | DEL | CCCCCGCGCCCGGGA | CCCCCGCGCCCGGGA | - | novel | c.1727_1741del | p.Val576_Gly580del | p.V576_G580del | NM_138576.4 | 845 | 761 | 84 | 308 | 306 | 0 |
| P786-PTsup | Trunk | ZFHX3 | 16 | + | Missense_Mutation | SNP | G | G | A | rs746242918 | c.3424C>T | p.Arg1142Cys | p.R1142C | NM_006885.4 | 542 | 504 | 38 | 365 | 364 | 0 |
| P786-PTsup | Trunk | TP53 | 17 | + | Missense_Mutation | SNP | G | G | A | rs28934574 | c.844C>T | p.Arg282Trp | p.R282W | NM_000546.5 | 1093 | 709 | 384 | 637 | 637 | 0 |
| P786-PTsup | Branch | TP53 | 17 | + | Nonsense_Mutation | SNP | G | G | A | rs397516436 | c.637C>T | p.Arg213Ter | p.R213* | NM_000546.5 | 876 | 867 | 8 | 465 | 464 | 0 |
| P786-PTsup | Branch | PPP2R1A | 19 | + | Missense_Mutation | SNP | T | T | C | novel | c.386T>C | p.Val129Ala | p.V129A | NM_014225.6 | 828 | 804 | 23 | 416 | 416 | 0 |
| P786-PTdeep | Trunk | ITPR1 | 3 | + | Missense_Mutation | SNP | C | C | A | novel | c.1459C>A | p.Pro487Thr | p.P487T | NM_001168272.1 | 499 | 437 | 62 | 654 | 654 | 0 |
| P786-PTdeep | Branch | FAT4 | 4 | + | Missense_Mutation | SNP | G | G | A | rs1406593216 | c.11515G>A | p.Ala3839Thr | p.A3839T | NM_024582.4 | 493 | 483 | 10 | 433 | 431 | 0 |
| P786-PTdeep | Trunk | DDB2 | 11 | + | Missense_Mutation | SNP | G | G | A | rs567082210 | c.1117G>A | p.Asp373Asn | p.D373N | NM_000107.2 | 504 | 418 | 86 | 545 | 544 | 1 |
| P786-PTdeep | Trunk | BCL11B | 14 | + | In_Frame_Del | DEL | CCCCCGCGCCCGGGA | CCCCCGCGCCCGGGA | - | novel | c.1727_1741del | p.Val576_Gly580del | p.V576_G580del | NM_138576.4 | 296 | 238 | 58 | 308 | 306 | 0 |
| P786-PTdeep | Branch | BLM | 15 | + | Frame_Shift_Del | DEL | A | A | - | rs367543043 | c.1544del | p.Asn515MetfsTer16 | p.N515Mfs*16 | NM_000057.4 | 434 | 424 | 10 | 417 | 416 | 1 |
| P786-PTdeep | Trunk | ZFHX3 | 16 | + | Missense_Mutation | SNP | G | G | A | rs746242918 | c.3424C>T | p.Arg1142Cys | p.R1142C | NM_006885.4 | 350 | 317 | 33 | 365 | 364 | 0 |
| P786-PTdeep | Trunk | TP53 | 17 | + | Missense_Mutation | SNP | G | G | A | rs28934574 | c.844C>T | p.Arg282Trp | p.R282W | NM_000546.5 | 972 | 592 | 380 | 637 | 637 | 0 |
| P786-LNmet | Branch | USH2A | 1 | + | Nonsense_Mutation | DNP | GG | GG | AT | novel | c.2859_2860delinsAT | p.Cys953_His954delinsTer | p.C953_H954delins* | NM_206933.3 | 362 | 348 | 12 | 341 | 340 | 0 |
| P786-LNmet | Trunk | DNAH7 | 2 | + | Missense_Mutation | SNP | G | G | T |  | c.6819C>A | p.Phe2273Leu | p.F2273L | NM_018897.3 | 421 | 400 | 21 | 343 | 343 | 0 |
| P786-LNmet | Trunk | ITPR1 | 3 | + | Missense_Mutation | SNP | C | C | A | novel | c.1459C>A | p.Pro487Thr | p.P487T | NM_001168272.1 | 638 | 607 | 28 | 654 | 654 | 0 |
| P786-LNmet | Branch | GARS | 7 | + | Missense_Mutation | SNP | T | T | C | novel | c.781T>C | p.Tyr261His | p.Y261H | NM_002047.4 | 390 | 379 | 11 | 342 | 342 | 0 |
| P786-LNmet | Branch | PREX2 | 8 | + | Missense_Mutation | SNP | C | C | G | novel | c.1114C>G | p.Gln372Glu | p.Q372E | NM_024870.4 | 410 | 396 | 13 | 292 | 292 | 0 |
| P786-LNmet | Trunk | DDB2 | 11 | + | Missense_Mutation | SNP | G | G | A | rs567082210 | c.1117G>A | p.Asp373Asn | p.D373N | NM_000107.2 | 647 | 591 | 53 | 545 | 544 | 1 |
| P786-LNmet | Trunk | BCL11B | 14 | + | In_Frame_Del | DEL | CCCCCGCGCCCGGGA | CCCCCGCGCCCGGGA | - | novel | c.1727_1741del | p.Val576_Gly580del | p.V576_G580del | NM_138576.4 | 517 | 476 | 41 | 308 | 306 | 0 |
| P786-LNmet | Branch | DNAH3 | 16 | + | Missense_Mutation | SNP | C | C | T | novel | c.9877G>A | p.Gly3293Ser | p.G3293S | NM_017539.2 | 440 | 420 | 20 | 349 | 348 | 0 |
| P786-LNmet | Trunk | ZFHX3 | 16 | + | Missense_Mutation | SNP | G | G | A | rs746242918 | c.3424C>T | p.Arg1142Cys | p.R1142C | NM_006885.4 | 506 | 471 | 35 | 365 | 364 | 0 |
| P786-LNmet | Trunk | TP53 | 17 | + | Missense_Mutation | SNP | G | G | A | rs28934574 | c.844C>T | p.Arg282Trp | p.R282W | NM_000546.5 | 1331 | 1100 | 230 | 637 | 637 | 0 |
| P973-LNmet | Trunk | SPTA1 | 1 | + | Missense_Mutation | SNP | C | C | A | rs540011978 | c.4309G>T | p.Asp1437Tyr | p.D1437Y | NM_003126.4 | 619 | 503 | 113 | 238 | 237 | 0 |
| P973-LNmet | Trunk | SOS1 | 2 | + | Missense_Mutation | SNP | G | G | A | novel | c.2455C>T | p.Pro819Ser | p.P819S | NM_005633.3 | 498 | 393 | 104 | 236 | 236 | 0 |
| P973-LNmet | Trunk | CNTNAP2 | 7 | + | Missense_Mutation | SNP | G | G | C |  | c.2666G>C | p.Arg889Thr | p.R889T | NM_014141.6 | 1275 | 1048 | 227 | 334 | 334 | 0 |
| P973-LNmet | Trunk | CSMD3 | 8 | + | Missense_Mutation | SNP | T | T | A | rs766890809 | c.6877A>T | p.Thr2293Ser | p.T2293S | NM_198123.2 | 455 | 363 | 92 | 192 | 192 | 0 |
| P973-LNmet | Trunk | TP53 | 17 | + | Missense_Mutation | SNP | C | C | T | rs587782664 | c.711G>A | p.Met237Ile | p.M237I | NM_000546.5 | 698 | 478 | 220 | 282 | 282 | 0 |
| P973-LNmet | Trunk | MYH2 | 17 | + | Missense_Mutation | SNP | G | G | T | novel | c.1990C>A | p.Leu664Met | p.L664M | NM_017534.6 | 249 | 201 | 48 | 153 | 153 | 0 |
| P973-LNmet | Branch | NCOR1 | 17 | + | Missense_Mutation | SNP | G | G | T |  | c.6728C>A | p.Thr2243Lys | p.T2243K | NM_006311.4 | 758 | 731 | 27 | 309 | 309 | 0 |
| P973-LNmet | Trunk | NCOR1 | 17 | + | Missense_Mutation | SNP | G | G | C | novel | c.3215C>G | p.Ala1072Gly | p.A1072G | NM_006311.4 | 688 | 587 | 100 | 349 | 346 | 1 |
| P973-LNmet | Branch | STAT5B | 17 | + | Missense_Mutation | SNP | G | G | T | novel | c.202C>A | p.Leu68Met | p.L68M | NM_012448.4 | 1478 | 1399 | 77 | 438 | 438 | 0 |
| P973-PTsup | Trunk | SPTA1 | 1 | + | Missense_Mutation | SNP | C | C | A | rs540011978 | c.4309G>T | p.Asp1437Tyr | p.D1437Y | NM_003126.4 | 870 | 728 | 138 | 238 | 237 | 0 |
| P973-PTsup | Trunk | SOS1 | 2 | + | Missense_Mutation | SNP | G | G | A | novel | c.2455C>T | p.Pro819Ser | p.P819S | NM_005633.3 | 695 | 612 | 83 | 236 | 236 | 0 |
| P973-PTsup | Branch | SFRP4 | 7 | + | Missense_Mutation | SNP | C | C | T | novel | c.403G>A | p.Val135Met | p.V135M | NM_003014.4 | 1394 | 1183 | 211 | 382 | 382 | 0 |
| P973-PTsup | Trunk | CNTNAP2 | 7 | + | Missense_Mutation | SNP | G | G | C |  | c.2666G>C | p.Arg889Thr | p.R889T | NM_014141.6 | 926 | 816 | 109 | 334 | 334 | 0 |
| P973-PTsup | Trunk | CSMD3 | 8 | + | Missense_Mutation | SNP | T | T | A | rs766890809 | c.6877A>T | p.Thr2293Ser | p.T2293S | NM_198123.2 | 708 | 639 | 68 | 192 | 192 | 0 |
| P973-PTsup | Branch | MLLT10 | 10 | + | Missense_Mutation | SNP | C | C | G | rs770775710 | c.1541C>G | p.Ser514Cys | p.S514C | NM_001195626.3 | 973 | 934 | 39 | 323 | 323 | 0 |
| P973-PTsup | Trunk | TP53 | 17 | + | Missense_Mutation | SNP | C | C | T | rs587782664 | c.711G>A | p.Met237Ile | p.M237I | NM_000546.5 | 595 | 508 | 87 | 282 | 282 | 0 |
| P973-PTsup | Trunk | MYH2 | 17 | + | Missense_Mutation | SNP | G | G | T | novel | c.1990C>A | p.Leu664Met | p.L664M | NM_017534.6 | 442 | 407 | 35 | 153 | 153 | 0 |
| P973-PTsup | Trunk | NCOR1 | 17 | + | Missense_Mutation | SNP | G | G | C | novel | c.3215C>G | p.Ala1072Gly | p.A1072G | NM_006311.4 | 833 | 767 | 66 | 349 | 346 | 1 |
| P973-PTsup | Branch | BRCA1 | 17 | + | Nonsense_Mutation | SNP | C | C | A | rs886040195 | c.4069G>T | p.Glu1357Ter | p.E1357* | NM_007294.4 | 895 | 783 | 111 | 337 | 337 | 0 |
| P973-PTdeep | Trunk | SPTA1 | 1 | + | Missense_Mutation | SNP | C | C | A | rs540011978 | c.4309G>T | p.Asp1437Tyr | p.D1437Y | NM_003126.4 | 646 | 484 | 162 | 238 | 237 | 0 |
| P973-PTdeep | Trunk | SOS1 | 2 | + | Missense_Mutation | SNP | G | G | A | novel | c.2455C>T | p.Pro819Ser | p.P819S | NM_005633.3 | 603 | 495 | 107 | 236 | 236 | 0 |
| P973-PTdeep | Branch | SFRP4 | 7 | + | Missense_Mutation | SNP | C | C | T | novel | c.403G>A | p.Val135Met | p.V135M | NM_003014.4 | 1132 | 884 | 248 | 382 | 382 | 0 |
| P973-PTdeep | Trunk | CNTNAP2 | 7 | + | Missense_Mutation | SNP | G | G | C |  | c.2666G>C | p.Arg889Thr | p.R889T | NM_014141.6 | 1063 | 866 | 197 | 334 | 334 | 0 |
| P973-PTdeep | Trunk | CSMD3 | 8 | + | Missense_Mutation | SNP | T | T | A | rs766890809 | c.6877A>T | p.Thr2293Ser | p.T2293S | NM_198123.2 | 438 | 377 | 61 | 192 | 192 | 0 |
| P973-PTdeep | Branch | MLLT10 | 10 | + | Missense_Mutation | SNP | C | C | G | rs770775710 | c.1541C>G | p.Ser514Cys | p.S514C | NM_001195626.3 | 677 | 650 | 27 | 323 | 323 | 0 |
| P973-PTdeep | Trunk | TP53 | 17 | + | Missense_Mutation | SNP | C | C | T | rs587782664 | c.711G>A | p.Met237Ile | p.M237I | NM_000546.5 | 535 | 400 | 134 | 282 | 282 | 0 |
| P973-PTdeep | Trunk | MYH2 | 17 | + | Missense_Mutation | SNP | G | G | T | novel | c.1990C>A | p.Leu664Met | p.L664M | NM_017534.6 | 244 | 186 | 58 | 153 | 153 | 0 |
| P973-PTdeep | Trunk | NCOR1 | 17 | + | Missense_Mutation | SNP | G | G | C | novel | c.3215C>G | p.Ala1072Gly | p.A1072G | NM_006311.4 | 552 | 487 | 65 | 349 | 346 | 1 |
| P973-PTdeep | Branch | BRCA1 | 17 | + | Nonsense_Mutation | SNP | C | C | A | rs886040195 | c.4069G>T | p.Glu1357Ter | p.E1357* | NM_007294.4 | 657 | 532 | 125 | 337 | 337 | 0 |
| P253-LNmet | Trunk | ARID1B | 6 | + | Missense_Mutation | SNP | G | G | A | rs1206220417 | c.5890G>A | p.Glu1964Lys | p.E1964K | NM_020732.3 | 783 | 667 | 116 | 566 | 566 | 0 |
| P253-LNmet | Trunk | TUBA3C | 13 | + | Missense_Mutation | SNP | G | G | A | rs763672067 | c.1015C>T | p.Arg339Cys | p.R339C | NM_006001.3 | 1169 | 923 | 245 | 784 | 781 | 2 |
| P253-LNmet | Trunk | TP53 | 17 | + | Nonsense_Mutation | SNP | C | C | A |  | c.610G>T | p.Glu204Ter | p.E204* | NM_000546.5 | 862 | 397 | 462 | 532 | 532 | 0 |
| P253-LNmet | Trunk | EP300 | 22 | + | Nonsense_Mutation | SNP | G | G | T | novel | c.3751G>T | p.Gly1251Ter | p.G1251* | NM_001429.4 | 631 | 244 | 385 | 350 | 350 | 0 |
| P253-PTsup | Trunk | ARID1B | 6 | + | Missense_Mutation | SNP | G | G | A | rs1206220417 | c.5890G>A | p.Glu1964Lys | p.E1964K | NM_020732.3 | 658 | 579 | 79 | 566 | 566 | 0 |
| P253-PTsup | Trunk | TUBA3C | 13 | + | Missense_Mutation | SNP | G | G | A | rs763672067 | c.1015C>T | p.Arg339Cys | p.R339C | NM_006001.3 | 1091 | 835 | 256 | 784 | 781 | 2 |
| P253-PTsup | Branch | DNAH3 | 16 | + | Missense_Mutation | SNP | G | G | C | novel | c.3861C>G | p.Ile1287Met | p.I1287M | NM_017539.2 | 505 | 483 | 22 | 248 | 248 | 0 |
| P253-PTsup | Trunk | TP53 | 17 | + | Nonsense_Mutation | SNP | C | C | A |  | c.610G>T | p.Glu204Ter | p.E204* | NM_000546.5 | 796 | 485 | 311 | 532 | 532 | 0 |
| P253-PTsup | Trunk | EP300 | 22 | + | Nonsense_Mutation | SNP | G | G | T | novel | c.3751G>T | p.Gly1251Ter | p.G1251* | NM_001429.4 | 747 | 331 | 416 | 350 | 350 | 0 |
| P253-PTdeep | Trunk | TUBA3C | 13 | + | Missense_Mutation | SNP | G | G | A | rs763672067 | c.1015C>T | p.Arg339Cys | p.R339C | NM_006001.3 | 1365 | 1196 | 169 | 784 | 781 | 2 |
| P253-PTdeep | Trunk | TP53 | 17 | + | Nonsense_Mutation | SNP | C | C | A |  | c.610G>T | p.Glu204Ter | p.E204* | NM_000546.5 | 807 | 662 | 145 | 532 | 532 | 0 |
| P253-PTdeep | Trunk | EP300 | 22 | + | Nonsense_Mutation | SNP | G | G | T | novel | c.3751G>T | p.Gly1251Ter | p.G1251* | NM_001429.4 | 577 | 418 | 157 | 350 | 350 | 0 |
| P848-PTsup | Trunk | AFF3 | 2 | + | Missense_Mutation | SNP | C | C | A | novel | c.3599G>T | p.Trp1200Leu | p.W1200L | NM_001025108.2 | 1520 | 1336 | 182 | 258 | 258 | 0 |
| P848-PTsup | Branch | LRP1B | 2 | + | Nonsense_Mutation | SNP | C | C | T | novel | c.4461G>A | p.Trp1487Ter | p.W1487* | NM_018557.3 | 637 | 493 | 143 | 203 | 202 | 0 |
| P848-PTsup | Trunk | NEB | 2 | + | Missense_Mutation | SNP | G | G | A | rs115623365 | c.20896C>T | p.Arg6966Cys | p.R6966C | NM_001271208.2 | 293 | 220 | 73 | 120 | 120 | 0 |
| P848-PTsup | Trunk | ERBB4 | 2 | + | Nonsense_Mutation | SNP | C | C | T | novel | c.2732G>A | p.Trp911Ter | p.W911* | NM_005235.3 | 472 | 374 | 98 | 167 | 167 | 0 |
| P848-PTsup | Trunk | CUL3 | 2 | + | Missense_Mutation | SNP | C | C | A | novel | c.2216G>T | p.Arg739Leu | p.R739L | NM_001257198.2 | 517 | 422 | 91 | 227 | 227 | 0 |
| P848-PTsup | Branch | GRIA2 | 4 | + | Missense_Mutation | SNP | A | A | C | novel | c.2431A>C | p.Ser811Arg | p.S811R | NM_001083619.1 | 809 | 683 | 124 | 148 | 146 | 0 |
| P848-PTsup | Trunk | KAT6A | 8 | + | Frame_Shift_Ins | INS | - | - | CTGG | novel | c.4473_4476dup | p.Ser1493ProfsTer15 | p.S1493Pfs*15 | NM_006766.5 | 989 | 828 | 147 | 327 | 327 | 0 |
| P848-PTsup | Trunk | CD163 | 12 | + | Nonsense_Mutation | SNP | C | C | A |  | c.2122G>T | p.Glu708Ter | p.E708* | NM_004244.5 | 734 | 478 | 256 | 187 | 187 | 0 |
| P848-PTsup | Trunk | TP53 | 17 | + | Nonsense_Mutation | SNP | T | T | A |  | c.871A>T | p.Lys291Ter | p.K291* | NM_000546.5 | 1447 | 897 | 550 | 662 | 661 | 1 |
| P848-PTsup | Branch | MUC16 | 19 | + | Missense_Mutation | SNP | C | C | T |  | c.30664G>A | p.Glu10222Lys | p.E10222K | NM_024690.2 | 1068 | 900 | 168 | 160 | 160 | 0 |
| P848-PTdeep | Trunk | AFF3 | 2 | + | Missense_Mutation | SNP | C | C | A | novel | c.3599G>T | p.Trp1200Leu | p.W1200L | NM_001025108.2 | 1024 | 885 | 138 | 258 | 258 | 0 |
| P848-PTdeep | Branch | LRP1B | 2 | + | Nonsense_Mutation | SNP | C | C | T | novel | c.4461G>A | p.Trp1487Ter | p.W1487* | NM_018557.3 | 438 | 344 | 92 | 203 | 202 | 0 |
| P848-PTdeep | Trunk | NEB | 2 | + | Missense_Mutation | SNP | G | G | A | rs115623365 | c.20896C>T | p.Arg6966Cys | p.R6966C | NM_001271208.2 | 198 | 164 | 34 | 120 | 120 | 0 |
| P848-PTdeep | Trunk | ERBB4 | 2 | + | Nonsense_Mutation | SNP | C | C | T | novel | c.2732G>A | p.Trp911Ter | p.W911* | NM_005235.3 | 351 | 282 | 69 | 167 | 167 | 0 |
| P848-PTdeep | Trunk | CUL3 | 2 | + | Missense_Mutation | SNP | C | C | A | novel | c.2216G>T | p.Arg739Leu | p.R739L | NM_001257198.2 | 410 | 329 | 80 | 227 | 227 | 0 |
| P848-PTdeep | Branch | GRIA2 | 4 | + | Missense_Mutation | SNP | A | A | C | novel | c.2431A>C | p.Ser811Arg | p.S811R | NM_001083619.1 | 570 | 519 | 49 | 148 | 146 | 0 |
| P848-PTdeep | Trunk | KAT6A | 8 | + | Frame_Shift_Ins | INS | - | - | CTGG | novel | c.4473_4476dup | p.Ser1493ProfsTer15 | p.S1493Pfs*15 | NM_006766.5 | 791 | 640 | 135 | 327 | 327 | 0 |
| P848-PTdeep | Trunk | CD163 | 12 | + | Nonsense_Mutation | SNP | C | C | A |  | c.2122G>T | p.Glu708Ter | p.E708* | NM_004244.5 | 542 | 361 | 180 | 187 | 187 | 0 |
| P848-PTdeep | Trunk | TP53 | 17 | + | Nonsense_Mutation | SNP | T | T | A |  | c.871A>T | p.Lys291Ter | p.K291* | NM_000546.5 | 1164 | 744 | 420 | 662 | 661 | 1 |
| P848-PTdeep | Branch | MUC16 | 19 | + | Missense_Mutation | SNP | C | C | T |  | c.30664G>A | p.Glu10222Lys | p.E10222K | NM_024690.2 | 631 | 546 | 85 | 160 | 160 | 0 |
| P848-LNmet | Trunk | AFF3 | 2 | + | Missense_Mutation | SNP | C | C | A | novel | c.3599G>T | p.Trp1200Leu | p.W1200L | NM_001025108.2 | 975 | 916 | 58 | 258 | 258 | 0 |
| P848-LNmet | Trunk | NEB | 2 | + | Missense_Mutation | SNP | G | G | A | rs115623365 | c.20896C>T | p.Arg6966Cys | p.R6966C | NM_001271208.2 | 233 | 207 | 26 | 120 | 120 | 0 |
| P848-LNmet | Trunk | ERBB4 | 2 | + | Nonsense_Mutation | SNP | C | C | T | novel | c.2732G>A | p.Trp911Ter | p.W911* | NM_005235.3 | 362 | 325 | 36 | 167 | 167 | 0 |
| P848-LNmet | Trunk | CUL3 | 2 | + | Missense_Mutation | SNP | C | C | A | novel | c.2216G>T | p.Arg739Leu | p.R739L | NM_001257198.2 | 373 | 343 | 29 | 227 | 227 | 0 |
| P848-LNmet | Branch | PTPN13 | 4 | + | Missense_Mutation | SNP | C | C | T | novel | c.6781C>T | p.Leu2261Phe | p.L2261F | NM_080685.2 | 388 | 375 | 13 | 222 | 222 | 0 |
| P848-LNmet | Branch | KMT2C | 7 | + | Frame_Shift_Ins | INS | - | - | T | rs747256476 | c.8390dup | p.Glu2798GlyfsTer11 | p.E2798Gfs*11 | NM_170606.3 | 405 | 392 | 11 | 291 | 290 | 0 |
| P848-LNmet | Trunk | KAT6A | 8 | + | Frame_Shift_Ins | INS | - | - | CTGG | novel | c.4473_4476dup | p.Ser1493ProfsTer15 | p.S1493Pfs*15 | NM_006766.5 | 952 | 860 | 86 | 327 | 327 | 0 |
| P848-LNmet | Trunk | CD163 | 12 | + | Nonsense_Mutation | SNP | C | C | A |  | c.2122G>T | p.Glu708Ter | p.E708* | NM_004244.5 | 467 | 361 | 106 | 187 | 187 | 0 |
| P848-LNmet | Trunk | TP53 | 17 | + | Nonsense_Mutation | SNP | T | T | A |  | c.871A>T | p.Lys291Ter | p.K291* | NM_000546.5 | 1347 | 1101 | 246 | 662 | 661 | 1 |
| P848-LNmet | Branch | BRCA1 | 17 | + | Missense_Mutation | SNP | G | G | A | rs80356994 | c.19C>T | p.Arg7Cys | p.R7C | NM_007294.4 | 1269 | 1240 | 29 | 812 | 809 | 3 |
| P768-PTsup | Trunk | OBSCN | 1 | + | Nonsense_Mutation | SNP | C | C | G | novel | c.24800C>G | p.Ser8267Ter | p.S8267* | NM_001271223.2 | 712 | 622 | 89 | 253 | 253 | 0 |
| P768-PTsup | Trunk | LRP1B | 2 | + | Missense_Mutation | SNP | C | C | T | rs1325525264 | c.8374G>A | p.Asp2792Asn | p.D2792N | NM_018557.3 | 555 | 376 | 179 | 150 | 150 | 0 |
| P768-PTsup | Trunk | BAP1 | 3 | + | Nonsense_Mutation | SNP | G | G | A |  | c.106C>T | p.Gln36Ter | p.Q36* | NM_004656.4 | 584 | 289 | 295 | 328 | 328 | 0 |
| P768-PTsup | Branch | ARID1B | 6 | + | In_Frame_Del | DEL | CAGCAGCAGCAGCAGCAGCAGCAA | CAGCAGCAGCAGCAGCAGCAGCAA | - | rs770869529 | c.369_392del | p.Gln124_Gln131del | p.Q124_Q131del | NM_020732.3 | 3029 | 2768 | 163 | 1634 | 1569 | 0 |
| P768-PTsup | Trunk | NBN | 8 | + | Nonsense_Mutation | SNP | G | G | C |  | c.1253C>G | p.Ser418Ter | p.S418* | NM_002485.4 | 520 | 304 | 216 | 224 | 224 | 0 |
| P768-PTsup | Branch | FAM135B | 8 | + | Missense_Mutation | SNP | C | C | A |  | c.3534G>T | p.Lys1178Asn | p.K1178N | NM_015912.4 | 365 | 338 | 27 | 116 | 116 | 0 |
| P768-PTsup | Branch | FGFR2 | 10 | + | Missense_Mutation | SNP | C | C | T |  | c.1393G>A | p.Glu465Lys | p.E465K | NM_000141.4 | 736 | 584 | 151 | 383 | 382 | 1 |
| P768-PTsup | Trunk | AKT1 | 14 | + | Missense_Mutation | SNP | C | C | T | rs121434592 | c.48G>A | p.Glu17Lys | p.E17K | NM_001014432.1 | 524 | 322 | 201 | 172 | 172 | 0 |
| P768-PTsup | Trunk | MYH8 | 17 | + | Missense_Mutation | SNP | C | C | T | novel | c.3745G>A | p.Glu1249Lys | p.E1249K | NM_002472.3 | 241 | 198 | 43 | 169 | 169 | 0 |
| P768-PTsup | Trunk | ZNF750 | 17 | + | Translation_Start_Site | SNP | C | C | A | rs200826990 | c.3G>T | p.Met1? | p.M1? | NM_024702.3 | 609 | 194 | 415 | 187 | 187 | 0 |
| P768-PTsup | Trunk | OLIG2 | 21 | + | Missense_Mutation | SNP | A | A | G | novel | c.784A>G | p.Lys262Glu | p.K262E | NM_005806.4 | 252 | 203 | 49 | 66 | 66 | 0 |
| P768-PTdeep | Trunk | OBSCN | 1 | + | Nonsense_Mutation | SNP | C | C | G | novel | c.24800C>G | p.Ser8267Ter | p.S8267* | NM_001271223.2 | 789 | 710 | 76 | 253 | 253 | 0 |
| P768-PTdeep | Trunk | LRP1B | 2 | + | Missense_Mutation | SNP | C | C | T | rs1325525264 | c.8374G>A | p.Asp2792Asn | p.D2792N | NM_018557.3 | 546 | 423 | 123 | 150 | 150 | 0 |
| P768-PTdeep | Trunk | BAP1 | 3 | + | Nonsense_Mutation | SNP | G | G | A |  | c.106C>T | p.Gln36Ter | p.Q36* | NM_004656.4 | 681 | 487 | 191 | 328 | 328 | 0 |
| P768-PTdeep | Branch | ARID1B | 6 | + | In_Frame_Del | DEL | CAGCAGCAGCAGCAGCAGCAGCAA | CAGCAGCAGCAGCAGCAGCAGCAA | - | rs770869529 | c.369_392del | p.Gln124_Gln131del | p.Q124_Q131del | NM_020732.3 | 3292 | 3084 | 105 | 1634 | 1569 | 0 |
| P768-PTdeep | Trunk | NBN | 8 | + | Nonsense_Mutation | SNP | G | G | C |  | c.1253C>G | p.Ser418Ter | p.S418* | NM_002485.4 | 763 | 521 | 240 | 224 | 224 | 0 |
| P768-PTdeep | Branch | FAM135B | 8 | + | Missense_Mutation | SNP | C | C | A |  | c.3534G>T | p.Lys1178Asn | p.K1178N | NM_015912.4 | 546 | 504 | 42 | 116 | 116 | 0 |
| P768-PTdeep | Branch | FGFR2 | 10 | + | Missense_Mutation | SNP | C | C | T |  | c.1393G>A | p.Glu465Lys | p.E465K | NM_000141.4 | 978 | 848 | 129 | 383 | 382 | 1 |
| P768-PTdeep | Trunk | AKT1 | 14 | + | Missense_Mutation | SNP | C | C | T | rs121434592 | c.48G>A | p.Glu17Lys | p.E17K | NM_001014432.1 | 521 | 394 | 127 | 172 | 172 | 0 |
| P768-PTdeep | Trunk | MYH8 | 17 | + | Missense_Mutation | SNP | C | C | T | novel | c.3745G>A | p.Glu1249Lys | p.E1249K | NM_002472.3 | 454 | 394 | 60 | 169 | 169 | 0 |
| P768-PTdeep | Trunk | ZNF750 | 17 | + | Translation_Start_Site | SNP | C | C | A | rs200826990 | c.3G>T | p.Met1? | p.M1? | NM_024702.3 | 701 | 364 | 336 | 187 | 187 | 0 |
| P768-PTdeep | Trunk | OLIG2 | 21 | + | Missense_Mutation | SNP | A | A | G | novel | c.784A>G | p.Lys262Glu | p.K262E | NM_005806.4 | 427 | 353 | 74 | 66 | 66 | 0 |
| P768-LNmet | Trunk | OBSCN | 1 | + | Nonsense_Mutation | SNP | C | C | G | novel | c.24800C>G | p.Ser8267Ter | p.S8267* | NM_001271223.2 | 321 | 298 | 23 | 253 | 253 | 0 |
| P768-LNmet | Trunk | LRP1B | 2 | + | Missense_Mutation | SNP | C | C | T | rs1325525264 | c.8374G>A | p.Asp2792Asn | p.D2792N | NM_018557.3 | 340 | 296 | 44 | 150 | 150 | 0 |
| P768-LNmet | Trunk | BAP1 | 3 | + | Nonsense_Mutation | SNP | G | G | A |  | c.106C>T | p.Gln36Ter | p.Q36* | NM_004656.4 | 430 | 365 | 65 | 328 | 328 | 0 |
| P768-LNmet | Trunk | NBN | 8 | + | Nonsense_Mutation | SNP | G | G | C |  | c.1253C>G | p.Ser418Ter | p.S418* | NM_002485.4 | 402 | 341 | 60 | 224 | 224 | 0 |
| P768-LNmet | Trunk | AKT1 | 14 | + | Missense_Mutation | SNP | C | C | T | rs121434592 | c.48G>A | p.Glu17Lys | p.E17K | NM_001014432.1 | 295 | 252 | 43 | 172 | 172 | 0 |
| P768-LNmet | Trunk | MYH8 | 17 | + | Missense_Mutation | SNP | C | C | T | novel | c.3745G>A | p.Glu1249Lys | p.E1249K | NM_002472.3 | 233 | 211 | 22 | 169 | 169 | 0 |
| P768-LNmet | Trunk | OLIG2 | 21 | + | Missense_Mutation | SNP | A | A | G | novel | c.784A>G | p.Lys262Glu | p.K262E | NM_005806.4 | 117 | 107 | 10 | 66 | 66 | 0 |
