# Supplementary material for: Multi‐omics analyses reveal spatial heterogeneity in primary and metastatic oesophageal squamous cell carcinoma
Source: Clin Transl Med. 2023 Nov 27;13(11):e1493. doi: 10.1002/ctm2.1493 (PMC10679972; doi:10.1002/ctm2.1493)
Supplement: Supplementary file 20 — Table S9. Number of genes in the whole transcriptome with significant differences in analyses of tumour subregions. [file CTM2-13-e1493-s006.docx]

**Supplementary Table 9. Number of genes in the whole transcriptome with significant differences in analyses of tumor subregions.**

| **LN_met_ versus PT_sup_ versus PT_deep_** | **ESCCs compared**  **n (A) versus n (B)** | **Genes sig different,**  **n** | **A > 2 × B**  **n** | **B > 2 × A**  **n** |
| --- | --- | --- | --- | --- |
| PT_sup_ versus PT_deep_ | 17 versus 19 | 2 | 0 | 2 |
| LN_met_ versus PT_sup_ | 17 versus 17 | 841 | 715 | 126 |
| LN_met_ versus PT_deep_ | 17 versus 19 | 373 | 221 | 152 |
| LN_met_ versus PT | 17 versus 36 | 807 | 606 | 201 |

A is the first subregion in the corresponding row of the first column (PT_sup_ vs PT_deep_ vs LN_met_ vs PT).

B is the second subregion in the corresponding row of the first column.

For example, in the first row, A = PT_sup_ and B = PT_deep_.

Expression of genes with a False Discovery Rate (FDR) value < 0.05, and the absolute value of log2 (Fold Change (FC)) > 1 were considered as significant difference.

ESCC, Esophageal squamous cell carcinoma; PT_sup_, primary tumor superficial; PT_deep_, primary tumor deep; LN_met_, lymph node metastasis; PT, primary tumor; sig, significant.
