# Supplementary material for: Multi‐omics analyses reveal spatial heterogeneity in primary and metastatic oesophageal squamous cell carcinoma
Source: Clin Transl Med. 2023 Nov 27;13(11):e1493. doi: 10.1002/ctm2.1493 (PMC10679972; doi:10.1002/ctm2.1493)
Supplement: Supplementary file 21 — Table S10. Genes in the whole transcriptome with significant differences in analyses of tumour subregions. [file CTM2-13-e1493-s023.docx]

**Supplementary Table 10. Genes in the whole transcriptome with significant differences in analyses of tumor subregions.**

| **Differential RNA expression between PT_sup_ and PT_deep_** | | | | | | | |
| --- | --- | --- | --- | --- | --- | --- | --- |
| **Gene ID** | **Length** | PT**_sup_**-Expression | PT_deep_-Expression | log2 FoldChange  (PT_sup_/PT_deep_) | **Padj** | **Up/Down-Regulation** | **P-value** |

| **CTNNA3** | 4037 | 2.20186531 | | 49.06176532 | 4.477801019 | 0.000135948 | Up | 3.03E-09 |
| --- | --- | --- | --- | --- | --- | --- | --- | --- |
| **THBS4** | 2846 | 240.6051456 | | 1416.124497 | 2.557208702 | 0.034159596 | Up | 1.52E-06 |
| **Differential RNA expression between LN_met_ and PT_sup_** | | | | | | | | |
| **Gene ID** | **Length** | PT**_sup_**-Expression | | LN**_met_**-Expression | log2 FoldChange (LN**_met_**/PT**_sup_**) | **Padj** | **Up/Down-Regulation** | **P-value** |
| ADIPOQ | 4562 | 0.171299954 | | 65.51369303 | 8.579127898 | 2.80E-07 | Up | 1.86E-11 |
| RP11-203B7.2 | 4159 | 0.672151266 | | 61.21924805 | 6.509055569 | 6.00E-05 | Up | 9.41E-08 |
| RP11-164H13.1 | 2738 | 0.222197614 | | 15.6436754 | 6.137592371 | 0.000947335 | Up | 4.10E-06 |
| RP5-1050D4.3 | 474 | 0.41235463 | | 28.22435124 | 6.096911005 | 2.41E-05 | Up | 1.87E-08 |
| SPIC | 1048 | 0.329317916 | | 14.49310667 | 5.459742066 | 5.90E-05 | Up | 9.00E-08 |
| AC132216.1 | 1150 | 0.166662997 | | 5.930147056 | 5.153062146 | 0.004794778 | Up | 3.98E-05 |
| TIMD4 | 1145 | 0.973069542 | | 33.43918771 | 5.102853079 | 2.51E-05 | Up | 2.28E-08 |
| RP11-148O21.3 | 435 | 0.330091491 | | 11.31904701 | 5.099742739 | 0.000694649 | Up | 2.65E-06 |
| CTCFL | 1647 | 0.338577839 | | 11.07375389 | 5.031513006 | 0.003406408 | Up | 2.50E-05 |
| RP11-856F16.2 | 593 | 0.298822234 | | 9.708705329 | 5.02191952 | 0.04770321 | Up | 0.001077026 |
| AICDA | 2819 | 2.67356141 | | 78.82650019 | 4.881846002 | 0.001117413 | Up | 5.06E-06 |
| CR2 | 3185 | 31.83482618 | | 885.7979327 | 4.798299838 | 1.30E-05 | Up | 7.48E-09 |
| AC084082.3 | 954 | 0.205694026 | | 5.668605207 | 4.784421993 | 0.002070559 | Up | 1.26E-05 |
| OR2T10 | 1049 | 0.194873021 | | 5.364572091 | 4.782856822 | 0.037011925 | Up | 0.000719536 |
| C7orf62 | 1023 | 0.225500075 | | 6.082765076 | 4.753527471 | 0.043228616 | Up | 0.000918701 |
| U62631.5 | 435 | 1.439111207 | | 37.11320046 | 4.688682432 | 0.00011272 | Up | 2.22E-07 |
| OR2T3 | 1008 | 0.163971681 | | 4.224669574 | 4.687319933 | 0.037515532 | Up | 0.000738443 |
| TUSC5 | 3596 | 0.767229109 | | 19.53306107 | 4.670116786 | 0.000338387 | Up | 1.02E-06 |
| CCR12P | 998 | 0.264008766 | | 6.434387021 | 4.607144978 | 0.007874612 | Up | 8.12E-05 |
| FCAMR | 1239 | 1.704096243 | | 40.85500206 | 4.583434002 | 2.03E-05 | Up | 1.34E-08 |
| TDGF1 | 1771 | 0.298357686 | | 7.059197657 | 4.564389371 | 0.021071929 | Up | 0.000314222 |
| RP11-855A2.5 | 414 | 0.262265404 | | 6.150817578 | 4.551678771 | 0.000371125 | Up | 1.24E-06 |
| NPY5R | 3183 | 0.283769357 | | 6.228982932 | 4.456205906 | 0.001554482 | Up | 8.00E-06 |
| FCER2 | 1560 | 7.220087687 | | 147.5708071 | 4.353247183 | 5.90E-05 | Up | 8.52E-08 |
| CLEC4M | 1472 | 4.503026585 | | 88.38596023 | 4.294850321 | 0.006953306 | Up | 6.84E-05 |
| MS4A1 | 2095 | 89.76268769 | | 1700.129458 | 4.243384922 | 3.07E-06 | Up | 7.45E-10 |
| RP11-564A8.8 | 466 | 2.857734672 | | 52.26699049 | 4.192956212 | 0.000128327 | Up | 2.58E-07 |
| RP11-84D1.2 | 2443 | 0.242836503 | | 4.290145178 | 4.142969264 | 0.046674073 | Up | 0.001036262 |
| CTD-2215E18.1 | 952 | 0.480136379 | | 8.20549655 | 4.095074485 | 0.031224733 | Up | 0.000553682 |
| RPL15P4 | 580 | 0.508569619 | | 8.536244919 | 4.069084386 | 0.007632176 | Up | 7.71E-05 |
| TDRD15 | 6135 | 2.28895795 | | 37.96925723 | 4.052068912 | 0.004306726 | Up | 3.43E-05 |
| AL355390.1 | 2328 | 1.027817546 | | 16.99830032 | 4.047734407 | 0.002858266 | Up | 2.00E-05 |
| RP11-78B10.2 | 682 | 0.245754943 | | 3.989315288 | 4.020848806 | 0.011307046 | Up | 0.000137385 |
| RP11-412P11.1 | 957 | 0.470852576 | | 7.513143065 | 3.996069247 | 0.047688386 | Up | 0.001075513 |
| TCL1A | 763 | 25.3798175 | | 400.5908424 | 3.980375841 | 0.000222681 | Up | 5.76E-07 |
| RP4-733B9.1 | 373 | 0.423691114 | | 6.347987856 | 3.905214593 | 0.025538687 | Up | 0.000410731 |
| PRAMENP | 2334 | 1.723130593 | | 25.45044581 | 3.884586977 | 0.002444 | Up | 1.61E-05 |
| FBXO40 | 5926 | 1.408429837 | | 20.47909423 | 3.861992307 | 0.000809549 | Up | 3.25E-06 |
| CNR2 | 5254 | 20.10471873 | | 280.6414714 | 3.80312216 | 0.000108854 | Up | 2.09E-07 |
| NPHS1 | 3741 | 1.403315668 | | 19.49019389 | 3.795836965 | 0.000876643 | Up | 3.66E-06 |
| RP11-542M13.3 | 750 | 0.42533205 | | 5.869663435 | 3.786616305 | 0.028367138 | Up | 0.000480034 |
| RP11-87G24.6 | 722 | 0.245051557 | | 3.349275889 | 3.772692004 | 0.029551766 | Up | 0.000501386 |
| FCRL1 | 1480 | 41.82699908 | | 569.572954 | 3.767374243 | 0.000327694 | Up | 9.63E-07 |
| RP11-523O18.7 | 559 | 1.017858266 | | 13.81790353 | 3.762930155 | 0.000595043 | Up | 2.18E-06 |
| OCM | 695 | 0.380199326 | | 5.088709578 | 3.742471976 | 0.025480239 | Up | 0.00040754 |
| AC079767.4 | 492 | 1.857529269 | | 24.05191773 | 3.69469508 | 3.88E-05 | Up | 4.48E-08 |
| PLCXD3 | 7538 | 5.870436322 | | 73.43741892 | 3.644975712 | 0.000338773 | Up | 1.05E-06 |
| GAPDHP31 | 1014 | 0.542533932 | | 6.71478403 | 3.629555721 | 0.025173573 | Up | 0.000401523 |
| NOL4 | 1375 | 0.669617631 | | 8.284538465 | 3.629011907 | 0.018410712 | Up | 0.000261929 |
| RP11-398A8.1 | 838 | 0.550596166 | | 6.79229017 | 3.624831623 | 0.039106709 | Up | 0.000782491 |
| OSTN | 3134 | 0.828452099 | | 10.02525581 | 3.597076957 | 0.005233831 | Up | 4.42E-05 |
| CTC-248O19.1 | 911 | 1.1460657 | | 13.72109684 | 3.581634156 | 0.005705023 | Up | 5.02E-05 |
| RP5-894D12.3 | 3265 | 0.933363647 | | 11.14844828 | 3.578259832 | 0.000151903 | Up | 3.36E-07 |
| CRB1 | 1952 | 1.40965717 | | 16.67882538 | 3.564601444 | 0.026041654 | Up | 0.000424509 |
| RP11-344B23.2 | 520 | 0.653710317 | | 7.705325678 | 3.559132564 | 0.037043584 | Up | 0.000722606 |
| CLEC17A | 1462 | 12.24585141 | | 143.1700437 | 3.547364672 | 8.16E-05 | Up | 1.41E-07 |
| RP11-210K20.3 | 283 | 0.570157624 | | 6.634113318 | 3.540470931 | 0.041913479 | Up | 0.000877789 |
| ITGAD | 3912 | 2.70450027 | | 31.46380214 | 3.540259073 | 3.02E-05 | Up | 3.00E-08 |
| RP11-428G5.5 | 852 | 3.622111433 | | 41.62365181 | 3.522500708 | 0.002053146 | Up | 1.22E-05 |
| KRT72 | 1679 | 1.718573728 | | 19.57275632 | 3.509563286 | 0.029677109 | Up | 0.000508066 |
| RP11-434H14.1 | 622 | 0.755486555 | | 8.600051236 | 3.508867269 | 0.001428445 | Up | 7.01E-06 |
| RP11-876N24.1 | 1161 | 0.793967343 | | 8.835321085 | 3.476130991 | 0.010936436 | Up | 0.000130473 |
| RP11-171I2.1 | 3590 | 0.632794925 | | 7.012070416 | 3.470030549 | 0.01143258 | Up | 0.000139668 |
| GDF10 | 2458 | 4.566919423 | | 50.27054367 | 3.460420051 | 0.000625496 | Up | 2.34E-06 |
| HLA-DPB2 | 776 | 1.943882577 | | 21.12481404 | 3.441925663 | 3.68E-05 | Up | 4.07E-08 |
| RP11-429P3.5 | 666 | 1.093752724 | | 11.7677871 | 3.427484536 | 0.023769773 | Up | 0.000369155 |
| RP11-433M22.1 | 333 | 0.429068732 | | 4.613369976 | 3.426540322 | 0.009484481 | Up | 0.000104973 |
| RP11-693J15.5 | 10626 | 22.16235028 | | 235.6344863 | 3.410367911 | 2.26E-05 | Up | 1.70E-08 |
| CD22 | 1064 | 154.506564 | | 1619.566679 | 3.389867831 | 5.90E-05 | Up | 8.93E-08 |
| RP1-245M18.2 | 465 | 2.095072212 | | 21.88660603 | 3.384976377 | 0.003189844 | Up | 2.31E-05 |
| MTRNR2L6 | 1447 | 1.394782815 | | 14.54719249 | 3.38262835 | 0.024373861 | Up | 0.000382306 |
| TCL1B | 1724 | 1.157746372 | | 11.96873873 | 3.369879987 | 0.048504682 | Up | 0.001101914 |
| RP11-861A13.4 | 2592 | 19.04973193 | | 196.3738096 | 3.365759929 | 0.00011272 | Up | 2.22E-07 |
| GLYCTK-AS1 | 643 | 3.01184017 | | 30.51257123 | 3.34068664 | 0.001601474 | Up | 8.46E-06 |
| RP11-8L8.2 | 943 | 1.590649023 | | 15.99100598 | 3.329573255 | 0.008823914 | Up | 9.43E-05 |
| ATP5A1P3 | 1507 | 0.800167293 | | 7.937509807 | 3.310312905 | 0.001089489 | Up | 4.89E-06 |
| PAX5 | 4065 | 149.2327268 | | 1452.399363 | 3.282802344 | 2.41E-06 | Up | 4.02E-10 |
| NCR3 | 875 | 5.004978561 | | 48.57970334 | 3.278917885 | 0.00299764 | Up | 2.13E-05 |
| RP11-210H10__A.1 | 499 | 1.108171432 | | 10.60874027 | 3.259000369 | 0.017995783 | Up | 0.000253429 |
| RP11-148O21.2 | 594 | 1.809583435 | | 17.18606131 | 3.247509414 | 0.017159205 | Up | 0.000236164 |
| TRAV29DV5 | 469 | 1.221390222 | | 11.55271581 | 3.241635934 | 0.003386802 | Up | 2.48E-05 |
| FAM129C | 3136 | 91.37342052 | | 860.3437628 | 3.235066757 | 0.000150951 | Up | 3.30E-07 |
| AGGF1P2 | 809 | 1.676060105 | | 15.64590206 | 3.222639048 | 0.01613687 | Up | 0.000217102 |
| RP1-164L12.1 | 802 | 1.152515131 | | 10.75567124 | 3.222239967 | 0.01566953 | Up | 0.000208046 |
| BANK1 | 2629 | 59.70043527 | | 553.000245 | 3.211466764 | 8.82E-06 | Up | 3.51E-09 |
| SPIB | 1433 | 47.21581112 | | 436.8532262 | 3.209806685 | 7.56E-05 | Up | 1.25E-07 |
| RP11-458D21.6 | 554 | 1.733658995 | | 15.86390135 | 3.193855552 | 0.025122102 | Up | 0.000400147 |
| FCRL2 | 2248 | 57.59212379 | | 520.5434697 | 3.176075216 | 5.90E-05 | Up | 8.81E-08 |
| FCRL3 | 3397 | 59.75412327 | | 537.3585148 | 3.168774773 | 2.51E-05 | Up | 2.22E-08 |
| TRAT1 | 1845 | 8.361609617 | | 74.91427406 | 3.16338804 | 0.000336665 | Up | 9.97E-07 |
| CIR1P2 | 1342 | 2.446030614 | | 21.88710748 | 3.16156694 | 0.004634141 | Up | 3.79E-05 |
| RP11-403N16.3 | 3007 | 1.530216562 | | 13.66641358 | 3.158826944 | 0.023788167 | Up | 0.000370491 |
| FAM177B | 1345 | 7.927211452 | | 70.50666093 | 3.152874194 | 0.000820285 | Up | 3.33E-06 |
| CTD-2547L16.3 | 1119 | 1.113835762 | | 9.877187111 | 3.148563722 | 0.015915958 | Up | 0.000212724 |
| PKHD1L1 | 9601 | 16.37934042 | | 144.2886827 | 3.139008979 | 2.26E-05 | Up | 1.70E-08 |
| ANKRD1 | 1979 | 1.010250528 | | 8.897260029 | 3.138648011 | 0.025985125 | Up | 0.000422298 |
| RP11-528G1.2 | 635 | 0.891549574 | | 7.811764716 | 3.131261571 | 0.015631737 | Up | 0.000206508 |
| MCCD1 | 955 | 2.699219789 | | 23.57514924 | 3.126652543 | 0.005513463 | Up | 4.76E-05 |
| SBF1P1 | 5631 | 1.676323489 | | 14.62240321 | 3.124807954 | 0.009769788 | Up | 0.000109642 |
| TTC24 | 1939 | 7.679802078 | | 66.4546823 | 3.113229817 | 0.000785533 | Up | 3.12E-06 |
| FDCSP | 566 | 14.98553386 | | 128.8199642 | 3.10371381 | 0.047174602 | Up | 0.001056754 |
| RP11-159H10.3 | 828 | 1.483820268 | | 12.6091876 | 3.08708707 | 0.024809937 | Up | 0.000392434 |
| KB-1507C5.4 | 563 | 0.404514517 | | 3.436032008 | 3.086480091 | 0.038556694 | Up | 0.000767454 |
| AC104809.4 | 2274 | 3.87707306 | | 32.87890188 | 3.084122289 | 0.021275949 | Up | 0.000320084 |
| CTB-133G6.1 | 1558 | 42.86301102 | | 361.090731 | 3.074556283 | 3.25E-05 | Up | 3.52E-08 |
| C4BPB | 1001 | 1.499598403 | | 12.46456118 | 3.055183994 | 0.006848689 | Up | 6.72E-05 |
| TLR10 | 2702 | 34.11293827 | | 283.0958374 | 3.052899606 | 7.63E-05 | Up | 1.30E-07 |
| CXXC11 | 2398 | 2.220280432 | | 18.09832453 | 3.027042332 | 0.016797542 | Up | 0.000230444 |
| OTOA | 1608 | 3.737442861 | | 30.19645801 | 3.014255906 | 0.001089489 | Up | 4.87E-06 |
| RP11-4M23.2 | 1267 | 7.809618958 | | 62.98566599 | 3.01169948 | 0.018635407 | Up | 0.000266355 |
| LTB | 916 | 153.1453846 | | 1231.541527 | 3.007491483 | 3.01E-05 | Up | 2.92E-08 |
| SETP9 | 832 | 0.442961092 | | 3.562033394 | 3.007449152 | 0.026168333 | Up | 0.000427795 |
| AP003774.1 | 672 | 1.299307828 | | 10.43587886 | 3.005736927 | 0.005762584 | Up | 5.09E-05 |
| AC005037.6 | 298 | 0.897282415 | | 7.194633346 | 3.003287123 | 0.02002261 | Up | 0.000293709 |
| BLK | 2205 | 76.81284395 | | 610.8015393 | 2.991284227 | 0.001343787 | Up | 6.47E-06 |
| ART4 | 1549 | 15.66385634 | | 123.9735836 | 2.984521399 | 3.77E-07 | Up | 3.33E-11 |
| SELL | 1644 | 92.98255948 | | 733.3812343 | 2.979531307 | 5.37E-05 | Up | 7.12E-08 |
| RP11-217B1.2 | 1109 | 3.244762729 | | 25.39865865 | 2.968567417 | 0.007539515 | Up | 7.56E-05 |
| ANTXRLP1 | 430 | 2.094049273 | | 16.38551745 | 2.968053939 | 0.000371125 | Up | 1.21E-06 |
| DNAH8 | 13049 | 12.82964692 | | 100.0730121 | 2.963499585 | 0.033521058 | Up | 0.000615714 |
| VPREB3 | 610 | 25.61789172 | | 197.4390844 | 2.946183954 | 9.58E-05 | Up | 1.80E-07 |
| CD79B | 1053 | 55.65160645 | | 428.1250991 | 2.943537179 | 0.000152559 | Up | 3.40E-07 |
| SNORA46 | 135 | 2.893920152 | | 21.35793504 | 2.883675147 | 0.006782906 | Up | 6.59E-05 |
| CD19 | 2040 | 79.15143988 | | 578.613146 | 2.869911601 | 0.000129707 | Up | 2.64E-07 |
| LINC00861 | 4675 | 81.67022058 | | 591.417193 | 2.856294155 | 3.75E-06 | Up | 9.94E-10 |
| TRBV29-1 | 402 | 3.533120537 | | 25.53601744 | 2.853518667 | 0.004570326 | Up | 3.72E-05 |
| AC022182.1 | 653 | 2.126535829 | | 15.26695099 | 2.843834898 | 0.002811491 | Up | 1.95E-05 |
| AC098824.6 | 980 | 1.283571515 | | 9.195255288 | 2.84072595 | 0.021428667 | Up | 0.000322855 |
| LILRA4 | 1133 | 8.464483184 | | 60.59167486 | 2.839625695 | 0.000391291 | Up | 1.33E-06 |
| LY86-AS1 | 2819 | 3.38596506 | | 24.11182153 | 2.832101652 | 0.000872535 | Up | 3.60E-06 |
| TRBV7-3 | 397 | 1.639012896 | | 11.61670672 | 2.825302019 | 0.024864661 | Up | 0.000394131 |
| TRAV26-1 | 537 | 0.868081363 | | 6.143173726 | 2.823082008 | 0.006581653 | Up | 6.34E-05 |
| CALM2P3 | 449 | 1.13288678 | | 7.978453365 | 2.816105419 | 0.038075283 | Up | 0.000755348 |
| SLC26A7 | 1685 | 6.751289405 | | 47.23588387 | 2.806648285 | 0.006053587 | Up | 5.56E-05 |
| LMOD3 | 4086 | 3.384254245 | | 23.60039047 | 2.801900868 | 0.000391291 | Up | 1.34E-06 |
| LRRC4C | 2246 | 3.733013368 | | 25.98565181 | 2.799302667 | 0.000977792 | Up | 4.27E-06 |
| CD1E | 861 | 10.5650464 | | 73.11962703 | 2.790959608 | 0.001146968 | Up | 5.22E-06 |
| RP11-534L6.3 | 584 | 1.242478091 | | 8.591570586 | 2.789701475 | 0.01491755 | Up | 0.000194466 |
| TSPAN8 | 1168 | 3.009817312 | | 20.69005027 | 2.781189323 | 0.014901737 | Up | 0.000193572 |
| TCL6 | 2527 | 30.96520181 | | 212.620904 | 2.77956369 | 0.001157648 | Up | 5.37E-06 |
| ENAM | 5679 | 9.377041479 | | 63.52545869 | 2.760130168 | 0.015692619 | Up | 0.000208699 |
| AC004906.3 | 969 | 2.035375084 | | 13.76442806 | 2.757578077 | 0.005838986 | Up | 5.21E-05 |
| GALNT8 | 2380 | 4.474059597 | | 30.22802781 | 2.756230476 | 0.010161554 | Up | 0.000118977 |
| GZMK | 1509 | 17.48002464 | | 117.4457133 | 2.748214933 | 0.000792536 | Up | 3.17E-06 |
| PLA2G2D | 2672 | 46.3665153 | | 309.8274764 | 2.740309883 | 0.005732028 | Up | 5.05E-05 |
| RP11-455G16.1 | 2097 | 1.679372336 | | 11.20947387 | 2.738724532 | 0.037011925 | Up | 0.000719513 |
| RP5-1073O3.2 | 773 | 0.843583035 | | 5.566320427 | 2.722121973 | 0.049533408 | Up | 0.001133668 |
| LINC00402 | 3340 | 19.93708461 | | 131.2485947 | 2.71877561 | 0.021088978 | Up | 0.000315823 |
| PRSS35 | 2440 | 3.382166975 | | 22.11748407 | 2.709167493 | 0.010161554 | Up | 0.000118729 |
| CTD-2306M10.1 | 975 | 3.303905591 | | 21.55380342 | 2.705698104 | 0.007375315 | Up | 7.35E-05 |
| RP1-102G20.2 | 395 | 2.817017072 | | 18.27352251 | 2.697514551 | 0.003815894 | Up | 2.92E-05 |
| RP11-172E10.1 | 545 | 5.405553061 | | 34.82484665 | 2.687602862 | 0.010634617 | Up | 0.000125926 |
| FCRLA | 2064 | 30.28768588 | | 194.3460079 | 2.681824214 | 0.006712131 | Up | 6.51E-05 |
| CTD-2311B13.7 | 4227 | 6.623904867 | | 42.30209625 | 2.674975299 | 0.011121605 | Up | 0.000134395 |
| PLIN1 | 2279 | 14.93721871 | | 94.47300207 | 2.660990558 | 0.00040072 | Up | 1.43E-06 |
| STAP1 | 1511 | 10.19462301 | | 64.34008693 | 2.657909458 | 0.000715328 | Up | 2.75E-06 |
| CR1 | 6710 | 112.816571 | | 709.2691552 | 2.652354217 | 2.26E-05 | Up | 1.62E-08 |
| GOLGA5P1 | 980 | 2.861251009 | | 17.98144086 | 2.651790658 | 0.006166254 | Up | 5.78E-05 |
| TRBV5-6 | 384 | 1.880774739 | | 11.80177656 | 2.649605077 | 0.006439807 | Up | 6.16E-05 |
| THEMIS | 3862 | 43.31697555 | | 270.2426609 | 2.641251018 | 1.44E-05 | Up | 8.97E-09 |
| CD48 | 1160 | 103.2409043 | | 643.0030266 | 2.638810845 | 2.10E-06 | Up | 2.78E-10 |
| CD40LG | 1817 | 7.68419031 | | 47.82543684 | 2.637812991 | 0.005292839 | Up | 4.49E-05 |
| LRMP | 1504 | 54.40359535 | | 338.4903872 | 2.63734096 | 0.000269183 | Up | 7.49E-07 |
| NPAP1P4 | 3310 | 13.42813984 | | 83.33231599 | 2.63361661 | 0.025585045 | Up | 0.000412042 |
| LINC00494 | 847 | 9.167851092 | | 56.86297932 | 2.632834174 | 0.003675552 | Up | 2.75E-05 |
| AC092667.2 | 2586 | 17.87537408 | | 110.4276434 | 2.627056028 | 0.045850997 | Up | 0.001012524 |
| CCL19 | 687 | 106.3651798 | | 655.1820564 | 2.622869906 | 0.002444 | Up | 1.61E-05 |
| FAM65B | 2583 | 174.9225646 | | 1077.348633 | 2.622696874 | 1.01E-05 | Up | 4.47E-09 |
| RP11-445F6.2 | 914 | 2.990409552 | | 18.41408456 | 2.622394689 | 0.038183472 | Up | 0.000759047 |
| BTLA | 1026 | 31.10848944 | | 191.3814591 | 2.621070821 | 0.015867206 | Up | 0.000211607 |
| RP11-564A8.4 | 611 | 11.52295907 | | 70.68303346 | 2.616852711 | 0.01403745 | Up | 0.000178623 |
| CCR6 | 3273 | 57.15819761 | | 349.4484356 | 2.612047259 | 3.88E-05 | Up | 4.54E-08 |
| RP11-570L15.2 | 506 | 2.236970876 | | 13.60997141 | 2.605045657 | 0.047154702 | Up | 0.001055267 |
| RP5-1051J4.6 | 836 | 7.264840045 | | 44.07751857 | 2.601040068 | 0.026942835 | Up | 0.000448945 |
| CYP1B1 | 5073 | 413.5366214 | | 2505.31776 | 2.598906598 | 4.28E-10 | Up | 9.46E-15 |
| C7 | 3495 | 426.0264905 | | 2575.720695 | 2.595961114 | 0.00552236 | Up | 4.78E-05 |
| CCR7 | 2173 | 132.3813919 | | 790.3060283 | 2.577711068 | 0.001605164 | Up | 8.51E-06 |
| FAIM3 | 1109 | 126.9017055 | | 757.417038 | 2.577376416 | 0.000338387 | Up | 1.02E-06 |
| SLITRK4 | 3074 | 14.11686802 | | 83.76289162 | 2.568891201 | 0.003741789 | Up | 2.85E-05 |
| VNN2 | 1608 | 14.78728012 | | 87.11770898 | 2.558609298 | 0.00057141 | Up | 2.08E-06 |
| DACT3-AS1 | 381 | 2.448114093 | | 14.38667063 | 2.554990061 | 0.00776959 | Up | 7.95E-05 |
| RP11-272K23.3 | 734 | 0.741616345 | | 4.332276169 | 2.546380268 | 0.034008774 | Up | 0.000627343 |
| CLECL1 | 1069 | 5.089092143 | | 29.64752041 | 2.542431231 | 8.30E-05 | Up | 1.49E-07 |
| TBC1D27 | 2662 | 42.6035192 | | 247.9135638 | 2.540792694 | 0.008363152 | Up | 8.79E-05 |
| CETP | 890 | 15.85574573 | | 91.99167626 | 2.536497595 | 0.002029093 | Up | 1.18E-05 |
| P2RX5 | 1836 | 185.4288412 | | 1071.007608 | 2.530031169 | 3.79E-05 | Up | 4.27E-08 |
| RP1-228H13.2 | 418 | 1.074178847 | | 6.193357971 | 2.527487619 | 0.017515135 | Up | 0.000242848 |
| RP11-111M22.5 | 710 | 3.876544064 | | 22.3383494 | 2.526679619 | 0.022449279 | Up | 0.000340712 |
| COL19A1 | 6232 | 22.02399587 | | 126.6652686 | 2.523872845 | 0.034344995 | Up | 0.000635063 |
| PARP15 | 3744 | 119.5310012 | | 684.4765757 | 2.51761633 | 3.25E-05 | Up | 3.46E-08 |
| CCDC141 | 3591 | 68.70249638 | | 389.4945862 | 2.503168849 | 0.000137071 | Up | 2.85E-07 |
| F5 | 7024 | 36.34400576 | | 206.0339885 | 2.503093008 | 6.98E-06 | Up | 2.31E-09 |
| AC008850.3 | 362 | 1.919945208 | | 10.88348405 | 2.503003425 | 0.007874612 | Up | 8.11E-05 |
| ABCD2 | 6238 | 16.00585018 | | 90.71024445 | 2.502666182 | 2.51E-05 | Up | 2.25E-08 |
| PVRIG | 1583 | 40.67259662 | | 229.3819758 | 2.495623029 | 0.005838986 | Up | 5.19E-05 |
| XKR4 | 19880 | 19.10818286 | | 107.605183 | 2.493485076 | 0.024373861 | Up | 0.000381723 |
| PLEKHG7 | 3358 | 6.796614683 | | 38.19446893 | 2.490475491 | 0.008440177 | Up | 8.89E-05 |
| TIFAB | 5923 | 32.35125503 | | 180.1757136 | 2.477510973 | 0.005172911 | Up | 4.35E-05 |
| RP11-713N11.3 | 1186 | 13.42201144 | | 74.73026062 | 2.477091661 | 0.009784188 | Up | 0.000110249 |
| CTD-2544H17.1 | 583 | 4.222844134 | | 23.49608329 | 2.476133381 | 0.00727839 | Up | 7.22E-05 |
| RP3-455J7.4 | 2523 | 16.25207393 | | 90.1577124 | 2.471827078 | 0.000213601 | Up | 5.29E-07 |
| ARHGAP15 | 1201 | 63.86243183 | | 352.6058771 | 2.465017125 | 5.82E-07 | Up | 6.43E-11 |
| PRKCB | 1957 | 237.832335 | | 1305.404388 | 2.456480016 | 1.10E-05 | Up | 5.45E-09 |
| GAPT | 1124 | 13.89031928 | | 76.14212137 | 2.454615004 | 0.005597266 | Up | 4.87E-05 |
| GRIA4 | 679 | 5.324834357 | | 29.1799349 | 2.454168113 | 0.002869805 | Up | 2.02E-05 |
| AC017035.2 | 1115 | 5.601396521 | | 30.64498074 | 2.451792333 | 0.030775261 | Up | 0.000541861 |
| C1orf200 | 1823 | 20.29823904 | | 110.1969595 | 2.44065794 | 0.003725804 | Up | 2.83E-05 |
| TRBV20-1 | 413 | 8.381917248 | | 45.41571949 | 2.437839553 | 0.002814001 | Up | 1.96E-05 |
| AHCYP1 | 1273 | 7.447387987 | | 40.35102663 | 2.437798955 | 0.014522109 | Up | 0.000186074 |
| RP11-1094M14.5 | 849 | 15.75695542 | | 85.33920041 | 2.437219791 | 8.30E-05 | Up | 1.49E-07 |
| RAX2 | 2145 | 2.811747645 | | 15.22292093 | 2.436706183 | 0.035480696 | Up | 0.000670172 |
| TESPA1 | 1312 | 40.56961164 | | 218.0873722 | 2.42643484 | 0.00238399 | Up | 1.52E-05 |
| SP140 | 2727 | 74.42469035 | | 398.2464661 | 2.419808341 | 4.00E-05 | Up | 4.78E-08 |
| BFSP2-AS1 | 894 | 2.559785519 | | 13.67763496 | 2.417723952 | 0.029677109 | Up | 0.000510905 |
| RP11-455F5.5 | 520 | 18.13707949 | | 96.84196839 | 2.416690238 | 0.020176024 | Up | 0.000296405 |
| LTA | 1377 | 5.631940166 | | 30.05710622 | 2.416002207 | 0.014182056 | Up | 0.00018109 |
| SERPINA9 | 1156 | 6.318041472 | | 33.7176863 | 2.41595623 | 0.048504682 | Up | 0.001102623 |
| CTA-250D10.23 | 1078 | 93.85758961 | | 496.8648563 | 2.404308187 | 0.001450639 | Up | 7.21E-06 |
| TXK | 1821 | 29.77953891 | | 157.5734698 | 2.403631331 | 0.000911206 | Up | 3.89E-06 |
| RP11-404F10.2 | 556 | 6.631847537 | | 34.82826693 | 2.39277594 | 0.011472405 | Up | 0.000140697 |
| LINC00892 | 1721 | 3.851739606 | | 20.08898467 | 2.38282257 | 0.027533229 | Up | 0.000461665 |
| DNAJC5B | 1318 | 3.893998373 | | 19.85635037 | 2.350276291 | 0.009691166 | Up | 0.000108131 |
| COMP | 2461 | 120.6512122 | | 615.2232579 | 2.350267633 | 0.006066748 | Up | 5.59E-05 |
| FGF9 | 4220 | 3.797604113 | | 19.36388866 | 2.350207281 | 0.035291927 | Up | 0.000663422 |
| ITGB2-AS1 | 2282 | 48.94350521 | | 249.4601087 | 2.349619799 | 3.25E-05 | Up | 3.41E-08 |
| PRG4 | 2745 | 7.293585982 | | 36.92784271 | 2.340008771 | 0.011832124 | Up | 0.000145856 |
| SLAMF6 | 2712 | 55.90604622 | | 282.62796 | 2.337827973 | 0.000214632 | Up | 5.36E-07 |
| RP11-664D1.1 | 678 | 3.242922118 | | 16.28578869 | 2.328247307 | 0.027952445 | Up | 0.00047072 |
| NT5DC4 | 267 | 4.812553051 | | 24.12712369 | 2.325781586 | 0.007773127 | Up | 7.97E-05 |
| RP11-436I9.3 | 901 | 1.5577197 | | 7.792726005 | 2.322692437 | 0.045241086 | Up | 0.000985568 |
| GPR174 | 1258 | 28.06249539 | | 140.161631 | 2.320376261 | 0.001150202 | Up | 5.26E-06 |
| CTD-2281E23.1 | 3014 | 2.976404647 | | 14.76962758 | 2.310990868 | 0.010517498 | Up | 0.00012361 |
| AC007386.2 | 2928 | 4.60945623 | | 22.74571683 | 2.302926429 | 0.006004054 | Up | 5.46E-05 |
| NAPSB | 957 | 81.06915353 | | 399.8297486 | 2.302160833 | 0.000295733 | Up | 8.30E-07 |
| RP11-222K16.2 | 1096 | 2.194398054 | | 10.76403413 | 2.294321717 | 0.047398153 | Up | 0.001064533 |
| TMEM156 | 1318 | 15.81425226 | | 77.15802323 | 2.286590839 | 0.002933986 | Up | 2.07E-05 |
| IRF8 | 1403 | 308.5784931 | | 1505.361188 | 2.286400261 | 2.56E-06 | Up | 5.10E-10 |
| TREML2 | 3302 | 18.35866308 | | 89.35467272 | 2.283082173 | 0.01613687 | Up | 0.00021701 |
| SCML4 | 2636 | 74.44762594 | | 360.7990988 | 2.276897986 | 4.41E-05 | Up | 5.36E-08 |
| AC006129.2 | 656 | 10.29068448 | | 49.82484329 | 2.275526322 | 0.019581748 | Up | 0.000285079 |
| KLRG1 | 1058 | 23.24559982 | | 112.3560153 | 2.273047808 | 0.001419931 | Up | 6.93E-06 |
| RP11-553L6.3 | 1624 | 7.054436896 | | 34.04552804 | 2.270862477 | 0.014814535 | Up | 0.000191457 |
| RHOH | 1029 | 142.2146649 | | 684.0542526 | 2.26604051 | 4.85E-05 | Up | 6.32E-08 |
| GIMAP7 | 1256 | 50.59499822 | | 242.8565366 | 2.263037644 | 7.56E-05 | Up | 1.24E-07 |
| MIR142 | 1625 | 236.6159069 | | 1129.498448 | 2.255063319 | 0.000236452 | Up | 6.43E-07 |
| MEF2C-AS1 | 711 | 6.119298664 | | 29.02994464 | 2.246103601 | 0.010105098 | Up | 0.000117151 |
| SIT1 | 1231 | 37.73392147 | | 178.5959271 | 2.242765236 | 0.009912235 | Up | 0.000114142 |
| RP11-960L18.1 | 605 | 5.836109855 | | 27.60385545 | 2.241790838 | 0.041646149 | Up | 0.000866766 |
| RP11-627G18.1 | 770 | 5.729157678 | | 27.00418031 | 2.236787808 | 0.015640139 | Up | 0.000206964 |
| TRDC | 720 | 4.202941332 | | 19.71913397 | 2.230124969 | 0.029677109 | Up | 0.000508683 |
| TNFRSF13B | 1242 | 43.04277056 | | 201.3781764 | 2.226064497 | 0.032213174 | Up | 0.000579276 |
| RP11-266L9.3 | 1869 | 4.018462533 | | 18.71303106 | 2.219327724 | 0.007887024 | Up | 8.16E-05 |
| CYSLTR2 | 2548 | 29.15413129 | | 135.4078182 | 2.215538799 | 0.004267233 | Up | 3.38E-05 |
| CXCL13 | 1203 | 148.6161421 | | 688.5649592 | 2.21200194 | 0.009502468 | Up | 0.000105382 |
| PPP1R1A | 1498 | 2.338886717 | | 10.81802593 | 2.209543369 | 0.022439114 | Up | 0.000339566 |
| CD79A | 1093 | 257.9962533 | | 1192.897467 | 2.209048025 | 0.013908303 | Up | 0.000176673 |
| CCR9 | 2512 | 3.799323112 | | 17.41057624 | 2.196149637 | 0.048303953 | Up | 0.001094858 |
| SLC12A3 | 3119 | 6.806971466 | | 31.15326488 | 2.194298399 | 0.03582353 | Up | 0.000682753 |
| NCF1C | 1075 | 41.23320481 | | 188.6031609 | 2.193475352 | 0.002403194 | Up | 1.55E-05 |
| CD180 | 2726 | 53.79754584 | | 245.8888538 | 2.192394073 | 0.000156085 | Up | 3.52E-07 |
| IKZF3 | 1709 | 148.9131652 | | 680.2074597 | 2.191503521 | 0.000217439 | Up | 5.50E-07 |
| ADAM28 | 2137 | 197.4879683 | | 898.7220421 | 2.186110225 | 1.10E-05 | Up | 5.43E-09 |
| SLC14A1 | 1972 | 11.44846694 | | 51.93717888 | 2.181613232 | 0.002403194 | Up | 1.56E-05 |
| GZMM | 940 | 14.21853059 | | 64.49087453 | 2.181322656 | 0.005341768 | Up | 4.54E-05 |
| RP11-830F9.6 | 1812 | 18.88832603 | | 85.0795999 | 2.1713184 | 0.002678588 | Up | 1.83E-05 |
| RASGRP2 | 1856 | 338.8198728 | | 1524.801454 | 2.170030999 | 0.003118562 | Up | 2.25E-05 |
| RP11-147L13.8 | 2919 | 18.32655718 | | 82.42672305 | 2.169176354 | 0.000180022 | Up | 4.18E-07 |
| FUT7 | 2584 | 28.42857333 | | 127.6900525 | 2.167232534 | 0.008177105 | Up | 8.53E-05 |
| C12orf42 | 1072 | 5.460021336 | | 24.37472257 | 2.158407305 | 0.02094981 | Up | 0.000311012 |
| RP11-730A19.5 | 793 | 3.479617021 | | 15.48291622 | 2.153676798 | 0.030458076 | Up | 0.000532906 |
| P2RY10 | 1597 | 29.96757939 | | 133.2747775 | 2.152929313 | 0.008653796 | Up | 9.18E-05 |
| SH2D1A | 2450 | 31.88337486 | | 141.7767152 | 2.152744358 | 0.00526673 | Up | 4.46E-05 |
| BZRAP1-AS1 | 1135 | 32.52904168 | | 144.2465666 | 2.148736754 | 0.016198491 | Up | 0.000219005 |
| RN7SL213P | 315 | 4.152435363 | | 18.36736358 | 2.145114943 | 0.022832186 | Up | 0.000347027 |
| WDFY4 | 5942 | 848.6180246 | | 3733.840218 | 2.137472965 | 1.31E-05 | Up | 7.83E-09 |
| RP11-284N8.3 | 3481 | 68.36082762 | | 300.4285228 | 2.135780021 | 0.001735234 | Up | 9.40E-06 |
| TNFRSF17 | 668 | 13.38097835 | | 58.73443884 | 2.134023071 | 0.000422145 | Up | 1.52E-06 |
| LINC00243 | 2052 | 7.276003164 | | 31.87914191 | 2.131394721 | 0.026942835 | Up | 0.000448932 |
| RP11-640B6.1 | 667 | 3.353424737 | | 14.69104558 | 2.13122995 | 0.045428461 | Up | 0.000993565 |
| IFNG-AS1 | 1791 | 20.75016818 | | 90.72670913 | 2.1284043 | 0.009788988 | Up | 0.00011202 |
| PZP | 1278 | 14.78823626 | | 64.59003823 | 2.126861676 | 0.002031107 | Up | 1.20E-05 |
| GVINP1 | 6825 | 257.5866096 | | 1117.974691 | 2.117758026 | 0.000182571 | Up | 4.32E-07 |
| IFNG | 1218 | 4.184659196 | | 18.16004399 | 2.11758566 | 0.044061528 | Up | 0.000942243 |
| CYP1B1-AS1 | 576 | 8.124369597 | | 35.129412 | 2.112351651 | 0.004401465 | Up | 3.53E-05 |
| SELP | 2966 | 37.41899058 | | 161.7135508 | 2.111598029 | 0.018303394 | Up | 0.000259998 |
| RP11-1094M14.8 | 958 | 32.09497882 | | 137.4939925 | 2.09894907 | 6.00E-05 | Up | 9.40E-08 |
| RP11-94L15.2 | 6116 | 419.9587992 | | 1793.266494 | 2.094270198 | 0.001554482 | Up | 7.94E-06 |
| GPA33 | 2549 | 5.390084992 | | 22.95450105 | 2.090397146 | 0.013589504 | Up | 0.000172023 |
| PTPRC | 2108 | 689.6098464 | | 2918.415615 | 2.081333075 | 2.26E-05 | Up | 1.59E-08 |
| HS3ST2 | 2306 | 20.05779009 | | 84.87860358 | 2.081238258 | 0.033521058 | Up | 0.000615096 |
| ATP8A1 | 6474 | 209.6253254 | | 885.6776499 | 2.07896869 | 8.53E-05 | Up | 1.56E-07 |
| CD96 | 3111 | 83.07090367 | | 349.8510265 | 2.074325571 | 0.002144266 | Up | 1.32E-05 |
| RN7SL243P | 330 | 3.762548653 | | 15.81654908 | 2.071652719 | 0.042901706 | Up | 0.000909858 |
| NUGGC | 3885 | 35.06965683 | | 147.1659445 | 2.069148639 | 0.014900335 | Up | 0.000193224 |
| CEACAM21 | 504 | 15.6130186 | | 65.0297013 | 2.058349304 | 0.01887487 | Up | 0.00027155 |
| HSPB1P2 | 546 | 1.539340445 | | 6.395464848 | 2.054736886 | 0.020969466 | Up | 0.000311767 |
| TLR7 | 5011 | 39.4893754 | | 163.5716668 | 2.050386418 | 0.000137516 | Up | 2.89E-07 |
| MCOLN2 | 1816 | 52.40408393 | | 216.5136493 | 2.046706824 | 0.00036185 | Up | 1.16E-06 |
| CNR1 | 6737 | 76.33557635 | | 314.831362 | 2.044151773 | 0.031216274 | Up | 0.000552384 |
| LINC00877 | 1229 | 8.788909479 | | 36.22530863 | 2.043241908 | 0.017995783 | Up | 0.000253358 |
| HVCN1 | 1175 | 63.59162177 | | 261.7155363 | 2.041090963 | 0.002437248 | Up | 1.60E-05 |
| CTGLF10P | 2418 | 5.906908109 | | 24.2816159 | 2.03938936 | 0.044567509 | Up | 0.000959955 |
| RP11-95M15.2 | 1779 | 7.105786636 | | 29.14193564 | 2.036030431 | 0.025590811 | Up | 0.0004127 |
| IKZF1 | 2946 | 694.6447739 | | 2838.831142 | 2.030949729 | 2.90E-05 | Up | 2.73E-08 |
| NPTX1 | 5122 | 60.04276011 | | 245.3096317 | 2.030541677 | 0.000190179 | Up | 4.58E-07 |
| CD3D | 396 | 48.22974176 | | 196.7256619 | 2.028190172 | 0.00588635 | Up | 5.28E-05 |
| PCDHAC2 | 5970 | 26.66270189 | | 108.6458239 | 2.026737832 | 0.045241086 | Up | 0.000986457 |
| CD72 | 831 | 47.75179168 | | 194.3548603 | 2.025066416 | 0.001559578 | Up | 8.11E-06 |
| CD28 | 4462 | 99.19555263 | | 402.5622629 | 2.020864592 | 0.002420277 | Up | 1.57E-05 |
| IRF4 | 4686 | 225.5837252 | | 914.5738647 | 2.019436705 | 0.001506006 | Up | 7.55E-06 |
| CD3G | 2678 | 72.23445218 | | 292.254374 | 2.016465618 | 0.001735026 | Up | 9.35E-06 |
| RP11-138I1.2 | 2166 | 3.511738408 | | 14.20695099 | 2.016339678 | 0.037043584 | Up | 0.000721108 |
| RP11-1094M14.9 | 1230 | 27.65897082 | | 111.5583705 | 2.011979386 | 0.002244641 | Up | 1.39E-05 |
| PYHIN1 | 1490 | 24.0210322 | | 96.87483076 | 2.011823739 | 0.006670522 | Up | 6.44E-05 |
| ZNF80 | 2555 | 11.09137002 | | 44.53171596 | 2.005395625 | 0.009788151 | Up | 0.000111145 |
| P2RY8 | 2264 | 197.9213536 | | 794.3781499 | 2.004898669 | 0.000357052 | Up | 1.13E-06 |
| FAM26F | 1109 | 55.04239572 | | 220.9109513 | 2.00484977 | 0.006323253 | Up | 6.02E-05 |
| FCRL5 | 4043 | 388.3696859 | | 1557.734442 | 2.003946807 | 0.004570326 | Up | 3.71E-05 |
| ADAMDEC1 | 1531 | 94.08333103 | | 373.6463264 | 1.989662293 | 0.000304959 | Up | 8.72E-07 |
| IL21R-AS1 | 2615 | 22.53560841 | | 89.44884133 | 1.988856394 | 0.026168333 | Up | 0.000427219 |
| ITGAL | 1412 | 475.4685379 | | 1883.484207 | 1.985982151 | 1.10E-05 | Up | 5.60E-09 |
| HLA-DOB | 1461 | 79.97875221 | | 316.1090179 | 1.982733515 | 0.00243499 | Up | 1.59E-05 |
| C11orf21 | 2063 | 70.31493738 | | 277.5762207 | 1.980980875 | 0.002338904 | Up | 1.48E-05 |
| ADRA2A | 3869 | 178.5289305 | | 704.684576 | 1.980819756 | 2.41E-06 | Up | 4.26E-10 |
| ZNF831 | 9404 | 208.1063706 | | 820.7583413 | 1.979636377 | 0.002480375 | Up | 1.65E-05 |
| CTC-548K16.6 | 889 | 10.38471674 | | 40.85996431 | 1.976226079 | 0.003845988 | Up | 2.98E-05 |
| RP11-126K1.6 | 618 | 3.554183541 | | 13.97637551 | 1.975400185 | 0.00757018 | Up | 7.61E-05 |
| RP11-327F22.2 | 1150 | 3.732588109 | | 14.66096262 | 1.973731611 | 0.015069495 | Up | 0.000197416 |
| LINC00426 | 1143 | 27.86175938 | | 109.4238069 | 1.973568387 | 0.011121605 | Up | 0.00013425 |
| CCL21 | 852 | 323.8435918 | | 1267.806809 | 1.96896582 | 0.035035573 | Up | 0.000656346 |
| C1orf186 | 1287 | 22.08147306 | | 86.34684753 | 1.967307089 | 0.0197567 | Up | 0.000289372 |
| S1PR4 | 1594 | 100.8284178 | | 393.3604655 | 1.963949659 | 0.005838986 | Up | 5.22E-05 |
| BTK | 1991 | 76.48611807 | | 297.4915745 | 1.959578976 | 0.000145864 | Up | 3.16E-07 |
| LINC00926 | 2437 | 157.3934528 | | 612.0859973 | 1.959358835 | 0.001588659 | Up | 8.35E-06 |
| GPR55 | 3828 | 15.13161453 | | 58.75561941 | 1.957160908 | 0.013583032 | Up | 0.000171361 |
| CLNK | 1903 | 19.97517445 | | 77.515251 | 1.956272083 | 0.002186045 | Up | 1.35E-05 |
| RLTPR | 1688 | 142.8423897 | | 551.7686198 | 1.949639235 | 0.001801935 | Up | 9.99E-06 |
| RP11-77C3.3 | 2795 | 19.30592154 | | 74.51658967 | 1.948518228 | 0.008816168 | Up | 9.40E-05 |
| MAP4K1 | 842 | 179.6428341 | | 692.9387782 | 1.947596507 | 0.000233339 | Up | 6.13E-07 |
| CXCL11 | 1606 | 43.52996202 | | 167.5346286 | 1.944378658 | 0.014128242 | Up | 0.000180091 |
| ZAP70 | 2077 | 266.7416184 | | 1023.293882 | 1.93970569 | 0.006045692 | Up | 5.54E-05 |
| PLAC8 | 1294 | 162.913134 | | 624.9776486 | 1.939701677 | 0.026550814 | Up | 0.000437567 |
| LY9 | 2099 | 85.75323545 | | 326.8758265 | 1.930479679 | 0.006240515 | Up | 5.90E-05 |
| CORO1A | 1217 | 1058.455196 | | 4008.611339 | 1.921142343 | 2.90E-05 | Up | 2.75E-08 |
| AC006160.5 | 750 | 4.807695617 | | 18.18589342 | 1.919402339 | 0.025639408 | Up | 0.00041451 |
| RGS7BP | 4160 | 8.852020481 | | 33.3413216 | 1.913232597 | 0.026824704 | Up | 0.000444451 |
| CD52 | 468 | 88.49506464 | | 332.2818316 | 1.908738508 | 0.001899 | Up | 1.07E-05 |
| PNMA3 | 3158 | 18.49166128 | | 68.98471008 | 1.899401794 | 0.022923756 | Up | 0.0003496 |
| NCF1B | 1455 | 48.40151156 | | 180.0414883 | 1.895205387 | 0.006563814 | Up | 6.31E-05 |
| RP11-876N24.2 | 575 | 15.29297497 | | 56.80655292 | 1.893188278 | 0.035663636 | Up | 0.000675991 |
| A2M-AS1 | 2187 | 26.70575227 | | 99.09633789 | 1.89168122 | 0.001801935 | Up | 9.97E-06 |
| MIR155HG | 1600 | 39.77566801 | | 146.9382944 | 1.885252368 | 0.00039838 | Up | 1.42E-06 |
| PLD4 | 671 | 56.25659337 | | 207.327173 | 1.881815116 | 0.021109349 | Up | 0.000316645 |
| SCIMP | 2313 | 132.8321649 | | 489.4911644 | 1.881678285 | 0.000136162 | Up | 2.80E-07 |
| AC008697.1 | 1580 | 7.229936175 | | 26.62155447 | 1.880539998 | 0.043456846 | Up | 0.000925472 |
| CD1C | 855 | 18.26799497 | | 66.7722418 | 1.86993018 | 0.039106709 | Up | 0.000782722 |
| CD80 | 1479 | 43.5076959 | | 158.6719427 | 1.866704524 | 9.28E-05 | Up | 1.72E-07 |
| SLC1A2 | 2481 | 37.84933756 | | 137.8204593 | 1.864450115 | 0.004687962 | Up | 3.87E-05 |
| ZMAT1 | 4711 | 100.7731926 | | 366.6995491 | 1.863486583 | 0.002031107 | Up | 1.19E-05 |
| CCR2 | 2339 | 50.20514395 | | 182.6172373 | 1.862915855 | 0.01485456 | Up | 0.000192303 |
| HLA-DQB2 | 1109 | 60.82666021 | | 221.1347392 | 1.862149985 | 0.039709818 | Up | 0.000798302 |
| TRABD2A | 2201 | 59.63913362 | | 216.2542383 | 1.858397203 | 0.000730922 | Up | 2.84E-06 |
| KCNA3 | 3346 | 163.9657107 | | 593.4194192 | 1.855657995 | 0.000987152 | Up | 4.36E-06 |
| ITK | 3746 | 192.6849738 | | 694.0175676 | 1.848728112 | 0.001588659 | Up | 8.34E-06 |
| CD53 | 1418 | 403.9630027 | | 1451.747577 | 1.845495552 | 9.64E-06 | Up | 4.04E-09 |
| PPP1R16B | 6251 | 468.2139472 | | 1682.185407 | 1.84509691 | 0.000193929 | Up | 4.71E-07 |
| TNFSF14 | 1553 | 48.09245576 | | 172.7835207 | 1.845083124 | 0.000978301 | Up | 4.30E-06 |
| RP3-323N1.2 | 430 | 19.79466029 | | 71.10025297 | 1.844743385 | 0.004652942 | Up | 3.81E-05 |
| CTD-2587H19.1 | 1904 | 14.21031583 | | 51.02348277 | 1.844222758 | 0.007666387 | Up | 7.77E-05 |
| FNDC9 | 2125 | 16.86374843 | | 60.19834118 | 1.835798482 | 0.030898984 | Up | 0.000546087 |
| BIRC3 | 4994 | 817.3184356 | | 2898.52055 | 1.826346532 | 0.010517498 | Up | 0.000123561 |
| MTND6P5 | 512 | 12.33247736 | | 43.70977152 | 1.825493199 | 0.041646149 | Up | 0.000869572 |
| HLA-DQA1 | 1341 | 542.2956629 | | 1917.485867 | 1.822064407 | 0.013583032 | Up | 0.000171641 |
| TNFSF8 | 1179 | 44.0907677 | | 155.8123036 | 1.821260657 | 0.000876455 | Up | 3.64E-06 |
| RAB37 | 1432 | 52.84194794 | | 186.6252334 | 1.820388509 | 0.016440722 | Up | 0.000223733 |
| CD69 | 1201 | 64.90076205 | | 228.7798783 | 1.817652846 | 0.011764303 | Up | 0.00014476 |
| SASH3 | 1720 | 328.319309 | | 1157.020234 | 1.81724259 | 1.23E-05 | Up | 6.53E-09 |
| UBASH3A | 2754 | 31.85759546 | | 112.0755164 | 1.814761862 | 0.00791074 | Up | 8.21E-05 |
| AC021188.4 | 740 | 10.96060094 | | 38.50172141 | 1.81259605 | 0.03130891 | Up | 0.00055679 |
| CTC-499B15.7 | 2515 | 7.203702128 | | 25.27151123 | 1.810701507 | 0.044904407 | Up | 0.00097118 |
| TNFRSF13C | 926 | 98.10454317 | | 343.0608413 | 1.806072605 | 0.046715569 | Up | 0.001038215 |
| P2RY12 | 1766 | 10.67841062 | | 37.02041969 | 1.79362432 | 0.045718533 | Up | 0.001006967 |
| KBTBD8 | 2684 | 65.72325695 | | 227.7571367 | 1.793020381 | 4.82E-05 | Up | 6.18E-08 |
| LIX1 | 529 | 7.872550341 | | 27.26669278 | 1.792236741 | 0.040671243 | Up | 0.000833803 |
| ANKRD44-IT1 | 1069 | 29.56454325 | | 102.3911196 | 1.792150701 | 0.01566953 | Up | 0.000207901 |
| AC013264.2 | 455 | 10.3556415 | | 35.80432335 | 1.789716875 | 0.023670275 | Up | 0.000365518 |
| CD3E | 1483 | 188.24157 | | 650.2533635 | 1.788416698 | 0.006166254 | Up | 5.75E-05 |
| RP11-59H7.1 | 467 | 4.721026635 | | 16.29749149 | 1.787477395 | 0.035450077 | Up | 0.000668027 |
| CD37 | 1108 | 534.1799482 | | 1841.231948 | 1.785273654 | 0.000304959 | Up | 8.68E-07 |
| JPH4 | 1550 | 20.54758736 | | 70.74988077 | 1.78375871 | 0.007874612 | Up | 8.12E-05 |
| RP11-285F7.2 | 348 | 8.792745328 | | 30.08277662 | 1.774552145 | 0.036960192 | Up | 0.000715264 |
| P2RY13 | 2764 | 36.46973653 | | 124.6421811 | 1.773020702 | 0.002300375 | Up | 1.44E-05 |
| SKAP1 | 1286 | 48.79869918 | | 166.7448289 | 1.772727425 | 0.017944884 | Up | 0.000250149 |
| LY86 | 1197 | 25.48286531 | | 87.04855535 | 1.772292851 | 0.00026745 | Up | 7.39E-07 |
| CD244 | 2463 | 18.18637149 | | 62.08887984 | 1.771477176 | 0.018635407 | Up | 0.000265859 |
| C1orf228 | 757 | 20.44700547 | | 69.69415319 | 1.769148058 | 0.009848105 | Up | 0.000113132 |
| CENPCP1 | 2578 | 12.59029395 | | 42.86623713 | 1.767529814 | 0.023085267 | Up | 0.000353424 |
| RP11-571M6.8 | 874 | 42.06009246 | | 143.0238592 | 1.76573191 | 0.005447962 | Up | 4.68E-05 |
| INPP5D | 1776 | 559.4967741 | | 1890.086039 | 1.756250191 | 0.000177915 | Up | 4.05E-07 |
| GIMAP1 | 4420 | 101.4401603 | | 342.404095 | 1.755071026 | 0.00727839 | Up | 7.17E-05 |
| PTPRCAP | 1292 | 375.9182034 | | 1267.917146 | 1.75396979 | 0.002008664 | Up | 1.16E-05 |
| GIMAP5 | 1671 | 301.7741259 | | 1016.016876 | 1.751383346 | 0.000177915 | Up | 4.09E-07 |
| POU2AF1 | 1802 | 686.5184518 | | 2307.974551 | 1.749256914 | 0.003923604 | Up | 3.05E-05 |
| RASA3-IT1 | 380 | 4.119142157 | | 13.82867914 | 1.747247541 | 0.041316107 | Up | 0.000851587 |
| RAB39B | 3505 | 22.12326978 | | 74.20253733 | 1.745903891 | 0.03182518 | Up | 0.000569487 |
| NCKAP1L | 3780 | 511.3827027 | | 1714.961076 | 1.745700564 | 1.30E-05 | Up | 7.24E-09 |
| AC147651.4 | 731 | 13.79017989 | | 46.07326893 | 1.740288685 | 0.034438544 | Up | 0.000639836 |
| CLEC10A | 1474 | 45.37261328 | | 150.8940161 | 1.733641935 | 0.041074386 | Up | 0.00084479 |
| GIMAP4 | 1975 | 135.2188182 | | 448.9392599 | 1.731224322 | 0.002566402 | Up | 1.72E-05 |
| GRAP2 | 1620 | 126.0603842 | | 417.9564476 | 1.729237652 | 0.00727839 | Up | 7.21E-05 |
| CYTIP | 1844 | 248.4740297 | | 821.4887229 | 1.7251457 | 0.000391291 | Up | 1.37E-06 |
| SCML2P2 | 540 | 10.48740456 | | 34.6685391 | 1.724969363 | 0.045241086 | Up | 0.000984473 |
| PTPN2P1 | 1072 | 26.05149733 | | 86.04652709 | 1.72375067 | 0.009284423 | Up | 0.000101734 |
| ANKRD44 | 1604 | 449.8898046 | | 1481.759941 | 1.719668159 | 7.14E-05 | Up | 1.14E-07 |
| CCR4 | 3095 | 77.19499304 | | 253.8350476 | 1.717312099 | 0.001915295 | Up | 1.09E-05 |
| CIITA | 3974 | 1622.992931 | | 5333.034432 | 1.716299926 | 0.000137516 | Up | 2.92E-07 |
| SLC19A3 | 3105 | 11.21975001 | | 36.80134424 | 1.713717933 | 0.041425354 | Up | 0.000856584 |
| LCK | 2117 | 148.2275156 | | 485.4685087 | 1.711564433 | 0.005447962 | Up | 4.69E-05 |
| RP11-645C24.5 | 1665 | 15.60400908 | | 50.74701363 | 1.701406182 | 0.008225475 | Up | 8.60E-05 |
| KMO | 4888 | 27.75237895 | | 90.20992781 | 1.700674769 | 0.013898297 | Up | 0.000176239 |
| KLRB1 | 1448 | 19.67070707 | | 63.59466504 | 1.692856926 | 0.032801796 | Up | 0.00059276 |
| FAM159A | 836 | 31.3430792 | | 101.2432423 | 1.691606791 | 0.022923756 | Up | 0.000349938 |
| SNX20 | 2893 | 149.2227236 | | 478.8298244 | 1.68204577 | 0.000371125 | Up | 1.23E-06 |
| DTHD1 | 2606 | 37.37082891 | | 119.5643845 | 1.677803239 | 0.011864352 | Up | 0.000146549 |
| ZC3H12D | 2150 | 288.6685169 | | 920.961281 | 1.673726732 | 0.000872535 | Up | 3.60E-06 |
| RP11-686D22.10 | 560 | 9.926700298 | | 31.57424865 | 1.669362263 | 0.04018638 | Up | 0.000818536 |
| RASAL3 | 3270 | 333.193959 | | 1057.326211 | 1.665986403 | 0.001525703 | Up | 7.68E-06 |
| EAF2 | 898 | 50.90606671 | | 161.1211726 | 1.662236583 | 0.001702592 | Up | 9.14E-06 |
| SLAMF1 | 1268 | 66.27049901 | | 209.5001797 | 1.660512794 | 0.017995783 | Up | 0.000254342 |
| USP30-AS1 | 656 | 15.65123755 | | 49.4723569 | 1.660345896 | 0.009476877 | Up | 0.00010468 |
| DOCK2 | 3449 | 599.1143391 | | 1893.271685 | 1.659978184 | 4.73E-05 | Up | 5.86E-08 |
| IL21R | 2247 | 189.8706268 | | 599.9615236 | 1.659853245 | 0.009912235 | Up | 0.000114306 |
| IL2RG | 1578 | 214.0614801 | | 676.238677 | 1.659507323 | 0.009649488 | Up | 0.000107226 |
| LAX1 | 2966 | 119.6054818 | | 377.7389135 | 1.659105901 | 0.036988935 | Up | 0.000716928 |
| POU2F2 | 4058 | 300.7608544 | | 949.8075127 | 1.659018363 | 0.001248706 | Up | 5.90E-06 |
| TAGAP | 3688 | 230.4390793 | | 725.501025 | 1.654592254 | 0.000236452 | Up | 6.42E-07 |
| SLFN12L | 2094 | 116.9593855 | | 368.1631253 | 1.654337499 | 0.00019415 | Up | 4.76E-07 |
| RP13-93L13.2 | 1197 | 54.29825947 | | 170.8669407 | 1.653895433 | 0.001941722 | Up | 1.12E-05 |
| SPOCK2 | 4842 | 851.0124958 | | 2672.75105 | 1.651073245 | 0.002300375 | Up | 1.44E-05 |
| RCSD1 | 2967 | 296.4717997 | | 930.6147474 | 1.650289167 | 0.010603986 | Up | 0.000125329 |
| CXCR4 | 1663 | 824.8654953 | | 2587.108971 | 1.649110029 | 0.009788988 | Up | 0.000111982 |
| CXCR3 | 1650 | 66.05770955 | | 207.0124672 | 1.647918804 | 0.016609254 | Up | 0.000227494 |
| ST6GAL1 | 2191 | 1215.122257 | | 3800.865479 | 1.645226491 | 0.000820285 | Up | 3.33E-06 |
| IPCEF1 | 4617 | 130.2889521 | | 407.4509601 | 1.644911675 | 0.002449829 | Up | 1.62E-05 |
| DOCK8 | 2725 | 898.7102851 | | 2801.166398 | 1.640099669 | 1.44E-05 | Up | 9.22E-09 |
| ARHGAP25 | 1433 | 202.0474931 | | 629.092241 | 1.638577117 | 3.25E-05 | Up | 3.38E-08 |
| RAB33A | 1258 | 10.19014857 | | 31.72088143 | 1.638257775 | 0.027952445 | Up | 0.000471164 |
| EMB | 4058 | 352.484501 | | 1096.932523 | 1.637843055 | 0.001313949 | Up | 6.27E-06 |
| HLA-DMB | 1602 | 464.3830529 | | 1444.916067 | 1.637598463 | 7.56E-05 | Up | 1.27E-07 |
| RASGRP3 | 1829 | 198.6941926 | | 617.9277262 | 1.636888402 | 0.001248706 | Up | 5.90E-06 |
| LILRB1 | 2646 | 175.3591546 | | 543.2766345 | 1.631374252 | 0.000614773 | Up | 2.27E-06 |
| AMICA1 | 2287 | 217.8205934 | | 674.5433072 | 1.630770713 | 0.00299764 | Up | 2.13E-05 |
| JAK3 | 4095 | 963.3492581 | | 2972.916684 | 1.625748192 | 0.000236452 | Up | 6.36E-07 |
| KIAA0226L | 959 | 114.4881854 | | 351.4050136 | 1.617936048 | 0.017995783 | Up | 0.000254436 |
| POPDC2 | 1744 | 41.84727126 | | 128.2784946 | 1.616073871 | 0.000181171 | Up | 4.24E-07 |
| EOMES | 2958 | 55.52578472 | | 170.0596109 | 1.61481076 | 0.007927498 | Up | 8.25E-05 |
| RP11-666A8.8 | 538 | 6.462786245 | | 19.66395369 | 1.605325242 | 0.037062278 | Up | 0.000724608 |
| C11orf65 | 2419 | 31.88767183 | | 96.34455351 | 1.595204343 | 0.000234929 | Up | 6.23E-07 |
| GRAP | 2478 | 126.0243901 | | 380.2163125 | 1.593117457 | 0.004574147 | Up | 3.73E-05 |
| GZMA | 894 | 38.90953341 | | 117.2217777 | 1.591045036 | 0.04081775 | Up | 0.000837708 |
| ERMN | 3760 | 27.21672524 | | 81.54275088 | 1.583063137 | 0.0367458 | Up | 0.000709491 |
| TMC8 | 1892 | 896.1046049 | | 2682.881416 | 1.582044231 | 0.002778547 | Up | 1.92E-05 |
| ST8SIA4 | 3638 | 191.2462158 | | 572.1666156 | 1.581004121 | 0.002673265 | Up | 1.82E-05 |
| IL12RB1 | 1788 | 132.7108438 | | 396.9077917 | 1.580517625 | 0.000357052 | Up | 1.12E-06 |
| TIGIT | 1548 | 139.260189 | | 416.1076506 | 1.579173927 | 0.011864352 | Up | 0.000146778 |
| RP11-551L14.1 | 1904 | 38.3408031 | | 114.502351 | 1.578424759 | 0.019672944 | Up | 0.00028771 |
| ZNF540 | 2927 | 18.97601013 | | 56.61670854 | 1.577051195 | 0.046202688 | Up | 0.001022734 |
| GMFG | 547 | 86.89536954 | | 258.9141822 | 1.575122786 | 0.006439807 | Up | 6.16E-05 |
| 1-Sep | 1068 | 262.5036583 | | 781.4361099 | 1.573790395 | 0.006004054 | Up | 5.44E-05 |
| RP11-571M6.7 | 699 | 194.8775479 | | 579.2962003 | 1.571733317 | 0.03782459 | Up | 0.000746209 |
| PLEK | 2496 | 285.6078678 | | 848.0769345 | 1.570159424 | 0.000872535 | Up | 3.58E-06 |
| FAM196B | 2999 | 91.84373503 | | 272.5577459 | 1.569308702 | 0.00263204 | Up | 1.79E-05 |
| GPR114 | 1646 | 149.2192103 | | 442.1155323 | 1.566990141 | 0.027996616 | Up | 0.000472527 |
| CHI3L2 | 629 | 55.57906157 | | 164.2015225 | 1.562854124 | 0.029677109 | Up | 0.000506408 |
| CD2 | 1609 | 84.9254106 | | 250.4386418 | 1.560188989 | 0.041958553 | Up | 0.000880586 |
| RNASE6 | 1061 | 54.53518192 | | 160.3844343 | 1.55627498 | 0.040113784 | Up | 0.000815285 |
| CD84 | 8009 | 514.3375922 | | 1509.935552 | 1.553699466 | 2.51E-05 | Up | 2.17E-08 |
| APBB1IP | 2771 | 140.1808528 | | 411.3178358 | 1.552964325 | 0.004267233 | Up | 3.38E-05 |
| ANGPTL1 | 2307 | 90.71433764 | | 266.15535 | 1.552866071 | 0.004345414 | Up | 3.48E-05 |
| CD1D | 2253 | 28.79960829 | | 84.2800921 | 1.549142701 | 0.036665707 | Up | 0.000704705 |
| YWHAQP5 | 1382 | 27.95180747 | | 81.73733904 | 1.548053701 | 0.022449279 | Up | 0.000340385 |
| GPR65 | 1786 | 27.71133937 | | 81.02136737 | 1.547825991 | 0.03582353 | Up | 0.000684053 |
| LINC00612 | 2213 | 30.14356665 | | 88.05623516 | 1.546575033 | 0.025538687 | Up | 0.000410613 |
| CTD-2020K17.1 | 2238 | 168.3045439 | | 489.7083769 | 1.540848748 | 0.040024601 | Up | 0.000811605 |
| TFEC | 2665 | 120.6524474 | | 350.9044858 | 1.54022121 | 0.001450639 | Up | 7.15E-06 |
| CLEC2D | 873 | 222.0642826 | | 644.4896357 | 1.537179794 | 0.004095832 | Up | 3.22E-05 |
| C5orf58 | 1180 | 27.93177179 | | 80.89350506 | 1.534116783 | 0.047351584 | Up | 0.001061765 |
| KLHL6 | 5388 | 788.1250207 | | 2280.962873 | 1.533146556 | 0.000247541 | Up | 6.78E-07 |
| PTPN7 | 1475 | 304.5952732 | | 878.950724 | 1.528888733 | 0.000371125 | Up | 1.20E-06 |
| ATP2A3 | 2346 | 1229.442048 | | 3532.927569 | 1.522860439 | 0.005367186 | Up | 4.60E-05 |
| EMR4P | 2732 | 39.32697342 | | 112.8352172 | 1.520626353 | 0.00261417 | Up | 1.76E-05 |
| CTD-2521M24.4 | 1258 | 20.8723259 | | 59.72971573 | 1.51685748 | 0.044454241 | Up | 0.000956533 |
| ARHGAP9 | 1126 | 265.3048931 | | 758.0633142 | 1.514667065 | 0.010564133 | Up | 0.00012449 |
| TRAC | 974 | 123.3869039 | | 352.4139536 | 1.514081771 | 0.047676822 | Up | 0.001073271 |
| RP11-16E12.1 | 894 | 30.00086647 | | 85.52837813 | 1.511399014 | 0.027773865 | Up | 0.000466313 |
| STAT4 | 2542 | 46.16637358 | | 131.1468329 | 1.506268652 | 0.020524352 | Up | 0.000302882 |
| FYB | 1815 | 344.9592426 | | 979.1573557 | 1.505114812 | 0.001450639 | Up | 7.18E-06 |
| HMHA1 | 2252 | 1458.630276 | | 4138.513444 | 1.504498399 | 0.001485842 | Up | 7.42E-06 |
| UCP2 | 1207 | 682.7955258 | | 1936.741624 | 1.504105991 | 5.90E-05 | Up | 8.25E-08 |
| PARVG | 1756 | 502.7167985 | | 1425.947554 | 1.504103118 | 0.001243068 | Up | 5.82E-06 |
| RASSF2 | 5427 | 489.1820226 | | 1385.38403 | 1.501842657 | 5.90E-05 | Up | 8.41E-08 |
| PIK3CG | 4233 | 264.57717 | | 748.7386043 | 1.500773562 | 0.003979363 | Up | 3.10E-05 |
| IL7R | 3106 | 982.7488792 | | 2778.310613 | 1.499313182 | 0.007659667 | Up | 7.75E-05 |
| FGD2 | 1663 | 635.5431419 | | 1796.080216 | 1.498789818 | 0.000391291 | Up | 1.33E-06 |
| HLA-DOA | 2005 | 313.9132309 | | 884.3037946 | 1.494176242 | 0.003328223 | Up | 2.43E-05 |
| SPN | 3519 | 455.7671158 | | 1281.104235 | 1.491019122 | 0.001646884 | Up | 8.77E-06 |
| TLR1 | 1516 | 114.0210376 | | 320.0895751 | 1.489175655 | 0.002814001 | Up | 1.96E-05 |
| ANKRD36BP2 | 1476 | 169.2363524 | | 473.891061 | 1.485515951 | 0.045850997 | Up | 0.001012924 |
| CD247 | 1788 | 144.3262636 | | 402.7349771 | 1.480496918 | 0.007520556 | Up | 7.53E-05 |
| FCGR2B | 3344 | 260.334368 | | 724.9454274 | 1.477506614 | 0.001929691 | Up | 1.10E-05 |
| VAV1 | 2107 | 231.8255273 | | 645.5103406 | 1.47740077 | 0.001747265 | Up | 9.57E-06 |
| LCP1 | 2353 | 1525.378321 | | 4245.336904 | 1.476711947 | 0.00261417 | Up | 1.77E-05 |
| MUC6 | 8006 | 96.31398236 | | 267.7201938 | 1.474908803 | 0.00776959 | Up | 7.94E-05 |
| PIK3AP1 | 3963 | 433.3849387 | | 1204.342624 | 1.474524962 | 8.30E-05 | Up | 1.50E-07 |
| CCR5 | 2093 | 184.4548604 | | 512.5447434 | 1.474410148 | 0.000910128 | Up | 3.86E-06 |
| ENPP2 | 2336 | 181.0893139 | | 501.7604129 | 1.470297237 | 0.000336665 | Up | 1.00E-06 |
| MEF2C | 2699 | 252.7788464 | | 699.0474646 | 1.467514678 | 0.012006174 | Up | 0.000148797 |
| RIC3 | 1518 | 79.42298239 | | 219.4774701 | 1.46644441 | 0.008659587 | Up | 9.20E-05 |
| BIN2 | 1937 | 109.1970158 | | 301.0501837 | 1.463070568 | 0.000145376 | Up | 3.12E-07 |
| CYFIP2 | 1846 | 501.8035204 | | 1383.280002 | 1.462898717 | 0.008276739 | Up | 8.67E-05 |
| RBBP8P1 | 1844 | 18.11039174 | | 49.85912973 | 1.461039948 | 0.023239737 | Up | 0.000358356 |
| FCGR2C | 757 | 206.7437207 | | 569.0254458 | 1.460649657 | 0.009407535 | Up | 0.000103703 |
| RGS13 | 1840 | 52.96334411 | | 145.2956891 | 1.455925777 | 0.045241086 | Up | 0.000984518 |
| RP11-792D21.2 | 1227 | 37.3893198 | | 102.5182833 | 1.455183095 | 0.003845988 | Up | 2.97E-05 |
| LPXN | 1588 | 206.9836801 | | 567.2131197 | 1.454373882 | 0.003725804 | Up | 2.81E-05 |
| LYL1 | 1502 | 109.0976345 | | 298.792942 | 1.453526249 | 0.003725804 | Up | 2.82E-05 |
| CELF2 | 6245 | 713.9785082 | | 1953.173229 | 1.451867356 | 0.004994693 | Up | 4.19E-05 |
| PTGIS | 2835 | 436.2246957 | | 1192.793167 | 1.451200546 | 0.01491755 | Up | 0.000194766 |
| MPEG1 | 4442 | 435.084309 | | 1188.171847 | 1.449376617 | 0.000688418 | Up | 2.62E-06 |
| C16orf54 | 2585 | 134.1579416 | | 365.9841717 | 1.447848796 | 0.049923577 | Up | 0.001149215 |
| DOCK10 | 2847 | 466.5890186 | | 1263.973333 | 1.437741768 | 5.90E-05 | Up | 8.63E-08 |
| ARHGEF6 | 5019 | 303.9685695 | | 822.6331483 | 1.436327051 | 0.000315937 | Up | 9.21E-07 |
| RP11-430C7.5 | 2219 | 44.29095761 | | 119.2494845 | 1.428898935 | 0.021088978 | Up | 0.000315873 |
| GPSM3 | 1468 | 300.4064724 | | 807.9291972 | 1.427312972 | 0.002065963 | Up | 1.25E-05 |
| CD5 | 1468 | 173.0523404 | | 465.0473306 | 1.426169102 | 0.002376998 | Up | 1.51E-05 |
| CECR1 | 2924 | 673.3383738 | | 1808.863345 | 1.425679828 | 0.000371125 | Up | 1.22E-06 |
| RP11-165F24.3 | 1273 | 33.99868718 | | 91.00745815 | 1.420505741 | 0.042834465 | Up | 0.000907486 |
| CYSLTR1 | 2915 | 42.55424177 | | 113.7614127 | 1.418636435 | 0.049556659 | Up | 0.00113721 |
| PLCL2 | 3348 | 163.046053 | | 435.4204819 | 1.417129755 | 0.004443255 | Up | 3.58E-05 |
| COL4A4 | 7101 | 179.5073823 | | 476.8514955 | 1.409496865 | 0.024782486 | Up | 0.000390905 |
| PLCG2 | 1637 | 535.375181 | | 1421.867997 | 1.409165369 | 0.000304959 | Up | 8.76E-07 |
| TLR6 | 5879 | 164.8038407 | | 434.9174352 | 1.399991681 | 0.000357052 | Up | 1.12E-06 |
| SNORA74A | 198 | 171.8149887 | | 453.2225355 | 1.399363699 | 0.032213174 | Up | 0.000578737 |
| CARD11 | 2194 | 238.3609797 | | 628.5427723 | 1.398862841 | 0.00776959 | Up | 7.91E-05 |
| HLA-DRA | 1273 | 2902.5643 | | 7632.845651 | 1.394892991 | 0.000887282 | Up | 3.74E-06 |
| RP11-796E2.4 | 4598 | 32.04196186 | | 84.19137859 | 1.393710022 | 0.011460766 | Up | 0.000140266 |
| AC011747.4 | 8839 | 35.04653941 | | 92.0340698 | 1.392896031 | 0.029815135 | Up | 0.000516393 |
| NLRC3 | 3040 | 486.5925575 | | 1276.999689 | 1.391972015 | 0.002054412 | Up | 1.23E-05 |
| BLNK | 625 | 104.2298572 | | 273.5330525 | 1.391946569 | 0.00354455 | Up | 2.62E-05 |
| SEL1L3 | 1925 | 1018.602687 | | 2671.100507 | 1.390842833 | 8.30E-05 | Up | 1.48E-07 |
| CD226 | 2331 | 46.43857143 | | 121.7719566 | 1.39078643 | 0.011121605 | Up | 0.000134152 |
| RP11-347P5.1 | 2065 | 237.3626668 | | 622.3507048 | 1.390634751 | 0.036724435 | Up | 0.000708267 |
| CSF2RB | 4863 | 657.6031056 | | 1723.750406 | 1.390261874 | 0.000357088 | Up | 1.14E-06 |
| ADAM22 | 3799 | 86.56192167 | | 226.7142781 | 1.38907082 | 0.002778547 | Up | 1.92E-05 |
| CTB-47B11.3 | 1932 | 22.2976437 | | 58.36840654 | 1.388296422 | 0.049556659 | Up | 0.001138579 |
| GPRIN3 | 13391 | 661.3175008 | | 1729.395699 | 1.386853021 | 0.000217938 | Up | 5.58E-07 |
| CXorf21 | 1855 | 25.94677573 | | 67.7314982 | 1.384271635 | 0.039894803 | Up | 0.00080552 |
| B3GAT1 | 5128 | 65.19122504 | | 170.1361892 | 1.383940355 | 0.030204631 | Up | 0.000525809 |
| RP11-43D4.2 | 411 | 33.31134464 | | 86.87868834 | 1.382988731 | 0.009077215 | Up | 9.81E-05 |
| SLC25A53 | 6224 | 38.54092267 | | 100.4416088 | 1.381894027 | 0.008467755 | Up | 8.94E-05 |
| CD6 | 3046 | 334.0220623 | | 869.3999234 | 1.380076572 | 0.023846069 | Up | 0.00037192 |
| AVPR2 | 1600 | 25.79783396 | | 67.04927896 | 1.377971878 | 0.015631737 | Up | 0.00020645 |
| DTX1 | 1817 | 196.8836502 | | 510.786748 | 1.375377785 | 0.006166254 | Up | 5.77E-05 |
| PATL2 | 1399 | 72.39421042 | | 187.5737051 | 1.373511368 | 0.029677109 | Up | 0.000507581 |
| CUBN | 11949 | 82.21310733 | | 212.9576292 | 1.373126088 | 0.004439347 | Up | 3.57E-05 |
| PPM1K | 3638 | 575.1153935 | | 1482.423827 | 1.366034617 | 0.002054412 | Up | 1.23E-05 |
| NCF1 | 1350 | 136.7061331 | | 351.8709733 | 1.363968539 | 0.044186831 | Up | 0.000948827 |
| TBC1D10C | 1247 | 569.2443366 | | 1463.233338 | 1.362039913 | 0.010155272 | Up | 0.000118231 |
| RP11-582J16.4 | 4076 | 46.8618727 | | 120.099421 | 1.357742682 | 0.031727254 | Up | 0.000566668 |
| MGAT3 | 5062 | 170.3355315 | | 436.1393759 | 1.356409838 | 0.004095832 | Up | 3.22E-05 |
| MYO1G | 3530 | 471.1907459 | | 1205.761686 | 1.355561682 | 0.001539795 | Up | 7.82E-06 |
| ARHGAP4 | 2015 | 766.7326112 | | 1949.225973 | 1.346105903 | 0.001358735 | Up | 6.57E-06 |
| PCED1B-AS1 | 1060 | 117.4731091 | | 298.5365298 | 1.345576929 | 0.00184633 | Up | 1.04E-05 |
| PLCB2 | 1082 | 397.6566382 | | 1008.453932 | 1.342550021 | 0.001003046 | Up | 4.45E-06 |
| PIM2 | 1383 | 758.5793716 | | 1922.288386 | 1.341452744 | 0.006782906 | Up | 6.61E-05 |
| GIMAP6 | 3440 | 240.1044768 | | 607.934484 | 1.340253552 | 0.001150392 | Up | 5.30E-06 |
| CD38 | 1928 | 318.2386223 | | 804.3082134 | 1.337639517 | 0.018301859 | Up | 0.000259572 |
| HCLS1 | 1663 | 695.9645705 | | 1756.283658 | 1.335440104 | 0.002046074 | Up | 1.21E-05 |
| LINC00341 | 2822 | 81.13541551 | | 204.3673954 | 1.332761357 | 0.009242093 | Up | 0.000100611 |
| HLA-DPA1 | 1043 | 1207.071768 | | 3039.577557 | 1.332359375 | 0.006066748 | Up | 5.60E-05 |
| SLAMF7 | 1779 | 280.9573115 | | 707.2557295 | 1.331881014 | 0.003845988 | Up | 2.95E-05 |
| RP11-517P14.2 | 760 | 16.1343736 | | 40.54872079 | 1.329518834 | 0.044254124 | Up | 0.00095125 |
| KCNK13 | 2522 | 30.56437043 | | 76.77748886 | 1.328832524 | 0.021072314 | Up | 0.000314693 |
| CELF2-AS1 | 2468 | 130.6571995 | | 327.8914992 | 1.327431876 | 0.032683609 | Up | 0.000589902 |
| 6-Sep | 2432 | 530.6165108 | | 1329.558357 | 1.325205629 | 0.001735234 | Up | 9.43E-06 |
| CMKLR1 | 2526 | 243.726086 | | 610.5986021 | 1.324963616 | 5.90E-05 | Up | 8.43E-08 |
| FERMT3 | 1995 | 466.1255279 | | 1167.744789 | 1.324934576 | 0.000977792 | Up | 4.28E-06 |
| CTC-457L16.2 | 2673 | 249.205767 | | 622.6908322 | 1.321178584 | 0.035035573 | Up | 0.000655858 |
| AIF1 | 607 | 69.19538954 | | 172.8368609 | 1.320663114 | 0.011472405 | Up | 0.000140915 |
| FAM46C | 5751 | 1100.23272 | | 2746.173228 | 1.31961392 | 0.04894421 | Up | 0.001116939 |
| LAT | 1407 | 240.4338811 | | 593.245613 | 1.302989317 | 0.025538687 | Up | 0.000409815 |
| SLC4A8 | 1113 | 63.21304365 | | 155.5377776 | 1.298970844 | 0.040556158 | Up | 0.000828736 |
| FAM78A | 4015 | 376.7880364 | | 926.9081502 | 1.298673227 | 0.002244641 | Up | 1.39E-05 |
| HLA-DMA | 1039 | 414.2292796 | | 1017.44163 | 1.296444592 | 0.012739601 | Up | 0.000159294 |
| HLA-DPB1 | 1258 | 815.8766606 | | 1997.985349 | 1.292123028 | 0.00603508 | Up | 5.51E-05 |
| ICAM3 | 1961 | 264.034122 | | 646.5617443 | 1.292063763 | 0.006708753 | Up | 6.49E-05 |
| CYBB | 4324 | 993.7049643 | | 2410.032611 | 1.278163191 | 0.026189843 | Up | 0.000428725 |
| GPR132 | 3098 | 193.5370682 | | 468.435501 | 1.275240504 | 0.013010467 | Up | 0.000163256 |
| SNX22 | 2181 | 222.3791508 | | 537.567532 | 1.273424471 | 0.034378903 | Up | 0.000637209 |
| LY75 | 3577 | 490.814259 | | 1183.268969 | 1.269528982 | 0.010105098 | Up | 0.0001172 |
| LAPTM5 | 2196 | 2680.026302 | | 6460.611733 | 1.269423615 | 0.000669481 | Up | 2.51E-06 |
| CD4 | 2872 | 660.7015929 | | 1592.085842 | 1.268847397 | 0.000391291 | Up | 1.33E-06 |
| PRKCQ | 2554 | 66.88832332 | | 161.0313046 | 1.26751489 | 0.020786533 | Up | 0.000308129 |
| MLANA | 2415 | 46.48014587 | | 111.5792044 | 1.263381668 | 0.034746069 | Up | 0.000647852 |
| FLI1 | 2435 | 302.3804603 | | 725.3900441 | 1.262392028 | 0.002894085 | Up | 2.04E-05 |
| N4BP2L1 | 1629 | 158.0609309 | | 378.9883874 | 1.261672833 | 0.01141224 | Up | 0.000139168 |
| KIF21B | 9103 | 455.3088149 | | 1088.011797 | 1.256776904 | 0.005838986 | Up | 5.21E-05 |
| CYTH4 | 3001 | 441.7359512 | | 1053.499352 | 1.253933269 | 0.004160788 | Up | 3.28E-05 |
| BCL2 | 2377 | 645.6022743 | | 1537.384077 | 1.251760065 | 0.004052956 | Up | 3.17E-05 |
| HLA-DRB1 | 1229 | 1157.182893 | | 2754.061071 | 1.25094365 | 0.046929158 | Up | 0.001047109 |
| LIMD2 | 1620 | 926.4049666 | | 2204.477222 | 1.250721677 | 0.028355492 | Up | 0.000479211 |
| GHRL | 868 | 55.41673231 | | 131.8533031 | 1.250540165 | 0.010650039 | Up | 0.000126579 |
| LYZ | 1388 | 1091.235096 | | 2595.281857 | 1.249929279 | 0.049556659 | Up | 0.001137485 |
| LAT2 | 1419 | 230.0144802 | | 545.75841 | 1.24653777 | 0.000338482 | Up | 1.04E-06 |
| HIN1L | 3067 | 35.68184369 | | 84.58180354 | 1.245157162 | 0.000126027 | Up | 2.51E-07 |
| RP1-111C20.4 | 1675 | 32.88595462 | | 77.77033883 | 1.241748475 | 0.024169062 | Up | 0.000377491 |
| CTSS | 1370 | 693.7339374 | | 1640.193164 | 1.241411362 | 0.001702592 | Up | 9.13E-06 |
| GIMAP8 | 4184 | 181.9777004 | | 430.0557533 | 1.240762033 | 0.001941722 | Up | 1.12E-05 |
| ARHGDIB | 1001 | 723.1777935 | | 1707.072434 | 1.239101992 | 0.006071658 | Up | 5.62E-05 |
| MGAT4A | 6483 | 461.3485252 | | 1086.931142 | 1.236331597 | 0.003055611 | Up | 2.18E-05 |
| IL10RA | 2483 | 859.735889 | | 2022.514563 | 1.234184654 | 0.000391291 | Up | 1.36E-06 |
| UNC13A | 9837 | 25.19051885 | | 59.24183795 | 1.233735563 | 0.026830737 | Up | 0.000445737 |
| FCHO1 | 2154 | 106.3839268 | | 250.1783785 | 1.233676915 | 0.025970302 | Up | 0.000421115 |
| SNORA53 | 249 | 110.3926457 | | 259.2286879 | 1.231581322 | 0.017278649 | Up | 0.000238571 |
| GAB3 | 1632 | 119.6774451 | | 280.0847947 | 1.226712383 | 0.029589632 | Up | 0.000502683 |
| FOXN3-AS2 | 1262 | 46.81789217 | | 109.3467101 | 1.223777927 | 0.01920573 | Up | 0.000277907 |
| MBNL1-AS1 | 6585 | 219.3701444 | | 511.860438 | 1.222383311 | 0.03782459 | Up | 0.000745565 |
| LAIR1 | 1169 | 366.8450942 | | 850.5945095 | 1.21330055 | 0.011034515 | Up | 0.000132611 |
| DENND2D | 1619 | 576.3464428 | | 1333.482402 | 1.210190602 | 0.003216072 | Up | 2.34E-05 |
| DOK3 | 1181 | 316.6517537 | | 732.6216182 | 1.210171203 | 0.006121326 | Up | 5.68E-05 |
| ATM | 6606 | 2324.27068 | | 5376.843983 | 1.209981519 | 0.002380343 | Up | 1.51E-05 |
| APLNR | 3609 | 320.3686904 | | 740.4429818 | 1.208655484 | 0.01502599 | Up | 0.000196514 |
| AKNA | 4402 | 1992.483227 | | 4603.768095 | 1.208247583 | 0.007887024 | Up | 8.17E-05 |
| FAM107B | 2412 | 638.9162661 | | 1474.928854 | 1.20694659 | 0.006043764 | Up | 5.53E-05 |
| C1orf162 | 1894 | 66.63212069 | | 153.379046 | 1.202811686 | 0.030458076 | Up | 0.000532912 |
| TNFSF13B | 1670 | 125.4249163 | | 288.3781495 | 1.20113788 | 0.020699139 | Up | 0.000305958 |
| FUCA1 | 2047 | 311.055207 | | 714.8431419 | 1.200456049 | 5.90E-05 | Up | 8.74E-08 |
| RP11-705C15.2 | 2588 | 318.8534524 | | 732.6771026 | 1.200284028 | 0.032052205 | Up | 0.000574966 |
| TRIM22 | 2153 | 1393.817856 | | 3201.390203 | 1.199656489 | 0.003111933 | Up | 2.23E-05 |
| GPR64 | 5240 | 48.13930383 | | 110.0366585 | 1.192697052 | 0.036783302 | Up | 0.000711028 |
| GP1BA | 2501 | 100.6556332 | | 229.3613786 | 1.188194566 | 0.009407535 | Up | 0.000103706 |
| ITPR1 | 3581 | 847.0449142 | | 1925.872944 | 1.185002152 | 0.005597266 | Up | 4.88E-05 |
| GNG2 | 2631 | 242.9063866 | | 552.0329023 | 1.184353835 | 0.03011375 | Up | 0.000522557 |
| JAK2 | 5285 | 409.4588434 | | 930.2764035 | 1.183940986 | 0.000233339 | Up | 6.09E-07 |
| GLCCI1 | 4420 | 370.6902802 | | 842.1729064 | 1.183902176 | 0.005592171 | Up | 4.86E-05 |
| ARL11 | 3760 | 78.65420529 | | 178.5972584 | 1.183114127 | 0.001150392 | Up | 5.31E-06 |
| ITGB2 | 1846 | 1864.643758 | | 4229.653408 | 1.18163942 | 0.017995783 | Up | 0.000251347 |
| IL18R1 | 2245 | 128.2026678 | | 290.4347674 | 1.179787882 | 0.023776266 | Up | 0.000369781 |
| NRROS | 2564 | 160.3057336 | | 362.5846021 | 1.177491633 | 0.014661082 | Up | 0.000188502 |
| SLC40A1 | 2287 | 772.0025932 | | 1736.710164 | 1.169679407 | 0.002031107 | Up | 1.20E-05 |
| C10orf128 | 1238 | 88.67178273 | | 199.1458253 | 1.167278251 | 0.001747265 | Up | 9.57E-06 |
| HHEX | 2824 | 128.8975227 | | 289.3954354 | 1.16681763 | 0.016577372 | Up | 0.000225959 |
| TXNIP | 2500 | 6719.075992 | | 15065.42996 | 1.164907095 | 0.003478631 | Up | 2.57E-05 |
| SIGLEC1 | 6284 | 1015.015344 | | 2270.886157 | 1.161753847 | 0.00251435 | Up | 1.68E-05 |
| SLA | 2390 | 759.5690645 | | 1698.969114 | 1.161406572 | 0.006004054 | Up | 5.46E-05 |
| RGS1 | 1555 | 897.0347651 | | 2002.647656 | 1.158672814 | 0.038852832 | Up | 0.000774207 |
| STK17B | 4802 | 1464.185494 | | 3268.660258 | 1.158601095 | 0.009772995 | Up | 0.000109894 |
| SERPINA1 | 856 | 295.7072953 | | 658.7040365 | 1.155460555 | 0.040024601 | Up | 0.000812588 |
| LCP2 | 1160 | 444.6861245 | | 989.50462 | 1.153919054 | 0.004810158 | Up | 4.01E-05 |
| LRRK2 | 4002 | 328.4032239 | | 730.1292501 | 1.152683588 | 0.003575 | Up | 2.67E-05 |
| ITGB7 | 1049 | 308.6791387 | | 685.8105436 | 1.151702099 | 0.002054412 | Up | 1.23E-05 |
| CD74 | 1258 | 9627.109032 | | 21373.05026 | 1.150618282 | 0.042109687 | Up | 0.000887316 |
| AC007038.7 | 2199 | 49.44409637 | | 109.4295276 | 1.146131897 | 0.014751368 | Up | 0.000190315 |
| PDE1B | 2301 | 104.1383495 | | 230.0088171 | 1.143187719 | 0.037344271 | Up | 0.000732596 |
| STX7 | 6750 | 1044.256283 | | 2300.839353 | 1.139684433 | 0.0111929 | Up | 0.000135736 |
| AC010761.13 | 499 | 49.44196072 | | 108.6935714 | 1.136458753 | 0.009769788 | Up | 0.000109484 |
| STAT5A | 2475 | 608.7922022 | | 1334.388369 | 1.132156834 | 0.000338482 | Up | 1.04E-06 |
| TRAF5 | 3767 | 514.1541651 | | 1126.62649 | 1.131736388 | 0.001801935 | Up | 9.96E-06 |
| RHOU | 4372 | 235.3068106 | | 514.9131192 | 1.12978595 | 0.003845988 | Up | 2.98E-05 |
| RASGRP1 | 2851 | 263.0243439 | | 575.4954126 | 1.129608096 | 0.010123529 | Up | 0.000117637 |
| MS4A6A | 1041 | 367.3994879 | | 803.8251289 | 1.129532064 | 0.003695149 | Up | 2.78E-05 |
| FGR | 2431 | 231.9368624 | | 506.5900699 | 1.127086668 | 0.01887487 | Up | 0.000272286 |
| APOE | 1108 | 2754.345325 | | 5999.84129 | 1.12321489 | 0.045598495 | Up | 0.001003316 |
| CMAHP | 1353 | 114.3625694 | | 249.0431773 | 1.12278095 | 0.02624123 | Up | 0.000430146 |
| TYROBP | 565 | 286.9798331 | | 622.3849411 | 1.116857796 | 0.010709084 | Up | 0.000127517 |
| MFNG | 1189 | 252.9504299 | | 547.2839548 | 1.113434869 | 0.008816168 | Up | 9.41E-05 |
| ARHGAP30 | 4109 | 1136.534261 | | 2452.784133 | 1.109779095 | 0.003415438 | Up | 2.51E-05 |
| CARD8 | 3356 | 700.1226946 | | 1500.992625 | 1.100237211 | 0.009242093 | Up | 0.000100998 |
| 1-Mar | 2168 | 441.9793593 | | 942.4712848 | 1.092469667 | 0.012950694 | Up | 0.00016222 |
| COLQ | 2849 | 69.0917896 | | 147.0966869 | 1.090178567 | 0.017603655 | Up | 0.000244614 |
| SLC7A7 | 2234 | 254.5453106 | | 541.8187151 | 1.08988774 | 0.035746308 | Up | 0.000679927 |
| GATA6 | 2520 | 149.6300989 | | 318.4306158 | 1.089578642 | 0.003845988 | Up | 2.98E-05 |
| TRAF1 | 5067 | 738.0457278 | | 1567.457102 | 1.086643849 | 0.006130364 | Up | 5.70E-05 |
| PIK3R5 | 2421 | 458.0818822 | | 970.3745528 | 1.082936214 | 0.00233814 | Up | 1.47E-05 |
| ADORA2A | 1291 | 174.6302377 | | 369.192578 | 1.080070163 | 0.026756582 | Up | 0.00044214 |
| NFATC2 | 6415 | 528.7584678 | | 1116.248571 | 1.077977561 | 0.029677109 | Up | 0.000509873 |
| ACAP1 | 1389 | 673.1621154 | | 1420.420574 | 1.077292271 | 0.01133658 | Up | 0.000137994 |
| CD86 | 1024 | 103.0209408 | | 217.1883138 | 1.076008858 | 0.019452105 | Up | 0.000282762 |
| SIRPB2 | 2642 | 58.34042896 | | 122.8815621 | 1.074700562 | 0.025970302 | Up | 0.000420925 |
| CTSH | 1153 | 1070.575609 | | 2248.889679 | 1.070826202 | 0.000926083 | Up | 3.99E-06 |
| SIRPB1 | 779 | 70.86178459 | | 148.2850818 | 1.065293759 | 0.040219914 | Up | 0.000820107 |
| ITGAX | 3861 | 1133.731683 | | 2370.824995 | 1.06430993 | 0.014648438 | Up | 0.000188016 |
| GREB1L | 8749 | 79.12737194 | | 165.4377492 | 1.064039716 | 0.017024091 | Up | 0.000233928 |
| RASSF4 | 2796 | 891.8662619 | | 1862.479013 | 1.062324874 | 0.005899489 | Up | 5.30E-05 |
| FPR3 | 2454 | 282.1213169 | | 589.0107159 | 1.061978202 | 0.030626961 | Up | 0.00053722 |
| ISG20 | 1113 | 920.4057923 | | 1917.545429 | 1.05891879 | 0.007615368 | Up | 7.67E-05 |
| ZNF101 | 3486 | 393.1210316 | | 818.4924237 | 1.057995514 | 0.004841472 | Up | 4.05E-05 |
| SYNE3 | 3331 | 343.4771752 | | 714.2794794 | 1.056274443 | 0.009242093 | Up | 0.000101066 |
| RP11-35G9.3 | 1557 | 73.59367569 | | 153.0252623 | 1.056116143 | 0.025371185 | Up | 0.000405235 |
| DDX26B | 4703 | 367.6829073 | | 758.8916095 | 1.045431734 | 0.001554482 | Up | 7.97E-06 |
| VAMP1 | 3449 | 614.987479 | | 1264.688222 | 1.040152825 | 0.015631737 | Up | 0.000205989 |
| LMO2 | 1629 | 164.6411399 | | 338.3262971 | 1.039090442 | 0.030775261 | Up | 0.000541386 |
| ZNF420 | 1049 | 68.49668169 | | 140.6642788 | 1.038150004 | 0.032242641 | Up | 0.000581231 |
| CD300A | 1620 | 120.8338084 | | 246.7794499 | 1.030198095 | 0.04909159 | Up | 0.001121387 |
| RP11-705C15.3 | 2508 | 420.0378877 | | 857.6221235 | 1.029822657 | 0.033038169 | Up | 0.000599975 |
| CEP85L | 2786 | 296.7461347 | | 605.2066818 | 1.028198678 | 0.000784934 | Up | 3.10E-06 |
| OXNAD1 | 3153 | 335.1258408 | | 683.1689579 | 1.027539489 | 0.001559578 | Up | 8.10E-06 |
| PARP8 | 2022 | 462.2513015 | | 935.7750772 | 1.017484424 | 0.006286354 | Up | 5.96E-05 |
| CSF2RA | 1067 | 111.5138221 | | 225.6367432 | 1.016779476 | 0.009788988 | Up | 0.000111449 |
| CCNG2 | 2442 | 737.9400632 | | 1491.375993 | 1.015068475 | 0.009242093 | Up | 0.00010052 |
| SFMBT2 | 6074 | 468.5060169 | | 945.2362972 | 1.012607454 | 0.000394593 | Up | 1.39E-06 |
| SMAP2 | 2939 | 1263.947424 | | 2548.923589 | 1.011951672 | 0.025639408 | Up | 0.000414617 |
| RNPC3 | 3467 | 276.1295072 | | 556.1529636 | 1.010136672 | 0.002747128 | Up | 1.88E-05 |
| KIAA1551 | 2951 | 1041.858766 | | 2096.030341 | 1.008499881 | 0.006439807 | Up | 6.17E-05 |
| ABI3 | 1712 | 199.8715963 | | 401.0870065 | 1.004841764 | 0.032905686 | Up | 0.000596072 |
| GAS2L1 | 1623 | 1904.646076 | | 952.0416294 | -1.000426375 | 0.044712062 | Down | 0.000965044 |
| NREP | 2013 | 1036.352986 | | 517.416715 | -1.002116909 | 0.030458076 | Down | 0.000530969 |
| SH3RF3 | 2717 | 836.9811864 | | 407.5019863 | -1.038388103 | 0.001845355 | Down | 1.03E-05 |
| MFAP2 | 1521 | 1461.620064 | | 707.0474634 | -1.047689373 | 0.044186831 | Down | 0.000947869 |
| EIF4EBP1 | 877 | 1203.903834 | | 578.5659597 | -1.057166807 | 0.041646149 | Down | 0.000865206 |
| RP11-127L20.3 | 954 | 105.3482527 | | 50.13255406 | -1.071346745 | 0.045922632 | Down | 0.001015521 |
| SH3RF3-AS1 | 1604 | 290.3005956 | | 137.8836731 | -1.074095895 | 0.026756582 | Down | 0.000441911 |
| TUBB2A | 1453 | 1216.201685 | | 566.284329 | -1.102783982 | 0.030866111 | Down | 0.000544143 |
| COL7A1 | 8069 | 35180.68675 | | 16309.70936 | -1.109052572 | 0.00902895 | Down | 9.73E-05 |
| MN1 | 7556 | 2744.889316 | | 1267.723356 | -1.114508023 | 0.032219591 | Down | 0.000580104 |
| MFSD2A | 1278 | 660.249863 | | 303.7638098 | -1.120062099 | 0.047972103 | Down | 0.001085217 |
| ANGPTL2 | 1940 | 1428.586959 | | 657.159614 | -1.12027313 | 0.016247629 | Down | 0.000220028 |
| MXRA5 | 9793 | 8358.518546 | | 3763.472941 | -1.151182665 | 0.045428461 | Down | 0.000993233 |
| PCDHB12 | 3406 | 137.6523916 | | 61.55477872 | -1.161086908 | 0.033521058 | Down | 0.000612763 |
| COL6A2 | 3203 | 22541.24683 | | 9941.988986 | -1.180960908 | 0.033207964 | Down | 0.000604501 |
| SMTN | 2938 | 3025.05094 | | 1330.170307 | -1.185348464 | 0.03363283 | Down | 0.000618923 |
| LDLR | 4191 | 3853.327568 | | 1652.719693 | -1.221262774 | 0.004681922 | Down | 3.86E-05 |
| FLNC | 9042 | 1447.858131 | | 614.8793488 | -1.235544987 | 0.010650039 | Down | 0.000126385 |
| SGCD | 4672 | 515.5678677 | | 217.469148 | -1.245351609 | 0.021267548 | Down | 0.000319488 |
| SORBS1 | 4731 | 1046.874858 | | 436.2516853 | -1.262856385 | 0.000761071 | Down | 2.98E-06 |
| TMEM158 | 1813 | 645.9969698 | | 267.7938079 | -1.270404795 | 0.046726262 | Down | 0.001039485 |
| LMOD1 | 3970 | 698.7746509 | | 287.4417032 | -1.281557884 | 0.009017287 | Down | 9.70E-05 |
| HECW1 | 5169 | 282.7872759 | | 115.7530925 | -1.28866647 | 0.027477376 | Down | 0.000460121 |
| BMP4 | 1917 | 262.4323359 | | 106.8541217 | -1.296302936 | 0.003575 | Down | 2.66E-05 |
| WNT5A | 5890 | 4487.231626 | | 1826.532793 | -1.296718001 | 0.006003802 | Down | 5.41E-05 |
| MRGPRF | 2282 | 470.4504235 | | 190.5489588 | -1.303880973 | 0.001197388 | Down | 5.58E-06 |
| LRP4 | 8076 | 1358.6874 | | 550.1315492 | -1.304365019 | 0.046387399 | Down | 0.001027848 |
| PCDHB11 | 1973 | 177.5149627 | | 71.51461547 | -1.311630613 | 0.024809937 | Down | 0.000392193 |
| HTRA3 | 2530 | 2548.967131 | | 1018.313869 | -1.323730466 | 0.003725804 | Down | 2.83E-05 |
| HSPB7 | 1541 | 127.3945269 | | 50.8178445 | -1.32589621 | 0.041425354 | Down | 0.000855151 |
| FOXF1 | 2568 | 384.0482289 | | 151.6694626 | -1.340356856 | 0.000911934 | Down | 3.91E-06 |
| CSPG4 | 8290 | 3704.291393 | | 1453.157677 | -1.350006338 | 0.027952445 | Down | 0.00047023 |
| TCF21 | 3010 | 145.3048146 | | 56.52582983 | -1.362100335 | 0.004291248 | Down | 3.41E-05 |
| RP11-424C20.2 | 1423 | 262.7874965 | | 102.060995 | -1.364465021 | 0.002144266 | Down | 1.31E-05 |
| MIR143HG | 7794 | 2762.775059 | | 1062.053975 | -1.379261017 | 0.00238399 | Down | 1.53E-05 |
| SPON2 | 1210 | 3165.714502 | | 1214.306287 | -1.382398791 | 0.000101507 | Down | 1.93E-07 |
| MYH11 | 2764 | 5287.482896 | | 2023.465351 | -1.385752946 | 0.024764376 | Down | 0.000390072 |
| ADAMTS14 | 5260 | 1373.08603 | | 523.8495398 | -1.390197614 | 0.036198713 | Down | 0.00069413 |
| COL6A1 | 3102 | 24711.6746 | | 9410.54637 | -1.392842387 | 0.013020405 | Down | 0.000163668 |
| PFKFB4 | 1083 | 1626.364971 | | 618.0378622 | -1.395883919 | 0.001323437 | Down | 6.34E-06 |
| PPAPDC3 | 1864 | 84.91076119 | | 31.90006346 | -1.412388112 | 0.01491755 | Down | 0.000194218 |
| UCN2 | 1481 | 844.7818676 | | 316.368716 | -1.416971923 | 0.018214422 | Down | 0.00025793 |
| COL12A1 | 7845 | 19884.6443 | | 7388.35643 | -1.428329384 | 0.016198491 | Down | 0.000218664 |
| MSX2 | 2241 | 116.9240154 | | 42.57680765 | -1.457431592 | 0.041506292 | Down | 0.000860091 |
| SLC24A2 | 10749 | 144.5108089 | | 51.92829431 | -1.476584662 | 0.027080009 | Down | 0.000452869 |
| NACAD | 4780 | 180.8693445 | | 63.44459997 | -1.511378626 | 0.00109658 | Down | 4.94E-06 |
| IGDCC4 | 4261 | 567.1813842 | | 197.7403166 | -1.520203135 | 0.000730922 | Down | 2.83E-06 |
| IL1B | 1055 | 317.4039254 | | 110.1823178 | -1.526427254 | 0.044983443 | Down | 0.000973883 |
| NKD2 | 1709 | 1357.171497 | | 467.3477243 | -1.538034763 | 0.008314057 | Down | 8.72E-05 |
| CDH6 | 4037 | 319.3056715 | | 109.8185631 | -1.539816238 | 0.0019047 | Down | 1.08E-05 |
| TWIST2 | 1176 | 93.65962015 | | 31.87197637 | -1.555138704 | 0.006240515 | Down | 5.90E-05 |
| AC131025.8 | 5131 | 1490.091121 | | 505.7389576 | -1.558935736 | 0.000619428 | Down | 2.30E-06 |
| PCSK9 | 3529 | 881.9902158 | | 299.2297508 | -1.55950903 | 0.031727254 | Down | 0.000565714 |
| ADAM33 | 3452 | 773.8608915 | | 257.7551448 | -1.586073028 | 0.017995783 | Down | 0.00025293 |
| RP11-681L8.1 | 500 | 24.76684544 | | 8.216986128 | -1.591728888 | 0.035035573 | Down | 0.000656283 |
| WNT2 | 1768 | 247.3352267 | | 81.93432997 | -1.593927766 | 0.040671243 | Down | 0.000832138 |
| CACNA1H | 6305 | 944.9419451 | | 312.167769 | -1.597904108 | 7.29E-05 | Down | 1.18E-07 |
| RN7SL67P | 297 | 42.57635794 | | 14.04226066 | -1.600277331 | 0.029815135 | Down | 0.000516157 |
| PAX9 | 3437 | 1369.141792 | | 450.6075814 | -1.603328372 | 0.029677109 | Down | 0.000512036 |
| MIR145 | 2549 | 717.1344169 | | 235.7510863 | -1.604979139 | 0.000391291 | Down | 1.36E-06 |
| FOXF2 | 2187 | 351.9775773 | | 114.3046824 | -1.622599022 | 2.21E-08 | Down | 9.78E-13 |
| RP3-342P20.2 | 725 | 107.8062941 | | 34.82947276 | -1.630060871 | 0.044145394 | Down | 0.000945421 |
| DIO2 | 3818 | 1268.053787 | | 403.2693228 | -1.652800375 | 0.000217439 | Down | 5.52E-07 |
| CNN1 | 1372 | 447.9787805 | | 142.3463907 | -1.654024485 | 0.035733419 | Down | 0.00067881 |
| NPW | 857 | 136.1393104 | | 42.61998626 | -1.675481673 | 0.043918445 | Down | 0.000937242 |
| APLN | 3220 | 192.6806654 | | 59.93334248 | -1.684781071 | 0.035291927 | Down | 0.000663488 |
| RTL1 | 4193 | 88.6117652 | | 27.49245909 | -1.688462306 | 0.045342496 | Down | 0.00098967 |
| MMP11 | 1645 | 10977.43283 | | 3388.377323 | -1.695874263 | 0.017245886 | Down | 0.000237738 |
| SLC2A4 | 1768 | 95.73172877 | | 28.91713507 | -1.727072537 | 0.018816396 | Down | 0.000270195 |
| TPTEP1 | 1195 | 201.0663516 | | 60.53981812 | -1.731715421 | 0.005364333 | Down | 4.58E-05 |
| IGF2 | 4884 | 5537.47782 | | 1571.176715 | -1.817383561 | 0.002244641 | Down | 1.40E-05 |
| ROBO2 | 4677 | 267.153546 | | 74.59506519 | -1.840517069 | 0.009691166 | Down | 0.000107923 |
| LRRN4CL | 2585 | 152.1511976 | | 42.31194692 | -1.846368714 | 0.0006813 | Down | 2.57E-06 |
| ADH5P4 | 1104 | 9.262802507 | | 2.499346924 | -1.889897584 | 0.009788988 | Down | 0.000111729 |
| RP11-598F7.5 | 1101 | 20.7175969 | | 5.560349301 | -1.897609249 | 0.03782459 | Down | 0.000747033 |
| MMP16 | 3142 | 280.6045754 | | 74.965002 | -1.904249409 | 0.00729183 | Down | 7.25E-05 |
| MASP1 | 3900 | 65.24758993 | | 17.32031255 | -1.913459649 | 0.011030797 | Down | 0.000132079 |
| LINC00578 | 533 | 31.60461527 | | 8.380001645 | -1.91511282 | 0.009784188 | Down | 0.000110487 |
| TGM1 | 1387 | 1832.211484 | | 474.3238974 | -1.949641576 | 0.00622511 | Down | 5.84E-05 |
| RP11-262D11.2 | 2269 | 19.61561932 | | 5.048106348 | -1.958188677 | 0.04553703 | Down | 0.00099794 |
| LAMC3 | 2029 | 376.2935587 | | 96.68468843 | -1.960499255 | 0.000880986 | Down | 3.70E-06 |
| GREM1 | 2747 | 5090.66275 | | 1304.186668 | -1.964703115 | 4.74E-05 | Down | 5.97E-08 |
| CTD-3064M3.3 | 1963 | 46.45967248 | | 11.68758978 | -1.991001536 | 0.000371125 | Down | 1.23E-06 |
| PCA3 | 3922 | 40.73103324 | | 9.937935333 | -2.035110353 | 0.023744726 | Down | 0.000367961 |
| RGMA | 3006 | 3015.107428 | | 734.2825264 | -2.037802232 | 0.001559578 | Down | 8.13E-06 |
| WFDC1 | 1971 | 115.9002775 | | 27.08164037 | -2.097496988 | 0.000478557 | Down | 1.73E-06 |
| RNU1-1 | 164 | 4355.569095 | | 1001.299893 | -2.120987101 | 0.035733419 | Down | 0.000678892 |
| GREM2 | 4170 | 87.93720695 | | 19.63839419 | -2.162796744 | 0.020181375 | Down | 0.000296929 |
| KRT16P1 | 810 | 152.4028172 | | 33.02881053 | -2.206092652 | 0.012725414 | Down | 0.000158836 |
| RP11-108F13.2 | 906 | 6.125354323 | | 1.327457961 | -2.206127126 | 0.03436104 | Down | 0.000636119 |
| CCL11 | 1079 | 45.78910469 | | 9.659329863 | -2.245009349 | 0.002058961 | Down | 1.24E-05 |
| JPH2 | 4787 | 562.7610721 | | 114.7452705 | -2.294087846 | 8.80E-06 | Down | 3.30E-09 |
| FENDRR | 2865 | 222.7577413 | | 42.48548709 | -2.390433559 | 2.78E-06 | Down | 6.13E-10 |
| SCUBE1 | 9808 | 120.4787194 | | 22.72631335 | -2.406342765 | 0.003118562 | Down | 2.25E-05 |
| RP11-368J22.2 | 1021 | 95.94411835 | | 18.0793965 | -2.407847847 | 0.047688386 | Down | 0.001075638 |
| C10orf111 | 1714 | 11.90376038 | | 2.222782057 | -2.420978986 | 0.016198491 | Down | 0.000218736 |
| ACAN | 5667 | 230.6542353 | | 42.00905976 | -2.456959382 | 2.41E-05 | Down | 1.92E-08 |
| NKX6-1 | 2566 | 138.142834 | | 25.14670447 | -2.457719476 | 7.08E-06 | Down | 2.50E-09 |
| RP11-616M22.2 | 476 | 10.73246637 | | 1.943919549 | -2.464941234 | 0.038075283 | Down | 0.000755195 |
| MYOCD | 8466 | 72.09891319 | | 12.65315923 | -2.510479872 | 0.009242093 | Down | 0.000100701 |
| LHX8 | 2244 | 8.705679631 | | 1.524820596 | -2.513317419 | 0.039766181 | Down | 0.000800314 |
| OR7E47P | 1296 | 43.46105099 | | 7.185665698 | -2.59652934 | 0.00018533 | Down | 4.42E-07 |
| FAM178B | 1209 | 168.0243929 | | 26.07134769 | -2.688133625 | 0.035480696 | Down | 0.00066956 |
| ALOX12B | 1608 | 343.501244 | | 51.70770337 | -2.731864191 | 0.043394483 | Down | 0.000923185 |
| RP4-736L20.3 | 797 | 7.742928405 | | 1.116094406 | -2.794420238 | 0.039106709 | Down | 0.000782115 |
| RP11-758N13.1 | 1738 | 408.7021087 | | 53.97009428 | -2.920817574 | 5.79E-06 | Down | 1.79E-09 |
| FGF5 | 5234 | 23.26342148 | | 2.974880702 | -2.967159577 | 0.00243499 | Down | 1.59E-05 |
| SOX11 | 8718 | 139.8119346 | | 16.49577131 | -3.083319375 | 5.37E-06 | Down | 1.54E-09 |
| KLF14 | 1488 | 12.72617342 | | 1.488714372 | -3.0956598 | 0.03421354 | Down | 0.000631876 |
| AL162151.3 | 158 | 41.45750586 | | 4.835307104 | -3.099953897 | 0.042538775 | Down | 0.000898402 |
| RN7SKP106 | 272 | 5.194524937 | | 0.575210016 | -3.174831111 | 0.041536935 | Down | 0.000861644 |
| HPSE2 | 2121 | 18.4335782 | | 1.996054851 | -3.207112872 | 0.023132054 | Down | 0.000355582 |
| RP11-69H7.2 | 720 | 12.33873392 | | 1.335847032 | -3.207367648 | 0.023744726 | Down | 0.000367843 |
| DES | 1192 | 1443.947415 | | 151.5671737 | -3.25198897 | 2.51E-05 | Down | 2.07E-08 |
| TGM3 | 2642 | 1301.463088 | | 134.8200434 | -3.271027494 | 0.031224733 | Down | 0.000553914 |
| IGF2-AS | 1867 | 20.2127778 | | 1.754616686 | -3.526039804 | 0.022923756 | Down | 0.000349092 |
| ACTC1 | 4106 | 41.72891808 | | 3.582634362 | -3.541954799 | 0.002144266 | Down | 1.32E-05 |
| RP11-252O2.2 | 318 | 3.318265823 | | 0.272624405 | -3.605442844 | 0.03011375 | Down | 0.000522896 |
| RP11-690I21.3 | 497 | 5.990755227 | | 0.41557225 | -3.849566662 | 0.006004054 | Down | 5.44E-05 |
| DMBT1 | 3826 | 48.81935995 | | 3.309674436 | -3.882692166 | 0.017995783 | Down | 0.000252763 |
| PCP4 | 587 | 4.02134232 | | 0.214521394 | -4.228483715 | 0.043938835 | Down | 0.000938648 |
| CTD-2297D10.1 | 454 | 4.69023623 | | 0.21523848 | -4.445652657 | 0.044145394 | Down | 0.000945987 |
| RP11-435I10.5 | 634 | 9.927375046 | | 0.372606261 | -4.735688474 | 0.008978614 | Down | 9.64E-05 |
| AC005358.1 | 2055 | 11.77209625 | | 0.368650136 | -4.996975142 | 0.004486873 | Down | 3.63E-05 |
| AL109763.2 | 615 | 8.490452943 | | 0.246171268 | -5.108107226 | 0.033521058 | Down | 0.000616062 |
| WI2-2998D17.2 | 886 | 11.42751641 | | 0.247202699 | -5.530673584 | 0.039894803 | Down | 0.000805843 |
| **Differential RNA expression between LN_met_ and PT_deep_** | | | | | | | | |
| **Gene ID** | **Length** | **LN_met_-Expression** | | **PT_deep_-Expression** | **log2 FoldChange(PT_deep_/LN_met_)** | **Padj** | **Up/Down-Regulation** | **P-value** |
| DES | 1630 | 147.7467149 | | 13705.57655 | 6.535493155 | 1.76E-21 | Up | 3.89E-26 |
| PCP4 | 660 | 0.210736088 | | 10.34889306 | 5.617895261 | 2.86E-06 | Up | 1.71E-09 |
| AC005358.1 | 2055 | 0.356427757 | | 13.95769584 | 5.291307296 | 0.005494957 | Up | 1.54E-05 |
| HPSE2 | 2169 | 1.952603751 | | 48.91209007 | 4.646720001 | 1.55E-05 | Up | 1.07E-08 |
| MYOCD | 5771 | 12.30118807 | | 242.7788206 | 4.302773005 | 8.88E-12 | Up | 6.32E-16 |
| LRRTM3 | 1942 | 0.429412728 | | 8.071280802 | 4.23236077 | 0.004172309 | Up | 1.09E-05 |
| ACTC1 | 1567 | 3.483375197 | | 61.24606539 | 4.136059384 | 1.57E-05 | Up | 1.11E-08 |
| FTH1P14 | 551 | 0.266667919 | | 4.006948979 | 3.909387958 | 0.0492388 | Up | 0.000447675 |
| SUPT20HL1 | 2472 | 0.425846777 | | 6.18743654 | 3.860935489 | 0.013191395 | Up | 5.49E-05 |
| NOBOX | 1980 | 0.82042095 | | 10.93540423 | 3.736498407 | 0.029787846 | Up | 0.000196602 |
| KLF14 | 1488 | 1.458784947 | | 19.04692302 | 3.70671883 | 0.00084837 | Up | 1.22E-06 |
| RP5-1198O20.5 | 520 | 0.399625231 | | 5.083171923 | 3.66900945 | 0.008007816 | Up | 2.70E-05 |
| RP5-1198O20.4 | 1707 | 5.207449425 | | 65.52827644 | 3.653468758 | 0.001099657 | Up | 1.70E-06 |
| RP11-1090M7.1 | 3679 | 0.961568002 | | 11.79228367 | 3.616310435 | 0.003976381 | Up | 1.01E-05 |
| ACTG2 | 1370 | 288.8195506 | | 3526.244749 | 3.609892304 | 2.10E-07 | Up | 1.02E-10 |
| DGKB | 3073 | 4.825783884 | | 53.80878718 | 3.479006576 | 0.002529393 | Up | 5.26E-06 |
| Z99756.1 | 1951 | 0.626603486 | | 6.618385058 | 3.400854529 | 0.006842438 | Up | 2.18E-05 |
| SYNM | 7097 | 544.4599389 | | 5730.679051 | 3.395808295 | 1.33E-07 | Up | 6.19E-11 |
| JPH2 | 4329 | 110.1230193 | | 1105.27641 | 3.327219231 | 3.34E-08 | Up | 1.11E-11 |
| CNN1 | 1458 | 136.0165871 | | 1361.903445 | 3.323769921 | 4.90E-07 | Up | 2.60E-10 |
| MYH11 | 5903 | 1944.398829 | | 19048.05807 | 3.292247849 | 1.95E-08 | Up | 5.17E-12 |
| SLC2A4 | 3055 | 27.78548774 | | 271.9926835 | 3.291164374 | 0.000752182 | Up | 1.02E-06 |
| C20orf166-AS1 | 2313 | 4.913431263 | | 47.51295596 | 3.273518187 | 0.003421292 | Up | 8.17E-06 |
| SCUBE1 | 5119 | 21.86607769 | | 198.9595405 | 3.185708721 | 2.81E-08 | Up | 8.70E-12 |
| PCA3 | 3922 | 9.524285346 | | 86.04354079 | 3.175384145 | 4.15E-05 | Up | 3.03E-08 |
| RN7SL815P | 291 | 1.041839738 | | 9.265230666 | 3.152693523 | 0.049744322 | Up | 0.000458874 |
| FGF5 | 1793 | 2.893411042 | | 25.33384599 | 3.130222918 | 0.000506476 | Up | 6.27E-07 |
| CASQ2 | 2674 | 6.719921088 | | 58.5872901 | 3.124071524 | 0.005190693 | Up | 1.41E-05 |
| NKX6-1 | 2527 | 23.93397393 | | 204.1925444 | 3.092798327 | 2.15E-07 | Up | 1.09E-10 |
| RP11-616M22.2 | 476 | 1.861414889 | | 14.96435552 | 3.007058589 | 0.010509553 | Up | 3.88E-05 |
| SLC27A6 | 2545 | 7.098802683 | | 56.98390927 | 3.00490698 | 0.008357012 | Up | 2.96E-05 |
| KHSRPP1 | 1768 | 1.9210087 | | 15.23566266 | 2.987516293 | 0.026571084 | Up | 0.000165169 |
| HSPB7 | 2184 | 48.8673905 | | 387.4349376 | 2.987010085 | 2.11E-08 | Up | 6.06E-12 |
| GPM6A | 1446 | 12.17087755 | | 91.84259861 | 2.915730269 | 0.000218885 | Up | 2.23E-07 |
| SGCA | 1253 | 12.35015774 | | 91.53085396 | 2.889728672 | 0.000165016 | Up | 1.57E-07 |
| LMOD1 | 3970 | 274.8545063 | | 1985.983841 | 2.853113847 | 1.19E-07 | Up | 5.28E-11 |
| FLNC | 9100 | 588.3436461 | | 4247.680029 | 2.851944123 | 5.32E-08 | Up | 2.24E-11 |
| PRUNE2 | 7232 | 246.5453403 | | 1777.076904 | 2.849581225 | 1.55E-08 | Up | 3.43E-12 |
| HAND2 | 2127 | 30.16489717 | | 205.414912 | 2.767598348 | 0.001984505 | Up | 3.91E-06 |
| KRT8P47 | 1398 | 9.401496381 | | 63.81505089 | 2.762934421 | 0.014116959 | Up | 6.21E-05 |
| C10orf111 | 1714 | 2.148738431 | | 14.46287591 | 2.75079268 | 0.002898752 | Up | 6.54E-06 |
| SORBS1 | 4096 | 417.8634066 | | 2807.785861 | 2.748329582 | 4.23E-11 | Up | 4.67E-15 |
| ELMOD1 | 1239 | 4.889561704 | | 32.54077788 | 2.734471685 | 0.024987636 | Up | 0.000148306 |
| KCNB1 | 11850 | 22.33942133 | | 144.6164707 | 2.694568153 | 0.014709057 | Up | 6.61E-05 |
| NRXN1 | 3930 | 21.03435339 | | 135.7689168 | 2.690333849 | 0.000191152 | Up | 1.86E-07 |
| NRK | 8062 | 24.87982199 | | 159.1147637 | 2.677019635 | 0.003397035 | Up | 8.04E-06 |
| TYRP1 | 1685 | 5.489985134 | | 35.05179099 | 2.674614014 | 0.01532008 | Up | 7.05E-05 |
| FENDRR | 2376 | 40.63169035 | | 259.2181537 | 2.673489468 | 0.000633098 | Up | 7.98E-07 |
| AC131025.8 | 5131 | 482.1102512 | | 2851.637611 | 2.564355643 | 1.57E-09 | Up | 2.44E-13 |
| CHRDL2 | 1754 | 37.23025034 | | 219.0099775 | 2.556449374 | 0.000154892 | Up | 1.40E-07 |
| LINC00578 | 563 | 7.994051956 | | 45.96795631 | 2.523629672 | 0.001134829 | Up | 1.81E-06 |
| FAM178B | 1001 | 13.77910233 | | 78.98429074 | 2.51908384 | 0.047407198 | Up | 0.000422632 |
| CACNA1H | 6656 | 298.1502745 | | 1592.632792 | 2.417302098 | 4.04E-09 | Up | 8.05E-13 |
| MYLK-AS2 | 754 | 2.668920825 | | 14.18116734 | 2.409647882 | 0.003492636 | Up | 8.50E-06 |
| RP11-85M11.2 | 906 | 5.206800691 | | 27.27235478 | 2.388970184 | 0.011113737 | Up | 4.33E-05 |
| MIR145 | 2549 | 224.5279638 | | 1171.328712 | 2.383178957 | 3.94E-08 | Up | 1.39E-11 |
| KDELC1P1 | 1509 | 3.839493324 | | 19.75163357 | 2.362984132 | 0.0077022 | Up | 2.50E-05 |
| CYP21A1P | 1971 | 5.96326599 | | 30.38284746 | 2.34908249 | 0.023712732 | Up | 0.000133757 |
| RGMB-AS1 | 1620 | 7.970476037 | | 40.18480835 | 2.333912405 | 0.021155941 | Up | 0.000113415 |
| SOX11 | 8718 | 15.73643331 | | 78.65464555 | 2.321423389 | 0.001678929 | Up | 3.01E-06 |
| LDB3 | 3900 | 25.63143914 | | 125.9442383 | 2.296798733 | 0.039496548 | Up | 0.000312201 |
| ATP1A2 | 4821 | 35.67790507 | | 175.0496593 | 2.294661443 | 0.040350791 | Up | 0.000327591 |
| MIR143HG | 5788 | 1011.838934 | | 4836.186691 | 2.25689028 | 1.61E-08 | Up | 3.93E-12 |
| WISP2 | 1612 | 95.57194057 | | 448.4571691 | 2.230311186 | 0.046781509 | Up | 0.000415338 |
| THBS4 | 2846 | 279.4353088 | | 1295.598582 | 2.213032561 | 0.009780089 | Up | 3.53E-05 |
| SLC35F1 | 4852 | 8.940160968 | | 38.73382912 | 2.115221417 | 0.006192125 | Up | 1.86E-05 |
| CTD-3064M3.3 | 1963 | 11.03599604 | | 46.51663453 | 2.075529877 | 0.000752182 | Up | 1.01E-06 |
| RP11-758N13.1 | 1325 | 51.29906421 | | 215.8224774 | 2.072840712 | 0.001499889 | Up | 2.65E-06 |
| RP11-122A3.2 | 894 | 10.53750979 | | 44.25037935 | 2.070155851 | 0.008007816 | Up | 2.70E-05 |
| LHCGR | 2180 | 8.652344672 | | 35.59715265 | 2.040598805 | 0.038643838 | Up | 0.000299671 |
| SCN7A | 4815 | 73.8643414 | | 300.6124906 | 2.02495499 | 0.01846592 | Up | 9.68E-05 |
| CCL11 | 1079 | 9.119500214 | | 36.34688515 | 1.994805067 | 0.027834255 | Up | 0.000176716 |
| SYNPO2 | 6739 | 1062.151245 | | 4156.270313 | 1.968300272 | 0.000826918 | Up | 1.17E-06 |
| GREM1 | 3387 | 1238.995301 | | 4759.887419 | 1.941756735 | 8.01E-07 | Up | 4.43E-10 |
| KCNK3 | 6162 | 15.64305603 | | 59.97505083 | 1.93884009 | 0.026265146 | Up | 0.00016236 |
| AOC3 | 3788 | 474.0846987 | | 1715.174412 | 1.855138553 | 2.23E-06 | Up | 1.28E-09 |
| DACT3 | 2646 | 149.5355781 | | 539.214806 | 1.850371334 | 0.000198967 | Up | 1.98E-07 |
| SOD3 | 1526 | 301.4846461 | | 1085.988893 | 1.848852912 | 0.002529393 | Up | 5.25E-06 |
| LINC00702 | 4931 | 34.08247252 | | 122.0353779 | 1.840197538 | 0.01532008 | Up | 7.02E-05 |
| MMP16 | 9653 | 71.05482144 | | 252.8922575 | 1.831518416 | 0.001384319 | Up | 2.33E-06 |
| MRGPRF | 2200 | 181.2441772 | | 631.0319402 | 1.799778384 | 0.006301047 | Up | 1.95E-05 |
| LRRN4CL | 2585 | 40.18731588 | | 139.7284462 | 1.79781363 | 0.015194736 | Up | 6.92E-05 |
| RGMA | 2496 | 695.3048897 | | 2393.671024 | 1.783507247 | 0.003397035 | Up | 8.03E-06 |
| CSDC2 | 2340 | 66.66894432 | | 224.3893762 | 1.750917585 | 0.010509553 | Up | 3.97E-05 |
| RSPO3 | 2431 | 47.65241613 | | 158.8426645 | 1.736977196 | 0.016642475 | Up | 8.10E-05 |
| TCF21 | 4140 | 53.38478202 | | 174.6645691 | 1.710086538 | 0.005536307 | Up | 1.58E-05 |
| F2RL2 | 3115 | 94.69969622 | | 303.2385982 | 1.679021698 | 0.000286393 | Up | 3.04E-07 |
| TBX5-AS1 | 3017 | 26.84582791 | | 84.99027825 | 1.662599928 | 0.022406236 | Up | 0.000124203 |
| DAAM2 | 4193 | 401.7262872 | | 1260.685464 | 1.6499236 | 0.000319537 | Up | 3.60E-07 |
| FHL1 | 2023 | 410.6921045 | | 1286.7635 | 1.647617801 | 0.003576765 | Up | 8.78E-06 |
| LYPD5 | 1785 | 76.46375585 | | 236.1966921 | 1.627140789 | 0.040406951 | Up | 0.000331785 |
| ADAM33 | 3243 | 244.1000338 | | 738.0759554 | 1.596296797 | 0.026265146 | Up | 0.000162418 |
| GJA5 | 2986 | 101.7851356 | | 301.3572727 | 1.565947993 | 0.030649646 | Up | 0.000206337 |
| LGI4 | 1841 | 91.95584915 | | 270.8352413 | 1.558402226 | 0.037830763 | Up | 0.000287047 |
| ADCY5 | 4642 | 265.2278332 | | 775.5135926 | 1.547919887 | 0.038643838 | Up | 0.000300055 |
| HTRA3 | 2508 | 962.7516377 | | 2793.336012 | 1.536753549 | 0.000112962 | Up | 9.50E-08 |
| CSPG4 | 8290 | 1372.69598 | | 3936.397149 | 1.519863645 | 0.006177514 | Up | 1.83E-05 |
| MYL9 | 1196 | 1909.195857 | | 5453.996839 | 1.514348753 | 0.014089702 | Up | 6.17E-05 |
| SMTN | 2347 | 1251.067341 | | 3568.774454 | 1.512269279 | 0.002127948 | Up | 4.24E-06 |
| FOXF2 | 2187 | 108.1103491 | | 308.3876572 | 1.51224039 | 0.000152093 | Up | 1.35E-07 |
| SPARCL1 | 2843 | 816.5687311 | | 2306.858461 | 1.498283261 | 0.001154453 | Up | 1.89E-06 |
| PDLIM3 | 1862 | 600.6281457 | | 1687.033695 | 1.489944802 | 0.000493773 | Up | 6.01E-07 |
| MSRB3 | 3787 | 451.5776719 | | 1253.941812 | 1.473424343 | 0.000103028 | Up | 7.98E-08 |
| PPAPDC3 | 1650 | 30.10226863 | | 82.46700429 | 1.453944782 | 0.008007816 | Up | 2.67E-05 |
| MRVI1 | 4346 | 521.9265397 | | 1428.395526 | 1.452476851 | 0.000109144 | Up | 8.93E-08 |
| KANK2 | 2344 | 1214.86455 | | 3298.028266 | 1.440808293 | 0.000152093 | Up | 1.32E-07 |
| TPM2 | 1353 | 777.4937863 | | 2100.125638 | 1.433572588 | 0.011824746 | Up | 4.70E-05 |
| RP11-114F10.3 | 753 | 16.86920657 | | 45.4896462 | 1.431146095 | 0.043762779 | Up | 0.000372661 |
| TAGLN | 1141 | 3709.885126 | | 9946.961644 | 1.422881398 | 0.029787846 | Up | 0.000197685 |
| TNS1 | 5302 | 5499.001755 | | 14656.01802 | 1.41425153 | 0.000430022 | Up | 5.14E-07 |
| ALDH1B1 | 3066 | 467.3802509 | | 1244.015041 | 1.412335249 | 0.003986375 | Up | 1.02E-05 |
| MYLK | 2483 | 1849.970182 | | 4851.770579 | 1.391009315 | 0.002744295 | Up | 6.01E-06 |
| SGCD | 7600 | 205.254469 | | 536.495627 | 1.386152777 | 0.005485427 | Up | 1.52E-05 |
| CLMP | 2125 | 266.8203004 | | 691.0069839 | 1.372831857 | 0.008007816 | Up | 2.75E-05 |
| GPC6 | 6467 | 363.0171501 | | 929.6706168 | 1.356681951 | 0.023712732 | Up | 0.000135133 |
| MXRA5 | 9793 | 3538.31814 | | 8892.037225 | 1.329450217 | 0.017395339 | Up | 8.70E-05 |
| BOC | 2826 | 634.3225032 | | 1593.101973 | 1.328550187 | 0.004122079 | Up | 1.07E-05 |
| S1PR3 | 4688 | 402.3027264 | | 999.32888 | 1.312678033 | 0.000360517 | Up | 4.15E-07 |
| COL15A1 | 4884 | 1077.628456 | | 2669.226018 | 1.308561618 | 0.011824746 | Up | 4.66E-05 |
| PCDHB11 | 3194 | 67.62467551 | | 166.5588574 | 1.300410405 | 0.024341338 | Up | 0.000141616 |
| NACAD | 1883 | 59.90056771 | | 146.4788031 | 1.290050327 | 0.03691641 | Up | 0.000273448 |
| FSTL3 | 2270 | 673.6811266 | | 1642.848796 | 1.286061917 | 0.018828806 | Up | 9.95E-05 |
| CACNB2 | 1930 | 70.27922324 | | 171.1166668 | 1.283810134 | 0.033713551 | Up | 0.000240145 |
| MFAP4 | 1568 | 434.7742182 | | 1056.779127 | 1.281335579 | 0.040273243 | Up | 0.000325297 |
| CYS1 | 2489 | 91.71423651 | | 222.3609065 | 1.277685567 | 0.046781509 | Up | 0.00041585 |
| DIO2 | 3647 | 379.2360864 | | 916.2687438 | 1.272674555 | 0.016438239 | Up | 7.93E-05 |
| IGDCC4 | 3997 | 186.2617924 | | 448.2267775 | 1.266897073 | 0.024165951 | Up | 0.000139527 |
| CASC14 | 1940 | 42.45355842 | | 102.1061697 | 1.266112653 | 0.028831725 | Up | 0.000186875 |
| FOXF1 | 2568 | 143.2536319 | | 339.5706241 | 1.245139942 | 0.043488591 | Up | 0.000366269 |
| COL6A1 | 2829 | 8862.822811 | | 20710.9301 | 1.224554169 | 0.026265146 | Up | 0.000161995 |
| SPON2 | 1346 | 1144.728794 | | 2668.106954 | 1.220810661 | 0.001907089 | Up | 3.63E-06 |
| RPLP0P2 | 1647 | 93.52266058 | | 217.3289289 | 1.216492348 | 0.043762779 | Up | 0.000372717 |
| PDE5A | 2931 | 424.7903523 | | 970.3040961 | 1.191685962 | 0.029787846 | Up | 0.000197321 |
| ITGA5 | 2352 | 2996.894833 | | 6732.338207 | 1.167639196 | 0.003976381 | Up | 1.01E-05 |
| TRPC6 | 2357 | 93.22376968 | | 209.0410231 | 1.165016333 | 0.005148765 | Up | 1.39E-05 |
| TBX2 | 4240 | 479.9874745 | | 1067.810146 | 1.153586499 | 0.015600638 | Up | 7.30E-05 |
| PFKFB4 | 1565 | 581.2210715 | | 1292.247675 | 1.152723694 | 0.024585838 | Up | 0.000144127 |
| ADAMTS4 | 2445 | 833.153734 | | 1829.561265 | 1.134843095 | 0.025480378 | Up | 0.00015388 |
| CASC15 | 2825 | 274.7577102 | | 598.232468 | 1.122546246 | 0.001391418 | Up | 2.43E-06 |
| MN1 | 4393 | 1188.482746 | | 2569.286693 | 1.112246923 | 0.031883309 | Up | 0.00022076 |
| CSRP1 | 2067 | 3114.022061 | | 6727.104344 | 1.111206471 | 0.01096452 | Up | 4.24E-05 |
| COL6A2 | 2964 | 9325.940821 | | 20068.47409 | 1.105609745 | 0.039923939 | Up | 0.000318826 |
| C14orf132 | 7565 | 350.7618974 | | 753.5089549 | 1.103132618 | 0.01808094 | Up | 9.32E-05 |
| PDZRN3 | 2293 | 304.6387647 | | 653.1947141 | 1.100413583 | 0.027682739 | Up | 0.000175141 |
| ANGPTL2 | 3087 | 614.674279 | | 1298.641507 | 1.079109204 | 0.048542724 | Up | 0.000439199 |
| ROR2 | 1763 | 373.4804041 | | 771.6099751 | 1.046839245 | 0.047558641 | Up | 0.000426087 |
| C14orf37 | 3115 | 89.86719834 | | 185.5874938 | 1.046232964 | 0.038723477 | Up | 0.000302406 |
| RBPMS | 1617 | 758.4396197 | | 1553.621818 | 1.034529131 | 0.011824746 | Up | 4.66E-05 |
| NREP | 1814 | 483.8080256 | | 989.0643777 | 1.031629727 | 0.031382955 | Up | 0.000213221 |
| DCHS1 | 10763 | 1217.209529 | | 2443.742035 | 1.005514467 | 0.047931916 | Up | 0.000430491 |
| FLNA | 5335 | 40544.21516 | | 81327.13869 | 1.004240771 | 0.024341338 | Up | 0.000141408 |
| TPM1 | 1485 | 2241.760257 | | 4494.757363 | 1.003611239 | 0.038723477 | Up | 0.000301788 |
| MRPS18C | 1386 | 300.6399739 | | 150.2841495 | -1.00034399 | 0.007838723 | Down | 2.58E-05 |
| ITGB7 | 1799 | 620.6593809 | | 308.7289803 | -1.007460817 | 0.03925233 | Down | 0.000309121 |
| ZNF101 | 3728 | 742.874049 | | 369.3417679 | -1.008161206 | 0.017791518 | Down | 9.07E-05 |
| PTPN6 | 1596 | 1361.226586 | | 674.9595478 | -1.012034289 | 0.037558234 | Down | 0.000283317 |
| SRGN | 1206 | 1608.25003 | | 795.2432242 | -1.016023636 | 0.024662595 | Down | 0.000145122 |
| ARHGAP30 | 4396 | 2221.742246 | | 1097.410754 | -1.017587835 | 0.033776224 | Down | 0.000241339 |
| SLC15A2 | 2635 | 507.2515342 | | 249.6581885 | -1.022747097 | 0.01325434 | Down | 5.58E-05 |
| LCP2 | 1130 | 895.749804 | | 440.4270822 | -1.024192639 | 0.01325434 | Down | 5.62E-05 |
| FLI1 | 3102 | 657.0174177 | | 322.9245231 | -1.024734613 | 0.040415364 | Down | 0.000334373 |
| FAM107B | 2190 | 1333.883758 | | 645.632036 | -1.046848876 | 0.037558234 | Down | 0.00028249 |
| HLA-DPB1 | 1078 | 1807.173788 | | 871.483888 | -1.052189354 | 0.044945025 | Down | 0.000385769 |
| LAPTM5 | 2103 | 5843.618113 | | 2795.519739 | -1.063745368 | 0.012838968 | Down | 5.28E-05 |
| ARHGEF6 | 5035 | 743.6484685 | | 353.4156191 | -1.073255003 | 0.027064262 | Down | 0.000169725 |
| SERPINA1 | 1404 | 598.6164579 | | 282.6025694 | -1.082857361 | 0.040273243 | Down | 0.00032607 |
| HLA-DMA | 1139 | 919.9706251 | | 433.6534998 | -1.085045043 | 0.013191395 | Down | 5.46E-05 |
| DENND2D | 1976 | 1206.491333 | | 564.3825124 | -1.096072361 | 0.017615369 | Down | 8.88E-05 |
| CD209 | 3847 | 341.8007979 | | 158.9070154 | -1.104972947 | 0.034806211 | Down | 0.000252548 |
| CTSS | 1395 | 1480.375678 | | 686.4285721 | -1.108781827 | 0.001897636 | Down | 3.57E-06 |
| MGAT4A | 2725 | 982.5918324 | | 455.2222348 | -1.110021221 | 0.017354492 | Down | 8.64E-05 |
| GPSM3 | 1470 | 731.1909454 | | 335.6675153 | -1.12321528 | 0.037988364 | Down | 0.000289923 |
| DOCK10 | 3629 | 1141.577204 | | 515.9664601 | -1.145679238 | 0.015374333 | Down | 7.11E-05 |
| HLA-DRA | 1279 | 6892.631549 | | 3109.158582 | -1.148530694 | 0.014463399 | Down | 6.46E-05 |
| PLCG2 | 1438 | 1285.489059 | | 578.2232875 | -1.152618712 | 0.029499871 | Down | 0.000192115 |
| SEL1L3 | 3231 | 2411.315846 | | 1080.988334 | -1.157469681 | 0.006332437 | Down | 1.98E-05 |
| HCLS1 | 1666 | 1585.683733 | | 707.088255 | -1.165142851 | 0.033611873 | Down | 0.000238677 |
| ARHGDIB | 1210 | 1539.972671 | | 678.1964269 | -1.18312966 | 0.015650687 | Down | 7.41E-05 |
| RAC2 | 1261 | 1490.192941 | | 647.4835198 | -1.202583756 | 0.031152905 | Down | 0.000210879 |
| FUCA1 | 2047 | 644.7969611 | | 278.8117642 | -1.209553509 | 0.000315784 | Down | 3.45E-07 |
| PPM1K | 3954 | 1337.606467 | | 572.4700314 | -1.224381652 | 0.018099992 | Down | 9.41E-05 |
| LPXN | 1591 | 511.4655086 | | 217.6683128 | -1.232505551 | 0.029544076 | Down | 0.000193453 |
| IL7R | 3105 | 2508.844946 | | 1065.909652 | -1.234938152 | 0.041385048 | Down | 0.000344281 |
| VAV1 | 1743 | 583.0270344 | | 244.9653254 | -1.250985229 | 0.016129766 | Down | 7.67E-05 |
| HLA-DMB | 1894 | 1303.594201 | | 537.9850452 | -1.276856865 | 0.00828142 | Down | 2.89E-05 |
| AIF1 | 708 | 155.4737115 | | 63.98436324 | -1.280879379 | 0.024987636 | Down | 0.000148693 |
| RASGRP3 | 1910 | 556.8418068 | | 228.7230579 | -1.283665717 | 0.037820958 | Down | 0.000286136 |
| DOCK2 | 3277 | 1706.908638 | | 690.6338103 | -1.305392972 | 0.031883309 | Down | 0.000220663 |
| PIK3AP1 | 4138 | 1085.478065 | | 432.8910402 | -1.326254728 | 0.000771555 | Down | 1.06E-06 |
| RPS23P8 | 330 | 63.55235481 | | 25.31833335 | -1.327763144 | 0.017615369 | Down | 8.92E-05 |
| KLHL6 | 4731 | 2056.309182 | | 819.074933 | -1.327989854 | 0.024165951 | Down | 0.000138652 |
| LCP1 | 3456 | 3826.678964 | | 1508.075898 | -1.343383834 | 0.014233948 | Down | 6.30E-05 |
| SPN | 4076 | 1155.664491 | | 453.0816489 | -1.350879655 | 0.011824746 | Down | 4.71E-05 |
| UCP2 | 1139 | 1749.345628 | | 685.327874 | -1.351949087 | 0.006282674 | Down | 1.90E-05 |
| ST6GAL1 | 2282 | 3413.894071 | | 1332.854271 | -1.356899244 | 0.048542724 | Down | 0.000438593 |
| ARHGAP25 | 2139 | 566.2679486 | | 221.045734 | -1.357139982 | 0.009055849 | Down | 3.23E-05 |
| NCKAP1L | 2549 | 1544.414009 | | 598.6778543 | -1.367207736 | 0.004690824 | Down | 1.25E-05 |
| GIMAP5 | 2688 | 915.6186988 | | 354.8867954 | -1.36738803 | 0.030056176 | Down | 0.000200796 |
| PLEK | 2536 | 763.7419063 | | 292.6729746 | -1.383795653 | 0.001897636 | Down | 3.55E-06 |
| ADRA2A | 3869 | 633.6761988 | | 240.6780295 | -1.396641378 | 0.023712732 | Down | 0.000134318 |
| ST8SIA4 | 4130 | 514.0179575 | | 192.5421223 | -1.416644664 | 0.007569928 | Down | 2.44E-05 |
| ALOX5AP | 875 | 159.5741696 | | 58.0943669 | -1.457756955 | 0.00178419 | Down | 3.28E-06 |
| P2RY13 | 2764 | 111.9131796 | | 40.72529428 | -1.458382918 | 0.038505188 | Down | 0.000296594 |
| EMB | 3075 | 986.9896976 | | 354.7101325 | -1.476394483 | 0.002401645 | Down | 4.89E-06 |
| CYFIP2 | 2234 | 1245.769129 | | 443.1598465 | -1.491137653 | 0.023712732 | Down | 0.00013487 |
| CHI3L2 | 1908 | 147.7496085 | | 52.45586066 | -1.493978435 | 0.046311939 | Down | 0.000407305 |
| GIMAP4 | 1589 | 403.1893686 | | 142.9297848 | -1.496151011 | 0.048044824 | Down | 0.000432568 |
| KBTBD8 | 2423 | 204.9687397 | | 72.60497581 | -1.497263569 | 0.002612566 | Down | 5.49E-06 |
| ITGAL | 2546 | 1694.904064 | | 598.7430469 | -1.501194713 | 0.016438239 | Down | 7.88E-05 |
| CD53 | 1161 | 1303.660732 | | 457.6604426 | -1.510218963 | 0.00111233 | Down | 1.75E-06 |
| MYBL1 | 2659 | 234.2087377 | | 81.98990902 | -1.514276635 | 0.049658566 | Down | 0.000453688 |
| WDFY4 | 6164 | 3358.012288 | | 1168.448752 | -1.523013051 | 0.023712732 | Down | 0.000134042 |
| BIRC3 | 4528 | 2598.831148 | | 890.2018972 | -1.545658421 | 0.034578397 | Down | 0.00025013 |
| CORO1A | 1850 | 3609.465904 | | 1229.570548 | -1.553630862 | 0.014249131 | Down | 6.34E-05 |
| MIR155HG | 1600 | 131.9109959 | | 44.86418606 | -1.555928688 | 0.026265146 | Down | 0.000162686 |
| IKZF1 | 2675 | 2552.637484 | | 863.9332219 | -1.562996958 | 0.018094235 | Down | 9.37E-05 |
| SASH3 | 2315 | 1039.717024 | | 346.4221647 | -1.585587784 | 0.001154453 | Down | 1.89E-06 |
| ATP8A1 | 3184 | 795.8105293 | | 263.0403595 | -1.597140812 | 0.031559744 | Down | 0.000217124 |
| BCL2A1 | 888 | 78.00002122 | | 25.30930073 | -1.623806869 | 0.024878387 | Down | 0.000146942 |
| RHOH | 1025 | 614.5226183 | | 199.1310321 | -1.625748047 | 0.043488591 | Down | 0.000366534 |
| RP11-147L13.8 | 2919 | 73.89762082 | | 23.6610755 | -1.643012264 | 0.033166664 | Down | 0.000232658 |
| GIMAP7 | 1256 | 217.7017876 | | 68.16515198 | -1.675246969 | 0.046311939 | Down | 0.000408595 |
| IRF4 | 2582 | 819.2577795 | | 255.5651159 | -1.680626542 | 0.017791518 | Down | 9.09E-05 |
| CD52 | 472 | 297.7204776 | | 92.5470282 | -1.685699886 | 0.015600638 | Down | 7.34E-05 |
| SCIMP | 2285 | 438.8374394 | | 135.7736166 | -1.69248345 | 0.008007816 | Down | 2.76E-05 |
| IRF8 | 1459 | 1351.295913 | | 417.2317162 | -1.695422902 | 0.002625324 | Down | 5.65E-06 |
| ARHGAP15 | 1236 | 316.1397291 | | 97.13245203 | -1.702537063 | 0.029499871 | Down | 0.000192511 |
| NAPSB | 1045 | 359.0119365 | | 109.7511347 | -1.709795956 | 0.017913059 | Down | 9.19E-05 |
| PTPRC | 2405 | 2615.947991 | | 793.9246626 | -1.72025984 | 0.000943889 | Down | 1.42E-06 |
| LINC00487 | 2131 | 17.68944454 | | 5.35387151 | -1.724234326 | 0.049744322 | Down | 0.000456875 |
| RP11-1094M14.8 | 958 | 123.1110846 | | 37.23195041 | -1.725347565 | 0.034355152 | Down | 0.000246995 |
| IGJ | 1183 | 2209.124957 | | 664.4096505 | -1.733330092 | 0.039496548 | Down | 0.000313666 |
| SH2D1A | 2450 | 126.8864742 | | 38.01623691 | -1.738850653 | 0.030649646 | Down | 0.000206794 |
| USP30-AS1 | 656 | 44.38322435 | | 13.04661367 | -1.766339085 | 0.01325434 | Down | 5.57E-05 |
| KCNJ10 | 5293 | 61.74726016 | | 18.1378122 | -1.767374676 | 0.021821464 | Down | 0.00011875 |
| PCNPP5 | 481 | 22.91054801 | | 6.613350711 | -1.792558652 | 0.023712732 | Down | 0.000135336 |
| F5 | 6770 | 183.2264594 | | 52.09509115 | -1.814408514 | 0.025342997 | Down | 0.000152315 |
| IPCEF1 | 2132 | 364.6499311 | | 102.9779901 | -1.824176104 | 0.004379862 | Down | 1.15E-05 |
| SCML2P2 | 540 | 31.0334061 | | 8.686088435 | -1.837043503 | 0.0492388 | Down | 0.000447589 |
| CD3G | 1527 | 261.4982429 | | 72.85254355 | -1.843750004 | 0.018755326 | Down | 9.87E-05 |
| MIR142 | 1625 | 1014.058893 | | 282.2417989 | -1.845137875 | 0.028758916 | Down | 0.000185332 |
| KIAA0226L | 2232 | 314.6200676 | | 87.19304199 | -1.851325776 | 0.01096452 | Down | 4.23E-05 |
| CR1 | 5468 | 635.7180774 | | 174.3941725 | -1.866035281 | 0.040406951 | Down | 0.00033341 |
| CD180 | 2565 | 220.0603569 | | 58.93822465 | -1.900623763 | 0.008173429 | Down | 2.84E-05 |
| RP11-94L15.2 | 6116 | 1604.516611 | | 429.2942625 | -1.902099929 | 0.01325434 | Down | 5.66E-05 |
| THEMIS | 3294 | 241.3522502 | | 63.44764433 | -1.927501772 | 0.015099615 | Down | 6.85E-05 |
| CLNK | 3870 | 68.98098668 | | 18.1329235 | -1.927587221 | 0.016716267 | Down | 8.17E-05 |
| RP11-624C23.1 | 664 | 24.82419718 | | 6.50855087 | -1.931338794 | 0.033166664 | Down | 0.000234782 |
| HVCN1 | 1400 | 234.1491259 | | 60.62065984 | -1.949546192 | 0.013859639 | Down |  |
| IKZF3 | 1921 | 607.5213761 | | 156.6243306 | -1.955626828 | 0.030078192 | Down | 0.000201608 |
| SP140 | 1124 | 355.8057717 | | 90.4679979 | -1.975610464 | 0.034578397 | Down | 0.000250028 |
| LTA | 1422 | 26.76903049 | | 6.748076364 | -1.988016684 | 0.049658566 | Down | 0.000453196 |
| LINC00861 | 3914 | 528.3295956 | | 132.9214422 | -1.990864377 | 0.006913625 | Down | 2.22E-05 |
| PARP15 | 3272 | 611.5196449 | | 153.3738069 | -1.995346725 | 0.005485427 | Down | 1.51E-05 |
| RP11-284N8.3 | 3481 | 267.6824516 | | 65.88938757 | -2.022404541 | 0.032473247 | Down | 0.000225564 |
| P2RY10 | 1088 | 118.6927786 | | 29.17996344 | -2.024182182 | 0.016958564 | Down | 8.37E-05 |
| TESPA1 | 2208 | 194.6658871 | | 47.44646584 | -2.036627557 | 0.03691641 | Down | 0.000273576 |
| SLC1A2 | 7164 | 123.1833741 | | 28.9953354 | -2.086914819 | 0.006301047 | Down | 1.95E-05 |
| HS3ST2 | 2314 | 74.67751172 | | 17.359482 | -2.104949958 | 0.041385048 | Down | 0.000345142 |
| HSPB1P2 | 546 | 5.625491091 | | 1.295636267 | -2.118318288 | 0.043508886 | Down | 0.000367667 |
| CD3D | 645 | 174.9862666 | | 39.93571581 | -2.131490222 | 0.005536307 | Down | 1.57E-05 |
| CTD-3105H18.13 | 1990 | 21.02291333 | | 4.786209325 | -2.13500721 | 0.032783019 | Down | 0.000229166 |
| TRIM31 | 1400 | 25.66129387 | | 5.778617492 | -2.150797634 | 0.046311939 | Down | 0.00040877 |
| MUC16 | 43816 | 1338.50845 | | 299.724277 | -2.158918398 | 0.036062008 | Down | 0.000264851 |
| GRIN2B | 27217 | 70.99531772 | | 15.6767996 | -2.179092814 | 0.023070243 | Down | 0.000128607 |
| CXCL13 | 1101 | 612.7941167 | | 134.3937136 | -2.18893679 | 0.040273243 | Down | 0.000325395 |
| RP11-404F10.2 | 1100 | 30.97693983 | | 6.763702985 | -2.195309416 | 0.008357012 | Down | 2.96E-05 |
| CCL21 | 835 | 1125.941939 | | 245.0718601 | -2.19985569 | 0.0103113 | Down | 3.74E-05 |
| GPR174 | 1258 | 124.6221412 | | 26.79146756 | -2.217714895 | 0.041288957 | Down | 0.000342514 |
| CTB-114C7.4 | 2182 | 19.68184557 | | 4.22948007 | -2.21831328 | 0.046311939 | Down | 0.000406586 |
| LIX1 | 4023 | 23.8784893 | | 5.12195723 | -2.220944455 | 0.006528527 | Down | 2.05E-05 |
| GAPT | 1595 | 67.68326611 | | 14.40820132 | -2.231908944 | 0.046245418 | Down | 0.000403068 |
| FAM65B | 2758 | 961.2063568 | | 202.9158411 | -2.243964693 | 0.005742067 | Down | 1.65E-05 |
| CETP | 1652 | 81.98876246 | | 17.287475 | -2.245699019 | 0.006108022 | Down | 1.77E-05 |
| PLA2G2D | 2672 | 276.4146269 | | 58.25685422 | -2.246334255 | 0.040406951 | Down | 0.000332646 |
| RP3-375P9.2 | 498 | 16.62238663 | | 3.491943418 | -2.251025451 | 0.010509553 | Down | 3.97E-05 |
| RP11-568N6.1 | 1907 | 23.42211937 | | 4.89928624 | -2.257228136 | 0.031526578 | Down | 0.000216159 |
| VNN2 | 1248 | 77.42068973 | | 16.19229028 | -2.257412101 | 0.01546096 | Down | 7.18E-05 |
| LY86-AS1 | 1098 | 21.35627622 | | 4.429056808 | -2.269588707 | 0.022094998 | Down | 0.00012155 |
| CD48 | 1216 | 572.9227424 | | 118.1165537 | -2.278129439 | 0.001384319 | Down | 2.35E-06 |
| FCRL3 | 2984 | 479.3615531 | | 98.72086507 | -2.279687261 | 0.040273243 | Down | 0.000325593 |
| AC008850.3 | 362 | 9.593014848 | | 1.967468976 | -2.285643403 | 0.040388917 | Down | 0.000328794 |
| OTOA | 3633 | 27.01399506 | | 5.495488724 | -2.29738732 | 0.037420016 | Down | 0.000278964 |
| DACT3-AS1 | 689 | 12.69920019 | | 2.467968343 | -2.363341844 | 0.01325434 | Down | 5.66E-05 |
| AC007386.2 | 2928 | 20.19839314 | | 3.91311937 | -2.3678495 | 0.005494957 | Down | 1.54E-05 |
| CCR6 | 3361 | 311.1713357 | | 58.30949411 | -2.415906458 | 0.002848832 | Down | 6.37E-06 |
| LRMP | 1291 | 300.8497668 | | 55.78474607 | -2.431100652 | 0.003132028 | Down | 7.21E-06 |
| KEL | 1150 | 49.93434994 | | 9.255735375 | -2.431613067 | 0.000411688 | Down | 4.83E-07 |
| RP11-693J15.5 | 10626 | 209.4442978 | | 37.40619456 | -2.485217498 | 0.033166664 | Down | 0.000234175 |
| TTC24 | 1012 | 58.98006826 | | 10.4583179 | -2.495576663 | 0.038505188 | Down | 0.000297275 |
| CXorf65 | 599 | 14.42657187 | | 2.546004152 | -2.502421842 | 0.013519172 | Down | 5.80E-05 |
| GRIA4 | 3738 | 25.8969117 | | 4.564297741 | -2.504315251 | 0.001069489 | Down | 1.63E-06 |
| LTB | 995 | 1096.121169 | | 190.2796649 | -2.526213993 | 0.017350123 | Down | 8.60E-05 |
| CTA-250D10.23 | 1078 | 442.43299 | | 76.59759161 | -2.530088028 | 0.00191214 | Down | 3.68E-06 |
| AE000661.37 | 614 | 19.65532182 | | 3.300193433 | -2.574297493 | 0.044378113 | Down | 0.000379921 |
| PLCXD3 | 7704 | 64.68637031 | | 10.85977318 | -2.574467792 | 0.006108022 | Down | 1.79E-05 |
| PLIN1 | 2331 | 83.86592657 | | 13.97904548 | -2.584818931 | 0.001385432 | Down | 2.39E-06 |
| CTD-2547L16.3 | 1119 | 8.698562844 | | 1.448551591 | -2.586165994 | 0.046311939 | Down | 0.000405692 |
| BANK1 | 2275 | 491.3852914 | | 81.41647462 | -2.593462016 | 0.010467476 | Down | 3.84E-05 |
| DNAH8 | 10190 | 88.55164458 | | 14.633596 | -2.597234766 | 0.037988364 | Down | 0.000289859 |
| PAX5 | 4493 | 1292.327738 | | 213.3440898 | -2.598717938 | 0.005148765 | Down | 1.39E-05 |
| RP11-172E10.1 | 545 | 31.22368873 | | 5.127897273 | -2.60620172 | 0.04595012 | Down | 0.000397633 |
| SLC26A7 | 1410 | 42.08848924 | | 6.859199358 | -2.617313633 | 0.013826281 | Down | 5.98E-05 |
| COL19A1 | 2496 | 112.5535801 | | 18.23739013 | -2.625640755 | 0.025342997 | Down | 0.00015249 |
| RP11-126K1.6 | 618 | 12.18940421 | | 1.97110657 | -2.628549927 | 0.001680448 | Down | 3.05E-06 |
| BTLA | 1587 | 169.7309091 | | 27.30951662 | -2.635773629 | 0.012838968 | Down | 5.27E-05 |
| SELL | 1819 | 650.8647879 | | 102.6375703 | -2.664798943 | 0.000896016 | Down | 1.31E-06 |
| FCRL2 | 2976 | 462.6446619 | | 72.03058774 | -2.683222964 | 0.022094998 | Down | 0.000121705 |
| MCCD1 | 955 | 20.89044545 | | 3.196050167 | -2.708481297 | 0.021975613 | Down | 0.000120075 |
| RP1-317E23.7 | 222 | 13.19158311 | | 2.013846805 | -2.711591866 | 0.02961145 | Down | 0.000194549 |
| AIRE | 3978 | 35.59749752 | | 5.416308623 | -2.716393972 | 0.049744322 | Down | 0.000457399 |
| RP1-245M18.2 | 465 | 19.35011805 | | 2.935262527 | -2.720780921 | 0.034355152 | Down | 0.000246697 |
| TRAT1 | 1653 | 66.16078067 | | 10.01969886 | -2.723137109 | 0.012544386 | Down | 5.08E-05 |
| TRBV28 | 364 | 21.29168359 | | 3.122009929 | -2.769743001 | 0.040406951 | Down | 0.000333313 |
| VPREB3 | 610 | 175.0043528 | | 25.01525798 | -2.80651057 | 0.008007816 | Down | 2.72E-05 |
| LINC00402 | 3340 | 116.2971842 | | 16.3536962 | -2.830127516 | 0.027064262 | Down | 0.000170031 |
| ADH1C | 1740 | 89.73398924 | | 12.5616357 | -2.836630214 | 0.022406236 | Down | 0.00012441 |
| FCRLA | 1552 | 172.0127167 | | 23.6501419 | -2.862594481 | 0.025651731 | Down | 0.000155482 |
| RP11-713N11.3 | 1186 | 66.19705632 | | 8.863596765 | -2.900802909 | 0.006177514 | Down | 1.84E-05 |
| SPIB | 1333 | 387.2289669 | | 51.14296986 | -2.920579033 | 0.003362825 | Down | 7.81E-06 |
| STAP1 | 1466 | 56.87305439 | | 7.230259212 | -2.975626012 | 0.000685511 | Down | 8.80E-07 |
| RNVU1-15 | 165 | 29.01571355 | | 3.613264412 | -3.005459671 | 0.007838723 | Down | 2.58E-05 |
| CNR2 | 5254 | 248.4881245 | | 30.59842371 | -3.021647667 | 0.01096452 | Down | 4.22E-05 |
| CLEC17A | 1835 | 126.7805837 | | 15.52137633 | -3.030005419 | 0.002625324 | Down | 5.66E-06 |
| RNU2-63P | 194 | 18.38072598 | | 2.235880967 | -3.03927846 | 0.016438239 | Down | 7.94E-05 |
| RP1-60O19.1 | 2232 | 6.828286641 | | 0.823925849 | -3.050937212 | 0.027682739 | Down | 0.000174614 |
| TLR10 | 3372 | 250.4221832 | | 30.21338641 | -3.051102567 | 0.002837261 | Down | 6.28E-06 |
| TREML2 | 3302 | 78.95261644 | | 9.501492867 | -3.054760965 | 0.010324487 | Down | 3.77E-05 |
| CP | 1784 | 360.8813101 | | 43.24682368 | -3.060858347 | 0.000716401 | Down | 9.35E-07 |
| RP1-55C23.7 | 581 | 8.279008054 | | 0.960829573 | -3.10710546 | 0.017615369 | Down | 8.85E-05 |
| AC079767.4 | 492 | 21.11084279 | | 2.374680064 | -3.152179115 | 0.008007816 | Down | 2.74E-05 |
| CD22 | 1475 | 1433.605928 | | 152.9219714 | -3.228780899 | 0.002625324 | Down | 5.69E-06 |
| RP11-861A13.4 | 2592 | 173.629697 | | 18.15992185 | -3.257183823 | 0.003461165 | Down | 8.35E-06 |
| ART4 | 2723 | 108.8408917 | | 11.20069407 | -3.280560642 | 2.26E-10 | Down | 3.00E-14 |
| RP11-159H10.3 | 787 | 11.29766121 | | 1.123011985 | -3.330578915 | 0.035103373 | Down | 0.000256257 |
| TIMD4 | 1369 | 29.63611439 | | 2.902584948 | -3.351946112 | 0.028758916 | Down | 0.000185767 |
| TDRD15 | 6135 | 13.67772926 | | 1.317485035 | -3.375970258 | 0.040273243 | Down | 0.000324016 |
| CTC-248O19.1 | 911 | 12.26521188 | | 1.149109982 | -3.415983366 | 0.015080497 | Down | 6.81E-05 |
| RP11-210H10__A.1 | 400 | 9.292880896 | | 0.868771055 | -3.419077975 | 0.031382955 | Down | 0.000213825 |
| RP11-564A8.4 | 611 | 62.39075945 | | 5.568742854 | -3.48590879 | 7.84E-06 | Down | 5.03E-09 |
| MTRNR2L6 | 1447 | 12.73873913 | | 1.128490671 | -3.496756091 | 0.012145363 | Down | 4.86E-05 |
| OSTN | 3134 | 8.698643645 | | 0.747015064 | -3.541581221 | 0.003879277 | Down | 9.70E-06 |
| TCL6 | 2314 | 187.5095639 | | 15.17416698 | -3.627274958 | 5.71E-06 | Down | 3.54E-09 |
| RP11-564A8.8 | 466 | 45.91762791 | | 3.688872059 | -3.637796457 | 0.000816357 | Down | 1.14E-06 |
| SPIC | 993 | 12.79093156 | | 0.996562126 | -3.682017783 | 0.024341406 | Down | 0.000142155 |
| RP11-225H22.7 | 657 | 7.982663781 | | 0.60619597 | -3.719014081 | 0.043488591 | Down | 0.000366103 |
| ADAM29 | 2307 | 13.8296981 | | 1.047611795 | -3.72259355 | 0.003589767 | Down | 8.89E-06 |
| MS4A1 | 2155 | 1499.250569 | | 111.0201799 | -3.755347679 | 0.001384319 | Down | 2.36E-06 |
| NLRP11 | 3064 | 3.824155067 | | 0.279688128 | -3.773250105 | 0.038931231 | Down | 0.000305731 |
| NKX6-3 | 1698 | 8.49771123 | | 0.619544149 | -3.77779532 | 0.021353357 | Down | 0.00011573 |
| Z95704.4 | 586 | 15.76753898 | | 1.1421611 | -3.78711944 | 0.025342997 | Down | 0.000151899 |
| FCAMR | 1326 | 35.9854201 | | 2.597145236 | -3.792413902 | 0.027064262 | Down | 0.000168953 |
| KRT72 | 2015 | 17.37119237 | | 1.193470981 | -3.863461393 | 0.021155941 | Down | 0.000113724 |
| RP11-1143G9.4 | 599 | 15.35666014 | | 1.007809278 | -3.929569935 | 0.032783019 | Down | 0.000228552 |
| KIAA0125 | 917 | 220.0757434 | | 14.03199902 | -3.971207684 | 0.000109144 | Down | 8.75E-08 |
| TUSC5 | 3596 | 17.1620737 | | 1.070037466 | -4.003490668 | 0.006108022 | Down | 1.80E-05 |
| NPY1R | 1572 | 57.82594248 | | 3.446889178 | -4.068350055 | 4.15E-08 | Down | 1.56E-11 |
| GAPDHP37 | 968 | 4.660913142 | | 0.272515727 | -4.096201234 | 0.019100529 | Down | 0.000101408 |
| TCL1A | 1076 | 352.8144642 | | 20.41670312 | -4.111087883 | 0.001984505 | Down | 3.89E-06 |
| BLK | 1691 | 538.3833528 | | 30.93839627 | -4.121163481 | 4.31E-08 | Down | 1.72E-11 |
| ANKRD20A19P | 856 | 33.62645143 | | 1.910757961 | -4.137379594 | 0.020130022 | Down | 0.000107319 |
| SERPINA9 | 1434 | 29.699017 | | 1.481684462 | -4.32510503 | 0.000252192 | Down | 2.62E-07 |
| FAM129C | 2313 | 757.4751164 | | 32.41772869 | -4.546343577 | 8.88E-12 | Down | 4.89E-16 |
| RP11-542M13.3 | 750 | 5.017206189 | | 0.206322481 | -4.603911297 | 0.01096452 | Down | 4.22E-05 |
| AL355390.1 | 2328 | 14.84618046 | | 0.56335148 | -4.719912688 | 0.000315784 | Down | 3.49E-07 |
| FDCSP | 566 | 112.7345512 | | 4.006152845 | -4.814568469 | 4.57E-05 | Down | 3.44E-08 |
| ADIPOQ | 4562 | 57.36949455 | | 1.868387342 | -4.940418326 | 0.024165951 | Down | 0.000139227 |
| RP11-148O21.3 | 435 | 9.914529972 | | 0.318122471 | -4.961890193 | 0.002382489 | Down | 4.80E-06 |
| AICDA | 2819 | 69.08296863 | | 1.660155177 | -5.378940076 | 1.39E-05 | Down | 9.21E-09 |
| RP11-203B7.2 | 4159 | 53.75273797 | | 1.284614042 | -5.386931365 | 0.000156996 | Down | 1.46E-07 |
| RP11-164H13.1 | 2738 | 13.64134486 | | 0.313088103 | -5.445273385 | 0.010509553 | Down | 3.96E-05 |
| FCER2 | 1567 | 129.4243999 | | 2.217905259 | -5.866768079 | 8.88E-12 | Down | 7.86E-16 |
| CLEC4M | 1669 | 77.27938382 | | 0.583066276 | -7.050279899 | 3.13E-09 | Down | 5.54E-13 |
| **Differential RNA expression between PT and LN_met_** | | | | | | | | |
| **Gene ID** | **Length** | **PT-Expression** | | **LN_met_-Expression** | **log2 FoldChange(PT/LN_met_)** | **Padj** | **Up/Down-Regulation** | **P-value** |
| RP11-164H13.1 | 2738 | 0.192886116 | 18.56091048 | | 6.588374366 | 2.05E-05 | Up | 2.52E-08 |
| ADIPOQ | 4562 | 0.718463515 | 42.03770627 | | 5.870625247 | 0.002946494 | Up | 2.14E-05 |
| CLEC4M | 1472 | 1.555757695 | 57.59653464 | | 5.210292726 | 7.39E-06 | Up | 5.27E-09 |
| AICDA | 2819 | 1.456359747 | 51.32587214 | | 5.139247561 | 5.43E-06 | Up | 3.52E-09 |
| RP11-148O21.3 | 435 | 0.212276968 | 7.325812837 | | 5.108969092 | 5.03E-05 | Up | 8.16E-08 |
| C7orf62 | 1023 | 0.130672907 | 3.920908036 | | 4.907155851 | 0.037901723 | Up | 0.0007895 |
| OR2T10 | 1049 | 0.127939855 | 3.400208632 | | 4.732085609 | 0.027957544 | Up | 0.000511002 |
| AC084082.3 | 954 | 0.149823538 | 3.638299604 | | 4.601928146 | 0.000358678 | Up | 1.16E-06 |
| RP11-203B7.2 | 4159 | 1.682446779 | 40.48411488 | | 4.588723162 | 0.011555711 | Up | 0.000145724 |
| ZPBP2 | 1277 | 0.136677771 | 3.196991405 | | 4.547864339 | 0.026091784 | Up | 0.000468434 |
| AC132216.1 | 1150 | 0.166074167 | 3.859229586 | | 4.538413291 | 0.01081697 | Up | 0.000134635 |
| OR2T3 | 1008 | 0.117928297 | 2.694080429 | | 4.513811078 | 0.038609374 | Up | 0.000808416 |
| TDRD15 | 6135 | 1.228316823 | 25.53791755 | | 4.377886255 | 0.000115553 | Up | 2.47E-07 |
| TUSC5 | 3596 | 0.64255064 | 12.97411258 | | 4.335681894 | 0.000127409 | Up | 2.81E-07 |
| SPIC | 1048 | 0.487120533 | 9.654888437 | | 4.308908888 | 0.000586189 | Up | 2.18E-06 |
| RP11-542M13.3 | 750 | 0.202799749 | 3.820986396 | | 4.235817353 | 0.002258295 | Up | 1.45E-05 |
| FCAMR | 1239 | 1.572710393 | 27.30685825 | | 4.117938401 | 0.000146317 | Up | 3.39E-07 |
| RP11-164H13.1 | 2738 | 0.192886116 | 18.56091048 | | 6.588374366 | 2.05E-05 | Up | 2.52E-08 |
| FCER2 | 1560 | 0.718463515 | 42.03770627 | | 5.870625247 | 0.000124856 | Up | 2.73E-07 |
| TCL1A | 763 | 1.555757695 | 57.59653464 | | 5.210292726 | 4.23E-05 | Up | 6.40E-08 |
| RP11-438F14.3 | 955 | 1.456359747 | 51.32587214 | | 5.139247561 | 0.033116516 | Up | 0.000657588 |
| MS4A1 | 2095 | 0.212276968 | 7.325812837 | | 5.108969092 | 1.25E-05 | Up | 1.16E-08 |
| TIMD4 | 1145 | 0.130672907 | 3.920908036 | | 4.907155851 | 0.000231995 | Up | 6.52E-07 |
| CR2 | 3185 | 0.127939855 | 3.400208632 | | 4.732085609 | 0.001002317 | Up | 4.51E-06 |
| TRBV7-4 | 347 | 0.146581023 | 3.638299604 | | 4.601928146 | 0.040239476 | Up | 0.000857916 |
| RP11-564A8.8 | 466 | 2.375249564 | 35.22333512 | | 3.890380508 | 4.81E-05 | Up | 7.70E-08 |
| FDCSP | 566 | 6.324473095 | 87.21724681 | | 3.785596253 | 0.007153215 | Up | 7.46E-05 |
| RP5-1050D4.3 | 474 | 1.406730243 | 19.33402566 | | 3.780724455 | 0.022485845 | Up | 0.000384385 |
| KRT72 | 1679 | 1.024583912 | 13.41117682 | | 3.710325789 | 0.002634395 | Up | 1.78E-05 |
| RP11-84D1.2 | 2443 | 0.22529093 | 2.87409511 | | 3.673246666 | 0.021790763 | Up | 0.000368592 |
| GAPDHP37 | 968 | 0.294119353 | 3.647074483 | | 3.632266041 | 0.003580223 | Up | 2.79E-05 |
| NLRP11 | 3777 | 0.241890596 | 2.928608825 | | 3.597788915 | 0.026069016 | Up | 0.000467462 |
| OSTN | 3134 | 0.55904164 | 6.759541494 | | 3.595897741 | 0.000819897 | Up | 3.51E-06 |
| CLEC4G | 1360 | 2.231808541 | 26.46280636 | | 3.567680892 | 0.000724002 | Up | 2.83E-06 |
| NPY5R | 3183 | 0.361128991 | 4.230110402 | | 3.550109171 | 0.023819381 | Up | 0.000413211 |
| CTC-248O19.1 | 911 | 0.834801728 | 9.541159274 | | 3.514659076 | 0.0002916 | Up | 8.70E-07 |
| RGS7 | 1686 | 0.551033354 | 6.203039139 | | 3.492763676 | 0.029361785 | Up | 0.000555086 |
| MTRNR2L6 | 1447 | 0.905593428 | 9.992531872 | | 3.463914875 | 0.001787061 | Up | 9.93E-06 |
| RP11-210K20.3 | 283 | 0.42406951 | 4.562171245 | | 3.427347937 | 0.019211116 | Up | 0.000305797 |
| AC079767.4 | 492 | 1.575285334 | 16.52569022 | | 3.391025454 | 9.94E-05 | Up | 1.96E-07 |
| RP11-457M11.6 | 1207 | 0.466673806 | 4.806513263 | | 3.364504317 | 0.003051288 | Up | 2.26E-05 |
| RP11-398A8.1 | 838 | 0.457677569 | 4.681841617 | | 3.354672638 | 0.009049417 | Up | 0.000104527 |
| CNR2 | 5254 | 19.14294719 | 195.1820425 | | 3.34993546 | 0.000525897 | Up | 1.88E-06 |
| RP11-210H10__A.1 | 499 | 0.715904302 | 7.271433897 | | 3.344401232 | 0.001445186 | Up | 7.60E-06 |
| TCL1B | 1724 | 0.824012385 | 8.30628132 | | 3.333464807 | 0.015535729 | Up | 0.000224973 |
| OLFM4 | 2897 | 3.679887113 | 36.82379512 | | 3.322904906 | 0.046478784 | Up | 0.001054622 |
| RP11-861A13.4 | 2592 | 13.66453878 | 136.705199 | | 3.322559441 | 3.19E-05 | Up | 4.56E-08 |
| RP11-855A2.5 | 414 | 0.426014562 | 4.248305955 | | 3.317913018 | 0.006917609 | Up | 6.96E-05 |
| CD22 | 1064 | 113.2979538 | 1129.575535 | | 3.317587036 | 1.85E-05 | Up | 2.04E-08 |
| AL355390.1 | 2328 | 1.220601555 | 11.77047746 | | 3.269508605 | 0.016739422 | Up | 0.000251285 |
| CLEC17A | 1462 | 10.40106525 | 100.1218725 | | 3.266953981 | 2.28E-05 | Up | 2.91E-08 |
| KIAA0125 | 2936 | 18.33191914 | 174.8119688 | | 3.253374232 | 0.001050468 | Up | 4.82E-06 |
| PRAMENP | 2334 | 1.86780809 | 17.77860271 | | 3.250723804 | 0.007198374 | Up | 7.52E-05 |
| NPHS1 | 3741 | 1.424805486 | 13.54441996 | | 3.24886173 | 0.00578959 | Up | 5.42E-05 |
| FCRL1 | 1480 | 42.13081577 | 399.0523367 | | 3.243630216 | 0.004390198 | Up | 3.54E-05 |
| RP11-159H10.3 | 828 | 0.940824561 | 8.894144111 | | 3.240858152 | 0.002810458 | Up | 1.96E-05 |
| C9orf57 | 1502 | 0.795953307 | 7.416365987 | | 3.219956734 | 0.016528228 | Up | 0.000247042 |
| OCM | 695 | 0.378632831 | 3.50024603 | | 3.208584914 | 0.011719872 | Up | 0.000148555 |
| TCL6 | 2527 | 16.38757399 | 149.1825548 | | 3.18640464 | 4.03E-06 | Up | 2.35E-09 |
| CCDC168 | 7359 | 0.744660728 | 6.770077902 | | 3.184517256 | 0.030947985 | Up | 0.000596233 |
| RP11-324I22.3 | 335 | 0.445148506 | 3.972588166 | | 3.15772062 | 0.017433896 | Up | 0.000267744 |
| ART4 | 1549 | 9.768496018 | 86.51375932 | | 3.146721237 | 1.66E-13 | Up | 3.59E-18 |
| SERPINA9 | 1156 | 2.684070993 | 23.75330384 | | 3.145633456 | 0.00260266 | Up | 1.75E-05 |
| RP11-452J21.2 | 414 | 1.100235827 | 9.659147923 | | 3.13408314 | 0.001407487 | Up | 7.28E-06 |
| RPL31P11 | 1558 | 0.806289882 | 6.803726291 | | 3.07695458 | 0.043282891 | Up | 0.000955892 |
| TLR10 | 2702 | 23.88934852 | 200.1400318 | | 3.066570346 | 1.67E-05 | Up | 1.77E-08 |
| FBXO40 | 5926 | 1.72217918 | 14.41321416 | | 3.065084937 | 0.010784122 | Up | 0.000133661 |
| SPIB | 1433 | 36.97201146 | 309.2548497 | | 3.064290779 | 1.98E-05 | Up | 2.31E-08 |
| CTSE | 2228 | 0.470417489 | 3.869079608 | | 3.039976811 | 0.04355663 | Up | 0.0009696 |
| U62631.5 | 435 | 3.225651102 | 26.45289342 | | 3.035763228 | 0.03353736 | Up | 0.000673199 |
| RP1-245M18.2 | 465 | 1.912713638 | 15.45899835 | | 3.014754043 | 0.002237675 | Up | 1.39E-05 |
| FAM129C | 3136 | 76.28852305 | 612.7782658 | | 3.005827191 | 0.000206602 | Up | 5.63E-07 |
| PLCXD3 | 7538 | 6.480401383 | 51.65767444 | | 2.994827618 | 0.001492129 | Up | 7.97E-06 |
| RP1-55C23.7 | 581 | 0.853283895 | 6.714383536 | | 2.976157225 | 0.005697352 | Up | 5.27E-05 |
| CREB3L3 | 2279 | 0.456992187 | 3.52953204 | | 2.949235512 | 0.049149324 | Up | 0.001163059 |
| LIX1 | 529 | 4.694627376 | 36.17274835 | | 2.945820658 | 1.76E-05 | Up | 1.90E-08 |
| RP11-428G5.5 | 852 | 3.859525683 | 29.57789695 | | 2.938024015 | 0.016269223 | Up | 0.000239652 |
| SNORA46 | 135 | 1.981553283 | 15.16789995 | | 2.936317686 | 0.000526485 | Up | 1.89E-06 |
| TRAT1 | 1845 | 7.002518714 | 53.18854577 | | 2.925169754 | 0.000503414 | Up | 1.77E-06 |
| FCRL2 | 2248 | 49.59121691 | 372.6856057 | | 2.909802566 | 0.000353857 | Up | 1.12E-06 |
| MCCD1 | 955 | 2.236755808 | 16.74697414 | | 2.904420784 | 0.000768489 | Up | 3.16E-06 |
| RP11-356M20.1 | 802 | 0.338526935 | 2.526684628 | | 2.899903071 | 0.02022611 | Up | 0.000325938 |
| PAX5 | 4065 | 139.9051907 | 1040.65992 | | 2.894977289 | 5.17E-06 | Up | 3.24E-09 |
| VPREB3 | 610 | 19.18713574 | 141.3739433 | | 2.881304974 | 0.000107422 | Up | 2.18E-07 |
| AC104809.4 | 2274 | 3.189526494 | 23.49699133 | | 2.881061872 | 0.017459116 | Up | 0.000268886 |
| CIR1P2 | 1342 | 2.153006806 | 15.78766786 | | 2.874373288 | 0.004886641 | Up | 4.18E-05 |
| NPY1R | 1588 | 6.460233022 | 47.24751999 | | 2.870580495 | 0.00123347 | Up | 5.87E-06 |
| BANK1 | 2629 | 54.40421994 | 396.5014421 | | 2.865535647 | 9.93E-06 | Up | 8.30E-09 |
| RP11-693J15.5 | 10626 | 23.21704434 | 168.8281148 | | 2.862298949 | 0.000228779 | Up | 6.38E-07 |
| CTD-2547L16.3 | 1119 | 0.982938994 | 7.016215118 | | 2.835519198 | 0.001431687 | Up | 7.43E-06 |
| STAP1 | 1511 | 6.464427614 | 46.12791846 | | 2.835045653 | 2.83E-05 | Up | 3.91E-08 |
| RP11-4M23.2 | 1267 | 6.329744019 | 45.0888422 | | 2.832551403 | 0.018152336 | Up | 0.000283097 |
| RP11-524O1.4 | 629 | 0.604697436 | 4.281771147 | | 2.823922323 | 0.04355663 | Up | 0.000970417 |
| SELL | 1644 | 74.64027129 | 527.2943386 | | 2.820582372 | 9.93E-06 | Up | 8.59E-09 |
| RP11-338L22.3 | 514 | 0.226144299 | 1.592600669 | | 2.816069038 | 0.036925399 | Up | 0.00075899 |
| LINC00402 | 3340 | 13.65271753 | 94.62882396 | | 2.793091552 | 0.005545358 | Up | 5.05E-05 |
| EIF5AP2 | 1047 | 0.691140156 | 4.789468389 | | 2.792815323 | 0.03898873 | Up | 0.000820576 |
| FCRLA | 2064 | 20.26629791 | 139.9229864 | | 2.787478508 | 0.003770563 | Up | 2.96E-05 |
| DNAH8 | 13049 | 10.54113076 | 71.96133928 | | 2.771192402 | 0.009063824 | Up | 0.000104996 |
| TTC24 | 1939 | 7.038317389 | 47.89499972 | | 2.766572567 | 0.001271063 | Up | 6.27E-06 |
| LTB | 916 | 132.6929519 | 891.5465154 | | 2.748218328 | 4.55E-05 | Up | 7.19E-08 |
| GLYCTK-AS1 | 643 | 3.332694679 | 22.22255132 | | 2.737263401 | 0.011267852 | Up | 0.000141363 |
| RP11-433M22.1 | 333 | 0.501525269 | 3.337072904 | | 2.734188908 | 0.0316264 | Up | 0.000619106 |
| NCR3 | 875 | 5.313402111 | 35.17397123 | | 2.726800425 | 0.002855835 | Up | 2.01E-05 |
| SLC26A7 | 1685 | 5.212118783 | 34.35883622 | | 2.720739303 | 0.001271063 | Up | 6.20E-06 |
| ASNSP5 | 153 | 0.730166057 | 4.791333258 | | 2.714130654 | 0.028144045 | Up | 0.000516237 |
| RP11-713N11.3 | 1186 | 8.322552353 | 54.22618442 | | 2.703891714 | 0.001104592 | Up | 5.09E-06 |
| C4BPB | 1001 | 1.377597958 | 8.950741442 | | 2.699852285 | 0.003576518 | Up | 2.77E-05 |
| PCDP1 | 589 | 0.850794404 | 5.517696221 | | 2.697183581 | 0.034049565 | Up | 0.000684217 |
| RP11-172E10.1 | 545 | 4.029496113 | 25.56065708 | | 2.665253577 | 0.001481664 | Up | 7.88E-06 |
| RP11-217B1.2 | 1109 | 2.898795531 | 18.33068278 | | 2.660735044 | 0.00886166 | Up | 0.000101241 |
| TREML2 | 3302 | 10.31952363 | 65.19351129 | | 2.659352005 | 0.000817528 | Up | 3.43E-06 |
| ENAM | 5679 | 7.332689839 | 46.19496722 | | 2.655321262 | 0.008976985 | Up | 0.000103496 |
| AC092667.2 | 2586 | 12.85490383 | 80.61682255 | | 2.648762105 | 0.018595205 | Up | 0.000292819 |
| FCRL3 | 3397 | 62.647773 | 392.0101395 | | 2.64555584 | 0.000166745 | Up | 4.11E-07 |
| BTLA | 1026 | 22.28233431 | 139.340803 | | 2.644645499 | 0.002284294 | Up | 1.48E-05 |
| PLIN1 | 2279 | 11.07318114 | 68.87043225 | | 2.636814987 | 1.19E-05 | Up | 1.09E-08 |
| BLK | 2205 | 73.51656059 | 447.8063528 | | 2.606733817 | 0.012227679 | Up | 0.000157901 |
| OTOA | 1608 | 3.632778992 | 22.12293678 | | 2.606397409 | 0.000845481 | Up | 3.66E-06 |
| RP11-338E21.2 | 281 | 0.996705409 | 6.03200449 | | 2.59739844 | 0.006962838 | Up | 7.14E-05 |
| RP11-561O23.5 | 2060 | 1.572291247 | 9.502777685 | | 2.595480795 | 0.007783932 | Up | 8.29E-05 |
| COL19A1 | 6232 | 15.39863377 | 92.94646836 | | 2.593597694 | 0.005630214 | Up | 5.18E-05 |
| SLC26A4-AS1 | 3687 | 1.886738604 | 11.37788529 | | 2.592265975 | 0.006820874 | Up | 6.77E-05 |
| TRBV7-3 | 397 | 1.39937575 | 8.423571601 | | 2.58964867 | 0.028144045 | Up | 0.000516187 |
| RP11-523O18.7 | 559 | 1.689708179 | 10.04316455 | | 2.571367914 | 0.008179665 | Up | 8.92E-05 |
| CD19 | 2040 | 71.65944552 | 424.8966819 | | 2.567883292 | 0.001271063 | Up | 6.26E-06 |
| TSPAN8 | 1168 | 2.540617026 | 14.95728853 | | 2.557597842 | 0.008927733 | Up | 0.000102349 |
| RP11-493L12.4 | 277 | 0.468433757 | 2.750207918 | | 2.553623741 | 0.038375662 | Up | 0.000801032 |
| TRBV4-2 | 455 | 0.877448446 | 5.14472096 | | 2.551706562 | 0.048834203 | Up | 0.001140683 |
| LRMP | 1504 | 42.77144122 | 248.4409473 | | 2.538183248 | 2.83E-05 | Up | 3.91E-08 |
| RP11-445P17.5 | 435 | 0.889269788 | 5.140168971 | | 2.531122708 | 0.041243515 | Up | 0.000897471 |
| AC005037.6 | 298 | 0.908405694 | 5.231383241 | | 2.525783809 | 0.027532524 | Up | 0.000500851 |
| CCR6 | 3273 | 44.8870883 | 257.5474654 | | 2.520465921 | 1.62E-05 | Up | 1.65E-08 |
| LY86-AS1 | 2819 | 3.076035966 | 17.64610864 | | 2.520205796 | 0.002237675 | Up | 1.39E-05 |
| AC112211.3 | 288 | 1.007680052 | 5.777994144 | | 2.519531099 | 0.037144004 | Up | 0.000765682 |
| GRIA4 | 679 | 3.809649971 | 21.43149745 | | 2.492002302 | 0.000122974 | Up | 2.66E-07 |
| CTA-250D10.23 | 1078 | 65.68498625 | 367.402246 | | 2.483724893 | 0.000108605 | Up | 2.26E-07 |
| PLA2G2D | 2672 | 41.363162 | 229.2450011 | | 2.470471893 | 0.00502514 | Up | 4.37E-05 |
| DACT3-AS1 | 381 | 1.905426123 | 10.51378687 | | 2.464096816 | 0.000496626 | Up | 1.74E-06 |
| PKHD1L1 | 9601 | 19.25614813 | 105.8735928 | | 2.458951745 | 0.018227512 | Up | 0.000284663 |
| CD48 | 1160 | 87.06278275 | 476.1890402 | | 2.451406378 | 1.64E-06 | Up | 6.39E-10 |
| AP003774.1 | 672 | 1.411403184 | 7.708911096 | | 2.449396921 | 0.005669157 | Up | 5.22E-05 |
| ATP5A1P3 | 1507 | 1.070018437 | 5.826064413 | | 2.444885997 | 0.002982928 | Up | 2.18E-05 |
| CD79B | 1053 | 58.71153067 | 318.0364907 | | 2.437476531 | 0.001307535 | Up | 6.67E-06 |
| CALM2P3 | 449 | 1.086357477 | 5.881623341 | | 2.436715482 | 0.03491714 | Up | 0.000706938 |
| SETP9 | 832 | 0.500801759 | 2.690881094 | | 2.425767107 | 0.030947985 | Up | 0.000596454 |
| FAM65B | 2583 | 149.0420131 | 800.3394029 | | 2.424892873 | 7.48E-06 | Up | 5.67E-09 |
| TRBV5-6 | 384 | 1.609689803 | 8.633841537 | | 2.423219915 | 0.008711703 | Up | 9.85E-05 |
| HLA-DPB2 | 776 | 2.91392958 | 15.61330326 | | 2.421737878 | 0.006536337 | Up | 6.37E-05 |
| TTC4P1 | 1158 | 0.612247358 | 3.249523222 | | 2.40804151 | 0.043538715 | Up | 0.000963674 |
| VNN2 | 1608 | 12.17929491 | 64.56158578 | | 2.406245401 | 0.000171069 | Up | 4.30E-07 |
| AC004906.3 | 969 | 1.92321642 | 10.143163 | | 2.398914583 | 0.01315858 | Up | 0.000175615 |
| AC008850.3 | 362 | 1.518440705 | 7.984136183 | | 2.394545756 | 0.001873799 | Up | 1.07E-05 |
| CETP | 890 | 13.06911339 | 68.57705307 | | 2.391564637 | 6.68E-05 | Up | 1.18E-07 |
| AC098824.6 | 980 | 1.306875907 | 6.850977297 | | 2.390187646 | 0.015110876 | Up | 0.000214339 |
| GZMK | 1509 | 16.62482759 | 87.0744122 | | 2.38890945 | 0.008409366 | Up | 9.28E-05 |
| RP11-111M22.5 | 710 | 3.205280999 | 16.74274082 | | 2.385012974 | 0.004357731 | Up | 3.51E-05 |
| CTB-133G6.1 | 1558 | 52.11781896 | 269.6244134 | | 2.371102517 | 0.002677639 | Up | 1.82E-05 |
| TRBV28 | 364 | 3.484118698 | 17.92542654 | | 2.363141768 | 0.012491877 | Up | 0.000162276 |
| FAM32B | 428 | 1.288899517 | 6.621649818 | | 2.361050922 | 0.031703553 | Up | 0.000621302 |
| AC007386.2 | 2928 | 3.314176578 | 16.90612178 | | 2.350823372 | 0.000784841 | Up | 3.25E-06 |
| LINC00861 | 4675 | 86.67687553 | 441.3849806 | | 2.348318484 | 1.19E-05 | Up | 1.06E-08 |
| GAPT | 1124 | 11.14915951 | 56.74893364 | | 2.347658328 | 0.003269556 | Up | 2.45E-05 |
| FAM177B | 1345 | 10.38647181 | 52.54453842 | | 2.338835149 | 0.04206749 | Up | 0.000919951 |
| CD40LG | 1817 | 7.076646114 | 35.67526715 | | 2.333786553 | 0.007742394 | Up | 8.22E-05 |
| RP11-455G16.1 | 2097 | 1.656545635 | 8.332740856 | | 2.330613166 | 0.012984762 | Up | 0.000172733 |
| RP5-1073O3.2 | 773 | 0.826293518 | 4.154912152 | | 2.330091719 | 0.035823997 | Up | 0.000727623 |
| GALNT8 | 2380 | 4.574570854 | 22.84244707 | | 2.320008899 | 0.007632716 | Up | 8.09E-05 |
| TPM3P8 | 643 | 0.457247257 | 2.270687751 | | 2.312082909 | 0.038712831 | Up | 0.000811419 |
| RP11-404F10.2 | 556 | 5.289263926 | 26.07213712 | | 2.301369974 | 0.001307535 | Up | 6.67E-06 |
| AC022182.1 | 653 | 2.310018623 | 11.37592019 | | 2.300006861 | 0.006599142 | Up | 6.46E-05 |
| RP11-126K1.6 | 618 | 2.100124294 | 10.32576033 | | 2.297701397 | 0.000208913 | Up | 5.74E-07 |
| RP11-564A8.4 | 611 | 10.86500711 | 53.14456838 | | 2.290233131 | 0.018271396 | Up | 0.000286534 |
| GPR174 | 1258 | 21.68957676 | 105.1319086 | | 2.277126802 | 0.001481664 | Up | 7.88E-06 |
| ANTXRLP1 | 430 | 2.510410426 | 12.15836504 | | 2.275954086 | 0.021563071 | Up | 0.000362408 |
| ITGAD | 3912 | 4.897357923 | 23.69216822 | | 2.274334691 | 0.011747048 | Up | 0.00014981 |
| INSM1 | 2826 | 3.048729737 | 14.71978517 | | 2.271476446 | 0.022441567 | Up | 0.000382519 |
| RP1-102G20.2 | 395 | 2.864355989 | 13.80514699 | | 2.268923539 | 0.005140208 | Up | 4.59E-05 |
| LINC00494 | 847 | 8.874977757 | 42.73330809 | | 2.267545595 | 0.00674149 | Up | 6.63E-05 |
| MIS18A-AS1 | 769 | 2.66213609 | 12.66284667 | | 2.249945537 | 0.008284045 | Up | 9.08E-05 |
| ABCB11 | 2822 | 1.593472373 | 7.571401606 | | 2.248386388 | 0.045109046 | Up | 0.001013785 |
| GPR15 | 1218 | 10.32331774 | 49.04225248 | | 2.24811854 | 0.021217779 | Up | 0.000354769 |
| TRAV26-1 | 537 | 0.956988312 | 4.540426487 | | 2.246254607 | 0.014812909 | Up | 0.000208587 |
| AE000661.37 | 1951 | 3.515420544 | 16.63545238 | | 2.242491907 | 0.015192434 | Up | 0.000216572 |
| TRBV29-1 | 402 | 4.145062657 | 19.59886874 | | 2.241304568 | 0.037381434 | Up | 0.000774786 |
| FAIM3 | 1109 | 120.8954796 | 571.5999108 | | 2.24124539 | 0.000537011 | Up | 1.94E-06 |
| KEL | 2812 | 8.999543948 | 42.4389502 | | 2.237465169 | 0.001508504 | Up | 8.09E-06 |
| AIRE | 3044 | 6.429789071 | 30.29787017 | | 2.236373065 | 0.02082266 | Up | 0.000343658 |
| THEMIS | 3862 | 43.27802345 | 203.3668968 | | 2.232378346 | 0.000154198 | Up | 3.64E-07 |
| PARP15 | 3744 | 109.9871283 | 516.5211977 | | 2.231492861 | 6.06E-06 | Up | 4.06E-09 |
| TESPA1 | 1312 | 35.30859521 | 164.640248 | | 2.221225732 | 0.001623689 | Up | 8.85E-06 |
| HNRNPA3P2 | 662 | 2.397350907 | 11.13318168 | | 2.215352949 | 0.028330423 | Up | 0.00052283 |
| LILRA4 | 1133 | 9.843820619 | 45.69557379 | | 2.214764155 | 0.004026151 | Up | 3.22E-05 |
| CXCL13 | 1203 | 112.0151951 | 519.334683 | | 2.212970126 | 0.003442406 | Up | 2.62E-05 |
| CCR7 | 2173 | 129.8585432 | 598.2007439 | | 2.203688775 | 0.001944958 | Up | 1.13E-05 |
| RP11-673E1.3 | 540 | 1.6780754 | 7.720537959 | | 2.201893835 | 0.016528228 | Up | 0.000246983 |
| CR1 | 6710 | 117.209404 | 537.8724276 | | 2.19817571 | 0.000308094 | Up | 9.33E-07 |
| RP11-960L18.1 | 605 | 4.569506781 | 20.85332196 | | 2.190166867 | 0.024382253 | Up | 0.000426465 |
| LTA | 1377 | 4.989640981 | 22.71900462 | | 2.186891709 | 0.004922031 | Up | 4.26E-05 |
| CP | 839 | 67.9602615 | 308.589883 | | 2.182927456 | 0.024524251 | Up | 0.000430213 |
| SP140 | 2727 | 66.55312251 | 301.8021646 | | 2.181024893 | 0.000145984 | Up | 3.35E-07 |
| CXorf65 | 863 | 2.740989195 | 12.3741598 | | 2.174562024 | 0.018678761 | Up | 0.000294539 |
| RPL13AP7 | 611 | 1.269977192 | 5.732203594 | | 2.174287264 | 0.032897074 | Up | 0.000651808 |
| CCL19 | 687 | 109.9875983 | 496.1518714 | | 2.173440934 | 0.015006504 | Up | 0.000212287 |
| SLAMF6 | 2712 | 47.54406947 | 214.4628809 | | 2.173390669 | 0.00128237 | Up | 6.38E-06 |
| CTB-114C7.4 | 2182 | 3.722637533 | 16.6983649 | | 2.165309785 | 0.004748292 | Up | 3.97E-05 |
| PLEKHG7 | 3358 | 6.433806257 | 28.82863478 | | 2.16375812 | 0.009950582 | Up | 0.000119456 |
| RP11-664D1.1 | 678 | 2.769819298 | 12.30396514 | | 2.151259558 | 0.002111649 | Up | 1.24E-05 |
| TNFRSF13B | 1242 | 34.60010617 | 153.2839363 | | 2.147358145 | 0.026984395 | Up | 0.000487962 |
| TXK | 1821 | 26.9913476 | 119.5734392 | | 2.147328047 | 0.002939277 | Up | 2.11E-05 |
| KLHL14 | 1884 | 15.48176414 | 68.15685427 | | 2.138288875 | 0.040302777 | Up | 0.000865452 |
| BFSP2-AS1 | 894 | 2.401397128 | 10.3921504 | | 2.113548303 | 0.036777927 | Up | 0.000752567 |
| F5 | 7024 | 36.19156009 | 156.0159127 | | 2.107967979 | 2.83E-05 | Up | 3.85E-08 |
| CCL21 | 852 | 224.7998523 | 965.7658831 | | 2.103032412 | 0.001256199 | Up | 6.01E-06 |
| HSPB1P2 | 546 | 1.138049179 | 4.87559124 | | 2.099014275 | 0.002618863 | Up | 1.76E-05 |
| P2RY10 | 1597 | 23.77031848 | 101.6751447 | | 2.096733905 | 0.002145092 | Up | 1.29E-05 |
| HS3ST2 | 2306 | 14.93402334 | 63.79611919 | | 2.094865774 | 0.003223217 | Up | 2.40E-05 |
| C1orf200 | 1823 | 19.77128712 | 84.44161764 | | 2.094547422 | 0.003442406 | Up | 2.62E-05 |
| CD3D | 396 | 35.1288188 | 149.9419472 | | 2.093677071 | 0.000415299 | Up | 1.37E-06 |
| RP11-284N8.3 | 3481 | 53.91445662 | 229.2284072 | | 2.088041767 | 0.000979235 | Up | 4.34E-06 |
| P2RX5 | 1836 | 193.6455859 | 821.7425456 | | 2.085267848 | 0.002957168 | Up | 2.16E-05 |
| TM4SF20 | 2296 | 1.188466105 | 5.016787806 | | 2.07766316 | 0.041864529 | Up | 0.000913148 |
| IKZF3 | 1709 | 123.5534394 | 520.3907668 | | 2.074460192 | 0.000253603 | Up | 7.35E-07 |
| LMOD3 | 4086 | 4.289136521 | 18.02196949 | | 2.070997539 | 0.006962838 | Up | 7.18E-05 |
| PRKCB | 1957 | 238.5714181 | 1001.930002 | | 2.070288603 | 0.00056388 | Up | 2.06E-06 |
| TRGV5 | 552 | 1.576755553 | 6.603122386 | | 2.066189371 | 0.030237343 | Up | 0.000577525 |
| TRBV20-1 | 413 | 8.331123482 | 34.72931279 | | 2.059570897 | 0.005785593 | Up | 5.41E-05 |
| CD180 | 2726 | 45.78750091 | 188.9937072 | | 2.045312469 | 9.05E-05 | Up | 1.72E-07 |
| MIR142 | 1625 | 212.0608976 | 870.6968612 | | 2.037691899 | 0.000254836 | Up | 7.50E-07 |
| ARHGAP15 | 1201 | 66.52015297 | 271.1830279 | | 2.027403498 | 9.45E-05 | Up | 1.84E-07 |
| TBC1D27 | 2662 | 46.97447102 | 191.0466535 | | 2.023976166 | 0.042456983 | Up | 0.000932142 |
| C12orf42 | 1072 | 4.637315886 | 18.67143158 | | 2.009470638 | 0.043235265 | Up | 0.000953905 |
| GPA33 | 2549 | 4.356806501 | 17.53092943 | | 2.00855954 | 0.007472229 | Up | 7.87E-05 |
| HVCN1 | 1175 | 50.21407729 | 202.051033 | | 2.008555948 | 0.000165679 | Up | 3.98E-07 |
| RP11-94L15.2 | 6116 | 345.3358972 | 1383.590658 | | 2.002344965 | 0.000152256 | Up | 3.56E-07 |
| SLC1A2 | 2481 | 26.73571016 | 106.5140437 | | 1.994203756 | 0.000358678 | Up | 1.16E-06 |
| RP11-455F5.5 | 520 | 19.00063965 | 75.22646823 | | 1.985192372 | 0.024243146 | Up | 0.000422398 |
| SIT1 | 1231 | 35.07190457 | 138.2534715 | | 1.978928024 | 0.009521049 | Up | 0.00011224 |
| CTD-3105H18.13 | 1990 | 4.634222262 | 18.18705582 | | 1.97251287 | 0.016860268 | Up | 0.000254923 |
| NAPSB | 957 | 79.03823244 | 309.9014259 | | 1.971186804 | 0.000428337 | Up | 1.45E-06 |
| SCML4 | 2636 | 71.32652388 | 279.1485746 | | 1.968522619 | 0.001260066 | Up | 6.05E-06 |
| LINC00877 | 1229 | 7.15217344 | 27.9763378 | | 1.967753493 | 0.005741849 | Up | 5.33E-05 |
| TIFAB | 5923 | 35.73756188 | 139.5911877 | | 1.96569475 | 0.011747048 | Up | 0.000149916 |
| IRF8 | 1403 | 300.6570571 | 1167.394676 | | 1.957101666 | 1.77E-06 | Up | 7.28E-10 |
| CTD-2024I7.1 | 208 | 2.575720067 | 9.972612982 | | 1.952995755 | 0.035874156 | Up | 0.000729418 |
| CLNK | 1903 | 15.46322696 | 59.85812996 | | 1.952705787 | 0.000559713 | Up | 2.03E-06 |
| RP3-455J7.4 | 2523 | 18.14706301 | 70.03090787 | | 1.948255714 | 0.00502514 | Up | 4.40E-05 |
| NUGGC | 3885 | 29.86214412 | 114.2560325 | | 1.935880677 | 0.014027494 | Up | 0.000192369 |
| CD3G | 2678 | 59.29337848 | 226.8561643 | | 1.935834955 | 0.000243692 | Up | 6.96E-07 |
| NCF1C | 1075 | 38.28169315 | 146.4231095 | | 1.935416723 | 0.009163033 | Up | 0.000106434 |
| GIMAP7 | 1256 | 49.2860205 | 188.449917 | | 1.934930756 | 0.000166522 | Up | 4.07E-07 |
| SH2D1A | 2450 | 28.83846385 | 109.98421 | | 1.931230193 | 0.001797942 | Up | 1.01E-05 |
| KLRG1 | 1058 | 22.88785016 | 87.15161054 | | 1.928945367 | 0.002300762 | Up | 1.49E-05 |
| APOA2 | 723 | 2.219445605 | 8.371715942 | | 1.91532401 | 0.039638299 | Up | 0.000841106 |
| CD79A | 1093 | 246.6177225 | 929.2584163 | | 1.913803371 | 0.045607719 | Up | 0.001028938 |
| FUT7 | 2584 | 26.39314649 | 99.40368536 | | 1.913135987 | 0.006962838 | Up | 7.18E-05 |
| RN7SL437P | 291 | 6.028013665 | 22.6731473 | | 1.911230075 | 0.033534657 | Up | 0.000671808 |
| RP11-731J8.1 | 505 | 3.215395044 | 12.06048364 | | 1.907219859 | 0.013389226 | Up | 0.000179562 |
| CCDC141 | 3591 | 81.0244733 | 303.4412919 | | 1.904987777 | 0.006820874 | Up | 6.79E-05 |
| RHOH | 1029 | 142.4034274 | 533.1432051 | | 1.904539231 | 0.000115553 | Up | 2.47E-07 |
| RP11-1094M14.8 | 958 | 28.62137189 | 107.0093643 | | 1.902572321 | 0.000390583 | Up | 1.28E-06 |
| CD96 | 3111 | 72.80030991 | 272.1013872 | | 1.902127814 | 0.004748687 | Up | 3.99E-05 |
| TNFRSF17 | 668 | 12.20846699 | 45.56312735 | | 1.899984721 | 0.001630852 | Up | 8.92E-06 |
| PVRIG | 1583 | 48.15662232 | 179.2887671 | | 1.896478992 | 0.043176804 | Up | 0.000951681 |
| PTPRC | 2108 | 612.3763198 | 2275.343999 | | 1.893594275 | 2.15E-06 | Up | 9.32E-10 |
| TMEM156 | 1318 | 16.22720404 | 60.25596524 | | 1.892689629 | 0.016927869 | Up | 0.000256311 |
| CEACAM21 | 504 | 13.66705516 | 50.71295269 | | 1.891651857 | 0.016639339 | Up | 0.000249423 |
| AC006129.2 | 656 | 10.46667441 | 38.80603262 | | 1.890477819 | 0.047399608 | Up | 0.001089088 |
| RP11-1094M14.5 | 849 | 18.10179331 | 66.91269682 | | 1.886147362 | 0.002664302 | Up | 1.80E-05 |
| RP11-147L13.8 | 2919 | 17.48059028 | 64.36208189 | | 1.880457091 | 0.000115446 | Up | 2.42E-07 |
| RN7SL243P | 330 | 3.377956154 | 12.41279888 | | 1.877605948 | 0.008607832 | Up | 9.63E-05 |
| PTCRA | 799 | 3.802918115 | 13.96472457 | | 1.87660834 | 0.011962401 | Up | 0.000153699 |
| ADAM28 | 2137 | 192.3308761 | 702.3877517 | | 1.868677301 | 0.00042537 | Up | 1.43E-06 |
| CD28 | 4462 | 86.30566727 | 314.9188505 | | 1.867452915 | 0.002194975 | Up | 1.32E-05 |
| LINC00487 | 2131 | 4.263421928 | 15.44755086 | | 1.85729438 | 0.01137678 | Up | 0.000142976 |
| LINC00243 | 2052 | 6.900128739 | 24.92617806 | | 1.852966507 | 0.031864462 | Up | 0.000625145 |
| GRIN2B | 27217 | 17.27102133 | 62.36480182 | | 1.852378614 | 0.012530674 | Up | 0.000163704 |
| GVINP1 | 6825 | 242.3692033 | 875.1183565 | | 1.852271755 | 0.002761242 | Up | 1.89E-05 |
| RP11-568N6.1 | 1907 | 5.715036378 | 20.54480406 | | 1.845938985 | 0.013907204 | Up | 0.000190118 |
| AC096579.13 | 1421 | 8.603870895 | 30.91802629 | | 1.845390443 | 0.026420651 | Up | 0.000475481 |
| IRF4 | 4686 | 199.7135265 | 716.1759427 | | 1.842382008 | 0.000233889 | Up | 6.63E-07 |
| LY9 | 2099 | 71.59541903 | 256.2002228 | | 1.839332544 | 0.006365597 | Up | 6.11E-05 |
| PZP | 1278 | 14.22420959 | 50.55146808 | | 1.829404503 | 0.002261822 | Up | 1.45E-05 |
| RP11-730A19.5 | 793 | 3.39790738 | 12.07175511 | | 1.828917011 | 0.024843396 | Up | 0.000437961 |
| SLC12A3 | 3119 | 6.848599788 | 24.30799104 | | 1.827549704 | 0.03221348 | Up | 0.000635476 |
| RP11-830F9.6 | 1812 | 19.06563586 | 67.26051118 | | 1.818785096 | 0.005784478 | Up | 5.38E-05 |
| ATP8A1 | 6474 | 197.416247 | 696.4348454 | | 1.818747662 | 0.000253099 | Up | 7.28E-07 |
| SCML2P2 | 540 | 7.806621895 | 27.25308392 | | 1.803649191 | 0.006364626 | Up | 6.10E-05 |
| CD52 | 468 | 75.13270157 | 261.6249066 | | 1.799987008 | 0.000818159 | Up | 3.47E-06 |
| P2RY12 | 1766 | 8.343547241 | 29.02112502 | | 1.79837067 | 0.031137901 | Up | 0.000602135 |
| CTGLF10P | 2418 | 5.502624242 | 19.08015253 | | 1.793880985 | 0.037381434 | Up | 0.00077614 |
| WDFY4 | 5942 | 849.7120576 | 2944.859915 | | 1.793153066 | 7.19E-05 | Up | 1.35E-07 |
| HLA-DOB | 1461 | 71.86093907 | 248.9275014 | | 1.792445934 | 0.002792353 | Up | 1.93E-05 |
| SCIMP | 2313 | 111.4866956 | 385.8503117 | | 1.791169717 | 4.52E-05 | Up | 7.02E-08 |
| NCF1B | 1455 | 40.99128183 | 141.8607733 | | 1.791086707 | 0.004767015 | Up | 4.01E-05 |
| PNMA3 | 3158 | 15.78279422 | 54.53085741 | | 1.788720194 | 0.028534432 | Up | 0.000531097 |
| AC090627.1 | 751 | 19.17201235 | 66.05328159 | | 1.784628463 | 0.011565715 | Up | 0.000146101 |
| DNAJC5B | 1318 | 4.505382435 | 15.51561193 | | 1.783999119 | 0.036925399 | Up | 0.000759578 |
| RP11-460N11.2 | 3275 | 29.24034503 | 100.3461116 | | 1.778952474 | 0.019900927 | Up | 0.000319407 |
| IKZF1 | 2946 | 654.1197193 | 2242.930805 | | 1.777758502 | 5.72E-05 | Up | 9.66E-08 |
| ABCD2 | 6238 | 20.9066282 | 71.55281655 | | 1.775048153 | 0.008711703 | Up | 9.87E-05 |
| CENPCP1 | 2578 | 9.909354029 | 33.87118666 | | 1.773195613 | 0.002946494 | Up | 2.13E-05 |
| CHIT1 | 5607 | 72.10052686 | 245.6247739 | | 1.768374372 | 0.015364188 | Up | 0.00022136 |
| PYHIN1 | 1490 | 22.49730577 | 76.53400438 | | 1.766348648 | 0.015192434 | Up | 0.0002171 |
| RASGRP2 | 1856 | 355.9533883 | 1210.460945 | | 1.765796293 | 0.010554285 | Up | 0.000129443 |
| BTK | 1991 | 69.03097617 | 234.6888564 | | 1.76543355 | 0.002252811 | Up | 1.41E-05 |
| CLECL1 | 1069 | 6.870625972 | 23.30410993 | | 1.76207096 | 0.028279096 | Up | 0.000520549 |
| CD69 | 1201 | 53.18400319 | 180.1722684 | | 1.760312694 | 0.004570902 | Up | 3.76E-05 |
| P2RY8 | 2264 | 186.30131 | 629.4375077 | | 1.756425331 | 0.003671862 | Up | 2.88E-05 |
| RP11-876N24.2 | 575 | 13.32503082 | 44.97984664 | | 1.755139872 | 0.013530627 | Up | 0.000182629 |
| PLAC8 | 1294 | 145.4471671 | 490.6499101 | | 1.754198798 | 0.029921264 | Up | 0.000567964 |
| KIAA0226L | 959 | 82.60071943 | 278.4731466 | | 1.753311962 | 0.000511139 | Up | 1.81E-06 |
| IPCEF1 | 4617 | 95.77799196 | 322.4806175 | | 1.751446356 | 0.000171069 | Up | 4.33E-07 |
| CTD-2587H19.1 | 1904 | 12.06636786 | 40.42002793 | | 1.744078848 | 0.002957168 | Up | 2.15E-05 |
| CCR9 | 2512 | 4.121902088 | 13.80419994 | | 1.743725135 | 0.040321316 | Up | 0.000868682 |
| LINC00926 | 2437 | 145.7881437 | 486.8861666 | | 1.739711115 | 0.008179665 | Up | 8.89E-05 |
| USP30-AS1 | 656 | 11.81911616 | 39.25011133 | | 1.731574591 | 0.000759477 | Up | 3.02E-06 |
| CORO1A | 1217 | 961.7643487 | 3188.046908 | | 1.728917503 | 2.22E-05 | Up | 2.79E-08 |
| ITGAL | 1412 | 453.7778226 | 1497.171012 | | 1.722181013 | 4.52E-05 | Up | 7.05E-08 |
| MAP4K1 | 842 | 167.1778924 | 550.7847348 | | 1.720104497 | 0.002468086 | Up | 1.63E-05 |
| NDUFB4P1 | 381 | 3.237264801 | 10.65144754 | | 1.718202223 | 0.022283154 | Up | 0.000378849 |
| MIR155HG | 1600 | 35.52152828 | 116.653088 | | 1.71545894 | 0.000586189 | Up | 2.17E-06 |
| FAM26F | 1109 | 53.38356948 | 175.2500644 | | 1.714947295 | 0.008284045 | Up | 9.07E-05 |
| SASH3 | 1720 | 282.8932891 | 921.4428834 | | 1.703636789 | 1.11E-06 | Up | 3.83E-10 |
| RN7SL213P | 315 | 4.502999124 | 14.65475866 | | 1.702411108 | 0.02898491 | Up | 0.000542946 |
| CD3E | 1483 | 158.9352844 | 517.2131689 | | 1.702319563 | 0.001877596 | Up | 1.08E-05 |
| ITGB2-AS1 | 2282 | 61.22309351 | 199.0457797 | | 1.700952433 | 0.011088408 | Up | 0.000138872 |
| CTC-548K16.6 | 889 | 10.0331975 | 32.55427696 | | 1.698065641 | 0.006503551 | Up | 6.32E-05 |
| S1PR4 | 1594 | 97.33986734 | 314.3324373 | | 1.691188444 | 0.009875173 | Up | 0.000117385 |
| BIRC3 | 4994 | 716.2520771 | 2303.028677 | | 1.684993053 | 0.002534153 | Up | 1.69E-05 |
| DNAJB13 | 1779 | 7.670865659 | 24.60438823 | | 1.681454345 | 0.043538715 | Up | 0.000967312 |
| TRABD2A | 2201 | 54.00013282 | 173.09454 | | 1.680525357 | 0.002220854 | Up | 1.36E-05 |
| RP11-95M15.2 | 1779 | 7.197546449 | 23.03706141 | | 1.6783796 | 0.020627755 | Up | 0.000336873 |
| CD53 | 1418 | 363.3164495 | 1157.535647 | | 1.671758031 | 1.64E-06 | Up | 6.11E-10 |
| AC006160.5 | 750 | 4.536139659 | 14.41021584 | | 1.667554981 | 0.02079244 | Up | 0.000341811 |
| KLRB1 | 1448 | 15.97810748 | 50.64319568 | | 1.664271905 | 0.024916969 | Up | 0.000441414 |
| ZNF80 | 2555 | 11.22477296 | 35.56744023 | | 1.663870883 | 0.029921264 | Up | 0.000568252 |
| GZMM | 940 | 16.29512324 | 51.54815635 | | 1.661480567 | 0.032667464 | Up | 0.000646552 |
| ADRA2A | 3869 | 178.538788 | 562.735687 | | 1.65621992 | 6.99E-05 | Up | 1.30E-07 |
| ANKRD44-IT1 | 1069 | 25.99445426 | 81.86242524 | | 1.654997542 | 0.019542937 | Up | 0.000312213 |
| RP11-1094M14.9 | 1230 | 28.34456702 | 89.12156838 | | 1.65270239 | 0.00886166 | Up | 0.0001014 |
| LY86 | 1197 | 22.12377813 | 69.40038985 | | 1.649345988 | 0.000767443 | Up | 3.11E-06 |
| FXYD7 | 717 | 7.768458744 | 24.30467893 | | 1.645533774 | 0.04284071 | Up | 0.00094242 |
| KBTBD8 | 2684 | 58.3425159 | 182.4447627 | | 1.644840231 | 3.77E-06 | Up | 2.12E-09 |
| KMO | 4888 | 23.16116729 | 72.41195555 | | 1.644519948 | 0.016366433 | Up | 0.0002425 |
| CD72 | 831 | 49.87768571 | 155.8462303 | | 1.643656827 | 0.001806907 | Up | 1.02E-05 |
| EOMES | 2958 | 43.62476361 | 136.1864795 | | 1.642364262 | 0.016144695 | Up | 0.00023698 |
| RP13-93L13.1 | 456 | 8.4445769 | 26.33819666 | | 1.641059524 | 0.032658943 | Up | 0.000645677 |
| DTHD1 | 2606 | 30.81277401 | 95.49728841 | | 1.631931198 | 0.008819259 | Up | 0.000100152 |
| ERP27 | 1475 | 8.723380525 | 26.95107178 | | 1.627383418 | 0.039362477 | Up | 0.000830145 |
| RP11-61K9.2 | 444 | 3.311241785 | 10.21617612 | | 1.625411037 | 0.039847453 | Up | 0.000847268 |
| ZAP70 | 2077 | 267.4637944 | 823.2478983 | | 1.621983311 | 0.016040951 | Up | 0.000233926 |
| GIMAP4 | 1975 | 117.4501382 | 360.1666205 | | 1.61661607 | 0.00243775 | Up | 1.60E-05 |
| P2RY13 | 2764 | 32.69440813 | 99.99181311 | | 1.612766072 | 0.004922031 | Up | 4.25E-05 |
| ZMAT1 | 4711 | 96.33522405 | 293.7284379 | | 1.608347643 | 0.001025748 | Up | 4.68E-06 |
| RAB37 | 1432 | 49.34873279 | 150.4327473 | | 1.608033714 | 0.023376038 | Up | 0.000404003 |
| HLA-DQA1 | 1341 | 506.4776951 | 1543.065688 | | 1.607228838 | 0.016283596 | Up | 0.000240216 |
| UBASH3A | 2754 | 29.58279659 | 90.12725322 | | 1.60720498 | 0.009270432 | Up | 0.000108684 |
| POU2AF1 | 1802 | 608.9101785 | 1855.007187 | | 1.607123442 | 0.018943966 | Up | 0.000300769 |
| CD244 | 2463 | 16.42044147 | 49.88819611 | | 1.60320559 | 0.006364626 | Up | 6.10E-05 |
| RLTPR | 1688 | 146.9210188 | 445.2869 | | 1.599694364 | 0.008255091 | Up | 9.02E-05 |
| GPR55 | 3828 | 15.59045531 | 47.21694719 | | 1.598641705 | 0.028330423 | Up | 0.000523751 |
| PTPRCAP | 1292 | 337.8604074 | 1023.083588 | | 1.59842482 | 0.003671862 | Up | 2.87E-05 |
| POU2F2 | 4058 | 253.2062927 | 766.2036036 | | 1.597414552 | 0.0019299 | Up | 1.11E-05 |
| CYTIP | 1844 | 218.7531521 | 660.3540379 | | 1.593935903 | 0.00132072 | Up | 6.77E-06 |
| TNFSF8 | 1179 | 41.47323189 | 125.0827991 | | 1.592631028 | 0.005167444 | Up | 4.65E-05 |
| KCNA3 | 3346 | 158.7026332 | 477.969692 | | 1.590593074 | 0.017987342 | Up | 0.000278578 |
| TRBC2 | 758 | 204.1788107 | 614.1504975 | | 1.588759077 | 0.020253616 | Up | 0.000328562 |
| IL2RG | 1578 | 181.3515342 | 545.216702 | | 1.588040808 | 0.002877047 | Up | 2.05E-05 |
| LCK | 2117 | 130.5240284 | 391.7676806 | | 1.585682965 | 0.002534153 | Up | 1.69E-05 |
| RP3-508I15.22 | 416 | 4.77285137 | 14.25850192 | | 1.578899097 | 0.021703842 | Up | 0.000365244 |
| KCNJ10 | 5293 | 18.85360019 | 55.93328735 | | 1.568867084 | 0.00921781 | Up | 0.000107668 |
| EMB | 4058 | 299.2187252 | 886.3791064 | | 1.566723412 | 5.87E-05 | Up | 1.00E-07 |
| PTPN2P1 | 1072 | 23.42851426 | 69.37047324 | | 1.566056259 | 0.014573839 | Up | 0.000203645 |
| ATP5G2P3 | 425 | 21.06174414 | 62.24814379 | | 1.563405905 | 0.046859698 | Up | 0.001068333 |
| AC007228.11 | 2045 | 6.380157511 | 18.84857249 | | 1.562791318 | 0.044737466 | Up | 0.001002646 |
| RAB33A | 1258 | 8.662954294 | 25.55915389 | | 1.560909068 | 0.00946905 | Up | 0.000111422 |
| TLR9 | 3978 | 49.69418757 | 146.2915793 | | 1.557697705 | 0.027676637 | Up | 0.000505269 |
| GRAP2 | 1620 | 114.8601484 | 338.0418351 | | 1.557323471 | 0.004858085 | Up | 4.13E-05 |
| RP3-323N1.2 | 430 | 19.52552409 | 57.33246228 | | 1.553990967 | 0.021130629 | Up | 0.000352398 |
| CD37 | 1108 | 508.8877175 | 1493.887327 | | 1.553652064 | 0.00200755 | Up | 1.18E-05 |
| ZNF831 | 9404 | 227.4546177 | 665.9669109 | | 1.549871774 | 0.030947985 | Up | 0.00059604 |
| GIMAP5 | 1671 | 280.9826654 | 822.5913107 | | 1.549694703 | 0.000157536 | Up | 3.75E-07 |
| NCKAP1L | 3780 | 475.0868348 | 1387.772098 | | 1.546507532 | 9.20E-06 | Up | 7.36E-09 |
| SLC24A4 | 3569 | 24.19850781 | 70.44590434 | | 1.541597745 | 0.040613897 | Up | 0.000880016 |
| CHI3L2 | 629 | 45.84387104 | 133.3152011 | | 1.540040516 | 0.002129846 | Up | 1.26E-05 |
| C1orf228 | 757 | 19.43793881 | 56.35938561 | | 1.53578064 | 0.018789812 | Up | 0.000296696 |
| GIMAP1 | 4420 | 95.8512325 | 277.7009851 | | 1.534663407 | 0.004570902 | Up | 3.78E-05 |
| RP11-571M6.8 | 874 | 40.37555558 | 116.6143198 | | 1.53019094 | 0.002917915 | Up | 2.08E-05 |
| PPP1R16B | 6251 | 474.414976 | 1366.435982 | | 1.526196413 | 0.001944958 | Up | 1.12E-05 |
| CYP1B1 | 5073 | 704.1126135 | 2023.39692 | | 1.522901262 | 0.002220854 | Up | 1.36E-05 |
| SLAMF1 | 1268 | 59.25547518 | 170.2606874 | | 1.522724991 | 0.021388449 | Up | 0.000358548 |
| RP11-286E11.2 | 3147 | 8.593114893 | 24.42181641 | | 1.506917417 | 0.046803427 | Up | 0.001066038 |
| TLR7 | 5011 | 46.7473468 | 132.8182011 | | 1.506496473 | 0.004886641 | Up | 4.19E-05 |
| SULT1B1 | 7115 | 54.61596249 | 154.9535927 | | 1.504441634 | 0.021246218 | Up | 0.000355704 |
| ST8SIA4 | 3638 | 163.7085467 | 464.3604604 | | 1.504115492 | 6.12E-05 | Up | 1.06E-07 |
| SNX20 | 2893 | 137.542836 | 389.9687693 | | 1.503477593 | 0.002129846 | Up | 1.27E-05 |
| RP11-496H15.2 | 337 | 4.486942652 | 12.6908624 | | 1.49998546 | 0.048295828 | Up | 0.001121896 |
| RP4-647J21.1 | 1911 | 29.0055428 | 81.97398959 | | 1.498837596 | 0.012997483 | Up | 0.000173184 |
| ARHGAP25 | 1433 | 181.6240579 | 511.8939795 | | 1.494889724 | 1.55E-05 | Up | 1.54E-08 |
| ST6GAL1 | 2191 | 1093.85136 | 3082.570675 | | 1.494717264 | 0.000908836 | Up | 3.99E-06 |
| GZMA | 894 | 33.73239799 | 94.93429638 | | 1.492794494 | 0.038998322 | Up | 0.000821621 |
| CYFIP2 | 1846 | 401.658761 | 1129.234486 | | 1.491302844 | 0.001271063 | Up | 6.22E-06 |
| ITK | 3746 | 201.2492619 | 565.4316161 | | 1.490369061 | 0.015322545 | Up | 0.000220072 |
| MCOLN2 | 1816 | 63.0038409 | 176.5683525 | | 1.486715095 | 0.00625597 | Up | 5.95E-05 |
| CD2 | 1609 | 72.81586237 | 203.8995402 | | 1.485533853 | 0.017915806 | Up | 0.000277082 |
| PLEK | 2496 | 247.1909984 | 691.4937792 | | 1.484090065 | 1.98E-05 | Up | 2.33E-08 |
| ERMN | 3760 | 23.83093745 | 66.45762482 | | 1.479599028 | 0.033454706 | Up | 0.000667941 |
| LINC01059 | 2471 | 9.846403112 | 27.44020664 | | 1.478622636 | 0.042526946 | Up | 0.000934598 |
| ANKRD44 | 1604 | 434.3281818 | 1209.508064 | | 1.477562916 | 0.00040224 | Up | 1.32E-06 |
| DOCK2 | 3449 | 555.9460529 | 1545.213483 | | 1.474789371 | 0.000108605 | Up | 2.24E-07 |
| ADAMDEC1 | 1531 | 110.0599134 | 304.8387261 | | 1.469757093 | 0.004744708 | Up | 3.96E-05 |
| RP13-93L13.2 | 1197 | 50.4605055 | 139.6648877 | | 1.468742803 | 0.002830794 | Up | 1.99E-05 |
| EAF2 | 898 | 47.4849777 | 131.3985345 | | 1.468406104 | 0.005866333 | Up | 5.52E-05 |
| SIGLEC5 | 2359 | 21.4336289 | 59.29342526 | | 1.467996009 | 0.043538715 | Up | 0.00096692 |
| 1-Sep | 1068 | 231.1360883 | 639.0968285 | | 1.467291996 | 0.002237675 | Up | 1.39E-05 |
| INPP5D | 1776 | 563.8717854 | 1547.208264 | | 1.456228344 | 0.000845542 | Up | 3.68E-06 |
| RASGRP3 | 1829 | 184.3879066 | 505.1396028 | | 1.453938115 | 0.000102055 | Up | 2.03E-07 |
| GPR114 | 1646 | 132.3884044 | 362.5478955 | | 1.453394834 | 0.02063198 | Up | 0.000337388 |
| HLA-DMB | 1602 | 433.1297447 | 1182.910607 | | 1.449469897 | 5.42E-05 | Up | 9.03E-08 |
| IL21R | 2247 | 180.9354906 | 492.3681651 | | 1.444262064 | 0.009940106 | Up | 0.0001189 |
| TRAC | 974 | 106.7088221 | 288.8040831 | | 1.436411685 | 0.006736145 | Up | 6.62E-05 |
| UCP2 | 1207 | 588.5277671 | 1591.735782 | | 1.435418489 | 3.82E-05 | Up | 5.68E-08 |
| GMFG | 547 | 78.50474048 | 212.2898095 | | 1.435183441 | 0.00505217 | Up | 4.46E-05 |
| TAGAP | 3688 | 220.2381398 | 595.0082589 | | 1.433845364 | 0.002211727 | Up | 1.34E-05 |
| KLHL6 | 5388 | 692.5397795 | 1869.801544 | | 1.432916307 | 0.000264221 | Up | 7.83E-07 |
| SPN | 3519 | 391.1194742 | 1052.775089 | | 1.428515981 | 0.000143125 | Up | 3.22E-07 |
| RP5-837J1.4 | 544 | 31.57473491 | 84.87259376 | | 1.426528146 | 0.026045799 | Up | 0.000466482 |
| BIN2 | 1937 | 92.16987905 | 246.9036475 | | 1.421580887 | 0.001696573 | Up | 9.39E-06 |
| LCP1 | 2353 | 1303.735367 | 3488.11196 | | 1.419795288 | 0.00049459 | Up | 1.72E-06 |
| RCSD1 | 2967 | 285.8705433 | 764.2523627 | | 1.418687138 | 0.004774295 | Up | 4.04E-05 |
| APBB1IP | 2771 | 126.8575312 | 337.7698222 | | 1.412831266 | 0.00886166 | Up | 0.000101341 |
| CXCR4 | 1663 | 799.2875145 | 2121.798896 | | 1.408501466 | 0.008620678 | Up | 9.66E-05 |
| PIK3AP1 | 3963 | 373.3536419 | 990.4991455 | | 1.407612926 | 2.71E-06 | Up | 1.29E-09 |
| ATP2A3 | 2346 | 1101.078756 | 2914.310795 | | 1.404237078 | 0.008175644 | Up | 8.84E-05 |
| SPOCK2 | 4842 | 833.1488789 | 2203.292831 | | 1.403015026 | 0.005143755 | Up | 4.61E-05 |
| DOCK8 | 2725 | 875.2402525 | 2308.409401 | | 1.399148117 | 0.000206602 | Up | 5.63E-07 |
| PIK3CG | 4233 | 233.3889268 | 615.4256917 | | 1.398848558 | 0.007253471 | Up | 7.59E-05 |
| MYBL1 | 3659 | 81.40470067 | 214.5030402 | | 1.397814086 | 0.003456518 | Up | 2.64E-05 |
| CXCR3 | 1650 | 65.14324784 | 170.823493 | | 1.390818844 | 0.043748838 | Up | 0.000975645 |
| IL12RB1 | 1788 | 125.2550104 | 327.8689128 | | 1.388250803 | 0.004971337 | Up | 4.31E-05 |
| RP3-325F22.5 | 1894 | 11.6890253 | 30.52077554 | | 1.384636987 | 0.036776851 | Up | 0.000751749 |
| C11orf65 | 2419 | 30.53732021 | 79.54214048 | | 1.381145921 | 0.000804611 | Up | 3.36E-06 |
| CCR4 | 3095 | 80.37056068 | 209.3327088 | | 1.381058703 | 0.021717259 | Up | 0.000365939 |
| RBBP8P1 | 1844 | 15.7898965 | 41.12536189 | | 1.38102666 | 0.012153768 | Up | 0.000156421 |
| CLEC2D | 873 | 204.3503522 | 531.7409861 | | 1.379678943 | 0.000966753 | Up | 4.27E-06 |
| PTPN7 | 1475 | 279.3405026 | 725.8561345 | | 1.377658862 | 0.001444523 | Up | 7.56E-06 |
| ARHGAP9 | 1126 | 241.7349427 | 626.4282219 | | 1.373723181 | 0.025142757 | Up | 0.000446501 |
| CTB-109A12.1 | 1992 | 28.61019141 | 74.13373421 | | 1.373601034 | 0.04560921 | Up | 0.001029959 |
| JAK3 | 4095 | 953.1330562 | 2462.818913 | | 1.369561021 | 0.001140678 | Up | 5.33E-06 |
| IL7R | 3106 | 889.5040109 | 2296.360577 | | 1.368276177 | 0.001148282 | Up | 5.39E-06 |
| VAV1 | 2107 | 207.2996744 | 534.2254036 | | 1.365730731 | 0.000464739 | Up | 1.60E-06 |
| FSD2 | 6418 | 17.32069331 | 44.55896237 | | 1.363218958 | 0.048207173 | Up | 0.001116869 |
| LYL1 | 1502 | 96.35820174 | 247.8613379 | | 1.363053878 | 0.012585016 | Up | 0.000165416 |
| RNASE6 | 1061 | 51.5532346 | 132.3670014 | | 1.360408654 | 0.040302777 | Up | 0.000867411 |
| BCL2A1 | 838 | 28.02744158 | 71.88406238 | | 1.358831885 | 0.030754754 | Up | 0.000589403 |
| CD247 | 1788 | 130.2727712 | 333.6159468 | | 1.356652679 | 0.002810458 | Up | 1.96E-05 |
| TMC8 | 1892 | 872.5202717 | 2228.874752 | | 1.353054993 | 0.009998465 | Up | 0.000120247 |
| TLR1 | 1516 | 103.5835654 | 264.4931285 | | 1.352435119 | 0.006886258 | Up | 6.91E-05 |
| EMR4P | 2732 | 36.44372984 | 93.04600627 | | 1.352273608 | 0.009209859 | Up | 0.000107177 |
| CIITA | 3974 | 1738.280191 | 4428.204838 | | 1.349061313 | 0.003965467 | Up | 3.13E-05 |
| RASAL3 | 3270 | 344.9308488 | 878.1729741 | | 1.348197974 | 0.014547775 | Up | 0.000202283 |
| LPXN | 1588 | 184.7271942 | 469.3845215 | | 1.345374003 | 0.000201017 | Up | 5.33E-07 |
| RGS13 | 1840 | 47.52020692 | 120.1618026 | | 1.338365337 | 0.022686674 | Up | 0.000390221 |
| ANGPTL1 | 2307 | 87.28544528 | 220.5640154 | | 1.337384425 | 0.008659602 | Up | 9.74E-05 |
| NCF1 | 1350 | 116.0645169 | 291.9610533 | | 1.33084895 | 0.031193353 | Up | 0.000605223 |
| SLFN12L | 2094 | 121.7067463 | 305.7245325 | | 1.328823184 | 0.001873799 | Up | 1.07E-05 |
| GRAP | 2478 | 126.317939 | 316.3196765 | | 1.324323763 | 0.028330423 | Up | 0.000523945 |
| TIGIT | 1548 | 138.5061417 | 345.9670155 | | 1.320684547 | 0.019211116 | Up | 0.000305842 |
| KCNK13 | 2522 | 25.66560867 | 63.97464708 | | 1.317663808 | 0.008438583 | Up | 9.34E-05 |
| FAM196B | 2999 | 91.05105985 | 226.8843468 | | 1.317209362 | 0.005140208 | Up | 4.58E-05 |
| LILRB1 | 2646 | 181.9553049 | 452.1880168 | | 1.313338647 | 0.002782519 | Up | 1.92E-05 |
| MGAT3 | 5062 | 146.1196959 | 362.8778667 | | 1.312333408 | 0.04548486 | Up | 0.001024199 |
| CD6 | 3046 | 291.9121247 | 724.6561917 | | 1.311762545 | 0.043805531 | Up | 0.000977857 |
| AIF1 | 607 | 57.75517227 | 143.3354671 | | 1.311373578 | 0.004010207 | Up | 3.18E-05 |
| CCR5 | 2093 | 172.4318597 | 426.441234 | | 1.306320583 | 0.003542986 | Up | 2.73E-05 |
| CD80 | 1479 | 53.61195456 | 132.5127252 | | 1.30550427 | 0.00400628 | Up | 3.17E-05 |
| ZC3H12D | 2150 | 311.3117543 | 769.3884136 | | 1.305352054 | 0.024734324 | Up | 0.000434433 |
| PPM1K | 3638 | 500.110489 | 1233.565309 | | 1.30251533 | 0.000165679 | Up | 4.01E-07 |
| RABGAP1L-IT1 | 737 | 18.4162711 | 45.34736455 | | 1.300037732 | 0.043538715 | Up | 0.000968134 |
| MEF2C | 2699 | 236.4059924 | 582.1003338 | | 1.30000124 | 0.002782519 | Up | 1.91E-05 |
| LRRC69 | 540 | 18.9383548 | 46.60886518 | | 1.299293379 | 0.047677389 | Up | 0.00109832 |
| DOCK10 | 2847 | 431.1370237 | 1053.522148 | | 1.289002281 | 1.98E-05 | Up | 2.40E-08 |
| BLNK | 625 | 93.78962709 | 228.8900286 | | 1.287154336 | 0.01081697 | Up | 0.000134771 |
| PLCG2 | 1637 | 488.724562 | 1187.885503 | | 1.281302268 | 6.99E-05 | Up | 1.30E-07 |
| MUC6 | 8006 | 92.43107835 | 224.1755897 | | 1.278179273 | 0.011705169 | Up | 0.000148115 |
| CCL22 | 2929 | 189.7488181 | 460.0186837 | | 1.277601559 | 0.048834203 | Up | 0.001139961 |
| HLA-DOA | 2005 | 305.2544865 | 739.5807117 | | 1.2766951 | 0.014274009 | Up | 0.000197294 |
| ICOS | 2645 | 74.03852108 | 179.3195594 | | 1.276184877 | 0.034311851 | Up | 0.000691714 |
| SEL1L3 | 1925 | 921.02743 | 2228.708144 | | 1.274891676 | 3.82E-05 | Up | 5.58E-08 |
| HLA-DRA | 1273 | 2637.738122 | 6370.976964 | | 1.272213282 | 0.000446074 | Up | 1.52E-06 |
| GPSM3 | 1468 | 280.2093935 | 676.3667098 | | 1.271300333 | 0.000358678 | Up | 1.15E-06 |
| PATL2 | 1399 | 64.87692454 | 156.5770497 | | 1.271095429 | 0.028534432 | Up | 0.000531206 |
| CECR1 | 2924 | 626.290212 | 1509.255168 | | 1.268933504 | 0.003416603 | Up | 2.58E-05 |
| TBC1D10C | 1247 | 511.7241061 | 1227.288333 | | 1.262036126 | 0.007876061 | Up | 8.44E-05 |
| RP11-415F23.2 | 549 | 26.56517548 | 63.66992018 | | 1.261075713 | 0.013639812 | Up | 0.000185098 |
| DTX1 | 1817 | 179.3115026 | 429.1713299 | | 1.259085665 | 0.00619137 | Up | 5.87E-05 |
| RP11-347P5.1 | 2065 | 217.5879601 | 520.3873224 | | 1.257987086 | 0.01174296 | Up | 0.000149102 |
| RP11-430C7.5 | 2219 | 41.80827946 | 99.90010057 | | 1.256697457 | 0.041864529 | Up | 0.000914607 |
| HCLS1 | 1663 | 615.7800834 | 1469.384173 | | 1.254724529 | 0.001481664 | Up | 7.88E-06 |
| CAMK4 | 6702 | 148.3315399 | 353.8872778 | | 1.254464505 | 0.010149583 | Up | 0.000122504 |
| CD84 | 8009 | 530.2176839 | 1264.445975 | | 1.253848705 | 0.001126237 | Up | 5.24E-06 |
| MPEG1 | 4442 | 419.661213 | 996.4629875 | | 1.24759109 | 0.006820874 | Up | 6.79E-05 |
| ARHGEF6 | 5019 | 290.4049533 | 688.9549898 | | 1.246343672 | 0.000167038 | Up | 4.16E-07 |
| HMHA1 | 2252 | 1469.495901 | 3482.313348 | | 1.244724692 | 0.008840256 | Up | 0.000100581 |
| CD5 | 1468 | 165.1953856 | 390.4998319 | | 1.241148539 | 0.002220854 | Up | 1.36E-05 |
| PLCL2 | 3348 | 154.6426136 | 364.9695861 | | 1.238838319 | 0.00739021 | Up | 7.77E-05 |
| NLRC3 | 3040 | 458.2177969 | 1074.783143 | | 1.229940201 | 0.003223217 | Up | 2.40E-05 |
| AC007690.1 | 1845 | 69.41062783 | 162.3435873 | | 1.225821914 | 0.034376738 | Up | 0.000695253 |
| FYB | 1815 | 351.344679 | 820.8539602 | | 1.224238522 | 0.002761242 | Up | 1.88E-05 |
| ARHGDIB | 1001 | 614.7113663 | 1433.363997 | | 1.221423956 | 0.000424986 | Up | 1.42E-06 |
| XXbac-BPG154L12.4 | 3458 | 40.8735461 | 95.21293674 | | 1.219990194 | 0.03209452 | Up | 0.000631741 |
| IL4I1 | 1863 | 153.2504852 | 356.9300551 | | 1.219749747 | 0.03092199 | Up | 0.000593277 |
| FUCA1 | 2047 | 258.4407864 | 600.7387351 | | 1.21690392 | 1.11E-06 | Up | 3.82E-10 |
| RP11-582J16.4 | 4076 | 43.48437133 | 100.9592365 | | 1.215204023 | 0.028103771 | Up | 0.000514282 |
| RP11-796E2.4 | 4598 | 30.51852316 | 70.68067844 | | 1.211630738 | 0.021862202 | Up | 0.000370746 |
| ENPP2 | 2336 | 182.5403498 | 422.0830983 | | 1.209311659 | 0.000358678 | Up | 1.16E-06 |
| RASSF2 | 5427 | 505.2884057 | 1167.307514 | | 1.208005691 | 0.010587969 | Up | 0.000130168 |
| TFEC | 2665 | 127.762421 | 294.9860338 | | 1.207183095 | 0.020072146 | Up | 0.000322589 |
| 6-Sep | 2432 | 485.8719989 | 1121.254367 | | 1.206465408 | 0.000683453 | Up | 2.65E-06 |
| CD226 | 2331 | 44.5362713 | 102.3590462 | | 1.200585927 | 0.029291717 | Up | 0.000552495 |
| SLC4A8 | 1113 | 57.00722311 | 130.9562082 | | 1.199867822 | 0.006873058 | Up | 6.87E-05 |
| RP4-728D4.3 | 1003 | 6.619011127 | 15.19923905 | | 1.199311496 | 0.046959219 | Up | 0.001071618 |
| CUBN | 11949 | 78.28479006 | 179.7093336 | | 1.198861401 | 0.02427048 | Up | 0.000423661 |
| RAC2 | 1353 | 607.1798333 | 1392.460001 | | 1.197440107 | 0.004655952 | Up | 3.87E-05 |
| TLR6 | 5879 | 159.7479354 | 366.204959 | | 1.196854042 | 0.002252811 | Up | 1.44E-05 |
| CELF2 | 6245 | 719.1184378 | 1647.159467 | | 1.195678929 | 0.008179665 | Up | 8.90E-05 |
| HLA-DMA | 1039 | 375.1555615 | 857.7809326 | | 1.193120301 | 0.002510654 | Up | 1.66E-05 |
| RPS23P8 | 330 | 25.99511787 | 59.33021507 | | 1.190526316 | 0.010088929 | Up | 0.000121553 |
| LIMD2 | 1620 | 819.9420102 | 1868.651008 | | 1.18840337 | 0.006885644 | Up | 6.90E-05 |
| CARD11 | 2194 | 234.043142 | 531.1163022 | | 1.182253321 | 0.040302777 | Up | 0.000866993 |
| CTSS | 1370 | 609.3316515 | 1382.097184 | | 1.181559477 | 0.000176659 | Up | 4.51E-07 |
| MGAT4A | 6483 | 404.57476 | 917.6516624 | | 1.181540297 | 0.000764435 | Up | 3.06E-06 |
| LYZ | 1388 | 969.5826445 | 2192.153501 | | 1.176913044 | 0.019723692 | Up | 0.000315709 |
| HLA-DPB1 | 1258 | 749.4862726 | 1688.699776 | | 1.171938903 | 0.002252811 | Up | 1.43E-05 |
| CXorf21 | 1855 | 25.44195551 | 57.32258078 | | 1.171894002 | 0.040302777 | Up | 0.000866535 |
| LAPTM5 | 2196 | 2428.31941 | 5461.415882 | | 1.169316821 | 0.000200828 | Up | 5.26E-07 |
| FERMT3 | 1995 | 439.4707547 | 987.3947708 | | 1.167859843 | 0.007054877 | Up | 7.34E-05 |
| CYBB | 4324 | 909.5948407 | 2038.883937 | | 1.164483676 | 0.009749015 | Up | 0.00011556 |
| CD38 | 1928 | 303.8939236 | 680.4226227 | | 1.16286328 | 0.048930075 | Up | 0.001146228 |
| DENND2D | 1619 | 504.9000781 | 1129.759129 | | 1.161945409 | 0.000129409 | Up | 2.88E-07 |
| RP11-556K13.1 | 880 | 8.792309448 | 19.67050087 | | 1.161719625 | 0.047399608 | Up | 0.00108987 |
| ARL11 | 3760 | 67.40841839 | 150.6912101 | | 1.160594585 | 0.002354683 | Up | 1.53E-05 |
| FAM78A | 4015 | 353.1006832 | 786.9844009 | | 1.156255427 | 0.003542986 | Up | 2.72E-05 |
| FGD2 | 1663 | 685.223966 | 1525.122152 | | 1.154277281 | 0.033534657 | Up | 0.00067242 |
| MYO1G | 3530 | 460.1661645 | 1024.160161 | | 1.154214533 | 0.005232784 | Up | 4.72E-05 |
| HLA-DPA1 | 1043 | 1156.463234 | 2573.618083 | | 1.154078578 | 0.006435623 | Up | 6.21E-05 |
| GPRIN3 | 13391 | 659.1313132 | 1465.5246 | | 1.152779371 | 0.000784841 | Up | 3.26E-06 |
| PARVG | 1756 | 545.6787479 | 1210.651002 | | 1.149659272 | 0.014547775 | Up | 0.000202017 |
| SLC25A53 | 6224 | 38.53847952 | 85.33297695 | | 1.146803726 | 0.010224372 | Up | 0.000123849 |
| FLI1 | 2435 | 278.4368608 | 615.9015714 | | 1.145349594 | 0.001006327 | Up | 4.57E-06 |
| CD209 | 3576 | 146.3031569 | 321.2577991 | | 1.134770579 | 0.009905794 | Up | 0.000118276 |
| SERPINA1 | 856 | 256.1972747 | 562.2862702 | | 1.134049691 | 0.001797942 | Up | 1.01E-05 |
| FCGR2B | 3344 | 280.3029627 | 614.7333833 | | 1.13297384 | 0.02673232 | Up | 0.000482825 |
| FAM107B | 2412 | 570.9386749 | 1251.841559 | | 1.13264428 | 0.001284446 | Up | 6.42E-06 |
| LAT | 1407 | 230.6973712 | 504.9434352 | | 1.130120219 | 0.02667875 | Up | 0.000480703 |
| SNX22 | 2181 | 210.2590638 | 457.9172428 | | 1.122918897 | 0.014414167 | Up | 0.000199543 |
| CD86 | 1024 | 84.77918246 | 184.2973632 | | 1.120253471 | 0.00863014 | Up | 9.69E-05 |
| ICAM3 | 1961 | 254.3330316 | 551.3583108 | | 1.116271342 | 0.010807769 | Up | 0.000134188 |
| N4BP2L1 | 1629 | 148.7839168 | 321.799686 | | 1.112944335 | 0.016047357 | Up | 0.000234648 |
| DOK3 | 1181 | 288.9560068 | 624.5579857 | | 1.111985661 | 0.004779156 | Up | 4.05E-05 |
| AC007038.7 | 2199 | 43.22291077 | 93.14256269 | | 1.107644345 | 0.020878979 | Up | 0.00034504 |
| PIM2 | 1383 | 759.1693319 | 1635.939082 | | 1.107625409 | 0.04004437 | Up | 0.000852321 |
| GNG2 | 2631 | 218.9023333 | 470.7728518 | | 1.104743794 | 0.005453499 | Up | 4.93E-05 |
| SLAMF7 | 1779 | 280.2729315 | 602.0627167 | | 1.103081363 | 0.017377482 | Up | 0.000265318 |
| PLCB2 | 1082 | 401.0169678 | 860.9171362 | | 1.102211103 | 0.008605363 | Up | 9.60E-05 |
| ATM | 6606 | 2143.122336 | 4589.492747 | | 1.098620503 | 0.000253898 | Up | 7.41E-07 |
| CSF2RB | 4863 | 687.6871633 | 1472.116551 | | 1.098067577 | 0.005127023 | Up | 4.56E-05 |
| LCP2 | 1160 | 394.4790935 | 844.1678115 | | 1.097580977 | 0.000588954 | Up | 2.23E-06 |
| KIF21B | 9103 | 436.7140816 | 931.1769167 | | 1.092366245 | 0.006921405 | Up | 7.04E-05 |
| ALOX5AP | 897 | 71.2259913 | 151.5431961 | | 1.089253379 | 0.024778801 | Up | 0.000436286 |
| HHEX | 2824 | 116.4469801 | 247.0703703 | | 1.085248782 | 0.017397329 | Up | 0.000266208 |
| ITGB7 | 1049 | 276.2642078 | 586.0696083 | | 1.085023364 | 0.000767443 | Up | 3.14E-06 |
| CD4 | 2872 | 641.4702826 | 1360.030496 | | 1.084184666 | 0.000767443 | Up | 3.12E-06 |
| ST3GAL5 | 1321 | 138.7702482 | 293.7790025 | | 1.082032992 | 0.025106178 | Up | 0.000445309 |
| PCED1B-AS1 | 1060 | 120.9384394 | 255.8448846 | | 1.08099652 | 0.013822997 | Up | 0.00018807 |
| CCR1 | 2731 | 134.4734027 | 283.9442322 | | 1.078286753 | 0.00921781 | Up | 0.000107485 |
| GIMAP6 | 3440 | 246.8567855 | 520.6066107 | | 1.07651933 | 0.017189887 | Up | 0.000261765 |
| RALGPS2 | 2297 | 575.4379376 | 1210.665573 | | 1.073068155 | 0.015535729 | Up | 0.00022541 |
| ARHGAP30 | 4109 | 998.3553627 | 2100.153793 | | 1.072869643 | 0.000710447 | Up | 2.77E-06 |
| LAT2 | 1419 | 222.8686706 | 468.2259494 | | 1.071011066 | 0.002252811 | Up | 1.43E-05 |
| SLA | 2390 | 693.3304216 | 1454.391839 | | 1.068801041 | 0.006353463 | Up | 6.06E-05 |
| BCL2 | 2377 | 629.8743253 | 1317.687588 | | 1.06487245 | 0.005084239 | Up | 4.50E-05 |
| TNFSF13B | 1670 | 119.1018503 | 246.9381732 | | 1.051954048 | 0.01397743 | Up | 0.000191381 |
| RP11-792D21.2 | 1227 | 42.61883186 | 88.27391085 | | 1.050496065 | 0.043491674 | Up | 0.000961443 |
| ITGB2 | 1846 | 1752.427587 | 3628.664774 | | 1.050083952 | 0.016854324 | Up | 0.000254468 |
| ZNF101 | 3486 | 341.28373 | 704.5851523 | | 1.045802435 | 9.40E-05 | Up | 1.81E-07 |
| ARHGAP4 | 2015 | 817.0757385 | 1680.300949 | | 1.040177929 | 0.023852868 | Up | 0.000414308 |
| ICAM2 | 1012 | 116.8477964 | 240.2739143 | | 1.040049501 | 0.048930075 | Up | 0.001146171 |
| CD74 | 1258 | 8934.002336 | 18348.63196 | | 1.038293965 | 0.022686674 | Up | 0.000390617 |
| APOE | 1108 | 2501.064893 | 5127.893609 | | 1.035823839 | 0.030055553 | Up | 0.000571453 |
| SRGN | 1208 | 744.6723898 | 1526.076073 | | 1.035149107 | 0.014547775 | Up | 0.000202966 |
| 1-Mar | 2168 | 397.9766927 | 811.1253187 | | 1.027240885 | 0.002405707 | Up | 1.57E-05 |
| GLCCI1 | 4420 | 356.2113252 | 724.8519448 | | 1.024952961 | 0.010353487 | Up | 0.000126085 |
| GP1BA | 2501 | 97.27998525 | 197.7215449 | | 1.023255169 | 0.012491877 | Up | 0.000162394 |
| RASGRP1 | 2851 | 244.2004917 | 495.2962614 | | 1.020225626 | 0.004767015 | Up | 4.02E-05 |
| LY75 | 3577 | 502.5446028 | 1018.582353 | | 1.019239077 | 0.022441567 | Up | 0.000382034 |
| CMKLR1 | 2526 | 259.3970688 | 525.2236049 | | 1.017769579 | 0.035557874 | Up | 0.000721449 |
| TRAF3IP3 | 1229 | 409.7493089 | 829.3674942 | | 1.017269988 | 0.016528228 | Up | 0.000246932 |
| LMO2 | 1629 | 144.0942589 | 291.6156213 | | 1.017055149 | 0.016528228 | Up | 0.000245726 |
| ACAP1 | 1389 | 609.0511696 | 1227.106701 | | 1.010625355 | 0.009270432 | Up | 0.000108615 |
| TNFRSF9 | 1923 | 50.34466329 | 101.3312676 | | 1.009168652 | 0.040302777 | Up | 0.000862521 |
| FGR | 2431 | 217.0271125 | 436.0042261 | | 1.006466834 | 0.028534432 | Up | 0.000529228 |
| AL133458.1 | 2298 | 198.5846952 | 398.6046438 | | 1.005204078 | 0.024872824 | Up | 0.000439556 |
| BACH2 | 7728 | 411.1195549 | 824.7814748 | | 1.004453934 | 0.049549512 | Up | 0.001175744 |
| CYTH4 | 3001 | 453.3219319 | 909.1393372 | | 1.003965462 | 0.015110876 | Up | 0.000214744 |
| TRAF5 | 3767 | 485.3820369 | 973.2504989 | | 1.003690461 | 0.002559577 | Up | 1.71E-05 |
| COL7A1 | 8069 | 35762.30335 | 17831.23067 | | -1.004033382 | 0.006921405 | Down | 7.00E-05 |
| NREP | 2013 | 1135.538423 | 564.0731779 | | -1.009422281 | 0.00502514 | Down | 4.40E-05 |
| C14orf132 | 2640 | 826.2814388 | 409.1841585 | | -1.013882968 | 0.005517785 | Down | 5.00E-05 |
| CTD-2206N4.4 | 1534 | 61.32898308 | 30.16475019 | | -1.023705394 | 0.048930075 | Down | 0.001145838 |
| TNS1 | 4030 | 13049.15897 | 6395.058636 | | -1.028927334 | 0.004512403 | Down | 3.70E-05 |
| BOC | 1464 | 1511.582339 | 738.7525823 | | -1.032896395 | 0.005545358 | Down | 5.04E-05 |
| ZNF469 | 13279 | 5707.444675 | 2779.762654 | | -1.037883264 | 0.046618649 | Down | 0.001058804 |
| MYLK | 2399 | 4427.089596 | 2154.744847 | | -1.038841528 | 0.00832354 | Down | 9.16E-05 |
| WNT5A | 5890 | 4114.200593 | 2002.223084 | | -1.03900941 | 0.024897122 | Down | 0.000440524 |
| TRPC6 | 4612 | 224.4705322 | 108.9679098 | | -1.04262273 | 0.00339807 | Down | 2.55E-05 |
| FSTL3 | 1579 | 1646.508388 | 787.5272784 | | -1.06400806 | 0.021026947 | Down | 0.000348531 |
| C3orf80 | 2577 | 207.6969062 | 99.14578976 | | -1.066856312 | 0.018038999 | Down | 0.000280222 |
| RPLP0P2 | 1943 | 229.7466189 | 109.4694973 | | -1.069514699 | 0.03898873 | Down | 0.00081965 |
| COL15A1 | 4089 | 2645.343463 | 1260.395139 | | -1.069578954 | 0.015212881 | Down | 0.000218168 |
| SEMA3B | 1011 | 381.2379771 | 180.9551685 | | -1.075059525 | 0.044051295 | Down | 0.000985249 |
| ANGPTL2 | 1940 | 1539.241655 | 722.5202021 | | -1.091109916 | 0.003416603 | Down | 2.58E-05 |
| ALDH1B1 | 3066 | 1164.650269 | 546.5188787 | | -1.091553558 | 0.007783932 | Down | 8.30E-05 |
| RP11-424C20.2 | 1423 | 240.8302308 | 112.3197687 | | -1.100404631 | 0.017146666 | Down | 0.000260736 |
| TBX5 | 2411 | 129.87654 | 60.51985277 | | -1.101660473 | 0.036122062 | Down | 0.0007356 |
| MN1 | 7556 | 3009.211752 | 1397.888679 | | -1.106136153 | 0.004482478 | Down | 3.67E-05 |
| CASC14 | 1940 | 107.2227103 | 49.79994169 | | -1.10639455 | 0.020247729 | Down | 0.000326725 |
| MFAP4 | 1257 | 1099.744906 | 510.6445312 | | -1.10677766 | 0.016423601 | Down | 0.000243702 |
| TPM2 | 1246 | 1968.113381 | 911.2069146 | | -1.110962735 | 0.021845918 | Down | 0.000369997 |
| ADAMTS12 | 7233 | 2905.076379 | 1342.725861 | | -1.11341131 | 0.032072714 | Down | 0.000630618 |
| NKD2 | 1709 | 1125.582326 | 518.1089957 | | -1.119344043 | 0.040415244 | Down | 0.000872454 |
| MYL9 | 1199 | 4877.682657 | 2237.179532 | | -1.124514863 | 0.028690367 | Down | 0.000536187 |
| TMEM158 | 1813 | 649.930783 | 297.2675178 | | -1.128524251 | 0.028147531 | Down | 0.00051691 |
| COL6A2 | 3203 | 24119.27564 | 11006.15649 | | -1.131875834 | 0.004886641 | Down | 4.17E-05 |
| GPC6 | 6467 | 940.9150934 | 427.1695613 | | -1.139255693 | 0.022111449 | Down | 0.000375451 |
| HECW1 | 5169 | 283.2187164 | 128.3632217 | | -1.141684704 | 0.016854324 | Down | 0.000254201 |
| CLMP | 2610 | 694.1987129 | 314.0504559 | | -1.144352327 | 0.005093583 | Down | 4.52E-05 |
| LDLR | 4191 | 4048.193901 | 1830.219752 | | -1.145261513 | 0.002871771 | Down | 2.04E-05 |
| KANK2 | 1725 | 3188.800347 | 1429.786457 | | -1.15721408 | 0.000206602 | Down | 5.62E-07 |
| CAP2 | 861 | 121.4990426 | 53.83748755 | | -1.174261956 | 0.024434521 | Down | 0.00042811 |
| MSRB3 | 4322 | 1202.417398 | 531.6251503 | | -1.177456526 | 0.000735872 | Down | 2.91E-06 |
| SPARCL1 | 2906 | 2187.201011 | 961.5234969 | | -1.185691797 | 0.008711703 | Down | 9.87E-05 |
| RASL12 | 2106 | 467.5804793 | 204.2094297 | | -1.195165214 | 0.030237343 | Down | 0.000576479 |
| NUS1P1 | 882 | 89.23244385 | 38.5622317 | | -1.210379806 | 0.040613897 | Down | 0.000880186 |
| COL4A6 | 4537 | 1439.23147 | 620.3124844 | | -1.214231572 | 0.013883121 | Down | 0.000189188 |
| COL12A1 | 7845 | 19052.06282 | 8207.341363 | | -1.214960346 | 0.006921405 | Down | 7.05E-05 |
| COL9A3 | 993 | 149.0688158 | 63.86872423 | | -1.222796949 | 0.036918551 | Down | 0.000757658 |
| MRVI1 | 2825 | 1451.835982 | 618.2674774 | | -1.231575454 | 1.93E-05 | Down | 2.17E-08 |
| MXRA5 | 9793 | 9916.102816 | 4202.836938 | | -1.238409747 | 0.002871771 | Down | 2.03E-05 |
| COL5A3 | 6174 | 4960.126768 | 2092.576934 | | -1.245096328 | 0.020627755 | Down | 0.000336585 |
| COL24A1 | 4597 | 173.1022868 | 73.0217882 | | -1.24522588 | 0.038939009 | Down | 0.000817002 |
| YBX3P1 | 1090 | 11.76956035 | 4.96277674 | | -1.245840971 | 0.049020637 | Down | 0.001151531 |
| FHL1 | 1831 | 1157.573876 | 485.9758806 | | -1.25214765 | 0.010554285 | Down | 0.000129299 |
| IGLON5 | 2606 | 74.1010514 | 30.90558142 | | -1.261626606 | 0.047361797 | Down | 0.001081829 |
| MMP2 | 2441 | 17060.0456 | 7105.070222 | | -1.26370069 | 0.010587969 | Down | 0.000130314 |
| PFKFB4 | 1083 | 1666.447335 | 693.4774503 | | -1.264854848 | 0.000845481 | Down | 3.65E-06 |
| PDLIM3 | 1710 | 1717.29736 | 713.0890902 | | -1.267985635 | 0.000905831 | Down | 3.96E-06 |
| NRG1 | 2806 | 530.0204514 | 219.5071574 | | -1.271780047 | 0.049020637 | Down | 0.001150746 |
| FOXF1 | 2568 | 415.6329278 | 171.1657473 | | -1.279915927 | 0.001620349 | Down | 8.80E-06 |
| UCN2 | 1481 | 864.4073894 | 355.5089874 | | -1.281825373 | 0.013486288 | Down | 0.000181739 |
| SPON2 | 1210 | 3341.111437 | 1368.119451 | | -1.288133904 | 7.48E-06 | Down | 5.62E-09 |
| COL6A1 | 3102 | 25983.77127 | 10597.17248 | | -1.293931459 | 0.001806907 | Down | 1.02E-05 |
| PCDHB11 | 1973 | 198.2062955 | 80.75262648 | | -1.295421697 | 0.00502514 | Down | 4.42E-05 |
| CDH6 | 4037 | 304.5696544 | 123.075869 | | -1.307224281 | 0.001167844 | Down | 5.51E-06 |
| SGCD | 4672 | 607.2527332 | 244.9736107 | | -1.309670732 | 0.00111641 | Down | 5.17E-06 |
| TWIST2 | 1176 | 90.79948557 | 36.03980358 | | -1.333092975 | 0.004655952 | Down | 3.86E-05 |
| DAAM2 | 5078 | 1216.149494 | 479.2814236 | | -1.343375653 | 0.000607401 | Down | 2.31E-06 |
| FAT4 | 6851 | 1421.596915 | 558.4221319 | | -1.348084429 | 0.006873058 | Down | 6.86E-05 |
| ADAMTS14 | 5260 | 1513.132376 | 593.3816966 | | -1.350505875 | 0.006962838 | Down | 7.15E-05 |
| SLC24A2 | 10749 | 150.5644833 | 58.83283418 | | -1.35568805 | 0.018271396 | Down | 0.000286251 |
| SOD3 | 1526 | 920.3268567 | 359.262367 | | -1.357108509 | 0.014821104 | Down | 0.000209023 |
| SYNPO2 | 6048 | 3247.971608 | 1264.71593 | | -1.360725647 | 0.033534657 | Down | 0.000671252 |
| SMTN | 2938 | 3845.182409 | 1497.233173 | | -1.360753121 | 0.000586189 | Down | 2.19E-06 |
| AOC3 | 1499 | 1455.865294 | 565.296695 | | -1.364796706 | 0.001271063 | Down | 6.23E-06 |
| RPS5P2 | 640 | 39.18877726 | 15.10557617 | | -1.375359346 | 0.015192434 | Down | 0.000217218 |
| PCSK9 | 3529 | 880.5878013 | 338.7012494 | | -1.37845355 | 0.014732727 | Down | 0.000206821 |
| IGDCC4 | 4261 | 583.8993413 | 224.2064388 | | -1.380891973 | 0.000325052 | Down | 9.91E-07 |
| NACAD | 4780 | 188.6756323 | 72.19857319 | | -1.385865878 | 0.001509509 | Down | 8.13E-06 |
| TBX5-AS1 | 3620 | 84.24382247 | 32.10023166 | | -1.39198719 | 0.039551591 | Down | 0.000837555 |
| GPR3 | 2115 | 53.61892016 | 20.36327937 | | -1.396772249 | 0.020253616 | Down | 0.000328079 |
| MMP11 | 1645 | 10193.56039 | 3843.614366 | | -1.40712254 | 0.013202213 | Down | 0.000176558 |
| IGF2 | 4884 | 4743.090587 | 1783.778185 | | -1.410891197 | 0.01403884 | Down | 0.000192829 |
| PPAPDC3 | 1864 | 97.51820364 | 36.32264415 | | -1.424802321 | 0.000998668 | Down | 4.47E-06 |
| WNT2 | 1768 | 250.3220558 | 93.23246593 | | -1.424881081 | 0.042986366 | Down | 0.000946554 |
| HTRA3 | 2530 | 3126.743742 | 1159.26144 | | -1.431455022 | 1.51E-05 | Down | 1.47E-08 |
| CSPG4 | 8290 | 4461.624872 | 1653.633626 | | -1.431929588 | 0.001271063 | Down | 6.17E-06 |
| F2RL2 | 1387 | 308.5239877 | 113.8744624 | | -1.43793842 | 0.002805941 | Down | 1.95E-05 |
| GJA5 | 1147 | 332.8808248 | 122.4345163 | | -1.442995433 | 0.010468391 | Down | 0.000127937 |
| WBSCR17 | 3884 | 200.8863789 | 73.68307518 | | -1.446974566 | 0.043538715 | Down | 0.000965431 |
| DIO2 | 3818 | 1258.312709 | 458.7568836 | | -1.455688787 | 3.09E-05 | Down | 4.35E-08 |
| WFDC1 | 1971 | 85.52520057 | 30.63683575 | | -1.481082283 | 0.04524761 | Down | 0.001017878 |
| DACT3 | 2667 | 503.5755764 | 180.0250898 | | -1.484010327 | 0.00065928 | Down | 2.52E-06 |
| TPTEP1 | 1195 | 200.9385173 | 70.09440524 | | -1.519382935 | 0.007313101 | Down | 7.67E-05 |
| ROBO2 | 4677 | 247.3185088 | 85.4852803 | | -1.532622283 | 0.00989901 | Down | 0.00011798 |
| TCF21 | 3010 | 189.539159 | 64.79609528 | | -1.54851716 | 0.000668973 | Down | 2.58E-06 |
| FOXF2 | 2187 | 386.8569913 | 131.6666893 | | -1.554909946 | 2.19E-08 | Down | 2.85E-12 |
| CSDC2 | 2294 | 237.2991067 | 80.76035552 | | -1.554987503 | 0.001307535 | Down | 6.62E-06 |
| MRGPRF | 2282 | 656.6052619 | 220.2255361 | | -1.576044546 | 0.000735872 | Down | 2.91E-06 |
| ADAM33 | 3452 | 890.8199773 | 297.6432537 | | -1.581549717 | 0.0027767 | Down | 1.90E-05 |
| KCND2 | 2051 | 273.0754268 | 91.18127337 | | -1.582490034 | 0.005785593 | Down | 5.40E-05 |
| RTL1 | 4193 | 95.63881427 | 31.64644793 | | -1.595552666 | 0.015411383 | Down | 0.000222682 |
| RP11-863P13.3 | 1313 | 102.4531505 | 33.74230316 | | -1.602333992 | 0.012623791 | Down | 0.000166566 |
| RP11-598F7.5 | 1101 | 19.45144768 | 6.386673172 | | -1.606741003 | 0.024382253 | Down | 0.000426667 |
| RP3-342P20.2 | 725 | 123.4464739 | 40.10284329 | | -1.622109195 | 0.014547775 | Down | 0.000202835 |
| TGM1 | 1387 | 1692.748648 | 547.0834937 | | -1.629534834 | 0.016144695 | Down | 0.000237119 |
| LAMC3 | 2029 | 352.0265313 | 113.289419 | | -1.635671042 | 0.002237675 | Down | 1.39E-05 |
| SMTNL2 | 1876 | 47.64121516 | 15.2388886 | | -1.644452523 | 0.039551591 | Down | 0.000836947 |
| ADH5P4 | 1104 | 9.367018772 | 2.918126873 | | -1.682547347 | 0.013639812 | Down | 0.00018464 |
| ST13P19 | 1124 | 3.772515318 | 1.154084495 | | -1.708777905 | 0.049020637 | Down | 0.001151188 |
| SLC35F1 | 4852 | 36.44229083 | 10.94964707 | | -1.734729283 | 0.003005855 | Down | 2.22E-05 |
| LHCGR | 1555 | 35.45340273 | 10.61544198 | | -1.739759661 | 0.029664637 | Down | 0.000562095 |
| RP11-122A3.2 | 894 | 44.00265654 | 12.884406 | | -1.771964598 | 0.018038999 | Down | 0.000280549 |
| LRRN4CL | 2585 | 174.4653194 | 49.76740165 | | -1.809667312 | 0.000209598 | Down | 5.80E-07 |
| KDELC1P1 | 1509 | 16.91112251 | 4.728098707 | | -1.838640365 | 0.017002273 | Down | 0.000257805 |
| MMP16 | 3142 | 318.2270823 | 87.97112449 | | -1.854954661 | 0.00019103 | Down | 4.96E-07 |
| LDB3 | 2800 | 114.2610512 | 31.57655406 | | -1.855408064 | 0.027199229 | Down | 0.000492435 |
| ADAMTS18 | 5913 | 47.89133015 | 12.94502856 | | -1.887366357 | 0.033454706 | Down | 0.000667521 |
| RGMB-AS1 | 1449 | 36.40060575 | 9.816866267 | | -1.890627992 | 0.02096927 | Down | 0.000346986 |
| MIR143HG | 7794 | 4668.861734 | 1251.531358 | | -1.899376425 | 6.26E-07 | Down | 1.89E-10 |
| KCNK3 | 6162 | 72.89230072 | 19.48825689 | | -1.903161382 | 0.010198264 | Down | 0.000123312 |
| RGMA | 3006 | 3236.873254 | 864.2086215 | | -1.905149346 | 0.000339328 | Down | 1.06E-06 |
| GREM1 | 2747 | 5926.047228 | 1543.215237 | | -1.941130831 | 1.03E-07 | Down | 2.01E-11 |
| S100A7A | 4279 | 591.5148131 | 153.4850811 | | -1.946315867 | 0.048834203 | Down | 0.001138203 |
| ACAN | 5667 | 197.7010219 | 49.693875 | | -1.992180379 | 0.000291764 | Down | 8.77E-07 |
| TMEM35 | 1462 | 35.39164438 | 8.506474493 | | -2.056775558 | 0.043906515 | Down | 0.000981061 |
| MIR145 | 2549 | 1168.817562 | 280.3293582 | | -2.059855013 | 1.09E-07 | Down | 2.36E-11 |
| CACNA1H | 6305 | 1575.692067 | 372.4263776 | | -2.080958458 | 3.60E-08 | Down | 5.45E-12 |
| HAND2 | 2780 | 160.4662322 | 37.63482524 | | -2.092129557 | 0.022858343 | Down | 0.000394067 |
| NRK | 3051 | 132.4639297 | 30.98139959 | | -2.096125339 | 0.006962838 | Down | 7.18E-05 |
| CCL11 | 1079 | 49.60865536 | 11.51953079 | | -2.106509899 | 0.000428337 | Down | 1.45E-06 |
| RP11-85M11.2 | 1075 | 28.09764403 | 6.515860375 | | -2.108421572 | 0.00502514 | Down | 4.40E-05 |
| RP11-368J22.2 | 1021 | 94.6231497 | 21.66411566 | | -2.126885838 | 0.036224506 | Down | 0.000738892 |
| KRT8P47 | 1398 | 51.51692057 | 11.74371446 | | -2.133157563 | 0.02022611 | Down | 0.000325767 |
| C20orf166-AS1 | 1568 | 27.14632516 | 6.153394576 | | -2.141302501 | 0.016817039 | Down | 0.000252849 |
| SYNM | 6713 | 3032.436092 | 678.5336344 | | -2.159985005 | 6.99E-05 | Down | 1.27E-07 |
| GPM6A | 550 | 67.95405336 | 15.19141859 | | -2.161303012 | 0.036122062 | Down | 0.000736021 |
| AC131025.8 | 5131 | 2717.312381 | 604.6839773 | | -2.16792717 | 5.62E-08 | Down | 9.72E-12 |
| ELMOD1 | 1662 | 27.59171904 | 6.13027309 | | -2.170212094 | 0.03221348 | Down | 0.000635207 |
| KCNB1 | 11850 | 126.9174799 | 28.06685896 | | -2.176951258 | 0.030343775 | Down | 0.000580215 |
| OR7E47P | 1296 | 39.12572947 | 8.601494935 | | -2.185458324 | 0.002996199 | Down | 2.20E-05 |
| MYLK-AS2 | 754 | 15.21003719 | 3.335095801 | | -2.189223572 | 0.004910555 | Down | 4.23E-05 |
| CHRDL2 | 669 | 216.6632378 | 46.94628556 | | -2.206371466 | 0.001438923 | Down | 7.50E-06 |
| NRXN1 | 2763 | 122.5813738 | 26.45048437 | | -2.212373732 | 0.003508569 | Down | 2.69E-05 |
| SORBS1 | 4731 | 2445.007407 | 524.8821554 | | -2.21977338 | 2.50E-07 | Down | 7.02E-11 |
| LINC00578 | 533 | 48.24169542 | 10.04393039 | | -2.263956676 | 6.12E-05 | Down | 1.07E-07 |
| FLNC | 9042 | 3635.986777 | 741.6980077 | | -2.293443154 | 8.28E-06 | Down | 6.44E-09 |
| LMOD1 | 3970 | 1714.020489 | 346.6587429 | | -2.305796304 | 6.54E-06 | Down | 4.53E-09 |
| PRUNE2 | 2260 | 1546.137653 | 311.2337838 | | -2.312598193 | 1.98E-05 | Down | 2.37E-08 |
| SGCA | 696 | 80.12188115 | 15.63586006 | | -2.357337716 | 0.00032742 | Down | 1.01E-06 |
| HSPB7 | 1541 | 330.8961298 | 61.96724907 | | -2.416800589 | 1.31E-05 | Down | 1.25E-08 |
| ACTG2 | 549 | 1975.59781 | 364.8187063 | | -2.437037662 | 3.82E-05 | Down | 5.70E-08 |
| MYH13 | 5992 | 9.297367308 | 1.710840801 | | -2.442116734 | 0.030754754 | Down | 0.000589231 |
| TYRP1 | 776 | 38.01107398 | 6.969141562 | | -2.447366923 | 0.002160809 | Down | 1.30E-05 |
| CASQ2 | 1369 | 47.56403458 | 8.536367695 | | -2.47817687 | 0.013639812 | Down | 0.000184739 |
| RP11-109I13.2 | 793 | 12.03057808 | 2.14673429 | | -2.486490427 | 0.012638885 | Down | 0.000167312 |
| RP5-1007G16.1 | 608 | 11.64075803 | 2.033972348 | | -2.516813036 | 0.018881363 | Down | 0.000299366 |
| FAM178B | 1209 | 183.6029366 | 32.02064628 | | -2.519514802 | 0.015325473 | Down | 0.000220446 |
| RP11-758N13.1 | 1738 | 380.1610659 | 65.98363576 | | -2.526430607 | 1.58E-07 | Down | 3.76E-11 |
| FENDRR | 2865 | 302.3026511 | 52.01545681 | | -2.538981331 | 2.71E-06 | Down | 1.27E-09 |
| RP11-528A10.1 | 1314 | 7.831924832 | 1.308815576 | | -2.581105095 | 0.034376738 | Down | 0.000694687 |
| SLC27A6 | 2294 | 54.66921957 | 9.097594164 | | -2.587171797 | 0.005622668 | Down | 5.15E-05 |
| C10orf111 | 1714 | 16.65639748 | 2.764896649 | | -2.590778943 | 0.000472473 | Down | 1.64E-06 |
| MYH2 | 6078 | 3.493266356 | 0.575857337 | | -2.600793304 | 0.047710678 | Down | 0.001100119 |
| MYH11 | 2764 | 15896.34187 | 2487.876393 | | -2.675708091 | 3.05E-06 | Down | 1.52E-09 |
| KHSRPP1 | 1768 | 16.21704676 | 2.477268151 | | -2.710689172 | 0.011747048 | Down | 0.000149862 |
| SOX11 | 8718 | 134.2227967 | 20.29369315 | | -2.72552638 | 3.50E-06 | Down | 1.82E-09 |
| PCA3 | 3922 | 81.80626987 | 12.24058352 | | -2.740539086 | 5.26E-05 | Down | 8.65E-08 |
| SLC2A4 | 1768 | 239.5259151 | 35.63731753 | | -2.748721102 | 0.000201017 | Down | 5.35E-07 |
| DUX4L4 | 1369 | 70.45734357 | 10.45598438 | | -2.752421191 | 0.020773092 | Down | 0.000340594 |
| CNN1 | 1372 | 1180.586341 | 174.6794058 | | -2.756722122 | 4.36E-06 | Down | 2.64E-09 |
| RP11-616M22.2 | 476 | 16.32640914 | 2.388397679 | | -2.77309254 | 0.00128237 | Down | 6.36E-06 |
| EPGN | 626 | 157.4291313 | 22.78269535 | | -2.788692184 | 0.006084864 | Down | 5.75E-05 |
| NKX6-1 | 2566 | 219.2155456 | 30.99120943 | | -2.822419148 | 5.93E-10 | Down | 5.13E-14 |
| DGKB | 6684 | 44.9718649 | 6.216204459 | | -2.854916849 | 0.005790324 | Down | 5.44E-05 |
| SCUBE1 | 9808 | 206.1500495 | 28.21868438 | | -2.868972174 | 3.77E-06 | Down | 2.04E-09 |
| RP11-566K19.5 | 3940 | 12.85854494 | 1.74357394 | | -2.882607947 | 0.032396668 | Down | 0.000639791 |
| IGF2-AS | 1867 | 16.30118467 | 2.186369571 | | -2.898367623 | 0.027602998 | Down | 0.000503327 |
| JPH2 | 4787 | 1080.452596 | 142.5715731 | | -2.921877515 | 8.15E-09 | Down | 8.82E-13 |
| RP5-1198O20.4 | 1569 | 51.10769585 | 6.723948916 | | -2.926159881 | 0.003002411 | Down | 2.21E-05 |
| RN7SL815P | 291 | 10.37435266 | 1.351351878 | | -2.940546026 | 0.021213892 | Down | 0.000354246 |
| RP11-805I24.3 | 4039 | 8.623437433 | 1.119911688 | | -2.944878093 | 0.033454706 | Down | 0.000668645 |
| HMGB4 | 927 | 16.90130128 | 2.177251798 | | -2.956554159 | 0.003826131 | Down | 3.01E-05 |
| FGF5 | 5234 | 30.95949085 | 3.774962941 | | -3.035847359 | 2.83E-05 | Down | 3.74E-08 |
| RP5-1198O20.5 | 520 | 4.616838191 | 0.523974543 | | -3.139336544 | 0.021538339 | Down | 0.000361527 |
| BMP5 | 3952 | 24.28165208 | 2.66659281 | | -3.186797137 | 0.021046356 | Down | 0.000350216 |
| EPYC | 790 | 25.0355504 | 2.74698229 | | -3.188058659 | 0.010225313 | Down | 0.000124081 |
| RP11-1090M7.1 | 3679 | 11.56702252 | 1.245636478 | | -3.215062543 | 0.012530674 | Down | 0.0001632 |
| Z99756.1 | 1951 | 7.857024203 | 0.817934198 | | -3.263926315 | 0.004907233 | Down | 4.21E-05 |
| RP11-811P12.3 | 459 | 3.03853621 | 0.305856432 | | -3.312449963 | 0.028534432 | Down | 0.000532039 |
| RP11-690I21.3 | 497 | 5.376553258 | 0.528762185 | | -3.345990693 | 0.006496666 | Down | 6.30E-05 |
| KLF14 | 1488 | 20.85323063 | 1.905238193 | | -3.452227626 | 0.000818159 | Down | 3.46E-06 |
| FTH1P14 | 551 | 3.916073476 | 0.353029192 | | -3.471548442 | 0.021769006 | Down | 0.000367753 |
| SUPT20HL1 | 2472 | 6.414664612 | 0.562120187 | | -3.512423305 | 0.002946494 | Down | 2.13E-05 |
| FGF19 | 2447 | 184.4482894 | 15.11886886 | | -3.608794299 | 0.006599142 | Down | 6.47E-05 |
| NOBOX | 1725 | 13.56611652 | 1.083288148 | | -3.646518842 | 0.020253616 | Down | 0.000328572 |
| MYOCD | 8466 | 212.1891324 | 16.21666756 | | -3.70980148 | 1.89E-07 | Down | 4.90E-11 |
| RP11-252O2.2 | 318 | 4.751012632 | 0.36087728 | | -3.718654821 | 0.007857058 | Down | 8.40E-05 |
| LRRTM3 | 4358 | 8.45688663 | 0.576257528 | | -3.87534104 | 0.012195156 | Down | 0.000157217 |
| ACTC1 | 4106 | 67.90453825 | 4.622757215 | | -3.876682497 | 9.93E-06 | Down | 8.37E-09 |
| CTD-2297D10.1 | 454 | 4.914208923 | 0.285313909 | | -4.106337209 | 0.0479516 | Down | 0.001107748 |
| HPSE2 | 2121 | 45.56362589 | 2.603545586 | | -4.129332987 | 2.78E-05 | Down | 3.60E-08 |
| AC005562.1 | 3219 | 19.06256625 | 0.831751557 | | -4.51844588 | 0.027602998 | Down | 0.00050305 |
| DES | 1192 | 6332.490695 | 198.9409266 | | -4.992361046 | 8.21E-13 | Down | 3.55E-17 |
| PCP4 | 587 | 9.891347215 | 0.285524001 | | -5.114483101 | 1.62E-05 | Down | 1.68E-08 |
| AC005358.1 | 2055 | 17.17910744 | 0.481694613 | | -5.156392482 | 0.000339328 | Down | 1.06E-06 |
| SLITRK3 | 4391 | 42.4706731 | 0.423081341 | | -6.649388102 | 4.86E-12 | Down | 3.15E-16 |
| MAGEA9B | 1814 | 103.0470433 | 0.115864099 | | -9.796653789 | 0.022579766 | Down | 0.000387032 |
