# Supplementary material for: Multi‐omics analyses reveal spatial heterogeneity in primary and metastatic oesophageal squamous cell carcinoma
Source: Clin Transl Med. 2023 Nov 27;13(11):e1493. doi: 10.1002/ctm2.1493 (PMC10679972; doi:10.1002/ctm2.1493)
Supplement: Supplementary file 22 — Table S11. Enriched pathways of DEGs in analyses of tumour subregions by KEGG analysis. [file CTM2-13-e1493-s002.docx]

**Supplementary Table 11. Enriched pathways of DEGs in analyses of tumor subregions by KEGG analysis.**

| **The top20 enriched pathways of up-regulated DEGs in comparison analyses of LN_met_ versus PT_sup_** | | | | | | | | |
| --- | --- | --- | --- | --- | --- | --- | --- | --- |
| **ID** | **Description** | **GeneRatio** | **BgRatio** | ***P*-value** | ***P*.adjust** | ***q-*value** | **geneID** | **Count** |
| hsa04060 | Cytokine-cytokine receptor interaction | 32/251 | 295/8115 | 3.99E-10 | 5.53E-09 | 4.54E-09 | CCR5/CXCL13/IL21R/CXCL11/LTB/IFNG/TNFRSF17/CSF2RB/IL2RG/CCL19/CXCR3/CCR6/IL10RA/GDF10/CD4/CCR7/CCR2/TNFRSF13C/TNFSF13B/TNFSF14/CXCR4/CCR9/CD40LG/IL7R/IL18R1/TNFSF8/  TNFRSF13B/CCR4/IL12RB1/CSF2RA/LTA/CCL21 | 32 |
| hsa04640 | Hematopoietic cell lineage | 29/251 | 99/8115 | 4.34E-21 | 1.02E-18 | 8.40E-19 | HLA-DMB/CD3E/CR1/CD1E/CD38/HLA-DRA/FCER2/CD1D/CD19/CD37/CD2/MS4A1/HLA-DMA/GP1BA/HLA-DRB1/CD4/HLA-DPB1/CD1C/CR2/CD22/CD3D/IL7R/HLA-DPA1/HLA-DOA/HLA-DQA1/  HLA-DOB/CSF2RA/CD3G/CD5 | 29 |
| hsa04514 | Cell adhesion molecules | 29/251 | 149/8115 | 8.16E-16 | 3.21E-14 | 2.64E-14 | HLA-DMB/SELP/HLA-DRA/ITGB7/CD6/SIGLEC1/CD2/HLA-DMA/CD80/HLA-DRB1/CD4/HLA-DPB1/SELL/CD22/CD40LG/ICAM3/LRRC4C/TIGIT/CD226/HLA-DPA1/SPN/HLA-DOA/CD28/ITGAL/  HLA-DQA1/ITGB2/HLA-DOB/CD86/PTPRC | 29 |
| hsa04659 | Th17 cell differentiation | 27/251 | 108/8115 | 9.90E-18 | 4.67E-16 | 3.84E-16 | HLA-DMB/CD3E/JAK3/IL21R/PRKCQ/HLA-DRA/STAT5A/IFNG/IL2RG/NFATC2/IRF4/HLA-DMA/LCK/HLA-DRB1/CD4/HLA-DPB1/CD247/CD3D/HLA-DPA1/HLA-DOA/IL12RB1/JAK2/HLA-DQA1/  HLA-DOB/CD3G/LAT/ZAP70 | 27 |
| hsa04658 | Th1 and Th2 cell differentiation | 26/251 | 92/8115 | 1.44E-18 | 1.13E-16 | 9.29E-17 | HLA-DMB/CD3E/JAK3/PRKCQ/HLA-DRA/STAT5A/IFNG/IL2RG/STAT4/NFATC2/HLA-DMA/LCK/HLA-DRB1/CD4/HLA-DPB1/CD247/CD3D/HLA-DPA1/HLA-DOA/IL12RB1/JAK2/HLA-DQA1/HLA-DOB/  CD3G/LAT/ZAP70 | 26 |
| hsa04062 | Chemokine signaling pathway | 26/251 | 192/8115 | 1.60E-10 | 2.91E-09 | 2.39E-09 | JAK3/NCF1/CCR5/PRKCB/CXCL13/RASGRP2/CXCL11/PIK3R5/  CCL19/CXCR3/ITK/CCR6/FGR/CCR7/CCR2/PIK3CG/DOCK2/CXCR4/  CCR9/VAV1/PLCB2/PLCG2/CCR4/JAK2/GNG2/CCL21 | 26 |
| hsa05152 | Tuberculosis | 25/251 | 180/8115 | 2.13E-10 | 3.34E-09 | 2.74E-09 | HLA-DMB/CORO1A/CTSS/CR1/HLA-DRA/ITGAX/IFNG/CLEC4M/CD74/HLA-DMA/IL10RA/HLA-DRB1/HLA-DPB1/FCGR2B/CIITA/HLA-DPA1/BCL2/HLA-DOA/TLR6/TLR1/JAK2/HLA-DQA1/ITGB2/  HLA-DOB/FCGR2C | 25 |
| hsa04064 | NF-kappa B signaling pathway | 22/251 | 104/8115 | 4.95E-13 | 1.67E-11 | 1.37E-11 | PRKCB/BIRC3/PRKCQ/BTK/TRAF5/LTB/CCL19/CARD11/LCK/ATM/TNFRSF13C/TNFSF13B/TNFSF14/CD40LG/BLNK/BCL2/PLCG2/  TRAF1/LTA/CCL21/LAT/ZAP70 | 22 |
| hsa04672 | Intestinal immune network for IgA production | 21/251 | 49/8115 | 1.56E-19 | 1.84E-17 | 1.51E-17 | HLA-DMB/HLA-DRA/ITGB7/AICDA/TNFRSF17/HLA-DMA/CD80/HLA-DRB1/HLA-DPB1/TNFRSF13C/TNFSF13B/CXCR4/CCR9/CD40LG/HLA-DPA1/HLA-DOA/TNFRSF13B/CD28/HLA-DQA1/HLA-DOB/  CD86 | 21 |
| hsa04660 | T cell receptor signaling pathway | 20/251 | 104/8115 | 3.80E-11 | 8.14E-10 | 6.68E-10 | CD3E/RASGRP1/PRKCQ/IFNG/NFATC2/ITK/LCP2/CARD11/LCK/CD4/CD247/CD40LG/CD3D/VAV1/CD28/CD3G/GRAP2/PTPRC/LAT/  ZAP70 | 20 |
| hsa04662 | B cell receptor signaling pathway | 19/251 | 82/8115 | 3.64E-12 | 9.55E-11 | 7.84E-11 | PRKCB/BTK/CD19/NFATC2/CD72/LILRA4/CD79A/CARD11/RASGRP3/INPP5D/FCGR2B/CR2/CD22/BLNK/VAV1/PLCG2/LILRB1/CD79B/PIK3AP1 | 19 |
| hsa05145 | Toxoplasmosis | 19/251 | 112/8115 | 1.13E-09 | 1.40E-08 | 1.15E-08 | HLA-DMB/CCR5/BIRC3/HLA-DRA/IFNG/PIK3R5/HLA-DMA/IL10RA/HLA-DRB1/HLA-DPB1/PIK3CG/CD40LG/CIITA/HLA-DPA1/BCL2/HLA-DOA/JAK2/HLA-DQA1/HLA-DOB | 19 |
| hsa05340 | Primary immunodeficiency | 18/251 | 38/8115 | 7.08E-18 | 4.18E-16 | 3.43E-16 | CD3E/JAK3/BTK/AICDA/CD19/IL2RG/CD79A/LCK/CD4/TNFRSF13C/CD40LG/CD3D/IL7R/CIITA/BLNK/TNFRSF13B/PTPRC/ZAP70 | 18 |
| hsa05140 | Leishmaniasis | 17/251 | 77/8115 | 1.18E-10 | 2.32E-09 | 1.91E-09 | HLA-DMB/NCF1/CR1/PRKCB/HLA-DRA/IFNG/HLA-DMA/CYBB/HLA-DRB1/HLA-DPB1/HLA-DPA1/HLA-DOA/JAK2/HLA-DQA1/ITGB2/HLA-DOB/FCGR2C | 17 |
| hsa05323 | Rheumatoid arthritis | 17/251 | 93/8115 | 2.67E-09 | 3.15E-08 | 2.58E-08 | HLA-DMB/HLA-DRA/LTB/IFNG/HLA-DMA/CD80/HLA-DRB1/HLA-DPB1/TNFSF13B/HLA-DPA1/HLA-DOA/CD28/ITGAL/HLA-DQA1  /ITGB2/HLA-DOB/CD86 | 17 |
| hsa05416 | Viral myocarditis | 15/251 | 60/8115 | 2.29E-10 | 3.38E-09 | 2.78E-09 | HLA-DMB/HLA-DRA/HLA-DMA/CD80/HLA-DRB1/HLA-DPB1/CD40LG/HLA-DPA1/HLA-DOA/CD28/ITGAL/HLA-DQA1/ITGB2/  HLA-DOB/CD86 | 15 |
| hsa05321 | Inflammatory bowel disease | 15/251 | 65/8115 | 7.79E-10 | 1.02E-08 | 8.38E-09 | HLA-DMB/IL21R/HLA-DRA/IFNG/IL2RG/STAT4/HLA-DMA/HLA-DRB1/HLA-DPB1/IL18R1/HLA-DPA1/HLA-DOA/IL12RB1/HLA-DQA1/HLA-DOB | 15 |
| hsa05330 | Allograft rejection | 14/251 | 38/8115 | 2.55E-12 | 7.53E-11 | 6.18E-11 | HLA-DMB/HLA-DRA/IFNG/HLA-DMA/CD80/HLA-DRB1/HLA-DPB1/CD40LG/HLA-DPA1/HLA-DOA/CD28/HLA-DQA1/HLA-DOB/CD86 | 14 |
| hsa04940 | Type I diabetes mellitus | 14/251 | 43/8115 | 1.80E-11 | 4.25E-10 | 3.49E-10 | HLA-DMB/HLA-DRA/IFNG/HLA-DMA/CD80/HLA-DRB1/HLA-DPB1/HLA-DPA1/HLA-DOA/CD28/HLA-DQA1/HLA-DOB/LTA/CD86 | 14 |
| hsa05332 | Graft-versus-host disease | 13/251 | 42/8115 | 2.00E-10 | 3.34E-09 | 2.74E-09 | HLA-DMB/HLA-DRA/IFNG/HLA-DMA/CD80/HLA-DRB1/HLA-DPB1/HLA-DPA1/HLA-DOA/CD28/HLA-DQA1/HLA-DOB/CD86 | 13 |
| **The top20 enriched pathways of down-regulated DEGs in comparison analyses of LN_met_ versus PT_sup_** | | | | | | | | |
| **ID** | **Description** | **GeneRatio** | **BgRatio** | ***P-*value** | ***P*.adjust** | ***q-*value** | **geneID** | **Count** |
| hsa05205 | Proteoglycans in cancer | 6/44 | 205/8115 | 0.00076529 | 0.094522066 | 0.083703751 | WNT2/HPSE2/TWIST2/IGF2/WNT5A/FLNC | 6 |
| hsa05165 | Human papillomavirus infection | 6/44 | 331/8115 | 0.008388739 | 0.158605458 | 0.140452619 | WNT2/LAMC3/COL6A2/COL6A1/WNT5A/EIF4EBP1 | 6 |
| hsa04151 | PI3K-Akt signaling pathway | 6/44 | 354/8115 | 0.01147493 | 0.158605458 | 0.140452619 | LAMC3/COL6A2/IGF2/COL6A1/FGF5/EIF4EBP1 | 6 |
| hsa04010 | MAPK signaling pathway | 5/44 | 294/8115 | 0.020631597 | 0.216631769 | 0.191837656 | CACNA1H/IGF2/FLNC/FGF5/IL1B | 5 |
| hsa04350 | TGF-beta signaling pathway | 4/44 | 94/8115 | 0.001608093 | 0.094522066 | 0.083703751 | RGMA/GREM1/BMP4/GREM2 | 4 |
| hsa04974 | Protein digestion and absorption | 4/44 | 103/8115 | 0.002250525 | 0.094522066 | 0.083703751 | COL6A2/COL7A1/COL12A1/COL6A1 | 4 |
| hsa04934 | Cushing syndrome | 4/44 | 155/8115 | 0.009604715 | 0.158605458 | 0.140452619 | WNT2/CACNA1H/WNT5A/LDLR | 4 |
| hsa04390 | Hippo signaling pathway | 4/44 | 157/8115 | 0.010036474 | 0.158605458 | 0.140452619 | WNT2/NKD2/BMP4/WNT5A | 4 |
| hsa04510 | Focal adhesion | 4/44 | 201/8115 | 0.022907965 | 0.222031043 | 0.196618969 | LAMC3/COL6A2/COL6A1/FLNC | 4 |
| hsa05217 | Basal cell carcinoma | 3/44 | 63/8115 | 0.004708519 | 0.148318357 | 0.131342906 | WNT2/BMP4/WNT5A | 3 |
| hsa04512 | ECM-receptor interaction | 3/44 | 88/8115 | 0.01184633 | 0.158605458 | 0.140452619 | LAMC3/COL6A2/COL6A1 | 3 |
| hsa05410 | Hypertrophic cardiomyopathy | 3/44 | 90/8115 | 0.012587735 | 0.158605458 | 0.140452619 | ACTC1/DES/SGCD | 3 |
| hsa05414 | Dilated cardiomyopathy | 3/44 | 96/8115 | 0.014969565 | 0.171469563 | 0.151844391 | ACTC1/DES/SGCD | 3 |
| hsa04152 | AMPK signaling pathway | 3/44 | 120/8115 | 0.026904481 | 0.24214033 | 0.214426692 | SLC2A4/PFKFB4/EIF4EBP1 | 3 |
| hsa04910 | Insulin signaling pathway | 3/44 | 137/8115 | 0.03770373 | 0.296916871 | 0.262933904 | SLC2A4/SORBS1/EIF4EBP1 | 3 |
| hsa04550 | Signaling pathways regulating pluripotency of stem cells | 3/44 | 143/8115 | 0.041973805 | 0.303555831 | 0.268813017 | WNT2/BMP4/WNT5A | 3 |
| hsa05224 | Breast cancer | 3/44 | 147/8115 | 0.044951295 | 0.303555831 | 0.268813017 | WNT2/WNT5A/FGF5 | 3 |
| hsa05226 | Gastric cancer | 3/44 | 149/8115 | 0.046478909 | 0.303555831 | 0.268813017 | WNT2/WNT5A/FGF5 | 3 |
| hsa04979 | Cholesterol metabolism | 2/44 | 50/8115 | 0.029857546 | 0.250803385 | 0.222098236 | PCSK9/LDLR | 2 |
| hsa04927 | Cortisol synthesis and secretion | 2/44 | 65/8115 | 0.048183465 | 0.303555831 | 0.268813017 | CACNA1H/LDLR | 2 |
| **The top20 enriched pathways of up-regulated DEGs in comparison analyses of LN_met_ versus PT_deep_.** | | | | | | | | |
| **ID** | **Description** | **GeneRatio** | **BgRatio** | ***P*-value** | ***P*.adjust** | ***q-*value** | **geneID** | **Count** |
| hsa04640 | Hematopoietic cell lineage | 11/79 | 99/8115 | 2.30E-09 | 3.87E-07 | 3.18E-07 | HLA-DMB/CR1/HLA-DRA/FCER2/MS4A1/HLA-DMA/HLA-DPB1/  CD22/CD3D/IL7R/CD3G | 11 |
| hsa04514 | Cell adhesion molecules | 10/79 | 149/8115 | 1.57E-06 | 0.00013175 | 0.000108141 | HLA-DMB/HLA-DRA/ITGB7/HLA-DMA/HLA-DPB1/SELL/CD22/SPN/ITGAL/PTPRC | 10 |
| hsa05152 | Tuberculosis | 9/79 | 180/8115 | 5.76E-05 | 0.001074844 | 0.000882234 | CD209/HLA-DMB/CORO1A/CTSS/CR1/HLA-DRA/CLEC4M/HLA-DMA/HLA-DPB1 | 9 |
| hsa05169 | Epstein-Barr virus infection | 9/79 | 202/8115 | 0.000139928 | 0.001808307 | 0.001484262 | HLA-DMB/HLA-DRA/FCER2/HLA-DMA/HLA-DPB1/CD3D/PLCG2/ITGAL/CD3G | 9 |
| hsa04650 | Natural killer cell mediated cytotoxicity | 8/79 | 131/8115 | 3.71E-05 | 0.000890728 | 0.000731111 | CD48/LCP2/VAV1/RAC2/SH2D1A/PLCG2/ITGAL/PTPN6 | 8 |
| hsa04145 | Phagosome | 8/79 | 152/8115 | 0.000106689 | 0.001493652 | 0.001225993 | CD209/HLA-DMB/CORO1A/CTSS/HLA-DRA/CLEC4M/HLA-DMA/HLA-DPB1 | 8 |
| hsa04662 | B cell receptor signaling pathway | 7/79 | 82/8115 | 1.34E-05 | 0.000562463 | 0.000461671 | RASGRP3/CD22/VAV1/RAC2/PLCG2/PTPN6/PIK3AP1 | 7 |
| hsa04659 | Th17 cell differentiation | 7/79 | 108/8115 | 8.02E-05 | 0.001347797 | 0.001106275 | HLA-DMB/HLA-DRA/IRF4/HLA-DMA/HLA-DPB1/CD3D/CD3G | 7 |
| hsa04672 | Intestinal immune network for IgA production | 6/79 | 49/8115 | 7.04E-06 | 0.000394207 | 0.000323566 | HLA-DMB/HLA-DRA/ITGB7/AICDA/HLA-DMA/HLA-DPB1 | 6 |
| hsa05416 | Viral myocarditis | 6/79 | 60/8115 | 2.31E-05 | 0.00077782 | 0.000638436 | HLA-DMB/HLA-DRA/HLA-DMA/HLA-DPB1/RAC2/ITGAL | 6 |
| hsa05140 | Leishmaniasis | 6/79 | 77/8115 | 9.62E-05 | 0.001468552 | 0.00120539 | HLA-DMB/CR1/HLA-DRA/HLA-DMA/HLA-DPB1/PTPN6 | 6 |
| hsa04658 | Th1 and Th2 cell differentiation | 6/79 | 92/8115 | 0.000257623 | 0.002869413 | 0.00235522 | HLA-DMB/HLA-DRA/HLA-DMA/HLA-DPB1/CD3D/CD3G | 6 |
| hsa05323 | Rheumatoid arthritis | 6/79 | 93/8115 | 0.000273277 | 0.002869413 | 0.00235522 | HLA-DMB/HLA-DRA/LTB/HLA-DMA/HLA-DPB1/ITGAL | 6 |
| hsa04064 | NF-kappa B signaling pathway | 6/79 | 104/8115 | 0.000499843 | 0.004198681 | 0.003446286 | BIRC3/LTB/BCL2A1/PLCG2/LTA/CCL21 | 6 |
| hsa04660 | T cell receptor signaling pathway | 6/79 | 104/8115 | 0.000499843 | 0.004198681 | 0.003446286 | LCP2/CD3D/VAV1/CD3G/PTPN6/PTPRC | 6 |
| hsa05340 | Primary immunodeficiency | 5/79 | 38/8115 | 3.00E-05 | 0.000841021 | 0.000690312 | AIRE/AICDA/CD3D/IL7R/PTPRC | 5 |
| hsa04940 | Type I diabetes mellitus | 5/79 | 43/8115 | 5.55E-05 | 0.001074844 | 0.000882234 | HLA-DMB/HLA-DRA/HLA-DMA/HLA-DPB1/LTA | 5 |
| hsa04664 | Fc epsilon RI signaling pathway | 5/79 | 68/8115 | 0.000496794 | 0.004198681 | 0.003446286 | LCP2/ALOX5AP/VAV1/RAC2/PLCG2 | 5 |
| hsa05310 | Asthma | 4/79 | 31/8115 | 0.000214388 | 0.002572651 | 0.002111637 | HLA-DMB/HLA-DRA/HLA-DMA/HLA-DPB1 | 4 |
| hsa05330 | Allograft rejection | 4/79 | 38/8115 | 0.000477631 | 0.004198681 | 0.003446286 | HLA-DMB/HLA-DRA/HLA-DMA/HLA-DPB1 | 4 |
| **The top20 enriched pathways of down-regulated DEGs in comparison analyses of LN_met_ versus PT_deep_.** | | | | | | | | |
| **ID** | **Description** | **GeneRatio** | **BgRatio** | **pvalue** | **p.adjust** | **qvalue** | **geneID** | **Count** |
| hsa05414 | Dilated cardiomyopathy | 9/54 | 96/8115 | 1.06E-08 | 1.46E-06 | 1.29E-06 | TPM2/SGCA/ACTC1/ADCY5/CACNB2/DES/SGCD/TPM1/ITGA5 | 9 |
| hsa05410 | Hypertrophic cardiomyopathy | 8/54 | 90/8115 | 1.15E-07 | 7.85E-06 | 6.93E-06 | TPM2/SGCA/ACTC1/CACNB2/DES/SGCD/TPM1/ITGA5 | 8 |
| hsa04510 | Focal adhesion | 8/54 | 201/8115 | 4.82E-05 | 0.001502014 | 0.001327173 | MYLK/COL6A2/THBS4/MYL9/FLNA/COL6A1/ITGA5/FLNC | 8 |
| hsa04261 | Adrenergic signaling in cardiomyocytes | 7/54 | 150/8115 | 5.48E-05 | 0.001502014 | 0.001327173 | TPM2/ACTC1/ADCY5/SCN7A/CACNB2/ATP1A2/TPM1 | 7 |
| hsa04022 | cGMP-PKG signaling pathway | 7/54 | 167/8115 | 0.000108144 | 0.002469288 | 0.002181853 | MYLK/MYL9/ADCY5/TRPC6/ATP1A2/PDE5A/MRVI1 | 7 |
| hsa04260 | Cardiac muscle contraction | 6/54 | 87/8115 | 2.18E-05 | 0.000996401 | 0.000880416 | TPM2/ACTC1/CASQ2/CACNB2/ATP1A2/TPM1 | 6 |
| hsa04270 | Vascular smooth muscle contraction | 6/54 | 134/8115 | 0.00024423 | 0.004182444 | 0.00369559 | MYLK/MYL9/ADCY5/MRVI1/MYH11/ACTG2 | 6 |
| hsa05412 | Arrhythmogenic right ventricular cardiomyopathy | 5/54 | 77/8115 | 0.000148407 | 0.002904541 | 0.002566441 | SGCA/CACNB2/DES/SGCD/ITGA5 | 5 |
| hsa04810 | Regulation of actin cytoskeleton | 5/54 | 218/8115 | 0.014525059 | 0.142138076 | 0.125592614 | MYLK/MYL9/ITGA5/FGF5/MYH11 | 5 |
| hsa04020 | Calcium signaling pathway | 5/54 | 240/8115 | 0.021145057 | 0.170404283 | 0.150568517 | CACNA1H/MYLK/LHCGR/CASQ2/FGF5 | 5 |
| hsa04010 | MAPK signaling pathway | 5/54 | 294/8115 | 0.044980262 | 0.292144867 | 0.258137992 | CACNA1H/CACNB2/FLNA/FLNC/FGF5 | 5 |
| hsa04512 | ECM-receptor interaction | 4/54 | 88/8115 | 0.002701529 | 0.041123271 | 0.036336351 | COL6A2/THBS4/COL6A1/ITGA5 | 4 |
| hsa04925 | Aldosterone synthesis and secretion | 4/54 | 98/8115 | 0.003985077 | 0.054595562 | 0.048240412 | CACNA1H/KCNK3/ADCY5/ATP1A2 | 4 |
| hsa04974 | Protein digestion and absorption | 4/54 | 103/8115 | 0.004759821 | 0.059281401 | 0.0523808 | COL15A1/COL6A2/ATP1A2/COL6A1 | 4 |
| hsa04921 | Oxytocin signaling pathway | 4/54 | 154/8115 | 0.018925726 | 0.16205153 | 0.143188059 | MYLK/MYL9/ADCY5/CACNB2 | 4 |
| hsa04360 | Axon guidance | 4/54 | 182/8115 | 0.03243874 | 0.233900389 | 0.206673413 | RGMA/BOC/MYL9/TRPC6 | 4 |
| hsa04927 | Cortisol synthesis and secretion | 3/54 | 65/8115 | 0.009096391 | 0.10385046 | 0.091761836 | CACNA1H/KCNK3/ADCY5 | 3 |
| hsa04971 | Gastric acid secretion | 3/54 | 76/8115 | 0.013909647 | 0.142138076 | 0.125592614 | MYLK/ADCY5/ATP1A2 | 3 |
| hsa00410 | beta-Alanine metabolism | 2/54 | 31/8115 | 0.017865214 | 0.16205153 | 0.143188059 | AOC3/ALDH1B1 | 2 |
| hsa00350 | Tyrosine metabolism | 2/54 | 36/8115 | 0.023698918 | 0.1803751 | 0.15937869 | AOC3/TYRP1 | 2 |
| **The top20 enriched pathways of up-regulated DEGs in comparison analyses of LN_met_ versus PT.** | | | | | | | | |
| **ID** | **Description** | **GeneRatio** | **BgRatio** | ***P*-value** | ***P*.adjust** | ***q-*value** | **geneID** | **Count** |
| hsa04514 | Cell adhesion molecules | 27/211 | 157/8163 | 2.34E-15 | 1.37E-13 | 1.14E-13 | PTPRC/HLA-DOA/CD86/HLA-DMA/ITGB2/CD226/ICAM3/HLA-DMB/SPN/CD22/SELL/CD40LG/CD80/CD2/HLA-DOB/ITGAL/CD6/CD28/HLA-DQA1/CD4/TIGIT/HLA-DPB1/ICOS/ITGB7/HLA-DPA1/HLA-DRA/ICAM2 | 27 |
| hsa04060 | Cytokine-cytokine receptor interaction | 26/211 | 295/8163 | 3.50E-08 | 4.32E-07 | 3.59E-07 | CCR6/TNFRSF13B/CXCR4/CXCL13/CCL22/CD40LG/LTB/CCL21/TNFRSF17/TNFRSF9/CCR4/CCR5/IL21R/TNFSF13B/TNFSF8/IL7R/CCR7/CD4/LTA/IL12RB1/CCR9/CXCR3/CSF2RB/IL2RG/CCR1/CCL19 | 26 |
| hsa04062 | Chemokine signaling pathway | 25/211 | 192/8163 | 1.75E-11 | 4.17E-10 | 3.47E-10 | RAC2/CCR6/PLCB2/PRKCB/CXCR4/JAK3/CXCL13/CCL22/GNG2/DOCK2/RASGRP2/CCL21/CCR4/CCR5/NCF1/VAV1/CCR7/CCR9/FGR/PIK3CG/PLCG2/CXCR3/ITK/CCR1/CCL19 | 25 |
| hsa04640 | Hematopoietic cell lineage | 24/211 | 99/8163 | 2.38E-17 | 1.86E-15 | 1.55E-15 | CD5/HLA-DOA/HLA-DMA/CR2/HLA-DMB/CD38/CD22/CR1/FCER2/GP1BA/CD2/HLA-DOB/CD37/MS4A1/CD19/CD3E/HLA-DQA1/IL7R/CD4/CD3D/HLA-DPB1/CD3G/HLA-DPA1/HLA-DRA | 24 |
| hsa05169 | Epstein-Barr virus infection | 22/211 | 202/8163 | 9.52E-09 | 1.31E-07 | 1.09E-07 | HLA-DOA/HLA-DMA/CR2/BTK/HLA-DMB/JAK3/FCER2/HLA-DOB/ITGAL/CD19/CD3E/HLA-DQA1/BLNK/CD3D/HLA-DPB1/CD3G/PLCG2/TRAF5/HLA-DPA1/HLA-DRA/CD247/BCL2 | 22 |
| hsa04659 | Th17 cell differentiation | 21/211 | 108/8163 | 2.89E-13 | 1.13E-11 | 9.40E-12 | HLA-DOA/IRF4/HLA-DMA/HLA-DMB/JAK3/LAT/LCK/ZAP70/HLA-DOB/IL21R/CD3E/HLA-DQA1/CD4/IL12RB1/CD3D/HLA-DPB1/CD3G/HLA-DPA1/IL2RG/HLA-DRA/CD247 | 21 |
| hsa05152 | Tuberculosis | 21/211 | 180/8163 | 6.12E-09 | 9.55E-08 | 7.95E-08 | HLA-DOA/HLA-DMA/ITGB2/CTSS/TLR9/HLA-DMB/CD209/CORO1A/TLR1/CR1/CIITA/HLA-DOB/CD74/TLR6/HLA-DQA1/FCGR2B/HLA-DPB1/CLEC4M/HLA-DPA1/HLA-DRA/BCL2 | 21 |
| hsa04672 | Intestinal immune network for IgA production | 20/211 | 49/8163 | 1.05E-19 | 1.23E-17 | 1.02E-17 | HLA-DOA/CD86/HLA-DMA/HLA-DMB/TNFRSF13B/CXCR4/AICDA/CD40LG/CD80/TNFRSF17/HLA-DOB/TNFSF13B/CD28/HLA-DQA1/HLA-DPB1/CCR9/ICOS/ITGB7/HLA-DPA1/HLA-DRA | 20 |
| hsa05340 | Primary immunodeficiency | 19/211 | 38/8163 | 6.98E-21 | 1.63E-18 | 1.36E-18 | PTPRC/BTK/TNFRSF13B/AICDA/JAK3/CD40LG/AIRE/LCK/CIITA/ZAP70/CD79A/CD19/CD3E/IL7R/CD4/BLNK/CD3D/ICOS/IL2RG | 19 |
| hsa04662 | B cell receptor signaling pathway | 19/211 | 82/8163 | 1.43E-13 | 6.71E-12 | 5.58E-12 | RAC2/PRKCB/PIK3AP1/CR2/BTK/INPP5D/CD22/CD79B/RASGRP3/CD79A/CD19/FCGR2B/CARD11/LILRB1/VAV1/BLNK/PLCG2/LILRA4/CD72 | 19 |
| hsa04658 | Th1 and Th2 cell differentiation | 19/211 | 92/8163 | 1.33E-12 | 4.43E-11 | 3.69E-11 | HLA-DOA/HLA-DMA/HLA-DMB/JAK3/LAT/LCK/ZAP70/HLA-DOB/CD3E/HLA-DQA1/CD4/IL12RB1/CD3D/HLA-DPB1/CD3G/HLA-DPA1/IL2RG/HLA-DRA/CD247 | 19 |
| hsa04064 | NF-kappa B signaling pathway | 19/211 | 104/8163 | 1.33E-11 | 3.88E-10 | 3.23E-10 | PRKCB/BTK/BIRC3/CD40LG/LAT/LTB/BCL2A1/LCK/CCL21/ZAP70/ATM/TNFSF13B/CARD11/BLNK/LTA/PLCG2/TRAF5/BCL2/CCL19 | 19 |
| hsa04660 | T cell receptor signaling pathway | 18/211 | 104/8163 | 1.20E-10 | 2.35E-09 | 1.95E-09 | PTPRC/GRAP2/CD40LG/LAT/RASGRP1/LCK/ZAP70/LCP2/CD28/CD3E/CARD11/VAV1/CD4/CD3D/CD3G/ICOS/ITK/CD247 | 18 |
| hsa05416 | Viral myocarditis | 15/211 | 60/8163 | 1.78E-11 | 4.17E-10 | 3.47E-10 | RAC2/HLA-DOA/CD86/HLA-DMA/ITGB2/HLA-DMB/CD40LG/CD80/HLA-DOB/ITGAL/CD28/HLA-DQA1/HLA-DPB1/HLA-DPA1/DRA | 15 |
| hsa05323 | Rheumatoid arthritis | 15/211 | 93/8163 | 1.23E-08 | 1.60E-07 | 1.33E-07 | HLA-DOA/CD86/HLA-DMA/ITGB2/HLA-DMB/LTB/CD80/HLA-DOB/ITGAL/TNFSF13B/CD28/HLA-DQA1/HLA-DPB1/HLA-DPA1/DRA | 15 |
| hsa05330 | Allograft rejection | 12/211 | 38/8163 | 9.76E-11 | 2.08E-09 | 1.73E-09 | HLA-DOA/CD86/HLA-DMA/HLA-DMB/CD40LG/CD80/HLA-DOB/CD28/HLA-DQA1/HLA-DPB1/HLA-DPA1/HLA-DRA | 12 |
| hsa04940 | Type I diabetes mellitus | 12/211 | 43/8163 | 4.94E-10 | 8.88E-09 | 7.39E-09 | HLA-DOA/CD86/HLA-DMA/HLA-DMB/CD80/HLA-DOB/CD28/HLA-DQA1/LTA/HLA-DPB1/HLA-DPA1/HLA-DRA | 12 |
| hsa05320 | Autoimmune thyroid disease | 12/211 | 53/8163 | 6.84E-09 | 1.00E-07 | 8.32E-08 | HLA-DOA/CD86/HLA-DMA/HLA-DMB/CD40LG/CD80/HLA-DOB/CD28/HLA-DQA1/HLA-DPB1/HLA-DPA1/HLA-DRA | 12 |
| hsa05332 | Graft-versus-host disease | 11/211 | 42/8163 | 5.62E-09 | 9.40E-08 | 7.82E-08 | HLA-DOA/CD86/HLA-DMA/HLA-DMB/CD80/HLA-DOB/CD28/HLA-DQA1/HLA-DPB1/HLA-DPA1/HLA-DRA | 11 |
| hsa05310 | Asthma | 9/211 | 31/8163 | 5.35E-08 | 6.25E-07 | 5.21E-07 | HLA-DOA/HLA-DMA/HLA-DMB/CD40LG/HLA-DOB/HLA-DQA1/HLA-DPB1/HLA-DPA1/HLA-DRA | 9 |
| **The top20 enriched pathways of down-regulated DEGs in comparison analyses of LN_met_ versus PT.** | | | | | | | | |
| **ID** | **Description** | **GeneRatio** | **BgRatio** | ***P*-value** | ***P*.adjust** | ***q-*value** | **geneID** | **Count** |
| hsa04974 | Protein digestion and absorption | 9/66 | 103/8163 | 1.16E-07 | 1.36E-05 | 1.11E-05 | COL5A3/COL12A1/COL4A6/COL7A1/COL24A1/COL6A1/COL15A1/COL9A3/COL6A2 | 9 |
| hsa04510 | Focal adhesion | 8/66 | 201/8163 | 0.000198332 | 0.011602418 | 0.009499056 | COL4A6/COL6A1/MYL9/FLNC/MYLK/COL9A3/LAMC3/COL6A2 | 8 |
| hsa04151 | PI3K-Akt signaling pathway | 8/66 | 354/8163 | 0.00737172 | 0.095404627 | 0.078109052 | FGF5/IGF2/COL4A6/COL6A1/FGF19/COL9A3/LAMC3/COL6A2 | 8 |
| hsa04360 | Axon guidance | 7/66 | 182/8163 | 0.000626371 | 0.017942553 | 0.014689809 | WNT5A/TRPC6/MYL9/ROBO2/RGMA/BOC/SEMA3B | 7 |
| hsa05205 | Proteoglycans in cancer | 7/66 | 205/8163 | 0.001261889 | 0.021091577 | 0.017267958 | TWIST2/IGF2/WNT5A/MMP2/WNT2/FLNC/HPSE2 | 7 |
| hsa05165 | Human papillomavirus infection | 7/66 | 331/8163 | 0.016930559 | 0.152375035 | 0.124751491 | WNT5A/COL4A6/WNT2/COL6A1/COL9A3/LAMC3/COL6A2 | 7 |
| hsa04020 | Calcium signaling pathway | 6/66 | 240/8163 | 0.012660191 | 0.134658395 | 0.110246639 | FGF5/CASQ2/LHCGR/FGF19/CACNA1H/MYLK | 6 |
| hsa04512 | ECM-receptor interaction | 5/66 | 88/8163 | 0.000692028 | 0.017942553 | 0.014689809 | COL4A6/COL6A1/COL9A3/LAMC3/COL6A2 | 5 |
| hsa05410 | Hypertrophic cardiomyopathy | 5/66 | 90/8163 | 0.000766776 | 0.017942553 | 0.014689809 | DES/ACTC1/TPM2/SGCA/SGCD | 5 |
| hsa05414 | Dilated cardiomyopathy | 5/66 | 96/8163 | 0.001027465 | 0.020035563 | 0.016403385 | DES/ACTC1/TPM2/SGCA/SGCD | 5 |
| hsa04270 | Vascular smooth muscle contraction | 5/66 | 134/8163 | 0.004436115 | 0.064878186 | 0.053116643 | MRVI1/ACTG2/MYL9/MYH11/MYLK | 5 |
| hsa04934 | Cushing syndrome | 5/66 | 155/8163 | 0.008154242 | 0.095404627 | 0.078109052 | LDLR/WNT5A/WNT2/KCNK3/CACNA1H | 5 |
| hsa04810 | Regulation of actin cytoskeleton | 5/66 | 218/8163 | 0.031078702 | 0.198903833 | 0.162845244 | FGF5/MYL9/FGF19/MYH11/MYLK | 5 |
| hsa05224 | Breast cancer | 4/66 | 147/8163 | 0.030768623 | 0.198903833 | 0.162845244 | FGF5/WNT5A/WNT2/FGF19 | 4 |
| hsa05226 | Gastric cancer | 4/66 | 149/8163 | 0.032112302 | 0.198903833 | 0.162845244 | FGF5/WNT5A/WNT2/FGF19 | 4 |
| hsa04927 | Cortisol synthesis and secretion | 3/66 | 65/8163 | 0.01543889 | 0.150529176 | 0.123240261 | LDLR/KCNK3/CACNA1H | 3 |
| hsa05412 | Arrhythmogenic right ventricular cardiomyopathy | 3/66 | 77/8163 | 0.024150413 | 0.198903833 | 0.162845244 | DES/SGCA/SGCD | 3 |
| hsa04260 | Cardiac muscle contraction | 3/66 | 87/8163 | 0.033067719 | 0.198903833 | 0.162845244 | CASQ2/ACTC1/TPM2 | 3 |
| hsa00410 | beta-Alanine metabolism | 2/66 | 31/8163 | 0.025749996 | 0.198903833 | 0.162845244 | AOC3/ALDH1B1 | 2 |
| hsa00350 | Tyrosine metabolism | 2/66 | 36/8163 | 0.034000655 | 0.198903833 | 0.162845244 | AOC3/TYRP1 | 2 |
