# Supplementary material for: Multi‐omics analyses reveal spatial heterogeneity in primary and metastatic oesophageal squamous cell carcinoma
Source: Clin Transl Med. 2023 Nov 27;13(11):e1493. doi: 10.1002/ctm2.1493 (PMC10679972; doi:10.1002/ctm2.1493)
Supplement: Supplementary file 23 — Table S12. Expression of immunomodulators across distinct regions. [file CTM2-13-e1493-s015.docx]

**Supplementary Table 12. Expression of immunomodulators across distinct regions.**

| **Gene_ID** | Ajusted P value PTsup vs. PTdeep | Ajusted P value LNmet vs. PTdeep | Ajusted P value PTsup vs. LNmet | Ajusted P value PT vs. LNmet |
| --- | --- | --- | --- | --- |
| ***CD48*** | 1 | 0.001384319 | 2.09678E-06 | 1.64193E-06 |
| ***CD28*** | 1 | 0.084336683 | 0.002420277 | 0.002194975 |
| ***BTLA*** | 1 | 0.012838968 | 0.015867206 | 0.002284294 |
| ***CD80*** | 1 | 0.176549783 | 9.27908E-05 | 0.00400628 |
| ***CD40LG*** | 1 | 0.088527028 | 0.005292839 | 0.007742394 |
| ***CD86*** | 1 | 0.054638101 | 0.019452105 | 0.00863014 |
| ***SLAMF7*** | 1 | 0.258482792 | 0.003845988 | 0.017377482 |
| ***TIGIT*** | 1 | 0.248094168 | 0.011864352 | 0.019211116 |
| ***ICOS*** | 1 | 0.248834565 | 0.066913135 | 0.034311851 |
| ***CD276*** | 1 | 0.261677755 | 0.079306304 | 0.036305934 |
| ***TNFRSF9*** | 1 | 0.277336035 | 0.142704396 | 0.040302777 |
| ***ADORA2A*** | 1 | 0.441762528 | 0.026756582 | 0.055396817 |
| ***CD27*** | 1 | 0.290265857 | 0.072915041 | 0.063491459 |
| ***CTLA4*** | 1 | 0.429954363 | 0.071771648 | 0.103054755 |
| ***CD40*** | 1 | 0.432252279 | 0.285590301 | 0.154276237 |
| ***PDCD1LG2*** | 1 | 0.751006893 | 0.107987297 | 0.18552892 |
| ***HAVCR2*** | 1 | 0.863180212 | 0.112840887 | 0.186883015 |
| ***PVR*** | 1 | 0.687616182 | 0.259343926 | 0.20859662 |
| ***IDO1*** | 1 | 0.627315694 | 0.427192119 | 0.33684438 |
| ***LAG3*** | 1 | 0.685449377 | 0.511638792 | 0.406213649 |
| ***LGALS9*** | 1 | 0.753951859 | 0.425944017 | 0.409734428 |
| ***CD47*** | 1 | 0.969766048 | 0.543313575 | 0.547770576 |
| ***PDCD1*** | 1 | 1 | 0.515477875 | 0.624921142 |
| ***CD274*** | 1 | 1 | 0.633526793 | 0.77895119 |
| ***ICOSLG*** | 1 | 1 | 0.924675 | 0.792509963 |
| ***TNFRSF14*** | 1 | 1 | 0.645794511 | 0.861273 |
| ***VTCN1*** | 1 | 1 | 0.589081528 | 0.99488154 |
| ***TNFSF4*** | 1 | 1 | 0.680501033 | 1 |
| ***IGSF11*** | 1 | 1 | 1 | 1 |
| ***CD70*** | 1 | 1 | 1 | 1 |
| ***TNFSF9*** | 1 | 1 | 1 | 1 |
| ***TNFRSF18*** | 1 | 1 | 1 | 1 |
| ***SIRPA*** | 1 | 1 | 1 | 1 |
