# Supplementary material for: Multi‐omics analyses reveal spatial heterogeneity in primary and metastatic oesophageal squamous cell carcinoma
Source: Clin Transl Med. 2023 Nov 27;13(11):e1493. doi: 10.1002/ctm2.1493 (PMC10679972; doi:10.1002/ctm2.1493)
Supplement: Supplementary file 24 — Table S13. The proportion of CD11c, CD8 and CD20 staining positive cells in each subregion. [file CTM2-13-e1493-s024.docx]

**Supplementary Table 13. The proportion of CD11c, CD8, and CD20 staining positive cells in each subregion.**

| **Patient_ID** | **The Proportion of CD11c Staining Positive Cells (%)** | | | **The Proportion of CD8 Staining Positive Cells (%)** | | | **The Proportion of CD20 Staining Positive Cells**  **(%)** | | |
| --- | --- | --- | --- | --- | --- | --- | --- | --- | --- |
|  | PTsup | PTdeep | LNmet | PTsup | PTdeep | LNmet | PTsup | PTdeep | LNmet |
| P253 | 8.31 | 14.9 | 33.99 | 7.35 | 5 | 11.88 | 5.62 | 3.05 | 8 |
| P316 | 17.27 | 18.07 | 17.82 | 14 | 11.01 | 33 | 3.05 | 5.35 | 10.02 |
| P937 | 20.98 | 21.29 | 40.24 | 11.7 | 9.71 | 14 | 2.63 | 7 | 15.72 |
| P926 | 14.55 | 19.84 | 69.51 | 3.34 | 9.2 | 8.69 | 11.29 | 10.23 | 47.2 |
| P351 | 23.71 | 24.01 | 34.66 | 12.51 | 10.62 | 43.17 | 13.6 | 12.07 | 43.54 |
| P768 | 31.47 | 32.47 | 72.41 | 14.32 | 14.37 | 40.32 | 15.05 | 11.24 | 45.17 |
| P848 | 15.82 | 20.24 | 88.26 | 11.35 | 15.62 | 49.62 | 17.82 | 16.35 | 19.88 |
| P334 | 34.66 | 31.61 | 35.56 | 22.61 | 20.33 | 50.43 | 16.83 | 18.94 | 23.57 |
| P035 | 43.05 | 32.3 | 15.81 | 27.51 | 18.5 | 20.05 | 12.9 | 9.21 | 21.27 |
| P270 | 19.03 | 18.51 | 28.28 | 32.27 | 27 | 30.62 | 20.64 | 12.41 | 40.16 |
| P348 | 23.21 | 24.27 | 64.32 | 10.68 | 15.44 | 16.52 | 21.57 | 7.6 | 13.17 |
| P685 | 16.87 | 17 | 61.07 | 21.65 | 20 | 18 | 11.62 | 8.31 | 47 |
| P575 | 11.41 | 9.57 | 60.24 | 7.37 | 21.57 | 10.54 | 17.03 | 10.26 | 19.04 |
