# Supplementary material for: Multi‐omics analyses reveal spatial heterogeneity in primary and metastatic oesophageal squamous cell carcinoma
Source: Clin Transl Med. 2023 Nov 27;13(11):e1493. doi: 10.1002/ctm2.1493 (PMC10679972; doi:10.1002/ctm2.1493)
Supplement: Supplementary file 25 — Table 14. Probe details in the NanoString Human Immune Cell Profiling panel. [file CTM2-13-e1493-s005.docx]

**Supplementary Table 14. Probe details in the NanoString Human Immune Cell Profiling panel.**

| **Protein Name** | **Gene** | **Protein ID** | **Target Notes** |
| --- | --- | --- | --- |
| PD-1 | PDCD1 | DPROT_00004.1 |  |
| CD68 | CD68 | DPROT_00006.1 |  |
| HLA-DR | CD74 | DPROT_00007.1 |  |
| Ki-67 | MKI67 | DPROT_00009.1 |  |
| Beta-2-microglobulin | B2M | DPROT_00010.1 |  |
| CD11c | ITGAX | DPROT_00011.1 |  |
| CD20 | MS4A1 | DPROT_00012.1 |  |
| CD3 | CD3G,CD3E,CD3D | DPROT_00013.1 |  |
| CD4 | CD4 | DPROT_00014.1 |  |
| CD45 | PTPRC | DPROT_00015.1 |  |
| CD56 | NCAM1 | DPROT_00016.1 |  |
| CD8 | CD8A | DPROT_00017.1 |  |
| CTLA4 | CTLA4 | DPROT_00395.1 |  |
| GZMB | GZMB | DPROT_00019.1 | High Background |
| PD-L1 | CD274 | DPROT_00021.1 |  |
| PanCK | KRT1,KRT10,KRT2,KRT16, KRT5,KRT6B,KRT19,KRT6A, KRT8,KRT14,KRT3 | DPROT_00022.1 |  |
| SMA | ACTA2 | DPROT_00023.1 |  |
| Fibronectin | FN1 | DPROT_00024.1 |  |
| **Controls** |  |  |  |
| Rb IgG |  | DPROT_00001.1 | Negative Control |
| Ms IgG1 |  | DPROT_00002.1 | Negative Control |
| Ms IgG2a |  | DPROT_00003.1 | Negative Control |
| Histone H3 | HIST1H3A | DPROT_00005.1 | Positive Control |
| S6 | RPS6 | DPROT_00008.1 | Positive Control |
| GAPDH | GAPDH | DPROT_00020.1 | Positive Control |
