# Supplementary material for: Multi‐omics analyses reveal spatial heterogeneity in primary and metastatic oesophageal squamous cell carcinoma
Source: Clin Transl Med. 2023 Nov 27;13(11):e1493. doi: 10.1002/ctm2.1493 (PMC10679972; doi:10.1002/ctm2.1493)
Supplement: Supplementary file 26 — Table S15. Information of all AOIs. [file CTM2-13-e1493-s030.docx]

**Supplementary Table 15. Information of all AOIs.**

| **Patient** | **Cancer_Position** | **Segment_Label** | **TMA_Position** | **Scan_ID** | **ROI_ID** | **AOI_ID** | **Cluster_Tree** |
| --- | --- | --- | --- | --- | --- | --- | --- |
| P481 | PTdeep | Stroma | D6 | 21R8355SLZA-2 | 33 | 21R8355SLZA-2_033_Stroma | C2 |
| P481 | PTdeep | Tumor | D6 | 21R8355SLZA-2 | 33 | 21R8355SLZA-2_033_Tumor | C3 |
| P481 | PTdeep | Stroma | D4 | 21R8355SLZA-2 | 31 | 21R8355SLZA-2_031_Stroma | C2 |
| P481 | PTdeep | Tumor | D4 | 21R8355SLZA-2 | 31 | 21R8355SLZA-2_031_Tumor | C3 |
| P937 | PTdeep | Stroma | D3 | 21R8355SLZA-2 | 30 | 21R8355SLZA-2_030_Stroma | C2 |
| P937 | PTdeep | Tumor | D3 | 21R8355SLZA-2 | 30 | 21R8355SLZA-2_030_Tumor | C3 |
| P937 | PTdeep | Stroma | D2 | 21R8355SLZA-2 | 29 | 21R8355SLZA-2_029_Stroma | C2 |
| P937 | PTdeep | Tumor | D2 | 21R8355SLZA-2 | 29 | 21R8355SLZA-2_029_Tumor | C3 |
| P937 | PTdeep | Stroma | D1 | 21R8355SLZA-2 | 28 | 21R8355SLZA-2_028_Stroma | C2 |
| P937 | PTdeep | Tumor | D1 | 21R8355SLZA-2 | 28 | 21R8355SLZA-2_028_Tumor | C3 |
| P541 | PTdeep | Stroma | C9 | 21R8355SLZA-2 | 27 | 21R8355SLZA-2_027_Stroma | C2 |
| P541 | PTdeep | Tumor | C9 | 21R8355SLZA-2 | 27 | 21R8355SLZA-2_027_Tumor | C3 |
| P541 | PTdeep | Stroma | C8 | 21R8355SLZA-2 | 26 | 21R8355SLZA-2_026_Stroma | C2 |
| P541 | PTdeep | Tumor | C8 | 21R8355SLZA-2 | 26 | 21R8355SLZA-2_026_Tumor | C3 |
| P541 | PTdeep | Stroma | C7 | 21R8355SLZA-2 | 25 | 21R8355SLZA-2_025_Stroma | C2 |
| P541 | PTdeep | Tumor | C7 | 21R8355SLZA-2 | 25 | 21R8355SLZA-2_025_Tumor | C3 |
| P435 | PTdeep | Stroma | C6 | 21R8355SLZA-2 | 24 | 21R8355SLZA-2_024_Stroma | C1 |
| P435 | PTdeep | Tumor | C6 | 21R8355SLZA-2 | 24 | 21R8355SLZA-2_024_Tumor | C3 |
| P435 | PTdeep | Stroma | C5 | 21R8355SLZA-2 | 23 | 21R8355SLZA-2_023_Stroma | C2 |
| P435 | PTdeep | Tumor | C5 | 21R8355SLZA-2 | 23 | 21R8355SLZA-2_023_Tumor | C3 |
| P435 | PTdeep | Stroma | C4 | 21R8355SLZA-2 | 22 | 21R8355SLZA-2_022_Stroma | C1 |
| P435 | PTdeep | Tumor | C4 | 21R8355SLZA-2 | 22 | 21R8355SLZA-2_022_Tumor | C3 |
| P324 | PTdeep | Stroma | C3 | 21R8355SLZA-2 | 21 | 21R8355SLZA-2_021_Stroma | C2 |
| P324 | PTdeep | Tumor | C3 | 21R8355SLZA-2 | 21 | 21R8355SLZA-2_021_Tumor | C3 |
| P324 | PTdeep | Stroma | C2 | 21R8355SLZA-2 | 20 | 21R8355SLZA-2_020_Stroma | C2 |
| P324 | PTdeep | Tumor | C2 | 21R8355SLZA-2 | 20 | 21R8355SLZA-2_020_Tumor | C3 |
| P324 | PTdeep | Tumor | C1 | 21R8355SLZA-2 | 19 | 21R8355SLZA-2_019_Tumor | C3 |
| P685 | PTdeep | Stroma | B9 | 21R8355SLZA-2 | 18 | 21R8355SLZA-2_018_Stroma | C2 |
| P685 | PTdeep | Tumor | B9 | 21R8355SLZA-2 | 18 | 21R8355SLZA-2_018_Tumor | C3 |
| P685 | PTdeep | Stroma | B8 | 21R8355SLZA-2 | 17 | 21R8355SLZA-2_017_Stroma | C1 |
| P685 | PTdeep | Tumor | B8 | 21R8355SLZA-2 | 17 | 21R8355SLZA-2_017_Tumor | C3 |
| P685 | PTdeep | Stroma | B7 | 21R8355SLZA-2 | 16 | 21R8355SLZA-2_016_Stroma | C2 |
| P685 | PTdeep | Tumor | B7 | 21R8355SLZA-2 | 16 | 21R8355SLZA-2_016_Tumor | C3 |
| P879 | PTdeep | Stroma | B6 | 21R8355SLZA-2 | 15 | 21R8355SLZA-2_015_Stroma | C1 |
| P879 | PTdeep | Tumor | B6 | 21R8355SLZA-2 | 15 | 21R8355SLZA-2_015_Tumor | C3 |
| P879 | PTdeep | Stroma | B5 | 21R8355SLZA-2 | 14 | 21R8355SLZA-2_014_Stroma | C2 |
| P879 | PTdeep | Tumor | B5 | 21R8355SLZA-2 | 14 | 21R8355SLZA-2_014_Tumor | C3 |
| P879 | PTdeep | Stroma | B4 | 21R8355SLZA-2 | 13 | 21R8355SLZA-2_013_Stroma | C2 |
| P879 | PTdeep | Tumor | B4 | 21R8355SLZA-2 | 13 | 21R8355SLZA-2_013_Tumor | C3 |
| P653 | PTdeep | Stroma | B3 | 21R8355SLZA-2 | 12 | 21R8355SLZA-2_012_Stroma | C1 |
| P653 | PTdeep | Tumor | B3 | 21R8355SLZA-2 | 12 | 21R8355SLZA-2_012_Tumor | C3 |
| P653 | PTdeep | Stroma | B2 | 21R8355SLZA-2 | 11 | 21R8355SLZA-2_011_Stroma | C1 |
| P653 | PTdeep | Tumor | B2 | 21R8355SLZA-2 | 11 | 21R8355SLZA-2_011_Tumor | C3 |
| P653 | PTdeep | Stroma | B1 | 21R8355SLZA-2 | 10 | 21R8355SLZA-2_010_Stroma | C1 |
| P653 | PTdeep | Tumor | B1 | 21R8355SLZA-2 | 10 | 21R8355SLZA-2_010_Tumor | C3 |
| P786 | PTdeep | Stroma | A9 | 21R8355SLZA-2 | 9 | 21R8355SLZA-2_009_Stroma | C2 |
| P786 | PTdeep | Tumor | A9 | 21R8355SLZA-2 | 9 | 21R8355SLZA-2_009_Tumor | C3 |
| P786 | PTdeep | Stroma | A8 | 21R8355SLZA-2 | 8 | 21R8355SLZA-2_008_Stroma | C1 |
| P786 | PTdeep | Tumor | A8 | 21R8355SLZA-2 | 8 | 21R8355SLZA-2_008_Tumor | C3 |
| P786 | PTdeep | Stroma | A7 | 21R8355SLZA-2 | 7 | 21R8355SLZA-2_007_Stroma | C2 |
| P786 | PTdeep | Tumor | A7 | 21R8355SLZA-2 | 7 | 21R8355SLZA-2_007_Tumor | C3 |
| P768 | PTdeep | Stroma | A6 | 21R8355SLZA-2 | 6 | 21R8355SLZA-2_006_Stroma | C1 |
| P768 | PTdeep | Tumor | A6 | 21R8355SLZA-2 | 6 | 21R8355SLZA-2_006_Tumor | C3 |
| P768 | PTdeep | Stroma | A5 | 21R8355SLZA-2 | 5 | 21R8355SLZA-2_005_Stroma | C1 |
| P768 | PTsup | Tumor | A5 | 21R8355SLZA-2 | 5 | 21R8355SLZA-2_005_Tumor | C3 |
| P768 | PTsup | Stroma | A4 | 21R8355SLZA-2 | 4 | 21R8355SLZA-2_004_Stroma | C1 |
| P768 | PTsup | Tumor | A4 | 21R8355SLZA-2 | 4 | 21R8355SLZA-2_004_Tumor | C3 |
| P253 | PTsup | Stroma | A3 | 21R8355SLZA-2 | 3 | 21R8355SLZA-2_003_Stroma | C2 |
| P253 | PTsup | Tumor | A3 | 21R8355SLZA-2 | 3 | 21R8355SLZA-2_003_Tumor | C3 |
| P253 | PTsup | Stroma | A2 | 21R8355SLZA-2 | 2 | 21R8355SLZA-2_002_Stroma | C2 |
| P253 | PTsup | Tumor | A2 | 21R8355SLZA-2 | 2 | 21R8355SLZA-2_002_Tumor | C3 |
| P253 | PTsup | Stroma | A1 | 21R8355SLZA-2 | 1 | 21R8355SLZA-2_001_Stroma | C2 |
| P253 | PTsup | Tumor | A1 | 21R8355SLZA-2 | 1 | 21R8355SLZA-2_001_Tumor | C3 |
| P575 | PTdeep | Stroma | F2 | 21R8355SLZA-2 | 50 | 21R8355SLZA-2_050_Stroma | C2 |
| P575 | PTdeep | Stroma | F1 | 21R8355SLZA-2 | 49 | 21R8355SLZA-2_049_Stroma | C2 |
| P575 | PTdeep | Stroma | F3 | 21R8355SLZA-2 | 48 | 21R8355SLZA-2_048_Stroma | C2 |
| P575 | PTdeep | Tumor | F3 | 21R8355SLZA-2 | 48 | 21R8355SLZA-2_048_Tumor | C3 |
| P575 | PTdeep | Tumor | F2 | 21R8355SLZA-2 | 47 | 21R8355SLZA-2_047_Tumor | C3 |
| P575 | PTdeep | Tumor | F1 | 21R8355SLZA-2 | 46 | 21R8355SLZA-2_046_Tumor | C3 |
| P848 | PTdeep | Stroma | E9 | 21R8355SLZA-2 | 45 | 21R8355SLZA-2_045_Stroma | C2 |
| P848 | PTdeep | Tumor | E9 | 21R8355SLZA-2 | 45 | 21R8355SLZA-2_045_Tumor | C3 |
| P848 | PTdeep | Stroma | E8 | 21R8355SLZA-2 | 44 | 21R8355SLZA-2_044_Stroma | C1 |
| P848 | PTdeep | Tumor | E8 | 21R8355SLZA-2 | 44 | 21R8355SLZA-2_044_Tumor | C3 |
| P848 | PTdeep | Stroma | E7 | 21R8355SLZA-2 | 43 | 21R8355SLZA-2_043_Stroma | C2 |
| P848 | PTdeep | Tumor | E7 | 21R8355SLZA-2 | 43 | 21R8355SLZA-2_043_Tumor | C3 |
| P348 | PTdeep | Tumor | E6 | 21R8355SLZA-2 | 42 | 21R8355SLZA-2_042_Tumor | C3 |
| P348 | PTdeep | Stroma | E5 | 21R8355SLZA-2 | 41 | 21R8355SLZA-2_041_Stroma | C2 |
| P348 | PTdeep | Tumor | E5 | 21R8355SLZA-2 | 41 | 21R8355SLZA-2_041_Tumor | C3 |
| P348 | PTdeep | Stroma | E4 | 21R8355SLZA-2 | 40 | 21R8355SLZA-2_040_Stroma | C2 |
| P348 | PTdeep | Tumor | E4 | 21R8355SLZA-2 | 40 | 21R8355SLZA-2_040_Tumor | C3 |
| P334 | PTdeep | Stroma | E3 | 21R8355SLZA-2 | 39 | 21R8355SLZA-2_039_Stroma | C2 |
| P334 | PTdeep | Tumor | E3 | 21R8355SLZA-2 | 39 | 21R8355SLZA-2_039_Tumor | C3 |
| P334 | PTdeep | Stroma | E2 | 21R8355SLZA-2 | 38 | 21R8355SLZA-2_038_Stroma | C2 |
| P334 | PTdeep | Tumor | E2 | 21R8355SLZA-2 | 38 | 21R8355SLZA-2_038_Tumor | C3 |
| P334 | PTdeep | Stroma | E1 | 21R8355SLZA-2 | 37 | 21R8355SLZA-2_037_Stroma | C2 |
| P334 | PTdeep | Tumor | E1 | 21R8355SLZA-2 | 37 | 21R8355SLZA-2_037_Tumor | C3 |
| P035 | PTdeep | Stroma | D9 | 21R8355SLZA-2 | 36 | 21R8355SLZA-2_036_Stroma | C2 |
| P035 | PTdeep | Tumor | D9 | 21R8355SLZA-2 | 36 | 21R8355SLZA-2_036_Tumor | C3 |
| P035 | PTdeep | Stroma | D8 | 21R8355SLZA-2 | 35 | 21R8355SLZA-2_035_Stroma | C2 |
| P035 | PTdeep | Tumor | D8 | 21R8355SLZA-2 | 35 | 21R8355SLZA-2_035_Tumor | C3 |
| P035 | PTdeep | Stroma | D7 | 21R8355SLZA-2 | 34 | 21R8355SLZA-2_034_Stroma | C1 |
| P035 | PTdeep | Tumor | D7 | 21R8355SLZA-2 | 34 | 21R8355SLZA-2_034_Tumor | C3 |
| P879 | LNmet | Tumor | B6 | 21R3908SLZA-2 | 13 | 21R3908SLZA-2_013_Tumor | C3 |
| P879 | LNmet | Tumor | B5 | 21R3908SLZA-2 | 14 | 21R3908SLZA-2_014_Tumor | C3 |
| P879 | LNmet | Stroma | B5 | 21R3908SLZA-2 | 14 | 21R3908SLZA-2_014_Stroma | C1 |
| P879 | LNmet | Tumor | B4 | 21R3908SLZA-2 | 15 | 21R3908SLZA-2_015_Tumor | C3 |
| P879 | LNmet | Stroma | B4 | 21R3908SLZA-2 | 15 | 21R3908SLZA-2_015_Stroma | C1 |
| P653 | LNmet | Tumor | B3 | 21R3908SLZA-2 | 16 | 21R3908SLZA-2_016_Tumor | C3 |
| P653 | LNmet | Stroma | B3 | 21R3908SLZA-2 | 16 | 21R3908SLZA-2_016_Stroma | C1 |
| P653 | LNmet | Tumor | B2 | 21R3908SLZA-2 | 17 | 21R3908SLZA-2_017_Tumor | C3 |
| P653 | LNmet | Stroma | B2 | 21R3908SLZA-2 | 17 | 21R3908SLZA-2_017_Stroma | C1 |
| P653 | LNmet | Tumor | B1 | 21R3908SLZA-2 | 18 | 21R3908SLZA-2_018_Tumor | C3 |
| P653 | LNmet | Stroma | B1 | 21R3908SLZA-2 | 18 | 21R3908SLZA-2_018_Stroma | C1 |
| P324 | LNmet | Tumor | C1 | 21R3908SLZA-2 | 19 | 21R3908SLZA-2_019_Tumor | C3 |
| P324 | LNmet | Stroma | C1 | 21R3908SLZA-2 | 19 | 21R3908SLZA-2_019_Stroma | C1 |
| P324 | LNmet | Tumor | C2 | 21R3908SLZA-2 | 20 | 21R3908SLZA-2_020_Tumor | C3 |
| P324 | LNmet | Stroma | C2 | 21R3908SLZA-2 | 20 | 21R3908SLZA-2_020_Stroma | C1 |
| P324 | LNmet | Tumor | C3 | 21R3908SLZA-2 | 21 | 21R3908SLZA-2_021_Tumor | C3 |
| P324 | LNmet | Stroma | C3 | 21R3908SLZA-2 | 21 | 21R3908SLZA-2_021_Stroma | C1 |
| P435 | LNmet | Tumor | C4 | 21R3908SLZA-2 | 22 | 21R3908SLZA-2_022_Tumor | C3 |
| P435 | LNmet | Stroma | C4 | 21R3908SLZA-2 | 22 | 21R3908SLZA-2_022_Stroma | C1 |
| P435 | LNmet | Tumor | C5 | 21R3908SLZA-2 | 23 | 21R3908SLZA-2_023_Tumor | C3 |
| P435 | LNmet | Stroma | C5 | 21R3908SLZA-2 | 23 | 21R3908SLZA-2_023_Stroma | C1 |
| P435 | LNmet | Tumor | C6 | 21R3908SLZA-2 | 24 | 21R3908SLZA-2_024_Tumor | C3 |
| P435 | LNmet | Stroma | C6 | 21R3908SLZA-2 | 24 | 21R3908SLZA-2_024_Stroma | C1 |
| P541 | LNmet | Tumor | C7 | 21R3908SLZA-2 | 25 | 21R3908SLZA-2_025_Tumor | C3 |
| P541 | LNmet | Stroma | C7 | 21R3908SLZA-2 | 25 | 21R3908SLZA-2_025_Stroma | C2 |
| P541 | LNmet | Tumor | C8 | 21R3908SLZA-2 | 26 | 21R3908SLZA-2_026_Tumor | C3 |
| P541 | LNmet | Stroma | C8 | 21R3908SLZA-2 | 26 | 21R3908SLZA-2_026_Stroma | C2 |
| P541 | LNmet | Tumor | C9 | 21R3908SLZA-2 | 27 | 21R3908SLZA-2_027_Tumor | C3 |
| P541 | LNmet | Stroma | C9 | 21R3908SLZA-2 | 27 | 21R3908SLZA-2_027_Stroma | C2 |
| P035 | LNmet | Tumor | D9 | 21R3908SLZA-2 | 28 | 21R3908SLZA-2_028_Tumor | C3 |
| P035 | LNmet | Tumor | D8 | 21R3908SLZA-2 | 29 | 21R3908SLZA-2_029_Tumor | C3 |
| P035 | LNmet | Stroma | D8 | 21R3908SLZA-2 | 29 | 21R3908SLZA-2_029_Stroma | C1 |
| P575 | LNmet | Tumor | F3 | 21R3908SLZA-2 | 46 | 21R3908SLZA-2_046_Tumor | C3 |
| P575 | LNmet | Tumor | F2 | 21R3908SLZA-2 | 45 | 21R3908SLZA-2_045_Tumor | C3 |
| P575 | LNmet | Stroma | F1 | 21R3908SLZA-2 | 44 | 21R3908SLZA-2_044_Stroma | C2 |
| P575 | LNmet | Tumor | F1 | 21R3908SLZA-2 | 44 | 21R3908SLZA-2_044_Tumor | C3 |
| P848 | LNmet | Stroma | E9 | 21R3908SLZA-2 | 43 | 21R3908SLZA-2_043_Stroma | C1 |
| P848 | LNmet | Tumor | E9 | 21R3908SLZA-2 | 43 | 21R3908SLZA-2_043_Tumor | C3 |
| P848 | LNmet | Stroma | E8 | 21R3908SLZA-2 | 42 | 21R3908SLZA-2_042_Stroma | C1 |
| P848 | LNmet | Tumor | E8 | 21R3908SLZA-2 | 42 | 21R3908SLZA-2_042_Tumor | C3 |
| P848 | LNmet | Stroma | E7 | 21R3908SLZA-2 | 41 | 21R3908SLZA-2_041_Stroma | C1 |
| P848 | LNmet | Tumor | E7 | 21R3908SLZA-2 | 41 | 21R3908SLZA-2_041_Tumor | C3 |
| P348 | LNmet | Stroma | E6 | 21R3908SLZA-2 | 40 | 21R3908SLZA-2_040_Stroma | C1 |
| P348 | LNmet | Tumor | E6 | 21R3908SLZA-2 | 40 | 21R3908SLZA-2_040_Tumor | C3 |
| P348 | LNmet | Stroma | E4 | 21R3908SLZA-2 | 39 | 21R3908SLZA-2_039_Stroma | C1 |
| P348 | LNmet | Tumor | E4 | 21R3908SLZA-2 | 39 | 21R3908SLZA-2_039_Tumor | C3 |
| P334 | LNmet | Stroma | E2 | 21R3908SLZA-2 | 38 | 21R3908SLZA-2_038_Stroma | C1 |
| P334 | LNmet | Tumor | E2 | 21R3908SLZA-2 | 38 | 21R3908SLZA-2_038_Tumor | C3 |
| P334 | LNmet | Stroma | E1 | 21R3908SLZA-2 | 37 | 21R3908SLZA-2_037_Stroma | C1 |
| P334 | LNmet | Tumor | E1 | 21R3908SLZA-2 | 37 | 21R3908SLZA-2_037_Tumor | C3 |
| P937 | LNmet | Tumor | D1 | 21R3908SLZA-2 | 36 | 21R3908SLZA-2_036_Tumor | C3 |
| P937 | LNmet | Stroma | D2 | 21R3908SLZA-2 | 35 | 21R3908SLZA-2_035_Stroma | C1 |
| P937 | LNmet | Tumor | D2 | 21R3908SLZA-2 | 35 | 21R3908SLZA-2_035_Tumor | C3 |
| P937 | LNmet | Stroma | D3 | 21R3908SLZA-2 | 34 | 21R3908SLZA-2_034_Stroma | C2 |
| P937 | LNmet | Tumor | D3 | 21R3908SLZA-2 | 34 | 21R3908SLZA-2_034_Tumor | C3 |
| P481 | LNmet | Stroma | D4 | 21R3908SLZA-2 | 33 | 21R3908SLZA-2_033_Stroma | C1 |
| P481 | LNmet | Tumor | D5 | 21R3908SLZA-2 | 33 | 21R3908SLZA-2_033_Tumor | C3 |
| P481 | LNmet | Stroma | D5 | 21R3908SLZA-2 | 32 | 21R3908SLZA-2_032_Stroma | C2 |
| P035 | LNmet | Tumor | D7 | 21R3908SLZA-2 | 30 | 21R3908SLZA-2_030_Tumor | C3 |
| P035 | LNmet | Stroma | D7 | 21R3908SLZA-2 | 30 | 21R3908SLZA-2_030_Stroma | C1 |
| P481 | LNmet | Tumor | D6 | 21R3908SLZA-2 | 31 | 21R3908SLZA-2_031_Tumor | C3 |
| P481 | LNmet | Stroma | D6 | 21R3908SLZA-2 | 31 | 21R3908SLZA-2_031_Stroma | C1 |
| P481 | LNmet | Tumor | D5 | 21R3908SLZA-2 | 32 | 21R3908SLZA-2_032_Tumor | C3 |
| P685 | LNmet | Tumor | B9 | 21R3908SLZA-2 | 1 | 21R3908SLZA-2_001_Tumor | C3 |
| P685 | LNmet | Stroma | B9 | 21R3908SLZA-2 | 1 | 21R3908SLZA-2_001_Stroma | C1 |
| P253 | LNmet | Tumor | A1 | 21R3908SLZA-2 | 2 | 21R3908SLZA-2_002_Tumor | C3 |
| P253 | LNmet | Stroma | A1 | 21R3908SLZA-2 | 2 | 21R3908SLZA-2_002_Stroma | C2 |
| P253 | LNmet | Tumor | A2 | 21R3908SLZA-2 | 3 | 21R3908SLZA-2_003_Tumor | C3 |
| P253 | LNmet | Stroma | A2 | 21R3908SLZA-2 | 3 | 21R3908SLZA-2_003_Stroma | C1 |
| P253 | LNmet | Tumor | A3 | 21R3908SLZA-2 | 4 | 21R3908SLZA-2_004_Tumor | C3 |
| P768 | LNmet | Tumor | A4 | 21R3908SLZA-2 | 5 | 21R3908SLZA-2_005_Tumor | C3 |
| P768 | LNmet | Stroma | A4 | 21R3908SLZA-2 | 5 | 21R3908SLZA-2_005_Stroma | C1 |
| P768 | LNmet | Tumor | A5 | 21R3908SLZA-2 | 6 | 21R3908SLZA-2_006_Tumor | C3 |
| P768 | LNmet | Stroma | A5 | 21R3908SLZA-2 | 6 | 21R3908SLZA-2_006_Stroma | C1 |
| P768 | LNmet | Stroma | A6 | 21R3908SLZA-2 | 7 | 21R3908SLZA-2_007_Stroma | C1 |
| P786 | LNmet | Tumor | A7 | 21R3908SLZA-2 | 8 | 21R3908SLZA-2_008_Tumor | C3 |
| P786 | LNmet | Stroma | A7 | 21R3908SLZA-2 | 8 | 21R3908SLZA-2_008_Stroma | C2 |
| P786 | LNmet | Tumor | A8 | 21R3908SLZA-2 | 9 | 21R3908SLZA-2_009_Tumor | C3 |
| P786 | LNmet | Stroma | A9 | 21R3908SLZA-2 | 10 | 21R3908SLZA-2_010_Stroma | C1 |
| P685 | LNmet | Stroma | B8 | 21R3908SLZA-2 | 11 | 21R3908SLZA-2_011_Stroma | C1 |
| P685 | LNmet | Tumor | B7 | 21R3908SLZA-2 | 12 | 21R3908SLZA-2_012_Tumor | C3 |
| P685 | LNmet | Stroma | B7 | 21R3908SLZA-2 | 12 | 21R3908SLZA-2_012_Stroma | C1 |
| P879 | LNmet | Stroma | B6 | 21R3908SLZA-2 | 13 | 21R3908SLZA-2_013_Stroma | C1 |
| P253 | PTsup | Stroma | A2 | 21R6148SLZA-2 | 2 | 21R6148SLZA-2_002_Stroma | C2 |
| P575 | PTsup | Stroma | F3 | 21R6148SLZA-2 | 45 | 21R6148SLZA-2_045_Stroma | C2 |
| P575 | PTsup | Tumor | F3 | 21R6148SLZA-2 | 45 | 21R6148SLZA-2_045_Tumor | C3 |
| P575 | PTsup | Stroma | F2 | 21R6148SLZA-2 | 44 | 21R6148SLZA-2_044_Stroma | C1 |
| P575 | PTsup | Tumor | F2 | 21R6148SLZA-2 | 44 | 21R6148SLZA-2_044_Tumor | C3 |
| P575 | PTsup | Stroma | F1 | 21R6148SLZA-2 | 43 | 21R6148SLZA-2_043_Stroma | C1 |
| P575 | PTsup | Tumor | F1 | 21R6148SLZA-2 | 43 | 21R6148SLZA-2_043_Tumor | C3 |
| P848 | PTsup | Stroma | E9 | 21R6148SLZA-2 | 42 | 21R6148SLZA-2_042_Stroma | C2 |
| P848 | PTsup | Tumor | E9 | 21R6148SLZA-2 | 42 | 21R6148SLZA-2_042_Tumor | C3 |
| P848 | PTsup | Stroma | E8 | 21R6148SLZA-2 | 41 | 21R6148SLZA-2_041_Stroma | C1 |
| P848 | PTsup | Tumor | E8 | 21R6148SLZA-2 | 41 | 21R6148SLZA-2_041_Tumor | C3 |
| P848 | PTsup | Stroma | E7 | 21R6148SLZA-2 | 40 | 21R6148SLZA-2_040_Stroma | C2 |
| P848 | PTsup | Tumor | E7 | 21R6148SLZA-2 | 40 | 21R6148SLZA-2_040_Tumor | C3 |
| P348 | PTsup | Stroma | E6 | 21R6148SLZA-2 | 39 | 21R6148SLZA-2_039_Stroma | C2 |
| P348 | PTsup | Tumor | E6 | 21R6148SLZA-2 | 39 | 21R6148SLZA-2_039_Tumor | C3 |
| P348 | PTsup | Stroma | E5 | 21R6148SLZA-2 | 38 | 21R6148SLZA-2_038_Stroma | C2 |
| P348 | PTsup | Tumor | E5 | 21R6148SLZA-2 | 38 | 21R6148SLZA-2_038_Tumor | C3 |
| P348 | PTsup | Stroma | E4 | 21R6148SLZA-2 | 37 | 21R6148SLZA-2_037_Stroma | C2 |
| P348 | PTsup | Tumor | E4 | 21R6148SLZA-2 | 37 | 21R6148SLZA-2_037_Tumor | C3 |
| P937 | PTsup | Tumor | D1 | 21R6148SLZA-2 | 36 | 21R6148SLZA-2_036_Tumor | C3 |
| P937 | PTsup | Tumor | D2 | 21R6148SLZA-2 | 35 | 21R6148SLZA-2_035_Tumor | C3 |
| P937 | PTsup | Stroma | D3 | 21R6148SLZA-2 | 34 | 21R6148SLZA-2_034_Stroma | C2 |
| P937 | PTsup | Tumor | D3 | 21R6148SLZA-2 | 34 | 21R6148SLZA-2_034_Tumor | C3 |
| P481 | PTsup | Stroma | D4 | 21R6148SLZA-2 | 33 | 21R6148SLZA-2_033_Stroma | C1 |
| P481 | PTsup | Tumor | D4 | 21R6148SLZA-2 | 33 | 21R6148SLZA-2_033_Tumor | C3 |
| P481 | PTsup | Stroma | D5 | 21R6148SLZA-2 | 32 | 21R6148SLZA-2_032_Stroma | C2 |
| P481 | PTsup | Tumor | D5 | 21R6148SLZA-2 | 32 | 21R6148SLZA-2_032_Tumor | C3 |
| P481 | PTsup | Stroma | D6 | 21R6148SLZA-2 | 31 | 21R6148SLZA-2_031_Stroma | C2 |
| P481 | PTsup | Tumor | D6 | 21R6148SLZA-2 | 31 | 21R6148SLZA-2_031_Tumor | C3 |
| P035 | PTsup | Stroma | D7 | 21R6148SLZA-2 | 30 | 21R6148SLZA-2_030_Stroma | C2 |
| P035 | PTsup | Tumor | D7 | 21R6148SLZA-2 | 30 | 21R6148SLZA-2_030_Tumor | C3 |
| P035 | PTsup | Stroma | D8 | 21R6148SLZA-2 | 29 | 21R6148SLZA-2_029_Stroma | C1 |
| P035 | PTsup | Tumor | D8 | 21R6148SLZA-2 | 29 | 21R6148SLZA-2_029_Tumor | C3 |
| P035 | PTsup | Stroma | D9 | 21R6148SLZA-2 | 28 | 21R6148SLZA-2_028_Stroma | C2 |
| P035 | PTsup | Tumor | D9 | 21R6148SLZA-2 | 28 | 21R6148SLZA-2_028_Tumor | C3 |
| P541 | PTsup | Stroma | C9 | 21R6148SLZA-2 | 27 | 21R6148SLZA-2_027_Stroma | C2 |
| P541 | PTsup | Tumor | C9 | 21R6148SLZA-2 | 27 | 21R6148SLZA-2_027_Tumor | C3 |
| P541 | PTsup | Stroma | C8 | 21R6148SLZA-2 | 26 | 21R6148SLZA-2_026_Stroma | C2 |
| P541 | PTsup | Tumor | C8 | 21R6148SLZA-2 | 26 | 21R6148SLZA-2_026_Tumor | C3 |
| P541 | PTsup | Stroma | C7 | 21R6148SLZA-2 | 25 | 21R6148SLZA-2_025_Stroma | C2 |
| P541 | PTsup | Tumor | C7 | 21R6148SLZA-2 | 25 | 21R6148SLZA-2_025_Tumor | C3 |
| P435 | PTsup | Stroma | C6 | 21R6148SLZA-2 | 24 | 21R6148SLZA-2_024_Stroma | C1 |
| P435 | PTsup | Tumor | C6 | 21R6148SLZA-2 | 24 | 21R6148SLZA-2_024_Tumor | C3 |
| P435 | PTsup | Stroma | C5 | 21R6148SLZA-2 | 23 | 21R6148SLZA-2_023_Stroma | C1 |
| P435 | PTsup | Tumor | C5 | 21R6148SLZA-2 | 23 | 21R6148SLZA-2_023_Tumor | C3 |
| P435 | PTsup | Stroma | C4 | 21R6148SLZA-2 | 22 | 21R6148SLZA-2_022_Stroma | C2 |
| P435 | PTsup | Tumor | C4 | 21R6148SLZA-2 | 22 | 21R6148SLZA-2_022_Tumor | C3 |
| P324 | PTsup | Stroma | C3 | 21R6148SLZA-2 | 21 | 21R6148SLZA-2_021_Stroma | C2 |
| P324 | PTsup | Tumor | C3 | 21R6148SLZA-2 | 21 | 21R6148SLZA-2_021_Tumor | C3 |
| P324 | PTsup | Stroma | C2 | 21R6148SLZA-2 | 20 | 21R6148SLZA-2_020_Stroma | C2 |
| P324 | PTsup | Tumor | C2 | 21R6148SLZA-2 | 20 | 21R6148SLZA-2_020_Tumor | C3 |
| P324 | PTsup | Stroma | C1 | 21R6148SLZA-2 | 19 | 21R6148SLZA-2_019_Stroma | C2 |
| P324 | PTsup | Tumor | C1 | 21R6148SLZA-2 | 19 | 21R6148SLZA-2_019_Tumor | C3 |
| P653 | PTsup | Stroma | B1 | 21R6148SLZA-2 | 18 | 21R6148SLZA-2_018_Stroma | C1 |
| P653 | PTsup | Tumor | B1 | 21R6148SLZA-2 | 18 | 21R6148SLZA-2_018_Tumor | C3 |
| P653 | PTsup | Tumor | B2 | 21R6148SLZA-2 | 17 | 21R6148SLZA-2_017_Tumor | C3 |
| P653 | PTsup | Tumor | B3 | 21R6148SLZA-2 | 16 | 21R6148SLZA-2_016_Tumor | C3 |
| P253 | PTsup | Tumor | A1 | 21R6148SLZA-2 | 1 | 21R6148SLZA-2_001_Tumor | C3 |
| P879 | PTsup | Stroma | B4 | 21R6148SLZA-2 | 15 | 21R6148SLZA-2_015_Stroma | C2 |
| P879 | PTsup | Tumor | B4 | 21R6148SLZA-2 | 15 | 21R6148SLZA-2_015_Tumor | C3 |
| P879 | PTsup | Stroma | B5 | 21R6148SLZA-2 | 14 | 21R6148SLZA-2_014_Stroma | C2 |
| P879 | PTsup | Tumor | B5 | 21R6148SLZA-2 | 14 | 21R6148SLZA-2_014_Tumor | C3 |
| P879 | PTsup | Stroma | B6 | 21R6148SLZA-2 | 13 | 21R6148SLZA-2_013_Stroma | C2 |
| P879 | PTsup | Tumor | B6 | 21R6148SLZA-2 | 13 | 21R6148SLZA-2_013_Tumor | C3 |
| P685 | PTsup | Stroma | B7 | 21R6148SLZA-2 | 12 | 21R6148SLZA-2_012_Stroma | C2 |
| P685 | PTsup | Tumor | B7 | 21R6148SLZA-2 | 12 | 21R6148SLZA-2_012_Tumor | C3 |
| P685 | PTsup | Tumor | B8 | 21R6148SLZA-2 | 11 | 21R6148SLZA-2_011_Tumor | C3 |
| P685 | PTsup | Stroma | B9 | 21R6148SLZA-2 | 10 | 21R6148SLZA-2_010_Stroma | C1 |
| P685 | PTsup | Tumor | B9 | 21R6148SLZA-2 | 10 | 21R6148SLZA-2_010_Tumor | C3 |
| P786 | PTsup | Stroma | A9 | 21R6148SLZA-2 | 9 | 21R6148SLZA-2_009_Stroma | C2 |
| P786 | PTsup | Tumor | A9 | 21R6148SLZA-2 | 9 | 21R6148SLZA-2_009_Tumor | C3 |
| P786 | PTsup | Stroma | A8 | 21R6148SLZA-2 | 8 | 21R6148SLZA-2_008_Stroma | C2 |
| P786 | PTsup | Tumor | A8 | 21R6148SLZA-2 | 8 | 21R6148SLZA-2_008_Tumor | C3 |
| P786 | PTsup | Stroma | A7 | 21R6148SLZA-2 | 7 | 21R6148SLZA-2_007_Stroma | C1 |
| P786 | PTsup | Tumor | A7 | 21R6148SLZA-2 | 7 | 21R6148SLZA-2_007_Tumor | C3 |
| P768 | PTsup | Stroma | A6 | 21R6148SLZA-2 | 6 | 21R6148SLZA-2_006_Stroma | C1 |
| P768 | PTsup | Tumor | A6 | 21R6148SLZA-2 | 6 | 21R6148SLZA-2_006_Tumor | C3 |
| P768 | PTsup | Stroma | A5 | 21R6148SLZA-2 | 5 | 21R6148SLZA-2_005_Stroma | C1 |
| P768 | PTsup | Tumor | A5 | 21R6148SLZA-2 | 5 | 21R6148SLZA-2_005_Tumor | C3 |
| P768 | PTsup | Stroma | A4 | 21R6148SLZA-2 | 4 | 21R6148SLZA-2_004_Stroma | C1 |
| P768 | PTsup | Tumor | A4 | 21R6148SLZA-2 | 4 | 21R6148SLZA-2_004_Tumor | C3 |
| P253 | PTsup | Stroma | A3 | 21R6148SLZA-2 | 3 | 21R6148SLZA-2_003_Stroma | C2 |
| P253 | PTsup | Tumor | A3 | 21R6148SLZA-2 | 3 | 21R6148SLZA-2_003_Tumor | C3 |
| P253 | PTsup | Tumor | A2 | 21R6148SLZA-2 | 2 | 21R6148SLZA-2_002_Tumor | C3 |
| P253 | PTsup | Stroma | A1 | 21R6148SLZA-2 | 1 | 21R6148SLZA-2_001_Stroma | C2 |
| P973 | LNmet | Stroma | D3 | 21R6202SLZA-2 | 29 | 21R6202SLZA-2_029_Stroma | C2 |
| P973 | LNmet | Tumor | D3 | 21R6202SLZA-2 | 29 | 21R6202SLZA-2_029_Tumor | C3 |
| P973 | LNmet | Stroma | D2 | 21R6202SLZA-2 | 28 | 21R6202SLZA-2_028_Stroma | C2 |
| P973 | LNmet | Tumor | D2 | 21R6202SLZA-2 | 28 | 21R6202SLZA-2_028_Tumor | C3 |
| P973 | LNmet | Stroma | D1 | 21R6202SLZA-2 | 27 | 21R6202SLZA-2_027_Stroma | C2 |
| P973 | LNmet | Tumor | D1 | 21R6202SLZA-2 | 27 | 21R6202SLZA-2_027_Tumor | C3 |
| P351 | PTdeep | Stroma | C9 | 21R6202SLZA-2 | 26 | 21R6202SLZA-2_026_Stroma | C2 |
| P351 | PTdeep | Stroma | C8 | 21R6202SLZA-2 | 25 | 21R6202SLZA-2_025_Stroma | C1 |
| P351 | PTdeep | Stroma | C7 | 21R6202SLZA-2 | 24 | 21R6202SLZA-2_024_Stroma | C1 |
| P351 | PTsup | Stroma | C6 | 21R6202SLZA-2 | 23 | 21R6202SLZA-2_023_Stroma | C1 |
| P351 | PTsup | Tumor | C6 | 21R6202SLZA-2 | 23 | 21R6202SLZA-2_023_Tumor | C3 |
| P351 | LNmet | Stroma | C3 | 21R6202SLZA-2 | 20 | 21R6202SLZA-2_020_Stroma | C2 |
| P351 | PTsup | Tumor | C4 | 21R6202SLZA-2 | 21 | 21R6202SLZA-2_021_Tumor | C3 |
| P351 | PTsup | Stroma | C4 | 21R6202SLZA-2 | 21 | 21R6202SLZA-2_021_Stroma | C2 |
| P351 | PTsup | Tumor | C5 | 21R6202SLZA-2 | 22 | 21R6202SLZA-2_022_Tumor | C3 |
| P351 | PTsup | Stroma | C5 | 21R6202SLZA-2 | 22 | 21R6202SLZA-2_022_Stroma | C2 |
| P973 | PTdeep | Stroma | D7 | 21R6202SLZA-2 | 33 | 21R6202SLZA-2_033_Stroma | C2 |
| P973 | PTdeep | Tumor | D8 | 21R6202SLZA-2 | 34 | 21R6202SLZA-2_034_Tumor | C3 |
| P973 | PTdeep | Stroma | D8 | 21R6202SLZA-2 | 34 | 21R6202SLZA-2_034_Stroma | C2 |
| P973 | PTdeep | Tumor | D9 | 21R6202SLZA-2 | 35 | 21R6202SLZA-2_035_Tumor | C3 |
| P973 | PTdeep | Stroma | D9 | 21R6202SLZA-2 | 35 | 21R6202SLZA-2_035_Stroma | C2 |
| P270 | LNmet | Stroma | E1 | 21R6202SLZA-2 | 36 | 21R6202SLZA-2_036_Stroma | C1 |
| P270 | LNmet | Stroma | E2 | 21R6202SLZA-2 | 37 | 21R6202SLZA-2_037_Stroma | C2 |
| P270 | LNmet | Stroma | E3 | 21R6202SLZA-2 | 38 | 21R6202SLZA-2_038_Stroma | C1 |
| P270 | PTsup | Stroma | E4 | 21R6202SLZA-2 | 39 | 21R6202SLZA-2_039_Stroma | C1 |
| P270 | PTsup | Stroma | E5 | 21R6202SLZA-2 | 40 | 21R6202SLZA-2_040_Stroma | C1 |
| P270 | PTsup | Tumor | E6 | 21R6202SLZA-2 | 41 | 21R6202SLZA-2_041_Tumor | C3 |
| P270 | PTsup | Stroma | E6 | 21R6202SLZA-2 | 41 | 21R6202SLZA-2_041_Stroma | C2 |
| P270 | PTdeep | Stroma | E7 | 21R6202SLZA-2 | 42 | 21R6202SLZA-2_042_Stroma | C2 |
| P270 | PTdeep | Tumor | E8 | 21R6202SLZA-2 | 43 | 21R6202SLZA-2_043_Tumor | C3 |
| P270 | PTdeep | Stroma | E9 | 21R6202SLZA-2 | 44 | 21R6202SLZA-2_044_Stroma | C2 |
| P270 | PTdeep | Stroma | E8 | 21R6202SLZA-2 | 43 | 21R6202SLZA-2_043_Stroma | C2 |
| P270 | PTdeep | Tumor | E9 | 21R6202SLZA-2 | 44 | 21R6202SLZA-2_044_Tumor | C3 |
| P316 | PTsup | Tumor | B5 | 21R6202SLZA-2 | 13 | 21R6202SLZA-2_013_Tumor | C3 |
| P316 | PTsup | Stroma | B5 | 21R6202SLZA-2 | 13 | 21R6202SLZA-2_013_Stroma | C1 |
| P316 | PTsup | Tumor | B6 | 21R6202SLZA-2 | 14 | 21R6202SLZA-2_014_Tumor | C3 |
| P316 | PTsup | Stroma | B6 | 21R6202SLZA-2 | 14 | 21R6202SLZA-2_014_Stroma | C2 |
| P316 | PTdeep | Tumor | B7 | 21R6202SLZA-2 | 15 | 21R6202SLZA-2_015_Tumor | C3 |
| P316 | PTdeep | Stroma | B7 | 21R6202SLZA-2 | 15 | 21R6202SLZA-2_015_Stroma | C2 |
| P316 | PTdeep | Tumor | B8 | 21R6202SLZA-2 | 16 | 21R6202SLZA-2_016_Tumor | C3 |
| P316 | PTdeep | Stroma | B8 | 21R6202SLZA-2 | 16 | 21R6202SLZA-2_016_Stroma | C1 |
| P316 | PTdeep | Tumor | B9 | 21R6202SLZA-2 | 17 | 21R6202SLZA-2_017_Tumor | C3 |
| P316 | PTdeep | Stroma | B9 | 21R6202SLZA-2 | 17 | 21R6202SLZA-2_017_Stroma | C1 |
| P351 | LNmet | Tumor | C1 | 21R6202SLZA-2 | 18 | 21R6202SLZA-2_018_Tumor | C3 |
| P351 | LNmet | Stroma | C1 | 21R6202SLZA-2 | 18 | 21R6202SLZA-2_018_Stroma | C1 |
| P351 | LNmet | Tumor | C2 | 21R6202SLZA-2 | 19 | 21R6202SLZA-2_019_Tumor | C3 |
| P351 | LNmet | Stroma | C2 | 21R6202SLZA-2 | 19 | 21R6202SLZA-2_019_Stroma | C1 |
| P253 | PTdeep | Tumor | A1 | 21R6202SLZA-2 | 1 | 21R6202SLZA-2_001_Tumor | C3 |
| P253 | PTdeep | Stroma | A1 | 21R6202SLZA-2 | 1 | 21R6202SLZA-2_001_Stroma | C1 |
| P253 | PTdeep | Tumor | A2 | 21R6202SLZA-2 | 2 | 21R6202SLZA-2_002_Tumor | C3 |
| P253 | PTdeep | Stroma | A2 | 21R6202SLZA-2 | 2 | 21R6202SLZA-2_002_Stroma | C1 |
| P253 | PTdeep | Tumor | A3 | 21R6202SLZA-2 | 3 | 21R6202SLZA-2_003_Tumor | C3 |
| P253 | PTdeep | Stroma | A3 | 21R6202SLZA-2 | 3 | 21R6202SLZA-2_003_Stroma | C1 |
| P768 | PTdeep | Tumor | A4 | 21R6202SLZA-2 | 4 | 21R6202SLZA-2_004_Tumor | C3 |
| P768 | PTdeep | Stroma | A4 | 21R6202SLZA-2 | 4 | 21R6202SLZA-2_004_Stroma | C1 |
| P768 | PTdeep | Tumor | A5 | 21R6202SLZA-2 | 5 | 21R6202SLZA-2_005_Tumor | C3 |
| P768 | PTdeep | Stroma | A5 | 21R6202SLZA-2 | 5 | 21R6202SLZA-2_005_Stroma | C1 |
| P768 | PTdeep | Tumor | A6 | 21R6202SLZA-2 | 6 | 21R6202SLZA-2_006_Tumor | C3 |
| P768 | PTdeep | Stroma | A6 | 21R6202SLZA-2 | 6 | 21R6202SLZA-2_006_Stroma | C1 |
| P926 | PTdeep | Tumor | A9 | 21R6202SLZA-2 | 7 | 21R6202SLZA-2_007_Tumor | C3 |
| P926 | PTdeep | Stroma | A9 | 21R6202SLZA-2 | 7 | 21R6202SLZA-2_007_Stroma | C1 |
| P937 | LNmet | Tumor | B1 | 21R6202SLZA-2 | 8 | 21R6202SLZA-2_008_Tumor | C3 |
| P937 | LNmet | Tumor | B2 | 21R6202SLZA-2 | 9 | 21R6202SLZA-2_009_Tumor | C3 |
| P937 | LNmet | Stroma | B2 | 21R6202SLZA-2 | 9 | 21R6202SLZA-2_009_Stroma | C2 |
| P937 | LNmet | Tumor | B3 | 21R6202SLZA-2 | 10 | 21R6202SLZA-2_010_Tumor | C3 |
| P937 | LNmet | Stroma | B3 | 21R6202SLZA-2 | 11 | 21R6202SLZA-2_011_Stroma | C2 |
| P316 | PTsup | Tumor | B4 | 21R6202SLZA-2 | 12 | 21R6202SLZA-2_012_Tumor | C3 |
| P316 | PTsup | Stroma | B4 | 21R6202SLZA-2 | 12 | 21R6202SLZA-2_012_Stroma | C2 |
| P973 | PTdeep | Tumor | D7 | 21R6202SLZA-2 | 33 | 21R6202SLZA-2_033_Tumor | C3 |
| P973 | PTsup | Stroma | D6 | 21R6202SLZA-2 | 32 | 21R6202SLZA-2_032_Stroma | C2 |
| P973 | PTsup | Tumor | D6 | 21R6202SLZA-2 | 32 | 21R6202SLZA-2_032_Tumor | C3 |
| P973 | PTsup | Stroma | D5 | 21R6202SLZA-2 | 31 | 21R6202SLZA-2_031_Stroma | C2 |
| P973 | PTsup | Tumor | D5 | 21R6202SLZA-2 | 31 | 21R6202SLZA-2_031_Tumor | C3 |
| P973 | PTsup | Stroma | D4 | 21R6202SLZA-2 | 30 | 21R6202SLZA-2_030_Stroma | C1 |
| P973 | PTsup | Tumor | D4 | 21R6202SLZA-2 | 30 | 21R6202SLZA-2_030_Tumor | C3 |
