# Supplementary material for: Multi‐omics analyses reveal spatial heterogeneity in primary and metastatic oesophageal squamous cell carcinoma
Source: Clin Transl Med. 2023 Nov 27;13(11):e1493. doi: 10.1002/ctm2.1493 (PMC10679972; doi:10.1002/ctm2.1493)
Supplement: Supplementary file 27 — Table S16. Differential proteins with FDR < .05 between subregions in the whole area, stromal and tumour compartments, respectively. [file CTM2-13-e1493-s029.docx]

**Supplementary Table 16. Differential proteins with FDR < 0.05 between subregions in the whole area, stromal, and tumor compartments, respectively.**

| **Differential proteins between PT_sup_ and PT_deep_ through mimic bulk sequencing in the whole area** | | | | | | | |
| --- | --- | --- | --- | --- | --- | --- | --- |
| Protein | PT_sup__Mean | PT_deep__Mean | Mean_difference | Log2FC | *P*-value | FDR | Change |
| CTLA4 | 38.56625669 | 65.55135476 | 26.98509806 | 0.765286475 | 1.59E-11 | 1.43E-10 | UP |
| PD-1 | 119.211483 | 100.1282114 | -19.08327162 | -0.251674695 | 9.04E-12 | 1.43E-10 | DOWN |
| GZMB | 456.8114371 | 332.5287905 | -124.2826465 | -0.458119516 | 2.75E-07 | 1.65E-06 | DOWN |
| CD56 | 200.3265159 | 179.7992038 | -20.52731213 | -0.155966762 | 0.00010976 | 0.000493922 | DOWN |
| CD20 | 55.84834041 | 50.00988164 | -5.83845877 | -0.15930122 | 0.000722949 | 0.002602617 | DOWN |
| PanCk | 8932.671082 | 6094.584747 | -2838.086335 | -0.551563714 | 0.010616669 | 0.031850006 | DOWN |
| Beta-2-microglobulin | 258.1954047 | 241.7071412 | -16.48826354 | -0.095203226 | 0.069530895 | 0.178793729 | NOT |
| PD-L1 | 60.66960503 | 69.11473431 | 8.44512928 | 0.188019387 | 0.079501085 | 0.17887744 | NOT |
| CD8 | 611.7403484 | 594.2705316 | -17.46981681 | -0.041799592 | 0.160877394 | 0.321754788 | NOT |
| Ki-67 | 1574.627785 | 1345.314564 | -229.3132212 | -0.227067295 | 0.180602938 | 0.325085288 | NOT |
| CD68 | 881.703784 | 942.3318604 | 60.62807645 | 0.095941171 | 0.23035999 | 0.376952712 | NOT |
| HLA-DR | 950.9733761 | 1176.607192 | 225.6338155 | 0.307155903 | 0.428253962 | 0.642380943 | NOT |
| SMA | 16050.47697 | 17298.83298 | 1248.356004 | 0.108058543 | 0.517544295 | 0.716599792 | NOT |
| CD11c | 508.1581568 | 604.7052119 | 96.54705516 | 0.250954429 | 0.575660234 | 0.730897158 | NOT |
| CD4 | 405.2130762 | 403.7869312 | -1.426145007 | -0.005086513 | 0.609080965 | 0.730897158 | NOT |
| CD3 | 367.2256307 | 385.5380068 | 18.31237608 | 0.070206334 | 0.660761561 | 0.743356756 | NOT |
| CD45 | 1709.490007 | 1633.487562 | -76.00244514 | -0.06561052 | 0.754728793 | 0.799124604 | NOT |
| Fibronectin | 3787.068516 | 3715.094236 | -71.97427993 | -0.027682717 | 0.989614157 | 0.989614157 | NOT |
| **Differential proteins between PT_sup_ and PT_deep_ in stromal compartment** | | | | | | | |
| Protein | PT_sup__Mean | PT_deep__Mean | Mean_difference | Log2FC | *P*-value | FDR | Change |
| CTLA4 | 38.56625669 | 65.55135476 | 26.98509806 | 0.765286475 | 1.59E-11 | 1.43E-10 | UP |
| PD-1 | 119.211483 | 100.1282114 | -19.08327162 | -0.251674695 | 9.04E-12 | 1.43E-10 | DOWN |
| GZMB | 456.8114371 | 332.5287905 | -124.2826465 | -0.458119516 | 2.75E-07 | 1.65E-06 | DOWN |
| CD56 | 200.3265159 | 179.7992038 | -20.52731213 | -0.155966762 | 0.00010976 | 0.000493922 | DOWN |
| CD20 | 55.84834041 | 50.00988164 | -5.83845877 | -0.15930122 | 0.000722949 | 0.002602617 | DOWN |
| PanCk | 8932.671082 | 6094.584747 | -2838.086335 | -0.551563714 | 0.010616669 | 0.031850006 | DOWN |
| Beta-2-microglobulin | 258.1954047 | 241.7071412 | -16.48826354 | -0.095203226 | 0.069530895 | 0.178793729 | NOT |
| PD-L1 | 60.66960503 | 69.11473431 | 8.44512928 | 0.188019387 | 0.079501085 | 0.17887744 | NOT |
| CD8 | 611.7403484 | 594.2705316 | -17.46981681 | -0.041799592 | 0.160877394 | 0.321754788 | NOT |
| Ki-67 | 1574.627785 | 1345.314564 | -229.3132212 | -0.227067295 | 0.180602938 | 0.325085288 | NOT |
| CD68 | 881.703784 | 942.3318604 | 60.62807645 | 0.095941171 | 0.23035999 | 0.376952712 | NOT |
| HLA-DR | 950.9733761 | 1176.607192 | 225.6338155 | 0.307155903 | 0.428253962 | 0.642380943 | NOT |
| SMA | 16050.47697 | 17298.83298 | 1248.356004 | 0.108058543 | 0.517544295 | 0.716599792 | NOT |
| CD11c | 508.1581568 | 604.7052119 | 96.54705516 | 0.250954429 | 0.575660234 | 0.730897158 | NOT |
| CD4 | 405.2130762 | 403.7869312 | -1.426145007 | -0.005086513 | 0.609080965 | 0.730897158 | NOT |
| CD3 | 367.2256307 | 385.5380068 | 18.31237608 | 0.070206334 | 0.660761561 | 0.743356756 | NOT |
| CD45 | 1709.490007 | 1633.487562 | -76.00244514 | -0.06561052 | 0.754728793 | 0.799124604 | NOT |
| Fibronectin | 3787.068516 | 3715.094236 | -71.97427993 | -0.027682717 | 0.989614157 | 0.989614157 | NOT |
| **Differential proteins between PT_sup_ and PT_deep_ in tumor compartment** | | | | | | | |
| Protein | PT_sup__Mean | PT_deep__Mean | Mean_difference | Log2FC | *P*-value | FDR | Change |
| CTLA4 | 27.92013174 | 43.93125524 | 16.01112349 | 0.653941973 | 1.40E-08 | 1.26E-07 | UP |
| PD-1 | 126.1697018 | 103.8558413 | -22.31386042 | -0.280783142 | 4.48E-09 | 8.07E-08 | DOWN |
| CD20 | 55.29532013 | 47.16274124 | -8.132578895 | -0.22950981 | 2.78E-05 | 0.00016668 | DOWN |
| GZMB | 463.3352252 | 359.146907 | -104.1883182 | -0.367482277 | 0.000267781 | 0.001205013 | DOWN |
| PanCk | 15811.46521 | 11004.6141 | -4806.851108 | -0.52286251 | 0.001283181 | 0.004619452 | DOWN |
| CD56 | 193.3610874 | 171.6493702 | -21.71171718 | -0.171832927 | 0.002192015 | 0.006576045 | DOWN |
| CD68 | 682.0923954 | 611.5313302 | -70.56106519 | -0.157540765 | 0.068215931 | 0.175412395 | NOT |
| PD-L1 | 55.89882875 | 72.05089136 | 16.15206261 | 0.366198224 | 0.099654406 | 0.224222413 | NOT |
| CD8 | 422.5790245 | 442.4809114 | 19.90188687 | 0.06639406 | 0.210580482 | 0.421160963 | NOT |
| Ki-67 | 2457.552389 | 2139.057712 | -318.4946771 | -0.200246765 | 0.308069426 | 0.554524966 | NOT |
| CD4 | 249.4197386 | 253.4301712 | 4.010432599 | 0.023012648 | 0.41396123 | 0.573177087 | NOT |
| Fibronectin | 1714.179614 | 1772.580636 | 58.40102218 | 0.048332973 | 0.405342338 | 0.573177087 | NOT |
| SMA | 3176.628713 | 3659.491068 | 482.8623548 | 0.204146548 | 0.402493378 | 0.573177087 | NOT |
| CD45 | 743.4650262 | 809.9488214 | 66.48379516 | 0.123565873 | 0.593570984 | 0.712285181 | NOT |
| Beta-2-microglobulin | 232.1653219 | 226.1427439 | -6.022577977 | -0.03791879 | 0.576188522 | 0.712285181 | NOT |
| CD11c | 354.5618248 | 361.9422019 | 7.380377049 | 0.029722122 | 0.829779095 | 0.87858963 | NOT |
| HLA-DR | 509.7379472 | 577.3631818 | 67.62523464 | 0.179723353 | 0.81403894 | 0.87858963 | NOT |
| CD3 | 160.4557386 | 193.4104077 | 32.95466913 | 0.269490043 | 0.961672053 | 0.961672053 | NOT |
| **Differential proteins between LN_met_ and PT_sup_ through mimic bulk sequencing in the whole area** | | | | | | | |
| Protein | LN_met_ _Mean | PT_sup_ _Mean | Mean_difference | Log2FC | *P*-value | FDR | Change |
| Fibronectin | 2897.796521 | 3787.068516 | 889.2719947 | 0.386125228 | 0.00071579 | 0.001288421 | UP |
| CD11c | 779.4948473 | 508.1581568 | -271.3366905 | -0.617261902 | 1.98E-09 | 3.56E-08 | DOWN |
| HLA-DR | 1777.533341 | 950.9733761 | -826.5599649 | -0.902399764 | 1.60E-08 | 1.44E-07 | DOWN |
| CD20 | 72.30120974 | 55.84834041 | -16.45286932 | -0.372505376 | 3.90E-07 | 2.34E-06 | DOWN |
| Beta-2-microglobulin | 315.5352045 | 258.1954047 | -57.33979977 | -0.289337652 | 1.56E-06 | 7.00E-06 | DOWN |
| CD45 | 3132.033349 | 1709.490007 | -1422.543342 | -0.873533584 | 5.08E-06 | 1.83E-05 | DOWN |
| CD8 | 1024.262439 | 611.7403484 | -412.5220908 | -0.743594074 | 9.19E-06 | 2.76E-05 | DOWN |
| CD56 | 237.1365563 | 200.3265159 | -36.81004035 | -0.243364688 | 7.35E-05 | 0.000189044 | DOWN |
| PD-L1 | 71.11127748 | 60.66960503 | -10.44167245 | -0.229104455 | 0.000435767 | 0.000980475 | DOWN |
| CD3 | 574.3357808 | 367.2256307 | -207.1101502 | -0.645227688 | 0.000642499 | 0.001284998 | DOWN |
| CD4 | 512.6107826 | 405.2130762 | -107.3977065 | -0.339183095 | 0.001264622 | 0.002069381 | DOWN |
| SMA | 9460.457734 | 16050.47697 | 6590.019239 | 0.762634277 | 0.049864376 | 0.074796564 | NOT |
| CTLA4 | 44.13106649 | 38.56625669 | -5.564809798 | -0.19445549 | 0.089297172 | 0.123642239 | NOT |
| PanCk | 6529.642594 | 8932.671082 | 2403.028488 | 0.452087614 | 0.098166723 | 0.126214358 | NOT |
| CD68 | 933.3654431 | 881.703784 | -51.66165917 | -0.082148003 | 0.178145018 | 0.203285403 | NOT |
| PD-1 | 124.8555795 | 119.211483 | -5.644096452 | -0.066737083 | 0.180698136 | 0.203285403 | NOT |
| GZMB | 477.2903794 | 456.8114371 | -20.47894231 | -0.063268485 | 0.213481522 | 0.226039259 | NOT |
| Ki-67 | 1570.953476 | 1574.627785 | 3.674308795 | 0.003370385 | 0.45826825 | 0.45826825 | NOT |
| **Differential proteins between LN_met_ and PT_sup_ in stromal compartment** | | | | | | | |
| Protein | LN_met_ _Mean | PT_sup_ _Mean | Mean_difference | Log2FC | *P*-value | FDR | Change |
| SMA | 15779.32278 | 30053.61017 | 14274.28739 | 0.929503015 | 1.11E-06 | 2.00E-05 | UP |
| Fibronectin | 4234.36438 | 6041.789777 | 1807.425397 | 0.512830564 | 0.000139864 | 0.000419592 | UP |
| HLA-DR | 2615.975648 | 1430.913667 | -1185.061981 | -0.87041248 | 3.48E-06 | 3.13E-05 | DOWN |
| CD20 | 82.02134307 | 56.44987124 | -25.57147183 | -0.539029077 | 5.95E-06 | 3.57E-05 | DOWN |
| CD8 | 1488.150478 | 817.4947709 | -670.6557075 | -0.864239008 | 6.41E-05 | 0.000288425 | DOWN |
| CD11c | 987.8715998 | 675.2278512 | -312.6437486 | -0.548949125 | 0.000127101 | 0.000419592 | DOWN |
| CD45 | 4914.216861 | 2760.254022 | -2153.962839 | -0.832160482 | 0.000218756 | 0.000562515 | DOWN |
| Beta-2-microglobulin | 346.1228441 | 286.5088282 | -59.61401586 | -0.272704569 | 0.000423144 | 0.000952075 | DOWN |
| Ki-67 | 832.3444073 | 614.2536543 | -218.090753 | -0.438346074 | 0.000983817 | 0.001967633 | DOWN |
| CD3 | 921.9523521 | 592.1332326 | -329.8191195 | -0.638770367 | 0.003208545 | 0.00577538 | DOWN |
| CD56 | 247.0445106 | 207.902947 | -39.14156358 | -0.248860791 | 0.004583727 | 0.007500644 | DOWN |
| PD-L1 | 67.45082279 | 65.85887046 | -1.591952333 | -0.034458273 | 0.009192206 | 0.01378831 | DOWN |
| GZMB | 495.373407 | 449.7153868 | -45.6580202 | -0.139504177 | 0.046455781 | 0.064323389 | NOT |
| CD4 | 706.942048 | 574.6724961 | -132.2695519 | -0.298851951 | 0.066087448 | 0.084969576 | NOT |
| PD-1 | 119.5292628 | 111.6428943 | -7.886368563 | -0.098472427 | 0.105332752 | 0.126399302 | NOT |
| CTLA4 | 53.58081971 | 50.14625226 | -3.434567454 | -0.095574769 | 0.127225811 | 0.143129037 | NOT |
| PanCk | 1538.926421 | 1450.473963 | -88.45245729 | -0.085399856 | 0.142611755 | 0.151000682 | NOT |
| CD68 | 1101.37287 | 1098.824943 | -2.547926626 | -0.003341411 | 0.82030902 | 0.82030902 | NOT |
| **Differential proteins between LN_met_ and PT_sup_ in tumor compartment** | | | | | | | |
| Protein | LN_met_ _Mean | PT_sup_ _Mean | Mean_difference | Log2FC | *P*-value | FDR | Change |
| PanCk | 11176.17144 | 15811.46521 | 4635.293763 | 0.500545007 | 0.002552733 | 0.00574365 | UP |
| CD11c | 585.4889053 | 354.5618248 | -230.9270805 | -0.723604621 | 3.27E-07 | 5.89E-06 | DOWN |
| HLA-DR | 996.9146412 | 509.7379472 | -487.176694 | -0.967714224 | 1.49E-06 | 1.34E-05 | DOWN |
| CD45 | 1472.759044 | 743.4650262 | -729.2940182 | -0.986184631 | 7.39E-06 | 4.43E-05 | DOWN |
| CD4 | 331.6816735 | 249.4197386 | -82.26193495 | -0.411223659 | 0.000102952 | 0.000463285 | DOWN |
| Beta-2-microglobulin | 287.0570573 | 232.1653219 | -54.89173545 | -0.306185029 | 0.000421217 | 0.001516383 | DOWN |
| CD3 | 250.6927662 | 160.4557386 | -90.2370276 | -0.64374498 | 0.000543898 | 0.001631693 | DOWN |
| CD8 | 592.3666785 | 422.5790245 | -169.787654 | -0.487269331 | 0.001039553 | 0.002673136 | DOWN |
| CD56 | 227.9119092 | 193.3610874 | -34.55082181 | -0.237178821 | 0.005974271 | 0.011948543 | DOWN |
| CD20 | 63.25143042 | 55.29532013 | -7.956110289 | -0.193940722 | 0.007575714 | 0.013636285 | DOWN |
| PD-L1 | 74.51928702 | 55.89882875 | -18.62045827 | -0.414795817 | 0.014084424 | 0.023047239 | DOWN |
| CD68 | 776.9447353 | 682.0923954 | -94.85233988 | -0.187844804 | 0.089342034 | 0.134013051 | NOT |
| Fibronectin | 1653.405756 | 1714.179614 | 60.77385844 | 0.052077471 | 0.158526958 | 0.219498865 | NOT |
| Ki-67 | 2258.623988 | 2457.552389 | 198.9284004 | 0.121778058 | 0.324787684 | 0.417584165 | NOT |
| CTLA4 | 35.33302039 | 27.92013174 | -7.41288865 | -0.339711332 | 0.390544905 | 0.468653886 | NOT |
| PD-1 | 129.814564 | 126.1697018 | -3.64486222 | -0.041086744 | 0.601301408 | 0.676464084 | NOT |
| SMA | 3577.376485 | 3176.628713 | -400.7477723 | -0.17140548 | 0.868610225 | 0.868610225 | NOT |
| GZMB | 460.4544571 | 463.3352252 | 2.880768112 | 0.008997899 | 0.868610225 | 0.868610225 | NOT |
| **Differential proteins between LN_met_ and PT_deep_ through mimic bulk sequencing in the whole area** | | | | | | | |
| Protein | LN_met_ _Mean | PT_deep_ _Mean | Mean_difference | Log2FC | *P*-value | FDR | Change |
| CTLA4 | 44.13106649 | 65.55135476 | 21.42028827 | 0.570830985 | 1.98E-06 | 4.12E-06 | UP |
| Fibronectin | 2897.796521 | 3715.094236 | 817.2977148 | 0.358442512 | 0.00039805 | 0.000597075 | UP |
| SMA | 9460.457734 | 17298.83298 | 7838.375243 | 0.87069282 | 0.011285866 | 0.0145104 | UP |
| CD68 | 933.3654431 | 942.3318604 | 8.966417283 | 0.013793168 | 0.019731643 | 0.023677971 | UP |
| PD-1 | 124.8555795 | 100.1282114 | -24.72736807 | -0.318411778 | 1.34E-14 | 1.32E-13 | DOWN |
| CD20 | 72.30120974 | 50.00988164 | -22.29132809 | -0.531806596 | 1.46E-14 | 1.32E-13 | DOWN |
| CD56 | 237.1365563 | 179.7992038 | -57.33735248 | -0.399331449 | 6.78E-11 | 4.07E-10 | DOWN |
| GZMB | 477.2903794 | 332.5287905 | -144.7615888 | -0.521388001 | 9.90E-10 | 4.45E-09 | DOWN |
| Beta-2-microglobulin | 315.5352045 | 241.7071412 | -73.82806331 | -0.384540879 | 8.27E-09 | 2.98E-08 | DOWN |
| CD8 | 1024.262439 | 594.2705316 | -429.9919076 | -0.785393666 | 4.31E-07 | 1.29E-06 | DOWN |
| CD45 | 3132.033349 | 1633.487562 | -1498.545787 | -0.939144104 | 1.13E-06 | 2.90E-06 | DOWN |
| CD11c | 779.4948473 | 604.7052119 | -174.7896354 | -0.366307473 | 2.06E-06 | 4.12E-06 | DOWN |
| HLA-DR | 1777.533341 | 1176.607192 | -600.9261495 | -0.595243861 | 5.49E-06 | 9.88E-06 | DOWN |
| CD4 | 512.6107826 | 403.7869312 | -108.8238515 | -0.344269608 | 0.000358884 | 0.000587265 | DOWN |
| CD3 | 574.3357808 | 385.5380068 | -188.7977741 | -0.575021354 | 0.002908531 | 0.004027196 | DOWN |
| Ki-67 | 1570.953476 | 1345.314564 | -225.6389125 | -0.22369691 | 0.031362185 | 0.035282458 | DOWN |
| PD-L1 | 71.11127748 | 69.11473431 | -1.996543172 | -0.041085068 | 0.141655152 | 0.149987808 | NOT |
| PanCk | 6529.642594 | 6094.584747 | -435.057847 | -0.0994761 | 0.315962559 | 0.315962559 | NOT |
| **Differential proteins between LN_met_ and PT_deep_ in stromal compartment** | | | | | | | |
| Protein | LN_met_ _Mean | PT_deep_ _Mean | Mean_difference | Log2FC | *P*-value | FDR | Change |
| SMA | 15779.32278 | 31165.49725 | 15386.17447 | 0.981914443 | 2.37E-06 | 1.07E-05 | UP |
| CTLA4 | 53.58081971 | 87.53178927 | 33.95096956 | 0.708090409 | 3.39E-05 | 6.78E-05 | UP |
| Fibronectin | 4234.36438 | 5689.983062 | 1455.618682 | 0.426278935 | 0.001357388 | 0.00187946 | UP |
| CD20 | 82.02134307 | 52.90447439 | -29.11686869 | -0.632609624 | 6.67E-08 | 1.20E-06 | DOWN |
| GZMB | 495.373407 | 305.4670388 | -189.9063682 | -0.697499707 | 1.60E-07 | 1.44E-06 | DOWN |
| PD-1 | 119.5292628 | 96.33845434 | -23.19080848 | -0.311180175 | 2.85E-07 | 1.71E-06 | DOWN |
| CD45 | 4914.216861 | 2470.751948 | -2443.464912 | -0.992011346 | 9.45E-06 | 2.36E-05 | DOWN |
| Beta-2-microglobulin | 346.1228441 | 257.5309451 | -88.59189895 | -0.426538364 | 1.05E-05 | 2.36E-05 | DOWN |
| CD56 | 247.0445106 | 188.084868 | -58.95964261 | -0.393387215 | 8.97E-06 | 2.36E-05 | DOWN |
| CD8 | 1488.150478 | 748.5899788 | -739.5604995 | -0.991272775 | 6.88E-06 | 2.36E-05 | DOWN |
| Ki-67 | 832.3444073 | 538.3423631 | -294.0020442 | -0.628656652 | 5.14E-05 | 9.25E-05 | DOWN |
| HLA-DR | 2615.975648 | 1785.838601 | -830.1370468 | -0.550747411 | 0.000873423 | 0.001429237 | DOWN |
| CD3 | 921.9523521 | 580.8677324 | -341.0846197 | -0.666482502 | 0.00118159 | 0.001772384 | DOWN |
| CD11c | 987.8715998 | 851.5142722 | -136.3573276 | -0.214292826 | 0.01146386 | 0.014739249 | DOWN |
| CD4 | 706.942048 | 556.6496372 | -150.2924108 | -0.344822393 | 0.014792029 | 0.017750435 | DOWN |
| PanCk | 1538.926421 | 1102.721571 | -436.2048493 | -0.480855687 | 0.077098348 | 0.086735642 | NOT |
| PD-L1 | 67.45082279 | 66.12964131 | -1.321181483 | -0.028538966 | 0.194718098 | 0.206172103 | NOT |
| CD68 | 1101.37287 | 1278.645733 | 177.2728627 | 0.215313624 | 0.739896775 | 0.739896775 | NOT |
| **Differential proteins between LN_met_ and PT_deep_ in tumor compartment** | | | | | | | |
| Protein | LN_met_ _Mean | PT_deep_ _Mean | Mean_difference | Log2FC | *P*-value | FDR | Change |
| CTLA4 | 35.33302039 | 43.93125524 | 8.598234844 | 0.314230641 | 4.30E-05 | 9.66E-05 | UP |
| Fibronectin | 1653.405756 | 1772.580636 | 119.1748806 | 0.100410444 | 0.029474373 | 0.037895623 | UP |
| PD-1 | 129.814564 | 103.8558413 | -25.95872264 | -0.321869887 | 4.89E-09 | 4.40E-08 | DOWN |
| CD20 | 63.25143042 | 47.16274124 | -16.08868918 | -0.423450532 | 2.57E-09 | 4.40E-08 | DOWN |
| CD56 | 227.9119092 | 171.6493702 | -56.26253899 | -0.409011749 | 5.73E-07 | 3.44E-06 | DOWN |
| CD11c | 585.4889053 | 361.9422019 | -223.5467034 | -0.693882499 | 2.64E-06 | 1.19E-05 | DOWN |
| CD45 | 1472.759044 | 809.9488214 | -662.8102231 | -0.862618757 | 1.23E-05 | 3.77E-05 | DOWN |
| HLA-DR | 996.9146412 | 577.3631818 | -419.5514593 | -0.787990872 | 1.26E-05 | 3.77E-05 | DOWN |
| CD4 | 331.6816735 | 253.4301712 | -78.25150235 | -0.388211011 | 4.10E-05 | 9.66E-05 | DOWN |
| Beta-2-microglobulin | 287.0570573 | 226.1427439 | -60.91431343 | -0.344103819 | 0.000155168 | 0.000310337 | DOWN |
| CD8 | 592.3666785 | 442.4809114 | -149.8857672 | -0.420875271 | 0.000419568 | 0.000755223 | DOWN |
| GZMB | 460.4544571 | 359.146907 | -101.3075501 | -0.358484378 | 0.001193741 | 0.001953394 | DOWN |
| CD68 | 776.9447353 | 611.5313302 | -165.4134051 | -0.345385569 | 0.002027361 | 0.003041041 | DOWN |
| CD3 | 250.6927662 | 193.4104077 | -57.28235847 | -0.374254937 | 0.002421114 | 0.003352311 | DOWN |
| PD-L1 | 74.51928702 | 72.05089136 | -2.468395662 | -0.048597593 | 0.42363865 | 0.50836638 | NOT |
| SMA | 3577.376485 | 3659.491068 | 82.11458249 | 0.032741068 | 0.45508764 | 0.511973595 | NOT |
| PanCk | 11176.17144 | 11004.6141 | -171.5573449 | -0.022317502 | 0.772010491 | 0.817422873 | NOT |
| Ki-67 | 2258.623988 | 2139.057712 | -119.5662767 | -0.078468707 | 0.892161798 | 0.892161798 | NOT |
