# Supplementary material for: Multi‐omics analyses reveal spatial heterogeneity in primary and metastatic oesophageal squamous cell carcinoma
Source: Clin Transl Med. 2023 Nov 27;13(11):e1493. doi: 10.1002/ctm2.1493 (PMC10679972; doi:10.1002/ctm2.1493)
Supplement: Supplementary file 28 — Table S17. Differential proteins with both FDR < 0.05 and |FC| > 1.5 between subregions in the whole area, stromal and tumour compartments, respectively. [file CTM2-13-e1493-s013.docx]

Supplementary Table 17. Differential proteins with both FDR < 0.05 and |FC| > 1.5 between subregions in the whole area, stromal, and

tumor compartments, respectively.

| Differential proteins between PTsup and PTdeep through mimic bulk sequencing in the whole area | | | | | | | |
| --- | --- | --- | --- | --- | --- | --- | --- |
| **Protein** | **PTsup_Mean** | **PTdeep_Mean** | **Mean_difference** | **Log2FC** | ***P*-value** | **FDR** | **Change** |
| CTLA4 | 37.99326851 | 64.22705388 | 26.23378537 | 0.757437291 | 1.69E-11 | 1.52E-10 | UP |
| PD-1 | 119.4055808 | 100.0934909 | -19.31208994 | -0.254522109 | 1.24E-11 | 1.52E-10 | NOT |
| GZMB | 446.6135642 | 324.6631512 | -121.950413 | -0.460083418 | 1.39E-07 | 8.33E-07 | NOT |
| CD56 | 201.1375957 | 178.7730949 | -22.36450079 | -0.170053139 | 4.41E-05 | 0.000198361 | NOT |
| CD20 | 56.03856567 | 50.12578017 | -5.912785491 | -0.160867243 | 0.00074735 | 0.002690458 | NOT |
| PanCk | 8826.341967 | 6102.505933 | -2723.836034 | -0.532413851 | 0.011600228 | 0.034800684 | NOT |
| Beta-2-microglobulin | 259.4109226 | 239.3175844 | -20.09333824 | -0.11631282 | 0.031795468 | 0.081759775 | NOT |
| Ki-67 | 1597.553511 | 1292.855836 | -304.6976753 | -0.305302844 | 0.083107334 | 0.18124859 | NOT |
| CD8 | 615.9312057 | 581.8581741 | -34.07303161 | -0.082101679 | 0.090624295 | 0.18124859 | NOT |
| PD-L1 | 61.15609527 | 69.10833927 | 7.952243999 | 0.176363515 | 0.113362533 | 0.20405256 | NOT |
| CD68 | 875.2471869 | 935.3903456 | 60.14315873 | 0.095878019 | 0.20328643 | 0.332650522 | NOT |
| CD4 | 404.9595997 | 396.0393782 | -8.920221483 | -0.032134102 | 0.494442464 | 0.684612643 | NOT |
| HLA-DR | 949.534598 | 1156.947663 | 207.4130651 | 0.285031129 | 0.490765573 | 0.684612643 | NOT |
| CD45 | 1722.264593 | 1595.610402 | -126.6541915 | -0.110198369 | 0.607149932 | 0.728579918 | NOT |
| SMA | 16233.44493 | 17651.81704 | 1418.372101 | 0.120847509 | 0.573610574 | 0.728579918 | NOT |
| CD11c | 498.4297253 | 596.4754061 | 98.04568083 | 0.259072544 | 0.665374671 | 0.748546505 | NOT |
| CD3 | 371.1194265 | 375.9491241 | 4.829697608 | 0.018653918 | 0.85272957 | 0.902890133 | NOT |
| Fibronectin | 3826.68698 | 3751.192713 | -75.494267 | -0.028746513 | 0.972863419 | 0.972863419 | NOT |
| Differential proteins between PTsup and PTdeep in stromal compartment | | | | | | | |
| **Protein** | **PTsup_Mean** | **PTdeep_Mean** | **Mean_difference** | **Log2FC** | ***P*-value** | **FDR** | **Change** |
| CTLA4 | 37.99326851 | 64.22705388 | 26.23378537 | 0.757437291 | 1.69E-11 | 1.52E-10 | UP |
| PD-1 | 119.4055808 | 100.0934909 | -19.31208994 | -0.254522109 | 1.24E-11 | 1.52E-10 | NOT |
| GZMB | 446.6135642 | 324.6631512 | -121.950413 | -0.460083418 | 1.39E-07 | 8.33E-07 | NOT |
| CD56 | 201.1375957 | 178.7730949 | -22.36450079 | -0.170053139 | 4.41E-05 | 0.000198361 | NOT |
| CD20 | 56.03856567 | 50.12578017 | -5.912785491 | -0.160867243 | 0.00074735 | 0.002690458 | NOT |
| PanCk | 8826.341967 | 6102.505933 | -2723.836034 | -0.532413851 | 0.011600228 | 0.034800684 | NOT |
| Beta-2-microglobulin | 259.4109226 | 239.3175844 | -20.09333824 | -0.11631282 | 0.031795468 | 0.081759775 | NOT |
| Ki-67 | 1597.553511 | 1292.855836 | -304.6976753 | -0.305302844 | 0.083107334 | 0.18124859 | NOT |
| CD8 | 615.9312057 | 581.8581741 | -34.07303161 | -0.082101679 | 0.090624295 | 0.18124859 | NOT |
| PD-L1 | 61.15609527 | 69.10833927 | 7.952243999 | 0.176363515 | 0.113362533 | 0.20405256 | NOT |
| CD68 | 875.2471869 | 935.3903456 | 60.14315873 | 0.095878019 | 0.20328643 | 0.332650522 | NOT |
| CD4 | 404.9595997 | 396.0393782 | -8.920221483 | -0.032134102 | 0.494442464 | 0.684612643 | NOT |
| HLA-DR | 949.534598 | 1156.947663 | 207.4130651 | 0.285031129 | 0.490765573 | 0.684612643 | NOT |
| CD45 | 1722.264593 | 1595.610402 | -126.6541915 | -0.110198369 | 0.607149932 | 0.728579918 | NOT |
| SMA | 16233.44493 | 17651.81704 | 1418.372101 | 0.120847509 | 0.573610574 | 0.728579918 | NOT |
| CD11c | 498.4297253 | 596.4754061 | 98.04568083 | 0.259072544 | 0.665374671 | 0.748546505 | NOT |
| CD3 | 371.1194265 | 375.9491241 | 4.829697608 | 0.018653918 | 0.85272957 | 0.902890133 | NOT |
| Fibronectin | 3826.68698 | 3751.192713 | -75.494267 | -0.028746513 | 0.972863419 | 0.972863419 | NOT |
| Differential proteins between PTsup and PTdeep in tumor compartment | | | | | | | |
| **Protein** | **PTsup_Mean** | **PTdeep_Mean** | **Mean_difference** | **Log2FC** | ***P*-value** | **FDR** | **Change** |
| PD-1 | 126.9134598 | 104.0461609 | -22.86729884 | -0.286621348 | 2.93E-09 | 5.27E-08 | NOT |
| CTLA4 | 26.63799849 | 39.69575347 | 13.05775497 | 0.575498994 | 8.91E-09 | 8.02E-08 | NOT |
| CD20 | 55.44692056 | 47.2008389 | -8.246081658 | -0.232294837 | 3.53E-05 | 0.000211945 | NOT |
| GZMB | 458.6166068 | 344.8695852 | -113.7470216 | -0.411237697 | 9.55E-05 | 0.00042996 | NOT |
| CD56 | 194.2108607 | 168.9712284 | -25.23963232 | -0.200846269 | 0.00074438 | 0.002679769 | NOT |
| PanCk | 15711.13275 | 11365.43684 | -4345.695907 | -0.467134064 | 0.005650463 | 0.016951389 | NOT |
| CD68 | 659.6811829 | 574.0688854 | -85.61229746 | -0.20054509 | 0.03699877 | 0.095139695 | NOT |
| CD8 | 422.8587494 | 406.3510112 | -16.5077382 | -0.057449345 | 0.061053182 | 0.137369659 | NOT |
| PD-L1 | 56.20702013 | 72.2438108 | 16.03679067 | 0.362123665 | 0.144652903 | 0.260375226 | NOT |
| Ki-67 | 2517.916318 | 2087.080544 | -430.8357738 | -0.270744059 | 0.131616421 | 0.260375226 | NOT |
| CD4 | 243.3160246 | 226.9759476 | -16.34007699 | -0.100291913 | 0.216790484 | 0.354748065 | NOT |
| Beta-2-microglobulin | 233.7816406 | 220.1456258 | -13.63601486 | -0.086703458 | 0.271863076 | 0.407794615 | NOT |
| CD45 | 730.7612985 | 674.4087741 | -56.35252433 | -0.115776924 | 0.319651183 | 0.442593946 | NOT |
| Fibronectin | 1720.826473 | 1710.360767 | -10.46570595 | -0.008800959 | 0.532384598 | 0.684494484 | NOT |
| CD11c | 328.5638088 | 328.0134418 | -0.550367055 | -0.00241864 | 0.583672781 | 0.700407337 | NOT |
| CD3 | 159.3437662 | 160.2453258 | 0.901559602 | 0.008139696 | 0.664640986 | 0.747721109 | NOT |
| SMA | 3213.754521 | 3426.890492 | 213.1359702 | 0.092640356 | 0.791416892 | 0.837970827 | NOT |
| HLA-DR | 493.9072728 | 494.9572016 | 1.049928813 | 0.00306357 | 0.96737753 | 0.96737753 | NOT |
| Differential proteins between LNmet and PTsup through mimic bulk sequencing in the whole area. | | | | | | | |
| **Protein** | **LNmet _Mean** | **PTsup _Mean** | **Mean_difference** | **Log2FC** | ***P*-value** | **FDR** | **Change** |
| CD11c | 773.4131004 | 498.4297253 | -274.9833752 | -0.633849092 | 4.48E-09 | 8.07E-08 | DOWN |
| HLA-DR | 1792.693539 | 949.534598 | -843.1589407 | -0.916836407 | 1.12E-07 | 6.73E-07 | DOWN |
| CD8 | 1055.436138 | 615.9312057 | -439.5049324 | -0.776998159 | 1.06E-05 | 3.83E-05 | DOWN |
| CD45 | 3183.426887 | 1722.264593 | -1461.162294 | -0.886273828 | 2.13E-05 | 6.38E-05 | DOWN |
| CD3 | 590.3360135 | 371.1194265 | -219.216587 | -0.669652834 | 0.001348827 | 0.002427888 | DOWN |
| CD20 | 73.90414805 | 56.03856567 | -17.86558238 | -0.399235312 | 7.86E-08 | 6.73E-07 | NOT |
| Beta-2-microglobulin | 317.3561949 | 259.4109226 | -57.94527227 | -0.290863779 | 6.56E-06 | 2.95E-05 | NOT |
| CD56 | 231.4144588 | 201.1375957 | -30.2768631 | -0.202296238 | 0.000118685 | 0.000305189 | NOT |
| Fibronectin | 2826.557925 | 3826.68698 | 1000.129055 | 0.437049632 | 0.000257033 | 0.000578324 | NOT |
| PD-L1 | 72.68195019 | 61.15609527 | -11.52585492 | -0.249100835 | 0.000512032 | 0.001024064 | NOT |
| CD4 | 515.7616209 | 404.9595997 | -110.8020212 | -0.348926436 | 0.00254314 | 0.004161502 | NOT |
| SMA | 9599.158922 | 16233.44493 | 6634.286012 | 0.757989282 | 0.034121712 | 0.051182568 | NOT |
| PD-1 | 127.3239935 | 119.4055808 | -7.918412699 | -0.092634046 | 0.055321359 | 0.076598804 | NOT |
| PanCk | 6538.588034 | 8826.341967 | 2287.753933 | 0.432836515 | 0.096000304 | 0.123428963 | NOT |
| CTLA4 | 40.26112369 | 37.99326851 | -2.267855184 | -0.083643608 | 0.205354207 | 0.246425048 | NOT |
| GZMB | 467.5474362 | 446.6135642 | -20.93387196 | -0.066085676 | 0.258110335 | 0.290374127 | NOT |
| CD68 | 892.562779 | 875.2471869 | -17.31559214 | -0.028263126 | 0.304150534 | 0.322041742 | NOT |
| Ki-67 | 1574.588507 | 1597.553511 | 22.96500462 | 0.020889403 | 0.652104068 | 0.652104068 | NOT |
| Differential proteins between LNmet and PTsup in stromal compartment. | | | | | | | |
| **Protein** | **LNmet _Mean** | **PTsup _Mean** | **Mean_difference** | **Log2FC** | ***P*-value** | **FDR** | **Change** |
| SMA | 15879.23651 | 30183.11323 | 14303.87673 | 0.926600073 | 1.97E-06 | 1.66E-05 | UP |
| HLA-DR | 2684.699679 | 1437.706732 | -1246.992947 | -0.900991291 | 2.77E-06 | 1.66E-05 | DOWN |
| CD8 | 1550.871096 | 822.7945517 | -728.0765444 | -0.914474633 | 1.32E-05 | 5.94E-05 | DOWN |
| CD45 | 5069.18342 | 2784.589552 | -2284.593868 | -0.864288676 | 0.000123494 | 0.000370481 | DOWN |
| CD3 | 958.3916605 | 598.0219196 | -360.3697409 | -0.680416989 | 0.001220428 | 0.002440855 | DOWN |
| CD20 | 84.09160893 | 56.67247114 | -27.41913779 | -0.569313738 | 1.24E-06 | 1.66E-05 | NOT |
| Fibronectin | 4212.564867 | 6082.966095 | 1870.401227 | 0.530076061 | 0.000120394 | 0.000370481 | NOT |
| CD11c | 995.6565958 | 680.4289214 | -315.2276744 | -0.549203776 | 0.000151142 | 0.00038865 | NOT |
| Beta-2-microglobulin | 349.6526957 | 286.8708677 | -62.78182803 | -0.285521161 | 0.000279871 | 0.00062971 | NOT |
| Ki-67 | 831.397582 | 611.4505039 | -219.9470781 | -0.443302833 | 0.001360627 | 0.002449128 | NOT |
| CD56 | 250.0227761 | 208.5590974 | -41.46367874 | -0.261603281 | 0.002839784 | 0.004646919 | NOT |
| PD-L1 | 68.03242969 | 66.45867578 | -1.573753912 | -0.033765067 | 0.01045429 | 0.015681435 | NOT |
| GZMB | 488.7741928 | 433.7531614 | -55.02103139 | -0.17229384 | 0.038670191 | 0.053543342 | NOT |
| CD4 | 721.2898325 | 578.1491444 | -143.1406882 | -0.319137377 | 0.049803894 | 0.064033577 | NOT |
| PD-1 | 120.0680393 | 111.3614248 | -8.706614523 | -0.108602599 | 0.075976491 | 0.091171789 | NOT |
| PanCk | 1410.516554 | 1449.780417 | 39.26386358 | 0.03961081 | 0.097718856 | 0.109933713 | NOT |
| CTLA4 | 53.06428171 | 50.15962924 | -2.904652472 | -0.081214406 | 0.125703229 | 0.133097536 | NOT |
| CD68 | 1061.635622 | 1106.210763 | 44.57514034 | 0.059337599 | 0.992535089 | 0.992535089 | NOT |
| Differential proteins between LNmet and PTsup in tumor compartment. | | | | | | | |
| **Protein** | **LNmet _Mean** | **PTsup _Mean** | **Mean_difference** | **Log2FC** | ***P*-value** | **FDR** | **Change** |
| CD11c | 551.1696051 | 328.5638088 | -222.6057962 | -0.746322754 | 6.58E-07 | 1.18E-05 | DOWN |
| HLA-DR | 900.6873984 | 493.9072728 | -406.7801256 | -0.866786264 | 1.58E-05 | 0.000142578 | DOWN |
| CD45 | 1297.670354 | 730.7612985 | -566.9090552 | -0.828451808 | 9.29E-05 | 0.000557566 | DOWN |
| CD4 | 310.2334092 | 243.3160246 | -66.91738464 | -0.350522721 | 0.000720346 | 0.003241556 | NOT |
| CD3 | 222.2803664 | 159.3437662 | -62.93660021 | -0.480237944 | 0.00463432 | 0.01390296 | NOT |
| Beta-2-microglobulin | 285.0596941 | 233.7816406 | -51.27805352 | -0.286102428 | 0.004383451 | 0.01390296 | NOT |
| CD8 | 560.0011801 | 422.8587494 | -137.1424307 | -0.405254038 | 0.005466851 | 0.014057617 | NOT |
| CD20 | 63.71668717 | 55.44692056 | -8.269766615 | -0.200563921 | 0.006432101 | 0.014472227 | NOT |
| PD-L1 | 77.3314707 | 56.20702013 | -21.12445057 | -0.46030532 | 0.015387871 | 0.025180152 | NOT |
| PanCk | 11666.65952 | 15711.13275 | 4044.473233 | 0.429395663 | 0.014413543 | 0.025180152 | NOT |
| CD56 | 212.8061414 | 194.2108607 | -18.59528068 | -0.131915904 | 0.01417863 | 0.025180152 | NOT |
| Fibronectin | 1440.550983 | 1720.826473 | 280.2754901 | 0.256480904 | 0.067929268 | 0.101893902 | NOT |
| CD68 | 723.4899358 | 659.6811829 | -63.8087529 | -0.133203995 | 0.184037539 | 0.254821208 | NOT |
| PD-1 | 134.5799477 | 126.9134598 | -7.666487942 | -0.084618384 | 0.217296296 | 0.279380952 | NOT |
| Ki-67 | 2317.779431 | 2517.916318 | 200.1368868 | 0.119487056 | 0.315872374 | 0.379046849 | NOT |
| SMA | 3319.081339 | 3213.754521 | -105.3268172 | -0.04652425 | 0.366853628 | 0.412710332 | NOT |
| GZMB | 446.3206796 | 458.6166068 | 12.29592727 | 0.039207944 | 0.68958724 | 0.730151195 | NOT |
| CTLA4 | 27.45796567 | 26.63799849 | -0.819967181 | -0.043739056 | 0.922222254 | 0.922222254 | NOT |
| Differential proteins between LNmet and PTdeep through mimic bulk sequencing in the whole area. | | | | | | | |
| **Protein** | **LNmet _Mean** | **PTdeep _Mean** | **Mean_difference** | **Log2FC** | ***P*-value** | **FDR** | **Change** |
| CTLA4 | 40.26112369 | 64.22705388 | 23.96593019 | 0.673793682 | 3.85E-07 | 9.89E-07 | UP |
| SMA | 9599.158922 | 17651.81704 | 8052.658113 | 0.878836791 | 0.010463342 | 0.013452869 | UP |
| CD8 | 1055.436138 | 581.8581741 | -473.577964 | -0.859099838 | 1.39E-07 | 4.17E-07 | DOWN |
| CD45 | 3183.426887 | 1595.610402 | -1587.816485 | -0.996472197 | 1.35E-06 | 3.03E-06 | DOWN |
| HLA-DR | 1792.693539 | 1156.947663 | -635.7458756 | -0.631805278 | 1.25E-05 | 2.25E-05 | DOWN |
| CD3 | 590.3360135 | 375.9491241 | -214.3868894 | -0.650998916 | 0.002459114 | 0.003404927 | DOWN |
| PD-1 | 127.3239935 | 100.0934909 | -27.23050264 | -0.347156155 | 9.67E-16 | 1.44E-14 | NOT |
| CD20 | 73.90414805 | 50.12578017 | -23.77836788 | -0.560102555 | 1.60E-15 | 1.44E-14 | NOT |
| CD56 | 231.4144588 | 178.7730949 | -52.64136389 | -0.372349378 | 8.02E-11 | 4.81E-10 | NOT |
| GZMB | 467.5474362 | 324.6631512 | -142.884285 | -0.526169094 | 2.11E-09 | 9.48E-09 | NOT |
| Beta-2-microglobulin | 317.3561949 | 239.3175844 | -78.03861051 | -0.407176599 | 9.88E-09 | 3.56E-08 | NOT |
| CD11c | 773.4131004 | 596.4754061 | -176.9376944 | -0.374776548 | 2.55E-06 | 5.09E-06 | NOT |
| Fibronectin | 2826.557925 | 3751.192713 | 924.6347878 | 0.408303119 | 0.000140935 | 0.000230621 | NOT |
| CD4 | 515.7616209 | 396.0393782 | -119.7222427 | -0.381060538 | 0.000401213 | 0.00060182 | NOT |
| Ki-67 | 1574.588507 | 1292.855836 | -281.7326707 | -0.284413441 | 0.024440444 | 0.029328533 | NOT |
| CD68 | 892.562779 | 935.3903456 | 42.82756659 | 0.067614893 | 0.035874818 | 0.040359171 | NOT |
| PD-L1 | 72.68195019 | 69.10833927 | -3.573610924 | -0.07273732 | 0.103523477 | 0.109613093 | NOT |
| PanCk | 6538.588034 | 6102.505933 | -436.0821012 | -0.099577336 | 0.345230762 | 0.345230762 | NOT |
| Differential Expression of proteins between LNmet and PTdeep in stromal compartment. | | | | | | | |
| **Protein** | **LNmet _Mean** | **PTdeep _Mean** | **Mean_difference** | **Log2FC** | ***P*-value** | **FDR** | **Change** |
| SMA | 15879.23651 | 31165.49725 | 15286.26075 | 0.972808184 | 4.09E-06 | 1.23E-05 | UP |
| CTLA4 | 53.06428171 | 87.53178927 | 34.46750756 | 0.722065971 | 3.67E-05 | 7.34E-05 | UP |
| CD20 | 84.09160893 | 52.90447439 | -31.18713454 | -0.668572105 | 6.56E-09 | 1.18E-07 | DOWN |
| GZMB | 488.7741928 | 305.4670388 | -183.3071539 | -0.678151397 | 2.76E-07 | 2.48E-06 | DOWN |
| CD8 | 1550.871096 | 748.5899788 | -802.2811173 | -1.050831138 | 1.48E-06 | 6.67E-06 | DOWN |
| CD45 | 5069.18342 | 2470.751948 | -2598.431472 | -1.036803188 | 3.87E-06 | 1.23E-05 | DOWN |
| Ki-67 | 831.397582 | 538.3423631 | -293.0552189 | -0.627014594 | 9.76E-05 | 0.000175654 | DOWN |
| CD3 | 958.3916605 | 580.8677324 | -377.5239281 | -0.722405665 | 0.000371938 | 0.000608626 | DOWN |
| HLA-DR | 2684.699679 | 1785.838601 | -898.8610774 | -0.588159012 | 0.000632642 | 0.000948963 | DOWN |
| PD-1 | 120.0680393 | 96.33845434 | -23.72958499 | -0.317668492 | 4.55E-07 | 2.73E-06 | NOT |
| CD56 | 250.0227761 | 188.084868 | -61.9379081 | -0.41067574 | 5.59E-06 | 1.44E-05 | NOT |
| Beta-2-microglobulin | 349.6526957 | 257.5309451 | -92.12175058 | -0.44117683 | 7.80E-06 | 1.76E-05 | NOT |
| Fibronectin | 4212.564867 | 5689.983062 | 1477.418194 | 0.433725456 | 0.001196548 | 0.001656759 | NOT |
| CD4 | 721.2898325 | 556.6496372 | -164.6401953 | -0.373809526 | 0.008989012 | 0.011557301 | NOT |
| CD11c | 995.6565958 | 851.5142722 | -144.1423236 | -0.225617528 | 0.011038912 | 0.013246694 | NOT |
| PanCk | 1410.516554 | 1102.721571 | -307.7949825 | -0.35515503 | 0.105582554 | 0.118780374 | NOT |
| PD-L1 | 68.03242969 | 66.12964131 | -1.902788377 | -0.040925538 | 0.193999082 | 0.205410793 | NOT |
| CD68 | 1061.635622 | 1278.645733 | 217.0101105 | 0.268327915 | 0.838239732 | 0.838239732 | NOT |
| Differential Expression of proteins between LNmet and PTdeep in tumor compartment. | | | | | | | |
| **Protein** | **LNmet _Mean** | **PTdeep _Mean** | **Mean_difference** | **Log2FC** | ***P*-value** | **FDR** | **Change** |
| CD11c | 551.1696051 | 328.0134418 | -223.1561633 | -0.748741394 | 1.85E-06 | 6.65E-06 | DOWN |
| CD45 | 1297.670354 | 674.4087741 | -623.2615796 | -0.944228732 | 7.90E-06 | 2.37E-05 | DOWN |
| HLA-DR | 900.6873984 | 494.9572016 | -405.7301968 | -0.863722694 | 2.17E-05 | 5.59E-05 | DOWN |
| PD-1 | 134.5799477 | 104.0461609 | -30.53378678 | -0.371239732 | 8.80E-11 | 1.58E-09 | NOT |
| CD20 | 63.71668717 | 47.2008389 | -16.51584827 | -0.432858758 | 2.05E-09 | 1.84E-08 | NOT |
| CTLA4 | 27.45796567 | 39.69575347 | 12.23778779 | 0.531759938 | 6.41E-07 | 3.07E-06 | NOT |
| CD56 | 212.8061414 | 168.9712284 | -43.83491299 | -0.332762174 | 6.83E-07 | 3.07E-06 | NOT |
| CD4 | 310.2334092 | 226.9759476 | -83.25746162 | -0.450814635 | 4.27E-05 | 9.61E-05 | NOT |
| CD8 | 560.0011801 | 406.3510112 | -153.6501689 | -0.462703383 | 0.000201608 | 0.000403215 | NOT |
| Beta-2-microglobulin | 285.0596941 | 220.1456258 | -64.91406838 | -0.372805886 | 0.000341488 | 0.000614679 | NOT |
| CD3 | 222.2803664 | 160.2453258 | -62.03504061 | -0.472098248 | 0.002134665 | 0.003201998 | NOT |
| GZMB | 446.3206796 | 344.8695852 | -101.4510943 | -0.372029753 | 0.00204832 | 0.003201998 | NOT |
| CD68 | 723.4899358 | 574.0688854 | -149.4210504 | -0.333749085 | 0.003534403 | 0.004893789 | NOT |
| Fibronectin | 1440.550983 | 1710.360767 | 269.8097841 | 0.247679945 | 0.013136508 | 0.016889796 | NOT |
| PD-L1 | 77.3314707 | 72.2438108 | -5.087659894 | -0.098181655 | 0.315814585 | 0.378977502 | NOT |
| SMA | 3319.081339 | 3426.890492 | 107.809153 | 0.046116105 | 0.378850283 | 0.426206568 | NOT |
| Ki-67 | 2317.779431 | 2087.080544 | -230.698887 | -0.151257003 | 0.657704028 | 0.6963925 | NOT |
| PanCk | 11666.65952 | 11365.43684 | -301.2226745 | -0.0377384 | 0.702798378 | 0.702798378 | NOT |
