# Supplementary material for: Multi‐omics analyses reveal spatial heterogeneity in primary and metastatic oesophageal squamous cell carcinoma
Source: Clin Transl Med. 2023 Nov 27;13(11):e1493. doi: 10.1002/ctm2.1493 (PMC10679972; doi:10.1002/ctm2.1493)
Supplement: Supplementary file 29 — Table S18. Protein markers for cell types. [file CTM2-13-e1493-s017.docx]

**Supplementary Table 18. Protein markers for cell types.**

| **Cell type** | **Markers** |
| --- | --- |
| Tumor | PanCK, Beta-2-microglobulin |
| Macrophage | CD68 |
| B cells | CD20 |
| CD8 T cells | CD8 |
| CD4 T cells | CD4 |
| DC | CD11c |
| NK cells | CD56 |
| Fibroblasts | Fibronectin |
| Cytotoxicity | GZMB |
