# Supplementary material for: Multi‐omics analyses reveal spatial heterogeneity in primary and metastatic oesophageal squamous cell carcinoma
Source: Clin Transl Med. 2023 Nov 27;13(11):e1493. doi: 10.1002/ctm2.1493 (PMC10679972; doi:10.1002/ctm2.1493)
Supplement: Supplementary file 30 — Table S19. Differentially expressed genes between genomic ITHhigh and ITHlow groups. [file CTM2-13-e1493-s025.docx]

**Supplementary Table 19. Differentially expressed genes between genomic ITH_high_ and ITH_low_ groups.**

| Differential RNA expression between genomic **ITH_high_ and ITH_low_** groups in PTsup | | | | | | | |
| --- | --- | --- | --- | --- | --- | --- | --- |
| Gene_ID | logFC | logCPM | LR | PValue | FDR | adjusted P-value | -log10 adjusted P-value |
| RP11-210L7.1 | -5.861821462 | -1.163651618 | 25.98232544 | 3.44557E-07 | 0.006481941 | 0.006481941 | 2.188294893 |
| AC069363.1 | 3.683714814 | -1.223139905 | 25.36116571 | 4.75393E-07 | 0.006481941 | 0.006481941 | 2.188294893 |
| CYP4Z2P | 5.945924515 | -3.126099354 | 25.30611156 | 4.89156E-07 | 0.006481941 | 0.006481941 | 2.188294893 |
| FOXI3 | -5.22744147 | -0.247096305 | 25.06141866 | 5.55329E-07 | 0.006481941 | 0.006481941 | 2.188294893 |
| CTB-96E2.7 | 8.574312744 | -1.061412967 | 23.66577918 | 1.14602E-06 | 0.009759198 | 0.009759198 | 2.010585851 |
| RP11-384F7.2 | 5.782659299 | -3.234542312 | 23.29779618 | 1.38759E-06 | 0.009759198 | 0.009759198 | 2.010585851 |
| LINC01071 | 5.830637543 | -3.204596922 | 23.1957814 | 1.46318E-06 | 0.009759198 | 0.009759198 | 2.010585851 |
| CTD-2026K11.3 | 5.454696414 | -2.917526194 | 22.13286853 | 2.54416E-06 | 0.014848047 | 0.014848047 | 1.828330662 |
| SLC15A1 | -2.996757581 | 2.232609059 | 21.4819233 | 3.5718E-06 | 0.017893821 | 0.017893821 | 1.747296902 |
| SLC22A24 | 5.476044918 | -3.404209859 | 21.33527331 | 3.85571E-06 | 0.017893821 | 0.017893821 | 1.747296902 |
| PGA3 | 4.09322485 | 0.385438044 | 21.00786476 | 4.57402E-06 | 0.017893821 | 0.017893821 | 1.747296902 |
| MPPED1 | -5.953505531 | 2.856331717 | 20.75523355 | 5.21888E-06 | 0.017893821 | 0.017893821 | 1.747296902 |
| ZNF804B | 8.297800477 | -1.32318467 | 20.66326329 | 5.47566E-06 | 0.017893821 | 0.017893821 | 1.747296902 |
| CTC-499B15.4 | 6.496755632 | -2.780436983 | 20.60040225 | 5.65842E-06 | 0.017893821 | 0.017893821 | 1.747296902 |
| CTD-2147F2.1 | -8.007540687 | -0.895289186 | 20.57005537 | 5.74883E-06 | 0.017893821 | 0.017893821 | 1.747296902 |
| HMGB3P8 | 6.504650409 | -0.699921502 | 20.07226659 | 7.45702E-06 | 0.021760057 | 0.021760057 | 1.662339976 |
| CTD-3116E22.6 | 4.325221408 | -1.833779932 | 19.77498791 | 8.71153E-06 | 0.023925436 | 0.023925436 | 1.62114014 |
| LDHC | 5.603945668 | -2.165306 | 19.53334042 | 9.88591E-06 | 0.025642405 | 0.025642405 | 1.591041247 |
| RP11-3J1.1 | -7.443612566 | -1.389195401 | 19.17153901 | 1.19482E-05 | 0.028699866 | 0.028699866 | 1.542120131 |
| RP11-511P7.4 | 4.999344121 | -3.623052148 | 19.03539708 | 1.28316E-05 | 0.028699866 | 0.028699866 | 1.542120131 |
| HTN1 | 7.155205266 | -2.250615111 | 18.99048594 | 1.31372E-05 | 0.028699866 | 0.028699866 | 1.542120131 |
| RP11-216M21.2 | -6.428472918 | -2.223515504 | 18.93519491 | 1.35235E-05 | 0.028699866 | 0.028699866 | 1.542120131 |
| UBE2FP2 | -7.211265406 | -1.580825824 | 18.73814191 | 1.49953E-05 | 0.030439787 | 0.030439787 | 1.516558387 |
| DMBT1 | 7.489399767 | 2.524839879 | 18.5524853 | 1.6529E-05 | 0.032155021 | 0.032155021 | 1.492751199 |
| BPIFB2 | 9.448975866 | -0.216070699 | 18.45485253 | 1.73977E-05 | 0.032491298 | 0.032491298 | 1.488232932 |
| RP11-331H2.4 | 4.938442313 | -3.640563994 | 17.84214994 | 2.40007E-05 | 0.043098856 | 0.043098856 | 1.365534259 |
| RPEP6 | 5.650782096 | -3.313077669 | 17.76277674 | 2.50231E-05 | 0.043270556 | 0.043270556 | 1.363807529 |
| SELV | -7.025994844 | -1.806789319 | 17.6269531 | 2.68752E-05 | 0.04337248 | 0.04337248 | 1.362785743 |
| CCL8 | -4.531265465 | -1.195173874 | 17.62237486 | 2.694E-05 | 0.04337248 | 0.04337248 | 1.362785743 |
| CICP17 | 6.209799274 | -2.980718671 | 17.49896438 | 2.87464E-05 | 0.044738058 | 0.044738058 | 1.349322875 |
| PNLIPRP3 | 6.797297286 | 1.581667714 | 17.31668504 | 3.16397E-05 | 0.047056609 | 0.047056609 | 1.327379373 |
| RP11-162K6.1 | 4.84183559 | -3.686524346 | 17.21720954 | 3.33402E-05 | 0.047056609 | 0.047056609 | 1.327379373 |
| RP5-1070A16.1 | 5.832504271 | -3.21449869 | 17.12690943 | 3.49634E-05 | 0.047056609 | 0.047056609 | 1.327379373 |
| RP11-307O13.1 | 5.762546654 | -3.269618262 | 17.11269718 | 3.5226E-05 | 0.047056609 | 0.047056609 | 1.327379373 |
| ERN2 | 2.752562688 | 0.061606971 | 17.05952089 | 3.62263E-05 | 0.047056609 | 0.047056609 | 1.327379373 |
| LINC00858 | -5.682617752 | -0.651045633 | 16.86466574 | 4.01419E-05 | 0.047056609 | 0.047056609 | 1.327379373 |
| RP11-1006G14.3 | 5.456565607 | -3.428553637 | 16.82242245 | 4.10455E-05 | 0.047056609 | 0.047056609 | 1.327379373 |
| RN7SKP232 | 6.346733205 | -1.750868482 | 16.82218554 | 4.10506E-05 | 0.047056609 | 0.047056609 | 1.327379373 |
| C6orf58 | 7.457778001 | -2.004590284 | 16.75266113 | 4.25825E-05 | 0.047056609 | 0.047056609 | 1.327379373 |
| RP11-417J1.3 | 5.933013196 | -3.173384272 | 16.74516461 | 4.27511E-05 | 0.047056609 | 0.047056609 | 1.327379373 |
| KHSRPP1 | 5.770885128 | 2.305127347 | 16.71816358 | 4.33638E-05 | 0.047056609 | 0.047056609 | 1.327379373 |
| CTC-338M12.9 | -6.787302339 | 0.379489102 | 16.71427801 | 4.34527E-05 | 0.047056609 | 0.047056609 | 1.327379373 |
| RP11-108B14.5 | 5.039100406 | -3.61177439 | 16.67841547 | 4.4282E-05 | 0.047056609 | 0.047056609 | 1.327379373 |
| ADH1B | 5.156629747 | 2.811005418 | 16.64938813 | 4.49648E-05 | 0.047056609 | 0.047056609 | 1.327379373 |
| RP11-629F19.1 | 4.828232636 | -2.18954641 | 16.62861735 | 4.54599E-05 | 0.047056609 | 0.047056609 | 1.327379373 |
| HOXC12 | -9.011538618 | 0.029982454 | 16.59134219 | 4.63622E-05 | 0.047056609 | 0.047056609 | 1.327379373 |
| CYP1D1P | 5.457664412 | -3.428553637 | 16.48232195 | 4.91058E-05 | 0.048780873 | 0.048780873 | 1.311750433 |
| Differential RNA expression between genomic **ITH_high_ and ITH_low_** groups in PTdeep | | | | | | | |
|  | logFC | logCPM | LR | PValue | FDR | adjusted P-value | -log10 adjusted P-value |
| LINC00355 | -8.519564872 | -0.122419888 | 27.62808514 | 1.47028E-07 | 0.006864609 | 0.006864609 | 2.163384195 |
| RP11-463O12.3 | -6.696508158 | -1.70053375 | 26.11680438 | 3.21375E-07 | 0.007502335 | 0.007502335 | 2.124803526 |
| ZIC4 | -10.39169291 | 1.678666664 | 24.69821416 | 6.70463E-07 | 0.010434411 | 0.010434411 | 1.981532053 |
| FAM178B | 4.439086173 | 2.511416557 | 23.48755839 | 1.25724E-06 | 0.01467484 | 0.01467484 | 1.833426637 |
| CTD-3099C6.7 | 6.145548739 | -1.791320793 | 22.67159576 | 1.92188E-06 | 0.015386049 | 0.015386049 | 1.812872882 |
| CTC-250I14.6 | 7.077460409 | -1.720660315 | 22.61701242 | 1.97726E-06 | 0.015386049 | 0.015386049 | 1.812872882 |
| CST4 | 5.936442006 | 0.410669594 | 22.19822892 | 2.45901E-06 | 0.01545482 | 0.01545482 | 1.810936052 |
| PTCHD2 | 4.001955194 | 2.067749327 | 21.96372018 | 2.77853E-06 | 0.01545482 | 0.01545482 | 1.810936052 |
| AC073464.7 | 6.735945716 | -1.289586013 | 21.53576296 | 3.47291E-06 | 0.01545482 | 0.01545482 | 1.810936052 |
| RP11-747H12.3 | 7.336983391 | -1.51551294 | 21.49749099 | 3.54292E-06 | 0.01545482 | 0.01545482 | 1.810936052 |
| FOXL1 | 2.919433056 | 3.127699761 | 21.44503346 | 3.64118E-06 | 0.01545482 | 0.01545482 | 1.810936052 |
| SCXB | 7.416000156 | -1.449404408 | 21.02509169 | 4.53308E-06 | 0.01672141 | 0.01672141 | 1.7767271 |
| DSCAM | 5.392515299 | -0.291539882 | 20.91629748 | 4.79795E-06 | 0.01672141 | 0.01672141 | 1.7767271 |
| AL590822.2 | 6.749845156 | -1.93511807 | 20.65216086 | 5.5075E-06 | 0.01672141 | 0.01672141 | 1.7767271 |
| RP11-747H12.6 | 5.128858077 | -2.989940874 | 20.61685196 | 5.61001E-06 | 0.01672141 | 0.01672141 | 1.7767271 |
| RP11-127B20.2 | -5.408532626 | -2.627799177 | 20.576233 | 5.73031E-06 | 0.01672141 | 0.01672141 | 1.7767271 |
| RP11-550A18.1 | -5.352384687 | -2.660376456 | 20.26753671 | 6.73334E-06 | 0.017704958 | 0.017704958 | 1.751905109 |
| ADCY8 | -10.72586575 | 2.010392852 | 20.24144864 | 6.82579E-06 | 0.017704958 | 0.017704958 | 1.751905109 |
| AC008753.6 | 4.76736149 | -3.158726289 | 19.97181205 | 7.85923E-06 | 0.019312598 | 0.019312598 | 1.71415929 |
| RP11-139K4.1 | -6.275897554 | -2.014177799 | 19.62841212 | 9.406E-06 | 0.021073201 | 0.021073201 | 1.67626949 |
| KRT4 | -7.980532007 | 5.589954366 | 19.61376138 | 9.4784E-06 | 0.021073201 | 0.021073201 | 1.67626949 |
| RP11-127B16.1 | 7.554686668 | -1.325673096 | 19.4350791 | 1.04078E-05 | 0.021838955 | 0.021838955 | 1.660768137 |
| DDC | -6.023396705 | -2.20313692 | 19.37181192 | 1.07583E-05 | 0.021838955 | 0.021838955 | 1.660768137 |
| BRDT | -8.193943641 | 0.711670518 | 19.16934797 | 1.19619E-05 | 0.02327035 | 0.02327035 | 1.633197076 |
| CICP24 | 4.768400499 | -3.15797608 | 18.98678203 | 1.31627E-05 | 0.024210744 | 0.024210744 | 1.615991866 |
| DYNAP | -5.713008719 | -2.420191782 | 18.9409983 | 1.34824E-05 | 0.024210744 | 0.024210744 | 1.615991866 |
| CAPN14 | -4.046287505 | 2.968077702 | 18.59920835 | 1.61288E-05 | 0.027890195 | 0.027890195 | 1.554548446 |
| CTBP2P5 | 4.789366517 | -3.146659943 | 18.17075457 | 2.01956E-05 | 0.033389085 | 0.033389085 | 1.476395478 |
| RP11-867G2.6 | -5.415448838 | -2.617812143 | 18.12019606 | 2.0739E-05 | 0.033389085 | 0.033389085 | 1.476395478 |
| FOXD1 | 3.976929485 | 1.26984229 | 17.81461256 | 2.43506E-05 | 0.037208398 | 0.037208398 | 1.429359031 |
| AC006372.4 | 3.300150143 | -0.919317302 | 17.78710586 | 2.47052E-05 | 0.037208398 | 0.037208398 | 1.429359031 |
| PIGR | -5.957174254 | 1.52637853 | 17.63771356 | 2.67236E-05 | 0.038990575 | 0.038990575 | 1.409040356 |
| RP11-350D23.4 | 5.631892986 | -2.734670628 | 17.54831886 | 2.80098E-05 | 0.039628819 | 0.039628819 | 1.401988869 |
| KRT39 | -5.577983496 | -2.51631163 | 17.4743141 | 2.91216E-05 | 0.039953889 | 0.039953889 | 1.398440944 |
| KRT8P37 | -5.175847561 | -2.759671243 | 17.42092325 | 2.99511E-05 | 0.039953889 | 0.039953889 | 1.398440944 |
| PWRN2 | 5.129979539 | -2.958995723 | 17.28653906 | 3.21456E-05 | 0.040801916 | 0.040801916 | 1.389319445 |
| RN7SL55P | -5.303882841 | -2.695271844 | 17.18728057 | 3.38697E-05 | 0.040801916 | 0.040801916 | 1.389319445 |
| RP11-579D7.2 | 7.33146127 | -1.510915047 | 17.0839258 | 3.57637E-05 | 0.040801916 | 0.040801916 | 1.389319445 |
| ATP6V0A4 | -5.80373236 | -0.301799333 | 17.06764558 | 3.60716E-05 | 0.040801916 | 0.040801916 | 1.389319445 |
| FUT6 | -4.127526306 | 2.088211729 | 17.04746645 | 3.6457E-05 | 0.040801916 | 0.040801916 | 1.389319445 |
| CTC-422A18.2 | -5.058161059 | -2.833684946 | 17.04290138 | 3.65447E-05 | 0.040801916 | 0.040801916 | 1.389319445 |
| RP11-146E13.3 | 6.721013908 | -2.004085273 | 17.03154263 | 3.6764E-05 | 0.040801916 | 0.040801916 | 1.389319445 |
| DUXAP10 | -7.94866107 | -0.653306514 | 16.98995678 | 3.75781E-05 | 0.040801916 | 0.040801916 | 1.389319445 |
| JAZF1-AS1 | 2.779562556 | -2.071237566 | 16.94199058 | 3.85396E-05 | 0.04089487 | 0.04089487 | 1.38833117 |
| TSPAN1 | -3.450208509 | 4.128247278 | 16.68907083 | 4.4034E-05 | 0.045301977 | 0.045301977 | 1.343882847 |
| RP11-163O19.8 | 8.005693516 | -0.979616713 | 16.62386651 | 4.55739E-05 | 0.045301977 | 0.045301977 | 1.343882847 |
| AP000593.5 | -5.974863779 | -2.309710043 | 16.60360121 | 4.60635E-05 | 0.045301977 | 0.045301977 | 1.343882847 |
| MIR3648 | 2.950753857 | 8.817104898 | 16.58269611 | 4.6574E-05 | 0.045301977 | 0.045301977 | 1.343882847 |
| UGT2A2 | -4.897358153 | -2.919270127 | 16.38971011 | 5.15644E-05 | 0.049132455 | 0.049132455 | 1.308631534 |
| RP11-63B19.1 | 5.344179609 | -2.906409246 | 16.22391979 | 5.6279E-05 | 0.0498897 | 0.0498897 | 1.301989105 |
| RP11-37O16.4 | -4.12017491 | -1.796035662 | 16.19192596 | 5.72376E-05 | 0.0498897 | 0.0498897 | 1.301989105 |
| CTC-513N18.4 | 5.332522164 | -2.655188561 | 16.1867165 | 5.73952E-05 | 0.0498897 | 0.0498897 | 1.301989105 |
| MIRLET7DHG | 7.49726689 | -1.420196377 | 16.18597822 | 5.74176E-05 | 0.0498897 | 0.0498897 | 1.301989105 |
| RP11-1281K21.3 | 5.066445108 | -3.029377346 | 16.17319391 | 5.78064E-05 | 0.0498897 | 0.0498897 | 1.301989105 |
| MYL6P4 | -5.295126087 | -2.688134853 | 16.13003345 | 5.91387E-05 | 0.0498897 | 0.0498897 | 1.301989105 |
| BPIFB1 | -7.429295996 | -1.073577842 | 16.01871068 | 6.27196E-05 | 0.0498897 | 0.0498897 | 1.301989105 |
| AC018804.7 | 4.284954099 | 0.277798508 | 16.00365924 | 6.32202E-05 | 0.0498897 | 0.0498897 | 1.301989105 |
| OR6C70 | -5.786774687 | -1.368362945 | 16.00000974 | 6.33422E-05 | 0.0498897 | 0.0498897 | 1.301989105 |
| RP11-513D5.5 | -4.96973091 | -2.889690892 | 15.99738221 | 6.34301E-05 | 0.0498897 | 0.0498897 | 1.301989105 |
| B4GALNT2 | -6.775711887 | -1.629887527 | 15.95116147 | 6.49979E-05 | 0.0498897 | 0.0498897 | 1.301989105 |
| CXorf22 | -6.753766961 | -1.628189631 | 15.94507545 | 6.52072E-05 | 0.0498897 | 0.0498897 | 1.301989105 |
| SLC13A2 | -4.835674077 | -2.948363408 | 15.90930908 | 6.64511E-05 | 0.0498897 | 0.0498897 | 1.301989105 |
| AL589739.1 | 3.321264093 | -0.531343802 | 15.88475272 | 6.73189E-05 | 0.0498897 | 0.0498897 | 1.301989105 |
| Differential RNA expression between genomic **ITH_high_ and ITH_low_** groups in LNmet | | | | | | | |
|  | logFC | logCPM | LR | PValue | FDR | adjusted P-value | -log10 adjusted P-value |
| RP11-619A14.2 | 6.214746554 | -1.529014346 | 33.25491519 | 8.08352E-09 | 0.000377411 | 0.000377411 | 3.423185037 |
| RP11-290D2.6 | 7.366654825 | -1.491595488 | 30.0230386 | 4.26944E-08 | 0.000996678 | 0.000996678 | 3.001444982 |
| TUBB2BP1 | 5.978776755 | -2.496349772 | 26.54216937 | 2.57848E-07 | 0.004012885 | 0.004012885 | 2.396543334 |
| FAM19A4 | 6.399449945 | -2.249749916 | 24.17640235 | 8.79028E-07 | 0.01026023 | 0.01026023 | 1.988842902 |
| FAM25HP | 5.682652976 | -2.664176199 | 23.16023948 | 1.49047E-06 | 0.01391775 | 0.01391775 | 1.856430964 |
| ABCB10P3 | 6.476058671 | -1.289639835 | 22.21033497 | 2.44355E-06 | 0.019014479 | 0.019014479 | 1.720915578 |
| REXO1L2P | 7.06701955 | -1.715682987 | 21.59585234 | 3.36579E-06 | 0.021358794 | 0.021358794 | 1.670423269 |
| MPL | 4.285500925 | -2.214998343 | 21.43527579 | 3.65976E-06 | 0.021358794 | 0.021358794 | 1.670423269 |
| HS6ST3 | 6.104151856 | -1.721587274 | 21.0202045 | 4.54465E-06 | 0.022419973 | 0.022419973 | 1.649364917 |
| RP11-1016B18.1 | 6.922803814 | -1.83083665 | 20.58869728 | 5.69312E-06 | 0.022419973 | 0.022419973 | 1.649364917 |
| CKS1BP7 | 6.160611365 | -2.414916766 | 20.53491317 | 5.85535E-06 | 0.022419973 | 0.022419973 | 1.649364917 |
| NR0B1 | 8.126166341 | -0.905651526 | 20.48196994 | 6.01956E-06 | 0.022419973 | 0.022419973 | 1.649364917 |
| RP11-244H18.1 | 6.606187008 | -2.06604409 | 20.41234609 | 6.24258E-06 | 0.022419973 | 0.022419973 | 1.649364917 |
| RP11-638F5.1 | 5.636251089 | -2.701373372 | 19.05013956 | 1.27328E-05 | 0.038852588 | 0.038852588 | 1.410580043 |
| CTAG1B | 9.039053139 | 0.448958622 | 18.98966947 | 1.31428E-05 | 0.038852588 | 0.038852588 | 1.410580043 |
| RP11-350E12.5 | 6.005109507 | -2.50173322 | 18.92488103 | 1.35968E-05 | 0.038852588 | 0.038852588 | 1.410580043 |
| ZCCHC16 | 5.537072938 | -2.768429496 | 18.84924982 | 1.41467E-05 | 0.038852588 | 0.038852588 | 1.410580043 |
| ARHGEF7-IT1 | 4.577871713 | -2.718206101 | 18.65055918 | 1.57001E-05 | 0.0407235 | 0.0407235 | 1.390154906 |
| RP11-893F2.5 | 5.516082205 | -2.771640126 | 18.51740367 | 1.6836E-05 | 0.041044559 | 0.041044559 | 1.386744405 |
| RP11-849I19.1 | 6.663751026 | -2.014348965 | 18.40377356 | 1.78704E-05 | 0.041044559 | 0.041044559 | 1.386744405 |
| CLDN22 | 8.132630142 | -0.893646681 | 18.34179662 | 1.84612E-05 | 0.041044559 | 0.041044559 | 1.386744405 |
| CTAG1A | 8.32969545 | -0.215340274 | 18.09729261 | 2.099E-05 | 0.042822767 | 0.042822767 | 1.368325277 |
| RP11-753A21.1 | 5.226786882 | -2.900320045 | 18.04878139 | 2.15316E-05 | 0.042822767 | 0.042822767 | 1.368325277 |
| LINC00261 | 5.650389087 | -2.714019562 | 18.0067244 | 2.20126E-05 | 0.042822767 | 0.042822767 | 1.368325277 |
