# Supplementary material for: Multi‐omics analyses reveal spatial heterogeneity in primary and metastatic oesophageal squamous cell carcinoma
Source: Clin Transl Med. 2023 Nov 27;13(11):e1493. doi: 10.1002/ctm2.1493 (PMC10679972; doi:10.1002/ctm2.1493)
Supplement: Supplementary file 16 — Table S5. Detailed information of all somatic mutations in 45 tumour subregions from 15 ESCC patients. [file CTM2-13-e1493-s026.docx]

**Supplementary Table 5. Detailed information of all somatic mutations in forty-five tumor subregions from 15 ESCC patients.**

| **Sample** | **Hugo_Symbol** | **Entrez_Gene_ID** | **NCBI_Build** | **Chromosome** | **Start_Position** | **End_Position** | **Variant** | **Variant_Type** | **Reference_Allele** | **Tumor_Seq_Allele1** | **Tumor_Seq_Allele2** | **dbSNP_RS** | **Mutation_Status** | **Protein_Change** | **TumorVAF_WU** | **transcript_name** | **t_depth** | **t_ref_count** | **t_alt_count** |
| --- | --- | --- | --- | --- | --- | --- | --- | --- | --- | --- | --- | --- | --- | --- | --- | --- | --- | --- | --- |
| 035-PTsup | AGO4 | 107 | hg19 | chr1 | 36291040 | 36291040 | Missense_Mutation | SNP | C | C | T | novel | Somatic | P145S | 0.304015 | NM_017629.4 | 519 | 360 | 159 |
| 035-PTsup | BEND5 | 960 | hg19 | chr1 | 49227065 | 49227065 | Missense_Mutation | SNP | C | C | G | novel | Somatic | E102Q | 0.225543 | NM_024603.4 | 368 | 285 | 83 |
| 035-PTsup | COL11A1 | 171 | hg19 | chr1 | 103427776 | 103427776 | Nonsense_Mutation | SNP | C | C | A | novel | Somatic | G1036Ter | 0.038043 | NM_080629.2 | 367 | 353 | 14 |
| 035-PTsup | S100A12 | 1754 | hg19 | chr1 | 153347008 | 153347008 | Missense_Mutation | SNP | G | G | A | novel | Somatic | R21W | 0.267081 | NM_005621.2 | 322 | 236 | 86 |
| 035-PTsup | SYT14 | 273 | hg19 | chr1 | 210267826 | 210267826 | Frame_Shift_Del | DEL | CA | CA | T | novel | Somatic | S246FfsTer15 | 0.225694 | NM_001146261.2 | 572 | 442 | 130 |
| 035-PTsup | ENAH | 288 | hg19 | chr1 | 225707032 | 225707032 | Missense_Mutation | SNP | G | G | A | novel | Somatic | R224W | 0.25732 | NM_001008493.3 | 1121 | 831 | 290 |
| 035-PTsup | MTR | 37 | hg19 | chr1 | 237024474 | 237024474 | Frame_Shift_Del | DEL | A | A | - | novel | Somatic | K700NfsTer8 | 0.029851 | NM_000254.2 | 602 | 584 | 18 |
| 035-PTsup | OR2W5 | 2474 | hg19 | chr1 | 247654830 | 247654830 | Missense_Mutation | SNP | C | C | A | novel | Somatic | A134E | 0.190283 | NM_001004698.2 | 493 | 399 | 94 |
| 035-PTsup | TRIM58 | 2477 | hg19 | chr1 | 248020931 | 248020931 | Missense_Mutation | SNP | G | G | A | novel | Somatic | R128H | 0.023379 | NM_015431.4 | 939 | 917 | 22 |
| 035-PTsup | OR2L5 | 2478 | hg19 | chr1 | 248185608 | 248185608 | Missense_Mutation | SNP | A | A | T | novel | Somatic | D120V | 0.228571 | NM_001258284.1 | 419 | 323 | 96 |
| 035-PTsup | KCNK3 | 790 | hg19 | chr2 | 26951045 | 26951045 | Missense_Mutation | SNP | C | C | T | novel | Somatic | T265M | 0.086043 | NM_002246.3 | 1476 | 1349 | 127 |
| 035-PTsup | RMDN2 | 876 | hg19 | chr2 | 38156790 | 38156790 | Missense_Mutation | SNP | C | C | T | novel | Somatic | H124Y | 0.082324 | NM_001170792.2 | 412 | 378 | 34 |
| 035-PTsup | POLR1A | 1243 | hg19 | chr2 | 86258591 | 86258591 | Silent | SNP | C | C | T | novel | Somatic | L1480= | 0.022663 | NM_015425.6 | 1056 | 1032 | 24 |
| 035-PTsup | RGPD3 | 1401 | hg19 | chr2 | 107049414 | 107049414 | Missense_Mutation | SNP | G | G | A | novel | Somatic | R816C | 0.022676 | NM_001144013.1 | 882 | 862 | 20 |
| 035-PTsup | CNTNAP5 | 192 | hg19 | chr2 | 125261876 | 125261876 | Missense_Mutation | SNP | T | T | C | novel | Somatic | V357A | 0.115512 | NM_001367498.1 | 303 | 268 | 35 |
| 035-PTsup | TMEM163 | 25 | hg19 | chr2 | 135476308 | 135476308 | Missense_Mutation | SNP | G | G | T | novel | Somatic | D66E | 0.183515 | NM_030923.5 | 643 | 525 | 118 |
| 035-PTsup | RAPH1 | 267 | hg19 | chr2 | 204305619 | 204305619 | Frame_Shift_Del | DEL | G | G | - | novel | Somatic | P765HfsTer19 | 0.029795 | NM_213589.3 | 534 | 518 | 16 |
| 035-PTsup | ZNF142 | 2259 | hg19 | chr2 | 219507889 | 219507889 | Missense_Mutation | SNP | C | C | T | novel | Somatic | R1317Q | 0.02275 | NM_001366290.3 | 1010 | 987 | 23 |
| 035-PTsup | EPHA4 | 35 | hg19 | chr2 | 222347074 | 222347074 | Missense_Mutation | SNP | G | G | A | novel | Somatic | A439V | 0.147727 | NM_001304536.1 | 351 | 299 | 52 |
| 035-PTsup | EPHA4 | 35 | hg19 | chr2 | 222347362 | 222347362 | Missense_Mutation | SNP | G | G | A | novel | Somatic | S343F | 0.189448 | NM_001304536.1 | 416 | 337 | 79 |
| 035-PTsup | TRPM8 | 4 | hg19 | chr2 | 234854632 | 234854632 | Missense_Mutation | SNP | C | C | T | novel | Somatic | R278W | 0.297968 | NM_024080.5 | 443 | 311 | 132 |
| 035-PTsup | OXTR | 652 | hg19 | chr3 | 8809460 | 8809460 | In_Frame_Del | DEL | GCA | GCA | - | novel | Somatic | C138del | 0.143135 | NM_000916.3 | 1026 | 879 | 147 |
| 035-PTsup | CAND2 | 85 | hg19 | chr3 | 12875432 | 12875432 | In_Frame_Del | DEL | AGA | AGA | - | novel | Somatic | K1222del | 0.124122 | NM_001162499.2 | 427 | 374 | 53 |
| 035-PTsup | RARB | 97 | hg19 | chr3 | 25622110 | 25622110 | Missense_Mutation | SNP | G | G | T | novel | Somatic | C235F | 0.223744 | NM_001290216.2 | 437 | 339 | 98 |
| 035-PTsup | BAP1 | 985 | hg19 | chr3 | 52437707 | 52437707 | Nonsense_Mutation | SNP | G | G | T | novel | Somatic | S485Ter | 0.251908 | NM_004656.4 | 1440 | 1077 | 363 |
| 035-PTsup | ARHGEF26 | 219 | hg19 | chr3 | 153840531 | 153840531 | Missense_Mutation | SNP | C | C | G | novel | Somatic | I250M | 0.127413 | NM_015595.4 | 1035 | 903 | 132 |
| 035-PTsup | PIK3CA | 243 | hg19 | chr3 | 178936091 | 178936091 | Missense_Mutation | SNP | G | G | A | novel | Somatic | E545K | 0.542289 | NM_006218.4 | 401 | 183 | 218 |
| 035-PTsup | DVL3 | 1987 | hg19 | chr3 | 183888232 | 183888232 | Missense_Mutation | SNP | C | C | T | novel | Somatic | R614W | 0.053474 | NM_004423.4 | 2732 | 2585 | 147 |
| 035-PTsup | MAP3K13 | 1996 | hg19 | chr3 | 185155325 | 185155325 | Missense_Mutation | SNP | C | C | T | novel | Somatic | A189V | 0.096517 | NM_004721.5 | 997 | 900 | 97 |
| 035-PTsup | SST | 2014 | hg19 | chr3 | 187387985 | 187387985 | Missense_Mutation | SNP | C | C | T | novel | Somatic | R32H | 0.063316 | NM_001048.4 | 2522 | 2362 | 160 |
| 035-PTsup | NKX3-2 | 688 | hg19 | chr4 | 13543740 | 13543740 | Frame_Shift_Del | DEL | GTA | GTA | CT | novel | Somatic | Y293SfsTer61 | 0.105946 | NM_001189.4 | 924 | 826 | 98 |
| 035-PTsup | SHISA3 | 908 | hg19 | chr4 | 42403406 | 42403406 | Missense_Mutation | SNP | T | T | A | novel | Somatic | Y219N | 0.15493 | NM_001080505.3 | 852 | 720 | 132 |
| 035-PTsup | BMP3 | 1210 | hg19 | chr4 | 81952770 | 81952770 | Intron | SNP | G | G | A | novel | Somatic |  | 0.119186 | NM_001201.4 | 688 | 606 | 82 |
| 035-PTsup | BMPR1B | 164 | hg19 | chr4 | 96052434 | 96052434 | Missense_Mutation | SNP | G | G | A | novel | Somatic | E283K | 0.366525 | NM_001203.3 | 472 | 299 | 173 |
| 035-PTsup | RAPGEF2 | 1807 | hg19 | chr4 | 160243635 | 160243635 | Missense_Mutation | SNP | G | G | C | novel | Somatic | E330D | 0.11898 | NM_001351724.2 | 353 | 311 | 42 |
| 035-PTsup | TRIP13 | 591 | hg19 | chr5 | 896849 | 896849 | Missense_Mutation | SNP | G | G | A | novel | Somatic | E110K | 0.144099 | NM_004237.4 | 803 | 687 | 116 |
| 035-PTsup | ZNF131 | 114 | hg19 | chr5 | 43161942 | 43161942 | Missense_Mutation | SNP | A | A | T | novel | Somatic | Q321H | 0.090196 | NM_001330714.2 | 762 | 693 | 69 |
| 035-PTsup | ZNF131 | 114 | hg19 | chr5 | 43161980 | 43161980 | Missense_Mutation | SNP | A | A | T | novel | Somatic | E334V | 0.079422 | NM_001330714.2 | 831 | 765 | 66 |
| 035-PTsup | CMYA5 | 148 | hg19 | chr5 | 79030322 | 79030322 | Missense_Mutation | SNP | G | G | A | novel | Somatic | G1912R | 0.139535 | NM_153610.5 | 258 | 222 | 36 |
| 035-PTsup | ADGRV1 | 19 | hg19 | chr5 | 90079012 | 90079012 | Missense_Mutation | SNP | G | G | C | novel | Somatic | D4435H | 0.228986 | NM_032119.4 | 345 | 266 | 79 |
| 035-PTsup | ADGRV1 | 19 | hg19 | chr5 | 90459703 | 90459703 | Missense_Mutation | SNP | G | G | A | novel | Somatic | D6303N | 0.147727 | NM_032119.4 | 176 | 150 | 26 |
| 035-PTsup | TIFAB | 1613 | hg19 | chr5 | 134785391 | 134785391 | Missense_Mutation | SNP | C | C | T | novel | Somatic | R80H | 0.133796 | NM_001099221.2 | 1008 | 873 | 135 |
| 035-PTsup | TCERG1 | 212 | hg19 | chr5 | 145886722 | 145886722 | Frame_Shift_Ins | INS | - | - | A | novel | Somatic | R958EfsTer5 | 0.025581 | NM_006706.4 | 430 | 419 | 11 |
| 035-PTsup | GRM6 | 1946 | hg19 | chr5 | 178418886 | 178418886 | Nonsense_Mutation | SNP | G | G | A | novel | Somatic | Q235Ter | 0.118598 | NM_000843.4 | 368 | 324 | 44 |
| 035-PTsup | GPLD1 | 771 | hg19 | chr6 | 24454334 | 24454334 | Missense_Mutation | SNP | C | C | T | novel | Somatic | R415H | 0.116972 | NM_001503.4 | 435 | 384 | 51 |
| 035-PTsup | PRSS16 | 792 | hg19 | chr6 | 27219583 | 27219583 | Missense_Mutation | SNP | G | G | A | novel | Somatic | G258S | 0.282171 | NM_005865.4 | 1288 | 924 | 364 |
| 035-PTsup | HLA-A | 594 | hg19 | chr6 | 29910756 | 29910756 | Missense_Mutation | SNP | G | G | C | novel | Somatic | R99P | 0.3064 | NM_002116.8 | 2201 | 1526 | 675 |
| 035-PTsup | MAN1A1 | 23 | hg19 | chr6 | 119623173 | 119623173 | Missense_Mutation | SNP | C | C | G | novel | Somatic | E266Q | 0.3125 | NM_005907.4 | 176 | 121 | 55 |
| 035-PTsup | NEUROD6 | 824 | hg19 | chr7 | 31378098 | 31378098 | Missense_Mutation | DNP | GG | GG | TT | novel | Somatic | P262N | 0.157051 | NM_022728.4 | 308 | 259 | 49 |
| 035-PTsup | CACNA2D1 | 18 | hg19 | chr7 | 81978949 | 81978949 | Missense_Mutation | SNP | C | C | A | novel | Somatic | D38Y | 0.283854 | NM_000722.4 | 383 | 274 | 109 |
| 035-PTsup | DMTF1 | 1247 | hg19 | chr7 | 86811560 | 86811560 | Missense_Mutation | SNP | G | G | C | novel | Somatic | G243R | 0.078984 | NM_021145.3 | 709 | 653 | 56 |
| 035-PTsup | PCOLCE | 1349 | hg19 | chr7 | 100205359 | 100205359 | Missense_Mutation | SNP | T | T | A | novel | Somatic | L371Q | 0.202864 | NM_002593.4 | 419 | 334 | 85 |
| 035-PTsup | FLNC | 1565 | hg19 | chr7 | 128489519 | 128489519 | Nonsense_Mutation | SNP | G | G | T | novel | Somatic | E1696Ter | 0.321777 | NM_001458.4 | 920 | 623 | 297 |
| 035-PTsup | PRSS37 | 1664 | hg19 | chr7 | 141536990 | 141536990 | Nonsense_Mutation | SNP | G | G | T | novel | Somatic | C163Ter | 0.066667 | NM_001008270.3 | 536 | 500 | 36 |
| 035-PTsup | XKR9 | 1131 | hg19 | chr8 | 71619170 | 71619170 | Missense_Mutation | SNP | A | A | G | novel | Somatic | Y92C | 0.127119 | NM_001011720.2 | 354 | 309 | 45 |
| 035-PTsup | CNBD1 | 19 | hg19 | chr8 | 88249244 | 88249244 | Missense_Mutation | SNP | C | C | A | novel | Somatic | D225E | 0.092683 | NM_173538.3 | 410 | 372 | 38 |
| 035-PTsup | KIFC2 | 1696 | hg19 | chr8 | 145693255 | 145693255 | Nonsense_Mutation | SNP | C | C | G | novel | Somatic | S232Ter | 0.097701 | NM_145754.5 | 1392 | 1256 | 136 |
| 035-PTsup | TRIM32 | 1496 | hg19 | chr9 | 119461775 | 119461775 | Missense_Mutation | SNP | C | C | A | novel | Somatic | A585D | 0.162614 | NM_012210.3 | 657 | 550 | 107 |
| 035-PTsup | BRINP1 | 189 | hg19 | chr9 | 122075585 | 122075585 | Missense_Mutation | SNP | G | G | A | novel | Somatic | R17C | 0.169265 | NM_014618.3 | 449 | 373 | 76 |
| 035-PTsup | GTF3C5 | 202 | hg19 | chr9 | 135917592 | 135917592 | Missense_Mutation | SNP | G | G | A | novel | Somatic | R91K | 0.108761 | NM_001122823.2 | 331 | 295 | 36 |
| 035-PTsup | KCNT1 | 205 | hg19 | chr9 | 138660693 | 138660693 | Missense_Mutation | SNP | C | C | T | novel | Somatic | R474C | 0.090909 | NM_020822.3 | 758 | 689 | 69 |
| 035-PTsup | SNAPC4 | 1647 | hg19 | chr9 | 139278072 | 139278072 | Missense_Mutation | SNP | G | G | A | novel | Somatic | R517W | 0.159036 | NM_003086.3 | 1243 | 1045 | 198 |
| 035-PTsup | SEC16A | 1648 | hg19 | chr9 | 139357965 | 139357965 | Missense_Mutation | SNP | A | A | G | novel | Somatic | S1533P | 0.417132 | NM_014866.2 | 533 | 309 | 224 |
| 035-PTsup | ANKRD30A | 108 | hg19 | chr10 | 37414974 | 37414974 | Intron | SNP | G | G | T | novel | Somatic |  | 0.108214 | NM_052997.2 | 767 | 684 | 83 |
| 035-PTsup | LRRTM3 | 138 | hg19 | chr10 | 68688050 | 68688050 | Missense_Mutation | SNP | G | G | A | novel | Somatic | R459Q | 0.238602 | NM_178011.5 | 657 | 500 | 157 |
| 035-PTsup | FAM149B1 | 144 | hg19 | chr10 | 74999109 | 74999109 | Missense_Mutation | SNP | C | C | A | novel | Somatic | P548T | 0.226107 | NM_173348.2 | 428 | 331 | 97 |
| 035-PTsup | KIF20B | 160 | hg19 | chr10 | 91479249 | 91479249 | Missense_Mutation | SNP | C | C | T | novel | Somatic | S503L | 0.245902 | NM_001284259.1 | 366 | 276 | 90 |
| 035-PTsup | GPAM | 1454 | hg19 | chr10 | 113923493 | 113923493 | Frame_Shift_Del | DEL | CCTCC | CCTCC | - | novel | Somatic | R462IfsTer7 | 0.174888 | NM_001244949.2 | 223 | 184 | 39 |
| 035-PTsup | VWA2 | 1470 | hg19 | chr10 | 116050118 | 116050118 | Missense_Mutation | SNP | G | G | A | novel | Somatic | E748K | 0.247368 | NM_001272046.2 | 570 | 429 | 141 |
| 035-PTsup | DNHD1 | 634 | hg19 | chr11 | 6566758 | 6566758 | Nonsense_Mutation | SNP | G | G | A | novel | Somatic | W1530Ter | 0.129225 | NM_144666.2 | 503 | 438 | 65 |
| 035-PTsup | OR10A4 | 637 | hg19 | chr11 | 6898039 | 6898039 | Missense_Mutation | SNP | C | C | T | novel | Somatic | S54F | 0.291367 | NM_207186.2 | 278 | 197 | 81 |
| 035-PTsup | ABCC8 | 89 | hg19 | chr11 | 17450183 | 17450183 | Nonsense_Mutation | SNP | C | C | A | novel | Somatic | E618Ter | 0.269601 | NM_000352.5 | 727 | 531 | 196 |
| 035-PTsup | ALX4 | 115 | hg19 | chr11 | 44297029 | 44297029 | Missense_Mutation | SNP | G | G | A | novel | Somatic | R216W | 0.199588 | NM_021926.4 | 484 | 387 | 97 |
| 035-PTsup | OR5D18 | 1009 | hg19 | chr11 | 55587484 | 55587484 | Missense_Mutation | SNP | A | A | C | novel | Somatic | I127L | 0.090186 | NM_001001952.1 | 376 | 342 | 34 |
| 035-PTsup | DAGLA | 131 | hg19 | chr11 | 61511150 | 61511150 | Missense_Mutation | SNP | G | G | A | novel | Somatic | R773Q | 0.221514 | NM_006133.3 | 1254 | 976 | 278 |
| 035-PTsup | EHBP1L1 | 1083 | hg19 | chr11 | 65352691 | 65352691 | Missense_Mutation | SNP | G | G | A | novel | Somatic | E1188K | 0.285194 | NM_001099409.3 | 821 | 586 | 235 |
| 035-PTsup | CNTN5 | 20 | hg19 | chr11 | 99715873 | 99715873 | Missense_Mutation | SNP | G | G | C | novel | Somatic | L152F | 0.227898 | NM_014361.4 | 509 | 393 | 116 |
| 035-PTsup | TIGAR | 77 | hg19 | chr12 | 4459027 | 4459027 | Missense_Mutation | SNP | A | A | C | novel | Somatic | M79L | 0.077586 | NM_020375.3 | 464 | 428 | 36 |
| 035-PTsup | FGF6 | 619 | hg19 | chr12 | 4554469 | 4554469 | Missense_Mutation | SNP | A | A | T | novel | Somatic | C90S | 0.18997 | NM_020996.2 | 1316 | 1066 | 250 |
| 035-PTsup | ANO2 | 78 | hg19 | chr12 | 5841702 | 5841702 | Missense_Mutation | SNP | G | G | A | novel | Somatic | T515M | 0.405959 | NM_001278596.3 | 535 | 317 | 218 |
| 035-PTsup | SPX | 750 | hg19 | chr12 | 21679800 | 21679800 | Intron | SNP | T | T | C | novel | Somatic |  | 0.066845 | NM_030572.4 | 374 | 349 | 25 |
| 035-PTsup | RAPGEF3 | 952 | hg19 | chr12 | 48132036 | 48132036 | Splice_Region | SNP | G | G | C | novel | Somatic |  | 0.206499 | NM_001098531.3 | 954 | 757 | 197 |
| 035-PTsup | CALCOCO1 | 997 | hg19 | chr12 | 54107992 | 54107992 | Missense_Mutation | SNP | G | G | A | novel | Somatic | R506C | 0.075 | NM_020898.3 | 397 | 367 | 30 |
| 035-PTsup | OSBPL8 | 146 | hg19 | chr12 | 76752578 | 76752578 | Missense_Mutation | SNP | T | T | C | novel | Somatic | K781R | 0.405473 | NM_020841.5 | 401 | 238 | 163 |
| 035-PTsup | SETD1B | 1517 | hg19 | chr12 | 122247822 | 122247822 | Missense_Mutation | SNP | G | G | A | novel | Somatic | R324H | 0.134056 | NM_001353345.1 | 1747 | 1512 | 235 |
| 035-PTsup | SETD1B | 1517 | hg19 | chr12 | 122248399 | 122248399 | Frame_Shift_Del | DEL | C | C | - | novel | Somatic | L517Ter | 0.20685 | NM_001353345.1 | 1484 | 1176 | 308 |
| 035-PTsup | LOC101928841 | 1455 | hg19 | chr13 | 114061634 | 114061634 | Missense_Mutation | SNP | C | C | T | novel | Somatic | V291M | 0.298799 | NM_001304433.1 | 1329 | 931 | 398 |
| 035-PTsup | PLEKHH1 | 137 | hg19 | chr14 | 68045349 | 68045349 | Splice_Site | SNP | G | G | A | novel | Somatic |  | 0.144105 | NM_020715.3 | 228 | 195 | 33 |
| 035-PTsup | TRIP11 | 1290 | hg19 | chr14 | 92471589 | 92471589 | Missense_Mutation | SNP | C | C | T | novel | Somatic | E911K | 0.18661 | NM_004239.4 | 701 | 570 | 131 |
| 035-PTsup | RYR3 | 105 | hg19 | chr15 | 34118903 | 34118903 | Missense_Mutation | SNP | C | C | T | novel | Somatic | T3732M | 0.148936 | NM_001036.5 | 281 | 239 | 42 |
| 035-PTsup | TGM7 | 917 | hg19 | chr15 | 43571969 | 43571969 | Missense_Mutation | SNP | C | C | T | novel | Somatic | R511H | 0.128146 | NM_052955.3 | 873 | 761 | 112 |
| 035-PTsup | CRAMP1 | 74 | hg19 | chr16 | 1682331 | 1682331 | Nonsense_Mutation | SNP | G | G | A | novel | Somatic | W220Ter | 0.02 | NM_020825.4 | 800 | 784 | 16 |
| 035-PTsup | CDH15 | 158 | hg19 | chr16 | 89246752 | 89246752 | Missense_Mutation | SNP | G | G | C | novel | Somatic | D116H | 0.040559 | NM_004933.3 | 715 | 686 | 29 |
| 035-PTsup | PRPF8 | 74 | hg19 | chr17 | 1562699 | 1562699 | Missense_Mutation | SNP | G | G | A | novel | Somatic | S1697L | 0.21718 | NM_006445.4 | 617 | 483 | 134 |
| 035-PTsup | TP53 | 642 | hg19 | chr17 | 7577529 | 7577529 | Missense_Mutation | SNP | A | A | T | novel | Somatic | I251N | 0.236842 | NM_000546.5 | 340 | 259 | 81 |
| 035-PTsup | DRC3 | 721 | hg19 | chr17 | 17897729 | 17897729 | In_Frame_Ins | INS | - | - | AAG | novel | Somatic | K237dup | 0.213483 | NM_031294.4 | 796 | 625 | 171 |
| 035-PTsup | KRT34 | 886 | hg19 | chr17 | 39535628 | 39535628 | Nonsense_Mutation | SNP | C | C | A | novel | Somatic | E327Ter | 0.216263 | NM_021013.3 | 1156 | 906 | 250 |
| 035-PTsup | CNTNAP1 | 896 | hg19 | chr17 | 40835856 | 40835856 | Frame_Shift_Ins | INS | - | - | T | novel | Somatic | V30GfsTer63 | 0.071813 | NM_003632.3 | 554 | 514 | 40 |
| 035-PTsup | SPAG9 | 959 | hg19 | chr17 | 49067114 | 49067114 | Missense_Mutation | SNP | C | C | T | novel | Somatic | V913I | 0.100186 | NM_001130528.3 | 538 | 484 | 54 |
| 035-PTsup | EPG5 | 114 | hg19 | chr18 | 43505835 | 43505835 | Missense_Mutation | SNP | A | A | G | novel | Somatic | Y863H | 0.05492 | NM_020964.3 | 436 | 412 | 24 |
| 035-PTsup | LIPG | 944 | hg19 | chr18 | 47088689 | 47088689 | Missense_Mutation | SNP | C | C | G | novel | Somatic | S4C | 0.24408 | NM_006033.4 | 549 | 415 | 134 |
| 035-PTsup | TMPRSS9 | 603 | hg19 | chr19 | 2415826 | 2415826 | Missense_Mutation | SNP | C | C | T | novel | Somatic | H544Y | 0.254386 | NM_182973.2 | 1138 | 848 | 290 |
| 035-PTsup | ICAM5 | 664 | hg19 | chr19 | 10404565 | 10404565 | Missense_Mutation | SNP | G | G | A | novel | Somatic | E553K | 0.215898 | NM_003259.4 | 1016 | 796 | 220 |
| 035-PTsup | MAP1S | 721 | hg19 | chr19 | 17838543 | 17838543 | Missense_Mutation | SNP | C | C | T | novel | Somatic | P784S | 0.020408 | NM_018174.6 | 1273 | 1247 | 26 |
| 035-PTsup | CILP2 | 734 | hg19 | chr19 | 19651000 | 19651000 | Intron | SNP | G | G | T | novel | Somatic |  | 0.082579 | NM_153221.2 | 883 | 810 | 73 |
| 035-PTsup | ARHGEF1 | 908 | hg19 | chr19 | 42400486 | 42400486 | Missense_Mutation | SNP | C | C | T | novel | Somatic | P366L | 0.104167 | NM_199002.2 | 815 | 730 | 85 |
| 035-PTsup | XRCC1 | 921 | hg19 | chr19 | 44047567 | 44047567 | Missense_Mutation | SNP | A | A | G | novel | Somatic | Y627H | 0.13189 | NM_006297.2 | 508 | 441 | 67 |
| 035-PTsup | AP2A1 | 968 | hg19 | chr19 | 50305242 | 50305242 | Missense_Mutation | SNP | C | C | T | novel | Somatic | T653M | 0.128165 | NM_014203.3 | 1263 | 1101 | 162 |
| 035-PTsup | ZNF71 | 1020 | hg19 | chr19 | 57133517 | 57133517 | Missense_Mutation | SNP | C | C | G | novel | Somatic | H348D | 0.020174 | NM_001370215.1 | 2177 | 2133 | 44 |
| 035-PTsup | ZNF544 | 1033 | hg19 | chr19 | 58772937 | 58772937 | Missense_Mutation | SNP | G | G | T | novel | Somatic | G322V | 0.303716 | NM_001320769.1 | 619 | 431 | 188 |
| 035-PTsup | CSNK2A1 | 73 | hg19 | chr20 | 472948 | 472948 | Nonsense_Mutation | SNP | G | G | A | novel | Somatic | R191Ter | 0.184959 | NM_177559.3 | 492 | 401 | 91 |
| 035-PTsup | SLX4IP | 10 | hg19 | chr20 | 10603872 | 10603872 | Missense_Mutation | SNP | G | G | C | novel | Somatic | E358Q | 0.140071 | NM_001009608.3 | 564 | 485 | 79 |
| 035-PTsup | RALGAPA2 | 92 | hg19 | chr20 | 20693099 | 20693099 | Missense_Mutation | SNP | C | C | T | novel | Somatic | D9N | 0.146959 | NM_020343.4 | 592 | 505 | 87 |
| 035-PTsup | ZNF831 | 128 | hg19 | chr20 | 57767896 | 57767896 | Missense_Mutation | SNP | G | G | A | novel | Somatic | G608S | 0.020858 | NM_178457.2 | 1677 | 1642 | 35 |
| 035-PTsup | FNDC11 | 1059 | hg19 | chr20 | 62187726 | 62187726 | Missense_Mutation | SNP | C | C | T | novel | Somatic | S237L | 0.246744 | NM_001319153.2 | 1380 | 1039 | 341 |
| 035-PTsup | PKNOX1 | 115 | hg19 | chr21 | 44430160 | 44430160 | Splice_Region | SNP | C | C | G | novel | Somatic |  | 0.213235 | NM_004571.5 | 136 | 107 | 29 |
| 035-PTsup | SLC25A18 | 722 | hg19 | chr22 | 18066255 | 18066255 | Missense_Mutation | SNP | C | C | T | novel | Somatic | P122S | 0.183217 | NM_031481.3 | 714 | 583 | 131 |
| 035-PTsup | MAPK1 | 94 | hg19 | chr22 | 22162084 | 22162084 | Missense_Mutation | SNP | G | G | T | novel | Somatic | S57R | 0.057878 | NM_138957.3 | 621 | 585 | 36 |
| 035-PTsup | SFI1 | 103 | hg19 | chr22 | 32007831 | 32007831 | Splice_Region | SNP | G | G | A | novel | Somatic |  | 0.160126 | NM_001007467.3 | 637 | 535 | 102 |
| 035-PTsup | WBP2NL | 908 | hg19 | chr22 | 42415364 | 42415364 | Missense_Mutation | SNP | G | G | A | novel | Somatic | E38K | 0.212707 | NM_152613.3 | 722 | 568 | 154 |
| 035-PTsup | TTC38 | 941 | hg19 | chr22 | 46688705 | 46688705 | Missense_Mutation | SNP | G | G | A | novel | Somatic | R445H | 0.048753 | NM_017931.3 | 882 | 839 | 43 |
| 035-PTsup | PRICKLE3 | 959 | hg19 | chrX | 49032583 | 49032583 | Silent | SNP | C | C | A | novel | Somatic | L429= | 0.345661 | NM_006150.5 | 702 | 459 | 243 |
| 035-PTsup | USP51 | 1008 | hg19 | chrX | 55514601 | 55514601 | Missense_Mutation | SNP | A | A | C | novel | Somatic | F258V | 0.383838 | NM_201286.3 | 594 | 366 | 228 |
| 035-PTsup | MID2 | 175 | hg19 | chrX | 107170203 | 107170203 | Missense_Mutation | SNP | G | G | C | novel | Somatic | G703A | 0.114558 | NM_012216.4 | 415 | 367 | 48 |
| 035-PTsup | GAB3 | 1759 | hg19 | chrX | 153941648 | 153941648 | Missense_Mutation | SNP | C | C | T | novel | Somatic | A144T | 0.101523 | NM_001081573.3 | 586 | 526 | 60 |
| 035-PTdeep | UBXN11 | 788 | hg19 | chr1 | 26610850 | 26610850 | Splice_Site | DEL | CA | CA | - | novel | Somatic |  | 0.022455 | NM_183008.2 | 668 | 653 | 15 |
| 035-PTdeep | AGO4 | 107 | hg19 | chr1 | 36291040 | 36291040 | Missense_Mutation | SNP | C | C | T | novel | Somatic | P145S | 0.29563 | NM_017629.4 | 388 | 273 | 115 |
| 035-PTdeep | BEND5 | 960 | hg19 | chr1 | 49227065 | 49227065 | Missense_Mutation | SNP | C | C | G | novel | Somatic | E102Q | 0.313993 | NM_024603.4 | 293 | 201 | 92 |
| 035-PTdeep | S100A12 | 1754 | hg19 | chr1 | 153347008 | 153347008 | Missense_Mutation | SNP | G | G | A | novel | Somatic | R21W | 0.198953 | NM_005621.2 | 190 | 152 | 38 |
| 035-PTdeep | SYT14 | 273 | hg19 | chr1 | 210267826 | 210267826 | Frame_Shift_Del | DEL | CA | CA | T | novel | Somatic | S246FfsTer15 | 0.193277 | NM_001146261.2 | 353 | 284 | 69 |
| 035-PTdeep | ENAH | 288 | hg19 | chr1 | 225707032 | 225707032 | Missense_Mutation | SNP | G | G | A | novel | Somatic | R224W | 0.224189 | NM_001008493.3 | 1013 | 785 | 228 |
| 035-PTdeep | MTR | 37 | hg19 | chr1 | 237024474 | 237024474 | Frame_Shift_Del | DEL | A | A | - | novel | Somatic | K700NfsTer8 | 0.027027 | NM_000254.2 | 444 | 432 | 12 |
| 035-PTdeep | OR2W5 | 2474 | hg19 | chr1 | 247654830 | 247654830 | Missense_Mutation | SNP | C | C | A | novel | Somatic | A134E | 0.184713 | NM_001004698.2 | 314 | 256 | 58 |
| 035-PTdeep | OR2L5 | 2478 | hg19 | chr1 | 248185608 | 248185608 | Missense_Mutation | SNP | A | A | T | novel | Somatic | D120V | 0.245675 | NM_001258284.1 | 288 | 217 | 71 |
| 035-PTdeep | KCNK3 | 790 | hg19 | chr2 | 26951045 | 26951045 | Missense_Mutation | SNP | C | C | T | novel | Somatic | T265M | 0.020906 | NM_002246.3 | 1431 | 1401 | 30 |
| 035-PTdeep | RMDN2 | 876 | hg19 | chr2 | 38156790 | 38156790 | Missense_Mutation | SNP | C | C | T | novel | Somatic | H124Y | 0.091483 | NM_001170792.2 | 316 | 287 | 29 |
| 035-PTdeep | CNTNAP5 | 192 | hg19 | chr2 | 125261876 | 125261876 | Missense_Mutation | SNP | T | T | C | novel | Somatic | V357A | 0.111111 | NM_001367498.1 | 189 | 168 | 21 |
| 035-PTdeep | TMEM163 | 25 | hg19 | chr2 | 135476308 | 135476308 | Missense_Mutation | SNP | G | G | T | novel | Somatic | D66E | 0.126488 | NM_030923.5 | 671 | 586 | 85 |
| 035-PTdeep | EPHA4 | 35 | hg19 | chr2 | 222347074 | 222347074 | Missense_Mutation | SNP | G | G | A | novel | Somatic | A439V | 0.104418 | NM_001304536.1 | 248 | 222 | 26 |
| 035-PTdeep | EPHA4 | 35 | hg19 | chr2 | 222347362 | 222347362 | Missense_Mutation | SNP | G | G | A | novel | Somatic | S343F | 0.158209 | NM_001304536.1 | 334 | 281 | 53 |
| 035-PTdeep | TRPM8 | 4 | hg19 | chr2 | 234854632 | 234854632 | Missense_Mutation | SNP | C | C | T | novel | Somatic | R278W | 0.245833 | NM_024080.5 | 240 | 181 | 59 |
| 035-PTdeep | OXTR | 652 | hg19 | chr3 | 8809460 | 8809460 | In_Frame_Del | DEL | GCA | GCA | - | novel | Somatic | C138del | 0.116016 | NM_000916.3 | 973 | 860 | 113 |
| 035-PTdeep | CAND2 | 85 | hg19 | chr3 | 12875432 | 12875432 | In_Frame_Del | DEL | AGA | AGA | - | novel | Somatic | K1222del | 0.156997 | NM_001162499.2 | 292 | 246 | 46 |
| 035-PTdeep | BAP1 | 985 | hg19 | chr3 | 52437707 | 52437707 | Nonsense_Mutation | SNP | G | G | T | novel | Somatic | S485Ter | 0.283307 | NM_004656.4 | 1246 | 893 | 353 |
| 035-PTdeep | ERICH6 | 1732 | hg19 | chr3 | 150421525 | 150421525 | Missense_Mutation | SNP | T | T | A | novel | Somatic | E54V | 0.02518 | NM_152394.5 | 545 | 531 | 14 |
| 035-PTdeep | ARHGEF26 | 219 | hg19 | chr3 | 153840531 | 153840531 | Missense_Mutation | SNP | C | C | G | novel | Somatic | I250M | 0.101266 | NM_015595.4 | 711 | 639 | 72 |
| 035-PTdeep | PIK3CA | 243 | hg19 | chr3 | 178936091 | 178936091 | Missense_Mutation | SNP | G | G | A | novel | Somatic | E545K | 0.6337 | NM_006218.4 | 273 | 100 | 173 |
| 035-PTdeep | DVL3 | 1987 | hg19 | chr3 | 183888232 | 183888232 | Missense_Mutation | SNP | C | C | T | novel | Somatic | R614W | 0.063703 | NM_004423.4 | 2845 | 2663 | 182 |
| 035-PTdeep | MAP3K13 | 1996 | hg19 | chr3 | 185155325 | 185155325 | Missense_Mutation | SNP | C | C | T | novel | Somatic | A189V | 0.052564 | NM_004721.5 | 776 | 735 | 41 |
| 035-PTdeep | SST | 2014 | hg19 | chr3 | 187387985 | 187387985 | Missense_Mutation | SNP | C | C | T | novel | Somatic | R32H | 0.054811 | NM_001048.4 | 2531 | 2392 | 139 |
| 035-PTdeep | ZNF721 | 588 | hg19 | chr4 | 437056 | 437056 | Missense_Mutation | SNP | A | A | C | novel | Somatic | S400R | 0.01996 | NM_133474.4 | 501 | 491 | 10 |
| 035-PTdeep | NKX3-2 | 688 | hg19 | chr4 | 13543740 | 13543740 | Frame_Shift_Del | DEL | GTA | GTA | CT | novel | Somatic | Y293SfsTer61 | 0.136108 | NM_001189.4 | 889 | 768 | 121 |
| 035-PTdeep | SHISA3 | 908 | hg19 | chr4 | 42403406 | 42403406 | Missense_Mutation | SNP | T | T | A | novel | Somatic | Y219N | 0.159527 | NM_001080505.3 | 676 | 568 | 108 |
| 035-PTdeep | BMP3 | 1210 | hg19 | chr4 | 81952770 | 81952770 | Intron | SNP | G | G | A | novel | Somatic |  | 0.152047 | NM_001201.4 | 684 | 580 | 104 |
| 035-PTdeep | BMPR1B | 164 | hg19 | chr4 | 96052434 | 96052434 | Missense_Mutation | SNP | G | G | A | novel | Somatic | E283K | 0.332378 | NM_001203.3 | 349 | 233 | 116 |
| 035-PTdeep | RAPGEF2 | 1807 | hg19 | chr4 | 160243635 | 160243635 | Missense_Mutation | SNP | G | G | C | novel | Somatic | E330D | 0.118081 | NM_001351724.2 | 271 | 239 | 32 |
| 035-PTdeep | TRIP13 | 591 | hg19 | chr5 | 896849 | 896849 | Missense_Mutation | SNP | G | G | A | novel | Somatic | E110K | 0.084656 | NM_004237.4 | 567 | 519 | 48 |
| 035-PTdeep | ZNF131 | 114 | hg19 | chr5 | 43161942 | 43161942 | Missense_Mutation | SNP | A | A | T | novel | Somatic | Q321H | 0.075838 | NM_001330714.2 | 566 | 523 | 43 |
| 035-PTdeep | ZNF131 | 114 | hg19 | chr5 | 43161980 | 43161980 | Missense_Mutation | SNP | A | A | T | novel | Somatic | E334V | 0.072607 | NM_001330714.2 | 605 | 561 | 44 |
| 035-PTdeep | CMYA5 | 148 | hg19 | chr5 | 79030322 | 79030322 | Missense_Mutation | SNP | G | G | A | novel | Somatic | G1912R | 0.190751 | NM_153610.5 | 173 | 140 | 33 |
| 035-PTdeep | ADGRV1 | 19 | hg19 | chr5 | 90079012 | 90079012 | Missense_Mutation | SNP | G | G | C | novel | Somatic | D4435H | 0.126923 | NM_032119.4 | 260 | 227 | 33 |
| 035-PTdeep | ADGRV1 | 19 | hg19 | chr5 | 90459703 | 90459703 | Missense_Mutation | SNP | G | G | A | novel | Somatic | D6303N | 0.247191 | NM_032119.4 | 89 | 67 | 22 |
| 035-PTdeep | TIFAB | 1613 | hg19 | chr5 | 134785391 | 134785391 | Missense_Mutation | SNP | C | C | T | novel | Somatic | R80H | 0.166082 | NM_001099221.2 | 854 | 712 | 142 |
| 035-PTdeep | GRM6 | 1946 | hg19 | chr5 | 178418886 | 178418886 | Nonsense_Mutation | SNP | G | G | A | novel | Somatic | Q235Ter | 0.121495 | NM_000843.4 | 321 | 282 | 39 |
| 035-PTdeep | GPLD1 | 771 | hg19 | chr6 | 24454334 | 24454334 | Missense_Mutation | SNP | C | C | T | novel | Somatic | R415H | 0.136842 | NM_001503.4 | 285 | 246 | 39 |
| 035-PTdeep | PRSS16 | 792 | hg19 | chr6 | 27219583 | 27219583 | Missense_Mutation | SNP | G | G | A | novel | Somatic | G258S | 0.290518 | NM_005865.4 | 1480 | 1048 | 432 |
| 035-PTdeep | HLA-A | 594 | hg19 | chr6 | 29910756 | 29910756 | Missense_Mutation | SNP | G | G | C | novel | Somatic | R99P | 0.30399 | NM_002116.8 | 2301 | 1600 | 701 |
| 035-PTdeep | SRF | 914 | hg19 | chr6 | 43139485 | 43139485 | In_Frame_Del | DEL | GGC | GGC | - | novel | Somatic | G35del | 0.020115 | NM_003131.4 | 696 | 682 | 14 |
| 035-PTdeep | MAN1A1 | 23 | hg19 | chr6 | 119623173 | 119623173 | Missense_Mutation | SNP | C | C | G | novel | Somatic | E266Q | 0.244094 | NM_005907.4 | 127 | 96 | 31 |
| 035-PTdeep | NEUROD6 | 824 | hg19 | chr7 | 31378098 | 31378098 | Missense_Mutation | DNP | GG | GG | TT | novel | Somatic | P262N | 0.208122 | NM_022728.4 | 196 | 155 | 41 |
| 035-PTdeep | CACNA2D1 | 18 | hg19 | chr7 | 81978949 | 81978949 | Missense_Mutation | SNP | C | C | A | novel | Somatic | D38Y | 0.262658 | NM_000722.4 | 316 | 233 | 83 |
| 035-PTdeep | DMTF1 | 1247 | hg19 | chr7 | 86811560 | 86811560 | Missense_Mutation | SNP | G | G | C | novel | Somatic | G243R | 0.070652 | NM_021145.3 | 552 | 513 | 39 |
| 035-PTdeep | PCOLCE | 1349 | hg19 | chr7 | 100205359 | 100205359 | Missense_Mutation | SNP | T | T | A | novel | Somatic | L371Q | 0.204947 | NM_002593.4 | 282 | 224 | 58 |
| 035-PTdeep | FLNC | 1565 | hg19 | chr7 | 128489519 | 128489519 | Nonsense_Mutation | SNP | G | G | T | novel | Somatic | E1696Ter | 0.30663 | NM_001458.4 | 723 | 501 | 222 |
| 035-PTdeep | PRSS37 | 1664 | hg19 | chr7 | 141536990 | 141536990 | Nonsense_Mutation | SNP | G | G | T | novel | Somatic | C163Ter | 0.119221 | NM_001008270.3 | 410 | 361 | 49 |
| 035-PTdeep | XKR9 | 1131 | hg19 | chr8 | 71619170 | 71619170 | Missense_Mutation | SNP | A | A | G | novel | Somatic | Y92C | 0.080882 | NM_001011720.2 | 272 | 250 | 22 |
| 035-PTdeep | CNBD1 | 19 | hg19 | chr8 | 88249244 | 88249244 | Missense_Mutation | SNP | C | C | A | novel | Somatic | D225E | 0.120567 | NM_173538.3 | 282 | 248 | 34 |
| 035-PTdeep | KIFC2 | 1696 | hg19 | chr8 | 145693255 | 145693255 | Nonsense_Mutation | SNP | C | C | G | novel | Somatic | S232Ter | 0.092204 | NM_145754.5 | 1332 | 1209 | 123 |
| 035-PTdeep | TRIM32 | 1496 | hg19 | chr9 | 119461775 | 119461775 | Missense_Mutation | SNP | C | C | A | novel | Somatic | A585D | 0.139423 | NM_012210.3 | 416 | 358 | 58 |
| 035-PTdeep | GTF3C5 | 202 | hg19 | chr9 | 135917592 | 135917592 | Missense_Mutation | SNP | G | G | A | novel | Somatic | R91K | 0.177778 | NM_001122823.2 | 270 | 222 | 48 |
| 035-PTdeep | KCNT1 | 205 | hg19 | chr9 | 138660693 | 138660693 | Missense_Mutation | SNP | C | C | T | novel | Somatic | R474C | 0.112374 | NM_020822.3 | 792 | 703 | 89 |
| 035-PTdeep | CAMSAP1 | 1643 | hg19 | chr9 | 138715799 | 138715799 | Frame_Shift_Ins | INS | - | - | T | novel | Somatic | T466NfsTer19 | 0.106061 | NM_015447.4 | 197 | 176 | 21 |
| 035-PTdeep | SNAPC4 | 1647 | hg19 | chr9 | 139278072 | 139278072 | Missense_Mutation | SNP | G | G | A | novel | Somatic | R517W | 0.146774 | NM_003086.3 | 1240 | 1058 | 182 |
| 035-PTdeep | SEC16A | 1648 | hg19 | chr9 | 139357965 | 139357965 | Missense_Mutation | SNP | A | A | G | novel | Somatic | S1533P | 0.386555 | NM_014866.2 | 356 | 218 | 138 |
| 035-PTdeep | ANKRD30A | 108 | hg19 | chr10 | 37414974 | 37414974 | Intron | SNP | G | G | T | novel | Somatic |  | 0.122421 | NM_052997.2 | 725 | 636 | 89 |
| 035-PTdeep | LRRTM3 | 138 | hg19 | chr10 | 68688050 | 68688050 | Missense_Mutation | SNP | G | G | A | novel | Somatic | R459Q | 0.209503 | NM_178011.5 | 462 | 365 | 97 |
| 035-PTdeep | FAM149B1 | 144 | hg19 | chr10 | 74999109 | 74999109 | Missense_Mutation | SNP | C | C | A | novel | Somatic | P548T | 0.30033 | NM_173348.2 | 303 | 212 | 91 |
| 035-PTdeep | KIF20B | 160 | hg19 | chr10 | 91479249 | 91479249 | Missense_Mutation | SNP | C | C | T | novel | Somatic | S503L | 0.236948 | NM_001284259.1 | 249 | 190 | 59 |
| 035-PTdeep | GPAM | 1454 | hg19 | chr10 | 113923493 | 113923493 | Frame_Shift_Del | DEL | CCTCC | CCTCC | - | novel | Somatic | R462IfsTer7 | 0.083969 | NM_001244949.2 | 131 | 120 | 11 |
| 035-PTdeep | VWA2 | 1470 | hg19 | chr10 | 116050118 | 116050118 | Missense_Mutation | SNP | G | G | A | novel | Somatic | E748K | 0.258206 | NM_001272046.2 | 456 | 338 | 118 |
| 035-PTdeep | DNHD1 | 634 | hg19 | chr11 | 6566758 | 6566758 | Nonsense_Mutation | SNP | G | G | A | novel | Somatic | W1530Ter | 0.130556 | NM_144666.2 | 360 | 313 | 47 |
| 035-PTdeep | OR10A4 | 637 | hg19 | chr11 | 6898039 | 6898039 | Missense_Mutation | SNP | C | C | T | novel | Somatic | S54F | 0.209756 | NM_207186.2 | 205 | 162 | 43 |
| 035-PTdeep | ABCC8 | 89 | hg19 | chr11 | 17450183 | 17450183 | Nonsense_Mutation | SNP | C | C | A | novel | Somatic | E618Ter | 0.292731 | NM_000352.5 | 509 | 360 | 149 |
| 035-PTdeep | ALX4 | 115 | hg19 | chr11 | 44297029 | 44297029 | Missense_Mutation | SNP | G | G | A | novel | Somatic | R216W | 0.187135 | NM_021926.4 | 338 | 274 | 64 |
| 035-PTdeep | OR5D18 | 1009 | hg19 | chr11 | 55587484 | 55587484 | Missense_Mutation | SNP | A | A | C | novel | Somatic | I127L | 0.089069 | NM_001001952.1 | 247 | 225 | 22 |
| 035-PTdeep | DAGLA | 131 | hg19 | chr11 | 61511150 | 61511150 | Missense_Mutation | SNP | G | G | A | novel | Somatic | R773Q | 0.238128 | NM_006133.3 | 1451 | 1105 | 346 |
| 035-PTdeep | EHBP1L1 | 1083 | hg19 | chr11 | 65352691 | 65352691 | Missense_Mutation | SNP | G | G | A | novel | Somatic | E1188K | 0.293144 | NM_001099409.3 | 845 | 597 | 248 |
| 035-PTdeep | NEU3 | 144 | hg19 | chr11 | 74716717 | 74716717 | Missense_Mutation | SNP | G | G | T | novel | Somatic | W189L | 0.054545 | NM_006656.6 | 274 | 259 | 15 |
| 035-PTdeep | CNTN5 | 20 | hg19 | chr11 | 99715873 | 99715873 | Missense_Mutation | SNP | G | G | C | novel | Somatic | L152F | 0.231672 | NM_014361.4 | 341 | 262 | 79 |
| 035-PTdeep | TIGAR | 77 | hg19 | chr12 | 4459027 | 4459027 | Missense_Mutation | SNP | A | A | C | novel | Somatic | M79L | 0.120401 | NM_020375.3 | 299 | 263 | 36 |
| 035-PTdeep | FGF6 | 619 | hg19 | chr12 | 4554469 | 4554469 | Missense_Mutation | SNP | A | A | T | novel | Somatic | C90S | 0.170523 | NM_020996.2 | 1166 | 967 | 199 |
| 035-PTdeep | ANO2 | 78 | hg19 | chr12 | 5841702 | 5841702 | Missense_Mutation | SNP | G | G | A | novel | Somatic | T515M | 0.379747 | NM_001278596.3 | 394 | 244 | 150 |
| 035-PTdeep | SPX | 750 | hg19 | chr12 | 21679800 | 21679800 | Intron | SNP | T | T | C | novel | Somatic |  | 0.109541 | NM_030572.4 | 283 | 252 | 31 |
| 035-PTdeep | RAPGEF3 | 952 | hg19 | chr12 | 48132036 | 48132036 | Splice_Region | SNP | G | G | C | novel | Somatic |  | 0.190083 | NM_001098531.3 | 726 | 588 | 138 |
| 035-PTdeep | CALCOCO1 | 997 | hg19 | chr12 | 54107992 | 54107992 | Missense_Mutation | SNP | G | G | A | novel | Somatic | R506C | 0.09292 | NM_020898.3 | 225 | 204 | 21 |
| 035-PTdeep | OSBPL8 | 146 | hg19 | chr12 | 76752578 | 76752578 | Missense_Mutation | SNP | T | T | C | novel | Somatic | K781R | 0.358621 | NM_020841.5 | 290 | 186 | 104 |
| 035-PTdeep | SETD1B | 1517 | hg19 | chr12 | 122247822 | 122247822 | Missense_Mutation | SNP | G | G | A | novel | Somatic | R324H | 0.111053 | NM_001353345.1 | 1914 | 1701 | 213 |
| 035-PTdeep | SETD1B | 1517 | hg19 | chr12 | 122248399 | 122248399 | Frame_Shift_Del | DEL | C | C | - | novel | Somatic | L517Ter | 0.207883 | NM_001353345.1 | 1567 | 1240 | 327 |
| 035-PTdeep | LOC101928841 | 1455 | hg19 | chr13 | 114061634 | 114061634 | Missense_Mutation | SNP | C | C | T | novel | Somatic | V291M | 0.269426 | NM_001304433.1 | 1372 | 1001 | 371 |
| 035-PTdeep | PLEKHH1 | 137 | hg19 | chr14 | 68045349 | 68045349 | Splice_Site | SNP | G | G | A | novel | Somatic |  | 0.255814 | NM_020715.3 | 169 | 125 | 44 |
| 035-PTdeep | TRIP11 | 1290 | hg19 | chr14 | 92471589 | 92471589 | Missense_Mutation | SNP | C | C | T | novel | Somatic | E911K | 0.259091 | NM_004239.4 | 440 | 326 | 114 |
| 035-PTdeep | RYR3 | 105 | hg19 | chr15 | 34118903 | 34118903 | Missense_Mutation | SNP | C | C | T | novel | Somatic | T3732M | 0.135417 | NM_001036.5 | 191 | 165 | 26 |
| 035-PTdeep | TGM7 | 917 | hg19 | chr15 | 43571969 | 43571969 | Missense_Mutation | SNP | C | C | T | novel | Somatic | R511H | 0.123839 | NM_052955.3 | 646 | 566 | 80 |
| 035-PTdeep | KIAA1024 | 1193 | hg19 | chr15 | 79750585 | 79750585 | Frame_Shift_Ins | INS | - | - | A | novel | Somatic | S702KfsTer9 | 0.038314 | NM_015206.3 | 261 | 251 | 10 |
| 035-PTdeep | CDH15 | 158 | hg19 | chr16 | 89246752 | 89246752 | Missense_Mutation | SNP | G | G | C | novel | Somatic | D116H | 0.116592 | NM_004933.3 | 669 | 591 | 78 |
| 035-PTdeep | PRPF8 | 74 | hg19 | chr17 | 1562699 | 1562699 | Missense_Mutation | SNP | G | G | A | novel | Somatic | S1697L | 0.25 | NM_006445.4 | 467 | 350 | 117 |
| 035-PTdeep | CAMTA2 | 622 | hg19 | chr17 | 4875738 | 4875738 | Frame_Shift_Del | DEL | G | G | - | novel | Somatic | P866LfsTer9 | 0.03481 | NM_015099.4 | 316 | 305 | 11 |
| 035-PTdeep | TP53 | 642 | hg19 | chr17 | 7577529 | 7577529 | Missense_Mutation | SNP | A | A | T | novel | Somatic | I251N | 0.220065 | NM_000546.5 | 309 | 241 | 68 |
| 035-PTdeep | DRC3 | 721 | hg19 | chr17 | 17897729 | 17897729 | In_Frame_Ins | INS | - | - | AAG | novel | Somatic | K237dup | 0.202401 | NM_031294.4 | 579 | 461 | 118 |
| 035-PTdeep | KRT34 | 886 | hg19 | chr17 | 39535628 | 39535628 | Nonsense_Mutation | SNP | C | C | A | novel | Somatic | E327Ter | 0.200634 | NM_021013.3 | 947 | 757 | 190 |
| 035-PTdeep | CNTNAP1 | 896 | hg19 | chr17 | 40835856 | 40835856 | Frame_Shift_Ins | INS | - | - | T | novel | Somatic | V30GfsTer63 | 0.084668 | NM_003632.3 | 437 | 400 | 37 |
| 035-PTdeep | SPAG9 | 959 | hg19 | chr17 | 49067114 | 49067114 | Missense_Mutation | SNP | C | C | T | novel | Somatic | V913I | 0.10733 | NM_001130528.3 | 381 | 340 | 41 |
| 035-PTdeep | LIPG | 944 | hg19 | chr18 | 47088689 | 47088689 | Missense_Mutation | SNP | C | C | G | novel | Somatic | S4C | 0.276712 | NM_006033.4 | 365 | 264 | 101 |
| 035-PTdeep | TMPRSS9 | 603 | hg19 | chr19 | 2415826 | 2415826 | Missense_Mutation | SNP | C | C | T | novel | Somatic | H544Y | 0.230228 | NM_182973.2 | 1136 | 874 | 262 |
| 035-PTdeep | ICAM5 | 664 | hg19 | chr19 | 10404565 | 10404565 | Missense_Mutation | SNP | G | G | A | novel | Somatic | E553K | 0.22493 | NM_003259.4 | 1064 | 824 | 240 |
| 035-PTdeep | CILP2 | 734 | hg19 | chr19 | 19651000 | 19651000 | Intron | SNP | G | G | T | novel | Somatic |  | 0.104575 | NM_153221.2 | 918 | 822 | 96 |
| 035-PTdeep | ARHGEF1 | 908 | hg19 | chr19 | 42400486 | 42400486 | Missense_Mutation | SNP | C | C | T | novel | Somatic | P366L | 0.148741 | NM_199002.2 | 873 | 743 | 130 |
| 035-PTdeep | AP2A1 | 968 | hg19 | chr19 | 50305242 | 50305242 | Missense_Mutation | SNP | C | C | T | novel | Somatic | T653M | 0.144699 | NM_014203.3 | 1392 | 1190 | 202 |
| 035-PTdeep | ZNF544 | 1033 | hg19 | chr19 | 58772937 | 58772937 | Missense_Mutation | SNP | G | G | T | novel | Somatic | G322V | 0.290123 | NM_001320769.1 | 485 | 344 | 141 |
| 035-PTdeep | CSNK2A1 | 73 | hg19 | chr20 | 472948 | 472948 | Nonsense_Mutation | SNP | G | G | A | novel | Somatic | R191Ter | 0.138889 | NM_177559.3 | 359 | 309 | 50 |
| 035-PTdeep | SLX4IP | 10 | hg19 | chr20 | 10603872 | 10603872 | Missense_Mutation | SNP | G | G | C | novel | Somatic | E358Q | 0.134715 | NM_001009608.3 | 385 | 333 | 52 |
| 035-PTdeep | RALGAPA2 | 92 | hg19 | chr20 | 20693099 | 20693099 | Missense_Mutation | SNP | C | C | T | novel | Somatic | D9N | 0.110307 | NM_020343.4 | 553 | 492 | 61 |
| 035-PTdeep | FNDC11 | 1059 | hg19 | chr20 | 62187726 | 62187726 | Missense_Mutation | SNP | C | C | T | novel | Somatic | S237L | 0.246407 | NM_001319153.2 | 1460 | 1100 | 360 |
| 035-PTdeep | PKNOX1 | 115 | hg19 | chr21 | 44430160 | 44430160 | Splice_Region | SNP | C | C | G | novel | Somatic |  | 0.171171 | NM_004571.5 | 111 | 92 | 19 |
| 035-PTdeep | SLC25A18 | 722 | hg19 | chr22 | 18066255 | 18066255 | Missense_Mutation | SNP | C | C | T | novel | Somatic | P122S | 0.161765 | NM_031481.3 | 609 | 510 | 99 |
| 035-PTdeep | MAPK1 | 94 | hg19 | chr22 | 22162084 | 22162084 | Missense_Mutation | SNP | G | G | T | novel | Somatic | S57R | 0.070081 | NM_138957.3 | 371 | 345 | 26 |
| 035-PTdeep | SFI1 | 103 | hg19 | chr22 | 32007831 | 32007831 | Splice_Region | SNP | G | G | A | novel | Somatic |  | 0.198661 | NM_001007467.3 | 448 | 359 | 89 |
| 035-PTdeep | WBP2NL | 908 | hg19 | chr22 | 42415364 | 42415364 | Missense_Mutation | SNP | G | G | A | novel | Somatic | E38K | 0.157424 | NM_152613.3 | 558 | 470 | 88 |
| 035-PTdeep | TTC38 | 941 | hg19 | chr22 | 46688705 | 46688705 | Missense_Mutation | SNP | G | G | A | novel | Somatic | R445H | 0.074029 | NM_017931.3 | 824 | 763 | 61 |
| 035-PTdeep | PRICKLE3 | 959 | hg19 | chrX | 49032583 | 49032583 | Silent | SNP | C | C | A | novel | Somatic | L429= | 0.318584 | NM_006150.5 | 678 | 462 | 216 |
| 035-PTdeep | USP51 | 1008 | hg19 | chrX | 55514601 | 55514601 | Missense_Mutation | SNP | A | A | C | novel | Somatic | F258V | 0.347291 | NM_201286.3 | 404 | 263 | 141 |
| 035-PTdeep | MID2 | 175 | hg19 | chrX | 107170203 | 107170203 | Missense_Mutation | SNP | G | G | C | novel | Somatic | G703A | 0.130909 | NM_012216.4 | 274 | 238 | 36 |
| 035-PTdeep | GAB3 | 1759 | hg19 | chrX | 153941648 | 153941648 | Missense_Mutation | SNP | C | C | T | novel | Somatic | A144T | 0.130631 | NM_001081573.3 | 442 | 384 | 58 |
| 035-LNmet | AGO4 | 107 | hg19 | chr1 | 36291040 | 36291040 | Missense_Mutation | SNP | C | C | T | novel | Somatic | P145S | 0.12987 | NM_017629.4 | 537 | 467 | 70 |
| 035-LNmet | STIL | 949 | hg19 | chr1 | 47746533 | 47746533 | Missense_Mutation | SNP | C | C | T | novel | Somatic | D533N | 0.091278 | NM_001048166.1 | 492 | 447 | 45 |
| 035-LNmet | BEND5 | 960 | hg19 | chr1 | 49227065 | 49227065 | Missense_Mutation | SNP | C | C | G | novel | Somatic | E102Q | 0.154839 | NM_024603.4 | 310 | 262 | 48 |
| 035-LNmet | WDR63 | 154 | hg19 | chr1 | 85559145 | 85559145 | Missense_Mutation | SNP | G | G | A | novel | Somatic | E288K | 0.043796 | NM_145172.5 | 411 | 393 | 18 |
| 035-LNmet | C1orf116 | 2165 | hg19 | chr1 | 207200866 | 207200866 | Missense_Mutation | SNP | C | C | T | novel | Somatic | M26I | 0.022046 | NM_023938.6 | 1133 | 1108 | 25 |
| 035-LNmet | SYT14 | 273 | hg19 | chr1 | 210267826 | 210267826 | Frame_Shift_Del | DEL | CA | CA | T | novel | Somatic | S246FfsTer15 | 0.167266 | NM_001146261.2 | 553 | 460 | 93 |
| 035-LNmet | ENAH | 288 | hg19 | chr1 | 225707032 | 225707032 | Missense_Mutation | SNP | G | G | A | novel | Somatic | R224W | 0.171815 | NM_001008493.3 | 1546 | 1279 | 267 |
| 035-LNmet | OR2W5 | 2474 | hg19 | chr1 | 247654830 | 247654830 | Missense_Mutation | SNP | C | C | A | novel | Somatic | A134E | 0.304527 | NM_001004698.2 | 485 | 337 | 148 |
| 035-LNmet | OR2L5 | 2478 | hg19 | chr1 | 248185608 | 248185608 | Missense_Mutation | SNP | A | A | T | novel | Somatic | D120V | 0.266515 | NM_001258284.1 | 439 | 322 | 117 |
| 035-LNmet | CMPK2 | 638 | hg19 | chr2 | 7005680 | 7005680 | Missense_Mutation | SNP | C | C | T | novel | Somatic | D50N | 0.047619 | NM_207315.3 | 685 | 652 | 33 |
| 035-LNmet | NCOA1 | 96 | hg19 | chr2 | 24929536 | 24929536 | In_Frame_Del | DEL | TGTGGC | TGTGGC | - | novel | Somatic | V400_A401del | 0.026279 | NM_003743.5 | 722 | 703 | 19 |
| 035-LNmet | ALMS1 | 143 | hg19 | chr2 | 73679577 | 73679577 | Missense_Mutation | SNP | G | G | A | novel | Somatic | E1976K | 0.157643 | NM_015120.4 | 627 | 528 | 99 |
| 035-LNmet | POU3F3 | 1389 | hg19 | chr2 | 105472382 | 105472382 | In_Frame_Del | DEL | GCC | GCC | - | novel | Somatic | P144del | 0.033333 | NM_006236.3 | 305 | 294 | 11 |
| 035-LNmet | CNTNAP5 | 192 | hg19 | chr2 | 125261876 | 125261876 | Missense_Mutation | SNP | T | T | C | novel | Somatic | V357A | 0.2 | NM_001367498.1 | 285 | 228 | 57 |
| 035-LNmet | TMEM163 | 25 | hg19 | chr2 | 135476308 | 135476308 | Missense_Mutation | SNP | G | G | T | novel | Somatic | D66E | 0.242634 | NM_030923.5 | 575 | 435 | 140 |
| 035-LNmet | COL5A2 | 254 | hg19 | chr2 | 189927623 | 189927623 | Missense_Mutation | SNP | C | C | T | novel | Somatic | E649K | 0.027555 | NM_000393.5 | 870 | 846 | 24 |
| 035-LNmet | EPHA4 | 35 | hg19 | chr2 | 222347074 | 222347074 | Missense_Mutation | SNP | G | G | A | novel | Somatic | A439V | 0.123333 | NM_001304536.1 | 299 | 262 | 37 |
| 035-LNmet | EPHA4 | 35 | hg19 | chr2 | 222347362 | 222347362 | Missense_Mutation | SNP | G | G | A | novel | Somatic | S343F | 0.163636 | NM_001304536.1 | 495 | 414 | 81 |
| 035-LNmet | PID1 | 292 | hg19 | chr2 | 229890590 | 229890590 | Missense_Mutation | SNP | C | C | T | novel | Somatic | A171T | 0.18541 | NM_001330156.1 | 327 | 266 | 61 |
| 035-LNmet | TRPM8 | 4 | hg19 | chr2 | 234854632 | 234854632 | Missense_Mutation | SNP | C | C | T | novel | Somatic | R278W | 0.240093 | NM_024080.5 | 429 | 326 | 103 |
| 035-LNmet | OXTR | 652 | hg19 | chr3 | 8809460 | 8809460 | In_Frame_Del | DEL | GCA | GCA | - | novel | Somatic | C138del | 0.195055 | NM_000916.3 | 1092 | 879 | 213 |
| 035-LNmet | CAND2 | 85 | hg19 | chr3 | 12875432 | 12875432 | In_Frame_Del | DEL | AGA | AGA | - | novel | Somatic | K1222del | 0.197211 | NM_001162499.2 | 502 | 403 | 99 |
| 035-LNmet | RARB | 97 | hg19 | chr3 | 25622110 | 25622110 | Missense_Mutation | SNP | G | G | T | novel | Somatic | C235F | 0.224417 | NM_001290216.2 | 554 | 429 | 125 |
| 035-LNmet | BSN | 120 | hg19 | chr3 | 49691173 | 49691173 | Missense_Mutation | SNP | G | G | A | novel | Somatic | R1395Q | 0.229058 | NM_003458.4 | 762 | 587 | 175 |
| 035-LNmet | BAP1 | 985 | hg19 | chr3 | 52437707 | 52437707 | Nonsense_Mutation | SNP | G | G | T | novel | Somatic | S485Ter | 0.239205 | NM_004656.4 | 1760 | 1339 | 421 |
| 035-LNmet | PLXND1 | 1571 | hg19 | chr3 | 129304863 | 129304863 | Missense_Mutation | SNP | C | C | T | novel | Somatic | E595K | 0.199687 | NM_015103.2 | 1272 | 1017 | 255 |
| 035-LNmet | CLSTN2 | 206 | hg19 | chr3 | 140281937 | 140281937 | Missense_Mutation | SNP | G | G | A | novel | Somatic | E792K | 0.037344 | NM_022131.3 | 481 | 463 | 18 |
| 035-LNmet | ARHGEF26 | 219 | hg19 | chr3 | 153840531 | 153840531 | Missense_Mutation | SNP | C | C | G | novel | Somatic | I250M | 0.172 | NM_015595.4 | 1000 | 828 | 172 |
| 035-LNmet | PIK3CA | 243 | hg19 | chr3 | 178936091 | 178936091 | Missense_Mutation | SNP | G | G | A | novel | Somatic | E545K | 0.737757 | NM_006218.4 | 633 | 166 | 467 |
| 035-LNmet | MAP3K13 | 1996 | hg19 | chr3 | 185155325 | 185155325 | Missense_Mutation | SNP | C | C | T | novel | Somatic | A189V | 0.15235 | NM_004721.5 | 613 | 519 | 94 |
| 035-LNmet | SST | 2014 | hg19 | chr3 | 187387985 | 187387985 | Missense_Mutation | SNP | C | C | T | novel | Somatic | R32H | 0.08021 | NM_001048.4 | 3028 | 2784 | 244 |
| 035-LNmet | SHISA3 | 908 | hg19 | chr4 | 42403406 | 42403406 | Missense_Mutation | SNP | T | T | A | novel | Somatic | Y219N | 0.304153 | NM_001080505.3 | 891 | 620 | 271 |
| 035-LNmet | BMP3 | 1210 | hg19 | chr4 | 81952770 | 81952770 | Intron | SNP | G | G | A | novel | Somatic |  | 0.305428 | NM_001201.4 | 1087 | 755 | 332 |
| 035-LNmet | WDFY3 | 154 | hg19 | chr4 | 85661470 | 85661470 | Nonsense_Mutation | SNP | G | G | A | novel | Somatic | Q2112Ter | 0.037468 | NM_014991.5 | 773 | 744 | 29 |
| 035-LNmet | BMPR1B | 164 | hg19 | chr4 | 96052434 | 96052434 | Missense_Mutation | SNP | G | G | A | novel | Somatic | E283K | 0.247619 | NM_001203.3 | 420 | 316 | 104 |
| 035-LNmet | NEK1 | 235 | hg19 | chr4 | 170510647 | 170510647 | Missense_Mutation | ONP | CTTTAGT | CTTTAGT | TTTTAGG | novel | Somatic | T137_D139delinsPKN | 0.152824 | NM_001199397.2 | 298 | 252 | 46 |
| 035-LNmet | TRIP13 | 591 | hg19 | chr5 | 896849 | 896849 | Missense_Mutation | SNP | G | G | A | novel | Somatic | E110K | 0.2125 | NM_004237.4 | 799 | 629 | 170 |
| 035-LNmet | CMYA5 | 148 | hg19 | chr5 | 79030322 | 79030322 | Missense_Mutation | SNP | G | G | A | novel | Somatic | G1912R | 0.319298 | NM_153610.5 | 285 | 194 | 91 |
| 035-LNmet | ADGRV1 | 19 | hg19 | chr5 | 90459703 | 90459703 | Missense_Mutation | SNP | G | G | A | novel | Somatic | D6303N | 0.291429 | NM_032119.4 | 175 | 124 | 51 |
| 035-LNmet | GRM6 | 1946 | hg19 | chr5 | 178418886 | 178418886 | Nonsense_Mutation | SNP | G | G | A | novel | Somatic | Q235Ter | 0.249448 | NM_000843.4 | 452 | 339 | 113 |
| 035-LNmet | PRSS16 | 792 | hg19 | chr6 | 27219583 | 27219583 | Missense_Mutation | SNP | G | G | A | novel | Somatic | G258S | 0.244063 | NM_005865.4 | 1511 | 1141 | 370 |
| 035-LNmet | HIST1H1B | 797 | hg19 | chr6 | 27835230 | 27835230 | Missense_Mutation | SNP | C | C | G | novel | Somatic | K26N | 0.043233 | NM_005322.2 | 532 | 509 | 23 |
| 035-LNmet | HLA-A | 594 | hg19 | chr6 | 29910756 | 29910756 | Missense_Mutation | SNP | G | G | C | novel | Somatic | R99P | 0.282237 | NM_002116.8 | 2625 | 1883 | 742 |
| 035-LNmet | SRF | 914 | hg19 | chr6 | 43139485 | 43139485 | In_Frame_Del | DEL | GGC | GGC | - | novel | Somatic | G35del | 0.025994 | NM_003131.4 | 654 | 637 | 17 |
| 035-LNmet | CD2AP | 118 | hg19 | chr6 | 47580228 | 47580228 | Nonsense_Mutation | SNP | C | C | T | novel | Somatic | R612Ter | 0.177632 | NM_012120.3 | 302 | 248 | 54 |
| 035-LNmet | MAN1A1 | 23 | hg19 | chr6 | 119623173 | 119623173 | Missense_Mutation | SNP | C | C | G | novel | Somatic | E266Q | 0.25 | NM_005907.4 | 200 | 150 | 50 |
| 035-LNmet | RNF146 | 1558 | hg19 | chr6 | 127608233 | 127608233 | Missense_Mutation | SNP | G | G | C | novel | Somatic | E159Q | 0.178042 | NM_001242849.1 | 337 | 277 | 60 |
| 035-LNmet | PCOLCE | 1349 | hg19 | chr7 | 100205359 | 100205359 | Missense_Mutation | SNP | T | T | A | novel | Somatic | L371Q | 0.451064 | NM_002593.4 | 469 | 257 | 212 |
| 035-LNmet | MUC17 | 1353 | hg19 | chr7 | 100679811 | 100679811 | Missense_Mutation | SNP | C | C | G | novel | Somatic | S1705C | 0.028986 | NM_001040105.2 | 1449 | 1407 | 42 |
| 035-LNmet | CADPS2 | 189 | hg19 | chr7 | 122033257 | 122033257 | Missense_Mutation | SNP | C | C | T | novel | Somatic | E1008K | 0.055375 | NM_001363389.1 | 614 | 580 | 34 |
| 035-LNmet | FLNC | 1565 | hg19 | chr7 | 128489519 | 128489519 | Nonsense_Mutation | SNP | G | G | T | novel | Somatic | E1696Ter | 0.221669 | NM_001458.4 | 801 | 623 | 178 |
| 035-LNmet | KCP | 1565 | hg19 | chr7 | 128531103 | 128531103 | Missense_Mutation | SNP | G | G | C | novel | Somatic | R673G | 0.022758 | NM_001366122.1 | 747 | 730 | 17 |
| 035-LNmet | PRDM14 | 1126 | hg19 | chr8 | 70981914 | 70981914 | Missense_Mutation | SNP | G | G | A | novel | Somatic | S61F | 0.020751 | NM_024504.4 | 1009 | 988 | 21 |
| 035-LNmet | XKR9 | 1131 | hg19 | chr8 | 71619170 | 71619170 | Missense_Mutation | SNP | A | A | G | novel | Somatic | Y92C | 0.205962 | NM_001011720.2 | 369 | 293 | 76 |
| 035-LNmet | SCRIB | 1690 | hg19 | chr8 | 144874944 | 144874944 | Missense_Mutation | SNP | C | C | T | novel | Somatic | E1371K | 0.032987 | NM_182706.5 | 2208 | 2135 | 73 |
| 035-LNmet | EPPK1 | 1690 | hg19 | chr8 | 144943084 | 144943084 | Frame_Shift_Del | DEL | GGTGT | GGTGT | - | novel | Somatic | H1445RfsTer17 | 0.040722 | NM_031308.4 | 2154 | 2066 | 88 |
| 035-LNmet | KIFC2 | 1696 | hg19 | chr8 | 145693255 | 145693255 | Nonsense_Mutation | SNP | C | C | G | novel | Somatic | S232Ter | 0.153005 | NM_145754.5 | 1462 | 1238 | 224 |
| 035-LNmet | RPL8 | 1699 | hg19 | chr8 | 146017394 | 146017394 | Missense_Mutation | SNP | T | T | A | novel | Somatic | I41F | 0.165362 | NM_001317782.2 | 2171 | 1812 | 359 |
| 035-LNmet | TNC | 185 | hg19 | chr9 | 117848514 | 117848514 | Missense_Mutation | SNP | C | C | T | novel | Somatic | R499H | 0.221041 | NM_002160.4 | 937 | 729 | 208 |
| 035-LNmet | BRINP1 | 189 | hg19 | chr9 | 122075585 | 122075585 | Missense_Mutation | SNP | G | G | A | novel | Somatic | R17C | 0.190157 | NM_014618.3 | 447 | 362 | 85 |
| 035-LNmet | SEC16A | 1648 | hg19 | chr9 | 139357965 | 139357965 | Missense_Mutation | SNP | A | A | G | novel | Somatic | S1533P | 0.463636 | NM_014866.2 | 549 | 294 | 255 |
| 035-LNmet | NSMF | 1655 | hg19 | chr9 | 140350900 | 140350900 | Missense_Mutation | SNP | G | G | C | novel | Somatic | L223V | 0.042781 | NM_001130969.1 | 374 | 358 | 16 |
| 035-LNmet | PCDH15 | 126 | hg19 | chr10 | 56077202 | 56077202 | Splice_Site | SNP | C | C | T | novel | Somatic |  | 0.153992 | NM_001142763.2 | 526 | 445 | 81 |
| 035-LNmet | LRRTM3 | 138 | hg19 | chr10 | 68688050 | 68688050 | Missense_Mutation | SNP | G | G | A | novel | Somatic | R459Q | 0.193103 | NM_178011.5 | 580 | 468 | 112 |
| 035-LNmet | FAM149B1 | 144 | hg19 | chr10 | 74999109 | 74999109 | Missense_Mutation | SNP | C | C | A | novel | Somatic | P548T | 0.209713 | NM_173348.2 | 452 | 357 | 95 |
| 035-LNmet | KIF20B | 160 | hg19 | chr10 | 91479249 | 91479249 | Missense_Mutation | SNP | C | C | T | novel | Somatic | S503L | 0.197279 | NM_001284259.1 | 294 | 236 | 58 |
| 035-LNmet | GPAM | 1454 | hg19 | chr10 | 113923493 | 113923493 | Frame_Shift_Del | DEL | CCTCC | CCTCC | - | novel | Somatic | R462IfsTer7 | 0.245192 | NM_001244949.2 | 208 | 157 | 51 |
| 035-LNmet | VWA2 | 1470 | hg19 | chr10 | 116050118 | 116050118 | Missense_Mutation | SNP | G | G | A | novel | Somatic | E748K | 0.214425 | NM_001272046.2 | 511 | 401 | 110 |
| 035-LNmet | MKI67 | 1576 | hg19 | chr10 | 129921168 | 129921168 | Missense_Mutation | SNP | T | T | C | novel | Somatic | I88M | 0.210084 | NM_002417.5 | 238 | 188 | 50 |
| 035-LNmet | MUC5B | 594 | hg19 | chr11 | 1263229 | 1263229 | Missense_Mutation | SNP | G | G | C | novel | Somatic | G1707R | 0.156008 | NM_002458.3 | 1422 | 1200 | 222 |
| 035-LNmet | DNHD1 | 634 | hg19 | chr11 | 6566758 | 6566758 | Nonsense_Mutation | SNP | G | G | A | novel | Somatic | W1530Ter | 0.199179 | NM_144666.2 | 487 | 390 | 97 |
| 035-LNmet | OR10A4 | 637 | hg19 | chr11 | 6898039 | 6898039 | Missense_Mutation | SNP | C | C | T | novel | Somatic | S54F | 0.148438 | NM_207186.2 | 256 | 218 | 38 |
| 035-LNmet | ABCC8 | 89 | hg19 | chr11 | 17450183 | 17450183 | Nonsense_Mutation | SNP | C | C | A | novel | Somatic | E618Ter | 0.147368 | NM_000352.5 | 760 | 648 | 112 |
| 035-LNmet | ALX4 | 115 | hg19 | chr11 | 44297029 | 44297029 | Missense_Mutation | SNP | G | G | A | novel | Somatic | R216W | 0.125514 | NM_021926.4 | 483 | 422 | 61 |
| 035-LNmet | DAGLA | 131 | hg19 | chr11 | 61511150 | 61511150 | Missense_Mutation | SNP | G | G | A | novel | Somatic | R773Q | 0.171157 | NM_006133.3 | 1259 | 1043 | 216 |
| 035-LNmet | EHBP1L1 | 1083 | hg19 | chr11 | 65352691 | 65352691 | Missense_Mutation | SNP | G | G | A | novel | Somatic | E1188K | 0.327243 | NM_001099409.3 | 601 | 404 | 197 |
| 035-LNmet | CNTN5 | 20 | hg19 | chr11 | 99715873 | 99715873 | Missense_Mutation | SNP | G | G | C | novel | Somatic | L152F | 0.185941 | NM_014361.4 | 441 | 359 | 82 |
| 035-LNmet | ARHGAP20 | 178 | hg19 | chr11 | 110450249 | 110450249 | Missense_Mutation | SNP | C | C | G | novel | Somatic | E1141Q | 0.077895 | NM_020809.3 | 475 | 438 | 37 |
| 035-LNmet | FGF6 | 619 | hg19 | chr12 | 4554469 | 4554469 | Missense_Mutation | SNP | A | A | T | novel | Somatic | C90S | 0.182109 | NM_020996.2 | 1250 | 1022 | 228 |
| 035-LNmet | ANO2 | 78 | hg19 | chr12 | 5841702 | 5841702 | Missense_Mutation | SNP | G | G | A | novel | Somatic | T515M | 0.377863 | NM_001278596.3 | 523 | 325 | 198 |
| 035-LNmet | ATF7IP | 86 | hg19 | chr12 | 14577801 | 14577801 | Frame_Shift_Ins | INS | - | - | A | novel | Somatic | N328KfsTer4 | 0.181172 | NM_181352.2 | 561 | 459 | 102 |
| 035-LNmet | SLCO1B7 | 93 | hg19 | chr12 | 21172221 | 21172221 | Missense_Mutation | SNP | C | C | G | novel | Somatic | S42C | 0.071207 | NM_001009562.4 | 646 | 600 | 46 |
| 035-LNmet | RAPGEF3 | 952 | hg19 | chr12 | 48132036 | 48132036 | Splice_Region | SNP | G | G | C | novel | Somatic |  | 0.174321 | NM_001098531.3 | 1066 | 880 | 186 |
| 035-LNmet | OSBPL8 | 146 | hg19 | chr12 | 76752578 | 76752578 | Missense_Mutation | SNP | T | T | C | novel | Somatic | K781R | 0.26506 | NM_020841.5 | 414 | 304 | 110 |
| 035-LNmet | SETD1B | 1517 | hg19 | chr12 | 122247822 | 122247822 | Missense_Mutation | SNP | G | G | A | novel | Somatic | R324H | 0.241206 | NM_001353345.1 | 1587 | 1203 | 384 |
| 035-LNmet | SETD1B | 1517 | hg19 | chr12 | 122248399 | 122248399 | Frame_Shift_Del | DEL | C | C | - | novel | Somatic | L517Ter | 0.221466 | NM_001353345.1 | 1322 | 1029 | 293 |
| 035-LNmet | MTUS2 | 101 | hg19 | chr13 | 29855979 | 29855979 | Missense_Mutation | SNP | C | C | T | novel | Somatic | P928L | 0.027586 | NM_001033602.3 | 580 | 564 | 16 |
| 035-LNmet | ALG5 | 871 | hg19 | chr13 | 37539722 | 37539722 | Missense_Mutation | SNP | C | C | T | novel | Somatic | V255I | 0.20985 | NM_013338.5 | 466 | 368 | 98 |
| 035-LNmet | LOC101928841 | 1455 | hg19 | chr13 | 114061634 | 114061634 | Missense_Mutation | SNP | C | C | T | novel | Somatic | V291M | 0.227571 | NM_001304433.1 | 1369 | 1057 | 312 |
| 035-LNmet | TRIP11 | 1290 | hg19 | chr14 | 92471589 | 92471589 | Missense_Mutation | SNP | C | C | T | novel | Somatic | E911K | 0.226446 | NM_004239.4 | 605 | 468 | 137 |
| 035-LNmet | PLA2G4D | 908 | hg19 | chr15 | 42371887 | 42371887 | Missense_Mutation | SNP | G | G | A | novel | Somatic | R389W | 0.186312 | NM_178034.4 | 1049 | 853 | 196 |
| 035-LNmet | TGM7 | 917 | hg19 | chr15 | 43571969 | 43571969 | Missense_Mutation | SNP | C | C | T | novel | Somatic | R511H | 0.206818 | NM_052955.3 | 878 | 696 | 182 |
| 035-LNmet | DMXL2 | 122 | hg19 | chr15 | 51766642 | 51766642 | Missense_Mutation | SNP | C | C | T | novel | Somatic | R2371Q | 0.170316 | NM_001174116.2 | 407 | 337 | 70 |
| 035-LNmet | TBL3 | 600 | hg19 | chr16 | 2027571 | 2027571 | Missense_Mutation | SNP | C | C | T | novel | Somatic | T600M | 0.112369 | NM_006453.3 | 1144 | 1015 | 129 |
| 035-LNmet | GRIN2A | 82 | hg19 | chr16 | 9857236 | 9857236 | Missense_Mutation | SNP | G | G | A | novel | Somatic | H1389Y | 0.215017 | NM_001134407.3 | 293 | 230 | 63 |
| 035-LNmet | YWHAE | 594 | hg19 | chr17 | 1303356 | 1303356 | Missense_Mutation | SNP | C | C | A | novel | Somatic | A17S | 0.222447 | NM_006761.5 | 981 | 761 | 220 |
| 035-LNmet | PRPF8 | 74 | hg19 | chr17 | 1562699 | 1562699 | Missense_Mutation | SNP | G | G | A | novel | Somatic | S1697L | 0.189985 | NM_006445.4 | 679 | 550 | 129 |
| 035-LNmet | PRPF8 | 74 | hg19 | chr17 | 1584941 | 1584941 | In_Frame_Del | DEL | GTA | GTA | - | novel | Somatic | L232del | 0.166311 | NM_006445.4 | 468 | 390 | 78 |
| 035-LNmet | PELP1 | 77 | hg19 | chr17 | 4576196 | 4576196 | Missense_Mutation | SNP | G | G | A | novel | Somatic | S697L | 0.027532 | NM_014389.3 | 1015 | 987 | 28 |
| 035-LNmet | TP53 | 642 | hg19 | chr17 | 7577529 | 7577529 | Missense_Mutation | SNP | A | A | T | novel | Somatic | I251N | 0.242667 | NM_000546.5 | 375 | 284 | 91 |
| 035-LNmet | KRT34 | 886 | hg19 | chr17 | 39535628 | 39535628 | Nonsense_Mutation | SNP | C | C | A | novel | Somatic | E327Ter | 0.196915 | NM_021013.3 | 1099 | 882 | 217 |
| 035-LNmet | BRCA1 | 899 | hg19 | chr17 | 41209088 | 41209088 | Missense_Mutation | SNP | C | C | A | novel | Somatic | R1753I | 0.022013 | NM_007294.4 | 950 | 929 | 21 |
| 035-LNmet | LIPG | 944 | hg19 | chr18 | 47088689 | 47088689 | Missense_Mutation | SNP | C | C | G | novel | Somatic | S4C | 0.234586 | NM_006033.4 | 665 | 509 | 156 |
| 035-LNmet | SLX4IP | 10 | hg19 | chr20 | 10603872 | 10603872 | Missense_Mutation | SNP | G | G | C | novel | Somatic | E358Q | 0.202465 | NM_001009608.3 | 568 | 453 | 115 |
| 035-LNmet | RALGAPA2 | 92 | hg19 | chr20 | 20693099 | 20693099 | Missense_Mutation | SNP | C | C | T | novel | Somatic | D9N | 0.216129 | NM_020343.4 | 618 | 484 | 134 |
| 035-LNmet | PKNOX1 | 115 | hg19 | chr21 | 44430160 | 44430160 | Splice_Region | SNP | C | C | G | novel | Somatic |  | 0.161111 | NM_004571.5 | 180 | 151 | 29 |
| 035-LNmet | SLC25A18 | 722 | hg19 | chr22 | 18066255 | 18066255 | Missense_Mutation | SNP | C | C | T | novel | Somatic | P122S | 0.232203 | NM_031481.3 | 588 | 451 | 137 |
| 035-LNmet | SFI1 | 103 | hg19 | chr22 | 32007831 | 32007831 | Splice_Region | SNP | G | G | A | novel | Somatic |  | 0.313008 | NM_001007467.3 | 491 | 337 | 154 |
| 035-LNmet | WBP2NL | 908 | hg19 | chr22 | 42415364 | 42415364 | Missense_Mutation | SNP | G | G | A | novel | Somatic | E38K | 0.22521 | NM_152613.3 | 595 | 461 | 134 |
| 035-LNmet | PRICKLE3 | 959 | hg19 | chrX | 49032583 | 49032583 | Silent | SNP | C | C | A | novel | Somatic | L429= | 0.312411 | NM_006150.5 | 700 | 481 | 219 |
| 035-LNmet | USP51 | 1008 | hg19 | chrX | 55514601 | 55514601 | Missense_Mutation | SNP | A | A | C | novel | Somatic | F258V | 0.265306 | NM_201286.3 | 489 | 359 | 130 |
| 253-PTsup | EPHA2 | 710 | hg19 | chr1 | 16461585 | 16461585 | Frame_Shift_Del | DEL | GTGCC | GTGCC | - | novel | Somatic | Q508HfsTer86 | 0.236067 | NM_004431.5 | 1893 | 1444 | 449 |
| 253-PTsup | EPHA2 | 710 | hg19 | chr1 | 16474922 | 16474922 | Frame_Shift_Del | DEL | C | C | - | novel | Somatic | Q259SfsTer134 | 0.206761 | NM_004431.5 | 1267 | 1004 | 263 |
| 253-PTsup | TARS2 | 216 | hg19 | chr1 | 150463144 | 150463144 | Missense_Mutation | SNP | G | G | T | novel | Somatic | C152F | 0.360981 | NM_025150.5 | 856 | 547 | 309 |
| 253-PTsup | ZNF687 | 217 | hg19 | chr1 | 151263039 | 151263039 | Missense_Mutation | SNP | C | C | T | novel | Somatic | A1070V | 0.192456 | NM_020832.3 | 1297 | 1047 | 250 |
| 253-PTsup | BLZF1 | 234 | hg19 | chr1 | 169345974 | 169345974 | Missense_Mutation | SNP | A | A | C | novel | Somatic | E75D | 0.337679 | NM_001320973.2 | 767 | 508 | 259 |
| 253-PTsup | DNAH6 | 19 | hg19 | chr2 | 84932758 | 84932758 | Missense_Mutation | SNP | G | G | A | novel | Somatic | E2872K | 0.060698 | NM_001370.2 | 656 | 616 | 40 |
| 253-PTsup | GAD1 | 1894 | hg19 | chr2 | 171687555 | 171687555 | Missense_Mutation | SNP | C | C | T | novel | Somatic | R134C | 0.158983 | NM_000817.3 | 628 | 528 | 100 |
| 253-PTsup | ABCA12 | 278 | hg19 | chr2 | 215910578 | 215910578 | Frame_Shift_Del | DEL | AACATCA | AACATCA | - | novel | Somatic | D284FfsTer49 | 0.033058 | NM_173076.3 | 361 | 349 | 12 |
| 253-PTsup | FEZF2 | 1060 | hg19 | chr3 | 62355764 | 62355764 | Missense_Mutation | SNP | C | C | G | novel | Somatic | Q458H | 0.035256 | NM_018008.4 | 312 | 301 | 11 |
| 253-PTsup | TRMT10C | 1357 | hg19 | chr3 | 101284752 | 101284752 | Missense_Mutation | SNP | T | T | C | novel | Somatic | V376A | 0.053541 | NM_017819.4 | 579 | 548 | 31 |
| 253-PTsup | ZIC1 | 1707 | hg19 | chr3 | 147128231 | 147128231 | Missense_Mutation | SNP | C | C | T | novel | Somatic | A111V | 0.267065 | NM_003412.4 | 1168 | 855 | 313 |
| 253-PTsup | ENPEP | 179 | hg19 | chr4 | 111397851 | 111397851 | Missense_Mutation | SNP | C | C | T | novel | Somatic | P94L | 0.198813 | NM_001977.4 | 672 | 538 | 134 |
| 253-PTsup | SSBP2 | 18 | hg19 | chr5 | 80736410 | 80736410 | Missense_Mutation | SNP | C | C | T | novel | Somatic | G307R | 0.287805 | NM_001256732.2 | 410 | 292 | 118 |
| 253-PTsup | PAM | 170 | hg19 | chr5 | 102296898 | 102296898 | Missense_Mutation | SNP | A | A | T | novel | Somatic | Q376L | 0.343333 | NM_001319943.1 | 598 | 392 | 206 |
| 253-PTsup | FBN2 | 194 | hg19 | chr5 | 127727828 | 127727828 | Missense_Mutation | SNP | C | C | T | novel | Somatic | D496N | 0.040956 | NM_001999.4 | 292 | 280 | 12 |
| 253-PTsup | PDLIM4 | 198 | hg19 | chr5 | 131606611 | 131606611 | Missense_Mutation | SNP | G | G | A | novel | Somatic | G111S | 0.087329 | NM_003687.4 | 582 | 531 | 51 |
| 253-PTsup | FAM53C | 1635 | hg19 | chr5 | 137681152 | 137681152 | Missense_Mutation | SNP | C | C | G | novel | Somatic | P259A | 0.099359 | NM_016605.3 | 1558 | 1403 | 155 |
| 253-PTsup | RELL2 | 1660 | hg19 | chr5 | 141019656 | 141019656 | Missense_Mutation | SNP | G | G | C | novel | Somatic | E225Q | 0.037196 | NM_173828.5 | 699 | 673 | 26 |
| 253-PTsup | GABRA1 | 1815 | hg19 | chr5 | 161309581 | 161309581 | Nonsense_Mutation | SNP | G | G | T | novel | Somatic | E193Ter | 0.103327 | NM_000806.5 | 571 | 512 | 59 |
| 253-PTsup | LRFN2 | 111 | hg19 | chr6 | 40359798 | 40359798 | Missense_Mutation | SNP | A | A | G | novel | Somatic | W752R | 0.181191 | NM_020737.3 | 1158 | 948 | 210 |
| 253-PTsup | TINAG | 998 | hg19 | chr6 | 54191685 | 54191685 | Missense_Mutation | SNP | C | C | T | novel | Somatic | P199S | 0.348189 | NM_014464.4 | 359 | 234 | 125 |
| 253-PTsup | ZNF292 | 156 | hg19 | chr6 | 87970675 | 87970675 | Missense_Mutation | SNP | G | G | T | novel | Somatic | G2443V | 0.115566 | NM_015021.3 | 419 | 370 | 49 |
| 253-PTsup | RSPO3 | 1557 | hg19 | chr6 | 127476428 | 127476428 | Missense_Mutation | SNP | C | C | T | novel | Somatic | T160M | 0.037618 | NM_032784.5 | 638 | 614 | 24 |
| 253-PTsup | CCDC28A | 1646 | hg19 | chr6 | 139094993 | 139094993 | Missense_Mutation | SNP | G | G | A | novel | Somatic | G61E | 0.214497 | NM_015439.2 | 676 | 531 | 145 |
| 253-PTsup | ARID1B | 27 | hg19 | chr6 | 157528165 | 157528165 | Missense_Mutation | SNP | G | G | A | novel | Somatic | E1964K | 0.120061 | NM_020732.3 | 658 | 579 | 79 |
| 253-PTsup | FNDC1 | 225 | hg19 | chr6 | 159688874 | 159688874 | Missense_Mutation | SNP | G | G | C | novel | Somatic | E1829Q | 0.089457 | NM_032532.3 | 313 | 285 | 28 |
| 253-PTsup | THSD7A | 10 | hg19 | chr7 | 11509595 | 11509595 | Missense_Mutation | SNP | G | G | T | novel | Somatic | T760N | 0.118812 | NM_015204.3 | 505 | 445 | 60 |
| 253-PTsup | THSD7A | 10 | hg19 | chr7 | 11675814 | 11675814 | Missense_Mutation | SNP | C | C | T | novel | Somatic | G322E | 0.146703 | NM_015204.3 | 742 | 633 | 109 |
| 253-PTsup | CACNA2D1 | 18 | hg19 | chr7 | 81662173 | 81662173 | Missense_Mutation | SNP | G | G | T | novel | Somatic | F361L | 0.025301 | NM_000722.4 | 828 | 807 | 21 |
| 253-PTsup | ATP5J2-PTCD1 | 1340 | hg19 | chr7 | 99021421 | 99021421 | Nonsense_Mutation | SNP | G | G | A | novel | Somatic | Q682Ter | 0.034735 | NM_001198879.2 | 545 | 526 | 19 |
| 253-PTsup | NAT16 | 1354 | hg19 | chr7 | 100817811 | 100817811 | Missense_Mutation | SNP | C | C | T | novel | Somatic | R93H | 0.040314 | NM_198571.3 | 890 | 854 | 36 |
| 253-PTsup | KMT2C | 27 | hg19 | chr7 | 151874147 | 151874147 | Missense_Mutation | SNP | C | C | A | novel | Somatic | K2797N | 0.201044 | NM_170606.3 | 372 | 295 | 77 |
| 253-PTsup | CNGB3 | 156 | hg19 | chr8 | 87751937 | 87751937 | Missense_Mutation | SNP | G | G | T | novel | Somatic | L53I | 0.049573 | NM_019098.4 | 585 | 556 | 29 |
| 253-PTsup | SNTB1 | 23 | hg19 | chr8 | 121644725 | 121644725 | Missense_Mutation | SNP | C | C | T | novel | Somatic | G319R | 0.100105 | NM_021021.4 | 949 | 854 | 95 |
| 253-PTsup | TG | 200 | hg19 | chr8 | 133912495 | 133912495 | Missense_Mutation | SNP | C | C | T | novel | Somatic | A1115V | 0.117347 | NM_003235.5 | 783 | 691 | 92 |
| 253-PTsup | ALDOB | 1379 | hg19 | chr9 | 104188886 | 104188886 | Missense_Mutation | SNP | A | A | G | novel | Somatic | I192T | 0.025572 | NM_000035.4 | 743 | 724 | 19 |
| 253-PTsup | GRIN3A | 172 | hg19 | chr9 | 104500122 | 104500122 | Missense_Mutation | SNP | G | G | A | novel | Somatic | A47V | 0.020588 | NM_133445.3 | 1017 | 996 | 21 |
| 253-PTsup | DUPD1 | 146 | hg19 | chr10 | 76797644 | 76797644 | Nonsense_Mutation | SNP | G | G | A | novel | Somatic | R205Ter | 0.287143 | NM_001003892.2 | 1400 | 998 | 402 |
| 253-PTsup | DNTT | 1333 | hg19 | chr10 | 98084108 | 98084108 | Missense_Mutation | SNP | C | C | T | novel | Somatic | S279L | 0.086567 | NM_004088.4 | 334 | 305 | 29 |
| 253-PTsup | AFAP1L2 | 183 | hg19 | chr10 | 116061095 | 116061095 | Intron | SNP | G | G | A | novel | Somatic |  | 0.255034 | NM_001287824.1 | 298 | 222 | 76 |
| 253-PTsup | EBF3 | 198 | hg19 | chr10 | 131666068 | 131666068 | Missense_Mutation | SNP | C | C | T | novel | Somatic | G288E | 0.317269 | NM_001005463.3 | 497 | 339 | 158 |
| 253-PTsup | LDHA | 725 | hg19 | chr11 | 18422419 | 18422419 | Missense_Mutation | SNP | A | A | G | novel | Somatic | I94V | 0.443662 | NM_005566.4 | 568 | 316 | 252 |
| 253-PTsup | MS4A14 | 1044 | hg19 | chr11 | 60183285 | 60183285 | Missense_Mutation | SNP | G | G | C | novel | Somatic | A315P | 0.191589 | NM_001261828.2 | 428 | 346 | 82 |
| 253-PTsup | MS4A1 | 1044 | hg19 | chr11 | 60233497 | 60233497 | Nonsense_Mutation | SNP | T | T | A | novel | Somatic | L147Ter | 0.230563 | NM_152866.2 | 373 | 287 | 86 |
| 253-PTsup | INTS5 | 1061 | hg19 | chr11 | 62415643 | 62415643 | Missense_Mutation | SNP | C | C | T | novel | Somatic | E637K | 0.024768 | NM_030628.2 | 646 | 630 | 16 |
| 253-PTsup | FLRT1 | 1072 | hg19 | chr11 | 63883860 | 63883860 | Missense_Mutation | SNP | C | C | A | novel | Somatic | L41I | 0.188504 | NM_013280.4 | 1618 | 1313 | 305 |
| 253-PTsup | NRXN2 | 1076 | hg19 | chr11 | 64457918 | 64457918 | Frame_Shift_Ins | INS | - | - | C | novel | Somatic | A270GfsTer27 | 0.030498 | NM_015080.4 | 613 | 594 | 19 |
| 253-PTsup | MAP4K2 | 1077 | hg19 | chr11 | 64563781 | 64563781 | Missense_Mutation | SNP | A | A | G | novel | Somatic | I572T | 0.050408 | NM_004579.5 | 1348 | 1280 | 68 |
| 253-PTsup | SNX32 | 1085 | hg19 | chr11 | 65617722 | 65617722 | Missense_Mutation | SNP | G | G | C | novel | Somatic | K118N | 0.032078 | NM_152760.3 | 717 | 694 | 23 |
| 253-PTsup | TBRG1 | 1534 | hg19 | chr11 | 124502061 | 124502061 | Missense_Mutation | SNP | A | A | C | novel | Somatic | E388A | 0.123397 | NM_032811.3 | 622 | 545 | 77 |
| 253-PTsup | IGSF9B | 200 | hg19 | chr11 | 133790906 | 133790906 | Missense_Mutation | SNP | A | A | G | novel | Somatic | V905A | 0.099715 | NM_001277285.2 | 702 | 632 | 70 |
| 253-PTsup | A2M | 655 | hg19 | chr12 | 9242541 | 9242541 | Missense_Mutation | SNP | T | T | G | novel | Somatic | E892A | 0.048458 | NM_000014.5 | 452 | 430 | 22 |
| 253-PTsup | MARS | 1026 | hg19 | chr12 | 57883052 | 57883052 | Frame_Shift_Ins | INS | - | - | T | novel | Somatic | L71FfsTer9 | 0.027505 | NM_004990.4 | 507 | 493 | 14 |
| 253-PTsup | TUBA3C | 735 | hg19 | chr13 | 19751108 | 19751108 | Missense_Mutation | SNP | G | G | A | novel | Somatic | R339C | 0.234647 | NM_006001.3 | 1091 | 835 | 256 |
| 253-PTsup | SACS | 767 | hg19 | chr13 | 23912864 | 23912864 | Frame_Shift_Del | DEL | T | T | - | novel | Somatic | K1717NfsTer8 | 0.023981 | NM_014363.6 | 417 | 407 | 10 |
| 253-PTsup | ZIC5 | 1352 | hg19 | chr13 | 100622668 | 100622668 | In_Frame_Del | DEL | GGCGGCGGC | GGCGGCGGC | - | novel | Somatic | P422_P424del | 0.246914 | NM_033132.4 | 153 | 113 | 40 |
| 253-PTsup | CLEC14A | 880 | hg19 | chr14 | 38724870 | 38724870 | Missense_Mutation | SNP | G | G | A | novel | Somatic | P120S | 0.302174 | NM_175060.3 | 919 | 641 | 278 |
| 253-PTsup | ABHD12B | 976 | hg19 | chr14 | 51368568 | 51368568 | Missense_Mutation | SNP | T | T | G | novel | Somatic | F268V | 0.080756 | NM_001206673.2 | 582 | 535 | 47 |
| 253-PTsup | NID2 | 985 | hg19 | chr14 | 52478311 | 52478311 | Missense_Mutation | SNP | G | G | C | novel | Somatic | L1171V | 0.13355 | NM_007361.4 | 307 | 266 | 41 |
| 253-PTsup | KIAA0586 | 129 | hg19 | chr14 | 58943908 | 58943908 | Missense_Mutation | SNP | A | A | G | novel | Somatic | S1018G | 0.107632 | NM_001244189.2 | 510 | 455 | 55 |
| 253-PTsup | TBC1D2B | 1182 | hg19 | chr15 | 78369829 | 78369829 | Missense_Mutation | SNP | G | G | C | novel | Somatic | R56G | 0.193103 | NM_144572.1 | 725 | 585 | 140 |
| 253-PTsup | ADAMTS7 | 1188 | hg19 | chr15 | 79051885 | 79051885 | Missense_Mutation | SNP | C | C | T | novel | Somatic | E1647K | 0.194474 | NM_014272.5 | 941 | 758 | 183 |
| 253-PTsup | BNC1 | 1225 | hg19 | chr15 | 83933476 | 83933476 | Missense_Mutation | SNP | G | G | T | novel | Somatic | T176N | 0.265849 | NM_001717.4 | 489 | 359 | 130 |
| 253-PTsup | ACAN | 158 | hg19 | chr15 | 89392768 | 89392768 | Missense_Mutation | SNP | T | T | C | novel | Somatic | L611P | 0.18811 | NM_001369268.1 | 1126 | 914 | 212 |
| 253-PTsup | BAIAP3 | 595 | hg19 | chr16 | 1397954 | 1397954 | Missense_Mutation | SNP | C | C | T | novel | Somatic | R1064C | 0.191781 | NM_003933.4 | 949 | 767 | 182 |
| 253-PTsup | CLEC16A | 83 | hg19 | chr16 | 11118706 | 11118706 | Missense_Mutation | SNP | C | C | A | novel | Somatic | L489M | 0.070423 | NM_015226.3 | 495 | 460 | 35 |
| 253-PTsup | ABCC1 | 88 | hg19 | chr16 | 16235067 | 16235067 | Missense_Mutation | SNP | G | G | A | novel | Somatic | G1509S | 0.031568 | NM_004996.4 | 981 | 950 | 31 |
| 253-PTsup | DNAH3 | 11 | hg19 | chr16 | 21069470 | 21069470 | Missense_Mutation | SNP | G | G | C | novel | Somatic | I1287M | 0.043564 | NM_017539.2 | 505 | 483 | 22 |
| 253-PTsup | RPGRIP1L | 994 | hg19 | chr16 | 53686790 | 53686790 | Silent | SNP | G | G | A | novel | Somatic | G603= | 0.085308 | NM_015272.5 | 419 | 383 | 36 |
| 253-PTsup | CAPNS2 | 1009 | hg19 | chr16 | 55600682 | 55600682 | Missense_Mutation | SNP | A | A | G | novel | Somatic | K5R | 0.255429 | NM_032330.3 | 967 | 720 | 247 |
| 253-PTsup | ADAMTS18 | 146 | hg19 | chr16 | 77353786 | 77353786 | Missense_Mutation | SNP | A | A | G | novel | Somatic | L831P | 0.2 | NM_199355.4 | 850 | 680 | 170 |
| 253-PTsup | ADAMTS18 | 146 | hg19 | chr16 | 77356310 | 77356310 | Frame_Shift_Ins | INS | - | - | A | novel | Somatic | A696CfsTer18 | 0.021021 | NM_199355.4 | 665 | 651 | 14 |
| 253-PTsup | NLRP1 | 626 | hg19 | chr17 | 5461780 | 5461780 | Missense_Mutation | SNP | C | C | G | novel | Somatic | E746Q | 0.129237 | NM_033004.4 | 470 | 409 | 61 |
| 253-PTsup | TP53 | 642 | hg19 | chr17 | 7578239 | 7578239 | Nonsense_Mutation | SNP | C | C | A | novel | Somatic | E204Ter | 0.390704 | NM_000546.5 | 796 | 485 | 311 |
| 253-PTsup | COX10 | 86 | hg19 | chr17 | 13980228 | 13980228 | Missense_Mutation | SNP | G | G | C | novel | Somatic | L118F | 0.04008 | NM_001303.4 | 498 | 478 | 20 |
| 253-PTsup | MPRIP | 89 | hg19 | chr17 | 17069668 | 17069668 | Nonsense_Mutation | SNP | C | C | G | novel | Somatic | S1620Ter | 0.055331 | NM_001364716.2 | 741 | 700 | 41 |
| 253-PTsup | SUPT6H | 98 | hg19 | chr17 | 27010824 | 27010824 | Missense_Mutation | SNP | A | A | G | novel | Somatic | Y740C | 0.255118 | NM_003170.5 | 634 | 472 | 162 |
| 253-PTsup | ATP9B | 146 | hg19 | chr18 | 77089192 | 77089192 | Missense_Mutation | SNP | G | G | A | novel | Somatic | D609N | 0.239159 | NM_198531.5 | 761 | 579 | 182 |
| 253-PTsup | ARHGAP45 | 593 | hg19 | chr19 | 1074862 | 1074862 | Missense_Mutation | SNP | G | G | A | novel | Somatic | R406H | 0.229576 | NM_001258328.3 | 967 | 745 | 222 |
| 253-PTsup | PPP2R1A | 987 | hg19 | chr19 | 52716301 | 52716301 | Missense_Mutation | SNP | C | C | T | novel | Somatic | R249C | 0.165881 | NM_014225.6 | 1269 | 1058 | 211 |
| 253-PTsup | CHD6 | 111 | hg19 | chr20 | 40045404 | 40045404 | Missense_Mutation | SNP | T | T | C | novel | Somatic | I2104V | 0.296741 | NM_032221.5 | 583 | 410 | 173 |
| 253-PTsup | SLC2A10 | 116 | hg19 | chr20 | 45354567 | 45354567 | Missense_Mutation | SNP | G | G | A | novel | Somatic | A298T | 0.090431 | NM_030777.4 | 951 | 865 | 86 |
| 253-PTsup | SALL4 | 969 | hg19 | chr20 | 50406698 | 50406698 | Missense_Mutation | SNP | C | C | T | novel | Somatic | R775Q | 0.340357 | NM_020436.5 | 617 | 407 | 210 |
| 253-PTsup | TCFL5 | 1054 | hg19 | chr20 | 61488766 | 61488766 | Missense_Mutation | SNP | G | G | C | novel | Somatic | R407G | 0.322086 | NM_006602.4 | 325 | 220 | 105 |
| 253-PTsup | NCAM2 | 94 | hg19 | chr21 | 22881199 | 22881199 | Missense_Mutation | SNP | G | G | A | novel | Somatic | G702E | 0.270588 | NM_004540.5 | 424 | 309 | 115 |
| 253-PTsup | SYNJ1 | 105 | hg19 | chr21 | 34042683 | 34042683 | Splice_Site | SNP | C | C | T | novel | Somatic |  | 0.125 | NM_003895.3 | 376 | 329 | 47 |
| 253-PTsup | MICAL3 | 90 | hg19 | chr22 | 18300278 | 18300278 | Missense_Mutation | SNP | C | C | T | novel | Somatic | E1717K | 0.199057 | NM_015241.3 | 1060 | 849 | 211 |
| 253-PTsup | EIF4ENIF1 | 103 | hg19 | chr22 | 31859047 | 31859047 | Missense_Mutation | SNP | C | C | T | novel | Somatic | E220K | 0.040559 | NM_019843.4 | 715 | 686 | 29 |
| 253-PTsup | PRR14L | 103 | hg19 | chr22 | 32108914 | 32108914 | Nonsense_Mutation | INS | - | - | T | novel | Somatic | Y1637Ter | 0.492152 | NM_173566.3 | 882 | 443 | 439 |
| 253-PTsup | BAIAP2L2 | 878 | hg19 | chr22 | 38494156 | 38494156 | Missense_Mutation | SNP | G | G | A | novel | Somatic | R127C | 0.314013 | NM_025045.6 | 1570 | 1077 | 493 |
| 253-PTsup | EP300 | 112 | hg19 | chr22 | 41560079 | 41560079 | Nonsense_Mutation | SNP | G | G | T | novel | Somatic | G1251Ter | 0.556894 | NM_001429.4 | 747 | 331 | 416 |
| 253-PTsup | CDKL5 | 90 | hg19 | chrX | 18664129 | 18664129 | In_Frame_Del | DEL | GGT | GGT | - | novel | Somatic | G907del | 0.404624 | NM_003159.2 | 173 | 103 | 70 |
| 253-PTsup | CXCR3 | 1125 | hg19 | chrX | 70836684 | 70836684 | Missense_Mutation | SNP | G | G | A | novel | Somatic | T260M | 0.492154 | NM_001142797.2 | 698 | 353 | 345 |
| 253-PTsup | NHSL2 | 17 | hg19 | chrX | 71360037 | 71360037 | Missense_Mutation | SNP | G | G | T | novel | Somatic | R880M | 0.043011 | NM_001013627.2 | 277 | 265 | 12 |
| 253-PTsup | NAP1L2 | 1137 | hg19 | chrX | 72433829 | 72433829 | Missense_Mutation | SNP | T | T | G | novel | Somatic | E167A | 0.535714 | NM_021963.4 | 336 | 156 | 180 |
| 253-PTsup | SMIM10 | 1608 | hg19 | chrX | 134125142 | 134125142 | Missense_Mutation | SNP | C | C | T | novel | Somatic | S6F | 0.463415 | NM_001163438.2 | 164 | 88 | 76 |
| 253-PTsup | MCF2 | 205 | hg19 | chrX | 138668563 | 138668563 | Missense_Mutation | SNP | G | G | A | novel | Somatic | A945V | 0.136364 | NM_001171876.2 | 286 | 247 | 39 |
| 253-PTdeep | EPHA2 | 710 | hg19 | chr1 | 16461585 | 16461585 | Frame_Shift_Del | DEL | GTGCC | GTGCC | - | novel | Somatic | Q508HfsTer86 | 0.11664 | NM_004431.5 | 1864 | 1646 | 218 |
| 253-PTdeep | EPHA2 | 710 | hg19 | chr1 | 16474922 | 16474922 | Frame_Shift_Del | DEL | C | C | - | novel | Somatic | Q259SfsTer134 | 0.095971 | NM_004431.5 | 1363 | 1232 | 131 |
| 253-PTdeep | NECAP2 | 1 | hg19 | chr1 | 16774404 | 16774404 | Missense_Mutation | SNP | G | G | T | novel | Somatic | G78V | 0.201859 | NM_018090.5 | 753 | 601 | 152 |
| 253-PTdeep | TARS2 | 216 | hg19 | chr1 | 150463144 | 150463144 | Missense_Mutation | SNP | G | G | T | novel | Somatic | C152F | 0.139818 | NM_025150.5 | 656 | 564 | 92 |
| 253-PTdeep | ZNF687 | 217 | hg19 | chr1 | 151263039 | 151263039 | Missense_Mutation | SNP | C | C | T | novel | Somatic | A1070V | 0.096234 | NM_020832.3 | 1193 | 1078 | 115 |
| 253-PTdeep | BLZF1 | 234 | hg19 | chr1 | 169345974 | 169345974 | Missense_Mutation | SNP | A | A | C | novel | Somatic | E75D | 0.113636 | NM_001320973.2 | 659 | 584 | 75 |
| 253-PTdeep | NLRC4 | 832 | hg19 | chr2 | 32475851 | 32475851 | Missense_Mutation | SNP | G | G | A | novel | Somatic | T361M | 0.080442 | NM_001199138.2 | 633 | 582 | 51 |
| 253-PTdeep | DNAH6 | 19 | hg19 | chr2 | 84932758 | 84932758 | Missense_Mutation | SNP | G | G | A | novel | Somatic | E2872K | 0.051364 | NM_001370.2 | 623 | 591 | 32 |
| 253-PTdeep | RNF103 | 1247 | hg19 | chr2 | 86831015 | 86831015 | Frame_Shift_Del | DEL | T | T | - | novel | Somatic | K670SfsTer27 | 0.021858 | NM_005667.4 | 549 | 537 | 12 |
| 253-PTdeep | GAD1 | 1894 | hg19 | chr2 | 171687555 | 171687555 | Missense_Mutation | SNP | C | C | T | novel | Somatic | R134C | 0.050955 | NM_000817.3 | 628 | 596 | 32 |
| 253-PTdeep | FEZF2 | 1060 | hg19 | chr3 | 62355764 | 62355764 | Missense_Mutation | SNP | C | C | G | novel | Somatic | Q458H | 0.050343 | NM_018008.4 | 437 | 415 | 22 |
| 253-PTdeep | TRMT10C | 1357 | hg19 | chr3 | 101284752 | 101284752 | Missense_Mutation | SNP | T | T | C | novel | Somatic | V376A | 0.025424 | NM_017819.4 | 472 | 460 | 12 |
| 253-PTdeep | ZIC1 | 1707 | hg19 | chr3 | 147128231 | 147128231 | Missense_Mutation | SNP | C | C | T | novel | Somatic | A111V | 0.106651 | NM_003412.4 | 870 | 777 | 93 |
| 253-PTdeep | EPHB3 | 248 | hg19 | chr3 | 184295745 | 184295745 | Missense_Mutation | SNP | G | G | A | novel | Somatic | G567R | 0.020619 | NM_004443.4 | 774 | 758 | 16 |
| 253-PTdeep | CLNK | 83 | hg19 | chr4 | 10529731 | 10529731 | Intron | DEL | A | A | - | novel | Somatic |  | 0.024038 | NM_052964.4 | 414 | 404 | 10 |
| 253-PTdeep | GRSF1 | 141 | hg19 | chr4 | 71705387 | 71705387 | In_Frame_Del | DEL | AGC | AGC | - | novel | Somatic | L53del | 0.021552 | NM_002092.4 | 693 | 678 | 15 |
| 253-PTdeep | ENPEP | 179 | hg19 | chr4 | 111397851 | 111397851 | Missense_Mutation | SNP | C | C | T | novel | Somatic | P94L | 0.080529 | NM_001977.4 | 831 | 764 | 67 |
| 253-PTdeep | SSBP2 | 18 | hg19 | chr5 | 80736410 | 80736410 | Missense_Mutation | SNP | C | C | T | novel | Somatic | G307R | 0.136253 | NM_001256732.2 | 411 | 355 | 56 |
| 253-PTdeep | PAM | 170 | hg19 | chr5 | 102296898 | 102296898 | Missense_Mutation | SNP | A | A | T | novel | Somatic | Q376L | 0.105615 | NM_001319943.1 | 748 | 669 | 79 |
| 253-PTdeep | PDLIM4 | 198 | hg19 | chr5 | 131606611 | 131606611 | Missense_Mutation | SNP | G | G | A | novel | Somatic | G111S | 0.033774 | NM_003687.4 | 680 | 657 | 23 |
| 253-PTdeep | FAM53C | 1635 | hg19 | chr5 | 137681152 | 137681152 | Missense_Mutation | SNP | C | C | G | novel | Somatic | P259A | 0.04321 | NM_016605.3 | 1457 | 1394 | 63 |
| 253-PTdeep | RELL2 | 1660 | hg19 | chr5 | 141019656 | 141019656 | Missense_Mutation | SNP | G | G | C | novel | Somatic | E225Q | 0.044077 | NM_173828.5 | 726 | 694 | 32 |
| 253-PTdeep | GABRA1 | 1815 | hg19 | chr5 | 161309581 | 161309581 | Nonsense_Mutation | SNP | G | G | T | novel | Somatic | E193Ter | 0.041199 | NM_000806.5 | 533 | 511 | 22 |
| 253-PTdeep | NOL7 | 688 | hg19 | chr6 | 13620992 | 13620992 | Frame_Shift_Del | DEL | A | A | - | novel | Somatic | K238NfsTer16 | 0.02057 | NM_016167.5 | 632 | 619 | 13 |
| 253-PTdeep | RANBP9 | 10 | hg19 | chr6 | 13711709 | 13711709 | Missense_Mutation | SNP | G | G | T | novel | Somatic | P10Q | 0.036398 | NM_005493.3 | 512 | 493 | 19 |
| 253-PTdeep | LRFN2 | 111 | hg19 | chr6 | 40359798 | 40359798 | Missense_Mutation | SNP | A | A | G | novel | Somatic | W752R | 0.108028 | NM_020737.3 | 1008 | 899 | 109 |
| 253-PTdeep | TINAG | 998 | hg19 | chr6 | 54191685 | 54191685 | Missense_Mutation | SNP | C | C | T | novel | Somatic | P199S | 0.175926 | NM_014464.4 | 323 | 266 | 57 |
| 253-PTdeep | CD109 | 17 | hg19 | chr6 | 74519764 | 74519764 | Missense_Mutation | SNP | G | G | A | novel | Somatic | R1138H | 0.02037 | NM_133493.5 | 539 | 528 | 11 |
| 253-PTdeep | ZNF292 | 156 | hg19 | chr6 | 87970675 | 87970675 | Missense_Mutation | SNP | G | G | T | novel | Somatic | G2443V | 0.036117 | NM_015021.3 | 440 | 424 | 16 |
| 253-PTdeep | CCDC28A | 1646 | hg19 | chr6 | 139094993 | 139094993 | Missense_Mutation | SNP | G | G | A | novel | Somatic | G61E | 0.092997 | NM_015439.2 | 870 | 789 | 81 |
| 253-PTdeep | THSD7A | 10 | hg19 | chr7 | 11509595 | 11509595 | Missense_Mutation | SNP | G | G | T | novel | Somatic | T760N | 0.038462 | NM_015204.3 | 520 | 500 | 20 |
| 253-PTdeep | THSD7A | 10 | hg19 | chr7 | 11675814 | 11675814 | Missense_Mutation | SNP | C | C | T | novel | Somatic | G322E | 0.075188 | NM_015204.3 | 530 | 490 | 40 |
| 253-PTdeep | ZNF727 | 1069 | hg19 | chr7 | 63529331 | 63529331 | Missense_Mutation | DNP | CT | CT | AA | novel | Somatic | D22_S23delinsET | 0.025451 | NM_001159522.3 | 942 | 918 | 24 |
| 253-PTdeep | NAT16 | 1354 | hg19 | chr7 | 100817811 | 100817811 | Missense_Mutation | SNP | C | C | T | novel | Somatic | R93H | 0.051546 | NM_198571.3 | 775 | 735 | 40 |
| 253-PTdeep | C7orf49 | 1613 | hg19 | chr7 | 134851406 | 134851406 | In_Frame_Del | DEL | TCC | TCC | - | novel | Somatic | E145del | 0.020216 | NM_001363329.1 | 740 | 725 | 15 |
| 253-PTdeep | KMT2C | 27 | hg19 | chr7 | 151874147 | 151874147 | Missense_Mutation | SNP | C | C | A | novel | Somatic | K2797N | 0.054945 | NM_170606.3 | 357 | 337 | 20 |
| 253-PTdeep | CNGB3 | 156 | hg19 | chr8 | 87751937 | 87751937 | Missense_Mutation | SNP | G | G | T | novel | Somatic | L53I | 0.037356 | NM_019098.4 | 347 | 334 | 13 |
| 253-PTdeep | SNTB1 | 23 | hg19 | chr8 | 121644725 | 121644725 | Missense_Mutation | SNP | C | C | T | novel | Somatic | G319R | 0.073746 | NM_021021.4 | 678 | 628 | 50 |
| 253-PTdeep | TG | 200 | hg19 | chr8 | 133912495 | 133912495 | Missense_Mutation | SNP | C | C | T | novel | Somatic | A1115V | 0.065922 | NM_003235.5 | 895 | 836 | 59 |
| 253-PTdeep | CHAT | 121 | hg19 | chr10 | 50859947 | 50859947 | Missense_Mutation | SNP | A | A | T | novel | Somatic | D510V | 0.033435 | NM_020549.4 | 329 | 318 | 11 |
| 253-PTdeep | JMJD1C | 16 | hg19 | chr10 | 64952698 | 64952698 | Frame_Shift_Ins | INS | - | - | T | novel | Somatic | E2026RfsTer8 | 0.023605 | NM_032776.3 | 464 | 453 | 11 |
| 253-PTdeep | DUPD1 | 146 | hg19 | chr10 | 76797644 | 76797644 | Nonsense_Mutation | SNP | G | G | A | novel | Somatic | R205Ter | 0.144426 | NM_001003892.2 | 1184 | 1013 | 171 |
| 253-PTdeep | DNTT | 1333 | hg19 | chr10 | 98084108 | 98084108 | Missense_Mutation | SNP | C | C | T | novel | Somatic | S279L | 0.050388 | NM_004088.4 | 256 | 243 | 13 |
| 253-PTdeep | AFAP1L2 | 183 | hg19 | chr10 | 116061095 | 116061095 | Intron | SNP | G | G | A | novel | Somatic |  | 0.117647 | NM_001287824.1 | 408 | 360 | 48 |
| 253-PTdeep | EBF3 | 198 | hg19 | chr10 | 131666068 | 131666068 | Missense_Mutation | SNP | C | C | T | novel | Somatic | G288E | 0.149819 | NM_001005463.3 | 552 | 469 | 83 |
| 253-PTdeep | LDHA | 725 | hg19 | chr11 | 18422419 | 18422419 | Missense_Mutation | SNP | A | A | G | novel | Somatic | I94V | 0.114566 | NM_005566.4 | 611 | 541 | 70 |
| 253-PTdeep | MS4A1 | 1044 | hg19 | chr11 | 60233497 | 60233497 | Nonsense_Mutation | SNP | T | T | A | novel | Somatic | L147Ter | 0.098191 | NM_152866.2 | 387 | 349 | 38 |
| 253-PTdeep | FLRT1 | 1072 | hg19 | chr11 | 63883860 | 63883860 | Missense_Mutation | SNP | C | C | A | novel | Somatic | L41I | 0.106107 | NM_013280.4 | 1305 | 1166 | 139 |
| 253-PTdeep | MAP4K2 | 1077 | hg19 | chr11 | 64563781 | 64563781 | Missense_Mutation | SNP | A | A | G | novel | Somatic | I572T | 0.034549 | NM_004579.5 | 1041 | 1005 | 36 |
| 253-PTdeep | SNX32 | 1085 | hg19 | chr11 | 65617722 | 65617722 | Missense_Mutation | SNP | G | G | C | novel | Somatic | K118N | 0.021944 | NM_152760.3 | 637 | 623 | 14 |
| 253-PTdeep | TBRG1 | 1534 | hg19 | chr11 | 124502061 | 124502061 | Missense_Mutation | SNP | A | A | C | novel | Somatic | E388A | 0.033566 | NM_032811.3 | 715 | 691 | 24 |
| 253-PTdeep | IGSF9B | 200 | hg19 | chr11 | 133790906 | 133790906 | Missense_Mutation | SNP | A | A | G | novel | Somatic | V905A | 0.036517 | NM_001277285.2 | 711 | 685 | 26 |
| 253-PTdeep | DYRK4 | 620 | hg19 | chr12 | 4719362 | 4719362 | Frame_Shift_Del | DEL | A | A | - | novel | Somatic | R470DfsTer12 | 0.043887 | NM_001371301.1 | 319 | 305 | 14 |
| 253-PTdeep | TUBA3C | 735 | hg19 | chr13 | 19751108 | 19751108 | Missense_Mutation | SNP | G | G | A | novel | Somatic | R339C | 0.12381 | NM_006001.3 | 1365 | 1196 | 169 |
| 253-PTdeep | RNASEH2B | 122 | hg19 | chr13 | 51530587 | 51530587 | Frame_Shift_Del | DEL | A | A | - | novel | Somatic | I309LfsTer26 | 0.033333 | NM_024570.4 | 360 | 348 | 12 |
| 253-PTdeep | ZIC5 | 1352 | hg19 | chr13 | 100622668 | 100622668 | In_Frame_Del | DEL | GGCGGCGGC | GGCGGCGGC | - | novel | Somatic | P422_P424del | 0.170543 | NM_033132.4 | 127 | 105 | 22 |
| 253-PTdeep | CLEC14A | 880 | hg19 | chr14 | 38724870 | 38724870 | Missense_Mutation | SNP | G | G | A | novel | Somatic | P120S | 0.133487 | NM_175060.3 | 866 | 750 | 116 |
| 253-PTdeep | ABHD12B | 976 | hg19 | chr14 | 51368568 | 51368568 | Missense_Mutation | SNP | T | T | G | novel | Somatic | F268V | 0.071429 | NM_001206673.2 | 629 | 584 | 45 |
| 253-PTdeep | NID2 | 985 | hg19 | chr14 | 52478311 | 52478311 | Missense_Mutation | SNP | G | G | C | novel | Somatic | L1171V | 0.057229 | NM_007361.4 | 332 | 313 | 19 |
| 253-PTdeep | KIAA0586 | 129 | hg19 | chr14 | 58943908 | 58943908 | Missense_Mutation | SNP | A | A | G | novel | Somatic | S1018G | 0.044444 | NM_001244189.2 | 495 | 473 | 22 |
| 253-PTdeep | TBC1D2B | 1182 | hg19 | chr15 | 78369829 | 78369829 | Missense_Mutation | SNP | G | G | C | novel | Somatic | R56G | 0.080214 | NM_144572.1 | 561 | 516 | 45 |
| 253-PTdeep | ADAMTS7 | 1188 | hg19 | chr15 | 79051885 | 79051885 | Missense_Mutation | SNP | C | C | T | novel | Somatic | E1647K | 0.12016 | NM_014272.5 | 749 | 659 | 90 |
| 253-PTdeep | BNC1 | 1225 | hg19 | chr15 | 83933476 | 83933476 | Missense_Mutation | SNP | G | G | T | novel | Somatic | T176N | 0.098237 | NM_001717.4 | 397 | 358 | 39 |
| 253-PTdeep | ACAN | 158 | hg19 | chr15 | 89392768 | 89392768 | Missense_Mutation | SNP | T | T | C | novel | Somatic | L611P | 0.097196 | NM_001369268.1 | 1069 | 965 | 104 |
| 253-PTdeep | BAIAP3 | 595 | hg19 | chr16 | 1397954 | 1397954 | Missense_Mutation | SNP | C | C | T | novel | Somatic | R1064C | 0.083908 | NM_003933.4 | 870 | 797 | 73 |
| 253-PTdeep | CLEC16A | 83 | hg19 | chr16 | 11118706 | 11118706 | Missense_Mutation | SNP | C | C | A | novel | Somatic | L489M | 0.028384 | NM_015226.3 | 458 | 445 | 13 |
| 253-PTdeep | ABCC1 | 88 | hg19 | chr16 | 16235067 | 16235067 | Missense_Mutation | SNP | G | G | A | novel | Somatic | G1509S | 0.033725 | NM_004996.4 | 1273 | 1230 | 43 |
| 253-PTdeep | RPGRIP1L | 994 | hg19 | chr16 | 53686790 | 53686790 | Silent | SNP | G | G | A | novel | Somatic | G603= | 0.025641 | NM_015272.5 | 389 | 379 | 10 |
| 253-PTdeep | CAPNS2 | 1009 | hg19 | chr16 | 55600682 | 55600682 | Missense_Mutation | SNP | A | A | G | novel | Somatic | K5R | 0.129458 | NM_032330.3 | 756 | 658 | 98 |
| 253-PTdeep | ADAMTS18 | 146 | hg19 | chr16 | 77353786 | 77353786 | Missense_Mutation | SNP | A | A | G | novel | Somatic | L831P | 0.087922 | NM_199355.4 | 1126 | 1027 | 99 |
| 253-PTdeep | ADAMTS18 | 146 | hg19 | chr16 | 77356310 | 77356310 | Frame_Shift_Ins | INS | - | - | A | novel | Somatic | A696CfsTer18 | 0.02 | NM_199355.4 | 649 | 636 | 13 |
| 253-PTdeep | NLRP1 | 626 | hg19 | chr17 | 5461780 | 5461780 | Missense_Mutation | SNP | C | C | G | novel | Somatic | E746Q | 0.054795 | NM_033004.4 | 584 | 552 | 32 |
| 253-PTdeep | TP53 | 642 | hg19 | chr17 | 7578239 | 7578239 | Nonsense_Mutation | SNP | C | C | A | novel | Somatic | E204Ter | 0.179678 | NM_000546.5 | 807 | 662 | 145 |
| 253-PTdeep | COX10 | 86 | hg19 | chr17 | 13980228 | 13980228 | Missense_Mutation | SNP | G | G | C | novel | Somatic | L118F | 0.038462 | NM_001303.4 | 467 | 449 | 18 |
| 253-PTdeep | MPRIP | 89 | hg19 | chr17 | 17069668 | 17069668 | Nonsense_Mutation | SNP | C | C | G | novel | Somatic | S1620Ter | 0.032168 | NM_001364716.2 | 713 | 690 | 23 |
| 253-PTdeep | SUPT6H | 98 | hg19 | chr17 | 27010824 | 27010824 | Missense_Mutation | SNP | A | A | G | novel | Somatic | Y740C | 0.088512 | NM_003170.5 | 531 | 484 | 47 |
| 253-PTdeep | ATP9B | 146 | hg19 | chr18 | 77089192 | 77089192 | Missense_Mutation | SNP | G | G | A | novel | Somatic | D609N | 0.080253 | NM_198531.5 | 947 | 871 | 76 |
| 253-PTdeep | ARHGAP45 | 593 | hg19 | chr19 | 1074862 | 1074862 | Missense_Mutation | SNP | G | G | A | novel | Somatic | R406H | 0.111262 | NM_001258328.3 | 736 | 654 | 82 |
| 253-PTdeep | PPP2R1A | 987 | hg19 | chr19 | 52716301 | 52716301 | Missense_Mutation | SNP | C | C | T | novel | Somatic | R249C | 0.078879 | NM_014225.6 | 1533 | 1412 | 121 |
| 253-PTdeep | FERMT1 | 631 | hg19 | chr20 | 6091014 | 6091014 | Frame_Shift_Ins | INS | - | - | G | novel | Somatic | Q226PfsTer17 | 0.020619 | NM_017671.4 | 679 | 665 | 14 |
| 253-PTdeep | DEFB118 | 813 | hg19 | chr20 | 29960672 | 29960672 | Frame_Shift_Del | DEL | A | A | - | novel | Somatic | K26NfsTer19 | 0.02381 | NM_054112.3 | 462 | 451 | 11 |
| 253-PTdeep | CHD6 | 111 | hg19 | chr20 | 40045404 | 40045404 | Missense_Mutation | SNP | T | T | C | novel | Somatic | I2104V | 0.111614 | NM_032221.5 | 663 | 589 | 74 |
| 253-PTdeep | SLC2A10 | 116 | hg19 | chr20 | 45354567 | 45354567 | Missense_Mutation | SNP | G | G | A | novel | Somatic | A298T | 0.052905 | NM_030777.4 | 964 | 913 | 51 |
| 253-PTdeep | SALL4 | 969 | hg19 | chr20 | 50406698 | 50406698 | Missense_Mutation | SNP | C | C | T | novel | Somatic | R775Q | 0.110012 | NM_020436.5 | 809 | 720 | 89 |
| 253-PTdeep | TCFL5 | 1054 | hg19 | chr20 | 61488766 | 61488766 | Missense_Mutation | SNP | G | G | C | novel | Somatic | R407G | 0.153348 | NM_006602.4 | 462 | 391 | 71 |
| 253-PTdeep | NCAM2 | 94 | hg19 | chr21 | 22881199 | 22881199 | Missense_Mutation | SNP | G | G | A | novel | Somatic | G702E | 0.086614 | NM_004540.5 | 381 | 348 | 33 |
| 253-PTdeep | SYNJ1 | 105 | hg19 | chr21 | 34042683 | 34042683 | Splice_Site | SNP | C | C | T | novel | Somatic |  | 0.034392 | NM_003895.3 | 378 | 365 | 13 |
| 253-PTdeep | MICAL3 | 90 | hg19 | chr22 | 18300278 | 18300278 | Missense_Mutation | SNP | C | C | T | novel | Somatic | E1717K | 0.112546 | NM_015241.3 | 1084 | 962 | 122 |
| 253-PTdeep | EIF4ENIF1 | 103 | hg19 | chr22 | 31859047 | 31859047 | Missense_Mutation | SNP | C | C | T | novel | Somatic | E220K | 0.023372 | NM_019843.4 | 599 | 585 | 14 |
| 253-PTdeep | PRR14L | 103 | hg19 | chr22 | 32108914 | 32108914 | Nonsense_Mutation | INS | - | - | T | novel | Somatic | Y1637Ter | 0.263554 | NM_173566.3 | 656 | 481 | 175 |
| 253-PTdeep | BAIAP2L2 | 878 | hg19 | chr22 | 38494156 | 38494156 | Missense_Mutation | SNP | G | G | A | novel | Somatic | R127C | 0.136187 | NM_025045.6 | 1541 | 1331 | 210 |
| 253-PTdeep | EP300 | 112 | hg19 | chr22 | 41560079 | 41560079 | Nonsense_Mutation | SNP | G | G | T | novel | Somatic | G1251Ter | 0.272097 | NM_001429.4 | 575 | 418 | 157 |
| 253-PTdeep | CDKL5 | 90 | hg19 | chrX | 18664129 | 18664129 | In_Frame_Del | DEL | GGT | GGT | - | novel | Somatic | G907del | 0.142857 | NM_003159.2 | 154 | 132 | 22 |
| 253-PTdeep | CXCR3 | 1125 | hg19 | chrX | 70836684 | 70836684 | Missense_Mutation | SNP | G | G | A | novel | Somatic | T260M | 0.268836 | NM_001142797.2 | 584 | 427 | 157 |
| 253-PTdeep | NAP1L2 | 1137 | hg19 | chrX | 72433829 | 72433829 | Missense_Mutation | SNP | T | T | G | novel | Somatic | E167A | 0.207885 | NM_021963.4 | 279 | 221 | 58 |
| 253-PTdeep | SMIM10 | 1608 | hg19 | chrX | 134125142 | 134125142 | Missense_Mutation | SNP | C | C | T | novel | Somatic | S6F | 0.223077 | NM_001163438.2 | 130 | 101 | 29 |
| 253-PTdeep | MCF2 | 205 | hg19 | chrX | 138668563 | 138668563 | Missense_Mutation | SNP | G | G | A | novel | Somatic | A945V | 0.049383 | NM_001171876.2 | 243 | 231 | 12 |
| 253-LNmet | MIB2 | 596 | hg19 | chr1 | 1564067 | 1564067 | Frame_Shift_Del | DEL | G | G | - | novel | Somatic | D783TfsTer22 | 0.02371 | NM_080875.3 | 717 | 700 | 17 |
| 253-LNmet | EPHA2 | 710 | hg19 | chr1 | 16461585 | 16461585 | Frame_Shift_Del | DEL | GTGCC | GTGCC | - | novel | Somatic | Q508HfsTer86 | 0.300469 | NM_004431.5 | 1913 | 1337 | 576 |
| 253-LNmet | EPHA2 | 710 | hg19 | chr1 | 16474922 | 16474922 | Frame_Shift_Del | DEL | C | C | - | novel | Somatic | Q259SfsTer134 | 0.237958 | NM_004431.5 | 1389 | 1058 | 331 |
| 253-LNmet | TARS2 | 216 | hg19 | chr1 | 150463144 | 150463144 | Missense_Mutation | SNP | G | G | T | novel | Somatic | C152F | 0.410377 | NM_025150.5 | 847 | 499 | 348 |
| 253-LNmet | ZNF687 | 217 | hg19 | chr1 | 151263039 | 151263039 | Missense_Mutation | SNP | C | C | T | novel | Somatic | A1070V | 0.228143 | NM_020832.3 | 1200 | 926 | 274 |
| 253-LNmet | DENND4B | 1759 | hg19 | chr1 | 153906234 | 153906234 | Frame_Shift_Ins | INS | - | - | A | novel | Somatic | G1019WfsTer13 | 0.086705 | NM_014856.3 | 1207 | 1102 | 105 |
| 253-LNmet | BLZF1 | 234 | hg19 | chr1 | 169345974 | 169345974 | Missense_Mutation | SNP | A | A | C | novel | Somatic | E75D | 0.41979 | NM_001320973.2 | 667 | 387 | 280 |
| 253-LNmet | HHIPL2 | 2284 | hg19 | chr1 | 222716993 | 222716993 | Missense_Mutation | SNP | C | C | T | novel | Somatic | R287H | 0.100813 | NM_024746.4 | 615 | 553 | 62 |
| 253-LNmet | MAP10 | 2362 | hg19 | chr1 | 232943490 | 232943490 | Frame_Shift_Del | DEL | T | T | - | novel | Somatic | S909QfsTer46 | 0.021142 | NM_019090.2 | 473 | 463 | 10 |
| 253-LNmet | NLRC4 | 832 | hg19 | chr2 | 32475851 | 32475851 | Missense_Mutation | SNP | G | G | A | novel | Somatic | T361M | 0.213008 | NM_001199138.2 | 615 | 484 | 131 |
| 253-LNmet | DNAH6 | 19 | hg19 | chr2 | 84932758 | 84932758 | Missense_Mutation | SNP | G | G | A | novel | Somatic | E2872K | 0.105691 | NM_001370.2 | 614 | 549 | 65 |
| 253-LNmet | GAD1 | 1894 | hg19 | chr2 | 171687555 | 171687555 | Missense_Mutation | SNP | C | C | T | novel | Somatic | R134C | 0.226779 | NM_000817.3 | 829 | 641 | 188 |
| 253-LNmet | DYNC1I2 | 1901 | hg19 | chr2 | 172549338 | 172549338 | Frame_Shift_Ins | INS | - | - | A | novel | Somatic | R57KfsTer5 | 0.048193 | NM_001378.3 | 498 | 474 | 24 |
| 253-LNmet | RFTN2 | 262 | hg19 | chr2 | 198498642 | 198498642 | Missense_Mutation | SNP | C | C | G | novel | Somatic | G173A | 0.123552 | NM_144629.3 | 518 | 454 | 64 |
| 253-LNmet | CMTM7 | 832 | hg19 | chr3 | 32483362 | 32483362 | Missense_Mutation | SNP | C | C | T | novel | Somatic | R64W | 0.141553 | NM_138410.4 | 875 | 751 | 124 |
| 253-LNmet | TRMT10C | 1357 | hg19 | chr3 | 101284752 | 101284752 | Missense_Mutation | SNP | T | T | C | novel | Somatic | V376A | 0.055031 | NM_017819.4 | 636 | 601 | 35 |
| 253-LNmet | ZIC1 | 1707 | hg19 | chr3 | 147128231 | 147128231 | Missense_Mutation | SNP | C | C | T | novel | Somatic | A111V | 0.262782 | NM_003412.4 | 840 | 619 | 221 |
| 253-LNmet | ENPEP | 179 | hg19 | chr4 | 111397851 | 111397851 | Missense_Mutation | SNP | C | C | T | novel | Somatic | P94L | 0.245752 | NM_001977.4 | 765 | 577 | 188 |
| 253-LNmet | SREK1IP1 | 1073 | hg19 | chr5 | 64036969 | 64036969 | Missense_Mutation | SNP | T | T | C | novel | Somatic | I40M | 0.130316 | NM_173829.4 | 729 | 634 | 95 |
| 253-LNmet | SSBP2 | 18 | hg19 | chr5 | 80736410 | 80736410 | Missense_Mutation | SNP | C | C | T | novel | Somatic | G307R | 0.399361 | NM_001256732.2 | 313 | 188 | 125 |
| 253-LNmet | PAM | 170 | hg19 | chr5 | 102296898 | 102296898 | Missense_Mutation | SNP | A | A | T | novel | Somatic | Q376L | 0.409186 | NM_001319943.1 | 479 | 283 | 196 |
| 253-LNmet | PDLIM4 | 198 | hg19 | chr5 | 131606611 | 131606611 | Missense_Mutation | SNP | G | G | A | novel | Somatic | G111S | 0.138425 | NM_003687.4 | 837 | 721 | 116 |
| 253-LNmet | FAM53C | 1635 | hg19 | chr5 | 137681152 | 137681152 | Missense_Mutation | SNP | C | C | G | novel | Somatic | P259A | 0.119756 | NM_016605.3 | 1475 | 1298 | 177 |
| 253-LNmet | GABRA1 | 1815 | hg19 | chr5 | 161309581 | 161309581 | Nonsense_Mutation | SNP | G | G | T | novel | Somatic | E193Ter | 0.130612 | NM_000806.5 | 490 | 426 | 64 |
| 253-LNmet | LRFN2 | 111 | hg19 | chr6 | 40359798 | 40359798 | Missense_Mutation | SNP | A | A | G | novel | Somatic | W752R | 0.244526 | NM_020737.3 | 1096 | 828 | 268 |
| 253-LNmet | TINAG | 998 | hg19 | chr6 | 54191685 | 54191685 | Missense_Mutation | SNP | C | C | T | novel | Somatic | P199S | 0.361011 | NM_014464.4 | 277 | 177 | 100 |
| 253-LNmet | ZNF292 | 156 | hg19 | chr6 | 87970675 | 87970675 | Missense_Mutation | SNP | G | G | T | novel | Somatic | G2443V | 0.135065 | NM_015021.3 | 385 | 333 | 52 |
| 253-LNmet | WISP3 | 1442 | hg19 | chr6 | 112389433 | 112389433 | Frame_Shift_Ins | INS | - | - | A | novel | Somatic | C209MfsTer21 | 0.048128 | NM_198239.2 | 371 | 353 | 18 |
| 253-LNmet | CCDC28A | 1646 | hg19 | chr6 | 139094993 | 139094993 | Missense_Mutation | SNP | G | G | A | novel | Somatic | G61E | 0.27216 | NM_015439.2 | 800 | 582 | 218 |
| 253-LNmet | ARID1B | 27 | hg19 | chr6 | 157528165 | 157528165 | Missense_Mutation | SNP | G | G | A | novel | Somatic | E1964K | 0.148148 | NM_020732.3 | 783 | 667 | 116 |
| 253-LNmet | FNDC1 | 225 | hg19 | chr6 | 159688874 | 159688874 | Missense_Mutation | SNP | G | G | C | novel | Somatic | E1829Q | 0.096654 | NM_032532.3 | 269 | 243 | 26 |
| 253-LNmet | GRID2IP | 79 | hg19 | chr7 | 6550551 | 6550551 | Missense_Mutation | SNP | C | C | A | novel | Somatic | G515C | 0.092063 | NM_001145118.1 | 630 | 572 | 58 |
| 253-LNmet | THSD7A | 10 | hg19 | chr7 | 11509595 | 11509595 | Missense_Mutation | SNP | G | G | T | novel | Somatic | T760N | 0.165236 | NM_015204.3 | 466 | 389 | 77 |
| 253-LNmet | THSD7A | 10 | hg19 | chr7 | 11675814 | 11675814 | Missense_Mutation | SNP | C | C | T | novel | Somatic | G322E | 0.228417 | NM_015204.3 | 554 | 427 | 127 |
| 253-LNmet | KMT2C | 27 | hg19 | chr7 | 151874147 | 151874147 | Missense_Mutation | SNP | C | C | A | novel | Somatic | K2797N | 0.187364 | NM_170606.3 | 449 | 363 | 86 |
| 253-LNmet | CNGB3 | 156 | hg19 | chr8 | 87751937 | 87751937 | Missense_Mutation | SNP | G | G | T | novel | Somatic | L53I | 0.042254 | NM_019098.4 | 497 | 476 | 21 |
| 253-LNmet | SNTB1 | 23 | hg19 | chr8 | 121644725 | 121644725 | Missense_Mutation | SNP | C | C | T | novel | Somatic | G319R | 0.130707 | NM_021021.4 | 1117 | 971 | 146 |
| 253-LNmet | TG | 200 | hg19 | chr8 | 133912495 | 133912495 | Missense_Mutation | SNP | C | C | T | novel | Somatic | A1115V | 0.155844 | NM_003235.5 | 1077 | 909 | 168 |
| 253-LNmet | NFX1 | 104 | hg19 | chr9 | 33303207 | 33303207 | Missense_Mutation | SNP | G | G | T | novel | Somatic | R404M | 0.08169 | NM_001318758.2 | 355 | 326 | 29 |
| 253-LNmet | ORM2 | 1478 | hg19 | chr9 | 117094204 | 117094204 | Frame_Shift_Del | DEL | A | A | - | novel | Somatic | K180RfsTer? | 0.024691 | NM_000608.2 | 486 | 474 | 12 |
| 253-LNmet | DUPD1 | 146 | hg19 | chr10 | 76797644 | 76797644 | Nonsense_Mutation | SNP | G | G | A | novel | Somatic | R205Ter | 0.368333 | NM_001003892.2 | 1198 | 756 | 442 |
| 253-LNmet | DNTT | 1333 | hg19 | chr10 | 98084108 | 98084108 | Missense_Mutation | SNP | C | C | T | novel | Somatic | S279L | 0.151515 | NM_004088.4 | 264 | 224 | 40 |
| 253-LNmet | AFAP1L2 | 183 | hg19 | chr10 | 116061095 | 116061095 | Intron | SNP | G | G | A | novel | Somatic |  | 0.382022 | NM_001287824.1 | 356 | 220 | 136 |
| 253-LNmet | EBF3 | 198 | hg19 | chr10 | 131666068 | 131666068 | Missense_Mutation | SNP | C | C | T | novel | Somatic | G288E | 0.384354 | NM_001005463.3 | 587 | 361 | 226 |
| 253-LNmet | LDHA | 725 | hg19 | chr11 | 18422419 | 18422419 | Missense_Mutation | SNP | A | A | G | novel | Somatic | I94V | 0.544218 | NM_005566.4 | 588 | 268 | 320 |
| 253-LNmet | MS4A1 | 1044 | hg19 | chr11 | 60233497 | 60233497 | Nonsense_Mutation | SNP | T | T | A | novel | Somatic | L147Ter | 0.267717 | NM_152866.2 | 254 | 186 | 68 |
| 253-LNmet | FLRT1 | 1072 | hg19 | chr11 | 63883860 | 63883860 | Missense_Mutation | SNP | C | C | A | novel | Somatic | L41I | 0.199826 | NM_013280.4 | 1150 | 920 | 230 |
| 253-LNmet | MAP4K2 | 1077 | hg19 | chr11 | 64563781 | 64563781 | Missense_Mutation | SNP | A | A | G | novel | Somatic | I572T | 0.060751 | NM_004579.5 | 1251 | 1175 | 76 |
| 253-LNmet | TBRG1 | 1534 | hg19 | chr11 | 124502061 | 124502061 | Missense_Mutation | SNP | A | A | C | novel | Somatic | E388A | 0.190391 | NM_032811.3 | 562 | 455 | 107 |
| 253-LNmet | MARS | 1026 | hg19 | chr12 | 57883052 | 57883052 | Frame_Shift_Ins | INS | - | - | T | novel | Somatic | L71FfsTer9 | 0.024712 | NM_004990.4 | 605 | 590 | 15 |
| 253-LNmet | TUBA3C | 735 | hg19 | chr13 | 19751108 | 19751108 | Missense_Mutation | SNP | G | G | A | novel | Somatic | R339C | 0.209581 | NM_006001.3 | 1168 | 923 | 245 |
| 253-LNmet | ZIC5 | 1352 | hg19 | chr13 | 100622668 | 100622668 | In_Frame_Del | DEL | GGCGGCGGC | GGCGGCGGC | - | novel | Somatic | P422_P424del | 0.272059 | NM_033132.4 | 132 | 95 | 37 |
| 253-LNmet | CLEC14A | 880 | hg19 | chr14 | 38724870 | 38724870 | Missense_Mutation | SNP | G | G | A | novel | Somatic | P120S | 0.334278 | NM_175060.3 | 706 | 470 | 236 |
| 253-LNmet | ABHD12B | 976 | hg19 | chr14 | 51368568 | 51368568 | Missense_Mutation | SNP | T | T | G | novel | Somatic | F268V | 0.175926 | NM_001206673.2 | 432 | 356 | 76 |
| 253-LNmet | NID2 | 985 | hg19 | chr14 | 52478311 | 52478311 | Missense_Mutation | SNP | G | G | C | novel | Somatic | L1171V | 0.140562 | NM_007361.4 | 498 | 428 | 70 |
| 253-LNmet | KIAA0586 | 129 | hg19 | chr14 | 58943908 | 58943908 | Missense_Mutation | SNP | A | A | G | novel | Somatic | S1018G | 0.119898 | NM_001244189.2 | 392 | 345 | 47 |
| 253-LNmet | TBC1D2B | 1182 | hg19 | chr15 | 78369829 | 78369829 | Missense_Mutation | SNP | G | G | C | novel | Somatic | R56G | 0.177858 | NM_144572.1 | 551 | 453 | 98 |
| 253-LNmet | ADAMTS7 | 1188 | hg19 | chr15 | 79051885 | 79051885 | Missense_Mutation | SNP | C | C | T | novel | Somatic | E1647K | 0.347578 | NM_014272.5 | 702 | 458 | 244 |
| 253-LNmet | BNC1 | 1225 | hg19 | chr15 | 83933476 | 83933476 | Missense_Mutation | SNP | G | G | T | novel | Somatic | T176N | 0.315493 | NM_001717.4 | 355 | 243 | 112 |
| 253-LNmet | ACAN | 158 | hg19 | chr15 | 89392768 | 89392768 | Missense_Mutation | SNP | T | T | C | novel | Somatic | L611P | 0.262799 | NM_001369268.1 | 877 | 646 | 231 |
| 253-LNmet | BAIAP3 | 595 | hg19 | chr16 | 1397954 | 1397954 | Missense_Mutation | SNP | C | C | T | novel | Somatic | R1064C | 0.148233 | NM_003933.4 | 877 | 747 | 130 |
| 253-LNmet | CLEC16A | 83 | hg19 | chr16 | 11118706 | 11118706 | Missense_Mutation | SNP | C | C | A | novel | Somatic | L489M | 0.091884 | NM_015226.3 | 653 | 593 | 60 |
| 253-LNmet | RPGRIP1L | 994 | hg19 | chr16 | 53686790 | 53686790 | Silent | SNP | G | G | A | novel | Somatic | G603= | 0.131868 | NM_015272.5 | 364 | 316 | 48 |
| 253-LNmet | CAPNS2 | 1009 | hg19 | chr16 | 55600682 | 55600682 | Missense_Mutation | SNP | A | A | G | novel | Somatic | K5R | 0.386029 | NM_032330.3 | 815 | 500 | 315 |
| 253-LNmet | ADAMTS18 | 146 | hg19 | chr16 | 77353786 | 77353786 | Missense_Mutation | SNP | A | A | G | novel | Somatic | L831P | 0.253452 | NM_199355.4 | 1231 | 919 | 312 |
| 253-LNmet | NLRP1 | 626 | hg19 | chr17 | 5461780 | 5461780 | Missense_Mutation | SNP | C | C | G | novel | Somatic | E746Q | 0.121951 | NM_033004.4 | 533 | 468 | 65 |
| 253-LNmet | TP53 | 642 | hg19 | chr17 | 7578239 | 7578239 | Nonsense_Mutation | SNP | C | C | A | novel | Somatic | E204Ter | 0.535963 | NM_000546.5 | 859 | 397 | 462 |
| 253-LNmet | SUPT6H | 98 | hg19 | chr17 | 27010824 | 27010824 | Missense_Mutation | SNP | A | A | G | novel | Somatic | Y740C | 0.277689 | NM_003170.5 | 623 | 450 | 173 |
| 253-LNmet | ATP9B | 146 | hg19 | chr18 | 77089192 | 77089192 | Missense_Mutation | SNP | G | G | A | novel | Somatic | D609N | 0.361463 | NM_198531.5 | 708 | 451 | 257 |
| 253-LNmet | ARHGAP45 | 593 | hg19 | chr19 | 1074862 | 1074862 | Missense_Mutation | SNP | G | G | A | novel | Somatic | R406H | 0.22542 | NM_001258328.3 | 834 | 646 | 188 |
| 253-LNmet | PPP2R1A | 987 | hg19 | chr19 | 52716301 | 52716301 | Missense_Mutation | SNP | C | C | T | novel | Somatic | R249C | 0.237988 | NM_014225.6 | 1332 | 1015 | 317 |
| 253-LNmet | CHD6 | 111 | hg19 | chr20 | 40045404 | 40045404 | Missense_Mutation | SNP | T | T | C | novel | Somatic | I2104V | 0.378855 | NM_032221.5 | 681 | 423 | 258 |
| 253-LNmet | SLC2A10 | 116 | hg19 | chr20 | 45354567 | 45354567 | Missense_Mutation | SNP | G | G | A | novel | Somatic | A298T | 0.129549 | NM_030777.4 | 687 | 598 | 89 |
| 253-LNmet | SALL4 | 969 | hg19 | chr20 | 50406698 | 50406698 | Missense_Mutation | SNP | C | C | T | novel | Somatic | R775Q | 0.36643 | NM_020436.5 | 845 | 535 | 310 |
| 253-LNmet | TCFL5 | 1054 | hg19 | chr20 | 61488766 | 61488766 | Missense_Mutation | SNP | G | G | C | novel | Somatic | R407G | 0.396078 | NM_006602.4 | 509 | 307 | 202 |
| 253-LNmet | NCAM2 | 94 | hg19 | chr21 | 22881199 | 22881199 | Missense_Mutation | SNP | G | G | A | novel | Somatic | G702E | 0.311897 | NM_004540.5 | 311 | 214 | 97 |
| 253-LNmet | SYNJ1 | 105 | hg19 | chr21 | 34042683 | 34042683 | Splice_Site | SNP | C | C | T | novel | Somatic |  | 0.133333 | NM_003895.3 | 269 | 233 | 36 |
| 253-LNmet | MICAL3 | 90 | hg19 | chr22 | 18300278 | 18300278 | Missense_Mutation | SNP | C | C | T | novel | Somatic | E1717K | 0.257418 | NM_015241.3 | 1246 | 925 | 321 |
| 253-LNmet | PRR14L | 103 | hg19 | chr22 | 32108914 | 32108914 | Nonsense_Mutation | INS | - | - | T | novel | Somatic | Y1637Ter | 0.580247 | NM_173566.3 | 789 | 319 | 470 |
| 253-LNmet | BAIAP2L2 | 878 | hg19 | chr22 | 38494156 | 38494156 | Missense_Mutation | SNP | G | G | A | novel | Somatic | R127C | 0.357641 | NM_025045.6 | 1573 | 1009 | 564 |
| 253-LNmet | EP300 | 112 | hg19 | chr22 | 41560079 | 41560079 | Nonsense_Mutation | SNP | G | G | T | novel | Somatic | G1251Ter | 0.610143 | NM_001429.4 | 629 | 244 | 385 |
| 253-LNmet | CDKL5 | 90 | hg19 | chrX | 18664129 | 18664129 | In_Frame_Del | DEL | GGT | GGT | - | novel | Somatic | G907del | 0.43609 | NM_003159.2 | 133 | 75 | 58 |
| 253-LNmet | CXCR3 | 1125 | hg19 | chrX | 70836684 | 70836684 | Missense_Mutation | SNP | G | G | A | novel | Somatic | T260M | 0.705293 | NM_001142797.2 | 699 | 206 | 493 |
| 253-LNmet | NAP1L2 | 1137 | hg19 | chrX | 72433829 | 72433829 | Missense_Mutation | SNP | T | T | G | novel | Somatic | E167A | 0.652038 | NM_021963.4 | 319 | 111 | 208 |
| 253-LNmet | SMIM10 | 1608 | hg19 | chrX | 134125142 | 134125142 | Missense_Mutation | SNP | C | C | T | novel | Somatic | S6F | 0.710526 | NM_001163438.2 | 114 | 33 | 81 |
| 253-LNmet | MCF2 | 205 | hg19 | chrX | 138668563 | 138668563 | Missense_Mutation | SNP | G | G | A | novel | Somatic | A945V | 0.296578 | NM_001171876.2 | 263 | 185 | 78 |
| 316-PTsup | ZNF436 | 765 | hg19 | chr1 | 23689220 | 23689220 | Missense_Mutation | SNP | C | C | T | novel | Somatic | E219K | 0.71116 | NM_001077195.2 | 455 | 130 | 325 |
| 316-PTsup | BCAR3 | 1302 | hg19 | chr1 | 94054822 | 94054822 | Missense_Mutation | SNP | C | C | T | novel | Somatic | R214H | 0.750221 | NM_001261408.2 | 1127 | 280 | 847 |
| 316-PTsup | WDR77 | 1439 | hg19 | chr1 | 111986004 | 111986004 | Missense_Mutation | SNP | G | G | T | novel | Somatic | P211H | 0.6 | NM_024102.4 | 477 | 189 | 288 |
| 316-PTsup | MCL1 | 1733 | hg19 | chr1 | 150551492 | 150551492 | In_Frame_Del | DEL | TCC | TCC | - | novel | Somatic | E171del | 0.030255 | NM_021960.5 | 624 | 605 | 19 |
| 316-PTsup | USF1 | 1813 | hg19 | chr1 | 161010052 | 161010052 | Missense_Mutation | SNP | C | C | A | novel | Somatic | L261F | 0.311258 | NM_007122.5 | 453 | 312 | 141 |
| 316-PTsup | BPNT1 | 2265 | hg19 | chr1 | 220253178 | 220253178 | Missense_Mutation | SNP | C | C | T | novel | Somatic | S4N | 0.206235 | NM_006085.6 | 416 | 330 | 86 |
| 316-PTsup | TMEM214 | 12 | hg19 | chr2 | 27258545 | 27258545 | Frame_Shift_Del | DEL | T | T | - | novel | Somatic | F197LfsTer42 | 0.030303 | NM_017727.5 | 396 | 384 | 12 |
| 316-PTsup | ANKRD36 | 166 | hg19 | chr2 | 97790292 | 97790292 | Missense_Mutation | SNP | G | G | A | novel | Somatic | R230Q | 0.067538 | NM_001354587.1 | 459 | 428 | 31 |
| 316-PTsup | TTN | 1955 | hg19 | chr2 | 179440435 | 179440435 | Missense_Mutation | SNP | C | C | T | novel | Somatic | R23475H | 0.28125 | NM_001267550.2 | 319 | 229 | 90 |
| 316-PTsup | MAP2 | 273 | hg19 | chr2 | 210557839 | 210557839 | Missense_Mutation | SNP | T | T | A | novel | Somatic | S315R | 0.217277 | NM_002374.3 | 382 | 299 | 83 |
| 316-PTsup | GRM7 | 9 | hg19 | chr3 | 7721913 | 7721913 | Missense_Mutation | SNP | T | T | A | novel | Somatic | S877T | 0.4 | NM_000844.4 | 169 | 101 | 68 |
| 316-PTsup | GUCA1C | 176 | hg19 | chr3 | 108626895 | 108626895 | Missense_Mutation | SNP | C | C | G | novel | Somatic | G202R | 0.315287 | NM_005459.4 | 314 | 215 | 99 |
| 316-PTsup | MYLK | 190 | hg19 | chr3 | 123383039 | 123383039 | Missense_Mutation | SNP | C | C | T | novel | Somatic | A1300T | 0.344304 | NM_053025.4 | 788 | 516 | 272 |
| 316-PTsup | VEPH1 | 1784 | hg19 | chr3 | 157081227 | 157081227 | Frame_Shift_Del | DEL | T | T | - | novel | Somatic | N554TfsTer5 | 0.032316 | NM_024621.2 | 554 | 536 | 18 |
| 316-PTsup | SCHIP1 | 1802 | hg19 | chr3 | 159482490 | 159482490 | Missense_Mutation | SNP | G | G | C | novel | Somatic | D108H | 0.190672 | NM_014575.3 | 1458 | 1180 | 278 |
| 316-PTsup | EIF4G1 | 1989 | hg19 | chr3 | 184043731 | 184043731 | Missense_Mutation | SNP | A | A | G | novel | Somatic | I1079V | 0.288991 | NM_001194947.1 | 436 | 310 | 126 |
| 316-PTsup | ATP13A5 | 257 | hg19 | chr3 | 193081961 | 193081961 | Missense_Mutation | SNP | T | T | C | novel | Somatic | R58G | 0.329949 | NM_198505.4 | 590 | 395 | 195 |
| 316-PTsup | PPARGC1A | 767 | hg19 | chr4 | 23831091 | 23831091 | Missense_Mutation | SNP | C | C | G | novel | Somatic | V183L | 0.650602 | NM_013261.5 | 249 | 87 | 162 |
| 316-PTsup | EPHA5 | 136 | hg19 | chr4 | 66230863 | 66230863 | Missense_Mutation | SNP | G | G | T | novel | Somatic | P704H | 0.093103 | NM_001281765.2 | 290 | 263 | 27 |
| 316-PTsup | HHIP | 211 | hg19 | chr4 | 145567851 | 145567851 | In_Frame_Del | DEL | GCT | GCT | - | novel | Somatic | L12del | 0.024176 | NM_022475.3 | 454 | 443 | 11 |
| 316-PTsup | DNAH5 | 86 | hg19 | chr5 | 13793771 | 13793771 | Missense_Mutation | SNP | C | C | G | novel | Somatic | E2693Q | 0.475921 | NM_001369.2 | 353 | 185 | 168 |
| 316-PTsup | AGXT2 | 852 | hg19 | chr5 | 35037139 | 35037139 | Missense_Mutation | SNP | C | C | T | novel | Somatic | G132S | 0.552688 | NM_031900.4 | 460 | 203 | 257 |
| 316-PTsup | DPYSL3 | 26 | hg19 | chr5 | 146780330 | 146780330 | Missense_Mutation | SNP | C | C | A | novel | Somatic | K459N | 0.17154 | NM_001197294.2 | 512 | 424 | 88 |
| 316-PTsup | ADAMTS2 | 243 | hg19 | chr5 | 178772259 | 178772259 | In_Frame_Ins | INS | - | - | GCAGCA | novel | Somatic | L22_L23dup | 0.116071 | NM_014244.5 | 83 | 57 | 26 |
| 316-PTsup | HIST1H1C | 783 | hg19 | chr6 | 26056530 | 26056530 | Missense_Mutation | SNP | G | G | C | novel | Somatic | L43V | 0.209375 | NM_005319.3 | 318 | 251 | 67 |
| 316-PTsup | TBCC | 910 | hg19 | chr6 | 42713485 | 42713485 | Missense_Mutation | SNP | G | G | T | novel | Somatic | D109E | 0.02 | NM_003192.3 | 1300 | 1274 | 26 |
| 316-PTsup | ZNF318 | 915 | hg19 | chr6 | 43305549 | 43305549 | Nonsense_Mutation | SNP | C | C | A | novel | Somatic | E2063Ter | 0.166023 | NM_014345.3 | 518 | 432 | 86 |
| 316-PTsup | MFSD4B | 1436 | hg19 | chr6 | 111587361 | 111587361 | Frame_Shift_Del | DEL | T | T | - | novel | Somatic | C202VfsTer4 | 0.044776 | NM_153369.3 | 401 | 383 | 18 |
| 316-PTsup | REV3L | 179 | hg19 | chr6 | 111672974 | 111672974 | Missense_Mutation | SNP | A | A | C | novel | Somatic | L2402R | 0.517241 | NM_002912.4 | 261 | 126 | 135 |
| 316-PTsup | PDE7B | 25 | hg19 | chr6 | 136502455 | 136502455 | Missense_Mutation | SNP | A | A | T | novel | Somatic | R347W | 0.226244 | NM_018945.4 | 221 | 171 | 50 |
| 316-PTsup | SYNE1 | 1748 | hg19 | chr6 | 152501394 | 152501394 | Missense_Mutation | SNP | C | C | G | novel | Somatic | L7779F | 0.451537 | NM_182961.4 | 421 | 230 | 191 |
| 316-PTsup | SYNE1 | 1748 | hg19 | chr6 | 152558085 | 152558085 | Splice_Site | SNP | T | T | C | novel | Somatic |  | 0.489104 | NM_182961.4 | 412 | 210 | 202 |
| 316-PTsup | LPA | 226 | hg19 | chr6 | 161026235 | 161026235 | Missense_Mutation | SNP | G | G | C | novel | Somatic | P930A | 0.045307 | NM_005577.4 | 309 | 295 | 14 |
| 316-PTsup | T | 1855 | hg19 | chr6 | 166580195 | 166580195 | Missense_Mutation | SNP | G | G | A | novel | Somatic | A119V | 0.537838 | NM_001366286.1 | 1104 | 507 | 597 |
| 316-PTsup | GRID2IP | 79 | hg19 | chr7 | 6590984 | 6590984 | Missense_Mutation | SNP | G | G | T | novel | Somatic | F28L | 0.364356 | NM_001145118.1 | 1514 | 962 | 552 |
| 316-PTsup | MACC1 | 92 | hg19 | chr7 | 20201463 | 20201463 | Missense_Mutation | SNP | T | T | G | novel | Somatic | H8P | 0.165375 | NM_182762.4 | 385 | 321 | 64 |
| 316-PTsup | ZMIZ2 | 926 | hg19 | chr7 | 44796064 | 44796064 | Missense_Mutation | SNP | A | A | G | novel | Somatic | S31G | 0.366423 | NM_031449.4 | 685 | 434 | 251 |
| 316-PTsup | VSTM2A | 1001 | hg19 | chr7 | 54614695 | 54614695 | Splice_Region | SNP | G | G | C | novel | Somatic |  | 0.151201 | NM_001317843.2 | 1538 | 1305 | 233 |
| 316-PTsup | KCND2 | 187 | hg19 | chr7 | 119915573 | 119915573 | Missense_Mutation | SNP | G | G | A | novel | Somatic | R296Q | 0.081301 | NM_012281.3 | 245 | 225 | 20 |
| 316-PTsup | TAS2R16 | 1520 | hg19 | chr7 | 122635207 | 122635207 | Missense_Mutation | SNP | G | G | T | novel | Somatic | P161Q | 0.27303 | NM_016945.3 | 901 | 655 | 246 |
| 316-PTsup | KCNU1 | 13 | hg19 | chr8 | 36663893 | 36663893 | Frame_Shift_Del | DEL | G | G | - | novel | Somatic | W192CfsTer2 | 0.153846 | NM_001031836.3 | 129 | 109 | 20 |
| 316-PTsup | PLEC | 1691 | hg19 | chr8 | 145003400 | 145003400 | Missense_Mutation | SNP | G | G | C | novel | Somatic | S1181C | 0.689459 | NM_201380.4 | 1753 | 543 | 1210 |
| 316-PTsup | CCDC171 | 10 | hg19 | chr9 | 15777829 | 15777829 | Splice_Region | SNP | G | G | T | novel | Somatic |  | 0.337143 | NM_173550.4 | 175 | 116 | 59 |
| 316-PTsup | C9orf43 | 1471 | hg19 | chr9 | 116191469 | 116191469 | Missense_Mutation | SNP | A | A | T | novel | Somatic | T415S | 0.696078 | NM_001278630.2 | 204 | 62 | 142 |
| 316-PTsup | ANKRD26 | 793 | hg19 | chr10 | 27326186 | 27326186 | Missense_Mutation | SNP | C | C | G | novel | Somatic | D876H | 0.534553 | NM_014915.2 | 491 | 228 | 263 |
| 316-PTsup | RASGEF1A | 918 | hg19 | chr10 | 43694587 | 43694587 | Missense_Mutation | SNP | C | C | T | novel | Somatic | R302Q | 0.480978 | NM_145313.4 | 368 | 191 | 177 |
| 316-PTsup | DPCD | 1373 | hg19 | chr10 | 103354467 | 103354467 | Missense_Mutation | SNP | A | A | T | novel | Somatic | E39D | 0.282723 | NM_001329742.2 | 382 | 274 | 108 |
| 316-PTsup | VWA2 | 1470 | hg19 | chr10 | 116046170 | 116046170 | In_Frame_Ins | INS | - | - | ACA | novel | Somatic | T491dup | 0.347287 | NM_001272046.2 | 643 | 419 | 224 |
| 316-PTsup | NANOS1 | 1506 | hg19 | chr10 | 120789596 | 120789596 | Missense_Mutation | SNP | G | G | A | novel | Somatic | A95T | 0.236016 | NM_199461.4 | 725 | 552 | 173 |
| 316-PTsup | OR52B4 | 618 | hg19 | chr11 | 4388914 | 4388914 | Frame_Shift_Del | DEL | A | A | - | novel | Somatic | L205Ter | 0.43578 | NM_001005161.3 | 217 | 122 | 95 |
| 316-PTsup | PSMC3 | 946 | hg19 | chr11 | 47445677 | 47445677 | Missense_Mutation | SNP | G | G | A | novel | Somatic | R171W | 0.510582 | NM_002804.4 | 378 | 185 | 193 |
| 316-PTsup | OR4C16 | 1007 | hg19 | chr11 | 55340236 | 55340236 | Missense_Mutation | SNP | G | G | A | novel | Somatic | M211I | 0.444444 | NM_001004701.2 | 225 | 125 | 100 |
| 316-PTsup | ARAP1 | 1137 | hg19 | chr11 | 72404510 | 72404510 | Missense_Mutation | SNP | G | G | C | novel | Somatic | R1272G | 0.603477 | NM_001040118.2 | 1780 | 704 | 1076 |
| 316-PTsup | NCAPD3 | 200 | hg19 | chr11 | 134047295 | 134047295 | Missense_Mutation | SNP | C | C | T | novel | Somatic | R947Q | 0.038596 | NM_015261.3 | 285 | 274 | 11 |
| 316-PTsup | CLEC4A | 648 | hg19 | chr12 | 8289394 | 8289394 | Missense_Mutation | SNP | T | T | C | novel | Somatic | F154S | 0.186667 | NM_016184.3 | 225 | 183 | 42 |
| 316-PTsup | OR6C1 | 1010 | hg19 | chr12 | 55714879 | 55714879 | Missense_Mutation | SNP | T | T | C | novel | Somatic | Y166H | 0.183857 | NM_001005182.1 | 223 | 182 | 41 |
| 316-PTsup | RAB3IP | 1120 | hg19 | chr12 | 70150214 | 70150214 | Missense_Mutation | SNP | C | C | G | novel | Somatic | S94C | 0.204852 | NM_022456.5 | 371 | 295 | 76 |
| 316-PTsup | NEDD1 | 1327 | hg19 | chr12 | 97311376 | 97311376 | Intron | DEL | CT | CT | - | novel | Somatic |  | 0.375 | NM_001135175.1 | 59 | 32 | 27 |
| 316-PTsup | APAF1 | 167 | hg19 | chr12 | 99059316 | 99059316 | Intron | INS | - | - | T | novel | Somatic |  | 0.110345 | NM_181861.2 | 144 | 128 | 16 |
| 316-PTsup | ANKLE2 | 1602 | hg19 | chr12 | 133306740 | 133306740 | Nonsense_Mutation | SNP | C | C | A | novel | Somatic | G670Ter | 0.321696 | NM_015114.3 | 400 | 271 | 129 |
| 316-PTsup | DYNC1H1 | 170 | hg19 | chr14 | 102442067 | 102442067 | Missense_Mutation | SNP | G | G | A | novel | Somatic | G92E | 0.195122 | NM_001376.5 | 448 | 360 | 88 |
| 316-PTsup | CPPED1 | 85 | hg19 | chr16 | 12798621 | 12798621 | Missense_Mutation | SNP | G | G | A | novel | Somatic | A192V | 0.43659 | NM_018340.3 | 481 | 271 | 210 |
| 316-PTsup | KIAA0556 | 99 | hg19 | chr16 | 27659994 | 27659994 | Missense_Mutation | SNP | G | G | A | novel | Somatic | D160N | 0.504155 | NM_015202.4 | 358 | 176 | 182 |
| 316-PTsup | XPO6 | 99 | hg19 | chr16 | 28117771 | 28117771 | Frame_Shift_Del | DEL | GG | GG | - | novel | Somatic | L849Ter | 0.033186 | NM_015171.4 | 451 | 436 | 15 |
| 316-PTsup | APOBR | 802 | hg19 | chr16 | 28508737 | 28508737 | Missense_Mutation | SNP | C | C | T | novel | Somatic | S792L | 0.190153 | NM_018690.4 | 588 | 476 | 112 |
| 316-PTsup | PHKB | 118 | hg19 | chr16 | 47732389 | 47732389 | Missense_Mutation | SNP | A | A | G | novel | Somatic | R1012G | 0.287197 | NM_000293.3 | 287 | 204 | 83 |
| 316-PTsup | UTP4 | 1112 | hg19 | chr16 | 69191041 | 69191041 | Missense_Mutation | SNP | G | G | A | novel | Somatic | D448N | 0.037406 | NM_032830.3 | 401 | 386 | 15 |
| 316-PTsup | ZNF276 | 1270 | hg19 | chr16 | 89800317 | 89800317 | Intron | DEL | TCTC | TCTC | - | novel | Somatic |  | 0.203438 | NM_001113525.2 | 349 | 278 | 71 |
| 316-PTsup | SMG6 | 74 | hg19 | chr17 | 1985129 | 1985129 | Missense_Mutation | SNP | T | T | A | novel | Somatic | Q1219L | 0.469586 | NM_017575.5 | 408 | 215 | 193 |
| 316-PTsup | ACADVL | 639 | hg19 | chr17 | 7127806 | 7127806 | Missense_Mutation | SNP | G | G | A | novel | Somatic | R538Q | 0.428295 | NM_000018.4 | 516 | 295 | 221 |
| 316-PTsup | TP53 | 642 | hg19 | chr17 | 7578281 | 7578281 | Missense_Mutation | SNP | G | G | T | novel | Somatic | P190T | 0.677419 | NM_000546.5 | 278 | 89 | 189 |
| 316-PTsup | USP43 | 82 | hg19 | chr17 | 9578291 | 9578291 | Missense_Mutation | SNP | G | G | A | novel | Somatic | R275H | 0.229381 | NM_153210.5 | 387 | 298 | 89 |
| 316-PTsup | MYH2 | 664 | hg19 | chr17 | 10448750 | 10448750 | Missense_Mutation | SNP | C | C | G | novel | Somatic | V140L | 0.203046 | NM_017534.6 | 590 | 470 | 120 |
| 316-PTsup | NOS2 | 784 | hg19 | chr17 | 26096017 | 26096017 | Missense_Mutation | SNP | C | C | T | novel | Somatic | V674M | 0.021552 | NM_000625.4 | 463 | 453 | 10 |
| 316-PTsup | DBF4B | 911 | hg19 | chr17 | 42828436 | 42828436 | Missense_Mutation | SNP | C | C | T | novel | Somatic | R555W | 0.022883 | NM_145663.3 | 437 | 427 | 10 |
| 316-PTsup | SMARCD2 | 1057 | hg19 | chr17 | 61920025 | 61920025 | Missense_Mutation | SNP | G | G | C | novel | Somatic | R4G | 0.204444 | NM_001098426.2 | 225 | 179 | 46 |
| 316-PTsup | COLEC12 | 73 | hg19 | chr18 | 334825 | 334825 | Missense_Mutation | SNP | C | C | G | novel | Somatic | G578A | 0.132916 | NM_130386.3 | 1433 | 1242 | 191 |
| 316-PTsup | ELAVL1 | 646 | hg19 | chr19 | 8028604 | 8028604 | Missense_Mutation | SNP | G | G | C | novel | Somatic | I248M | 0.322335 | NM_001419.3 | 788 | 534 | 254 |
| 316-PTsup | FBN3 | 647 | hg19 | chr19 | 8161355 | 8161355 | Missense_Mutation | SNP | C | C | T | novel | Somatic | A1838T | 0.351852 | NM_032447.5 | 322 | 208 | 114 |
| 316-PTsup | WIZ | 703 | hg19 | chr19 | 15547874 | 15547874 | Missense_Mutation | SNP | G | G | T | novel | Somatic | T828K | 0.236125 | NM_001371589.1 | 988 | 754 | 234 |
| 316-PTsup | OCEL1 | 717 | hg19 | chr19 | 17337610 | 17337610 | Missense_Mutation | SNP | C | C | T | novel | Somatic | R60W | 0.237132 | NM_024578.3 | 1088 | 830 | 258 |
| 316-PTsup | RYR1 | 110 | hg19 | chr19 | 39076818 | 39076818 | Missense_Mutation | SNP | C | C | G | novel | Somatic | L4986V | 0.036789 | NM_000540.3 | 299 | 288 | 11 |
| 316-PTsup | AKT1S1 | 969 | hg19 | chr19 | 50374816 | 50374816 | Missense_Mutation | SNP | C | C | G | novel | Somatic | E225D | 0.028169 | NM_032375.5 | 638 | 620 | 18 |
| 316-PTsup | ZNF528 | 988 | hg19 | chr19 | 52919592 | 52919592 | Missense_Mutation | SNP | A | A | T | novel | Somatic | N496I | 0.297436 | NM_032423.3 | 390 | 274 | 116 |
| 316-PTsup | ZNF468 | 123 | hg19 | chr19 | 53345047 | 53345047 | Missense_Mutation | SNP | G | G | C | novel | Somatic | S167C | 0.289532 | NM_001008801.2 | 448 | 318 | 130 |
| 316-PTsup | ZNF816-ZNF321P | 992 | hg19 | chr19 | 53432619 | 53432619 | Missense_Mutation | SNP | C | C | T | novel | Somatic | G80D | 0.314685 | NM_001202473.1 | 429 | 294 | 135 |
| 316-PTsup | ZFP28 | 1020 | hg19 | chr19 | 57065951 | 57065951 | Missense_Mutation | SNP | G | G | T | novel | Somatic | R599S | 0.284753 | NM_020828.2 | 446 | 319 | 127 |
| 316-PTsup | DZANK1 | 725 | hg19 | chr20 | 18371058 | 18371058 | Missense_Mutation | SNP | C | C | T | novel | Somatic | G647R | 0.487365 | NM_001351683.2 | 554 | 284 | 270 |
| 316-PTsup | ASXL1 | 821 | hg19 | chr20 | 31023249 | 31023249 | Nonsense_Mutation | SNP | A | A | T | novel | Somatic | K912Ter | 0.569686 | NM_015338.6 | 574 | 247 | 327 |
| 316-PTsup | BPIFA3 | 827 | hg19 | chr20 | 31812987 | 31812987 | Missense_Mutation | SNP | G | G | A | novel | Somatic | R157Q | 0.168207 | NM_178466.5 | 541 | 450 | 91 |
| 316-PTsup | ZSWIM3 | 924 | hg19 | chr20 | 44505856 | 44505856 | Missense_Mutation | SNP | A | A | G | novel | Somatic | N220S | 0.222591 | NM_080752.4 | 601 | 467 | 134 |
| 316-PTsup | TOB2 | 904 | hg19 | chr22 | 41832688 | 41832688 | Missense_Mutation | SNP | C | C | T | novel | Somatic | R221H | 0.024758 | NM_016272.4 | 929 | 906 | 23 |
| 316-PTdeep | ZNF436 | 765 | hg19 | chr1 | 23689220 | 23689220 | Missense_Mutation | SNP | C | C | T | novel | Somatic | E219K | 0.288538 | NM_001077195.2 | 253 | 180 | 73 |
| 316-PTdeep | BCAR3 | 1302 | hg19 | chr1 | 94054822 | 94054822 | Missense_Mutation | SNP | C | C | T | novel | Somatic | R214H | 0.363636 | NM_001261408.2 | 626 | 398 | 228 |
| 316-PTdeep | SYPL2 | 1424 | hg19 | chr1 | 110009654 | 110009654 | Intron | DEL | A | A | - | novel | Somatic |  | 0.032037 | NM_001040709.2 | 435 | 421 | 14 |
| 316-PTdeep | WDR77 | 1439 | hg19 | chr1 | 111986004 | 111986004 | Missense_Mutation | SNP | G | G | T | novel | Somatic | P211H | 0.332103 | NM_024102.4 | 271 | 181 | 90 |
| 316-PTdeep | USF1 | 1813 | hg19 | chr1 | 161010052 | 161010052 | Missense_Mutation | SNP | C | C | A | novel | Somatic | L261F | 0.105263 | NM_007122.5 | 284 | 254 | 30 |
| 316-PTdeep | BPNT1 | 2265 | hg19 | chr1 | 220253178 | 220253178 | Missense_Mutation | SNP | C | C | T | novel | Somatic | S4N | 0.076271 | NM_006085.6 | 236 | 218 | 18 |
| 316-PTdeep | MAP2 | 273 | hg19 | chr2 | 210557839 | 210557839 | Missense_Mutation | SNP | T | T | A | novel | Somatic | S315R | 0.138889 | NM_002374.3 | 216 | 186 | 30 |
| 316-PTdeep | GRM7 | 9 | hg19 | chr3 | 7721913 | 7721913 | Missense_Mutation | SNP | T | T | A | novel | Somatic | S877T | 0.171171 | NM_000844.4 | 109 | 90 | 19 |
| 316-PTdeep | GUCA1C | 176 | hg19 | chr3 | 108626895 | 108626895 | Missense_Mutation | SNP | C | C | G | novel | Somatic | G202R | 0.181287 | NM_005459.4 | 171 | 140 | 31 |
| 316-PTdeep | MYLK | 190 | hg19 | chr3 | 123383039 | 123383039 | Missense_Mutation | SNP | C | C | T | novel | Somatic | A1300T | 0.17907 | NM_053025.4 | 429 | 352 | 77 |
| 316-PTdeep | RHO | 1571 | hg19 | chr3 | 129251434 | 129251434 | Missense_Mutation | SNP | G | G | A | novel | Somatic | R252H | 0.026316 | NM_000539.3 | 380 | 370 | 10 |
| 316-PTdeep | SCHIP1 | 1802 | hg19 | chr3 | 159482490 | 159482490 | Missense_Mutation | SNP | G | G | C | novel | Somatic | D108H | 0.07636 | NM_014575.3 | 954 | 881 | 73 |
| 316-PTdeep | PLD1 | 236 | hg19 | chr3 | 171405255 | 171405255 | Missense_Mutation | SNP | C | C | G | novel | Somatic | K553N | 0.073171 | NM_002662.5 | 205 | 190 | 15 |
| 316-PTdeep | EIF4G1 | 1989 | hg19 | chr3 | 184043731 | 184043731 | Missense_Mutation | SNP | A | A | G | novel | Somatic | I1079V | 0.212644 | NM_001194947.1 | 174 | 137 | 37 |
| 316-PTdeep | ATP13A5 | 257 | hg19 | chr3 | 193081961 | 193081961 | Missense_Mutation | SNP | T | T | C | novel | Somatic | R58G | 0.196078 | NM_198505.4 | 255 | 205 | 50 |
| 316-PTdeep | PPARGC1A | 767 | hg19 | chr4 | 23831091 | 23831091 | Missense_Mutation | SNP | C | C | G | novel | Somatic | V183L | 0.347826 | NM_013261.5 | 161 | 105 | 56 |
| 316-PTdeep | ANTXR2 | 1202 | hg19 | chr4 | 80905990 | 80905990 | Frame_Shift_Del | DEL | C | C | - | novel | Somatic | A357PfsTer52 | 0.064655 | NM_058172.6 | 15 | 0 | 15 |
| 316-PTdeep | DNAH5 | 86 | hg19 | chr5 | 13793771 | 13793771 | Missense_Mutation | SNP | C | C | G | novel | Somatic | E2693Q | 0.141509 | NM_001369.2 | 212 | 182 | 30 |
| 316-PTdeep | AGXT2 | 852 | hg19 | chr5 | 35037139 | 35037139 | Missense_Mutation | SNP | C | C | T | novel | Somatic | G132S | 0.177665 | NM_031900.4 | 197 | 162 | 35 |
| 316-PTdeep | DPYSL3 | 26 | hg19 | chr5 | 146780330 | 146780330 | Missense_Mutation | SNP | C | C | A | novel | Somatic | K459N | 0.092251 | NM_001197294.2 | 271 | 246 | 25 |
| 316-PTdeep | HIST1H1C | 783 | hg19 | chr6 | 26056530 | 26056530 | Missense_Mutation | SNP | G | G | C | novel | Somatic | L43V | 0.208333 | NM_005319.3 | 144 | 114 | 30 |
| 316-PTdeep | ZNF318 | 915 | hg19 | chr6 | 43305549 | 43305549 | Nonsense_Mutation | SNP | C | C | A | novel | Somatic | E2063Ter | 0.11032 | NM_014345.3 | 281 | 250 | 31 |
| 316-PTdeep | REV3L | 179 | hg19 | chr6 | 111672974 | 111672974 | Missense_Mutation | SNP | A | A | C | novel | Somatic | L2402R | 0.257143 | NM_002912.4 | 140 | 104 | 36 |
| 316-PTdeep | WISP3 | 1442 | hg19 | chr6 | 112389434 | 112389434 | Frame_Shift_Del | DEL | A | A | - | novel | Somatic | K208NfsTer24 | 0.052239 | NM_198239.2 | 266 | 252 | 14 |
| 316-PTdeep | HDAC2 | 1456 | hg19 | chr6 | 114292040 | 114292040 | Frame_Shift_Del | DEL | T | T | - | novel | Somatic | V12SfsTer20 | 0.028889 | NM_001527.4 | 450 | 437 | 13 |
| 316-PTdeep | SYNE1 | 1748 | hg19 | chr6 | 152501394 | 152501394 | Missense_Mutation | SNP | C | C | G | novel | Somatic | L7779F | 0.186235 | NM_182961.4 | 247 | 201 | 46 |
| 316-PTdeep | SYNE1 | 1748 | hg19 | chr6 | 152558085 | 152558085 | Splice_Site | SNP | T | T | C | novel | Somatic |  | 0.217213 | NM_182961.4 | 243 | 190 | 53 |
| 316-PTdeep | T | 1855 | hg19 | chr6 | 166580195 | 166580195 | Missense_Mutation | SNP | G | G | A | novel | Somatic | A119V | 0.276892 | NM_001366286.1 | 499 | 360 | 139 |
| 316-PTdeep | INTS1 | 596 | hg19 | chr7 | 1527075 | 1527075 | Frame_Shift_Del | DEL | G | G | - | novel | Somatic | R843GfsTer11 | 0.02716 | NM_001080453.3 | 405 | 394 | 11 |
| 316-PTdeep | GRID2IP | 79 | hg19 | chr7 | 6590984 | 6590984 | Missense_Mutation | SNP | G | G | T | novel | Somatic | F28L | 0.186755 | NM_001145118.1 | 750 | 609 | 141 |
| 316-PTdeep | MACC1 | 92 | hg19 | chr7 | 20201463 | 20201463 | Missense_Mutation | SNP | T | T | G | novel | Somatic | H8P | 0.102857 | NM_182762.4 | 175 | 157 | 18 |
| 316-PTdeep | ZMIZ2 | 926 | hg19 | chr7 | 44796064 | 44796064 | Missense_Mutation | SNP | A | A | G | novel | Somatic | S31G | 0.220779 | NM_031449.4 | 302 | 234 | 68 |
| 316-PTdeep | VSTM2A | 1001 | hg19 | chr7 | 54614695 | 54614695 | Splice_Region | SNP | G | G | C | novel | Somatic |  | 0.097222 | NM_001317843.2 | 720 | 650 | 70 |
| 316-PTdeep | TAS2R16 | 1520 | hg19 | chr7 | 122635207 | 122635207 | Missense_Mutation | SNP | G | G | T | novel | Somatic | P161Q | 0.152905 | NM_016945.3 | 327 | 277 | 50 |
| 316-PTdeep | JRK | 1681 | hg19 | chr8 | 143746089 | 143746089 | Missense_Mutation | SNP | A | A | C | novel | Somatic | W463G | 0.054618 | NM_003724.4 | 134 | 79 | 55 |
| 316-PTdeep | PLEC | 1691 | hg19 | chr8 | 145000951 | 145000951 | Splice_Site | DEL | CCT | CCT | - | novel | Somatic |  | 0.042373 | NM_201380.4 | 236 | 226 | 10 |
| 316-PTdeep | PLEC | 1691 | hg19 | chr8 | 145003400 | 145003400 | Missense_Mutation | SNP | G | G | C | novel | Somatic | S1181C | 0.47141 | NM_201380.4 | 786 | 415 | 371 |
| 316-PTdeep | C9orf43 | 1471 | hg19 | chr9 | 116191469 | 116191469 | Missense_Mutation | SNP | A | A | T | novel | Somatic | T415S | 0.326389 | NM_001278630.2 | 144 | 97 | 47 |
| 316-PTdeep | RASGEF1A | 918 | hg19 | chr10 | 43694587 | 43694587 | Missense_Mutation | SNP | C | C | T | novel | Somatic | R302Q | 0.144828 | NM_145313.4 | 145 | 124 | 21 |
| 316-PTdeep | DPCD | 1373 | hg19 | chr10 | 103354467 | 103354467 | Missense_Mutation | SNP | A | A | T | novel | Somatic | E39D | 0.111111 | NM_001329742.2 | 207 | 184 | 23 |
| 316-PTdeep | VWA2 | 1470 | hg19 | chr10 | 116046170 | 116046170 | In_Frame_Ins | INS | - | - | ACA | novel | Somatic | T491dup | 0.07196 | NM_001272046.2 | 401 | 372 | 29 |
| 316-PTdeep | NANOS1 | 1506 | hg19 | chr10 | 120789596 | 120789596 | Missense_Mutation | SNP | G | G | A | novel | Somatic | A95T | 0.080247 | NM_199461.4 | 482 | 443 | 39 |
| 316-PTdeep | OR52B4 | 618 | hg19 | chr11 | 4388914 | 4388914 | Frame_Shift_Del | DEL | A | A | - | novel | Somatic | L205Ter | 0.224138 | NM_001005161.3 | 115 | 89 | 26 |
| 316-PTdeep | PSMC3 | 946 | hg19 | chr11 | 47445677 | 47445677 | Missense_Mutation | SNP | G | G | A | novel | Somatic | R171W | 0.217899 | NM_002804.4 | 257 | 201 | 56 |
| 316-PTdeep | OR4C16 | 1007 | hg19 | chr11 | 55340236 | 55340236 | Missense_Mutation | SNP | G | G | A | novel | Somatic | M211I | 0.22069 | NM_001004701.2 | 145 | 113 | 32 |
| 316-PTdeep | ARAP1 | 1137 | hg19 | chr11 | 72404510 | 72404510 | Missense_Mutation | SNP | G | G | C | novel | Somatic | R1272G | 0.31652 | NM_001040118.2 | 791 | 540 | 251 |
| 316-PTdeep | TPBGL | 1156 | hg19 | chr11 | 74952977 | 74952977 | Missense_Mutation | SNP | G | G | A | novel | Somatic | E295K | 0.19856 | NM_001195528.2 | 490 | 297 | 193 |
| 316-PTdeep | CLEC4A | 648 | hg19 | chr12 | 8289394 | 8289394 | Missense_Mutation | SNP | T | T | C | novel | Somatic | F154S | 0.084746 | NM_016184.3 | 117 | 107 | 10 |
| 316-PTdeep | RAB3IP | 1120 | hg19 | chr12 | 70150214 | 70150214 | Missense_Mutation | SNP | C | C | G | novel | Somatic | S94C | 0.119565 | NM_022456.5 | 184 | 162 | 22 |
| 316-PTdeep | LRRIQ1 | 154 | hg19 | chr12 | 85638646 | 85638646 | Frame_Shift_Del | DEL | A | A | - | novel | Somatic | N1702IfsTer20 | 0.056338 | NM_001079910.2 | 212 | 200 | 12 |
| 316-PTdeep | ANKLE2 | 1602 | hg19 | chr12 | 133306740 | 133306740 | Nonsense_Mutation | SNP | C | C | A | novel | Somatic | G670Ter | 0.141304 | NM_015114.3 | 276 | 237 | 39 |
| 316-PTdeep | DYNC1H1 | 170 | hg19 | chr14 | 102442067 | 102442067 | Missense_Mutation | SNP | G | G | A | novel | Somatic | G92E | 0.127854 | NM_001376.5 | 219 | 191 | 28 |
| 316-PTdeep | TELO2 | 596 | hg19 | chr16 | 1544301 | 1544301 | Missense_Mutation | SNP | G | G | C | novel | Somatic | E7Q | 0.060086 | NM_016111.4 | 139 | 125 | 14 |
| 316-PTdeep | CPPED1 | 85 | hg19 | chr16 | 12798621 | 12798621 | Missense_Mutation | SNP | G | G | A | novel | Somatic | A192V | 0.217241 | NM_018340.3 | 288 | 225 | 63 |
| 316-PTdeep | KIAA0556 | 99 | hg19 | chr16 | 27659994 | 27659994 | Missense_Mutation | SNP | G | G | A | novel | Somatic | D160N | 0.25431 | NM_015202.4 | 232 | 173 | 59 |
| 316-PTdeep | APOBR | 802 | hg19 | chr16 | 28508737 | 28508737 | Missense_Mutation | SNP | C | C | T | novel | Somatic | S792L | 0.079027 | NM_018690.4 | 329 | 303 | 26 |
| 316-PTdeep | PHKB | 118 | hg19 | chr16 | 47732389 | 47732389 | Missense_Mutation | SNP | A | A | G | novel | Somatic | R1012G | 0.163265 | NM_000293.3 | 147 | 123 | 24 |
| 316-PTdeep | ZNF276 | 1270 | hg19 | chr16 | 89800317 | 89800317 | Intron | DEL | TCTC | TCTC | - | novel | Somatic |  | 0.068323 | NM_001113525.2 | 161 | 150 | 11 |
| 316-PTdeep | SMG6 | 74 | hg19 | chr17 | 1985129 | 1985129 | Missense_Mutation | SNP | T | T | A | novel | Somatic | Q1219L | 0.301887 | NM_017575.5 | 212 | 148 | 64 |
| 316-PTdeep | ACADVL | 639 | hg19 | chr17 | 7127806 | 7127806 | Missense_Mutation | SNP | G | G | A | novel | Somatic | R538Q | 0.240506 | NM_000018.4 | 316 | 240 | 76 |
| 316-PTdeep | TP53 | 642 | hg19 | chr17 | 7578281 | 7578281 | Missense_Mutation | SNP | G | G | T | novel | Somatic | P190T | 0.27551 | NM_000546.5 | 196 | 142 | 54 |
| 316-PTdeep | USP43 | 82 | hg19 | chr17 | 9578291 | 9578291 | Missense_Mutation | SNP | G | G | A | novel | Somatic | R275H | 0.12766 | NM_153210.5 | 188 | 164 | 24 |
| 316-PTdeep | MYH2 | 664 | hg19 | chr17 | 10448750 | 10448750 | Missense_Mutation | SNP | C | C | G | novel | Somatic | V140L | 0.083851 | NM_017534.6 | 322 | 295 | 27 |
| 316-PTdeep | SMARCD2 | 1057 | hg19 | chr17 | 61920025 | 61920025 | Missense_Mutation | SNP | G | G | C | novel | Somatic | R4G | 0.084507 | NM_001098426.2 | 213 | 195 | 18 |
| 316-PTdeep | ERN1 | 132 | hg19 | chr17 | 62133223 | 62133223 | Missense_Mutation | SNP | T | T | A | novel | Somatic | Q495L | 0.031646 | NM_001433.5 | 311 | 301 | 10 |
| 316-PTdeep | COLEC12 | 73 | hg19 | chr18 | 334825 | 334825 | Missense_Mutation | SNP | C | C | G | novel | Somatic | G578A | 0.110075 | NM_130386.3 | 535 | 476 | 59 |
| 316-PTdeep | ELAVL1 | 646 | hg19 | chr19 | 8028604 | 8028604 | Missense_Mutation | SNP | G | G | C | novel | Somatic | I248M | 0.14978 | NM_001419.3 | 454 | 386 | 68 |
| 316-PTdeep | FBN3 | 647 | hg19 | chr19 | 8161355 | 8161355 | Missense_Mutation | SNP | C | C | T | novel | Somatic | A1838T | 0.182243 | NM_032447.5 | 213 | 174 | 39 |
| 316-PTdeep | WIZ | 703 | hg19 | chr19 | 15547874 | 15547874 | Missense_Mutation | SNP | G | G | T | novel | Somatic | T828K | 0.104688 | NM_001371589.1 | 640 | 573 | 67 |
| 316-PTdeep | OCEL1 | 717 | hg19 | chr19 | 17337610 | 17337610 | Missense_Mutation | SNP | C | C | T | novel | Somatic | R60W | 0.105714 | NM_024578.3 | 687 | 613 | 74 |
| 316-PTdeep | ZNF528 | 988 | hg19 | chr19 | 52919592 | 52919592 | Missense_Mutation | SNP | A | A | T | novel | Somatic | N496I | 0.191304 | NM_032423.3 | 230 | 186 | 44 |
| 316-PTdeep | ZNF468 | 123 | hg19 | chr19 | 53345047 | 53345047 | Missense_Mutation | SNP | G | G | C | novel | Somatic | S167C | 0.087079 | NM_001008801.2 | 355 | 324 | 31 |
| 316-PTdeep | ZFP28 | 1020 | hg19 | chr19 | 57065951 | 57065951 | Missense_Mutation | SNP | G | G | T | novel | Somatic | R599S | 0.111888 | NM_020828.2 | 285 | 253 | 32 |
| 316-PTdeep | DZANK1 | 725 | hg19 | chr20 | 18371058 | 18371058 | Missense_Mutation | SNP | C | C | T | novel | Somatic | G647R | 0.302789 | NM_001351683.2 | 251 | 175 | 76 |
| 316-PTdeep | ASXL1 | 821 | hg19 | chr20 | 31023249 | 31023249 | Nonsense_Mutation | SNP | A | A | T | novel | Somatic | K912Ter | 0.259649 | NM_015338.6 | 285 | 211 | 74 |
| 316-PTdeep | BPIFA3 | 827 | hg19 | chr20 | 31812987 | 31812987 | Missense_Mutation | SNP | G | G | A | novel | Somatic | R157Q | 0.068027 | NM_178466.5 | 294 | 274 | 20 |
| 316-PTdeep | ZSWIM3 | 924 | hg19 | chr20 | 44505856 | 44505856 | Missense_Mutation | SNP | A | A | G | novel | Somatic | N220S | 0.162791 | NM_080752.4 | 301 | 252 | 49 |
| 316-LNmet | ZNF436 | 765 | hg19 | chr1 | 23689220 | 23689220 | Missense_Mutation | SNP | C | C | T | novel | Somatic | E219K | 0.277487 | NM_001077195.2 | 191 | 138 | 53 |
| 316-LNmet | BCAR3 | 1302 | hg19 | chr1 | 94054822 | 94054822 | Missense_Mutation | SNP | C | C | T | novel | Somatic | R214H | 0.470085 | NM_001261408.2 | 584 | 309 | 275 |
| 316-LNmet | WDR77 | 1439 | hg19 | chr1 | 111986004 | 111986004 | Missense_Mutation | SNP | G | G | T | novel | Somatic | P211H | 0.243119 | NM_024102.4 | 218 | 165 | 53 |
| 316-LNmet | FLG2 | 1747 | hg19 | chr1 | 152326963 | 152326963 | Missense_Mutation | SNP | G | G | C | novel | Somatic | S1100C | 0.141058 | NM_001014342.3 | 396 | 340 | 56 |
| 316-LNmet | USF1 | 1813 | hg19 | chr1 | 161010052 | 161010052 | Missense_Mutation | SNP | C | C | A | novel | Somatic | L261F | 0.197368 | NM_007122.5 | 228 | 183 | 45 |
| 316-LNmet | BPNT1 | 2265 | hg19 | chr1 | 220253178 | 220253178 | Missense_Mutation | SNP | C | C | T | novel | Somatic | S4N | 0.257937 | NM_006085.6 | 251 | 186 | 65 |
| 316-LNmet | TTN | 1955 | hg19 | chr2 | 179440435 | 179440435 | Missense_Mutation | SNP | C | C | T | novel | Somatic | R23475H | 0.119205 | NM_001267550.2 | 151 | 133 | 18 |
| 316-LNmet | NBEAL1 | 267 | hg19 | chr2 | 203922058 | 203922058 | Frame_Shift_Del | DEL | A | A | - | novel | Somatic | E136RfsTer14 | 0.040741 | NM_001114132.1 | 270 | 259 | 11 |
| 316-LNmet | MAP2 | 273 | hg19 | chr2 | 210557839 | 210557839 | Missense_Mutation | SNP | T | T | A | novel | Somatic | S315R | 0.176101 | NM_002374.3 | 159 | 131 | 28 |
| 316-LNmet | SPHKAP | 291 | hg19 | chr2 | 228881429 | 228881429 | Nonsense_Mutation | SNP | G | G | A | novel | Somatic | Q1381Ter | 0.111588 | NM_001142644.2 | 233 | 207 | 26 |
| 316-LNmet | GRM7 | 9 | hg19 | chr3 | 7721913 | 7721913 | Missense_Mutation | SNP | T | T | A | novel | Somatic | S877T | 0.132653 | NM_000844.4 | 97 | 84 | 13 |
| 316-LNmet | C3orf67 | 129 | hg19 | chr3 | 58834787 | 58834787 | Nonsense_Mutation | SNP | C | C | A | novel | Somatic | E593Ter | 0.17734 | NM_001351530.2 | 203 | 167 | 36 |
| 316-LNmet | GUCA1C | 176 | hg19 | chr3 | 108626895 | 108626895 | Missense_Mutation | SNP | C | C | G | novel | Somatic | G202R | 0.091503 | NM_005459.4 | 153 | 139 | 14 |
| 316-LNmet | MYLK | 190 | hg19 | chr3 | 123383039 | 123383039 | Missense_Mutation | SNP | C | C | T | novel | Somatic | A1300T | 0.135678 | NM_053025.4 | 398 | 344 | 54 |
| 316-LNmet | EIF4G1 | 1989 | hg19 | chr3 | 184043731 | 184043731 | Missense_Mutation | SNP | A | A | G | novel | Somatic | I1079V | 0.121076 | NM_001194947.1 | 223 | 196 | 27 |
| 316-LNmet | ATP13A5 | 257 | hg19 | chr3 | 193081961 | 193081961 | Missense_Mutation | SNP | T | T | C | novel | Somatic | R58G | 0.144578 | NM_198505.4 | 249 | 213 | 36 |
| 316-LNmet | PPARGC1A | 767 | hg19 | chr4 | 23831091 | 23831091 | Missense_Mutation | SNP | C | C | G | novel | Somatic | V183L | 0.33125 | NM_013261.5 | 160 | 107 | 53 |
| 316-LNmet | ANK2 | 22 | hg19 | chr4 | 114294510 | 114294510 | Missense_Mutation | SNP | G | G | C | novel | Somatic | G1828R | 0.245614 | NM_001127493.2 | 171 | 129 | 42 |
| 316-LNmet | DNAH5 | 86 | hg19 | chr5 | 13793771 | 13793771 | Missense_Mutation | SNP | C | C | G | novel | Somatic | E2693Q | 0.368821 | NM_001369.2 | 263 | 166 | 97 |
| 316-LNmet | AGXT2 | 852 | hg19 | chr5 | 35037139 | 35037139 | Missense_Mutation | SNP | C | C | T | novel | Somatic | G132S | 0.348123 | NM_031900.4 | 293 | 191 | 102 |
| 316-LNmet | PCDH1 | 1662 | hg19 | chr5 | 141243872 | 141243872 | Missense_Mutation | SNP | C | C | T | novel | Somatic | R675Q | 0.176923 | NM_032420.4 | 130 | 107 | 23 |
| 316-LNmet | C6orf229 | 774 | hg19 | chr6 | 24798949 | 24798949 | Frame_Shift_Ins | INS | - | - | T | novel | Somatic | I46NfsTer5 | 0.04065 | NM_001282492.2 | 240 | 230 | 10 |
| 316-LNmet | HIST1H1C | 783 | hg19 | chr6 | 26056530 | 26056530 | Missense_Mutation | SNP | G | G | C | novel | Somatic | L43V | 0.155556 | NM_005319.3 | 180 | 152 | 28 |
| 316-LNmet | CYB5R4 | 1230 | hg19 | chr6 | 84573961 | 84573961 | Missense_Mutation | SNP | C | C | T | novel | Somatic | T48M | 0.151786 | NM_016230.4 | 223 | 189 | 34 |
| 316-LNmet | EPHA7 | 1303 | hg19 | chr6 | 93964468 | 93964468 | Missense_Mutation | SNP | T | T | C | novel | Somatic | Y810C | 0.147368 | NM_004440.4 | 190 | 162 | 28 |
| 316-LNmet | REV3L | 179 | hg19 | chr6 | 111672974 | 111672974 | Missense_Mutation | SNP | A | A | C | novel | Somatic | L2402R | 0.21118 | NM_002912.4 | 161 | 127 | 34 |
| 316-LNmet | HDAC2 | 1456 | hg19 | chr6 | 114292040 | 114292040 | Frame_Shift_Del | DEL | T | T | - | novel | Somatic | V12SfsTer20 | 0.03632 | NM_001527.4 | 412 | 397 | 15 |
| 316-LNmet | PDE7B | 25 | hg19 | chr6 | 136502455 | 136502455 | Missense_Mutation | SNP | A | A | T | novel | Somatic | R347W | 0.182609 | NM_018945.4 | 115 | 94 | 21 |
| 316-LNmet | SYNE1 | 1748 | hg19 | chr6 | 152501394 | 152501394 | Missense_Mutation | SNP | C | C | G | novel | Somatic | L7779F | 0.291262 | NM_182961.4 | 206 | 146 | 60 |
| 316-LNmet | SYNE1 | 1748 | hg19 | chr6 | 152558085 | 152558085 | Splice_Site | SNP | T | T | C | novel | Somatic |  | 0.280769 | NM_182961.4 | 260 | 187 | 73 |
| 316-LNmet | T | 1855 | hg19 | chr6 | 166580195 | 166580195 | Missense_Mutation | SNP | G | G | A | novel | Somatic | A119V | 0.292784 | NM_001366286.1 | 481 | 339 | 142 |
| 316-LNmet | GRID2IP | 79 | hg19 | chr7 | 6590984 | 6590984 | Missense_Mutation | SNP | G | G | T | novel | Somatic | F28L | 0.229457 | NM_001145118.1 | 643 | 495 | 148 |
| 316-LNmet | ZMIZ2 | 926 | hg19 | chr7 | 44796064 | 44796064 | Missense_Mutation | SNP | A | A | G | novel | Somatic | S31G | 0.258333 | NM_031449.4 | 354 | 261 | 93 |
| 316-LNmet | TAS2R16 | 1520 | hg19 | chr7 | 122635207 | 122635207 | Missense_Mutation | SNP | G | G | T | novel | Somatic | P161Q | 0.198925 | NM_016945.3 | 372 | 298 | 74 |
| 316-LNmet | PLEC | 1691 | hg19 | chr8 | 145003400 | 145003400 | Missense_Mutation | SNP | G | G | C | novel | Somatic | S1181C | 0.547458 | NM_201380.4 | 589 | 266 | 323 |
| 316-LNmet | CCDC171 | 10 | hg19 | chr9 | 15777829 | 15777829 | Splice_Region | SNP | G | G | T | novel | Somatic |  | 0.28125 | NM_173550.4 | 96 | 69 | 27 |
| 316-LNmet | AQP7 | 839 | hg19 | chr9 | 33386510 | 33386510 | Missense_Mutation | SNP | C | C | T | novel | Somatic | A100T | 0.034335 | NM_001170.3 | 274 | 258 | 16 |
| 316-LNmet | PALM2-AKAP2 | 180 | hg19 | chr9 | 112778239 | 112778239 | Missense_Mutation | SNP | G | G | C | novel | Somatic | G168R | 0.176136 | NM_007203.5 | 176 | 145 | 31 |
| 316-LNmet | C9orf43 | 1471 | hg19 | chr9 | 116191469 | 116191469 | Missense_Mutation | SNP | A | A | T | novel | Somatic | T415S | 0.301887 | NM_001278630.2 | 106 | 74 | 32 |
| 316-LNmet | GPR158 | 97 | hg19 | chr10 | 25887088 | 25887088 | Nonsense_Mutation | SNP | G | G | T | novel | Somatic | E845Ter | 0.082759 | NM_020752.3 | 144 | 132 | 12 |
| 316-LNmet | ANKRD26 | 793 | hg19 | chr10 | 27326186 | 27326186 | Missense_Mutation | SNP | C | C | G | novel | Somatic | D876H | 0.284404 | NM_014915.2 | 218 | 156 | 62 |
| 316-LNmet | RASGEF1A | 918 | hg19 | chr10 | 43694587 | 43694587 | Missense_Mutation | SNP | C | C | T | novel | Somatic | R302Q | 0.366667 | NM_145313.4 | 150 | 95 | 55 |
| 316-LNmet | DPCD | 1373 | hg19 | chr10 | 103354467 | 103354467 | Missense_Mutation | SNP | A | A | T | novel | Somatic | E39D | 0.145349 | NM_001329742.2 | 172 | 147 | 25 |
| 316-LNmet | SORCS1 | 176 | hg19 | chr10 | 108459060 | 108459060 | Missense_Mutation | SNP | T | T | C | novel | Somatic | D442G | 0.145946 | NM_001013031.3 | 185 | 158 | 27 |
| 316-LNmet | VWA2 | 1470 | hg19 | chr10 | 116046170 | 116046170 | In_Frame_Ins | INS | - | - | ACA | novel | Somatic | T491dup | 0.158501 | NM_001272046.2 | 347 | 292 | 55 |
| 316-LNmet | NANOS1 | 1506 | hg19 | chr10 | 120789596 | 120789596 | Missense_Mutation | SNP | G | G | A | novel | Somatic | A95T | 0.15493 | NM_199461.4 | 422 | 356 | 66 |
| 316-LNmet | PWWP2B | 0 | hg19 | chr10 | 134218255 | 134218255 | Frame_Shift_Del | DEL | C | C | - | novel | Somatic | P86LfsTer100 | 0.167857 | NM_138499.4 | 278 | 231 | 47 |
| 316-LNmet | PWWP2B | 0 | hg19 | chr10 | 134218530 | 134218530 | Missense_Mutation | SNP | G | G | A | novel | Somatic | V176M | 0.154122 | NM_138499.4 | 834 | 705 | 129 |
| 316-LNmet | OR52B4 | 618 | hg19 | chr11 | 4388914 | 4388914 | Frame_Shift_Del | DEL | A | A | - | novel | Somatic | L205Ter | 0.213115 | NM_001005161.3 | 122 | 96 | 26 |
| 316-LNmet | PSMC3 | 946 | hg19 | chr11 | 47445677 | 47445677 | Missense_Mutation | SNP | G | G | A | novel | Somatic | R171W | 0.268775 | NM_002804.4 | 249 | 181 | 68 |
| 316-LNmet | OR4C16 | 1007 | hg19 | chr11 | 55340236 | 55340236 | Missense_Mutation | SNP | G | G | A | novel | Somatic | M211I | 0.259259 | NM_001004701.2 | 108 | 80 | 28 |
| 316-LNmet | DTX4 | 1034 | hg19 | chr11 | 58949308 | 58949308 | Missense_Mutation | SNP | C | C | T | novel | Somatic | T103M | 0.144022 | NM_015177.2 | 367 | 314 | 53 |
| 316-LNmet | ARAP1 | 1137 | hg19 | chr11 | 72404510 | 72404510 | Missense_Mutation | SNP | G | G | C | novel | Somatic | R1272G | 0.447619 | NM_001040118.2 | 733 | 404 | 329 |
| 316-LNmet | TPBGL | 1156 | hg19 | chr11 | 74952977 | 74952977 | Missense_Mutation | SNP | G | G | A | novel | Somatic | E295K | 0.220917 | NM_001195528.2 | 450 | 262 | 188 |
| 316-LNmet | MMP12 | 1368 | hg19 | chr11 | 102742630 | 102742630 | Missense_Mutation | SNP | G | G | A | novel | Somatic | R135W | 0.072626 | NM_002426.6 | 358 | 332 | 26 |
| 316-LNmet | RAB3IP | 1120 | hg19 | chr12 | 70150214 | 70150214 | Missense_Mutation | SNP | C | C | G | novel | Somatic | S94C | 0.110497 | NM_022456.5 | 181 | 161 | 20 |
| 316-LNmet | UNC119B | 1509 | hg19 | chr12 | 121154535 | 121154535 | Missense_Mutation | SNP | G | G | A | novel | Somatic | G155R | 0.114754 | NM_001080533.3 | 243 | 215 | 28 |
| 316-LNmet | ANKLE2 | 1602 | hg19 | chr12 | 133306740 | 133306740 | Nonsense_Mutation | SNP | C | C | A | novel | Somatic | G670Ter | 0.22541 | NM_015114.3 | 244 | 189 | 55 |
| 316-LNmet | GAS6 | 182 | hg19 | chr13 | 114523828 | 114523828 | Nonstop_Mutation | DEL | CGTGGGGGCCTAGGCTGCGGCGGGCTCCA | CGTGGGGGCCTAGGCTGCGGCGGGCTCCA | - | novel | Somatic |  | 0.042959 | NM_000820.4 | 415 | 397 | 18 |
| 316-LNmet | DYNC1H1 | 170 | hg19 | chr14 | 102442067 | 102442067 | Missense_Mutation | SNP | G | G | A | novel | Somatic | G92E | 0.195652 | NM_001376.5 | 276 | 222 | 54 |
| 316-LNmet | CPPED1 | 85 | hg19 | chr16 | 12798621 | 12798621 | Missense_Mutation | SNP | G | G | A | novel | Somatic | A192V | 0.172535 | NM_018340.3 | 284 | 235 | 49 |
| 316-LNmet | KIAA0556 | 99 | hg19 | chr16 | 27659994 | 27659994 | Missense_Mutation | SNP | G | G | A | novel | Somatic | D160N | 0.184049 | NM_015202.4 | 163 | 133 | 30 |
| 316-LNmet | XPO6 | 99 | hg19 | chr16 | 28117771 | 28117771 | Frame_Shift_Del | DEL | GG | GG | - | novel | Somatic | L849Ter | 0.068421 | NM_015171.4 | 190 | 177 | 13 |
| 316-LNmet | PHKB | 118 | hg19 | chr16 | 47732389 | 47732389 | Missense_Mutation | SNP | A | A | G | novel | Somatic | R1012G | 0.161491 | NM_000293.3 | 161 | 135 | 26 |
| 316-LNmet | UTP4 | 1112 | hg19 | chr16 | 69191041 | 69191041 | Missense_Mutation | SNP | G | G | A | novel | Somatic | D448N | 0.110169 | NM_032830.3 | 236 | 210 | 26 |
| 316-LNmet | SMG6 | 74 | hg19 | chr17 | 1985129 | 1985129 | Missense_Mutation | SNP | T | T | A | novel | Somatic | Q1219L | 0.123223 | NM_017575.5 | 210 | 184 | 26 |
| 316-LNmet | ACADVL | 639 | hg19 | chr17 | 7127806 | 7127806 | Missense_Mutation | SNP | G | G | A | novel | Somatic | R538Q | 0.089457 | NM_000018.4 | 313 | 285 | 28 |
| 316-LNmet | TP53 | 642 | hg19 | chr17 | 7578281 | 7578281 | Missense_Mutation | SNP | G | G | T | novel | Somatic | P190T | 0.370968 | NM_000546.5 | 186 | 117 | 69 |
| 316-LNmet | USP43 | 82 | hg19 | chr17 | 9578291 | 9578291 | Missense_Mutation | SNP | G | G | A | novel | Somatic | R275H | 0.245399 | NM_153210.5 | 162 | 122 | 40 |
| 316-LNmet | MYH4 | 82 | hg19 | chr17 | 10363546 | 10363546 | Missense_Mutation | SNP | C | C | G | novel | Somatic | V414L | 0.164894 | NM_017533.2 | 188 | 157 | 31 |
| 316-LNmet | ELAVL1 | 646 | hg19 | chr19 | 8028604 | 8028604 | Missense_Mutation | SNP | G | G | C | novel | Somatic | I248M | 0.171053 | NM_001419.3 | 456 | 378 | 78 |
| 316-LNmet | FBN3 | 647 | hg19 | chr19 | 8161355 | 8161355 | Missense_Mutation | SNP | C | C | T | novel | Somatic | A1838T | 0.24537 | NM_032447.5 | 216 | 163 | 53 |
| 316-LNmet | WIZ | 703 | hg19 | chr19 | 15547874 | 15547874 | Missense_Mutation | SNP | G | G | T | novel | Somatic | T828K | 0.113636 | NM_001371589.1 | 484 | 429 | 55 |
| 316-LNmet | OCEL1 | 717 | hg19 | chr19 | 17337610 | 17337610 | Missense_Mutation | SNP | C | C | T | novel | Somatic | R60W | 0.180769 | NM_024578.3 | 513 | 419 | 94 |
| 316-LNmet | FBXO17 | 885 | hg19 | chr19 | 39439234 | 39439234 | In_Frame_Del | DEL | GAA | GAA | - | novel | Somatic | S145del | 0.094937 | NM_024907.7 | 158 | 143 | 15 |
| 316-LNmet | ZNF528 | 988 | hg19 | chr19 | 52919592 | 52919592 | Missense_Mutation | SNP | A | A | T | novel | Somatic | N496I | 0.161765 | NM_032423.3 | 272 | 228 | 44 |
| 316-LNmet | ZNF816-ZNF321P | 992 | hg19 | chr19 | 53432619 | 53432619 | Missense_Mutation | SNP | C | C | T | novel | Somatic | G80D | 0.10585 | NM_001202473.1 | 358 | 320 | 38 |
| 316-LNmet | ZFP28 | 1020 | hg19 | chr19 | 57065951 | 57065951 | Missense_Mutation | SNP | G | G | T | novel | Somatic | R599S | 0.288079 | NM_020828.2 | 300 | 213 | 87 |
| 316-LNmet | DZANK1 | 725 | hg19 | chr20 | 18371058 | 18371058 | Missense_Mutation | SNP | C | C | T | novel | Somatic | G647R | 0.186364 | NM_001351683.2 | 218 | 177 | 41 |
| 316-LNmet | ASXL1 | 821 | hg19 | chr20 | 31023249 | 31023249 | Nonsense_Mutation | SNP | A | A | T | novel | Somatic | K912Ter | 0.363057 | NM_015338.6 | 314 | 200 | 114 |
| 316-LNmet | BPIFA3 | 827 | hg19 | chr20 | 31812987 | 31812987 | Missense_Mutation | SNP | G | G | A | novel | Somatic | R157Q | 0.14741 | NM_178466.5 | 251 | 214 | 37 |
| 316-LNmet | FAM83D | 871 | hg19 | chr20 | 37580769 | 37580769 | Missense_Mutation | SNP | G | G | T | novel | Somatic | G455V | 0.072464 | NM_030919.3 | 276 | 256 | 20 |
| 316-LNmet | ZSWIM3 | 924 | hg19 | chr20 | 44505856 | 44505856 | Missense_Mutation | SNP | A | A | G | novel | Somatic | N220S | 0.183562 | NM_080752.4 | 365 | 298 | 67 |
| 316-LNmet | DIP2A | 950 | hg19 | chr21 | 47978256 | 47978256 | Missense_Mutation | SNP | G | G | A | novel | Somatic | A1307T | 0.118644 | NM_015151.4 | 470 | 414 | 56 |
| 348--PTsup | MORN1 | 602 | hg19 | chr1 | 2305899 | 2305899 | Splice_Site | SNP | C | C | T | novel | Somatic |  | 0.438202 | NM_024848.3 | 89 | 50 | 39 |
| 348--PTsup | CHD5 | 632 | hg19 | chr1 | 6206795 | 6206795 | Missense_Mutation | SNP | G | G | C | novel | Somatic | P507R | 0.25 | NM_015557.3 | 312 | 234 | 78 |
| 348--PTsup | DISP3 | 673 | hg19 | chr1 | 11580878 | 11580878 | Missense_Mutation | SNP | G | G | A | novel | Somatic | E779K | 0.255941 | NM_020780.2 | 547 | 407 | 140 |
| 348--PTsup | VPS13D | 84 | hg19 | chr1 | 12337738 | 12337738 | Missense_Mutation | SNP | C | C | G | novel | Somatic | L1365V | 0.2 | NM_015378.4 | 130 | 104 | 26 |
| 348--PTsup | HP1BP3 | 93 | hg19 | chr1 | 21106402 | 21106402 | Splice_Region | SNP | C | C | T | novel | Somatic | K33= | 0.108911 | NM_001372052.1 | 101 | 90 | 11 |
| 348--PTsup | SLC44A5 | 145 | hg19 | chr1 | 75699753 | 75699753 | Missense_Mutation | SNP | C | C | A | novel | Somatic | W257C | 0.248804 | NM_152697.5 | 208 | 156 | 52 |
| 348--PTsup | ZZZ3 | 147 | hg19 | chr1 | 78098624 | 78098624 | Nonsense_Mutation | SNP | G | G | C | novel | Somatic | S139Ter | 0.452941 | NM_015534.6 | 170 | 93 | 77 |
| 348--PTsup | ARHGAP29 | 1307 | hg19 | chr1 | 94643497 | 94643497 | Missense_Mutation | SNP | C | C | T | novel | Somatic | D903N | 0.106509 | NM_004815.4 | 169 | 151 | 18 |
| 348--PTsup | SLC44A3 | 1312 | hg19 | chr1 | 95286524 | 95286524 | Missense_Mutation | SNP | C | C | A | novel | Somatic | P16H | 0.086022 | NM_001114106.3 | 186 | 170 | 16 |
| 348--PTsup | TMIGD3 | 1439 | hg19 | chr1 | 112031623 | 112031623 | Missense_Mutation | SNP | C | C | T | novel | Somatic | V161I | 0.13806 | NM_020683.7 | 268 | 231 | 37 |
| 348--PTsup | IQGAP3 | 222 | hg19 | chr1 | 156504298 | 156504298 | Missense_Mutation | SNP | C | C | A | novel | Somatic | K1245N | 0.272727 | NM_178229.5 | 187 | 136 | 51 |
| 348--PTsup | SH2D2A | 1781 | hg19 | chr1 | 156784898 | 156784898 | Missense_Mutation | SNP | T | T | G | novel | Somatic | K80T | 0.113636 | NM_003975.4 | 132 | 117 | 15 |
| 348--PTsup | ARHGEF11 | 1782 | hg19 | chr1 | 156926326 | 156926326 | Nonsense_Mutation | SNP | G | G | T | novel | Somatic | Y519Ter | 0.290909 | NM_198236.3 | 165 | 117 | 48 |
| 348--PTsup | GPR161 | 1867 | hg19 | chr1 | 168066228 | 168066228 | Frame_Shift_Ins | INS | - | - | CCAG | novel | Somatic | V226AfsTer89 | 0.246575 | NM_001267609.1 | 145 | 109 | 36 |
| 348--PTsup | F5 | 1878 | hg19 | chr1 | 169487772 | 169487772 | Missense_Mutation | SNP | C | C | T | novel | Somatic | G2075R | 0.214286 | NM_000130.5 | 154 | 121 | 33 |
| 348--PTsup | ZBTB41 | 32 | hg19 | chr1 | 197128819 | 197128819 | Missense_Mutation | SNP | C | C | G | novel | Somatic | Q800H | 0.130682 | NM_194314.2 | 175 | 152 | 23 |
| 348--PTsup | NAV1 | 265 | hg19 | chr1 | 201687796 | 201687796 | Missense_Mutation | SNP | C | C | T | novel | Somatic | S380L | 0.458262 | NM_020443.4 | 585 | 316 | 269 |
| 348--PTsup | KCNH1 | 274 | hg19 | chr1 | 211307265 | 211307265 | Missense_Mutation | SNP | C | C | T | novel | Somatic | R8K | 0.267416 | NM_172362.3 | 443 | 324 | 119 |
| 348--PTsup | CENPF | 277 | hg19 | chr1 | 214818141 | 214818141 | Missense_Mutation | SNP | C | C | T | novel | Somatic | S1743L | 0.146667 | NM_016343.4 | 150 | 128 | 22 |
| 348--PTsup | DNAH14 | 2302 | hg19 | chr1 | 225477692 | 225477692 | Missense_Mutation | SNP | G | G | A | novel | Somatic | E2793K | 0.11828 | NM_001367479.1 | 186 | 164 | 22 |
| 348--PTsup | DNAH14 | 2302 | hg19 | chr1 | 225488547 | 225488547 | Intron | SNP | C | C | A | novel | Somatic |  | 0.083333 | NM_001367479.1 | 144 | 132 | 12 |
| 348--PTsup | ACBD3 | 288 | hg19 | chr1 | 226352490 | 226352490 | Frame_Shift_Ins | INS | - | - | T | novel | Somatic | R190KfsTer11 | 0.051643 | NM_022735.4 | 210 | 199 | 11 |
| 348--PTsup | CHRM3 | 301 | hg19 | chr1 | 240071836 | 240071836 | Missense_Mutation | SNP | G | G | C | novel | Somatic | R362T | 0.126866 | NM_001347716.2 | 134 | 117 | 17 |
| 348--PTsup | ROCK2 | 83 | hg19 | chr2 | 11338637 | 11338637 | Missense_Mutation | SNP | C | C | G | novel | Somatic | E1027Q | 0.192308 | NM_004850.5 | 78 | 63 | 15 |
| 348--PTsup | AGBL5 | 793 | hg19 | chr2 | 27277616 | 27277616 | Missense_Mutation | SNP | C | C | G | novel | Somatic | L224V | 0.202703 | NM_021831.6 | 148 | 118 | 30 |
| 348--PTsup | SLC4A1AP | 797 | hg19 | chr2 | 27911547 | 27911547 | Intron | INS | - | - | T | novel | Somatic |  | 0.129412 | NM_018158.2 | 85 | 74 | 11 |
| 348--PTsup | PTCD3 | 1243 | hg19 | chr2 | 86333397 | 86333397 | Nonsense_Mutation | SNP | G | G | A | novel | Somatic | W9Ter | 0.259615 | NM_017952.6 | 103 | 76 | 27 |
| 348--PTsup | TFCP2L1 | 189 | hg19 | chr2 | 122004491 | 122004491 | Missense_Mutation | SNP | A | A | T | novel | Somatic | V187E | 0.42029 | NM_014553.3 | 138 | 80 | 58 |
| 348--PTsup | NCKAP5 | 0 | hg19 | chr2 | 133489543 | 133489543 | Missense_Mutation | SNP | C | C | T | novel | Somatic | R1737H | 0.185484 | NM_207363.3 | 124 | 101 | 23 |
| 348--PTsup | NEB | 218 | hg19 | chr2 | 152398036 | 152398036 | Missense_Mutation | SNP | C | C | A | novel | Somatic | Q6869H | 0.315068 | NM_001271208.2 | 73 | 50 | 23 |
| 348--PTsup | TTN | 1955 | hg19 | chr2 | 179495007 | 179495007 | Missense_Mutation | SNP | C | C | T | novel | Somatic | A14748T | 0.134021 | NM_001267550.2 | 194 | 168 | 26 |
| 348--PTsup | ORC2 | 2124 | hg19 | chr2 | 201778067 | 201778067 | Missense_Mutation | SNP | C | C | T | novel | Somatic | R533Q | 0.114094 | NM_006190.5 | 149 | 132 | 17 |
| 348--PTsup | CNPPD1 | 2263 | hg19 | chr2 | 220037597 | 220037597 | Nonsense_Mutation | SNP | G | G | C | novel | Somatic | S315Ter | 0.14346 | NM_015680.6 | 237 | 203 | 34 |
| 348--PTsup | NKTR | 910 | hg19 | chr3 | 42680716 | 42680716 | Missense_Mutation | SNP | G | G | C | novel | Somatic | E1174Q | 0.203704 | NM_005385.4 | 108 | 86 | 22 |
| 348--PTsup | CCDC191 | 181 | hg19 | chr3 | 113724522 | 113724522 | Missense_Mutation | SNP | C | C | A | novel | Somatic | K567N | 0.103321 | NM_020817.2 | 269 | 241 | 28 |
| 348--PTsup | MLF1 | 1792 | hg19 | chr3 | 158322988 | 158322988 | Missense_Mutation | SNP | A | A | C | novel | Somatic | K299N | 0.156069 | NM_001195432.2 | 172 | 145 | 27 |
| 348--PTsup | PIK3CA | 243 | hg19 | chr3 | 178952085 | 178952085 | Missense_Mutation | SNP | A | A | G | novel | Somatic | H1047R | 0.211538 | NM_006218.4 | 104 | 82 | 22 |
| 348--PTsup | ATP13A5 | 257 | hg19 | chr3 | 192992892 | 192992892 | Missense_Mutation | SNP | G | G | A | novel | Somatic | P1199L | 0.093525 | NM_198505.4 | 138 | 125 | 13 |
| 348--PTsup | ATP13A5 | 257 | hg19 | chr3 | 192992952 | 192992952 | Missense_Mutation | SNP | G | G | A | novel | Somatic | S1179L | 0.125 | NM_198505.4 | 159 | 139 | 20 |
| 348--PTsup | PRKG2 | 151 | hg19 | chr4 | 82092896 | 82092896 | Missense_Mutation | SNP | A | A | C | novel | Somatic | F231V | 0.2 | NM_001363401.2 | 165 | 132 | 33 |
| 348--PTsup | WDFY3 | 154 | hg19 | chr4 | 85609253 | 85609253 | Missense_Mutation | SNP | T | T | C | novel | Somatic | I3177V | 0.225225 | NM_014991.5 | 111 | 86 | 25 |
| 348--PTsup | KIAA1109 | 190 | hg19 | chr4 | 123195582 | 123195582 | Missense_Mutation | SNP | C | C | G | novel | Somatic | P2844R | 0.240876 | NM_015312.3 | 137 | 104 | 33 |
| 348--PTsup | INTU | 195 | hg19 | chr4 | 128635181 | 128635181 | Missense_Mutation | SNP | G | G | C | novel | Somatic | E884Q | 0.210526 | NM_015693.4 | 133 | 105 | 28 |
| 348--PTsup | WDR17 | 241 | hg19 | chr4 | 177049909 | 177049909 | Missense_Mutation | SNP | C | C | T | novel | Somatic | R295C | 0.134328 | NM_170710.5 | 133 | 115 | 18 |
| 348--PTsup | TENM3 | 30 | hg19 | chr4 | 183635251 | 183635251 | Missense_Mutation | SNP | G | G | C | novel | Somatic | G745R | 0.290698 | NM_001080477.4 | 86 | 61 | 25 |
| 348--PTsup | NIM1K | 114 | hg19 | chr5 | 43280334 | 43280334 | Missense_Mutation | SNP | C | C | T | novel | Somatic | R272W | 0.26 | NM_153361.4 | 100 | 74 | 26 |
| 348--PTsup | TRIM23 | 1080 | hg19 | chr5 | 64920052 | 64920052 | Missense_Mutation | SNP | C | C | G | novel | Somatic | R17P | 0.05618 | NM_001656.4 | 176 | 166 | 10 |
| 348--PTsup | MTRNR2L2 | 1194 | hg19 | chr5 | 79945871 | 79945871 | Missense_Mutation | SNP | G | G | A | novel | Somatic | S12L | 0.140496 | NM_001190470.2 | 121 | 104 | 17 |
| 348--PTsup | ZCCHC9 | 1199 | hg19 | chr5 | 80600728 | 80600728 | Missense_Mutation | SNP | A | A | G | novel | Somatic | N51S | 0.217021 | NM_032280.2 | 235 | 184 | 51 |
| 348--PTsup | PRRC1 | 24 | hg19 | chr5 | 126887532 | 126887532 | Missense_Mutation | SNP | G | G | A | novel | Somatic | R421H | 0.224299 | NM_130809.5 | 107 | 83 | 24 |
| 348--PTsup | NUP153 | 89 | hg19 | chr6 | 17616382 | 17616382 | In_Frame_Del | DEL | AGA | AGA | - | novel | Somatic | S1489del | 0.140845 | NM_001278209.1 | 71 | 61 | 10 |
| 348--PTsup | HIST1H2AK | 797 | hg19 | chr6 | 27806095 | 27806095 | Missense_Mutation | SNP | C | C | G | novel | Somatic | G8A | 0.221014 | NM_003510.2 | 275 | 214 | 61 |
| 348--PTsup | SOGA3 | 194 | hg19 | chr6 | 127796827 | 127796827 | Missense_Mutation | SNP | C | C | T | novel | Somatic | D782N | 0.342084 | NM_001012279.3 | 1214 | 797 | 417 |
| 348--PTsup | LAMA2 | 196 | hg19 | chr6 | 129470144 | 129470144 | Missense_Mutation | SNP | G | G | C | novel | Somatic | E310D | 0.279412 | NM_000426.3 | 68 | 49 | 19 |
| 348--PTsup | LPA | 226 | hg19 | chr6 | 161026093 | 161026093 | Missense_Mutation | SNP | G | G | T | novel | Somatic | A977E | 0.050808 | NM_005577.4 | 433 | 411 | 22 |
| 348--PTsup | PCLO | 151 | hg19 | chr7 | 82476497 | 82476497 | Missense_Mutation | SNP | A | A | G | novel | Somatic | I4574T | 0.349206 | NM_033026.6 | 63 | 41 | 22 |
| 348--PTsup | CALCR | 161 | hg19 | chr7 | 93055823 | 93055823 | Missense_Mutation | SNP | G | G | A | novel | Somatic | R424C | 0.06867 | NM_001742.4 | 233 | 217 | 16 |
| 348--PTsup | CALCR | 161 | hg19 | chr7 | 93065415 | 93065415 | Missense_Mutation | SNP | G | G | A | novel | Somatic | A333V | 0.039683 | NM_001742.4 | 252 | 242 | 10 |
| 348--PTsup | LHFPL3 | 172 | hg19 | chr7 | 104377253 | 104377253 | Missense_Mutation | SNP | G | G | A | novel | Somatic | A193T | 0.077586 | NM_199000.2 | 232 | 214 | 18 |
| 348--PTsup | DPYSL2 | 98 | hg19 | chr8 | 26492389 | 26492389 | Missense_Mutation | SNP | G | G | A | novel | Somatic | E367K | 0.135057 | NM_001197293.3 | 347 | 300 | 47 |
| 348--PTsup | RB1CC1 | 124 | hg19 | chr8 | 53568744 | 53568744 | Missense_Mutation | SNP | G | G | C | novel | Somatic | D1215E | 0.591928 | NM_014781.5 | 222 | 90 | 132 |
| 348--PTsup | RP1 | 1008 | hg19 | chr8 | 55542638 | 55542638 | Missense_Mutation | SNP | G | G | A | novel | Somatic | D2066N | 0.24183 | NM_006269.2 | 153 | 116 | 37 |
| 348--PTsup | TMEM67 | 1308 | hg19 | chr8 | 94772156 | 94772156 | Missense_Mutation | SNP | T | T | A | novel | Somatic | L33H | 0.047059 | NM_001142301.1 | 231 | 219 | 12 |
| 348--PTsup | MAL2 | 1502 | hg19 | chr8 | 120220771 | 120220771 | Frame_Shift_Del | DEL | G | G | - | novel | Somatic | R22GfsTer52 | 0.022523 | NM_052886.3 | 857 | 837 | 20 |
| 348--PTsup | FAM135B | 25 | hg19 | chr8 | 139165041 | 139165041 | Missense_Mutation | SNP | G | G | C | novel | Somatic | S559R | 0.302703 | NM_015912.4 | 185 | 129 | 56 |
| 348--PTsup | NRBP2 | 1690 | hg19 | chr8 | 144919465 | 144919465 | Silent | SNP | C | C | T | novel | Somatic | S361= | 0.031858 | NM_178564.4 | 563 | 545 | 18 |
| 348--PTsup | PHYHD1 | 1589 | hg19 | chr9 | 131689395 | 131689395 | Missense_Mutation | SNP | G | G | A | novel | Somatic | E38K | 0.155462 | NM_174933.4 | 238 | 201 | 37 |
| 348--PTsup | PRRC2B | 201 | hg19 | chr9 | 134358148 | 134358148 | Missense_Mutation | SNP | G | G | A | novel | Somatic | G1743S | 0.268657 | NM_013318.3 | 134 | 98 | 36 |
| 348--PTsup | PHYHIPL | 131 | hg19 | chr10 | 61005333 | 61005333 | Frame_Shift_Ins | INS | - | - | AC | novel | Somatic | I372TfsTer38 | 0.132812 | NM_032439.4 | 127 | 110 | 17 |
| 348--PTsup | CTNNA3 | 17 | hg19 | chr10 | 67680249 | 67680249 | Missense_Mutation | SNP | G | G | T | novel | Somatic | H843N | 0.157407 | NM_013266.4 | 108 | 91 | 17 |
| 348--PTsup | DMBT1 | 191 | hg19 | chr10 | 124395547 | 124395547 | Missense_Mutation | SNP | G | G | A | novel | Somatic | D2068N | 0.162602 | NM_007329.2 | 123 | 103 | 20 |
| 348--PTsup | C10orf120 | 1534 | hg19 | chr10 | 124457413 | 124457413 | Nonsense_Mutation | SNP | G | G | A | novel | Somatic | R282Ter | 0.24 | NM_001010912.4 | 100 | 76 | 24 |
| 348--PTsup | LHPP | 1547 | hg19 | chr10 | 126150487 | 126150487 | Missense_Mutation | SNP | C | C | T | novel | Somatic | S19L | 0.174447 | NM_022126.4 | 402 | 331 | 71 |
| 348--PTsup | CHID1 | 591 | hg19 | chr11 | 902267 | 902267 | Missense_Mutation | SNP | C | C | T | novel | Somatic | V109I | 0.154762 | NM_023947.4 | 84 | 71 | 13 |
| 348--PTsup | OR51G1 | 622 | hg19 | chr11 | 4944875 | 4944875 | Missense_Mutation | SNP | G | G | A | novel | Somatic | S232F | 0.142105 | NM_001005237.1 | 190 | 163 | 27 |
| 348--PTsup | IPO7 | 10 | hg19 | chr11 | 9442030 | 9442030 | Missense_Mutation | SNP | A | A | G | novel | Somatic | I267V | 0.220472 | NM_006391.3 | 127 | 99 | 28 |
| 348--PTsup | NAV2 | 92 | hg19 | chr11 | 19735269 | 19735269 | Missense_Mutation | SNP | A | A | G | novel | Somatic | M10V | 0.367144 | NM_145117.5 | 905 | 572 | 333 |
| 348--PTsup | OR5A1 | 1036 | hg19 | chr11 | 59211095 | 59211095 | Missense_Mutation | SNP | T | T | A | novel | Somatic | Y152N | 0.32 | NM_001004728.1 | 125 | 85 | 40 |
| 348--PTsup | TM7SF2 | 134 | hg19 | chr11 | 64882391 | 64882391 | Missense_Mutation | SNP | G | G | A | novel | Somatic | V244I | 0.131356 | NM_003273.5 | 236 | 205 | 31 |
| 348--PTsup | SART1 | 1086 | hg19 | chr11 | 65745258 | 65745258 | Missense_Mutation | SNP | G | G | A | novel | Somatic | R687Q | 0.024213 | NM_005146.5 | 409 | 399 | 10 |
| 348--PTsup | CCDC81 | 155 | hg19 | chr11 | 86123559 | 86123559 | Missense_Mutation | SNP | G | G | C | novel | Somatic | R450T | 0.265957 | NM_001156474.2 | 94 | 69 | 25 |
| 348--PTsup | PGR | 169 | hg19 | chr11 | 100998606 | 100998606 | Missense_Mutation | SNP | C | C | T | novel | Somatic | R399H | 0.259424 | NM_000926.4 | 440 | 323 | 117 |
| 348--PTsup | OR8B12 | 1534 | hg19 | chr11 | 124412656 | 124412656 | Missense_Mutation | SNP | C | C | A | novel | Somatic | A299S | 0.377358 | NM_001005195.1 | 53 | 33 | 20 |
| 348--PTsup | LTBR | 634 | hg19 | chr12 | 6499462 | 6499462 | Missense_Mutation | SNP | G | G | A | novel | Somatic | R329K | 0.202765 | NM_002342.3 | 217 | 173 | 44 |
| 348--PTsup | TPH2 | 17 | hg19 | chr12 | 72425090 | 72425090 | Missense_Mutation | SNP | G | G | T | novel | Somatic | C406F | 0.287671 | NM_173353.4 | 146 | 104 | 42 |
| 348--PTsup | MYF6 | 1203 | hg19 | chr12 | 81102337 | 81102337 | Missense_Mutation | SNP | C | C | A | novel | Somatic | S185Y | 0.164975 | NM_002469.3 | 394 | 329 | 65 |
| 348--PTsup | ANO4 | 169 | hg19 | chr12 | 101520711 | 101520711 | Missense_Mutation | SNP | A | A | G | novel | Somatic | K911E | 0.134752 | NM_001286615.2 | 141 | 122 | 19 |
| 348--PTsup | MYO16 | 177 | hg19 | chr13 | 109318232 | 109318232 | Splice_Site | SNP | A | A | G | novel | Somatic |  | 0.456522 | NM_001198950.3 | 92 | 50 | 42 |
| 348--PTsup | TTC6 | 109 | hg19 | chr14 | 38222307 | 38222307 | Frame_Shift_Ins | INS | - | - | T | novel | Somatic | Y1063LfsTer2 | 0.151603 | NM_001310135.2 | 686 | 582 | 104 |
| 348--PTsup | FANCM | 116 | hg19 | chr14 | 45650640 | 45650640 | Missense_Mutation | SNP | A | A | T | novel | Somatic | Q1410H | 0.36 | NM_020937.4 | 50 | 32 | 18 |
| 348--PTsup | MAGEL2 | 767 | hg19 | chr15 | 23891875 | 23891875 | Missense_Mutation | SNP | G | G | T | novel | Somatic | Q339K | 0.022911 | NM_019066.5 | 739 | 722 | 17 |
| 348--PTsup | CATSPER2 | 920 | hg19 | chr15 | 43941017 | 43941017 | Splice_Region | SNP | C | C | T | novel | Somatic |  | 0.178313 | NM_001282310.2 | 826 | 678 | 148 |
| 348--PTsup | KBTBD13 | 1083 | hg19 | chr15 | 65369982 | 65369982 | Missense_Mutation | SNP | C | C | T | novel | Somatic | R277C | 0.164577 | NM_001101362.2 | 631 | 526 | 105 |
| 348--PTsup | LRRK1 | 169 | hg19 | chr15 | 101562106 | 101562106 | Missense_Mutation | SNP | A | A | G | novel | Somatic | Q599R | 0.202797 | NM_024652.6 | 143 | 114 | 29 |
| 348--PTsup | TELO2 | 596 | hg19 | chr16 | 1544301 | 1544301 | Missense_Mutation | SNP | G | G | C | novel | Somatic | E7Q | 0.141026 | NM_016111.4 | 34 | 1 | 33 |
| 348--PTsup | ZNF629 | 819 | hg19 | chr16 | 30793308 | 30793308 | Missense_Mutation | SNP | C | C | T | novel | Somatic | V781M | 0.424821 | NM_001080417.3 | 416 | 238 | 178 |
| 348--PTsup | HSD3B7 | 821 | hg19 | chr16 | 30997511 | 30997511 | Missense_Mutation | SNP | A | A | G | novel | Somatic | E103G | 0.266129 | NM_025193.4 | 248 | 182 | 66 |
| 348--PTsup | PIEZO1 | 1262 | hg19 | chr16 | 88800395 | 88800395 | Missense_Mutation | SNP | C | C | G | novel | Somatic | E750Q | 0.053388 | NM_001142864.4 | 486 | 460 | 26 |
| 348--PTsup | PABPN1L | 1263 | hg19 | chr16 | 88931506 | 88931506 | Missense_Mutation | SNP | C | C | T | novel | Somatic | E164K | 0.200793 | NM_001080487.2 | 754 | 602 | 152 |
| 348--PTsup | TP53 | 642 | hg19 | chr17 | 7577556 | 7577556 | Missense_Mutation | SNP | C | C | A | novel | Somatic | C242F | 0.669903 | NM_000546.5 | 103 | 34 | 69 |
| 348--PTsup | MYO15A | 722 | hg19 | chr17 | 18045547 | 18045547 | Missense_Mutation | SNP | G | G | C | novel | Somatic | R1935P | 0.263889 | NM_016239.4 | 144 | 106 | 38 |
| 348--PTsup | NF1 | 101 | hg19 | chr17 | 29576048 | 29576048 | Nonsense_Mutation | SNP | C | C | T | novel | Somatic | Q1341Ter | 0.329193 | NM_001042492.3 | 161 | 108 | 53 |
| 348--PTsup | RASL10B | 844 | hg19 | chr17 | 34068173 | 34068173 | Missense_Mutation | SNP | G | G | T | novel | Somatic | G154V | 0.152921 | NM_033315.4 | 580 | 491 | 89 |
| 348--PTsup | ADAM11 | 911 | hg19 | chr17 | 42837203 | 42837203 | Missense_Mutation | SNP | G | G | A | novel | Somatic | G59R | 0.163957 | NM_002390.6 | 730 | 609 | 121 |
| 348--PTsup | HOXB3 | 940 | hg19 | chr17 | 46628465 | 46628465 | In_Frame_Del | DEL | CCG | CCG | - | novel | Somatic | G178del | 0.027778 | NM_002146.4 | 502 | 488 | 14 |
| 348--PTsup | EPN3 | 955 | hg19 | chr17 | 48616320 | 48616320 | Missense_Mutation | SNP | G | G | A | novel | Somatic | E253K | 0.173664 | NM_017957.3 | 522 | 431 | 91 |
| 348--PTsup | ANKFN1 | 15 | hg19 | chr17 | 54588484 | 54588484 | Missense_Mutation | SNP | G | G | A | novel | Somatic | V1099M | 0.180498 | NM_001370326.1 | 963 | 789 | 174 |
| 348--PTsup | FAM104A | 1128 | hg19 | chr17 | 71205681 | 71205681 | Missense_Mutation | SNP | C | C | T | novel | Somatic | R204Q | 0.031185 | NM_001098832.1 | 481 | 466 | 15 |
| 348--PTsup | DNAH17 | 18 | hg19 | chr17 | 76450732 | 76450732 | Missense_Mutation | SNP | G | G | A | novel | Somatic | A3409V | 0.337349 | NM_173628.4 | 331 | 219 | 112 |
| 348--PTsup | CEP295NL | 1171 | hg19 | chr17 | 76888378 | 76888378 | Missense_Mutation | SNP | C | C | G | novel | Somatic | D70H | 0.173554 | NM_001243540.2 | 121 | 100 | 21 |
| 348--PTsup | ZNF750 | 1201 | hg19 | chr17 | 80790311 | 80790311 | Missense_Mutation | SNP | C | C | T | novel | Somatic | R7Q | 0.317308 | NM_024702.3 | 104 | 71 | 33 |
| 348--PTsup | DSG1 | 805 | hg19 | chr18 | 28914059 | 28914059 | Nonsense_Mutation | SNP | C | C | G | novel | Somatic | S300Ter | 0.235772 | NM_001942.4 | 123 | 94 | 29 |
| 348--PTsup | MEX3C | 956 | hg19 | chr18 | 48703671 | 48703671 | Missense_Mutation | SNP | G | G | T | novel | Somatic | P344T | 0.28866 | NM_016626.5 | 191 | 135 | 56 |
| 348--PTsup | CDH7 | 133 | hg19 | chr18 | 63477114 | 63477114 | Missense_Mutation | SNP | A | A | T | novel | Somatic | T129S | 0.395604 | NM_001362438.2 | 91 | 55 | 36 |
| 348--PTsup | CD226 | 1100 | hg19 | chr18 | 67563011 | 67563011 | Missense_Mutation | SNP | C | C | A | novel | Somatic | G218V | 0.259259 | NM_001303618.2 | 81 | 60 | 21 |
| 348--PTsup | NETO1 | 140 | hg19 | chr18 | 70526241 | 70526241 | Missense_Mutation | SNP | T | T | C | novel | Somatic | K97E | 0.261905 | NM_138966.5 | 126 | 93 | 33 |
| 348--PTsup | ZNF564 | 681 | hg19 | chr19 | 12637453 | 12637453 | Missense_Mutation | SNP | C | C | A | novel | Somatic | S490I | 0.301282 | NM_144976.4 | 156 | 109 | 47 |
| 348--PTsup | ZNF429 | 750 | hg19 | chr19 | 21720557 | 21720557 | Missense_Mutation | SNP | A | A | G | novel | Somatic | K568E | 0.060686 | NM_001001415.4 | 753 | 707 | 46 |
| 348--PTsup | ZNF536 | 102 | hg19 | chr19 | 30934847 | 30934847 | Missense_Mutation | SNP | G | G | C | novel | Somatic | K126N | 0.203451 | NM_014717.3 | 1391 | 1108 | 283 |
| 348--PTsup | IGFLR1 | 861 | hg19 | chr19 | 36230697 | 36230697 | Missense_Mutation | SNP | G | G | T | novel | Somatic | P212H | 0.207921 | NM_024660.4 | 303 | 240 | 63 |
| 348--PTsup | ZNF585B | 872 | hg19 | chr19 | 37676788 | 37676788 | Missense_Mutation | SNP | C | C | G | novel | Somatic | E551Q | 0.169576 | NM_152279.4 | 401 | 333 | 68 |
| 348--PTsup | SLC8A2 | 118 | hg19 | chr19 | 47960617 | 47960617 | Nonsense_Mutation | SNP | C | C | A | novel | Somatic | E304Ter | 0.102244 | NM_015063.3 | 790 | 708 | 82 |
| 348--PTsup | SLC8A2 | 118 | hg19 | chr19 | 47960704 | 47960704 | Missense_Mutation | SNP | C | C | T | novel | Somatic | D275N | 0.079503 | NM_015063.3 | 800 | 736 | 64 |
| 348--PTsup | SIGLEC12 | 981 | hg19 | chr19 | 52000614 | 52000614 | Silent | SNP | G | G | T | novel | Somatic | I497= | 0.171053 | NM_053003.4 | 152 | 126 | 26 |
| 348--PTsup | ZNF28 | 991 | hg19 | chr19 | 53303933 | 53303933 | Missense_Mutation | SNP | C | C | T | novel | Somatic | E389K | 0.21573 | NM_006969.5 | 445 | 349 | 96 |
| 348--PTsup | ZNF888 | 992 | hg19 | chr19 | 53410157 | 53410157 | Missense_Mutation | SNP | T | T | G | novel | Somatic | K473T | 0.026564 | NM_001310127.1 | 1162 | 1131 | 31 |
| 348--PTsup | LILRA2 | 1005 | hg19 | chr19 | 55085379 | 55085379 | Missense_Mutation | SNP | G | G | T | novel | Somatic | G12W | 0.111111 | NM_001130917.3 | 368 | 327 | 41 |
| 348--PTsup | NLRP7 | 125 | hg19 | chr19 | 55439141 | 55439141 | Missense_Mutation | SNP | A | A | T | novel | Somatic | L938H | 0.063291 | NM_001127255.1 | 157 | 147 | 10 |
| 348--PTsup | ZNF547 | 1026 | hg19 | chr19 | 57889047 | 57889047 | Missense_Mutation | SNP | T | T | A | novel | Somatic | S235T | 0.02994 | NM_173631.3 | 334 | 324 | 10 |
| 348--PTsup | ZNF416 | 1028 | hg19 | chr19 | 58084178 | 58084178 | Missense_Mutation | SNP | G | G | T | novel | Somatic | S365Y | 0.11157 | NM_017879.3 | 242 | 215 | 27 |
| 348--PTsup | ASXL1 | 821 | hg19 | chr20 | 31022440 | 31022440 | Frame_Shift_Del | DEL | GA | GA | - | novel | Somatic | G645WfsTer12 | 0.045991 | NM_015338.6 | 1671 | 1593 | 78 |
| 348--PTsup | ASXL1 | 821 | hg19 | chr20 | 31022441 | 31022441 | Frame_Shift_Del | DEL | A | A | - | novel | Somatic | G645VfsTer58 | 0.225519 | NM_015338.6 | 1392 | 1012 | 380 |
| 348--PTsup | ZNF831 | 128 | hg19 | chr20 | 57828149 | 57828149 | Missense_Mutation | SNP | A | A | G | novel | Somatic | T1382A | 0.191388 | NM_178457.2 | 208 | 168 | 40 |
| 348--PTsup | SYCP2 | 128 | hg19 | chr20 | 58467314 | 58467314 | Missense_Mutation | SNP | G | G | T | novel | Somatic | H699N | 0.193966 | NM_014258.4 | 232 | 187 | 45 |
| 348--PTsup | PRPF6 | 132 | hg19 | chr20 | 62654231 | 62654231 | Missense_Mutation | SNP | G | G | A | novel | Somatic | R590Q | 0.271429 | NM_012469.4 | 210 | 153 | 57 |
| 348--PTsup | CECR2 | 90 | hg19 | chr22 | 18028845 | 18028845 | Missense_Mutation | SNP | C | C | A | novel | Somatic | A1398D | 0.270677 | NM_001290047.2 | 131 | 95 | 36 |
| 348--PTsup | TUBA8 | 90 | hg19 | chr22 | 18604438 | 18604438 | Missense_Mutation | SNP | G | G | A | novel | Somatic | V66I | 0.226415 | NM_018943.3 | 106 | 82 | 24 |
| 348--PTsup | SCARF2 | 743 | hg19 | chr22 | 20779946 | 20779946 | Missense_Mutation | SNP | C | C | G | novel | Somatic | V776L | 0.13602 | NM_153334.7 | 54 | 0 | 54 |
| 348--PTsup | MAGEB6 | 784 | hg19 | chrX | 26212535 | 26212535 | Missense_Mutation | SNP | G | G | A | novel | Somatic | S191N | 0.568966 | NM_173523.2 | 58 | 25 | 33 |
| 348--PTsup | MAGEB5 | 785 | hg19 | chrX | 26235461 | 26235461 | Missense_Mutation | SNP | G | G | A | novel | Somatic | A15T | 0.378378 | NM_001271752.1 | 37 | 23 | 14 |
| 348--PTsup | IL1RAPL1 | 12 | hg19 | chrX | 29973260 | 29973260 | Missense_Mutation | SNP | C | C | T | novel | Somatic | R472W | 0.28 | NM_014271.4 | 50 | 36 | 14 |
| 348--PTdeep | MORN1 | 602 | hg19 | chr1 | 2305899 | 2305899 | Splice_Site | SNP | C | C | T | novel | Somatic |  | 0.305263 | NM_024848.3 | 94 | 65 | 29 |
| 348--PTdeep | CHD5 | 632 | hg19 | chr1 | 6206795 | 6206795 | Missense_Mutation | SNP | G | G | C | novel | Somatic | P507R | 0.113333 | NM_015557.3 | 150 | 133 | 17 |
| 348--PTdeep | DISP3 | 673 | hg19 | chr1 | 11580878 | 11580878 | Missense_Mutation | SNP | G | G | A | novel | Somatic | E779K | 0.105413 | NM_020780.2 | 349 | 312 | 37 |
| 348--PTdeep | SLC44A5 | 145 | hg19 | chr1 | 75699753 | 75699753 | Missense_Mutation | SNP | C | C | A | novel | Somatic | W257C | 0.193717 | NM_152697.5 | 190 | 153 | 37 |
| 348--PTdeep | ZZZ3 | 147 | hg19 | chr1 | 78098624 | 78098624 | Nonsense_Mutation | SNP | G | G | C | novel | Somatic | S139Ter | 0.296443 | NM_015534.6 | 253 | 178 | 75 |
| 348--PTdeep | TMIGD3 | 1439 | hg19 | chr1 | 112031623 | 112031623 | Missense_Mutation | SNP | C | C | T | novel | Somatic | V161I | 0.117021 | NM_020683.7 | 188 | 166 | 22 |
| 348--PTdeep | IQGAP3 | 222 | hg19 | chr1 | 156504298 | 156504298 | Missense_Mutation | SNP | C | C | A | novel | Somatic | K1245N | 0.144578 | NM_178229.5 | 83 | 71 | 12 |
| 348--PTdeep | ARHGEF11 | 1782 | hg19 | chr1 | 156926326 | 156926326 | Nonsense_Mutation | SNP | G | G | T | novel | Somatic | Y519Ter | 0.116438 | NM_198236.3 | 145 | 128 | 17 |
| 348--PTdeep | GPR161 | 1867 | hg19 | chr1 | 168066228 | 168066228 | Frame_Shift_Ins | INS | - | - | CCAG | novel | Somatic | V226AfsTer89 | 0.132743 | NM_001267609.1 | 113 | 98 | 15 |
| 348--PTdeep | F5 | 1878 | hg19 | chr1 | 169487772 | 169487772 | Missense_Mutation | SNP | C | C | T | novel | Somatic | G2075R | 0.168831 | NM_000130.5 | 76 | 63 | 13 |
| 348--PTdeep | ZBTB41 | 32 | hg19 | chr1 | 197128819 | 197128819 | Missense_Mutation | SNP | C | C | G | novel | Somatic | Q800H | 0.096618 | NM_194314.2 | 207 | 187 | 20 |
| 348--PTdeep | NAV1 | 265 | hg19 | chr1 | 201687796 | 201687796 | Missense_Mutation | SNP | C | C | T | novel | Somatic | S380L | 0.312661 | NM_020443.4 | 386 | 265 | 121 |
| 348--PTdeep | KCNH1 | 274 | hg19 | chr1 | 211307265 | 211307265 | Missense_Mutation | SNP | C | C | T | novel | Somatic | R8K | 0.144522 | NM_172362.3 | 428 | 366 | 62 |
| 348--PTdeep | CENPF | 277 | hg19 | chr1 | 214818141 | 214818141 | Missense_Mutation | SNP | C | C | T | novel | Somatic | S1743L | 0.075758 | NM_016343.4 | 132 | 122 | 10 |
| 348--PTdeep | DNAH14 | 2302 | hg19 | chr1 | 225477692 | 225477692 | Missense_Mutation | SNP | G | G | A | novel | Somatic | E2793K | 0.201299 | NM_001367479.1 | 154 | 123 | 31 |
| 348--PTdeep | TFCP2L1 | 189 | hg19 | chr2 | 122004491 | 122004491 | Missense_Mutation | SNP | A | A | T | novel | Somatic | V187E | 0.340782 | NM_014553.3 | 179 | 118 | 61 |
| 348--PTdeep | NEB | 218 | hg19 | chr2 | 152398036 | 152398036 | Missense_Mutation | SNP | C | C | A | novel | Somatic | Q6869H | 0.119565 | NM_001271208.2 | 91 | 80 | 11 |
| 348--PTdeep | TTN | 1955 | hg19 | chr2 | 179495007 | 179495007 | Missense_Mutation | SNP | C | C | T | novel | Somatic | A14748T | 0.110048 | NM_001267550.2 | 209 | 186 | 23 |
| 348--PTdeep | CERKL | 30 | hg19 | chr2 | 182413290 | 182413290 | Missense_Mutation | SNP | G | G | C | novel | Somatic | S398C | 0.096491 | NM_001030311.2 | 114 | 103 | 11 |
| 348--PTdeep | ORC2 | 2124 | hg19 | chr2 | 201778067 | 201778067 | Missense_Mutation | SNP | C | C | T | novel | Somatic | R533Q | 0.097902 | NM_006190.5 | 142 | 128 | 14 |
| 348--PTdeep | DAZL | 88 | hg19 | chr3 | 16638998 | 16638998 | Missense_Mutation | SNP | T | T | C | novel | Somatic | E98G | 0.072464 | NM_001351.4 | 136 | 126 | 10 |
| 348--PTdeep | NKTR | 910 | hg19 | chr3 | 42680716 | 42680716 | Missense_Mutation | SNP | G | G | C | novel | Somatic | E1174Q | 0.141176 | NM_005385.4 | 170 | 146 | 24 |
| 348--PTdeep | CCDC191 | 181 | hg19 | chr3 | 113724522 | 113724522 | Missense_Mutation | SNP | C | C | A | novel | Somatic | K567N | 0.062696 | NM_020817.2 | 319 | 299 | 20 |
| 348--PTdeep | MLF1 | 1792 | hg19 | chr3 | 158322988 | 158322988 | Missense_Mutation | SNP | A | A | C | novel | Somatic | K299N | 0.090909 | NM_001195432.2 | 209 | 190 | 19 |
| 348--PTdeep | PIK3CA | 243 | hg19 | chr3 | 178952085 | 178952085 | Missense_Mutation | SNP | A | A | G | novel | Somatic | H1047R | 0.19685 | NM_006218.4 | 126 | 101 | 25 |
| 348--PTdeep | ATP13A5 | 257 | hg19 | chr3 | 192992892 | 192992892 | Missense_Mutation | SNP | G | G | A | novel | Somatic | P1199L | 0.136364 | NM_198505.4 | 130 | 112 | 18 |
| 348--PTdeep | ATP13A5 | 257 | hg19 | chr3 | 192992952 | 192992952 | Missense_Mutation | SNP | G | G | A | novel | Somatic | S1179L | 0.107914 | NM_198505.4 | 138 | 123 | 15 |
| 348--PTdeep | PRKG2 | 151 | hg19 | chr4 | 82092896 | 82092896 | Missense_Mutation | SNP | A | A | C | novel | Somatic | F231V | 0.077778 | NM_001363401.2 | 180 | 166 | 14 |
| 348--PTdeep | HPSE | 1227 | hg19 | chr4 | 84223307 | 84223307 | Missense_Mutation | SNP | C | C | A | novel | Somatic | D441Y | 0.23913 | NM_006665.5 | 46 | 35 | 11 |
| 348--PTdeep | WDFY3 | 154 | hg19 | chr4 | 85609253 | 85609253 | Missense_Mutation | SNP | T | T | C | novel | Somatic | I3177V | 0.252101 | NM_014991.5 | 118 | 88 | 30 |
| 348--PTdeep | NDST4 | 183 | hg19 | chr4 | 115767059 | 115767059 | Nonsense_Mutation | SNP | G | G | A | novel | Somatic | R679Ter | 0.064103 | NM_022569.3 | 156 | 146 | 10 |
| 348--PTdeep | INTU | 195 | hg19 | chr4 | 128635181 | 128635181 | Missense_Mutation | SNP | G | G | C | novel | Somatic | E884Q | 0.232759 | NM_015693.4 | 116 | 89 | 27 |
| 348--PTdeep | WDR17 | 241 | hg19 | chr4 | 177049909 | 177049909 | Missense_Mutation | SNP | C | C | T | novel | Somatic | R295C | 0.195652 | NM_170710.5 | 138 | 111 | 27 |
| 348--PTdeep | TENM3 | 30 | hg19 | chr4 | 183635251 | 183635251 | Missense_Mutation | SNP | G | G | C | novel | Somatic | G745R | 0.145833 | NM_001080477.4 | 96 | 82 | 14 |
| 348--PTdeep | NIM1K | 114 | hg19 | chr5 | 43280334 | 43280334 | Missense_Mutation | SNP | C | C | T | novel | Somatic | R272W | 0.144928 | NM_153361.4 | 137 | 117 | 20 |
| 348--PTdeep | ZCCHC9 | 1199 | hg19 | chr5 | 80600728 | 80600728 | Missense_Mutation | SNP | A | A | G | novel | Somatic | N51S | 0.158974 | NM_032280.2 | 195 | 164 | 31 |
| 348--PTdeep | PRRC1 | 24 | hg19 | chr5 | 126887532 | 126887532 | Missense_Mutation | SNP | G | G | A | novel | Somatic | R421H | 0.111111 | NM_130809.5 | 106 | 94 | 12 |
| 348--PTdeep | HIST1H2AK | 797 | hg19 | chr6 | 27806095 | 27806095 | Missense_Mutation | SNP | C | C | G | novel | Somatic | G8A | 0.141026 | NM_003510.2 | 156 | 134 | 22 |
| 348--PTdeep | SOGA3 | 194 | hg19 | chr6 | 127796827 | 127796827 | Missense_Mutation | SNP | C | C | T | novel | Somatic | D782N | 0.254522 | NM_001012279.3 | 771 | 574 | 197 |
| 348--PTdeep | LAMA2 | 196 | hg19 | chr6 | 129470144 | 129470144 | Missense_Mutation | SNP | G | G | C | novel | Somatic | E310D | 0.166667 | NM_000426.3 | 78 | 65 | 13 |
| 348--PTdeep | CCDC129 | 826 | hg19 | chr7 | 31614312 | 31614312 | Missense_Mutation | SNP | A | A | G | novel | Somatic | E211G | 0.102041 | NM_001257968.2 | 97 | 87 | 10 |
| 348--PTdeep | SPDYE1 | 921 | hg19 | chr7 | 44046934 | 44046934 | Missense_Mutation | SNP | G | G | A | novel | Somatic | G234R | 0.050562 | NM_175064.2 | 887 | 842 | 45 |
| 348--PTdeep | PCLO | 151 | hg19 | chr7 | 82476497 | 82476497 | Missense_Mutation | SNP | A | A | G | novel | Somatic | I4574T | 0.271186 | NM_033026.6 | 59 | 43 | 16 |
| 348--PTdeep | LHFPL3 | 172 | hg19 | chr7 | 104377253 | 104377253 | Missense_Mutation | SNP | G | G | A | novel | Somatic | A193T | 0.073826 | NM_199000.2 | 296 | 274 | 22 |
| 348--PTdeep | DPYSL2 | 98 | hg19 | chr8 | 26492389 | 26492389 | Missense_Mutation | SNP | G | G | A | novel | Somatic | E367K | 0.058651 | NM_001197293.3 | 341 | 321 | 20 |
| 348--PTdeep | RB1CC1 | 124 | hg19 | chr8 | 53568744 | 53568744 | Missense_Mutation | SNP | G | G | C | novel | Somatic | D1215E | 0.379032 | NM_014781.5 | 248 | 154 | 94 |
| 348--PTdeep | RP1 | 1008 | hg19 | chr8 | 55542638 | 55542638 | Missense_Mutation | SNP | G | G | A | novel | Somatic | D2066N | 0.22449 | NM_006269.2 | 147 | 114 | 33 |
| 348--PTdeep | PKHD1L1 | 178 | hg19 | chr8 | 110535532 | 110535532 | Missense_Mutation | SNP | A | A | C | novel | Somatic | Q4134P | 0.08042 | NM_177531.6 | 286 | 263 | 23 |
| 348--PTdeep | FAM135B | 25 | hg19 | chr8 | 139165041 | 139165041 | Missense_Mutation | SNP | G | G | C | novel | Somatic | S559R | 0.171271 | NM_015912.4 | 181 | 150 | 31 |
| 348--PTdeep | PHYHD1 | 1589 | hg19 | chr9 | 131689395 | 131689395 | Missense_Mutation | SNP | G | G | A | novel | Somatic | E38K | 0.097315 | NM_174933.4 | 298 | 269 | 29 |
| 348--PTdeep | PRRC2B | 201 | hg19 | chr9 | 134358148 | 134358148 | Missense_Mutation | SNP | G | G | A | novel | Somatic | G1743S | 0.13245 | NM_013318.3 | 151 | 131 | 20 |
| 348--PTdeep | CTNNA3 | 17 | hg19 | chr10 | 67680249 | 67680249 | Missense_Mutation | SNP | G | G | T | novel | Somatic | H843N | 0.086667 | NM_013266.4 | 149 | 136 | 13 |
| 348--PTdeep | DMBT1 | 191 | hg19 | chr10 | 124395547 | 124395547 | Missense_Mutation | SNP | G | G | A | novel | Somatic | D2068N | 0.078313 | NM_007329.2 | 166 | 153 | 13 |
| 348--PTdeep | LHPP | 1547 | hg19 | chr10 | 126150487 | 126150487 | Missense_Mutation | SNP | C | C | T | novel | Somatic | S19L | 0.19186 | NM_022126.4 | 170 | 137 | 33 |
| 348--PTdeep | OR51G1 | 622 | hg19 | chr11 | 4944875 | 4944875 | Missense_Mutation | SNP | G | G | A | novel | Somatic | S232F | 0.120419 | NM_001005237.1 | 191 | 168 | 23 |
| 348--PTdeep | IPO7 | 10 | hg19 | chr11 | 9442030 | 9442030 | Missense_Mutation | SNP | A | A | G | novel | Somatic | I267V | 0.210526 | NM_006391.3 | 114 | 90 | 24 |
| 348--PTdeep | NAV2 | 92 | hg19 | chr11 | 19735269 | 19735269 | Missense_Mutation | SNP | A | A | G | novel | Somatic | M10V | 0.181373 | NM_145117.5 | 612 | 501 | 111 |
| 348--PTdeep | TRIM48 | 1004 | hg19 | chr11 | 55035844 | 55035844 | Missense_Mutation | SNP | T | T | C | novel | Somatic | Y192H | 0.032028 | NM_024114.5 | 279 | 270 | 9 |
| 348--PTdeep | OR5A1 | 1036 | hg19 | chr11 | 59211095 | 59211095 | Missense_Mutation | SNP | T | T | A | novel | Somatic | Y152N | 0.208333 | NM_001004728.1 | 142 | 112 | 30 |
| 348--PTdeep | TM7SF2 | 134 | hg19 | chr11 | 64882391 | 64882391 | Missense_Mutation | SNP | G | G | A | novel | Somatic | V244I | 0.097222 | NM_003273.5 | 144 | 130 | 14 |
| 348--PTdeep | PGR | 169 | hg19 | chr11 | 100998606 | 100998606 | Missense_Mutation | SNP | C | C | T | novel | Somatic | R399H | 0.159722 | NM_000926.4 | 419 | 350 | 69 |
| 348--PTdeep | OR8B12 | 1534 | hg19 | chr11 | 124412656 | 124412656 | Missense_Mutation | SNP | C | C | A | novel | Somatic | A299S | 0.289855 | NM_001005195.1 | 69 | 49 | 20 |
| 348--PTdeep | KCNA5 | 624 | hg19 | chr12 | 5153347 | 5153347 | Missense_Mutation | SNP | G | G | A | novel | Somatic | G12S | 0.04375 | NM_002234.4 | 480 | 459 | 21 |
| 348--PTdeep | LTBR | 634 | hg19 | chr12 | 6499462 | 6499462 | Missense_Mutation | SNP | G | G | A | novel | Somatic | R329K | 0.216346 | NM_002342.3 | 208 | 163 | 45 |
| 348--PTdeep | TPH2 | 17 | hg19 | chr12 | 72425090 | 72425090 | Missense_Mutation | SNP | G | G | T | novel | Somatic | C406F | 0.165217 | NM_173353.4 | 115 | 96 | 19 |
| 348--PTdeep | MYF6 | 1203 | hg19 | chr12 | 81102337 | 81102337 | Missense_Mutation | SNP | C | C | A | novel | Somatic | S185Y | 0.117207 | NM_002469.3 | 401 | 354 | 47 |
| 348--PTdeep | MYO16 | 177 | hg19 | chr13 | 109318232 | 109318232 | Splice_Site | SNP | A | A | G | novel | Somatic |  | 0.212766 | NM_001198950.3 | 94 | 74 | 20 |
| 348--PTdeep | SRP54 | 855 | hg19 | chr14 | 35476596 | 35476596 | Splice_Region | DEL | AAATTGAACTTAATTTAAAAAGA | AAATTGAACTTAATTTAAAAAGA | - | novel | Somatic |  | 0.044855 | NM_003136.4 | 378 | 361 | 17 |
| 348--PTdeep | TTC6 | 109 | hg19 | chr14 | 38222307 | 38222307 | Frame_Shift_Ins | INS | - | - | T | novel | Somatic | Y1063LfsTer2 | 0.161184 | NM_001310135.2 | 301 | 252 | 49 |
| 348--PTdeep | SYNE2 | 1078 | hg19 | chr14 | 64612843 | 64612843 | Missense_Mutation | SNP | A | A | G | novel | Somatic | I5181V | 0.134615 | NM_182914.2 | 104 | 90 | 14 |
| 348--PTdeep | CATSPER2 | 920 | hg19 | chr15 | 43941017 | 43941017 | Splice_Region | SNP | C | C | T | novel | Somatic |  | 0.096953 | NM_001282310.2 | 1799 | 1624 | 175 |
| 348--PTdeep | SPATA5L1 | 933 | hg19 | chr15 | 45697614 | 45697614 | Missense_Mutation | SNP | C | C | G | novel | Somatic | L396V | 0.093525 | NM_024063.3 | 139 | 126 | 13 |
| 348--PTdeep | KBTBD13 | 1083 | hg19 | chr15 | 65369982 | 65369982 | Missense_Mutation | SNP | C | C | T | novel | Somatic | R277C | 0.110759 | NM_001101362.2 | 311 | 276 | 35 |
| 348--PTdeep | LRRK1 | 169 | hg19 | chr15 | 101562106 | 101562106 | Missense_Mutation | SNP | A | A | G | novel | Somatic | Q599R | 0.132911 | NM_024652.6 | 158 | 137 | 21 |
| 348--PTdeep | TELO2 | 596 | hg19 | chr16 | 1544301 | 1544301 | Missense_Mutation | SNP | G | G | C | novel | Somatic | E7Q | 0.12963 | NM_016111.4 | 14 | 0 | 14 |
| 348--PTdeep | ZNF629 | 819 | hg19 | chr16 | 30793308 | 30793308 | Missense_Mutation | SNP | C | C | T | novel | Somatic | V781M | 0.358621 | NM_001080417.3 | 682 | 422 | 260 |
| 348--PTdeep | PABPN1L | 1263 | hg19 | chr16 | 88931506 | 88931506 | Missense_Mutation | SNP | C | C | T | novel | Somatic | E164K | 0.140351 | NM_001080487.2 | 453 | 389 | 64 |
| 348--PTdeep | TP53 | 642 | hg19 | chr17 | 7577556 | 7577556 | Missense_Mutation | SNP | C | C | A | novel | Somatic | C242F | 0.454545 | NM_000546.5 | 153 | 83 | 70 |
| 348--PTdeep | MYO15A | 722 | hg19 | chr17 | 18045547 | 18045547 | Missense_Mutation | SNP | G | G | C | novel | Somatic | R1935P | 0.186667 | NM_016239.4 | 150 | 122 | 28 |
| 348--PTdeep | NF1 | 101 | hg19 | chr17 | 29576048 | 29576048 | Nonsense_Mutation | SNP | C | C | T | novel | Somatic | Q1341Ter | 0.322835 | NM_001042492.3 | 127 | 86 | 41 |
| 348--PTdeep | RASL10B | 844 | hg19 | chr17 | 34068173 | 34068173 | Missense_Mutation | SNP | G | G | T | novel | Somatic | G154V | 0.099359 | NM_033315.4 | 308 | 277 | 31 |
| 348--PTdeep | ADAM11 | 911 | hg19 | chr17 | 42837203 | 42837203 | Missense_Mutation | SNP | G | G | A | novel | Somatic | G59R | 0.105882 | NM_002390.6 | 422 | 377 | 45 |
| 348--PTdeep | EPN3 | 955 | hg19 | chr17 | 48616320 | 48616320 | Missense_Mutation | SNP | G | G | A | novel | Somatic | E253K | 0.098507 | NM_017957.3 | 332 | 299 | 33 |
| 348--PTdeep | ANKFN1 | 15 | hg19 | chr17 | 54588484 | 54588484 | Missense_Mutation | SNP | G | G | A | novel | Somatic | V1099M | 0.10719 | NM_001370326.1 | 765 | 683 | 82 |
| 348--PTdeep | FAM104A | 1128 | hg19 | chr17 | 71205681 | 71205681 | Missense_Mutation | SNP | C | C | T | novel | Somatic | R204Q | 0.025126 | NM_001098832.1 | 398 | 388 | 10 |
| 348--PTdeep | DNAH17 | 18 | hg19 | chr17 | 76450732 | 76450732 | Missense_Mutation | SNP | G | G | A | novel | Somatic | A3409V | 0.224719 | NM_173628.4 | 178 | 138 | 40 |
| 348--PTdeep | CEP295NL | 1171 | hg19 | chr17 | 76888378 | 76888378 | Missense_Mutation | SNP | C | C | G | novel | Somatic | D70H | 0.134752 | NM_001243540.2 | 141 | 122 | 19 |
| 348--PTdeep | ZNF750 | 1201 | hg19 | chr17 | 80790311 | 80790311 | Missense_Mutation | SNP | C | C | T | novel | Somatic | R7Q | 0.214815 | NM_024702.3 | 135 | 106 | 29 |
| 348--PTdeep | DSG1 | 805 | hg19 | chr18 | 28914059 | 28914059 | Nonsense_Mutation | SNP | C | C | G | novel | Somatic | S300Ter | 0.16092 | NM_001942.4 | 87 | 73 | 14 |
| 348--PTdeep | MEX3C | 956 | hg19 | chr18 | 48703671 | 48703671 | Missense_Mutation | SNP | G | G | T | novel | Somatic | P344T | 0.221805 | NM_016626.5 | 266 | 207 | 59 |
| 348--PTdeep | CDH7 | 133 | hg19 | chr18 | 63477114 | 63477114 | Missense_Mutation | SNP | A | A | T | novel | Somatic | T129S | 0.244681 | NM_001362438.2 | 94 | 71 | 23 |
| 348--PTdeep | NETO1 | 140 | hg19 | chr18 | 70526241 | 70526241 | Missense_Mutation | SNP | T | T | C | novel | Somatic | K97E | 0.204545 | NM_138966.5 | 88 | 70 | 18 |
| 348--PTdeep | ZNF429 | 750 | hg19 | chr19 | 21720557 | 21720557 | Missense_Mutation | SNP | A | A | G | novel | Somatic | K568E | 0.060561 | NM_001001415.4 | 675 | 634 | 41 |
| 348--PTdeep | PLEKHF1 | 815 | hg19 | chr19 | 30164786 | 30164786 | Missense_Mutation | SNP | C | C | T | novel | Somatic | R14C | 0.05341 | NM_024310.5 | 1209 | 1144 | 65 |
| 348--PTdeep | ZNF536 | 102 | hg19 | chr19 | 30934847 | 30934847 | Missense_Mutation | SNP | G | G | C | novel | Somatic | K126N | 0.192453 | NM_014717.3 | 795 | 642 | 153 |
| 348--PTdeep | KMT2B | 861 | hg19 | chr19 | 36223858 | 36223858 | Frame_Shift_Del | DEL | C | C | - | novel | Somatic | P2138RfsTer15 | 0.022508 | NM_014727.2 | 620 | 606 | 14 |
| 348--PTdeep | ZNF585B | 872 | hg19 | chr19 | 37676788 | 37676788 | Missense_Mutation | SNP | C | C | G | novel | Somatic | E551Q | 0.139785 | NM_152279.4 | 372 | 320 | 52 |
| 348--PTdeep | SLC8A2 | 118 | hg19 | chr19 | 47960617 | 47960617 | Nonsense_Mutation | SNP | C | C | A | novel | Somatic | E304Ter | 0.187638 | NM_015063.3 | 447 | 362 | 85 |
| 348--PTdeep | SIGLEC12 | 981 | hg19 | chr19 | 52000614 | 52000614 | Silent | SNP | G | G | T | novel | Somatic | I497= | 0.120192 | NM_053003.4 | 208 | 183 | 25 |
| 348--PTdeep | ZNF415 | 994 | hg19 | chr19 | 53612007 | 53612007 | Missense_Mutation | SNP | G | G | A | novel | Somatic | R508W | 0.055961 | NM_001352130.1 | 403 | 380 | 23 |
| 348--PTdeep | LILRA2 | 1005 | hg19 | chr19 | 55085379 | 55085379 | Missense_Mutation | SNP | G | G | T | novel | Somatic | G12W | 0.119048 | NM_001130917.3 | 293 | 258 | 35 |
| 348--PTdeep | NLRP7 | 125 | hg19 | chr19 | 55439141 | 55439141 | Missense_Mutation | SNP | A | A | T | novel | Somatic | L938H | 0.053333 | NM_001127255.1 | 225 | 213 | 12 |
| 348--PTdeep | SHISA7 | 1011 | hg19 | chr19 | 55944700 | 55944700 | In_Frame_Del | DEL | GTG | GTG | - | novel | Somatic | H480del | 0.020286 | NM_001145176.1 | 832 | 815 | 17 |
| 348--PTdeep | FIZ1 | 1013 | hg19 | chr19 | 56108981 | 56108981 | Missense_Mutation | SNP | C | C | A | novel | Somatic | C84F | 0.032333 | NM_032836.3 | 860 | 832 | 28 |
| 348--PTdeep | ZNF416 | 1028 | hg19 | chr19 | 58084178 | 58084178 | Missense_Mutation | SNP | G | G | T | novel | Somatic | S365Y | 0.086957 | NM_017879.3 | 228 | 208 | 20 |
| 348--PTdeep | ASXL1 | 821 | hg19 | chr20 | 31022440 | 31022440 | Frame_Shift_Del | DEL | GA | GA | - | novel | Somatic | G645WfsTer12 | 0.037662 | NM_015338.6 | 1523 | 1465 | 58 |
| 348--PTdeep | ASXL1 | 821 | hg19 | chr20 | 31022441 | 31022441 | Frame_Shift_Del | DEL | A | A | - | novel | Somatic | G645VfsTer58 | 0.199219 | NM_015338.6 | 1291 | 985 | 306 |
| 348--PTdeep | ZNF831 | 128 | hg19 | chr20 | 57828149 | 57828149 | Missense_Mutation | SNP | A | A | G | novel | Somatic | T1382A | 0.129353 | NM_178457.2 | 200 | 174 | 26 |
| 348--PTdeep | SYCP2 | 128 | hg19 | chr20 | 58467314 | 58467314 | Missense_Mutation | SNP | G | G | T | novel | Somatic | H699N | 0.123188 | NM_014258.4 | 137 | 120 | 17 |
| 348--PTdeep | PRPF6 | 132 | hg19 | chr20 | 62654231 | 62654231 | Missense_Mutation | SNP | G | G | A | novel | Somatic | R590Q | 0.175 | NM_012469.4 | 160 | 132 | 28 |
| 348--PTdeep | CECR2 | 90 | hg19 | chr22 | 18028845 | 18028845 | Missense_Mutation | SNP | C | C | A | novel | Somatic | A1398D | 0.173913 | NM_001290047.2 | 115 | 95 | 20 |
| 348--PTdeep | TUBA8 | 90 | hg19 | chr22 | 18604438 | 18604438 | Missense_Mutation | SNP | G | G | A | novel | Somatic | V66I | 0.118812 | NM_018943.3 | 101 | 89 | 12 |
| 348--PTdeep | MAGEB5 | 785 | hg19 | chrX | 26235461 | 26235461 | Missense_Mutation | SNP | G | G | A | novel | Somatic | A15T | 0.25 | NM_001271752.1 | 48 | 36 | 12 |
| 348--PTdeep | IL1RAPL1 | 12 | hg19 | chrX | 29973260 | 29973260 | Missense_Mutation | SNP | C | C | T | novel | Somatic | R472W | 0.241379 | NM_014271.4 | 58 | 44 | 14 |
| 348--PTdeep | RBM10 | 943 | hg19 | chrX | 47030585 | 47030585 | Missense_Mutation | SNP | T | T | G | novel | Somatic | D120E | 0.050193 | NM_005676.5 | 250 | 237 | 13 |
| 348--LNmet | MORN1 | 602 | hg19 | chr1 | 2305899 | 2305899 | Splice_Site | SNP | C | C | T | novel | Somatic |  | 0.086643 | NM_024848.3 | 277 | 253 | 24 |
| 348--LNmet | CHD5 | 632 | hg19 | chr1 | 6206795 | 6206795 | Missense_Mutation | SNP | G | G | C | novel | Somatic | P507R | 0.061425 | NM_015557.3 | 406 | 381 | 25 |
| 348--LNmet | DISP3 | 673 | hg19 | chr1 | 11580878 | 11580878 | Missense_Mutation | SNP | G | G | A | novel | Somatic | E779K | 0.065811 | NM_020780.2 | 623 | 582 | 41 |
| 348--LNmet | VPS13D | 84 | hg19 | chr1 | 12337738 | 12337738 | Missense_Mutation | SNP | C | C | G | novel | Somatic | L1365V | 0.054968 | NM_015378.4 | 472 | 446 | 26 |
| 348--LNmet | OSCP1 | 866 | hg19 | chr1 | 36888394 | 36888394 | Nonsense_Mutation | SNP | G | G | A | novel | Somatic | R242Ter | 0.044231 | NM_145047.5 | 518 | 495 | 23 |
| 348--LNmet | WLS | 138 | hg19 | chr1 | 68624837 | 68624837 | Missense_Mutation | SNP | C | C | T | novel | Somatic | R158Q | 0.048035 | NM_024911.7 | 458 | 436 | 22 |
| 348--LNmet | SLC44A5 | 145 | hg19 | chr1 | 75699753 | 75699753 | Missense_Mutation | SNP | C | C | A | novel | Somatic | W257C | 0.044369 | NM_152697.5 | 585 | 559 | 26 |
| 348--LNmet | ZZZ3 | 147 | hg19 | chr1 | 78098624 | 78098624 | Nonsense_Mutation | SNP | G | G | C | novel | Somatic | S139Ter | 0.115619 | NM_015534.6 | 492 | 435 | 57 |
| 348--LNmet | LRIF1 | 1435 | hg19 | chr1 | 111494839 | 111494839 | Missense_Mutation | SNP | C | C | A | novel | Somatic | A223S | 0.060475 | NM_018372.4 | 462 | 434 | 28 |
| 348--LNmet | FLG | 1746 | hg19 | chr1 | 152286202 | 152286202 | Missense_Mutation | DNP | CC | CC | TG | novel | Somatic | G387H | 0.028942 | NM_002016.2 | 988 | 959 | 29 |
| 348--LNmet | IQGAP3 | 222 | hg19 | chr1 | 156504298 | 156504298 | Missense_Mutation | SNP | C | C | A | novel | Somatic | K1245N | 0.046205 | NM_178229.5 | 303 | 289 | 14 |
| 348--LNmet | ARHGEF11 | 1782 | hg19 | chr1 | 156926326 | 156926326 | Nonsense_Mutation | SNP | G | G | T | novel | Somatic | Y519Ter | 0.043956 | NM_198236.3 | 546 | 522 | 24 |
| 348--LNmet | GPR161 | 1867 | hg19 | chr1 | 168066228 | 168066228 | Frame_Shift_Ins | INS | - | - | CCAG | novel | Somatic | V226AfsTer89 | 0.063218 | NM_001267609.1 | 345 | 323 | 22 |
| 348--LNmet | F5 | 1878 | hg19 | chr1 | 169487772 | 169487772 | Missense_Mutation | SNP | C | C | T | novel | Somatic | G2075R | 0.053012 | NM_000130.5 | 414 | 392 | 22 |
| 348--LNmet | NAV1 | 265 | hg19 | chr1 | 201687796 | 201687796 | Missense_Mutation | SNP | C | C | T | novel | Somatic | S380L | 0.13176 | NM_020443.4 | 1015 | 881 | 134 |
| 348--LNmet | C1orf116 | 2165 | hg19 | chr1 | 207200850 | 207200850 | Missense_Mutation | SNP | G | G | A | novel | Somatic | R32C | 0.038647 | NM_023938.6 | 827 | 795 | 32 |
| 348--LNmet | KCNH1 | 274 | hg19 | chr1 | 211307265 | 211307265 | Missense_Mutation | SNP | C | C | T | novel | Somatic | R8K | 0.036078 | NM_172362.3 | 1079 | 1040 | 39 |
| 348--LNmet | EXOC8 | 293 | hg19 | chr1 | 231472927 | 231472927 | Missense_Mutation | SNP | G | G | C | novel | Somatic | L189V | 0.046875 | NM_175876.5 | 639 | 609 | 30 |
| 348--LNmet | FBXO11 | 118 | hg19 | chr2 | 48036767 | 48036767 | Missense_Mutation | SNP | T | T | A | novel | Somatic | L806F | 0.068966 | NM_001190274.1 | 348 | 324 | 24 |
| 348--LNmet | GCFC2 | 1164 | hg19 | chr2 | 75921501 | 75921501 | Missense_Mutation | SNP | A | A | T | novel | Somatic | Y296N | 0.081784 | NM_003203.5 | 269 | 247 | 22 |
| 348--LNmet | NCKAP5 | 0 | hg19 | chr2 | 133489543 | 133489543 | Missense_Mutation | SNP | C | C | T | novel | Somatic | R1737H | 0.04931 | NM_207363.3 | 507 | 482 | 25 |
| 348--LNmet | ACVR2A | 214 | hg19 | chr2 | 148683685 | 148683685 | Frame_Shift_Ins | INS | - | - | A | novel | Somatic | R438EfsTer19 | 0.026087 | NM_001616.5 | 460 | 448 | 12 |
| 348--LNmet | PLA2R1 | 226 | hg19 | chr2 | 160801441 | 160801441 | Frame_Shift_Ins | INS | - | - | T | novel | Somatic | G1374RfsTer5 | 0.043478 | NM_007366.5 | 367 | 351 | 16 |
| 348--LNmet | TTN | 1955 | hg19 | chr2 | 179584803 | 179584803 | Missense_Mutation | SNP | C | C | A | novel | Somatic | G7856W | 0.04119 | NM_001267550.2 | 435 | 417 | 18 |
| 348--LNmet | TNNC1 | 985 | hg19 | chr3 | 52485532 | 52485532 | Missense_Mutation | SNP | C | C | A | novel | Somatic | G110V | 0.060294 | NM_003280.3 | 679 | 638 | 41 |
| 348--LNmet | CCDC191 | 181 | hg19 | chr3 | 113724522 | 113724522 | Missense_Mutation | SNP | C | C | A | novel | Somatic | K567N | 0.063063 | NM_020817.2 | 551 | 516 | 35 |
| 348--LNmet | PIK3CA | 243 | hg19 | chr3 | 178952085 | 178952085 | Missense_Mutation | SNP | A | A | G | novel | Somatic | H1047R | 0.104545 | NM_006218.4 | 440 | 394 | 46 |
| 348--LNmet | LDB2 | 1 | hg19 | chr4 | 16504481 | 16504481 | Missense_Mutation | SNP | C | C | T | novel | Somatic | G303R | 0.050691 | NM_001290.5 | 434 | 412 | 22 |
| 348--LNmet | REST | 1025 | hg19 | chr4 | 57797575 | 57797575 | Missense_Mutation | SNP | C | C | G | novel | Somatic | L851V | 0.03752 | NM_005612.5 | 612 | 589 | 23 |
| 348--LNmet | HPSE | 1227 | hg19 | chr4 | 84223307 | 84223307 | Missense_Mutation | SNP | C | C | A | novel | Somatic | D441Y | 0.055319 | NM_006665.5 | 235 | 222 | 13 |
| 348--LNmet | WDFY3 | 154 | hg19 | chr4 | 85609253 | 85609253 | Missense_Mutation | SNP | T | T | C | novel | Somatic | I3177V | 0.032419 | NM_014991.5 | 401 | 388 | 13 |
| 348--LNmet | INTU | 195 | hg19 | chr4 | 128635181 | 128635181 | Missense_Mutation | SNP | G | G | C | novel | Somatic | E884Q | 0.053191 | NM_015693.4 | 469 | 444 | 25 |
| 348--LNmet | WDR17 | 241 | hg19 | chr4 | 177049909 | 177049909 | Missense_Mutation | SNP | C | C | T | novel | Somatic | R295C | 0.060748 | NM_170710.5 | 427 | 401 | 26 |
| 348--LNmet | NIM1K | 114 | hg19 | chr5 | 43280334 | 43280334 | Missense_Mutation | SNP | C | C | T | novel | Somatic | R272W | 0.064024 | NM_153361.4 | 325 | 304 | 21 |
| 348--LNmet | MTRNR2L2 | 1194 | hg19 | chr5 | 79945871 | 79945871 | Missense_Mutation | SNP | G | G | A | novel | Somatic | S12L | 0.112202 | NM_001190470.2 | 713 | 633 | 80 |
| 348--LNmet | ADAMTS19 | 24 | hg19 | chr5 | 128844868 | 128844868 | Missense_Mutation | SNP | A | A | C | novel | Somatic | K282N | 0.030023 | NM_133638.5 | 433 | 420 | 13 |
| 348--LNmet | PCDHB6 | 1657 | hg19 | chr5 | 140531735 | 140531735 | Missense_Mutation | SNP | G | G | A | novel | Somatic | A633T | 0.03608 | NM_018939.3 | 5369 | 5175 | 194 |
| 348--LNmet | HIST1H2AK | 797 | hg19 | chr6 | 27806095 | 27806095 | Missense_Mutation | SNP | C | C | G | novel | Somatic | G8A | 0.039916 | NM_003510.2 | 476 | 457 | 19 |
| 348--LNmet | GLP1R | 882 | hg19 | chr6 | 39040774 | 39040774 | Missense_Mutation | SNP | G | G | T | novel | Somatic | G216W | 0.065672 | NM_002062.5 | 335 | 313 | 22 |
| 348--LNmet | CUL7 | 913 | hg19 | chr6 | 43011227 | 43011227 | Missense_Mutation | SNP | G | G | A | novel | Somatic | P1105L | 0.029297 | NM_014780.4 | 511 | 496 | 15 |
| 348--LNmet | RAB23 | 1020 | hg19 | chr6 | 57059614 | 57059614 | Missense_Mutation | SNP | T | T | G | novel | Somatic | L145F | 0.064576 | NM_016277.5 | 541 | 506 | 35 |
| 348--LNmet | PHACTR2 | 210 | hg19 | chr6 | 144081695 | 144081695 | Frame_Shift_Ins | INS | - | - | A | novel | Somatic | N207KfsTer7 | 0.033248 | NM_001100164.2 | 390 | 377 | 13 |
| 348--LNmet | CCDC129 | 826 | hg19 | chr7 | 31614312 | 31614312 | Missense_Mutation | SNP | A | A | G | novel | Somatic | E211G | 0.041056 | NM_001257968.2 | 341 | 327 | 14 |
| 348--LNmet | CALCR | 161 | hg19 | chr7 | 93065415 | 93065415 | Missense_Mutation | SNP | G | G | A | novel | Somatic | A333V | 0.04936 | NM_001742.4 | 543 | 516 | 27 |
| 348--LNmet | LAMB4 | 175 | hg19 | chr7 | 107738938 | 107738938 | Missense_Mutation | SNP | G | G | A | novel | Somatic | L424F | 0.031792 | NM_007356.3 | 346 | 335 | 11 |
| 348--LNmet | PRAG1 | 647 | hg19 | chr8 | 8235405 | 8235405 | Missense_Mutation | SNP | C | C | T | novel | Somatic | E172K | 0.022222 | NM_001080826.3 | 495 | 484 | 11 |
| 348--LNmet | RB1CC1 | 124 | hg19 | chr8 | 53568744 | 53568744 | Missense_Mutation | SNP | G | G | C | novel | Somatic | D1215E | 0.051702 | NM_014781.5 | 791 | 750 | 41 |
| 348--LNmet | DCSTAMP | 1388 | hg19 | chr8 | 105361698 | 105361698 | Missense_Mutation | SNP | C | C | G | novel | Somatic | I306M | 0.06383 | NM_030788.4 | 423 | 396 | 27 |
| 348--LNmet | FAM135B | 25 | hg19 | chr8 | 139165041 | 139165041 | Missense_Mutation | SNP | G | G | C | novel | Somatic | S559R | 0.06789 | NM_015912.4 | 542 | 505 | 37 |
| 348--LNmet | ADAMTSL1 | 90 | hg19 | chr9 | 18680350 | 18680350 | Frame_Shift_Del | DEL | G | G | - | novel | Somatic | G395AfsTer56 | 0.028953 | NM_001040272.6 | 449 | 436 | 13 |
| 348--LNmet | PRRC2B | 201 | hg19 | chr9 | 134358148 | 134358148 | Missense_Mutation | SNP | G | G | A | novel | Somatic | G1743S | 0.123737 | NM_013318.3 | 395 | 346 | 49 |
| 348--LNmet | CTNNA3 | 17 | hg19 | chr10 | 67680249 | 67680249 | Missense_Mutation | SNP | G | G | T | novel | Somatic | H843N | 0.038983 | NM_013266.4 | 588 | 565 | 23 |
| 348--LNmet | DMBT1 | 191 | hg19 | chr10 | 124395547 | 124395547 | Missense_Mutation | SNP | G | G | A | novel | Somatic | D2068N | 0.050633 | NM_007329.2 | 552 | 524 | 28 |
| 348--LNmet | NAV2 | 92 | hg19 | chr11 | 19735269 | 19735269 | Missense_Mutation | SNP | A | A | G | novel | Somatic | M10V | 0.055948 | NM_145117.5 | 1984 | 1873 | 111 |
| 348--LNmet | DBX1 | 738 | hg19 | chr11 | 20181734 | 20181734 | Missense_Mutation | SNP | C | C | T | novel | Somatic | R46H | 0.037352 | NM_001029865.4 | 2698 | 2597 | 101 |
| 348--LNmet | NELL1 | 11 | hg19 | chr11 | 21135197 | 21135197 | Missense_Mutation | SNP | C | C | T | novel | Somatic | P455S | 0.061404 | NM_006157.5 | 341 | 320 | 21 |
| 348--LNmet | LUZP2 | 96 | hg19 | chr11 | 25100135 | 25100135 | Frame_Shift_Ins | INS | - | - | A | novel | Somatic | A327SfsTer10 | 0.046154 | NM_001009909.4 | 584 | 557 | 27 |
| 348--LNmet | OR5A1 | 1036 | hg19 | chr11 | 59211095 | 59211095 | Missense_Mutation | SNP | T | T | A | novel | Somatic | Y152N | 0.070922 | NM_001004728.1 | 423 | 393 | 30 |
| 348--LNmet | SLC22A10 | 1066 | hg19 | chr11 | 63069924 | 63069924 | Missense_Mutation | SNP | G | G | C | novel | Somatic | L398F | 0.050992 | NM_001039752.4 | 353 | 335 | 18 |
| 348--LNmet | SNX32 | 1085 | hg19 | chr11 | 65617620 | 65617620 | Splice_Site | SNP | G | G | C | novel | Somatic |  | 0.033613 | NM_152760.3 | 594 | 574 | 20 |
| 348--LNmet | OR8B12 | 1534 | hg19 | chr11 | 124412656 | 124412656 | Missense_Mutation | SNP | C | C | A | novel | Somatic | A299S | 0.056716 | NM_001005195.1 | 334 | 315 | 19 |
| 348--LNmet | LTBR | 634 | hg19 | chr12 | 6499462 | 6499462 | Missense_Mutation | SNP | G | G | A | novel | Somatic | R329K | 0.045649 | NM_002342.3 | 701 | 669 | 32 |
| 348--LNmet | SYT10 | 1 | hg19 | chr12 | 33579333 | 33579333 | Nonsense_Mutation | SNP | G | G | T | novel | Somatic | C83Ter | 0.040153 | NM_198992.4 | 521 | 500 | 21 |
| 348--LNmet | DIP2B | 121 | hg19 | chr12 | 51125183 | 51125183 | Missense_Mutation | SNP | C | C | T | novel | Somatic | P1225S | 0.045455 | NM_173602.3 | 637 | 608 | 29 |
| 348--LNmet | TPH2 | 17 | hg19 | chr12 | 72425090 | 72425090 | Missense_Mutation | SNP | G | G | T | novel | Somatic | C406F | 0.067797 | NM_173353.4 | 469 | 437 | 32 |
| 348--LNmet | SYT1 | 18 | hg19 | chr12 | 79842792 | 79842792 | Missense_Mutation | SNP | C | C | T | novel | Somatic | A386V | 0.040107 | NM_005639.3 | 373 | 358 | 15 |
| 348--LNmet | MYF6 | 1203 | hg19 | chr12 | 81102337 | 81102337 | Missense_Mutation | SNP | C | C | A | novel | Somatic | S185Y | 0.081042 | NM_002469.3 | 690 | 634 | 56 |
| 348--LNmet | ANO4 | 169 | hg19 | chr12 | 101520711 | 101520711 | Missense_Mutation | SNP | A | A | G | novel | Somatic | K911E | 0.052301 | NM_001286615.2 | 478 | 453 | 25 |
| 348--LNmet | DTX1 | 181 | hg19 | chr12 | 113515603 | 113515603 | Missense_Mutation | SNP | C | C | T | novel | Somatic | R212C | 0.025329 | NM_004416.2 | 2958 | 2883 | 75 |
| 348--LNmet | HS6ST3 | 165 | hg19 | chr13 | 97485053 | 97485053 | Missense_Mutation | SNP | G | G | C | novel | Somatic | Q339H | 0.037464 | NM_153456.3 | 346 | 333 | 13 |
| 348--LNmet | COCH | 824 | hg19 | chr14 | 31348048 | 31348048 | Nonsense_Mutation | SNP | C | C | T | novel | Somatic | R91Ter | 0.025641 | NM_004086.3 | 467 | 455 | 12 |
| 348--LNmet | BDKRB1 | 1322 | hg19 | chr14 | 96730335 | 96730335 | Missense_Mutation | SNP | G | G | A | novel | Somatic | G106R | 0.040598 | NM_000710.4 | 467 | 448 | 19 |
| 348--LNmet | SPATA5L1 | 933 | hg19 | chr15 | 45697614 | 45697614 | Missense_Mutation | SNP | C | C | G | novel | Somatic | L396V | 0.034483 | NM_024063.3 | 377 | 364 | 13 |
| 348--LNmet | KBTBD13 | 1083 | hg19 | chr15 | 65369982 | 65369982 | Missense_Mutation | SNP | C | C | T | novel | Somatic | R277C | 0.054962 | NM_001101362.2 | 3174 | 2999 | 175 |
| 348--LNmet | SRRM2 | 606 | hg19 | chr16 | 2819233 | 2819233 | Missense_Mutation | SNP | C | C | G | novel | Somatic | Q2657E | 0.03796 | NM_016333.4 | 840 | 808 | 32 |
| 348--LNmet | PRKCB | 11 | hg19 | chr16 | 24192159 | 24192159 | Missense_Mutation | SNP | G | G | C | novel | Somatic | K481N | 0.046154 | NM_002738.7 | 454 | 433 | 21 |
| 348--LNmet | PRRT2 | 812 | hg19 | chr16 | 29825016 | 29825016 | Frame_Shift_Del | DEL | C | C | - | novel | Somatic | R217EfsTer12 | 0.049065 | NM_145239.3 | 421 | 400 | 21 |
| 348--LNmet | ZNF629 | 819 | hg19 | chr16 | 30793308 | 30793308 | Missense_Mutation | SNP | C | C | T | novel | Somatic | V781M | 0.102823 | NM_001080417.3 | 992 | 890 | 102 |
| 348--LNmet | HSD3B7 | 821 | hg19 | chr16 | 30997511 | 30997511 | Missense_Mutation | SNP | A | A | G | novel | Somatic | E103G | 0.043764 | NM_025193.4 | 457 | 437 | 20 |
| 348--LNmet | PABPN1L | 1263 | hg19 | chr16 | 88931506 | 88931506 | Missense_Mutation | SNP | C | C | T | novel | Somatic | E164K | 0.061947 | NM_001080487.2 | 1016 | 953 | 63 |
| 348--LNmet | OR3A1 | 609 | hg19 | chr17 | 3195728 | 3195728 | Missense_Mutation | SNP | G | G | T | novel | Somatic | A50E | 0.059102 | NM_002550.2 | 423 | 398 | 25 |
| 348--LNmet | TP53 | 642 | hg19 | chr17 | 7577556 | 7577556 | Missense_Mutation | SNP | C | C | A | novel | Somatic | C242F | 0.13215 | NM_000546.5 | 505 | 438 | 67 |
| 348--LNmet | MYO15A | 722 | hg19 | chr17 | 18045547 | 18045547 | Missense_Mutation | SNP | G | G | C | novel | Somatic | R1935P | 0.081818 | NM_016239.4 | 438 | 402 | 36 |
| 348--LNmet | FLOT2 | 792 | hg19 | chr17 | 27211287 | 27211287 | Missense_Mutation | SNP | C | C | G | novel | Somatic | E60Q | 0.031818 | NM_004475.3 | 439 | 425 | 14 |
| 348--LNmet | NF1 | 101 | hg19 | chr17 | 29576048 | 29576048 | Nonsense_Mutation | SNP | C | C | T | novel | Somatic | Q1341Ter | 0.049213 | NM_001042492.3 | 508 | 483 | 25 |
| 348--LNmet | QRICH2 | 1151 | hg19 | chr17 | 74288760 | 74288760 | Missense_Mutation | SNP | G | G | C | novel | Somatic | P517R | 0.030361 | NM_032134.2 | 526 | 510 | 16 |
| 348--LNmet | DNAH17 | 18 | hg19 | chr17 | 76450732 | 76450732 | Missense_Mutation | SNP | G | G | A | novel | Somatic | A3409V | 0.058647 | NM_173628.4 | 660 | 621 | 39 |
| 348--LNmet | ZNF750 | 1201 | hg19 | chr17 | 80790311 | 80790311 | Missense_Mutation | SNP | C | C | T | novel | Somatic | R7Q | 0.037855 | NM_024702.3 | 317 | 305 | 12 |
| 348--LNmet | DSG1 | 805 | hg19 | chr18 | 28914059 | 28914059 | Nonsense_Mutation | SNP | C | C | G | novel | Somatic | S300Ter | 0.06135 | NM_001942.4 | 325 | 305 | 20 |
| 348--LNmet | MYO5B | 118 | hg19 | chr18 | 47352977 | 47352977 | Missense_Mutation | SNP | C | C | T | novel | Somatic | R1804Q | 0.035484 | NM_001080467.3 | 618 | 596 | 22 |
| 348--LNmet | MEX3C | 956 | hg19 | chr18 | 48703671 | 48703671 | Missense_Mutation | SNP | G | G | T | novel | Somatic | P344T | 0.042991 | NM_016626.5 | 533 | 510 | 23 |
| 348--LNmet | CDH7 | 133 | hg19 | chr18 | 63477114 | 63477114 | Missense_Mutation | SNP | A | A | T | novel | Somatic | T129S | 0.054662 | NM_001362438.2 | 311 | 294 | 17 |
| 348--LNmet | CD226 | 1100 | hg19 | chr18 | 67563011 | 67563011 | Missense_Mutation | SNP | C | C | A | novel | Somatic | G218V | 0.068592 | NM_001303618.2 | 276 | 257 | 19 |
| 348--LNmet | NETO1 | 140 | hg19 | chr18 | 70526241 | 70526241 | Missense_Mutation | SNP | T | T | C | novel | Somatic | K97E | 0.052632 | NM_138966.5 | 303 | 287 | 16 |
| 348--LNmet | ZBTB7A | 76 | hg19 | chr19 | 4054999 | 4054999 | Missense_Mutation | SNP | C | C | T | novel | Somatic | D78N | 0.05557 | NM_015898.4 | 3952 | 3732 | 220 |
| 348--LNmet | RGL3 | 672 | hg19 | chr19 | 11526634 | 11526634 | Missense_Mutation | SNP | C | C | T | novel | Somatic | E206K | 0.05395 | NM_001161616.3 | 519 | 491 | 28 |
| 348--LNmet | ZNF564 | 681 | hg19 | chr19 | 12637453 | 12637453 | Missense_Mutation | SNP | C | C | A | novel | Somatic | S490I | 0.057192 | NM_144976.4 | 577 | 544 | 33 |
| 348--LNmet | OR10H4 | 707 | hg19 | chr19 | 16060572 | 16060572 | Missense_Mutation | SNP | C | C | T | novel | Somatic | T252M | 0.022779 | NM_001004465.1 | 439 | 429 | 10 |
| 348--LNmet | ZNF429 | 750 | hg19 | chr19 | 21720557 | 21720557 | Missense_Mutation | SNP | A | A | G | novel | Somatic | K568E | 0.06746 | NM_001001415.4 | 1759 | 1640 | 119 |
| 348--LNmet | ZNF536 | 102 | hg19 | chr19 | 30934847 | 30934847 | Missense_Mutation | SNP | G | G | C | novel | Somatic | K126N | 0.051937 | NM_014717.3 | 2346 | 2224 | 122 |
| 348--LNmet | IGFLR1 | 861 | hg19 | chr19 | 36230697 | 36230697 | Missense_Mutation | SNP | G | G | T | novel | Somatic | P212H | 0.040134 | NM_024660.4 | 598 | 574 | 24 |
| 348--LNmet | SLC8A2 | 118 | hg19 | chr19 | 47960617 | 47960617 | Nonsense_Mutation | SNP | C | C | A | novel | Somatic | E304Ter | 0.067041 | NM_015063.3 | 2846 | 2655 | 191 |
| 348--LNmet | SLC8A2 | 118 | hg19 | chr19 | 47960704 | 47960704 | Missense_Mutation | SNP | C | C | T | novel | Somatic | D275N | 0.052778 | NM_015063.3 | 2156 | 2042 | 114 |
| 348--LNmet | SIGLEC12 | 981 | hg19 | chr19 | 52000614 | 52000614 | Silent | SNP | G | G | T | novel | Somatic | I497= | 0.058586 | NM_053003.4 | 495 | 466 | 29 |
| 348--LNmet | ZNF28 | 991 | hg19 | chr19 | 53303933 | 53303933 | Missense_Mutation | SNP | C | C | T | novel | Somatic | E389K | 0.041372 | NM_006969.5 | 987 | 946 | 41 |
| 348--LNmet | MBOAT7 | 1002 | hg19 | chr19 | 54678007 | 54678007 | Missense_Mutation | SNP | G | G | A | novel | Somatic | R384W | 0.036995 | NM_024298.5 | 1752 | 1687 | 65 |
| 348--LNmet | PAX1 | 750 | hg19 | chr20 | 21687108 | 21687108 | Missense_Mutation | SNP | G | G | A | novel | Somatic | G107S | 0.050336 | NM_006192.5 | 1191 | 1131 | 60 |
| 348--LNmet | ASXL1 | 821 | hg19 | chr20 | 31022441 | 31022441 | Frame_Shift_Del | DEL | A | A | - | novel | Somatic | G645VfsTer58 | 0.057294 | NM_015338.6 | 1821 | 1713 | 108 |
| 348--LNmet | ACTL10 | 831 | hg19 | chr20 | 32255361 | 32255361 | Missense_Mutation | SNP | G | G | A | novel | Somatic | V20M | 0.026224 | NM_001024675.2 | 4002 | 3897 | 105 |
| 348--LNmet | CTCFL | 126 | hg19 | chr20 | 56094367 | 56094367 | Missense_Mutation | SNP | C | C | T | novel | Somatic | R274H | 0.049275 | NM_001269043.1 | 345 | 328 | 17 |
| 348--LNmet | ZNF831 | 128 | hg19 | chr20 | 57828149 | 57828149 | Missense_Mutation | SNP | A | A | G | novel | Somatic | T1382A | 0.024948 | NM_178457.2 | 481 | 469 | 12 |
| 348--LNmet | SYCP2 | 128 | hg19 | chr20 | 58467314 | 58467314 | Missense_Mutation | SNP | G | G | T | novel | Somatic | H699N | 0.067485 | NM_014258.4 | 489 | 456 | 33 |
| 348--LNmet | PRPF6 | 132 | hg19 | chr20 | 62654231 | 62654231 | Missense_Mutation | SNP | G | G | A | novel | Somatic | R590Q | 0.043178 | NM_012469.4 | 578 | 553 | 25 |
| 348--LNmet | CECR2 | 90 | hg19 | chr22 | 18028845 | 18028845 | Missense_Mutation | SNP | C | C | A | novel | Somatic | A1398D | 0.051685 | NM_001290047.2 | 443 | 420 | 23 |
| 348--LNmet | ARFGAP3 | 914 | hg19 | chr22 | 43213780 | 43213780 | Frame_Shift_Del | DEL | T | T | - | novel | Somatic | N299MfsTer22 | 0.022727 | NM_014570.5 | 614 | 600 | 14 |
| 348--LNmet | MAGEB6 | 784 | hg19 | chrX | 26212535 | 26212535 | Missense_Mutation | SNP | G | G | A | novel | Somatic | S191N | 0.115741 | NM_173523.2 | 216 | 191 | 25 |
| 348--LNmet | MAGEB5 | 785 | hg19 | chrX | 26235461 | 26235461 | Missense_Mutation | SNP | G | G | A | novel | Somatic | A15T | 0.07732 | NM_001271752.1 | 193 | 178 | 15 |
| 348--LNmet | RBM10 | 943 | hg19 | chrX | 47030585 | 47030585 | Missense_Mutation | SNP | T | T | G | novel | Somatic | D120E | 0.05095 | NM_005676.5 | 1145 | 1086 | 59 |
| 435-PTsup | MST1L | 715 | hg19 | chr1 | 17084347 | 17084347 | Missense_Mutation | SNP | G | G | C | novel | Somatic | T557S | 0.021948 | NM_001271733.1 | 2592 | 2535 | 57 |
| 435-PTsup | IFNLR1 | 96 | hg19 | chr1 | 24513708 | 24513708 | Missense_Mutation | SNP | G | G | A | novel | Somatic | L11F | 0.092697 | NM_170743.4 | 711 | 645 | 66 |
| 435-PTsup | PHC2 | 842 | hg19 | chr1 | 33799853 | 33799853 | Missense_Mutation | SNP | G | G | T | novel | Somatic | S532R | 0.120567 | NM_198040.2 | 423 | 372 | 51 |
| 435-PTsup | ZSCAN20 | 105 | hg19 | chr1 | 33960310 | 33960310 | Missense_Mutation | SNP | C | C | T | novel | Somatic | T789M | 0.04486 | NM_145238.4 | 534 | 510 | 24 |
| 435-PTsup | ATP1A1 | 1477 | hg19 | chr1 | 116932312 | 116932312 | Missense_Mutation | SNP | T | T | G | novel | Somatic | L336V | 0.118619 | NM_001160233.1 | 664 | 585 | 79 |
| 435-PTsup | GJA8 | 1709 | hg19 | chr1 | 147380773 | 147380773 | Missense_Mutation | SNP | G | G | A | novel | Somatic | G231R | 0.104822 | NM_005267.5 | 476 | 426 | 50 |
| 435-PTsup | CHRNB2 | 1764 | hg19 | chr1 | 154542030 | 154542030 | Missense_Mutation | SNP | T | T | C | novel | Somatic | S53P | 0.124197 | NM_000748.3 | 467 | 409 | 58 |
| 435-PTsup | EFNA1 | 1768 | hg19 | chr1 | 155106258 | 155106258 | Splice_Site | SNP | T | T | C | novel | Somatic |  | 0.160839 | NM_004428.3 | 568 | 476 | 92 |
| 435-PTsup | HCN3 | 1769 | hg19 | chr1 | 155254548 | 155254548 | Splice_Site | DEL | GGT | GGT | - | novel | Somatic |  | 0.115607 | NM_020897.3 | 346 | 306 | 40 |
| 435-PTsup | LEFTY1 | 2309 | hg19 | chr1 | 226074608 | 226074608 | Missense_Mutation | SNP | T | T | G | novel | Somatic | K307T | 0.09232 | NM_020997.4 | 2446 | 2220 | 226 |
| 435-PTsup | ARV1 | 2348 | hg19 | chr1 | 231132996 | 231132996 | Missense_Mutation | SNP | C | C | G | novel | Somatic | S268C | 0.155052 | NM_022786.3 | 574 | 485 | 89 |
| 435-PTsup | ZNF496 | 2473 | hg19 | chr1 | 247464275 | 247464275 | Missense_Mutation | SNP | C | C | T | novel | Somatic | C437Y | 0.090637 | NM_032752.3 | 2008 | 1826 | 182 |
| 435-PTsup | OR2M4 | 2480 | hg19 | chr1 | 248402295 | 248402295 | Missense_Mutation | SNP | C | C | T | novel | Somatic | P22L | 0.148936 | NM_017504.1 | 517 | 440 | 77 |
| 435-PTsup | SUPT7L | 797 | hg19 | chr2 | 27884121 | 27884121 | Missense_Mutation | SNP | G | G | A | novel | Somatic | P50L | 0.041314 | NM_014860.3 | 942 | 903 | 39 |
| 435-PTsup | WDR43 | 807 | hg19 | chr2 | 29158455 | 29158455 | Missense_Mutation | SNP | T | T | G | novel | Somatic | I502M | 0.053333 | NM_015131.3 | 375 | 355 | 20 |
| 435-PTsup | C2orf42 | 140 | hg19 | chr2 | 70377673 | 70377673 | Missense_Mutation | SNP | C | C | G | novel | Somatic | E514Q | 0.103053 | NM_001348758.1 | 261 | 234 | 27 |
| 435-PTsup | MYO3B | 236 | hg19 | chr2 | 171375987 | 171375987 | Missense_Mutation | SNP | C | C | T | novel | Somatic | A1171V | 0.099119 | NM_138995.5 | 452 | 407 | 45 |
| 435-PTsup | NFE2L2 | 242 | hg19 | chr2 | 178098815 | 178098815 | Missense_Mutation | SNP | T | T | A | novel | Somatic | D77V | 0.121094 | NM_006164.5 | 512 | 450 | 62 |
| 435-PTsup | HRH1 | 671 | hg19 | chr3 | 11301493 | 11301493 | Missense_Mutation | SNP | G | G | T | novel | Somatic | W257L | 0.139373 | NM_001098213.2 | 286 | 246 | 40 |
| 435-PTsup | MBD4 | 1570 | hg19 | chr3 | 129152818 | 129152818 | Missense_Mutation | SNP | G | G | A | novel | Somatic | P429L | 0.051948 | NM_003925.3 | 693 | 657 | 36 |
| 435-PTsup | B3GALNT1 | 1811 | hg19 | chr3 | 160803865 | 160803865 | Nonsense_Mutation | SNP | G | G | C | novel | Somatic | Y346Ter | 0.077731 | NM_001349162.1 | 473 | 436 | 37 |
| 435-PTsup | UGT2B7 | 1118 | hg19 | chr4 | 69962686 | 69962686 | Missense_Mutation | SNP | G | G | A | novel | Somatic | A150T | 0.10757 | NM_001074.4 | 498 | 444 | 54 |
| 435-PTsup | STPG2 | 20 | hg19 | chr4 | 98865102 | 98865102 | Missense_Mutation | SNP | G | G | C | novel | Somatic | N330K | 0.08316 | NM_174952.3 | 481 | 441 | 40 |
| 435-PTsup | DIAPH1 | 207 | hg19 | chr5 | 140903716 | 140903716 | Missense_Mutation | SNP | G | G | A | novel | Somatic | R1219C | 0.070866 | NM_005219.5 | 379 | 352 | 27 |
| 435-PTsup | TRIM41 | 1963 | hg19 | chr5 | 180661473 | 180661473 | Missense_Mutation | SNP | C | C | G | novel | Somatic | H531D | 0.043685 | NM_033549.5 | 2104 | 2012 | 92 |
| 435-PTsup | HIST1H2BH | 785 | hg19 | chr6 | 26252007 | 26252007 | Nonsense_Mutation | SNP | C | C | G | novel | Somatic | Y43Ter | 0.077864 | NM_003524.2 | 899 | 829 | 70 |
| 435-PTsup | TREML2 | 899 | hg19 | chr6 | 41160232 | 41160232 | Missense_Mutation | SNP | C | C | A | novel | Somatic | C300F | 0.046341 | NM_024807.4 | 407 | 388 | 19 |
| 435-PTsup | AARS2 | 922 | hg19 | chr6 | 44271982 | 44271982 | Missense_Mutation | SNP | G | G | T | novel | Somatic | P648H | 0.040842 | NM_020745.4 | 808 | 775 | 33 |
| 435-PTsup | TRAM2 | 15 | hg19 | chr6 | 52369400 | 52369400 | Missense_Mutation | SNP | T | T | G | novel | Somatic | K343T | 0.054085 | NM_012288.4 | 1737 | 1643 | 94 |
| 435-PTsup | TTLL2 | 1864 | hg19 | chr6 | 167753992 | 167753992 | Missense_Mutation | SNP | C | C | A | novel | Somatic | L202M | 0.053619 | NM_031949.5 | 370 | 350 | 20 |
| 435-PTsup | ZNF117 | 1076 | hg19 | chr7 | 64439383 | 64439383 | Missense_Mutation | SNP | T | T | C | novel | Somatic | E189G | 0.035714 | NM_015852.4 | 1090 | 1051 | 39 |
| 435-PTsup | CLCN1 | 1676 | hg19 | chr7 | 143042675 | 143042675 | Missense_Mutation | SNP | C | C | G | novel | Somatic | H664Q | 0.113111 | NM_000083.3 | 1166 | 1034 | 132 |
| 435-PTsup | PKHD1L1 | 178 | hg19 | chr8 | 110448573 | 110448573 | Missense_Mutation | SNP | C | C | G | novel | Somatic | T1171S | 0.114723 | NM_177531.6 | 523 | 463 | 60 |
| 435-PTsup | PKHD1L1 | 178 | hg19 | chr8 | 110520435 | 110520435 | Missense_Mutation | SNP | A | A | T | novel | Somatic | E3779D | 0.145791 | NM_177531.6 | 487 | 416 | 71 |
| 435-PTsup | CSMD3 | 22 | hg19 | chr8 | 113504859 | 113504859 | Missense_Mutation | SNP | G | G | T | novel | Somatic | L1713I | 0.054201 | NM_198123.2 | 368 | 348 | 20 |
| 435-PTsup | PLEC | 1691 | hg19 | chr8 | 145000031 | 145000031 | Missense_Mutation | SNP | C | C | T | novel | Somatic | A1493T | 0.149265 | NM_201380.4 | 1360 | 1157 | 203 |
| 435-PTsup | KLHL9 | 747 | hg19 | chr9 | 21333271 | 21333271 | Missense_Mutation | SNP | C | C | T | novel | Somatic | A530T | 0.11335 | NM_018847.4 | 393 | 348 | 45 |
| 435-PTsup | TMEM8B | 858 | hg19 | chr9 | 35846584 | 35846584 | Nonsense_Mutation | INS | - | - | A | novel | Somatic | Y658Ter | 0.155272 | NM_001042590.4 | 1725 | 1457 | 268 |
| 435-PTsup | PGM5 | 140 | hg19 | chr9 | 71114243 | 71114243 | Missense_Mutation | SNP | A | A | T | novel | Somatic | E527V | 0.050505 | NM_021965.4 | 494 | 469 | 25 |
| 435-PTsup | NIPSNAP3A | 1405 | hg19 | chr9 | 107513323 | 107513323 | Missense_Mutation | SNP | G | G | A | novel | Somatic | M49I | 0.159329 | NM_015469.3 | 477 | 401 | 76 |
| 435-PTsup | IKBKAP | Unknown | hg19 | chr9 | 111693370 | 111693370 | Frame_Shift_Del | DEL | C | C | - | novel | Somatic | N20IfsTer22 | 0.117391 | NM_003640.5 | 460 | 406 | 54 |
| 435-PTsup | PDCD11 | 1387 | hg19 | chr10 | 105185096 | 105185096 | Missense_Mutation | SNP | C | C | T | novel | Somatic | T1040I | 0.07483 | NM_014976.2 | 294 | 272 | 22 |
| 435-PTsup | CFAP46 | 201 | hg19 | chr10 | 134699339 | 134699339 | Frame_Shift_Del | DEL | CC | CC | G | novel | Somatic | R1143TfsTer7 | 0.036969 | NM_001200049.3 | 1619 | 1559 | 60 |
| 435-PTsup | DDB2 | 945 | hg19 | chr11 | 47238418 | 47238418 | Nonsense_Mutation | SNP | C | C | T | novel | Somatic | Q92Ter | 0.071233 | NM_000107.2 | 365 | 339 | 26 |
| 435-PTsup | OR9G4 | 1016 | hg19 | chr11 | 56510878 | 56510878 | Missense_Mutation | SNP | C | C | T | novel | Somatic | R137H | 0.088028 | NM_001005284.1 | 282 | 257 | 25 |
| 435-PTsup | PPFIA1 | 139 | hg19 | chr11 | 70172459 | 70172459 | Missense_Mutation | SNP | C | C | T | novel | Somatic | T233M | 0.066815 | NM_003626.4 | 446 | 416 | 30 |
| 435-PTsup | CEP164 | 1479 | hg19 | chr11 | 117241956 | 117241956 | Missense_Mutation | SNP | G | G | T | novel | Somatic | G309V | 0.069307 | NM_014956.5 | 503 | 468 | 35 |
| 435-PTsup | MED21 | 792 | hg19 | chr12 | 27180351 | 27180351 | Missense_Mutation | SNP | C | C | G | novel | Somatic | P77A | 0.100559 | NM_004264.5 | 356 | 320 | 36 |
| 435-PTsup | OTOGL | 18 | hg19 | chr12 | 80696424 | 80696424 | Frame_Shift_Ins | INS | - | - | T | novel | Somatic | L1019SfsTer31 | 0.10181 | NM_173591.3 | 438 | 393 | 45 |
| 435-PTsup | ANAPC5 | 189 | hg19 | chr12 | 121766207 | 121766207 | Missense_Mutation | SNP | C | C | T | novel | Somatic | D406N | 0.109756 | NM_016237.5 | 488 | 434 | 54 |
| 435-PTsup | HIP1R | 190 | hg19 | chr12 | 123343622 | 123343622 | Missense_Mutation | SNP | T | T | G | novel | Somatic | C725G | 0.043624 | NM_003959.3 | 1191 | 1139 | 52 |
| 435-PTsup | ZNF891 | 1605 | hg19 | chr12 | 133697292 | 133697292 | Nonsense_Mutation | SNP | C | C | A | novel | Somatic | E405Ter | 0.102007 | NM_001277291.1 | 596 | 535 | 61 |
| 435-PTsup | ZFP36L1 | 1113 | hg19 | chr14 | 69256608 | 69256608 | Frame_Shift_Ins | INS | - | - | GCAGCCCG | novel | Somatic | L289PfsTer16 | 0.068817 | NM_001244701.1 | 2319 | 2159 | 160 |
| 435-PTsup | ZC3H14 | 1264 | hg19 | chr14 | 89076131 | 89076131 | Missense_Mutation | SNP | C | C | G | novel | Somatic | P694R | 0.044527 | NM_024824.5 | 535 | 511 | 24 |
| 435-PTsup | SERPINA9 | 1309 | hg19 | chr14 | 94933669 | 94933669 | Missense_Mutation | SNP | A | A | C | novel | Somatic | F227V | 0.049853 | NM_175739.4 | 340 | 323 | 17 |
| 435-PTsup | KIF26A | 1383 | hg19 | chr14 | 104618379 | 104618379 | Missense_Mutation | SNP | G | G | A | novel | Somatic | D106N | 0.097173 | NM_015656.1 | 1129 | 1019 | 110 |
| 435-PTsup | STARD9 | 14 | hg19 | chr15 | 42983821 | 42983821 | Missense_Mutation | SNP | G | G | T | novel | Somatic | D3349Y | 0.066406 | NM_020759.3 | 508 | 474 | 34 |
| 435-PTsup | CD276 | 1149 | hg19 | chr15 | 73996147 | 73996147 | Missense_Mutation | SNP | T | T | C | novel | Somatic | V294A | 0.05973 | NM_001024736.2 | 2074 | 1950 | 124 |
| 435-PTsup | SALL1 | 975 | hg19 | chr16 | 51174554 | 51174554 | Missense_Mutation | SNP | G | G | T | novel | Somatic | P527T | 0.060914 | NM_002968.3 | 590 | 554 | 36 |
| 435-PTsup | TOX3 | 123 | hg19 | chr16 | 52473235 | 52473235 | Missense_Mutation | SNP | G | G | A | novel | Somatic | P545S | 0.035608 | NM_001080430.4 | 1008 | 972 | 36 |
| 435-PTsup | MT1X | 1017 | hg19 | chr16 | 56716475 | 56716475 | Missense_Mutation | SNP | C | C | T | novel | Somatic | S8L | 0.055928 | NM_005952.4 | 893 | 843 | 50 |
| 435-PTsup | SGSM2 | 602 | hg19 | chr17 | 2274512 | 2274512 | Intron | DEL | GAAGGTGTCTCAGGCCCT | GAAGGTGTCTCAGGCCCT | - | novel | Somatic |  | 0.090435 | NM_014853.3 | 574 | 522 | 52 |
| 435-PTsup | TP53 | 642 | hg19 | chr17 | 7574003 | 7574003 | Nonsense_Mutation | SNP | G | G | A | novel | Somatic | R342Ter | 0.084507 | NM_000546.5 | 354 | 324 | 30 |
| 435-PTsup | TP53 | 642 | hg19 | chr17 | 7576851 | 7576851 | Splice_Site | SNP | A | A | G | novel | Somatic |  | 0.098507 | NM_000546.5 | 333 | 300 | 33 |
| 435-PTsup | HS3ST3B1 | 693 | hg19 | chr17 | 14204973 | 14204973 | Missense_Mutation | SNP | G | G | A | novel | Somatic | M46I | 0.106117 | NM_006041.3 | 801 | 716 | 85 |
| 435-PTsup | KRTAP1-1 | 884 | hg19 | chr17 | 39197634 | 39197634 | Missense_Mutation | SNP | T | T | C | novel | Somatic | T6A | 0.087766 | NM_030967.3 | 750 | 684 | 66 |
| 435-PTsup | CNTNAP1 | 896 | hg19 | chr17 | 40845339 | 40845339 | Missense_Mutation | SNP | C | C | T | novel | Somatic | P926L | 0.039514 | NM_003632.3 | 984 | 945 | 39 |
| 435-PTsup | HEATR6 | 1028 | hg19 | chr17 | 58149662 | 58149662 | Missense_Mutation | SNP | G | G | A | novel | Somatic | P205S | 0.098182 | NM_022070.5 | 275 | 248 | 27 |
| 435-PTsup | CEP95 | 132 | hg19 | chr17 | 62525545 | 62525545 | Missense_Mutation | SNP | G | G | T | novel | Somatic | Q482H | 0.090349 | NM_138363.3 | 483 | 439 | 44 |
| 435-PTsup | TXNL4A | 1178 | hg19 | chr18 | 77737679 | 77737679 | Missense_Mutation | SNP | T | T | C | novel | Somatic | Y59C | 0.123711 | NM_006701.5 | 291 | 255 | 36 |
| 435-PTsup | MUC16 | 81 | hg19 | chr19 | 9083751 | 9083751 | Missense_Mutation | SNP | C | C | T | novel | Somatic | M2688I | 0.168539 | NM_024690.2 | 178 | 148 | 30 |
| 435-PTsup | ZNF700 | 84 | hg19 | chr19 | 12060008 | 12060008 | Missense_Mutation | SNP | A | A | G | novel | Somatic | Y390C | 0.124567 | NM_144566.3 | 866 | 758 | 108 |
| 435-PTsup | ZNF429 | 750 | hg19 | chr19 | 21720557 | 21720557 | Missense_Mutation | SNP | A | A | G | novel | Somatic | K568E | 0.02238 | NM_001001415.4 | 983 | 961 | 22 |
| 435-PTsup | ZNF729 | 756 | hg19 | chr19 | 22496921 | 22496921 | Missense_Mutation | SNP | A | A | T | novel | Somatic | K234N | 0.09872 | NM_001242680.2 | 547 | 493 | 54 |
| 435-PTsup | ZNF229 | 927 | hg19 | chr19 | 44934516 | 44934516 | Missense_Mutation | SNP | G | G | T | novel | Somatic | S147Y | 0.103761 | NM_014518.4 | 769 | 689 | 80 |
| 435-PTsup | RTN2 | 935 | hg19 | chr19 | 45998252 | 45998252 | Missense_Mutation | SNP | C | C | A | novel | Somatic | D31Y | 0.072363 | NM_005619.5 | 1144 | 1061 | 83 |
| 435-PTsup | PRR12 | 967 | hg19 | chr19 | 50094915 | 50094915 | Missense_Mutation | SNP | G | G | T | novel | Somatic | D2Y | 0.046486 | NM_020719.3 | 919 | 876 | 43 |
| 435-PTsup | ZNF578 | 989 | hg19 | chr19 | 53014350 | 53014350 | Missense_Mutation | SNP | C | C | A | novel | Somatic | A239D | 0.067321 | NM_001099694.2 | 709 | 661 | 48 |
| 435-PTsup | ZNF582 | 1019 | hg19 | chr19 | 56895283 | 56895283 | Missense_Mutation | SNP | C | C | A | novel | Somatic | M532I | 0.085239 | NM_001320371.2 | 480 | 439 | 41 |
| 435-PTsup | PPP1R3D | 1031 | hg19 | chr20 | 58514167 | 58514167 | Missense_Mutation | SNP | C | C | T | novel | Somatic | D274N | 0.04084 | NM_006242.4 | 1708 | 1638 | 70 |
| 435-PTsup | AIRE | 933 | hg19 | chr21 | 45708294 | 45708294 | Missense_Mutation | SNP | C | C | G | novel | Somatic | A202G | 0.086331 | NM_000383.4 | 694 | 634 | 60 |
| 435-PTsup | TBC1D25 | 954 | hg19 | chrX | 48417372 | 48417372 | Missense_Mutation | SNP | C | C | T | novel | Somatic | T159M | 0.082067 | NM_002536.4 | 329 | 302 | 27 |
| 435-PTsup | NUDT10 | 974 | hg19 | chrX | 51076075 | 51076075 | Missense_Mutation | SNP | C | C | G | novel | Somatic | N86K | 0.193724 | NM_153183.3 | 732 | 590 | 142 |
| 435-PTsup | RBMXL3 | 182 | hg19 | chrX | 114426006 | 114426006 | Missense_Mutation | SNP | C | C | T | novel | Somatic | R668C | 0.177546 | NM_001145346.1 | 1529 | 1257 | 272 |
| 435-PTsup | PLXNB3 | 1752 | hg19 | chrX | 153042691 | 153042691 | Frame_Shift_Del | DEL | G | G | - | novel | Somatic | V1678CfsTer7 | 0.033133 | NM_001163257.1 | 663 | 641 | 22 |
| 435-PTdeep | MST1L | 715 | hg19 | chr1 | 17084347 | 17084347 | Missense_Mutation | SNP | G | G | C | novel | Somatic | T557S | 0.049765 | NM_001271733.1 | 2549 | 2422 | 127 |
| 435-PTdeep | KDM1A | 763 | hg19 | chr1 | 23346117 | 23346117 | In_Frame_Del | DEL | GCG | GCG | - | novel | Somatic | A18del | 0.02651 | NM_001009999.3 | 678 | 660 | 18 |
| 435-PTdeep | IFNLR1 | 96 | hg19 | chr1 | 24513708 | 24513708 | Missense_Mutation | SNP | G | G | A | novel | Somatic | L11F | 0.368421 | NM_170743.4 | 607 | 383 | 224 |
| 435-PTdeep | PHC2 | 842 | hg19 | chr1 | 33799853 | 33799853 | Missense_Mutation | SNP | G | G | T | novel | Somatic | S532R | 0.301587 | NM_198040.2 | 441 | 308 | 133 |
| 435-PTdeep | ZSCAN20 | 105 | hg19 | chr1 | 33960310 | 33960310 | Missense_Mutation | SNP | C | C | T | novel | Somatic | T789M | 0.180987 | NM_145238.4 | 544 | 445 | 99 |
| 435-PTdeep | CYP4Z1 | 118 | hg19 | chr1 | 47581260 | 47581260 | Missense_Mutation | SNP | C | C | G | novel | Somatic | P421A | 0.189616 | NM_178134.3 | 441 | 357 | 84 |
| 435-PTdeep | ATP1A1 | 1477 | hg19 | chr1 | 116932312 | 116932312 | Missense_Mutation | SNP | T | T | G | novel | Somatic | L336V | 0.345902 | NM_001160233.1 | 610 | 399 | 211 |
| 435-PTdeep | GJA8 | 1709 | hg19 | chr1 | 147380773 | 147380773 | Missense_Mutation | SNP | G | G | A | novel | Somatic | G231R | 0.146965 | NM_005267.5 | 624 | 532 | 92 |
| 435-PTdeep | CHRNB2 | 1764 | hg19 | chr1 | 154542030 | 154542030 | Missense_Mutation | SNP | T | T | C | novel | Somatic | S53P | 0.279938 | NM_000748.3 | 643 | 463 | 180 |
| 435-PTdeep | EFNA1 | 1768 | hg19 | chr1 | 155106258 | 155106258 | Splice_Site | SNP | T | T | C | novel | Somatic |  | 0.231156 | NM_004428.3 | 789 | 605 | 184 |
| 435-PTdeep | HCN3 | 1769 | hg19 | chr1 | 155254548 | 155254548 | Splice_Site | DEL | GGT | GGT | - | novel | Somatic |  | 0.206349 | NM_020897.3 | 503 | 399 | 104 |
| 435-PTdeep | LEFTY1 | 2309 | hg19 | chr1 | 226074608 | 226074608 | Missense_Mutation | SNP | T | T | G | novel | Somatic | K307T | 0.124512 | NM_020997.4 | 2816 | 2465 | 351 |
| 435-PTdeep | ARV1 | 2348 | hg19 | chr1 | 231132996 | 231132996 | Missense_Mutation | SNP | C | C | G | novel | Somatic | S268C | 0.204735 | NM_022786.3 | 716 | 569 | 147 |
| 435-PTdeep | ZNF496 | 2473 | hg19 | chr1 | 247464275 | 247464275 | Missense_Mutation | SNP | C | C | T | novel | Somatic | C437Y | 0.10733 | NM_032752.3 | 2289 | 2043 | 246 |
| 435-PTdeep | OR2M4 | 2480 | hg19 | chr1 | 248402295 | 248402295 | Missense_Mutation | SNP | C | C | T | novel | Somatic | P22L | 0.221239 | NM_017504.1 | 563 | 438 | 125 |
| 435-PTdeep | SUPT7L | 797 | hg19 | chr2 | 27884121 | 27884121 | Missense_Mutation | SNP | G | G | A | novel | Somatic | P50L | 0.189636 | NM_014860.3 | 906 | 734 | 172 |
| 435-PTdeep | WDR43 | 807 | hg19 | chr2 | 29158455 | 29158455 | Missense_Mutation | SNP | T | T | G | novel | Somatic | I502M | 0.177778 | NM_015131.3 | 405 | 333 | 72 |
| 435-PTdeep | KIAA1841 | 131 | hg19 | chr2 | 61343204 | 61343204 | Missense_Mutation | SNP | A | A | C | novel | Somatic | K612T | 0.09434 | NM_001129993.3 | 422 | 382 | 40 |
| 435-PTdeep | C2orf42 | 140 | hg19 | chr2 | 70377673 | 70377673 | Missense_Mutation | SNP | C | C | G | novel | Somatic | E514Q | 0.174757 | NM_001348758.1 | 308 | 254 | 54 |
| 435-PTdeep | MYO3B | 236 | hg19 | chr2 | 171375987 | 171375987 | Missense_Mutation | SNP | C | C | T | novel | Somatic | A1171V | 0.44382 | NM_138995.5 | 354 | 196 | 158 |
| 435-PTdeep | NFE2L2 | 242 | hg19 | chr2 | 178098815 | 178098815 | Missense_Mutation | SNP | T | T | A | novel | Somatic | D77V | 0.522248 | NM_006164.5 | 427 | 204 | 223 |
| 435-PTdeep | HRH1 | 671 | hg19 | chr3 | 11301493 | 11301493 | Missense_Mutation | SNP | G | G | T | novel | Somatic | W257L | 0.441964 | NM_001098213.2 | 224 | 125 | 99 |
| 435-PTdeep | SLC4A7 | 794 | hg19 | chr3 | 27478041 | 27478041 | Splice_Site | SNP | T | T | A | novel | Somatic |  | 0.162679 | NM_001321103.2 | 208 | 174 | 34 |
| 435-PTdeep | DLEC1 | 109 | hg19 | chr3 | 38139028 | 38139028 | Frame_Shift_Del | DEL | ACTTT | ACTTT | - | novel | Somatic | F823WfsTer14 | 0.089983 | NM_007337.3 | 589 | 536 | 53 |
| 435-PTdeep | BBX | 175 | hg19 | chr3 | 107517396 | 107517396 | Missense_Mutation | SNP | A | A | G | novel | Somatic | M794V | 0.077374 | NM_001142568.3 | 852 | 786 | 66 |
| 435-PTdeep | TMCC1 | 196 | hg19 | chr3 | 129547050 | 129547050 | Missense_Mutation | SNP | G | G | T | novel | Somatic | H58N | 0.089132 | NM_001349263.2 | 1275 | 1161 | 114 |
| 435-PTdeep | COPB2 | 1646 | hg19 | chr3 | 139097976 | 139097976 | Missense_Mutation | SNP | C | C | G | novel | Somatic | E90Q | 0.082927 | NM_004766.3 | 816 | 748 | 68 |
| 435-PTdeep | B3GALNT1 | 1811 | hg19 | chr3 | 160803865 | 160803865 | Nonsense_Mutation | SNP | G | G | C | novel | Somatic | Y346Ter | 0.103933 | NM_001349162.1 | 711 | 637 | 74 |
| 435-PTdeep | UGT2B7 | 1118 | hg19 | chr4 | 69962686 | 69962686 | Missense_Mutation | SNP | G | G | A | novel | Somatic | A150T | 0.28268 | NM_001074.4 | 608 | 435 | 173 |
| 435-PTdeep | STPG2 | 20 | hg19 | chr4 | 98865102 | 98865102 | Missense_Mutation | SNP | G | G | C | novel | Somatic | N330K | 0.22736 | NM_174952.3 | 519 | 401 | 118 |
| 435-PTdeep | IQGAP2 | 1164 | hg19 | chr5 | 75902129 | 75902129 | Splice_Site | SNP | G | G | T | novel | Somatic |  | 0.149007 | NM_006633.4 | 299 | 254 | 45 |
| 435-PTdeep | DIAPH1 | 207 | hg19 | chr5 | 140903716 | 140903716 | Missense_Mutation | SNP | G | G | A | novel | Somatic | R1219C | 0.12605 | NM_005219.5 | 355 | 310 | 45 |
| 435-PTdeep | HIST1H2BH | 785 | hg19 | chr6 | 26252007 | 26252007 | Nonsense_Mutation | SNP | C | C | G | novel | Somatic | Y43Ter | 0.224829 | NM_003524.2 | 1023 | 793 | 230 |
| 435-PTdeep | DNAH8 | 13 | hg19 | chr6 | 38980248 | 38980248 | Missense_Mutation | SNP | A | A | G | novel | Somatic | M4517V | 0.113872 | NM_001206927.2 | 482 | 427 | 55 |
| 435-PTdeep | KIF6 | 887 | hg19 | chr6 | 39607392 | 39607392 | Missense_Mutation | SNP | T | T | A | novel | Somatic | L131F | 0.120853 | NM_145027.6 | 420 | 369 | 51 |
| 435-PTdeep | TREML2 | 899 | hg19 | chr6 | 41160232 | 41160232 | Missense_Mutation | SNP | C | C | A | novel | Somatic | C300F | 0.110647 | NM_024807.4 | 477 | 424 | 53 |
| 435-PTdeep | AARS2 | 922 | hg19 | chr6 | 44271982 | 44271982 | Missense_Mutation | SNP | G | G | T | novel | Somatic | P648H | 0.12233 | NM_020745.4 | 1028 | 902 | 126 |
| 435-PTdeep | MEP1A | 117 | hg19 | chr6 | 46803179 | 46803179 | Frame_Shift_Del | DEL | CC | CC | A | novel | Somatic | L661WfsTer26 | 0.088859 | NM_005588.3 | 738 | 671 | 67 |
| 435-PTdeep | TRAM2 | 15 | hg19 | chr6 | 52369400 | 52369400 | Missense_Mutation | SNP | T | T | G | novel | Somatic | K343T | 0.111235 | NM_012288.4 | 1788 | 1589 | 199 |
| 435-PTdeep | TTLL2 | 1864 | hg19 | chr6 | 167753992 | 167753992 | Missense_Mutation | SNP | C | C | A | novel | Somatic | L202M | 0.200565 | NM_031949.5 | 352 | 281 | 71 |
| 435-PTdeep | AGBL3 | 201 | hg19 | chr7 | 134718867 | 134718867 | Intron | SNP | C | C | G | novel | Somatic |  | 0.331933 | NM_178563.4 | 238 | 159 | 79 |
| 435-PTdeep | CLCN1 | 1676 | hg19 | chr7 | 143042675 | 143042675 | Missense_Mutation | SNP | C | C | G | novel | Somatic | H664Q | 0.3897 | NM_000083.3 | 1164 | 710 | 454 |
| 435-PTdeep | PKHD1L1 | 178 | hg19 | chr8 | 110448573 | 110448573 | Missense_Mutation | SNP | C | C | G | novel | Somatic | T1171S | 0.346429 | NM_177531.6 | 559 | 365 | 194 |
| 435-PTdeep | PKHD1L1 | 178 | hg19 | chr8 | 110520435 | 110520435 | Missense_Mutation | SNP | A | A | T | novel | Somatic | E3779D | 0.370629 | NM_177531.6 | 572 | 360 | 212 |
| 435-PTdeep | CSMD3 | 22 | hg19 | chr8 | 113504859 | 113504859 | Missense_Mutation | SNP | G | G | T | novel | Somatic | L1713I | 0.077307 | NM_198123.2 | 401 | 370 | 31 |
| 435-PTdeep | KCNQ3 | 24 | hg19 | chr8 | 133150232 | 133150232 | Frame_Shift_Ins | INS | - | - | T | novel | Somatic | F534IfsTer15 | 0.050279 | NM_004519.4 | 535 | 508 | 27 |
| 435-PTdeep | PLEC | 1691 | hg19 | chr8 | 145000031 | 145000031 | Missense_Mutation | SNP | C | C | T | novel | Somatic | A1493T | 0.382883 | NM_201380.4 | 1331 | 821 | 510 |
| 435-PTdeep | KLHL9 | 747 | hg19 | chr9 | 21333271 | 21333271 | Missense_Mutation | SNP | C | C | T | novel | Somatic | A530T | 0.348195 | NM_018847.4 | 469 | 305 | 164 |
| 435-PTdeep | TMEM8B | 858 | hg19 | chr9 | 35846584 | 35846584 | Nonsense_Mutation | INS | - | - | A | novel | Somatic | Y658Ter | 0.344431 | NM_001042590.4 | 1643 | 1074 | 569 |
| 435-PTdeep | NIPSNAP3A | 1405 | hg19 | chr9 | 107513323 | 107513323 | Missense_Mutation | SNP | G | G | A | novel | Somatic | M49I | 0.393281 | NM_015469.3 | 505 | 306 | 199 |
| 435-PTdeep | IKBKAP | Unknown | hg19 | chr9 | 111693370 | 111693370 | Frame_Shift_Del | DEL | C | C | - | novel | Somatic | N20IfsTer22 | 0.216912 | NM_003640.5 | 541 | 423 | 118 |
| 435-PTdeep | ANKRD30A | 108 | hg19 | chr10 | 37442530 | 37442530 | Missense_Mutation | SNP | G | G | A | novel | Somatic | V524M | 0.092199 | NM_052997.2 | 1267 | 1150 | 117 |
| 435-PTdeep | CFAP46 | 201 | hg19 | chr10 | 134699339 | 134699339 | Frame_Shift_Del | DEL | CC | CC | G | novel | Somatic | R1143TfsTer7 | 0.196676 | NM_001200049.3 | 1079 | 866 | 213 |
| 435-PTdeep | DNHD1 | 634 | hg19 | chr11 | 6568863 | 6568863 | Missense_Mutation | SNP | C | C | G | novel | Somatic | L2232V | 0.12336 | NM_144666.2 | 381 | 334 | 47 |
| 435-PTdeep | DDB2 | 945 | hg19 | chr11 | 47238418 | 47238418 | Nonsense_Mutation | SNP | C | C | T | novel | Somatic | Q92Ter | 0.166667 | NM_000107.2 | 390 | 325 | 65 |
| 435-PTdeep | OR9G4 | 1016 | hg19 | chr11 | 56510878 | 56510878 | Missense_Mutation | SNP | C | C | T | novel | Somatic | R137H | 0.447368 | NM_001005284.1 | 228 | 126 | 102 |
| 435-PTdeep | PPFIA1 | 139 | hg19 | chr11 | 70172459 | 70172459 | Missense_Mutation | SNP | C | C | T | novel | Somatic | T233M | 0.334928 | NM_003626.4 | 418 | 278 | 140 |
| 435-PTdeep | CEP164 | 1479 | hg19 | chr11 | 117241956 | 117241956 | Missense_Mutation | SNP | G | G | T | novel | Somatic | G309V | 0.188017 | NM_014956.5 | 482 | 391 | 91 |
| 435-PTdeep | MED21 | 792 | hg19 | chr12 | 27180351 | 27180351 | Missense_Mutation | SNP | C | C | G | novel | Somatic | P77A | 0.254593 | NM_004264.5 | 380 | 283 | 97 |
| 435-PTdeep | SCN8A | 122 | hg19 | chr12 | 52115532 | 52115532 | In_Frame_Del | DEL | GCTACAGCG | GCTACAGCG | - | novel | Somatic | G619_S621del | 0.088977 | NM_014191.4 | 1505 | 1371 | 134 |
| 435-PTdeep | OTOGL | 18 | hg19 | chr12 | 80696424 | 80696424 | Frame_Shift_Ins | INS | - | - | T | novel | Somatic | L1019SfsTer31 | 0.292576 | NM_173591.3 | 447 | 313 | 134 |
| 435-PTdeep | ANAPC5 | 189 | hg19 | chr12 | 121766207 | 121766207 | Missense_Mutation | SNP | C | C | T | novel | Somatic | D406N | 0.359292 | NM_016237.5 | 560 | 357 | 203 |
| 435-PTdeep | HIP1R | 190 | hg19 | chr12 | 123343622 | 123343622 | Missense_Mutation | SNP | T | T | G | novel | Somatic | C725G | 0.137148 | NM_003959.3 | 1099 | 948 | 151 |
| 435-PTdeep | ZNF891 | 1605 | hg19 | chr12 | 133697292 | 133697292 | Nonsense_Mutation | SNP | C | C | A | novel | Somatic | E405Ter | 0.305723 | NM_001277291.1 | 661 | 458 | 203 |
| 435-PTdeep | RB1 | 119 | hg19 | chr13 | 48954188 | 48954188 | Splice_Site | SNP | G | G | T | novel | Somatic |  | 0.413793 | NM_000321.2 | 115 | 67 | 48 |
| 435-PTdeep | ZFP36L1 | 1113 | hg19 | chr14 | 69256608 | 69256608 | Frame_Shift_Ins | INS | - | - | GCAGCCCG | novel | Somatic | L289PfsTer16 | 0.205911 | NM_001244701.1 | 2039 | 1614 | 425 |
| 435-PTdeep | ZC3H14 | 1264 | hg19 | chr14 | 89076131 | 89076131 | Missense_Mutation | SNP | C | C | G | novel | Somatic | P694R | 0.154135 | NM_024824.5 | 531 | 449 | 82 |
| 435-PTdeep | KIF26A | 1383 | hg19 | chr14 | 104618379 | 104618379 | Missense_Mutation | SNP | G | G | A | novel | Somatic | D106N | 0.302495 | NM_015656.1 | 961 | 670 | 291 |
| 435-PTdeep | STARD9 | 14 | hg19 | chr15 | 42983821 | 42983821 | Missense_Mutation | SNP | G | G | T | novel | Somatic | D3349Y | 0.171756 | NM_020759.3 | 521 | 431 | 90 |
| 435-PTdeep | CD276 | 1149 | hg19 | chr15 | 73996147 | 73996147 | Missense_Mutation | SNP | T | T | C | novel | Somatic | V294A | 0.175824 | NM_001024736.2 | 1729 | 1425 | 304 |
| 435-PTdeep | SALL1 | 975 | hg19 | chr16 | 51174554 | 51174554 | Missense_Mutation | SNP | G | G | T | novel | Somatic | P527T | 0.164894 | NM_002968.3 | 564 | 471 | 93 |
| 435-PTdeep | TOX3 | 123 | hg19 | chr16 | 52473235 | 52473235 | Missense_Mutation | SNP | G | G | A | novel | Somatic | P545S | 0.171399 | NM_001080430.4 | 950 | 787 | 163 |
| 435-PTdeep | MT1X | 1017 | hg19 | chr16 | 56716475 | 56716475 | Missense_Mutation | SNP | C | C | T | novel | Somatic | S8L | 0.160169 | NM_005952.4 | 946 | 794 | 152 |
| 435-PTdeep | SGSM2 | 602 | hg19 | chr17 | 2274512 | 2274512 | Intron | DEL | GAAGGTGTCTCAGGCCCT | GAAGGTGTCTCAGGCCCT | - | novel | Somatic |  | 0.248485 | NM_014853.3 | 494 | 371 | 123 |
| 435-PTdeep | TP53 | 642 | hg19 | chr17 | 7574003 | 7574003 | Nonsense_Mutation | SNP | G | G | A | novel | Somatic | R342Ter | 0.29064 | NM_000546.5 | 406 | 288 | 118 |
| 435-PTdeep | TP53 | 642 | hg19 | chr17 | 7576851 | 7576851 | Splice_Site | SNP | A | A | G | novel | Somatic |  | 0.35 | NM_000546.5 | 397 | 257 | 140 |
| 435-PTdeep | HS3ST3B1 | 693 | hg19 | chr17 | 14204973 | 14204973 | Missense_Mutation | SNP | G | G | A | novel | Somatic | M46I | 0.290191 | NM_006041.3 | 734 | 521 | 213 |
| 435-PTdeep | KRTAP1-1 | 884 | hg19 | chr17 | 39197634 | 39197634 | Missense_Mutation | SNP | T | T | C | novel | Somatic | T6A | 0.25 | NM_030967.3 | 819 | 614 | 205 |
| 435-PTdeep | CNTNAP1 | 896 | hg19 | chr17 | 40845339 | 40845339 | Missense_Mutation | SNP | C | C | T | novel | Somatic | P926L | 0.178357 | NM_003632.3 | 995 | 817 | 178 |
| 435-PTdeep | HEATR6 | 1028 | hg19 | chr17 | 58149662 | 58149662 | Missense_Mutation | SNP | G | G | A | novel | Somatic | P205S | 0.276968 | NM_022070.5 | 342 | 247 | 95 |
| 435-PTdeep | CEP95 | 132 | hg19 | chr17 | 62525545 | 62525545 | Missense_Mutation | SNP | G | G | T | novel | Somatic | Q482H | 0.323232 | NM_138363.3 | 493 | 333 | 160 |
| 435-PTdeep | SYNGR2 | 1166 | hg19 | chr17 | 76167591 | 76167591 | Missense_Mutation | SNP | C | C | A | novel | Somatic | A113D | 0.102763 | NM_001363778.1 | 1156 | 1037 | 119 |
| 435-PTdeep | TXNL4A | 1178 | hg19 | chr18 | 77737679 | 77737679 | Missense_Mutation | SNP | T | T | C | novel | Somatic | Y59C | 0.429167 | NM_006701.5 | 239 | 136 | 103 |
| 435-PTdeep | HSD11B1L | 628 | hg19 | chr19 | 5687904 | 5687904 | Missense_Mutation | SNP | C | C | G | novel | Somatic | P317R | 0.186613 | NM_001267868.2 | 986 | 802 | 184 |
| 435-PTdeep | MUC16 | 81 | hg19 | chr19 | 9083751 | 9083751 | Missense_Mutation | SNP | C | C | T | novel | Somatic | M2688I | 0.403846 | NM_024690.2 | 156 | 93 | 63 |
| 435-PTdeep | ZNF700 | 84 | hg19 | chr19 | 12060008 | 12060008 | Missense_Mutation | SNP | A | A | G | novel | Somatic | Y390C | 0.428571 | NM_144566.3 | 700 | 400 | 300 |
| 435-PTdeep | ZNF429 | 750 | hg19 | chr19 | 21720557 | 21720557 | Missense_Mutation | SNP | A | A | G | novel | Somatic | K568E | 0.035088 | NM_001001415.4 | 1083 | 1045 | 38 |
| 435-PTdeep | ZNF729 | 756 | hg19 | chr19 | 22496921 | 22496921 | Missense_Mutation | SNP | A | A | T | novel | Somatic | K234N | 0.292332 | NM_001242680.2 | 625 | 442 | 183 |
| 435-PTdeep | ZNF229 | 927 | hg19 | chr19 | 44934516 | 44934516 | Missense_Mutation | SNP | G | G | T | novel | Somatic | S147Y | 0.257116 | NM_014518.4 | 1053 | 782 | 271 |
| 435-PTdeep | RTN2 | 935 | hg19 | chr19 | 45998252 | 45998252 | Missense_Mutation | SNP | C | C | A | novel | Somatic | D31Y | 0.237752 | NM_005619.5 | 1387 | 1057 | 330 |
| 435-PTdeep | PRR12 | 967 | hg19 | chr19 | 50094915 | 50094915 | Missense_Mutation | SNP | G | G | T | novel | Somatic | D2Y | 0.096939 | NM_020719.3 | 977 | 882 | 95 |
| 435-PTdeep | ZNF578 | 989 | hg19 | chr19 | 53014350 | 53014350 | Missense_Mutation | SNP | C | C | A | novel | Somatic | A239D | 0.183766 | NM_001099694.2 | 877 | 714 | 163 |
| 435-PTdeep | ZNF582 | 1019 | hg19 | chr19 | 56895283 | 56895283 | Missense_Mutation | SNP | C | C | A | novel | Somatic | M532I | 0.266553 | NM_001320371.2 | 589 | 432 | 157 |
| 435-PTdeep | PPP1R3D | 1031 | hg19 | chr20 | 58514167 | 58514167 | Missense_Mutation | SNP | C | C | T | novel | Somatic | D274N | 0.194199 | NM_006242.4 | 1580 | 1272 | 308 |
| 435-PTdeep | AIRE | 933 | hg19 | chr21 | 45708294 | 45708294 | Missense_Mutation | SNP | C | C | G | novel | Somatic | A202G | 0.231207 | NM_000383.4 | 876 | 673 | 203 |
| 435-PTdeep | CLTCL1 | 91 | hg19 | chr22 | 19223307 | 19223307 | Missense_Mutation | SNP | C | C | T | novel | Somatic | C294Y | 0.134904 | NM_007098.4 | 465 | 402 | 63 |
| 435-PTdeep | CRYBB1 | 98 | hg19 | chr22 | 27008070 | 27008070 | Missense_Mutation | SNP | C | C | T | novel | Somatic | D89N | 0.280182 | NM_001887.4 | 438 | 315 | 123 |
| 435-PTdeep | MAPK12 | 971 | hg19 | chr22 | 50694122 | 50694122 | Missense_Mutation | SNP | G | G | C | novel | Somatic | H231Q | 0.030896 | NM_002969.6 | 970 | 940 | 30 |
| 435-PTdeep | NUDT10 | 974 | hg19 | chrX | 51076075 | 51076075 | Missense_Mutation | SNP | C | C | G | novel | Somatic | N86K | 0.613139 | NM_153183.3 | 683 | 263 | 420 |
| 435-PTdeep | RBMXL3 | 182 | hg19 | chrX | 114426006 | 114426006 | Missense_Mutation | SNP | C | C | T | novel | Somatic | R668C | 0.593544 | NM_001145346.1 | 1358 | 549 | 809 |
| 435-PTdeep | MAMLD1 | 1726 | hg19 | chrX | 149680775 | 149680775 | Missense_Mutation | SNP | C | C | T | novel | Somatic | P810L | 0.209476 | NM_001177465.3 | 399 | 315 | 84 |
| 435-LNmet | MST1L | 715 | hg19 | chr1 | 17084347 | 17084347 | Missense_Mutation | SNP | G | G | C | novel | Somatic | T557S | 0.059865 | NM_001271733.1 | 1184 | 1113 | 71 |
| 435-LNmet | IFNLR1 | 96 | hg19 | chr1 | 24513708 | 24513708 | Missense_Mutation | SNP | G | G | A | novel | Somatic | L11F | 0.566011 | NM_170743.4 | 709 | 306 | 403 |
| 435-LNmet | PHC2 | 842 | hg19 | chr1 | 33799853 | 33799853 | Missense_Mutation | SNP | G | G | T | novel | Somatic | S532R | 0.453608 | NM_198040.2 | 193 | 105 | 88 |
| 435-LNmet | EIF2B3 | 116 | hg19 | chr1 | 45407181 | 45407181 | Frame_Shift_Ins | INS | - | - | T | novel | Somatic | A151SfsTer6 | 0.052265 | NM_020365.5 | 287 | 272 | 15 |
| 435-LNmet | CYP4Z1 | 118 | hg19 | chr1 | 47581260 | 47581260 | Missense_Mutation | SNP | C | C | G | novel | Somatic | P421A | 0.115385 | NM_178134.3 | 234 | 207 | 27 |
| 435-LNmet | ATP1A1 | 1477 | hg19 | chr1 | 116932312 | 116932312 | Missense_Mutation | SNP | T | T | G | novel | Somatic | L336V | 0.493464 | NM_001160233.1 | 306 | 155 | 151 |
| 435-LNmet | GJA8 | 1709 | hg19 | chr1 | 147380773 | 147380773 | Missense_Mutation | SNP | G | G | A | novel | Somatic | G231R | 0.249191 | NM_005267.5 | 309 | 232 | 77 |
| 435-LNmet | CHRNB2 | 1764 | hg19 | chr1 | 154542030 | 154542030 | Missense_Mutation | SNP | T | T | C | novel | Somatic | S53P | 0.537815 | NM_000748.3 | 356 | 164 | 192 |
| 435-LNmet | EFNA1 | 1768 | hg19 | chr1 | 155106258 | 155106258 | Splice_Site | SNP | T | T | C | novel | Somatic |  | 0.481481 | NM_004428.3 | 536 | 276 | 260 |
| 435-LNmet | THBS3 | 1768 | hg19 | chr1 | 155174943 | 155174943 | Missense_Mutation | SNP | G | G | A | novel | Somatic | H151Y | 0.025556 | NM_007112.5 | 900 | 877 | 23 |
| 435-LNmet | HCN3 | 1769 | hg19 | chr1 | 155254548 | 155254548 | Splice_Site | DEL | GGT | GGT | - | novel | Somatic |  | 0.366071 | NM_020897.3 | 221 | 139 | 82 |
| 435-LNmet | LEFTY1 | 2309 | hg19 | chr1 | 226074608 | 226074608 | Missense_Mutation | SNP | T | T | G | novel | Somatic | K307T | 0.261737 | NM_020997.4 | 2407 | 1777 | 630 |
| 435-LNmet | ARV1 | 2348 | hg19 | chr1 | 231132996 | 231132996 | Missense_Mutation | SNP | C | C | G | novel | Somatic | S268C | 0.440329 | NM_022786.3 | 486 | 272 | 214 |
| 435-LNmet | TRIM67 | 293 | hg19 | chr1 | 231333130 | 231333130 | Missense_Mutation | SNP | A | A | G | novel | Somatic | Q353R | 0.052632 | NM_001004342.4 | 361 | 342 | 19 |
| 435-LNmet | RYR2 | 299 | hg19 | chr1 | 237754093 | 237754093 | Frame_Shift_Del | DEL | GG | GG | - | novel | Somatic | G1321AfsTer12 | 0.05915 | NM_001035.3 | 540 | 508 | 32 |
| 435-LNmet | ZNF496 | 2473 | hg19 | chr1 | 247464275 | 247464275 | Missense_Mutation | SNP | C | C | T | novel | Somatic | C437Y | 0.202333 | NM_032752.3 | 1969 | 1570 | 399 |
| 435-LNmet | OR2M4 | 2480 | hg19 | chr1 | 248402295 | 248402295 | Missense_Mutation | SNP | C | C | T | novel | Somatic | P22L | 0.484108 | NM_017504.1 | 409 | 211 | 198 |
| 435-LNmet | SUPT7L | 797 | hg19 | chr2 | 27884121 | 27884121 | Missense_Mutation | SNP | G | G | A | novel | Somatic | P50L | 0.305389 | NM_014860.3 | 498 | 345 | 153 |
| 435-LNmet | WDR43 | 807 | hg19 | chr2 | 29158455 | 29158455 | Missense_Mutation | SNP | T | T | G | novel | Somatic | I502M | 0.315789 | NM_015131.3 | 247 | 169 | 78 |
| 435-LNmet | CYP1B1 | 877 | hg19 | chr2 | 38298328 | 38298328 | Missense_Mutation | SNP | C | C | T | novel | Somatic | R390H | 0.117871 | NM_000104.3 | 261 | 230 | 31 |
| 435-LNmet | KIAA1841 | 131 | hg19 | chr2 | 61343204 | 61343204 | Missense_Mutation | SNP | A | A | C | novel | Somatic | K612T | 0.114804 | NM_001129993.3 | 330 | 292 | 38 |
| 435-LNmet | C2orf42 | 140 | hg19 | chr2 | 70377673 | 70377673 | Missense_Mutation | SNP | C | C | G | novel | Somatic | E514Q | 0.314917 | NM_001348758.1 | 180 | 123 | 57 |
| 435-LNmet | MYO3B | 236 | hg19 | chr2 | 171375987 | 171375987 | Missense_Mutation | SNP | C | C | T | novel | Somatic | A1171V | 0.651899 | NM_138995.5 | 158 | 55 | 103 |
| 435-LNmet | NFE2L2 | 242 | hg19 | chr2 | 178098815 | 178098815 | Missense_Mutation | SNP | T | T | A | novel | Somatic | D77V | 0.679245 | NM_006164.5 | 211 | 67 | 144 |
| 435-LNmet | HRH1 | 671 | hg19 | chr3 | 11301493 | 11301493 | Missense_Mutation | SNP | G | G | T | novel | Somatic | W257L | 0.7 | NM_001098213.2 | 110 | 33 | 77 |
| 435-LNmet | BBX | 175 | hg19 | chr3 | 107517396 | 107517396 | Missense_Mutation | SNP | A | A | G | novel | Somatic | M794V | 0.07868 | NM_001142568.3 | 787 | 725 | 62 |
| 435-LNmet | TMCC1 | 196 | hg19 | chr3 | 129547050 | 129547050 | Missense_Mutation | SNP | G | G | T | novel | Somatic | H58N | 0.076179 | NM_001349263.2 | 827 | 764 | 63 |
| 435-LNmet | COPB2 | 1646 | hg19 | chr3 | 139097976 | 139097976 | Missense_Mutation | SNP | C | C | G | novel | Somatic | E90Q | 0.101064 | NM_004766.3 | 564 | 507 | 57 |
| 435-LNmet | B3GALNT1 | 1811 | hg19 | chr3 | 160803865 | 160803865 | Nonsense_Mutation | SNP | G | G | C | novel | Somatic | Y346Ter | 0.207436 | NM_001349162.1 | 510 | 404 | 106 |
| 435-LNmet | EIF4G1 | 1989 | hg19 | chr3 | 184041658 | 184041658 | Missense_Mutation | SNP | G | G | A | novel | Somatic | D796N | 0.037132 | NM_001194947.1 | 779 | 750 | 29 |
| 435-LNmet | UGT2B7 | 1118 | hg19 | chr4 | 69962686 | 69962686 | Missense_Mutation | SNP | G | G | A | novel | Somatic | A150T | 0.425 | NM_001074.4 | 239 | 137 | 102 |
| 435-LNmet | STPG2 | 20 | hg19 | chr4 | 98865102 | 98865102 | Missense_Mutation | SNP | G | G | C | novel | Somatic | N330K | 0.382353 | NM_174952.3 | 204 | 126 | 78 |
| 435-LNmet | IQGAP2 | 1164 | hg19 | chr5 | 75902129 | 75902129 | Splice_Site | SNP | G | G | T | novel | Somatic |  | 0.369318 | NM_006633.4 | 176 | 111 | 65 |
| 435-LNmet | MTRNR2L2 | 1194 | hg19 | chr5 | 79945871 | 79945871 | Missense_Mutation | SNP | G | G | A | novel | Somatic | S12L | 0.060748 | NM_001190470.2 | 213 | 200 | 13 |
| 435-LNmet | ARRDC3 | 1276 | hg19 | chr5 | 90678789 | 90678789 | Nonsense_Mutation | SNP | C | C | A | novel | Somatic | E41Ter | 0.045833 | NM_020801.4 | 240 | 229 | 11 |
| 435-LNmet | TSLP | 1427 | hg19 | chr5 | 110409272 | 110409272 | Missense_Mutation | SNP | G | G | C | novel | Somatic | A94P | 0.047619 | NM_033035.5 | 273 | 260 | 13 |
| 435-LNmet | DIAPH1 | 207 | hg19 | chr5 | 140903716 | 140903716 | Missense_Mutation | SNP | G | G | A | novel | Somatic | R1219C | 0.42515 | NM_005219.5 | 165 | 94 | 71 |
| 435-LNmet | HIST1H2BH | 785 | hg19 | chr6 | 26252007 | 26252007 | Nonsense_Mutation | SNP | C | C | G | novel | Somatic | Y43Ter | 0.261161 | NM_003524.2 | 448 | 331 | 117 |
| 435-LNmet | DNAH8 | 13 | hg19 | chr6 | 38980248 | 38980248 | Missense_Mutation | SNP | A | A | G | novel | Somatic | M4517V | 0.158076 | NM_001206927.2 | 291 | 245 | 46 |
| 435-LNmet | AARS2 | 922 | hg19 | chr6 | 44271982 | 44271982 | Missense_Mutation | SNP | G | G | T | novel | Somatic | P648H | 0.165217 | NM_020745.4 | 457 | 381 | 76 |
| 435-LNmet | MEP1A | 117 | hg19 | chr6 | 46803179 | 46803179 | Frame_Shift_Del | DEL | CC | CC | A | novel | Somatic | L661WfsTer26 | 0.064626 | NM_005588.3 | 287 | 268 | 19 |
| 435-LNmet | TRAM2 | 15 | hg19 | chr6 | 52369400 | 52369400 | Missense_Mutation | SNP | T | T | G | novel | Somatic | K343T | 0.17004 | NM_012288.4 | 741 | 615 | 126 |
| 435-LNmet | LCA5 | 149 | hg19 | chr6 | 80228571 | 80228571 | Missense_Mutation | SNP | C | C | G | novel | Somatic | R14T | 0.095477 | NM_181714.4 | 199 | 180 | 19 |
| 435-LNmet | FNDC1 | 225 | hg19 | chr6 | 159618514 | 159618514 | Missense_Mutation | SNP | G | G | A | novel | Somatic | G54D | 0.09901 | NM_032532.3 | 202 | 182 | 20 |
| 435-LNmet | TTLL2 | 1864 | hg19 | chr6 | 167753992 | 167753992 | Missense_Mutation | SNP | C | C | A | novel | Somatic | L202M | 0.187845 | NM_031949.5 | 181 | 147 | 34 |
| 435-LNmet | AGBL3 | 201 | hg19 | chr7 | 134718867 | 134718867 | Intron | SNP | C | C | G | novel | Somatic |  | 0.471074 | NM_178563.4 | 121 | 64 | 57 |
| 435-LNmet | CLCN1 | 1676 | hg19 | chr7 | 143042675 | 143042675 | Missense_Mutation | SNP | C | C | G | novel | Somatic | H664Q | 0.551931 | NM_000083.3 | 1162 | 519 | 643 |
| 435-LNmet | CYP7B1 | 135 | hg19 | chr8 | 65711107 | 65711107 | Missense_Mutation | SNP | G | G | A | novel | Somatic | S13L | 0.126501 | NM_004820.5 | 1249 | 1091 | 158 |
| 435-LNmet | PKHD1L1 | 178 | hg19 | chr8 | 110448573 | 110448573 | Missense_Mutation | SNP | C | C | G | novel | Somatic | T1171S | 0.256667 | NM_177531.6 | 300 | 223 | 77 |
| 435-LNmet | PKHD1L1 | 178 | hg19 | chr8 | 110520435 | 110520435 | Missense_Mutation | SNP | A | A | T | novel | Somatic | E3779D | 0.318008 | NM_177531.6 | 261 | 178 | 83 |
| 435-LNmet | THEM6 | 1682 | hg19 | chr8 | 143809078 | 143809078 | Missense_Mutation | SNP | C | C | T | novel | Somatic | A105V | 0.031963 | NM_016647.3 | 1095 | 1060 | 35 |
| 435-LNmet | PLEC | 1691 | hg19 | chr8 | 145000031 | 145000031 | Missense_Mutation | SNP | C | C | T | novel | Somatic | A1493T | 0.326339 | NM_201380.4 | 1381 | 930 | 451 |
| 435-LNmet | KLHL9 | 747 | hg19 | chr9 | 21333271 | 21333271 | Missense_Mutation | SNP | C | C | T | novel | Somatic | A530T | 0.553279 | NM_018847.4 | 244 | 109 | 135 |
| 435-LNmet | NUDT2 | 105 | hg19 | chr9 | 34343337 | 34343337 | Missense_Mutation | SNP | C | C | T | novel | Somatic | R115C | 0.055046 | NM_001161.5 | 326 | 308 | 18 |
| 435-LNmet | TMEM8B | 858 | hg19 | chr9 | 35846584 | 35846584 | Nonsense_Mutation | INS | - | - | A | novel | Somatic | Y658Ter | 0.489459 | NM_001042590.4 | 2172 | 1104 | 1068 |
| 435-LNmet | NIPSNAP3A | 1405 | hg19 | chr9 | 107513323 | 107513323 | Missense_Mutation | SNP | G | G | A | novel | Somatic | M49I | 0.4 | NM_015469.3 | 285 | 171 | 114 |
| 435-LNmet | IKBKAP | Unknown | hg19 | chr9 | 111693370 | 111693370 | Frame_Shift_Del | DEL | C | C | - | novel | Somatic | N20IfsTer22 | 0.312102 | NM_003640.5 | 313 | 215 | 98 |
| 435-LNmet | LRRTM3 | 138 | hg19 | chr10 | 68687614 | 68687614 | Missense_Mutation | SNP | T | T | A | novel | Somatic | W314R | 0.087432 | NM_178011.5 | 183 | 167 | 16 |
| 435-LNmet | CFAP46 | 201 | hg19 | chr10 | 134699339 | 134699339 | Frame_Shift_Del | DEL | CC | CC | G | novel | Somatic | R1143TfsTer7 | 0.361285 | NM_001200049.3 | 1263 | 802 | 461 |
| 435-LNmet | OTOG | 719 | hg19 | chr11 | 17653444 | 17653444 | Missense_Mutation | SNP | G | G | A | novel | Somatic | R2315H | 0.042316 | NM_001277269.2 | 448 | 429 | 19 |
| 435-LNmet | KCNC1 | 720 | hg19 | chr11 | 17801129 | 17801129 | Missense_Mutation | SNP | G | G | A | novel | Somatic | G544E | 0.030697 | NM_001112741.1 | 847 | 821 | 26 |
| 435-LNmet | DDB2 | 945 | hg19 | chr11 | 47238418 | 47238418 | Nonsense_Mutation | SNP | C | C | T | novel | Somatic | Q92Ter | 0.333333 | NM_000107.2 | 174 | 116 | 58 |
| 435-LNmet | OR5T1 | 1012 | hg19 | chr11 | 56043214 | 56043214 | Missense_Mutation | SNP | G | G | C | novel | Somatic | D34H | 0.109589 | NM_001004745.1 | 146 | 130 | 16 |
| 435-LNmet | OR9G4 | 1016 | hg19 | chr11 | 56510878 | 56510878 | Missense_Mutation | SNP | C | C | T | novel | Somatic | R137H | 0.585366 | NM_001005284.1 | 163 | 67 | 96 |
| 435-LNmet | AHNAK | 1060 | hg19 | chr11 | 62294964 | 62294964 | Missense_Mutation | SNP | T | T | C | novel | Somatic | T2309A | 0.031496 | NM_001620.3 | 380 | 368 | 12 |
| 435-LNmet | AHNAK | 1060 | hg19 | chr11 | 62294991 | 62294991 | Missense_Mutation | SNP | A | A | G | novel | Somatic | F2300L | 0.026549 | NM_001620.3 | 452 | 440 | 12 |
| 435-LNmet | BSCL2 | 1061 | hg19 | chr11 | 62469965 | 62469965 | Missense_Mutation | SNP | G | G | A | novel | Somatic | S90L | 0.071429 | NM_032667.6 | 321 | 298 | 23 |
| 435-LNmet | PPFIA1 | 139 | hg19 | chr11 | 70172459 | 70172459 | Missense_Mutation | SNP | C | C | T | novel | Somatic | T233M | 0.207447 | NM_003626.4 | 187 | 148 | 39 |
| 435-LNmet | CEP164 | 1479 | hg19 | chr11 | 117241956 | 117241956 | Missense_Mutation | SNP | G | G | T | novel | Somatic | G309V | 0.57971 | NM_014956.5 | 207 | 87 | 120 |
| 435-LNmet | MED21 | 792 | hg19 | chr12 | 27180351 | 27180351 | Missense_Mutation | SNP | C | C | G | novel | Somatic | P77A | 0.378378 | NM_004264.5 | 259 | 161 | 98 |
| 435-LNmet | ASIC1 | 970 | hg19 | chr12 | 50453613 | 50453613 | Missense_Mutation | SNP | G | G | A | novel | Somatic | R145H | 0.100457 | NM_020039.4 | 436 | 392 | 44 |
| 435-LNmet | OTOGL | 18 | hg19 | chr12 | 80696424 | 80696424 | Frame_Shift_Ins | INS | - | - | T | novel | Somatic | L1019SfsTer31 | 0.4375 | NM_173591.3 | 255 | 143 | 112 |
| 435-LNmet | RBM19 | 22 | hg19 | chr12 | 114384186 | 114384186 | Frame_Shift_Ins | INS | - | - | CC | novel | Somatic | E501GfsTer69 | 0.05379 | NM_016196.4 | 408 | 386 | 22 |
| 435-LNmet | ANAPC5 | 189 | hg19 | chr12 | 121766207 | 121766207 | Missense_Mutation | SNP | C | C | T | novel | Somatic | D406N | 0.463259 | NM_016237.5 | 313 | 168 | 145 |
| 435-LNmet | HIP1R | 190 | hg19 | chr12 | 123343622 | 123343622 | Missense_Mutation | SNP | T | T | G | novel | Somatic | C725G | 0.197235 | NM_003959.3 | 1083 | 869 | 214 |
| 435-LNmet | ZNF891 | 1605 | hg19 | chr12 | 133697292 | 133697292 | Nonsense_Mutation | SNP | C | C | A | novel | Somatic | E405Ter | 0.377976 | NM_001277291.1 | 335 | 208 | 127 |
| 435-LNmet | RB1 | 119 | hg19 | chr13 | 48954188 | 48954188 | Splice_Site | SNP | G | G | T | novel | Somatic |  | 0.585366 | NM_000321.2 | 41 | 17 | 24 |
| 435-LNmet | ZFP36L1 | 1113 | hg19 | chr14 | 69256608 | 69256608 | Frame_Shift_Ins | INS | - | - | GCAGCCCG | novel | Somatic | L289PfsTer16 | 0.320108 | NM_001244701.1 | 1464 | 988 | 476 |
| 435-LNmet | ZC3H14 | 1264 | hg19 | chr14 | 89076131 | 89076131 | Missense_Mutation | SNP | C | C | G | novel | Somatic | P694R | 0.080321 | NM_024824.5 | 248 | 228 | 20 |
| 435-LNmet | KIF26A | 1383 | hg19 | chr14 | 104618379 | 104618379 | Missense_Mutation | SNP | G | G | A | novel | Somatic | D106N | 0.313964 | NM_015656.1 | 1065 | 730 | 335 |
| 435-LNmet | STARD9 | 14 | hg19 | chr15 | 42983821 | 42983821 | Missense_Mutation | SNP | G | G | T | novel | Somatic | D3349Y | 0.240385 | NM_020759.3 | 208 | 158 | 50 |
| 435-LNmet | SALL1 | 975 | hg19 | chr16 | 51174554 | 51174554 | Missense_Mutation | SNP | G | G | T | novel | Somatic | P527T | 0.205882 | NM_002968.3 | 237 | 188 | 49 |
| 435-LNmet | MT1X | 1017 | hg19 | chr16 | 56716475 | 56716475 | Missense_Mutation | SNP | C | C | T | novel | Somatic | S8L | 0.217391 | NM_005952.4 | 411 | 321 | 90 |
| 435-LNmet | SGSM2 | 602 | hg19 | chr17 | 2274512 | 2274512 | Intron | DEL | GAAGGTGTCTCAGGCCCT | GAAGGTGTCTCAGGCCCT | - | novel | Somatic |  | 0.288462 | NM_014853.3 | 364 | 259 | 105 |
| 435-LNmet | ZMYND15 | 620 | hg19 | chr17 | 4644320 | 4644320 | Missense_Mutation | SNP | C | C | G | novel | Somatic | N159K | 0.07728 | NM_001267822.1 | 645 | 595 | 50 |
| 435-LNmet | TP53 | 642 | hg19 | chr17 | 7574003 | 7574003 | Nonsense_Mutation | SNP | G | G | A | novel | Somatic | R342Ter | 0.365639 | NM_000546.5 | 227 | 144 | 83 |
| 435-LNmet | TP53 | 642 | hg19 | chr17 | 7576851 | 7576851 | Splice_Site | SNP | A | A | G | novel | Somatic |  | 0.33913 | NM_000546.5 | 230 | 152 | 78 |
| 435-LNmet | PSMD3 | 109 | hg19 | chr17 | 38152442 | 38152442 | Missense_Mutation | SNP | C | C | T | novel | Somatic | R443W | 0.08642 | NM_002809.4 | 888 | 811 | 77 |
| 435-LNmet | KRTAP1-1 | 884 | hg19 | chr17 | 39197634 | 39197634 | Missense_Mutation | SNP | T | T | C | novel | Somatic | T6A | 0.310897 | NM_030967.3 | 311 | 214 | 97 |
| 435-LNmet | HEATR6 | 1028 | hg19 | chr17 | 58149662 | 58149662 | Missense_Mutation | SNP | G | G | A | novel | Somatic | P205S | 0.359551 | NM_022070.5 | 175 | 111 | 64 |
| 435-LNmet | CEP95 | 132 | hg19 | chr17 | 62525545 | 62525545 | Missense_Mutation | SNP | G | G | T | novel | Somatic | Q482H | 0.401361 | NM_138363.3 | 294 | 176 | 118 |
| 435-LNmet | TXNL4A | 1178 | hg19 | chr18 | 77737679 | 77737679 | Missense_Mutation | SNP | T | T | C | novel | Somatic | Y59C | 0.675439 | NM_006701.5 | 114 | 37 | 77 |
| 435-LNmet | MUC16 | 81 | hg19 | chr19 | 9083751 | 9083751 | Missense_Mutation | SNP | C | C | T | novel | Somatic | M2688I | 0.612245 | NM_024690.2 | 49 | 19 | 30 |
| 435-LNmet | ZNF700 | 84 | hg19 | chr19 | 12060008 | 12060008 | Missense_Mutation | SNP | A | A | G | novel | Somatic | Y390C | 0.715254 | NM_144566.3 | 295 | 84 | 211 |
| 435-LNmet | ZNF429 | 750 | hg19 | chr19 | 21720557 | 21720557 | Missense_Mutation | SNP | A | A | G | novel | Somatic | K568E | 0.03858 | NM_001001415.4 | 646 | 621 | 25 |
| 435-LNmet | ZNF729 | 756 | hg19 | chr19 | 22496921 | 22496921 | Missense_Mutation | SNP | A | A | T | novel | Somatic | K234N | 0.338608 | NM_001242680.2 | 316 | 209 | 107 |
| 435-LNmet | MAP3K10 | 895 | hg19 | chr19 | 40719955 | 40719955 | Missense_Mutation | SNP | G | G | C | novel | Somatic | S790T | 0.071611 | NM_002446.4 | 2342 | 2174 | 168 |
| 435-LNmet | ZNF229 | 927 | hg19 | chr19 | 44934516 | 44934516 | Missense_Mutation | SNP | G | G | T | novel | Somatic | S147Y | 0.236364 | NM_014518.4 | 550 | 420 | 130 |
| 435-LNmet | RTN2 | 935 | hg19 | chr19 | 45998252 | 45998252 | Missense_Mutation | SNP | C | C | A | novel | Somatic | D31Y | 0.264817 | NM_005619.5 | 793 | 583 | 210 |
| 435-LNmet | ZNF578 | 989 | hg19 | chr19 | 53014350 | 53014350 | Missense_Mutation | SNP | C | C | A | novel | Somatic | A239D | 0.247818 | NM_001099694.2 | 572 | 430 | 142 |
| 435-LNmet | ZNF582 | 1019 | hg19 | chr19 | 56895283 | 56895283 | Missense_Mutation | SNP | C | C | A | novel | Somatic | M532I | 0.308571 | NM_001320371.2 | 350 | 242 | 108 |
| 435-LNmet | PPP1R3D | 1031 | hg19 | chr20 | 58514167 | 58514167 | Missense_Mutation | SNP | C | C | T | novel | Somatic | D274N | 0.354415 | NM_006242.4 | 1672 | 1078 | 594 |
| 435-LNmet | AIRE | 933 | hg19 | chr21 | 45708294 | 45708294 | Missense_Mutation | SNP | C | C | G | novel | Somatic | A202G | 0.241758 | NM_000383.4 | 455 | 345 | 110 |
| 435-LNmet | CRYBB1 | 98 | hg19 | chr22 | 27008070 | 27008070 | Missense_Mutation | SNP | C | C | T | novel | Somatic | D89N | 0.223958 | NM_001887.4 | 191 | 148 | 43 |
| 435-LNmet | TCF20 | 113 | hg19 | chr22 | 42611146 | 42611146 | Missense_Mutation | SNP | C | C | T | novel | Somatic | G56S | 0.030418 | NM_005650.3 | 526 | 510 | 16 |
| 435-LNmet | NUDT10 | 974 | hg19 | chrX | 51076075 | 51076075 | Missense_Mutation | SNP | C | C | G | novel | Somatic | N86K | 0.80344 | NM_153183.3 | 407 | 80 | 327 |
| 435-LNmet | RBMXL3 | 182 | hg19 | chrX | 114426006 | 114426006 | Missense_Mutation | SNP | C | C | T | novel | Somatic | R668C | 0.77693 | NM_001145346.1 | 1173 | 257 | 916 |
| 481-PTsup | C1orf167 | 675 | hg19 | chr1 | 11826885 | 11826885 | Missense_Mutation | SNP | G | G | T | novel | Somatic | A348S | 0.26556 | NM_001010881.2 | 241 | 177 | 64 |
| 481-PTsup | RHCE | 97 | hg19 | chr1 | 25715490 | 25715490 | Missense_Mutation | SNP | T | T | C | novel | Somatic | I306V | 0.305389 | NM_020485.5 | 166 | 115 | 51 |
| 481-PTsup | COL24A1 | 155 | hg19 | chr1 | 86289269 | 86289269 | Missense_Mutation | SNP | C | C | T | novel | Somatic | E1247K | 0.240506 | NM_152890.7 | 237 | 180 | 57 |
| 481-PTsup | SYPL2 | 1424 | hg19 | chr1 | 110009654 | 110009654 | Intron | DEL | A | A | - | novel | Somatic |  | 0.048309 | NM_001040709.2 | 411 | 391 | 20 |
| 481-PTsup | KCND3 | 180 | hg19 | chr1 | 112524505 | 112524505 | Missense_Mutation | SNP | C | C | T | novel | Somatic | V282M | 0.31236 | NM_004980.4 | 445 | 306 | 139 |
| 481-PTsup | ADAMTSL4 | 1733 | hg19 | chr1 | 150525554 | 150525554 | Missense_Mutation | SNP | C | C | T | novel | Somatic | R87W | 0.394977 | NM_001288608.1 | 874 | 528 | 346 |
| 481-PTsup | RIT1 | 1774 | hg19 | chr1 | 155874568 | 155874568 | Missense_Mutation | SNP | A | A | G | novel | Somatic | I64T | 0.086207 | NM_006912.6 | 116 | 106 | 10 |
| 481-PTsup | LMNA | 221 | hg19 | chr1 | 156106009 | 156106009 | Missense_Mutation | SNP | C | C | T | novel | Somatic | R388C | 0.021739 | NM_170708.3 | 872 | 853 | 19 |
| 481-PTsup | SPTA1 | 224 | hg19 | chr1 | 158612728 | 158612728 | Missense_Mutation | SNP | G | G | T | novel | Somatic | T1494K | 0.031447 | NM_003126.4 | 317 | 307 | 10 |
| 481-PTsup | COPA | 225 | hg19 | chr1 | 160261988 | 160261988 | Nonsense_Mutation | SNP | C | C | T | novel | Somatic | W987Ter | 0.215686 | NM_004371.4 | 102 | 80 | 22 |
| 481-PTsup | MROH9 | 29 | hg19 | chr1 | 170983294 | 170983294 | Missense_Mutation | SNP | G | G | A | novel | Somatic | E545K | 0.245763 | NM_001163629.1 | 118 | 89 | 29 |
| 481-PTsup | NAV1 | 265 | hg19 | chr1 | 201786380 | 201786380 | Frame_Shift_Del | DEL | C | C | - | novel | Somatic | D1835EfsTer12 | 0.195846 | NM_020443.4 | 335 | 269 | 66 |
| 481-PTsup | NVL | 2297 | hg19 | chr1 | 224424202 | 224424202 | Splice_Region | DEL | ACTT | ACTT | - | novel | Somatic |  | 0.188119 | NM_002533.4 | 101 | 82 | 19 |
| 481-PTsup | DNAH14 | 2302 | hg19 | chr1 | 225140364 | 225140364 | Splice_Region | SNP | T | T | G | novel | Somatic |  | 0.253521 | NM_001367479.1 | 71 | 53 | 18 |
| 481-PTsup | DNAH14 | 2302 | hg19 | chr1 | 225152201 | 225152201 | Missense_Mutation | SNP | A | A | G | novel | Somatic | I130V | 0.238971 | NM_001367479.1 | 271 | 206 | 65 |
| 481-PTsup | ARV1 | 2348 | hg19 | chr1 | 231132905 | 231132905 | Missense_Mutation | SNP | G | G | A | novel | Somatic | V238M | 0.215447 | NM_022786.3 | 242 | 189 | 53 |
| 481-PTsup | LPIN1 | 84 | hg19 | chr2 | 11955237 | 11955237 | Missense_Mutation | SNP | G | G | C | novel | Somatic | C807S | 0.427536 | NM_001261428.3 | 138 | 79 | 59 |
| 481-PTsup | FAM228B | 96 | hg19 | chr2 | 24387082 | 24387082 | Missense_Mutation | SNP | C | C | G | novel | Somatic | S270C | 0.278607 | NM_001145710.2 | 199 | 143 | 56 |
| 481-PTsup | ITSN2 | 96 | hg19 | chr2 | 24480817 | 24480817 | Missense_Mutation | SNP | C | C | A | novel | Somatic | R943I | 0.181818 | NM_006277.3 | 165 | 135 | 30 |
| 481-PTsup | DPYSL5 | 98 | hg19 | chr2 | 27121531 | 27121531 | Missense_Mutation | SNP | G | G | A | novel | Somatic | G55E | 0.342657 | NM_020134.4 | 142 | 93 | 49 |
| 481-PTsup | PKDCC | 907 | hg19 | chr2 | 42275428 | 42275428 | Missense_Mutation | SNP | C | C | T | novel | Somatic | S30L | 0.255771 | NM_138370.3 | 1070 | 793 | 277 |
| 481-PTsup | C2orf78 | 1149 | hg19 | chr2 | 74042938 | 74042938 | Missense_Mutation | SNP | G | G | C | novel | Somatic | E530Q | 0.214634 | NM_001080474.2 | 205 | 161 | 44 |
| 481-PTsup | MAP4K4 | 170 | hg19 | chr2 | 102452436 | 102452436 | Missense_Mutation | SNP | A | A | G | novel | Somatic | K257E | 0.079498 | NM_145686.3 | 239 | 220 | 19 |
| 481-PTsup | IL1R2 | 170 | hg19 | chr2 | 102644697 | 102644697 | Missense_Mutation | SNP | C | C | T | novel | Somatic | T347M | 0.098101 | NM_004633.4 | 315 | 284 | 31 |
| 481-PTsup | AMMECR1L | 1566 | hg19 | chr2 | 128631778 | 128631778 | Missense_Mutation | SNP | C | C | T | novel | Somatic | E11K | 0.056716 | NM_001199140.1 | 335 | 316 | 19 |
| 481-PTsup | ARHGAP15 | 210 | hg19 | chr2 | 144244956 | 144244956 | Missense_Mutation | SNP | C | C | G | novel | Somatic | H240D | 0.142857 | NM_018460.4 | 231 | 198 | 33 |
| 481-PTsup | TTN | 1955 | hg19 | chr2 | 179489297 | 179489297 | Missense_Mutation | SNP | C | C | T | novel | Somatic | G14904S | 0.15625 | NM_001267550.2 | 224 | 189 | 35 |
| 481-PTsup | TTN | 1955 | hg19 | chr2 | 179588247 | 179588247 | Missense_Mutation | SNP | G | G | T | novel | Somatic | L7194M | 0.149466 | NM_001267550.2 | 281 | 239 | 42 |
| 481-PTsup | FRZB | 1986 | hg19 | chr2 | 183707206 | 183707206 | Missense_Mutation | SNP | C | C | A | novel | Somatic | V198F | 0.548387 | NM_001463.4 | 91 | 40 | 51 |
| 481-PTsup | PMS1 | 2039 | hg19 | chr2 | 190656659 | 190656659 | Missense_Mutation | SNP | G | G | A | novel | Somatic | V42I | 0.125828 | NM_000534.4 | 151 | 132 | 19 |
| 481-PTsup | SLC4A3 | 2267 | hg19 | chr2 | 220504227 | 220504227 | Missense_Mutation | SNP | G | G | A | novel | Somatic | R1016Q | 0.360778 | NM_005070.4 | 668 | 427 | 241 |
| 481-PTsup | ARMC9 | 294 | hg19 | chr2 | 232087474 | 232087474 | Missense_Mutation | SNP | A | A | G | novel | Somatic | I180V | 0.149425 | NM_001271466.4 | 13 | 0 | 13 |
| 481-PTsup | ECEL1 | 2365 | hg19 | chr2 | 233348779 | 233348779 | Missense_Mutation | SNP | G | G | A | novel | Somatic | R447C | 0.288889 | NM_004826.4 | 358 | 254 | 104 |
| 481-PTsup | NGLY1 | 97 | hg19 | chr3 | 25805799 | 25805799 | Missense_Mutation | SNP | C | C | G | novel | Somatic | E84Q | 0.230263 | NM_018297.4 | 152 | 117 | 35 |
| 481-PTsup | SCN5A | 109 | hg19 | chr3 | 38622603 | 38622603 | Missense_Mutation | SNP | G | G | A | novel | Somatic | T1016M | 0.023952 | NM_001099404.1 | 1163 | 1135 | 28 |
| 481-PTsup | CDC25A | 952 | hg19 | chr3 | 48215868 | 48215868 | Missense_Mutation | SNP | G | G | A | novel | Somatic | S279F | 0.240506 | NM_001789.3 | 158 | 120 | 38 |
| 481-PTsup | PQLC2L | 27 | hg19 | chr3 | 157318018 | 157318018 | Silent | SNP | C | C | T | novel | Somatic | T113= | 0.069892 | NM_001130002.2 | 184 | 171 | 13 |
| 481-PTsup | ECT2 | 237 | hg19 | chr3 | 172491738 | 172491738 | Missense_Mutation | SNP | A | A | G | novel | Somatic | I492V | 0.086817 | NM_001349094.2 | 621 | 567 | 54 |
| 481-PTsup | LMLN | 2093 | hg19 | chr3 | 197712718 | 197712718 | Missense_Mutation | SNP | C | C | G | novel | Somatic | L296V | 0.055066 | NM_001136049.2 | 453 | 428 | 25 |
| 481-PTsup | JAKMIP1 | 78 | hg19 | chr4 | 6087158 | 6087158 | Missense_Mutation | SNP | T | T | G | novel | Somatic | M275L | 0.240385 | NM_001099433.2 | 104 | 79 | 25 |
| 481-PTsup | SLIT2 | 92 | hg19 | chr4 | 20552515 | 20552515 | Missense_Mutation | SNP | C | C | T | novel | Somatic | S852L | 0.240506 | NM_004787.4 | 79 | 60 | 19 |
| 481-PTsup | DSPP | 1260 | hg19 | chr4 | 88535772 | 88535772 | Missense_Mutation | SNP | A | A | G | novel | Somatic | N653S | 0.032051 | NM_014208.3 | 310 | 300 | 10 |
| 481-PTsup | ADH1B | 1349 | hg19 | chr4 | 100232705 | 100232705 | Missense_Mutation | SNP | G | G | A | novel | Somatic | R313C | 0.324324 | NM_000668.6 | 220 | 148 | 72 |
| 481-PTsup | TRAM1L1 | 1485 | hg19 | chr4 | 118006507 | 118006507 | Missense_Mutation | SNP | T | T | A | novel | Somatic | S15C | 0.15534 | NM_152402.3 | 614 | 518 | 96 |
| 481-PTsup | ADAD1 | 190 | hg19 | chr4 | 123333817 | 123333817 | Missense_Mutation | SNP | C | C | G | novel | Somatic | L368V | 0.207407 | NM_139243.4 | 135 | 107 | 28 |
| 481-PTsup | ZCCHC9 | 1199 | hg19 | chr5 | 80600551 | 80600551 | Intron | SNP | A | A | G | novel | Somatic |  | 0.238095 | NM_032280.2 | 126 | 96 | 30 |
| 481-PTsup | PCDHA2 | 1654 | hg19 | chr5 | 140174842 | 140174842 | Missense_Mutation | SNP | G | G | A | novel | Somatic | R98Q | 0.186099 | NM_018905.3 | 1334 | 1085 | 249 |
| 481-PTsup | PCDHA10 | 1654 | hg19 | chr5 | 140237414 | 140237414 | Missense_Mutation | SNP | G | G | A | novel | Somatic | R594H | 0.043438 | NM_018901.4 | 1077 | 1030 | 47 |
| 481-PTsup | RANBP17 | 235 | hg19 | chr5 | 170610183 | 170610183 | Frame_Shift_Del | DEL | A | A | - | novel | Somatic | K628NfsTer3 | 0.304878 | NM_022897.5 | 81 | 56 | 25 |
| 481-PTsup | CDHR2 | 1927 | hg19 | chr5 | 176002580 | 176002580 | Missense_Mutation | SNP | C | C | G | novel | Somatic | S281C | 0.046332 | NM_017675.5 | 259 | 247 | 12 |
| 481-PTsup | SSR1 | 640 | hg19 | chr6 | 7290149 | 7290149 | Missense_Mutation | SNP | C | C | T | novel | Somatic | R270K | 0.229508 | NM_003144.5 | 122 | 94 | 28 |
| 481-PTsup | SLC35B3 | 649 | hg19 | chr6 | 8428186 | 8428186 | Missense_Mutation | SNP | G | G | C | novel | Somatic | Q135E | 0.073034 | NM_001370476.2 | 178 | 165 | 13 |
| 481-PTsup | RARS2 | 1258 | hg19 | chr6 | 88251727 | 88251727 | Intron | SNP | C | C | T | novel | Somatic |  | 0.2625 | NM_020320.5 | 80 | 59 | 21 |
| 481-PTsup | MYB | 202 | hg19 | chr6 | 135518269 | 135518269 | Missense_Mutation | SNP | G | G | C | novel | Somatic | E458D | 0.22069 | NM_001130173.2 | 145 | 113 | 32 |
| 481-PTsup | GUSB | 1084 | hg19 | chr7 | 65425965 | 65425965 | Frame_Shift_Del | DEL | TC | TC | - | novel | Somatic | R625IfsTer7 | 0.177966 | NM_000181.4 | 118 | 97 | 21 |
| 481-PTsup | COL1A2 | 1302 | hg19 | chr7 | 94055747 | 94055747 | Missense_Mutation | SNP | A | A | T | novel | Somatic | I1004F | 0.184713 | NM_000089.4 | 156 | 127 | 29 |
| 481-PTsup | GNB2 | 1350 | hg19 | chr7 | 100275225 | 100275225 | Nonsense_Mutation | SNP | C | C | A | novel | Somatic | Y124Ter | 0.463845 | NM_005273.4 | 567 | 304 | 263 |
| 481-PTsup | SYPL1 | 1391 | hg19 | chr7 | 105752637 | 105752637 | Missense_Mutation | SNP | G | G | C | novel | Somatic | I24M | 0.020649 | NM_006754.4 | 1354 | 1326 | 28 |
| 481-PTsup | ZNF775 | 216 | hg19 | chr7 | 150095049 | 150095049 | Missense_Mutation | SNP | C | C | T | novel | Somatic | R494C | 0.023832 | NM_173680.4 | 2132 | 2081 | 51 |
| 481-PTsup | KMT2C | 27 | hg19 | chr7 | 151843734 | 151843734 | Missense_Mutation | SNP | C | C | G | novel | Somatic | E4661Q | 0.191176 | NM_170606.3 | 135 | 109 | 26 |
| 481-PTsup | SORBS3 | 756 | hg19 | chr8 | 22412472 | 22412472 | Nonsense_Mutation | SNP | C | C | T | novel | Somatic | R67Ter | 0.048023 | NM_005775.5 | 352 | 335 | 17 |
| 481-PTsup | TEX15 | 819 | hg19 | chr8 | 30725009 | 30725009 | Missense_Mutation | SNP | C | C | A | novel | Somatic | M104I | 0.152174 | NM_001350162.2 | 92 | 78 | 14 |
| 481-PTsup | CDH17 | 163 | hg19 | chr8 | 95201495 | 95201495 | Missense_Mutation | SNP | C | C | G | novel | Somatic | E24Q | 0.276836 | NM_004063.4 | 174 | 125 | 49 |
| 481-PTsup | MAL2 | 1502 | hg19 | chr8 | 120220771 | 120220771 | Frame_Shift_Del | DEL | G | G | - | novel | Somatic | R22GfsTer52 | 0.029353 | NM_052886.3 | 1906 | 1847 | 59 |
| 481-PTsup | TRIB1 | 1549 | hg19 | chr8 | 126448220 | 126448220 | Splice_Region | DEL | ATAATGGCTTGGTTCCTCTCTCTTGC | ATAATGGCTTGGTTCCTCTCTCTTGC | - | novel | Somatic |  | 0.192982 | NM_025195.4 | 56 | 45 | 11 |
| 481-PTsup | JRK | 1681 | hg19 | chr8 | 143746089 | 143746089 | Missense_Mutation | SNP | A | A | C | novel | Somatic | W463G | 0.056767 | NM_003724.4 | 441 | 290 | 151 |
| 481-PTsup | CYC1 | 1692 | hg19 | chr8 | 145151988 | 145151988 | Missense_Mutation | SNP | G | G | A | novel | Somatic | R275H | 0.168033 | NM_001916.5 | 244 | 203 | 41 |
| 481-PTsup | TONSL | 1696 | hg19 | chr8 | 145666438 | 145666438 | Nonsense_Mutation | SNP | G | G | A | novel | Somatic | Q308Ter | 0.300207 | NM_013432.5 | 959 | 669 | 290 |
| 481-PTsup | COMMD5 | 1699 | hg19 | chr8 | 146076704 | 146076704 | Missense_Mutation | SNP | G | G | A | novel | Somatic | A7V | 0.024653 | NM_014066.4 | 643 | 627 | 16 |
| 481-PTsup | DOCK8 | 73 | hg19 | chr9 | 441906 | 441906 | Missense_Mutation | SNP | G | G | A | novel | Somatic | R1796Q | 0.208696 | NM_203447.3 | 115 | 91 | 24 |
| 481-PTsup | KDM4C | 636 | hg19 | chr9 | 6984204 | 6984204 | Missense_Mutation | SNP | C | C | G | novel | Somatic | A385G | 0.2 | NM_015061.5 | 80 | 64 | 16 |
| 481-PTsup | CDKN2A | 752 | hg19 | chr9 | 21971036 | 21971036 | Missense_Mutation | SNP | C | C | T | novel | Somatic | D108N | 0.69511 | NM_000077.4 | 1041 | 316 | 725 |
| 481-PTsup | NR4A3 | 1367 | hg19 | chr9 | 102625947 | 102625947 | Missense_Mutation | SNP | A | A | G | novel | Somatic | N560S | 0.075342 | NM_006981.4 | 146 | 135 | 11 |
| 481-PTsup | CDC26 | 1470 | hg19 | chr9 | 116034777 | 116034777 | Missense_Mutation | SNP | C | C | G | novel | Somatic | R4P | 0.549618 | NM_139286.4 | 130 | 58 | 72 |
| 481-PTsup | RC3H2 | 1543 | hg19 | chr9 | 125616533 | 125616533 | Silent | SNP | A | A | G | novel | Somatic | I971= | 0.182927 | NM_001100588.3 | 82 | 67 | 15 |
| 481-PTsup | RNF224 | 1654 | hg19 | chr9 | 140123254 | 140123254 | Missense_Mutation | SNP | C | C | T | novel | Somatic | R63C | 0.236546 | NM_001190228.1 | 789 | 600 | 189 |
| 481-PTsup | RGR | 1241 | hg19 | chr10 | 86012687 | 86012687 | Missense_Mutation | SNP | C | C | G | novel | Somatic | L149V | 0.339806 | NM_002921.3 | 206 | 136 | 70 |
| 481-PTsup | PGGHG | 587 | hg19 | chr11 | 292999 | 292999 | Splice_Region | INS | - | - | G | novel | Somatic |  | 0.024313 | NM_025092.5 | 939 | 916 | 23 |
| 481-PTsup | EPS8L2 | 590 | hg19 | chr11 | 725827 | 725827 | Missense_Mutation | SNP | G | G | A | novel | Somatic | A554T | 0.310214 | NM_022772.4 | 782 | 536 | 246 |
| 481-PTsup | LUZP2 | 96 | hg19 | chr11 | 24750732 | 24750732 | Missense_Mutation | SNP | T | T | C | novel | Somatic | L27P | 0.292453 | NM_001009909.4 | 105 | 74 | 31 |
| 481-PTsup | TRIM48 | 1004 | hg19 | chr11 | 55035844 | 55035844 | Missense_Mutation | SNP | T | T | C | novel | Somatic | Y192H | 0.052288 | NM_024114.5 | 153 | 145 | 8 |
| 481-PTsup | VWCE | 1050 | hg19 | chr11 | 61058300 | 61058300 | Missense_Mutation | SNP | G | G | A | novel | Somatic | T132M | 0.031891 | NM_152718.2 | 423 | 409 | 14 |
| 481-PTsup | ZBTB3 | 132 | hg19 | chr11 | 62520061 | 62520061 | Missense_Mutation | SNP | C | C | G | novel | Somatic | G359A | 0.19697 | NM_001370809.1 | 132 | 106 | 26 |
| 481-PTsup | MARK2 | 1070 | hg19 | chr11 | 63673579 | 63673579 | Missense_Mutation | SNP | G | G | T | novel | Somatic | A652S | 0.171429 | NM_001039469.2 | 525 | 435 | 90 |
| 481-PTsup | NRXN2 | 1076 | hg19 | chr11 | 64421194 | 64421194 | Missense_Mutation | SNP | T | T | C | novel | Somatic | D797G | 0.170984 | NM_015080.4 | 375 | 309 | 66 |
| 481-PTsup | CHKA | 1102 | hg19 | chr11 | 67842184 | 67842184 | Splice_Region | SNP | C | C | A | novel | Somatic | P210= | 0.698473 | NM_001277.3 | 258 | 75 | 183 |
| 481-PTsup | ARHGEF17 | 1142 | hg19 | chr11 | 73073283 | 73073283 | Missense_Mutation | SNP | C | C | T | novel | Somatic | R1565W | 0.255172 | NM_014786.4 | 866 | 644 | 222 |
| 481-PTsup | C11orf71 | 1456 | hg19 | chr11 | 114270974 | 114270974 | Missense_Mutation | SNP | C | C | G | novel | Somatic | R27T | 0.223926 | NM_019021.4 | 650 | 504 | 146 |
| 481-PTsup | FGF23 | 619 | hg19 | chr12 | 4479799 | 4479799 | Nonsense_Mutation | SNP | G | G | A | novel | Somatic | Q156Ter | 0.022308 | NM_020638.3 | 1297 | 1268 | 29 |
| 481-PTsup | ACRBP | 636 | hg19 | chr12 | 6754383 | 6754383 | Splice_Region | SNP | C | C | G | novel | Somatic |  | 0.2875 | NM_032489.3 | 240 | 171 | 69 |
| 481-PTsup | CD163L1 | 642 | hg19 | chr12 | 7531771 | 7531771 | In_Frame_Del | DEL | TCAGCAATGTTC | TCAGCAATGTTC | - | novel | Somatic | M721_E725delinsI | 0.122047 | NM_174941.5 | 253 | 222 | 31 |
| 481-PTsup | ABCC9 | 11 | hg19 | chr12 | 21997422 | 21997422 | Missense_Mutation | SNP | C | C | G | novel | Somatic | D1104H | 0.246753 | NM_005691.3 | 153 | 115 | 38 |
| 481-PTsup | REP15 | 797 | hg19 | chr12 | 27849796 | 27849796 | Missense_Mutation | SNP | A | A | G | novel | Somatic | N101D | 0.710938 | NM_001029874.3 | 91 | 0 | 91 |
| 481-PTsup | PTHLH | 799 | hg19 | chr12 | 28116708 | 28116708 | Splice_Region | SNP | G | G | C | novel | Somatic |  | 0.19403 | NM_198965.2 | 134 | 108 | 26 |
| 481-PTsup | KRT5 | 988 | hg19 | chr12 | 52913855 | 52913855 | In_Frame_Del | DEL | TGGATATCCTCT | TGGATATCCTCT | - | novel | Somatic | K72_S75del | 0.144371 | NM_000424.4 | 749 | 640 | 109 |
| 481-PTsup | KRT76 | 990 | hg19 | chr12 | 53169361 | 53169361 | Missense_Mutation | SNP | T | T | A | novel | Somatic | K209M | 0.122449 | NM_015848.4 | 97 | 85 | 12 |
| 481-PTsup | SMARCC2 | 1016 | hg19 | chr12 | 56572825 | 56572825 | Frame_Shift_Del | DEL | TCTT | TCTT | - | novel | Somatic | K365TfsTer12 | 0.245614 | NM_001330288.2 | 113 | 85 | 28 |
| 481-PTsup | NXPH4 | 1024 | hg19 | chr12 | 57619423 | 57619423 | Missense_Mutation | SNP | T | T | G | novel | Somatic | F274V | 0.233881 | NM_007224.4 | 760 | 575 | 185 |
| 481-PTsup | TRHDE | 142 | hg19 | chr12 | 72667063 | 72667063 | Missense_Mutation | SNP | G | G | A | novel | Somatic | V169M | 0.19909 | NM_013381.2 | 877 | 702 | 175 |
| 481-PTsup | ACACB | 1421 | hg19 | chr12 | 109689929 | 109689929 | Missense_Mutation | SNP | A | A | G | novel | Somatic | I1957V | 0.21875 | NM_001093.4 | 159 | 124 | 35 |
| 481-PTsup | SH2B3 | 1438 | hg19 | chr12 | 111856595 | 111856595 | Missense_Mutation | SNP | C | C | T | novel | Somatic | R216C | 0.275168 | NM_005475.3 | 592 | 428 | 164 |
| 481-PTsup | OAS3 | 181 | hg19 | chr12 | 113405938 | 113405938 | Missense_Mutation | SNP | G | G | C | novel | Somatic | K1021N | 0.175325 | NM_006187.4 | 153 | 126 | 27 |
| 481-PTsup | EP400 | 199 | hg19 | chr12 | 132547070 | 132547070 | Missense_Mutation | SNP | C | C | G | novel | Somatic | Q2720E | 0.035714 | NM_015409.5 | 680 | 655 | 25 |
| 481-PTsup | SHISA2 | 788 | hg19 | chr13 | 26624876 | 26624876 | Missense_Mutation | SNP | C | C | T | novel | Somatic | E80K | 0.286701 | NM_001007538.1 | 572 | 406 | 166 |
| 481-PTsup | RXFP2 | 103 | hg19 | chr13 | 32366145 | 32366145 | Missense_Mutation | SNP | C | C | T | novel | Somatic | H450Y | 0.318182 | NM_130806.5 | 88 | 60 | 28 |
| 481-PTsup | ADCY4 | 774 | hg19 | chr14 | 24795357 | 24795357 | Missense_Mutation | SNP | C | C | T | novel | Somatic | R528Q | 0.066929 | NM_001198568.2 | 253 | 236 | 17 |
| 481-PTsup | MIA2 | 888 | hg19 | chr14 | 39763224 | 39763224 | Missense_Mutation | SNP | G | G | T | novel | Somatic | R780S | 0.063241 | NM_001329214.3 | 252 | 236 | 16 |
| 481-PTsup | MIA2 | 888 | hg19 | chr14 | 39763267 | 39763267 | Missense_Mutation | SNP | G | G | A | novel | Somatic | V795I | 0.054902 | NM_001329214.3 | 254 | 240 | 14 |
| 481-PTsup | SPTB | 135 | hg19 | chr14 | 65216751 | 65216751 | Missense_Mutation | SNP | T | T | C | novel | Somatic | I2242V | 0.04065 | NM_001024858.3 | 246 | 236 | 10 |
| 481-PTsup | SYNDIG1L | 1156 | hg19 | chr14 | 74876246 | 74876246 | Missense_Mutation | SNP | A | A | G | novel | Somatic | Y68H | 0.265403 | NM_001105579.2 | 622 | 454 | 168 |
| 481-PTsup | FLRT2 | 1241 | hg19 | chr14 | 86089539 | 86089539 | Missense_Mutation | SNP | G | G | A | novel | Somatic | V561I | 0.263959 | NM_001346143.2 | 196 | 144 | 52 |
| 481-PTsup | DUOX1 | 931 | hg19 | chr15 | 45448072 | 45448072 | Missense_Mutation | SNP | G | G | A | novel | Somatic | R1216H | 0.026385 | NM_017434.5 | 378 | 368 | 10 |
| 481-PTsup | HDC | 970 | hg19 | chr15 | 50546377 | 50546377 | Missense_Mutation | SNP | G | G | C | novel | Somatic | L224V | 0.316062 | NM_002112.4 | 192 | 131 | 61 |
| 481-PTsup | LIPC | 129 | hg19 | chr15 | 58855717 | 58855717 | Missense_Mutation | SNP | G | G | T | novel | Somatic | A395S | 0.394495 | NM_000236.3 | 109 | 66 | 43 |
| 481-PTsup | HERC1 | 16 | hg19 | chr15 | 64005673 | 64005673 | Missense_Mutation | SNP | G | G | A | novel | Somatic | R1448W | 0.283333 | NM_003922.4 | 120 | 86 | 34 |
| 481-PTsup | IGF1R | 167 | hg19 | chr15 | 99459989 | 99459989 | Missense_Mutation | SNP | G | G | C | novel | Somatic | E695D | 0.072052 | NM_000875.5 | 455 | 422 | 33 |
| 481-PTsup | PKD1 | 601 | hg19 | chr16 | 2161581 | 2161581 | Missense_Mutation | SNP | G | G | A | novel | Somatic | T1196M | 0.028703 | NM_001009944.3 | 867 | 842 | 25 |
| 481-PTsup | DNASE1L2 | 602 | hg19 | chr16 | 2287753 | 2287753 | Splice_Region | DEL | CCGCGGCGC | CCGCGGCGC | - | novel | Somatic |  | 0.229149 | NM_001374.3 | 1166 | 894 | 272 |
| 481-PTsup | TRAP1 | 613 | hg19 | chr16 | 3712953 | 3712953 | Missense_Mutation | SNP | G | G | A | novel | Somatic | S575L | 0.181481 | NM_016292.3 | 266 | 217 | 49 |
| 481-PTsup | CIITA | 83 | hg19 | chr16 | 11001325 | 11001325 | Missense_Mutation | SNP | A | A | T | novel | Somatic | E659V | 0.031469 | NM_000246.3 | 1124 | 1088 | 36 |
| 481-PTsup | ZKSCAN2 | 777 | hg19 | chr16 | 25263271 | 25263271 | Missense_Mutation | SNP | C | C | T | novel | Somatic | R255Q | 0.336134 | NM_001012981.5 | 119 | 79 | 40 |
| 481-PTsup | CORO1A | 815 | hg19 | chr16 | 30198518 | 30198518 | Missense_Mutation | SNP | G | G | C | novel | Somatic | E204Q | 0.042493 | NM_001193333.2 | 353 | 338 | 15 |
| 481-PTsup | ITGAX | 824 | hg19 | chr16 | 31383014 | 31383014 | Missense_Mutation | SNP | C | C | A | novel | Somatic | A690D | 0.139803 | NM_000887.5 | 606 | 521 | 85 |
| 481-PTsup | NUP93 | 1018 | hg19 | chr16 | 56866268 | 56866268 | Missense_Mutation | SNP | C | C | T | novel | Somatic | S438L | 0.252381 | NM_014669.5 | 210 | 157 | 53 |
| 481-PTsup | ADAMTS18 | 146 | hg19 | chr16 | 77396098 | 77396098 | Missense_Mutation | SNP | C | C | A | novel | Somatic | A374S | 0.167832 | NM_199355.4 | 143 | 119 | 24 |
| 481-PTsup | OR1E1 | 610 | hg19 | chr17 | 3301479 | 3301479 | Missense_Mutation | SNP | C | C | T | novel | Somatic | V76M | 0.138889 | NM_003553.3 | 323 | 278 | 45 |
| 481-PTsup | GPS2 | 640 | hg19 | chr17 | 7216782 | 7216782 | Missense_Mutation | SNP | G | G | A | novel | Somatic | S214L | 0.103448 | NM_004489.5 | 143 | 128 | 15 |
| 481-PTsup | DRC3 | 721 | hg19 | chr17 | 17910398 | 17910398 | Missense_Mutation | SNP | C | C | G | novel | Somatic | I421M | 0.186646 | NM_031294.4 | 1317 | 1071 | 246 |
| 481-PTsup | EFCAB5 | 12 | hg19 | chr17 | 28269284 | 28269284 | Missense_Mutation | SNP | G | G | T | novel | Somatic | R23S | 0.2375 | NM_198529.4 | 160 | 122 | 38 |
| 481-PTsup | NF1 | 101 | hg19 | chr17 | 29562641 | 29562641 | Nonsense_Mutation | SNP | C | C | T | novel | Somatic | R1241Ter | 0.035 | NM_001042492.3 | 200 | 193 | 7 |
| 481-PTsup | HOXB9 | 941 | hg19 | chr17 | 46703139 | 46703139 | Missense_Mutation | SNP | C | C | T | novel | Somatic | E165K | 0.313846 | NM_024017.5 | 648 | 444 | 204 |
| 481-PTsup | LAMA1 | 79 | hg19 | chr18 | 6959455 | 6959455 | Missense_Mutation | SNP | C | C | T | novel | Somatic | E2555K | 0.116505 | NM_005559.4 | 308 | 272 | 36 |
| 481-PTsup | ABCA7 | 9 | hg19 | chr19 | 1058878 | 1058878 | Missense_Mutation | SNP | G | G | A | novel | Somatic | R1780Q | 0.235577 | NM_019112.4 | 205 | 156 | 49 |
| 481-PTsup | TJP3 | 613 | hg19 | chr19 | 3740743 | 3740743 | Nonsense_Mutation | SNP | C | C | T | novel | Somatic | R609Ter | 0.393298 | NM_001267560.2 | 561 | 338 | 223 |
| 481-PTsup | APBA3 | 613 | hg19 | chr19 | 3752703 | 3752703 | Missense_Mutation | SNP | G | G | A | novel | Somatic | R400W | 0.0251 | NM_004886.4 | 983 | 958 | 25 |
| 481-PTsup | DUS3L | 629 | hg19 | chr19 | 5789485 | 5789485 | Missense_Mutation | SNP | G | G | T | novel | Somatic | D211E | 0.029135 | NM_020175.3 | 1062 | 1031 | 31 |
| 481-PTsup | MYO1F | 650 | hg19 | chr19 | 8609331 | 8609331 | Missense_Mutation | SNP | C | C | T | novel | Somatic | M458I | 0.023992 | NM_012335.4 | 1040 | 1015 | 25 |
| 481-PTsup | MUC16 | 81 | hg19 | chr19 | 9089582 | 9089582 | Missense_Mutation | SNP | G | G | A | novel | Somatic | H745Y | 0.202479 | NM_024690.2 | 242 | 193 | 49 |
| 481-PTsup | TMEM221 | 718 | hg19 | chr19 | 17547667 | 17547667 | Missense_Mutation | SNP | G | G | A | novel | Somatic | S159L | 0.16101 | NM_001190844.2 | 1255 | 1051 | 204 |
| 481-PTsup | TMEM59L | 727 | hg19 | chr19 | 18727861 | 18727861 | Missense_Mutation | SNP | G | G | A | novel | Somatic | V205M | 0.216667 | NM_012109.3 | 120 | 94 | 26 |
| 481-PTsup | HOMER3 | 730 | hg19 | chr19 | 19043812 | 19043812 | Nonsense_Mutation | SNP | C | C | A | novel | Somatic | E152Ter | 0.274809 | NM_001145722.2 | 1042 | 754 | 288 |
| 481-PTsup | ZNF429 | 750 | hg19 | chr19 | 21720557 | 21720557 | Missense_Mutation | SNP | A | A | G | novel | Somatic | K568E | 0.062822 | NM_001001415.4 | 970 | 909 | 61 |
| 481-PTsup | ZNF43 | 752 | hg19 | chr19 | 21990758 | 21990758 | Missense_Mutation | SNP | G | G | A | novel | Somatic | S694F | 0.277388 | NM_003423.4 | 1848 | 1334 | 514 |
| 481-PTsup | ZNF529 | 867 | hg19 | chr19 | 37045605 | 37045605 | Missense_Mutation | SNP | T | T | C | novel | Somatic | M68V | 0.257576 | NM_020951.5 | 198 | 147 | 51 |
| 481-PTsup | ZNF568 | 870 | hg19 | chr19 | 37488389 | 37488389 | Missense_Mutation | SNP | C | C | A | novel | Somatic | A599D | 0.271028 | NM_001204838.1 | 107 | 78 | 29 |
| 481-PTsup | ZNF569 | 874 | hg19 | chr19 | 37917266 | 37917266 | Missense_Mutation | SNP | G | G | C | novel | Somatic | F10L | 0.284404 | NM_152484.3 | 109 | 78 | 31 |
| 481-PTsup | DYRK1B | 892 | hg19 | chr19 | 40316896 | 40316896 | Missense_Mutation | SNP | C | C | T | novel | Somatic | R481Q | 0.051064 | NM_004714.3 | 232 | 220 | 12 |
| 481-PTsup | ZNF234 | 925 | hg19 | chr19 | 44660987 | 44660987 | Missense_Mutation | SNP | C | C | A | novel | Somatic | S273Y | 0.246914 | NM_006630.3 | 162 | 122 | 40 |
| 481-PTsup | RASIP1 | 960 | hg19 | chr19 | 49232588 | 49232588 | Missense_Mutation | SNP | C | C | G | novel | Somatic | G480A | 0.054324 | NM_017805.3 | 900 | 851 | 49 |
| 481-PTsup | KCNC3 | 972 | hg19 | chr19 | 50826237 | 50826237 | Missense_Mutation | SNP | C | C | T | novel | Somatic | R658Q | 0.033688 | NM_004977.3 | 1122 | 1084 | 38 |
| 481-PTsup | SYT3 | 975 | hg19 | chr19 | 51133094 | 51133094 | Missense_Mutation | SNP | G | G | A | novel | Somatic | P337S | 0.27907 | NM_032298.2 | 556 | 400 | 156 |
| 481-PTsup | ZNF766 | 987 | hg19 | chr19 | 52794308 | 52794308 | Missense_Mutation | SNP | C | C | G | novel | Somatic | Q422E | 0.195652 | NM_001010851.3 | 230 | 185 | 45 |
| 481-PTsup | ZNF701 | 123 | hg19 | chr19 | 53077411 | 53077411 | Missense_Mutation | SNP | T | T | C | novel | Somatic | L4P | 0.03012 | NM_018260.2 | 332 | 322 | 10 |
| 481-PTsup | ZNF765 | 996 | hg19 | chr19 | 53911722 | 53911722 | Missense_Mutation | SNP | A | A | G | novel | Somatic | D305G | 0.031646 | NM_001040185.3 | 472 | 457 | 15 |
| 481-PTsup | ZNF765-ZNF761 | 996 | hg19 | chr19 | 53959420 | 53959420 | Missense_Mutation | SNP | G | G | T | novel | Somatic | K553N | 0.05 | NM_001350496.2 | 279 | 265 | 14 |
| 481-PTsup | ZNF552 | 1029 | hg19 | chr19 | 58319454 | 58319454 | Missense_Mutation | SNP | G | G | C | novel | Somatic | S393C | 0.257426 | NM_024762.3 | 202 | 150 | 52 |
| 481-PTsup | ZSCAN1 | 1031 | hg19 | chr19 | 58549368 | 58549368 | Missense_Mutation | SNP | C | C | T | novel | Somatic | A55V | 0.087452 | NM_182572.4 | 1041 | 949 | 92 |
| 481-PTsup | ZSCAN18 | 1032 | hg19 | chr19 | 58596071 | 58596071 | Missense_Mutation | SNP | G | G | T | novel | Somatic | P561H | 0.029364 | NM_001145542.1 | 613 | 595 | 18 |
| 481-PTsup | SLC27A5 | 1035 | hg19 | chr19 | 59022950 | 59022950 | Nonsense_Mutation | SNP | G | G | A | novel | Somatic | R125Ter | 0.419038 | NM_012254.3 | 1012 | 585 | 427 |
| 481-PTsup | CST1 | 766 | hg19 | chr20 | 23731367 | 23731367 | Missense_Mutation | SNP | C | C | T | novel | Somatic | R46H | 0.096154 | NM_001898.2 | 259 | 234 | 25 |
| 481-PTsup | ZNF341 | 103 | hg19 | chr20 | 32341114 | 32341114 | Missense_Mutation | SNP | G | G | A | novel | Somatic | R209H | 0.166335 | NM_001282933.2 | 995 | 828 | 167 |
| 481-PTsup | GGT7 | 840 | hg19 | chr20 | 33449337 | 33449337 | Nonsense_Mutation | SNP | G | G | A | novel | Somatic | R196Ter | 0.236025 | NM_178026.3 | 161 | 123 | 38 |
| 481-PTsup | EIF6 | 843 | hg19 | chr20 | 33868540 | 33868540 | Missense_Mutation | SNP | G | G | A | novel | Somatic | R96W | 0.270893 | NM_002212.4 | 345 | 251 | 94 |
| 481-PTsup | SYCP2 | 128 | hg19 | chr20 | 58467046 | 58467046 | Frame_Shift_Ins | INS | - | - | T | novel | Somatic | M788NfsTer23 | 0.075342 | NM_014258.4 | 146 | 135 | 11 |
| 481-PTsup | TPTE | 668 | hg19 | chr21 | 10944774 | 10944774 | Missense_Mutation | SNP | A | A | T | novel | Somatic | F154I | 0.073955 | NM_199261.3 | 1244 | 1152 | 92 |
| 481-PTsup | IL2RB | 871 | hg19 | chr22 | 37533724 | 37533724 | Missense_Mutation | SNP | C | C | T | novel | Somatic | R147K | 0.085427 | NM_000878.5 | 199 | 182 | 17 |
| 481-PTsup | PDXP | 875 | hg19 | chr22 | 38055213 | 38055213 | Missense_Mutation | SNP | C | C | T | novel | Somatic | R142C | 0.181034 | NM_020315.5 | 229 | 187 | 42 |
| 481-PTsup | EP300 | 112 | hg19 | chr22 | 41513449 | 41513449 | Missense_Mutation | SNP | T | T | A | novel | Somatic | I118K | 0.385321 | NM_001429.4 | 217 | 133 | 84 |
| 481-PTsup | ATXN10 | 14 | hg19 | chr22 | 46114328 | 46114328 | Missense_Mutation | SNP | C | C | T | novel | Somatic | P228L | 0.12766 | NM_013236.4 | 278 | 242 | 36 |
| 481-PTsup | BRD1 | 120 | hg19 | chr22 | 50167938 | 50167938 | Missense_Mutation | SNP | C | C | T | novel | Somatic | M1171I | 0.16943 | NM_001304808.3 | 1385 | 1150 | 235 |
| 481-PTsup | CFAP47 | 859 | hg19 | chrX | 36403047 | 36403047 | Missense_Mutation | SNP | C | C | T | novel | Somatic | R3164C | 0.657143 | NM_001304548.2 | 70 | 24 | 46 |
| 481-PTsup | HDX | 152 | hg19 | chrX | 83723676 | 83723676 | Nonsense_Mutation | SNP | G | G | T | novel | Somatic | S352Ter | 0.333333 | NM_001177479.2 | 78 | 52 | 26 |
| 481-PTsup | AMMECR1 | 177 | hg19 | chrX | 109561119 | 109561119 | Missense_Mutation | SNP | C | C | T | novel | Somatic | G61R | 0.516732 | NM_015365.3 | 1015 | 490 | 525 |
| 481-PTsup | DCAF12L2 | 1540 | hg19 | chrX | 125299340 | 125299340 | Missense_Mutation | SNP | G | G | A | novel | Somatic | R190C | 0.213319 | NM_001013628.3 | 1914 | 1504 | 410 |
| 481-PTdeep | C1orf167 | 675 | hg19 | chr1 | 11826885 | 11826885 | Missense_Mutation | SNP | G | G | T | novel | Somatic | A348S | 0.105 | NM_001010881.2 | 199 | 178 | 21 |
| 481-PTdeep | RHCE | 97 | hg19 | chr1 | 25715490 | 25715490 | Missense_Mutation | SNP | T | T | C | novel | Somatic | I306V | 0.059783 | NM_020485.5 | 184 | 173 | 11 |
| 481-PTdeep | FOXD2 | 950 | hg19 | chr1 | 47903913 | 47903913 | Frame_Shift_Del | DEL | G | G | - | novel | Somatic | E38SfsTer30 | 0.026801 | NM_004474.4 | 596 | 580 | 16 |
| 481-PTdeep | COL24A1 | 155 | hg19 | chr1 | 86289269 | 86289269 | Missense_Mutation | SNP | C | C | T | novel | Somatic | E1247K | 0.111111 | NM_152890.7 | 135 | 120 | 15 |
| 481-PTdeep | KCND3 | 180 | hg19 | chr1 | 112524505 | 112524505 | Missense_Mutation | SNP | C | C | T | novel | Somatic | V282M | 0.136905 | NM_004980.4 | 503 | 434 | 69 |
| 481-PTdeep | ADAMTSL4 | 1733 | hg19 | chr1 | 150525554 | 150525554 | Missense_Mutation | SNP | C | C | T | novel | Somatic | R87W | 0.157761 | NM_001288608.1 | 393 | 331 | 62 |
| 481-PTdeep | NAV1 | 265 | hg19 | chr1 | 201786380 | 201786380 | Frame_Shift_Del | DEL | C | C | - | novel | Somatic | D1835EfsTer12 | 0.052402 | NM_020443.4 | 228 | 216 | 12 |
| 481-PTdeep | DNAH14 | 2302 | hg19 | chr1 | 225152201 | 225152201 | Missense_Mutation | SNP | A | A | G | novel | Somatic | I130V | 0.059172 | NM_001367479.1 | 169 | 159 | 10 |
| 481-PTdeep | ARV1 | 2348 | hg19 | chr1 | 231132905 | 231132905 | Missense_Mutation | SNP | G | G | A | novel | Somatic | V238M | 0.123288 | NM_022786.3 | 146 | 128 | 18 |
| 481-PTdeep | LPIN1 | 84 | hg19 | chr2 | 11955237 | 11955237 | Missense_Mutation | SNP | G | G | C | novel | Somatic | C807S | 0.190083 | NM_001261428.3 | 121 | 98 | 23 |
| 481-PTdeep | FAM228B | 96 | hg19 | chr2 | 24387082 | 24387082 | Missense_Mutation | SNP | C | C | G | novel | Somatic | S270C | 0.075862 | NM_001145710.2 | 145 | 134 | 11 |
| 481-PTdeep | PKDCC | 907 | hg19 | chr2 | 42275428 | 42275428 | Missense_Mutation | SNP | C | C | T | novel | Somatic | S30L | 0.105548 | NM_138370.3 | 737 | 659 | 78 |
| 481-PTdeep | C2orf78 | 1149 | hg19 | chr2 | 74042938 | 74042938 | Missense_Mutation | SNP | G | G | C | novel | Somatic | E530Q | 0.112426 | NM_001080474.2 | 169 | 150 | 19 |
| 481-PTdeep | RGPD3 | 1401 | hg19 | chr2 | 107049414 | 107049414 | Missense_Mutation | SNP | G | G | A | novel | Somatic | R816C | 0.032432 | NM_001144013.1 | 370 | 358 | 12 |
| 481-PTdeep | ARHGAP15 | 210 | hg19 | chr2 | 144244956 | 144244956 | Missense_Mutation | SNP | C | C | G | novel | Somatic | H240D | 0.118644 | NM_018460.4 | 118 | 104 | 14 |
| 481-PTdeep | TTN | 1955 | hg19 | chr2 | 179489297 | 179489297 | Missense_Mutation | SNP | C | C | T | novel | Somatic | G14904S | 0.093567 | NM_001267550.2 | 171 | 155 | 16 |
| 481-PTdeep | TTN | 1955 | hg19 | chr2 | 179588247 | 179588247 | Missense_Mutation | SNP | G | G | T | novel | Somatic | L7194M | 0.070707 | NM_001267550.2 | 198 | 184 | 14 |
| 481-PTdeep | FRZB | 1986 | hg19 | chr2 | 183707206 | 183707206 | Missense_Mutation | SNP | C | C | A | novel | Somatic | V198F | 0.244186 | NM_001463.4 | 86 | 65 | 21 |
| 481-PTdeep | SLC4A3 | 2267 | hg19 | chr2 | 220504227 | 220504227 | Missense_Mutation | SNP | G | G | A | novel | Somatic | R1016Q | 0.109053 | NM_005070.4 | 483 | 430 | 53 |
| 481-PTdeep | ECEL1 | 2365 | hg19 | chr2 | 233348779 | 233348779 | Missense_Mutation | SNP | G | G | A | novel | Somatic | R447C | 0.122677 | NM_004826.4 | 269 | 236 | 33 |
| 481-PTdeep | SCN5A | 109 | hg19 | chr3 | 38622603 | 38622603 | Missense_Mutation | SNP | G | G | A | novel | Somatic | T1016M | 0.028646 | NM_001099404.1 | 763 | 741 | 22 |
| 481-PTdeep | CDC25A | 952 | hg19 | chr3 | 48215868 | 48215868 | Missense_Mutation | SNP | G | G | A | novel | Somatic | S279F | 0.130435 | NM_001789.3 | 91 | 79 | 12 |
| 481-PTdeep | JAKMIP1 | 78 | hg19 | chr4 | 6087158 | 6087158 | Missense_Mutation | SNP | T | T | G | novel | Somatic | M275L | 0.173077 | NM_001099433.2 | 104 | 86 | 18 |
| 481-PTdeep | ADH1B | 1349 | hg19 | chr4 | 100232705 | 100232705 | Missense_Mutation | SNP | G | G | A | novel | Somatic | R313C | 0.12 | NM_000668.6 | 200 | 176 | 24 |
| 481-PTdeep | ZCCHC9 | 1199 | hg19 | chr5 | 80600551 | 80600551 | Intron | SNP | A | A | G | novel | Somatic |  | 0.087719 | NM_032280.2 | 113 | 103 | 10 |
| 481-PTdeep | PCDHA2 | 1654 | hg19 | chr5 | 140174842 | 140174842 | Missense_Mutation | SNP | G | G | A | novel | Somatic | R98Q | 0.04757 | NM_018905.3 | 966 | 920 | 46 |
| 481-PTdeep | GNB2 | 1350 | hg19 | chr7 | 100275225 | 100275225 | Nonsense_Mutation | SNP | C | C | A | novel | Somatic | Y124Ter | 0.215596 | NM_005273.4 | 436 | 342 | 94 |
| 481-PTdeep | SRRT | 1351 | hg19 | chr7 | 100479332 | 100479332 | Frame_Shift_Del | DEL | G | G | - | novel | Somatic | G104VfsTer45 | 0.071895 | NM_015908.6 | 153 | 142 | 11 |
| 481-PTdeep | DLGAP2 | 9 | hg19 | chr8 | 1616767 | 1616767 | Missense_Mutation | SNP | C | C | T | novel | Somatic | R695W | 0.021991 | NM_001346810.2 | 861 | 842 | 19 |
| 481-PTdeep | CDH17 | 163 | hg19 | chr8 | 95201495 | 95201495 | Missense_Mutation | SNP | C | C | G | novel | Somatic | E24Q | 0.1875 | NM_004063.4 | 128 | 104 | 24 |
| 481-PTdeep | CYC1 | 1692 | hg19 | chr8 | 145151988 | 145151988 | Missense_Mutation | SNP | G | G | A | novel | Somatic | R275H | 0.125 | NM_001916.5 | 144 | 126 | 18 |
| 481-PTdeep | TONSL | 1696 | hg19 | chr8 | 145666438 | 145666438 | Nonsense_Mutation | SNP | G | G | A | novel | Somatic | Q308Ter | 0.1639 | NM_013432.5 | 482 | 403 | 79 |
| 481-PTdeep | COMMD5 | 1699 | hg19 | chr8 | 146076704 | 146076704 | Missense_Mutation | SNP | G | G | A | novel | Somatic | A7V | 0.0317 | NM_014066.4 | 345 | 334 | 11 |
| 481-PTdeep | DOCK8 | 73 | hg19 | chr9 | 441906 | 441906 | Missense_Mutation | SNP | G | G | A | novel | Somatic | R1796Q | 0.092199 | NM_203447.3 | 141 | 128 | 13 |
| 481-PTdeep | CDKN2A | 752 | hg19 | chr9 | 21971036 | 21971036 | Missense_Mutation | SNP | C | C | T | novel | Somatic | D108N | 0.25831 | NM_000077.4 | 1051 | 779 | 272 |
| 481-PTdeep | CDC26 | 1470 | hg19 | chr9 | 116034777 | 116034777 | Missense_Mutation | SNP | C | C | G | novel | Somatic | R4P | 0.292453 | NM_139286.4 | 106 | 75 | 31 |
| 481-PTdeep | RNF224 | 1654 | hg19 | chr9 | 140123254 | 140123254 | Missense_Mutation | SNP | C | C | T | novel | Somatic | R63C | 0.069091 | NM_001190228.1 | 821 | 764 | 57 |
| 481-PTdeep | EPS8L2 | 590 | hg19 | chr11 | 725827 | 725827 | Missense_Mutation | SNP | G | G | A | novel | Somatic | A554T | 0.109231 | NM_022772.4 | 643 | 572 | 71 |
| 481-PTdeep | MARK2 | 1070 | hg19 | chr11 | 63673579 | 63673579 | Missense_Mutation | SNP | G | G | T | novel | Somatic | A652S | 0.090508 | NM_001039469.2 | 453 | 412 | 41 |
| 481-PTdeep | NRXN2 | 1076 | hg19 | chr11 | 64421194 | 64421194 | Missense_Mutation | SNP | T | T | C | novel | Somatic | D797G | 0.111111 | NM_015080.4 | 257 | 228 | 29 |
| 481-PTdeep | CHKA | 1102 | hg19 | chr11 | 67842184 | 67842184 | Splice_Region | SNP | C | C | A | novel | Somatic | P210= | 0.413793 | NM_001277.3 | 115 | 67 | 48 |
| 481-PTdeep | ARHGEF17 | 1142 | hg19 | chr11 | 73073283 | 73073283 | Missense_Mutation | SNP | C | C | T | novel | Somatic | R1565W | 0.084694 | NM_014786.4 | 970 | 887 | 83 |
| 481-PTdeep | C11orf71 | 1456 | hg19 | chr11 | 114270974 | 114270974 | Missense_Mutation | SNP | C | C | G | novel | Somatic | R27T | 0.106312 | NM_019021.4 | 301 | 269 | 32 |
| 481-PTdeep | ACRBP | 636 | hg19 | chr12 | 6754383 | 6754383 | Splice_Region | SNP | C | C | G | novel | Somatic |  | 0.131387 | NM_032489.3 | 137 | 119 | 18 |
| 481-PTdeep | CD163L1 | 642 | hg19 | chr12 | 7531771 | 7531771 | In_Frame_Del | DEL | TCAGCAATGTTC | TCAGCAATGTTC | - | novel | Somatic | M721_E725delinsI | 0.076433 | NM_174941.5 | 157 | 145 | 12 |
| 481-PTdeep | REP15 | 797 | hg19 | chr12 | 27849796 | 27849796 | Missense_Mutation | SNP | A | A | G | novel | Somatic | N101D | 0.702128 | NM_001029874.3 | 66 | 0 | 66 |
| 481-PTdeep | SMARCC2 | 1016 | hg19 | chr12 | 56572825 | 56572825 | Frame_Shift_Del | DEL | TCTT | TCTT | - | novel | Somatic | K365TfsTer12 | 0.154545 | NM_001330288.2 | 110 | 93 | 17 |
| 481-PTdeep | NXPH4 | 1024 | hg19 | chr12 | 57619423 | 57619423 | Missense_Mutation | SNP | T | T | G | novel | Somatic | F274V | 0.14121 | NM_007224.4 | 341 | 292 | 49 |
| 481-PTdeep | TRHDE | 142 | hg19 | chr12 | 72667063 | 72667063 | Missense_Mutation | SNP | G | G | A | novel | Somatic | V169M | 0.084127 | NM_013381.2 | 629 | 576 | 53 |
| 481-PTdeep | SH2B3 | 1438 | hg19 | chr12 | 111856595 | 111856595 | Missense_Mutation | SNP | C | C | T | novel | Somatic | R216C | 0.113095 | NM_005475.3 | 502 | 445 | 57 |
| 481-PTdeep | OAS3 | 181 | hg19 | chr12 | 113405938 | 113405938 | Missense_Mutation | SNP | G | G | C | novel | Somatic | K1021N | 0.092593 | NM_006187.4 | 108 | 98 | 10 |
| 481-PTdeep | ORAI1 | 1516 | hg19 | chr12 | 122079191 | 122079191 | Missense_Mutation | SNP | G | G | C | novel | Somatic | G183A | 0.026455 | NM_032790.3 | 756 | 736 | 20 |
| 481-PTdeep | ORAI1 | 1516 | hg19 | chr12 | 122079446 | 122079446 | Missense_Mutation | SNP | G | G | A | novel | Somatic | R268Q | 0.037736 | NM_032790.3 | 474 | 456 | 18 |
| 481-PTdeep | SHISA2 | 788 | hg19 | chr13 | 26624876 | 26624876 | Missense_Mutation | SNP | C | C | T | novel | Somatic | E80K | 0.10992 | NM_001007538.1 | 733 | 651 | 82 |
| 481-PTdeep | SYNDIG1L | 1156 | hg19 | chr14 | 74876246 | 74876246 | Missense_Mutation | SNP | A | A | G | novel | Somatic | Y68H | 0.126801 | NM_001105579.2 | 338 | 294 | 44 |
| 481-PTdeep | FLRT2 | 1241 | hg19 | chr14 | 86089539 | 86089539 | Missense_Mutation | SNP | G | G | A | novel | Somatic | V561I | 0.145833 | NM_001346143.2 | 144 | 123 | 21 |
| 481-PTdeep | HDC | 970 | hg19 | chr15 | 50546377 | 50546377 | Missense_Mutation | SNP | G | G | C | novel | Somatic | L224V | 0.223214 | NM_002112.4 | 112 | 87 | 25 |
| 481-PTdeep | HERC1 | 16 | hg19 | chr15 | 64005673 | 64005673 | Missense_Mutation | SNP | G | G | A | novel | Somatic | R1448W | 0.174419 | NM_003922.4 | 86 | 71 | 15 |
| 481-PTdeep | PKD1 | 601 | hg19 | chr16 | 2161581 | 2161581 | Missense_Mutation | SNP | G | G | A | novel | Somatic | T1196M | 0.028926 | NM_001009944.3 | 724 | 703 | 21 |
| 481-PTdeep | DNASE1L2 | 602 | hg19 | chr16 | 2287753 | 2287753 | Splice_Region | DEL | CCGCGGCGC | CCGCGGCGC | - | novel | Somatic |  | 0.068901 | NM_001374.3 | 1067 | 993 | 74 |
| 481-PTdeep | TRAP1 | 613 | hg19 | chr16 | 3712953 | 3712953 | Missense_Mutation | SNP | G | G | A | novel | Somatic | S575L | 0.071006 | NM_016292.3 | 169 | 157 | 12 |
| 481-PTdeep | CIITA | 83 | hg19 | chr16 | 11001325 | 11001325 | Missense_Mutation | SNP | A | A | T | novel | Somatic | E659V | 0.026816 | NM_000246.3 | 873 | 849 | 24 |
| 481-PTdeep | ITGAX | 824 | hg19 | chr16 | 31383014 | 31383014 | Missense_Mutation | SNP | C | C | A | novel | Somatic | A690D | 0.068249 | NM_000887.5 | 336 | 313 | 23 |
| 481-PTdeep | ADAMTS18 | 146 | hg19 | chr16 | 77396098 | 77396098 | Missense_Mutation | SNP | C | C | A | novel | Somatic | A374S | 0.153285 | NM_199355.4 | 137 | 116 | 21 |
| 481-PTdeep | OR1E1 | 610 | hg19 | chr17 | 3301479 | 3301479 | Missense_Mutation | SNP | C | C | T | novel | Somatic | V76M | 0.063492 | NM_003553.3 | 189 | 177 | 12 |
| 481-PTdeep | DRC3 | 721 | hg19 | chr17 | 17910398 | 17910398 | Missense_Mutation | SNP | C | C | G | novel | Somatic | I421M | 0.077922 | NM_031294.4 | 769 | 709 | 60 |
| 481-PTdeep | SEZ6 | 793 | hg19 | chr17 | 27290989 | 27290989 | Missense_Mutation | SNP | C | C | T | novel | Somatic | V411I | 0.064516 | NM_178860.5 | 155 | 145 | 10 |
| 481-PTdeep | EFCAB5 | 12 | hg19 | chr17 | 28269284 | 28269284 | Missense_Mutation | SNP | G | G | T | novel | Somatic | R23S | 0.126506 | NM_198529.4 | 164 | 143 | 21 |
| 481-PTdeep | FBXO47 | 108 | hg19 | chr17 | 37111137 | 37111137 | Missense_Mutation | SNP | C | C | G | novel | Somatic | G160A | 0.15942 | NM_001008777.3 | 138 | 116 | 22 |
| 481-PTdeep | HOXB9 | 941 | hg19 | chr17 | 46703139 | 46703139 | Missense_Mutation | SNP | C | C | T | novel | Somatic | E165K | 0.15736 | NM_024017.5 | 394 | 332 | 62 |
| 481-PTdeep | LAMA1 | 79 | hg19 | chr18 | 6959455 | 6959455 | Missense_Mutation | SNP | C | C | T | novel | Somatic | E2555K | 0.089041 | NM_005559.4 | 145 | 132 | 13 |
| 481-PTdeep | ABCA7 | 9 | hg19 | chr19 | 1058878 | 1058878 | Missense_Mutation | SNP | G | G | A | novel | Somatic | R1780Q | 0.063694 | NM_019112.4 | 157 | 147 | 10 |
| 481-PTdeep | TJP3 | 613 | hg19 | chr19 | 3740743 | 3740743 | Nonsense_Mutation | SNP | C | C | T | novel | Somatic | R609Ter | 0.121372 | NM_001267560.2 | 751 | 659 | 92 |
| 481-PTdeep | MUC16 | 81 | hg19 | chr19 | 9070548 | 9070548 | Missense_Mutation | SNP | G | G | A | novel | Somatic | S5633F | 0.152941 | NM_024690.2 | 84 | 71 | 13 |
| 481-PTdeep | TMEM221 | 718 | hg19 | chr19 | 17547667 | 17547667 | Missense_Mutation | SNP | G | G | A | novel | Somatic | S159L | 0.084046 | NM_001190844.2 | 697 | 638 | 59 |
| 481-PTdeep | TMEM59L | 727 | hg19 | chr19 | 18727861 | 18727861 | Missense_Mutation | SNP | G | G | A | novel | Somatic | V205M | 0.096 | NM_012109.3 | 124 | 112 | 12 |
| 481-PTdeep | HOMER3 | 730 | hg19 | chr19 | 19043812 | 19043812 | Nonsense_Mutation | SNP | C | C | A | novel | Somatic | E152Ter | 0.121827 | NM_001145722.2 | 394 | 346 | 48 |
| 481-PTdeep | ZNF43 | 752 | hg19 | chr19 | 21990758 | 21990758 | Missense_Mutation | SNP | G | G | A | novel | Somatic | S694F | 0.125 | NM_003423.4 | 885 | 774 | 111 |
| 481-PTdeep | ZNF529 | 867 | hg19 | chr19 | 37045605 | 37045605 | Missense_Mutation | SNP | T | T | C | novel | Somatic | M68V | 0.082645 | NM_020951.5 | 121 | 111 | 10 |
| 481-PTdeep | ZNF568 | 870 | hg19 | chr19 | 37488389 | 37488389 | Missense_Mutation | SNP | C | C | A | novel | Somatic | A599D | 0.125 | NM_001204838.1 | 104 | 91 | 13 |
| 481-PTdeep | ZNF569 | 874 | hg19 | chr19 | 37917266 | 37917266 | Missense_Mutation | SNP | G | G | C | novel | Somatic | F10L | 0.144068 | NM_152484.3 | 118 | 101 | 17 |
| 481-PTdeep | TMEM160 | 947 | hg19 | chr19 | 47551797 | 47551797 | Nonsense_Mutation | SNP | G | G | A | novel | Somatic | Q26Ter | 0.024943 | NM_017854.2 | 440 | 429 | 11 |
| 481-PTdeep | RASIP1 | 960 | hg19 | chr19 | 49232588 | 49232588 | Missense_Mutation | SNP | C | C | G | novel | Somatic | G480A | 0.049745 | NM_017805.3 | 783 | 744 | 39 |
| 481-PTdeep | C19orf73 | 963 | hg19 | chr19 | 49621975 | 49621975 | Nonsense_Mutation | SNP | A | A | T | novel | Somatic | L102Ter | 0.026738 | NM_018111.3 | 374 | 364 | 10 |
| 481-PTdeep | SYT3 | 975 | hg19 | chr19 | 51133094 | 51133094 | Missense_Mutation | SNP | G | G | A | novel | Somatic | P337S | 0.105802 | NM_032298.2 | 293 | 262 | 31 |
| 481-PTdeep | ZNF552 | 1029 | hg19 | chr19 | 58319454 | 58319454 | Missense_Mutation | SNP | G | G | C | novel | Somatic | S393C | 0.080745 | NM_024762.3 | 161 | 148 | 13 |
| 481-PTdeep | ZSCAN1 | 1031 | hg19 | chr19 | 58549368 | 58549368 | Missense_Mutation | SNP | C | C | T | novel | Somatic | A55V | 0.068038 | NM_182572.4 | 1258 | 1172 | 86 |
| 481-PTdeep | SLC27A5 | 1035 | hg19 | chr19 | 59022950 | 59022950 | Nonsense_Mutation | SNP | G | G | A | novel | Somatic | R125Ter | 0.115178 | NM_012254.3 | 927 | 820 | 107 |
| 481-PTdeep | KAT14 | 723 | hg19 | chr20 | 18123477 | 18123477 | Missense_Mutation | SNP | C | C | T | novel | Somatic | S58F | 0.040486 | NM_020536.4 | 245 | 235 | 10 |
| 481-PTdeep | ZNF341 | 103 | hg19 | chr20 | 32341114 | 32341114 | Missense_Mutation | SNP | G | G | A | novel | Somatic | R209H | 0.112875 | NM_001282933.2 | 565 | 501 | 64 |
| 481-PTdeep | GGT7 | 840 | hg19 | chr20 | 33449337 | 33449337 | Nonsense_Mutation | SNP | G | G | A | novel | Somatic | R196Ter | 0.12381 | NM_178026.3 | 105 | 92 | 13 |
| 481-PTdeep | EIF6 | 843 | hg19 | chr20 | 33868540 | 33868540 | Missense_Mutation | SNP | G | G | A | novel | Somatic | R96W | 0.09633 | NM_002212.4 | 216 | 195 | 21 |
| 481-PTdeep | KCNS1 | 918 | hg19 | chr20 | 43726465 | 43726465 | Missense_Mutation | SNP | G | G | C | novel | Somatic | F316L | 0.0251 | NM_002251.5 | 995 | 970 | 25 |
| 481-PTdeep | PDXP | 875 | hg19 | chr22 | 38055213 | 38055213 | Missense_Mutation | SNP | C | C | T | novel | Somatic | R142C | 0.096774 | NM_020315.5 | 153 | 138 | 15 |
| 481-PTdeep | EP300 | 112 | hg19 | chr22 | 41513449 | 41513449 | Missense_Mutation | SNP | T | T | A | novel | Somatic | I118K | 0.192053 | NM_001429.4 | 151 | 122 | 29 |
| 481-PTdeep | BRD1 | 120 | hg19 | chr22 | 50167938 | 50167938 | Missense_Mutation | SNP | C | C | T | novel | Somatic | M1171I | 0.078385 | NM_001304808.3 | 842 | 776 | 66 |
| 481-PTdeep | CFAP47 | 859 | hg19 | chrX | 36403047 | 36403047 | Missense_Mutation | SNP | C | C | T | novel | Somatic | R3164C | 0.5 | NM_001304548.2 | 40 | 20 | 20 |
| 481-PTdeep | HDX | 152 | hg19 | chrX | 83723676 | 83723676 | Nonsense_Mutation | SNP | G | G | T | novel | Somatic | S352Ter | 0.20339 | NM_001177479.2 | 59 | 47 | 12 |
| 481-PTdeep | AMMECR1 | 177 | hg19 | chrX | 109561119 | 109561119 | Missense_Mutation | SNP | C | C | T | novel | Somatic | G61R | 0.234409 | NM_015365.3 | 465 | 356 | 109 |
| 481-PTdeep | DCAF12L2 | 1540 | hg19 | chrX | 125299340 | 125299340 | Missense_Mutation | SNP | G | G | A | novel | Somatic | R190C | 0.06679 | NM_001013628.3 | 1078 | 1006 | 72 |
| 481-LNmet | C1orf167 | 675 | hg19 | chr1 | 11826885 | 11826885 | Missense_Mutation | SNP | G | G | T | novel | Somatic | A348S | 0.210526 | NM_001010881.2 | 470 | 370 | 100 |
| 481-LNmet | EPHB2 | 11 | hg19 | chr1 | 23239090 | 23239090 | Missense_Mutation | SNP | G | G | T | novel | Somatic | M950I | 0.198795 | NM_017449.4 | 166 | 133 | 33 |
| 481-LNmet | RHCE | 97 | hg19 | chr1 | 25715490 | 25715490 | Missense_Mutation | SNP | T | T | C | novel | Somatic | I306V | 0.15534 | NM_020485.5 | 205 | 173 | 32 |
| 481-LNmet | COL24A1 | 155 | hg19 | chr1 | 86289269 | 86289269 | Missense_Mutation | SNP | C | C | T | novel | Somatic | E1247K | 0.197183 | NM_152890.7 | 211 | 169 | 42 |
| 481-LNmet | KCND3 | 180 | hg19 | chr1 | 112524505 | 112524505 | Missense_Mutation | SNP | C | C | T | novel | Somatic | V282M | 0.20354 | NM_004980.4 | 678 | 540 | 138 |
| 481-LNmet | ADAMTSL4 | 1733 | hg19 | chr1 | 150525554 | 150525554 | Missense_Mutation | SNP | C | C | T | novel | Somatic | R87W | 0.323276 | NM_001288608.1 | 923 | 623 | 300 |
| 481-LNmet | GLMP | 1777 | hg19 | chr1 | 156264149 | 156264149 | Splice_Region | SNP | G | G | A | novel | Somatic |  | 0.191667 | NM_144580.3 | 120 | 97 | 23 |
| 481-LNmet | COPA | 225 | hg19 | chr1 | 160261988 | 160261988 | Nonsense_Mutation | SNP | C | C | T | novel | Somatic | W987Ter | 0.153226 | NM_004371.4 | 124 | 105 | 19 |
| 481-LNmet | MROH9 | 29 | hg19 | chr1 | 170983294 | 170983294 | Missense_Mutation | SNP | G | G | A | novel | Somatic | E545K | 0.152439 | NM_001163629.1 | 162 | 137 | 25 |
| 481-LNmet | NAV1 | 265 | hg19 | chr1 | 201786380 | 201786380 | Frame_Shift_Del | DEL | C | C | - | novel | Somatic | D1835EfsTer12 | 0.167147 | NM_020443.4 | 346 | 288 | 58 |
| 481-LNmet | NVL | 2297 | hg19 | chr1 | 224424202 | 224424202 | Splice_Region | DEL | ACTT | ACTT | - | novel | Somatic |  | 0.178947 | NM_002533.4 | 95 | 78 | 17 |
| 481-LNmet | DNAH14 | 2302 | hg19 | chr1 | 225152201 | 225152201 | Missense_Mutation | SNP | A | A | G | novel | Somatic | I130V | 0.136364 | NM_001367479.1 | 308 | 266 | 42 |
| 481-LNmet | ARV1 | 2348 | hg19 | chr1 | 231132905 | 231132905 | Missense_Mutation | SNP | G | G | A | novel | Somatic | V238M | 0.178082 | NM_022786.3 | 219 | 180 | 39 |
| 481-LNmet | LPIN1 | 84 | hg19 | chr2 | 11955237 | 11955237 | Missense_Mutation | SNP | G | G | C | novel | Somatic | C807S | 0.20678 | NM_001261428.3 | 294 | 233 | 61 |
| 481-LNmet | FAM228B | 96 | hg19 | chr2 | 24387082 | 24387082 | Missense_Mutation | SNP | C | C | G | novel | Somatic | S270C | 0.12963 | NM_001145710.2 | 216 | 188 | 28 |
| 481-LNmet | ITSN2 | 96 | hg19 | chr2 | 24480817 | 24480817 | Missense_Mutation | SNP | C | C | A | novel | Somatic | R943I | 0.215517 | NM_006277.3 | 232 | 182 | 50 |
| 481-LNmet | DPYSL5 | 98 | hg19 | chr2 | 27121531 | 27121531 | Missense_Mutation | SNP | G | G | A | novel | Somatic | G55E | 0.29927 | NM_020134.4 | 272 | 190 | 82 |
| 481-LNmet | PKDCC | 907 | hg19 | chr2 | 42275428 | 42275428 | Missense_Mutation | SNP | C | C | T | novel | Somatic | S30L | 0.156301 | NM_138370.3 | 1221 | 1030 | 191 |
| 481-LNmet | C2orf78 | 1149 | hg19 | chr2 | 74042938 | 74042938 | Missense_Mutation | SNP | G | G | C | novel | Somatic | E530Q | 0.185022 | NM_001080474.2 | 227 | 185 | 42 |
| 481-LNmet | MAP4K4 | 170 | hg19 | chr2 | 102452436 | 102452436 | Missense_Mutation | SNP | A | A | G | novel | Somatic | K257E | 0.125654 | NM_145686.3 | 191 | 167 | 24 |
| 481-LNmet | IL1R2 | 170 | hg19 | chr2 | 102644697 | 102644697 | Missense_Mutation | SNP | C | C | T | novel | Somatic | T347M | 0.165254 | NM_004633.4 | 235 | 196 | 39 |
| 481-LNmet | ARHGAP15 | 210 | hg19 | chr2 | 144244956 | 144244956 | Missense_Mutation | SNP | C | C | G | novel | Somatic | H240D | 0.126904 | NM_018460.4 | 196 | 171 | 25 |
| 481-LNmet | CCDC173 | 235 | hg19 | chr2 | 170518927 | 170518927 | Missense_Mutation | SNP | C | C | T | novel | Somatic | E228K | 0.094862 | NM_001085447.2 | 252 | 228 | 24 |
| 481-LNmet | TTN | 1955 | hg19 | chr2 | 179489297 | 179489297 | Missense_Mutation | SNP | C | C | T | novel | Somatic | G14904S | 0.07767 | NM_001267550.2 | 309 | 285 | 24 |
| 481-LNmet | FRZB | 1986 | hg19 | chr2 | 183707206 | 183707206 | Missense_Mutation | SNP | C | C | A | novel | Somatic | V198F | 0.543307 | NM_001463.4 | 127 | 58 | 69 |
| 481-LNmet | PMS1 | 2039 | hg19 | chr2 | 190656659 | 190656659 | Missense_Mutation | SNP | G | G | A | novel | Somatic | V42I | 0.154589 | NM_000534.4 | 207 | 175 | 32 |
| 481-LNmet | SLC4A3 | 2267 | hg19 | chr2 | 220504227 | 220504227 | Missense_Mutation | SNP | G | G | A | novel | Somatic | R1016Q | 0.064516 | NM_005070.4 | 526 | 492 | 34 |
| 481-LNmet | ECEL1 | 2365 | hg19 | chr2 | 233348779 | 233348779 | Missense_Mutation | SNP | G | G | A | novel | Somatic | R447C | 0.029536 | NM_004826.4 | 473 | 459 | 14 |
| 481-LNmet | NGLY1 | 97 | hg19 | chr3 | 25805799 | 25805799 | Missense_Mutation | SNP | C | C | G | novel | Somatic | E84Q | 0.142857 | NM_018297.4 | 140 | 120 | 20 |
| 481-LNmet | CDC25A | 952 | hg19 | chr3 | 48215868 | 48215868 | Missense_Mutation | SNP | G | G | A | novel | Somatic | S279F | 0.196262 | NM_001789.3 | 107 | 86 | 21 |
| 481-LNmet | PQLC2L | 27 | hg19 | chr3 | 157318018 | 157318018 | Silent | SNP | C | C | T | novel | Somatic | T113= | 0.146465 | NM_001130002.2 | 198 | 169 | 29 |
| 481-LNmet | ECT2 | 237 | hg19 | chr3 | 172491738 | 172491738 | Missense_Mutation | SNP | A | A | G | novel | Somatic | I492V | 0.064267 | NM_001349094.2 | 389 | 364 | 25 |
| 481-LNmet | JAKMIP1 | 78 | hg19 | chr4 | 6087158 | 6087158 | Missense_Mutation | SNP | T | T | G | novel | Somatic | M275L | 0.230263 | NM_001099433.2 | 152 | 117 | 35 |
| 481-LNmet | SLIT2 | 92 | hg19 | chr4 | 20552515 | 20552515 | Missense_Mutation | SNP | C | C | T | novel | Somatic | S852L | 0.191919 | NM_004787.4 | 99 | 80 | 19 |
| 481-LNmet | ADH1B | 1349 | hg19 | chr4 | 100232705 | 100232705 | Missense_Mutation | SNP | G | G | A | novel | Somatic | R313C | 0.208029 | NM_000668.6 | 274 | 217 | 57 |
| 481-LNmet | ADAD1 | 190 | hg19 | chr4 | 123333817 | 123333817 | Missense_Mutation | SNP | C | C | G | novel | Somatic | L368V | 0.221675 | NM_139243.4 | 202 | 157 | 45 |
| 481-LNmet | ITGA1 | 122 | hg19 | chr5 | 52227884 | 52227884 | Missense_Mutation | SNP | G | G | A | novel | Somatic | E927K | 0.168317 | NM_181501.2 | 101 | 84 | 17 |
| 481-LNmet | SRP19 | 22 | hg19 | chr5 | 112203167 | 112203167 | Missense_Mutation | SNP | G | G | T | novel | Somatic | G123V | 0.05802 | NM_003135.3 | 292 | 275 | 17 |
| 481-LNmet | CCDC69 | 216 | hg19 | chr5 | 150563006 | 150563006 | Missense_Mutation | SNP | C | C | T | novel | Somatic | A295T | 0.086957 | NM_015621.3 | 229 | 209 | 20 |
| 481-LNmet | RANBP17 | 235 | hg19 | chr5 | 170610183 | 170610183 | Frame_Shift_Del | DEL | A | A | - | novel | Somatic | K628NfsTer3 | 0.10989 | NM_022897.5 | 90 | 80 | 10 |
| 481-LNmet | SSR1 | 640 | hg19 | chr6 | 7290149 | 7290149 | Missense_Mutation | SNP | C | C | T | novel | Somatic | R270K | 0.179012 | NM_003144.5 | 162 | 133 | 29 |
| 481-LNmet | ANKRD66 | 941 | hg19 | chr6 | 46726498 | 46726498 | Missense_Mutation | SNP | A | A | G | novel | Somatic | Q199R | 0.053097 | NM_001162435.2 | 225 | 213 | 12 |
| 481-LNmet | GPR6 | 1426 | hg19 | chr6 | 110300794 | 110300794 | Missense_Mutation | SNP | T | T | G | novel | Somatic | L175R | 0.084314 | NM_001286099.1 | 1019 | 933 | 86 |
| 481-LNmet | GPR6 | 1426 | hg19 | chr6 | 110300808 | 110300808 | Missense_Mutation | SNP | G | G | C | novel | Somatic | D180H | 0.084562 | NM_001286099.1 | 1017 | 931 | 86 |
| 481-LNmet | EPB41L2 | 198 | hg19 | chr6 | 131276371 | 131276371 | Missense_Mutation | SNP | C | C | G | novel | Somatic | E193D | 0.173913 | NM_001350299.1 | 299 | 247 | 52 |
| 481-LNmet | FRMD1 | 1870 | hg19 | chr6 | 168457809 | 168457809 | Missense_Mutation | SNP | C | C | T | novel | Somatic | G540R | 0.028786 | NM_024919.5 | 799 | 776 | 23 |
| 481-LNmet | GUSB | 1084 | hg19 | chr7 | 65425965 | 65425965 | Frame_Shift_Del | DEL | TC | TC | - | novel | Somatic | R625IfsTer7 | 0.161017 | NM_000181.4 | 118 | 99 | 19 |
| 481-LNmet | GNB2 | 1350 | hg19 | chr7 | 100275225 | 100275225 | Nonsense_Mutation | SNP | C | C | A | novel | Somatic | Y124Ter | 0.275605 | NM_005273.4 | 537 | 389 | 148 |
| 481-LNmet | SLC26A3 | 1404 | hg19 | chr7 | 107416955 | 107416955 | Missense_Mutation | SNP | C | C | A | novel | Somatic | C540F | 0.141844 | NM_000111.3 | 141 | 121 | 20 |
| 481-LNmet | KMT2C | 27 | hg19 | chr7 | 151843734 | 151843734 | Missense_Mutation | SNP | C | C | G | novel | Somatic | E4661Q | 0.202899 | NM_170606.3 | 137 | 109 | 28 |
| 481-LNmet | TEX15 | 819 | hg19 | chr8 | 30725009 | 30725009 | Missense_Mutation | SNP | C | C | A | novel | Somatic | M104I | 0.344086 | NM_001350162.2 | 93 | 61 | 32 |
| 481-LNmet | CDH17 | 163 | hg19 | chr8 | 95201495 | 95201495 | Missense_Mutation | SNP | C | C | G | novel | Somatic | E24Q | 0.192118 | NM_004063.4 | 202 | 163 | 39 |
| 481-LNmet | TRIB1 | 1549 | hg19 | chr8 | 126448220 | 126448220 | Splice_Region | DEL | ATAATGGCTTGGTTCCTCTCTCTTGC | ATAATGGCTTGGTTCCTCTCTCTTGC | - | novel | Somatic |  | 0.210526 | NM_025195.4 | 57 | 45 | 12 |
| 481-LNmet | CYC1 | 1692 | hg19 | chr8 | 145151988 | 145151988 | Missense_Mutation | SNP | G | G | A | novel | Somatic | R275H | 0.120482 | NM_001916.5 | 249 | 219 | 30 |
| 481-LNmet | TONSL | 1696 | hg19 | chr8 | 145666438 | 145666438 | Nonsense_Mutation | SNP | G | G | A | novel | Somatic | Q308Ter | 0.263419 | NM_013432.5 | 1206 | 887 | 319 |
| 481-LNmet | DOCK8 | 73 | hg19 | chr9 | 441906 | 441906 | Missense_Mutation | SNP | G | G | A | novel | Somatic | R1796Q | 0.257282 | NM_203447.3 | 206 | 153 | 53 |
| 481-LNmet | KDM4C | 636 | hg19 | chr9 | 6984204 | 6984204 | Missense_Mutation | SNP | C | C | G | novel | Somatic | A385G | 0.12 | NM_015061.5 | 100 | 88 | 12 |
| 481-LNmet | CDKN2A | 752 | hg19 | chr9 | 21971036 | 21971036 | Missense_Mutation | SNP | C | C | T | novel | Somatic | D108N | 0.453178 | NM_000077.4 | 1461 | 798 | 663 |
| 481-LNmet | CAAP1 | 98 | hg19 | chr9 | 26842510 | 26842510 | Missense_Mutation | SNP | T | T | C | novel | Somatic | D292G | 0.109589 | NM_024828.4 | 146 | 130 | 16 |
| 481-LNmet | CDC26 | 1470 | hg19 | chr9 | 116034777 | 116034777 | Missense_Mutation | SNP | C | C | G | novel | Somatic | R4P | 0.340659 | NM_139286.4 | 90 | 59 | 31 |
| 481-LNmet | RC3H2 | 1543 | hg19 | chr9 | 125616533 | 125616533 | Silent | SNP | A | A | G | novel | Somatic | I971= | 0.235294 | NM_001100588.3 | 119 | 91 | 28 |
| 481-LNmet | TTF1 | 25 | hg19 | chr9 | 135277388 | 135277388 | Frame_Shift_Del | DEL | T | T | - | novel | Somatic | K274SfsTer149 | 0.032051 | NM_007344.4 | 311 | 301 | 10 |
| 481-LNmet | RNF224 | 1654 | hg19 | chr9 | 140123254 | 140123254 | Missense_Mutation | SNP | C | C | T | novel | Somatic | R63C | 0.239719 | NM_001190228.1 | 994 | 755 | 239 |
| 481-LNmet | RGR | 1241 | hg19 | chr10 | 86012687 | 86012687 | Missense_Mutation | SNP | C | C | G | novel | Somatic | L149V | 0.107955 | NM_002921.3 | 176 | 157 | 19 |
| 481-LNmet | EPS8L2 | 590 | hg19 | chr11 | 725827 | 725827 | Missense_Mutation | SNP | G | G | A | novel | Somatic | A554T | 0.210863 | NM_022772.4 | 924 | 726 | 198 |
| 481-LNmet | LUZP2 | 96 | hg19 | chr11 | 24750732 | 24750732 | Missense_Mutation | SNP | T | T | C | novel | Somatic | L27P | 0.173469 | NM_001009909.4 | 95 | 78 | 17 |
| 481-LNmet | STIP1 | 16 | hg19 | chr11 | 63971012 | 63971012 | Missense_Mutation | SNP | C | C | A | novel | Somatic | P493T | 0.062706 | NM_006819.3 | 302 | 283 | 19 |
| 481-LNmet | NRXN2 | 1076 | hg19 | chr11 | 64421194 | 64421194 | Missense_Mutation | SNP | T | T | C | novel | Somatic | D797G | 0.205446 | NM_015080.4 | 394 | 311 | 83 |
| 481-LNmet | FAM89B | 1083 | hg19 | chr11 | 65340833 | 65340833 | Splice_Site | SNP | G | G | T | novel | Somatic |  | 0.055684 | NM_001098785.2 | 430 | 406 | 24 |
| 481-LNmet | CHKA | 1102 | hg19 | chr11 | 67842184 | 67842184 | Splice_Region | SNP | C | C | A | novel | Somatic | P210= | 0.593607 | NM_001277.3 | 218 | 88 | 130 |
| 481-LNmet | C11orf71 | 1456 | hg19 | chr11 | 114270974 | 114270974 | Missense_Mutation | SNP | C | C | G | novel | Somatic | R27T | 0.177099 | NM_019021.4 | 655 | 539 | 116 |
| 481-LNmet | ACRBP | 636 | hg19 | chr12 | 6754383 | 6754383 | Splice_Region | SNP | C | C | G | novel | Somatic |  | 0.187166 | NM_032489.3 | 186 | 151 | 35 |
| 481-LNmet | CD163L1 | 642 | hg19 | chr12 | 7531771 | 7531771 | In_Frame_Del | DEL | TCAGCAATGTTC | TCAGCAATGTTC | - | novel | Somatic | M721_E725delinsI | 0.119565 | NM_174941.5 | 274 | 241 | 33 |
| 481-LNmet | ABCC9 | 11 | hg19 | chr12 | 21997422 | 21997422 | Missense_Mutation | SNP | C | C | G | novel | Somatic | D1104H | 0.196629 | NM_005691.3 | 178 | 143 | 35 |
| 481-LNmet | REP15 | 797 | hg19 | chr12 | 27849796 | 27849796 | Missense_Mutation | SNP | A | A | G | novel | Somatic | N101D | 0.68932 | NM_001029874.3 | 71 | 0 | 71 |
| 481-LNmet | KRT5 | 988 | hg19 | chr12 | 52913855 | 52913855 | In_Frame_Del | DEL | TGGATATCCTCT | TGGATATCCTCT | - | novel | Somatic | K72_S75del | 0.109479 | NM_000424.4 | 744 | 662 | 82 |
| 481-LNmet | SMARCC2 | 1016 | hg19 | chr12 | 56572825 | 56572825 | Frame_Shift_Del | DEL | TCTT | TCTT | - | novel | Somatic | K365TfsTer12 | 0.266667 | NM_001330288.2 | 177 | 129 | 48 |
| 481-LNmet | NXPH4 | 1024 | hg19 | chr12 | 57619423 | 57619423 | Missense_Mutation | SNP | T | T | G | novel | Somatic | F274V | 0.260274 | NM_007224.4 | 574 | 422 | 152 |
| 481-LNmet | TRHDE | 142 | hg19 | chr12 | 72667063 | 72667063 | Missense_Mutation | SNP | G | G | A | novel | Somatic | V169M | 0.213529 | NM_013381.2 | 1226 | 964 | 262 |
| 481-LNmet | ACACB | 1421 | hg19 | chr12 | 109689929 | 109689929 | Missense_Mutation | SNP | A | A | G | novel | Somatic | I1957V | 0.112426 | NM_001093.4 | 169 | 150 | 19 |
| 481-LNmet | SH2B3 | 1438 | hg19 | chr12 | 111856595 | 111856595 | Missense_Mutation | SNP | C | C | T | novel | Somatic | R216C | 0.210243 | NM_005475.3 | 738 | 582 | 156 |
| 481-LNmet | SHISA2 | 788 | hg19 | chr13 | 26624876 | 26624876 | Missense_Mutation | SNP | C | C | T | novel | Somatic | E80K | 0.196803 | NM_001007538.1 | 985 | 788 | 197 |
| 481-LNmet | RXFP2 | 103 | hg19 | chr13 | 32366145 | 32366145 | Missense_Mutation | SNP | C | C | T | novel | Somatic | H450Y | 0.1875 | NM_130806.5 | 112 | 91 | 21 |
| 481-LNmet | G2E3 | 102 | hg19 | chr14 | 31074771 | 31074771 | Frame_Shift_Ins | INS | - | - | A | novel | Somatic | T361NfsTer2 | 0.119266 | NM_017769.5 | 109 | 96 | 13 |
| 481-LNmet | SYNDIG1L | 1156 | hg19 | chr14 | 74876246 | 74876246 | Missense_Mutation | SNP | A | A | G | novel | Somatic | Y68H | 0.144284 | NM_001105579.2 | 885 | 755 | 130 |
| 481-LNmet | FLRT2 | 1241 | hg19 | chr14 | 86089539 | 86089539 | Missense_Mutation | SNP | G | G | A | novel | Somatic | V561I | 0.125 | NM_001346143.2 | 232 | 203 | 29 |
| 481-LNmet | CHGA | 1297 | hg19 | chr14 | 93399035 | 93399035 | Missense_Mutation | SNP | C | C | T | novel | Somatic | R377W | 0.020027 | NM_001275.4 | 1481 | 1451 | 30 |
| 481-LNmet | HDC | 970 | hg19 | chr15 | 50546377 | 50546377 | Missense_Mutation | SNP | G | G | C | novel | Somatic | L224V | 0.341176 | NM_002112.4 | 170 | 112 | 58 |
| 481-LNmet | LIPC | 129 | hg19 | chr15 | 58855717 | 58855717 | Missense_Mutation | SNP | G | G | T | novel | Somatic | A395S | 0.239583 | NM_000236.3 | 94 | 71 | 23 |
| 481-LNmet | HERC1 | 16 | hg19 | chr15 | 64005673 | 64005673 | Missense_Mutation | SNP | G | G | A | novel | Somatic | R1448W | 0.220472 | NM_003922.4 | 126 | 98 | 28 |
| 481-LNmet | IGF1R | 167 | hg19 | chr15 | 99459989 | 99459989 | Missense_Mutation | SNP | G | G | C | novel | Somatic | E695D | 0.060325 | NM_000875.5 | 430 | 404 | 26 |
| 481-LNmet | DNASE1L2 | 602 | hg19 | chr16 | 2287753 | 2287753 | Splice_Region | DEL | CCGCGGCGC | CCGCGGCGC | - | novel | Somatic |  | 0.174133 | NM_001374.3 | 1376 | 1135 | 241 |
| 481-LNmet | TRAP1 | 613 | hg19 | chr16 | 3712953 | 3712953 | Missense_Mutation | SNP | G | G | A | novel | Somatic | S575L | 0.208219 | NM_016292.3 | 365 | 289 | 76 |
| 481-LNmet | ZKSCAN2 | 777 | hg19 | chr16 | 25263271 | 25263271 | Missense_Mutation | SNP | C | C | T | novel | Somatic | R255Q | 0.163399 | NM_001012981.5 | 153 | 128 | 25 |
| 481-LNmet | ITGAX | 824 | hg19 | chr16 | 31383014 | 31383014 | Missense_Mutation | SNP | C | C | A | novel | Somatic | A690D | 0.14711 | NM_000887.5 | 566 | 482 | 84 |
| 481-LNmet | NUP93 | 1018 | hg19 | chr16 | 56866268 | 56866268 | Missense_Mutation | SNP | C | C | T | novel | Somatic | S438L | 0.1875 | NM_014669.5 | 160 | 130 | 30 |
| 481-LNmet | ADAMTS18 | 146 | hg19 | chr16 | 77396098 | 77396098 | Missense_Mutation | SNP | C | C | A | novel | Somatic | A374S | 0.149485 | NM_199355.4 | 194 | 165 | 29 |
| 481-LNmet | ZNF469 | 1260 | hg19 | chr16 | 88495829 | 88495829 | Missense_Mutation | SNP | C | C | G | novel | Somatic | P651A | 0.050736 | NM_001367624.2 | 1219 | 1157 | 62 |
| 481-LNmet | ZFPM1 | 1260 | hg19 | chr16 | 88600302 | 88600302 | Missense_Mutation | SNP | G | G | A | novel | Somatic | E646K | 0.259669 | NM_153813.3 | 181 | 134 | 47 |
| 481-LNmet | GGT6 | 619 | hg19 | chr17 | 4462146 | 4462146 | Missense_Mutation | SNP | C | C | T | novel | Somatic | D222N | 0.141115 | NM_001288702.2 | 1144 | 982 | 162 |
| 481-LNmet | DRC3 | 721 | hg19 | chr17 | 17910398 | 17910398 | Missense_Mutation | SNP | C | C | G | novel | Somatic | I421M | 0.184987 | NM_031294.4 | 1118 | 911 | 207 |
| 481-LNmet | MFAP4 | 732 | hg19 | chr17 | 19288491 | 19288491 | Silent | SNP | G | G | A | novel | Somatic | F147= | 0.194118 | NM_002404.3 | 169 | 136 | 33 |
| 481-LNmet | EFCAB5 | 12 | hg19 | chr17 | 28269284 | 28269284 | Missense_Mutation | SNP | G | G | T | novel | Somatic | R23S | 0.299652 | NM_198529.4 | 286 | 200 | 86 |
| 481-LNmet | HOXB9 | 941 | hg19 | chr17 | 46703139 | 46703139 | Missense_Mutation | SNP | C | C | T | novel | Somatic | E165K | 0.180272 | NM_024017.5 | 587 | 481 | 106 |
| 481-LNmet | LAMA1 | 79 | hg19 | chr18 | 6959455 | 6959455 | Missense_Mutation | SNP | C | C | T | novel | Somatic | E2555K | 0.095833 | NM_005559.4 | 240 | 217 | 23 |
| 481-LNmet | TJP3 | 613 | hg19 | chr19 | 3740743 | 3740743 | Nonsense_Mutation | SNP | C | C | T | novel | Somatic | R609Ter | 0.26988 | NM_001267560.2 | 823 | 599 | 224 |
| 481-LNmet | MUC16 | 81 | hg19 | chr19 | 9070548 | 9070548 | Missense_Mutation | SNP | G | G | A | novel | Somatic | S5633F | 0.164835 | NM_024690.2 | 91 | 76 | 15 |
| 481-LNmet | MUC16 | 81 | hg19 | chr19 | 9089582 | 9089582 | Missense_Mutation | SNP | G | G | A | novel | Somatic | H745Y | 0.193966 | NM_024690.2 | 232 | 187 | 45 |
| 481-LNmet | TMEM221 | 718 | hg19 | chr19 | 17547667 | 17547667 | Missense_Mutation | SNP | G | G | A | novel | Somatic | S159L | 0.219037 | NM_001190844.2 | 864 | 673 | 191 |
| 481-LNmet | TMEM59L | 727 | hg19 | chr19 | 18727861 | 18727861 | Missense_Mutation | SNP | G | G | A | novel | Somatic | V205M | 0.174359 | NM_012109.3 | 195 | 161 | 34 |
| 481-LNmet | ZNF429 | 750 | hg19 | chr19 | 21720557 | 21720557 | Missense_Mutation | SNP | A | A | G | novel | Somatic | K568E | 0.062136 | NM_001001415.4 | 512 | 480 | 32 |
| 481-LNmet | GPATCH1 | 841 | hg19 | chr19 | 33609996 | 33609996 | Missense_Mutation | SNP | C | C | T | novel | Somatic | P832S | 0.141304 | NM_018025.3 | 91 | 78 | 13 |
| 481-LNmet | ZNF529 | 867 | hg19 | chr19 | 37045605 | 37045605 | Missense_Mutation | SNP | T | T | C | novel | Somatic | M68V | 0.159664 | NM_020951.5 | 119 | 100 | 19 |
| 481-LNmet | ZNF569 | 874 | hg19 | chr19 | 37917266 | 37917266 | Missense_Mutation | SNP | G | G | C | novel | Somatic | F10L | 0.172414 | NM_152484.3 | 174 | 144 | 30 |
| 481-LNmet | CYP2B6 | 901 | hg19 | chr19 | 41518244 | 41518244 | Missense_Mutation | SNP | C | C | T | novel | Somatic | R336C | 0.070968 | NM_000767.5 | 82 | 71 | 11 |
| 481-LNmet | ZNF234 | 925 | hg19 | chr19 | 44660987 | 44660987 | Missense_Mutation | SNP | C | C | A | novel | Somatic | S273Y | 0.219512 | NM_006630.3 | 164 | 128 | 36 |
| 481-LNmet | SYT3 | 975 | hg19 | chr19 | 51133094 | 51133094 | Missense_Mutation | SNP | G | G | A | novel | Somatic | P337S | 0.2578 | NM_032298.2 | 608 | 451 | 157 |
| 481-LNmet | ZNF766 | 987 | hg19 | chr19 | 52794308 | 52794308 | Missense_Mutation | SNP | C | C | G | novel | Somatic | Q422E | 0.178138 | NM_001010851.3 | 247 | 203 | 44 |
| 481-LNmet | ZNF552 | 1029 | hg19 | chr19 | 58319454 | 58319454 | Missense_Mutation | SNP | G | G | C | novel | Somatic | S393C | 0.190299 | NM_024762.3 | 267 | 216 | 51 |
| 481-LNmet | ZSCAN1 | 1031 | hg19 | chr19 | 58549368 | 58549368 | Missense_Mutation | SNP | C | C | T | novel | Somatic | A55V | 0.213308 | NM_182572.4 | 1598 | 1255 | 343 |
| 481-LNmet | SLC27A5 | 1035 | hg19 | chr19 | 59022950 | 59022950 | Nonsense_Mutation | SNP | G | G | A | novel | Somatic | R125Ter | 0.224537 | NM_012254.3 | 1292 | 1001 | 291 |
| 481-LNmet | ZNF341 | 103 | hg19 | chr20 | 32341114 | 32341114 | Missense_Mutation | SNP | G | G | A | novel | Somatic | R209H | 0.339243 | NM_001282933.2 | 838 | 551 | 287 |
| 481-LNmet | GGT7 | 840 | hg19 | chr20 | 33449337 | 33449337 | Nonsense_Mutation | SNP | G | G | A | novel | Somatic | R196Ter | 0.317829 | NM_178026.3 | 129 | 88 | 41 |
| 481-LNmet | TPTE | 668 | hg19 | chr21 | 10944774 | 10944774 | Missense_Mutation | SNP | A | A | T | novel | Somatic | F154I | 0.048276 | NM_199261.3 | 724 | 689 | 35 |
| 481-LNmet | IL2RB | 871 | hg19 | chr22 | 37533724 | 37533724 | Missense_Mutation | SNP | C | C | T | novel | Somatic | R147K | 0.166667 | NM_000878.5 | 150 | 125 | 25 |
| 481-LNmet | PDXP | 875 | hg19 | chr22 | 38055213 | 38055213 | Missense_Mutation | SNP | C | C | T | novel | Somatic | R142C | 0.05614 | NM_020315.5 | 284 | 268 | 16 |
| 481-LNmet | EP300 | 112 | hg19 | chr22 | 41513449 | 41513449 | Missense_Mutation | SNP | T | T | A | novel | Somatic | I118K | 0.32618 | NM_001429.4 | 233 | 157 | 76 |
| 481-LNmet | ATXN10 | 14 | hg19 | chr22 | 46114328 | 46114328 | Missense_Mutation | SNP | C | C | T | novel | Somatic | P228L | 0.139738 | NM_013236.4 | 229 | 197 | 32 |
| 481-LNmet | BRD1 | 120 | hg19 | chr22 | 50167938 | 50167938 | Missense_Mutation | SNP | C | C | T | novel | Somatic | M1171I | 0.038083 | NM_001304808.3 | 1523 | 1465 | 58 |
| 481-LNmet | CFAP47 | 859 | hg19 | chrX | 36403047 | 36403047 | Missense_Mutation | SNP | C | C | T | novel | Somatic | R3164C | 0.402985 | NM_001304548.2 | 65 | 38 | 27 |
| 481-LNmet | HDX | 152 | hg19 | chrX | 83723676 | 83723676 | Nonsense_Mutation | SNP | G | G | T | novel | Somatic | S352Ter | 0.306818 | NM_001177479.2 | 88 | 61 | 27 |
| 481-LNmet | AMMECR1 | 177 | hg19 | chrX | 109561119 | 109561119 | Missense_Mutation | SNP | C | C | T | novel | Somatic | G61R | 0.266862 | NM_015365.3 | 680 | 498 | 182 |
| 575-PTsup | WDTC1 | 795 | hg19 | chr1 | 27621107 | 27621107 | Frame_Shift_Ins | INS | - | - | G | novel | Somatic | E290GfsTer7 | 0.028497 | NM_001276252.2 | 386 | 375 | 11 |
| 575-PTsup | DMRTB1 | 996 | hg19 | chr1 | 53927311 | 53927311 | Missense_Mutation | SNP | G | G | C | novel | Somatic | G248A | 0.351613 | NM_033067.2 | 928 | 601 | 327 |
| 575-PTsup | GBP6 | 1270 | hg19 | chr1 | 89846028 | 89846028 | Missense_Mutation | SNP | G | G | C | novel | Somatic | D237H | 0.040541 | NM_198460.2 | 296 | 284 | 12 |
| 575-PTsup | CELF3 | 1742 | hg19 | chr1 | 151681825 | 151681825 | Splice_Site | SNP | C | C | G | novel | Somatic |  | 0.04142 | NM_007185.7 | 507 | 486 | 21 |
| 575-PTsup | FLG2 | 1747 | hg19 | chr1 | 152324413 | 152324413 | Missense_Mutation | SNP | C | C | T | novel | Somatic | R1950K | 0.180165 | NM_001014342.3 | 602 | 493 | 109 |
| 575-PTsup | DENND4B | 1759 | hg19 | chr1 | 153906649 | 153906649 | Missense_Mutation | SNP | C | C | T | novel | Somatic | R968Q | 0.07483 | NM_014856.3 | 588 | 544 | 44 |
| 575-PTsup | ISG20L2 | 1780 | hg19 | chr1 | 156694023 | 156694023 | Frame_Shift_Del | DEL | G | G | - | novel | Somatic | L289SfsTer15 | 0.028409 | NM_001370151.1 | 527 | 512 | 15 |
| 575-PTsup | OR1C1 | 2476 | hg19 | chr1 | 247921610 | 247921610 | Silent | SNP | A | A | G | novel | Somatic | C33= | 0.356643 | NM_012353.2 | 286 | 184 | 102 |
| 575-PTsup | APLF | 1109 | hg19 | chr2 | 68805045 | 68805045 | Missense_Mutation | SNP | A | A | G | novel | Somatic | Y476C | 0.362264 | NM_173545.3 | 530 | 338 | 192 |
| 575-PTsup | IL1R2 | 170 | hg19 | chr2 | 102644852 | 102644852 | Nonstop_Mutation | SNP | T | T | C | novel | Somatic | Ter399RextTer41 | 0.035821 | NM_004633.4 | 335 | 323 | 12 |
| 575-PTsup | CACNB4 | 218 | hg19 | chr2 | 152739856 | 152739856 | Missense_Mutation | SNP | G | G | A | novel | Somatic | P59L | 0.091691 | NM_000726.4 | 347 | 315 | 32 |
| 575-PTsup | PLA2R1 | 226 | hg19 | chr2 | 160918826 | 160918826 | Missense_Mutation | SNP | T | T | A | novel | Somatic | E30V | 0.348977 | NM_007366.5 | 821 | 531 | 290 |
| 575-PTsup | LRP2 | 235 | hg19 | chr2 | 169997024 | 169997024 | Frame_Shift_Ins | INS | - | - | G | novel | Somatic | C4381MfsTer12 | 0.057402 | NM_004525.3 | 330 | 311 | 19 |
| 575-PTsup | DNAH7 | 260 | hg19 | chr2 | 196788373 | 196788373 | Frame_Shift_Ins | INS | - | - | T | novel | Somatic | N1257KfsTer3 | 0.024291 | NM_018897.3 | 494 | 482 | 12 |
| 575-PTsup | LANCL1 | 2197 | hg19 | chr2 | 211301109 | 211301109 | Missense_Mutation | SNP | C | C | G | novel | Somatic | R294T | 0.266667 | NM_006055.3 | 285 | 209 | 76 |
| 575-PTsup | STK11IP | 283 | hg19 | chr2 | 220471231 | 220471231 | Missense_Mutation | SNP | G | G | T | novel | Somatic | D327Y | 0.295615 | NM_052902.4 | 704 | 495 | 209 |
| 575-PTsup | DIS3L2 | 295 | hg19 | chr2 | 232995366 | 232995366 | Missense_Mutation | SNP | G | G | C | novel | Somatic | E213D | 0.036364 | NM_152383.4 | 495 | 477 | 18 |
| 575-PTsup | TGFBR2 | 102 | hg19 | chr3 | 30715660 | 30715660 | Missense_Mutation | SNP | G | G | A | novel | Somatic | E465K | 0.475472 | NM_001024847.2 | 265 | 139 | 126 |
| 575-PTsup | SMARCC1 | 118 | hg19 | chr3 | 47663802 | 47663802 | Frame_Shift_Del | DEL | G | G | - | novel | Somatic | K893SfsTer4 | 0.517073 | NM_003074.4 | 199 | 93 | 106 |
| 575-PTsup | ACOX2 | 1031 | hg19 | chr3 | 58519264 | 58519264 | Missense_Mutation | SNP | C | C | A | novel | Somatic | G164V | 0.518519 | NM_003500.4 | 216 | 104 | 112 |
| 575-PTsup | KBTBD12 | 194 | hg19 | chr3 | 127642154 | 127642154 | Missense_Mutation | SNP | G | G | T | novel | Somatic | V84L | 0.163174 | NM_207335.3 | 666 | 557 | 109 |
| 575-PTsup | NLGN1 | 238 | hg19 | chr3 | 173998371 | 173998371 | Missense_Mutation | SNP | C | C | A | novel | Somatic | L604I | 0.184466 | NM_001365923.2 | 720 | 587 | 133 |
| 575-PTsup | TRPC3 | 190 | hg19 | chr4 | 122853910 | 122853910 | Missense_Mutation | SNP | T | T | C | novel | Somatic | K168R | 0.338789 | NM_001130698.2 | 1222 | 808 | 414 |
| 575-PTsup | ANKRD50 | 1543 | hg19 | chr4 | 125593158 | 125593158 | Missense_Mutation | SNP | T | T | C | novel | Somatic | Y425C | 0.365724 | NM_020337.3 | 566 | 359 | 207 |
| 575-PTsup | NEK1 | 235 | hg19 | chr4 | 170508706 | 170508706 | Splice_Region | DEL | T | T | - | novel | Somatic |  | 0.301587 | NM_001199397.2 | 252 | 176 | 76 |
| 575-PTsup | PDE4D | 1029 | hg19 | chr5 | 58286633 | 58286633 | Missense_Mutation | SNP | G | G | C | novel | Somatic | Q429E | 0.336493 | NM_001104631.2 | 211 | 140 | 71 |
| 575-PTsup | ADGRV1 | 19 | hg19 | chr5 | 90449220 | 90449220 | Splice_Region | SNP | G | G | C | novel | Somatic |  | 0.337778 | NM_032119.4 | 225 | 149 | 76 |
| 575-PTsup | TMED7 | 182 | hg19 | chr5 | 114956292 | 114956292 | Missense_Mutation | SNP | T | T | C | novel | Somatic | Y93C | 0.350309 | NM_181836.6 | 647 | 420 | 227 |
| 575-PTsup | TXNDC15 | 0 | hg19 | chr5 | 134223753 | 134223753 | Missense_Mutation | SNP | G | G | A | novel | Somatic | A158T | 0.020284 | NM_024715.3 | 493 | 483 | 10 |
| 575-PTsup | KIF6 | 887 | hg19 | chr6 | 39353422 | 39353422 | Missense_Mutation | SNP | G | G | A | novel | Somatic | R613W | 0.275424 | NM_145027.6 | 236 | 171 | 65 |
| 575-PTsup | BAG2 | 1020 | hg19 | chr6 | 57037545 | 57037545 | Missense_Mutation | SNP | G | G | T | novel | Somatic | R17L | 0.354902 | NM_004282.4 | 1018 | 656 | 362 |
| 575-PTsup | EYS | 16 | hg19 | chr6 | 66205125 | 66205125 | Frame_Shift_Del | DEL | A | A | - | novel | Somatic | L60WfsTer3 | 0.026596 | NM_001292009.1 | 376 | 366 | 10 |
| 575-PTsup | MCHR2 | 168 | hg19 | chr6 | 100395774 | 100395774 | Frame_Shift_Del | DEL | T | T | - | novel | Somatic | M86CfsTer39 | 0.355932 | NM_001040179.2 | 408 | 261 | 147 |
| 575-PTsup | EIF2AK1 | 631 | hg19 | chr7 | 6068664 | 6068664 | Splice_Site | SNP | C | C | T | novel | Somatic |  | 0.374126 | NM_014413.4 | 284 | 177 | 107 |
| 575-PTsup | HECW1 | 114 | hg19 | chr7 | 43484548 | 43484548 | Missense_Mutation | SNP | G | G | T | novel | Somatic | D593Y | 0.358195 | NM_015052.5 | 1083 | 694 | 389 |
| 575-PTsup | EGFR | 125 | hg19 | chr7 | 55210050 | 55210050 | Missense_Mutation | SNP | A | A | T | novel | Somatic | M54L | 0.352294 | NM_005228.5 | 545 | 353 | 192 |
| 575-PTsup | LANCL2 | 125 | hg19 | chr7 | 55496065 | 55496065 | Splice_Region | SNP | C | C | G | novel | Somatic |  | 0.055556 | NM_018697.3 | 270 | 255 | 15 |
| 575-PTsup | STYXL1 | 145 | hg19 | chr7 | 75634695 | 75634695 | Missense_Mutation | SNP | T | T | C | novel | Somatic | I161V | 0.354067 | NM_016086.2 | 418 | 270 | 148 |
| 575-PTsup | MSC | 1140 | hg19 | chr8 | 72754901 | 72754901 | Missense_Mutation | SNP | C | C | T | novel | Somatic | A206T | 0.313253 | NM_005098.3 | 415 | 285 | 130 |
| 575-PTsup | TG | 200 | hg19 | chr8 | 133900745 | 133900745 | Missense_Mutation | SNP | G | G | T | novel | Somatic | C898F | 0.257437 | NM_003235.5 | 873 | 648 | 225 |
| 575-PTsup | PTP4A3 | 1671 | hg19 | chr8 | 142441102 | 142441102 | Missense_Mutation | SNP | C | C | G | novel | Somatic | F160L | 0.030403 | NM_032611.3 | 1215 | 1178 | 37 |
| 575-PTsup | MROH1 | 211 | hg19 | chr8 | 145278020 | 145278020 | Missense_Mutation | SNP | G | G | C | novel | Somatic | A409P | 0.257781 | NM_032450.2 | 1249 | 926 | 323 |
| 575-PTsup | ZNF250 | 1699 | hg19 | chr8 | 146106921 | 146106921 | Missense_Mutation | SNP | C | C | G | novel | Somatic | Q554H | 0.030377 | NM_001363099.2 | 823 | 798 | 25 |
| 575-PTsup | ARHGAP22 | 120 | hg19 | chr10 | 49658764 | 49658764 | Missense_Mutation | SNP | C | C | T | novel | Somatic | G470R | 0.368691 | NM_021226.4 | 1366 | 862 | 504 |
| 575-PTsup | FAM170B | 969 | hg19 | chr10 | 50339855 | 50339855 | Missense_Mutation | SNP | G | G | C | novel | Somatic | L219V | 0.026316 | NM_001164484.1 | 1101 | 1072 | 29 |
| 575-PTsup | MMP21 | 1557 | hg19 | chr10 | 127455460 | 127455460 | Missense_Mutation | SNP | G | G | T | novel | Somatic | P494Q | 0.222973 | NM_147191.1 | 294 | 228 | 66 |
| 575-PTsup | NLRP14 | 79 | hg19 | chr11 | 7059989 | 7059989 | Missense_Mutation | SNP | C | C | G | novel | Somatic | L58V | 0.486631 | NM_176822.3 | 187 | 96 | 91 |
| 575-PTsup | SBF2 | 82 | hg19 | chr11 | 10051370 | 10051370 | Missense_Mutation | TNP | AAG | AAG | CAA | novel | Somatic | L152W | 0.260047 | NM_030962.3 | 423 | 313 | 110 |
| 575-PTsup | SOX6 | 88 | hg19 | chr11 | 16362643 | 16362643 | Missense_Mutation | SNP | G | G | C | novel | Somatic | P51R | 0.314 | NM_033326.3 | 499 | 342 | 157 |
| 575-PTsup | ZNF408 | 941 | hg19 | chr11 | 46726106 | 46726106 | Missense_Mutation | SNP | G | G | C | novel | Somatic | D286H | 0.025994 | NM_024741.3 | 650 | 633 | 17 |
| 575-PTsup | FOLH1 | 960 | hg19 | chr11 | 49214441 | 49214441 | Missense_Mutation | SNP | G | G | T | novel | Somatic | F139L | 0.272727 | NM_004476.3 | 296 | 215 | 81 |
| 575-PTsup | NXF1 | 1062 | hg19 | chr11 | 62563543 | 62563543 | Missense_Mutation | SNP | G | G | C | novel | Somatic | Q486E | 0.212707 | NM_006362.5 | 360 | 283 | 77 |
| 575-PTsup | EHBP1L1 | 1083 | hg19 | chr11 | 65351836 | 65351836 | Missense_Mutation | SNP | G | G | T | novel | Somatic | C1073F | 0.523517 | NM_001099409.3 | 977 | 465 | 512 |
| 575-PTsup | CCDC87 | 1091 | hg19 | chr11 | 66360047 | 66360047 | Missense_Mutation | SNP | T | T | C | novel | Somatic | E147G | 0.497938 | NM_018219.3 | 967 | 484 | 483 |
| 575-PTsup | PLEKHB1 | 1144 | hg19 | chr11 | 73366898 | 73366898 | Missense_Mutation | SNP | G | G | C | novel | Somatic | K147N | 0.496774 | NM_021200.3 | 310 | 156 | 154 |
| 575-PTsup | MUC19 | 13 | hg19 | chr12 | 40874282 | 40874282 | Nonsense_Mutation | SNP | G | G | T | novel | Somatic | G2510Ter | 0.083744 | NM_173600.2 | 405 | 371 | 34 |
| 575-PTsup | NELL2 | 14 | hg19 | chr12 | 44913810 | 44913810 | Missense_Mutation | SNP | T | T | C | novel | Somatic | E843G | 0.225309 | NM_001145107.1 | 324 | 251 | 73 |
| 575-PTsup | PTPRB | 140 | hg19 | chr12 | 70960481 | 70960481 | Missense_Mutation | SNP | G | G | T | novel | Somatic | P1213Q | 0.361478 | NM_001109754.4 | 374 | 237 | 137 |
| 575-PTsup | NAV3 | 147 | hg19 | chr12 | 78582110 | 78582110 | Missense_Mutation | SNP | G | G | A | novel | Somatic | R1958K | 0.292359 | NM_001024383.2 | 299 | 211 | 88 |
| 575-PTsup | UHRF1BP1L | 168 | hg19 | chr12 | 100480074 | 100480074 | Missense_Mutation | SNP | A | A | G | novel | Somatic | Y362H | 0.303406 | NM_015054.2 | 323 | 225 | 98 |
| 575-PTsup | ANAPC5 | 189 | hg19 | chr12 | 121773386 | 121773386 | Missense_Mutation | SNP | C | C | G | novel | Somatic | L300F | 0.032203 | NM_016237.5 | 590 | 571 | 19 |
| 575-PTsup | TMEM132C | 24 | hg19 | chr12 | 128899942 | 128899942 | Missense_Mutation | SNP | G | G | A | novel | Somatic | A251T | 0.285897 | NM_001136103.2 | 779 | 556 | 223 |
| 575-PTsup | EP400 | 199 | hg19 | chr12 | 132498165 | 132498165 | Missense_Mutation | SNP | G | G | A | novel | Somatic | E1248K | 0.037736 | NM_015409.5 | 477 | 459 | 18 |
| 575-PTsup | ATP12A | 777 | hg19 | chr13 | 25275049 | 25275049 | Missense_Mutation | SNP | G | G | T | novel | Somatic | A624S | 0.314991 | NM_001676.6 | 526 | 360 | 166 |
| 575-PTsup | PCCA | 169 | hg19 | chr13 | 100909838 | 100909838 | Intron | SNP | C | C | G | novel | Somatic |  | 0.269802 | NM_000282.4 | 404 | 295 | 109 |
| 575-PTsup | CUL4A | 1453 | hg19 | chr13 | 113897411 | 113897411 | Missense_Mutation | SNP | C | C | G | novel | Somatic | L389V | 0.25 | NM_001008895.4 | 631 | 473 | 158 |
| 575-PTsup | FRMD6 | 983 | hg19 | chr14 | 52171594 | 52171594 | Missense_Mutation | SNP | T | T | C | novel | Somatic | F167L | 0.288089 | NM_001267046.2 | 360 | 256 | 104 |
| 575-PTsup | HERC1 | 16 | hg19 | chr15 | 63942064 | 63942064 | Frame_Shift_Del | DEL | T | T | - | novel | Somatic | E3536GfsTer26 | 0.336158 | NM_003922.4 | 349 | 230 | 119 |
| 575-PTsup | EEF2KMT | 624 | hg19 | chr16 | 5147731 | 5147731 | Missense_Mutation | SNP | C | C | T | novel | Somatic | E4K | 0.02886 | NM_201400.4 | 1382 | 1342 | 40 |
| 575-PTsup | TP53 | 642 | hg19 | chr17 | 7577046 | 7577046 | Nonsense_Mutation | SNP | C | C | A | novel | Somatic | E298Ter | 0.546547 | NM_000546.5 | 333 | 151 | 182 |
| 575-PTsup | AURKB | 646 | hg19 | chr17 | 8109958 | 8109958 | Splice_Site | SNP | C | C | A | novel | Somatic |  | 0.229167 | NM_004217.4 | 958 | 738 | 220 |
| 575-PTsup | NCOR1 | 707 | hg19 | chr17 | 15971414 | 15971414 | Missense_Mutation | SNP | G | G | C | novel | Somatic | S1512C | 0.52968 | NM_006311.4 | 218 | 102 | 116 |
| 575-PTsup | KRTAP4-5 | 884 | hg19 | chr17 | 39305595 | 39305595 | Missense_Mutation | SNP | C | C | T | novel | Somatic | R142H | 0.297393 | NM_033188.4 | 2816 | 1972 | 844 |
| 575-PTsup | SCRN2 | 935 | hg19 | chr17 | 45916908 | 45916908 | Missense_Mutation | SNP | G | G | A | novel | Somatic | A153V | 0.362353 | NM_138355.4 | 423 | 269 | 154 |
| 575-PTsup | ICAM2 | 1058 | hg19 | chr17 | 62080147 | 62080147 | Missense_Mutation | SNP | C | C | T | novel | Somatic | R263Q | 0.302368 | NM_001099786.1 | 549 | 383 | 166 |
| 575-PTsup | FHOD3 | 105 | hg19 | chr18 | 34174834 | 34174834 | Missense_Mutation | SNP | G | G | A | novel | Somatic | A231T | 0.400641 | NM_001281740.3 | 310 | 185 | 125 |
| 575-PTsup | C3 | 79 | hg19 | chr19 | 6709730 | 6709730 | Missense_Mutation | SNP | C | C | G | novel | Somatic | V604L | 0.328194 | NM_000064.4 | 454 | 305 | 149 |
| 575-PTsup | DCAF15 | 692 | hg19 | chr19 | 14069919 | 14069919 | Missense_Mutation | SNP | C | C | G | novel | Somatic | P283A | 0.024925 | NM_138353.3 | 1002 | 977 | 25 |
| 575-PTsup | FAM98C | 881 | hg19 | chr19 | 38899416 | 38899416 | Frame_Shift_Del | DEL | G | G | - | novel | Somatic | G317AfsTer49 | 0.020654 | NM_174905.3 | 581 | 569 | 12 |
| 575-PTsup | EID2 | 890 | hg19 | chr19 | 40030299 | 40030299 | Missense_Mutation | SNP | G | G | T | novel | Somatic | R141S | 0.350471 | NM_153232.4 | 1167 | 758 | 409 |
| 575-PTsup | PSG3 | 914 | hg19 | chr19 | 43234159 | 43234159 | Missense_Mutation | SNP | C | C | A | novel | Somatic | E253D | 0.278894 | NM_021016.4 | 1587 | 1143 | 444 |
| 575-PTsup | CDH22 | 115 | hg19 | chr20 | 44869794 | 44869794 | Missense_Mutation | SNP | G | G | T | novel | Somatic | H120N | 0.206587 | NM_021248.3 | 668 | 530 | 138 |
| 575-PTsup | HRH3 | 1048 | hg19 | chr20 | 60794957 | 60794957 | In_Frame_Del | DEL | CCG | CCG | - | novel | Somatic | A23del | 0.038741 | NM_007232.3 | 412 | 396 | 16 |
| 575-PTsup | DIDO1 | 1054 | hg19 | chr20 | 61510611 | 61510611 | Missense_Mutation | SNP | C | C | T | novel | Somatic | D2233N | 0.080469 | NM_033081.2 | 1276 | 1173 | 103 |
| 575-PTsup | HIC2 | 751 | hg19 | chr22 | 21799233 | 21799233 | Missense_Mutation | SNP | G | G | A | novel | Somatic | G17R | 0.303887 | NM_015094.3 | 848 | 590 | 258 |
| 575-PTdeep | DMRTB1 | 996 | hg19 | chr1 | 53927311 | 53927311 | Missense_Mutation | SNP | G | G | C | novel | Somatic | G248A | 0.225722 | NM_033067.2 | 381 | 295 | 86 |
| 575-PTdeep | SERBP1 | 137 | hg19 | chr1 | 67885862 | 67885862 | Missense_Mutation | SNP | C | C | T | novel | Somatic | E276K | 0.205263 | NM_001018067.2 | 190 | 151 | 39 |
| 575-PTdeep | SDCCAG8 | 305 | hg19 | chr1 | 243581304 | 243581304 | Missense_Mutation | SNP | T | T | G | novel | Somatic | N593K | 0.26087 | NM_006642.5 | 205 | 151 | 54 |
| 575-PTdeep | GREB1 | 674 | hg19 | chr2 | 11770084 | 11770084 | Missense_Mutation | SNP | C | C | T | novel | Somatic | S1487F | 0.168421 | NM_014668.4 | 188 | 156 | 32 |
| 575-PTdeep | DNMT3A | 779 | hg19 | chr2 | 25469149 | 25469149 | Missense_Mutation | SNP | T | T | G | novel | Somatic | T437P | 0.183246 | NM_022552.4 | 189 | 154 | 35 |
| 575-PTdeep | APLF | 1109 | hg19 | chr2 | 68805045 | 68805045 | Missense_Mutation | SNP | A | A | G | novel | Somatic | Y476C | 0.302905 | NM_173545.3 | 240 | 167 | 73 |
| 575-PTdeep | PLA2R1 | 226 | hg19 | chr2 | 160918826 | 160918826 | Missense_Mutation | SNP | T | T | A | novel | Somatic | E30V | 0.214929 | NM_007366.5 | 708 | 541 | 167 |
| 575-PTdeep | NFE2L2 | 242 | hg19 | chr2 | 178098968 | 178098968 | Missense_Mutation | SNP | T | T | G | novel | Somatic | Q26P | 0.195219 | NM_006164.5 | 250 | 201 | 49 |
| 575-PTdeep | LANCL1 | 2197 | hg19 | chr2 | 211301109 | 211301109 | Missense_Mutation | SNP | C | C | G | novel | Somatic | R294T | 0.271186 | NM_006055.3 | 118 | 86 | 32 |
| 575-PTdeep | STK11IP | 283 | hg19 | chr2 | 220471231 | 220471231 | Missense_Mutation | SNP | G | G | T | novel | Somatic | D327Y | 0.223969 | NM_052902.4 | 505 | 391 | 114 |
| 575-PTdeep | ARMC9 | 294 | hg19 | chr2 | 232087474 | 232087474 | Missense_Mutation | SNP | A | A | G | novel | Somatic | I180V | 0.131579 | NM_001271466.4 | 20 | 0 | 20 |
| 575-PTdeep | TGFBR2 | 102 | hg19 | chr3 | 30715660 | 30715660 | Missense_Mutation | SNP | G | G | A | novel | Somatic | E465K | 0.393162 | NM_001024847.2 | 117 | 71 | 46 |
| 575-PTdeep | SMARCC1 | 118 | hg19 | chr3 | 47663802 | 47663802 | Frame_Shift_Del | DEL | G | G | - | novel | Somatic | K893SfsTer4 | 0.231481 | NM_003074.4 | 104 | 79 | 25 |
| 575-PTdeep | ACOX2 | 1031 | hg19 | chr3 | 58519264 | 58519264 | Missense_Mutation | SNP | C | C | A | novel | Somatic | G164V | 0.258621 | NM_003500.4 | 58 | 43 | 15 |
| 575-PTdeep | NLGN1 | 238 | hg19 | chr3 | 173998371 | 173998371 | Missense_Mutation | SNP | C | C | A | novel | Somatic | L604I | 0.152416 | NM_001365923.2 | 268 | 227 | 41 |
| 575-PTdeep | OPA1 | 257 | hg19 | chr3 | 193361201 | 193361201 | Missense_Mutation | SNP | A | A | G | novel | Somatic | M449V | 0.169291 | NM_130837.2 | 253 | 210 | 43 |
| 575-PTdeep | TRPC3 | 190 | hg19 | chr4 | 122853910 | 122853910 | Missense_Mutation | SNP | T | T | C | novel | Somatic | K168R | 0.267456 | NM_001130698.2 | 842 | 616 | 226 |
| 575-PTdeep | ANKRD50 | 1543 | hg19 | chr4 | 125593158 | 125593158 | Missense_Mutation | SNP | T | T | C | novel | Somatic | Y425C | 0.235808 | NM_020337.3 | 229 | 175 | 54 |
| 575-PTdeep | NEK1 | 235 | hg19 | chr4 | 170508706 | 170508706 | Splice_Region | DEL | T | T | - | novel | Somatic |  | 0.366667 | NM_001199397.2 | 120 | 76 | 44 |
| 575-PTdeep | CDH12 | 11 | hg19 | chr5 | 21751911 | 21751911 | Missense_Mutation | SNP | G | G | A | novel | Somatic | R774C | 0.266187 | NM_001364105.2 | 139 | 102 | 37 |
| 575-PTdeep | PDE4D | 1029 | hg19 | chr5 | 58286633 | 58286633 | Missense_Mutation | SNP | G | G | C | novel | Somatic | Q429E | 0.260163 | NM_001104631.2 | 123 | 91 | 32 |
| 575-PTdeep | ADGRV1 | 19 | hg19 | chr5 | 90449220 | 90449220 | Splice_Region | SNP | G | G | C | novel | Somatic |  | 0.193333 | NM_032119.4 | 150 | 121 | 29 |
| 575-PTdeep | TMED7 | 182 | hg19 | chr5 | 114956292 | 114956292 | Missense_Mutation | SNP | T | T | C | novel | Somatic | Y93C | 0.217082 | NM_181836.6 | 281 | 220 | 61 |
| 575-PTdeep | ADAMTS19 | 24 | hg19 | chr5 | 128796962 | 128796962 | Missense_Mutation | SNP | C | C | T | novel | Somatic | R87C | 0.243572 | NM_133638.5 | 733 | 553 | 180 |
| 575-PTdeep | KIF6 | 887 | hg19 | chr6 | 39353422 | 39353422 | Missense_Mutation | SNP | G | G | A | novel | Somatic | R613W | 0.260638 | NM_145027.6 | 188 | 139 | 49 |
| 575-PTdeep | DAAM2 | 13 | hg19 | chr6 | 39856480 | 39856480 | Missense_Mutation | SNP | C | C | T | novel | Somatic | R696W | 0.044843 | NM_001201427.2 | 221 | 211 | 10 |
| 575-PTdeep | BAG2 | 1020 | hg19 | chr6 | 57037545 | 57037545 | Missense_Mutation | SNP | G | G | T | novel | Somatic | R17L | 0.220605 | NM_004282.4 | 874 | 677 | 197 |
| 575-PTdeep | MCHR2 | 168 | hg19 | chr6 | 100395774 | 100395774 | Frame_Shift_Del | DEL | T | T | - | novel | Somatic | M86CfsTer39 | 0.252688 | NM_001040179.2 | 186 | 139 | 47 |
| 575-PTdeep | FAM184A | 1495 | hg19 | chr6 | 119288012 | 119288012 | Missense_Mutation | SNP | T | T | C | novel | Somatic | I1007M | 0.239437 | NM_024581.6 | 213 | 162 | 51 |
| 575-PTdeep | LPA | 226 | hg19 | chr6 | 161026093 | 161026093 | Missense_Mutation | SNP | G | G | T | novel | Somatic | A977E | 0.055556 | NM_005577.4 | 449 | 424 | 25 |
| 575-PTdeep | TMEM184A | 597 | hg19 | chr7 | 1586653 | 1586653 | Missense_Mutation | SNP | A | A | C | novel | Somatic | S393A | 0.107534 | NM_001097620.2 | 421 | 264 | 157 |
| 575-PTdeep | HECW1 | 114 | hg19 | chr7 | 43484548 | 43484548 | Missense_Mutation | SNP | G | G | T | novel | Somatic | D593Y | 0.208402 | NM_015052.5 | 1212 | 959 | 253 |
| 575-PTdeep | EGFR | 125 | hg19 | chr7 | 55210050 | 55210050 | Missense_Mutation | SNP | A | A | T | novel | Somatic | M54L | 0.246575 | NM_005228.5 | 217 | 163 | 54 |
| 575-PTdeep | ZNF479 | 1021 | hg19 | chr7 | 57187809 | 57187809 | Missense_Mutation | SNP | T | T | G | novel | Somatic | K438T | 0.011029 | NM_033273.2 | 1086 | 1074 | 12 |
| 575-PTdeep | STYXL1 | 145 | hg19 | chr7 | 75634695 | 75634695 | Missense_Mutation | SNP | T | T | C | novel | Somatic | I161V | 0.236181 | NM_016086.2 | 198 | 151 | 47 |
| 575-PTdeep | DLC1 | 683 | hg19 | chr8 | 12957475 | 12957475 | Missense_Mutation | DNP | CC | CC | TG | novel | Somatic | V791M | 0.312195 | NM_182643.3 | 64 | 0 | 64 |
| 575-PTdeep | SLC18A1 | 737 | hg19 | chr8 | 20031968 | 20031968 | Intron | SNP | A | A | G | novel | Somatic |  | 0.326733 | NM_001135691.2 | 101 | 68 | 33 |
| 575-PTdeep | TG | 200 | hg19 | chr8 | 133900745 | 133900745 | Missense_Mutation | SNP | G | G | T | novel | Somatic | C898F | 0.178344 | NM_003235.5 | 467 | 383 | 84 |
| 575-PTdeep | MROH1 | 211 | hg19 | chr8 | 145278020 | 145278020 | Missense_Mutation | SNP | G | G | C | novel | Somatic | A409P | 0.225564 | NM_032450.2 | 399 | 309 | 90 |
| 575-PTdeep | ARHGAP22 | 120 | hg19 | chr10 | 49658764 | 49658764 | Missense_Mutation | SNP | C | C | T | novel | Somatic | G470R | 0.225322 | NM_021226.4 | 1392 | 1077 | 315 |
| 575-PTdeep | DENND5A | 81 | hg19 | chr11 | 9286597 | 9286597 | In_Frame_Del | DEL | CCG | CCG | - | novel | Somatic | G9del | 0.021154 | NM_015213.4 | 519 | 508 | 11 |
| 575-PTdeep | SOX6 | 88 | hg19 | chr11 | 16362643 | 16362643 | Missense_Mutation | SNP | G | G | C | novel | Somatic | P51R | 0.2 | NM_033326.3 | 175 | 140 | 35 |
| 575-PTdeep | FOLH1 | 960 | hg19 | chr11 | 49214441 | 49214441 | Missense_Mutation | SNP | G | G | T | novel | Somatic | F139L | 0.275862 | NM_004476.3 | 116 | 84 | 32 |
| 575-PTdeep | NXF1 | 1062 | hg19 | chr11 | 62563543 | 62563543 | Missense_Mutation | SNP | G | G | C | novel | Somatic | Q486E | 0.203008 | NM_006362.5 | 133 | 106 | 27 |
| 575-PTdeep | EHBP1L1 | 1083 | hg19 | chr11 | 65351836 | 65351836 | Missense_Mutation | SNP | G | G | T | novel | Somatic | C1073F | 0.419192 | NM_001099409.3 | 395 | 229 | 166 |
| 575-PTdeep | CCDC87 | 1091 | hg19 | chr11 | 66360047 | 66360047 | Missense_Mutation | SNP | T | T | C | novel | Somatic | E147G | 0.419492 | NM_018219.3 | 235 | 136 | 99 |
| 575-PTdeep | PLEKHB1 | 1144 | hg19 | chr11 | 73366898 | 73366898 | Missense_Mutation | SNP | G | G | C | novel | Somatic | K147N | 0.4 | NM_021200.3 | 175 | 105 | 70 |
| 575-PTdeep | NFRKB | 196 | hg19 | chr11 | 129752495 | 129752495 | Frame_Shift_Ins | INS | - | - | T | novel | Somatic | V337GfsTer2 | 0.085366 | NM_006165.3 | 162 | 148 | 14 |
| 575-PTdeep | NAV3 | 147 | hg19 | chr12 | 78582110 | 78582110 | Missense_Mutation | SNP | G | G | A | novel | Somatic | R1958K | 0.258065 | NM_001024383.2 | 152 | 112 | 40 |
| 575-PTdeep | UHRF1BP1L | 168 | hg19 | chr12 | 100480074 | 100480074 | Missense_Mutation | SNP | A | A | G | novel | Somatic | Y362H | 0.183099 | NM_015054.2 | 141 | 115 | 26 |
| 575-PTdeep | TMEM132C | 24 | hg19 | chr12 | 128899942 | 128899942 | Missense_Mutation | SNP | G | G | A | novel | Somatic | A251T | 0.184466 | NM_001136103.2 | 206 | 168 | 38 |
| 575-PTdeep | ATP12A | 777 | hg19 | chr13 | 25275049 | 25275049 | Missense_Mutation | SNP | G | G | T | novel | Somatic | A624S | 0.160428 | NM_001676.6 | 185 | 155 | 30 |
| 575-PTdeep | PCCA | 169 | hg19 | chr13 | 100909838 | 100909838 | Intron | SNP | C | C | G | novel | Somatic |  | 0.276243 | NM_000282.4 | 181 | 131 | 50 |
| 575-PTdeep | CUL4A | 1453 | hg19 | chr13 | 113897411 | 113897411 | Missense_Mutation | SNP | C | C | G | novel | Somatic | L389V | 0.130841 | NM_001008895.4 | 213 | 185 | 28 |
| 575-PTdeep | FRMD6 | 983 | hg19 | chr14 | 52171594 | 52171594 | Missense_Mutation | SNP | T | T | C | novel | Somatic | F167L | 0.258621 | NM_001267046.2 | 174 | 129 | 45 |
| 575-PTdeep | HERC1 | 16 | hg19 | chr15 | 63942064 | 63942064 | Frame_Shift_Del | DEL | T | T | - | novel | Somatic | E3536GfsTer26 | 0.163265 | NM_003922.4 | 98 | 82 | 16 |
| 575-PTdeep | TP53 | 642 | hg19 | chr17 | 7577046 | 7577046 | Nonsense_Mutation | SNP | C | C | A | novel | Somatic | E298Ter | 0.264901 | NM_000546.5 | 151 | 111 | 40 |
| 575-PTdeep | CDK12 | 872 | hg19 | chr17 | 37673805 | 37673805 | Missense_Mutation | SNP | T | T | C | novel | Somatic | S987P | 0.195652 | NM_016507.4 | 138 | 111 | 27 |
| 575-PTdeep | KRTAP4-5 | 884 | hg19 | chr17 | 39305595 | 39305595 | Missense_Mutation | SNP | C | C | T | novel | Somatic | R142H | 0.22717 | NM_033188.4 | 1765 | 1362 | 403 |
| 575-PTdeep | ICAM2 | 1058 | hg19 | chr17 | 62080147 | 62080147 | Missense_Mutation | SNP | C | C | T | novel | Somatic | R263Q | 0.167742 | NM_001099786.1 | 153 | 127 | 26 |
| 575-PTdeep | FASN | 1195 | hg19 | chr17 | 80042743 | 80042743 | Missense_Mutation | SNP | C | C | T | novel | Somatic | G1499E | 0.180052 | NM_004104.5 | 765 | 626 | 139 |
| 575-PTdeep | SMCHD1 | 75 | hg19 | chr18 | 2751275 | 2751275 | Splice_Site | SNP | G | G | A | novel | Somatic |  | 0.313043 | NM_015295.2 | 115 | 79 | 36 |
| 575-PTdeep | FHOD3 | 105 | hg19 | chr18 | 34174834 | 34174834 | Missense_Mutation | SNP | G | G | A | novel | Somatic | A231T | 0.186207 | NM_001281740.3 | 145 | 118 | 27 |
| 575-PTdeep | C3 | 79 | hg19 | chr19 | 6709730 | 6709730 | Missense_Mutation | SNP | C | C | G | novel | Somatic | V604L | 0.240964 | NM_000064.4 | 249 | 189 | 60 |
| 575-PTdeep | ZNF429 | 750 | hg19 | chr19 | 21720557 | 21720557 | Missense_Mutation | SNP | A | A | G | novel | Somatic | K568E | 0.039024 | NM_001001415.4 | 405 | 389 | 16 |
| 575-PTdeep | EID2 | 890 | hg19 | chr19 | 40030299 | 40030299 | Missense_Mutation | SNP | G | G | T | novel | Somatic | R141S | 0.264036 | NM_153232.4 | 657 | 483 | 174 |
| 575-PTdeep | PSG3 | 914 | hg19 | chr19 | 43234159 | 43234159 | Missense_Mutation | SNP | C | C | A | novel | Somatic | E253D | 0.165049 | NM_021016.4 | 823 | 687 | 136 |
| 575-PTdeep | KCNK15 | 915 | hg19 | chr20 | 43374838 | 43374838 | Splice_Region | DEL | A | A | - | novel | Somatic |  | 0.027739 | NM_022358.4 | 680 | 660 | 20 |
| 575-PTdeep | HIC2 | 751 | hg19 | chr22 | 21799233 | 21799233 | Missense_Mutation | SNP | G | G | A | novel | Somatic | G17R | 0.168467 | NM_015094.3 | 459 | 381 | 78 |
| 575-LNmet | DMRTB1 | 996 | hg19 | chr1 | 53927311 | 53927311 | Missense_Mutation | SNP | G | G | C | novel | Somatic | G248A | 0.127358 | NM_033067.2 | 212 | 185 | 27 |
| 575-LNmet | SLC41A1 | 2154 | hg19 | chr1 | 205768201 | 205768201 | Missense_Mutation | SNP | G | G | A | novel | Somatic | A194V | 0.179894 | NM_173854.6 | 189 | 155 | 34 |
| 575-LNmet | OR1C1 | 2476 | hg19 | chr1 | 247921610 | 247921610 | Silent | SNP | A | A | G | novel | Somatic | C33= | 0.161616 | NM_012353.2 | 98 | 82 | 16 |
| 575-LNmet | APLF | 1109 | hg19 | chr2 | 68805045 | 68805045 | Missense_Mutation | SNP | A | A | G | novel | Somatic | Y476C | 0.211712 | NM_173545.3 | 222 | 175 | 47 |
| 575-LNmet | PLA2R1 | 226 | hg19 | chr2 | 160918826 | 160918826 | Missense_Mutation | SNP | T | T | A | novel | Somatic | E30V | 0.176238 | NM_007366.5 | 461 | 372 | 89 |
| 575-LNmet | LANCL1 | 2197 | hg19 | chr2 | 211301109 | 211301109 | Missense_Mutation | SNP | C | C | G | novel | Somatic | R294T | 0.22549 | NM_006055.3 | 102 | 79 | 23 |
| 575-LNmet | STK11IP | 283 | hg19 | chr2 | 220471231 | 220471231 | Missense_Mutation | SNP | G | G | T | novel | Somatic | D327Y | 0.188755 | NM_052902.4 | 248 | 201 | 47 |
| 575-LNmet | SGO1 | 739 | hg19 | chr3 | 20216050 | 20216050 | Frame_Shift_Del | DEL | T | T | - | novel | Somatic | M325CfsTer28 | 0.047619 | NM_001199252.3 | 209 | 199 | 10 |
| 575-LNmet | TGFBR2 | 102 | hg19 | chr3 | 30715660 | 30715660 | Missense_Mutation | SNP | G | G | A | novel | Somatic | E465K | 0.29703 | NM_001024847.2 | 101 | 71 | 30 |
| 575-LNmet | SMARCC1 | 118 | hg19 | chr3 | 47663802 | 47663802 | Frame_Shift_Del | DEL | G | G | - | novel | Somatic | K893SfsTer4 | 0.176471 | NM_003074.4 | 101 | 83 | 18 |
| 575-LNmet | ACOX2 | 1031 | hg19 | chr3 | 58519264 | 58519264 | Missense_Mutation | SNP | C | C | A | novel | Somatic | G164V | 0.139241 | NM_003500.4 | 79 | 68 | 11 |
| 575-LNmet | KBTBD12 | 194 | hg19 | chr3 | 127642154 | 127642154 | Missense_Mutation | SNP | G | G | T | novel | Somatic | V84L | 0.077778 | NM_207335.3 | 180 | 166 | 14 |
| 575-LNmet | TRPC3 | 190 | hg19 | chr4 | 122853910 | 122853910 | Missense_Mutation | SNP | T | T | C | novel | Somatic | K168R | 0.285372 | NM_001130698.2 | 417 | 298 | 119 |
| 575-LNmet | ANKRD50 | 1543 | hg19 | chr4 | 125593158 | 125593158 | Missense_Mutation | SNP | T | T | C | novel | Somatic | Y425C | 0.21 | NM_020337.3 | 200 | 158 | 42 |
| 575-LNmet | NEK1 | 235 | hg19 | chr4 | 170508706 | 170508706 | Splice_Region | DEL | T | T | - | novel | Somatic |  | 0.1 | NM_001199397.2 | 120 | 108 | 12 |
| 575-LNmet | PDE4D | 1029 | hg19 | chr5 | 58286633 | 58286633 | Missense_Mutation | SNP | G | G | C | novel | Somatic | Q429E | 0.186275 | NM_001104631.2 | 102 | 83 | 19 |
| 575-LNmet | KIF6 | 887 | hg19 | chr6 | 39353422 | 39353422 | Missense_Mutation | SNP | G | G | A | novel | Somatic | R613W | 0.177419 | NM_145027.6 | 123 | 101 | 22 |
| 575-LNmet | BAG2 | 1020 | hg19 | chr6 | 57037545 | 57037545 | Missense_Mutation | SNP | G | G | T | novel | Somatic | R17L | 0.181976 | NM_004282.4 | 569 | 464 | 105 |
| 575-LNmet | MCHR2 | 168 | hg19 | chr6 | 100395774 | 100395774 | Frame_Shift_Del | DEL | T | T | - | novel | Somatic | M86CfsTer39 | 0.152318 | NM_001040179.2 | 150 | 127 | 23 |
| 575-LNmet | FAM184A | 1495 | hg19 | chr6 | 119288012 | 119288012 | Missense_Mutation | SNP | T | T | C | novel | Somatic | I1007M | 0.212389 | NM_024581.6 | 113 | 89 | 24 |
| 575-LNmet | TMEM184A | 597 | hg19 | chr7 | 1586653 | 1586653 | Missense_Mutation | SNP | A | A | C | novel | Somatic | S393A | 0.085795 | NM_001097620.2 | 173 | 112 | 61 |
| 575-LNmet | HECW1 | 114 | hg19 | chr7 | 43484548 | 43484548 | Missense_Mutation | SNP | G | G | T | novel | Somatic | D593Y | 0.170274 | NM_015052.5 | 691 | 573 | 118 |
| 575-LNmet | EGFR | 125 | hg19 | chr7 | 55210050 | 55210050 | Missense_Mutation | SNP | A | A | T | novel | Somatic | M54L | 0.157895 | NM_005228.5 | 209 | 176 | 33 |
| 575-LNmet | STYXL1 | 145 | hg19 | chr7 | 75634695 | 75634695 | Missense_Mutation | SNP | T | T | C | novel | Somatic | I161V | 0.187097 | NM_016086.2 | 155 | 126 | 29 |
| 575-LNmet | ZFHX4 | 18 | hg19 | chr8 | 77763216 | 77763216 | Missense_Mutation | SNP | A | A | T | novel | Somatic | E1353D | 0.114286 | NM_024721.5 | 175 | 155 | 20 |
| 575-LNmet | TG | 200 | hg19 | chr8 | 133900745 | 133900745 | Missense_Mutation | SNP | G | G | T | novel | Somatic | C898F | 0.120623 | NM_003235.5 | 257 | 226 | 31 |
| 575-LNmet | MROH1 | 211 | hg19 | chr8 | 145278020 | 145278020 | Missense_Mutation | SNP | G | G | C | novel | Somatic | A409P | 0.164659 | NM_032450.2 | 249 | 208 | 41 |
| 575-LNmet | ARHGAP22 | 120 | hg19 | chr10 | 49658764 | 49658764 | Missense_Mutation | SNP | C | C | T | novel | Somatic | G470R | 0.196429 | NM_021226.4 | 950 | 763 | 187 |
| 575-LNmet | SUFU | 172 | hg19 | chr10 | 104375029 | 104375029 | Missense_Mutation | SNP | C | C | T | novel | Somatic | R343C | 0.188811 | NM_016169.3 | 143 | 116 | 27 |
| 575-LNmet | SOX6 | 88 | hg19 | chr11 | 16362643 | 16362643 | Missense_Mutation | SNP | G | G | C | novel | Somatic | P51R | 0.13245 | NM_033326.3 | 151 | 131 | 20 |
| 575-LNmet | FOLH1 | 960 | hg19 | chr11 | 49214441 | 49214441 | Missense_Mutation | SNP | G | G | T | novel | Somatic | F139L | 0.125 | NM_004476.3 | 128 | 112 | 16 |
| 575-LNmet | TMEM132A | 1048 | hg19 | chr11 | 60704108 | 60704108 | Missense_Mutation | SNP | C | C | T | novel | Somatic | P935L | 0.11236 | NM_017870.4 | 263 | 233 | 30 |
| 575-LNmet | NXF1 | 1062 | hg19 | chr11 | 62563543 | 62563543 | Missense_Mutation | SNP | G | G | C | novel | Somatic | Q486E | 0.105263 | NM_006362.5 | 95 | 85 | 10 |
| 575-LNmet | EHBP1L1 | 1083 | hg19 | chr11 | 65351836 | 65351836 | Missense_Mutation | SNP | G | G | T | novel | Somatic | C1073F | 0.263158 | NM_001099409.3 | 227 | 167 | 60 |
| 575-LNmet | CCDC87 | 1091 | hg19 | chr11 | 66360047 | 66360047 | Missense_Mutation | SNP | T | T | C | novel | Somatic | E147G | 0.35443 | NM_018219.3 | 157 | 101 | 56 |
| 575-LNmet | PLEKHB1 | 1144 | hg19 | chr11 | 73366898 | 73366898 | Missense_Mutation | SNP | G | G | C | novel | Somatic | K147N | 0.220472 | NM_021200.3 | 127 | 99 | 28 |
| 575-LNmet | NAV3 | 147 | hg19 | chr12 | 78582110 | 78582110 | Missense_Mutation | SNP | G | G | A | novel | Somatic | R1958K | 0.226277 | NM_001024383.2 | 137 | 106 | 31 |
| 575-LNmet | UHRF1BP1L | 168 | hg19 | chr12 | 100480074 | 100480074 | Missense_Mutation | SNP | A | A | G | novel | Somatic | Y362H | 0.127273 | NM_015054.2 | 110 | 96 | 14 |
| 575-LNmet | TMEM132C | 24 | hg19 | chr12 | 128899942 | 128899942 | Missense_Mutation | SNP | G | G | A | novel | Somatic | A251T | 0.134503 | NM_001136103.2 | 171 | 148 | 23 |
| 575-LNmet | ATP12A | 777 | hg19 | chr13 | 25275049 | 25275049 | Missense_Mutation | SNP | G | G | T | novel | Somatic | A624S | 0.141304 | NM_001676.6 | 183 | 157 | 26 |
| 575-LNmet | PCCA | 169 | hg19 | chr13 | 100909838 | 100909838 | Intron | SNP | C | C | G | novel | Somatic |  | 0.2 | NM_000282.4 | 129 | 103 | 26 |
| 575-LNmet | TNFSF13B | 1416 | hg19 | chr13 | 108955706 | 108955706 | Missense_Mutation | SNP | A | A | G | novel | Somatic | Y196C | 0.217054 | NM_006573.4 | 129 | 101 | 28 |
| 575-LNmet | CUL4A | 1453 | hg19 | chr13 | 113897411 | 113897411 | Missense_Mutation | SNP | C | C | G | novel | Somatic | L389V | 0.171875 | NM_001008895.4 | 192 | 159 | 33 |
| 575-LNmet | FRMD6 | 983 | hg19 | chr14 | 52171594 | 52171594 | Missense_Mutation | SNP | T | T | C | novel | Somatic | F167L | 0.12766 | NM_001267046.2 | 94 | 82 | 12 |
| 575-LNmet | MGA | 905 | hg19 | chr15 | 42003383 | 42003383 | In_Frame_Del | DEL | CAG | CAG | - | novel | Somatic | Q981del | 0.046729 | NM_001164273.1 | 214 | 204 | 10 |
| 575-LNmet | HERC1 | 16 | hg19 | chr15 | 63942064 | 63942064 | Frame_Shift_Del | DEL | T | T | - | novel | Somatic | E3536GfsTer26 | 0.223214 | NM_003922.4 | 111 | 86 | 25 |
| 575-LNmet | MC1R | 1271 | hg19 | chr16 | 89986186 | 89986186 | Missense_Mutation | SNP | G | G | A | novel | Somatic | V174I | 0.135831 | NM_002386.3 | 427 | 369 | 58 |
| 575-LNmet | TP53 | 642 | hg19 | chr17 | 7577046 | 7577046 | Nonsense_Mutation | SNP | C | C | A | novel | Somatic | E298Ter | 0.228814 | NM_000546.5 | 118 | 91 | 27 |
| 575-LNmet | KRTAP4-5 | 884 | hg19 | chr17 | 39305595 | 39305595 | Missense_Mutation | SNP | C | C | T | novel | Somatic | R142H | 0.134315 | NM_033188.4 | 1152 | 997 | 155 |
| 575-LNmet | SCRN2 | 935 | hg19 | chr17 | 45916908 | 45916908 | Missense_Mutation | SNP | G | G | A | novel | Somatic | A153V | 0.31405 | NM_138355.4 | 121 | 83 | 38 |
| 575-LNmet | ICAM2 | 1058 | hg19 | chr17 | 62080147 | 62080147 | Missense_Mutation | SNP | C | C | T | novel | Somatic | R263Q | 0.114286 | NM_001099786.1 | 105 | 93 | 12 |
| 575-LNmet | FHOD3 | 105 | hg19 | chr18 | 34174834 | 34174834 | Missense_Mutation | SNP | G | G | A | novel | Somatic | A231T | 0.186275 | NM_001281740.3 | 102 | 83 | 19 |
| 575-LNmet | C3 | 79 | hg19 | chr19 | 6709730 | 6709730 | Missense_Mutation | SNP | C | C | G | novel | Somatic | V604L | 0.184783 | NM_000064.4 | 184 | 150 | 34 |
| 575-LNmet | ZNF429 | 750 | hg19 | chr19 | 21720557 | 21720557 | Missense_Mutation | SNP | A | A | G | novel | Somatic | K568E | 0.053097 | NM_001001415.4 | 451 | 427 | 24 |
| 575-LNmet | EID2 | 890 | hg19 | chr19 | 40030299 | 40030299 | Missense_Mutation | SNP | G | G | T | novel | Somatic | R141S | 0.215933 | NM_153232.4 | 475 | 372 | 103 |
| 575-LNmet | PSG3 | 914 | hg19 | chr19 | 43234159 | 43234159 | Missense_Mutation | SNP | C | C | A | novel | Somatic | E253D | 0.128763 | NM_021016.4 | 598 | 521 | 77 |
| 575-LNmet | PPP6R1 | 1010 | hg19 | chr19 | 55752847 | 55752847 | Nonsense_Mutation | SNP | T | T | A | novel | Somatic | K336Ter | 0.180828 | NM_014931.4 | 459 | 376 | 83 |
| 575-LNmet | HIC2 | 751 | hg19 | chr22 | 21799233 | 21799233 | Missense_Mutation | SNP | G | G | A | novel | Somatic | G17R | 0.154472 | NM_015094.3 | 246 | 208 | 38 |
| 575-LNmet | ARAF | 946 | hg19 | chrX | 47426120 | 47426120 | Missense_Mutation | SNP | T | T | G | novel | Somatic | S214A | 0.307692 | NM_001654.5 | 139 | 95 | 44 |
| 653-PTsup | SSU72 | 596 | hg19 | chr1 | 1477512 | 1477512 | In_Frame_Ins | INS | - | - | TCGATC | novel | Somatic | E171_I172dup | 0.190945 | NM_014188.3 | 508 | 411 | 97 |
| 653-PTsup | DNAJC16 | 88 | hg19 | chr1 | 15886055 | 15886055 | In_Frame_Del | DEL | TCA | TCA | - | novel | Somatic | I354del | 0.186441 | NM_015291.4 | 412 | 335 | 77 |
| 653-PTsup | UQCRHL | 708 | hg19 | chr1 | 16133928 | 16133928 | Missense_Mutation | SNP | C | C | G | novel | Somatic | D73H | 0.205128 | NM_001089591.1 | 740 | 588 | 152 |
| 653-PTsup | ZMYM6 | 855 | hg19 | chr1 | 35477556 | 35477556 | Missense_Mutation | SNP | G | G | A | novel | Somatic | H333Y | 0.188552 | NM_007167.4 | 297 | 241 | 56 |
| 653-PTsup | ZMYM1 | 856 | hg19 | chr1 | 35578657 | 35578657 | Missense_Mutation | SNP | C | C | T | novel | Somatic | S409L | 0.218472 | NM_024772.5 | 561 | 438 | 123 |
| 653-PTsup | AGO4 | 107 | hg19 | chr1 | 36291050 | 36291050 | Nonsense_Mutation | SNP | C | C | G | novel | Somatic | S148Ter | 0.194553 | NM_017629.4 | 513 | 413 | 100 |
| 653-PTsup | CSF3R | 866 | hg19 | chr1 | 36935409 | 36935409 | Nonsense_Mutation | SNP | G | G | A | novel | Somatic | R440Ter | 0.183226 | NM_156039.3 | 775 | 633 | 142 |
| 653-PTsup | HIVEP3 | 113 | hg19 | chr1 | 42048097 | 42048097 | In_Frame_Del | DEL | CTC | CTC | - | novel | Somatic | R791del | 0.18797 | NM_024503.5 | 661 | 536 | 125 |
| 653-PTsup | FAF1 | 15 | hg19 | chr1 | 51050417 | 51050417 | Missense_Mutation | SNP | C | C | G | novel | Somatic | G303R | 0.189956 | NM_007051.3 | 458 | 371 | 87 |
| 653-PTsup | NRDC | 122 | hg19 | chr1 | 52257726 | 52257726 | Missense_Mutation | SNP | C | C | G | novel | Somatic | Q1024H | 0.222772 | NM_002525.2 | 202 | 157 | 45 |
| 653-PTsup | LRRC7 | 17 | hg19 | chr1 | 70144122 | 70144122 | Missense_Mutation | SNP | G | G | C | novel | Somatic | E21Q | 0.170259 | NM_001370785.1 | 462 | 383 | 79 |
| 653-PTsup | TGFBR3 | 2 | hg19 | chr1 | 92185448 | 92185448 | Splice_Site | SNP | A | A | G | novel | Somatic |  | 0.190341 | NM_003243.5 | 352 | 285 | 67 |
| 653-PTsup | LRIF1 | 1435 | hg19 | chr1 | 111494431 | 111494431 | Missense_Mutation | SNP | T | T | A | novel | Somatic | M359L | 0.235192 | NM_018372.4 | 574 | 439 | 135 |
| 653-PTsup | CEPT1 | 1437 | hg19 | chr1 | 111690538 | 111690538 | Frame_Shift_Del | DEL | ATGCAAG | ATGCAAG | - | novel | Somatic | M68GfsTer26 | 0.182927 | NM_006090.5 | 573 | 468 | 105 |
| 653-PTsup | NBPF10 | 26 | hg19 | chr1 | 145311150 | 145311150 | Missense_Mutation | SNP | C | C | G | novel | Somatic | P617A | 0.07839 | NM_001039703.6 | 3302 | 3043 | 259 |
| 653-PTsup | SETDB1 | 1736 | hg19 | chr1 | 150915441 | 150915441 | Missense_Mutation | SNP | C | C | T | novel | Somatic | R263W | 0.192982 | NM_001366418.1 | 568 | 458 | 110 |
| 653-PTsup | CELF3 | 1742 | hg19 | chr1 | 151682224 | 151682224 | Missense_Mutation | SNP | C | C | T | novel | Somatic | R91Q | 0.215753 | NM_007185.7 | 584 | 458 | 126 |
| 653-PTsup | PAQR6 | 1776 | hg19 | chr1 | 156215359 | 156215359 | Missense_Mutation | SNP | G | G | C | novel | Somatic | S54C | 0.02994 | NM_024897.3 | 1001 | 971 | 30 |
| 653-PTsup | IPO9 | 265 | hg19 | chr1 | 201817642 | 201817642 | Missense_Mutation | SNP | C | C | T | novel | Somatic | P145L | 0.204007 | NM_018085.5 | 549 | 437 | 112 |
| 653-PTsup | TARBP1 | 2374 | hg19 | chr1 | 234563135 | 234563135 | Splice_Site | SNP | C | C | G | novel | Somatic |  | 0.209877 | NM_005646.4 | 647 | 511 | 136 |
| 653-PTsup | LYST | 2385 | hg19 | chr1 | 235956889 | 235956889 | Missense_Mutation | SNP | G | G | T | novel | Somatic | L1344I | 0.151571 | NM_001301365.1 | 539 | 457 | 82 |
| 653-PTsup | TSSC1 | Unknown | hg19 | chr2 | 3261143 | 3261143 | Missense_Mutation | SNP | C | C | T | novel | Somatic | D115N | 0.175311 | NM_003310.4 | 964 | 795 | 169 |
| 653-PTsup | RSAD2 | 638 | hg19 | chr2 | 7036022 | 7036022 | Missense_Mutation | SNP | G | G | C | novel | Somatic | K345N | 0.133333 | NM_080657.5 | 555 | 481 | 74 |
| 653-PTsup | PRR30 | 793 | hg19 | chr2 | 27360083 | 27360083 | Missense_Mutation | SNP | C | C | T | novel | Somatic | R372Q | 0.363988 | NM_178553.4 | 1292 | 821 | 471 |
| 653-PTsup | PSME4 | 124 | hg19 | chr2 | 54163906 | 54163906 | Missense_Mutation | SNP | C | C | G | novel | Somatic | E252Q | 0.021834 | NM_014614.3 | 687 | 672 | 15 |
| 653-PTsup | PROKR1 | 1110 | hg19 | chr2 | 68882505 | 68882505 | Missense_Mutation | SNP | G | G | A | novel | Somatic | V327I | 0.35063 | NM_138964.3 | 1349 | 876 | 473 |
| 653-PTsup | ARHGAP25 | 1111 | hg19 | chr2 | 69045015 | 69045015 | Missense_Mutation | SNP | G | G | A | novel | Somatic | E298K | 0.154047 | NM_001007231.3 | 380 | 321 | 59 |
| 653-PTsup | ZNF638 | 141 | hg19 | chr2 | 71650548 | 71650548 | Frame_Shift_Ins | INS | - | - | A | novel | Somatic | G1305RfsTer2 | 0.340659 | NM_014497.5 | 538 | 352 | 186 |
| 653-PTsup | C2orf81 | 1154 | hg19 | chr2 | 74642859 | 74642859 | Missense_Mutation | SNP | C | C | T | novel | Somatic | G149S | 0.178651 | NM_001316764.3 | 1348 | 1107 | 241 |
| 653-PTsup | TEKT4 | 1313 | hg19 | chr2 | 95541339 | 95541339 | Missense_Mutation | SNP | C | C | T | novel | Somatic | R315W | 0.134002 | NM_144705.4 | 1096 | 949 | 147 |
| 653-PTsup | KIAA1211L | 167 | hg19 | chr2 | 99438908 | 99438908 | Missense_Mutation | SNP | C | C | T | novel | Somatic | E610K | 0.278932 | NM_207362.3 | 1011 | 729 | 282 |
| 653-PTsup | ST6GAL2 | 175 | hg19 | chr2 | 107459567 | 107459567 | Missense_Mutation | SNP | G | G | T | novel | Somatic | H289Q | 0.177104 | NM_001322362.2 | 1021 | 840 | 181 |
| 653-PTsup | DPP10 | 22 | hg19 | chr2 | 116599880 | 116599880 | Missense_Mutation | SNP | G | G | A | novel | Somatic | E801K | 0.135747 | NM_001321905.2 | 442 | 382 | 60 |
| 653-PTsup | ARHGEF4 | 198 | hg19 | chr2 | 131672852 | 131672852 | Missense_Mutation | SNP | G | G | C | novel | Somatic | E445Q | 0.132128 | NM_001367493.1 | 719 | 624 | 95 |
| 653-PTsup | MBD5 | 26 | hg19 | chr2 | 149240922 | 149240922 | Missense_Mutation | SNP | C | C | T | novel | Somatic | S921F | 0.021782 | NM_018328.4 | 1007 | 985 | 22 |
| 653-PTsup | KCNJ3 | 1771 | hg19 | chr2 | 155711277 | 155711277 | Missense_Mutation | SNP | G | G | A | novel | Somatic | E320K | 0.260677 | NM_002239.4 | 679 | 502 | 177 |
| 653-PTsup | LY75-CD302 | 226 | hg19 | chr2 | 160676274 | 160676274 | Missense_Mutation | SNP | A | A | T | novel | Somatic | F1372L | 0.15427 | NM_001198759.1 | 363 | 307 | 56 |
| 653-PTsup | KCNH7 | 28 | hg19 | chr2 | 163693178 | 163693178 | Missense_Mutation | SNP | A | A | G | novel | Somatic | V59A | 0.238701 | NM_033272.4 | 706 | 537 | 169 |
| 653-PTsup | KLHL41 | 1884 | hg19 | chr2 | 170366628 | 170366628 | Missense_Mutation | SNP | C | C | G | novel | Somatic | Q114E | 0.276252 | NM_006063.3 | 619 | 448 | 171 |
| 653-PTsup | NFE2L2 | 242 | hg19 | chr2 | 178098960 | 178098960 | Missense_Mutation | SNP | C | C | G | novel | Somatic | D29H | 0.263785 | NM_006164.5 | 670 | 493 | 177 |
| 653-PTsup | ZNF804A | 31 | hg19 | chr2 | 185801390 | 185801390 | Missense_Mutation | SNP | G | G | C | novel | Somatic | E423Q | 0.257862 | NM_194250.2 | 636 | 472 | 164 |
| 653-PTsup | ABCA12 | 278 | hg19 | chr2 | 215891652 | 215891652 | Missense_Mutation | SNP | G | G | C | novel | Somatic | L358V | 0.200436 | NM_173076.3 | 459 | 367 | 92 |
| 653-PTsup | IGFBP2 | 2244 | hg19 | chr2 | 217498593 | 217498593 | Missense_Mutation | SNP | G | G | C | novel | Somatic | G116A | 0.190345 | NM_000597.3 | 725 | 587 | 138 |
| 653-PTsup | SLC19A3 | 2328 | hg19 | chr2 | 228563751 | 228563751 | Missense_Mutation | SNP | C | C | G | novel | Somatic | G227A | 0.271756 | NM_025243.4 | 655 | 477 | 178 |
| 653-PTsup | GRM7 | 9 | hg19 | chr3 | 7340373 | 7340373 | Nonsense_Mutation | SNP | G | G | T | novel | Somatic | G247Ter | 0.203846 | NM_000844.4 | 519 | 413 | 106 |
| 653-PTsup | CAND2 | 85 | hg19 | chr3 | 12838250 | 12838250 | Missense_Mutation | SNP | C | C | T | novel | Somatic | L11F | 0.264286 | NM_001162499.2 | 700 | 515 | 185 |
| 653-PTsup | GOLGA4 | 108 | hg19 | chr3 | 37388770 | 37388770 | Missense_Mutation | SNP | A | A | G | novel | Somatic | M2202V | 0.253623 | NM_001172713.2 | 414 | 309 | 105 |
| 653-PTsup | USP19 | 119 | hg19 | chr3 | 49146570 | 49146570 | Missense_Mutation | SNP | C | C | T | novel | Somatic | E1362K | 0.46997 | NM_001351100.1 | 666 | 353 | 313 |
| 653-PTsup | PBRM1 | 123 | hg19 | chr3 | 52678784 | 52678784 | Frame_Shift_Del | DEL | T | T | - | novel | Somatic | I279YfsTer4 | 0.352505 | NM_018313.5 | 538 | 348 | 190 |
| 653-PTsup | PDZRN3 | 1145 | hg19 | chr3 | 73433781 | 73433781 | Missense_Mutation | SNP | A | A | G | novel | Somatic | F646L | 0.241309 | NM_015009.3 | 974 | 738 | 236 |
| 653-PTsup | GOLGB1 | 1511 | hg19 | chr3 | 121416186 | 121416186 | Missense_Mutation | SNP | C | C | T | novel | Somatic | E1062K | 0.211494 | NM_001256486.2 | 435 | 343 | 92 |
| 653-PTsup | KALRN | 191 | hg19 | chr3 | 124380746 | 124380746 | Missense_Mutation | SNP | A | A | G | novel | Somatic | M2105V | 0.182008 | NM_001024660.4 | 478 | 391 | 87 |
| 653-PTsup | ATR | 208 | hg19 | chr3 | 142226756 | 142226756 | Intron | SNP | C | C | G | novel | Somatic |  | 0.164516 | NM_001184.4 | 309 | 258 | 51 |
| 653-PTsup | SCHIP1 | 1802 | hg19 | chr3 | 159583964 | 159583964 | Frame_Shift_Del | DEL | GA | GA | - | novel | Somatic | E260VfsTer13 | 0.117537 | NM_014575.3 | 536 | 473 | 63 |
| 653-PTsup | BCHE | 230 | hg19 | chr3 | 165548026 | 165548026 | Missense_Mutation | SNP | C | C | T | novel | Somatic | E266K | 0.319213 | NM_000055.4 | 661 | 450 | 211 |
| 653-PTsup | MSX1 | 622 | hg19 | chr4 | 4864727 | 4864727 | Missense_Mutation | SNP | C | C | G | novel | Somatic | L257V | 0.221344 | NM_002448.3 | 759 | 591 | 168 |
| 653-PTsup | SEL1L3 | 97 | hg19 | chr4 | 25780780 | 25780780 | Missense_Mutation | SNP | T | T | C | novel | Somatic | M835V | 0.167539 | NM_015187.5 | 380 | 316 | 64 |
| 653-PTsup | NFXL1 | 950 | hg19 | chr4 | 47886406 | 47886406 | Missense_Mutation | SNP | G | G | C | novel | Somatic | Q625E | 0.314961 | NM_001278624.2 | 635 | 435 | 200 |
| 653-PTsup | ANKRD17 | 143 | hg19 | chr4 | 73985976 | 73985976 | Missense_Mutation | SNP | C | C | T | novel | Somatic | D1310N | 0.257851 | NM_032217.5 | 605 | 449 | 156 |
| 653-PTsup | TET2 | 1394 | hg19 | chr4 | 106156531 | 106156531 | Missense_Mutation | SNP | G | G | A | novel | Somatic | E478K | 0.236749 | NM_001127208.2 | 564 | 430 | 134 |
| 653-PTsup | GIMD1 | 1403 | hg19 | chr4 | 107288225 | 107288225 | Missense_Mutation | SNP | A | A | G | novel | Somatic | V123A | 0.204887 | NM_001195138.1 | 532 | 423 | 109 |
| 653-PTsup | ALPK1 | 22 | hg19 | chr4 | 113298937 | 113298937 | Missense_Mutation | SNP | A | A | C | novel | Somatic | N2H | 0.47561 | NM_025144.4 | 328 | 172 | 156 |
| 653-PTsup | PLRG1 | 1771 | hg19 | chr4 | 155461792 | 155461792 | Frame_Shift_Del | DEL | G | G | - | novel | Somatic | P298RfsTer7 | 0.205082 | NM_002669.4 | 551 | 438 | 113 |
| 653-PTsup | SLC6A3 | 74 | hg19 | chr5 | 1401052 | 1401052 | Missense_Mutation | SNP | C | C | T | novel | Somatic | R606K | 0.152985 | NM_001044.5 | 803 | 680 | 123 |
| 653-PTsup | ADAMTS16 | 9 | hg19 | chr5 | 5146365 | 5146365 | Missense_Mutation | SNP | C | C | T | novel | Somatic | R100W | 0.2144 | NM_139056.4 | 625 | 491 | 134 |
| 653-PTsup | PAPD7 | 636 | hg19 | chr5 | 6714482 | 6714482 | Missense_Mutation | SNP | C | C | T | novel | Somatic | S129L | 0.216216 | NM_006999.6 | 294 | 230 | 64 |
| 653-PTsup | SEMA5A | 10 | hg19 | chr5 | 9052090 | 9052090 | Missense_Mutation | SNP | G | G | T | novel | Somatic | Q914K | 0.148736 | NM_003966.3 | 1383 | 1177 | 206 |
| 653-PTsup | 6-Mar | 82 | hg19 | chr5 | 10403604 | 10403604 | Missense_Mutation | SNP | G | G | A | novel | Somatic | G428E | 0.191011 | NM_005885.4 | 355 | 287 | 68 |
| 653-PTsup | SPEF2 | 13 | hg19 | chr5 | 35740257 | 35740257 | Missense_Mutation | SNP | G | G | T | novel | Somatic | R1073L | 0.147583 | NM_024867.4 | 393 | 335 | 58 |
| 653-PTsup | FBN2 | 194 | hg19 | chr5 | 127680126 | 127680126 | Nonsense_Mutation | SNP | G | G | T | novel | Somatic | C1098Ter | 0.242358 | NM_001999.4 | 458 | 347 | 111 |
| 653-PTsup | PKD2L2 | 203 | hg19 | chr5 | 137260794 | 137260794 | Splice_Site | SNP | G | G | T | novel | Somatic |  | 0.219885 | NM_001300921.2 | 520 | 405 | 115 |
| 653-PTsup | SLC34A1 | 241 | hg19 | chr5 | 176815371 | 176815371 | Nonsense_Mutation | SNP | C | C | T | novel | Somatic | Q312Ter | 0.218137 | NM_003052.5 | 408 | 319 | 89 |
| 653-PTsup | HIST1H1B | 797 | hg19 | chr6 | 27834675 | 27834675 | In_Frame_Del | DEL | TGC | TGC | - | novel | Somatic | A211del | 0.197598 | NM_005322.2 | 916 | 735 | 181 |
| 653-PTsup | TREM2 | 898 | hg19 | chr6 | 41129020 | 41129020 | Silent | SNP | G | G | T | novel | Somatic | V124= | 0.133705 | NM_018965.4 | 358 | 310 | 48 |
| 653-PTsup | CUL9 | 914 | hg19 | chr6 | 43190294 | 43190294 | Missense_Mutation | SNP | G | G | A | novel | Somatic | R2316Q | 0.173592 | NM_015089.4 | 1083 | 895 | 188 |
| 653-PTsup | ZNF318 | 915 | hg19 | chr6 | 43306322 | 43306322 | Missense_Mutation | SNP | C | C | A | novel | Somatic | S1805I | 0.16318 | NM_014345.3 | 478 | 400 | 78 |
| 653-PTsup | LGSN | 1073 | hg19 | chr6 | 63990395 | 63990395 | Missense_Mutation | SNP | G | G | A | novel | Somatic | A354V | 0.313981 | NM_016571.3 | 843 | 578 | 265 |
| 653-PTsup | PTPRK | 195 | hg19 | chr6 | 128540093 | 128540093 | Missense_Mutation | SNP | G | G | A | novel | Somatic | S281F | 0.180873 | NM_001291981.2 | 480 | 393 | 87 |
| 653-PTsup | PHF14 | 83 | hg19 | chr7 | 11101655 | 11101655 | Frame_Shift_Ins | INS | - | - | T | novel | Somatic | L867FfsTer4 | 0.205797 | NM_001007157.2 | 343 | 272 | 71 |
| 653-PTsup | HOXA6 | 792 | hg19 | chr7 | 27186940 | 27186940 | Missense_Mutation | SNP | C | C | T | novel | Somatic | M143I | 0.331593 | NM_024014.4 | 766 | 512 | 254 |
| 653-PTsup | NOD1 | 817 | hg19 | chr7 | 30491628 | 30491628 | Missense_Mutation | SNP | G | G | A | novel | Somatic | R469W | 0.17181 | NM_006092.4 | 1198 | 992 | 206 |
| 653-PTsup | STAG3 | 1346 | hg19 | chr7 | 99796499 | 99796499 | Missense_Mutation | SNP | G | G | T | novel | Somatic | M462I | 0.198598 | NM_001282717.1 | 428 | 343 | 85 |
| 653-PTsup | PIK3CG | 1397 | hg19 | chr7 | 106524631 | 106524631 | Missense_Mutation | SNP | C | C | G | novel | Somatic | S931C | 0.232598 | NM_002649.3 | 589 | 452 | 137 |
| 653-PTsup | CADPS2 | 189 | hg19 | chr7 | 122255307 | 122255307 | Missense_Mutation | SNP | C | C | T | novel | Somatic | R384Q | 0.22093 | NM_001363389.1 | 430 | 335 | 95 |
| 653-PTsup | ADRB3 | 873 | hg19 | chr8 | 37823224 | 37823224 | Missense_Mutation | SNP | G | G | A | novel | Somatic | S255L | 0.244094 | NM_000025.3 | 380 | 287 | 93 |
| 653-PTsup | LETM2 | 876 | hg19 | chr8 | 38250329 | 38250329 | Missense_Mutation | SNP | A | A | T | novel | Somatic | Q106L | 0.441538 | NM_001286819.1 | 650 | 363 | 287 |
| 653-PTsup | ZFHX4 | 18 | hg19 | chr8 | 77767085 | 77767085 | Missense_Mutation | SNP | G | G | C | novel | Somatic | G2643A | 0.247717 | NM_024721.5 | 876 | 659 | 217 |
| 653-PTsup | SNX16 | 1216 | hg19 | chr8 | 82736029 | 82736029 | Missense_Mutation | SNP | G | G | C | novel | Somatic | N203K | 0.171014 | NM_022133.3 | 345 | 286 | 59 |
| 653-PTsup | CNGB3 | 156 | hg19 | chr8 | 87683211 | 87683211 | Missense_Mutation | SNP | C | C | G | novel | Somatic | E152Q | 0.473605 | NM_019098.4 | 662 | 348 | 314 |
| 653-PTsup | SLC26A7 | 2 | hg19 | chr8 | 92375765 | 92375765 | Missense_Mutation | SNP | G | G | A | novel | Somatic | S496N | 0.166113 | NM_134266.2 | 300 | 250 | 50 |
| 653-PTsup | RGS22 | 169 | hg19 | chr8 | 101059824 | 101059824 | Missense_Mutation | SNP | T | T | C | novel | Somatic | K564E | 0.199134 | NM_015668.5 | 231 | 185 | 46 |
| 653-PTsup | PKHD1L1 | 178 | hg19 | chr8 | 110412510 | 110412510 | Missense_Mutation | SNP | C | C | G | novel | Somatic | F406L | 0.188976 | NM_177531.6 | 508 | 412 | 96 |
| 653-PTsup | SNTB1 | 23 | hg19 | chr8 | 121706103 | 121706103 | Missense_Mutation | SNP | G | G | A | novel | Somatic | P206L | 0.084435 | NM_021021.4 | 1362 | 1247 | 115 |
| 653-PTsup | EFR3A | 1599 | hg19 | chr8 | 132966087 | 132966087 | Missense_Mutation | SNP | G | G | A | novel | Somatic | G171S | 0.074359 | NM_015137.6 | 1558 | 1442 | 116 |
| 653-PTsup | JAK2 | 77 | hg19 | chr9 | 5050712 | 5050712 | Missense_Mutation | SNP | G | G | C | novel | Somatic | W165C | 0.194631 | NM_004972.3 | 596 | 480 | 116 |
| 653-PTsup | KDM4C | 636 | hg19 | chr9 | 7103848 | 7103848 | Missense_Mutation | SNP | G | G | C | novel | Somatic | R863P | 0.164345 | NM_015061.5 | 359 | 300 | 59 |
| 653-PTsup | MPDZ | 85 | hg19 | chr9 | 13188782 | 13188782 | Splice_Site | SNP | C | C | T | novel | Somatic |  | 0.40458 | NM_001330637.1 | 262 | 156 | 106 |
| 653-PTsup | FOCAD | 11 | hg19 | chr9 | 20717846 | 20717846 | Missense_Mutation | SNP | G | G | C | novel | Somatic | K37N | 0.233058 | NM_017794.4 | 605 | 464 | 141 |
| 653-PTsup | DDX58 | 12 | hg19 | chr9 | 32459463 | 32459463 | Missense_Mutation | SNP | T | T | A | novel | Somatic | K796I | 0.187845 | NM_014314.4 | 542 | 440 | 102 |
| 653-PTsup | PCSK5 | 18 | hg19 | chr9 | 78686768 | 78686768 | Missense_Mutation | SNP | G | G | A | novel | Somatic | G283E | 0.150502 | NM_001372043.1 | 1194 | 1014 | 180 |
| 653-PTsup | ROR2 | 163 | hg19 | chr9 | 94487206 | 94487206 | Missense_Mutation | SNP | C | C | T | novel | Somatic | E524K | 0.153907 | NM_004560.4 | 1254 | 1061 | 193 |
| 653-PTsup | FBP1 | 165 | hg19 | chr9 | 97369228 | 97369228 | Missense_Mutation | SNP | C | C | T | novel | Somatic | G192R | 0.143232 | NM_000507.4 | 1902 | 1629 | 273 |
| 653-PTsup | RNF20 | 1380 | hg19 | chr9 | 104309662 | 104309662 | Intron | SNP | T | T | G | novel | Somatic |  | 0.125 | NM_019592.7 | 280 | 245 | 35 |
| 653-PTsup | SVEP1 | 22 | hg19 | chr9 | 113217997 | 113217997 | Intron | SNP | C | C | T | novel | Somatic |  | 0.121212 | NM_153366.4 | 363 | 319 | 44 |
| 653-PTsup | TLR4 | 1504 | hg19 | chr9 | 120476729 | 120476729 | Nonsense_Mutation | SNP | G | G | T | novel | Somatic | E775Ter | 0.13522 | NM_138554.5 | 636 | 550 | 86 |
| 653-PTsup | OR1L3 | 1542 | hg19 | chr9 | 125437535 | 125437535 | Missense_Mutation | SNP | C | C | A | novel | Somatic | L43M | 0.296355 | NM_001005234.1 | 631 | 444 | 187 |
| 653-PTsup | SET | 1587 | hg19 | chr9 | 131456277 | 131456277 | Missense_Mutation | SNP | G | G | A | novel | Somatic | E269K | 0.190581 | NM_001122821.2 | 912 | 738 | 174 |
| 653-PTsup | SET | 1587 | hg19 | chr9 | 131456286 | 131456286 | Missense_Mutation | SNP | G | G | A | novel | Somatic | E272K | 0.177096 | NM_001122821.2 | 845 | 695 | 150 |
| 653-PTsup | AK8 | 202 | hg19 | chr9 | 135739089 | 135739089 | Missense_Mutation | SNP | G | G | A | novel | Somatic | T99M | 0.192793 | NM_152572.3 | 553 | 446 | 107 |
| 653-PTsup | ADAMTS13 | 25 | hg19 | chr9 | 136293772 | 136293772 | Missense_Mutation | SNP | C | C | G | novel | Somatic | D235E | 0.165803 | NM_139025.4 | 193 | 161 | 32 |
| 653-PTsup | COL5A1 | 204 | hg19 | chr9 | 137686952 | 137686952 | Missense_Mutation | SNP | C | C | T | novel | Somatic | R909W | 0.231604 | NM_001278074.1 | 828 | 636 | 192 |
| 653-PTsup | C10orf67 | 765 | hg19 | chr10 | 23592373 | 23592373 | Missense_Mutation | SNP | C | C | G | novel | Somatic | E189Q | 0.255172 | NM_001365862.2 | 290 | 216 | 74 |
| 653-PTsup | LOC100505502 | Unknown | hg19 | chr10 | 31652039 | 31652039 | Missense_Mutation | SNP | G | G | A | novel | Somatic | P239S | 0.206456 | NM_001368169.1 | 1487 | 1180 | 307 |
| 653-PTsup | WDFY4 | 120 | hg19 | chr10 | 49944069 | 49944069 | Missense_Mutation | SNP | G | G | C | novel | Somatic | C611S | 0.209343 | NM_020945.2 | 578 | 457 | 121 |
| 653-PTsup | CDHR1 | 1240 | hg19 | chr10 | 85974270 | 85974270 | Missense_Mutation | SNP | C | C | G | novel | Somatic | P825A | 0.210261 | NM_033100.4 | 1185 | 935 | 250 |
| 653-PTsup | LGI1 | 164 | hg19 | chr10 | 95557238 | 95557238 | Missense_Mutation | SNP | T | T | A | novel | Somatic | F451Y | 0.173278 | NM_005097.4 | 479 | 396 | 83 |
| 653-PTsup | NT5C2 | 21 | hg19 | chr10 | 104850373 | 104850373 | Missense_Mutation | SNP | C | C | A | novel | Somatic | V490F | 0.21671 | NM_001351173.1 | 383 | 300 | 83 |
| 653-PTsup | ITPRIP | 1394 | hg19 | chr10 | 106075218 | 106075218 | Missense_Mutation | SNP | C | C | T | novel | Somatic | E198K | 0.20595 | NM_033397.3 | 874 | 694 | 180 |
| 653-PTsup | JAKMIP3 | 200 | hg19 | chr10 | 133981856 | 133981856 | Missense_Mutation | SNP | G | G | A | novel | Somatic | V978I | 0.197727 | NM_001323086.2 | 880 | 706 | 174 |
| 653-PTsup | MUC5B | 594 | hg19 | chr11 | 1262533 | 1262533 | Missense_Mutation | SNP | C | C | T | novel | Somatic | R1475W | 0.215363 | NM_002458.3 | 1392 | 1092 | 300 |
| 653-PTsup | OR51H1 | 622 | hg19 | chr11 | 4881331 | 4881331 | Missense_Mutation | SNP | A | A | G | novel | Somatic | V155A | 0.167857 | NM_001348291.1 | 559 | 465 | 94 |
| 653-PTsup | OR52N5 | 629 | hg19 | chr11 | 5799805 | 5799805 | Missense_Mutation | SNP | A | A | T | novel | Somatic | F20L | 0.163043 | NM_001001922.2 | 368 | 308 | 60 |
| 653-PTsup | TAF10 | 635 | hg19 | chr11 | 6632922 | 6632922 | Missense_Mutation | SNP | C | C | G | novel | Somatic | L120F | 0.191795 | NM_006284.4 | 974 | 787 | 187 |
| 653-PTsup | RNF141 | 665 | hg19 | chr11 | 10536581 | 10536581 | Missense_Mutation | SNP | C | C | T | novel | Somatic | R192H | 0.128451 | NM_016422.4 | 830 | 723 | 107 |
| 653-PTsup | NAV2 | 92 | hg19 | chr11 | 20114706 | 20114706 | Missense_Mutation | SNP | G | G | C | novel | Somatic | K1959N | 0.504491 | NM_145117.5 | 668 | 331 | 337 |
| 653-PTsup | DBX1 | 738 | hg19 | chr11 | 20181645 | 20181645 | Missense_Mutation | SNP | C | C | G | novel | Somatic | D76H | 0.202899 | NM_001029865.4 | 1171 | 933 | 238 |
| 653-PTsup | DCDC1 | 102 | hg19 | chr11 | 30946913 | 30946913 | Missense_Mutation | SNP | C | C | A | novel | Somatic | L980F | 0.328165 | NM_001367979.1 | 774 | 520 | 254 |
| 653-PTsup | SLC43A3 | 1021 | hg19 | chr11 | 57175323 | 57175323 | Missense_Mutation | SNP | G | G | A | novel | Somatic | P486L | 0.392265 | NM_001278206.2 | 359 | 217 | 142 |
| 653-PTsup | OR5B12 | 1029 | hg19 | chr11 | 58206931 | 58206931 | Missense_Mutation | SNP | G | G | A | novel | Somatic | R232C | 0.352174 | NM_001004733.2 | 459 | 297 | 162 |
| 653-PTsup | PITPNM1 | 1098 | hg19 | chr11 | 67269450 | 67269450 | Missense_Mutation | SNP | C | C | T | novel | Somatic | D175N | 0.429303 | NM_004910.3 | 975 | 556 | 419 |
| 653-PTsup | ALDH3B2 | 1099 | hg19 | chr11 | 67432882 | 67432882 | Missense_Mutation | SNP | C | C | A | novel | Somatic | D194Y | 0.233887 | NM_001354345.1 | 1501 | 1149 | 352 |
| 653-PTsup | FGF3 | 1116 | hg19 | chr11 | 69625234 | 69625234 | Missense_Mutation | SNP | C | C | T | novel | Somatic | D187N | 0.056715 | NM_005247.4 | 3735 | 3523 | 212 |
| 653-PTsup | ANO1 | 139 | hg19 | chr11 | 69978117 | 69978117 | Missense_Mutation | SNP | C | C | T | novel | Somatic | T397M | 0.02867 | NM_018043.6 | 5194 | 5045 | 149 |
| 653-PTsup | PPFIA1 | 139 | hg19 | chr11 | 70118289 | 70118289 | Missense_Mutation | SNP | A | A | T | novel | Somatic | E4V | 0.034762 | NM_003626.4 | 2589 | 2499 | 90 |
| 653-PTsup | FAM181B | 151 | hg19 | chr11 | 82443699 | 82443699 | Missense_Mutation | SNP | G | G | A | novel | Somatic | A358V | 0.496901 | NM_175885.4 | 966 | 485 | 481 |
| 653-PTsup | GRIA4 | 173 | hg19 | chr11 | 105845164 | 105845164 | Missense_Mutation | SNP | G | G | A | novel | Somatic | R846K | 0.230769 | NM_000829.4 | 520 | 400 | 120 |
| 653-PTsup | APOA4 | 1475 | hg19 | chr11 | 116693892 | 116693892 | Missense_Mutation | SNP | C | C | T | novel | Somatic | V6M | 0.411095 | NM_000482.4 | 703 | 414 | 289 |
| 653-PTsup | PEX5 | 641 | hg19 | chr12 | 7360267 | 7360267 | Missense_Mutation | SNP | G | G | A | novel | Somatic | E348K | 0.207297 | NM_001300789.1 | 603 | 478 | 125 |
| 653-PTsup | PEX5 | 641 | hg19 | chr12 | 7360309 | 7360309 | Missense_Mutation | SNP | G | G | A | novel | Somatic | E362K | 0.240576 | NM_001300789.1 | 902 | 685 | 217 |
| 653-PTsup | KMT2D | 120 | hg19 | chr12 | 49420432 | 49420432 | Missense_Mutation | SNP | C | C | T | novel | Somatic | R5106H | 0.209073 | NM_003482.3 | 1014 | 802 | 212 |
| 653-PTsup | KRT72 | 989 | hg19 | chr12 | 52995200 | 52995200 | Missense_Mutation | SNP | G | G | A | novel | Somatic | R13C | 0.28513 | NM_080747.3 | 733 | 524 | 209 |
| 653-PTsup | NACA | 1020 | hg19 | chr12 | 57111473 | 57111473 | Missense_Mutation | SNP | G | G | A | novel | Somatic | P1281S | 0.235915 | NM_001365896.1 | 850 | 649 | 201 |
| 653-PTsup | FRY | 104 | hg19 | chr13 | 32869463 | 32869463 | Nonsense_Mutation | SNP | C | C | T | novel | Somatic | Q2970Ter | 0.254321 | NM_023037.3 | 405 | 302 | 103 |
| 653-PTsup | STARD13 | 842 | hg19 | chr13 | 33703085 | 33703085 | Missense_Mutation | SNP | A | A | T | novel | Somatic | S577T | 0.257525 | NM_178006.4 | 598 | 444 | 154 |
| 653-PTsup | COG3 | 936 | hg19 | chr13 | 46085931 | 46085931 | Missense_Mutation | SNP | C | C | T | novel | Somatic | A584V | 0.15534 | NM_031431.4 | 206 | 174 | 32 |
| 653-PTsup | OR4K3 | 740 | hg19 | chr14 | 20336372 | 20336372 | Missense_Mutation | SNP | G | G | T | novel | Somatic | T310N | 0.107456 | NM_001348266.2 | 456 | 407 | 49 |
| 653-PTsup | SLC7A7 | 762 | hg19 | chr14 | 23242892 | 23242892 | Frame_Shift_Del | DEL | AT | AT | - | novel | Somatic | M488VfsTer23 | 0.166096 | NM_001126106.2 | 583 | 486 | 97 |
| 653-PTsup | AJUBA | 763 | hg19 | chr14 | 23443312 | 23443312 | Frame_Shift_Del | DEL | TG | TG | - | novel | Somatic | I479RfsTer13 | 0.153302 | NM_032876.6 | 424 | 359 | 65 |
| 653-PTsup | BRMS1L | 107 | hg19 | chr14 | 36331851 | 36331851 | Missense_Mutation | SNP | G | G | C | novel | Somatic | R166T | 0.189815 | NM_032352.4 | 648 | 525 | 123 |
| 653-PTsup | FANCM | 116 | hg19 | chr14 | 45605671 | 45605671 | Missense_Mutation | SNP | T | T | G | novel | Somatic | L146W | 0.208167 | NM_020937.4 | 1003 | 794 | 209 |
| 653-PTsup | FRMD6 | 983 | hg19 | chr14 | 52186748 | 52186748 | Intron | SNP | A | A | G | novel | Somatic |  | 0.144351 | NM_001267046.2 | 478 | 409 | 69 |
| 653-PTsup | CCDC175 | 130 | hg19 | chr14 | 59992099 | 59992099 | Frame_Shift_Ins | INS | - | - | T | novel | Somatic | N632KfsTer6 | 0.135283 | NM_001164399.2 | 541 | 467 | 74 |
| 653-PTsup | EIF5 | 21 | hg19 | chr14 | 103806764 | 103806764 | Missense_Mutation | SNP | G | G | T | novel | Somatic | A358S | 0.164444 | NM_001969.5 | 224 | 187 | 37 |
| 653-PTsup | NPAP1 | 775 | hg19 | chr15 | 24921279 | 24921279 | Missense_Mutation | SNP | G | G | T | novel | Somatic | G89C | 0.431866 | NM_018958.3 | 953 | 541 | 412 |
| 653-PTsup | ATP10A | 97 | hg19 | chr15 | 25925971 | 25925971 | Missense_Mutation | SNP | C | C | T | novel | Somatic | E1222K | 0.261803 | NM_024490.3 | 465 | 343 | 122 |
| 653-PTsup | ISLR2 | 1152 | hg19 | chr15 | 74425667 | 74425667 | Missense_Mutation | SNP | G | G | T | novel | Somatic | W191L | 0.27449 | NM_020851.3 | 978 | 709 | 269 |
| 653-PTsup | EDC3 | 144 | hg19 | chr15 | 74925234 | 74925234 | Missense_Mutation | DNP | TC | TC | AT | novel | Somatic | N416Y | 0.408299 | NM_025083.5 | 1182 | 690 | 492 |
| 653-PTsup | TBC1D2B | 1182 | hg19 | chr15 | 78305643 | 78305643 | Missense_Mutation | SNP | C | C | T | novel | Somatic | G598R | 0.46732 | NM_144572.1 | 306 | 163 | 143 |
| 653-PTsup | LINS1 | 1356 | hg19 | chr15 | 101110045 | 101110045 | Missense_Mutation | SNP | C | C | T | novel | Somatic | G558S | 0.489796 | NM_001040616.3 | 490 | 250 | 240 |
| 653-PTsup | ZNF263 | 610 | hg19 | chr16 | 3340047 | 3340047 | Missense_Mutation | SNP | G | G | A | novel | Somatic | G514E | 0.399694 | NM_005741.5 | 652 | 391 | 261 |
| 653-PTsup | DNAH3 | 11 | hg19 | chr16 | 21117900 | 21117900 | Missense_Mutation | SNP | G | G | T | novel | Somatic | S732Y | 0.176157 | NM_017539.2 | 561 | 462 | 99 |
| 653-PTsup | ZP2 | 746 | hg19 | chr16 | 21213522 | 21213522 | Missense_Mutation | SNP | C | C | G | novel | Somatic | R397T | 0.199616 | NM_003460.2 | 520 | 416 | 104 |
| 653-PTsup | SCNN1G | 95 | hg19 | chr16 | 23197680 | 23197680 | Missense_Mutation | SNP | C | C | T | novel | Somatic | R30W | 0.233971 | NM_001039.4 | 886 | 678 | 208 |
| 653-PTsup | GTF3C1 | 99 | hg19 | chr16 | 27475965 | 27475965 | Nonsense_Mutation | SNP | G | G | A | novel | Somatic | Q1850Ter | 0.215591 | NM_001520.4 | 820 | 643 | 177 |
| 653-PTsup | PLLP | 1022 | hg19 | chr16 | 57290846 | 57290846 | Missense_Mutation | SNP | C | C | G | novel | Somatic | Q176H | 0.143349 | NM_015993.3 | 872 | 747 | 125 |
| 653-PTsup | CCDC102A | 1024 | hg19 | chr16 | 57562710 | 57562710 | Missense_Mutation | SNP | T | T | C | novel | Somatic | Q127R | 0.112164 | NM_033212.4 | 632 | 561 | 71 |
| 653-PTsup | DNAAF1 | 1227 | hg19 | chr16 | 84209716 | 84209716 | Nonsense_Mutation | SNP | A | A | T | novel | Somatic | K626Ter | 0.154265 | NM_178452.6 | 1102 | 932 | 170 |
| 653-PTsup | ZCCHC14 | 1252 | hg19 | chr16 | 87525633 | 87525633 | Missense_Mutation | SNP | T | T | A | novel | Somatic | D71V | 0.141087 | NM_015144.3 | 1046 | 898 | 148 |
| 653-PTsup | ZNF469 | 1260 | hg19 | chr16 | 88496865 | 88496865 | Missense_Mutation | SNP | G | G | A | novel | Somatic | R996H | 0.182711 | NM_001367624.2 | 509 | 416 | 93 |
| 653-PTsup | PRDM7 | 1272 | hg19 | chr16 | 90141408 | 90141408 | Missense_Mutation | SNP | A | A | C | novel | Somatic | F73V | 0.093548 | NM_001098173.1 | 930 | 843 | 87 |
| 653-PTsup | ZZEF1 | 76 | hg19 | chr17 | 4045957 | 4045957 | Missense_Mutation | SNP | C | C | A | novel | Somatic | G78V | 0.139009 | NM_015113.4 | 927 | 798 | 129 |
| 653-PTsup | NLRP1 | 626 | hg19 | chr17 | 5462779 | 5462779 | Missense_Mutation | SNP | C | C | T | novel | Somatic | D413N | 0.14503 | NM_033004.4 | 986 | 843 | 143 |
| 653-PTsup | PLSCR3 | 640 | hg19 | chr17 | 7296681 | 7296681 | Missense_Mutation | SNP | A | A | G | novel | Somatic | F97L | 0.162437 | NM_020360.4 | 788 | 660 | 128 |
| 653-PTsup | TP53 | 642 | hg19 | chr17 | 7578413 | 7578413 | Missense_Mutation | SNP | C | C | A | novel | Somatic | V173L | 0.525017 | NM_000546.5 | 1499 | 712 | 787 |
| 653-PTsup | CFAP52 | 657 | hg19 | chr17 | 9515797 | 9515797 | Splice_Site | SNP | G | G | C | novel | Somatic |  | 0.160305 | NM_145054.5 | 393 | 330 | 63 |
| 653-PTsup | DNAH9 | 84 | hg19 | chr17 | 11511499 | 11511499 | Missense_Mutation | SNP | G | G | C | novel | Somatic | M157I | 0.191153 | NM_001372.4 | 632 | 511 | 121 |
| 653-PTsup | DNAH9 | 84 | hg19 | chr17 | 11593754 | 11593754 | Splice_Site | SNP | G | G | A | novel | Somatic |  | 0.2 | NM_001372.4 | 565 | 452 | 113 |
| 653-PTsup | KCNJ12 | 747 | hg19 | chr17 | 21319451 | 21319451 | Missense_Mutation | SNP | C | C | T | novel | Somatic | S266L | 0.083085 | NM_021012.5 | 4354 | 3992 | 362 |
| 653-PTsup | TTC25 | 111 | hg19 | chr17 | 40094807 | 40094807 | Splice_Region | SNP | C | C | A | novel | Somatic |  | 0.223448 | NM_031421.5 | 724 | 562 | 162 |
| 653-PTsup | UBTF | 907 | hg19 | chr17 | 42284701 | 42284701 | In_Frame_Del | DEL | TCGTCG | TCGTCG | - | novel | Somatic | D733_D734del | 0.112601 | NM_014233.4 | 746 | 662 | 84 |
| 653-PTsup | KPNA2 | 1088 | hg19 | chr17 | 66040561 | 66040561 | Missense_Mutation | SNP | C | C | G | novel | Somatic | T430S | 0.184486 | NM_002266.4 | 477 | 389 | 88 |
| 653-PTsup | ACOX1 | 1149 | hg19 | chr17 | 73947542 | 73947542 | Missense_Mutation | SNP | C | C | T | novel | Somatic | R354Q | 0.423529 | NM_004035.7 | 595 | 343 | 252 |
| 653-PTsup | TCF3 | 597 | hg19 | chr19 | 1611735 | 1611735 | Missense_Mutation | SNP | C | C | T | novel | Somatic | E646K | 0.180174 | NM_003200.5 | 1381 | 1132 | 249 |
| 653-PTsup | ADGRE1 | 637 | hg19 | chr19 | 6897177 | 6897177 | Missense_Mutation | SNP | C | C | G | novel | Somatic | Q86E | 0.145161 | NM_001974.5 | 496 | 424 | 72 |
| 653-PTsup | CLEC4M | 644 | hg19 | chr19 | 7831545 | 7831545 | Missense_Mutation | SNP | G | G | A | novel | Somatic | R263H | 0.157488 | NM_014257.5 | 1030 | 867 | 163 |
| 653-PTsup | SLC44A2 | 83 | hg19 | chr19 | 10736941 | 10736941 | Missense_Mutation | SNP | A | A | G | novel | Somatic | K17R | 0.140725 | NM_001363611.2 | 469 | 403 | 66 |
| 653-PTsup | RGL3 | 672 | hg19 | chr19 | 11526646 | 11526646 | Missense_Mutation | SNP | C | C | G | novel | Somatic | E202Q | 0.364627 | NM_001161616.3 | 1193 | 758 | 435 |
| 653-PTsup | ECSIT | 673 | hg19 | chr19 | 11618361 | 11618361 | Missense_Mutation | SNP | G | G | A | novel | Somatic | P321L | 0.137868 | NM_016581.5 | 544 | 469 | 75 |
| 653-PTsup | ADGRL1 | 86 | hg19 | chr19 | 14273838 | 14273838 | Missense_Mutation | SNP | C | C | T | novel | Somatic | G264R | 0.184302 | NM_001008701.3 | 1719 | 1402 | 317 |
| 653-PTsup | BRD4 | 702 | hg19 | chr19 | 15367789 | 15367789 | Missense_Mutation | SNP | C | C | T | novel | Somatic | E513K | 0.182453 | NM_058243.2 | 1002 | 819 | 183 |
| 653-PTsup | CYP4F2 | 88 | hg19 | chr19 | 16003384 | 16003384 | Missense_Mutation | SNP | C | C | T | novel | Somatic | A118T | 0.168224 | NM_001082.5 | 1177 | 979 | 198 |
| 653-PTsup | CALR3 | 711 | hg19 | chr19 | 16589968 | 16589968 | Nonsense_Mutation | SNP | G | G | A | novel | Somatic | Q377Ter | 0.184453 | NM_145046.4 | 759 | 619 | 140 |
| 653-PTsup | NWD1 | 89 | hg19 | chr19 | 16842022 | 16842022 | Missense_Mutation | SNP | A | A | G | novel | Somatic | K5R | 0.206897 | NM_001007525.5 | 520 | 412 | 108 |
| 653-PTsup | ANKRD27 | 104 | hg19 | chr19 | 33135312 | 33135312 | Missense_Mutation | SNP | G | G | T | novel | Somatic | H148Q | 0.130809 | NM_032139.3 | 1160 | 1008 | 152 |
| 653-PTsup | HAUS5 | 860 | hg19 | chr19 | 36113830 | 36113830 | Missense_Mutation | SNP | T | T | A | novel | Somatic | S613T | 0.158028 | NM_015302.2 | 791 | 666 | 125 |
| 653-PTsup | RYR1 | 110 | hg19 | chr19 | 38964291 | 38964291 | Missense_Mutation | SNP | G | G | T | novel | Somatic | G1347V | 0.147249 | NM_000540.3 | 1849 | 1576 | 273 |
| 653-PTsup | LTBP4 | 898 | hg19 | chr19 | 41114264 | 41114264 | Missense_Mutation | SNP | G | G | A | novel | Somatic | R499Q | 0.115412 | NM_001042544.1 | 1418 | 1254 | 164 |
| 653-PTsup | KCNA7 | 963 | hg19 | chr19 | 49574036 | 49574036 | Missense_Mutation | SNP | A | A | G | novel | Somatic | W219R | 0.130841 | NM_031886.2 | 1284 | 1116 | 168 |
| 653-PTsup | KLK6 | 977 | hg19 | chr19 | 51465038 | 51465038 | In_Frame_Del | DEL | ACA | ACA | - | novel | Somatic | L181del | 0.081081 | NM_002774.4 | 444 | 408 | 36 |
| 653-PTsup | ZNF468 | 123 | hg19 | chr19 | 53345382 | 53345382 | Missense_Mutation | SNP | C | C | G | novel | Somatic | L55F | 0.112483 | NM_001008801.2 | 729 | 647 | 82 |
| 653-PTsup | NLRP9 | 126 | hg19 | chr19 | 56244334 | 56244334 | Missense_Mutation | SNP | C | C | T | novel | Somatic | R288Q | 0.116667 | NM_176820.4 | 480 | 424 | 56 |
| 653-PTsup | ZNF582 | 1019 | hg19 | chr19 | 56895719 | 56895719 | Missense_Mutation | SNP | C | C | T | novel | Somatic | R387Q | 0.109983 | NM_001320371.2 | 591 | 526 | 65 |
| 653-PTsup | TRIM28 | 1035 | hg19 | chr19 | 59060457 | 59060457 | Silent | SNP | C | C | T | novel | Somatic | P504= | 0.145134 | NM_005762.3 | 1190 | 1017 | 173 |
| 653-PTsup | TMX4 | 80 | hg19 | chr20 | 7963005 | 7963005 | Missense_Mutation | SNP | C | C | G | novel | Somatic | E315Q | 0.224453 | NM_021156.4 | 548 | 425 | 123 |
| 653-PTsup | SPTLC3 | 85 | hg19 | chr20 | 13074168 | 13074168 | Missense_Mutation | SNP | C | C | T | novel | Somatic | S257L | 0.18894 | NM_018327.4 | 433 | 351 | 82 |
| 653-PTsup | ACTR5 | 870 | hg19 | chr20 | 37384617 | 37384617 | Missense_Mutation | SNP | G | G | A | novel | Somatic | A371T | 0.25227 | NM_024855.4 | 989 | 739 | 250 |
| 653-PTsup | ZNF334 | 929 | hg19 | chr20 | 45130480 | 45130480 | Missense_Mutation | SNP | C | C | A | novel | Somatic | V523L | 0.180685 | NM_001353825.1 | 642 | 526 | 116 |
| 653-PTsup | ATP9A | 1 | hg19 | chr20 | 50255904 | 50255904 | Missense_Mutation | SNP | T | T | G | novel | Somatic | K549T | 0.127941 | NM_006045.3 | 680 | 593 | 87 |
| 653-PTsup | SYCP2 | 128 | hg19 | chr20 | 58467047 | 58467047 | Frame_Shift_Del | DEL | T | T | - | novel | Somatic | M788Ter | 0.023707 | NM_014258.4 | 464 | 453 | 11 |
| 653-PTsup | CDH26 | 128 | hg19 | chr20 | 58574656 | 58574656 | Missense_Mutation | SNP | G | G | A | novel | Somatic | E679K | 0.218299 | NM_177980.4 | 623 | 487 | 136 |
| 653-PTsup | NCAM2 | 94 | hg19 | chr21 | 22782690 | 22782690 | Nonsense_Mutation | SNP | C | C | G | novel | Somatic | S431Ter | 0.169072 | NM_004540.5 | 485 | 403 | 82 |
| 653-PTsup | URB1 | 105 | hg19 | chr21 | 33706558 | 33706558 | Missense_Mutation | SNP | C | C | A | novel | Somatic | G1591W | 0.211694 | NM_014825.3 | 992 | 782 | 210 |
| 653-PTsup | KRTAP10-9 | 936 | hg19 | chr21 | 46047486 | 46047486 | Missense_Mutation | SNP | C | C | T | novel | Somatic | S133F | 0.208202 | NM_198690.3 | 1267 | 1003 | 264 |
| 653-PTsup | ZNRF3 | 12 | hg19 | chr22 | 29446070 | 29446070 | Missense_Mutation | SNP | C | C | G | novel | Somatic | P634R | 0.182008 | NM_001206998.2 | 954 | 780 | 174 |
| 653-PTsup | CACNA1I | 111 | hg19 | chr22 | 40075295 | 40075295 | Missense_Mutation | SNP | G | G | T | novel | Somatic | D1747Y | 0.179515 | NM_021096.4 | 907 | 744 | 163 |
| 653-PTsup | MKL1 | 896 | hg19 | chr22 | 40813025 | 40813025 | Missense_Mutation | SNP | A | A | G | novel | Somatic | M848T | 0.182495 | NM_020831.6 | 1071 | 875 | 196 |
| 653-PTsup | FBLN1 | 935 | hg19 | chr22 | 45929685 | 45929685 | Missense_Mutation | SNP | G | G | A | novel | Somatic | E231K | 0.367347 | NM_006486.3 | 833 | 527 | 306 |
| 653-PTsup | IL17REL | 969 | hg19 | chr22 | 50439591 | 50439591 | Missense_Mutation | SNP | G | G | A | novel | Somatic | T67M | 0.188609 | NM_001371417.1 | 1352 | 1097 | 255 |
| 653-PTsup | GK | 819 | hg19 | chrX | 30737535 | 30737535 | Splice_Site | SNP | G | G | C | novel | Somatic |  | 0.345679 | NM_001205019.1 | 79 | 51 | 28 |
| 653-PTsup | GPRASP1 | 1362 | hg19 | chrX | 101910070 | 101910070 | Missense_Mutation | SNP | G | G | C | novel | Somatic | W410S | 0.44523 | NM_001184727.2 | 282 | 156 | 126 |
| 653-PTdeep | SSU72 | 596 | hg19 | chr1 | 1477512 | 1477512 | In_Frame_Ins | INS | - | - | TCGATC | novel | Somatic | E171_I172dup | 0.099804 | NM_014188.3 | 508 | 457 | 51 |
| 653-PTdeep | DNAJC16 | 88 | hg19 | chr1 | 15886055 | 15886055 | In_Frame_Del | DEL | TCA | TCA | - | novel | Somatic | I354del | 0.074074 | NM_015291.4 | 432 | 400 | 32 |
| 653-PTdeep | UQCRHL | 708 | hg19 | chr1 | 16133928 | 16133928 | Missense_Mutation | SNP | C | C | G | novel | Somatic | D73H | 0.117409 | NM_001089591.1 | 741 | 654 | 87 |
| 653-PTdeep | ZMYM6 | 855 | hg19 | chr1 | 35477556 | 35477556 | Missense_Mutation | SNP | G | G | A | novel | Somatic | H333Y | 0.130814 | NM_007167.4 | 344 | 299 | 45 |
| 653-PTdeep | ZMYM1 | 856 | hg19 | chr1 | 35578657 | 35578657 | Missense_Mutation | SNP | C | C | T | novel | Somatic | S409L | 0.102079 | NM_024772.5 | 528 | 474 | 54 |
| 653-PTdeep | AGO4 | 107 | hg19 | chr1 | 36291050 | 36291050 | Nonsense_Mutation | SNP | C | C | G | novel | Somatic | S148Ter | 0.131448 | NM_017629.4 | 600 | 521 | 79 |
| 653-PTdeep | CSF3R | 866 | hg19 | chr1 | 36935409 | 36935409 | Nonsense_Mutation | SNP | G | G | A | novel | Somatic | R440Ter | 0.11898 | NM_156039.3 | 704 | 620 | 84 |
| 653-PTdeep | HIVEP3 | 113 | hg19 | chr1 | 42048097 | 42048097 | In_Frame_Del | DEL | CTC | CTC | - | novel | Somatic | R791del | 0.117829 | NM_024503.5 | 644 | 568 | 76 |
| 653-PTdeep | FAF1 | 15 | hg19 | chr1 | 51050417 | 51050417 | Missense_Mutation | SNP | C | C | G | novel | Somatic | G303R | 0.108108 | NM_007051.3 | 481 | 429 | 52 |
| 653-PTdeep | NRDC | 122 | hg19 | chr1 | 52257726 | 52257726 | Missense_Mutation | SNP | C | C | G | novel | Somatic | Q1024H | 0.16055 | NM_002525.2 | 218 | 183 | 35 |
| 653-PTdeep | LRRC7 | 17 | hg19 | chr1 | 70144122 | 70144122 | Missense_Mutation | SNP | G | G | C | novel | Somatic | E21Q | 0.115385 | NM_001370785.1 | 468 | 414 | 54 |
| 653-PTdeep | TGFBR3 | 2 | hg19 | chr1 | 92185448 | 92185448 | Splice_Site | SNP | A | A | G | novel | Somatic |  | 0.125 | NM_003243.5 | 359 | 314 | 45 |
| 653-PTdeep | LRIF1 | 1435 | hg19 | chr1 | 111494431 | 111494431 | Missense_Mutation | SNP | T | T | A | novel | Somatic | M359L | 0.148342 | NM_018372.4 | 573 | 488 | 85 |
| 653-PTdeep | CEPT1 | 1437 | hg19 | chr1 | 111690538 | 111690538 | Frame_Shift_Del | DEL | ATGCAAG | ATGCAAG | - | novel | Somatic | M68GfsTer26 | 0.099839 | NM_006090.5 | 621 | 559 | 62 |
| 653-PTdeep | CELF3 | 1742 | hg19 | chr1 | 151682224 | 151682224 | Missense_Mutation | SNP | C | C | T | novel | Somatic | R91Q | 0.148325 | NM_007185.7 | 627 | 534 | 93 |
| 653-PTdeep | UBAP2L | 1761 | hg19 | chr1 | 154223643 | 154223643 | Missense_Mutation | SNP | C | C | G | novel | Somatic | A447G | 0.032357 | NM_014847.4 | 647 | 626 | 21 |
| 653-PTdeep | IPO9 | 265 | hg19 | chr1 | 201817642 | 201817642 | Missense_Mutation | SNP | C | C | T | novel | Somatic | P145L | 0.135452 | NM_018085.5 | 598 | 517 | 81 |
| 653-PTdeep | LYST | 2385 | hg19 | chr1 | 235956889 | 235956889 | Missense_Mutation | SNP | G | G | T | novel | Somatic | L1344I | 0.120354 | NM_001301365.1 | 565 | 497 | 68 |
| 653-PTdeep | TSSC1 | Unknown | hg19 | chr2 | 3261143 | 3261143 | Missense_Mutation | SNP | C | C | T | novel | Somatic | D115N | 0.095982 | NM_003310.4 | 895 | 809 | 86 |
| 653-PTdeep | RSAD2 | 638 | hg19 | chr2 | 7036022 | 7036022 | Missense_Mutation | SNP | G | G | C | novel | Somatic | K345N | 0.100604 | NM_080657.5 | 497 | 447 | 50 |
| 653-PTdeep | PRR30 | 793 | hg19 | chr2 | 27360083 | 27360083 | Missense_Mutation | SNP | C | C | T | novel | Somatic | R372Q | 0.25684 | NM_178553.4 | 1128 | 837 | 291 |
| 653-PTdeep | PROKR1 | 1110 | hg19 | chr2 | 68882505 | 68882505 | Missense_Mutation | SNP | G | G | A | novel | Somatic | V327I | 0.195671 | NM_138964.3 | 1152 | 926 | 226 |
| 653-PTdeep | ARHGAP25 | 1111 | hg19 | chr2 | 69045015 | 69045015 | Missense_Mutation | SNP | G | G | A | novel | Somatic | E298K | 0.100686 | NM_001007231.3 | 437 | 393 | 44 |
| 653-PTdeep | ZNF638 | 141 | hg19 | chr2 | 71650548 | 71650548 | Frame_Shift_Ins | INS | - | - | A | novel | Somatic | G1305RfsTer2 | 0.21558 | NM_014497.5 | 545 | 426 | 119 |
| 653-PTdeep | C2orf81 | 1154 | hg19 | chr2 | 74642859 | 74642859 | Missense_Mutation | SNP | C | C | T | novel | Somatic | G149S | 0.140351 | NM_001316764.3 | 1140 | 980 | 160 |
| 653-PTdeep | TEKT4 | 1313 | hg19 | chr2 | 95541339 | 95541339 | Missense_Mutation | SNP | C | C | T | novel | Somatic | R315W | 0.125152 | NM_144705.4 | 822 | 719 | 103 |
| 653-PTdeep | KIAA1211L | 167 | hg19 | chr2 | 99438908 | 99438908 | Missense_Mutation | SNP | C | C | T | novel | Somatic | E610K | 0.198171 | NM_207362.3 | 656 | 526 | 130 |
| 653-PTdeep | ST6GAL2 | 175 | hg19 | chr2 | 107459567 | 107459567 | Missense_Mutation | SNP | G | G | T | novel | Somatic | H289Q | 0.108955 | NM_001322362.2 | 670 | 597 | 73 |
| 653-PTdeep | DPP10 | 22 | hg19 | chr2 | 116599880 | 116599880 | Missense_Mutation | SNP | G | G | A | novel | Somatic | E801K | 0.123223 | NM_001321905.2 | 422 | 370 | 52 |
| 653-PTdeep | ARHGEF4 | 198 | hg19 | chr2 | 131672852 | 131672852 | Missense_Mutation | SNP | G | G | C | novel | Somatic | E445Q | 0.104312 | NM_001367493.1 | 719 | 644 | 75 |
| 653-PTdeep | KCNJ3 | 1771 | hg19 | chr2 | 155711277 | 155711277 | Missense_Mutation | SNP | G | G | A | novel | Somatic | E320K | 0.199295 | NM_002239.4 | 566 | 453 | 113 |
| 653-PTdeep | LY75-CD302 | 226 | hg19 | chr2 | 160676274 | 160676274 | Missense_Mutation | SNP | A | A | T | novel | Somatic | F1372L | 0.117834 | NM_001198759.1 | 314 | 277 | 37 |
| 653-PTdeep | KCNH7 | 28 | hg19 | chr2 | 163693178 | 163693178 | Missense_Mutation | SNP | A | A | G | novel | Somatic | V59A | 0.170313 | NM_033272.4 | 640 | 531 | 109 |
| 653-PTdeep | KLHL41 | 1884 | hg19 | chr2 | 170366628 | 170366628 | Missense_Mutation | SNP | C | C | G | novel | Somatic | Q114E | 0.223622 | NM_006063.3 | 635 | 493 | 142 |
| 653-PTdeep | NFE2L2 | 242 | hg19 | chr2 | 178098960 | 178098960 | Missense_Mutation | SNP | C | C | G | novel | Somatic | D29H | 0.229709 | NM_006164.5 | 653 | 503 | 150 |
| 653-PTdeep | ZNF804A | 31 | hg19 | chr2 | 185801390 | 185801390 | Missense_Mutation | SNP | G | G | C | novel | Somatic | E423Q | 0.215852 | NM_194250.2 | 593 | 465 | 128 |
| 653-PTdeep | ABCA12 | 278 | hg19 | chr2 | 215891652 | 215891652 | Missense_Mutation | SNP | G | G | C | novel | Somatic | L358V | 0.144638 | NM_173076.3 | 401 | 343 | 58 |
| 653-PTdeep | IGFBP2 | 2244 | hg19 | chr2 | 217498593 | 217498593 | Missense_Mutation | SNP | G | G | C | novel | Somatic | G116A | 0.126543 | NM_000597.3 | 647 | 565 | 82 |
| 653-PTdeep | SLC19A3 | 2328 | hg19 | chr2 | 228563751 | 228563751 | Missense_Mutation | SNP | C | C | G | novel | Somatic | G227A | 0.148377 | NM_025243.4 | 647 | 551 | 96 |
| 653-PTdeep | GRM7 | 9 | hg19 | chr3 | 7340373 | 7340373 | Nonsense_Mutation | SNP | G | G | T | novel | Somatic | G247Ter | 0.126273 | NM_000844.4 | 490 | 428 | 62 |
| 653-PTdeep | CAND2 | 85 | hg19 | chr3 | 12838250 | 12838250 | Missense_Mutation | SNP | C | C | T | novel | Somatic | L11F | 0.11878 | NM_001162499.2 | 623 | 549 | 74 |
| 653-PTdeep | GOLGA4 | 108 | hg19 | chr3 | 37388770 | 37388770 | Missense_Mutation | SNP | A | A | G | novel | Somatic | M2202V | 0.136919 | NM_001172713.2 | 409 | 353 | 56 |
| 653-PTdeep | USP19 | 119 | hg19 | chr3 | 49146570 | 49146570 | Missense_Mutation | SNP | C | C | T | novel | Somatic | E1362K | 0.254962 | NM_001351100.1 | 655 | 488 | 167 |
| 653-PTdeep | PBRM1 | 123 | hg19 | chr3 | 52678784 | 52678784 | Frame_Shift_Del | DEL | T | T | - | novel | Somatic | I279YfsTer4 | 0.2512 | NM_018313.5 | 624 | 467 | 157 |
| 653-PTdeep | DNAH12 | 1023 | hg19 | chr3 | 57509313 | 57509313 | Frame_Shift_Del | DEL | T | T | - | novel | Somatic | G93DfsTer7 | 0.028571 | NM_001366028.2 | 419 | 407 | 12 |
| 653-PTdeep | PDZRN3 | 1145 | hg19 | chr3 | 73433781 | 73433781 | Missense_Mutation | SNP | A | A | G | novel | Somatic | F646L | 0.136415 | NM_015009.3 | 886 | 765 | 121 |
| 653-PTdeep | GOLGB1 | 1511 | hg19 | chr3 | 121416186 | 121416186 | Missense_Mutation | SNP | C | C | T | novel | Somatic | E1062K | 0.116114 | NM_001256486.2 | 422 | 373 | 49 |
| 653-PTdeep | KALRN | 191 | hg19 | chr3 | 124380746 | 124380746 | Missense_Mutation | SNP | A | A | G | novel | Somatic | M2105V | 0.121019 | NM_001024660.4 | 471 | 414 | 57 |
| 653-PTdeep | ATR | 208 | hg19 | chr3 | 142226756 | 142226756 | Intron | SNP | C | C | G | novel | Somatic |  | 0.135838 | NM_001184.4 | 346 | 299 | 47 |
| 653-PTdeep | SCHIP1 | 1802 | hg19 | chr3 | 159583964 | 159583964 | Frame_Shift_Del | DEL | GA | GA | - | novel | Somatic | E260VfsTer13 | 0.083673 | NM_014575.3 | 489 | 448 | 41 |
| 653-PTdeep | BCHE | 230 | hg19 | chr3 | 165548026 | 165548026 | Missense_Mutation | SNP | C | C | T | novel | Somatic | E266K | 0.271307 | NM_000055.4 | 704 | 513 | 191 |
| 653-PTdeep | MSX1 | 622 | hg19 | chr4 | 4864727 | 4864727 | Missense_Mutation | SNP | C | C | G | novel | Somatic | L257V | 0.130312 | NM_002448.3 | 706 | 614 | 92 |
| 653-PTdeep | SEL1L3 | 97 | hg19 | chr4 | 25780780 | 25780780 | Missense_Mutation | SNP | T | T | C | novel | Somatic | M835V | 0.130326 | NM_015187.5 | 399 | 347 | 52 |
| 653-PTdeep | NFXL1 | 950 | hg19 | chr4 | 47886406 | 47886406 | Missense_Mutation | SNP | G | G | C | novel | Somatic | Q625E | 0.19646 | NM_001278624.2 | 564 | 453 | 111 |
| 653-PTdeep | ANKRD17 | 143 | hg19 | chr4 | 73985976 | 73985976 | Missense_Mutation | SNP | C | C | T | novel | Somatic | D1310N | 0.139373 | NM_032217.5 | 573 | 493 | 80 |
| 653-PTdeep | SEC31A | 152 | hg19 | chr4 | 83785565 | 83785565 | Frame_Shift_Del | DEL | T | T | - | novel | Somatic | I462LfsTer16 | 0.028463 | NM_001318120.1 | 527 | 512 | 15 |
| 653-PTdeep | TET2 | 1394 | hg19 | chr4 | 106156531 | 106156531 | Missense_Mutation | SNP | G | G | A | novel | Somatic | E478K | 0.140449 | NM_001127208.2 | 533 | 458 | 75 |
| 653-PTdeep | GIMD1 | 1403 | hg19 | chr4 | 107288225 | 107288225 | Missense_Mutation | SNP | A | A | G | novel | Somatic | V123A | 0.143403 | NM_001195138.1 | 523 | 448 | 75 |
| 653-PTdeep | ALPK1 | 22 | hg19 | chr4 | 113298937 | 113298937 | Missense_Mutation | SNP | A | A | C | novel | Somatic | N2H | 0.332447 | NM_025144.4 | 376 | 251 | 125 |
| 653-PTdeep | PLRG1 | 1771 | hg19 | chr4 | 155461792 | 155461792 | Frame_Shift_Del | DEL | G | G | - | novel | Somatic | P298RfsTer7 | 0.107926 | NM_002669.4 | 592 | 528 | 64 |
| 653-PTdeep | SLC6A3 | 74 | hg19 | chr5 | 1401052 | 1401052 | Missense_Mutation | SNP | C | C | T | novel | Somatic | R606K | 0.11396 | NM_001044.5 | 702 | 622 | 80 |
| 653-PTdeep | ADAMTS16 | 9 | hg19 | chr5 | 5146365 | 5146365 | Missense_Mutation | SNP | C | C | T | novel | Somatic | R100W | 0.11526 | NM_139056.4 | 616 | 545 | 71 |
| 653-PTdeep | PAPD7 | 636 | hg19 | chr5 | 6714482 | 6714482 | Missense_Mutation | SNP | C | C | T | novel | Somatic | S129L | 0.123223 | NM_006999.6 | 211 | 185 | 26 |
| 653-PTdeep | SEMA5A | 10 | hg19 | chr5 | 9052090 | 9052090 | Missense_Mutation | SNP | G | G | T | novel | Somatic | Q914K | 0.120599 | NM_003966.3 | 1136 | 999 | 137 |
| 653-PTdeep | 6-Mar | 82 | hg19 | chr5 | 10403604 | 10403604 | Missense_Mutation | SNP | G | G | A | novel | Somatic | G428E | 0.110193 | NM_005885.4 | 363 | 323 | 40 |
| 653-PTdeep | SPEF2 | 13 | hg19 | chr5 | 35740257 | 35740257 | Missense_Mutation | SNP | G | G | T | novel | Somatic | R1073L | 0.093264 | NM_024867.4 | 385 | 349 | 36 |
| 653-PTdeep | FBN2 | 194 | hg19 | chr5 | 127680126 | 127680126 | Nonsense_Mutation | SNP | G | G | T | novel | Somatic | C1098Ter | 0.112676 | NM_001999.4 | 426 | 378 | 48 |
| 653-PTdeep | PKD2L2 | 203 | hg19 | chr5 | 137260794 | 137260794 | Splice_Site | SNP | G | G | T | novel | Somatic |  | 0.141414 | NM_001300921.2 | 589 | 505 | 84 |
| 653-PTdeep | SLC34A1 | 241 | hg19 | chr5 | 176815371 | 176815371 | Nonsense_Mutation | SNP | C | C | T | novel | Somatic | Q312Ter | 0.128571 | NM_003052.5 | 420 | 366 | 54 |
| 653-PTdeep | HIST1H1B | 797 | hg19 | chr6 | 27834675 | 27834675 | In_Frame_Del | DEL | TGC | TGC | - | novel | Somatic | A211del | 0.114685 | NM_005322.2 | 715 | 633 | 82 |
| 653-PTdeep | TREM2 | 898 | hg19 | chr6 | 41129020 | 41129020 | Silent | SNP | G | G | T | novel | Somatic | V124= | 0.132075 | NM_018965.4 | 318 | 276 | 42 |
| 653-PTdeep | CUL9 | 914 | hg19 | chr6 | 43190294 | 43190294 | Missense_Mutation | SNP | G | G | A | novel | Somatic | R2316Q | 0.127711 | NM_015089.4 | 829 | 723 | 106 |
| 653-PTdeep | ZNF318 | 915 | hg19 | chr6 | 43306322 | 43306322 | Missense_Mutation | SNP | C | C | A | novel | Somatic | S1805I | 0.106132 | NM_014345.3 | 423 | 378 | 45 |
| 653-PTdeep | LGSN | 1073 | hg19 | chr6 | 63990395 | 63990395 | Missense_Mutation | SNP | G | G | A | novel | Somatic | A354V | 0.238276 | NM_016571.3 | 788 | 600 | 188 |
| 653-PTdeep | CEP85L | 186 | hg19 | chr6 | 118886893 | 118886893 | Frame_Shift_Del | DEL | T | T | - | novel | Somatic | E273DfsTer6 | 0.028626 | NM_001042475.3 | 524 | 509 | 15 |
| 653-PTdeep | PTPRK | 195 | hg19 | chr6 | 128540093 | 128540093 | Missense_Mutation | SNP | G | G | A | novel | Somatic | S281F | 0.132632 | NM_001291981.2 | 474 | 411 | 63 |
| 653-PTdeep | PHF14 | 83 | hg19 | chr7 | 11101655 | 11101655 | Frame_Shift_Ins | INS | - | - | T | novel | Somatic | L867FfsTer4 | 0.079208 | NM_001007157.2 | 302 | 278 | 24 |
| 653-PTdeep | HOXA6 | 792 | hg19 | chr7 | 27186940 | 27186940 | Missense_Mutation | SNP | C | C | T | novel | Somatic | M143I | 0.248311 | NM_024014.4 | 591 | 444 | 147 |
| 653-PTdeep | NOD1 | 817 | hg19 | chr7 | 30491628 | 30491628 | Missense_Mutation | SNP | G | G | A | novel | Somatic | R469W | 0.126953 | NM_006092.4 | 1023 | 893 | 130 |
| 653-PTdeep | STAG3 | 1346 | hg19 | chr7 | 99796499 | 99796499 | Missense_Mutation | SNP | G | G | T | novel | Somatic | M462I | 0.12844 | NM_001282717.1 | 436 | 380 | 56 |
| 653-PTdeep | GIGYF1 | 1350 | hg19 | chr7 | 100285169 | 100285169 | Frame_Shift_Ins | INS | - | - | G | novel | Somatic | L111PfsTer20 | 0.025298 | NM_022574.5 | 669 | 652 | 17 |
| 653-PTdeep | PIK3CG | 1397 | hg19 | chr7 | 106524631 | 106524631 | Missense_Mutation | SNP | C | C | G | novel | Somatic | S931C | 0.147249 | NM_002649.3 | 618 | 527 | 91 |
| 653-PTdeep | CADPS2 | 189 | hg19 | chr7 | 122255307 | 122255307 | Missense_Mutation | SNP | C | C | T | novel | Somatic | R384Q | 0.132353 | NM_001363389.1 | 476 | 413 | 63 |
| 653-PTdeep | KMT2C | 27 | hg19 | chr7 | 151874148 | 151874148 | Frame_Shift_Del | DEL | T | T | - | novel | Somatic | K2797RfsTer26 | 0.041262 | NM_170606.3 | 409 | 392 | 17 |
| 653-PTdeep | ADRB3 | 873 | hg19 | chr8 | 37823224 | 37823224 | Missense_Mutation | SNP | G | G | A | novel | Somatic | S255L | 0.170213 | NM_000025.3 | 234 | 194 | 40 |
| 653-PTdeep | LETM2 | 876 | hg19 | chr8 | 38250329 | 38250329 | Missense_Mutation | SNP | A | A | T | novel | Somatic | Q106L | 0.258359 | NM_001286819.1 | 658 | 488 | 170 |
| 653-PTdeep | ZFHX4 | 18 | hg19 | chr8 | 77767085 | 77767085 | Missense_Mutation | SNP | G | G | C | novel | Somatic | G2643A | 0.186312 | NM_024721.5 | 788 | 641 | 147 |
| 653-PTdeep | SNX16 | 1216 | hg19 | chr8 | 82736029 | 82736029 | Missense_Mutation | SNP | G | G | C | novel | Somatic | N203K | 0.108401 | NM_022133.3 | 369 | 329 | 40 |
| 653-PTdeep | CNGB3 | 156 | hg19 | chr8 | 87683211 | 87683211 | Missense_Mutation | SNP | C | C | G | novel | Somatic | E152Q | 0.285047 | NM_019098.4 | 642 | 459 | 183 |
| 653-PTdeep | SLC26A7 | 2 | hg19 | chr8 | 92375765 | 92375765 | Missense_Mutation | SNP | G | G | A | novel | Somatic | S496N | 0.128571 | NM_134266.2 | 418 | 364 | 54 |
| 653-PTdeep | RGS22 | 169 | hg19 | chr8 | 101059824 | 101059824 | Missense_Mutation | SNP | T | T | C | novel | Somatic | K564E | 0.158537 | NM_015668.5 | 246 | 207 | 39 |
| 653-PTdeep | PKHD1L1 | 178 | hg19 | chr8 | 110412510 | 110412510 | Missense_Mutation | SNP | C | C | G | novel | Somatic | F406L | 0.152344 | NM_177531.6 | 512 | 434 | 78 |
| 653-PTdeep | SNTB1 | 23 | hg19 | chr8 | 121706103 | 121706103 | Missense_Mutation | SNP | G | G | A | novel | Somatic | P206L | 0.064161 | NM_021021.4 | 1088 | 1018 | 70 |
| 653-PTdeep | EFR3A | 1599 | hg19 | chr8 | 132966087 | 132966087 | Missense_Mutation | SNP | G | G | A | novel | Somatic | G171S | 0.050749 | NM_015137.6 | 1198 | 1137 | 61 |
| 653-PTdeep | JAK2 | 77 | hg19 | chr9 | 5050712 | 5050712 | Missense_Mutation | SNP | G | G | C | novel | Somatic | W165C | 0.112299 | NM_004972.3 | 560 | 497 | 63 |
| 653-PTdeep | KDM4C | 636 | hg19 | chr9 | 7103848 | 7103848 | Missense_Mutation | SNP | G | G | C | novel | Somatic | R863P | 0.099476 | NM_015061.5 | 381 | 343 | 38 |
| 653-PTdeep | MPDZ | 85 | hg19 | chr9 | 13188782 | 13188782 | Splice_Site | SNP | C | C | T | novel | Somatic |  | 0.250794 | NM_001330637.1 | 314 | 235 | 79 |
| 653-PTdeep | FOCAD | 11 | hg19 | chr9 | 20717846 | 20717846 | Missense_Mutation | SNP | G | G | C | novel | Somatic | K37N | 0.137931 | NM_017794.4 | 665 | 573 | 92 |
| 653-PTdeep | DDX58 | 12 | hg19 | chr9 | 32459463 | 32459463 | Missense_Mutation | SNP | T | T | A | novel | Somatic | K796I | 0.084279 | NM_014314.4 | 617 | 565 | 52 |
| 653-PTdeep | PCSK5 | 18 | hg19 | chr9 | 78686768 | 78686768 | Missense_Mutation | SNP | G | G | A | novel | Somatic | G283E | 0.111216 | NM_001372043.1 | 1061 | 943 | 118 |
| 653-PTdeep | ROR2 | 163 | hg19 | chr9 | 94487206 | 94487206 | Missense_Mutation | SNP | C | C | T | novel | Somatic | E524K | 0.118708 | NM_004560.4 | 1053 | 928 | 125 |
| 653-PTdeep | FBP1 | 165 | hg19 | chr9 | 97369228 | 97369228 | Missense_Mutation | SNP | C | C | T | novel | Somatic | G192R | 0.111648 | NM_000507.4 | 1654 | 1469 | 185 |
| 653-PTdeep | RNF20 | 1380 | hg19 | chr9 | 104309662 | 104309662 | Intron | SNP | T | T | G | novel | Somatic |  | 0.140794 | NM_019592.7 | 277 | 238 | 39 |
| 653-PTdeep | SVEP1 | 22 | hg19 | chr9 | 113217997 | 113217997 | Intron | SNP | C | C | T | novel | Somatic |  | 0.091429 | NM_153366.4 | 350 | 318 | 32 |
| 653-PTdeep | TLR4 | 1504 | hg19 | chr9 | 120476729 | 120476729 | Nonsense_Mutation | SNP | G | G | T | novel | Somatic | E775Ter | 0.111732 | NM_138554.5 | 537 | 477 | 60 |
| 653-PTdeep | OR1L3 | 1542 | hg19 | chr9 | 125437535 | 125437535 | Missense_Mutation | SNP | C | C | A | novel | Somatic | L43M | 0.231281 | NM_001005234.1 | 599 | 460 | 139 |
| 653-PTdeep | AK8 | 202 | hg19 | chr9 | 135739089 | 135739089 | Missense_Mutation | SNP | G | G | A | novel | Somatic | T99M | 0.164179 | NM_152572.3 | 469 | 392 | 77 |
| 653-PTdeep | COL5A1 | 204 | hg19 | chr9 | 137686952 | 137686952 | Missense_Mutation | SNP | C | C | T | novel | Somatic | R909W | 0.135032 | NM_001278074.1 | 785 | 679 | 106 |
| 653-PTdeep | C10orf67 | 765 | hg19 | chr10 | 23592373 | 23592373 | Missense_Mutation | SNP | C | C | G | novel | Somatic | E189Q | 0.13355 | NM_001365862.2 | 306 | 265 | 41 |
| 653-PTdeep | LOC100505502 | Unknown | hg19 | chr10 | 31652039 | 31652039 | Missense_Mutation | SNP | G | G | A | novel | Somatic | P239S | 0.128187 | NM_001368169.1 | 1451 | 1265 | 186 |
| 653-PTdeep | WDFY4 | 120 | hg19 | chr10 | 49944069 | 49944069 | Missense_Mutation | SNP | G | G | C | novel | Somatic | C611S | 0.150228 | NM_020945.2 | 657 | 558 | 99 |
| 653-PTdeep | CDHR1 | 1240 | hg19 | chr10 | 85974270 | 85974270 | Missense_Mutation | SNP | C | C | G | novel | Somatic | P825A | 0.1331 | NM_033100.4 | 1141 | 989 | 152 |
| 653-PTdeep | LGI1 | 164 | hg19 | chr10 | 95557238 | 95557238 | Missense_Mutation | SNP | T | T | A | novel | Somatic | F451Y | 0.099415 | NM_005097.4 | 513 | 462 | 51 |
| 653-PTdeep | HPS1 | 1349 | hg19 | chr10 | 100186986 | 100186986 | Frame_Shift_Ins | INS | - | - | G | novel | Somatic | M325HfsTer128 | 0.032037 | NM_000195.5 | 436 | 422 | 14 |
| 653-PTdeep | NT5C2 | 21 | hg19 | chr10 | 104850373 | 104850373 | Missense_Mutation | SNP | C | C | A | novel | Somatic | V490F | 0.148052 | NM_001351173.1 | 385 | 328 | 57 |
| 653-PTdeep | ITPRIP | 1394 | hg19 | chr10 | 106075218 | 106075218 | Missense_Mutation | SNP | C | C | T | novel | Somatic | E198K | 0.148883 | NM_033397.3 | 806 | 686 | 120 |
| 653-PTdeep | JAKMIP3 | 200 | hg19 | chr10 | 133981856 | 133981856 | Missense_Mutation | SNP | G | G | A | novel | Somatic | V978I | 0.141998 | NM_001323086.2 | 831 | 713 | 118 |
| 653-PTdeep | MUC5B | 594 | hg19 | chr11 | 1262533 | 1262533 | Missense_Mutation | SNP | C | C | T | novel | Somatic | R1475W | 0.106929 | NM_002458.3 | 1168 | 1043 | 125 |
| 653-PTdeep | OR51H1 | 622 | hg19 | chr11 | 4881331 | 4881331 | Missense_Mutation | SNP | A | A | G | novel | Somatic | V155A | 0.096386 | NM_001348291.1 | 497 | 449 | 48 |
| 653-PTdeep | OR52N5 | 629 | hg19 | chr11 | 5799805 | 5799805 | Missense_Mutation | SNP | A | A | T | novel | Somatic | F20L | 0.096606 | NM_001001922.2 | 383 | 346 | 37 |
| 653-PTdeep | TAF10 | 635 | hg19 | chr11 | 6632922 | 6632922 | Missense_Mutation | SNP | C | C | G | novel | Somatic | L120F | 0.096591 | NM_006284.4 | 880 | 795 | 85 |
| 653-PTdeep | RNF141 | 665 | hg19 | chr11 | 10536581 | 10536581 | Missense_Mutation | SNP | C | C | T | novel | Somatic | R192H | 0.108219 | NM_016422.4 | 729 | 650 | 79 |
| 653-PTdeep | NAV2 | 92 | hg19 | chr11 | 20114706 | 20114706 | Missense_Mutation | SNP | G | G | C | novel | Somatic | K1959N | 0.358553 | NM_145117.5 | 608 | 390 | 218 |
| 653-PTdeep | DBX1 | 738 | hg19 | chr11 | 20181645 | 20181645 | Missense_Mutation | SNP | C | C | G | novel | Somatic | D76H | 0.127784 | NM_001029865.4 | 853 | 744 | 109 |
| 653-PTdeep | DCDC1 | 102 | hg19 | chr11 | 30946913 | 30946913 | Missense_Mutation | SNP | C | C | A | novel | Somatic | L980F | 0.189433 | NM_001367979.1 | 776 | 629 | 147 |
| 653-PTdeep | SLC43A3 | 1021 | hg19 | chr11 | 57175323 | 57175323 | Missense_Mutation | SNP | G | G | A | novel | Somatic | P486L | 0.268852 | NM_001278206.2 | 305 | 223 | 82 |
| 653-PTdeep | OR5B12 | 1029 | hg19 | chr11 | 58206931 | 58206931 | Missense_Mutation | SNP | G | G | A | novel | Somatic | R232C | 0.248307 | NM_001004733.2 | 443 | 333 | 110 |
| 653-PTdeep | PITPNM1 | 1098 | hg19 | chr11 | 67269450 | 67269450 | Missense_Mutation | SNP | C | C | T | novel | Somatic | D175N | 0.290608 | NM_004910.3 | 905 | 642 | 263 |
| 653-PTdeep | ANO1 | 139 | hg19 | chr11 | 69978117 | 69978117 | Missense_Mutation | SNP | C | C | T | novel | Somatic | T397M | 0.034558 | NM_018043.6 | 3646 | 3520 | 126 |
| 653-PTdeep | PPFIA1 | 139 | hg19 | chr11 | 70118289 | 70118289 | Missense_Mutation | SNP | A | A | T | novel | Somatic | E4V | 0.036592 | NM_003626.4 | 1747 | 1683 | 64 |
| 653-PTdeep | FAM181B | 151 | hg19 | chr11 | 82443699 | 82443699 | Missense_Mutation | SNP | G | G | A | novel | Somatic | A358V | 0.355392 | NM_175885.4 | 815 | 525 | 290 |
| 653-PTdeep | GRIA4 | 173 | hg19 | chr11 | 105845164 | 105845164 | Missense_Mutation | SNP | G | G | A | novel | Somatic | R846K | 0.16548 | NM_000829.4 | 560 | 467 | 93 |
| 653-PTdeep | C11orf53 | 22 | hg19 | chr11 | 111156397 | 111156397 | Missense_Mutation | SNP | C | C | G | novel | Somatic | T162R | 0.02026 | NM_198498.3 | 691 | 677 | 14 |
| 653-PTdeep | APOA4 | 1475 | hg19 | chr11 | 116693892 | 116693892 | Missense_Mutation | SNP | C | C | T | novel | Somatic | V6M | 0.244094 | NM_000482.4 | 634 | 479 | 155 |
| 653-PTdeep | PATE3 | 1543 | hg19 | chr11 | 125659352 | 125659352 | Missense_Mutation | SNP | G | G | T | novel | Somatic | V41F | 0.054118 | NM_001129883.4 | 424 | 401 | 23 |
| 653-PTdeep | PEX5 | 641 | hg19 | chr12 | 7360267 | 7360267 | Missense_Mutation | SNP | G | G | A | novel | Somatic | E348K | 0.178368 | NM_001300789.1 | 527 | 433 | 94 |
| 653-PTdeep | PEX5 | 641 | hg19 | chr12 | 7360309 | 7360309 | Missense_Mutation | SNP | G | G | A | novel | Somatic | E362K | 0.194049 | NM_001300789.1 | 770 | 620 | 150 |
| 653-PTdeep | KMT2D | 120 | hg19 | chr12 | 49420432 | 49420432 | Missense_Mutation | SNP | C | C | T | novel | Somatic | R5106H | 0.131441 | NM_003482.3 | 950 | 825 | 125 |
| 653-PTdeep | KRT72 | 989 | hg19 | chr12 | 52995200 | 52995200 | Missense_Mutation | SNP | G | G | A | novel | Somatic | R13C | 0.139573 | NM_080747.3 | 607 | 522 | 85 |
| 653-PTdeep | NACA | 1020 | hg19 | chr12 | 57111473 | 57111473 | Missense_Mutation | SNP | G | G | A | novel | Somatic | P1281S | 0.1598 | NM_001365896.1 | 798 | 670 | 128 |
| 653-PTdeep | FRY | 104 | hg19 | chr13 | 32869463 | 32869463 | Nonsense_Mutation | SNP | C | C | T | novel | Somatic | Q2970Ter | 0.161692 | NM_023037.3 | 402 | 337 | 65 |
| 653-PTdeep | STARD13 | 842 | hg19 | chr13 | 33703085 | 33703085 | Missense_Mutation | SNP | A | A | T | novel | Somatic | S577T | 0.156187 | NM_178006.4 | 493 | 416 | 77 |
| 653-PTdeep | COG3 | 936 | hg19 | chr13 | 46085931 | 46085931 | Missense_Mutation | SNP | C | C | T | novel | Somatic | A584V | 0.124444 | NM_031431.4 | 225 | 197 | 28 |
| 653-PTdeep | OR4K3 | 740 | hg19 | chr14 | 20336372 | 20336372 | Missense_Mutation | SNP | G | G | T | novel | Somatic | T310N | 0.065076 | NM_001348266.2 | 460 | 430 | 30 |
| 653-PTdeep | SLC7A7 | 762 | hg19 | chr14 | 23242892 | 23242892 | Frame_Shift_Del | DEL | AT | AT | - | novel | Somatic | M488VfsTer23 | 0.085821 | NM_001126106.2 | 536 | 490 | 46 |
| 653-PTdeep | AJUBA | 763 | hg19 | chr14 | 23443312 | 23443312 | Frame_Shift_Del | DEL | TG | TG | - | novel | Somatic | I479RfsTer13 | 0.143939 | NM_032876.6 | 396 | 339 | 57 |
| 653-PTdeep | BRMS1L | 107 | hg19 | chr14 | 36331851 | 36331851 | Missense_Mutation | SNP | G | G | C | novel | Somatic | R166T | 0.122542 | NM_032352.4 | 661 | 580 | 81 |
| 653-PTdeep | FANCM | 116 | hg19 | chr14 | 45605671 | 45605671 | Missense_Mutation | SNP | T | T | G | novel | Somatic | L146W | 0.122358 | NM_020937.4 | 898 | 788 | 110 |
| 653-PTdeep | FRMD6 | 983 | hg19 | chr14 | 52186748 | 52186748 | Intron | SNP | A | A | G | novel | Somatic |  | 0.143868 | NM_001267046.2 | 424 | 363 | 61 |
| 653-PTdeep | CCDC175 | 130 | hg19 | chr14 | 59992099 | 59992099 | Frame_Shift_Ins | INS | - | - | T | novel | Somatic | N632KfsTer6 | 0.102317 | NM_001164399.2 | 509 | 456 | 53 |
| 653-PTdeep | CCDC175 | 130 | hg19 | chr14 | 60031870 | 60031870 | Missense_Mutation | SNP | C | C | G | novel | Somatic | K205N | 0.083045 | NM_001164399.2 | 577 | 529 | 48 |
| 653-PTdeep | BTBD7 | 1300 | hg19 | chr14 | 93761192 | 93761192 | Frame_Shift_Ins | INS | - | - | T | novel | Somatic | R59EfsTer14 | 0.02 | NM_001002860.4 | 594 | 582 | 12 |
| 653-PTdeep | EIF5 | 21 | hg19 | chr14 | 103806764 | 103806764 | Missense_Mutation | SNP | G | G | T | novel | Somatic | A358S | 0.087786 | NM_001969.5 | 260 | 237 | 23 |
| 653-PTdeep | NPAP1 | 775 | hg19 | chr15 | 24921279 | 24921279 | Missense_Mutation | SNP | G | G | T | novel | Somatic | G89C | 0.280874 | NM_018958.3 | 914 | 657 | 257 |
| 653-PTdeep | ATP10A | 97 | hg19 | chr15 | 25925971 | 25925971 | Missense_Mutation | SNP | C | C | T | novel | Somatic | E1222K | 0.154613 | NM_024490.3 | 401 | 339 | 62 |
| 653-PTdeep | ISLR2 | 1152 | hg19 | chr15 | 74425667 | 74425667 | Missense_Mutation | SNP | G | G | T | novel | Somatic | W191L | 0.168172 | NM_020851.3 | 885 | 736 | 149 |
| 653-PTdeep | EDC3 | 144 | hg19 | chr15 | 74925234 | 74925234 | Missense_Mutation | DNP | TC | TC | AT | novel | Somatic | N416Y | 0.277045 | NM_025083.5 | 1127 | 812 | 315 |
| 653-PTdeep | TBC1D2B | 1182 | hg19 | chr15 | 78305643 | 78305643 | Missense_Mutation | SNP | C | C | T | novel | Somatic | G598R | 0.289562 | NM_144572.1 | 297 | 211 | 86 |
| 653-PTdeep | LINS1 | 1356 | hg19 | chr15 | 101110045 | 101110045 | Missense_Mutation | SNP | C | C | T | novel | Somatic | G558S | 0.286344 | NM_001040616.3 | 453 | 323 | 130 |
| 653-PTdeep | ZNF263 | 610 | hg19 | chr16 | 3340047 | 3340047 | Missense_Mutation | SNP | G | G | A | novel | Somatic | G514E | 0.273038 | NM_005741.5 | 584 | 424 | 160 |
| 653-PTdeep | DNAH3 | 11 | hg19 | chr16 | 21117900 | 21117900 | Missense_Mutation | SNP | G | G | T | novel | Somatic | S732Y | 0.118443 | NM_017539.2 | 590 | 520 | 70 |
| 653-PTdeep | ZP2 | 746 | hg19 | chr16 | 21213522 | 21213522 | Missense_Mutation | SNP | C | C | G | novel | Somatic | R397T | 0.115596 | NM_003460.2 | 545 | 482 | 63 |
| 653-PTdeep | SCNN1G | 95 | hg19 | chr16 | 23197680 | 23197680 | Missense_Mutation | SNP | C | C | T | novel | Somatic | R30W | 0.137157 | NM_001039.4 | 802 | 692 | 110 |
| 653-PTdeep | GTF3C1 | 99 | hg19 | chr16 | 27475965 | 27475965 | Nonsense_Mutation | SNP | G | G | A | novel | Somatic | Q1850Ter | 0.155612 | NM_001520.4 | 782 | 660 | 122 |
| 653-PTdeep | PLLP | 1022 | hg19 | chr16 | 57290846 | 57290846 | Missense_Mutation | SNP | C | C | G | novel | Somatic | Q176H | 0.113287 | NM_015993.3 | 715 | 634 | 81 |
| 653-PTdeep | CCDC102A | 1024 | hg19 | chr16 | 57562710 | 57562710 | Missense_Mutation | SNP | T | T | C | novel | Somatic | Q127R | 0.054863 | NM_033212.4 | 401 | 379 | 22 |
| 653-PTdeep | DNAAF1 | 1227 | hg19 | chr16 | 84209716 | 84209716 | Nonsense_Mutation | SNP | A | A | T | novel | Somatic | K626Ter | 0.079384 | NM_178452.6 | 844 | 777 | 67 |
| 653-PTdeep | ZCCHC14 | 1252 | hg19 | chr16 | 87525633 | 87525633 | Missense_Mutation | SNP | T | T | A | novel | Somatic | D71V | 0.092191 | NM_015144.3 | 921 | 836 | 85 |
| 653-PTdeep | ZNF469 | 1260 | hg19 | chr16 | 88496865 | 88496865 | Missense_Mutation | SNP | G | G | A | novel | Somatic | R996H | 0.158273 | NM_001367624.2 | 277 | 233 | 44 |
| 653-PTdeep | PRDM7 | 1272 | hg19 | chr16 | 90141408 | 90141408 | Missense_Mutation | SNP | A | A | C | novel | Somatic | F73V | 0.083333 | NM_001098173.1 | 840 | 770 | 70 |
| 653-PTdeep | ZZEF1 | 76 | hg19 | chr17 | 4045957 | 4045957 | Missense_Mutation | SNP | C | C | A | novel | Somatic | G78V | 0.116547 | NM_015113.4 | 694 | 613 | 81 |
| 653-PTdeep | NLRP1 | 626 | hg19 | chr17 | 5462779 | 5462779 | Missense_Mutation | SNP | C | C | T | novel | Somatic | D413N | 0.11236 | NM_033004.4 | 801 | 711 | 90 |
| 653-PTdeep | PLSCR3 | 640 | hg19 | chr17 | 7296681 | 7296681 | Missense_Mutation | SNP | A | A | G | novel | Somatic | F97L | 0.142659 | NM_020360.4 | 722 | 619 | 103 |
| 653-PTdeep | TP53 | 642 | hg19 | chr17 | 7578413 | 7578413 | Missense_Mutation | SNP | C | C | A | novel | Somatic | V173L | 0.393322 | NM_000546.5 | 1227 | 744 | 483 |
| 653-PTdeep | CFAP52 | 657 | hg19 | chr17 | 9515797 | 9515797 | Splice_Site | SNP | G | G | C | novel | Somatic |  | 0.090439 | NM_145054.5 | 387 | 352 | 35 |
| 653-PTdeep | DNAH9 | 84 | hg19 | chr17 | 11511499 | 11511499 | Missense_Mutation | SNP | G | G | C | novel | Somatic | M157I | 0.110368 | NM_001372.4 | 598 | 532 | 66 |
| 653-PTdeep | DNAH9 | 84 | hg19 | chr17 | 11593754 | 11593754 | Splice_Site | SNP | G | G | A | novel | Somatic |  | 0.064516 | NM_001372.4 | 464 | 434 | 30 |
| 653-PTdeep | KCNJ12 | 747 | hg19 | chr17 | 21319451 | 21319451 | Missense_Mutation | SNP | C | C | T | novel | Somatic | S266L | 0.059969 | NM_021012.5 | 3849 | 3618 | 231 |
| 653-PTdeep | TTC25 | 111 | hg19 | chr17 | 40094807 | 40094807 | Splice_Region | SNP | C | C | A | novel | Somatic |  | 0.137313 | NM_031421.5 | 669 | 577 | 92 |
| 653-PTdeep | UBTF | 907 | hg19 | chr17 | 42284701 | 42284701 | In_Frame_Del | DEL | TCGTCG | TCGTCG | - | novel | Somatic | D733_D734del | 0.076712 | NM_014233.4 | 730 | 674 | 56 |
| 653-PTdeep | KPNA2 | 1088 | hg19 | chr17 | 66040561 | 66040561 | Missense_Mutation | SNP | C | C | G | novel | Somatic | T430S | 0.090361 | NM_002266.4 | 498 | 453 | 45 |
| 653-PTdeep | ACOX1 | 1149 | hg19 | chr17 | 73947542 | 73947542 | Missense_Mutation | SNP | C | C | T | novel | Somatic | R354Q | 0.313725 | NM_004035.7 | 611 | 419 | 192 |
| 653-PTdeep | TCF3 | 597 | hg19 | chr19 | 1611735 | 1611735 | Missense_Mutation | SNP | C | C | T | novel | Somatic | E646K | 0.135314 | NM_003200.5 | 1211 | 1047 | 164 |
| 653-PTdeep | CLEC4M | 644 | hg19 | chr19 | 7831545 | 7831545 | Missense_Mutation | SNP | G | G | A | novel | Somatic | R263H | 0.091904 | NM_014257.5 | 910 | 826 | 84 |
| 653-PTdeep | SLC44A2 | 83 | hg19 | chr19 | 10736941 | 10736941 | Missense_Mutation | SNP | A | A | G | novel | Somatic | K17R | 0.05492 | NM_001363611.2 | 437 | 413 | 24 |
| 653-PTdeep | RGL3 | 672 | hg19 | chr19 | 11526646 | 11526646 | Missense_Mutation | SNP | C | C | G | novel | Somatic | E202Q | 0.215886 | NM_001161616.3 | 982 | 770 | 212 |
| 653-PTdeep | ECSIT | 673 | hg19 | chr19 | 11618361 | 11618361 | Missense_Mutation | SNP | G | G | A | novel | Somatic | P321L | 0.120902 | NM_016581.5 | 488 | 429 | 59 |
| 653-PTdeep | ADGRL1 | 86 | hg19 | chr19 | 14273838 | 14273838 | Missense_Mutation | SNP | C | C | T | novel | Somatic | G264R | 0.123345 | NM_001008701.3 | 1433 | 1256 | 177 |
| 653-PTdeep | ADGRE2 | 698 | hg19 | chr19 | 14863254 | 14863254 | Missense_Mutation | SNP | G | G | T | novel | Somatic | L559I | 0.022929 | NM_013447.4 | 1351 | 1320 | 31 |
| 653-PTdeep | BRD4 | 702 | hg19 | chr19 | 15367789 | 15367789 | Missense_Mutation | SNP | C | C | T | novel | Somatic | E513K | 0.120283 | NM_058243.2 | 848 | 746 | 102 |
| 653-PTdeep | CYP4F2 | 88 | hg19 | chr19 | 16003384 | 16003384 | Missense_Mutation | SNP | C | C | T | novel | Somatic | A118T | 0.099203 | NM_001082.5 | 1127 | 1015 | 112 |
| 653-PTdeep | CALR3 | 711 | hg19 | chr19 | 16589968 | 16589968 | Nonsense_Mutation | SNP | G | G | A | novel | Somatic | Q377Ter | 0.148905 | NM_145046.4 | 684 | 582 | 102 |
| 653-PTdeep | NWD1 | 89 | hg19 | chr19 | 16842022 | 16842022 | Missense_Mutation | SNP | A | A | G | novel | Somatic | K5R | 0.132075 | NM_001007525.5 | 477 | 414 | 63 |
| 653-PTdeep | ANKRD27 | 104 | hg19 | chr19 | 33135312 | 33135312 | Missense_Mutation | SNP | G | G | T | novel | Somatic | H148Q | 0.083877 | NM_032139.3 | 1072 | 982 | 90 |
| 653-PTdeep | HAUS5 | 860 | hg19 | chr19 | 36113830 | 36113830 | Missense_Mutation | SNP | T | T | A | novel | Somatic | S613T | 0.108451 | NM_015302.2 | 710 | 633 | 77 |
| 653-PTdeep | RYR1 | 110 | hg19 | chr19 | 38964291 | 38964291 | Missense_Mutation | SNP | G | G | T | novel | Somatic | G1347V | 0.131126 | NM_000540.3 | 1508 | 1310 | 198 |
| 653-PTdeep | LTBP4 | 898 | hg19 | chr19 | 41114264 | 41114264 | Missense_Mutation | SNP | G | G | A | novel | Somatic | R499Q | 0.117219 | NM_001042544.1 | 1236 | 1091 | 145 |
| 653-PTdeep | KCNA7 | 963 | hg19 | chr19 | 49574036 | 49574036 | Missense_Mutation | SNP | A | A | G | novel | Somatic | W219R | 0.107666 | NM_031886.2 | 1161 | 1036 | 125 |
| 653-PTdeep | KLK6 | 977 | hg19 | chr19 | 51465038 | 51465038 | In_Frame_Del | DEL | ACA | ACA | - | novel | Somatic | L181del | 0.095455 | NM_002774.4 | 440 | 398 | 42 |
| 653-PTdeep | NLRP9 | 126 | hg19 | chr19 | 56244334 | 56244334 | Missense_Mutation | SNP | C | C | T | novel | Somatic | R288Q | 0.086022 | NM_176820.4 | 465 | 425 | 40 |
| 653-PTdeep | ZNF582 | 1019 | hg19 | chr19 | 56895719 | 56895719 | Missense_Mutation | SNP | C | C | T | novel | Somatic | R387Q | 0.102888 | NM_001320371.2 | 553 | 496 | 57 |
| 653-PTdeep | TRIM28 | 1035 | hg19 | chr19 | 59060457 | 59060457 | Silent | SNP | C | C | T | novel | Somatic | P504= | 0.113225 | NM_005762.3 | 1051 | 932 | 119 |
| 653-PTdeep | TMX4 | 80 | hg19 | chr20 | 7963005 | 7963005 | Missense_Mutation | SNP | C | C | G | novel | Somatic | E315Q | 0.142602 | NM_021156.4 | 559 | 479 | 80 |
| 653-PTdeep | SPTLC3 | 85 | hg19 | chr20 | 13074168 | 13074168 | Missense_Mutation | SNP | C | C | T | novel | Somatic | S257L | 0.162679 | NM_018327.4 | 418 | 350 | 68 |
| 653-PTdeep | ACTR5 | 870 | hg19 | chr20 | 37384617 | 37384617 | Missense_Mutation | SNP | G | G | A | novel | Somatic | A371T | 0.170996 | NM_024855.4 | 924 | 766 | 158 |
| 653-PTdeep | ZNF334 | 929 | hg19 | chr20 | 45130480 | 45130480 | Missense_Mutation | SNP | C | C | A | novel | Somatic | V523L | 0.189015 | NM_001353825.1 | 619 | 502 | 117 |
| 653-PTdeep | ATP9A | 1 | hg19 | chr20 | 50255904 | 50255904 | Missense_Mutation | SNP | T | T | G | novel | Somatic | K549T | 0.086384 | NM_006045.3 | 683 | 624 | 59 |
| 653-PTdeep | SYCP2 | 128 | hg19 | chr20 | 58467047 | 58467047 | Frame_Shift_Del | DEL | T | T | - | novel | Somatic | M788Ter | 0.029412 | NM_014258.4 | 508 | 493 | 15 |
| 653-PTdeep | CDH26 | 128 | hg19 | chr20 | 58574656 | 58574656 | Missense_Mutation | SNP | G | G | A | novel | Somatic | E679K | 0.185374 | NM_177980.4 | 587 | 478 | 109 |
| 653-PTdeep | NCAM2 | 94 | hg19 | chr21 | 22782690 | 22782690 | Nonsense_Mutation | SNP | C | C | G | novel | Somatic | S431Ter | 0.109375 | NM_004540.5 | 448 | 399 | 49 |
| 653-PTdeep | URB1 | 105 | hg19 | chr21 | 33706558 | 33706558 | Missense_Mutation | SNP | C | C | A | novel | Somatic | G1591W | 0.138889 | NM_014825.3 | 864 | 744 | 120 |
| 653-PTdeep | TRAPPC10 | 932 | hg19 | chr21 | 45522859 | 45522859 | Splice_Region | SNP | G | G | C | novel | Somatic |  | 0.03263 | NM_003274.5 | 1041 | 1007 | 34 |
| 653-PTdeep | KRTAP10-9 | 936 | hg19 | chr21 | 46047486 | 46047486 | Missense_Mutation | SNP | C | C | T | novel | Somatic | S133F | 0.133125 | NM_198690.3 | 1276 | 1106 | 170 |
| 653-PTdeep | ZNRF3 | 12 | hg19 | chr22 | 29446070 | 29446070 | Missense_Mutation | SNP | C | C | G | novel | Somatic | P634R | 0.139142 | NM_001206998.2 | 768 | 661 | 107 |
| 653-PTdeep | CACNA1I | 111 | hg19 | chr22 | 40075295 | 40075295 | Missense_Mutation | SNP | G | G | T | novel | Somatic | D1747Y | 0.131436 | NM_021096.4 | 737 | 640 | 97 |
| 653-PTdeep | MKL1 | 896 | hg19 | chr22 | 40813025 | 40813025 | Missense_Mutation | SNP | A | A | G | novel | Somatic | M848T | 0.109785 | NM_020831.6 | 838 | 746 | 92 |
| 653-PTdeep | FBLN1 | 935 | hg19 | chr22 | 45929685 | 45929685 | Missense_Mutation | SNP | G | G | A | novel | Somatic | E231K | 0.256082 | NM_006486.3 | 780 | 580 | 200 |
| 653-PTdeep | IL17REL | 969 | hg19 | chr22 | 50439591 | 50439591 | Missense_Mutation | SNP | G | G | A | novel | Somatic | T67M | 0.125957 | NM_001371417.1 | 1174 | 1026 | 148 |
| 653-PTdeep | GK | 819 | hg19 | chrX | 30737535 | 30737535 | Splice_Site | SNP | G | G | C | novel | Somatic |  | 0.315789 | NM_001205019.1 | 114 | 78 | 36 |
| 653-PTdeep | GPRASP1 | 1362 | hg19 | chrX | 101910070 | 101910070 | Missense_Mutation | SNP | G | G | C | novel | Somatic | W410S | 0.289037 | NM_001184727.2 | 301 | 214 | 87 |
| 653-PTdeep | AMMECR1 | 177 | hg19 | chrX | 109561207 | 109561207 | In_Frame_Del | DEL | GGA | GGA | - | novel | Somatic | S31del | 0.024038 | NM_015365.3 | 416 | 406 | 10 |
| 653-LNmet | CFAP74 | 599 | hg19 | chr1 | 1857284 | 1857284 | Nonsense_Mutation | SNP | C | C | A | novel | Somatic | E1348Ter | 0.103189 | NM_001304360.2 | 532 | 477 | 55 |
| 653-LNmet | DNAJC16 | 88 | hg19 | chr1 | 15886055 | 15886055 | In_Frame_Del | DEL | TCA | TCA | - | novel | Somatic | I354del | 0.098 | NM_015291.4 | 500 | 451 | 49 |
| 653-LNmet | UQCRHL | 708 | hg19 | chr1 | 16133928 | 16133928 | Missense_Mutation | SNP | C | C | G | novel | Somatic | D73H | 0.111669 | NM_001089591.1 | 797 | 708 | 89 |
| 653-LNmet | KDF1 | 793 | hg19 | chr1 | 27278424 | 27278424 | Missense_Mutation | SNP | G | G | A | novel | Somatic | R150W | 0.16476 | NM_152365.3 | 437 | 365 | 72 |
| 653-LNmet | ZMYM1 | 856 | hg19 | chr1 | 35578657 | 35578657 | Missense_Mutation | SNP | C | C | T | novel | Somatic | S409L | 0.123457 | NM_024772.5 | 405 | 355 | 50 |
| 653-LNmet | AGO4 | 107 | hg19 | chr1 | 36291050 | 36291050 | Nonsense_Mutation | SNP | C | C | G | novel | Somatic | S148Ter | 0.118871 | NM_017629.4 | 673 | 593 | 80 |
| 653-LNmet | HIVEP3 | 113 | hg19 | chr1 | 42048097 | 42048097 | In_Frame_Del | DEL | CTC | CTC | - | novel | Somatic | R791del | 0.136471 | NM_024503.5 | 424 | 366 | 58 |
| 653-LNmet | FAM183A | 917 | hg19 | chr1 | 43621973 | 43621973 | Missense_Mutation | SNP | C | C | T | novel | Somatic | H132Y | 0.135667 | NM_001101376.3 | 457 | 395 | 62 |
| 653-LNmet | FAF1 | 15 | hg19 | chr1 | 51050417 | 51050417 | Missense_Mutation | SNP | C | C | G | novel | Somatic | G303R | 0.132316 | NM_007051.3 | 393 | 341 | 52 |
| 653-LNmet | NRDC | 122 | hg19 | chr1 | 52257726 | 52257726 | Missense_Mutation | SNP | C | C | G | novel | Somatic | Q1024H | 0.127737 | NM_002525.2 | 273 | 238 | 35 |
| 653-LNmet | WDR78 | 137 | hg19 | chr1 | 67299385 | 67299385 | Missense_Mutation | SNP | C | C | G | novel | Somatic | L632F | 0.149813 | NM_024763.5 | 265 | 225 | 40 |
| 653-LNmet | LRRC7 | 17 | hg19 | chr1 | 70144122 | 70144122 | Missense_Mutation | SNP | G | G | C | novel | Somatic | E21Q | 0.126253 | NM_001370785.1 | 499 | 436 | 63 |
| 653-LNmet | TGFBR3 | 2 | hg19 | chr1 | 92185448 | 92185448 | Splice_Site | SNP | A | A | G | novel | Somatic |  | 0.142857 | NM_003243.5 | 437 | 374 | 63 |
| 653-LNmet | SETDB1 | 1736 | hg19 | chr1 | 150915441 | 150915441 | Missense_Mutation | SNP | C | C | T | novel | Somatic | R263W | 0.193473 | NM_001366418.1 | 854 | 688 | 166 |
| 653-LNmet | INTS3 | 1757 | hg19 | chr1 | 153736631 | 153736631 | Missense_Mutation | SNP | C | C | T | novel | Somatic | S620F | 0.052716 | NM_023015.5 | 625 | 592 | 33 |
| 653-LNmet | AIM2 | 1798 | hg19 | chr1 | 159032487 | 159032487 | Missense_Mutation | SNP | T | T | G | novel | Somatic | T343P | 0.077579 | NM_004833.3 | 1139 | 1048 | 91 |
| 653-LNmet | CADM3 | 1799 | hg19 | chr1 | 159162470 | 159162470 | Nonsense_Mutation | SNP | C | C | G | novel | Somatic | S145Ter | 0.102653 | NM_021189.5 | 864 | 775 | 89 |
| 653-LNmet | GPR161 | 1867 | hg19 | chr1 | 168059870 | 168059870 | Missense_Mutation | SNP | G | G | T | novel | Somatic | A399E | 0.045521 | NM_001267609.1 | 681 | 650 | 31 |
| 653-LNmet | GLUL | 1976 | hg19 | chr1 | 182353613 | 182353613 | Missense_Mutation | SNP | G | G | A | novel | Somatic | S350L | 0.107327 | NM_002065.6 | 968 | 864 | 104 |
| 653-LNmet | BRINP3 | 254 | hg19 | chr1 | 190195215 | 190195215 | Missense_Mutation | SNP | A | A | C | novel | Somatic | S320A | 0.083779 | NM_199051.3 | 561 | 514 | 47 |
| 653-LNmet | REN | 2142 | hg19 | chr1 | 204125842 | 204125842 | Frame_Shift_Del | DEL | G | G | - | novel | Somatic | L261SfsTer11 | 0.121693 | NM_000537.4 | 566 | 497 | 69 |
| 653-LNmet | IL19 | 2164 | hg19 | chr1 | 206972315 | 206972315 | Missense_Mutation | SNP | G | G | A | novel | Somatic | V26M | 0.077372 | NM_153758.3 | 685 | 632 | 53 |
| 653-LNmet | OBSCN | 290 | hg19 | chr1 | 228482557 | 228482557 | Missense_Mutation | SNP | G | G | C | novel | Somatic | E4253D | 0.037872 | NM_001271223.2 | 1108 | 1066 | 42 |
| 653-LNmet | OBSCN | 290 | hg19 | chr1 | 228520919 | 228520919 | Missense_Mutation | SNP | G | G | C | novel | Somatic | E6208Q | 0.074906 | NM_001271223.2 | 267 | 247 | 20 |
| 653-LNmet | GNPAT | 2350 | hg19 | chr1 | 231396424 | 231396424 | Missense_Mutation | SNP | C | C | G | novel | Somatic | Q145E | 0.052209 | NM_014236.4 | 496 | 470 | 26 |
| 653-LNmet | TARBP1 | 2374 | hg19 | chr1 | 234563135 | 234563135 | Splice_Site | SNP | C | C | G | novel | Somatic |  | 0.268975 | NM_005646.4 | 751 | 549 | 202 |
| 653-LNmet | KIF26B | 38 | hg19 | chr1 | 245530393 | 245530393 | Missense_Mutation | SNP | G | G | T | novel | Somatic | Q241H | 0.026277 | NM_018012.4 | 685 | 667 | 18 |
| 653-LNmet | SMYD3 | 38 | hg19 | chr1 | 246021990 | 246021990 | Intron | SNP | T | T | G | novel | Somatic |  | 0.11465 | NM_001167740.2 | 471 | 417 | 54 |
| 653-LNmet | OR2B11 | 2474 | hg19 | chr1 | 247615143 | 247615143 | Missense_Mutation | SNP | C | C | T | novel | Somatic | A48T | 0.070848 | NM_001004492.1 | 860 | 799 | 61 |
| 653-LNmet | TDRD15 | 93 | hg19 | chr2 | 21363579 | 21363579 | Frame_Shift_Del | DEL | T | T | - | novel | Somatic | L1082CfsTer7 | 0.138743 | NM_001306137.1 | 381 | 328 | 53 |
| 653-LNmet | PRR30 | 793 | hg19 | chr2 | 27360083 | 27360083 | Missense_Mutation | SNP | C | C | T | novel | Somatic | R372Q | 0.15733 | NM_178553.4 | 838 | 706 | 132 |
| 653-LNmet | CAD | 794 | hg19 | chr2 | 27446870 | 27446870 | Missense_Mutation | SNP | A | A | G | novel | Somatic | T361A | 0.232432 | NM_004341.5 | 551 | 422 | 129 |
| 653-LNmet | FOSL2 | 803 | hg19 | chr2 | 28635247 | 28635247 | Missense_Mutation | SNP | C | C | T | novel | Somatic | R305C | 0.214286 | NM_005253.4 | 629 | 494 | 135 |
| 653-LNmet | FANCL | 128 | hg19 | chr2 | 58392870 | 58392870 | Missense_Mutation | SNP | C | C | T | novel | Somatic | R227K | 0.105978 | NM_018062.3 | 1471 | 1315 | 156 |
| 653-LNmet | VAX2 | 1127 | hg19 | chr2 | 71148399 | 71148399 | Missense_Mutation | SNP | A | A | G | novel | Somatic | N140S | 0.233161 | NM_012476.3 | 577 | 442 | 135 |
| 653-LNmet | SFXN5 | 142 | hg19 | chr2 | 73298797 | 73298797 | In_Frame_Del | DEL | GCC | GCC | - | novel | Somatic | A13del | 0.024226 | NM_144579.3 | 722 | 704 | 18 |
| 653-LNmet | REG1B | 1190 | hg19 | chr2 | 79313534 | 79313534 | Missense_Mutation | SNP | C | C | T | novel | Somatic | D94N | 0.115007 | NM_006507.4 | 712 | 630 | 82 |
| 653-LNmet | SH2D6 | 1238 | hg19 | chr2 | 85662870 | 85662870 | Missense_Mutation | SNP | G | G | A | novel | Somatic | V80I | 0.083244 | NM_201594.2 | 937 | 859 | 78 |
| 653-LNmet | KDM3A | 1246 | hg19 | chr2 | 86693679 | 86693679 | Missense_Mutation | SNP | G | G | A | novel | Somatic | D398N | 0.118866 | NM_018433.6 | 917 | 808 | 109 |
| 653-LNmet | TEKT4 | 1313 | hg19 | chr2 | 95541339 | 95541339 | Missense_Mutation | SNP | C | C | T | novel | Somatic | R315W | 0.087766 | NM_144705.4 | 376 | 343 | 33 |
| 653-LNmet | DPP10 | 22 | hg19 | chr2 | 116599880 | 116599880 | Missense_Mutation | SNP | G | G | A | novel | Somatic | E801K | 0.104294 | NM_001321905.2 | 326 | 292 | 34 |
| 653-LNmet | ARHGEF4 | 198 | hg19 | chr2 | 131672852 | 131672852 | Missense_Mutation | SNP | G | G | C | novel | Somatic | E445Q | 0.122047 | NM_001367493.1 | 508 | 446 | 62 |
| 653-LNmet | KCNJ3 | 1771 | hg19 | chr2 | 155711277 | 155711277 | Missense_Mutation | SNP | G | G | A | novel | Somatic | E320K | 0.081967 | NM_002239.4 | 488 | 448 | 40 |
| 653-LNmet | KLHL41 | 1884 | hg19 | chr2 | 170366628 | 170366628 | Missense_Mutation | SNP | C | C | G | novel | Somatic | Q114E | 0.25723 | NM_006063.3 | 657 | 488 | 169 |
| 653-LNmet | NFE2L2 | 242 | hg19 | chr2 | 178098960 | 178098960 | Missense_Mutation | SNP | C | C | G | novel | Somatic | D29H | 0.219092 | NM_006164.5 | 639 | 499 | 140 |
| 653-LNmet | TTN | 1955 | hg19 | chr2 | 179543185 | 179543185 | Missense_Mutation | SNP | A | A | G | novel | Somatic | V11289A | 0.078675 | NM_001267550.2 | 483 | 445 | 38 |
| 653-LNmet | TTN | 1955 | hg19 | chr2 | 179614010 | 179614010 | Missense_Mutation | SNP | G | G | T | novel | Somatic | H4373N | 0.215881 | NM_133379.5 | 403 | 316 | 87 |
| 653-LNmet | ZNF804A | 31 | hg19 | chr2 | 185801390 | 185801390 | Missense_Mutation | SNP | G | G | C | novel | Somatic | E423Q | 0.194561 | NM_194250.2 | 477 | 384 | 93 |
| 653-LNmet | ASDURF | 2038 | hg19 | chr2 | 190530150 | 190530150 | Nonsense_Mutation | SNP | C | C | T | novel | Somatic | R65Ter | 0.21558 | NM_001353493.1 | 552 | 433 | 119 |
| 653-LNmet | PGAP1 | 261 | hg19 | chr2 | 197710632 | 197710632 | Missense_Mutation | SNP | G | G | A | novel | Somatic | L754F | 0.093074 | NM_024989.4 | 461 | 418 | 43 |
| 653-LNmet | UNC80 | 34 | hg19 | chr2 | 210694004 | 210694004 | Nonsense_Mutation | SNP | C | C | T | novel | Somatic | R843Ter | 0.214431 | NM_001371986.1 | 982 | 771 | 211 |
| 653-LNmet | SLC19A3 | 2328 | hg19 | chr2 | 228563751 | 228563751 | Missense_Mutation | SNP | C | C | G | novel | Somatic | G227A | 0.092405 | NM_025243.4 | 790 | 717 | 73 |
| 653-LNmet | COL6A3 | 300 | hg19 | chr2 | 238285810 | 238285810 | Missense_Mutation | SNP | T | T | C | novel | Somatic | E892G | 0.079762 | NM_004369.4 | 840 | 773 | 67 |
| 653-LNmet | ANO7 | 2432 | hg19 | chr2 | 242142824 | 242142824 | Missense_Mutation | SNP | C | C | T | novel | Somatic | A267V | 0.367036 | NM_001370694.2 | 721 | 456 | 265 |
| 653-LNmet | HDLBP | 2432 | hg19 | chr2 | 242194520 | 242194520 | Missense_Mutation | SNP | G | G | T | novel | Somatic | F378L | 0.078534 | NM_005336.5 | 955 | 880 | 75 |
| 653-LNmet | CAV3 | 81 | hg19 | chr3 | 8787330 | 8787330 | Missense_Mutation | SNP | C | C | T | novel | Somatic | T78M | 0.140127 | NM_033337.3 | 471 | 405 | 66 |
| 653-LNmet | CAND2 | 85 | hg19 | chr3 | 12856837 | 12856837 | Missense_Mutation | SNP | G | G | A | novel | Somatic | V402M | 0.143426 | NM_001162499.2 | 753 | 645 | 108 |
| 653-LNmet | ZNF385D | 93 | hg19 | chr3 | 21467033 | 21467033 | Missense_Mutation | SNP | T | T | A | novel | Somatic | H268L | 0.024142 | NM_024697.3 | 787 | 768 | 19 |
| 653-LNmet | CMTM6 | 833 | hg19 | chr3 | 32525525 | 32525525 | Missense_Mutation | SNP | C | C | T | novel | Somatic | R160Q | 0.090511 | NM_017801.3 | 684 | 622 | 62 |
| 653-LNmet | USP19 | 119 | hg19 | chr3 | 49146570 | 49146570 | Missense_Mutation | SNP | C | C | T | novel | Somatic | E1362K | 0.252273 | NM_001351100.1 | 440 | 329 | 111 |
| 653-LNmet | PBRM1 | 123 | hg19 | chr3 | 52678784 | 52678784 | Frame_Shift_Del | DEL | T | T | - | novel | Somatic | I279YfsTer4 | 0.259091 | NM_018313.5 | 659 | 488 | 171 |
| 653-LNmet | GLT8D1 | 987 | hg19 | chr3 | 52730659 | 52730659 | Missense_Mutation | SNP | C | C | G | novel | Somatic | D116H | 0.098765 | NM_001010983.2 | 405 | 365 | 40 |
| 653-LNmet | ADAMTS9 | 134 | hg19 | chr3 | 64580050 | 64580050 | Missense_Mutation | SNP | C | C | T | novel | Somatic | G1414S | 0.137552 | NM_182920.2 | 727 | 627 | 100 |
| 653-LNmet | PDZRN3 | 1145 | hg19 | chr3 | 73433781 | 73433781 | Missense_Mutation | SNP | A | A | G | novel | Somatic | F646L | 0.116635 | NM_015009.3 | 523 | 462 | 61 |
| 653-LNmet | CNTN3 | 17 | hg19 | chr3 | 74418484 | 74418484 | Missense_Mutation | SNP | G | G | T | novel | Somatic | P268T | 0.101031 | NM_020872.2 | 483 | 434 | 49 |
| 653-LNmet | IMPG2 | 1355 | hg19 | chr3 | 100976579 | 100976579 | Missense_Mutation | SNP | T | T | C | novel | Somatic | E316G | 0.077307 | NM_016247.4 | 800 | 738 | 62 |
| 653-LNmet | SENP7 | 169 | hg19 | chr3 | 101056452 | 101056452 | Nonsense_Mutation | SNP | G | G | C | novel | Somatic | S794Ter | 0.082569 | NM_020654.5 | 545 | 500 | 45 |
| 653-LNmet | ARHGAP31 | 186 | hg19 | chr3 | 119133391 | 119133391 | Nonsense_Mutation | SNP | C | C | G | novel | Somatic | S872Ter | 0.082777 | NM_020754.4 | 749 | 687 | 62 |
| 653-LNmet | GTF2E1 | 1504 | hg19 | chr3 | 120499913 | 120499913 | Missense_Mutation | SNP | C | C | A | novel | Somatic | Q306K | 0.080226 | NM_005513.3 | 885 | 814 | 71 |
| 653-LNmet | UMPS | 1534 | hg19 | chr3 | 124456637 | 124456637 | Missense_Mutation | SNP | A | A | C | novel | Somatic | K178T | 0.092117 | NM_000373.4 | 1129 | 1025 | 104 |
| 653-LNmet | ZNF148 | 192 | hg19 | chr3 | 124952521 | 124952521 | Missense_Mutation | SNP | G | G | A | novel | Somatic | S350F | 0.098765 | NM_021964.2 | 646 | 582 | 64 |
| 653-LNmet | PLXND1 | 1571 | hg19 | chr3 | 129278617 | 129278617 | Missense_Mutation | SNP | G | G | T | novel | Somatic | Q1715K | 0.091062 | NM_015103.2 | 593 | 539 | 54 |
| 653-LNmet | SRPRB | 1603 | hg19 | chr3 | 133538425 | 133538425 | Missense_Mutation | SNP | G | G | A | novel | Somatic | A211T | 0.081439 | NM_021203.4 | 1054 | 968 | 86 |
| 653-LNmet | FOXL2 | 1642 | hg19 | chr3 | 138664910 | 138664910 | Nonsense_Mutation | SNP | G | G | A | novel | Somatic | Q219Ter | 0.13079 | NM_023067.4 | 366 | 318 | 48 |
| 653-LNmet | ZBTB38 | 207 | hg19 | chr3 | 141161861 | 141161861 | Missense_Mutation | SNP | G | G | A | novel | Somatic | E211K | 0.073344 | NM_001080412.3 | 1268 | 1175 | 93 |
| 653-LNmet | MBNL1 | 27 | hg19 | chr3 | 152163077 | 152163077 | Nonsense_Mutation | SNP | C | C | T | novel | Somatic | R186Ter | 0.060311 | NM_207293.1 | 513 | 482 | 31 |
| 653-LNmet | DHX36 | 219 | hg19 | chr3 | 154032851 | 154032851 | Missense_Mutation | SNP | C | C | T | novel | Somatic | R196Q | 0.095855 | NM_020865.3 | 386 | 349 | 37 |
| 653-LNmet | PLCH1 | 221 | hg19 | chr3 | 155198804 | 155198804 | Missense_Mutation | SNP | G | G | A | novel | Somatic | P1671S | 0.154762 | NM_014996.3 | 671 | 567 | 104 |
| 653-LNmet | SHOX2 | 1789 | hg19 | chr3 | 157820625 | 157820625 | Missense_Mutation | SNP | C | C | T | novel | Somatic | E157K | 0.079534 | NM_003030.4 | 1030 | 948 | 82 |
| 653-LNmet | BCHE | 230 | hg19 | chr3 | 165548026 | 165548026 | Missense_Mutation | SNP | C | C | T | novel | Somatic | E266K | 0.11809 | NM_000055.4 | 796 | 702 | 94 |
| 653-LNmet | ST6GAL1 | 251 | hg19 | chr3 | 186760638 | 186760638 | Missense_Mutation | SNP | G | G | C | novel | Somatic | K49N | 0.048421 | NM_173216.2 | 950 | 904 | 46 |
| 653-LNmet | MUC4 | 2076 | hg19 | chr3 | 195508207 | 195508207 | Missense_Mutation | SNP | G | G | T | novel | Somatic | S3415Y | 0.038871 | NM_018406.7 | 4597 | 4418 | 179 |
| 653-LNmet | HGFAC | 611 | hg19 | chr4 | 3444583 | 3444583 | Missense_Mutation | SNP | G | G | T | novel | Somatic | G81V | 0.23822 | NM_001297439.2 | 382 | 291 | 91 |
| 653-LNmet | ADAMTS3 | 17 | hg19 | chr4 | 73186461 | 73186461 | Missense_Mutation | SNP | T | T | A | novel | Somatic | R358W | 0.129032 | NM_014243.3 | 806 | 702 | 104 |
| 653-LNmet | BMPR1B | 164 | hg19 | chr4 | 96070017 | 96070017 | Missense_Mutation | SNP | G | G | A | novel | Somatic | D399N | 0.130933 | NM_001203.3 | 611 | 531 | 80 |
| 653-LNmet | ALPK1 | 22 | hg19 | chr4 | 113298937 | 113298937 | Missense_Mutation | SNP | A | A | C | novel | Somatic | N2H | 0.308668 | NM_025144.4 | 472 | 326 | 146 |
| 653-LNmet | DCHS2 | 221 | hg19 | chr4 | 155412414 | 155412414 | Missense_Mutation | SNP | G | G | T | novel | Somatic | P32T | 0.181435 | NM_001358235.2 | 473 | 387 | 86 |
| 653-LNmet | TENM3 | 30 | hg19 | chr4 | 183594211 | 183594211 | Missense_Mutation | SNP | G | G | C | novel | Somatic | D389H | 0.131274 | NM_001080477.4 | 518 | 450 | 68 |
| 653-LNmet | TENM3 | 30 | hg19 | chr4 | 183674714 | 183674714 | Missense_Mutation | SNP | C | C | T | novel | Somatic | S1325L | 0.089317 | NM_001080477.4 | 571 | 520 | 51 |
| 653-LNmet | SLC6A3 | 74 | hg19 | chr5 | 1401052 | 1401052 | Missense_Mutation | SNP | C | C | T | novel | Somatic | R606K | 0.096774 | NM_001044.5 | 402 | 363 | 39 |
| 653-LNmet | ADAMTS16 | 9 | hg19 | chr5 | 5146365 | 5146365 | Missense_Mutation | SNP | C | C | T | novel | Somatic | R100W | 0.080592 | NM_139056.4 | 607 | 558 | 49 |
| 653-LNmet | PAPD7 | 636 | hg19 | chr5 | 6714482 | 6714482 | Missense_Mutation | SNP | C | C | T | novel | Somatic | S129L | 0.112613 | NM_006999.6 | 222 | 197 | 25 |
| 653-LNmet | PARP8 | 120 | hg19 | chr5 | 50056123 | 50056123 | Splice_Region | SNP | C | C | T | novel | Somatic |  | 0.030093 | NM_024615.4 | 430 | 417 | 13 |
| 653-LNmet | FLT4 | 1958 | hg19 | chr5 | 180039637 | 180039637 | Intron | SNP | C | C | G | novel | Somatic |  | 0.156627 | NM_182925.5 | 166 | 140 | 26 |
| 653-LNmet | HIST1H2AC | 784 | hg19 | chr6 | 26124853 | 26124853 | Nonstop_Mutation | SNP | A | A | G | novel | Somatic | Ter131WextTer? | 0.079886 | NM_003512.3 | 701 | 645 | 56 |
| 653-LNmet | CUL9 | 914 | hg19 | chr6 | 43190294 | 43190294 | Missense_Mutation | SNP | G | G | A | novel | Somatic | R2316Q | 0.186047 | NM_015089.4 | 558 | 454 | 104 |
| 653-LNmet | COL19A1 | 140 | hg19 | chr6 | 70778351 | 70778351 | Missense_Mutation | SNP | G | G | A | novel | Somatic | G403R | 0.05698 | NM_001858.6 | 702 | 662 | 40 |
| 653-LNmet | CYB5R4 | 1230 | hg19 | chr6 | 84618732 | 84618732 | Missense_Mutation | SNP | A | A | T | novel | Somatic | H112L | 0.091153 | NM_016230.4 | 373 | 339 | 34 |
| 653-LNmet | UFL1 | 165 | hg19 | chr6 | 97001218 | 97001218 | Missense_Mutation | SNP | C | C | G | novel | Somatic | Q742E | 0.078261 | NM_015323.5 | 345 | 318 | 27 |
| 653-LNmet | MICAL1 | 1422 | hg19 | chr6 | 109765519 | 109765519 | Missense_Mutation | SNP | G | G | A | novel | Somatic | R1027W | 0.067602 | NM_022765.4 | 783 | 730 | 53 |
| 653-LNmet | WISP3 | 1442 | hg19 | chr6 | 112389566 | 112389566 | Missense_Mutation | SNP | C | C | A | novel | Somatic | Q250K | 0.108844 | NM_198239.2 | 294 | 262 | 32 |
| 653-LNmet | RNF217 | 192 | hg19 | chr6 | 125283935 | 125283935 | Missense_Mutation | SNP | G | G | T | novel | Somatic | R82L | 0.059055 | NM_001286398.2 | 508 | 478 | 30 |
| 653-LNmet | PTPRK | 195 | hg19 | chr6 | 128540093 | 128540093 | Missense_Mutation | SNP | G | G | A | novel | Somatic | S281F | 0.189716 | NM_001291981.2 | 563 | 456 | 107 |
| 653-LNmet | ADGB | 213 | hg19 | chr6 | 147045448 | 147045448 | Missense_Mutation | SNP | T | T | C | novel | Somatic | V741A | 0.104628 | NM_024694.4 | 497 | 445 | 52 |
| 653-LNmet | TULP4 | 224 | hg19 | chr6 | 158900784 | 158900784 | Missense_Mutation | SNP | G | G | T | novel | Somatic | R343L | 0.056818 | NM_020245.5 | 439 | 414 | 25 |
| 653-LNmet | RPS6KA2 | 232 | hg19 | chr6 | 166827389 | 166827389 | Missense_Mutation | SNP | G | G | A | novel | Somatic | P657S | 0.102467 | NM_021135.6 | 527 | 473 | 54 |
| 653-LNmet | GPR31 | 1863 | hg19 | chr6 | 167570570 | 167570570 | Missense_Mutation | SNP | A | A | C | novel | Somatic | N250K | 0.097222 | NM_005299.2 | 576 | 520 | 56 |
| 653-LNmet | PHF14 | 83 | hg19 | chr7 | 11101655 | 11101655 | Frame_Shift_Ins | INS | - | - | T | novel | Somatic | L867FfsTer4 | 0.095588 | NM_001007157.2 | 272 | 246 | 26 |
| 653-LNmet | HOXA6 | 792 | hg19 | chr7 | 27186940 | 27186940 | Missense_Mutation | SNP | C | C | T | novel | Somatic | M143I | 0.120582 | NM_024014.4 | 481 | 423 | 58 |
| 653-LNmet | NOD1 | 817 | hg19 | chr7 | 30491628 | 30491628 | Missense_Mutation | SNP | G | G | A | novel | Somatic | R469W | 0.078859 | NM_006092.4 | 596 | 549 | 47 |
| 653-LNmet | STARD3NL | 876 | hg19 | chr7 | 38247164 | 38247164 | Missense_Mutation | SNP | C | C | A | novel | Somatic | A20D | 0.071508 | NM_001363339.2 | 895 | 831 | 64 |
| 653-LNmet | MYO1G | 928 | hg19 | chr7 | 45009309 | 45009309 | Missense_Mutation | SNP | G | G | A | novel | Somatic | R500C | 0.104911 | NM_033054.3 | 448 | 401 | 47 |
| 653-LNmet | HUS1 | 951 | hg19 | chr7 | 48019035 | 48019035 | Intron | SNP | C | C | G | novel | Somatic |  | 0.121662 | NM_004507.4 | 337 | 296 | 41 |
| 653-LNmet | ZPBP | 120 | hg19 | chr7 | 50023060 | 50023060 | Missense_Mutation | SNP | C | C | T | novel | Somatic | R280H | 0.107872 | NM_007009.3 | 341 | 304 | 37 |
| 653-LNmet | ZPBP | 120 | hg19 | chr7 | 50057869 | 50057869 | Missense_Mutation | SNP | G | G | C | novel | Somatic | D250E | 0.109875 | NM_007009.3 | 717 | 638 | 79 |
| 653-LNmet | ZNF713 | 126 | hg19 | chr7 | 56006801 | 56006801 | Missense_Mutation | SNP | G | G | A | novel | Somatic | G145E | 0.10219 | NM_182633.3 | 410 | 368 | 42 |
| 653-LNmet | CCDC146 | 146 | hg19 | chr7 | 76912079 | 76912079 | Missense_Mutation | SNP | G | G | A | novel | Somatic | D709N | 0.165301 | NM_020879.3 | 731 | 610 | 121 |
| 653-LNmet | ASB4 | 1310 | hg19 | chr7 | 95157450 | 95157450 | Missense_Mutation | SNP | G | G | T | novel | Somatic | M271I | 0.196916 | NM_016116.3 | 842 | 676 | 166 |
| 653-LNmet | LMTK2 | 166 | hg19 | chr7 | 97823708 | 97823708 | Missense_Mutation | SNP | G | G | A | novel | Somatic | E1311K | 0.074244 | NM_014916.4 | 1090 | 1009 | 81 |
| 653-LNmet | PIK3CG | 1397 | hg19 | chr7 | 106524631 | 106524631 | Missense_Mutation | SNP | C | C | G | novel | Somatic | S931C | 0.30559 | NM_002649.3 | 803 | 557 | 246 |
| 653-LNmet | GRM8 | 24 | hg19 | chr7 | 126086298 | 126086298 | Missense_Mutation | SNP | C | C | G | novel | Somatic | K853N | 0.106227 | NM_000845.3 | 817 | 730 | 87 |
| 653-LNmet | KMT2C | 27 | hg19 | chr7 | 151874148 | 151874148 | Frame_Shift_Del | DEL | T | T | - | novel | Somatic | K2797RfsTer26 | 0.042654 | NM_170606.3 | 417 | 399 | 18 |
| 653-LNmet | MCPH1 | 9 | hg19 | chr8 | 6289099 | 6289099 | Frame_Shift_Del | DEL | A | A | - | novel | Somatic | K107NfsTer39 | 0.032454 | NM_024596.5 | 493 | 477 | 16 |
| 653-LNmet | XKR6 | 83 | hg19 | chr8 | 10755630 | 10755630 | Missense_Mutation | SNP | C | C | A | novel | Somatic | K586N | 0.17773 | NM_173683.4 | 467 | 384 | 83 |
| 653-LNmet | MTMR9 | 670 | hg19 | chr8 | 11180205 | 11180205 | Missense_Mutation | SNP | G | G | A | novel | Somatic | E520K | 0.040856 | NM_015458.4 | 514 | 493 | 21 |
| 653-LNmet | RP1 | 1008 | hg19 | chr8 | 55533617 | 55533617 | Missense_Mutation | SNP | C | C | T | novel | Somatic | H31Y | 0.051833 | NM_006269.2 | 791 | 750 | 41 |
| 653-LNmet | C8orf89 | 1150 | hg19 | chr8 | 74169224 | 74169224 | Missense_Mutation | SNP | C | C | T | novel | Somatic | E89K | 0.103825 | NM_001243237.1 | 366 | 328 | 38 |
| 653-LNmet | PSKH2 | 1249 | hg19 | chr8 | 87060862 | 87060862 | Missense_Mutation | SNP | C | C | T | novel | Somatic | M329I | 0.065187 | NM_033126.3 | 721 | 674 | 47 |
| 653-LNmet | CNGB3 | 156 | hg19 | chr8 | 87683211 | 87683211 | Missense_Mutation | SNP | C | C | G | novel | Somatic | E152Q | 0.259082 | NM_019098.4 | 1045 | 774 | 271 |
| 653-LNmet | RBM12B | 1307 | hg19 | chr8 | 94747816 | 94747816 | Frame_Shift_Del | DEL | T | T | - | novel | Somatic | R275EfsTer14 | 0.071834 | NM_203390.3 | 529 | 491 | 38 |
| 653-LNmet | ESRP1 | 164 | hg19 | chr8 | 95674530 | 95674530 | Missense_Mutation | SNP | C | C | T | novel | Somatic | S174F | 0.056637 | NM_017697.4 | 565 | 533 | 32 |
| 653-LNmet | PKHD1L1 | 178 | hg19 | chr8 | 110412510 | 110412510 | Missense_Mutation | SNP | C | C | G | novel | Somatic | F406L | 0.075391 | NM_177531.6 | 701 | 648 | 53 |
| 653-LNmet | CSMD3 | 22 | hg19 | chr8 | 113484904 | 113484904 | Missense_Mutation | SNP | C | C | T | novel | Somatic | G1771S | 0.055696 | NM_198123.2 | 394 | 372 | 22 |
| 653-LNmet | SNTB1 | 23 | hg19 | chr8 | 121706103 | 121706103 | Missense_Mutation | SNP | G | G | A | novel | Somatic | P206L | 0.083628 | NM_021021.4 | 849 | 778 | 71 |
| 653-LNmet | EFR3A | 1599 | hg19 | chr8 | 132966087 | 132966087 | Missense_Mutation | SNP | G | G | A | novel | Somatic | G171S | 0.092537 | NM_015137.6 | 670 | 608 | 62 |
| 653-LNmet | PLEC | 1691 | hg19 | chr8 | 144999083 | 144999083 | Missense_Mutation | SNP | C | C | T | novel | Somatic | E1809K | 0.120805 | NM_201380.4 | 595 | 523 | 72 |
| 653-LNmet | WDR97 | 1692 | hg19 | chr8 | 145169477 | 145169477 | Missense_Mutation | SNP | C | C | A | novel | Somatic | F1271L | 0.131524 | NM_001316309.2 | 478 | 415 | 63 |
| 653-LNmet | MPDZ | 85 | hg19 | chr9 | 13188782 | 13188782 | Splice_Site | SNP | C | C | T | novel | Somatic |  | 0.244499 | NM_001330637.1 | 409 | 309 | 100 |
| 653-LNmet | FOCAD | 11 | hg19 | chr9 | 20717846 | 20717846 | Missense_Mutation | SNP | G | G | C | novel | Somatic | K37N | 0.102479 | NM_017794.4 | 605 | 543 | 62 |
| 653-LNmet | LINGO2 | 12 | hg19 | chr9 | 27950117 | 27950117 | Missense_Mutation | SNP | C | C | T | novel | Somatic | E185K | 0.090153 | NM_001258282.3 | 721 | 656 | 65 |
| 653-LNmet | OR13J1 | 858 | hg19 | chr9 | 35869813 | 35869813 | Missense_Mutation | SNP | C | C | T | novel | Somatic | E196K | 0.137331 | NM_001004487.1 | 517 | 446 | 71 |
| 653-LNmet | TJP2 | 141 | hg19 | chr9 | 71836042 | 71836042 | In_Frame_Del | DEL | CCGGAGCCGTGG | CCGGAGCCGTGG | - | novel | Somatic | R197_S200del | 0.081132 | NM_004817.4 | 529 | 486 | 43 |
| 653-LNmet | TRPM3 | 17 | hg19 | chr9 | 73255521 | 73255521 | Silent | SNP | G | G | T | novel | Somatic | V492= | 0.098039 | NM_001366147.2 | 611 | 551 | 60 |
| 653-LNmet | PCSK5 | 18 | hg19 | chr9 | 78686768 | 78686768 | Missense_Mutation | SNP | G | G | A | novel | Somatic | G283E | 0.091248 | NM_001372043.1 | 1072 | 974 | 98 |
| 653-LNmet | ROR2 | 163 | hg19 | chr9 | 94487206 | 94487206 | Missense_Mutation | SNP | C | C | T | novel | Somatic | E524K | 0.110169 | NM_004560.4 | 590 | 525 | 65 |
| 653-LNmet | FBP1 | 165 | hg19 | chr9 | 97369228 | 97369228 | Missense_Mutation | SNP | C | C | T | novel | Somatic | G192R | 0.116453 | NM_000507.4 | 936 | 827 | 109 |
| 653-LNmet | TEX10 | 1371 | hg19 | chr9 | 103082642 | 103082642 | Nonsense_Mutation | SNP | G | G | A | novel | Somatic | R703Ter | 0.063406 | NM_017746.4 | 552 | 517 | 35 |
| 653-LNmet | SVEP1 | 22 | hg19 | chr9 | 113217997 | 113217997 | Intron | SNP | C | C | T | novel | Somatic |  | 0.063325 | NM_153366.4 | 379 | 355 | 24 |
| 653-LNmet | C9orf84 | 1458 | hg19 | chr9 | 114467600 | 114467600 | Nonsense_Mutation | SNP | G | G | A | novel | Somatic | Q864Ter | 0.073661 | NM_173521.5 | 448 | 415 | 33 |
| 653-LNmet | TLR4 | 1504 | hg19 | chr9 | 120476729 | 120476729 | Nonsense_Mutation | SNP | G | G | T | novel | Somatic | E775Ter | 0.09113 | NM_138554.5 | 823 | 748 | 75 |
| 653-LNmet | BRINP1 | 189 | hg19 | chr9 | 122004335 | 122004335 | Missense_Mutation | SNP | C | C | G | novel | Somatic | G190A | 0.117296 | NM_014618.3 | 501 | 442 | 59 |
| 653-LNmet | OR1L3 | 1542 | hg19 | chr9 | 125437535 | 125437535 | Missense_Mutation | SNP | C | C | A | novel | Somatic | L43M | 0.215356 | NM_001005234.1 | 533 | 418 | 115 |
| 653-LNmet | SET | 1587 | hg19 | chr9 | 131456277 | 131456277 | Missense_Mutation | SNP | G | G | A | novel | Somatic | E269K | 0.084402 | NM_001122821.2 | 936 | 857 | 79 |
| 653-LNmet | SET | 1587 | hg19 | chr9 | 131456286 | 131456286 | Missense_Mutation | SNP | G | G | A | novel | Somatic | E272K | 0.08296 | NM_001122821.2 | 892 | 818 | 74 |
| 653-LNmet | AK8 | 202 | hg19 | chr9 | 135739089 | 135739089 | Missense_Mutation | SNP | G | G | A | novel | Somatic | T99M | 0.168016 | NM_152572.3 | 494 | 411 | 83 |
| 653-LNmet | COL5A1 | 204 | hg19 | chr9 | 137686952 | 137686952 | Missense_Mutation | SNP | C | C | T | novel | Somatic | R909W | 0.117783 | NM_001278074.1 | 433 | 382 | 51 |
| 653-LNmet | C10orf67 | 765 | hg19 | chr10 | 23592373 | 23592373 | Missense_Mutation | SNP | C | C | G | novel | Somatic | E189Q | 0.134276 | NM_001365862.2 | 283 | 245 | 38 |
| 653-LNmet | SVIL | 101 | hg19 | chr10 | 29822346 | 29822346 | Missense_Mutation | SNP | C | C | T | novel | Somatic | R317Q | 0.127923 | NM_021738.3 | 727 | 634 | 93 |
| 653-LNmet | WDFY4 | 120 | hg19 | chr10 | 49944069 | 49944069 | Missense_Mutation | SNP | G | G | C | novel | Somatic | C611S | 0.121986 | NM_020945.2 | 705 | 619 | 86 |
| 653-LNmet | LGI1 | 164 | hg19 | chr10 | 95557238 | 95557238 | Missense_Mutation | SNP | T | T | A | novel | Somatic | F451Y | 0.132827 | NM_005097.4 | 527 | 457 | 70 |
| 653-LNmet | BLNK | 1332 | hg19 | chr10 | 97976469 | 97976469 | Missense_Mutation | SNP | C | C | G | novel | Somatic | E183Q | 0.091803 | NM_013314.3 | 304 | 276 | 28 |
| 653-LNmet | ITPRIP | 1394 | hg19 | chr10 | 106075218 | 106075218 | Missense_Mutation | SNP | C | C | T | novel | Somatic | E198K | 0.097804 | NM_033397.3 | 501 | 452 | 49 |
| 653-LNmet | TAF10 | 635 | hg19 | chr11 | 6632922 | 6632922 | Missense_Mutation | SNP | C | C | G | novel | Somatic | L120F | 0.164009 | NM_006284.4 | 878 | 734 | 144 |
| 653-LNmet | EIF4G2 | 667 | hg19 | chr11 | 10822068 | 10822068 | Missense_Mutation | SNP | C | C | G | novel | Somatic | D591H | 0.105372 | NM_001172705.1 | 483 | 432 | 51 |
| 653-LNmet | SPTY2D1 | 727 | hg19 | chr11 | 18636621 | 18636621 | Missense_Mutation | SNP | C | C | G | novel | Somatic | Q400H | 0.123457 | NM_194285.3 | 729 | 639 | 90 |
| 653-LNmet | SPTY2D1 | 727 | hg19 | chr11 | 18636949 | 18636949 | Missense_Mutation | SNP | C | C | A | novel | Somatic | G291V | 0.120666 | NM_194285.3 | 721 | 634 | 87 |
| 653-LNmet | NAV2 | 92 | hg19 | chr11 | 20114706 | 20114706 | Missense_Mutation | SNP | G | G | C | novel | Somatic | K1959N | 0.225524 | NM_145117.5 | 572 | 443 | 129 |
| 653-LNmet | SLC43A3 | 1021 | hg19 | chr11 | 57175323 | 57175323 | Missense_Mutation | SNP | G | G | A | novel | Somatic | P486L | 0.1 | NM_001278206.2 | 360 | 324 | 36 |
| 653-LNmet | OR5B12 | 1029 | hg19 | chr11 | 58206931 | 58206931 | Missense_Mutation | SNP | G | G | A | novel | Somatic | R232C | 0.081019 | NM_001004733.2 | 431 | 396 | 35 |
| 653-LNmet | PITPNM1 | 1098 | hg19 | chr11 | 67269450 | 67269450 | Missense_Mutation | SNP | C | C | T | novel | Somatic | D175N | 0.1231 | NM_004910.3 | 658 | 577 | 81 |
| 653-LNmet | UVRAG | 145 | hg19 | chr11 | 75851867 | 75851867 | Nonsense_Mutation | SNP | C | C | T | novel | Somatic | R504Ter | 0.109489 | NM_003369.4 | 685 | 610 | 75 |
| 653-LNmet | TENM4 | 18 | hg19 | chr11 | 78399143 | 78399143 | Missense_Mutation | SNP | G | G | T | novel | Somatic | T1739N | 0.146245 | NM_001098816.3 | 253 | 216 | 37 |
| 653-LNmet | FAM181B | 151 | hg19 | chr11 | 82443699 | 82443699 | Missense_Mutation | SNP | G | G | A | novel | Somatic | A358V | 0.15011 | NM_175885.4 | 452 | 384 | 68 |
| 653-LNmet | DEUP1 | 1295 | hg19 | chr11 | 93122280 | 93122280 | Missense_Mutation | SNP | G | G | C | novel | Somatic | E344Q | 0.102459 | NM_181645.3 | 488 | 438 | 50 |
| 653-LNmet | PGR | 169 | hg19 | chr11 | 100999023 | 100999023 | Missense_Mutation | SNP | G | G | A | novel | Somatic | A260V | 0.125767 | NM_000926.4 | 325 | 284 | 41 |
| 653-LNmet | APOA4 | 1475 | hg19 | chr11 | 116693892 | 116693892 | Missense_Mutation | SNP | C | C | T | novel | Somatic | V6M | 0.114173 | NM_000482.4 | 508 | 450 | 58 |
| 653-LNmet | FOXR1 | 1491 | hg19 | chr11 | 118851273 | 118851273 | Missense_Mutation | SNP | G | G | C | novel | Somatic | D229H | 0.161937 | NM_181721.3 | 599 | 502 | 97 |
| 653-LNmet | FKBP4 | 607 | hg19 | chr12 | 2904327 | 2904327 | Missense_Mutation | SNP | G | G | A | novel | Somatic | A8T | 0.116601 | NM_002014.4 | 506 | 447 | 59 |
| 653-LNmet | PEX5 | 641 | hg19 | chr12 | 7360267 | 7360267 | Missense_Mutation | SNP | G | G | A | novel | Somatic | E348K | 0.187063 | NM_001300789.1 | 572 | 465 | 107 |
| 653-LNmet | PEX5 | 641 | hg19 | chr12 | 7360309 | 7360309 | Missense_Mutation | SNP | G | G | A | novel | Somatic | E362K | 0.193287 | NM_001300789.1 | 864 | 697 | 167 |
| 653-LNmet | KMT2D | 120 | hg19 | chr12 | 49420432 | 49420432 | Missense_Mutation | SNP | C | C | T | novel | Somatic | R5106H | 0.13913 | NM_003482.3 | 804 | 692 | 112 |
| 653-LNmet | KCNMB4 | 140 | hg19 | chr12 | 70760824 | 70760824 | Missense_Mutation | SNP | G | G | C | novel | Somatic | E104Q | 0.039514 | NM_014505.6 | 658 | 632 | 26 |
| 653-LNmet | APPL2 | 1390 | hg19 | chr12 | 105629745 | 105629745 | Missense_Mutation | SNP | T | T | G | novel | Somatic | S16R | 0.106272 | NM_018171.5 | 572 | 511 | 61 |
| 653-LNmet | ACACB | 1421 | hg19 | chr12 | 109617746 | 109617746 | Missense_Mutation | SNP | G | G | A | novel | Somatic | V558M | 0.109489 | NM_001093.4 | 684 | 609 | 75 |
| 653-LNmet | NAA25 | 1443 | hg19 | chr12 | 112477055 | 112477055 | Missense_Mutation | SNP | T | T | G | novel | Somatic | K876T | 0.119086 | NM_024953.4 | 415 | 342 | 73 |
| 653-LNmet | NAA25 | 1443 | hg19 | chr12 | 112480913 | 112480913 | Missense_Mutation | SNP | C | C | G | novel | Somatic | E785Q | 0.188034 | NM_024953.4 | 234 | 190 | 44 |
| 653-LNmet | NCOR2 | 192 | hg19 | chr12 | 124829333 | 124829333 | In_Frame_Del | DEL | CAGGCTCTCCTCGTA | CAGGCTCTCCTCGTA | - | novel | Somatic | Y1504_L1508del | 0.094891 | NM_006312.6 | 411 | 372 | 39 |
| 653-LNmet | FRY | 104 | hg19 | chr13 | 32869463 | 32869463 | Nonsense_Mutation | SNP | C | C | T | novel | Somatic | Q2970Ter | 0.244807 | NM_023037.3 | 673 | 508 | 165 |
| 653-LNmet | STARD13 | 842 | hg19 | chr13 | 33703085 | 33703085 | Missense_Mutation | SNP | A | A | T | novel | Somatic | S577T | 0.219477 | NM_178006.4 | 688 | 537 | 151 |
| 653-LNmet | SLC7A7 | 762 | hg19 | chr14 | 23242892 | 23242892 | Frame_Shift_Del | DEL | AT | AT | - | novel | Somatic | M488VfsTer23 | 0.109375 | NM_001126106.2 | 703 | 626 | 77 |
| 653-LNmet | AJUBA | 763 | hg19 | chr14 | 23443312 | 23443312 | Frame_Shift_Del | DEL | TG | TG | - | novel | Somatic | I479RfsTer13 | 0.077453 | NM_032876.6 | 580 | 535 | 45 |
| 653-LNmet | SLC7A8 | 765 | hg19 | chr14 | 23635540 | 23635540 | Splice_Region | SNP | C | C | G | novel | Somatic |  | 0.157277 | NM_012244.4 | 426 | 359 | 67 |
| 653-LNmet | MYH6 | 95 | hg19 | chr14 | 23865568 | 23865568 | Missense_Mutation | SNP | C | C | T | novel | Somatic | R785H | 0.120125 | NM_002471.3 | 641 | 564 | 77 |
| 653-LNmet | BRMS1L | 107 | hg19 | chr14 | 36331851 | 36331851 | Missense_Mutation | SNP | G | G | C | novel | Somatic | R166T | 0.126984 | NM_032352.4 | 567 | 495 | 72 |
| 653-LNmet | KLHDC1 | 120 | hg19 | chr14 | 50196214 | 50196214 | Missense_Mutation | SNP | A | A | G | novel | Somatic | R220G | 0.072193 | NM_172193.3 | 748 | 694 | 54 |
| 653-LNmet | FRMD6 | 983 | hg19 | chr14 | 52186748 | 52186748 | Intron | SNP | A | A | G | novel | Somatic |  | 0.138514 | NM_001267046.2 | 296 | 255 | 41 |
| 653-LNmet | TBPL2 | 1011 | hg19 | chr14 | 55907214 | 55907214 | Missense_Mutation | SNP | C | C | T | novel | Somatic | R17H | 0.121857 | NM_199047.2 | 516 | 453 | 63 |
| 653-LNmet | DACT1 | 129 | hg19 | chr14 | 59112729 | 59112729 | Missense_Mutation | SNP | A | A | C | novel | Somatic | H463P | 0.144144 | NM_016651.6 | 777 | 665 | 112 |
| 653-LNmet | NEK9 | 1161 | hg19 | chr14 | 75567827 | 75567827 | Missense_Mutation | SNP | T | T | G | novel | Somatic | N624H | 0.107323 | NM_033116.6 | 792 | 707 | 85 |
| 653-LNmet | TSHR | 150 | hg19 | chr14 | 81558939 | 81558939 | Missense_Mutation | SNP | G | G | C | novel | Somatic | E178Q | 0.049133 | NM_000369.3 | 342 | 325 | 17 |
| 653-LNmet | MARK3 | 172 | hg19 | chr14 | 103934491 | 103934491 | Nonsense_Mutation | SNP | C | C | G | novel | Somatic | S411Ter | 0.120098 | NM_001128918.3 | 815 | 717 | 98 |
| 653-LNmet | NPAP1 | 775 | hg19 | chr15 | 24921279 | 24921279 | Missense_Mutation | SNP | G | G | T | novel | Somatic | G89C | 0.272727 | NM_018958.3 | 450 | 327 | 123 |
| 653-LNmet | FMN1 | 840 | hg19 | chr15 | 33069078 | 33069078 | Missense_Mutation | SNP | C | C | G | novel | Somatic | E1391D | 0.104294 | NM_001277313.2 | 652 | 584 | 68 |
| 653-LNmet | SPG11 | 927 | hg19 | chr15 | 44900791 | 44900791 | Missense_Mutation | SNP | C | C | T | novel | Somatic | E1102K | 0.07967 | NM_025137.4 | 364 | 335 | 29 |
| 653-LNmet | SECISBP2L | 14 | hg19 | chr15 | 49284926 | 49284926 | Missense_Mutation | SNP | T | T | C | novel | Somatic | S941G | 0.066214 | NM_001193489.2 | 589 | 550 | 39 |
| 653-LNmet | SECISBP2L | 14 | hg19 | chr15 | 49284937 | 49284937 | In_Frame_Ins | INS | - | - | TCCCAGCACTTG | novel | Somatic | T933_G936dup | 0.052356 | NM_001193489.2 | 572 | 542 | 30 |
| 653-LNmet | SKOR1 | 1104 | hg19 | chr15 | 68120444 | 68120444 | Missense_Mutation | SNP | G | G | A | novel | Somatic | E760K | 0.068063 | NM_001365915.1 | 191 | 178 | 13 |
| 653-LNmet | EDC3 | 144 | hg19 | chr15 | 74925234 | 74925234 | Missense_Mutation | DNP | TC | TC | AT | novel | Somatic | N416Y | 0.257932 | NM_025083.5 | 962 | 710 | 252 |
| 653-LNmet | PTPN9 | 145 | hg19 | chr15 | 75871117 | 75871117 | Translation_Start_Site | SNP | T | T | C | novel | Somatic | M1? | 0.063694 | NM_002833.4 | 156 | 146 | 10 |
| 653-LNmet | TBC1D2B | 1182 | hg19 | chr15 | 78305643 | 78305643 | Missense_Mutation | SNP | C | C | T | novel | Somatic | G598R | 0.142433 | NM_144572.1 | 337 | 289 | 48 |
| 653-LNmet | MEX3B | 1213 | hg19 | chr15 | 82336523 | 82336523 | Missense_Mutation | SNP | C | C | T | novel | Somatic | G230S | 0.113009 | NM_032246.6 | 761 | 675 | 86 |
| 653-LNmet | LINS1 | 1356 | hg19 | chr15 | 101110045 | 101110045 | Missense_Mutation | SNP | C | C | T | novel | Somatic | G558S | 0.202847 | NM_001040616.3 | 562 | 448 | 114 |
| 653-LNmet | RHBDF1 | 585 | hg19 | chr16 | 111989 | 111989 | Missense_Mutation | SNP | G | G | A | novel | Somatic | R339W | 0.079019 | NM_022450.5 | 367 | 338 | 29 |
| 653-LNmet | ZNF263 | 610 | hg19 | chr16 | 3340047 | 3340047 | Missense_Mutation | SNP | G | G | A | novel | Somatic | G514E | 0.176166 | NM_005741.5 | 768 | 632 | 136 |
| 653-LNmet | RBBP6 | 772 | hg19 | chr16 | 24580362 | 24580362 | Missense_Mutation | SNP | G | G | A | novel | Somatic | R784H | 0.088339 | NM_006910.5 | 565 | 515 | 50 |
| 653-LNmet | ZNF747 | 818 | hg19 | chr16 | 30545884 | 30545884 | Missense_Mutation | SNP | G | G | T | novel | Somatic | F39L | 0.088117 | NM_001305018.1 | 749 | 683 | 66 |
| 653-LNmet | ZNF469 | 1260 | hg19 | chr16 | 88496865 | 88496865 | Missense_Mutation | SNP | G | G | A | novel | Somatic | R996H | 0.104938 | NM_001367624.2 | 162 | 145 | 17 |
| 653-LNmet | SGSM2 | 602 | hg19 | chr17 | 2266817 | 2266817 | Missense_Mutation | SNP | G | G | T | novel | Somatic | R244L | 0.029762 | NM_014853.3 | 503 | 488 | 15 |
| 653-LNmet | TRPV1 | 611 | hg19 | chr17 | 3477162 | 3477162 | Missense_Mutation | SNP | G | G | T | novel | Somatic | P623H | 0.17484 | NM_080706.3 | 469 | 387 | 82 |
| 653-LNmet | ZZEF1 | 76 | hg19 | chr17 | 4045957 | 4045957 | Missense_Mutation | SNP | C | C | A | novel | Somatic | G78V | 0.115625 | NM_015113.4 | 640 | 566 | 74 |
| 653-LNmet | ZFP3 | 623 | hg19 | chr17 | 4995203 | 4995203 | Missense_Mutation | SNP | C | C | A | novel | Somatic | S135Y | 0.154255 | NM_153018.3 | 563 | 476 | 87 |
| 653-LNmet | C1QBP | 625 | hg19 | chr17 | 5336378 | 5336378 | Missense_Mutation | SNP | A | A | G | novel | Somatic | I269T | 0.101604 | NM_001212.4 | 935 | 840 | 95 |
| 653-LNmet | NLRP1 | 626 | hg19 | chr17 | 5461658 | 5461658 | Splice_Site | SNP | C | C | T | novel | Somatic |  | 0.11413 | NM_033004.4 | 368 | 326 | 42 |
| 653-LNmet | PLSCR3 | 640 | hg19 | chr17 | 7296681 | 7296681 | Missense_Mutation | SNP | A | A | G | novel | Somatic | F97L | 0.097872 | NM_020360.4 | 470 | 424 | 46 |
| 653-LNmet | TP53 | 642 | hg19 | chr17 | 7578413 | 7578413 | Missense_Mutation | SNP | C | C | A | novel | Somatic | V173L | 0.415842 | NM_000546.5 | 808 | 472 | 336 |
| 653-LNmet | CFAP52 | 657 | hg19 | chr17 | 9515797 | 9515797 | Splice_Site | SNP | G | G | C | novel | Somatic |  | 0.129412 | NM_145054.5 | 425 | 370 | 55 |
| 653-LNmet | KCNJ12 | 747 | hg19 | chr17 | 21319451 | 21319451 | Missense_Mutation | SNP | C | C | T | novel | Somatic | S266L | 0.051629 | NM_021012.5 | 1993 | 1890 | 103 |
| 653-LNmet | LRRC37B | 816 | hg19 | chr17 | 30376371 | 30376371 | Missense_Mutation | SNP | G | G | C | novel | Somatic | E878D | 0.106667 | NM_052888.3 | 675 | 603 | 72 |
| 653-LNmet | TTC25 | 111 | hg19 | chr17 | 40094807 | 40094807 | Splice_Region | SNP | C | C | A | novel | Somatic |  | 0.107143 | NM_031421.5 | 588 | 525 | 63 |
| 653-LNmet | PHB | 947 | hg19 | chr17 | 47489050 | 47489050 | In_Frame_Del | DEL | AGTGATGACTGG | AGTGATGACTGG | - | novel | Somatic | P77_T80del | 0.037613 | NM_002634.4 | 771 | 742 | 29 |
| 653-LNmet | ABCC3 | 956 | hg19 | chr17 | 48753812 | 48753812 | Missense_Mutation | SNP | G | G | A | novel | Somatic | E1081K | 0.184211 | NM_003786.4 | 722 | 589 | 133 |
| 653-LNmet | HELZ | 135 | hg19 | chr17 | 65105469 | 65105469 | Nonsense_Mutation | SNP | G | G | A | novel | Somatic | Q1418Ter | 0.134912 | NM_014877.4 | 971 | 840 | 131 |
| 653-LNmet | BTBD17 | 1137 | hg19 | chr17 | 72356250 | 72356250 | Missense_Mutation | SNP | T | T | C | novel | Somatic | T74A | 0.146978 | NM_001080466.2 | 724 | 617 | 107 |
| 653-LNmet | GRIN2C | 1140 | hg19 | chr17 | 72847822 | 72847822 | Missense_Mutation | SNP | A | A | C | novel | Somatic | L336R | 0.121849 | NM_000835.6 | 238 | 209 | 29 |
| 653-LNmet | ACOX1 | 1149 | hg19 | chr17 | 73947542 | 73947542 | Missense_Mutation | SNP | C | C | T | novel | Somatic | R354Q | 0.342799 | NM_004035.7 | 985 | 647 | 338 |
| 653-LNmet | ARHGAP28 | 637 | hg19 | chr18 | 6837315 | 6837315 | Missense_Mutation | SNP | G | G | A | novel | Somatic | V149M | 0.084775 | NM_001366230.1 | 578 | 529 | 49 |
| 653-LNmet | LAMA1 | 79 | hg19 | chr18 | 6995394 | 6995394 | Missense_Mutation | SNP | C | C | T | novel | Somatic | E1620K | 0.099455 | NM_005559.4 | 734 | 661 | 73 |
| 653-LNmet | RNMT | 86 | hg19 | chr18 | 13731654 | 13731654 | Missense_Mutation | SNP | G | G | C | novel | Somatic | K46N | 0.100471 | NM_003799.3 | 635 | 571 | 64 |
| 653-LNmet | PALM | 590 | hg19 | chr19 | 746527 | 746527 | Missense_Mutation | SNP | G | G | A | novel | Somatic | G293S | 0.115124 | NM_002579.3 | 443 | 392 | 51 |
| 653-LNmet | TCF3 | 597 | hg19 | chr19 | 1611735 | 1611735 | Missense_Mutation | SNP | C | C | T | novel | Somatic | E646K | 0.105036 | NM_003200.5 | 695 | 622 | 73 |
| 653-LNmet | LINGO3 | 602 | hg19 | chr19 | 2290317 | 2290317 | Missense_Mutation | SNP | C | C | T | novel | Somatic | G487S | 0.071642 | NM_001101391.2 | 335 | 311 | 24 |
| 653-LNmet | ADGRE1 | 637 | hg19 | chr19 | 6897177 | 6897177 | Missense_Mutation | SNP | C | C | G | novel | Somatic | Q86E | 0.076305 | NM_001974.5 | 498 | 460 | 38 |
| 653-LNmet | CD209 | 644 | hg19 | chr19 | 7812395 | 7812395 | Translation_Start_Site | SNP | C | C | G | novel | Somatic | M1? | 0.083176 | NM_021155.4 | 529 | 485 | 44 |
| 653-LNmet | MUC16 | 81 | hg19 | chr19 | 9057709 | 9057709 | Missense_Mutation | SNP | C | C | G | novel | Somatic | E9913Q | 0.113793 | NM_024690.2 | 580 | 514 | 66 |
| 653-LNmet | MUC16 | 81 | hg19 | chr19 | 9064257 | 9064257 | Missense_Mutation | SNP | T | T | C | novel | Somatic | D7730G | 0.128698 | NM_024690.2 | 676 | 589 | 87 |
| 653-LNmet | RGL3 | 672 | hg19 | chr19 | 11526646 | 11526646 | Missense_Mutation | SNP | C | C | G | novel | Somatic | E202Q | 0.200913 | NM_001161616.3 | 657 | 525 | 132 |
| 653-LNmet | ECSIT | 673 | hg19 | chr19 | 11618361 | 11618361 | Missense_Mutation | SNP | G | G | A | novel | Somatic | P321L | 0.08209 | NM_016581.5 | 402 | 369 | 33 |
| 653-LNmet | NFIX | 685 | hg19 | chr19 | 13189438 | 13189438 | Missense_Mutation | SNP | C | C | G | novel | Somatic | L331V | 0.076 | NM_001271043.2 | 250 | 231 | 19 |
| 653-LNmet | PODNL1 | 692 | hg19 | chr19 | 14043705 | 14043705 | Missense_Mutation | SNP | C | C | T | novel | Somatic | G451D | 0.020202 | NM_001370095.1 | 495 | 485 | 10 |
| 653-LNmet | NOTCH3 | 701 | hg19 | chr19 | 15284912 | 15284912 | Missense_Mutation | SNP | C | C | T | novel | Somatic | R1568Q | 0.110108 | NM_000435.3 | 552 | 491 | 61 |
| 653-LNmet | NOTCH3 | 701 | hg19 | chr19 | 15298137 | 15298137 | Missense_Mutation | SNP | G | G | A | novel | Somatic | T540M | 0.148607 | NM_000435.3 | 323 | 275 | 48 |
| 653-LNmet | BRD4 | 702 | hg19 | chr19 | 15367789 | 15367789 | Missense_Mutation | SNP | C | C | T | novel | Somatic | E513K | 0.120818 | NM_058243.2 | 538 | 473 | 65 |
| 653-LNmet | CALR3 | 711 | hg19 | chr19 | 16589968 | 16589968 | Nonsense_Mutation | SNP | G | G | A | novel | Somatic | Q377Ter | 0.109489 | NM_145046.4 | 684 | 609 | 75 |
| 653-LNmet | VSTM2B | 814 | hg19 | chr19 | 30018144 | 30018144 | In_Frame_Del | DEL | GTGACA | GTGACA | - | novel | Somatic | T38_V39del | 0.041667 | NM_001146339.1 | 575 | 551 | 24 |
| 653-LNmet | DMKN | 859 | hg19 | chr19 | 36001155 | 36001155 | Splice_Site | SNP | C | C | T | novel | Somatic |  | 0.108209 | NM_033317.5 | 534 | 476 | 58 |
| 653-LNmet | WDR87 | 877 | hg19 | chr19 | 38385106 | 38385106 | Missense_Mutation | SNP | C | C | A | novel | Somatic | V413L | 0.118443 | NM_001291088.2 | 591 | 521 | 70 |
| 653-LNmet | LMTK3 | 958 | hg19 | chr19 | 49002273 | 49002273 | Missense_Mutation | SNP | G | G | A | novel | Somatic | R714W | 0.129808 | NM_001080434.1 | 415 | 361 | 54 |
| 653-LNmet | IL4I1 | 969 | hg19 | chr19 | 50393727 | 50393727 | Missense_Mutation | SNP | C | C | G | novel | Somatic | E324Q | 0.037736 | NM_001258018.2 | 583 | 561 | 22 |
| 653-LNmet | NLRP9 | 126 | hg19 | chr19 | 56244334 | 56244334 | Missense_Mutation | SNP | C | C | T | novel | Somatic | R288Q | 0.107955 | NM_176820.4 | 351 | 313 | 38 |
| 653-LNmet | TRIM28 | 1035 | hg19 | chr19 | 59060457 | 59060457 | Silent | SNP | C | C | T | novel | Somatic | P504= | 0.107042 | NM_005762.3 | 710 | 634 | 76 |
| 653-LNmet | ACTR5 | 870 | hg19 | chr20 | 37384617 | 37384617 | Missense_Mutation | SNP | G | G | A | novel | Somatic | A371T | 0.075492 | NM_024855.4 | 914 | 845 | 69 |
| 653-LNmet | CDH26 | 128 | hg19 | chr20 | 58574656 | 58574656 | Missense_Mutation | SNP | G | G | A | novel | Somatic | E679K | 0.12611 | NM_177980.4 | 562 | 491 | 71 |
| 653-LNmet | URB1 | 105 | hg19 | chr21 | 33706558 | 33706558 | Missense_Mutation | SNP | C | C | A | novel | Somatic | G1591W | 0.124272 | NM_014825.3 | 514 | 450 | 64 |
| 653-LNmet | KRTAP10-9 | 936 | hg19 | chr21 | 46047486 | 46047486 | Missense_Mutation | SNP | C | C | T | novel | Somatic | S133F | 0.10961 | NM_198690.3 | 666 | 593 | 73 |
| 653-LNmet | CLTCL1 | 91 | hg19 | chr22 | 19168265 | 19168265 | Missense_Mutation | SNP | C | C | T | novel | Somatic | E1628K | 0.10087 | NM_007098.4 | 575 | 517 | 58 |
| 653-LNmet | DDX17 | 881 | hg19 | chr22 | 38884088 | 38884088 | Missense_Mutation | SNP | C | C | G | novel | Somatic | D494H | 0.101515 | NM_001098504.2 | 660 | 593 | 67 |
| 653-LNmet | NFAM1 | 911 | hg19 | chr22 | 42783071 | 42783071 | Missense_Mutation | SNP | C | C | T | novel | Somatic | R226H | 0.205776 | NM_145912.8 | 553 | 439 | 114 |
| 653-LNmet | FBLN1 | 935 | hg19 | chr22 | 45929685 | 45929685 | Missense_Mutation | SNP | G | G | A | novel | Somatic | E231K | 0.136816 | NM_006486.3 | 397 | 342 | 55 |
| 653-LNmet | ZBED4 | 968 | hg19 | chr22 | 50280804 | 50280804 | Missense_Mutation | SNP | C | C | G | novel | Somatic | P1165R | 0.089059 | NM_014838.3 | 393 | 358 | 35 |
| 653-LNmet | IL17REL | 969 | hg19 | chr22 | 50439591 | 50439591 | Missense_Mutation | SNP | G | G | A | novel | Somatic | T67M | 0.228966 | NM_001371417.1 | 724 | 558 | 166 |
| 653-LNmet | FANCB | 698 | hg19 | chrX | 14861739 | 14861739 | Missense_Mutation | SNP | C | C | G | novel | Somatic | E844Q | 0.169154 | NM_001018113.3 | 201 | 167 | 34 |
| 653-LNmet | UBA1 | 117 | hg19 | chrX | 47074225 | 47074225 | Missense_Mutation | SNP | G | G | A | novel | Somatic | R1025Q | 0.154362 | NM_003334.4 | 298 | 252 | 46 |
| 653-LNmet | ITIH6 | 125 | hg19 | chrX | 54823403 | 54823403 | Missense_Mutation | SNP | G | G | A | novel | Somatic | H77Y | 0.159744 | NM_198510.3 | 312 | 262 | 50 |
| 653-LNmet | AR | 136 | hg19 | chrX | 66942684 | 66942684 | Missense_Mutation | SNP | T | T | A | novel | Somatic | L822Q | 0.132159 | NM_000044.6 | 227 | 197 | 30 |
| 653-LNmet | GPR174 | 1183 | hg19 | chrX | 78426636 | 78426636 | Missense_Mutation | SNP | C | C | G | novel | Somatic | F44L | 0.096386 | NM_032553.2 | 249 | 225 | 24 |
| 653-LNmet | PCDH11X | 19 | hg19 | chrX | 91134187 | 91134187 | Missense_Mutation | SNP | C | C | T | novel | Somatic | S983L | 0.133916 | NM_032968.4 | 687 | 595 | 92 |
| 653-LNmet | GPRASP1 | 1362 | hg19 | chrX | 101910070 | 101910070 | Missense_Mutation | SNP | G | G | C | novel | Somatic | W410S | 0.182018 | NM_001184727.2 | 456 | 373 | 83 |
| 685-PTsup | CAMTA1 | 644 | hg19 | chr1 | 7723734 | 7723734 | Missense_Mutation | SNP | C | C | T | novel | Somatic | P376L | 0.231198 | NM_015215.4 | 716 | 550 | 166 |
| 685-PTsup | COL16A1 | 830 | hg19 | chr1 | 32127969 | 32127969 | Missense_Mutation | SNP | C | C | T | novel | Somatic | G1216D | 0.168632 | NM_001856.4 | 848 | 705 | 143 |
| 685-PTsup | COL9A2 | 896 | hg19 | chr1 | 40767555 | 40767555 | Missense_Mutation | SNP | C | C | T | novel | Somatic | R600H | 0.188285 | NM_001852.4 | 239 | 194 | 45 |
| 685-PTsup | TOE1 | 934 | hg19 | chr1 | 45806886 | 45806886 | Missense_Mutation | SNP | C | C | T | novel | Somatic | T65M | 0.241685 | NM_025077.4 | 451 | 342 | 109 |
| 685-PTsup | FLAD1 | 1767 | hg19 | chr1 | 154962868 | 154962868 | Missense_Mutation | SNP | A | A | G | novel | Somatic | E473G | 0.204545 | NM_025207.5 | 352 | 280 | 72 |
| 685-PTsup | CD1E | 1792 | hg19 | chr1 | 158325670 | 158325670 | Missense_Mutation | SNP | C | C | A | novel | Somatic | Q227K | 0.182886 | NM_030893.4 | 595 | 486 | 109 |
| 685-PTsup | RALGPS2 | 243 | hg19 | chr1 | 178777229 | 178777229 | Missense_Mutation | SNP | C | C | A | novel | Somatic | P88Q | 0.257463 | NM_152663.5 | 266 | 197 | 69 |
| 685-PTsup | KIF21B | 2118 | hg19 | chr1 | 200965373 | 200965373 | Missense_Mutation | SNP | C | C | T | novel | Somatic | R743K | 0.219072 | NM_001252100.2 | 388 | 303 | 85 |
| 685-PTsup | USH2A | 279 | hg19 | chr1 | 216420019 | 216420019 | Missense_Mutation | SNP | C | C | A | novel | Somatic | G906V | 0.273171 | NM_206933.3 | 409 | 297 | 112 |
| 685-PTsup | USP34 | 131 | hg19 | chr2 | 61570965 | 61570965 | Missense_Mutation | SNP | C | C | T | novel | Somatic | E829K | 0.565111 | NM_014709.4 | 407 | 177 | 230 |
| 685-PTsup | FIGLA | 1126 | hg19 | chr2 | 71017734 | 71017734 | Missense_Mutation | SNP | C | C | T | novel | Somatic | A13T | 0.59863 | NM_001004311.3 | 728 | 291 | 437 |
| 685-PTsup | LRRTM1 | 1199 | hg19 | chr2 | 80529822 | 80529822 | Missense_Mutation | SNP | G | G | C | novel | Somatic | L375V | 0.024155 | NM_178839.5 | 1034 | 1009 | 25 |
| 685-PTsup | TGOLN2 | 1237 | hg19 | chr2 | 85554654 | 85554654 | Frame_Shift_Ins | INS | - | - | G | novel | Somatic | S68Ter | 0.383681 | NM_006464.4 | 574 | 353 | 221 |
| 685-PTsup | REV1 | 1348 | hg19 | chr2 | 100017796 | 100017796 | In_Frame_Del | DEL | CCA | CCA | - | novel | Somatic | V1221del | 0.111475 | NM_016316.4 | 304 | 270 | 34 |
| 685-PTsup | DPP10 | 22 | hg19 | chr2 | 116539930 | 116539930 | Missense_Mutation | SNP | C | C | A | novel | Somatic | P513T | 0.301493 | NM_001321905.2 | 335 | 234 | 101 |
| 685-PTsup | PRPF40A | 1756 | hg19 | chr2 | 153526794 | 153526794 | Missense_Mutation | SNP | C | C | G | novel | Somatic | Q569H | 0.230548 | NM_001365596.2 | 346 | 266 | 80 |
| 685-PTsup | TTN | 1955 | hg19 | chr2 | 179481856 | 179481856 | Missense_Mutation | SNP | C | C | T | novel | Somatic | D15956N | 0.082581 | NM_001267550.2 | 774 | 710 | 64 |
| 685-PTsup | CPS1 | 2198 | hg19 | chr2 | 211473152 | 211473152 | Missense_Mutation | SNP | G | G | A | novel | Somatic | V754I | 0.231317 | NM_001875.5 | 281 | 216 | 65 |
| 685-PTsup | CNTN6 | 74 | hg19 | chr3 | 1445041 | 1445041 | Missense_Mutation | SNP | C | C | A | novel | Somatic | T1009N | 0.300469 | NM_001349351.2 | 212 | 148 | 64 |
| 685-PTsup | LRRN1 | 614 | hg19 | chr3 | 3887647 | 3887647 | Missense_Mutation | SNP | C | C | T | novel | Somatic | T441M | 0.256158 | NM_020873.7 | 203 | 151 | 52 |
| 685-PTsup | SRGAP3 | 81 | hg19 | chr3 | 9074383 | 9074383 | Missense_Mutation | SNP | G | G | A | novel | Somatic | S497L | 0.441799 | NM_014850.4 | 377 | 210 | 167 |
| 685-PTsup | FYCO1 | 116 | hg19 | chr3 | 46009433 | 46009433 | Missense_Mutation | SNP | C | C | T | novel | Somatic | E465K | 0.234421 | NM_024513.4 | 337 | 258 | 79 |
| 685-PTsup | DOCK3 | 15 | hg19 | chr3 | 51417604 | 51417604 | Frame_Shift_Del | DEL | C | C | - | novel | Somatic | P1852QfsTer45 | 0.022177 | NM_004947.5 | 496 | 485 | 11 |
| 685-PTsup | CSNKA2IP | Unknown | hg19 | chr3 | 88515123 | 88515123 | Frame_Shift_Del | DEL | C | C | - | novel | Somatic | K207NfsTer35 | 0.236196 | NM_001368165.1 | 322 | 245 | 77 |
| 685-PTsup | MORC1 | 176 | hg19 | chr3 | 108723661 | 108723661 | Splice_Region | SNP | C | C | T | novel | Somatic |  | 0.138636 | NM_014429.4 | 439 | 378 | 61 |
| 685-PTsup | C3orf52 | 1438 | hg19 | chr3 | 111835488 | 111835488 | Splice_Site | SNP | G | G | A | novel | Somatic |  | 0.124611 | NM_024616.2 | 321 | 281 | 40 |
| 685-PTsup | XRN1 | 1669 | hg19 | chr3 | 142122569 | 142122569 | Missense_Mutation | SNP | G | G | C | novel | Somatic | R684G | 0.171701 | NM_019001.5 | 629 | 521 | 108 |
| 685-PTsup | PHC3 | 29 | hg19 | chr3 | 169815155 | 169815155 | Missense_Mutation | SNP | C | C | T | novel | Somatic | D951N | 0.245375 | NM_024947.4 | 1836 | 1385 | 451 |
| 685-PTsup | TNIK | 1890 | hg19 | chr3 | 170802067 | 170802067 | Nonsense_Mutation | SNP | C | C | A | novel | Somatic | E1016Ter | 0.776874 | NM_015028.4 | 2334 | 520 | 1814 |
| 685-PTsup | FNDC3B | 29 | hg19 | chr3 | 172115023 | 172115023 | Missense_Mutation | SNP | G | G | A | novel | Somatic | V1125I | 0.179724 | NM_022763.4 | 434 | 356 | 78 |
| 685-PTsup | ZNF639 | 243 | hg19 | chr3 | 179051897 | 179051897 | Missense_Mutation | SNP | G | G | A | novel | Somatic | R382Q | 0.302419 | NM_001303425.2 | 494 | 344 | 150 |
| 685-PTsup | PEX5L | 244 | hg19 | chr3 | 179533791 | 179533791 | Missense_Mutation | SNP | C | C | G | novel | Somatic | G314A | 0.174242 | NM_016559.3 | 396 | 327 | 69 |
| 685-PTsup | MAP6D1 | 1985 | hg19 | chr3 | 183543193 | 183543193 | Missense_Mutation | SNP | G | G | A | novel | Somatic | S48L | 0.161089 | NM_024871.3 | 1284 | 1077 | 207 |
| 685-PTsup | KNG1 | 2007 | hg19 | chr3 | 186442948 | 186442948 | Missense_Mutation | SNP | G | G | A | novel | Somatic | D155N | 0.169451 | NM_001102416.3 | 419 | 348 | 71 |
| 685-PTsup | GUF1 | 115 | hg19 | chr4 | 44688546 | 44688546 | Missense_Mutation | SNP | A | A | G | novel | Somatic | N252D | 0.288136 | NM_021927.3 | 177 | 126 | 51 |
| 685-PTsup | ADGRL3 | 16 | hg19 | chr4 | 62812686 | 62812686 | Missense_Mutation | SNP | C | C | T | novel | Somatic | T825M | 0.333333 | NM_001322402.2 | 233 | 155 | 78 |
| 685-PTsup | ADAMTS3 | 17 | hg19 | chr4 | 73414436 | 73414436 | Missense_Mutation | SNP | C | C | A | novel | Somatic | G88V | 0.226044 | NM_014243.3 | 406 | 314 | 92 |
| 685-PTsup | WDFY3 | 154 | hg19 | chr4 | 85672857 | 85672857 | Missense_Mutation | SNP | C | C | T | novel | Somatic | D1918N | 0.423333 | NM_014991.5 | 300 | 173 | 127 |
| 685-PTsup | ADCY2 | 80 | hg19 | chr5 | 7709500 | 7709500 | Splice_Site | DNP | GG | GG | TT | novel | Somatic |  | 0.202105 | NM_020546.3 | 463 | 367 | 96 |
| 685-PTsup | FGF10 | 923 | hg19 | chr5 | 44310530 | 44310530 | Missense_Mutation | SNP | G | G | A | novel | Somatic | S143L | 0.092486 | NM_004465.2 | 173 | 157 | 16 |
| 685-PTsup | POC5 | 144 | hg19 | chr5 | 74998445 | 74998445 | Missense_Mutation | SNP | C | C | G | novel | Somatic | W166C | 0.215976 | NM_001099271.2 | 338 | 265 | 73 |
| 685-PTsup | MTRNR2L2 | 1194 | hg19 | chr5 | 79945871 | 79945871 | Missense_Mutation | SNP | G | G | A | novel | Somatic | S12L | 0.050898 | NM_001190470.2 | 334 | 317 | 17 |
| 685-PTsup | NUDT12 | 171 | hg19 | chr5 | 102886645 | 102886645 | Nonsense_Mutation | SNP | G | G | A | novel | Somatic | Q436Ter | 0.203704 | NM_031438.4 | 323 | 257 | 66 |
| 685-PTsup | CXCL14 | 1614 | hg19 | chr5 | 134907551 | 134907551 | Missense_Mutation | SNP | C | C | T | novel | Somatic | E98K | 0.288026 | NM_004887.5 | 309 | 220 | 89 |
| 685-PTsup | FAM193B | 241 | hg19 | chr5 | 176965990 | 176965990 | Silent | SNP | G | G | A | novel | Somatic | G123= | 0.226829 | NM_001190946.3 | 410 | 317 | 93 |
| 685-PTsup | ENPP1 | 1593 | hg19 | chr6 | 132198274 | 132198274 | Missense_Mutation | SNP | T | T | A | novel | Somatic | D622E | 0.20339 | NM_006208.3 | 354 | 282 | 72 |
| 685-PTsup | TBPL1 | 1609 | hg19 | chr6 | 134303713 | 134303713 | Nonsense_Mutation | SNP | T | T | G | novel | Somatic | L48Ter | 0.21813 | NM_004865.4 | 353 | 276 | 77 |
| 685-PTsup | MAP7 | 203 | hg19 | chr6 | 136704883 | 136704883 | Missense_Mutation | SNP | G | G | A | novel | Somatic | S210L | 0.289941 | NM_001198609.1 | 169 | 120 | 49 |
| 685-PTsup | PCMT1 | 216 | hg19 | chr6 | 150123393 | 150123393 | Missense_Mutation | SNP | G | G | A | novel | Somatic | G246R | 0.224561 | NM_001252049.1 | 284 | 220 | 64 |
| 685-PTsup | SUN1 | 591 | hg19 | chr7 | 905651 | 905651 | Missense_Mutation | SNP | C | C | G | novel | Somatic | L781V | 0.170683 | NM_001367651.1 | 498 | 413 | 85 |
| 685-PTsup | SLC29A4 | 625 | hg19 | chr7 | 5334535 | 5334535 | Missense_Mutation | SNP | C | C | T | novel | Somatic | R197W | 0.17484 | NM_001040661.2 | 469 | 387 | 82 |
| 685-PTsup | SP4 | 748 | hg19 | chr7 | 21468306 | 21468306 | Missense_Mutation | SNP | G | G | A | novel | Somatic | E7K | 0.027915 | NM_003112.5 | 598 | 581 | 17 |
| 685-PTsup | NPC1L1 | 925 | hg19 | chr7 | 44556890 | 44556890 | Missense_Mutation | SNP | G | G | T | novel | Somatic | A1095E | 0.285276 | NM_013389.3 | 325 | 232 | 93 |
| 685-PTsup | SRRT | 1351 | hg19 | chr7 | 100484957 | 100484957 | Missense_Mutation | SNP | G | G | C | novel | Somatic | K664N | 0.209677 | NM_015908.6 | 310 | 245 | 65 |
| 685-PTsup | ARFGEF1 | 17 | hg19 | chr8 | 68170429 | 68170429 | Missense_Mutation | SNP | C | C | T | novel | Somatic | V778I | 0.228723 | NM_006421.5 | 375 | 289 | 86 |
| 685-PTsup | OSR2 | 1347 | hg19 | chr8 | 99961377 | 99961377 | Missense_Mutation | SNP | G | G | A | novel | Somatic | R187H | 0.203673 | NM_001286841.1 | 1794 | 1428 | 366 |
| 685-PTsup | RIMS2 | 173 | hg19 | chr8 | 105105860 | 105105860 | Missense_Mutation | SNP | C | C | G | novel | Somatic | N1285K | 0.131403 | NM_001348484.2 | 448 | 389 | 59 |
| 685-PTsup | TRHR | 22 | hg19 | chr8 | 110100305 | 110100305 | Nonsense_Mutation | SNP | C | C | G | novel | Somatic | Y188Ter | 0.068852 | NM_003301.6 | 610 | 568 | 42 |
| 685-PTsup | PLEC | 1691 | hg19 | chr8 | 144993013 | 144993013 | Missense_Mutation | SNP | G | G | C | novel | Somatic | S3796C | 0.10163 | NM_201380.4 | 1043 | 937 | 106 |
| 685-PTsup | SPTAN1 | 198 | hg19 | chr9 | 131383524 | 131383524 | Missense_Mutation | SNP | C | C | G | novel | Somatic | L1941V | 0.199095 | NM_001363759.2 | 221 | 177 | 44 |
| 685-PTsup | NUP214 | 200 | hg19 | chr9 | 134103627 | 134103627 | Missense_Mutation | SNP | G | G | A | novel | Somatic | G1995S | 0.275401 | NM_005085.4 | 374 | 271 | 103 |
| 685-PTsup | SURF2 | 1624 | hg19 | chr9 | 136226880 | 136226880 | In_Frame_Del | DEL | GGA | GGA | - | novel | Somatic | R135del | 0.025455 | NM_017503.5 | 549 | 535 | 14 |
| 685-PTsup | EXD3 | 1655 | hg19 | chr9 | 140247165 | 140247165 | Missense_Mutation | SNP | G | G | A | novel | Somatic | T315M | 0.183851 | NM_017820.5 | 804 | 656 | 148 |
| 685-PTsup | CACNB2 | 90 | hg19 | chr10 | 18690886 | 18690886 | Missense_Mutation | SNP | G | G | A | novel | Somatic | D83N | 0.454082 | NM_201596.3 | 195 | 106 | 89 |
| 685-PTsup | 8-Mar | 116 | hg19 | chr10 | 45958876 | 45958876 | Missense_Mutation | SNP | C | C | T | novel | Somatic | A271T | 0.216172 | NM_001282866.2 | 606 | 475 | 131 |
| 685-PTsup | DNA2 | 1120 | hg19 | chr10 | 70228005 | 70228005 | Missense_Mutation | SNP | C | C | G | novel | Somatic | D106H | 0.245734 | NM_001080449.3 | 291 | 219 | 72 |
| 685-PTsup | LIPJ | 1274 | hg19 | chr10 | 90366537 | 90366537 | Missense_Mutation | SNP | C | C | T | novel | Somatic | S325F | 0.230769 | NM_001010939.2 | 221 | 170 | 51 |
| 685-PTsup | CHUK | 170 | hg19 | chr10 | 101982628 | 101982628 | Nonsense_Mutation | SNP | G | G | A | novel | Somatic | R104Ter | 0.435065 | NM_001278.5 | 154 | 87 | 67 |
| 685-PTsup | LZTS2 | 1369 | hg19 | chr10 | 102763855 | 102763855 | Nonsense_Mutation | SNP | C | C | T | novel | Somatic | R334Ter | 0.241033 | NM_032429.4 | 696 | 528 | 168 |
| 685-PTsup | CFAP46 | 201 | hg19 | chr10 | 134738257 | 134738257 | Missense_Mutation | SNP | C | C | T | novel | Somatic | R400Q | 0.312609 | NM_001200049.3 | 1142 | 785 | 357 |
| 685-PTsup | AMBRA1 | 117 | hg19 | chr11 | 46539702 | 46539702 | Missense_Mutation | SNP | A | A | G | novel | Somatic | W693R | 0.036961 | NM_001267782.2 | 484 | 466 | 18 |
| 685-PTsup | CKAP5 | 117 | hg19 | chr11 | 46783636 | 46783636 | Missense_Mutation | SNP | C | C | A | novel | Somatic | D1379Y | 0.173575 | NM_001008938.4 | 386 | 319 | 67 |
| 685-PTsup | DTX4 | 1034 | hg19 | chr11 | 58962827 | 58962827 | Frame_Shift_Del | DEL | C | C | - | novel | Somatic | G510AfsTer35 | 0.021073 | NM_015177.2 | 520 | 509 | 11 |
| 685-PTsup | KMT5B | 1103 | hg19 | chr11 | 67925388 | 67925388 | Frame_Shift_Del | DEL | AGAG | AGAG | - | novel | Somatic | L808WfsTer50 | 0.168317 | NM_017635.5 | 303 | 252 | 51 |
| 685-PTsup | INPPL1 | 1133 | hg19 | chr11 | 71948767 | 71948767 | Missense_Mutation | SNP | C | C | T | novel | Somatic | S1160L | 0.236025 | NM_001567.4 | 966 | 738 | 228 |
| 685-PTsup | CCDC89 | 1236 | hg19 | chr11 | 85396056 | 85396056 | Missense_Mutation | SNP | G | G | C | novel | Somatic | S373C | 0.282443 | NM_152723.3 | 261 | 187 | 74 |
| 685-PTsup | CNTN5 | 20 | hg19 | chr11 | 100061859 | 100061859 | Missense_Mutation | SNP | A | A | T | novel | Somatic | I528L | 0.229885 | NM_014361.4 | 174 | 134 | 40 |
| 685-PTsup | PDGFD | 21 | hg19 | chr11 | 103797672 | 103797672 | Missense_Mutation | SNP | T | T | C | novel | Somatic | N319D | 0.223684 | NM_025208.5 | 304 | 236 | 68 |
| 685-PTsup | ARHGAP32 | 195 | hg19 | chr11 | 128844234 | 128844234 | Missense_Mutation | SNP | C | C | G | novel | Somatic | W939S | 0.201613 | NM_001142685.1 | 372 | 297 | 75 |
| 685-PTsup | DCP1B | 601 | hg19 | chr12 | 2074768 | 2074768 | Missense_Mutation | SNP | C | C | T | novel | Somatic | D160N | 0.227621 | NM_152640.5 | 390 | 301 | 89 |
| 685-PTsup | PEX5 | 641 | hg19 | chr12 | 7361148 | 7361148 | Missense_Mutation | SNP | T | T | G | novel | Somatic | L447R | 0.294606 | NM_001300789.1 | 239 | 168 | 71 |
| 685-PTsup | LRP6 | 84 | hg19 | chr12 | 12315157 | 12315157 | Missense_Mutation | SNP | G | G | T | novel | Somatic | P750H | 0.272727 | NM_002336.3 | 307 | 223 | 84 |
| 685-PTsup | MUC19 | 13 | hg19 | chr12 | 40835680 | 40835680 | Nonsense_Mutation | SNP | C | C | A | novel | Somatic | C1260Ter | 0.215753 | NM_173600.2 | 291 | 228 | 63 |
| 685-PTsup | PUS7L | 921 | hg19 | chr12 | 44139908 | 44139908 | Frame_Shift_Ins | INS | - | - | T | novel | Somatic | Q402TfsTer4 | 0.182432 | NM_001098615.2 | 148 | 121 | 27 |
| 685-PTsup | MARS | 1026 | hg19 | chr12 | 57909000 | 57909000 | Missense_Mutation | SNP | C | C | G | novel | Somatic | P758A | 0.260188 | NM_004990.4 | 318 | 235 | 83 |
| 685-PTsup | DCTN2 | 128 | hg19 | chr12 | 57928842 | 57928842 | Frame_Shift_Del | DEL | G | G | - | novel | Somatic | K166RfsTer4 | 0.196429 | NM_001348065.1 | 224 | 180 | 44 |
| 685-PTsup | KIF5A | 1027 | hg19 | chr12 | 57969045 | 57969045 | Missense_Mutation | SNP | T | T | G | novel | Somatic | L632R | 0.227468 | NM_004984.4 | 233 | 180 | 53 |
| 685-PTsup | DYRK2 | 1104 | hg19 | chr12 | 68043649 | 68043649 | Missense_Mutation | SNP | G | G | C | novel | Somatic | G41A | 0.216695 | NM_006482.3 | 1185 | 928 | 257 |
| 685-PTsup | MYRFL | 17 | hg19 | chr12 | 70321441 | 70321441 | Intron | SNP | A | A | C | novel | Somatic |  | 0.342466 | NM_182530.3 | 146 | 96 | 50 |
| 685-PTsup | AMER2 | 781 | hg19 | chr13 | 25744182 | 25744182 | Missense_Mutation | SNP | C | C | A | novel | Somatic | D526Y | 0.392857 | NM_152704.3 | 978 | 593 | 385 |
| 685-PTsup | COL4A1 | 178 | hg19 | chr13 | 110844637 | 110844637 | Splice_Region | SNP | G | G | C | novel | Somatic |  | 0.614458 | NM_001845.6 | 332 | 128 | 204 |
| 685-PTsup | CARMIL3 | 772 | hg19 | chr14 | 24538366 | 24538366 | Missense_Mutation | SNP | C | C | T | novel | Somatic | R1335W | 0.214477 | NM_138360.4 | 372 | 292 | 80 |
| 685-PTsup | FSCB | 928 | hg19 | chr14 | 44973945 | 44973945 | Missense_Mutation | SNP | T | T | C | novel | Somatic | D749G | 0.190299 | NM_032135.4 | 268 | 217 | 51 |
| 685-PTsup | FANCM | 116 | hg19 | chr14 | 45639896 | 45639896 | Missense_Mutation | SNP | A | A | G | novel | Somatic | I703V | 0.22807 | NM_020937.4 | 228 | 176 | 52 |
| 685-PTsup | RPL36AL | 967 | hg19 | chr14 | 50085549 | 50085549 | Missense_Mutation | SNP | C | C | T | novel | Somatic | E92K | 0.191011 | NM_001001.5 | 356 | 288 | 68 |
| 685-PTsup | MTHFD1 | 134 | hg19 | chr14 | 64892478 | 64892478 | Missense_Mutation | SNP | G | G | A | novel | Somatic | E296K | 0.303571 | NM_005956.4 | 335 | 233 | 102 |
| 685-PTsup | SRSF5 | 1120 | hg19 | chr14 | 70237949 | 70237949 | Missense_Mutation | SNP | C | C | T | novel | Somatic | S197F | 0.326606 | NM_001039465.2 | 545 | 367 | 178 |
| 685-PTsup | PROX2 | 1159 | hg19 | chr14 | 75329708 | 75329708 | Missense_Mutation | SNP | C | C | T | novel | Somatic | G277E | 0.293532 | NM_001243007.1 | 402 | 284 | 118 |
| 685-PTsup | VIPAS39 | 1179 | hg19 | chr14 | 77895366 | 77895366 | Missense_Mutation | SNP | G | G | A | novel | Somatic | H447Y | 0.152334 | NM_001193314.2 | 406 | 344 | 62 |
| 685-PTsup | ISM2 | 1179 | hg19 | chr14 | 77942267 | 77942267 | Missense_Mutation | SNP | C | C | T | novel | Somatic | E463K | 0.316583 | NM_199296.3 | 1193 | 815 | 378 |
| 685-PTsup | BTBD7 | 1300 | hg19 | chr14 | 93708879 | 93708879 | Missense_Mutation | SNP | C | C | T | novel | Somatic | A1047T | 0.288828 | NM_001002860.4 | 367 | 261 | 106 |
| 685-PTsup | GABRA5 | 98 | hg19 | chr15 | 27193183 | 27193183 | Missense_Mutation | SNP | T | T | C | novel | Somatic | S398P | 0.277344 | NM_000810.4 | 256 | 185 | 71 |
| 685-PTsup | PPIP5K1 | 919 | hg19 | chr15 | 43827140 | 43827140 | Missense_Mutation | DNP | TC | TC | AA | novel | Somatic | E1345L | 0.192308 | NM_001130858.2 | 336 | 271 | 65 |
| 685-PTsup | RFX7 | 126 | hg19 | chr15 | 56387789 | 56387789 | Nonsense_Mutation | SNP | G | G | A | novel | Somatic | Q713Ter | 0.276515 | NM_001370561.1 | 264 | 191 | 73 |
| 685-PTsup | TMEM266 | 145 | hg19 | chr15 | 76494606 | 76494606 | Splice_Site | SNP | G | G | T | novel | Somatic |  | 0.212766 | NM_152335.3 | 235 | 185 | 50 |
| 685-PTsup | PEAK1 | 18 | hg19 | chr15 | 77473084 | 77473084 | Missense_Mutation | SNP | C | C | A | novel | Somatic | Q395H | 0.184 | NM_024776.4 | 374 | 305 | 69 |
| 685-PTsup | NR2F2 | 1324 | hg19 | chr15 | 96880718 | 96880718 | Missense_Mutation | SNP | G | G | A | novel | Somatic | R371H | 0.220779 | NM_021005.4 | 461 | 359 | 102 |
| 685-PTsup | JMJD8 | 590 | hg19 | chr16 | 734114 | 734114 | Missense_Mutation | SNP | C | C | T | novel | Somatic | C43Y | 0.041929 | NM_001005920.4 | 477 | 457 | 20 |
| 685-PTsup | ZP2 | 746 | hg19 | chr16 | 21218254 | 21218254 | Missense_Mutation | SNP | C | C | T | novel | Somatic | G130R | 0.280236 | NM_003460.2 | 337 | 242 | 95 |
| 685-PTsup | CDH11 | 16 | hg19 | chr16 | 64981858 | 64981858 | Missense_Mutation | SNP | G | G | T | novel | Somatic | T680N | 0.270742 | NM_001797.4 | 229 | 167 | 62 |
| 685-PTsup | CBFB | 0 | hg19 | chr16 | 67070641 | 67070641 | Nonsense_Mutation | SNP | G | G | T | novel | Somatic | E89Ter | 0.509804 | NM_022845.3 | 204 | 100 | 104 |
| 685-PTsup | PLEKHG4 | 1098 | hg19 | chr16 | 67318167 | 67318167 | Intron | DEL | TG | TG | - | novel | Somatic |  | 0.242308 | NM_001129727.3 | 260 | 197 | 63 |
| 685-PTsup | HYDIN | 140 | hg19 | chr16 | 71103285 | 71103285 | Missense_Mutation | SNP | A | A | G | novel | Somatic | V620A | 0.26383 | NM_001270974.2 | 235 | 173 | 62 |
| 685-PTsup | TP53 | 642 | hg19 | chr17 | 7576897 | 7576897 | Nonsense_Mutation | SNP | G | G | A | novel | Somatic | Q317Ter | 0.265734 | NM_000546.5 | 285 | 209 | 76 |
| 685-PTsup | PIK3R6 | 651 | hg19 | chr17 | 8731962 | 8731962 | Missense_Mutation | SNP | C | C | A | novel | Somatic | R412L | 0.300116 | NM_001010855.4 | 860 | 601 | 259 |
| 685-PTsup | UBB | 709 | hg19 | chr17 | 16285891 | 16285891 | Missense_Mutation | SNP | C | C | T | novel | Somatic | R224C | 0.344262 | NM_018955.4 | 426 | 279 | 147 |
| 685-PTsup | KPNB1 | 116 | hg19 | chr17 | 45734425 | 45734425 | Missense_Mutation | SNP | T | T | C | novel | Somatic | I161T | 0.195219 | NM_002265.6 | 251 | 202 | 49 |
| 685-PTsup | CA10 | 120 | hg19 | chr17 | 49825102 | 49825102 | Missense_Mutation | SNP | C | C | G | novel | Somatic | G119A | 0.07326 | NM_020178.5 | 272 | 252 | 20 |
| 685-PTsup | GPR142 | 1137 | hg19 | chr17 | 72368179 | 72368179 | Missense_Mutation | SNP | C | C | T | novel | Somatic | R277W | 0.161947 | NM_181790.1 | 1130 | 947 | 183 |
| 685-PTsup | DNAH17 | 18 | hg19 | chr17 | 76464789 | 76464789 | In_Frame_Del | DEL | GGA | GGA | - | novel | Somatic | S2896del | 0.180693 | NM_173628.4 | 404 | 331 | 73 |
| 685-PTsup | LRRC45 | 1195 | hg19 | chr17 | 79987021 | 79987021 | Missense_Mutation | SNP | G | G | A | novel | Somatic | R454Q | 0.173403 | NM_144999.4 | 766 | 633 | 133 |
| 685-PTsup | CSNK1D | 149 | hg19 | chr17 | 80210978 | 80210978 | Missense_Mutation | SNP | C | C | T | novel | Somatic | R160H | 0.385965 | NM_001363749.2 | 627 | 385 | 242 |
| 685-PTsup | LMAN1 | 127 | hg19 | chr18 | 57022603 | 57022603 | Missense_Mutation | SNP | G | G | A | novel | Somatic | S140L | 0.061033 | NM_005570.4 | 213 | 200 | 13 |
| 685-PTsup | SALL3 | 1170 | hg19 | chr18 | 76752521 | 76752521 | Missense_Mutation | SNP | C | C | T | novel | Somatic | A177V | 0.021954 | NM_171999.3 | 910 | 890 | 20 |
| 685-PTsup | ATCAY | 614 | hg19 | chr19 | 3909529 | 3909529 | Missense_Mutation | SNP | G | G | A | novel | Somatic | M231I | 0.167488 | NM_033064.5 | 406 | 338 | 68 |
| 685-PTsup | CYP4F12 | 705 | hg19 | chr19 | 15793235 | 15793235 | Missense_Mutation | DNP | TG | TG | CA | novel | Somatic | R188H | 0.247911 | NM_023944.4 | 89 | 0 | 89 |
| 685-PTsup | NXNL1 | 719 | hg19 | chr19 | 17566520 | 17566520 | Missense_Mutation | SNP | G | G | A | novel | Somatic | A192V | 0.221176 | NM_138454.2 | 834 | 646 | 188 |
| 685-PTsup | GGN | 881 | hg19 | chr19 | 38876137 | 38876137 | Missense_Mutation | SNP | G | G | A | novel | Somatic | L589F | 0.255587 | NM_152657.4 | 714 | 531 | 183 |
| 685-PTsup | DYRK1B | 892 | hg19 | chr19 | 40321074 | 40321074 | Missense_Mutation | SNP | C | C | T | novel | Somatic | E105K | 0.233236 | NM_004714.3 | 343 | 263 | 80 |
| 685-PTsup | GRIN2D | 958 | hg19 | chr19 | 48946238 | 48946238 | Missense_Mutation | SNP | C | C | T | novel | Somatic | P1019S | 0.23166 | NM_000836.2 | 259 | 199 | 60 |
| 685-PTsup | GRIN2D | 958 | hg19 | chr19 | 48946554 | 48946554 | Missense_Mutation | SNP | C | C | T | novel | Somatic | A1124V | 0.247078 | NM_000836.2 | 597 | 449 | 148 |
| 685-PTsup | OTOR | 712 | hg19 | chr20 | 16729605 | 16729605 | Nonsense_Mutation | SNP | C | C | G | novel | Somatic | S70Ter | 0.271028 | NM_020157.3 | 214 | 156 | 58 |
| 685-PTsup | NKX2-2 | 748 | hg19 | chr20 | 21492817 | 21492817 | Missense_Mutation | SNP | T | T | A | novel | Somatic | E189V | 0.224691 | NM_002509.4 | 810 | 628 | 182 |
| 685-PTsup | TM9SF4 | 819 | hg19 | chr20 | 30753177 | 30753177 | Missense_Mutation | SNP | C | C | T | novel | Somatic | T620M | 0.179039 | NM_014742.4 | 458 | 376 | 82 |
| 685-PTsup | ASXL1 | 821 | hg19 | chr20 | 31022982 | 31022982 | Frame_Shift_Ins | INS | - | - | TAGA | novel | Somatic | E824DfsTer10 | 0.447433 | NM_015338.6 | 405 | 222 | 183 |
| 685-PTsup | SUN5 | 103 | hg19 | chr20 | 31589024 | 31589024 | Missense_Mutation | SNP | G | G | C | novel | Somatic | T91R | 0.169435 | NM_080675.4 | 600 | 498 | 102 |
| 685-PTsup | PMEPA1 | 126 | hg19 | chr20 | 56284608 | 56284608 | Missense_Mutation | SNP | C | C | T | novel | Somatic | A11T | 0.020231 | NM_020182.5 | 668 | 654 | 14 |
| 685-PTsup | LIPI | 703 | hg19 | chr21 | 15538701 | 15538701 | Missense_Mutation | SNP | G | G | C | novel | Somatic | P260A | 0.331034 | NM_198996.3 | 145 | 97 | 48 |
| 685-PTsup | COL6A1 | 946 | hg19 | chr21 | 47404280 | 47404280 | Missense_Mutation | SNP | G | G | A | novel | Somatic | G109S | 0.3906 | NM_001848.3 | 617 | 376 | 241 |
| 685-PTsup | CACNA1I | 111 | hg19 | chr22 | 40054959 | 40054959 | Missense_Mutation | SNP | C | C | G | novel | Somatic | A723G | 0.178838 | NM_021096.4 | 671 | 551 | 120 |
| 685-PTsup | TUBGCP6 | 971 | hg19 | chr22 | 50671810 | 50671810 | Missense_Mutation | SNP | C | C | T | novel | Somatic | E351K | 0.202736 | NM_020461.4 | 804 | 641 | 163 |
| 685-PTsup | FAM9A | 651 | hg19 | chrX | 8764319 | 8764319 | Frame_Shift_Ins | INS | - | - | T | novel | Somatic | Q160TfsTer8 | 0.070093 | NM_174951.3 | 212 | 197 | 15 |
| 685-PTsup | GRPR | 708 | hg19 | chrX | 16142299 | 16142299 | Missense_Mutation | SNP | C | C | A | novel | Somatic | P75T | 0.282895 | NM_005314.3 | 152 | 109 | 43 |
| 685-PTdeep | CAMTA1 | 644 | hg19 | chr1 | 7723734 | 7723734 | Missense_Mutation | SNP | C | C | T | novel | Somatic | P376L | 0.151822 | NM_015215.4 | 493 | 418 | 75 |
| 685-PTdeep | COL16A1 | 830 | hg19 | chr1 | 32127969 | 32127969 | Missense_Mutation | SNP | C | C | T | novel | Somatic | G1216D | 0.147518 | NM_001856.4 | 705 | 601 | 104 |
| 685-PTdeep | COL9A2 | 896 | hg19 | chr1 | 40767555 | 40767555 | Missense_Mutation | SNP | C | C | T | novel | Somatic | R600H | 0.193277 | NM_001852.4 | 118 | 95 | 23 |
| 685-PTdeep | TOE1 | 934 | hg19 | chr1 | 45806886 | 45806886 | Missense_Mutation | SNP | C | C | T | novel | Somatic | T65M | 0.14497 | NM_025077.4 | 337 | 288 | 49 |
| 685-PTdeep | FNDC7 | 1418 | hg19 | chr1 | 109276138 | 109276138 | Frame_Shift_Del | DEL | A | A | - | novel | Somatic | I711YfsTer4 | 0.051643 | NM_001144937.3 | 213 | 202 | 11 |
| 685-PTdeep | SYPL2 | 1424 | hg19 | chr1 | 110009654 | 110009654 | Intron | DEL | A | A | - | novel | Somatic |  | 0.048632 | NM_001040709.2 | 329 | 313 | 16 |
| 685-PTdeep | FLAD1 | 1767 | hg19 | chr1 | 154962868 | 154962868 | Missense_Mutation | SNP | A | A | G | novel | Somatic | E473G | 0.14 | NM_025207.5 | 199 | 171 | 28 |
| 685-PTdeep | CD1E | 1792 | hg19 | chr1 | 158325670 | 158325670 | Missense_Mutation | SNP | C | C | A | novel | Somatic | Q227K | 0.105159 | NM_030893.4 | 499 | 446 | 53 |
| 685-PTdeep | RALGPS2 | 243 | hg19 | chr1 | 178777229 | 178777229 | Missense_Mutation | SNP | C | C | A | novel | Somatic | P88Q | 0.168776 | NM_152663.5 | 237 | 197 | 40 |
| 685-PTdeep | KIF21B | 2118 | hg19 | chr1 | 200965373 | 200965373 | Missense_Mutation | SNP | C | C | T | novel | Somatic | R743K | 0.155779 | NM_001252100.2 | 199 | 168 | 31 |
| 685-PTdeep | USH2A | 279 | hg19 | chr1 | 216420019 | 216420019 | Missense_Mutation | SNP | C | C | A | novel | Somatic | G906V | 0.12766 | NM_206933.3 | 375 | 327 | 48 |
| 685-PTdeep | USP34 | 131 | hg19 | chr2 | 61570965 | 61570965 | Missense_Mutation | SNP | C | C | T | novel | Somatic | E829K | 0.442568 | NM_014709.4 | 296 | 165 | 131 |
| 685-PTdeep | FIGLA | 1126 | hg19 | chr2 | 71017734 | 71017734 | Missense_Mutation | SNP | C | C | T | novel | Somatic | A13T | 0.446108 | NM_001004311.3 | 646 | 348 | 298 |
| 685-PTdeep | TGOLN2 | 1237 | hg19 | chr2 | 85554654 | 85554654 | Frame_Shift_Ins | INS | - | - | G | novel | Somatic | S68Ter | 0.280864 | NM_006464.4 | 321 | 230 | 91 |
| 685-PTdeep | REV1 | 1348 | hg19 | chr2 | 100017796 | 100017796 | In_Frame_Del | DEL | CCA | CCA | - | novel | Somatic | V1221del | 0.158192 | NM_016316.4 | 174 | 146 | 28 |
| 685-PTdeep | DPP10 | 22 | hg19 | chr2 | 116539930 | 116539930 | Missense_Mutation | SNP | C | C | A | novel | Somatic | P513T | 0.186235 | NM_001321905.2 | 244 | 198 | 46 |
| 685-PTdeep | PRPF40A | 1756 | hg19 | chr2 | 153526794 | 153526794 | Missense_Mutation | SNP | C | C | G | novel | Somatic | Q569H | 0.142349 | NM_001365596.2 | 280 | 240 | 40 |
| 685-PTdeep | TTN | 1955 | hg19 | chr2 | 179481856 | 179481856 | Missense_Mutation | SNP | C | C | T | novel | Somatic | D15956N | 0.055666 | NM_001267550.2 | 502 | 474 | 28 |
| 685-PTdeep | CPS1 | 2198 | hg19 | chr2 | 211473152 | 211473152 | Missense_Mutation | SNP | G | G | A | novel | Somatic | V754I | 0.13242 | NM_001875.5 | 219 | 190 | 29 |
| 685-PTdeep | CNTN6 | 74 | hg19 | chr3 | 1445041 | 1445041 | Missense_Mutation | SNP | C | C | A | novel | Somatic | T1009N | 0.142857 | NM_001349351.2 | 140 | 120 | 20 |
| 685-PTdeep | SRGAP3 | 81 | hg19 | chr3 | 9074383 | 9074383 | Missense_Mutation | SNP | G | G | A | novel | Somatic | S497L | 0.303725 | NM_014850.4 | 349 | 243 | 106 |
| 685-PTdeep | FYCO1 | 116 | hg19 | chr3 | 46009433 | 46009433 | Missense_Mutation | SNP | C | C | T | novel | Somatic | E465K | 0.144628 | NM_024513.4 | 241 | 206 | 35 |
| 685-PTdeep | CSNKA2IP | Unknown | hg19 | chr3 | 88515123 | 88515123 | Frame_Shift_Del | DEL | C | C | - | novel | Somatic | K207NfsTer35 | 0.108696 | NM_001368165.1 | 230 | 205 | 25 |
| 685-PTdeep | MORC1 | 176 | hg19 | chr3 | 108723661 | 108723661 | Splice_Region | SNP | C | C | T | novel | Somatic |  | 0.068441 | NM_014429.4 | 263 | 245 | 18 |
| 685-PTdeep | C3orf52 | 1438 | hg19 | chr3 | 111835488 | 111835488 | Splice_Site | SNP | G | G | A | novel | Somatic |  | 0.114894 | NM_024616.2 | 235 | 208 | 27 |
| 685-PTdeep | XRN1 | 1669 | hg19 | chr3 | 142122569 | 142122569 | Missense_Mutation | SNP | G | G | C | novel | Somatic | R684G | 0.083077 | NM_019001.5 | 324 | 297 | 27 |
| 685-PTdeep | PHC3 | 29 | hg19 | chr3 | 169815155 | 169815155 | Missense_Mutation | SNP | C | C | T | novel | Somatic | D951N | 0.225444 | NM_024947.4 | 1069 | 828 | 241 |
| 685-PTdeep | TNIK | 1890 | hg19 | chr3 | 170802067 | 170802067 | Nonsense_Mutation | SNP | C | C | A | novel | Somatic | E1016Ter | 0.71401 | NM_015028.4 | 1553 | 442 | 1111 |
| 685-PTdeep | FNDC3B | 29 | hg19 | chr3 | 172115023 | 172115023 | Missense_Mutation | SNP | G | G | A | novel | Somatic | V1125I | 0.185393 | NM_022763.4 | 178 | 145 | 33 |
| 685-PTdeep | ZNF639 | 243 | hg19 | chr3 | 179051897 | 179051897 | Missense_Mutation | SNP | G | G | A | novel | Somatic | R382Q | 0.232843 | NM_001303425.2 | 406 | 311 | 95 |
| 685-PTdeep | PEX5L | 244 | hg19 | chr3 | 179533791 | 179533791 | Missense_Mutation | SNP | C | C | G | novel | Somatic | G314A | 0.130112 | NM_016559.3 | 268 | 233 | 35 |
| 685-PTdeep | MAP6D1 | 1985 | hg19 | chr3 | 183543193 | 183543193 | Missense_Mutation | SNP | G | G | A | novel | Somatic | S48L | 0.166511 | NM_024871.3 | 1064 | 886 | 178 |
| 685-PTdeep | KNG1 | 2007 | hg19 | chr3 | 186442948 | 186442948 | Missense_Mutation | SNP | G | G | A | novel | Somatic | D155N | 0.098291 | NM_001102416.3 | 233 | 210 | 23 |
| 685-PTdeep | GUF1 | 115 | hg19 | chr4 | 44688546 | 44688546 | Missense_Mutation | SNP | A | A | G | novel | Somatic | N252D | 0.201613 | NM_021927.3 | 124 | 99 | 25 |
| 685-PTdeep | ADGRL3 | 16 | hg19 | chr4 | 62812686 | 62812686 | Missense_Mutation | SNP | C | C | T | novel | Somatic | T825M | 0.164948 | NM_001322402.2 | 192 | 160 | 32 |
| 685-PTdeep | ADAMTS3 | 17 | hg19 | chr4 | 73414436 | 73414436 | Missense_Mutation | SNP | C | C | A | novel | Somatic | G88V | 0.156522 | NM_014243.3 | 230 | 194 | 36 |
| 685-PTdeep | WDFY3 | 154 | hg19 | chr4 | 85672857 | 85672857 | Missense_Mutation | SNP | C | C | T | novel | Somatic | D1918N | 0.227979 | NM_014991.5 | 192 | 148 | 44 |
| 685-PTdeep | DCTD | 1987 | hg19 | chr4 | 183814272 | 183814272 | Nonsense_Mutation | SNP | C | C | A | novel | Somatic | E135Ter | 0.162791 | NM_001012732.1 | 128 | 107 | 21 |
| 685-PTdeep | ADCY2 | 80 | hg19 | chr5 | 7709500 | 7709500 | Splice_Site | DNP | GG | GG | TT | novel | Somatic |  | 0.071856 | NM_020546.3 | 333 | 309 | 24 |
| 685-PTdeep | FGF10 | 923 | hg19 | chr5 | 44310530 | 44310530 | Missense_Mutation | SNP | G | G | A | novel | Somatic | S143L | 0.084848 | NM_004465.2 | 165 | 151 | 14 |
| 685-PTdeep | POC5 | 144 | hg19 | chr5 | 74998445 | 74998445 | Missense_Mutation | SNP | C | C | G | novel | Somatic | W166C | 0.184211 | NM_001099271.2 | 227 | 185 | 42 |
| 685-PTdeep | MTRNR2L2 | 1194 | hg19 | chr5 | 79945871 | 79945871 | Missense_Mutation | SNP | G | G | A | novel | Somatic | S12L | 0.073077 | NM_001190470.2 | 260 | 241 | 19 |
| 685-PTdeep | NUDT12 | 171 | hg19 | chr5 | 102886645 | 102886645 | Nonsense_Mutation | SNP | G | G | A | novel | Somatic | Q436Ter | 0.177665 | NM_031438.4 | 196 | 161 | 35 |
| 685-PTdeep | CXCL14 | 1614 | hg19 | chr5 | 134907551 | 134907551 | Missense_Mutation | SNP | C | C | T | novel | Somatic | E98K | 0.232558 | NM_004887.5 | 128 | 98 | 30 |
| 685-PTdeep | FAM193B | 241 | hg19 | chr5 | 176965990 | 176965990 | Silent | SNP | G | G | A | novel | Somatic | G123= | 0.17284 | NM_001190946.3 | 242 | 200 | 42 |
| 685-PTdeep | IP6K3 | 842 | hg19 | chr6 | 33694507 | 33694507 | Splice_Site | SNP | C | C | T | novel | Somatic |  | 0.031579 | NM_054111.5 | 378 | 366 | 12 |
| 685-PTdeep | ENPP1 | 1593 | hg19 | chr6 | 132198274 | 132198274 | Missense_Mutation | SNP | T | T | A | novel | Somatic | D622E | 0.158879 | NM_006208.3 | 321 | 270 | 51 |
| 685-PTdeep | TBPL1 | 1609 | hg19 | chr6 | 134303713 | 134303713 | Nonsense_Mutation | SNP | T | T | G | novel | Somatic | L48Ter | 0.168498 | NM_004865.4 | 273 | 227 | 46 |
| 685-PTdeep | MAP7 | 203 | hg19 | chr6 | 136704883 | 136704883 | Missense_Mutation | SNP | G | G | A | novel | Somatic | S210L | 0.198198 | NM_001198609.1 | 111 | 89 | 22 |
| 685-PTdeep | PCMT1 | 216 | hg19 | chr6 | 150123393 | 150123393 | Missense_Mutation | SNP | G | G | A | novel | Somatic | G246R | 0.171429 | NM_001252049.1 | 209 | 173 | 36 |
| 685-PTdeep | SUN1 | 591 | hg19 | chr7 | 905651 | 905651 | Missense_Mutation | SNP | C | C | G | novel | Somatic | L781V | 0.174041 | NM_001367651.1 | 338 | 279 | 59 |
| 685-PTdeep | SLC29A4 | 625 | hg19 | chr7 | 5334535 | 5334535 | Missense_Mutation | SNP | C | C | T | novel | Somatic | R197W | 0.109718 | NM_001040661.2 | 319 | 284 | 35 |
| 685-PTdeep | NPC1L1 | 925 | hg19 | chr7 | 44556890 | 44556890 | Missense_Mutation | SNP | G | G | T | novel | Somatic | A1095E | 0.252788 | NM_013389.3 | 266 | 198 | 68 |
| 685-PTdeep | SRRM3 | 145 | hg19 | chr7 | 75890695 | 75890695 | In_Frame_Del | DEL | AAG | AAG | - | novel | Somatic | K204del | 0.025575 | NM_001110199.3 | 388 | 378 | 10 |
| 685-PTdeep | SRRT | 1351 | hg19 | chr7 | 100484957 | 100484957 | Missense_Mutation | SNP | G | G | C | novel | Somatic | K664N | 0.146018 | NM_015908.6 | 226 | 193 | 33 |
| 685-PTdeep | ARFGEF1 | 17 | hg19 | chr8 | 68170429 | 68170429 | Missense_Mutation | SNP | C | C | T | novel | Somatic | V778I | 0.157343 | NM_006421.5 | 284 | 239 | 45 |
| 685-PTdeep | OSR2 | 1347 | hg19 | chr8 | 99961377 | 99961377 | Missense_Mutation | SNP | G | G | A | novel | Somatic | R187H | 0.16289 | NM_001286841.1 | 1405 | 1175 | 230 |
| 685-PTdeep | RIMS2 | 173 | hg19 | chr8 | 105105860 | 105105860 | Missense_Mutation | SNP | C | C | G | novel | Somatic | N1285K | 0.099174 | NM_001348484.2 | 363 | 327 | 36 |
| 685-PTdeep | PLEC | 1691 | hg19 | chr8 | 144993013 | 144993013 | Missense_Mutation | SNP | G | G | C | novel | Somatic | S3796C | 0.100116 | NM_201380.4 | 858 | 772 | 86 |
| 685-PTdeep | AQP7 | 839 | hg19 | chr9 | 33386510 | 33386510 | Missense_Mutation | SNP | C | C | T | novel | Somatic | A100T | 0.024831 | NM_001170.3 | 267 | 256 | 11 |
| 685-PTdeep | SPTAN1 | 198 | hg19 | chr9 | 131383524 | 131383524 | Missense_Mutation | SNP | C | C | G | novel | Somatic | L1941V | 0.153226 | NM_001363759.2 | 124 | 105 | 19 |
| 685-PTdeep | NUP214 | 200 | hg19 | chr9 | 134103627 | 134103627 | Missense_Mutation | SNP | G | G | A | novel | Somatic | G1995S | 0.16 | NM_005085.4 | 273 | 229 | 44 |
| 685-PTdeep | SURF2 | 1624 | hg19 | chr9 | 136226880 | 136226880 | In_Frame_Del | DEL | GGA | GGA | - | novel | Somatic | R135del | 0.036082 | NM_017503.5 | 387 | 373 | 14 |
| 685-PTdeep | EXD3 | 1655 | hg19 | chr9 | 140247165 | 140247165 | Missense_Mutation | SNP | G | G | A | novel | Somatic | T315M | 0.150097 | NM_017820.5 | 1022 | 868 | 154 |
| 685-PTdeep | CACNB2 | 90 | hg19 | chr10 | 18690886 | 18690886 | Missense_Mutation | SNP | G | G | A | novel | Somatic | D83N | 0.335227 | NM_201596.3 | 176 | 117 | 59 |
| 685-PTdeep | 8-Mar | 116 | hg19 | chr10 | 45958876 | 45958876 | Missense_Mutation | SNP | C | C | T | novel | Somatic | A271T | 0.111702 | NM_001282866.2 | 373 | 331 | 42 |
| 685-PTdeep | DNA2 | 1120 | hg19 | chr10 | 70228005 | 70228005 | Missense_Mutation | SNP | C | C | G | novel | Somatic | D106H | 0.178322 | NM_001080449.3 | 286 | 235 | 51 |
| 685-PTdeep | LIPJ | 1274 | hg19 | chr10 | 90366537 | 90366537 | Missense_Mutation | SNP | C | C | T | novel | Somatic | S325F | 0.10828 | NM_001010939.2 | 157 | 140 | 17 |
| 685-PTdeep | CHUK | 170 | hg19 | chr10 | 101982628 | 101982628 | Nonsense_Mutation | SNP | G | G | A | novel | Somatic | R104Ter | 0.376712 | NM_001278.5 | 145 | 90 | 55 |
| 685-PTdeep | LZTS2 | 1369 | hg19 | chr10 | 102763855 | 102763855 | Nonsense_Mutation | SNP | C | C | T | novel | Somatic | R334Ter | 0.152395 | NM_032429.4 | 688 | 583 | 105 |
| 685-PTdeep | CFAP46 | 201 | hg19 | chr10 | 134738257 | 134738257 | Missense_Mutation | SNP | C | C | T | novel | Somatic | R400Q | 0.170616 | NM_001200049.3 | 1265 | 1049 | 216 |
| 685-PTdeep | CKAP5 | 117 | hg19 | chr11 | 46783636 | 46783636 | Missense_Mutation | SNP | C | C | A | novel | Somatic | D1379Y | 0.128755 | NM_001008938.4 | 233 | 203 | 30 |
| 685-PTdeep | KMT5B | 1103 | hg19 | chr11 | 67925388 | 67925388 | Frame_Shift_Del | DEL | AGAG | AGAG | - | novel | Somatic | L808WfsTer50 | 0.13253 | NM_017635.5 | 249 | 216 | 33 |
| 685-PTdeep | INPPL1 | 1133 | hg19 | chr11 | 71948767 | 71948767 | Missense_Mutation | SNP | C | C | T | novel | Somatic | S1160L | 0.155086 | NM_001567.4 | 992 | 838 | 154 |
| 685-PTdeep | CCDC89 | 1236 | hg19 | chr11 | 85396056 | 85396056 | Missense_Mutation | SNP | G | G | C | novel | Somatic | S373C | 0.180328 | NM_152723.3 | 183 | 150 | 33 |
| 685-PTdeep | CNTN5 | 20 | hg19 | chr11 | 100061859 | 100061859 | Missense_Mutation | SNP | A | A | T | novel | Somatic | I528L | 0.155172 | NM_014361.4 | 116 | 98 | 18 |
| 685-PTdeep | PDGFD | 21 | hg19 | chr11 | 103797672 | 103797672 | Missense_Mutation | SNP | T | T | C | novel | Somatic | N319D | 0.147208 | NM_025208.5 | 197 | 168 | 29 |
| 685-PTdeep | ARHGAP32 | 195 | hg19 | chr11 | 128844234 | 128844234 | Missense_Mutation | SNP | C | C | G | novel | Somatic | W939S | 0.178218 | NM_001142685.1 | 303 | 249 | 54 |
| 685-PTdeep | DCP1B | 601 | hg19 | chr12 | 2074768 | 2074768 | Missense_Mutation | SNP | C | C | T | novel | Somatic | D160N | 0.148594 | NM_152640.5 | 248 | 211 | 37 |
| 685-PTdeep | PEX5 | 641 | hg19 | chr12 | 7361148 | 7361148 | Missense_Mutation | SNP | T | T | G | novel | Somatic | L447R | 0.198758 | NM_001300789.1 | 158 | 126 | 32 |
| 685-PTdeep | LRP6 | 84 | hg19 | chr12 | 12315157 | 12315157 | Missense_Mutation | SNP | G | G | T | novel | Somatic | P750H | 0.157895 | NM_002336.3 | 171 | 144 | 27 |
| 685-PTdeep | MUC19 | 13 | hg19 | chr12 | 40835680 | 40835680 | Nonsense_Mutation | SNP | C | C | A | novel | Somatic | C1260Ter | 0.124138 | NM_173600.2 | 290 | 254 | 36 |
| 685-PTdeep | PUS7L | 921 | hg19 | chr12 | 44139908 | 44139908 | Frame_Shift_Ins | INS | - | - | T | novel | Somatic | Q402TfsTer4 | 0.204082 | NM_001098615.2 | 96 | 76 | 20 |
| 685-PTdeep | MARS | 1026 | hg19 | chr12 | 57909000 | 57909000 | Missense_Mutation | SNP | C | C | G | novel | Somatic | P758A | 0.190813 | NM_004990.4 | 282 | 228 | 54 |
| 685-PTdeep | DCTN2 | 128 | hg19 | chr12 | 57928842 | 57928842 | Frame_Shift_Del | DEL | G | G | - | novel | Somatic | K166RfsTer4 | 0.153846 | NM_001348065.1 | 195 | 165 | 30 |
| 685-PTdeep | KIF5A | 1027 | hg19 | chr12 | 57969045 | 57969045 | Missense_Mutation | SNP | T | T | G | novel | Somatic | L632R | 0.155556 | NM_004984.4 | 133 | 112 | 21 |
| 685-PTdeep | DYRK2 | 1104 | hg19 | chr12 | 68043649 | 68043649 | Missense_Mutation | SNP | G | G | C | novel | Somatic | G41A | 0.18047 | NM_006482.3 | 1616 | 1324 | 292 |
| 685-PTdeep | MYRFL | 17 | hg19 | chr12 | 70321441 | 70321441 | Intron | SNP | A | A | C | novel | Somatic |  | 0.175325 | NM_182530.3 | 154 | 127 | 27 |
| 685-PTdeep | AMER2 | 781 | hg19 | chr13 | 25744182 | 25744182 | Missense_Mutation | SNP | C | C | A | novel | Somatic | D526Y | 0.295583 | NM_152704.3 | 881 | 620 | 261 |
| 685-PTdeep | COL4A1 | 178 | hg19 | chr13 | 110844637 | 110844637 | Splice_Region | SNP | G | G | C | novel | Somatic |  | 0.431095 | NM_001845.6 | 283 | 161 | 122 |
| 685-PTdeep | CARMIL3 | 772 | hg19 | chr14 | 24538366 | 24538366 | Missense_Mutation | SNP | C | C | T | novel | Somatic | R1335W | 0.095057 | NM_138360.4 | 261 | 236 | 25 |
| 685-PTdeep | FSCB | 928 | hg19 | chr14 | 44973945 | 44973945 | Missense_Mutation | SNP | T | T | C | novel | Somatic | D749G | 0.104167 | NM_032135.4 | 144 | 129 | 15 |
| 685-PTdeep | FANCM | 116 | hg19 | chr14 | 45639896 | 45639896 | Missense_Mutation | SNP | A | A | G | novel | Somatic | I703V | 0.142012 | NM_020937.4 | 169 | 145 | 24 |
| 685-PTdeep | RPL36AL | 967 | hg19 | chr14 | 50085549 | 50085549 | Missense_Mutation | SNP | C | C | T | novel | Somatic | E92K | 0.092105 | NM_001001.5 | 228 | 207 | 21 |
| 685-PTdeep | MTHFD1 | 134 | hg19 | chr14 | 64892478 | 64892478 | Missense_Mutation | SNP | G | G | A | novel | Somatic | E296K | 0.25 | NM_005956.4 | 275 | 206 | 69 |
| 685-PTdeep | SRSF5 | 1120 | hg19 | chr14 | 70237949 | 70237949 | Missense_Mutation | SNP | C | C | T | novel | Somatic | S197F | 0.363409 | NM_001039465.2 | 398 | 253 | 145 |
| 685-PTdeep | PROX2 | 1159 | hg19 | chr14 | 75329708 | 75329708 | Missense_Mutation | SNP | C | C | T | novel | Somatic | G277E | 0.299539 | NM_001243007.1 | 215 | 150 | 65 |
| 685-PTdeep | VIPAS39 | 1179 | hg19 | chr14 | 77895366 | 77895366 | Missense_Mutation | SNP | G | G | A | novel | Somatic | H447Y | 0.105023 | NM_001193314.2 | 218 | 195 | 23 |
| 685-PTdeep | ISM2 | 1179 | hg19 | chr14 | 77942267 | 77942267 | Missense_Mutation | SNP | C | C | T | novel | Somatic | E463K | 0.252025 | NM_199296.3 | 1110 | 830 | 280 |
| 685-PTdeep | BTBD7 | 1300 | hg19 | chr14 | 93708879 | 93708879 | Missense_Mutation | SNP | C | C | T | novel | Somatic | A1047T | 0.181416 | NM_001002860.4 | 225 | 184 | 41 |
| 685-PTdeep | GABRA5 | 98 | hg19 | chr15 | 27193183 | 27193183 | Missense_Mutation | SNP | T | T | C | novel | Somatic | S398P | 0.124424 | NM_000810.4 | 212 | 185 | 27 |
| 685-PTdeep | PPIP5K1 | 919 | hg19 | chr15 | 43827140 | 43827140 | Missense_Mutation | DNP | TC | TC | AA | novel | Somatic | E1345L | 0.156863 | NM_001130858.2 | 203 | 171 | 32 |
| 685-PTdeep | RFX7 | 126 | hg19 | chr15 | 56387789 | 56387789 | Nonsense_Mutation | SNP | G | G | A | novel | Somatic | Q713Ter | 0.213018 | NM_001370561.1 | 168 | 132 | 36 |
| 685-PTdeep | TMEM266 | 145 | hg19 | chr15 | 76494606 | 76494606 | Splice_Site | SNP | G | G | T | novel | Somatic |  | 0.171053 | NM_152335.3 | 228 | 189 | 39 |
| 685-PTdeep | PEAK1 | 18 | hg19 | chr15 | 77473084 | 77473084 | Missense_Mutation | SNP | C | C | A | novel | Somatic | Q395H | 0.16791 | NM_024776.4 | 267 | 222 | 45 |
| 685-PTdeep | NR2F2 | 1324 | hg19 | chr15 | 96880718 | 96880718 | Missense_Mutation | SNP | G | G | A | novel | Somatic | R371H | 0.117117 | NM_021005.4 | 333 | 294 | 39 |
| 685-PTdeep | ZP2 | 746 | hg19 | chr16 | 21218254 | 21218254 | Missense_Mutation | SNP | C | C | T | novel | Somatic | G130R | 0.173516 | NM_003460.2 | 218 | 180 | 38 |
| 685-PTdeep | CDH11 | 16 | hg19 | chr16 | 64981858 | 64981858 | Missense_Mutation | SNP | G | G | T | novel | Somatic | T680N | 0.136986 | NM_001797.4 | 146 | 126 | 20 |
| 685-PTdeep | CBFB | 0 | hg19 | chr16 | 67070641 | 67070641 | Nonsense_Mutation | SNP | G | G | T | novel | Somatic | E89Ter | 0.319149 | NM_022845.3 | 140 | 95 | 45 |
| 685-PTdeep | PLEKHG4 | 1098 | hg19 | chr16 | 67318167 | 67318167 | Intron | DEL | TG | TG | - | novel | Somatic |  | 0.084507 | NM_001129727.3 | 142 | 130 | 12 |
| 685-PTdeep | HYDIN | 140 | hg19 | chr16 | 71103285 | 71103285 | Missense_Mutation | SNP | A | A | G | novel | Somatic | V620A | 0.150235 | NM_001270974.2 | 212 | 180 | 32 |
| 685-PTdeep | TP53 | 642 | hg19 | chr17 | 7576897 | 7576897 | Nonsense_Mutation | SNP | G | G | A | novel | Somatic | Q317Ter | 0.185965 | NM_000546.5 | 285 | 232 | 53 |
| 685-PTdeep | PIK3R6 | 651 | hg19 | chr17 | 8731962 | 8731962 | Missense_Mutation | SNP | C | C | A | novel | Somatic | R412L | 0.19419 | NM_001010855.4 | 1299 | 1045 | 254 |
| 685-PTdeep | UBB | 709 | hg19 | chr17 | 16285891 | 16285891 | Missense_Mutation | SNP | C | C | T | novel | Somatic | R224C | 0.231834 | NM_018955.4 | 288 | 221 | 67 |
| 685-PTdeep | KPNB1 | 116 | hg19 | chr17 | 45734425 | 45734425 | Missense_Mutation | SNP | T | T | C | novel | Somatic | I161T | 0.06962 | NM_002265.6 | 158 | 147 | 11 |
| 685-PTdeep | CA10 | 120 | hg19 | chr17 | 49825102 | 49825102 | Missense_Mutation | SNP | C | C | G | novel | Somatic | G119A | 0.085714 | NM_020178.5 | 175 | 160 | 15 |
| 685-PTdeep | GPR142 | 1137 | hg19 | chr17 | 72368179 | 72368179 | Missense_Mutation | SNP | C | C | T | novel | Somatic | R277W | 0.129317 | NM_181790.1 | 1237 | 1076 | 161 |
| 685-PTdeep | DNAH17 | 18 | hg19 | chr17 | 76464789 | 76464789 | In_Frame_Del | DEL | GGA | GGA | - | novel | Somatic | S2896del | 0.133065 | NM_173628.4 | 248 | 215 | 33 |
| 685-PTdeep | LRRC45 | 1195 | hg19 | chr17 | 79987021 | 79987021 | Missense_Mutation | SNP | G | G | A | novel | Somatic | R454Q | 0.121212 | NM_144999.4 | 460 | 404 | 56 |
| 685-PTdeep | CSNK1D | 149 | hg19 | chr17 | 80210978 | 80210978 | Missense_Mutation | SNP | C | C | T | novel | Somatic | R160H | 0.251716 | NM_001363749.2 | 437 | 327 | 110 |
| 685-PTdeep | ATCAY | 614 | hg19 | chr19 | 3909529 | 3909529 | Missense_Mutation | SNP | G | G | A | novel | Somatic | M231I | 0.122449 | NM_033064.5 | 294 | 258 | 36 |
| 685-PTdeep | CYP4F12 | 705 | hg19 | chr19 | 15793235 | 15793235 | Missense_Mutation | DNP | TG | TG | CA | novel | Somatic | R188H | 0.164 | NM_023944.4 | 42 | 1 | 41 |
| 685-PTdeep | NXNL1 | 719 | hg19 | chr19 | 17566520 | 17566520 | Missense_Mutation | SNP | G | G | A | novel | Somatic | A192V | 0.118914 | NM_138454.2 | 1018 | 891 | 127 |
| 685-PTdeep | ZNF429 | 750 | hg19 | chr19 | 21720557 | 21720557 | Missense_Mutation | SNP | A | A | G | novel | Somatic | K568E | 0.053763 | NM_001001415.4 | 650 | 615 | 35 |
| 685-PTdeep | GGN | 881 | hg19 | chr19 | 38876137 | 38876137 | Missense_Mutation | SNP | G | G | A | novel | Somatic | L589F | 0.134454 | NM_152657.4 | 711 | 615 | 96 |
| 685-PTdeep | DYRK1B | 892 | hg19 | chr19 | 40321074 | 40321074 | Missense_Mutation | SNP | C | C | T | novel | Somatic | E105K | 0.1341 | NM_004714.3 | 261 | 226 | 35 |
| 685-PTdeep | GRIN2D | 958 | hg19 | chr19 | 48946238 | 48946238 | Missense_Mutation | SNP | C | C | T | novel | Somatic | P1019S | 0.215909 | NM_000836.2 | 439 | 344 | 95 |
| 685-PTdeep | GRIN2D | 958 | hg19 | chr19 | 48946554 | 48946554 | Missense_Mutation | SNP | C | C | T | novel | Somatic | A1124V | 0.196522 | NM_000836.2 | 573 | 460 | 113 |
| 685-PTdeep | SDCBP2 | 594 | hg19 | chr20 | 1294062 | 1294062 | Missense_Mutation | SNP | G | G | C | novel | Somatic | I102M | 0.023562 | NM_080489.5 | 1443 | 1409 | 34 |
| 685-PTdeep | OTOR | 712 | hg19 | chr20 | 16729605 | 16729605 | Nonsense_Mutation | SNP | C | C | G | novel | Somatic | S70Ter | 0.118143 | NM_020157.3 | 237 | 209 | 28 |
| 685-PTdeep | NKX2-2 | 748 | hg19 | chr20 | 21492817 | 21492817 | Missense_Mutation | SNP | T | T | A | novel | Somatic | E189V | 0.176225 | NM_002509.4 | 956 | 787 | 169 |
| 685-PTdeep | TM9SF4 | 819 | hg19 | chr20 | 30753177 | 30753177 | Missense_Mutation | SNP | C | C | T | novel | Somatic | T620M | 0.081699 | NM_014742.4 | 304 | 279 | 25 |
| 685-PTdeep | ASXL1 | 821 | hg19 | chr20 | 31022982 | 31022982 | Frame_Shift_Ins | INS | - | - | TAGA | novel | Somatic | E824DfsTer10 | 0.327217 | NM_015338.6 | 323 | 216 | 107 |
| 685-PTdeep | SUN5 | 103 | hg19 | chr20 | 31589024 | 31589024 | Missense_Mutation | SNP | G | G | C | novel | Somatic | T91R | 0.12069 | NM_080675.4 | 462 | 406 | 56 |
| 685-PTdeep | LIPI | 703 | hg19 | chr21 | 15538701 | 15538701 | Missense_Mutation | SNP | G | G | C | novel | Somatic | P260A | 0.1 | NM_198996.3 | 170 | 153 | 17 |
| 685-PTdeep | COL6A1 | 946 | hg19 | chr21 | 47404280 | 47404280 | Missense_Mutation | SNP | G | G | A | novel | Somatic | G109S | 0.257937 | NM_001848.3 | 754 | 559 | 195 |
| 685-PTdeep | CACNA1I | 111 | hg19 | chr22 | 40054959 | 40054959 | Missense_Mutation | SNP | C | C | G | novel | Somatic | A723G | 0.068554 | NM_021096.4 | 671 | 625 | 46 |
| 685-PTdeep | TUBGCP6 | 971 | hg19 | chr22 | 50671810 | 50671810 | Missense_Mutation | SNP | C | C | T | novel | Somatic | E351K | 0.127536 | NM_020461.4 | 688 | 600 | 88 |
| 685-PTdeep | GRPR | 708 | hg19 | chrX | 16142299 | 16142299 | Missense_Mutation | SNP | C | C | A | novel | Somatic | P75T | 0.221311 | NM_005314.3 | 122 | 95 | 27 |
| 685-LNmet | COL16A1 | 830 | hg19 | chr1 | 32127969 | 32127969 | Missense_Mutation | SNP | C | C | T | novel | Somatic | G1216D | 0.021038 | NM_001856.4 | 712 | 697 | 15 |
| 685-LNmet | TOE1 | 934 | hg19 | chr1 | 45806886 | 45806886 | Missense_Mutation | SNP | C | C | T | novel | Somatic | T65M | 0.047739 | NM_025077.4 | 395 | 376 | 19 |
| 685-LNmet | SERBP1 | 137 | hg19 | chr1 | 67885763 | 67885763 | Nonsense_Mutation | SNP | G | G | A | novel | Somatic | R309Ter | 0.05 | NM_001018067.2 | 260 | 247 | 13 |
| 685-LNmet | SYPL2 | 1424 | hg19 | chr1 | 110009654 | 110009654 | Intron | DEL | A | A | - | novel | Somatic |  | 0.069054 | NM_001040709.2 | 387 | 360 | 27 |
| 685-LNmet | DENND4B | 1759 | hg19 | chr1 | 153907278 | 153907278 | Missense_Mutation | SNP | C | C | G | novel | Somatic | E911Q | 0.038394 | NM_014856.3 | 569 | 547 | 22 |
| 685-LNmet | FMO2 | 236 | hg19 | chr1 | 171168584 | 171168584 | Missense_Mutation | SNP | C | C | T | novel | Somatic | S195L | 0.071823 | NM_001460.5 | 13 | 0 | 13 |
| 685-LNmet | RALGPS2 | 243 | hg19 | chr1 | 178777229 | 178777229 | Missense_Mutation | SNP | C | C | A | novel | Somatic | P88Q | 0.042403 | NM_152663.5 | 283 | 271 | 12 |
| 685-LNmet | ZNF678 | 290 | hg19 | chr1 | 227842419 | 227842419 | Missense_Mutation | SNP | C | C | A | novel | Somatic | D211E | 0.050877 | NM_178549.4 | 560 | 531 | 29 |
| 685-LNmet | USP34 | 131 | hg19 | chr2 | 61570965 | 61570965 | Missense_Mutation | SNP | C | C | T | novel | Somatic | E829K | 0.075397 | NM_014709.4 | 252 | 233 | 19 |
| 685-LNmet | FIGLA | 1126 | hg19 | chr2 | 71017734 | 71017734 | Missense_Mutation | SNP | C | C | T | novel | Somatic | A13T | 0.08209 | NM_001004311.3 | 781 | 715 | 66 |
| 685-LNmet | RAB11FIP5 | 1144 | hg19 | chr2 | 73308223 | 73308223 | Missense_Mutation | SNP | T | T | C | novel | Somatic | R713G | 0.051095 | NM_001371272.1 | 822 | 780 | 42 |
| 685-LNmet | TGOLN2 | 1237 | hg19 | chr2 | 85554654 | 85554654 | Frame_Shift_Ins | INS | - | - | G | novel | Somatic | S68Ter | 0.051613 | NM_006464.4 | 309 | 293 | 16 |
| 685-LNmet | DPP10 | 22 | hg19 | chr2 | 116539930 | 116539930 | Missense_Mutation | SNP | C | C | A | novel | Somatic | P513T | 0.04717 | NM_001321905.2 | 211 | 201 | 10 |
| 685-LNmet | PRPF40A | 1756 | hg19 | chr2 | 153526794 | 153526794 | Missense_Mutation | SNP | C | C | G | novel | Somatic | Q569H | 0.042553 | NM_001365596.2 | 235 | 225 | 10 |
| 685-LNmet | SRGAP3 | 81 | hg19 | chr3 | 9074383 | 9074383 | Missense_Mutation | SNP | G | G | A | novel | Somatic | S497L | 0.076142 | NM_014850.4 | 393 | 363 | 30 |
| 685-LNmet | MORC1 | 176 | hg19 | chr3 | 108723661 | 108723661 | Splice_Region | SNP | C | C | T | novel | Somatic |  | 0.052885 | NM_014429.4 | 208 | 197 | 11 |
| 685-LNmet | TRIM59 | 1806 | hg19 | chr3 | 160156368 | 160156368 | Frame_Shift_Del | DEL | T | T | - | novel | Somatic | S202VfsTer3 | 0.044068 | NM_173084.3 | 291 | 278 | 13 |
| 685-LNmet | PHC3 | 29 | hg19 | chr3 | 169815155 | 169815155 | Missense_Mutation | SNP | C | C | T | novel | Somatic | D951N | 0.079452 | NM_024947.4 | 363 | 334 | 29 |
| 685-LNmet | TNIK | 1890 | hg19 | chr3 | 170802067 | 170802067 | Nonsense_Mutation | SNP | C | C | A | novel | Somatic | E1016Ter | 0.30198 | NM_015028.4 | 601 | 418 | 183 |
| 685-LNmet | PEX5L | 244 | hg19 | chr3 | 179533791 | 179533791 | Missense_Mutation | SNP | C | C | G | novel | Somatic | G314A | 0.048544 | NM_016559.3 | 308 | 293 | 15 |
| 685-LNmet | MAP6D1 | 1985 | hg19 | chr3 | 183543193 | 183543193 | Missense_Mutation | SNP | G | G | A | novel | Somatic | S48L | 0.034164 | NM_024871.3 | 1393 | 1345 | 48 |
| 685-LNmet | UGT2B4 | 1121 | hg19 | chr4 | 70346458 | 70346458 | Missense_Mutation | SNP | G | G | A | novel | Somatic | T494I | 0.06379 | NM_021139.3 | 529 | 495 | 34 |
| 685-LNmet | ADAMTS3 | 17 | hg19 | chr4 | 73414436 | 73414436 | Missense_Mutation | SNP | C | C | A | novel | Somatic | G88V | 0.052632 | NM_014243.3 | 245 | 232 | 13 |
| 685-LNmet | MAPK10 | 19 | hg19 | chr4 | 86938355 | 86938355 | Missense_Mutation | SNP | G | G | A | novel | Somatic | A474V | 0.024719 | NM_001318069.2 | 445 | 434 | 11 |
| 685-LNmet | POC5 | 144 | hg19 | chr5 | 74998445 | 74998445 | Missense_Mutation | SNP | C | C | G | novel | Somatic | W166C | 0.038095 | NM_001099271.2 | 313 | 301 | 12 |
| 685-LNmet | MTRNR2L2 | 1194 | hg19 | chr5 | 79945871 | 79945871 | Missense_Mutation | SNP | G | G | A | novel | Somatic | S12L | 0.126722 | NM_001190470.2 | 363 | 317 | 46 |
| 685-LNmet | PCDHA2 | 1654 | hg19 | chr5 | 140174909 | 140174909 | Missense_Mutation | SNP | A | A | C | novel | Somatic | E120D | 0.030457 | NM_018905.3 | 385 | 373 | 12 |
| 685-LNmet | FAM193B | 241 | hg19 | chr5 | 176965990 | 176965990 | Silent | SNP | G | G | A | novel | Somatic | G123= | 0.043668 | NM_001190946.3 | 228 | 218 | 10 |
| 685-LNmet | LPA | 226 | hg19 | chr6 | 161006168 | 161006168 | Missense_Mutation | DNP | AG | AG | TA | novel | Somatic | L1400Y | 0.045283 | NM_005577.4 | 263 | 251 | 12 |
| 685-LNmet | OSR2 | 1347 | hg19 | chr8 | 99961377 | 99961377 | Missense_Mutation | SNP | G | G | A | novel | Somatic | R187H | 0.068602 | NM_001286841.1 | 379 | 353 | 26 |
| 685-LNmet | JRK | 1681 | hg19 | chr8 | 143746089 | 143746089 | Missense_Mutation | SNP | A | A | C | novel | Somatic | W463G | 0.067174 | NM_003724.4 | 158 | 105 | 53 |
| 685-LNmet | SURF2 | 1624 | hg19 | chr9 | 136226880 | 136226880 | In_Frame_Del | DEL | GGA | GGA | - | novel | Somatic | R135del | 0.02864 | NM_017503.5 | 414 | 402 | 12 |
| 685-LNmet | DNA2 | 1120 | hg19 | chr10 | 70228005 | 70228005 | Missense_Mutation | SNP | C | C | G | novel | Somatic | D106H | 0.034014 | NM_001080449.3 | 294 | 284 | 10 |
| 685-LNmet | CAMK2G | 145 | hg19 | chr10 | 75576499 | 75576499 | Missense_Mutation | SNP | A | A | G | novel | Somatic | Y583H | 0.200351 | NM_001320898.1 | 558 | 444 | 114 |
| 685-LNmet | CFAP46 | 201 | hg19 | chr10 | 134738257 | 134738257 | Missense_Mutation | SNP | C | C | T | novel | Somatic | R400Q | 0.029575 | NM_001200049.3 | 1076 | 1044 | 32 |
| 685-LNmet | INPPL1 | 1133 | hg19 | chr11 | 71948767 | 71948767 | Missense_Mutation | SNP | C | C | T | novel | Somatic | S1160L | 0.027211 | NM_001567.4 | 584 | 568 | 16 |
| 685-LNmet | CCDC89 | 1236 | hg19 | chr11 | 85396056 | 85396056 | Missense_Mutation | SNP | G | G | C | novel | Somatic | S373C | 0.072917 | NM_152723.3 | 192 | 178 | 14 |
| 685-LNmet | ARHGAP32 | 195 | hg19 | chr11 | 128844234 | 128844234 | Missense_Mutation | SNP | C | C | G | novel | Somatic | W939S | 0.046809 | NM_001142685.1 | 235 | 224 | 11 |
| 685-LNmet | KRT18 | 123 | hg19 | chr12 | 53346096 | 53346096 | Missense_Mutation | SNP | G | G | A | novel | Somatic | R381H | 0.065934 | NM_199187.1 | 259 | 241 | 18 |
| 685-LNmet | OR6C75 | 1010 | hg19 | chr12 | 55759192 | 55759192 | Frame_Shift_Del | DEL | T | T | - | novel | Somatic | F102SfsTer6 | 0.044534 | NM_001005497.1 | 247 | 236 | 11 |
| 685-LNmet | DYRK2 | 1104 | hg19 | chr12 | 68043649 | 68043649 | Missense_Mutation | SNP | G | G | C | novel | Somatic | G41A | 0.041047 | NM_006482.3 | 1408 | 1350 | 58 |
| 685-LNmet | AMER2 | 781 | hg19 | chr13 | 25744182 | 25744182 | Missense_Mutation | SNP | C | C | A | novel | Somatic | D526Y | 0.0401 | NM_152704.3 | 399 | 383 | 16 |
| 685-LNmet | COL4A1 | 178 | hg19 | chr13 | 110844637 | 110844637 | Splice_Region | SNP | G | G | C | novel | Somatic |  | 0.060606 | NM_001845.6 | 297 | 279 | 18 |
| 685-LNmet | MTHFD1 | 134 | hg19 | chr14 | 64892478 | 64892478 | Missense_Mutation | SNP | G | G | A | novel | Somatic | E296K | 0.075 | NM_005956.4 | 280 | 259 | 21 |
| 685-LNmet | SRSF5 | 1120 | hg19 | chr14 | 70237949 | 70237949 | Missense_Mutation | SNP | C | C | T | novel | Somatic | S197F | 0.034591 | NM_001039465.2 | 318 | 307 | 11 |
| 685-LNmet | ISM2 | 1179 | hg19 | chr14 | 77942267 | 77942267 | Missense_Mutation | SNP | C | C | T | novel | Somatic | E463K | 0.055118 | NM_199296.3 | 634 | 599 | 35 |
| 685-LNmet | MOK | 1368 | hg19 | chr14 | 102700053 | 102700053 | Missense_Mutation | SNP | C | C | T | novel | Somatic | A222T | 0.026954 | NM_014226.3 | 368 | 358 | 10 |
| 685-LNmet | LOC283710 | 825 | hg19 | chr15 | 31521506 | 31521506 | In_Frame_Del | DEL | GGG | GGG | - | novel | Somatic | P25del | 0.042296 | NM_001243538.3 | 126 | 112 | 14 |
| 685-LNmet | PEAK1 | 18 | hg19 | chr15 | 77473084 | 77473084 | Missense_Mutation | SNP | C | C | A | novel | Somatic | Q395H | 0.045643 | NM_024776.4 | 241 | 230 | 11 |
| 685-LNmet | NR2F2 | 1324 | hg19 | chr15 | 96880718 | 96880718 | Missense_Mutation | SNP | G | G | A | novel | Somatic | R371H | 0.036675 | NM_021005.4 | 403 | 388 | 15 |
| 685-LNmet | USP10 | 153 | hg19 | chr16 | 84792991 | 84792991 | Missense_Mutation | SNP | G | G | T | novel | Somatic | C440F | 0.041522 | NM_001272075.2 | 289 | 277 | 12 |
| 685-LNmet | USP10 | 153 | hg19 | chr16 | 84806189 | 84806189 | Missense_Mutation | SNP | G | G | C | novel | Somatic | V685L | 0.029703 | NM_001272075.2 | 505 | 490 | 15 |
| 685-LNmet | TP53 | 642 | hg19 | chr17 | 7576897 | 7576897 | Nonsense_Mutation | SNP | G | G | A | novel | Somatic | Q317Ter | 0.020619 | NM_000546.5 | 484 | 474 | 10 |
| 685-LNmet | PIK3R6 | 651 | hg19 | chr17 | 8731962 | 8731962 | Missense_Mutation | SNP | C | C | A | novel | Somatic | R412L | 0.02343 | NM_001010855.4 | 1062 | 1037 | 25 |
| 685-LNmet | RPL23 | 867 | hg19 | chr17 | 37008901 | 37008901 | Missense_Mutation | SNP | A | A | C | novel | Somatic | V61G | 0.044444 | NM_000978.4 | 269 | 257 | 12 |
| 685-LNmet | KRT14 | 888 | hg19 | chr17 | 39740027 | 39740027 | Missense_Mutation | SNP | T | T | A | novel | Somatic | E304D | 0.03096 | NM_000526.5 | 322 | 312 | 10 |
| 685-LNmet | TMEM94 | 1145 | hg19 | chr17 | 73482037 | 73482037 | In_Frame_Del | DEL | TGC | TGC | - | novel | Somatic | L81del | 0.057078 | NM_014738.6 | 435 | 410 | 25 |
| 685-LNmet | QRICH2 | 1151 | hg19 | chr17 | 74288760 | 74288760 | Missense_Mutation | SNP | G | G | C | novel | Somatic | P517R | 0.054745 | NM_032134.2 | 274 | 259 | 15 |
| 685-LNmet | LRRC45 | 1195 | hg19 | chr17 | 79987021 | 79987021 | Missense_Mutation | SNP | G | G | A | novel | Somatic | R454Q | 0.041016 | NM_144999.4 | 512 | 491 | 21 |
| 685-LNmet | OR1M1 | 655 | hg19 | chr19 | 9204109 | 9204109 | Missense_Mutation | DNP | GG | GG | CT | novel | Somatic | A64S | 0.033557 | NM_001004456.1 | 591 | 571 | 20 |
| 685-LNmet | ZNF429 | 750 | hg19 | chr19 | 21720557 | 21720557 | Missense_Mutation | SNP | A | A | G | novel | Somatic | K568E | 0.073171 | NM_001001415.4 | 656 | 608 | 48 |
| 685-LNmet | GGN | 881 | hg19 | chr19 | 38876137 | 38876137 | Missense_Mutation | SNP | G | G | A | novel | Somatic | L589F | 0.031008 | NM_152657.4 | 514 | 498 | 16 |
| 685-LNmet | CYP2B6 | 901 | hg19 | chr19 | 41497295 | 41497295 | Missense_Mutation | SNP | C | C | T | novel | Somatic | R29C | 0.02864 | NM_000767.5 | 417 | 405 | 12 |
| 685-LNmet | CIC | 911 | hg19 | chr19 | 42798993 | 42798993 | Missense_Mutation | SNP | T | T | C | novel | Somatic | F2402L | 0.020638 | NM_001304815.1 | 529 | 518 | 11 |
| 685-LNmet | CIC | 911 | hg19 | chr19 | 42799003 | 42799003 | Missense_Mutation | SNP | G | G | A | novel | Somatic | R2405H | 0.024575 | NM_001304815.1 | 520 | 507 | 13 |
| 685-LNmet | DHX34 | 950 | hg19 | chr19 | 47858479 | 47858479 | Missense_Mutation | SNP | C | C | T | novel | Somatic | R297C | 0.021053 | NM_014681.6 | 471 | 461 | 10 |
| 685-LNmet | GRIN2D | 958 | hg19 | chr19 | 48946554 | 48946554 | Missense_Mutation | SNP | C | C | T | novel | Somatic | A1124V | 0.071942 | NM_000836.2 | 823 | 763 | 60 |
| 685-LNmet | ZNF28 | 991 | hg19 | chr19 | 53303893 | 53303893 | Missense_Mutation | SNP | T | T | C | novel | Somatic | K402R | 0.051813 | NM_006969.5 | 385 | 365 | 20 |
| 685-LNmet | ZNF845 | 995 | hg19 | chr19 | 53855552 | 53855552 | Missense_Mutation | SNP | T | T | C | novel | Somatic | C542R | 0.054755 | NM_138374.3 | 345 | 326 | 19 |
| 685-LNmet | NKX2-2 | 748 | hg19 | chr20 | 21492817 | 21492817 | Missense_Mutation | SNP | T | T | A | novel | Somatic | E189V | 0.03125 | NM_002509.4 | 604 | 585 | 19 |
| 685-LNmet | ASXL1 | 821 | hg19 | chr20 | 31022982 | 31022982 | Frame_Shift_Ins | INS | - | - | TAGA | novel | Somatic | E824DfsTer10 | 0.055944 | NM_015338.6 | 286 | 270 | 16 |
| 685-LNmet | SUN5 | 103 | hg19 | chr20 | 31589024 | 31589024 | Missense_Mutation | SNP | G | G | C | novel | Somatic | T91R | 0.020036 | NM_080675.4 | 548 | 537 | 11 |
| 685-LNmet | COL6A1 | 946 | hg19 | chr21 | 47404280 | 47404280 | Missense_Mutation | SNP | G | G | A | novel | Somatic | G109S | 0.035868 | NM_001848.3 | 696 | 671 | 25 |
| 685-LNmet | HIC2 | 751 | hg19 | chr22 | 21800371 | 21800371 | In_Frame_Del | DEL | AGG | AGG | - | novel | Somatic | E400del | 0.046595 | NM_015094.3 | 277 | 264 | 13 |
| 685-LNmet | FAM9A | 651 | hg19 | chrX | 8764319 | 8764319 | Frame_Shift_Ins | INS | - | - | T | novel | Somatic | Q160TfsTer8 | 0.075117 | NM_174951.3 | 211 | 195 | 16 |
| 685-LNmet | FAM47C | 867 | hg19 | chrX | 37027450 | 37027450 | Missense_Mutation | SNP | T | T | C | novel | Somatic | S323P | 0.029412 | NM_001013736.2 | 339 | 329 | 10 |
| 685-LNmet | FAM47C | 867 | hg19 | chrX | 37027856 | 37027856 | Missense_Mutation | SNP | G | G | A | novel | Somatic | R458Q | 0.02381 | NM_001013736.2 | 418 | 408 | 10 |
| 685-LNmet | TAF1 | 1124 | hg19 | chrX | 70598785 | 70598785 | Missense_Mutation | SNP | G | G | A | novel | Somatic | V442I | 0.028384 | NM_004606.4 | 456 | 443 | 13 |
| 685-LNmet | RBM41 | 1396 | hg19 | chrX | 106312573 | 106312573 | Silent | SNP | G | G | A | novel | Somatic | F388= | 0.056911 | NM_001324243.1 | 245 | 231 | 14 |
| 685-LNmet | F8 | 1760 | hg19 | chrX | 154158427 | 154158427 | Frame_Shift_Ins | INS | - | - | T | novel | Somatic | I1213NfsTer28 | 0.040984 | NM_000132.3 | 240 | 230 | 10 |
| 768-PTsup | AJAP1 | 77 | hg19 | chr1 | 4772321 | 4772321 | Missense_Mutation | SNP | G | G | A | novel | Somatic | A131T | 0.122078 | NM_018836.4 | 1153 | 1012 | 141 |
| 768-PTsup | TNFRSF1B | 678 | hg19 | chr1 | 12248919 | 12248919 | Missense_Mutation | SNP | A | A | G | novel | Somatic | T49A | 0.435655 | NM_001066.3 | 846 | 477 | 369 |
| 768-PTsup | CDC42 | 94 | hg19 | chr1 | 22413346 | 22413346 | Missense_Mutation | SNP | C | C | G | novel | Somatic | S158C | 0.05914 | NM_001791.4 | 372 | 350 | 22 |
| 768-PTsup | PATJ | 132 | hg19 | chr1 | 62321714 | 62321714 | Missense_Mutation | SNP | C | C | T | novel | Somatic | P709S | 0.070922 | NM_001350145.2 | 421 | 391 | 30 |
| 768-PTsup | RABGGTB | 1166 | hg19 | chr1 | 76255688 | 76255688 | Nonsense_Mutation | SNP | G | G | T | novel | Somatic | E121Ter | 0.123457 | NM_004582.4 | 567 | 497 | 70 |
| 768-PTsup | IGSF9 | 225 | hg19 | chr1 | 159897582 | 159897582 | Missense_Mutation | SNP | G | G | A | novel | Somatic | S1109F | 0.237705 | NM_001135050.2 | 488 | 372 | 116 |
| 768-PTsup | SLAMF1 | 1810 | hg19 | chr1 | 160589600 | 160589600 | Frame_Shift_Ins | INS | - | - | T | novel | Somatic | A305SfsTer60 | 0.028658 | NM_001330754.1 | 663 | 644 | 19 |
| 768-PTsup | AXDND1 | 244 | hg19 | chr1 | 179380303 | 179380303 | Frame_Shift_Del | DEL | TT | TT | - | novel | Somatic | L378IfsTer11 | 0.103352 | NM_144696.6 | 358 | 321 | 37 |
| 768-PTsup | IGFN1 | 264 | hg19 | chr1 | 201185708 | 201185708 | Missense_Mutation | SNP | G | G | A | novel | Somatic | R3141Q | 0.26146 | NM_001164586.2 | 1178 | 870 | 308 |
| 768-PTsup | SLC41A1 | 2154 | hg19 | chr1 | 205779283 | 205779283 | Missense_Mutation | SNP | A | A | C | novel | Somatic | F96C | 0.123384 | NM_173854.6 | 851 | 746 | 105 |
| 768-PTsup | CCDC185 | 2290 | hg19 | chr1 | 223568412 | 223568412 | Missense_Mutation | SNP | T | T | A | novel | Somatic | L532H | 0.283847 | NM_152610.3 | 1068 | 764 | 304 |
| 768-PTsup | OBSCN | 290 | hg19 | chr1 | 228560408 | 228560408 | Nonsense_Mutation | SNP | C | C | G | novel | Somatic | S8267Ter | 0.125 | NM_001271223.2 | 711 | 622 | 89 |
| 768-PTsup | TARBP1 | 2374 | hg19 | chr1 | 234569179 | 234569179 | Missense_Mutation | SNP | G | G | A | novel | Somatic | L791F | 0.03268 | NM_005646.4 | 611 | 591 | 20 |
| 768-PTsup | LYST | 2385 | hg19 | chr1 | 235875399 | 235875399 | Missense_Mutation | SNP | G | G | T | novel | Somatic | P3295T | 0.080808 | NM_001301365.1 | 593 | 545 | 48 |
| 768-PTsup | KIF26B | 38 | hg19 | chr1 | 245851667 | 245851667 | In_Frame_Del | DEL | CCTGGCCTCCAAGCTTCC | CCTGGCCTCCAAGCTTCC | - | novel | Somatic | A1796_L1801del | 0.048619 | NM_018012.4 | 903 | 859 | 44 |
| 768-PTsup | SOX11 | 629 | hg19 | chr2 | 5833962 | 5833962 | Missense_Mutation | SNP | C | C | G | novel | Somatic | S370C | 0.442568 | NM_003108.4 | 592 | 330 | 262 |
| 768-PTsup | PLEK | 1108 | hg19 | chr2 | 68622898 | 68622898 | Missense_Mutation | SNP | C | C | T | novel | Somatic | R335C | 0.159375 | NM_002664.3 | 320 | 269 | 51 |
| 768-PTsup | C2orf81 | 1154 | hg19 | chr2 | 74642696 | 74642696 | Missense_Mutation | SNP | C | C | T | novel | Somatic | R203Q | 0.46093 | NM_001316764.3 | 1008 | 542 | 466 |
| 768-PTsup | RANBP2 | 1419 | hg19 | chr2 | 109382636 | 109382636 | Missense_Mutation | SNP | G | G | A | novel | Somatic | E1881K | 0.084524 | NM_006267.5 | 1678 | 1536 | 142 |
| 768-PTsup | NT5DC4 | 1450 | hg19 | chr2 | 113480080 | 113480080 | Missense_Mutation | SNP | A | A | G | novel | Somatic | E102G | 0.321762 | NM_001350494.2 | 748 | 507 | 241 |
| 768-PTsup | LCT | 203 | hg19 | chr2 | 136570058 | 136570058 | Missense_Mutation | SNP | G | G | A | novel | Somatic | R726C | 0.334331 | NM_002299.4 | 1002 | 667 | 335 |
| 768-PTsup | LRP1B | 3 | hg19 | chr2 | 141267521 | 141267521 | Missense_Mutation | SNP | C | C | T | novel | Somatic | D2792N | 0.322523 | NM_018557.3 | 555 | 376 | 179 |
| 768-PTsup | METAP1D | 237 | hg19 | chr2 | 172942627 | 172942627 | Splice_Region | SNP | G | G | A | novel | Somatic |  | 0.08818 | NM_199227.3 | 533 | 486 | 47 |
| 768-PTsup | PDE1A | 1981 | hg19 | chr2 | 183104988 | 183104988 | Missense_Mutation | SNP | C | C | T | novel | Somatic | D87N | 0.358634 | NM_001258312.1 | 526 | 337 | 189 |
| 768-PTsup | WDR12 | 2139 | hg19 | chr2 | 203765843 | 203765843 | Splice_Site | SNP | C | C | G | novel | Somatic |  | 0.089466 | NM_018256.4 | 693 | 631 | 62 |
| 768-PTsup | MAP2 | 273 | hg19 | chr2 | 210543334 | 210543334 | Missense_Mutation | SNP | G | G | A | novel | Somatic | E101K | 0.078261 | NM_002374.3 | 690 | 636 | 54 |
| 768-PTsup | USP37 | 282 | hg19 | chr2 | 219341589 | 219341589 | Missense_Mutation | SNP | C | C | T | novel | Somatic | E673K | 0.168246 | NM_020935.3 | 422 | 351 | 71 |
| 768-PTsup | USP40 | 2373 | hg19 | chr2 | 234386078 | 234386078 | Missense_Mutation | SNP | G | G | A | novel | Somatic | P1224L | 0.411079 | NM_001365479.1 | 685 | 403 | 282 |
| 768-PTsup | OTOS | 2424 | hg19 | chr2 | 241078760 | 241078760 | Missense_Mutation | SNP | C | C | T | novel | Somatic | E33K | 0.199195 | NM_148961.4 | 496 | 397 | 99 |
| 768-PTsup | NKTR | 910 | hg19 | chr3 | 42679764 | 42679764 | Frame_Shift_Del | DEL | A | A | - | novel | Somatic | R859EfsTer3 | 0.028133 | NM_005385.4 | 391 | 380 | 11 |
| 768-PTsup | BAP1 | 985 | hg19 | chr3 | 52443586 | 52443586 | Nonsense_Mutation | SNP | G | G | A | novel | Somatic | Q36Ter | 0.505137 | NM_004656.4 | 584 | 289 | 295 |
| 768-PTsup | ERC2 | 15 | hg19 | chr3 | 56330423 | 56330423 | Missense_Mutation | SNP | C | C | T | novel | Somatic | R233Q | 0.191853 | NM_015576.3 | 760 | 614 | 146 |
| 768-PTsup | OR5H14 | 1331 | hg19 | chr3 | 97868484 | 97868484 | Missense_Mutation | SNP | C | C | G | novel | Somatic | F85L | 0.101289 | NM_001005514.1 | 1086 | 976 | 110 |
| 768-PTsup | EIF4G1 | 1989 | hg19 | chr3 | 184042091 | 184042091 | Missense_Mutation | SNP | G | G | C | novel | Somatic | E866Q | 0.384289 | NM_001194947.1 | 471 | 290 | 181 |
| 768-PTsup | MUC4 | 2076 | hg19 | chr3 | 195512594 | 195512594 | Missense_Mutation | SNP | G | G | A | novel | Somatic | L1953F | 0.26616 | NM_018406.7 | 3679 | 2699 | 980 |
| 768-PTsup | NKX1-1 | 595 | hg19 | chr4 | 1399930 | 1399930 | Missense_Mutation | SNP | C | C | T | novel | Somatic | V101M | 0.070248 | NM_001290079.1 | 242 | 225 | 17 |
| 768-PTsup | CCDC96 | 638 | hg19 | chr4 | 7043867 | 7043867 | Missense_Mutation | SNP | C | C | T | novel | Somatic | E267K | 0.471963 | NM_153376.3 | 642 | 339 | 303 |
| 768-PTsup | WDR19 | 110 | hg19 | chr4 | 39206803 | 39206803 | Frame_Shift_Ins | INS | - | - | T | novel | Somatic | L214FfsTer5 | 0.175066 | NM_025132.4 | 371 | 305 | 66 |
| 768-PTsup | FAM13A | 158 | hg19 | chr4 | 89671659 | 89671659 | Missense_Mutation | SNP | C | C | T | novel | Somatic | R619Q | 0.173442 | NM_014883.4 | 737 | 609 | 128 |
| 768-PTsup | USP38 | 1684 | hg19 | chr4 | 144116919 | 144116919 | Missense_Mutation | SNP | T | T | C | novel | Somatic | V290A | 0.472477 | NM_032557.6 | 218 | 115 | 103 |
| 768-PTsup | TENM3 | 30 | hg19 | chr4 | 183721137 | 183721137 | Missense_Mutation | SNP | C | C | T | novel | Somatic | T2578M | 0.072072 | NM_001080477.4 | 666 | 618 | 48 |
| 768-PTsup | SORBS2 | 250 | hg19 | chr4 | 186544674 | 186544674 | Missense_Mutation | SNP | C | C | T | novel | Somatic | E733K | 0.387331 | NM_001270771.2 | 963 | 590 | 373 |
| 768-PTsup | H3.Y | 719 | hg19 | chr5 | 17655622 | 17655622 | Missense_Mutation | SNP | C | C | T | novel | Somatic | R9H | 0.515611 | NM_001355258.2 | 2303 | 1114 | 1189 |
| 768-PTsup | CDH10 | 96 | hg19 | chr5 | 24491793 | 24491793 | Missense_Mutation | SNP | C | C | T | novel | Somatic | D590N | 0.169471 | NM_006727.5 | 832 | 691 | 141 |
| 768-PTsup | AGXT2 | 852 | hg19 | chr5 | 35035385 | 35035385 | Missense_Mutation | SNP | C | C | T | novel | Somatic | E175K | 0.08838 | NM_031900.4 | 611 | 557 | 54 |
| 768-PTsup | ZNF608 | 191 | hg19 | chr5 | 123980275 | 123980275 | Missense_Mutation | SNP | C | C | T | novel | Somatic | R1262K | 0.483117 | NM_020747.2 | 385 | 199 | 186 |
| 768-PTsup | PCDHB12 | 1657 | hg19 | chr5 | 140589591 | 140589591 | Missense_Mutation | SNP | G | G | C | novel | Somatic | R371P | 0.083333 | NM_018932.4 | 528 | 484 | 44 |
| 768-PTsup | GRIA1 | 27 | hg19 | chr5 | 153035421 | 153035421 | Missense_Mutation | SNP | C | C | T | novel | Somatic | L230F | 0.494208 | NM_000827.4 | 259 | 131 | 128 |
| 768-PTsup | ATXN1 | 88 | hg19 | chr6 | 16327912 | 16327912 | Missense_Mutation | SNP | C | C | A | novel | Somatic | Q210H | 0.077196 | NM_000332.3 | 2217 | 2043 | 174 |
| 768-PTsup | FAM8A1 | 719 | hg19 | chr6 | 17600647 | 17600647 | Missense_Mutation | SNP | G | G | A | novel | Somatic | E3K | 0.125382 | NM_016255.3 | 325 | 284 | 41 |
| 768-PTsup | DNAH8 | 13 | hg19 | chr6 | 38825390 | 38825390 | Missense_Mutation | SNP | C | C | A | novel | Somatic | Q1944K | 0.327434 | NM_001206927.2 | 339 | 228 | 111 |
| 768-PTsup | COL19A1 | 140 | hg19 | chr6 | 70639566 | 70639566 | Nonsense_Mutation | SNP | C | C | T | novel | Somatic | R214Ter | 0.26284 | NM_001858.6 | 329 | 242 | 87 |
| 768-PTsup | GJA10 | 1276 | hg19 | chr6 | 90605586 | 90605586 | Missense_Mutation | SNP | C | C | G | novel | Somatic | P467A | 0.05915 | NM_032602.2 | 540 | 508 | 32 |
| 768-PTsup | SYNE1 | 1748 | hg19 | chr6 | 152527355 | 152527355 | Missense_Mutation | SNP | G | G | A | novel | Somatic | S7656L | 0.145275 | NM_182961.4 | 709 | 606 | 103 |
| 768-PTsup | ARID1B | 27 | hg19 | chr6 | 157099403 | 157099403 | In_Frame_Del | DEL | CAGCAGCAGCAGCAGCAGCAGCAA | CAGCAGCAGCAGCAGCAGCAGCAA | - | novel | Somatic | Q124_Q131del | 0.053813 | NM_020732.3 | 2931 | 2768 | 163 |
| 768-PTsup | FNDC1 | 225 | hg19 | chr6 | 159672439 | 159672439 | Missense_Mutation | SNP | A | A | T | novel | Somatic | D1647V | 0.166369 | NM_032532.3 | 559 | 466 | 93 |
| 768-PTsup | LPA | 226 | hg19 | chr6 | 160952810 | 160952810 | Missense_Mutation | SNP | C | C | T | novel | Somatic | R2025H | 0.29979 | NM_005577.4 | 953 | 667 | 286 |
| 768-PTsup | PRR18 | 1856 | hg19 | chr6 | 166721128 | 166721128 | Missense_Mutation | SNP | G | G | C | novel | Somatic | S168W | 0.204819 | NM_175922.4 | 332 | 264 | 68 |
| 768-PTsup | UNC93A | 1864 | hg19 | chr6 | 167711503 | 167711503 | Missense_Mutation | SNP | C | C | A | novel | Somatic | N190K | 0.295808 | NM_018974.4 | 835 | 588 | 247 |
| 768-PTsup | CYP2W1 | 592 | hg19 | chr7 | 1024112 | 1024112 | Missense_Mutation | SNP | C | C | T | novel | Somatic | T80M | 0.161657 | NM_017781.3 | 1230 | 1031 | 199 |
| 768-PTsup | SDK1 | 9 | hg19 | chr7 | 4272926 | 4272926 | Missense_Mutation | SNP | G | G | A | novel | Somatic | R1956Q | 0.147358 | NM_152744.4 | 984 | 839 | 145 |
| 768-PTsup | DNAH11 | 93 | hg19 | chr7 | 21913114 | 21913114 | Missense_Mutation | SNP | G | G | C | novel | Somatic | D4064H | 0.144928 | NM_001277115.2 | 345 | 295 | 50 |
| 768-PTsup | NPY | 770 | hg19 | chr7 | 24324876 | 24324876 | Missense_Mutation | SNP | G | G | A | novel | Somatic | R6Q | 0.053517 | NM_000905.4 | 654 | 619 | 35 |
| 768-PTsup | ZNF716 | 1023 | hg19 | chr7 | 57529513 | 57529513 | Nonsense_Mutation | SNP | C | C | A | novel | Somatic | S449Ter | 0.050078 | NM_001159279.1 | 639 | 607 | 32 |
| 768-PTsup | GRM3 | 155 | hg19 | chr7 | 86415818 | 86415818 | Missense_Mutation | SNP | G | G | A | novel | Somatic | R237H | 0.315837 | NM_000840.2 | 1105 | 756 | 349 |
| 768-PTsup | KMT2E | 172 | hg19 | chr7 | 104707230 | 104707230 | Missense_Mutation | SNP | A | A | G | novel | Somatic | Y158C | 0.29916 | NM_182931.3 | 595 | 417 | 178 |
| 768-PTsup | NBN | 159 | hg19 | chr8 | 90967655 | 90967655 | Nonsense_Mutation | SNP | G | G | C | novel | Somatic | S418Ter | 0.415385 | NM_002485.4 | 520 | 304 | 216 |
| 768-PTsup | VPS13B | 1348 | hg19 | chr8 | 100796636 | 100796636 | Missense_Mutation | SNP | G | G | T | novel | Somatic | D2650Y | 0.475789 | NM_017890.4 | 475 | 249 | 226 |
| 768-PTsup | PKHD1L1 | 178 | hg19 | chr8 | 110432825 | 110432825 | Missense_Mutation | SNP | C | C | A | novel | Somatic | T868K | 0.099248 | NM_177531.6 | 663 | 597 | 66 |
| 768-PTsup | ZHX2 | 191 | hg19 | chr8 | 123964798 | 123964798 | Missense_Mutation | SNP | G | G | A | novel | Somatic | A350T | 0.109549 | NM_014943.5 | 1704 | 1517 | 187 |
| 768-PTsup | KCNQ3 | 24 | hg19 | chr8 | 133150232 | 133150232 | Frame_Shift_Ins | INS | - | - | T | novel | Somatic | F534IfsTer15 | 0.049065 | NM_004519.4 | 427 | 406 | 21 |
| 768-PTsup | FAM135B | 25 | hg19 | chr8 | 139158208 | 139158208 | Missense_Mutation | SNP | C | C | A | novel | Somatic | K1178N | 0.073973 | NM_015912.4 | 365 | 338 | 27 |
| 768-PTsup | FRMPD1 | 872 | hg19 | chr9 | 37732361 | 37732361 | Nonsense_Mutation | SNP | C | C | T | novel | Somatic | R307Ter | 0.473881 | NM_014907.3 | 267 | 140 | 127 |
| 768-PTsup | NIPSNAP3B | 1405 | hg19 | chr9 | 107528646 | 107528646 | Missense_Mutation | SNP | G | G | A | novel | Somatic | G34E | 0.455801 | NM_018376.4 | 361 | 196 | 165 |
| 768-PTsup | KCNT1 | 205 | hg19 | chr9 | 138606490 | 138606490 | Missense_Mutation | SNP | G | G | A | novel | Somatic | E60K | 0.522353 | NM_020822.3 | 425 | 203 | 222 |
| 768-PTsup | CLIC3 | 1652 | hg19 | chr9 | 139889210 | 139889210 | Missense_Mutation | SNP | C | C | T | novel | Somatic | E212K | 0.214539 | NM_004669.3 | 564 | 443 | 121 |
| 768-PTsup | KCNMA1 | 1190 | hg19 | chr10 | 79397364 | 79397364 | Missense_Mutation | SNP | C | C | T | novel | Somatic | G13S | 0.025739 | NM_001161352.2 | 1048 | 1021 | 27 |
| 768-PTsup | ALDH18A1 | 165 | hg19 | chr10 | 97387306 | 97387306 | Missense_Mutation | SNP | G | G | T | novel | Somatic | S324Y | 0.146982 | NM_002860.4 | 381 | 325 | 56 |
| 768-PTsup | SFRP5 | 1344 | hg19 | chr10 | 99531359 | 99531359 | Missense_Mutation | SNP | C | C | G | novel | Somatic | E78Q | 0.401327 | NM_003015.3 | 603 | 361 | 242 |
| 768-PTsup | NRAP | 1465 | hg19 | chr10 | 115381738 | 115381738 | Nonsense_Mutation | SNP | G | G | A | novel | Somatic | Q887Ter | 0.096859 | NM_001261463.1 | 382 | 345 | 37 |
| 768-PTsup | FGFR2 | 1525 | hg19 | chr10 | 123263350 | 123263350 | Missense_Mutation | SNP | C | C | T | novel | Somatic | E465K | 0.205163 | NM_000141.4 | 735 | 584 | 151 |
| 768-PTsup | DMBT1 | 191 | hg19 | chr10 | 124402699 | 124402699 | Missense_Mutation | SNP | C | C | T | novel | Somatic | R2343W | 0.316736 | NM_007329.2 | 723 | 494 | 229 |
| 768-PTsup | E2F8 | 731 | hg19 | chr11 | 19252218 | 19252218 | Missense_Mutation | SNP | C | C | G | novel | Somatic | K410N | 0.204152 | NM_024680.4 | 288 | 229 | 59 |
| 768-PTsup | PAX6 | 827 | hg19 | chr11 | 31816252 | 31816252 | Missense_Mutation | SNP | C | C | T | novel | Somatic | R203Q | 0.197002 | NM_000280.4 | 466 | 374 | 92 |
| 768-PTsup | AHNAK | 1060 | hg19 | chr11 | 62295012 | 62295012 | Missense_Mutation | SNP | C | C | T | novel | Somatic | G2293R | 0.194357 | NM_001620.3 | 637 | 513 | 124 |
| 768-PTsup | CPT1A | 138 | hg19 | chr11 | 68548137 | 68548137 | Missense_Mutation | SNP | C | C | T | novel | Somatic | D477N | 0.081818 | NM_001876.4 | 880 | 808 | 72 |
| 768-PTsup | CLPB | 141 | hg19 | chr11 | 72069957 | 72069957 | Missense_Mutation | SNP | C | C | T | novel | Somatic | D248N | 0.417891 | NM_001258392.3 | 748 | 435 | 313 |
| 768-PTsup | WNT5B | 598 | hg19 | chr12 | 1755207 | 1755207 | Missense_Mutation | SNP | G | G | A | novel | Somatic | R290H | 0.227594 | NM_032642.3 | 848 | 655 | 193 |
| 768-PTsup | GNB3 | 638 | hg19 | chr12 | 6952180 | 6952180 | Missense_Mutation | SNP | G | G | A | novel | Somatic | R48Q | 0.314121 | NM_002075.4 | 694 | 476 | 218 |
| 768-PTsup | USP5 | 638 | hg19 | chr12 | 6972416 | 6972416 | Missense_Mutation | SNP | G | G | A | novel | Somatic | G610E | 0.303258 | NM_001098536.2 | 797 | 555 | 242 |
| 768-PTsup | KIAA1551 | 103 | hg19 | chr12 | 32138043 | 32138043 | Missense_Mutation | SNP | T | T | G | novel | Somatic | M1385R | 0.176849 | NM_018169.4 | 311 | 256 | 55 |
| 768-PTsup | ARID2 | 14 | hg19 | chr12 | 46245223 | 46245223 | Missense_Mutation | SNP | C | C | T | novel | Somatic | A1106V | 0.172566 | NM_152641.4 | 450 | 372 | 78 |
| 768-PTsup | ANO4 | 169 | hg19 | chr12 | 101336303 | 101336303 | Missense_Mutation | SNP | T | T | G | novel | Somatic | M149R | 0.121681 | NM_001286615.2 | 452 | 397 | 55 |
| 768-PTsup | FOXN4 | 1422 | hg19 | chr12 | 109725301 | 109725301 | Frame_Shift_Del | DEL | G | G | - | novel | Somatic | P166HfsTer36 | 0.17913 | NM_213596.3 | 575 | 472 | 103 |
| 768-PTsup | RXFP2 | 103 | hg19 | chr13 | 32339215 | 32339215 | Missense_Mutation | SNP | G | G | A | novel | Somatic | E121K | 0.217573 | NM_130806.5 | 478 | 374 | 104 |
| 768-PTsup | ABCC4 | 164 | hg19 | chr13 | 95696016 | 95696016 | Frame_Shift_Del | DEL | T | T | - | novel | Somatic | I1219SfsTer11 | 0.057743 | NM_005845.4 | 381 | 359 | 22 |
| 768-PTsup | UGGT2 | 20 | hg19 | chr13 | 96599314 | 96599314 | Missense_Mutation | SNP | C | C | T | novel | Somatic | E552K | 0.147249 | NM_020121.4 | 618 | 527 | 91 |
| 768-PTsup | RNASE9 | 745 | hg19 | chr14 | 21024652 | 21024652 | Missense_Mutation | SNP | T | T | G | novel | Somatic | S198R | 0.094915 | NM_001110359.1 | 590 | 534 | 56 |
| 768-PTsup | PRPF39 | 932 | hg19 | chr14 | 45571797 | 45571797 | Missense_Mutation | SNP | A | A | T | novel | Somatic | Y212F | 0.103348 | NM_017922.4 | 686 | 615 | 71 |
| 768-PTsup | MTHFD1 | 134 | hg19 | chr14 | 64920579 | 64920579 | Missense_Mutation | SNP | G | G | C | novel | Somatic | Q855H | 0.172757 | NM_005956.4 | 301 | 249 | 52 |
| 768-PTsup | SPATA7 | 157 | hg19 | chr14 | 88859755 | 88859755 | Missense_Mutation | SNP | C | C | T | novel | Somatic | S38F | 0.335088 | NM_018418.5 | 570 | 379 | 191 |
| 768-PTsup | AKT1 | 173 | hg19 | chr14 | 105246551 | 105246551 | Missense_Mutation | SNP | C | C | T | novel | Somatic | E17K | 0.383588 | NM_001014432.1 | 523 | 322 | 201 |
| 768-PTsup | UNC13C | 15 | hg19 | chr15 | 54919052 | 54919052 | Missense_Mutation | SNP | G | G | A | novel | Somatic | G2129E | 0.184615 | NM_001080534.2 | 324 | 264 | 60 |
| 768-PTsup | TRIP4 | 1078 | hg19 | chr15 | 64747217 | 64747217 | Missense_Mutation | SNP | A | A | G | novel | Somatic | H567R | 0.354582 | NM_016213.5 | 251 | 162 | 89 |
| 768-PTsup | GOLGA6B | 1141 | hg19 | chr15 | 72954811 | 72954811 | Nonsense_Mutation | SNP | G | G | T | novel | Somatic | E356Ter | 0.305242 | NM_018652.4 | 970 | 673 | 297 |
| 768-PTsup | SCNN1B | 95 | hg19 | chr16 | 23360214 | 23360214 | Nonsense_Mutation | SNP | C | C | A | novel | Somatic | C98Ter | 0.488372 | NM_000336.3 | 516 | 264 | 252 |
| 768-PTsup | MAZ | 812 | hg19 | chr16 | 29818053 | 29818053 | Frame_Shift_Ins | INS | - | - | C | novel | Somatic | F14LfsTer34 | 0.020522 | NM_002383.4 | 533 | 522 | 11 |
| 768-PTsup | ZFHX3 | 142 | hg19 | chr16 | 72828114 | 72828114 | Missense_Mutation | SNP | G | G | C | novel | Somatic | L2823V | 0.462745 | NM_006885.4 | 510 | 274 | 236 |
| 768-PTsup | CPHXL | 145 | hg19 | chr16 | 75748362 | 75748362 | Frame_Shift_Del | DEL | CT | CT | - | novel | Somatic | E326GfsTer36 | 0.181818 | NM_001355613.1 | 439 | 359 | 80 |
| 768-PTsup | ABR | 593 | hg19 | chr17 | 1028596 | 1028596 | In_Frame_Del | DEL | CATGGTGGGCGACTCATCGATGTACGG | CATGGTGGGCGACTCATCGATGTACGG | - | novel | Somatic | P307_M315del | 0.055675 | NM_001322841.2 | 466 | 440 | 26 |
| 768-PTsup | RPA1 | 598 | hg19 | chr17 | 1747967 | 1747967 | Missense_Mutation | SNP | C | C | G | novel | Somatic | L87V | 0.20915 | NM_002945.5 | 306 | 242 | 64 |
| 768-PTsup | PLD2 | 77 | hg19 | chr17 | 4713302 | 4713302 | Missense_Mutation | SNP | G | G | A | novel | Somatic | V280M | 0.254786 | NM_002663.5 | 679 | 506 | 173 |
| 768-PTsup | NUP88 | 625 | hg19 | chr17 | 5294965 | 5294965 | Missense_Mutation | SNP | C | C | T | novel | Somatic | R467Q | 0.116667 | NM_002532.6 | 179 | 158 | 21 |
| 768-PTsup | TP53 | 642 | hg19 | chr17 | 7578406 | 7578406 | Missense_Mutation | SNP | C | C | T | novel | Somatic | R175H | 0.446746 | NM_000546.5 | 676 | 374 | 302 |
| 768-PTsup | MYH8 | 663 | hg19 | chr17 | 10302977 | 10302977 | Missense_Mutation | SNP | C | C | T | novel | Somatic | E1249K | 0.178423 | NM_002472.3 | 241 | 198 | 43 |
| 768-PTsup | MPRIP | 89 | hg19 | chr17 | 17070310 | 17070310 | Missense_Mutation | SNP | C | C | T | novel | Somatic | S1834F | 0.181197 | NM_001364716.2 | 584 | 478 | 106 |
| 768-PTsup | DRG2 | 722 | hg19 | chr17 | 18002994 | 18002994 | Missense_Mutation | SNP | G | G | A | novel | Somatic | V142I | 0.231317 | NM_001388.5 | 562 | 432 | 130 |
| 768-PTsup | KSR1 | 97 | hg19 | chr17 | 25924391 | 25924391 | Missense_Mutation | SNP | C | C | G | novel | Somatic | S466C | 0.56093 | NM_001367810.1 | 1590 | 697 | 893 |
| 768-PTsup | SYNRG | 107 | hg19 | chr17 | 35913322 | 35913322 | Nonsense_Mutation | SNP | G | G | A | novel | Somatic | Q835Ter | 0.511864 | NM_007247.6 | 295 | 144 | 151 |
| 768-PTsup | NAGS | 906 | hg19 | chr17 | 42082220 | 42082220 | Nonsense_Mutation | SNP | C | C | A | novel | Somatic | Y63Ter | 0.227273 | NM_153006.3 | 242 | 187 | 55 |
| 768-PTsup | KIF18B | 913 | hg19 | chr17 | 43005934 | 43005934 | Missense_Mutation | SNP | G | G | C | novel | Somatic | S579C | 0.428023 | NM_001265577.2 | 521 | 298 | 223 |
| 768-PTsup | TLK2 | 130 | hg19 | chr17 | 60655864 | 60655864 | Silent | SNP | G | G | A | novel | Somatic | L405= | 0.02763 | NM_006852.4 | 941 | 915 | 26 |
| 768-PTsup | DDX42 | 16 | hg19 | chr17 | 61895231 | 61895231 | Missense_Mutation | SNP | C | C | T | novel | Somatic | P764S | 0.672048 | NM_007372.3 | 1174 | 383 | 791 |
| 768-PTsup | ABCA5 | 1098 | hg19 | chr17 | 67293416 | 67293416 | Missense_Mutation | SNP | G | G | A | novel | Somatic | S451L | 0.139966 | NM_018672.4 | 592 | 509 | 83 |
| 768-PTsup | ZNF750 | 1201 | hg19 | chr17 | 80790328 | 80790328 | Translation_Start_Site | SNP | C | C | A | novel | Somatic | M1? | 0.681445 | NM_024702.3 | 609 | 194 | 415 |
| 768-PTsup | TYMS | 590 | hg19 | chr18 | 672903 | 672903 | Missense_Mutation | SNP | G | G | A | novel | Somatic | R283Q | 0.185738 | NM_001071.4 | 603 | 491 | 112 |
| 768-PTsup | TTC39C | 750 | hg19 | chr18 | 21645958 | 21645958 | Intron | DEL | TTCT | TTCT | - | novel | Somatic |  | 0.09571 | NM_001135993.2 | 303 | 274 | 29 |
| 768-PTsup | DSEL | 1082 | hg19 | chr18 | 65179606 | 65179606 | Missense_Mutation | SNP | C | C | A | novel | Somatic | R747L | 0.402844 | NM_032160.3 | 420 | 250 | 170 |
| 768-PTsup | DSEL | 1082 | hg19 | chr18 | 65180867 | 65180867 | Missense_Mutation | SNP | C | C | G | novel | Somatic | V327L | 0.440087 | NM_032160.3 | 459 | 257 | 202 |
| 768-PTsup | ANKRD24 | 9 | hg19 | chr19 | 4217531 | 4217531 | Missense_Mutation | SNP | G | G | C | novel | Somatic | E792Q | 0.122449 | NM_133475.1 | 294 | 258 | 36 |
| 768-PTsup | ANKRD24 | 9 | hg19 | chr19 | 4217603 | 4217603 | Missense_Mutation | SNP | G | G | A | novel | Somatic | D816N | 0.174026 | NM_133475.1 | 384 | 317 | 67 |
| 768-PTsup | RGL3 | 672 | hg19 | chr19 | 11513325 | 11513325 | Splice_Site | SNP | C | C | A | novel | Somatic |  | 0.184576 | NM_001161616.3 | 791 | 645 | 146 |
| 768-PTsup | CNN1 | 673 | hg19 | chr19 | 11658613 | 11658613 | Missense_Mutation | SNP | C | C | T | novel | Somatic | A131V | 0.144258 | NM_001299.6 | 714 | 611 | 103 |
| 768-PTsup | CPAMD8 | 714 | hg19 | chr19 | 17008795 | 17008795 | Missense_Mutation | SNP | C | C | T | novel | Somatic | R1620Q | 0.254516 | NM_015692.5 | 609 | 454 | 155 |
| 768-PTsup | NCAN | 732 | hg19 | chr19 | 19338285 | 19338285 | Nonsense_Mutation | SNP | G | G | A | novel | Somatic | W619Ter | 0.138322 | NM_004386.3 | 882 | 760 | 122 |
| 768-PTsup | ZNF729 | 756 | hg19 | chr19 | 22496725 | 22496725 | Missense_Mutation | SNP | G | G | C | novel | Somatic | R169T | 0.147766 | NM_001242680.2 | 291 | 248 | 43 |
| 768-PTsup | ZNF726 | 768 | hg19 | chr19 | 24116590 | 24116590 | Missense_Mutation | SNP | A | A | G | novel | Somatic | I558V | 0.1 | NM_001244038.2 | 440 | 396 | 44 |
| 768-PTsup | URI1 | 817 | hg19 | chr19 | 30500119 | 30500119 | In_Frame_Del | DEL | TGA | TGA | - | novel | Somatic | D311del | 0.040179 | NM_003796.3 | 668 | 641 | 27 |
| 768-PTsup | ECH1 | 110 | hg19 | chr19 | 39322031 | 39322031 | Missense_Mutation | SNP | C | C | G | novel | Somatic | V60L | 0.274847 | NM_001398.3 | 815 | 591 | 224 |
| 768-PTsup | LGALS14 | 891 | hg19 | chr19 | 40197975 | 40197975 | Missense_Mutation | SNP | G | G | A | novel | Somatic | E84K | 0.23057 | NM_020129.3 | 385 | 296 | 89 |
| 768-PTsup | ZNF296 | 932 | hg19 | chr19 | 45574867 | 45574867 | Missense_Mutation | SNP | C | C | T | novel | Somatic | E474K | 0.042076 | NM_145288.3 | 713 | 683 | 30 |
| 768-PTsup | RTN2 | 935 | hg19 | chr19 | 45998093 | 45998093 | Missense_Mutation | SNP | G | G | A | novel | Somatic | R84C | 0.161716 | NM_005619.5 | 1212 | 1016 | 196 |
| 768-PTsup | GPR32 | 976 | hg19 | chr19 | 51274575 | 51274575 | Missense_Mutation | SNP | C | C | T | novel | Somatic | R240W | 0.112112 | NM_001506.2 | 999 | 887 | 112 |
| 768-PTsup | SIGLEC8 | 981 | hg19 | chr19 | 51958723 | 51958723 | Missense_Mutation | SNP | G | G | T | novel | Somatic | Q334K | 0.137835 | NM_014442.3 | 1081 | 932 | 149 |
| 768-PTsup | ZNF614 | 985 | hg19 | chr19 | 52521681 | 52521681 | Missense_Mutation | SNP | C | C | T | novel | Somatic | A28T | 0.116197 | NM_025040.4 | 283 | 250 | 33 |
| 768-PTsup | ZNF616 | 986 | hg19 | chr19 | 52620014 | 52620014 | Missense_Mutation | SNP | G | G | C | novel | Somatic | H135D | 0.17782 | NM_178523.5 | 523 | 430 | 93 |
| 768-PTsup | ZNF611 | 123 | hg19 | chr19 | 53209932 | 53209932 | Missense_Mutation | SNP | C | C | T | novel | Somatic | E126K | 0.17364 | NM_030972.3 | 955 | 789 | 166 |
| 768-PTsup | NLRP2 | 1008 | hg19 | chr19 | 55494912 | 55494912 | Missense_Mutation | SNP | G | G | C | novel | Somatic | E616Q | 0.332005 | NM_017852.5 | 751 | 501 | 250 |
| 768-PTsup | TTPAL | 114 | hg19 | chr20 | 43108823 | 43108823 | Missense_Mutation | SNP | C | C | T | novel | Somatic | R62C | 0.143023 | NM_024331.5 | 860 | 737 | 123 |
| 768-PTsup | CDH4 | 130 | hg19 | chr20 | 60348207 | 60348207 | Missense_Mutation | SNP | C | C | T | novel | Somatic | S182L | 0.046674 | NM_001794.5 | 856 | 816 | 40 |
| 768-PTsup | OLIG2 | 847 | hg19 | chr21 | 34399954 | 34399954 | Missense_Mutation | SNP | A | A | G | novel | Somatic | K262E | 0.194444 | NM_005806.4 | 252 | 203 | 49 |
| 768-PTsup | PRODH | 729 | hg19 | chr22 | 18907113 | 18907113 | Splice_Region | SNP | G | G | C | novel | Somatic |  | 0.139771 | NM_016335.5 | 787 | 677 | 110 |
| 768-PTsup | SHROOM4 | 121 | hg19 | chrX | 50438919 | 50438919 | Missense_Mutation | SNP | C | C | T | novel | Somatic | A46T | 0.601064 | NM_020717.3 | 188 | 75 | 113 |
| 768-PTsup | NAP1L2 | 1137 | hg19 | chrX | 72433499 | 72433499 | Nonsense_Mutation | SNP | G | G | C | novel | Somatic | S277Ter | 0.219595 | NM_021963.4 | 296 | 231 | 65 |
| 768-PTsup | COL4A5 | 175 | hg19 | chrX | 107867491 | 107867491 | Missense_Mutation | SNP | A | A | T | novel | Somatic | K981N | 0.344538 | NM_033380.3 | 238 | 156 | 82 |
| 768-PTsup | RBMXL3 | 182 | hg19 | chrX | 114426351 | 114426351 | Nonsense_Mutation | SNP | C | C | T | novel | Somatic | R783Ter | 0.367188 | NM_001145346.1 | 767 | 485 | 282 |
| 768-PTsup | CUL4B | 187 | hg19 | chrX | 119676848 | 119676848 | Missense_Mutation | SNP | C | C | T | novel | Somatic | E451K | 0.664706 | NM_003588.3 | 340 | 114 | 226 |
| 768-PTdeep | AJAP1 | 77 | hg19 | chr1 | 4772321 | 4772321 | Missense_Mutation | SNP | G | G | A | novel | Somatic | A131T | 0.089455 | NM_018836.4 | 1370 | 1247 | 123 |
| 768-PTdeep | TNFRSF1B | 678 | hg19 | chr1 | 12248919 | 12248919 | Missense_Mutation | SNP | A | A | G | novel | Somatic | T49A | 0.256244 | NM_001066.3 | 1079 | 802 | 277 |
| 768-PTdeep | CDC42 | 94 | hg19 | chr1 | 22413346 | 22413346 | Missense_Mutation | SNP | C | C | G | novel | Somatic | S158C | 0.055462 | NM_001791.4 | 594 | 561 | 33 |
| 768-PTdeep | RABGGTB | 1166 | hg19 | chr1 | 76255688 | 76255688 | Nonsense_Mutation | SNP | G | G | T | novel | Somatic | E121Ter | 0.090476 | NM_004582.4 | 839 | 763 | 76 |
| 768-PTdeep | IGSF9 | 225 | hg19 | chr1 | 159897582 | 159897582 | Missense_Mutation | SNP | G | G | A | novel | Somatic | S1109F | 0.15619 | NM_001135050.2 | 524 | 442 | 82 |
| 768-PTdeep | AXDND1 | 244 | hg19 | chr1 | 179380303 | 179380303 | Frame_Shift_Del | DEL | TT | TT | - | novel | Somatic | L378IfsTer11 | 0.099278 | NM_144696.6 | 552 | 497 | 55 |
| 768-PTdeep | HMCN1 | 250 | hg19 | chr1 | 185966644 | 185966644 | Missense_Mutation | SNP | T | T | C | novel | Somatic | C1288R | 0.061728 | NM_031935.3 | 405 | 380 | 25 |
| 768-PTdeep | IGFN1 | 264 | hg19 | chr1 | 201185708 | 201185708 | Missense_Mutation | SNP | G | G | A | novel | Somatic | R3141Q | 0.18012 | NM_001164586.2 | 1335 | 1094 | 241 |
| 768-PTdeep | SLC41A1 | 2154 | hg19 | chr1 | 205779283 | 205779283 | Missense_Mutation | SNP | A | A | C | novel | Somatic | F96C | 0.121242 | NM_173854.6 | 998 | 877 | 121 |
| 768-PTdeep | CCDC185 | 2290 | hg19 | chr1 | 223568412 | 223568412 | Missense_Mutation | SNP | T | T | A | novel | Somatic | L532H | 0.198391 | NM_152610.3 | 1118 | 896 | 222 |
| 768-PTdeep | OBSCN | 290 | hg19 | chr1 | 228560408 | 228560408 | Nonsense_Mutation | SNP | C | C | G | novel | Somatic | S8267Ter | 0.096324 | NM_001271223.2 | 786 | 710 | 76 |
| 768-PTdeep | LYST | 2385 | hg19 | chr1 | 235875399 | 235875399 | Missense_Mutation | SNP | G | G | T | novel | Somatic | P3295T | 0.086823 | NM_001301365.1 | 975 | 890 | 85 |
| 768-PTdeep | KIF26B | 38 | hg19 | chr1 | 245851667 | 245851667 | In_Frame_Del | DEL | CCTGGCCTCCAAGCTTCC | CCTGGCCTCCAAGCTTCC | - | novel | Somatic | A1796_L1801del | 0.034667 | NM_018012.4 | 1122 | 1083 | 39 |
| 768-PTdeep | SOX11 | 629 | hg19 | chr2 | 5833962 | 5833962 | Missense_Mutation | SNP | C | C | G | novel | Somatic | S370C | 0.307692 | NM_003108.4 | 792 | 548 | 244 |
| 768-PTdeep | FNDC4 | 796 | hg19 | chr2 | 27717516 | 27717516 | Frame_Shift_Ins | INS | - | - | G | novel | Somatic | S11QfsTer28 | 0.031421 | NM_022823.3 | 732 | 709 | 23 |
| 768-PTdeep | PLEK | 1108 | hg19 | chr2 | 68622898 | 68622898 | Missense_Mutation | SNP | C | C | T | novel | Somatic | R335C | 0.105495 | NM_002664.3 | 454 | 406 | 48 |
| 768-PTdeep | C2orf81 | 1154 | hg19 | chr2 | 74642696 | 74642696 | Missense_Mutation | SNP | C | C | T | novel | Somatic | R203Q | 0.343415 | NM_001316764.3 | 1025 | 673 | 352 |
| 768-PTdeep | NT5DC4 | 1450 | hg19 | chr2 | 113480080 | 113480080 | Missense_Mutation | SNP | A | A | G | novel | Somatic | E102G | 0.231944 | NM_001350494.2 | 720 | 553 | 167 |
| 768-PTdeep | LCT | 203 | hg19 | chr2 | 136570058 | 136570058 | Missense_Mutation | SNP | G | G | A | novel | Somatic | R726C | 0.254254 | NM_002299.4 | 999 | 745 | 254 |
| 768-PTdeep | LRP1B | 3 | hg19 | chr2 | 141267521 | 141267521 | Missense_Mutation | SNP | C | C | T | novel | Somatic | D2792N | 0.225275 | NM_018557.3 | 546 | 423 | 123 |
| 768-PTdeep | METAP1D | 237 | hg19 | chr2 | 172942627 | 172942627 | Splice_Region | SNP | G | G | A | novel | Somatic |  | 0.047934 | NM_199227.3 | 605 | 576 | 29 |
| 768-PTdeep | PDE1A | 1981 | hg19 | chr2 | 183104988 | 183104988 | Missense_Mutation | SNP | C | C | T | novel | Somatic | D87N | 0.22252 | NM_001258312.1 | 746 | 580 | 166 |
| 768-PTdeep | WDR12 | 2139 | hg19 | chr2 | 203765843 | 203765843 | Splice_Site | SNP | C | C | G | novel | Somatic |  | 0.077325 | NM_018256.4 | 955 | 881 | 74 |
| 768-PTdeep | MAP2 | 273 | hg19 | chr2 | 210543334 | 210543334 | Missense_Mutation | SNP | G | G | A | novel | Somatic | E101K | 0.043353 | NM_002374.3 | 1037 | 992 | 45 |
| 768-PTdeep | USP37 | 282 | hg19 | chr2 | 219341589 | 219341589 | Missense_Mutation | SNP | C | C | T | novel | Somatic | E673K | 0.09465 | NM_020935.3 | 729 | 660 | 69 |
| 768-PTdeep | USP40 | 2373 | hg19 | chr2 | 234386078 | 234386078 | Missense_Mutation | SNP | G | G | A | novel | Somatic | P1224L | 0.203065 | NM_001365479.1 | 782 | 623 | 159 |
| 768-PTdeep | OTOS | 2424 | hg19 | chr2 | 241078760 | 241078760 | Missense_Mutation | SNP | C | C | T | novel | Somatic | E33K | 0.122288 | NM_148961.4 | 507 | 445 | 62 |
| 768-PTdeep | BAP1 | 985 | hg19 | chr3 | 52443586 | 52443586 | Nonsense_Mutation | SNP | G | G | A | novel | Somatic | Q36Ter | 0.28047 | NM_004656.4 | 678 | 487 | 191 |
| 768-PTdeep | ERC2 | 15 | hg19 | chr3 | 56330423 | 56330423 | Missense_Mutation | SNP | C | C | T | novel | Somatic | R233Q | 0.103211 | NM_015576.3 | 872 | 782 | 90 |
| 768-PTdeep | OR5H14 | 1331 | hg19 | chr3 | 97868484 | 97868484 | Missense_Mutation | SNP | C | C | G | novel | Somatic | F85L | 0.074224 | NM_001005514.1 | 1481 | 1371 | 110 |
| 768-PTdeep | HTR3E | 1987 | hg19 | chr3 | 183823111 | 183823111 | Missense_Mutation | SNP | C | C | T | novel | Somatic | R232W | 0.035109 | NM_001256614.1 | 824 | 795 | 29 |
| 768-PTdeep | EIF4G1 | 1989 | hg19 | chr3 | 184042091 | 184042091 | Missense_Mutation | SNP | G | G | C | novel | Somatic | E866Q | 0.224368 | NM_001194947.1 | 673 | 522 | 151 |
| 768-PTdeep | FETUB | 2006 | hg19 | chr3 | 186362544 | 186362544 | Frame_Shift_Del | DEL | A | A | - | novel | Somatic | K146RfsTer4 | 0.030686 | NM_014375.3 | 554 | 537 | 17 |
| 768-PTdeep | MUC4 | 2076 | hg19 | chr3 | 195512594 | 195512594 | Missense_Mutation | SNP | G | G | A | novel | Somatic | L1953F | 0.165576 | NM_018406.7 | 3659 | 3052 | 607 |
| 768-PTdeep | NKX1-1 | 595 | hg19 | chr4 | 1399930 | 1399930 | Missense_Mutation | SNP | C | C | T | novel | Somatic | V101M | 0.051873 | NM_001290079.1 | 347 | 329 | 18 |
| 768-PTdeep | CCDC96 | 638 | hg19 | chr4 | 7043867 | 7043867 | Missense_Mutation | SNP | C | C | T | novel | Somatic | E267K | 0.245399 | NM_153376.3 | 815 | 615 | 200 |
| 768-PTdeep | WDR19 | 110 | hg19 | chr4 | 39206803 | 39206803 | Frame_Shift_Ins | INS | - | - | T | novel | Somatic | L214FfsTer5 | 0.143836 | NM_025132.4 | 583 | 499 | 84 |
| 768-PTdeep | FAM13A | 158 | hg19 | chr4 | 89671659 | 89671659 | Missense_Mutation | SNP | C | C | T | novel | Somatic | R619Q | 0.115124 | NM_014883.4 | 884 | 782 | 102 |
| 768-PTdeep | USP38 | 1684 | hg19 | chr4 | 144116919 | 144116919 | Missense_Mutation | SNP | T | T | C | novel | Somatic | V290A | 0.260766 | NM_032557.6 | 418 | 309 | 109 |
| 768-PTdeep | TENM3 | 30 | hg19 | chr4 | 183721137 | 183721137 | Missense_Mutation | SNP | C | C | T | novel | Somatic | T2578M | 0.051844 | NM_001080477.4 | 1003 | 951 | 52 |
| 768-PTdeep | SORBS2 | 250 | hg19 | chr4 | 186544674 | 186544674 | Missense_Mutation | SNP | C | C | T | novel | Somatic | E733K | 0.228407 | NM_001270771.2 | 1042 | 804 | 238 |
| 768-PTdeep | H3.Y | 719 | hg19 | chr5 | 17655622 | 17655622 | Missense_Mutation | SNP | C | C | T | novel | Somatic | R9H | 0.400854 | NM_001355258.2 | 2574 | 1541 | 1033 |
| 768-PTdeep | CDH10 | 96 | hg19 | chr5 | 24491793 | 24491793 | Missense_Mutation | SNP | C | C | T | novel | Somatic | D590N | 0.141522 | NM_006727.5 | 749 | 643 | 106 |
| 768-PTdeep | AGXT2 | 852 | hg19 | chr5 | 35035385 | 35035385 | Missense_Mutation | SNP | C | C | T | novel | Somatic | E175K | 0.069767 | NM_031900.4 | 817 | 760 | 57 |
| 768-PTdeep | ZNF608 | 191 | hg19 | chr5 | 123980275 | 123980275 | Missense_Mutation | SNP | C | C | T | novel | Somatic | R1262K | 0.296928 | NM_020747.2 | 586 | 412 | 174 |
| 768-PTdeep | GRIA1 | 27 | hg19 | chr5 | 153035421 | 153035421 | Missense_Mutation | SNP | C | C | T | novel | Somatic | L230F | 0.291581 | NM_000827.4 | 487 | 345 | 142 |
| 768-PTdeep | ATXN1 | 88 | hg19 | chr6 | 16327912 | 16327912 | Missense_Mutation | SNP | C | C | A | novel | Somatic | Q210H | 0.049355 | NM_000332.3 | 2675 | 2541 | 134 |
| 768-PTdeep | FAM8A1 | 719 | hg19 | chr6 | 17600647 | 17600647 | Missense_Mutation | SNP | G | G | A | novel | Somatic | E3K | 0.108747 | NM_016255.3 | 422 | 376 | 46 |
| 768-PTdeep | DNAH8 | 13 | hg19 | chr6 | 38825390 | 38825390 | Missense_Mutation | SNP | C | C | A | novel | Somatic | Q1944K | 0.201566 | NM_001206927.2 | 511 | 408 | 103 |
| 768-PTdeep | COL19A1 | 140 | hg19 | chr6 | 70639566 | 70639566 | Nonsense_Mutation | SNP | C | C | T | novel | Somatic | R214Ter | 0.148402 | NM_001858.6 | 437 | 372 | 65 |
| 768-PTdeep | SYNE1 | 1748 | hg19 | chr6 | 152527355 | 152527355 | Missense_Mutation | SNP | G | G | A | novel | Somatic | S7656L | 0.08245 | NM_182961.4 | 848 | 778 | 70 |
| 768-PTdeep | ARID1B | 27 | hg19 | chr6 | 157099403 | 157099403 | In_Frame_Del | DEL | CAGCAGCAGCAGCAGCAGCAGCAA | CAGCAGCAGCAGCAGCAGCAGCAA | - | novel | Somatic | Q124_Q131del | 0.031896 | NM_020732.3 | 3189 | 3084 | 105 |
| 768-PTdeep | FNDC1 | 225 | hg19 | chr6 | 159672439 | 159672439 | Missense_Mutation | SNP | A | A | T | novel | Somatic | D1647V | 0.087989 | NM_032532.3 | 716 | 653 | 63 |
| 768-PTdeep | LPA | 226 | hg19 | chr6 | 160952810 | 160952810 | Missense_Mutation | SNP | C | C | T | novel | Somatic | R2025H | 0.183079 | NM_005577.4 | 1440 | 1176 | 264 |
| 768-PTdeep | PRR18 | 1856 | hg19 | chr6 | 166721128 | 166721128 | Missense_Mutation | SNP | G | G | C | novel | Somatic | S168W | 0.115646 | NM_175922.4 | 441 | 390 | 51 |
| 768-PTdeep | UNC93A | 1864 | hg19 | chr6 | 167711503 | 167711503 | Missense_Mutation | SNP | C | C | A | novel | Somatic | N190K | 0.170282 | NM_018974.4 | 922 | 765 | 157 |
| 768-PTdeep | CYP2W1 | 592 | hg19 | chr7 | 1024112 | 1024112 | Missense_Mutation | SNP | C | C | T | novel | Somatic | T80M | 0.102972 | NM_017781.3 | 1447 | 1298 | 149 |
| 768-PTdeep | SDK1 | 9 | hg19 | chr7 | 4272926 | 4272926 | Missense_Mutation | SNP | G | G | A | novel | Somatic | R1956Q | 0.091362 | NM_152744.4 | 1204 | 1094 | 110 |
| 768-PTdeep | DNAH11 | 93 | hg19 | chr7 | 21913114 | 21913114 | Missense_Mutation | SNP | G | G | C | novel | Somatic | D4064H | 0.110092 | NM_001277115.2 | 545 | 485 | 60 |
| 768-PTdeep | NPY | 770 | hg19 | chr7 | 24324876 | 24324876 | Missense_Mutation | SNP | G | G | A | novel | Somatic | R6Q | 0.046448 | NM_000905.4 | 731 | 697 | 34 |
| 768-PTdeep | GRM3 | 155 | hg19 | chr7 | 86415818 | 86415818 | Missense_Mutation | SNP | G | G | A | novel | Somatic | R237H | 0.234637 | NM_000840.2 | 1253 | 959 | 294 |
| 768-PTdeep | KMT2E | 172 | hg19 | chr7 | 104707230 | 104707230 | Missense_Mutation | SNP | A | A | G | novel | Somatic | Y158C | 0.188634 | NM_182931.3 | 827 | 671 | 156 |
| 768-PTdeep | LRRCC1 | 1241 | hg19 | chr8 | 86050700 | 86050700 | Missense_Mutation | SNP | G | G | A | novel | Somatic | E944K | 0.096033 | NM_033402.5 | 477 | 431 | 46 |
| 768-PTdeep | NBN | 159 | hg19 | chr8 | 90967655 | 90967655 | Nonsense_Mutation | SNP | G | G | C | novel | Somatic | S418Ter | 0.314548 | NM_002485.4 | 761 | 521 | 240 |
| 768-PTdeep | VPS13B | 1348 | hg19 | chr8 | 100796636 | 100796636 | Missense_Mutation | SNP | G | G | T | novel | Somatic | D2650Y | 0.244838 | NM_017890.4 | 678 | 512 | 166 |
| 768-PTdeep | PKHD1L1 | 178 | hg19 | chr8 | 110432825 | 110432825 | Missense_Mutation | SNP | C | C | A | novel | Somatic | T868K | 0.053879 | NM_177531.6 | 924 | 874 | 50 |
| 768-PTdeep | ZHX2 | 191 | hg19 | chr8 | 123964798 | 123964798 | Missense_Mutation | SNP | G | G | A | novel | Somatic | A350T | 0.066581 | NM_014943.5 | 1561 | 1457 | 104 |
| 768-PTdeep | FAM135B | 25 | hg19 | chr8 | 139158208 | 139158208 | Missense_Mutation | SNP | C | C | A | novel | Somatic | K1178N | 0.076923 | NM_015912.4 | 546 | 504 | 42 |
| 768-PTdeep | FRMPD1 | 872 | hg19 | chr9 | 37732361 | 37732361 | Nonsense_Mutation | SNP | C | C | T | novel | Somatic | R307Ter | 0.296037 | NM_014907.3 | 428 | 301 | 127 |
| 768-PTdeep | NIPSNAP3B | 1405 | hg19 | chr9 | 107528646 | 107528646 | Missense_Mutation | SNP | G | G | A | novel | Somatic | G34E | 0.251291 | NM_018376.4 | 580 | 434 | 146 |
| 768-PTdeep | KCNT1 | 205 | hg19 | chr9 | 138606490 | 138606490 | Missense_Mutation | SNP | G | G | A | novel | Somatic | E60K | 0.297428 | NM_020822.3 | 622 | 437 | 185 |
| 768-PTdeep | CLIC3 | 1652 | hg19 | chr9 | 139889210 | 139889210 | Missense_Mutation | SNP | C | C | T | novel | Somatic | E212K | 0.148718 | NM_004669.3 | 779 | 663 | 116 |
| 768-PTdeep | SKIDA1 | 751 | hg19 | chr10 | 21805466 | 21805466 | In_Frame_Ins | INS | - | - | CCT | novel | Somatic | E428dup | 0.037267 | NM_207371.4 | 280 | 256 | 24 |
| 768-PTdeep | ALDH18A1 | 165 | hg19 | chr10 | 97387306 | 97387306 | Missense_Mutation | SNP | G | G | T | novel | Somatic | S324Y | 0.113069 | NM_002860.4 | 680 | 603 | 77 |
| 768-PTdeep | SFRP5 | 1344 | hg19 | chr10 | 99531359 | 99531359 | Missense_Mutation | SNP | C | C | G | novel | Somatic | E78Q | 0.248399 | NM_003015.3 | 781 | 587 | 194 |
| 768-PTdeep | FGFR2 | 1525 | hg19 | chr10 | 123263350 | 123263350 | Missense_Mutation | SNP | C | C | T | novel | Somatic | E465K | 0.131902 | NM_000141.4 | 977 | 848 | 129 |
| 768-PTdeep | DMBT1 | 191 | hg19 | chr10 | 124402699 | 124402699 | Missense_Mutation | SNP | C | C | T | novel | Somatic | R2343W | 0.236671 | NM_007329.2 | 768 | 586 | 182 |
| 768-PTdeep | E2F8 | 731 | hg19 | chr11 | 19252218 | 19252218 | Missense_Mutation | SNP | C | C | G | novel | Somatic | K410N | 0.104034 | NM_024680.4 | 471 | 422 | 49 |
| 768-PTdeep | PAX6 | 827 | hg19 | chr11 | 31816252 | 31816252 | Missense_Mutation | SNP | C | C | T | novel | Somatic | R203Q | 0.120466 | NM_000280.4 | 772 | 679 | 93 |
| 768-PTdeep | MYRF | 1054 | hg19 | chr11 | 61539013 | 61539013 | Frame_Shift_Del | DEL | C | C | - | novel | Somatic | S264AfsTer8 | 0.023887 | NM_001127392.3 | 919 | 897 | 22 |
| 768-PTdeep | AHNAK | 1060 | hg19 | chr11 | 62295012 | 62295012 | Missense_Mutation | SNP | C | C | T | novel | Somatic | G2293R | 0.1001 | NM_001620.3 | 999 | 899 | 100 |
| 768-PTdeep | CPT1A | 138 | hg19 | chr11 | 68548137 | 68548137 | Missense_Mutation | SNP | C | C | T | novel | Somatic | D477N | 0.082751 | NM_001876.4 | 858 | 787 | 71 |
| 768-PTdeep | CLPB | 141 | hg19 | chr11 | 72069957 | 72069957 | Missense_Mutation | SNP | C | C | T | novel | Somatic | D248N | 0.320833 | NM_001258392.3 | 719 | 488 | 231 |
| 768-PTdeep | WNT5B | 598 | hg19 | chr12 | 1755207 | 1755207 | Missense_Mutation | SNP | G | G | A | novel | Somatic | R290H | 0.124424 | NM_032642.3 | 1083 | 948 | 135 |
| 768-PTdeep | GNB3 | 638 | hg19 | chr12 | 6952180 | 6952180 | Missense_Mutation | SNP | G | G | A | novel | Somatic | R48Q | 0.212414 | NM_002075.4 | 725 | 571 | 154 |
| 768-PTdeep | USP5 | 638 | hg19 | chr12 | 6972416 | 6972416 | Missense_Mutation | SNP | G | G | A | novel | Somatic | G610E | 0.222222 | NM_001098536.2 | 728 | 566 | 162 |
| 768-PTdeep | KIAA1551 | 103 | hg19 | chr12 | 32138043 | 32138043 | Missense_Mutation | SNP | T | T | G | novel | Somatic | M1385R | 0.111954 | NM_018169.4 | 527 | 468 | 59 |
| 768-PTdeep | ARID2 | 14 | hg19 | chr12 | 46245223 | 46245223 | Missense_Mutation | SNP | C | C | T | novel | Somatic | A1106V | 0.117647 | NM_152641.4 | 594 | 524 | 70 |
| 768-PTdeep | ANO4 | 169 | hg19 | chr12 | 101336303 | 101336303 | Missense_Mutation | SNP | T | T | G | novel | Somatic | M149R | 0.086455 | NM_001286615.2 | 693 | 633 | 60 |
| 768-PTdeep | FOXN4 | 1422 | hg19 | chr12 | 109725301 | 109725301 | Frame_Shift_Del | DEL | G | G | - | novel | Somatic | P166HfsTer36 | 0.11526 | NM_213596.3 | 616 | 545 | 71 |
| 768-PTdeep | RXFP2 | 103 | hg19 | chr13 | 32339215 | 32339215 | Missense_Mutation | SNP | G | G | A | novel | Somatic | E121K | 0.133733 | NM_130806.5 | 1001 | 867 | 134 |
| 768-PTdeep | UGGT2 | 20 | hg19 | chr13 | 96599314 | 96599314 | Missense_Mutation | SNP | C | C | T | novel | Somatic | E552K | 0.11615 | NM_020121.4 | 902 | 797 | 105 |
| 768-PTdeep | RNASE9 | 745 | hg19 | chr14 | 21024652 | 21024652 | Missense_Mutation | SNP | T | T | G | novel | Somatic | S198R | 0.078692 | NM_001110359.1 | 826 | 761 | 65 |
| 768-PTdeep | PRPF39 | 932 | hg19 | chr14 | 45571797 | 45571797 | Missense_Mutation | SNP | A | A | T | novel | Somatic | Y212F | 0.074795 | NM_017922.4 | 976 | 903 | 73 |
| 768-PTdeep | SPATA7 | 157 | hg19 | chr14 | 88859755 | 88859755 | Missense_Mutation | SNP | C | C | T | novel | Somatic | S38F | 0.235005 | NM_018418.5 | 1016 | 777 | 239 |
| 768-PTdeep | PTPN21 | 157 | hg19 | chr14 | 88945628 | 88945628 | In_Frame_Del | DEL | TCC | TCC | - | novel | Somatic | E715del | 0.026316 | NM_007039.4 | 720 | 701 | 19 |
| 768-PTdeep | AKT1 | 173 | hg19 | chr14 | 105246551 | 105246551 | Missense_Mutation | SNP | C | C | T | novel | Somatic | E17K | 0.243762 | NM_001014432.1 | 521 | 394 | 127 |
| 768-PTdeep | UNC13C | 15 | hg19 | chr15 | 54919052 | 54919052 | Missense_Mutation | SNP | G | G | A | novel | Somatic | G2129E | 0.114701 | NM_001080534.2 | 617 | 546 | 71 |
| 768-PTdeep | TRIP4 | 1078 | hg19 | chr15 | 64747217 | 64747217 | Missense_Mutation | SNP | A | A | G | novel | Somatic | H567R | 0.207983 | NM_016213.5 | 475 | 376 | 99 |
| 768-PTdeep | GOLGA6B | 1141 | hg19 | chr15 | 72954811 | 72954811 | Nonsense_Mutation | SNP | G | G | T | novel | Somatic | E356Ter | 0.183348 | NM_018652.4 | 1126 | 919 | 207 |
| 768-PTdeep | SCNN1B | 95 | hg19 | chr16 | 23360214 | 23360214 | Nonsense_Mutation | SNP | C | C | A | novel | Somatic | C98Ter | 0.276639 | NM_000336.3 | 488 | 353 | 135 |
| 768-PTdeep | ZFHX3 | 142 | hg19 | chr16 | 72828114 | 72828114 | Missense_Mutation | SNP | G | G | C | novel | Somatic | L2823V | 0.26875 | NM_006885.4 | 640 | 468 | 172 |
| 768-PTdeep | CPHXL | 145 | hg19 | chr16 | 75748362 | 75748362 | Frame_Shift_Del | DEL | CT | CT | - | novel | Somatic | E326GfsTer36 | 0.130282 | NM_001355613.1 | 568 | 494 | 74 |
| 768-PTdeep | ABR | 593 | hg19 | chr17 | 1028596 | 1028596 | In_Frame_Del | DEL | CATGGTGGGCGACTCATCGATGTACGG | CATGGTGGGCGACTCATCGATGTACGG | - | novel | Somatic | P307_M315del | 0.028967 | NM_001322841.2 | 792 | 769 | 23 |
| 768-PTdeep | WDR81 | 597 | hg19 | chr17 | 1631749 | 1631749 | In_Frame_Del | DEL | GAG | GAG | - | novel | Somatic | E1169del | 0.020619 | NM_001163809.1 | 970 | 950 | 20 |
| 768-PTdeep | RPA1 | 598 | hg19 | chr17 | 1747967 | 1747967 | Missense_Mutation | SNP | C | C | G | novel | Somatic | L87V | 0.118774 | NM_002945.5 | 522 | 460 | 62 |
| 768-PTdeep | PLD2 | 77 | hg19 | chr17 | 4713302 | 4713302 | Missense_Mutation | SNP | G | G | A | novel | Somatic | V280M | 0.131792 | NM_002663.5 | 865 | 751 | 114 |
| 768-PTdeep | TP53 | 642 | hg19 | chr17 | 7578406 | 7578406 | Missense_Mutation | SNP | C | C | T | novel | Somatic | R175H | 0.272081 | NM_000546.5 | 985 | 717 | 268 |
| 768-PTdeep | MYH8 | 663 | hg19 | chr17 | 10302977 | 10302977 | Missense_Mutation | SNP | C | C | T | novel | Somatic | E1249K | 0.132159 | NM_002472.3 | 454 | 394 | 60 |
| 768-PTdeep | MPRIP | 89 | hg19 | chr17 | 17070310 | 17070310 | Missense_Mutation | SNP | C | C | T | novel | Somatic | S1834F | 0.102964 | NM_001364716.2 | 640 | 574 | 66 |
| 768-PTdeep | DRG2 | 722 | hg19 | chr17 | 18002994 | 18002994 | Missense_Mutation | SNP | G | G | A | novel | Somatic | V142I | 0.128302 | NM_001388.5 | 795 | 693 | 102 |
| 768-PTdeep | KSR1 | 97 | hg19 | chr17 | 25924391 | 25924391 | Missense_Mutation | SNP | C | C | G | novel | Somatic | S466C | 0.33999 | NM_001367810.1 | 1951 | 1287 | 664 |
| 768-PTdeep | SYNRG | 107 | hg19 | chr17 | 35913322 | 35913322 | Nonsense_Mutation | SNP | G | G | A | novel | Somatic | Q835Ter | 0.280672 | NM_007247.6 | 595 | 428 | 167 |
| 768-PTdeep | NAGS | 906 | hg19 | chr17 | 42082220 | 42082220 | Nonsense_Mutation | SNP | C | C | A | novel | Somatic | Y63Ter | 0.142494 | NM_153006.3 | 392 | 336 | 56 |
| 768-PTdeep | KIF18B | 913 | hg19 | chr17 | 43005934 | 43005934 | Missense_Mutation | SNP | G | G | C | novel | Somatic | S579C | 0.275103 | NM_001265577.2 | 726 | 526 | 200 |
| 768-PTdeep | DDX42 | 16 | hg19 | chr17 | 61895231 | 61895231 | Missense_Mutation | SNP | C | C | T | novel | Somatic | P764S | 0.489194 | NM_007372.3 | 1016 | 518 | 498 |
| 768-PTdeep | ABCA5 | 1098 | hg19 | chr17 | 67293416 | 67293416 | Missense_Mutation | SNP | G | G | A | novel | Somatic | S451L | 0.055749 | NM_018672.4 | 861 | 813 | 48 |
| 768-PTdeep | SYNGR2 | 1166 | hg19 | chr17 | 76167813 | 76167813 | Missense_Mutation | SNP | C | C | T | novel | Somatic | P187L | 0.030651 | NM_001363778.1 | 1043 | 1011 | 32 |
| 768-PTdeep | ZNF750 | 1201 | hg19 | chr17 | 80790328 | 80790328 | Translation_Start_Site | SNP | C | C | A | novel | Somatic | M1? | 0.479315 | NM_024702.3 | 700 | 364 | 336 |
| 768-PTdeep | TYMS | 590 | hg19 | chr18 | 672903 | 672903 | Missense_Mutation | SNP | G | G | A | novel | Somatic | R283Q | 0.110874 | NM_001071.4 | 937 | 833 | 104 |
| 768-PTdeep | TTC39C | 750 | hg19 | chr18 | 21645958 | 21645958 | Intron | DEL | TTCT | TTCT | - | novel | Somatic |  | 0.055556 | NM_001135993.2 | 432 | 408 | 24 |
| 768-PTdeep | DSEL | 1082 | hg19 | chr18 | 65179606 | 65179606 | Missense_Mutation | SNP | C | C | A | novel | Somatic | R747L | 0.226744 | NM_032160.3 | 686 | 530 | 156 |
| 768-PTdeep | DSEL | 1082 | hg19 | chr18 | 65180867 | 65180867 | Missense_Mutation | SNP | C | C | G | novel | Somatic | V327L | 0.213483 | NM_032160.3 | 712 | 560 | 152 |
| 768-PTdeep | ANKRD24 | 9 | hg19 | chr19 | 4217531 | 4217531 | Missense_Mutation | SNP | G | G | C | novel | Somatic | E792Q | 0.101167 | NM_133475.1 | 514 | 462 | 52 |
| 768-PTdeep | ANKRD24 | 9 | hg19 | chr19 | 4217603 | 4217603 | Missense_Mutation | SNP | G | G | A | novel | Somatic | D816N | 0.099698 | NM_133475.1 | 661 | 595 | 66 |
| 768-PTdeep | RGL3 | 672 | hg19 | chr19 | 11513325 | 11513325 | Splice_Site | SNP | C | C | A | novel | Somatic |  | 0.099355 | NM_001161616.3 | 774 | 697 | 77 |
| 768-PTdeep | CNN1 | 673 | hg19 | chr19 | 11658613 | 11658613 | Missense_Mutation | SNP | C | C | T | novel | Somatic | A131V | 0.082729 | NM_001299.6 | 687 | 630 | 57 |
| 768-PTdeep | CPAMD8 | 714 | hg19 | chr19 | 17008795 | 17008795 | Missense_Mutation | SNP | C | C | T | novel | Somatic | R1620Q | 0.24812 | NM_015692.5 | 798 | 600 | 198 |
| 768-PTdeep | NCAN | 732 | hg19 | chr19 | 19338285 | 19338285 | Nonsense_Mutation | SNP | G | G | A | novel | Somatic | W619Ter | 0.097926 | NM_004386.3 | 868 | 783 | 85 |
| 768-PTdeep | ZNF729 | 756 | hg19 | chr19 | 22496725 | 22496725 | Missense_Mutation | SNP | G | G | C | novel | Somatic | R169T | 0.106635 | NM_001242680.2 | 420 | 375 | 45 |
| 768-PTdeep | ZNF726 | 768 | hg19 | chr19 | 24116590 | 24116590 | Missense_Mutation | SNP | A | A | G | novel | Somatic | I558V | 0.029827 | NM_001244038.2 | 637 | 618 | 19 |
| 768-PTdeep | ECH1 | 110 | hg19 | chr19 | 39322031 | 39322031 | Missense_Mutation | SNP | C | C | G | novel | Somatic | V60L | 0.185507 | NM_001398.3 | 690 | 562 | 128 |
| 768-PTdeep | LGALS14 | 891 | hg19 | chr19 | 40197975 | 40197975 | Missense_Mutation | SNP | G | G | A | novel | Somatic | E84K | 0.173815 | NM_020129.3 | 443 | 366 | 77 |
| 768-PTdeep | ZNF296 | 932 | hg19 | chr19 | 45574867 | 45574867 | Missense_Mutation | SNP | C | C | T | novel | Somatic | E474K | 0.023697 | NM_145288.3 | 844 | 824 | 20 |
| 768-PTdeep | RTN2 | 935 | hg19 | chr19 | 45998093 | 45998093 | Missense_Mutation | SNP | G | G | A | novel | Somatic | R84C | 0.096774 | NM_005619.5 | 1579 | 1426 | 153 |
| 768-PTdeep | GPR32 | 976 | hg19 | chr19 | 51274575 | 51274575 | Missense_Mutation | SNP | C | C | T | novel | Somatic | R240W | 0.087034 | NM_001506.2 | 1124 | 1026 | 98 |
| 768-PTdeep | SIGLEC8 | 981 | hg19 | chr19 | 51958723 | 51958723 | Missense_Mutation | SNP | G | G | T | novel | Somatic | Q334K | 0.089005 | NM_014442.3 | 1145 | 1043 | 102 |
| 768-PTdeep | ZNF614 | 985 | hg19 | chr19 | 52521681 | 52521681 | Missense_Mutation | SNP | C | C | T | novel | Somatic | A28T | 0.099548 | NM_025040.4 | 441 | 397 | 44 |
| 768-PTdeep | ZNF616 | 986 | hg19 | chr19 | 52620014 | 52620014 | Missense_Mutation | SNP | G | G | C | novel | Somatic | H135D | 0.122563 | NM_178523.5 | 718 | 630 | 88 |
| 768-PTdeep | ZNF611 | 123 | hg19 | chr19 | 53209932 | 53209932 | Missense_Mutation | SNP | C | C | T | novel | Somatic | E126K | 0.102429 | NM_030972.3 | 1522 | 1366 | 156 |
| 768-PTdeep | NLRP2 | 1008 | hg19 | chr19 | 55494912 | 55494912 | Missense_Mutation | SNP | G | G | C | novel | Somatic | E616Q | 0.230769 | NM_017852.5 | 754 | 580 | 174 |
| 768-PTdeep | CD93 | 760 | hg19 | chr20 | 23066215 | 23066215 | Missense_Mutation | SNP | G | G | C | novel | Somatic | F205L | 0.045949 | NM_012072.4 | 827 | 789 | 38 |
| 768-PTdeep | TTPAL | 114 | hg19 | chr20 | 43108823 | 43108823 | Missense_Mutation | SNP | C | C | T | novel | Somatic | R62C | 0.078813 | NM_024331.5 | 976 | 899 | 77 |
| 768-PTdeep | CDH4 | 130 | hg19 | chr20 | 60348207 | 60348207 | Missense_Mutation | SNP | C | C | T | novel | Somatic | S182L | 0.040338 | NM_001794.5 | 1066 | 1023 | 43 |
| 768-PTdeep | OLIG2 | 847 | hg19 | chr21 | 34399954 | 34399954 | Missense_Mutation | SNP | A | A | G | novel | Somatic | K262E | 0.173302 | NM_005806.4 | 427 | 353 | 74 |
| 768-PTdeep | PRODH | 729 | hg19 | chr22 | 18907113 | 18907113 | Splice_Region | SNP | G | G | C | novel | Somatic |  | 0.071875 | NM_016335.5 | 960 | 891 | 69 |
| 768-PTdeep | SHROOM4 | 121 | hg19 | chrX | 50438919 | 50438919 | Missense_Mutation | SNP | C | C | T | novel | Somatic | A46T | 0.369048 | NM_020717.3 | 336 | 212 | 124 |
| 768-PTdeep | NAP1L2 | 1137 | hg19 | chrX | 72433499 | 72433499 | Nonsense_Mutation | SNP | G | G | C | novel | Somatic | S277Ter | 0.168539 | NM_021963.4 | 444 | 369 | 75 |
| 768-PTdeep | COL4A5 | 175 | hg19 | chrX | 107867491 | 107867491 | Missense_Mutation | SNP | A | A | T | novel | Somatic | K981N | 0.222222 | NM_033380.3 | 333 | 259 | 74 |
| 768-PTdeep | RBMXL3 | 182 | hg19 | chrX | 114426351 | 114426351 | Nonsense_Mutation | SNP | C | C | T | novel | Somatic | R783Ter | 0.267252 | NM_001145346.1 | 795 | 582 | 213 |
| 768-PTdeep | CUL4B | 187 | hg19 | chrX | 119676848 | 119676848 | Missense_Mutation | SNP | C | C | T | novel | Somatic | E451K | 0.431452 | NM_003588.3 | 495 | 281 | 214 |
| 768-LNmet | AJAP1 | 77 | hg19 | chr1 | 4772321 | 4772321 | Missense_Mutation | SNP | G | G | A | novel | Somatic | A131T | 0.087591 | NM_018836.4 | 685 | 625 | 60 |
| 768-LNmet | TNFRSF1B | 678 | hg19 | chr1 | 12248919 | 12248919 | Missense_Mutation | SNP | A | A | G | novel | Somatic | T49A | 0.18225 | NM_001066.3 | 631 | 516 | 115 |
| 768-LNmet | MED8 | 919 | hg19 | chr1 | 43850143 | 43850143 | Frame_Shift_Del | DEL | T | T | - | novel | Somatic | N295MfsTer62 | 0.038922 | NM_052877.5 | 334 | 321 | 13 |
| 768-LNmet | RABGGTB | 1166 | hg19 | chr1 | 76255688 | 76255688 | Nonsense_Mutation | SNP | G | G | T | novel | Somatic | E121Ter | 0.054054 | NM_004582.4 | 480 | 454 | 26 |
| 768-LNmet | IGSF9 | 225 | hg19 | chr1 | 159897582 | 159897582 | Missense_Mutation | SNP | G | G | A | novel | Somatic | S1109F | 0.108844 | NM_001135050.2 | 292 | 260 | 32 |
| 768-LNmet | IGFN1 | 264 | hg19 | chr1 | 201185708 | 201185708 | Missense_Mutation | SNP | G | G | A | novel | Somatic | R3141Q | 0.111111 | NM_001164586.2 | 711 | 632 | 79 |
| 768-LNmet | PKP1 | 2120 | hg19 | chr1 | 201252997 | 201252997 | Missense_Mutation | SNP | C | C | T | novel | Somatic | S56F | 0.041071 | NM_000299.3 | 560 | 537 | 23 |
| 768-LNmet | SLC41A1 | 2154 | hg19 | chr1 | 205779283 | 205779283 | Missense_Mutation | SNP | A | A | C | novel | Somatic | F96C | 0.072041 | NM_173854.6 | 583 | 541 | 42 |
| 768-LNmet | CCDC185 | 2290 | hg19 | chr1 | 223568412 | 223568412 | Missense_Mutation | SNP | T | T | A | novel | Somatic | L532H | 0.146087 | NM_152610.3 | 575 | 491 | 84 |
| 768-LNmet | OBSCN | 290 | hg19 | chr1 | 228560408 | 228560408 | Nonsense_Mutation | SNP | C | C | G | novel | Somatic | S8267Ter | 0.071651 | NM_001271223.2 | 321 | 298 | 23 |
| 768-LNmet | SOX11 | 629 | hg19 | chr2 | 5833962 | 5833962 | Missense_Mutation | SNP | C | C | G | novel | Somatic | S370C | 0.188406 | NM_003108.4 | 345 | 280 | 65 |
| 768-LNmet | PLEK | 1108 | hg19 | chr2 | 68622898 | 68622898 | Missense_Mutation | SNP | C | C | T | novel | Somatic | R335C | 0.069767 | NM_002664.3 | 214 | 199 | 15 |
| 768-LNmet | C2orf81 | 1154 | hg19 | chr2 | 74642696 | 74642696 | Missense_Mutation | SNP | C | C | T | novel | Somatic | R203Q | 0.188552 | NM_001316764.3 | 594 | 482 | 112 |
| 768-LNmet | NT5DC4 | 1450 | hg19 | chr2 | 113480080 | 113480080 | Missense_Mutation | SNP | A | A | G | novel | Somatic | E102G | 0.126238 | NM_001350494.2 | 404 | 353 | 51 |
| 768-LNmet | LCT | 203 | hg19 | chr2 | 136570058 | 136570058 | Missense_Mutation | SNP | G | G | A | novel | Somatic | R726C | 0.163148 | NM_002299.4 | 521 | 436 | 85 |
| 768-LNmet | LRP1B | 3 | hg19 | chr2 | 141267521 | 141267521 | Missense_Mutation | SNP | C | C | T | novel | Somatic | D2792N | 0.129412 | NM_018557.3 | 340 | 296 | 44 |
| 768-LNmet | LRP2 | 235 | hg19 | chr2 | 169997024 | 169997024 | Frame_Shift_Ins | INS | - | - | G | novel | Somatic | C4381MfsTer12 | 0.033854 | NM_004525.3 | 384 | 371 | 13 |
| 768-LNmet | METAP1D | 237 | hg19 | chr2 | 172942627 | 172942627 | Splice_Region | SNP | G | G | A | novel | Somatic |  | 0.068493 | NM_199227.3 | 292 | 272 | 20 |
| 768-LNmet | TTN | 1955 | hg19 | chr2 | 179510734 | 179510734 | Missense_Mutation | SNP | C | C | G | novel | Somatic | E13441Q | 0.036585 | NM_001267550.2 | 328 | 316 | 12 |
| 768-LNmet | PDE1A | 1981 | hg19 | chr2 | 183104988 | 183104988 | Missense_Mutation | SNP | C | C | T | novel | Somatic | D87N | 0.116959 | NM_001258312.1 | 342 | 302 | 40 |
| 768-LNmet | MAP2 | 273 | hg19 | chr2 | 210543334 | 210543334 | Missense_Mutation | SNP | G | G | A | novel | Somatic | E101K | 0.037736 | NM_002374.3 | 424 | 408 | 16 |
| 768-LNmet | USP40 | 2373 | hg19 | chr2 | 234386078 | 234386078 | Missense_Mutation | SNP | G | G | A | novel | Somatic | P1224L | 0.099291 | NM_001365479.1 | 423 | 381 | 42 |
| 768-LNmet | BAP1 | 985 | hg19 | chr3 | 52443586 | 52443586 | Nonsense_Mutation | SNP | G | G | A | novel | Somatic | Q36Ter | 0.151163 | NM_004656.4 | 430 | 365 | 65 |
| 768-LNmet | ERC2 | 15 | hg19 | chr3 | 56330423 | 56330423 | Missense_Mutation | SNP | C | C | T | novel | Somatic | R233Q | 0.065517 | NM_015576.3 | 580 | 542 | 38 |
| 768-LNmet | USF3 | 181 | hg19 | chr3 | 113376475 | 113376475 | Missense_Mutation | SNP | G | G | A | novel | Somatic | L1352F | 0.048991 | NM_001009899.4 | 346 | 329 | 17 |
| 768-LNmet | EIF4G1 | 1989 | hg19 | chr3 | 184042091 | 184042091 | Missense_Mutation | SNP | G | G | C | novel | Somatic | E866Q | 0.126289 | NM_001194947.1 | 388 | 339 | 49 |
| 768-LNmet | MUC4 | 2076 | hg19 | chr3 | 195512594 | 195512594 | Missense_Mutation | SNP | G | G | A | novel | Somatic | L1953F | 0.087645 | NM_018406.7 | 2588 | 2361 | 227 |
| 768-LNmet | CCDC96 | 638 | hg19 | chr4 | 7043867 | 7043867 | Missense_Mutation | SNP | C | C | T | novel | Somatic | E267K | 0.158111 | NM_153376.3 | 487 | 410 | 77 |
| 768-LNmet | WDR19 | 110 | hg19 | chr4 | 39206803 | 39206803 | Frame_Shift_Ins | INS | - | - | T | novel | Somatic | L214FfsTer5 | 0.078261 | NM_025132.4 | 343 | 316 | 27 |
| 768-LNmet | FAM13A | 158 | hg19 | chr4 | 89671659 | 89671659 | Missense_Mutation | SNP | C | C | T | novel | Somatic | R619Q | 0.07971 | NM_014883.4 | 550 | 506 | 44 |
| 768-LNmet | USP38 | 1684 | hg19 | chr4 | 144116919 | 144116919 | Missense_Mutation | SNP | T | T | C | novel | Somatic | V290A | 0.148515 | NM_032557.6 | 303 | 258 | 45 |
| 768-LNmet | SORBS2 | 250 | hg19 | chr4 | 186544674 | 186544674 | Missense_Mutation | SNP | C | C | T | novel | Somatic | E733K | 0.150862 | NM_001270771.2 | 696 | 591 | 105 |
| 768-LNmet | H3.Y | 719 | hg19 | chr5 | 17655622 | 17655622 | Missense_Mutation | SNP | C | C | T | novel | Somatic | R9H | 0.322508 | NM_001355258.2 | 1321 | 894 | 427 |
| 768-LNmet | CDH10 | 96 | hg19 | chr5 | 24491793 | 24491793 | Missense_Mutation | SNP | C | C | T | novel | Somatic | D590N | 0.075 | NM_006727.5 | 479 | 443 | 36 |
| 768-LNmet | MROH2B | 112 | hg19 | chr5 | 41067246 | 41067246 | Missense_Mutation | SNP | C | C | G | novel | Somatic | L55F | 0.039416 | NM_173489.5 | 683 | 656 | 27 |
| 768-LNmet | ZNF608 | 191 | hg19 | chr5 | 123980275 | 123980275 | Missense_Mutation | SNP | C | C | T | novel | Somatic | R1262K | 0.248603 | NM_020747.2 | 358 | 269 | 89 |
| 768-LNmet | GRIA1 | 27 | hg19 | chr5 | 153035421 | 153035421 | Missense_Mutation | SNP | C | C | T | novel | Somatic | L230F | 0.101887 | NM_000827.4 | 265 | 238 | 27 |
| 768-LNmet | FOXI1 | 1878 | hg19 | chr5 | 169533506 | 169533506 | Missense_Mutation | SNP | A | A | C | novel | Somatic | K182T | 0.021713 | NM_012188.5 | 829 | 811 | 18 |
| 768-LNmet | FAM8A1 | 719 | hg19 | chr6 | 17600647 | 17600647 | Missense_Mutation | SNP | G | G | A | novel | Somatic | E3K | 0.083682 | NM_016255.3 | 239 | 219 | 20 |
| 768-LNmet | DNAH8 | 13 | hg19 | chr6 | 38825390 | 38825390 | Missense_Mutation | SNP | C | C | A | novel | Somatic | Q1944K | 0.100346 | NM_001206927.2 | 289 | 260 | 29 |
| 768-LNmet | TDRD6 | 117 | hg19 | chr6 | 46657612 | 46657612 | Missense_Mutation | SNP | G | G | A | novel | Somatic | V583I | 0.062147 | NM_001010870.3 | 353 | 331 | 22 |
| 768-LNmet | COL19A1 | 140 | hg19 | chr6 | 70639566 | 70639566 | Nonsense_Mutation | SNP | C | C | T | novel | Somatic | R214Ter | 0.125 | NM_001858.6 | 263 | 230 | 33 |
| 768-LNmet | FNDC1 | 225 | hg19 | chr6 | 159672439 | 159672439 | Missense_Mutation | SNP | A | A | T | novel | Somatic | D1647V | 0.086957 | NM_032532.3 | 368 | 336 | 32 |
| 768-LNmet | LPA | 226 | hg19 | chr6 | 160952810 | 160952810 | Missense_Mutation | SNP | C | C | T | novel | Somatic | R2025H | 0.144379 | NM_005577.4 | 845 | 723 | 122 |
| 768-LNmet | UNC93A | 1864 | hg19 | chr6 | 167711503 | 167711503 | Missense_Mutation | SNP | C | C | A | novel | Somatic | N190K | 0.103806 | NM_018974.4 | 577 | 517 | 60 |
| 768-LNmet | DLL1 | 1886 | hg19 | chr6 | 170592616 | 170592616 | Missense_Mutation | SNP | C | C | T | novel | Somatic | R584Q | 0.055659 | NM_005618.4 | 539 | 509 | 30 |
| 768-LNmet | CYP2W1 | 592 | hg19 | chr7 | 1024112 | 1024112 | Missense_Mutation | SNP | C | C | T | novel | Somatic | T80M | 0.068733 | NM_017781.3 | 741 | 690 | 51 |
| 768-LNmet | GRM3 | 155 | hg19 | chr7 | 86415818 | 86415818 | Missense_Mutation | SNP | G | G | A | novel | Somatic | R237H | 0.138889 | NM_000840.2 | 684 | 589 | 95 |
| 768-LNmet | PPP1R9A | 163 | hg19 | chr7 | 94898813 | 94898813 | Missense_Mutation | SNP | G | G | A | novel | Somatic | E1040K | 0.046729 | NM_001166160.2 | 214 | 204 | 10 |
| 768-LNmet | KMT2E | 172 | hg19 | chr7 | 104707230 | 104707230 | Missense_Mutation | SNP | A | A | G | novel | Somatic | Y158C | 0.147343 | NM_182931.3 | 414 | 353 | 61 |
| 768-LNmet | PRSS1 | 1671 | hg19 | chr7 | 142458479 | 142458479 | Missense_Mutation | SNP | G | G | C | novel | Somatic | Q38H | 0.048128 | NM_002769.5 | 747 | 711 | 36 |
| 768-LNmet | NBN | 159 | hg19 | chr8 | 90967655 | 90967655 | Nonsense_Mutation | SNP | G | G | C | novel | Somatic | S418Ter | 0.149254 | NM_002485.4 | 401 | 341 | 60 |
| 768-LNmet | VPS13B | 1348 | hg19 | chr8 | 100796636 | 100796636 | Missense_Mutation | SNP | G | G | T | novel | Somatic | D2650Y | 0.120253 | NM_017890.4 | 316 | 278 | 38 |
| 768-LNmet | PKHD1L1 | 178 | hg19 | chr8 | 110432825 | 110432825 | Missense_Mutation | SNP | C | C | A | novel | Somatic | T868K | 0.050704 | NM_177531.6 | 355 | 337 | 18 |
| 768-LNmet | FRMPD1 | 872 | hg19 | chr9 | 37732361 | 37732361 | Nonsense_Mutation | SNP | C | C | T | novel | Somatic | R307Ter | 0.221239 | NM_014907.3 | 226 | 176 | 50 |
| 768-LNmet | NIPSNAP3B | 1405 | hg19 | chr9 | 107528646 | 107528646 | Missense_Mutation | SNP | G | G | A | novel | Somatic | G34E | 0.147436 | NM_018376.4 | 311 | 265 | 46 |
| 768-LNmet | PAPPA | 186 | hg19 | chr9 | 119139859 | 119139859 | Missense_Mutation | SNP | T | T | C | novel | Somatic | V1537A | 0.082474 | NM_002581.5 | 194 | 178 | 16 |
| 768-LNmet | OLFML2A | 1558 | hg19 | chr9 | 127561721 | 127561721 | Missense_Mutation | SNP | C | C | T | novel | Somatic | A207V | 0.020408 | NM_182487.4 | 539 | 528 | 11 |
| 768-LNmet | KCNT1 | 205 | hg19 | chr9 | 138606490 | 138606490 | Missense_Mutation | SNP | G | G | A | novel | Somatic | E60K | 0.187291 | NM_020822.3 | 299 | 243 | 56 |
| 768-LNmet | ALDH18A1 | 165 | hg19 | chr10 | 97387306 | 97387306 | Missense_Mutation | SNP | G | G | T | novel | Somatic | S324Y | 0.061333 | NM_002860.4 | 375 | 352 | 23 |
| 768-LNmet | SFRP5 | 1344 | hg19 | chr10 | 99531359 | 99531359 | Missense_Mutation | SNP | C | C | G | novel | Somatic | E78Q | 0.132505 | NM_003015.3 | 483 | 419 | 64 |
| 768-LNmet | DMBT1 | 191 | hg19 | chr10 | 124402699 | 124402699 | Missense_Mutation | SNP | C | C | T | novel | Somatic | R2343W | 0.13242 | NM_007329.2 | 438 | 380 | 58 |
| 768-LNmet | E2F8 | 731 | hg19 | chr11 | 19252218 | 19252218 | Missense_Mutation | SNP | C | C | G | novel | Somatic | K410N | 0.078947 | NM_024680.4 | 266 | 245 | 21 |
| 768-LNmet | PAX6 | 827 | hg19 | chr11 | 31816252 | 31816252 | Missense_Mutation | SNP | C | C | T | novel | Somatic | R203Q | 0.06713 | NM_000280.4 | 432 | 403 | 29 |
| 768-LNmet | AHNAK | 1060 | hg19 | chr11 | 62295012 | 62295012 | Missense_Mutation | SNP | C | C | T | novel | Somatic | G2293R | 0.075404 | NM_001620.3 | 556 | 514 | 42 |
| 768-LNmet | LTBP3 | 1083 | hg19 | chr11 | 65325326 | 65325326 | In_Frame_Del | DEL | CAG | CAG | - | novel | Somatic | L35del | 0.202128 | NM_001130144.2 | 92 | 73 | 19 |
| 768-LNmet | GAL3ST3 | 1087 | hg19 | chr11 | 65810700 | 65810700 | Missense_Mutation | SNP | G | G | A | novel | Somatic | R192C | 0.029213 | NM_033036.3 | 445 | 432 | 13 |
| 768-LNmet | CLPB | 141 | hg19 | chr11 | 72069957 | 72069957 | Missense_Mutation | SNP | C | C | T | novel | Somatic | D248N | 0.417808 | NM_001258392.3 | 583 | 339 | 244 |
| 768-LNmet | WNT5B | 598 | hg19 | chr12 | 1755207 | 1755207 | Missense_Mutation | SNP | G | G | A | novel | Somatic | R290H | 0.109737 | NM_032642.3 | 646 | 575 | 71 |
| 768-LNmet | GNB3 | 638 | hg19 | chr12 | 6952180 | 6952180 | Missense_Mutation | SNP | G | G | A | novel | Somatic | R48Q | 0.14781 | NM_002075.4 | 548 | 467 | 81 |
| 768-LNmet | USP5 | 638 | hg19 | chr12 | 6972416 | 6972416 | Missense_Mutation | SNP | G | G | A | novel | Somatic | G610E | 0.10772 | NM_001098536.2 | 557 | 497 | 60 |
| 768-LNmet | KIAA1551 | 103 | hg19 | chr12 | 32138043 | 32138043 | Missense_Mutation | SNP | T | T | G | novel | Somatic | M1385R | 0.069231 | NM_018169.4 | 260 | 242 | 18 |
| 768-LNmet | ARID2 | 14 | hg19 | chr12 | 46245223 | 46245223 | Missense_Mutation | SNP | C | C | T | novel | Somatic | A1106V | 0.059278 | NM_152641.4 | 388 | 365 | 23 |
| 768-LNmet | ANO4 | 169 | hg19 | chr12 | 101336303 | 101336303 | Missense_Mutation | SNP | T | T | G | novel | Somatic | M149R | 0.059459 | NM_001286615.2 | 370 | 348 | 22 |
| 768-LNmet | FOXN4 | 1422 | hg19 | chr12 | 109725301 | 109725301 | Frame_Shift_Del | DEL | G | G | - | novel | Somatic | P166HfsTer36 | 0.050761 | NM_213596.3 | 394 | 374 | 20 |
| 768-LNmet | SACS | 767 | hg19 | chr13 | 23914687 | 23914687 | Frame_Shift_Del | DEL | T | T | - | novel | Somatic | I1110LfsTer16 | 0.042208 | NM_014363.6 | 307 | 294 | 13 |
| 768-LNmet | PRPF39 | 932 | hg19 | chr14 | 45571797 | 45571797 | Missense_Mutation | SNP | A | A | T | novel | Somatic | Y212F | 0.069705 | NM_017922.4 | 373 | 347 | 26 |
| 768-LNmet | NRXN3 | 149 | hg19 | chr14 | 79111726 | 79111726 | Frame_Shift_Del | DEL | G | G | - | novel | Somatic | A341PfsTer7 | 0.032483 | NM_001330195.2 | 430 | 416 | 14 |
| 768-LNmet | SPATA7 | 157 | hg19 | chr14 | 88859755 | 88859755 | Missense_Mutation | SNP | C | C | T | novel | Somatic | S38F | 0.146245 | NM_018418.5 | 506 | 432 | 74 |
| 768-LNmet | INF2 | 1387 | hg19 | chr14 | 105167890 | 105167890 | Missense_Mutation | SNP | G | G | A | novel | Somatic | G63D | 0.022026 | NM_022489.4 | 454 | 444 | 10 |
| 768-LNmet | AKT1 | 173 | hg19 | chr14 | 105246551 | 105246551 | Missense_Mutation | SNP | C | C | T | novel | Somatic | E17K | 0.145763 | NM_001014432.1 | 295 | 252 | 43 |
| 768-LNmet | UNC13C | 15 | hg19 | chr15 | 54919052 | 54919052 | Missense_Mutation | SNP | G | G | A | novel | Somatic | G2129E | 0.071642 | NM_001080534.2 | 335 | 311 | 24 |
| 768-LNmet | TRIP4 | 1078 | hg19 | chr15 | 64747217 | 64747217 | Missense_Mutation | SNP | A | A | G | novel | Somatic | H567R | 0.131818 | NM_016213.5 | 220 | 191 | 29 |
| 768-LNmet | GOLGA6B | 1141 | hg19 | chr15 | 72954811 | 72954811 | Nonsense_Mutation | SNP | G | G | T | novel | Somatic | E356Ter | 0.088068 | NM_018652.4 | 702 | 640 | 62 |
| 768-LNmet | SCNN1B | 95 | hg19 | chr16 | 23360214 | 23360214 | Nonsense_Mutation | SNP | C | C | A | novel | Somatic | C98Ter | 0.106742 | NM_000336.3 | 356 | 318 | 38 |
| 768-LNmet | SPNS1 | 806 | hg19 | chr16 | 28992903 | 28992903 | Missense_Mutation | SNP | C | C | T | novel | Somatic | S259L | 0.053398 | NM_032038.3 | 617 | 584 | 33 |
| 768-LNmet | INO80E | 101 | hg19 | chr16 | 30007665 | 30007665 | Frame_Shift_Del | DEL | A | A | - | novel | Somatic | K14NfsTer5 | 0.043928 | NM_173618.3 | 387 | 370 | 17 |
| 768-LNmet | ZFHX3 | 142 | hg19 | chr16 | 72828114 | 72828114 | Missense_Mutation | SNP | G | G | C | novel | Somatic | L2823V | 0.172507 | NM_006885.4 | 371 | 307 | 64 |
| 768-LNmet | CPHXL | 145 | hg19 | chr16 | 75748362 | 75748362 | Frame_Shift_Del | DEL | CT | CT | - | novel | Somatic | E326GfsTer36 | 0.05305 | NM_001355613.1 | 376 | 356 | 20 |
| 768-LNmet | RPA1 | 598 | hg19 | chr17 | 1747967 | 1747967 | Missense_Mutation | SNP | C | C | G | novel | Somatic | L87V | 0.074074 | NM_002945.5 | 243 | 225 | 18 |
| 768-LNmet | PLD2 | 77 | hg19 | chr17 | 4713302 | 4713302 | Missense_Mutation | SNP | G | G | A | novel | Somatic | V280M | 0.061567 | NM_002663.5 | 535 | 502 | 33 |
| 768-LNmet | TP53 | 642 | hg19 | chr17 | 7578406 | 7578406 | Missense_Mutation | SNP | C | C | T | novel | Somatic | R175H | 0.144216 | NM_000546.5 | 630 | 539 | 91 |
| 768-LNmet | MYH8 | 663 | hg19 | chr17 | 10302977 | 10302977 | Missense_Mutation | SNP | C | C | T | novel | Somatic | E1249K | 0.094421 | NM_002472.3 | 233 | 211 | 22 |
| 768-LNmet | MPRIP | 89 | hg19 | chr17 | 17070310 | 17070310 | Missense_Mutation | SNP | C | C | T | novel | Somatic | S1834F | 0.074813 | NM_001364716.2 | 400 | 370 | 30 |
| 768-LNmet | DRG2 | 722 | hg19 | chr17 | 18002994 | 18002994 | Missense_Mutation | SNP | G | G | A | novel | Somatic | V142I | 0.077626 | NM_001388.5 | 438 | 404 | 34 |
| 768-LNmet | KSR1 | 97 | hg19 | chr17 | 25924391 | 25924391 | Missense_Mutation | SNP | C | C | G | novel | Somatic | S466C | 0.224889 | NM_001367810.1 | 1122 | 869 | 253 |
| 768-LNmet | KIF18B | 913 | hg19 | chr17 | 43005934 | 43005934 | Missense_Mutation | SNP | G | G | C | novel | Somatic | S579C | 0.152941 | NM_001265577.2 | 425 | 360 | 65 |
| 768-LNmet | HOXB3 | 940 | hg19 | chr17 | 46628165 | 46628165 | Missense_Mutation | SNP | A | A | G | novel | Somatic | M276T | 0.041121 | NM_002146.4 | 534 | 512 | 22 |
| 768-LNmet | DDX42 | 16 | hg19 | chr17 | 61895231 | 61895231 | Missense_Mutation | SNP | C | C | T | novel | Somatic | P764S | 0.350087 | NM_007372.3 | 577 | 375 | 202 |
| 768-LNmet | TYMS | 590 | hg19 | chr18 | 672903 | 672903 | Missense_Mutation | SNP | G | G | A | novel | Somatic | R283Q | 0.07563 | NM_001071.4 | 476 | 440 | 36 |
| 768-LNmet | CCDC178 | 102 | hg19 | chr18 | 30913143 | 30913143 | Frame_Shift_Del | DEL | T | T | - | novel | Somatic | M292WfsTer3 | 0.056497 | NM_001371121.1 | 177 | 167 | 10 |
| 768-LNmet | SERPINB10 | 1054 | hg19 | chr18 | 61584738 | 61584738 | Frame_Shift_Ins | INS | - | - | A | novel | Somatic | R76KfsTer16 | 0.03304 | NM_005024.3 | 453 | 438 | 15 |
| 768-LNmet | DSEL | 1082 | hg19 | chr18 | 65179606 | 65179606 | Missense_Mutation | SNP | C | C | A | novel | Somatic | R747L | 0.082418 | NM_032160.3 | 364 | 334 | 30 |
| 768-LNmet | DSEL | 1082 | hg19 | chr18 | 65180867 | 65180867 | Missense_Mutation | SNP | C | C | G | novel | Somatic | V327L | 0.155718 | NM_032160.3 | 411 | 347 | 64 |
| 768-LNmet | AMH | 602 | hg19 | chr19 | 2250998 | 2250998 | Missense_Mutation | SNP | C | C | T | novel | Somatic | P272L | 0.088235 | NM_000479.5 | 136 | 124 | 12 |
| 768-LNmet | ANKRD24 | 9 | hg19 | chr19 | 4217531 | 4217531 | Missense_Mutation | SNP | G | G | C | novel | Somatic | E792Q | 0.083871 | NM_133475.1 | 155 | 142 | 13 |
| 768-LNmet | ANKRD24 | 9 | hg19 | chr19 | 4217603 | 4217603 | Missense_Mutation | SNP | G | G | A | novel | Somatic | D816N | 0.109948 | NM_133475.1 | 190 | 169 | 21 |
| 768-LNmet | CNN1 | 673 | hg19 | chr19 | 11658613 | 11658613 | Missense_Mutation | SNP | C | C | T | novel | Somatic | A131V | 0.084951 | NM_001299.6 | 411 | 376 | 35 |
| 768-LNmet | ZNF44 | 679 | hg19 | chr19 | 12384448 | 12384448 | Frame_Shift_Del | DEL | A | A | - | novel | Somatic | W256GfsTer34 | 0.023305 | NM_001164276.2 | 472 | 461 | 11 |
| 768-LNmet | CPAMD8 | 714 | hg19 | chr19 | 17008795 | 17008795 | Missense_Mutation | SNP | C | C | T | novel | Somatic | R1620Q | 0.161017 | NM_015692.5 | 353 | 296 | 57 |
| 768-LNmet | URI1 | 817 | hg19 | chr19 | 30500119 | 30500119 | In_Frame_Del | DEL | TGA | TGA | - | novel | Somatic | D311del | 0.044807 | NM_003796.3 | 489 | 467 | 22 |
| 768-LNmet | WDR87 | 877 | hg19 | chr19 | 38378243 | 38378243 | Missense_Mutation | SNP | A | A | G | novel | Somatic | L2023P | 0.035256 | NM_001291088.2 | 312 | 301 | 11 |
| 768-LNmet | SPRED3 | 881 | hg19 | chr19 | 38881062 | 38881062 | Frame_Shift_Del | DEL | G | G | - | novel | Somatic | A43PfsTer24 | 0.0271 | NM_001042522.2 | 369 | 359 | 10 |
| 768-LNmet | ECH1 | 110 | hg19 | chr19 | 39322031 | 39322031 | Missense_Mutation | SNP | C | C | G | novel | Somatic | V60L | 0.09407 | NM_001398.3 | 489 | 443 | 46 |
| 768-LNmet | LGALS14 | 891 | hg19 | chr19 | 40197975 | 40197975 | Missense_Mutation | SNP | G | G | A | novel | Somatic | E84K | 0.109589 | NM_020129.3 | 219 | 195 | 24 |
| 768-LNmet | RTN2 | 935 | hg19 | chr19 | 45998093 | 45998093 | Missense_Mutation | SNP | G | G | A | novel | Somatic | R84C | 0.062176 | NM_005619.5 | 772 | 724 | 48 |
| 768-LNmet | GPR32 | 976 | hg19 | chr19 | 51274575 | 51274575 | Missense_Mutation | SNP | C | C | T | novel | Somatic | R240W | 0.077165 | NM_001506.2 | 635 | 586 | 49 |
| 768-LNmet | SIGLEC8 | 981 | hg19 | chr19 | 51958723 | 51958723 | Missense_Mutation | SNP | G | G | T | novel | Somatic | Q334K | 0.057234 | NM_014442.3 | 629 | 593 | 36 |
| 768-LNmet | ZNF616 | 986 | hg19 | chr19 | 52620014 | 52620014 | Missense_Mutation | SNP | G | G | C | novel | Somatic | H135D | 0.033708 | NM_178523.5 | 356 | 344 | 12 |
| 768-LNmet | NLRP2 | 1008 | hg19 | chr19 | 55494912 | 55494912 | Missense_Mutation | SNP | G | G | C | novel | Somatic | E616Q | 0.112094 | NM_017852.5 | 339 | 301 | 38 |
| 768-LNmet | GDAP1L1 | 912 | hg19 | chr20 | 42885971 | 42885971 | Missense_Mutation | SNP | G | G | A | novel | Somatic | R120H | 0.035928 | NM_024034.5 | 501 | 483 | 18 |
| 768-LNmet | TTPAL | 114 | hg19 | chr20 | 43108823 | 43108823 | Missense_Mutation | SNP | C | C | T | novel | Somatic | R62C | 0.072086 | NM_024331.5 | 652 | 605 | 47 |
| 768-LNmet | OLIG2 | 847 | hg19 | chr21 | 34399954 | 34399954 | Missense_Mutation | SNP | A | A | G | novel | Somatic | K262E | 0.08547 | NM_005806.4 | 117 | 107 | 10 |
| 768-LNmet | NLGN4X | 78 | hg19 | chrX | 5827217 | 5827217 | Missense_Mutation | SNP | G | G | A | novel | Somatic | A230V | 0.033613 | NM_001282145.2 | 357 | 345 | 12 |
| 768-LNmet | SHROOM4 | 121 | hg19 | chrX | 50438919 | 50438919 | Missense_Mutation | SNP | C | C | T | novel | Somatic | A46T | 0.225564 | NM_020717.3 | 133 | 103 | 30 |
| 768-LNmet | NAP1L2 | 1137 | hg19 | chrX | 72433499 | 72433499 | Nonsense_Mutation | SNP | G | G | C | novel | Somatic | S277Ter | 0.107143 | NM_021963.4 | 251 | 224 | 27 |
| 768-LNmet | COL4A5 | 175 | hg19 | chrX | 107867491 | 107867491 | Missense_Mutation | SNP | A | A | T | novel | Somatic | K981N | 0.146667 | NM_033380.3 | 150 | 128 | 22 |
| 768-LNmet | RBMXL3 | 182 | hg19 | chrX | 114426351 | 114426351 | Nonsense_Mutation | SNP | C | C | T | novel | Somatic | R783Ter | 0.123223 | NM_001145346.1 | 422 | 370 | 52 |
| 768-LNmet | CUL4B | 187 | hg19 | chrX | 119676848 | 119676848 | Missense_Mutation | SNP | C | C | T | novel | Somatic | E451K | 0.25641 | NM_003588.3 | 234 | 174 | 60 |
| 786-PTsup | VPS13D | 84 | hg19 | chr1 | 12337663 | 12337663 | Missense_Mutation | SNP | G | G | C | novel | Somatic | V1340L | 0.082631 | NM_015378.4 | 590 | 541 | 49 |
| 786-PTsup | RLF | 111 | hg19 | chr1 | 40702375 | 40702375 | Missense_Mutation | SNP | C | C | G | novel | Somatic | D667E | 0.053892 | NM_012421.4 | 833 | 788 | 45 |
| 786-PTsup | TIE1 | 114 | hg19 | chr1 | 43778945 | 43778945 | Missense_Mutation | SNP | C | C | A | novel | Somatic | D689E | 0.132019 | NM_005424.5 | 1668 | 1447 | 221 |
| 786-PTsup | LRRC53 | Unknown | hg19 | chr1 | 74941228 | 74941228 | Missense_Mutation | SNP | C | C | T | novel | Somatic | E391K | 0.083643 | NM_001364665.1 | 538 | 493 | 45 |
| 786-PTsup | GBP3 | 1267 | hg19 | chr1 | 89473442 | 89473442 | Frame_Shift_Del | DEL | T | T | - | novel | Somatic | T585PfsTer9 | 0.02164 | NM_018284.3 | 878 | 859 | 19 |
| 786-PTsup | CCDC18 | 162 | hg19 | chr1 | 93676474 | 93676474 | Missense_Mutation | SNP | C | C | T | novel | Somatic | S442L | 0.125 | NM_001306076.1 | 607 | 531 | 76 |
| 786-PTsup | DPYD | 166 | hg19 | chr1 | 97771805 | 97771805 | Missense_Mutation | SNP | G | G | C | novel | Somatic | Q703E | 0.09375 | NM_000110.4 | 895 | 811 | 84 |
| 786-PTsup | PI4KB | 1739 | hg19 | chr1 | 151265889 | 151265889 | Silent | SNP | C | C | T | novel | Somatic | V773= | 0.182747 | NM_001369626.1 | 881 | 720 | 161 |
| 786-PTsup | ATP8B2 | 1762 | hg19 | chr1 | 154315769 | 154315769 | Missense_Mutation | SNP | C | C | T | novel | Somatic | S564L | 0.195489 | NM_001367934.1 | 664 | 534 | 130 |
| 786-PTsup | IQGAP3 | 222 | hg19 | chr1 | 156503808 | 156503808 | Missense_Mutation | SNP | G | G | A | novel | Somatic | T1289M | 0.122984 | NM_178229.5 | 496 | 435 | 61 |
| 786-PTsup | FCRL5 | 1786 | hg19 | chr1 | 157497419 | 157497419 | Missense_Mutation | SNP | G | G | A | novel | Somatic | L650F | 0.055866 | NM_031281.3 | 536 | 506 | 30 |
| 786-PTsup | CD5L | 223 | hg19 | chr1 | 157804287 | 157804287 | Missense_Mutation | SNP | C | C | T | novel | Somatic | G210R | 0.077939 | NM_005894.3 | 757 | 698 | 59 |
| 786-PTsup | TNN | 29 | hg19 | chr1 | 175087684 | 175087684 | Intron | DEL | T | T | - | novel | Somatic |  | 0.037667 | NM_022093.2 | 823 | 792 | 31 |
| 786-PTsup | LHX4 | 244 | hg19 | chr1 | 180235585 | 180235585 | Missense_Mutation | SNP | C | C | T | novel | Somatic | R103C | 0.097291 | NM_033343.4 | 812 | 733 | 79 |
| 786-PTsup | USH2A | 279 | hg19 | chr1 | 216052262 | 216052262 | Missense_Mutation | SNP | C | C | A | novel | Somatic | G2801V | 0.06044 | NM_206933.3 | 546 | 513 | 33 |
| 786-PTsup | OR2L13 | 309 | hg19 | chr1 | 248263030 | 248263030 | Missense_Mutation | SNP | C | C | A | novel | Somatic | A118D | 0.174691 | NM_175911.3 | 727 | 600 | 127 |
| 786-PTsup | MYT1L | 9 | hg19 | chr2 | 1947002 | 1947002 | Missense_Mutation | SNP | G | G | A | novel | Somatic | S86L | 0.084625 | NM_001329844.2 | 839 | 768 | 71 |
| 786-PTsup | FOSL2 | 803 | hg19 | chr2 | 28631638 | 28631638 | In_Frame_Del | DEL | GAG | GAG | - | novel | Somatic | E125del | 0.104895 | NM_005253.4 | 858 | 768 | 90 |
| 786-PTsup | PPP4R3B | 126 | hg19 | chr2 | 55805468 | 55805468 | Frame_Shift_Ins | INS | - | - | A | novel | Somatic | L493FfsTer7 | 0.035242 | NM_001122964.3 | 451 | 435 | 16 |
| 786-PTsup | EFEMP1 | 126 | hg19 | chr2 | 56103844 | 56103844 | Missense_Mutation | SNP | G | G | A | novel | Somatic | A265V | 0.119691 | NM_001039348.3 | 518 | 456 | 62 |
| 786-PTsup | ITPRIPL1 | 165 | hg19 | chr2 | 96993304 | 96993304 | Missense_Mutation | SNP | A | A | G | novel | Somatic | H320R | 0.074166 | NM_178495.6 | 809 | 749 | 60 |
| 786-PTsup | PDE11A | 243 | hg19 | chr2 | 178592756 | 178592756 | Missense_Mutation | SNP | C | C | G | novel | Somatic | E645Q | 0.150838 | NM_016953.4 | 537 | 456 | 81 |
| 786-PTsup | TTN | 1955 | hg19 | chr2 | 179431582 | 179431582 | Missense_Mutation | SNP | C | C | G | novel | Somatic | R26426T | 0.046358 | NM_001267550.2 | 755 | 720 | 35 |
| 786-PTsup | TTN | 1955 | hg19 | chr2 | 179432156 | 179432156 | Missense_Mutation | SNP | C | C | T | novel | Somatic | E26235K | 0.072808 | NM_001267550.2 | 673 | 624 | 49 |
| 786-PTsup | TTN | 1955 | hg19 | chr2 | 179432170 | 179432170 | Missense_Mutation | SNP | C | C | T | novel | Somatic | G26230E | 0.075916 | NM_001267550.2 | 764 | 706 | 58 |
| 786-PTsup | TTN | 1955 | hg19 | chr2 | 179433944 | 179433944 | Missense_Mutation | SNP | C | C | T | novel | Somatic | E25639K | 0.105185 | NM_001267550.2 | 673 | 602 | 71 |
| 786-PTsup | TTN | 1955 | hg19 | chr2 | 179455905 | 179455905 | Missense_Mutation | DNP | CC | CC | TT | novel | Somatic | D20183N | 0.091286 | NM_001267550.2 | 721 | 655 | 66 |
| 786-PTsup | TTN | 1955 | hg19 | chr2 | 179456389 | 179456389 | Nonsense_Mutation | SNP | C | C | A | novel | Somatic | E20053Ter | 0.08872 | NM_001267550.2 | 788 | 718 | 70 |
| 786-PTsup | TTN | 1955 | hg19 | chr2 | 179457005 | 179457005 | Splice_Site | SNP | C | C | G | novel | Somatic |  | 0.115546 | NM_001267550.2 | 476 | 421 | 55 |
| 786-PTsup | TTN | 1955 | hg19 | chr2 | 179458008 | 179458008 | Missense_Mutation | SNP | C | C | G | novel | Somatic | D19643H | 0.097059 | NM_001267550.2 | 679 | 613 | 66 |
| 786-PTsup | TTN | 1955 | hg19 | chr2 | 179458511 | 179458511 | Missense_Mutation | SNP | C | C | A | novel | Somatic | D19506Y | 0.103759 | NM_001267550.2 | 665 | 596 | 69 |
| 786-PTsup | FAM171B | 251 | hg19 | chr2 | 187626251 | 187626251 | Missense_Mutation | SNP | A | A | C | novel | Somatic | K394N | 0.023438 | NM_177454.4 | 768 | 750 | 18 |
| 786-PTsup | DNAH7 | 260 | hg19 | chr2 | 196729560 | 196729560 | Missense_Mutation | SNP | G | G | T | novel | Somatic | F2273L | 0.076647 | NM_018897.3 | 835 | 771 | 64 |
| 786-PTsup | ALS2CR11 | Unknown | hg19 | chr2 | 202352352 | 202352352 | Frame_Shift_Del | DEL | T | T | - | novel | Somatic | I1816LfsTer19 | 0.022375 | NM_001168221.2 | 581 | 568 | 13 |
| 786-PTsup | KANSL1L | 274 | hg19 | chr2 | 210894528 | 210894528 | Missense_Mutation | SNP | C | C | T | novel | Somatic | R757Q | 0.126095 | NM_152519.4 | 571 | 499 | 72 |
| 786-PTsup | C2orf54 | 303 | hg19 | chr2 | 241835333 | 241835333 | Missense_Mutation | SNP | G | G | A | novel | Somatic | R28W | 0.105799 | NM_001085437.3 | 983 | 879 | 104 |
| 786-PTsup | ITPR1 | 77 | hg19 | chr3 | 4704840 | 4704840 | Missense_Mutation | SNP | C | C | A | novel | Somatic | P487T | 0.097052 | NM_001168272.1 | 814 | 735 | 79 |
| 786-PTsup | ZNF35 | 115 | hg19 | chr3 | 44700582 | 44700582 | Missense_Mutation | SNP | A | A | T | novel | Somatic | I243F | 0.024233 | NM_003420.4 | 619 | 604 | 15 |
| 786-PTsup | ADAMTS9 | 134 | hg19 | chr3 | 64601699 | 64601699 | Missense_Mutation | SNP | G | G | C | novel | Somatic | S987R | 0.076336 | NM_182920.2 | 524 | 484 | 40 |
| 786-PTsup | TRMT10C | 1357 | hg19 | chr3 | 101283906 | 101283906 | Missense_Mutation | SNP | C | C | A | novel | Somatic | T94N | 0.118926 | NM_017819.4 | 779 | 686 | 93 |
| 786-PTsup | RNF7 | 1664 | hg19 | chr3 | 141462372 | 141462372 | Missense_Mutation | SNP | C | C | A | novel | Somatic | A66D | 0.132061 | NM_014245.5 | 1308 | 1135 | 173 |
| 786-PTsup | TM4SF18 | 1722 | hg19 | chr3 | 149051163 | 149051163 | Missense_Mutation | SNP | A | A | G | novel | Somatic | S3P | 0.11746 | NM_138786.4 | 630 | 556 | 74 |
| 786-PTsup | CCDC39 | 30 | hg19 | chr3 | 180349364 | 180349364 | Missense_Mutation | SNP | G | G | A | novel | Somatic | R631W | 0.033898 | NM_181426.2 | 530 | 512 | 18 |
| 786-PTsup | ABCF3 | 1988 | hg19 | chr3 | 183908824 | 183908824 | Missense_Mutation | SNP | G | G | A | novel | Somatic | V477I | 0.31519 | NM_018358.2 | 789 | 540 | 249 |
| 786-PTsup | GABRA4 | 117 | hg19 | chr4 | 46979602 | 46979602 | Frame_Shift_Del | DEL | T | T | - | novel | Somatic | R107DfsTer2 | 0.056923 | NM_000809.4 | 649 | 612 | 37 |
| 786-PTsup | EXOSC9 | 1521 | hg19 | chr4 | 122731167 | 122731167 | Missense_Mutation | SNP | G | G | A | novel | Somatic | M217I | 0.059347 | NM_005033.3 | 674 | 634 | 40 |
| 786-PTsup | GALNT7 | 239 | hg19 | chr4 | 174219256 | 174219256 | Intron | SNP | C | C | G | novel | Somatic |  | 0.122711 | NM_017423.3 | 546 | 479 | 67 |
| 786-PTsup | SLC12A7 | 593 | hg19 | chr5 | 1085509 | 1085509 | Missense_Mutation | SNP | C | C | T | novel | Somatic | R252H | 0.189751 | NM_006598.3 | 721 | 584 | 137 |
| 786-PTsup | DNAJC21 | 851 | hg19 | chr5 | 34954061 | 34954061 | Missense_Mutation | SNP | A | A | T | novel | Somatic | K463N | 0.03712 | NM_001012339.3 | 889 | 856 | 33 |
| 786-PTsup | EGFLAM | 878 | hg19 | chr5 | 38352350 | 38352350 | Silent | SNP | G | G | A | novel | Somatic | S154= | 0.081509 | NM_152403.4 | 821 | 754 | 67 |
| 786-PTsup | CAST | 1318 | hg19 | chr5 | 96077047 | 96077047 | Missense_Mutation | SNP | G | G | C | novel | Somatic | K332N | 0.273369 | NM_001750.7 | 567 | 412 | 155 |
| 786-PTsup | RAD50 | 1591 | hg19 | chr5 | 131923294 | 131923294 | Missense_Mutation | SNP | T | T | G | novel | Somatic | M266R | 0.321117 | NM_005732.4 | 1146 | 778 | 368 |
| 786-PTsup | SMAD5 | 1618 | hg19 | chr5 | 135508165 | 135508165 | Nonsense_Mutation | SNP | C | C | G | novel | Somatic | S273Ter | 0.025543 | NM_005903.7 | 783 | 763 | 20 |
| 786-PTsup | PCDHA4 | 1654 | hg19 | chr5 | 140188648 | 140188648 | Missense_Mutation | SNP | C | C | A | novel | Somatic | L626M | 0.067764 | NM_018907.4 | 1579 | 1472 | 107 |
| 786-PTsup | PCDHA13 | 1655 | hg19 | chr5 | 140263242 | 140263242 | Missense_Mutation | SNP | C | C | A | novel | Somatic | F463L | 0.522133 | NM_018904.3 | 1988 | 950 | 1038 |
| 786-PTsup | PCDHB6 | 1657 | hg19 | chr5 | 140531975 | 140531975 | Missense_Mutation | SNP | A | A | G | novel | Somatic | R713G | 0.147024 | NM_018939.3 | 2503 | 2135 | 368 |
| 786-PTsup | PCDHGB2 | 1658 | hg19 | chr5 | 140741872 | 140741872 | Missense_Mutation | SNP | T | T | C | novel | Somatic | W724R | 0.189139 | NM_018923.2 | 1068 | 866 | 202 |
| 786-PTsup | PCDHGB7 | 1659 | hg19 | chr5 | 140797796 | 140797796 | Missense_Mutation | SNP | G | G | C | novel | Somatic | E124Q | 0.056159 | NM_018927.4 | 1104 | 1042 | 62 |
| 786-PTsup | LARP1 | 27 | hg19 | chr5 | 154183118 | 154183118 | Missense_Mutation | SNP | C | C | A | novel | Somatic | A751D | 0.046484 | NM_033551.3 | 839 | 800 | 39 |
| 786-PTsup | FGF18 | 1888 | hg19 | chr5 | 170863173 | 170863173 | Missense_Mutation | SNP | G | G | A | novel | Somatic | R49H | 0.090671 | NM_003862.3 | 1147 | 1043 | 104 |
| 786-PTsup | UIMC1 | 241 | hg19 | chr5 | 176338382 | 176338382 | Missense_Mutation | SNP | G | G | A | novel | Somatic | R536W | 0.144309 | NM_001199297.2 | 491 | 420 | 71 |
| 786-PTsup | HIST1H2BD | 784 | hg19 | chr6 | 26158708 | 26158708 | Missense_Mutation | SNP | C | C | T | novel | Somatic | P104L | 0.210965 | NM_138720.2 | 2877 | 2269 | 608 |
| 786-PTsup | ITPR3 | 841 | hg19 | chr6 | 33636332 | 33636332 | Missense_Mutation | SNP | A | A | G | novel | Somatic | I643V | 0.092478 | NM_002224.4 | 809 | 734 | 75 |
| 786-PTsup | CLIC5 | 935 | hg19 | chr6 | 46047778 | 46047778 | Missense_Mutation | SNP | C | C | G | novel | Somatic | E68Q | 0.198157 | NM_001370650.1 | 651 | 522 | 129 |
| 786-PTsup | PKHD1 | 122 | hg19 | chr6 | 51768466 | 51768466 | Missense_Mutation | SNP | C | C | T | novel | Somatic | E2309K | 0.166107 | NM_138694.4 | 594 | 495 | 99 |
| 786-PTsup | HACE1 | 173 | hg19 | chr6 | 105178234 | 105178234 | Missense_Mutation | SNP | G | G | C | novel | Somatic | N857K | 0.0282 | NM_020771.4 | 461 | 448 | 13 |
| 786-PTsup | ENPP3 | 198 | hg19 | chr6 | 131973973 | 131973973 | Missense_Mutation | SNP | G | G | C | novel | Somatic | E136Q | 0.131579 | NM_005021.5 | 532 | 462 | 70 |
| 786-PTsup | VNN2 | 1600 | hg19 | chr6 | 133065485 | 133065485 | Missense_Mutation | SNP | A | A | T | novel | Somatic | F506Y | 0.094253 | NM_004665.5 | 435 | 394 | 41 |
| 786-PTsup | HECA | 25 | hg19 | chr6 | 139488129 | 139488129 | Missense_Mutation | SNP | C | C | T | novel | Somatic | A327V | 0.20479 | NM_016217.3 | 834 | 663 | 171 |
| 786-PTsup | UTRN | 26 | hg19 | chr6 | 144749911 | 144749911 | Splice_Region | INS | C | C | TAGACCT | novel | Somatic |  | 0.08686 | NM_007124.2 | 449 | 410 | 39 |
| 786-PTsup | ADGB | 213 | hg19 | chr6 | 146956516 | 146956516 | Missense_Mutation | SNP | A | A | T | novel | Somatic | Y27F | 0.099804 | NM_024694.4 | 511 | 460 | 51 |
| 786-PTsup | MAS1 | 1808 | hg19 | chr6 | 160328663 | 160328663 | Missense_Mutation | SNP | C | C | T | novel | Somatic | L226F | 0.098446 | NM_002377.4 | 771 | 695 | 76 |
| 786-PTsup | DNAH11 | 93 | hg19 | chr7 | 21675499 | 21675499 | Missense_Mutation | SNP | A | A | T | novel | Somatic | Q1504L | 0.131399 | NM_001277115.2 | 586 | 509 | 77 |
| 786-PTsup | ZNF804B | 157 | hg19 | chr7 | 88966101 | 88966101 | Missense_Mutation | SNP | C | C | A | novel | Somatic | H1269N | 0.137466 | NM_181646.5 | 1112 | 959 | 153 |
| 786-PTsup | PEG10 | 1304 | hg19 | chr7 | 94293685 | 94293685 | Missense_Mutation | SNP | C | C | G | novel | Somatic | P349A | 0.084004 | NM_001172437.2 | 1117 | 1023 | 94 |
| 786-PTsup | ACTL6B | 1349 | hg19 | chr7 | 100252706 | 100252706 | Missense_Mutation | SNP | T | T | C | novel | Somatic | H102R | 0.2 | NM_016188.5 | 785 | 628 | 157 |
| 786-PTsup | POLR2J | 1364 | hg19 | chr7 | 102119287 | 102119287 | Missense_Mutation | SNP | G | G | C | novel | Somatic | F7L | 0.165902 | NM_006234.5 | 1307 | 1090 | 217 |
| 786-PTsup | FAM110B | 129 | hg19 | chr8 | 59059902 | 59059902 | Nonstop_Mutation | SNP | A | A | T | novel | Somatic | Ter371YextTer24 | 0.187123 | NM_147189.3 | 495 | 402 | 93 |
| 786-PTsup | RALYL | 154 | hg19 | chr8 | 85774661 | 85774661 | Missense_Mutation | SNP | A | A | G | novel | Somatic | T195A | 0.024745 | NM_001100391.2 | 686 | 669 | 17 |
| 786-PTsup | RUNX1T1 | 1294 | hg19 | chr8 | 92988171 | 92988171 | Missense_Mutation | SNP | G | G | A | novel | Somatic | A496V | 0.171123 | NM_001198679.1 | 747 | 619 | 128 |
| 786-PTsup | WDR97 | 1692 | hg19 | chr8 | 145164670 | 145164670 | Missense_Mutation | SNP | C | C | A | novel | Somatic | A478E | 0.075145 | NM_001316309.2 | 519 | 480 | 39 |
| 786-PTsup | NTRK2 | 156 | hg19 | chr9 | 87339207 | 87339207 | Missense_Mutation | SNP | G | G | C | novel | Somatic | Q263H | 0.094881 | NM_006180.4 | 799 | 723 | 76 |
| 786-PTsup | SHC3 | 160 | hg19 | chr9 | 91727466 | 91727466 | Splice_Region | SNP | C | C | G | novel | Somatic |  | 0.057803 | NM_016848.6 | 518 | 488 | 30 |
| 786-PTsup | CRB2 | 1547 | hg19 | chr9 | 126136139 | 126136139 | Missense_Mutation | SNP | C | C | T | novel | Somatic | T1110M | 0.087635 | NM_173689.7 | 833 | 760 | 73 |
| 786-PTsup | MVB12B | 196 | hg19 | chr9 | 129089245 | 129089245 | In_Frame_Del | DEL | CCG | CCG | - | novel | Somatic | P16del | 0.03178 | NM_033446.3 | 471 | 456 | 15 |
| 786-PTsup | LCN1 | 1641 | hg19 | chr9 | 138415088 | 138415088 | Missense_Mutation | SNP | C | C | T | novel | Somatic | R78W | 0.139262 | NM_001252618.2 | 595 | 512 | 83 |
| 786-PTsup | NPDC1 | 1652 | hg19 | chr9 | 139935154 | 139935154 | Missense_Mutation | SNP | C | C | T | novel | Somatic | A198T | 0.13918 | NM_015392.4 | 1047 | 901 | 146 |
| 786-PTsup | MRLN | 1054 | hg19 | chr10 | 61496922 | 61496922 | Missense_Mutation | SNP | T | T | G | novel | Somatic | T13P | 0.039049 | NM_001304732.2 | 589 | 566 | 23 |
| 786-PTsup | CDH23 | 1146 | hg19 | chr10 | 73548732 | 73548732 | Missense_Mutation | SNP | G | G | T | novel | Somatic | E1952D | 0.073579 | NM_022124.6 | 597 | 553 | 44 |
| 786-PTsup | ECD | 1156 | hg19 | chr10 | 74896497 | 74896497 | Missense_Mutation | SNP | A | A | C | novel | Somatic | C557G | 0.212177 | NM_007265.3 | 542 | 427 | 115 |
| 786-PTsup | TNKS2 | 162 | hg19 | chr10 | 93619393 | 93619393 | Missense_Mutation | SNP | A | A | G | novel | Somatic | Y1090C | 0.229008 | NM_025235.4 | 655 | 505 | 150 |
| 786-PTsup | LCOR | 167 | hg19 | chr10 | 98711858 | 98711858 | Splice_Site | SNP | A | A | T | novel | Somatic |  | 0.170874 | NM_001346516.2 | 515 | 427 | 88 |
| 786-PTsup | TMEM80 | 590 | hg19 | chr11 | 704540 | 704540 | Missense_Mutation | SNP | A | A | C | novel | Somatic | E249A | 0.16035 | NM_001276274.1 | 1029 | 864 | 165 |
| 786-PTsup | CALCA | 699 | hg19 | chr11 | 14992709 | 14992709 | Missense_Mutation | TNP | CAG | CAG | AAT | novel | Somatic | L10I | 0.179245 | NM_001033952.2 | 424 | 348 | 76 |
| 786-PTsup | NELL1 | 11 | hg19 | chr11 | 20968923 | 20968923 | Missense_Mutation | SNP | G | G | C | novel | Somatic | L371F | 0.12763 | NM_006157.5 | 713 | 622 | 91 |
| 786-PTsup | NELL1 | 11 | hg19 | chr11 | 21596493 | 21596493 | Intron | SNP | T | T | G | novel | Somatic |  | 0.088889 | NM_006157.5 | 540 | 492 | 48 |
| 786-PTsup | DDB2 | 945 | hg19 | chr11 | 47259481 | 47259481 | Missense_Mutation | SNP | G | G | A | novel | Somatic | D373N | 0.183832 | NM_000107.2 | 903 | 737 | 166 |
| 786-PTsup | OR4C3 | 953 | hg19 | chr11 | 48347107 | 48347107 | Missense_Mutation | SNP | C | C | G | novel | Somatic | D178E | 0.031429 | NM_001004702.2 | 1050 | 1017 | 33 |
| 786-PTsup | OR5T3 | 1012 | hg19 | chr11 | 56020588 | 56020588 | Missense_Mutation | SNP | T | T | A | novel | Somatic | S305T | 0.334356 | NM_001004747.1 | 651 | 433 | 218 |
| 786-PTsup | FAT3 | 2 | hg19 | chr11 | 92532370 | 92532370 | Missense_Mutation | SNP | G | G | A | novel | Somatic | R2064H | 0.226619 | NM_001367949.1 | 556 | 430 | 126 |
| 786-PTsup | OR4D5 | 1529 | hg19 | chr11 | 123810436 | 123810436 | Missense_Mutation | SNP | C | C | G | novel | Somatic | T38S | 0.09571 | NM_001001965.1 | 605 | 547 | 58 |
| 786-PTsup | ANO2 | 78 | hg19 | chr12 | 5941621 | 5941621 | Missense_Mutation | SNP | T | T | C | novel | Somatic | Y262C | 0.038585 | NM_001278596.3 | 622 | 598 | 24 |
| 786-PTsup | PZP | 81 | hg19 | chr12 | 9317889 | 9317889 | Missense_Mutation | SNP | A | A | G | novel | Somatic | L778P | 0.062992 | NM_002864.3 | 1143 | 1071 | 72 |
| 786-PTsup | TAS2R7 | 668 | hg19 | chr12 | 10955058 | 10955058 | Missense_Mutation | SNP | T | T | C | novel | Somatic | K38E | 0.080808 | NM_023919.2 | 692 | 636 | 56 |
| 786-PTsup | PLBD1 | 10 | hg19 | chr12 | 14659897 | 14659897 | Missense_Mutation | SNP | C | C | T | novel | Somatic | A448T | 0.068479 | NM_024829.6 | 991 | 923 | 68 |
| 786-PTsup | ITPR2 | 98 | hg19 | chr12 | 26784949 | 26784949 | Missense_Mutation | SNP | C | C | G | novel | Somatic | M928I | 0.08172 | NM_002223.4 | 930 | 854 | 76 |
| 786-PTsup | ITPR2 | 98 | hg19 | chr12 | 26784992 | 26784992 | Missense_Mutation | SNP | C | C | T | novel | Somatic | G914E | 0.055882 | NM_002223.4 | 680 | 642 | 38 |
| 786-PTsup | SENP1 | 119 | hg19 | chr12 | 48458895 | 48458895 | Frame_Shift_Ins | INS | - | - | T | novel | Somatic | G410RfsTer3 | 0.020408 | NM_001267595.2 | 538 | 527 | 11 |
| 786-PTsup | KRT81 | 986 | hg19 | chr12 | 52685152 | 52685152 | Missense_Mutation | SNP | C | C | T | novel | Somatic | R33H | 0.096563 | NM_002281.4 | 1833 | 1656 | 177 |
| 786-PTsup | ITGA5 | 1003 | hg19 | chr12 | 54797710 | 54797710 | Missense_Mutation | SNP | C | C | T | novel | Somatic | V536I | 0.043646 | NM_002205.5 | 779 | 745 | 34 |
| 786-PTsup | DGKA | 1014 | hg19 | chr12 | 56332722 | 56332722 | Silent | SNP | G | G | A | novel | Somatic | Q141= | 0.263473 | NM_001345.5 | 668 | 492 | 176 |
| 786-PTsup | AVIL | 128 | hg19 | chr12 | 58195985 | 58195985 | Missense_Mutation | SNP | G | G | C | novel | Somatic | I703M | 0.284672 | NM_006576.3 | 959 | 686 | 273 |
| 786-PTsup | IKBIP | 1340 | hg19 | chr12 | 99038376 | 99038376 | Missense_Mutation | SNP | C | C | T | novel | Somatic | S35N | 0.038896 | NM_153687.4 | 796 | 765 | 31 |
| 786-PTsup | EP400 | 199 | hg19 | chr12 | 132534890 | 132534890 | Missense_Mutation | SNP | G | G | A | novel | Somatic | R2408H | 0.023622 | NM_015409.5 | 762 | 744 | 18 |
| 786-PTsup | LACC1 | 924 | hg19 | chr13 | 44464265 | 44464265 | Missense_Mutation | SNP | G | G | T | novel | Somatic | Q383H | 0.028169 | NM_153218.4 | 639 | 621 | 18 |
| 786-PTsup | SCEL | 147 | hg19 | chr13 | 78177197 | 78177197 | Intron | DEL | T | T | - | novel | Somatic |  | 0.035514 | NM_144777.3 | 535 | 516 | 19 |
| 786-PTsup | MYO16 | 177 | hg19 | chr13 | 109793062 | 109793062 | Missense_Mutation | SNP | C | C | T | novel | Somatic | P1501L | 0.287456 | NM_001198950.3 | 573 | 408 | 165 |
| 786-PTsup | NOVA1 | 98 | hg19 | chr14 | 26917586 | 26917586 | Missense_Mutation | SNP | T | T | C | novel | Somatic | E368G | 0.31477 | NM_002515.3 | 825 | 565 | 260 |
| 786-PTsup | SYNE2 | 1078 | hg19 | chr14 | 64688292 | 64688292 | Splice_Site | SNP | A | A | T | novel | Somatic |  | 0.068966 | NM_182914.2 | 696 | 648 | 48 |
| 786-PTsup | BCL11B | 1345 | hg19 | chr14 | 99641432 | 99641432 | In_Frame_Del | DEL | CCCCCGCGCCCGGGA | CCCCCGCGCCCGGGA | - | novel | Somatic | V576_G580del | 0.099408 | NM_138576.4 | 845 | 761 | 84 |
| 786-PTsup | CTXN2 | 954 | hg19 | chr15 | 48493566 | 48493566 | Missense_Mutation | SNP | G | G | C | novel | Somatic | L23F | 0.299242 | NM_001145668.2 | 528 | 370 | 158 |
| 786-PTsup | SEPT12 | 621 | hg19 | chr16 | 4835884 | 4835884 | Missense_Mutation | SNP | C | C | G | novel | Somatic | E100Q | 0.061111 | NM_144605.5 | 900 | 845 | 55 |
| 786-PTsup | ABCC6 | 709 | hg19 | chr16 | 16253377 | 16253377 | In_Frame_Del | DEL | CTGACACGATGCTGTTCTCTAGGT | CTGACACGATGCTGTTCTCTAGGT | - | novel | Somatic | D1225_S1232del | 0.038621 | NM_001171.5 | 725 | 697 | 28 |
| 786-PTsup | SRCAP | 819 | hg19 | chr16 | 30722379 | 30722379 | Missense_Mutation | SNP | G | G | T | novel | Somatic | A430S | 0.098124 | NM_006662.3 | 693 | 625 | 68 |
| 786-PTsup | ZFHX3 | 142 | hg19 | chr16 | 72923654 | 72923654 | Missense_Mutation | SNP | G | G | A | novel | Somatic | R1142C | 0.070111 | NM_006885.4 | 542 | 504 | 38 |
| 786-PTsup | C16orf95 | 1251 | hg19 | chr16 | 87350781 | 87350781 | Missense_Mutation | SNP | G | G | C | novel | Somatic | A23G | 0.073063 | NM_001195124.2 | 1136 | 1053 | 83 |
| 786-PTsup | C16orf95 | 1251 | hg19 | chr16 | 87350821 | 87350821 | Missense_Mutation | SNP | G | G | T | novel | Somatic | P10T | 0.073722 | NM_001195124.2 | 839 | 777 | 62 |
| 786-PTsup | TP53 | 642 | hg19 | chr17 | 7576872 | 7576872 | Missense_Mutation | SNP | C | C | G | novel | Somatic | G325A | 0.060638 | NM_000546.5 | 940 | 883 | 57 |
| 786-PTsup | TP53 | 642 | hg19 | chr17 | 7577094 | 7577094 | Missense_Mutation | SNP | G | G | A | novel | Somatic | R282W | 0.351327 | NM_000546.5 | 1093 | 709 | 384 |
| 786-PTsup | TP53 | 642 | hg19 | chr17 | 7578212 | 7578212 | Nonsense_Mutation | SNP | G | G | A | novel | Somatic | R213Ter | 0.009132 | NM_000546.5 | 875 | 867 | 8 |
| 786-PTsup | DNAH2 | 643 | hg19 | chr17 | 7719990 | 7719990 | Missense_Mutation | SNP | G | G | C | novel | Somatic | E3277D | 0.08561 | NM_020877.4 | 1097 | 1003 | 94 |
| 786-PTsup | CCDC42 | 650 | hg19 | chr17 | 8644964 | 8644964 | Missense_Mutation | SNP | G | G | A | novel | Somatic | A107V | 0.098375 | NM_144681.3 | 1169 | 1054 | 115 |
| 786-PTsup | ZNF287 | 710 | hg19 | chr17 | 16469877 | 16469877 | Missense_Mutation | SNP | C | C | T | novel | Somatic | A155T | 0.072632 | NM_001346167.1 | 950 | 881 | 69 |
| 786-PTsup | FLII | 723 | hg19 | chr17 | 18149571 | 18149571 | Intron | SNP | G | G | A | novel | Somatic |  | 0.309585 | NM_002018.4 | 771 | 532 | 239 |
| 786-PTsup | TMEM132E | 836 | hg19 | chr17 | 32959750 | 32959750 | Missense_Mutation | SNP | G | G | T | novel | Somatic | V504L | 0.226772 | NM_001304438.2 | 635 | 491 | 144 |
| 786-PTsup | SRCIN1 | 13 | hg19 | chr17 | 36700087 | 36700087 | Nonsense_Mutation | SNP | C | C | A | novel | Somatic | E1130Ter | 0.120516 | NM_025248.3 | 696 | 612 | 84 |
| 786-PTsup | KRT14 | 888 | hg19 | chr17 | 39738757 | 39738757 | Missense_Mutation | SNP | G | G | A | novel | Somatic | T450I | 0.03011 | NM_000526.5 | 1726 | 1674 | 52 |
| 786-PTsup | SLC4A1 | 113 | hg19 | chr17 | 42328788 | 42328788 | Missense_Mutation | SNP | C | C | A | novel | Somatic | R827L | 0.213811 | NM_000342.4 | 752 | 591 | 161 |
| 786-PTsup | ABCA6 | 0 | hg19 | chr17 | 67132294 | 67132294 | Missense_Mutation | SNP | G | G | C | novel | Somatic | F133L | 0.344444 | NM_080284.3 | 630 | 413 | 217 |
| 786-PTsup | UBE2O | 17 | hg19 | chr17 | 74388088 | 74388088 | Frame_Shift_Ins | INS | - | - | G | novel | Somatic | H1018PfsTer18 | 0.040373 | NM_022066.4 | 964 | 925 | 39 |
| 786-PTsup | FN3KRP | 1200 | hg19 | chr17 | 80680740 | 80680740 | Missense_Mutation | SNP | C | C | T | novel | Somatic | T149M | 0.125926 | NM_024619.4 | 810 | 708 | 102 |
| 786-PTsup | DLGAP1 | 76 | hg19 | chr18 | 3879700 | 3879700 | Nonsense_Mutation | SNP | G | G | T | novel | Somatic | Y123Ter | 0.05423 | NM_004746.4 | 1383 | 1308 | 75 |
| 786-PTsup | ARHGAP28 | 637 | hg19 | chr18 | 6868227 | 6868227 | Missense_Mutation | SNP | A | A | G | novel | Somatic | I269V | 0.030303 | NM_001366230.1 | 692 | 671 | 21 |
| 786-PTsup | ANKRD12 | 81 | hg19 | chr18 | 9258237 | 9258237 | Missense_Mutation | SNP | G | G | A | novel | Somatic | G1658R | 0.068966 | NM_015208.5 | 609 | 567 | 42 |
| 786-PTsup | ANKRD12 | 81 | hg19 | chr18 | 9258794 | 9258794 | Frame_Shift_Ins | INS | - | - | AA | novel | Somatic | I1846KfsTer2 | 0.049444 | NM_015208.5 | 803 | 763 | 40 |
| 786-PTsup | MEP1B | 812 | hg19 | chr18 | 29787254 | 29787254 | Nonsense_Mutation | SNP | C | C | G | novel | Somatic | S196Ter | 0.182336 | NM_005925.3 | 351 | 287 | 64 |
| 786-PTsup | ZNF532 | 15 | hg19 | chr18 | 56587042 | 56587042 | Missense_Mutation | SNP | C | C | T | novel | Somatic | P508L | 0.088339 | NM_001353526.2 | 566 | 516 | 50 |
| 786-PTsup | POLRMT | 589 | hg19 | chr19 | 633469 | 633469 | Missense_Mutation | SNP | C | C | T | novel | Somatic | R15Q | 0.032895 | NM_005035.4 | 456 | 441 | 15 |
| 786-PTsup | FSTL3 | 590 | hg19 | chr19 | 681344 | 681344 | Missense_Mutation | SNP | C | C | T | novel | Somatic | H173Y | 0.06015 | NM_005860.3 | 665 | 625 | 40 |
| 786-PTsup | PALM | 590 | hg19 | chr19 | 746417 | 746417 | Missense_Mutation | SNP | C | C | A | novel | Somatic | T256N | 0.102163 | NM_002579.3 | 832 | 747 | 85 |
| 786-PTsup | MUC16 | 81 | hg19 | chr19 | 9061158 | 9061158 | Missense_Mutation | SNP | G | G | A | novel | Somatic | P8763L | 0.081877 | NM_024690.2 | 1087 | 998 | 89 |
| 786-PTsup | ZNF121 | 658 | hg19 | chr19 | 9676921 | 9676921 | Frame_Shift_Ins | INS | - | - | A | novel | Somatic | G290WfsTer12 | 0.196667 | NM_001308269.2 | 598 | 480 | 118 |
| 786-PTsup | UNC13A | 720 | hg19 | chr19 | 17778978 | 17778978 | Missense_Mutation | SNP | C | C | T | novel | Somatic | R139H | 0.160093 | NM_001080421.2 | 431 | 362 | 69 |
| 786-PTsup | CCNE1 | 816 | hg19 | chr19 | 30312922 | 30312922 | Missense_Mutation | SNP | G | G | C | novel | Somatic | S242T | 0.26455 | NM_001238.4 | 378 | 278 | 100 |
| 786-PTsup | KCTD15 | 846 | hg19 | chr19 | 34292217 | 34292217 | Missense_Mutation | SNP | T | T | C | novel | Somatic | L71P | 0.036706 | NM_001129994.2 | 1008 | 971 | 37 |
| 786-PTsup | SELENOV | 890 | hg19 | chr19 | 40005917 | 40005917 | Missense_Mutation | SNP | C | C | G | novel | Somatic | S22C | 0.025641 | NM_182704.1 | 585 | 570 | 15 |
| 786-PTsup | AXL | 903 | hg19 | chr19 | 41727944 | 41727944 | Missense_Mutation | SNP | G | G | A | novel | Somatic | R190H | 0.120909 | NM_021913.5 | 1100 | 967 | 133 |
| 786-PTsup | SLC17A7 | 120 | hg19 | chr19 | 49935820 | 49935820 | Missense_Mutation | SNP | C | C | T | novel | Somatic | R369H | 0.092166 | NM_020309.4 | 651 | 591 | 60 |
| 786-PTsup | BCL2L12 | 967 | hg19 | chr19 | 50169198 | 50169198 | In_Frame_Del | DEL | CGCTGGGCCGGTTATCGACCCGGCCCAGTG | CGCTGGGCCGGTTATCGACCCGGCCCAGTG | - | novel | Somatic | W41_R50del | 0.037433 | NM_138639.1 | 561 | 540 | 21 |
| 786-PTsup | PPP2R1A | 987 | hg19 | chr19 | 52714628 | 52714628 | Missense_Mutation | SNP | T | T | C | novel | Somatic | V129A | 0.027778 | NM_014225.6 | 827 | 804 | 23 |
| 786-PTsup | ZNF667 | 1019 | hg19 | chr19 | 56972076 | 56972076 | Missense_Mutation | SNP | G | G | A | novel | Somatic | R48W | 0.102273 | NM_001321356.2 | 350 | 314 | 36 |
| 786-PTsup | ZNF71 | 1020 | hg19 | chr19 | 57134055 | 57134055 | Missense_Mutation | SNP | G | G | A | novel | Somatic | R527Q | 0.113102 | NM_001370215.1 | 892 | 791 | 101 |
| 786-PTsup | PSMF1 | 593 | hg19 | chr20 | 1106228 | 1106228 | Missense_Mutation | SNP | G | G | A | novel | Somatic | D73N | 0.085575 | NM_178578.3 | 818 | 748 | 70 |
| 786-PTsup | GINS1 | 97 | hg19 | chr20 | 25422396 | 25422396 | Frame_Shift_Ins | INS | - | - | A | novel | Somatic | N172KfsTer2 | 0.022284 | NM_021067.5 | 1076 | 1052 | 24 |
| 786-PTsup | XKR7 | 818 | hg19 | chr20 | 30584887 | 30584887 | Missense_Mutation | SNP | G | G | A | novel | Somatic | R456H | 0.149499 | NM_001011718.2 | 1097 | 933 | 164 |
| 786-PTsup | MYH7B | 1 | hg19 | chr20 | 33588157 | 33588157 | Missense_Mutation | SNP | C | C | T | novel | Somatic | R1657C | 0.115602 | NM_020884.5 | 1064 | 941 | 123 |
| 786-PTsup | AP1B1 | 101 | hg19 | chr22 | 29727810 | 29727810 | Missense_Mutation | SNP | G | G | A | novel | Somatic | S802L | 0.097849 | NM_001127.3 | 930 | 839 | 91 |
| 786-PTsup | CENPI | 1350 | hg19 | chrX | 100416521 | 100416521 | Missense_Mutation | SNP | G | G | A | novel | Somatic | S700N | 0.108779 | NM_006733.3 | 1046 | 932 | 114 |
| 786-PTsup | PASD1 | 216 | hg19 | chrX | 150780169 | 150780169 | Missense_Mutation | SNP | G | G | A | novel | Somatic | D51N | 0.033259 | NM_173493.3 | 451 | 436 | 15 |
| 786-PTdeep | VPS13D | 84 | hg19 | chr1 | 12337663 | 12337663 | Missense_Mutation | SNP | G | G | C | novel | Somatic | V1340L | 0.089701 | NM_015378.4 | 301 | 274 | 27 |
| 786-PTdeep | RLF | 111 | hg19 | chr1 | 40702375 | 40702375 | Missense_Mutation | SNP | C | C | G | novel | Somatic | D667E | 0.038462 | NM_012421.4 | 468 | 450 | 18 |
| 786-PTdeep | TIE1 | 114 | hg19 | chr1 | 43778945 | 43778945 | Missense_Mutation | SNP | C | C | A | novel | Somatic | D689E | 0.138889 | NM_005424.5 | 933 | 803 | 130 |
| 786-PTdeep | LRRC53 | Unknown | hg19 | chr1 | 74941228 | 74941228 | Missense_Mutation | SNP | C | C | T | novel | Somatic | E391K | 0.05988 | NM_001364665.1 | 334 | 314 | 20 |
| 786-PTdeep | CCDC18 | 162 | hg19 | chr1 | 93676474 | 93676474 | Missense_Mutation | SNP | C | C | T | novel | Somatic | S442L | 0.111111 | NM_001306076.1 | 420 | 373 | 47 |
| 786-PTdeep | DPYD | 166 | hg19 | chr1 | 97771805 | 97771805 | Missense_Mutation | SNP | G | G | C | novel | Somatic | Q703E | 0.129412 | NM_000110.4 | 510 | 444 | 66 |
| 786-PTdeep | AGL | 1350 | hg19 | chr1 | 100378028 | 100378028 | Frame_Shift_Del | DEL | A | A | - | novel | Somatic | N1304IfsTer10 | 0.027027 | NM_000028.2 | 555 | 540 | 15 |
| 786-PTdeep | PI4KB | 1739 | hg19 | chr1 | 151265889 | 151265889 | Silent | SNP | C | C | T | novel | Somatic | V773= | 0.174603 | NM_001369626.1 | 566 | 467 | 99 |
| 786-PTdeep | ATP8B2 | 1762 | hg19 | chr1 | 154315769 | 154315769 | Missense_Mutation | SNP | C | C | T | novel | Somatic | S564L | 0.171913 | NM_001367934.1 | 413 | 342 | 71 |
| 786-PTdeep | IQGAP3 | 222 | hg19 | chr1 | 156503808 | 156503808 | Missense_Mutation | SNP | G | G | A | novel | Somatic | T1289M | 0.199446 | NM_178229.5 | 361 | 289 | 72 |
| 786-PTdeep | CD5L | 223 | hg19 | chr1 | 157804287 | 157804287 | Missense_Mutation | SNP | C | C | T | novel | Somatic | G210R | 0.083871 | NM_005894.3 | 620 | 568 | 52 |
| 786-PTdeep | LHX4 | 244 | hg19 | chr1 | 180235585 | 180235585 | Missense_Mutation | SNP | C | C | T | novel | Somatic | R103C | 0.112595 | NM_033343.4 | 523 | 464 | 59 |
| 786-PTdeep | GNPAT | 2350 | hg19 | chr1 | 231403606 | 231403606 | Frame_Shift_Del | DEL | AA | AA | - | novel | Somatic | K413RfsTer14 | 0.052493 | NM_014236.4 | 381 | 361 | 20 |
| 786-PTdeep | OR2L13 | 309 | hg19 | chr1 | 248263030 | 248263030 | Missense_Mutation | SNP | C | C | A | novel | Somatic | A118D | 0.195857 | NM_175911.3 | 531 | 427 | 104 |
| 786-PTdeep | MYT1L | 9 | hg19 | chr2 | 1947002 | 1947002 | Missense_Mutation | SNP | G | G | A | novel | Somatic | S86L | 0.079861 | NM_001329844.2 | 575 | 529 | 46 |
| 786-PTdeep | FOSL2 | 803 | hg19 | chr2 | 28631638 | 28631638 | In_Frame_Del | DEL | GAG | GAG | - | novel | Somatic | E125del | 0.094203 | NM_005253.4 | 552 | 500 | 52 |
| 786-PTdeep | ZFP36L2 | 916 | hg19 | chr2 | 43452520 | 43452520 | In_Frame_Del | DEL | CTG | CTG | - | novel | Somatic | Q141del | 0.020484 | NM_006887.5 | 536 | 525 | 11 |
| 786-PTdeep | EFEMP1 | 126 | hg19 | chr2 | 56103844 | 56103844 | Missense_Mutation | SNP | G | G | A | novel | Somatic | A265V | 0.186544 | NM_001039348.3 | 327 | 266 | 61 |
| 786-PTdeep | ITPRIPL1 | 165 | hg19 | chr2 | 96993304 | 96993304 | Missense_Mutation | SNP | A | A | G | novel | Somatic | H320R | 0.077044 | NM_178495.6 | 636 | 587 | 49 |
| 786-PTdeep | PDE11A | 243 | hg19 | chr2 | 178592756 | 178592756 | Missense_Mutation | SNP | C | C | G | novel | Somatic | E645Q | 0.189003 | NM_016953.4 | 291 | 236 | 55 |
| 786-PTdeep | TTN | 1955 | hg19 | chr2 | 179431582 | 179431582 | Missense_Mutation | SNP | C | C | G | novel | Somatic | R26426T | 0.028497 | NM_001267550.2 | 386 | 375 | 11 |
| 786-PTdeep | TTN | 1955 | hg19 | chr2 | 179432156 | 179432156 | Missense_Mutation | SNP | C | C | T | novel | Somatic | E26235K | 0.07561 | NM_001267550.2 | 410 | 379 | 31 |
| 786-PTdeep | TTN | 1955 | hg19 | chr2 | 179432170 | 179432170 | Missense_Mutation | SNP | C | C | T | novel | Somatic | G26230E | 0.078947 | NM_001267550.2 | 456 | 420 | 36 |
| 786-PTdeep | TTN | 1955 | hg19 | chr2 | 179433944 | 179433944 | Missense_Mutation | SNP | C | C | T | novel | Somatic | E25639K | 0.104046 | NM_001267550.2 | 346 | 310 | 36 |
| 786-PTdeep | TTN | 1955 | hg19 | chr2 | 179455905 | 179455905 | Missense_Mutation | DNP | CC | CC | TT | novel | Somatic | D20183N | 0.080357 | NM_001267550.2 | 444 | 408 | 36 |
| 786-PTdeep | TTN | 1955 | hg19 | chr2 | 179456389 | 179456389 | Nonsense_Mutation | SNP | C | C | A | novel | Somatic | E20053Ter | 0.076605 | NM_001267550.2 | 482 | 445 | 37 |
| 786-PTdeep | TTN | 1955 | hg19 | chr2 | 179458008 | 179458008 | Missense_Mutation | SNP | C | C | G | novel | Somatic | D19643H | 0.082051 | NM_001267550.2 | 390 | 358 | 32 |
| 786-PTdeep | TTN | 1955 | hg19 | chr2 | 179458511 | 179458511 | Missense_Mutation | SNP | C | C | A | novel | Somatic | D19506Y | 0.077994 | NM_001267550.2 | 356 | 328 | 28 |
| 786-PTdeep | KANSL1L | 274 | hg19 | chr2 | 210894528 | 210894528 | Missense_Mutation | SNP | C | C | T | novel | Somatic | R757Q | 0.147343 | NM_152519.4 | 414 | 353 | 61 |
| 786-PTdeep | C2orf54 | 303 | hg19 | chr2 | 241835333 | 241835333 | Missense_Mutation | SNP | G | G | A | novel | Somatic | R28W | 0.105386 | NM_001085437.3 | 427 | 382 | 45 |
| 786-PTdeep | ITPR1 | 77 | hg19 | chr3 | 4704840 | 4704840 | Missense_Mutation | SNP | C | C | A | novel | Somatic | P487T | 0.124248 | NM_001168272.1 | 499 | 437 | 62 |
| 786-PTdeep | ADAMTS9 | 134 | hg19 | chr3 | 64601699 | 64601699 | Missense_Mutation | SNP | G | G | C | novel | Somatic | S987R | 0.091743 | NM_182920.2 | 327 | 297 | 30 |
| 786-PTdeep | TRMT10C | 1357 | hg19 | chr3 | 101283906 | 101283906 | Missense_Mutation | SNP | C | C | A | novel | Somatic | T94N | 0.117117 | NM_017819.4 | 444 | 392 | 52 |
| 786-PTdeep | RNF7 | 1664 | hg19 | chr3 | 141462372 | 141462372 | Missense_Mutation | SNP | C | C | A | novel | Somatic | A66D | 0.132468 | NM_014245.5 | 770 | 668 | 102 |
| 786-PTdeep | TM4SF18 | 1722 | hg19 | chr3 | 149051163 | 149051163 | Missense_Mutation | SNP | A | A | G | novel | Somatic | S3P | 0.125373 | NM_138786.4 | 335 | 293 | 42 |
| 786-PTdeep | KLHL6 | 247 | hg19 | chr3 | 183210492 | 183210492 | Missense_Mutation | SNP | C | C | T | novel | Somatic | A452T | 0.068462 | NM_130446.4 | 888 | 827 | 61 |
| 786-PTdeep | ABCF3 | 1988 | hg19 | chr3 | 183908824 | 183908824 | Missense_Mutation | SNP | G | G | A | novel | Somatic | V477I | 0.273305 | NM_018358.2 | 472 | 343 | 129 |
| 786-PTdeep | GABRA4 | 117 | hg19 | chr4 | 46979602 | 46979602 | Frame_Shift_Del | DEL | T | T | - | novel | Somatic | R107DfsTer2 | 0.072539 | NM_000809.4 | 386 | 358 | 28 |
| 786-PTdeep | EXOSC9 | 1521 | hg19 | chr4 | 122731167 | 122731167 | Missense_Mutation | SNP | G | G | A | novel | Somatic | M217I | 0.046584 | NM_005033.3 | 321 | 306 | 15 |
| 786-PTdeep | FAT4 | 193 | hg19 | chr4 | 126373686 | 126373686 | Missense_Mutation | SNP | G | G | A | novel | Somatic | A3839T | 0.020284 | NM_024582.4 | 493 | 483 | 10 |
| 786-PTdeep | GALNT7 | 239 | hg19 | chr4 | 174219256 | 174219256 | Intron | SNP | C | C | G | novel | Somatic |  | 0.179941 | NM_017423.3 | 338 | 277 | 61 |
| 786-PTdeep | BRD9 | 591 | hg19 | chr5 | 891919 | 891919 | Missense_Mutation | SNP | C | C | T | novel | Somatic | G35R | 0.020218 | NM_023924.5 | 643 | 630 | 13 |
| 786-PTdeep | SLC12A7 | 593 | hg19 | chr5 | 1085509 | 1085509 | Missense_Mutation | SNP | C | C | T | novel | Somatic | R252H | 0.168103 | NM_006598.3 | 232 | 193 | 39 |
| 786-PTdeep | DNAJC21 | 851 | hg19 | chr5 | 34954061 | 34954061 | Missense_Mutation | SNP | A | A | T | novel | Somatic | K463N | 0.062257 | NM_001012339.3 | 514 | 482 | 32 |
| 786-PTdeep | EGFLAM | 878 | hg19 | chr5 | 38352350 | 38352350 | Silent | SNP | G | G | A | novel | Somatic | S154= | 0.083333 | NM_152403.4 | 576 | 528 | 48 |
| 786-PTdeep | CAST | 1318 | hg19 | chr5 | 96077047 | 96077047 | Missense_Mutation | SNP | G | G | C | novel | Somatic | K332N | 0.300353 | NM_001750.7 | 283 | 198 | 85 |
| 786-PTdeep | RAD50 | 1591 | hg19 | chr5 | 131923294 | 131923294 | Missense_Mutation | SNP | T | T | G | novel | Somatic | M266R | 0.301444 | NM_005732.4 | 553 | 386 | 167 |
| 786-PTdeep | PCDHA4 | 1654 | hg19 | chr5 | 140188648 | 140188648 | Missense_Mutation | SNP | C | C | A | novel | Somatic | L626M | 0.040704 | NM_018907.4 | 906 | 869 | 37 |
| 786-PTdeep | PCDHA13 | 1655 | hg19 | chr5 | 140263242 | 140263242 | Missense_Mutation | SNP | C | C | A | novel | Somatic | F463L | 0.455725 | NM_018904.3 | 1307 | 710 | 597 |
| 786-PTdeep | PCDHB6 | 1657 | hg19 | chr5 | 140531975 | 140531975 | Missense_Mutation | SNP | A | A | G | novel | Somatic | R713G | 0.126263 | NM_018939.3 | 1782 | 1557 | 225 |
| 786-PTdeep | PCDHGB2 | 1658 | hg19 | chr5 | 140741872 | 140741872 | Missense_Mutation | SNP | T | T | C | novel | Somatic | W724R | 0.220443 | NM_018923.2 | 812 | 633 | 179 |
| 786-PTdeep | PCDHGB7 | 1659 | hg19 | chr5 | 140797796 | 140797796 | Missense_Mutation | SNP | G | G | C | novel | Somatic | E124Q | 0.066566 | NM_018927.4 | 660 | 616 | 44 |
| 786-PTdeep | LARP1 | 27 | hg19 | chr5 | 154183118 | 154183118 | Missense_Mutation | SNP | C | C | A | novel | Somatic | A751D | 0.063927 | NM_033551.3 | 654 | 612 | 42 |
| 786-PTdeep | FGF18 | 1888 | hg19 | chr5 | 170863173 | 170863173 | Missense_Mutation | SNP | G | G | A | novel | Somatic | R49H | 0.073427 | NM_003862.3 | 572 | 530 | 42 |
| 786-PTdeep | CPLX2 | 1922 | hg19 | chr5 | 175306965 | 175306965 | In_Frame_Del | DEL | GAG | GAG | - | novel | Somatic | E114del | 0.027508 | NM_001008220.2 | 618 | 601 | 17 |
| 786-PTdeep | UIMC1 | 241 | hg19 | chr5 | 176338382 | 176338382 | Missense_Mutation | SNP | G | G | A | novel | Somatic | R536W | 0.130435 | NM_001199297.2 | 206 | 179 | 27 |
| 786-PTdeep | HIST1H2BD | 784 | hg19 | chr6 | 26158708 | 26158708 | Missense_Mutation | SNP | C | C | T | novel | Somatic | P104L | 0.188119 | NM_138720.2 | 2520 | 2045 | 475 |
| 786-PTdeep | ITPR3 | 841 | hg19 | chr6 | 33636332 | 33636332 | Missense_Mutation | SNP | A | A | G | novel | Somatic | I643V | 0.083916 | NM_002224.4 | 428 | 392 | 36 |
| 786-PTdeep | CLIC5 | 935 | hg19 | chr6 | 46047778 | 46047778 | Missense_Mutation | SNP | C | C | G | novel | Somatic | E68Q | 0.369803 | NM_001370650.1 | 457 | 288 | 169 |
| 786-PTdeep | PKHD1 | 122 | hg19 | chr6 | 51768466 | 51768466 | Missense_Mutation | SNP | C | C | T | novel | Somatic | E2309K | 0.128049 | NM_138694.4 | 328 | 286 | 42 |
| 786-PTdeep | ENPP3 | 198 | hg19 | chr6 | 131973973 | 131973973 | Missense_Mutation | SNP | G | G | C | novel | Somatic | E136Q | 0.090498 | NM_005021.5 | 221 | 201 | 20 |
| 786-PTdeep | VNN2 | 1600 | hg19 | chr6 | 133065485 | 133065485 | Missense_Mutation | SNP | A | A | T | novel | Somatic | F506Y | 0.064516 | NM_004665.5 | 216 | 202 | 14 |
| 786-PTdeep | HECA | 25 | hg19 | chr6 | 139488129 | 139488129 | Missense_Mutation | SNP | C | C | T | novel | Somatic | A327V | 0.137803 | NM_016217.3 | 537 | 463 | 74 |
| 786-PTdeep | UTRN | 26 | hg19 | chr6 | 144749911 | 144749911 | Splice_Region | INS | C | C | TAGACCT | novel | Somatic |  | 0.102041 | NM_007124.2 | 196 | 176 | 20 |
| 786-PTdeep | ADGB | 213 | hg19 | chr6 | 146956516 | 146956516 | Missense_Mutation | SNP | A | A | T | novel | Somatic | Y27F | 0.06867 | NM_024694.4 | 233 | 217 | 16 |
| 786-PTdeep | MAS1 | 1808 | hg19 | chr6 | 160328663 | 160328663 | Missense_Mutation | SNP | C | C | T | novel | Somatic | L226F | 0.041763 | NM_002377.4 | 431 | 413 | 18 |
| 786-PTdeep | DNAH11 | 93 | hg19 | chr7 | 21675499 | 21675499 | Missense_Mutation | SNP | A | A | T | novel | Somatic | Q1504L | 0.172185 | NM_001277115.2 | 302 | 250 | 52 |
| 786-PTdeep | TYW1 | 136 | hg19 | chr7 | 66703377 | 66703377 | Missense_Mutation | SNP | G | G | A | novel | Somatic | G687E | 0.059722 | NM_018264.4 | 719 | 676 | 43 |
| 786-PTdeep | ZNF804B | 157 | hg19 | chr7 | 88966101 | 88966101 | Missense_Mutation | SNP | C | C | A | novel | Somatic | H1269N | 0.125234 | NM_181646.5 | 535 | 468 | 67 |
| 786-PTdeep | PEG10 | 1304 | hg19 | chr7 | 94293685 | 94293685 | Missense_Mutation | SNP | C | C | G | novel | Somatic | P349A | 0.0721 | NM_001172437.2 | 637 | 591 | 46 |
| 786-PTdeep | ACTL6B | 1349 | hg19 | chr7 | 100252706 | 100252706 | Missense_Mutation | SNP | T | T | C | novel | Somatic | H102R | 0.191244 | NM_016188.5 | 434 | 351 | 83 |
| 786-PTdeep | POLR2J | 1364 | hg19 | chr7 | 102119287 | 102119287 | Missense_Mutation | SNP | G | G | C | novel | Somatic | F7L | 0.207723 | NM_006234.5 | 749 | 593 | 156 |
| 786-PTdeep | FGF17 | 752 | hg19 | chr8 | 21905599 | 21905599 | Missense_Mutation | SNP | C | C | T | novel | Somatic | R164C | 0.052632 | NM_003867.4 | 361 | 342 | 19 |
| 786-PTdeep | FAM110B | 129 | hg19 | chr8 | 59059902 | 59059902 | Nonstop_Mutation | SNP | A | A | T | novel | Somatic | Ter371YextTer24 | 0.13764 | NM_147189.3 | 356 | 307 | 49 |
| 786-PTdeep | RUNX1T1 | 1294 | hg19 | chr8 | 92988171 | 92988171 | Missense_Mutation | SNP | G | G | A | novel | Somatic | A496V | 0.224868 | NM_001198679.1 | 378 | 293 | 85 |
| 786-PTdeep | NTRK2 | 156 | hg19 | chr9 | 87339207 | 87339207 | Missense_Mutation | SNP | G | G | C | novel | Somatic | Q263H | 0.075949 | NM_006180.4 | 395 | 365 | 30 |
| 786-PTdeep | SHC3 | 160 | hg19 | chr9 | 91727466 | 91727466 | Splice_Region | SNP | C | C | G | novel | Somatic |  | 0.098246 | NM_016848.6 | 285 | 257 | 28 |
| 786-PTdeep | CRB2 | 1547 | hg19 | chr9 | 126136139 | 126136139 | Missense_Mutation | SNP | C | C | T | novel | Somatic | T1110M | 0.05618 | NM_173689.7 | 267 | 252 | 15 |
| 786-PTdeep | LCN1 | 1641 | hg19 | chr9 | 138415088 | 138415088 | Missense_Mutation | SNP | C | C | T | novel | Somatic | R78W | 0.162791 | NM_001252618.2 | 258 | 216 | 42 |
| 786-PTdeep | NPDC1 | 1652 | hg19 | chr9 | 139935154 | 139935154 | Missense_Mutation | SNP | C | C | T | novel | Somatic | A198T | 0.148225 | NM_015392.4 | 475 | 404 | 71 |
| 786-PTdeep | CDH23 | 1146 | hg19 | chr10 | 73548732 | 73548732 | Missense_Mutation | SNP | G | G | T | novel | Somatic | E1952D | 0.090439 | NM_022124.6 | 387 | 352 | 35 |
| 786-PTdeep | ECD | 1156 | hg19 | chr10 | 74896497 | 74896497 | Missense_Mutation | SNP | A | A | C | novel | Somatic | C557G | 0.213376 | NM_007265.3 | 314 | 247 | 67 |
| 786-PTdeep | TNKS2 | 162 | hg19 | chr10 | 93619393 | 93619393 | Missense_Mutation | SNP | A | A | G | novel | Somatic | Y1090C | 0.170588 | NM_025235.4 | 340 | 282 | 58 |
| 786-PTdeep | LCOR | 167 | hg19 | chr10 | 98711858 | 98711858 | Splice_Site | SNP | A | A | T | novel | Somatic |  | 0.193662 | NM_001346516.2 | 284 | 229 | 55 |
| 786-PTdeep | TMEM80 | 590 | hg19 | chr11 | 704540 | 704540 | Missense_Mutation | SNP | A | A | C | novel | Somatic | E249A | 0.110345 | NM_001276274.1 | 435 | 387 | 48 |
| 786-PTdeep | CALCA | 699 | hg19 | chr11 | 14992709 | 14992709 | Missense_Mutation | TNP | CAG | CAG | AAT | novel | Somatic | L10I | 0.15 | NM_001033952.2 | 300 | 255 | 45 |
| 786-PTdeep | OTOG | 719 | hg19 | chr11 | 17594807 | 17594807 | Missense_Mutation | SNP | G | G | A | novel | Somatic | V767I | 0.026178 | NM_001277269.2 | 382 | 372 | 10 |
| 786-PTdeep | NELL1 | 11 | hg19 | chr11 | 20968923 | 20968923 | Missense_Mutation | SNP | G | G | C | novel | Somatic | L371F | 0.171355 | NM_006157.5 | 390 | 323 | 67 |
| 786-PTdeep | NELL1 | 11 | hg19 | chr11 | 21596493 | 21596493 | Intron | SNP | T | T | G | novel | Somatic |  | 0.119898 | NM_006157.5 | 392 | 345 | 47 |
| 786-PTdeep | DDB2 | 945 | hg19 | chr11 | 47259481 | 47259481 | Missense_Mutation | SNP | G | G | A | novel | Somatic | D373N | 0.170635 | NM_000107.2 | 504 | 418 | 86 |
| 786-PTdeep | OR4P4 | 1007 | hg19 | chr11 | 55406213 | 55406213 | Missense_Mutation | SNP | C | C | A | novel | Somatic | P127H | 0.031847 | NM_001004124.2 | 313 | 303 | 10 |
| 786-PTdeep | OR5T3 | 1012 | hg19 | chr11 | 56020588 | 56020588 | Missense_Mutation | SNP | T | T | A | novel | Somatic | S305T | 0.273183 | NM_001004747.1 | 399 | 290 | 109 |
| 786-PTdeep | FAT3 | 2 | hg19 | chr11 | 92532370 | 92532370 | Missense_Mutation | SNP | G | G | A | novel | Somatic | R2064H | 0.224784 | NM_001367949.1 | 347 | 269 | 78 |
| 786-PTdeep | OR4D5 | 1529 | hg19 | chr11 | 123810436 | 123810436 | Missense_Mutation | SNP | C | C | G | novel | Somatic | T38S | 0.144876 | NM_001001965.1 | 282 | 241 | 41 |
| 786-PTdeep | PZP | 81 | hg19 | chr12 | 9317889 | 9317889 | Missense_Mutation | SNP | A | A | G | novel | Somatic | L778P | 0.062428 | NM_002864.3 | 865 | 811 | 54 |
| 786-PTdeep | TAS2R7 | 668 | hg19 | chr12 | 10955058 | 10955058 | Missense_Mutation | SNP | T | T | C | novel | Somatic | K38E | 0.076087 | NM_023919.2 | 368 | 340 | 28 |
| 786-PTdeep | PLBD1 | 10 | hg19 | chr12 | 14659897 | 14659897 | Missense_Mutation | SNP | C | C | T | novel | Somatic | A448T | 0.086242 | NM_024829.6 | 484 | 442 | 42 |
| 786-PTdeep | ITPR2 | 98 | hg19 | chr12 | 26784949 | 26784949 | Missense_Mutation | SNP | C | C | G | novel | Somatic | M928I | 0.117096 | NM_002223.4 | 854 | 754 | 100 |
| 786-PTdeep | ITPR2 | 98 | hg19 | chr12 | 26784992 | 26784992 | Missense_Mutation | SNP | C | C | T | novel | Somatic | G914E | 0.093023 | NM_002223.4 | 557 | 505 | 52 |
| 786-PTdeep | KRT81 | 986 | hg19 | chr12 | 52685152 | 52685152 | Missense_Mutation | SNP | C | C | T | novel | Somatic | R33H | 0.102871 | NM_002281.4 | 835 | 749 | 86 |
| 786-PTdeep | ITGA5 | 1003 | hg19 | chr12 | 54797710 | 54797710 | Missense_Mutation | SNP | C | C | T | novel | Somatic | V536I | 0.044534 | NM_002205.5 | 494 | 472 | 22 |
| 786-PTdeep | DGKA | 1014 | hg19 | chr12 | 56332722 | 56332722 | Silent | SNP | G | G | A | novel | Somatic | Q141= | 0.247678 | NM_001345.5 | 323 | 243 | 80 |
| 786-PTdeep | AVIL | 128 | hg19 | chr12 | 58195985 | 58195985 | Missense_Mutation | SNP | G | G | C | novel | Somatic | I703M | 0.315385 | NM_006576.3 | 780 | 534 | 246 |
| 786-PTdeep | MYO16 | 177 | hg19 | chr13 | 109793062 | 109793062 | Missense_Mutation | SNP | C | C | T | novel | Somatic | P1501L | 0.253571 | NM_001198950.3 | 280 | 209 | 71 |
| 786-PTdeep | NOVA1 | 98 | hg19 | chr14 | 26917586 | 26917586 | Missense_Mutation | SNP | T | T | C | novel | Somatic | E368G | 0.237037 | NM_002515.3 | 810 | 618 | 192 |
| 786-PTdeep | SYNE2 | 1078 | hg19 | chr14 | 64688292 | 64688292 | Splice_Site | SNP | A | A | T | novel | Somatic |  | 0.089069 | NM_182914.2 | 493 | 449 | 44 |
| 786-PTdeep | LIN52 | 144 | hg19 | chr14 | 74564551 | 74564551 | Missense_Mutation | SNP | G | G | A | novel | Somatic | D63N | 0.022624 | NM_001372005.1 | 441 | 431 | 10 |
| 786-PTdeep | BCL11B | 1345 | hg19 | chr14 | 99641432 | 99641432 | In_Frame_Del | DEL | CCCCCGCGCCCGGGA | CCCCCGCGCCCGGGA | - | novel | Somatic | V576_G580del | 0.195946 | NM_138576.4 | 296 | 238 | 58 |
| 786-PTdeep | CTXN2 | 954 | hg19 | chr15 | 48493566 | 48493566 | Missense_Mutation | SNP | G | G | C | novel | Somatic | L23F | 0.291228 | NM_001145668.2 | 285 | 202 | 83 |
| 786-PTdeep | PIGB | 1009 | hg19 | chr15 | 55631503 | 55631503 | Frame_Shift_Del | DEL | T | T | - | novel | Somatic | F280LfsTer12 | 0.022026 | NM_004855.5 | 454 | 444 | 10 |
| 786-PTdeep | BLM | 160 | hg19 | chr15 | 91304139 | 91304139 | Frame_Shift_Del | DEL | A | A | - | novel | Somatic | N515MfsTer16 | 0.023041 | NM_000057.4 | 434 | 424 | 10 |
| 786-PTdeep | SEPT12 | 621 | hg19 | chr16 | 4835884 | 4835884 | Missense_Mutation | SNP | C | C | G | novel | Somatic | E100Q | 0.066667 | NM_144605.5 | 495 | 462 | 33 |
| 786-PTdeep | ABCC6 | 709 | hg19 | chr16 | 16253377 | 16253377 | In_Frame_Del | DEL | CTGACACGATGCTGTTCTCTAGGT | CTGACACGATGCTGTTCTCTAGGT | - | novel | Somatic | D1225_S1232del | 0.036538 | NM_001171.5 | 520 | 501 | 19 |
| 786-PTdeep | SRCAP | 819 | hg19 | chr16 | 30722379 | 30722379 | Missense_Mutation | SNP | G | G | T | novel | Somatic | A430S | 0.123077 | NM_006662.3 | 260 | 228 | 32 |
| 786-PTdeep | ZFHX3 | 142 | hg19 | chr16 | 72923654 | 72923654 | Missense_Mutation | SNP | G | G | A | novel | Somatic | R1142C | 0.094286 | NM_006885.4 | 350 | 317 | 33 |
| 786-PTdeep | ADAMTS18 | 146 | hg19 | chr16 | 77356310 | 77356310 | Frame_Shift_Ins | INS | - | - | A | novel | Somatic | A696CfsTer18 | 0.028818 | NM_199355.4 | 345 | 335 | 10 |
| 786-PTdeep | C16orf95 | 1251 | hg19 | chr16 | 87350781 | 87350781 | Missense_Mutation | SNP | G | G | C | novel | Somatic | A23G | 0.080537 | NM_001195124.2 | 596 | 548 | 48 |
| 786-PTdeep | C16orf95 | 1251 | hg19 | chr16 | 87350821 | 87350821 | Missense_Mutation | SNP | G | G | T | novel | Somatic | P10T | 0.086498 | NM_001195124.2 | 474 | 433 | 41 |
| 786-PTdeep | ZFP3 | 623 | hg19 | chr17 | 4994828 | 4994828 | Missense_Mutation | SNP | C | C | A | novel | Somatic | P10H | 0.071429 | NM_153018.3 | 322 | 299 | 23 |
| 786-PTdeep | TP53 | 642 | hg19 | chr17 | 7576872 | 7576872 | Missense_Mutation | SNP | C | C | G | novel | Somatic | G325A | 0.069583 | NM_000546.5 | 503 | 468 | 35 |
| 786-PTdeep | TP53 | 642 | hg19 | chr17 | 7577094 | 7577094 | Missense_Mutation | SNP | G | G | A | novel | Somatic | R282W | 0.390947 | NM_000546.5 | 972 | 592 | 380 |
| 786-PTdeep | DNAH2 | 643 | hg19 | chr17 | 7719990 | 7719990 | Missense_Mutation | SNP | G | G | C | novel | Somatic | E3277D | 0.094301 | NM_020877.4 | 965 | 874 | 91 |
| 786-PTdeep | CCDC42 | 650 | hg19 | chr17 | 8644964 | 8644964 | Missense_Mutation | SNP | G | G | A | novel | Somatic | A107V | 0.130653 | NM_144681.3 | 594 | 516 | 78 |
| 786-PTdeep | ZNF287 | 710 | hg19 | chr17 | 16469877 | 16469877 | Missense_Mutation | SNP | C | C | T | novel | Somatic | A155T | 0.070938 | NM_001346167.1 | 436 | 405 | 31 |
| 786-PTdeep | FLII | 723 | hg19 | chr17 | 18149571 | 18149571 | Intron | SNP | G | G | A | novel | Somatic |  | 0.272727 | NM_002018.4 | 351 | 255 | 96 |
| 786-PTdeep | TMEM132E | 836 | hg19 | chr17 | 32959750 | 32959750 | Missense_Mutation | SNP | G | G | T | novel | Somatic | V504L | 0.181347 | NM_001304438.2 | 386 | 316 | 70 |
| 786-PTdeep | SRCIN1 | 13 | hg19 | chr17 | 36700087 | 36700087 | Nonsense_Mutation | SNP | C | C | A | novel | Somatic | E1130Ter | 0.117438 | NM_025248.3 | 281 | 248 | 33 |
| 786-PTdeep | SLC4A1 | 113 | hg19 | chr17 | 42328788 | 42328788 | Missense_Mutation | SNP | C | C | A | novel | Somatic | R827L | 0.238443 | NM_000342.4 | 409 | 311 | 98 |
| 786-PTdeep | ABCA6 | 0 | hg19 | chr17 | 67132294 | 67132294 | Missense_Mutation | SNP | G | G | C | novel | Somatic | F133L | 0.379404 | NM_080284.3 | 369 | 229 | 140 |
| 786-PTdeep | UBE2O | 17 | hg19 | chr17 | 74388088 | 74388088 | Frame_Shift_Ins | INS | - | - | G | novel | Somatic | H1018PfsTer18 | 0.077465 | NM_022066.4 | 567 | 523 | 44 |
| 786-PTdeep | FN3KRP | 1200 | hg19 | chr17 | 80680740 | 80680740 | Missense_Mutation | SNP | C | C | T | novel | Somatic | T149M | 0.161638 | NM_024619.4 | 463 | 388 | 75 |
| 786-PTdeep | ANKRD12 | 81 | hg19 | chr18 | 9258237 | 9258237 | Missense_Mutation | SNP | G | G | A | novel | Somatic | G1658R | 0.052632 | NM_015208.5 | 341 | 323 | 18 |
| 786-PTdeep | ANKRD12 | 81 | hg19 | chr18 | 9258794 | 9258794 | Frame_Shift_Ins | INS | - | - | AA | novel | Somatic | I1846KfsTer2 | 0.043379 | NM_015208.5 | 438 | 419 | 19 |
| 786-PTdeep | MEP1B | 812 | hg19 | chr18 | 29787254 | 29787254 | Nonsense_Mutation | SNP | C | C | G | novel | Somatic | S196Ter | 0.151042 | NM_005925.3 | 192 | 163 | 29 |
| 786-PTdeep | ZNF532 | 15 | hg19 | chr18 | 56587042 | 56587042 | Missense_Mutation | SNP | C | C | T | novel | Somatic | P508L | 0.086066 | NM_001353526.2 | 487 | 445 | 42 |
| 786-PTdeep | PALM | 590 | hg19 | chr19 | 746417 | 746417 | Missense_Mutation | SNP | C | C | A | novel | Somatic | T256N | 0.107345 | NM_002579.3 | 354 | 316 | 38 |
| 786-PTdeep | MUC16 | 81 | hg19 | chr19 | 9061158 | 9061158 | Missense_Mutation | SNP | G | G | A | novel | Somatic | P8763L | 0.073585 | NM_024690.2 | 530 | 491 | 39 |
| 786-PTdeep | ZNF121 | 658 | hg19 | chr19 | 9676921 | 9676921 | Frame_Shift_Ins | INS | - | - | A | novel | Somatic | G290WfsTer12 | 0.256798 | NM_001308269.2 | 328 | 243 | 85 |
| 786-PTdeep | AKAP8 | 87 | hg19 | chr19 | 15473013 | 15473013 | Missense_Mutation | SNP | C | C | G | novel | Somatic | E404Q | 0.025974 | NM_005858.4 | 538 | 524 | 14 |
| 786-PTdeep | UNC13A | 720 | hg19 | chr19 | 17778978 | 17778978 | Missense_Mutation | SNP | C | C | T | novel | Somatic | R139H | 0.097656 | NM_001080421.2 | 256 | 231 | 25 |
| 786-PTdeep | CCNE1 | 816 | hg19 | chr19 | 30312922 | 30312922 | Missense_Mutation | SNP | G | G | C | novel | Somatic | S242T | 0.301802 | NM_001238.4 | 222 | 155 | 67 |
| 786-PTdeep | AXL | 903 | hg19 | chr19 | 41727944 | 41727944 | Missense_Mutation | SNP | G | G | A | novel | Somatic | R190H | 0.097879 | NM_021913.5 | 611 | 551 | 60 |
| 786-PTdeep | SLC17A7 | 120 | hg19 | chr19 | 49935820 | 49935820 | Missense_Mutation | SNP | C | C | T | novel | Somatic | R369H | 0.06746 | NM_020309.4 | 252 | 235 | 17 |
| 786-PTdeep | BCL2L12 | 967 | hg19 | chr19 | 50169198 | 50169198 | In_Frame_Del | DEL | CGCTGGGCCGGTTATCGACCCGGCCCAGTG | CGCTGGGCCGGTTATCGACCCGGCCCAGTG | - | novel | Somatic | W41_R50del | 0.040169 | NM_138639.1 | 473 | 454 | 19 |
| 786-PTdeep | ZNF71 | 1020 | hg19 | chr19 | 57134055 | 57134055 | Missense_Mutation | SNP | G | G | A | novel | Somatic | R527Q | 0.123667 | NM_001370215.1 | 468 | 410 | 58 |
| 786-PTdeep | PSMF1 | 593 | hg19 | chr20 | 1106228 | 1106228 | Missense_Mutation | SNP | G | G | A | novel | Somatic | D73N | 0.090069 | NM_178578.3 | 433 | 394 | 39 |
| 786-PTdeep | XKR7 | 818 | hg19 | chr20 | 30584887 | 30584887 | Missense_Mutation | SNP | G | G | A | novel | Somatic | R456H | 0.214971 | NM_001011718.2 | 521 | 409 | 112 |
| 786-PTdeep | MYH7B | 1 | hg19 | chr20 | 33588157 | 33588157 | Missense_Mutation | SNP | C | C | T | novel | Somatic | R1657C | 0.158192 | NM_020884.5 | 531 | 447 | 84 |
| 786-PTdeep | AP1B1 | 101 | hg19 | chr22 | 29727810 | 29727810 | Missense_Mutation | SNP | G | G | A | novel | Somatic | S802L | 0.091153 | NM_001127.3 | 372 | 338 | 34 |
| 786-PTdeep | CENPI | 1350 | hg19 | chrX | 100416521 | 100416521 | Missense_Mutation | SNP | G | G | A | novel | Somatic | S700N | 0.102174 | NM_006733.3 | 459 | 412 | 47 |
| 786-LNmet | EPHA8 | 759 | hg19 | chr1 | 22915511 | 22915511 | Missense_Mutation | SNP | G | G | A | novel | Somatic | C376Y | 0.036891 | NM_020526.5 | 759 | 731 | 28 |
| 786-LNmet | ZMYND12 | 912 | hg19 | chr1 | 42905586 | 42905586 | Missense_Mutation | SNP | C | C | T | novel | Somatic | G179R | 0.037647 | NM_032257.5 | 424 | 408 | 16 |
| 786-LNmet | TIE1 | 114 | hg19 | chr1 | 43778945 | 43778945 | Missense_Mutation | SNP | C | C | A | novel | Somatic | D689E | 0.083945 | NM_005424.5 | 953 | 873 | 80 |
| 786-LNmet | LRRC53 | Unknown | hg19 | chr1 | 74941228 | 74941228 | Missense_Mutation | SNP | C | C | T | novel | Somatic | E391K | 0.040816 | NM_001364665.1 | 294 | 282 | 12 |
| 786-LNmet | CCDC18 | 162 | hg19 | chr1 | 93676474 | 93676474 | Missense_Mutation | SNP | C | C | T | novel | Somatic | S442L | 0.05914 | NM_001306076.1 | 558 | 525 | 33 |
| 786-LNmet | DPYD | 166 | hg19 | chr1 | 97771805 | 97771805 | Missense_Mutation | SNP | G | G | C | novel | Somatic | Q703E | 0.084936 | NM_000110.4 | 624 | 571 | 53 |
| 786-LNmet | PI4KB | 1739 | hg19 | chr1 | 151265889 | 151265889 | Silent | SNP | C | C | T | novel | Somatic | V773= | 0.079038 | NM_001369626.1 | 872 | 803 | 69 |
| 786-LNmet | ATP8B2 | 1762 | hg19 | chr1 | 154315769 | 154315769 | Missense_Mutation | SNP | C | C | T | novel | Somatic | S564L | 0.049731 | NM_001367934.1 | 744 | 707 | 37 |
| 786-LNmet | IQGAP3 | 222 | hg19 | chr1 | 156503808 | 156503808 | Missense_Mutation | SNP | G | G | A | novel | Somatic | T1289M | 0.078059 | NM_178229.5 | 474 | 437 | 37 |
| 786-LNmet | SLAMF9 | 1805 | hg19 | chr1 | 159922278 | 159922278 | Silent | SNP | C | C | G | novel | Somatic | G146= | 0.049488 | NM_033438.4 | 585 | 556 | 29 |
| 786-LNmet | LHX4 | 244 | hg19 | chr1 | 180235585 | 180235585 | Missense_Mutation | SNP | C | C | T | novel | Somatic | R103C | 0.054276 | NM_033343.4 | 608 | 575 | 33 |
| 786-LNmet | USH2A | 279 | hg19 | chr1 | 216405428 | 216405428 | Nonsense_Mutation | DNP | GG | GG | AT | novel | Somatic | C953_H954delinsTer | 0.033149 | NM_206933.3 | 360 | 348 | 12 |
| 786-LNmet | OR2L13 | 309 | hg19 | chr1 | 248263030 | 248263030 | Missense_Mutation | SNP | C | C | A | novel | Somatic | A118D | 0.087413 | NM_175911.3 | 572 | 522 | 50 |
| 786-LNmet | MYT1L | 9 | hg19 | chr2 | 1947002 | 1947002 | Missense_Mutation | SNP | G | G | A | novel | Somatic | S86L | 0.05668 | NM_001329844.2 | 494 | 466 | 28 |
| 786-LNmet | FOSL2 | 803 | hg19 | chr2 | 28631638 | 28631638 | In_Frame_Del | DEL | GAG | GAG | - | novel | Somatic | E125del | 0.03444 | NM_005253.4 | 812 | 784 | 28 |
| 786-LNmet | RTN4 | 1006 | hg19 | chr2 | 55253865 | 55253865 | Missense_Mutation | SNP | C | C | T | novel | Somatic | R457H | 0.020284 | NM_020532.5 | 492 | 482 | 10 |
| 786-LNmet | EFEMP1 | 126 | hg19 | chr2 | 56103844 | 56103844 | Missense_Mutation | SNP | G | G | A | novel | Somatic | A265V | 0.066929 | NM_001039348.3 | 254 | 237 | 17 |
| 786-LNmet | USP34 | 131 | hg19 | chr2 | 61510315 | 61510315 | Missense_Mutation | SNP | C | C | G | novel | Somatic | D1655H | 0.05802 | NM_014709.4 | 293 | 276 | 17 |
| 786-LNmet | PDE11A | 243 | hg19 | chr2 | 178592756 | 178592756 | Missense_Mutation | SNP | C | C | G | novel | Somatic | E645Q | 0.044983 | NM_016953.4 | 289 | 276 | 13 |
| 786-LNmet | TTN | 1955 | hg19 | chr2 | 179432156 | 179432156 | Missense_Mutation | SNP | C | C | T | novel | Somatic | E26235K | 0.041344 | NM_001267550.2 | 385 | 369 | 16 |
| 786-LNmet | TTN | 1955 | hg19 | chr2 | 179432170 | 179432170 | Missense_Mutation | SNP | C | C | T | novel | Somatic | G26230E | 0.038278 | NM_001267550.2 | 418 | 402 | 16 |
| 786-LNmet | TTN | 1955 | hg19 | chr2 | 179433944 | 179433944 | Missense_Mutation | SNP | C | C | T | novel | Somatic | E25639K | 0.035928 | NM_001267550.2 | 334 | 322 | 12 |
| 786-LNmet | TTN | 1955 | hg19 | chr2 | 179455905 | 179455905 | Missense_Mutation | DNP | CC | CC | TT | novel | Somatic | D20183N | 0.027624 | NM_001267550.2 | 358 | 348 | 10 |
| 786-LNmet | TTN | 1955 | hg19 | chr2 | 179456389 | 179456389 | Nonsense_Mutation | SNP | C | C | A | novel | Somatic | E20053Ter | 0.041096 | NM_001267550.2 | 365 | 350 | 15 |
| 786-LNmet | TTN | 1955 | hg19 | chr2 | 179457005 | 179457005 | Splice_Site | SNP | C | C | G | novel | Somatic |  | 0.040293 | NM_001267550.2 | 273 | 262 | 11 |
| 786-LNmet | TTN | 1955 | hg19 | chr2 | 179458511 | 179458511 | Missense_Mutation | SNP | C | C | A | novel | Somatic | D19506Y | 0.053872 | NM_001267550.2 | 297 | 281 | 16 |
| 786-LNmet | DNAH7 | 260 | hg19 | chr2 | 196729560 | 196729560 | Missense_Mutation | SNP | G | G | T | novel | Somatic | F2273L | 0.049881 | NM_018897.3 | 421 | 400 | 21 |
| 786-LNmet | KANSL1L | 274 | hg19 | chr2 | 210894528 | 210894528 | Missense_Mutation | SNP | C | C | T | novel | Somatic | R757Q | 0.07767 | NM_152519.4 | 412 | 380 | 32 |
| 786-LNmet | C2orf54 | 303 | hg19 | chr2 | 241835333 | 241835333 | Missense_Mutation | SNP | G | G | A | novel | Somatic | R28W | 0.105702 | NM_001085437.3 | 718 | 642 | 76 |
| 786-LNmet | ITPR1 | 77 | hg19 | chr3 | 4704840 | 4704840 | Missense_Mutation | SNP | C | C | A | novel | Somatic | P487T | 0.043887 | NM_001168272.1 | 635 | 607 | 28 |
| 786-LNmet | DNAH12 | 1023 | hg19 | chr3 | 57528534 | 57528534 | Missense_Mutation | SNP | G | G | T | novel | Somatic | P22T | 0.038674 | NM_001366028.2 | 361 | 347 | 14 |
| 786-LNmet | ADAMTS9 | 134 | hg19 | chr3 | 64601699 | 64601699 | Missense_Mutation | SNP | G | G | C | novel | Somatic | S987R | 0.040107 | NM_182920.2 | 373 | 358 | 15 |
| 786-LNmet | TRMT10C | 1357 | hg19 | chr3 | 101283906 | 101283906 | Missense_Mutation | SNP | C | C | A | novel | Somatic | T94N | 0.037778 | NM_017819.4 | 447 | 430 | 17 |
| 786-LNmet | RNF7 | 1664 | hg19 | chr3 | 141462372 | 141462372 | Missense_Mutation | SNP | C | C | A | novel | Somatic | A66D | 0.057663 | NM_014245.5 | 659 | 621 | 38 |
| 786-LNmet | TM4SF18 | 1722 | hg19 | chr3 | 149051163 | 149051163 | Missense_Mutation | SNP | A | A | G | novel | Somatic | S3P | 0.075472 | NM_138786.4 | 264 | 244 | 20 |
| 786-LNmet | ABCF3 | 1988 | hg19 | chr3 | 183908824 | 183908824 | Missense_Mutation | SNP | G | G | A | novel | Somatic | V477I | 0.179739 | NM_018358.2 | 611 | 501 | 110 |
| 786-LNmet | GABRA4 | 117 | hg19 | chr4 | 46979602 | 46979602 | Frame_Shift_Del | DEL | T | T | - | novel | Somatic | R107DfsTer2 | 0.052117 | NM_000809.4 | 307 | 291 | 16 |
| 786-LNmet | NDST4 | 183 | hg19 | chr4 | 115767053 | 115767053 | Missense_Mutation | SNP | C | C | T | novel | Somatic | A681T | 0.032432 | NM_022569.3 | 370 | 358 | 12 |
| 786-LNmet | EXOSC9 | 1521 | hg19 | chr4 | 122731167 | 122731167 | Missense_Mutation | SNP | G | G | A | novel | Somatic | M217I | 0.064838 | NM_005033.3 | 401 | 375 | 26 |
| 786-LNmet | GALNT7 | 239 | hg19 | chr4 | 174219256 | 174219256 | Intron | SNP | C | C | G | novel | Somatic |  | 0.050781 | NM_017423.3 | 256 | 243 | 13 |
| 786-LNmet | SLC12A7 | 593 | hg19 | chr5 | 1085509 | 1085509 | Missense_Mutation | SNP | C | C | T | novel | Somatic | R252H | 0.085627 | NM_006598.3 | 327 | 299 | 28 |
| 786-LNmet | EGFLAM | 878 | hg19 | chr5 | 38352350 | 38352350 | Silent | SNP | G | G | A | novel | Somatic | S154= | 0.026667 | NM_152403.4 | 600 | 584 | 16 |
| 786-LNmet | SHLD3 | Unknown | hg19 | chr5 | 64925871 | 64925871 | Missense_Mutation | SNP | C | C | G | novel | Somatic | Q153E | 0.045558 | NM_001365341.2 | 437 | 417 | 20 |
| 786-LNmet | CAST | 1318 | hg19 | chr5 | 96077047 | 96077047 | Missense_Mutation | SNP | G | G | C | novel | Somatic | K332N | 0.12931 | NM_001750.7 | 348 | 303 | 45 |
| 786-LNmet | RAD50 | 1591 | hg19 | chr5 | 131923294 | 131923294 | Missense_Mutation | SNP | T | T | G | novel | Somatic | M266R | 0.129799 | NM_005732.4 | 547 | 476 | 71 |
| 786-LNmet | PCDHA13 | 1655 | hg19 | chr5 | 140263242 | 140263242 | Missense_Mutation | SNP | C | C | A | novel | Somatic | F463L | 0.234093 | NM_018904.3 | 1267 | 969 | 298 |
| 786-LNmet | PCDHB6 | 1657 | hg19 | chr5 | 140531975 | 140531975 | Missense_Mutation | SNP | A | A | G | novel | Somatic | R713G | 0.072131 | NM_018939.3 | 1523 | 1413 | 110 |
| 786-LNmet | PCDHGA3 | 1658 | hg19 | chr5 | 140725137 | 140725137 | Missense_Mutation | SNP | G | G | A | novel | Somatic | V513I | 0.02719 | NM_018916.4 | 661 | 643 | 18 |
| 786-LNmet | PCDHGB2 | 1658 | hg19 | chr5 | 140741872 | 140741872 | Missense_Mutation | SNP | T | T | C | novel | Somatic | W724R | 0.113445 | NM_018923.2 | 714 | 633 | 81 |
| 786-LNmet | PCDHGB7 | 1659 | hg19 | chr5 | 140797796 | 140797796 | Missense_Mutation | SNP | G | G | C | novel | Somatic | E124Q | 0.024896 | NM_018927.4 | 482 | 470 | 12 |
| 786-LNmet | WWC1 | 3 | hg19 | chr5 | 167855047 | 167855047 | Missense_Mutation | SNP | C | C | T | novel | Somatic | T607M | 0.023211 | NM_015238.3 | 517 | 505 | 12 |
| 786-LNmet | CLIC5 | 935 | hg19 | chr6 | 46047778 | 46047778 | Missense_Mutation | SNP | C | C | G | novel | Somatic | E68Q | 0.106383 | NM_001370650.1 | 282 | 252 | 30 |
| 786-LNmet | PKHD1 | 122 | hg19 | chr6 | 51768466 | 51768466 | Missense_Mutation | SNP | C | C | T | novel | Somatic | E2309K | 0.162264 | NM_138694.4 | 265 | 222 | 43 |
| 786-LNmet | SOGA3 | 194 | hg19 | chr6 | 127834083 | 127834083 | Missense_Mutation | SNP | C | C | G | novel | Somatic | E480Q | 0.028916 | NM_001012279.3 | 415 | 403 | 12 |
| 786-LNmet | ENPP3 | 198 | hg19 | chr6 | 131973973 | 131973973 | Missense_Mutation | SNP | G | G | C | novel | Somatic | E136Q | 0.045833 | NM_005021.5 | 240 | 229 | 11 |
| 786-LNmet | VNN2 | 1600 | hg19 | chr6 | 133065485 | 133065485 | Missense_Mutation | SNP | A | A | T | novel | Somatic | F506Y | 0.041667 | NM_004665.5 | 240 | 230 | 10 |
| 786-LNmet | HECA | 25 | hg19 | chr6 | 139488129 | 139488129 | Missense_Mutation | SNP | C | C | T | novel | Somatic | A327V | 0.069405 | NM_016217.3 | 705 | 656 | 49 |
| 786-LNmet | DNAH11 | 93 | hg19 | chr7 | 21675499 | 21675499 | Missense_Mutation | SNP | A | A | T | novel | Somatic | Q1504L | 0.08658 | NM_001277115.2 | 231 | 211 | 20 |
| 786-LNmet | GARS | 102 | hg19 | chr7 | 30649246 | 30649246 | Missense_Mutation | SNP | T | T | C | novel | Somatic | Y261H | 0.028205 | NM_002047.4 | 390 | 379 | 11 |
| 786-LNmet | PEG10 | 1304 | hg19 | chr7 | 94293685 | 94293685 | Missense_Mutation | SNP | C | C | G | novel | Somatic | P349A | 0.072546 | NM_001172437.2 | 702 | 651 | 51 |
| 786-LNmet | ACTL6B | 1349 | hg19 | chr7 | 100252706 | 100252706 | Missense_Mutation | SNP | T | T | C | novel | Somatic | H102R | 0.111111 | NM_016188.5 | 540 | 480 | 60 |
| 786-LNmet | SRRT | 1351 | hg19 | chr7 | 100483976 | 100483976 | Missense_Mutation | SNP | C | C | G | novel | Somatic | L523V | 0.035811 | NM_015908.6 | 1479 | 1426 | 53 |
| 786-LNmet | POLR2J | 1364 | hg19 | chr7 | 102119287 | 102119287 | Missense_Mutation | SNP | G | G | C | novel | Somatic | F7L | 0.076296 | NM_006234.5 | 1349 | 1246 | 103 |
| 786-LNmet | KMT2E | 172 | hg19 | chr7 | 104746351 | 104746351 | Missense_Mutation | SNP | C | C | T | novel | Somatic | H833Y | 0.029221 | NM_182931.3 | 616 | 598 | 18 |
| 786-LNmet | FAM110B | 129 | hg19 | chr8 | 59059902 | 59059902 | Nonstop_Mutation | SNP | A | A | T | novel | Somatic | Ter371YextTer24 | 0.15331 | NM_147189.3 | 287 | 243 | 44 |
| 786-LNmet | PREX2 | 138 | hg19 | chr8 | 68968085 | 68968085 | Missense_Mutation | SNP | C | C | G | novel | Somatic | Q372E | 0.031707 | NM_024870.4 | 409 | 396 | 13 |
| 786-LNmet | RUNX1T1 | 1294 | hg19 | chr8 | 92988171 | 92988171 | Missense_Mutation | SNP | G | G | A | novel | Somatic | A496V | 0.089069 | NM_001198679.1 | 494 | 450 | 44 |
| 786-LNmet | RGS22 | 169 | hg19 | chr8 | 100990177 | 100990177 | Frame_Shift_Ins | INS | - | - | T | novel | Somatic | L1163IfsTer32 | 0.037475 | NM_015668.5 | 503 | 484 | 19 |
| 786-LNmet | YWHAZ | 1362 | hg19 | chr8 | 101960832 | 101960832 | Missense_Mutation | SNP | C | C | T | novel | Somatic | D96N | 0.021687 | NM_145690.3 | 830 | 812 | 18 |
| 786-LNmet | WDR97 | 1692 | hg19 | chr8 | 145164670 | 145164670 | Missense_Mutation | SNP | C | C | A | novel | Somatic | A478E | 0.153061 | NM_001316309.2 | 390 | 330 | 60 |
| 786-LNmet | NTRK2 | 156 | hg19 | chr9 | 87339207 | 87339207 | Missense_Mutation | SNP | G | G | C | novel | Somatic | Q263H | 0.060976 | NM_006180.4 | 328 | 308 | 20 |
| 786-LNmet | SHC3 | 160 | hg19 | chr9 | 91727466 | 91727466 | Splice_Region | SNP | C | C | G | novel | Somatic |  | 0.040161 | NM_016848.6 | 249 | 239 | 10 |
| 786-LNmet | NTNG2 | 1615 | hg19 | chr9 | 135042430 | 135042430 | Missense_Mutation | SNP | A | A | T | novel | Somatic | H71L | 0.031073 | NM_032536.4 | 354 | 343 | 11 |
| 786-LNmet | LCN1 | 1641 | hg19 | chr9 | 138415088 | 138415088 | Missense_Mutation | SNP | C | C | T | novel | Somatic | R78W | 0.031414 | NM_001252618.2 | 382 | 370 | 12 |
| 786-LNmet | NPDC1 | 1652 | hg19 | chr9 | 139935154 | 139935154 | Missense_Mutation | SNP | C | C | T | novel | Somatic | A198T | 0.033479 | NM_015392.4 | 686 | 663 | 23 |
| 786-LNmet | CHAT | 121 | hg19 | chr10 | 50828626 | 50828626 | Missense_Mutation | SNP | G | G | A | novel | Somatic | R222Q | 0.053131 | NM_020549.4 | 527 | 499 | 28 |
| 786-LNmet | CDH23 | 1146 | hg19 | chr10 | 73548732 | 73548732 | Missense_Mutation | SNP | G | G | T | novel | Somatic | E1952D | 0.025581 | NM_022124.6 | 429 | 418 | 11 |
| 786-LNmet | ECD | 1156 | hg19 | chr10 | 74896497 | 74896497 | Missense_Mutation | SNP | A | A | C | novel | Somatic | C557G | 0.099462 | NM_007265.3 | 372 | 335 | 37 |
| 786-LNmet | TNKS2 | 162 | hg19 | chr10 | 93619393 | 93619393 | Missense_Mutation | SNP | A | A | G | novel | Somatic | Y1090C | 0.083102 | NM_025235.4 | 361 | 331 | 30 |
| 786-LNmet | LCOR | 167 | hg19 | chr10 | 98711858 | 98711858 | Splice_Site | SNP | A | A | T | novel | Somatic |  | 0.076246 | NM_001346516.2 | 341 | 315 | 26 |
| 786-LNmet | TMEM80 | 590 | hg19 | chr11 | 704540 | 704540 | Missense_Mutation | SNP | A | A | C | novel | Somatic | E249A | 0.109772 | NM_001276274.1 | 747 | 665 | 82 |
| 786-LNmet | NELL1 | 11 | hg19 | chr11 | 20968923 | 20968923 | Missense_Mutation | SNP | G | G | C | novel | Somatic | L371F | 0.128319 | NM_006157.5 | 452 | 394 | 58 |
| 786-LNmet | NELL1 | 11 | hg19 | chr11 | 21596493 | 21596493 | Intron | SNP | T | T | G | novel | Somatic |  | 0.075802 | NM_006157.5 | 343 | 317 | 26 |
| 786-LNmet | LDLRAD3 | 107 | hg19 | chr11 | 36103250 | 36103250 | Missense_Mutation | SNP | G | G | C | novel | Somatic | G81R | 0.066079 | NM_174902.4 | 681 | 636 | 45 |
| 786-LNmet | DDB2 | 945 | hg19 | chr11 | 47259481 | 47259481 | Missense_Mutation | SNP | G | G | A | novel | Somatic | D373N | 0.081917 | NM_000107.2 | 644 | 591 | 53 |
| 786-LNmet | OR5T3 | 1012 | hg19 | chr11 | 56020588 | 56020588 | Missense_Mutation | SNP | T | T | A | novel | Somatic | S305T | 0.138047 | NM_001004747.1 | 296 | 255 | 41 |
| 786-LNmet | MRPL49 | 1080 | hg19 | chr11 | 64893043 | 64893043 | Missense_Mutation | SNP | C | C | T | novel | Somatic | R105W | 0.030984 | NM_004927.4 | 1646 | 1595 | 51 |
| 786-LNmet | RELT | 1142 | hg19 | chr11 | 73106130 | 73106130 | Splice_Site | SNP | A | A | G | novel | Somatic |  | 0.048843 | NM_032871.3 | 778 | 740 | 38 |
| 786-LNmet | FAT3 | 2 | hg19 | chr11 | 92532370 | 92532370 | Missense_Mutation | SNP | G | G | A | novel | Somatic | R2064H | 0.105145 | NM_001367949.1 | 447 | 400 | 47 |
| 786-LNmet | C11orf54 | 1298 | hg19 | chr11 | 93486914 | 93486914 | Frame_Shift_Del | DEL | A | A | - | novel | Somatic | V77FfsTer4 | 0.020794 | NM_001351985.2 | 529 | 518 | 11 |
| 786-LNmet | ITPR2 | 98 | hg19 | chr12 | 26784949 | 26784949 | Missense_Mutation | SNP | C | C | G | novel | Somatic | M928I | 0.052863 | NM_002223.4 | 681 | 645 | 36 |
| 786-LNmet | ITPR2 | 98 | hg19 | chr12 | 26784992 | 26784992 | Missense_Mutation | SNP | C | C | T | novel | Somatic | G914E | 0.052885 | NM_002223.4 | 416 | 394 | 22 |
| 786-LNmet | DGKA | 1014 | hg19 | chr12 | 56332722 | 56332722 | Silent | SNP | G | G | A | novel | Somatic | Q141= | 0.122936 | NM_001345.5 | 545 | 478 | 67 |
| 786-LNmet | AVIL | 128 | hg19 | chr12 | 58195985 | 58195985 | Missense_Mutation | SNP | G | G | C | novel | Somatic | I703M | 0.109474 | NM_006576.3 | 950 | 846 | 104 |
| 786-LNmet | MYO16 | 177 | hg19 | chr13 | 109793062 | 109793062 | Missense_Mutation | SNP | C | C | T | novel | Somatic | P1501L | 0.177898 | NM_001198950.3 | 371 | 305 | 66 |
| 786-LNmet | NOVA1 | 98 | hg19 | chr14 | 26917586 | 26917586 | Missense_Mutation | SNP | T | T | C | novel | Somatic | E368G | 0.109137 | NM_002515.3 | 787 | 701 | 86 |
| 786-LNmet | SIPA1L1 | 141 | hg19 | chr14 | 72138294 | 72138294 | Missense_Mutation | SNP | T | T | A | novel | Somatic | I905K | 0.026667 | NM_015556.4 | 375 | 365 | 10 |
| 786-LNmet | BCL11B | 1345 | hg19 | chr14 | 99641432 | 99641432 | In_Frame_Del | DEL | CCCCCGCGCCCGGGA | CCCCCGCGCCCGGGA | - | novel | Somatic | V576_G580del | 0.079304 | NM_138576.4 | 517 | 476 | 41 |
| 786-LNmet | CTXN2 | 954 | hg19 | chr15 | 48493566 | 48493566 | Missense_Mutation | SNP | G | G | C | novel | Somatic | L23F | 0.103139 | NM_001145668.2 | 223 | 200 | 23 |
| 786-LNmet | IGDCC4 | 1086 | hg19 | chr15 | 65681166 | 65681166 | Missense_Mutation | SNP | C | C | A | novel | Somatic | W896L | 0.029316 | NM_020962.3 | 613 | 595 | 18 |
| 786-LNmet | MEIOB | 599 | hg19 | chr16 | 1910438 | 1910438 | Missense_Mutation | SNP | C | C | G | novel | Somatic | E100Q | 0.071834 | NM_001163560.3 | 528 | 490 | 38 |
| 786-LNmet | TMC5 | 733 | hg19 | chr16 | 19451830 | 19451830 | Missense_Mutation | SNP | G | G | C | novel | Somatic | G157A | 0.034694 | NM_001105248.1 | 488 | 471 | 17 |
| 786-LNmet | DNAH3 | 11 | hg19 | chr16 | 20975329 | 20975329 | Missense_Mutation | SNP | C | C | T | novel | Somatic | G3293S | 0.045455 | NM_017539.2 | 440 | 420 | 20 |
| 786-LNmet | SRCAP | 819 | hg19 | chr16 | 30722379 | 30722379 | Missense_Mutation | SNP | G | G | T | novel | Somatic | A430S | 0.033679 | NM_006662.3 | 385 | 372 | 13 |
| 786-LNmet | ZFHX3 | 142 | hg19 | chr16 | 72923654 | 72923654 | Missense_Mutation | SNP | G | G | A | novel | Somatic | R1142C | 0.06917 | NM_006885.4 | 506 | 471 | 35 |
| 786-LNmet | C16orf95 | 1251 | hg19 | chr16 | 87350781 | 87350781 | Missense_Mutation | SNP | G | G | C | novel | Somatic | A23G | 0.053115 | NM_001195124.2 | 979 | 927 | 52 |
| 786-LNmet | C16orf95 | 1251 | hg19 | chr16 | 87350821 | 87350821 | Missense_Mutation | SNP | G | G | T | novel | Somatic | P10T | 0.051047 | NM_001195124.2 | 759 | 720 | 39 |
| 786-LNmet | ZFP3 | 623 | hg19 | chr17 | 4994828 | 4994828 | Missense_Mutation | SNP | C | C | A | novel | Somatic | P10H | 0.030641 | NM_153018.3 | 358 | 347 | 11 |
| 786-LNmet | WSCD1 | 630 | hg19 | chr17 | 6023788 | 6023788 | Missense_Mutation | SNP | T | T | C | novel | Somatic | V512A | 0.054201 | NM_015253.1 | 368 | 348 | 20 |
| 786-LNmet | TP53 | 642 | hg19 | chr17 | 7576872 | 7576872 | Missense_Mutation | SNP | C | C | G | novel | Somatic | G325A | 0.041528 | NM_000546.5 | 602 | 577 | 25 |
| 786-LNmet | TP53 | 642 | hg19 | chr17 | 7577094 | 7577094 | Missense_Mutation | SNP | G | G | A | novel | Somatic | R282W | 0.172802 | NM_000546.5 | 1330 | 1100 | 230 |
| 786-LNmet | DNAH2 | 643 | hg19 | chr17 | 7719990 | 7719990 | Missense_Mutation | SNP | G | G | C | novel | Somatic | E3277D | 0.053282 | NM_020877.4 | 1294 | 1225 | 69 |
| 786-LNmet | CCDC42 | 650 | hg19 | chr17 | 8644964 | 8644964 | Missense_Mutation | SNP | G | G | A | novel | Somatic | A107V | 0.083064 | NM_144681.3 | 926 | 849 | 77 |
| 786-LNmet | ZNF287 | 710 | hg19 | chr17 | 16469877 | 16469877 | Missense_Mutation | SNP | C | C | T | novel | Somatic | A155T | 0.080786 | NM_001346167.1 | 458 | 421 | 37 |
| 786-LNmet | FLII | 723 | hg19 | chr17 | 18149571 | 18149571 | Intron | SNP | G | G | A | novel | Somatic |  | 0.145068 | NM_002018.4 | 517 | 442 | 75 |
| 786-LNmet | GIT1 | 797 | hg19 | chr17 | 27901911 | 27901911 | Missense_Mutation | SNP | G | G | A | novel | Somatic | R699W | 0.023891 | NM_014030.4 | 879 | 858 | 21 |
| 786-LNmet | TMEM132E | 836 | hg19 | chr17 | 32959750 | 32959750 | Missense_Mutation | SNP | G | G | T | novel | Somatic | V504L | 0.080247 | NM_001304438.2 | 486 | 447 | 39 |
| 786-LNmet | SRCIN1 | 13 | hg19 | chr17 | 36700087 | 36700087 | Nonsense_Mutation | SNP | C | C | A | novel | Somatic | E1130Ter | 0.054422 | NM_025248.3 | 440 | 416 | 24 |
| 786-LNmet | SLC4A1 | 113 | hg19 | chr17 | 42328788 | 42328788 | Missense_Mutation | SNP | C | C | A | novel | Somatic | R827L | 0.099291 | NM_000342.4 | 564 | 508 | 56 |
| 786-LNmet | DDX5 | 1061 | hg19 | chr17 | 62496673 | 62496673 | Missense_Mutation | SNP | C | C | G | novel | Somatic | G479R | 0.029781 | NM_004396.5 | 637 | 618 | 19 |
| 786-LNmet | ABCA6 | 0 | hg19 | chr17 | 67132294 | 67132294 | Missense_Mutation | SNP | G | G | C | novel | Somatic | F133L | 0.199475 | NM_080284.3 | 381 | 305 | 76 |
| 786-LNmet | FN3KRP | 1200 | hg19 | chr17 | 80680740 | 80680740 | Missense_Mutation | SNP | C | C | T | novel | Somatic | T149M | 0.06274 | NM_024619.4 | 780 | 731 | 49 |
| 786-LNmet | ANKRD12 | 81 | hg19 | chr18 | 9258237 | 9258237 | Missense_Mutation | SNP | G | G | A | novel | Somatic | G1658R | 0.03413 | NM_015208.5 | 293 | 283 | 10 |
| 786-LNmet | ANKRD12 | 81 | hg19 | chr18 | 9258794 | 9258794 | Frame_Shift_Ins | INS | - | - | AA | novel | Somatic | I1846KfsTer2 | 0.029412 | NM_015208.5 | 407 | 395 | 12 |
| 786-LNmet | MEP1B | 812 | hg19 | chr18 | 29787254 | 29787254 | Nonsense_Mutation | SNP | C | C | G | novel | Somatic | S196Ter | 0.081967 | NM_005925.3 | 183 | 168 | 15 |
| 786-LNmet | ZNF532 | 15 | hg19 | chr18 | 56587042 | 56587042 | Missense_Mutation | SNP | C | C | T | novel | Somatic | P508L | 0.046605 | NM_001353526.2 | 751 | 716 | 35 |
| 786-LNmet | PALM | 590 | hg19 | chr19 | 746417 | 746417 | Missense_Mutation | SNP | C | C | A | novel | Somatic | T256N | 0.069343 | NM_002579.3 | 548 | 510 | 38 |
| 786-LNmet | ZNF121 | 658 | hg19 | chr19 | 9676921 | 9676921 | Frame_Shift_Ins | INS | - | - | A | novel | Somatic | G290WfsTer12 | 0.132075 | NM_001308269.2 | 314 | 272 | 42 |
| 786-LNmet | SYDE1 | 701 | hg19 | chr19 | 15224456 | 15224456 | Missense_Mutation | SNP | C | C | A | novel | Somatic | D630E | 0.039116 | NM_033025.6 | 586 | 563 | 23 |
| 786-LNmet | UNC13A | 720 | hg19 | chr19 | 17778978 | 17778978 | Missense_Mutation | SNP | C | C | T | novel | Somatic | R139H | 0.036545 | NM_001080421.2 | 301 | 290 | 11 |
| 786-LNmet | CCNE1 | 816 | hg19 | chr19 | 30312922 | 30312922 | Missense_Mutation | SNP | G | G | C | novel | Somatic | S242T | 0.096774 | NM_001238.4 | 248 | 224 | 24 |
| 786-LNmet | ZNF567 | 868 | hg19 | chr19 | 37211080 | 37211080 | Missense_Mutation | SNP | C | C | T | novel | Somatic | A485V | 0.044379 | NM_001322914.1 | 337 | 322 | 15 |
| 786-LNmet | AXL | 903 | hg19 | chr19 | 41727944 | 41727944 | Missense_Mutation | SNP | G | G | A | novel | Somatic | R190H | 0.120205 | NM_021913.5 | 781 | 687 | 94 |
| 786-LNmet | SLC17A7 | 120 | hg19 | chr19 | 49935820 | 49935820 | Missense_Mutation | SNP | C | C | T | novel | Somatic | R369H | 0.040268 | NM_020309.4 | 447 | 429 | 18 |
| 786-LNmet | BCL2L12 | 967 | hg19 | chr19 | 50169198 | 50169198 | In_Frame_Del | DEL | CGCTGGGCCGGTTATCGACCCGGCCCAGTG | CGCTGGGCCGGTTATCGACCCGGCCCAGTG | - | novel | Somatic | W41_R50del | 0.028678 | NM_138639.1 | 801 | 778 | 23 |
| 786-LNmet | PNKP | 969 | hg19 | chr19 | 50367312 | 50367312 | Missense_Mutation | SNP | T | T | A | novel | Somatic | N218I | 0.029312 | NM_007254.4 | 887 | 861 | 26 |
| 786-LNmet | ZNF71 | 1020 | hg19 | chr19 | 57134055 | 57134055 | Missense_Mutation | SNP | G | G | A | novel | Somatic | R527Q | 0.051237 | NM_001370215.1 | 566 | 537 | 29 |
| 786-LNmet | PSMF1 | 593 | hg19 | chr20 | 1106228 | 1106228 | Missense_Mutation | SNP | G | G | A | novel | Somatic | D73N | 0.043548 | NM_178578.3 | 619 | 592 | 27 |
| 786-LNmet | GINS1 | 97 | hg19 | chr20 | 25422396 | 25422396 | Frame_Shift_Ins | INS | - | - | A | novel | Somatic | N172KfsTer2 | 0.026273 | NM_021067.5 | 609 | 593 | 16 |
| 786-LNmet | MYH7B | 1 | hg19 | chr20 | 33588157 | 33588157 | Missense_Mutation | SNP | C | C | T | novel | Somatic | R1657C | 0.08322 | NM_020884.5 | 733 | 672 | 61 |
| 786-LNmet | PTGIS | 952 | hg19 | chr20 | 48140626 | 48140626 | Missense_Mutation | SNP | C | C | G | novel | Somatic | R275P | 0.043812 | NM_000961.4 | 911 | 871 | 40 |
| 786-LNmet | AP1B1 | 101 | hg19 | chr22 | 29727810 | 29727810 | Missense_Mutation | SNP | G | G | A | novel | Somatic | S802L | 0.053623 | NM_001127.3 | 690 | 653 | 37 |
| 786-LNmet | APOBEC3C | 885 | hg19 | chr22 | 39413943 | 39413943 | Missense_Mutation | SNP | T | T | C | novel | Somatic | L116P | 0.038359 | NM_014508.3 | 2241 | 2155 | 86 |
| 786-LNmet | JADE3 | 117 | hg19 | chrX | 46893170 | 46893170 | Missense_Mutation | SNP | T | T | C | novel | Somatic | C279R | 0.090909 | NM_014735.5 | 220 | 200 | 20 |
| 848-PTsup | PADI1 | 89 | hg19 | chr1 | 17548884 | 17548884 | Silent | SNP | C | C | T | novel | Somatic | G64= | 0.241935 | NM_013358.3 | 620 | 470 | 150 |
| 848-PTsup | ARHGEF10L | 90 | hg19 | chr1 | 17953892 | 17953892 | Missense_Mutation | SNP | T | T | C | novel | Somatic | L493P | 0.051553 | NM_018125.4 | 1511 | 1433 | 78 |
| 848-PTsup | MTF2 | 162 | hg19 | chr1 | 93602520 | 93602520 | Missense_Mutation | SNP | G | G | A | novel | Somatic | R573H | 0.191223 | NM_007358.4 | 638 | 516 | 122 |
| 848-PTsup | COL11A1 | 171 | hg19 | chr1 | 103463910 | 103463910 | Missense_Mutation | SNP | C | C | T | novel | Somatic | G730R | 0.186047 | NM_080629.2 | 686 | 558 | 128 |
| 848-PTsup | KCND3 | 180 | hg19 | chr1 | 112322918 | 112322918 | Missense_Mutation | SNP | G | G | T | novel | Somatic | H464N | 0.198142 | NM_004980.4 | 323 | 259 | 64 |
| 848-PTsup | SPAG17 | 186 | hg19 | chr1 | 118527339 | 118527339 | Missense_Mutation | SNP | A | A | T | novel | Somatic | F1912L | 0.230882 | NM_206996.4 | 678 | 521 | 157 |
| 848-PTsup | OR6Y1 | 1794 | hg19 | chr1 | 158517290 | 158517290 | Missense_Mutation | SNP | C | C | T | novel | Somatic | M202I | 0.30911 | NM_001005189.1 | 977 | 675 | 302 |
| 848-PTsup | LAX1 | 2139 | hg19 | chr1 | 203743576 | 203743576 | Missense_Mutation | SNP | G | G | A | novel | Somatic | E322K | 0.290718 | NM_017773.4 | 1140 | 808 | 332 |
| 848-PTsup | SOX13 | 267 | hg19 | chr1 | 204093869 | 204093869 | Missense_Mutation | SNP | G | G | T | novel | Somatic | K492N | 0.3269 | NM_005686.3 | 1657 | 1115 | 542 |
| 848-PTsup | NFASC | 2148 | hg19 | chr1 | 204981927 | 204981927 | Nonsense_Mutation | SNP | C | C | T | novel | Somatic | R1142Ter | 0.285276 | NM_001005388.2 | 652 | 466 | 186 |
| 848-PTsup | CR2 | 2169 | hg19 | chr1 | 207649749 | 207649749 | Missense_Mutation | SNP | G | G | T | novel | Somatic | A963S | 0.260032 | NM_001006658.2 | 623 | 461 | 162 |
| 848-PTsup | GUK1 | 2327 | hg19 | chr1 | 228328021 | 228328021 | Frame_Shift_Del | DEL | GGCCGGGCTGGCTGCGGCCGCCCTG | GGCCGGGCTGGCTGCGGCCGCCCTG | - | novel | Somatic | L9PfsTer14 | 0.080505 | NM_001242840.2 | 1263 | 1161 | 102 |
| 848-PTsup | PCNX2 | 2365 | hg19 | chr1 | 233161036 | 233161036 | Missense_Mutation | SNP | G | G | T | novel | Somatic | N1487K | 0.327572 | NM_014801.4 | 1215 | 817 | 398 |
| 848-PTsup | ADCY3 | 776 | hg19 | chr2 | 25141376 | 25141376 | Missense_Mutation | SNP | G | G | A | novel | Somatic | R161C | 0.161241 | NM_001320613.2 | 1318 | 1105 | 213 |
| 848-PTsup | GAREM2 | 786 | hg19 | chr2 | 26407588 | 26407588 | Missense_Mutation | SNP | C | C | G | novel | Somatic | L291V | 0.366931 | NM_001168241.2 | 882 | 558 | 324 |
| 848-PTsup | KCNG3 | 910 | hg19 | chr2 | 42720473 | 42720473 | Missense_Mutation | SNP | C | C | T | novel | Somatic | E57K | 0.202685 | NM_133329.6 | 745 | 594 | 151 |
| 848-PTsup | AFF3 | 2 | hg19 | chr2 | 100170808 | 100170808 | Missense_Mutation | SNP | C | C | A | novel | Somatic | W1200L | 0.119737 | NM_001025108.2 | 1518 | 1336 | 182 |
| 848-PTsup | ST6GAL2 | 175 | hg19 | chr2 | 107460187 | 107460187 | Missense_Mutation | SNP | C | C | T | novel | Somatic | A83T | 0.449785 | NM_001322362.2 | 1163 | 639 | 524 |
| 848-PTsup | CNTNAP5 | 192 | hg19 | chr2 | 125521709 | 125521709 | Nonsense_Mutation | SNP | C | C | T | novel | Somatic | R840Ter | 0.176842 | NM_001367498.1 | 475 | 391 | 84 |
| 848-PTsup | AMER3 | 1588 | hg19 | chr2 | 131519833 | 131519833 | Missense_Mutation | SNP | A | A | G | novel | Somatic | D63G | 0.205882 | NM_152698.3 | 575 | 456 | 119 |
| 848-PTsup | LRP1B | 3 | hg19 | chr2 | 141625277 | 141625277 | Nonsense_Mutation | SNP | C | C | T | novel | Somatic | W1487Ter | 0.22449 | NM_018557.3 | 636 | 493 | 143 |
| 848-PTsup | NEB | 218 | hg19 | chr2 | 152394755 | 152394755 | Missense_Mutation | SNP | G | G | A | novel | Somatic | R6966C | 0.249147 | NM_001271208.2 | 293 | 220 | 73 |
| 848-PTsup | TTN | 1955 | hg19 | chr2 | 179489290 | 179489290 | Missense_Mutation | SNP | A | A | G | novel | Somatic | V14906A | 0.442777 | NM_001267550.2 | 532 | 296 | 236 |
| 848-PTsup | TTN | 1955 | hg19 | chr2 | 179580313 | 179580313 | Missense_Mutation | SNP | G | G | T | novel | Somatic | H8610N | 0.425439 | NM_001267550.2 | 684 | 393 | 291 |
| 848-PTsup | ERBB4 | 34 | hg19 | chr2 | 212289014 | 212289014 | Nonsense_Mutation | SNP | C | C | T | novel | Somatic | W911Ter | 0.207627 | NM_005235.3 | 472 | 374 | 98 |
| 848-PTsup | PLCD4 | 2259 | hg19 | chr2 | 219500624 | 219500624 | Missense_Mutation | SNP | C | C | T | novel | Somatic | R668W | 0.182779 | NM_032726.4 | 662 | 541 | 121 |
| 848-PTsup | CUL3 | 2304 | hg19 | chr2 | 225339071 | 225339071 | Missense_Mutation | SNP | C | C | A | novel | Somatic | R739L | 0.176015 | NM_001257198.2 | 513 | 422 | 91 |
| 848-PTsup | KCNH8 | 91 | hg19 | chr3 | 19554522 | 19554522 | Missense_Mutation | SNP | G | G | A | novel | Somatic | E714K | 0.225554 | NM_144633.3 | 767 | 594 | 173 |
| 848-PTsup | CCR8 | 885 | hg19 | chr3 | 39374178 | 39374178 | Missense_Mutation | SNP | G | G | A | novel | Somatic | S119N | 0.17047 | NM_005201.4 | 744 | 617 | 127 |
| 848-PTsup | CSPG5 | 948 | hg19 | chr3 | 47618698 | 47618698 | Missense_Mutation | SNP | T | T | G | novel | Somatic | D273A | 0.271717 | NM_006574.4 | 987 | 718 | 269 |
| 848-PTsup | KLHDC8B | 960 | hg19 | chr3 | 49212285 | 49212285 | Missense_Mutation | SNP | G | G | A | novel | Somatic | A218T | 0.22898 | NM_173546.3 | 1117 | 861 | 256 |
| 848-PTsup | EPHA3 | 158 | hg19 | chr3 | 89521723 | 89521723 | Missense_Mutation | SNP | G | G | A | novel | Somatic | G934S | 0.488889 | NM_005233.6 | 717 | 365 | 352 |
| 848-PTsup | TRIM42 | 1656 | hg19 | chr3 | 140401662 | 140401662 | Missense_Mutation | SNP | G | G | A | novel | Somatic | G234R | 0.106017 | NM_152616.5 | 1394 | 1246 | 148 |
| 848-PTsup | GK5 | 1667 | hg19 | chr3 | 141934501 | 141934501 | Missense_Mutation | SNP | C | C | T | novel | Somatic | E61K | 0.107886 | NM_001039547.3 | 1584 | 1413 | 171 |
| 848-PTsup | ANKUB1 | 1725 | hg19 | chr3 | 149485876 | 149485876 | Missense_Mutation | SNP | C | C | A | novel | Somatic | Q191H | 0.10883 | NM_001144960.3 | 487 | 434 | 53 |
| 848-PTsup | KCNAB1 | 27 | hg19 | chr3 | 156232230 | 156232230 | Missense_Mutation | SNP | G | G | A | novel | Somatic | E246K | 0.115686 | NM_172160.2 | 510 | 451 | 59 |
| 848-PTsup | B3GNT5 | 247 | hg19 | chr3 | 182988544 | 182988544 | Missense_Mutation | SNP | A | A | G | novel | Somatic | M320V | 0.070894 | NM_032047.5 | 2002 | 1860 | 142 |
| 848-PTsup | VWA5B2 | 1988 | hg19 | chr3 | 183954152 | 183954152 | Missense_Mutation | SNP | G | G | A | novel | Somatic | E412K | 0.05417 | NM_138345.3 | 1403 | 1327 | 76 |
| 848-PTsup | IL1RAP | 254 | hg19 | chr3 | 190321919 | 190321919 | Missense_Mutation | SNP | C | C | A | novel | Somatic | R23S | 0.056628 | NM_001167931.2 | 1554 | 1466 | 88 |
| 848-PTsup | LMLN | 2093 | hg19 | chr3 | 197729927 | 197729927 | Frame_Shift_Del | DEL | G | G | - | novel | Somatic | G426AfsTer27 | 0.064014 | NM_001136049.2 | 1155 | 1081 | 74 |
| 848-PTsup | AFAP1 | 80 | hg19 | chr4 | 7795457 | 7795457 | Missense_Mutation | SNP | C | C | A | novel | Somatic | D455Y | 0.268105 | NM_001134647.1 | 649 | 475 | 174 |
| 848-PTsup | LIMCH1 | 112 | hg19 | chr4 | 41634839 | 41634839 | Missense_Mutation | SNP | G | G | C | novel | Somatic | E559Q | 0.259594 | NM_001330672.2 | 443 | 328 | 115 |
| 848-PTsup | BANK1 | 21 | hg19 | chr4 | 102776269 | 102776269 | Nonsense_Mutation | SNP | G | G | T | novel | Somatic | E183Ter | 0.030303 | NM_017935.5 | 725 | 703 | 22 |
| 848-PTsup | GRIA2 | 223 | hg19 | chr4 | 158283975 | 158283975 | Missense_Mutation | SNP | A | A | C | novel | Somatic | S811R | 0.153276 | NM_001083619.1 | 807 | 683 | 124 |
| 848-PTsup | DDX60 | 234 | hg19 | chr4 | 169196671 | 169196671 | Intron | SNP | G | G | C | novel | Somatic |  | 0.303393 | NM_017631.6 | 500 | 348 | 152 |
| 848-PTsup | TRIML2 | 2027 | hg19 | chr4 | 189012729 | 189012729 | Missense_Mutation | SNP | C | C | T | novel | Somatic | G396D | 0.314145 | NM_001303419.1 | 606 | 415 | 191 |
| 848-PTsup | ADAMTS16 | 9 | hg19 | chr5 | 5262779 | 5262779 | Missense_Mutation | SNP | C | C | G | novel | Somatic | T891S | 0.194712 | NM_139056.4 | 416 | 335 | 81 |
| 848-PTsup | PCDHAC2 | 1655 | hg19 | chr5 | 140347516 | 140347516 | Missense_Mutation | SNP | G | G | T | novel | Somatic | V389F | 0.532526 | NM_018899.6 | 661 | 309 | 352 |
| 848-PTsup | PCDHB11 | 1657 | hg19 | chr5 | 140579789 | 140579789 | Frame_Shift_Ins | INS | - | - | T | novel | Somatic | G149WfsTer33 | 0.492829 | NM_018931.3 | 764 | 386 | 378 |
| 848-PTsup | NMUR2 | 217 | hg19 | chr5 | 151784001 | 151784001 | Missense_Mutation | SNP | T | T | C | novel | Somatic | Y225C | 0.163686 | NM_020167.4 | 725 | 606 | 119 |
| 848-PTsup | DRD1 | 1919 | hg19 | chr5 | 174869394 | 174869394 | Missense_Mutation | SNP | G | G | A | novel | Somatic | H237Y | 0.151206 | NM_000794.5 | 1077 | 914 | 163 |
| 848-PTsup | SFXN1 | 1919 | hg19 | chr5 | 174919169 | 174919169 | Missense_Mutation | SNP | C | C | A | novel | Somatic | F21L | 0.149057 | NM_022754.7 | 1590 | 1353 | 237 |
| 848-PTsup | CPLX2 | 1922 | hg19 | chr5 | 175306051 | 175306051 | Missense_Mutation | SNP | G | G | A | novel | Somatic | E58K | 0.1622 | NM_001008220.2 | 1417 | 1187 | 230 |
| 848-PTsup | HUS1B | 590 | hg19 | chr6 | 656542 | 656542 | Missense_Mutation | SNP | G | G | A | novel | Somatic | R135W | 0.163583 | NM_148959.3 | 1027 | 859 | 168 |
| 848-PTsup | PGBD1 | 800 | hg19 | chr6 | 28269624 | 28269624 | Missense_Mutation | SNP | C | C | A | novel | Somatic | H665N | 0.156313 | NM_001184743.1 | 498 | 420 | 78 |
| 848-PTsup | C6orf89 | 866 | hg19 | chr6 | 36870003 | 36870003 | Missense_Mutation | SNP | G | G | A | novel | Somatic | A73T | 0.288952 | NM_152734.4 | 704 | 500 | 204 |
| 848-PTsup | DEFB113 | 965 | hg19 | chr6 | 49936573 | 49936573 | Missense_Mutation | SNP | C | C | G | novel | Somatic | Q22H | 0.173252 | NM_001037729.1 | 658 | 544 | 114 |
| 848-PTsup | MTRF1L | 1754 | hg19 | chr6 | 153319684 | 153319684 | Splice_Site | SNP | C | C | A | novel | Somatic |  | 0.186888 | NM_019041.7 | 1021 | 830 | 191 |
| 848-PTsup | AGPAT4 | 227 | hg19 | chr6 | 161560504 | 161560504 | Missense_Mutation | SNP | C | C | A | novel | Somatic | R331M | 0.178601 | NM_020133.3 | 1215 | 998 | 217 |
| 848-PTsup | FBXL18 | 627 | hg19 | chr7 | 5541643 | 5541643 | Frame_Shift_Del | DEL | CTCA | CTCA | - | novel | Somatic | V85GfsTer4 | 0.020649 | NM_024963.6 | 678 | 664 | 14 |
| 848-PTsup | TWISTNB | 735 | hg19 | chr7 | 19739753 | 19739753 | Missense_Mutation | SNP | G | G | A | novel | Somatic | R183C | 0.054645 | NM_001002926.2 | 732 | 692 | 40 |
| 848-PTsup | MAGI2 | 18 | hg19 | chr7 | 79082546 | 79082546 | Missense_Mutation | SNP | C | C | G | novel | Somatic | E31Q | 0.221311 | NM_012301.4 | 730 | 568 | 162 |
| 848-PTsup | COL1A2 | 1302 | hg19 | chr7 | 94053715 | 94053715 | Missense_Mutation | SNP | C | C | T | novel | Somatic | S878L | 0.182618 | NM_000089.4 | 908 | 742 | 166 |
| 848-PTsup | SLC25A13 | 164 | hg19 | chr7 | 95818605 | 95818605 | Splice_Site | SNP | C | C | T | novel | Somatic |  | 0.228457 | NM_014251.3 | 499 | 385 | 114 |
| 848-PTsup | ATG9B | 1734 | hg19 | chr7 | 150721484 | 150721484 | Frame_Shift_Del | DEL | C | C | - | novel | Somatic | R10EfsTer96 | 0.02449 | NM_001317056.1 | 734 | 716 | 18 |
| 848-PTsup | CSMD1 | 9 | hg19 | chr8 | 3081326 | 3081326 | Missense_Mutation | SNP | G | G | A | novel | Somatic | P1470L | 0.519203 | NM_033225.6 | 703 | 338 | 365 |
| 848-PTsup | KAT6A | 112 | hg19 | chr8 | 41791261 | 41791261 | Frame_Shift_Ins | INS | - | - | CTGG | novel | Somatic | S1493PfsTer15 | 0.148635 | NM_006766.5 | 975 | 828 | 147 |
| 848-PTsup | YTHDF3 | 134 | hg19 | chr8 | 64099908 | 64099908 | Missense_Mutation | SNP | C | C | T | novel | Somatic | R446C | 0.182505 | NM_152758.6 | 924 | 755 | 169 |
| 848-PTsup | CSMD3 | 22 | hg19 | chr8 | 113599378 | 113599378 | Missense_Mutation | SNP | T | T | C | novel | Somatic | I1268V | 0.172028 | NM_198123.2 | 715 | 592 | 123 |
| 848-PTsup | DEPTOR | 188 | hg19 | chr8 | 120940713 | 120940713 | Missense_Mutation | SNP | G | G | A | novel | Somatic | A66T | 0.172766 | NM_022783.4 | 1175 | 972 | 203 |
| 848-PTsup | ATAD2 | 191 | hg19 | chr8 | 124384893 | 124384893 | Frame_Shift_Del | DEL | T | T | - | novel | Somatic | E119KfsTer8 | 0.023356 | NM_014109.4 | 1156 | 1129 | 27 |
| 848-PTsup | PAPPA | 186 | hg19 | chr9 | 118997469 | 118997469 | Missense_Mutation | SNP | C | C | T | novel | Somatic | P762L | 0.204545 | NM_002581.5 | 660 | 525 | 135 |
| 848-PTsup | NELFB | 1654 | hg19 | chr9 | 140149972 | 140149972 | Missense_Mutation | SNP | G | G | A | novel | Somatic | G52E | 0.405941 | NM_015456.5 | 707 | 420 | 287 |
| 848-PTsup | CUBN | 89 | hg19 | chr10 | 17107611 | 17107611 | Missense_Mutation | SNP | G | G | A | novel | Somatic | S1012F | 0.263427 | NM_001081.4 | 782 | 576 | 206 |
| 848-PTsup | ZNF33B | 114 | hg19 | chr10 | 43127384 | 43127384 | Missense_Mutation | SNP | G | G | T | novel | Somatic | S81R | 0.154018 | NM_006955.3 | 448 | 379 | 69 |
| 848-PTsup | KCNMA1 | 1190 | hg19 | chr10 | 78844467 | 78844467 | Missense_Mutation | SNP | G | G | A | novel | Somatic | A484V | 0.18442 | NM_001161352.2 | 626 | 510 | 116 |
| 848-PTsup | OR4C15 | 1007 | hg19 | chr11 | 55322321 | 55322321 | Missense_Mutation | SNP | G | G | T | novel | Somatic | C126F | 0.227468 | NM_001001920.2 | 698 | 539 | 159 |
| 848-PTsup | VPS37C | 1049 | hg19 | chr11 | 60899539 | 60899539 | Missense_Mutation | SNP | C | C | T | novel | Somatic | G274E | 0.248528 | NM_017966.5 | 848 | 637 | 211 |
| 848-PTsup | ARHGAP20 | 178 | hg19 | chr11 | 110485337 | 110485337 | Missense_Mutation | SNP | G | G | A | novel | Somatic | P193L | 0.195833 | NM_020809.3 | 959 | 771 | 188 |
| 848-PTsup | CD163 | 643 | hg19 | chr12 | 7639511 | 7639511 | Nonsense_Mutation | SNP | C | C | A | novel | Somatic | E708Ter | 0.348774 | NM_004244.5 | 734 | 478 | 256 |
| 848-PTsup | KLRF1 | 661 | hg19 | chr12 | 9997090 | 9997090 | In_Frame_Ins | INS | - | - | GCA | novel | Somatic | S223dup | 0.142736 | NM_016523.3 | 1169 | 1001 | 168 |
| 848-PTsup | LOC255308 | Unknown | hg19 | chr12 | 10659547 | 10659547 | Missense_Mutation | SNP | G | G | A | novel | Somatic | R349Q | 0.339468 | NM_001357734.1 | 978 | 646 | 332 |
| 848-PTsup | ARID2 | 14 | hg19 | chr12 | 46245781 | 46245781 | Missense_Mutation | SNP | G | G | A | novel | Somatic | S1292N | 0.176403 | NM_152641.4 | 871 | 717 | 154 |
| 848-PTsup | SMARCC2 | 1016 | hg19 | chr12 | 56575296 | 56575296 | Missense_Mutation | SNP | G | G | C | novel | Somatic | P309R | 0.171159 | NM_001330288.2 | 741 | 614 | 127 |
| 848-PTsup | STAT2 | 1017 | hg19 | chr12 | 56737279 | 56737279 | Missense_Mutation | SNP | G | G | A | novel | Somatic | P817L | 0.021613 | NM_005419.4 | 1199 | 1173 | 26 |
| 848-PTsup | LYZ | 1117 | hg19 | chr12 | 69746053 | 69746053 | Missense_Mutation | SNP | C | C | T | novel | Somatic | R119C | 0.162643 | NM_000239.3 | 786 | 658 | 128 |
| 848-PTsup | NAV3 | 147 | hg19 | chr12 | 78400658 | 78400658 | Missense_Mutation | SNP | C | C | T | novel | Somatic | A447V | 0.168512 | NM_001024383.2 | 809 | 672 | 137 |
| 848-PTsup | SLC6A15 | 1235 | hg19 | chr12 | 85266555 | 85266555 | Missense_Mutation | SNP | C | C | T | novel | Somatic | M376I | 0.15858 | NM_182767.6 | 845 | 711 | 134 |
| 848-PTsup | CEP290 | 157 | hg19 | chr12 | 88512305 | 88512305 | Frame_Shift_Del | DEL | T | T | - | novel | Somatic | I556FfsTer17 | 0.024327 | NM_025114.4 | 1147 | 1119 | 28 |
| 848-PTsup | ANKS1B | 167 | hg19 | chr12 | 99837530 | 99837530 | Missense_Mutation | SNP | G | G | A | novel | Somatic | S499L | 0.145598 | NM_001352186.1 | 885 | 756 | 129 |
| 848-PTsup | FANCM | 116 | hg19 | chr14 | 45628301 | 45628301 | Missense_Mutation | SNP | G | G | A | novel | Somatic | E467K | 0.139752 | NM_020937.4 | 322 | 277 | 45 |
| 848-PTsup | C15orf39 | 2 | hg19 | chr15 | 75500089 | 75500089 | Nonsense_Mutation | SNP | C | C | A | novel | Somatic | S567Ter | 0.34672 | NM_015492.5 | 747 | 488 | 259 |
| 848-PTsup | ARPIN | 1275 | hg19 | chr15 | 90451604 | 90451604 | Missense_Mutation | SNP | T | T | C | novel | Somatic | H70R | 0.347032 | NM_182616.4 | 874 | 570 | 304 |
| 848-PTsup | ECI1 | 602 | hg19 | chr16 | 2290078 | 2290078 | Missense_Mutation | SNP | C | C | T | novel | Somatic | D271N | 0.164699 | NM_001919.4 | 1779 | 1486 | 293 |
| 848-PTsup | ABCC1 | 88 | hg19 | chr16 | 16200707 | 16200707 | Missense_Mutation | SNP | G | G | A | novel | Somatic | A950T | 0.22569 | NM_004996.4 | 832 | 644 | 188 |
| 848-PTsup | ZNF646 | 822 | hg19 | chr16 | 31090337 | 31090337 | Missense_Mutation | SNP | C | C | T | novel | Somatic | L898F | 0.178054 | NM_014699.4 | 1446 | 1188 | 258 |
| 848-PTsup | ZNF267 | 828 | hg19 | chr16 | 31926927 | 31926927 | Missense_Mutation | SNP | C | C | A | novel | Somatic | Q453K | 0.135484 | NM_003414.6 | 775 | 670 | 105 |
| 848-PTsup | IRX5 | 1004 | hg19 | chr16 | 54967457 | 54967457 | Missense_Mutation | SNP | G | G | T | novel | Somatic | R375L | 0.384201 | NM_005853.6 | 1114 | 686 | 428 |
| 848-PTsup | LPCAT2 | 15 | hg19 | chr16 | 55563831 | 55563831 | Missense_Mutation | SNP | G | G | T | novel | Somatic | R205L | 0.080285 | NM_017839.5 | 980 | 901 | 79 |
| 848-PTsup | CES1 | 1011 | hg19 | chr16 | 55844469 | 55844469 | Missense_Mutation | SNP | C | C | T | novel | Somatic | M426I | 0.224103 | NM_001025195.2 | 1476 | 1145 | 331 |
| 848-PTsup | TP53 | 642 | hg19 | chr17 | 7577067 | 7577067 | Nonsense_Mutation | SNP | T | T | A | novel | Somatic | K291Ter | 0.380097 | NM_000546.5 | 1447 | 897 | 550 |
| 848-PTsup | PIK3R6 | 651 | hg19 | chr17 | 8726793 | 8726793 | Silent | SNP | G | G | T | novel | Somatic | R513= | 0.161054 | NM_001010855.4 | 681 | 571 | 110 |
| 848-PTsup | EVPLL | 724 | hg19 | chr17 | 18285955 | 18285955 | Splice_Region | SNP | G | G | A | novel | Somatic |  | 0.20935 | NM_001145127.2 | 492 | 389 | 103 |
| 848-PTsup | NLE1 | 840 | hg19 | chr17 | 33464561 | 33464561 | Splice_Site | SNP | C | C | T | novel | Somatic |  | 0.137736 | NM_018096.5 | 530 | 457 | 73 |
| 848-PTsup | KRT14 | 888 | hg19 | chr17 | 39742678 | 39742678 | Nonsense_Mutation | SNP | C | C | A | novel | Somatic | E137Ter | 0.405773 | NM_000526.5 | 1836 | 1091 | 745 |
| 848-PTsup | DBF4B | 911 | hg19 | chr17 | 42809600 | 42809600 | Missense_Mutation | SNP | C | C | T | novel | Somatic | R175C | 0.160271 | NM_145663.3 | 886 | 744 | 142 |
| 848-PTsup | TUBB6 | 84 | hg19 | chr18 | 12325600 | 12325600 | Missense_Mutation | SNP | C | C | T | novel | Somatic | A271V | 0.022436 | NM_032525.3 | 1872 | 1830 | 42 |
| 848-PTsup | ASXL3 | 102 | hg19 | chr18 | 31326113 | 31326113 | Missense_Mutation | SNP | G | G | A | novel | Somatic | V2101I | 0.207891 | NM_030632.3 | 659 | 522 | 137 |
| 848-PTsup | TLE2 | 75 | hg19 | chr19 | 3000686 | 3000686 | Missense_Mutation | SNP | G | G | T | novel | Somatic | L695I | 0.197917 | NM_003260.5 | 958 | 768 | 190 |
| 848-PTsup | CD70 | 635 | hg19 | chr19 | 6586080 | 6586080 | Missense_Mutation | SNP | G | G | T | novel | Somatic | S178Y | 0.134367 | NM_001252.5 | 1546 | 1338 | 208 |
| 848-PTsup | ZNF557 | 79 | hg19 | chr19 | 7083388 | 7083388 | Missense_Mutation | SNP | C | C | G | novel | Somatic | S309W | 0.391901 | NM_024341.3 | 1457 | 886 | 571 |
| 848-PTsup | MUC16 | 81 | hg19 | chr19 | 9056653 | 9056653 | Missense_Mutation | SNP | C | C | T | novel | Somatic | D10265N | 0.139973 | NM_024690.2 | 743 | 639 | 104 |
| 848-PTsup | MUC16 | 81 | hg19 | chr19 | 9056782 | 9056782 | Missense_Mutation | SNP | C | C | T | novel | Somatic | E10222K | 0.157303 | NM_024690.2 | 1068 | 900 | 168 |
| 848-PTsup | ZNF440 | 84 | hg19 | chr19 | 11942890 | 11942890 | Missense_Mutation | SNP | G | G | A | novel | Somatic | R300H | 0.152511 | NM_152357.3 | 1094 | 927 | 167 |
| 848-PTsup | RFX1 | 692 | hg19 | chr19 | 14077237 | 14077237 | Missense_Mutation | SNP | C | C | A | novel | Somatic | A653S | 0.147335 | NM_002918.5 | 637 | 543 | 94 |
| 848-PTsup | ZNF738 | 749 | hg19 | chr19 | 21558109 | 21558109 | Nonsense_Mutation | SNP | C | C | T | novel | Somatic | Q56Ter | 0.128995 | NM_001355237.2 | 876 | 763 | 113 |
| 848-PTsup | ZNF738 | 749 | hg19 | chr19 | 21558740 | 21558740 | Missense_Mutation | SNP | G | G | C | novel | Somatic | R98T | 0.183511 | NM_001355237.2 | 752 | 614 | 138 |
| 848-PTsup | NPHS1 | 862 | hg19 | chr19 | 36335301 | 36335301 | Missense_Mutation | SNP | G | G | A | novel | Somatic | A664V | 0.312389 | NM_004646.3 | 1684 | 1157 | 527 |
| 848-PTsup | ZNF529 | 867 | hg19 | chr19 | 37047043 | 37047043 | Missense_Mutation | SNP | A | A | G | novel | Somatic | F24S | 0.139535 | NM_020951.5 | 903 | 777 | 126 |
| 848-PTsup | ZNF585A | 872 | hg19 | chr19 | 37642547 | 37642547 | Missense_Mutation | SNP | C | C | A | novel | Somatic | G752W | 0.186267 | NM_001288800.2 | 1369 | 1114 | 255 |
| 848-PTsup | RINL | 885 | hg19 | chr19 | 39360749 | 39360749 | In_Frame_Ins | INS | - | - | GCCCCCGGAGGCCCC | novel | Somatic | G388_A392dup | 0.037702 | NM_001195833.2 | 1104 | 1062 | 42 |
| 848-PTsup | ZNF227 | 926 | hg19 | chr19 | 44739011 | 44739011 | Missense_Mutation | SNP | C | C | G | novel | Somatic | S143C | 0.122129 | NM_182490.3 | 957 | 840 | 117 |
| 848-PTsup | RELB | 932 | hg19 | chr19 | 45528957 | 45528957 | Missense_Mutation | SNP | G | G | A | novel | Somatic | R283Q | 0.248879 | NM_006509.4 | 891 | 669 | 222 |
| 848-PTsup | PPP1R13L | 935 | hg19 | chr19 | 45898771 | 45898771 | Missense_Mutation | SNP | G | G | A | novel | Somatic | P426L | 0.12633 | NM_006663.4 | 1504 | 1314 | 190 |
| 848-PTsup | PPP1R13L | 935 | hg19 | chr19 | 45898781 | 45898781 | Nonsense_Mutation | SNP | G | G | A | novel | Somatic | Q423Ter | 0.128651 | NM_006663.4 | 1438 | 1253 | 185 |
| 848-PTsup | DHX34 | 950 | hg19 | chr19 | 47858386 | 47858386 | Missense_Mutation | SNP | C | C | T | novel | Somatic | R266W | 0.149284 | NM_014681.6 | 976 | 830 | 146 |
| 848-PTsup | DPRX | 998 | hg19 | chr19 | 54139917 | 54139917 | Missense_Mutation | SNP | A | A | C | novel | Somatic | E84A | 0.146168 | NM_001012728.1 | 1122 | 958 | 164 |
| 848-PTsup | DEFB119 | 813 | hg19 | chr20 | 29965166 | 29965166 | Nonsense_Mutation | SNP | G | G | T | novel | Somatic | Y46Ter | 0.139706 | NM_153289.4 | 813 | 699 | 114 |
| 848-PTsup | XKR7 | 818 | hg19 | chr20 | 30556280 | 30556280 | Missense_Mutation | SNP | C | C | G | novel | Somatic | S101W | 0.183374 | NM_001011718.2 | 817 | 667 | 150 |
| 848-PTsup | DSCAM | 1 | hg19 | chr21 | 41668063 | 41668063 | Missense_Mutation | SNP | C | C | T | novel | Somatic | G701R | 0.42915 | NM_001389.5 | 494 | 282 | 212 |
| 848-PTsup | PKDREJ | 940 | hg19 | chr22 | 46656184 | 46656184 | Missense_Mutation | SNP | C | C | A | novel | Somatic | L1012F | 0.173028 | NM_006071.2 | 786 | 650 | 136 |
| 848-PTsup | SHROOM2 | 660 | hg19 | chrX | 9900238 | 9900238 | Missense_Mutation | SNP | A | A | G | novel | Somatic | E972G | 0.243802 | NM_001649.4 | 726 | 549 | 177 |
| 848-PTsup | HCCS | 83 | hg19 | chrX | 11139860 | 11139860 | Missense_Mutation | SNP | G | G | T | novel | Somatic | R246L | 0.177835 | NM_005333.5 | 773 | 635 | 138 |
| 848-PTsup | CYBB | 872 | hg19 | chrX | 37652977 | 37652977 | Missense_Mutation | SNP | A | A | G | novel | Somatic | N133D | 0.260763 | NM_000397.4 | 813 | 601 | 212 |
| 848-PTsup | CASK | 112 | hg19 | chrX | 41481918 | 41481918 | Intron | SNP | C | C | A | novel | Somatic |  | 0.144348 | NM_001367721.1 | 575 | 492 | 83 |
| 848-PTsup | ZNF157 | 945 | hg19 | chrX | 47271884 | 47271884 | Nonsense_Mutation | SNP | G | G | T | novel | Somatic | G138Ter | 0.129108 | NM_003446.3 | 851 | 741 | 110 |
| 848-PTsup | LRCH2 | 182 | hg19 | chrX | 114347878 | 114347878 | Missense_Mutation | SNP | A | A | T | novel | Somatic | H733Q | 0.417112 | NM_020871.4 | 561 | 327 | 234 |
| 848-PTsup | AGTR2 | 1464 | hg19 | chrX | 115303867 | 115303867 | Missense_Mutation | SNP | T | T | C | novel | Somatic | F112L | 0.249564 | NM_000686.5 | 573 | 430 | 143 |
| 848-PTsup | GLUD2 | 1501 | hg19 | chrX | 120182895 | 120182895 | Missense_Mutation | SNP | C | C | T | novel | Somatic | R453C | 0.207044 | NM_012084.4 | 936 | 742 | 194 |
| 848-PTdeep | PADI1 | 89 | hg19 | chr1 | 17548884 | 17548884 | Silent | SNP | C | C | T | novel | Somatic | G64= | 0.231801 | NM_013358.3 | 522 | 401 | 121 |
| 848-PTdeep | MTF2 | 162 | hg19 | chr1 | 93602520 | 93602520 | Missense_Mutation | SNP | G | G | A | novel | Somatic | R573H | 0.204593 | NM_007358.4 | 479 | 381 | 98 |
| 848-PTdeep | COL11A1 | 171 | hg19 | chr1 | 103463910 | 103463910 | Missense_Mutation | SNP | C | C | T | novel | Somatic | G730R | 0.174632 | NM_080629.2 | 544 | 449 | 95 |
| 848-PTdeep | KCND3 | 180 | hg19 | chr1 | 112322918 | 112322918 | Missense_Mutation | SNP | G | G | T | novel | Somatic | H464N | 0.27381 | NM_004980.4 | 252 | 183 | 69 |
| 848-PTdeep | SPAG17 | 186 | hg19 | chr1 | 118527339 | 118527339 | Missense_Mutation | SNP | A | A | T | novel | Somatic | F1912L | 0.237903 | NM_206996.4 | 496 | 378 | 118 |
| 848-PTdeep | OR6Y1 | 1794 | hg19 | chr1 | 158517290 | 158517290 | Missense_Mutation | SNP | C | C | T | novel | Somatic | M202I | 0.297218 | NM_001005189.1 | 682 | 479 | 203 |
| 848-PTdeep | LAX1 | 2139 | hg19 | chr1 | 203743576 | 203743576 | Missense_Mutation | SNP | G | G | A | novel | Somatic | E322K | 0.286275 | NM_017773.4 | 765 | 546 | 219 |
| 848-PTdeep | SOX13 | 267 | hg19 | chr1 | 204093869 | 204093869 | Missense_Mutation | SNP | G | G | T | novel | Somatic | K492N | 0.326953 | NM_005686.3 | 1306 | 879 | 427 |
| 848-PTdeep | NFASC | 2148 | hg19 | chr1 | 204981927 | 204981927 | Nonsense_Mutation | SNP | C | C | T | novel | Somatic | R1142Ter | 0.218062 | NM_001005388.2 | 454 | 355 | 99 |
| 848-PTdeep | CR2 | 2169 | hg19 | chr1 | 207649749 | 207649749 | Missense_Mutation | SNP | G | G | T | novel | Somatic | A963S | 0.314904 | NM_001006658.2 | 415 | 284 | 131 |
| 848-PTdeep | GUK1 | 2327 | hg19 | chr1 | 228328021 | 228328021 | Frame_Shift_Del | DEL | GGCCGGGCTGGCTGCGGCCGCCCTG | GGCCGGGCTGGCTGCGGCCGCCCTG | - | novel | Somatic | L9PfsTer14 | 0.066185 | NM_001242840.2 | 831 | 776 | 55 |
| 848-PTdeep | PCNX2 | 2365 | hg19 | chr1 | 233161036 | 233161036 | Missense_Mutation | SNP | G | G | T | novel | Somatic | N1487K | 0.283964 | NM_014801.4 | 897 | 642 | 255 |
| 848-PTdeep | ADCY3 | 776 | hg19 | chr2 | 25141376 | 25141376 | Missense_Mutation | SNP | G | G | A | novel | Somatic | R161C | 0.143555 | NM_001320613.2 | 1024 | 877 | 147 |
| 848-PTdeep | GAREM2 | 786 | hg19 | chr2 | 26407588 | 26407588 | Missense_Mutation | SNP | C | C | G | novel | Somatic | L291V | 0.404075 | NM_001168241.2 | 588 | 350 | 238 |
| 848-PTdeep | KCNG3 | 910 | hg19 | chr2 | 42720473 | 42720473 | Missense_Mutation | SNP | C | C | T | novel | Somatic | E57K | 0.210692 | NM_133329.6 | 636 | 502 | 134 |
| 848-PTdeep | AFF3 | 2 | hg19 | chr2 | 100170808 | 100170808 | Missense_Mutation | SNP | C | C | A | novel | Somatic | W1200L | 0.134766 | NM_001025108.2 | 1023 | 885 | 138 |
| 848-PTdeep | ST6GAL2 | 175 | hg19 | chr2 | 107460187 | 107460187 | Missense_Mutation | SNP | C | C | T | novel | Somatic | A83T | 0.46071 | NM_001322362.2 | 928 | 500 | 428 |
| 848-PTdeep | CNTNAP5 | 192 | hg19 | chr2 | 125521709 | 125521709 | Nonsense_Mutation | SNP | C | C | T | novel | Somatic | R840Ter | 0.187204 | NM_001367498.1 | 422 | 343 | 79 |
| 848-PTdeep | AMER3 | 1588 | hg19 | chr2 | 131519833 | 131519833 | Missense_Mutation | SNP | A | A | G | novel | Somatic | D63G | 0.242 | NM_152698.3 | 499 | 378 | 121 |
| 848-PTdeep | LRP1B | 3 | hg19 | chr2 | 141625277 | 141625277 | Nonsense_Mutation | SNP | C | C | T | novel | Somatic | W1487Ter | 0.210046 | NM_018557.3 | 436 | 344 | 92 |
| 848-PTdeep | NEB | 218 | hg19 | chr2 | 152394755 | 152394755 | Missense_Mutation | SNP | G | G | A | novel | Somatic | R6966C | 0.171717 | NM_001271208.2 | 198 | 164 | 34 |
| 848-PTdeep | TTN | 1955 | hg19 | chr2 | 179489290 | 179489290 | Missense_Mutation | SNP | A | A | G | novel | Somatic | V14906A | 0.430851 | NM_001267550.2 | 372 | 210 | 162 |
| 848-PTdeep | TTN | 1955 | hg19 | chr2 | 179580313 | 179580313 | Missense_Mutation | SNP | G | G | T | novel | Somatic | H8610N | 0.452336 | NM_001267550.2 | 534 | 292 | 242 |
| 848-PTdeep | ERBB4 | 34 | hg19 | chr2 | 212289014 | 212289014 | Nonsense_Mutation | SNP | C | C | T | novel | Somatic | W911Ter | 0.196581 | NM_005235.3 | 351 | 282 | 69 |
| 848-PTdeep | PLCD4 | 2259 | hg19 | chr2 | 219500624 | 219500624 | Missense_Mutation | SNP | C | C | T | novel | Somatic | R668W | 0.168675 | NM_032726.4 | 498 | 414 | 84 |
| 848-PTdeep | CUL3 | 2304 | hg19 | chr2 | 225339071 | 225339071 | Missense_Mutation | SNP | C | C | A | novel | Somatic | R739L | 0.195122 | NM_001257198.2 | 409 | 329 | 80 |
| 848-PTdeep | KCNH8 | 91 | hg19 | chr3 | 19554522 | 19554522 | Missense_Mutation | SNP | G | G | A | novel | Somatic | E714K | 0.245098 | NM_144633.3 | 510 | 385 | 125 |
| 848-PTdeep | CCR8 | 885 | hg19 | chr3 | 39374178 | 39374178 | Missense_Mutation | SNP | G | G | A | novel | Somatic | S119N | 0.184502 | NM_005201.4 | 541 | 441 | 100 |
| 848-PTdeep | CSPG5 | 948 | hg19 | chr3 | 47618698 | 47618698 | Missense_Mutation | SNP | T | T | G | novel | Somatic | D273A | 0.219761 | NM_006574.4 | 587 | 458 | 129 |
| 848-PTdeep | KLHDC8B | 960 | hg19 | chr3 | 49212285 | 49212285 | Missense_Mutation | SNP | G | G | A | novel | Somatic | A218T | 0.24833 | NM_173546.3 | 895 | 672 | 223 |
| 848-PTdeep | EPHA3 | 158 | hg19 | chr3 | 89521723 | 89521723 | Missense_Mutation | SNP | G | G | A | novel | Somatic | G934S | 0.416813 | NM_005233.6 | 570 | 332 | 238 |
| 848-PTdeep | TRIM42 | 1656 | hg19 | chr3 | 140401662 | 140401662 | Missense_Mutation | SNP | G | G | A | novel | Somatic | G234R | 0.144641 | NM_152616.5 | 961 | 822 | 139 |
| 848-PTdeep | GK5 | 1667 | hg19 | chr3 | 141934501 | 141934501 | Missense_Mutation | SNP | C | C | T | novel | Somatic | E61K | 0.130739 | NM_001039547.3 | 1000 | 869 | 131 |
| 848-PTdeep | ANKUB1 | 1725 | hg19 | chr3 | 149485876 | 149485876 | Missense_Mutation | SNP | C | C | A | novel | Somatic | Q191H | 0.187879 | NM_001144960.3 | 330 | 268 | 62 |
| 848-PTdeep | KCNAB1 | 27 | hg19 | chr3 | 156232230 | 156232230 | Missense_Mutation | SNP | G | G | A | novel | Somatic | E246K | 0.177019 | NM_172160.2 | 322 | 265 | 57 |
| 848-PTdeep | B3GNT5 | 247 | hg19 | chr3 | 182988544 | 182988544 | Missense_Mutation | SNP | A | A | G | novel | Somatic | M320V | 0.106952 | NM_032047.5 | 1121 | 1001 | 120 |
| 848-PTdeep | AFAP1 | 80 | hg19 | chr4 | 7795457 | 7795457 | Missense_Mutation | SNP | C | C | A | novel | Somatic | D455Y | 0.230303 | NM_001134647.1 | 495 | 381 | 114 |
| 848-PTdeep | LIMCH1 | 112 | hg19 | chr4 | 41634839 | 41634839 | Missense_Mutation | SNP | G | G | C | novel | Somatic | E559Q | 0.317073 | NM_001330672.2 | 369 | 252 | 117 |
| 848-PTdeep | POU4F2 | 1710 | hg19 | chr4 | 147560504 | 147560504 | Missense_Mutation | SNP | G | G | T | novel | Somatic | S71I | 0.116071 | NM_004575.3 | 446 | 394 | 52 |
| 848-PTdeep | GRIA2 | 223 | hg19 | chr4 | 158283975 | 158283975 | Missense_Mutation | SNP | A | A | C | novel | Somatic | S811R | 0.085965 | NM_001083619.1 | 568 | 519 | 49 |
| 848-PTdeep | DDX60 | 234 | hg19 | chr4 | 169196671 | 169196671 | Intron | SNP | G | G | C | novel | Somatic |  | 0.263587 | NM_017631.6 | 368 | 271 | 97 |
| 848-PTdeep | TRIML2 | 2027 | hg19 | chr4 | 189012729 | 189012729 | Missense_Mutation | SNP | C | C | T | novel | Somatic | G396D | 0.303748 | NM_001303419.1 | 507 | 353 | 154 |
| 848-PTdeep | ADAMTS16 | 9 | hg19 | chr5 | 5262779 | 5262779 | Missense_Mutation | SNP | C | C | G | novel | Somatic | T891S | 0.202091 | NM_139056.4 | 287 | 229 | 58 |
| 848-PTdeep | PCDHAC2 | 1655 | hg19 | chr5 | 140347516 | 140347516 | Missense_Mutation | SNP | G | G | T | novel | Somatic | V389F | 0.516917 | NM_018899.6 | 532 | 257 | 275 |
| 848-PTdeep | PCDHB11 | 1657 | hg19 | chr5 | 140579789 | 140579789 | Frame_Shift_Ins | INS | - | - | T | novel | Somatic | G149WfsTer33 | 0.504082 | NM_018931.3 | 488 | 241 | 247 |
| 848-PTdeep | KCTD16 | 26 | hg19 | chr5 | 143853531 | 143853531 | Frame_Shift_Del | DEL | A | A | - | novel | Somatic | A384LfsTer20 | 0.021534 | NM_020768.4 | 741 | 725 | 16 |
| 848-PTdeep | NMUR2 | 217 | hg19 | chr5 | 151784001 | 151784001 | Missense_Mutation | SNP | T | T | C | novel | Somatic | Y225C | 0.119617 | NM_020167.4 | 418 | 368 | 50 |
| 848-PTdeep | DRD1 | 1919 | hg19 | chr5 | 174869394 | 174869394 | Missense_Mutation | SNP | G | G | A | novel | Somatic | H237Y | 0.178344 | NM_000794.5 | 783 | 643 | 140 |
| 848-PTdeep | SFXN1 | 1919 | hg19 | chr5 | 174919169 | 174919169 | Missense_Mutation | SNP | C | C | A | novel | Somatic | F21L | 0.131021 | NM_022754.7 | 1037 | 901 | 136 |
| 848-PTdeep | CPLX2 | 1922 | hg19 | chr5 | 175306051 | 175306051 | Missense_Mutation | SNP | G | G | A | novel | Somatic | E58K | 0.206237 | NM_001008220.2 | 993 | 788 | 205 |
| 848-PTdeep | HUS1B | 590 | hg19 | chr6 | 656542 | 656542 | Missense_Mutation | SNP | G | G | A | novel | Somatic | R135W | 0.203682 | NM_148959.3 | 869 | 692 | 177 |
| 848-PTdeep | PGBD1 | 800 | hg19 | chr6 | 28269624 | 28269624 | Missense_Mutation | SNP | C | C | A | novel | Somatic | H665N | 0.125786 | NM_001184743.1 | 318 | 278 | 40 |
| 848-PTdeep | C6orf89 | 866 | hg19 | chr6 | 36870003 | 36870003 | Missense_Mutation | SNP | G | G | A | novel | Somatic | A73T | 0.321121 | NM_152734.4 | 464 | 315 | 149 |
| 848-PTdeep | DEFB113 | 965 | hg19 | chr6 | 49936573 | 49936573 | Missense_Mutation | SNP | C | C | G | novel | Somatic | Q22H | 0.153518 | NM_001037729.1 | 469 | 397 | 72 |
| 848-PTdeep | MTRF1L | 1754 | hg19 | chr6 | 153319684 | 153319684 | Splice_Site | SNP | C | C | A | novel | Somatic |  | 0.186577 | NM_019041.7 | 745 | 606 | 139 |
| 848-PTdeep | AGPAT4 | 227 | hg19 | chr6 | 161560504 | 161560504 | Missense_Mutation | SNP | C | C | A | novel | Somatic | R331M | 0.200448 | NM_020133.3 | 893 | 714 | 179 |
| 848-PTdeep | TWISTNB | 735 | hg19 | chr7 | 19739753 | 19739753 | Missense_Mutation | SNP | G | G | A | novel | Somatic | R183C | 0.162835 | NM_001002926.2 | 522 | 437 | 85 |
| 848-PTdeep | MAGI2 | 18 | hg19 | chr7 | 79082546 | 79082546 | Missense_Mutation | SNP | C | C | G | novel | Somatic | E31Q | 0.246753 | NM_012301.4 | 462 | 348 | 114 |
| 848-PTdeep | COL1A2 | 1302 | hg19 | chr7 | 94053715 | 94053715 | Missense_Mutation | SNP | C | C | T | novel | Somatic | S878L | 0.181818 | NM_000089.4 | 561 | 459 | 102 |
| 848-PTdeep | SLC25A13 | 164 | hg19 | chr7 | 95818605 | 95818605 | Splice_Site | SNP | C | C | T | novel | Somatic |  | 0.243836 | NM_014251.3 | 365 | 276 | 89 |
| 848-PTdeep | CSMD1 | 9 | hg19 | chr8 | 3081326 | 3081326 | Missense_Mutation | SNP | G | G | A | novel | Somatic | P1470L | 0.509843 | NM_033225.6 | 507 | 248 | 259 |
| 848-PTdeep | MCPH1 | 9 | hg19 | chr8 | 6289099 | 6289099 | Frame_Shift_Del | DEL | A | A | - | novel | Somatic | K107NfsTer39 | 0.025292 | NM_024596.5 | 514 | 501 | 13 |
| 848-PTdeep | KAT6A | 112 | hg19 | chr8 | 41791261 | 41791261 | Frame_Shift_Ins | INS | - | - | CTGG | novel | Somatic | S1493PfsTer15 | 0.17067 | NM_006766.5 | 775 | 640 | 135 |
| 848-PTdeep | YTHDF3 | 134 | hg19 | chr8 | 64099908 | 64099908 | Missense_Mutation | SNP | C | C | T | novel | Somatic | R446C | 0.178515 | NM_152758.6 | 631 | 518 | 113 |
| 848-PTdeep | CSMD3 | 22 | hg19 | chr8 | 113599378 | 113599378 | Missense_Mutation | SNP | T | T | C | novel | Somatic | I1268V | 0.148855 | NM_198123.2 | 524 | 446 | 78 |
| 848-PTdeep | DEPTOR | 188 | hg19 | chr8 | 120940713 | 120940713 | Missense_Mutation | SNP | G | G | A | novel | Somatic | A66T | 0.177945 | NM_022783.4 | 798 | 656 | 142 |
| 848-PTdeep | PAPPA | 186 | hg19 | chr9 | 118997469 | 118997469 | Missense_Mutation | SNP | C | C | T | novel | Somatic | P762L | 0.204499 | NM_002581.5 | 489 | 389 | 100 |
| 848-PTdeep | NELFB | 1654 | hg19 | chr9 | 140149972 | 140149972 | Missense_Mutation | SNP | G | G | A | novel | Somatic | G52E | 0.480603 | NM_015456.5 | 463 | 240 | 223 |
| 848-PTdeep | CUBN | 89 | hg19 | chr10 | 17107611 | 17107611 | Missense_Mutation | SNP | G | G | A | novel | Somatic | S1012F | 0.183486 | NM_001081.4 | 544 | 444 | 100 |
| 848-PTdeep | ZNF33B | 114 | hg19 | chr10 | 43127384 | 43127384 | Missense_Mutation | SNP | G | G | T | novel | Somatic | S81R | 0.190939 | NM_006955.3 | 309 | 250 | 59 |
| 848-PTdeep | KCNMA1 | 1190 | hg19 | chr10 | 78844467 | 78844467 | Missense_Mutation | SNP | G | G | A | novel | Somatic | A484V | 0.194444 | NM_001161352.2 | 467 | 376 | 91 |
| 848-PTdeep | OR4C15 | 1007 | hg19 | chr11 | 55322321 | 55322321 | Missense_Mutation | SNP | G | G | T | novel | Somatic | C126F | 0.220302 | NM_001001920.2 | 461 | 359 | 102 |
| 848-PTdeep | VPS37C | 1049 | hg19 | chr11 | 60899539 | 60899539 | Missense_Mutation | SNP | C | C | T | novel | Somatic | G274E | 0.248062 | NM_017966.5 | 644 | 484 | 160 |
| 848-PTdeep | ARHGAP20 | 178 | hg19 | chr11 | 110485337 | 110485337 | Missense_Mutation | SNP | G | G | A | novel | Somatic | P193L | 0.173853 | NM_020809.3 | 717 | 592 | 125 |
| 848-PTdeep | CD163 | 643 | hg19 | chr12 | 7639511 | 7639511 | Nonsense_Mutation | SNP | C | C | A | novel | Somatic | E708Ter | 0.332103 | NM_004244.5 | 541 | 361 | 180 |
| 848-PTdeep | KLRF1 | 661 | hg19 | chr12 | 9997090 | 9997090 | In_Frame_Ins | INS | - | - | GCA | novel | Somatic | S223dup | 0.140312 | NM_016523.3 | 895 | 769 | 126 |
| 848-PTdeep | LOC255308 | Unknown | hg19 | chr12 | 10659547 | 10659547 | Missense_Mutation | SNP | G | G | A | novel | Somatic | R349Q | 0.332491 | NM_001357734.1 | 790 | 527 | 263 |
| 848-PTdeep | ARID2 | 14 | hg19 | chr12 | 46245781 | 46245781 | Missense_Mutation | SNP | G | G | A | novel | Somatic | S1292N | 0.167539 | NM_152641.4 | 571 | 475 | 96 |
| 848-PTdeep | SMARCC2 | 1016 | hg19 | chr12 | 56575296 | 56575296 | Missense_Mutation | SNP | G | G | C | novel | Somatic | P309R | 0.157303 | NM_001330288.2 | 534 | 450 | 84 |
| 848-PTdeep | STAT2 | 1017 | hg19 | chr12 | 56737279 | 56737279 | Missense_Mutation | SNP | G | G | A | novel | Somatic | P817L | 0.024194 | NM_005419.4 | 866 | 845 | 21 |
| 848-PTdeep | LYZ | 1117 | hg19 | chr12 | 69746053 | 69746053 | Missense_Mutation | SNP | C | C | T | novel | Somatic | R119C | 0.170418 | NM_000239.3 | 622 | 516 | 106 |
| 848-PTdeep | NAV3 | 147 | hg19 | chr12 | 78400658 | 78400658 | Missense_Mutation | SNP | C | C | T | novel | Somatic | A447V | 0.157699 | NM_001024383.2 | 535 | 450 | 85 |
| 848-PTdeep | SLC6A15 | 1235 | hg19 | chr12 | 85266555 | 85266555 | Missense_Mutation | SNP | C | C | T | novel | Somatic | M376I | 0.187713 | NM_182767.6 | 586 | 476 | 110 |
| 848-PTdeep | ANKS1B | 167 | hg19 | chr12 | 99837530 | 99837530 | Missense_Mutation | SNP | G | G | A | novel | Somatic | S499L | 0.173835 | NM_001352186.1 | 558 | 461 | 97 |
| 848-PTdeep | FANCM | 116 | hg19 | chr14 | 45628301 | 45628301 | Missense_Mutation | SNP | G | G | A | novel | Somatic | E467K | 0.184549 | NM_020937.4 | 233 | 190 | 43 |
| 848-PTdeep | C15orf39 | 2 | hg19 | chr15 | 75500089 | 75500089 | Nonsense_Mutation | SNP | C | C | A | novel | Somatic | S567Ter | 0.333333 | NM_015492.5 | 624 | 416 | 208 |
| 848-PTdeep | ARPIN | 1275 | hg19 | chr15 | 90451604 | 90451604 | Missense_Mutation | SNP | T | T | C | novel | Somatic | H70R | 0.392105 | NM_182616.4 | 759 | 461 | 298 |
| 848-PTdeep | ECI1 | 602 | hg19 | chr16 | 2290078 | 2290078 | Missense_Mutation | SNP | C | C | T | novel | Somatic | D271N | 0.198464 | NM_001919.4 | 1561 | 1251 | 310 |
| 848-PTdeep | ABCC1 | 88 | hg19 | chr16 | 16200707 | 16200707 | Missense_Mutation | SNP | G | G | A | novel | Somatic | A950T | 0.229907 | NM_004996.4 | 534 | 411 | 123 |
| 848-PTdeep | ZNF646 | 822 | hg19 | chr16 | 31090337 | 31090337 | Missense_Mutation | SNP | C | C | T | novel | Somatic | L898F | 0.166542 | NM_014699.4 | 1332 | 1110 | 222 |
| 848-PTdeep | ZNF267 | 828 | hg19 | chr16 | 31926927 | 31926927 | Missense_Mutation | SNP | C | C | A | novel | Somatic | Q453K | 0.167883 | NM_003414.6 | 548 | 456 | 92 |
| 848-PTdeep | IRX5 | 1004 | hg19 | chr16 | 54967457 | 54967457 | Missense_Mutation | SNP | G | G | T | novel | Somatic | R375L | 0.346363 | NM_005853.6 | 921 | 602 | 319 |
| 848-PTdeep | LPCAT2 | 15 | hg19 | chr16 | 55563831 | 55563831 | Missense_Mutation | SNP | G | G | T | novel | Somatic | R205L | 0.146611 | NM_017839.5 | 721 | 615 | 106 |
| 848-PTdeep | CES1 | 1011 | hg19 | chr16 | 55844469 | 55844469 | Missense_Mutation | SNP | C | C | T | novel | Somatic | M426I | 0.202566 | NM_001025195.2 | 1088 | 867 | 221 |
| 848-PTdeep | TP53 | 642 | hg19 | chr17 | 7577067 | 7577067 | Nonsense_Mutation | SNP | T | T | A | novel | Somatic | K291Ter | 0.360825 | NM_000546.5 | 1164 | 744 | 420 |
| 848-PTdeep | PIK3R6 | 651 | hg19 | chr17 | 8726793 | 8726793 | Silent | SNP | G | G | T | novel | Somatic | R513= | 0.15367 | NM_001010855.4 | 435 | 368 | 67 |
| 848-PTdeep | NLE1 | 840 | hg19 | chr17 | 33464561 | 33464561 | Splice_Site | SNP | C | C | T | novel | Somatic |  | 0.133178 | NM_018096.5 | 428 | 371 | 57 |
| 848-PTdeep | KRT14 | 888 | hg19 | chr17 | 39742678 | 39742678 | Nonsense_Mutation | SNP | C | C | A | novel | Somatic | E137Ter | 0.455521 | NM_000526.5 | 1585 | 863 | 722 |
| 848-PTdeep | DBF4B | 911 | hg19 | chr17 | 42809600 | 42809600 | Missense_Mutation | SNP | C | C | T | novel | Somatic | R175C | 0.131397 | NM_145663.3 | 721 | 626 | 95 |
| 848-PTdeep | ASXL3 | 102 | hg19 | chr18 | 31326113 | 31326113 | Missense_Mutation | SNP | G | G | A | novel | Somatic | V2101I | 0.165842 | NM_030632.3 | 404 | 337 | 67 |
| 848-PTdeep | TLE2 | 75 | hg19 | chr19 | 3000686 | 3000686 | Missense_Mutation | SNP | G | G | T | novel | Somatic | L695I | 0.201379 | NM_003260.5 | 725 | 579 | 146 |
| 848-PTdeep | CD70 | 635 | hg19 | chr19 | 6586080 | 6586080 | Missense_Mutation | SNP | G | G | T | novel | Somatic | S178Y | 0.137675 | NM_001252.5 | 1211 | 1044 | 167 |
| 848-PTdeep | ZNF557 | 79 | hg19 | chr19 | 7083388 | 7083388 | Missense_Mutation | SNP | C | C | G | novel | Somatic | S309W | 0.391477 | NM_024341.3 | 1007 | 612 | 395 |
| 848-PTdeep | MUC16 | 81 | hg19 | chr19 | 9056653 | 9056653 | Missense_Mutation | SNP | C | C | T | novel | Somatic | D10265N | 0.131886 | NM_024690.2 | 599 | 520 | 79 |
| 848-PTdeep | MUC16 | 81 | hg19 | chr19 | 9056782 | 9056782 | Missense_Mutation | SNP | C | C | T | novel | Somatic | E10222K | 0.134707 | NM_024690.2 | 631 | 546 | 85 |
| 848-PTdeep | ZNF440 | 84 | hg19 | chr19 | 11942890 | 11942890 | Missense_Mutation | SNP | G | G | A | novel | Somatic | R300H | 0.133065 | NM_152357.3 | 742 | 643 | 99 |
| 848-PTdeep | RFX1 | 692 | hg19 | chr19 | 14077237 | 14077237 | Missense_Mutation | SNP | C | C | A | novel | Somatic | A653S | 0.179104 | NM_002918.5 | 535 | 439 | 96 |
| 848-PTdeep | ZNF738 | 749 | hg19 | chr19 | 21558109 | 21558109 | Nonsense_Mutation | SNP | C | C | T | novel | Somatic | Q56Ter | 0.109306 | NM_001355237.2 | 676 | 602 | 74 |
| 848-PTdeep | ZNF738 | 749 | hg19 | chr19 | 21558740 | 21558740 | Missense_Mutation | SNP | G | G | C | novel | Somatic | R98T | 0.146296 | NM_001355237.2 | 540 | 461 | 79 |
| 848-PTdeep | NPHS1 | 862 | hg19 | chr19 | 36335301 | 36335301 | Missense_Mutation | SNP | G | G | A | novel | Somatic | A664V | 0.316952 | NM_004646.3 | 1403 | 958 | 445 |
| 848-PTdeep | ZNF529 | 867 | hg19 | chr19 | 37047043 | 37047043 | Missense_Mutation | SNP | A | A | G | novel | Somatic | F24S | 0.121417 | NM_020951.5 | 593 | 521 | 72 |
| 848-PTdeep | ZNF585A | 872 | hg19 | chr19 | 37642547 | 37642547 | Missense_Mutation | SNP | C | C | A | novel | Somatic | G752W | 0.173673 | NM_001288800.2 | 903 | 746 | 157 |
| 848-PTdeep | RINL | 885 | hg19 | chr19 | 39360749 | 39360749 | In_Frame_Ins | INS | - | - | GCCCCCGGAGGCCCC | novel | Somatic | G388_A392dup | 0.027108 | NM_001195833.2 | 659 | 641 | 18 |
| 848-PTdeep | ZNF227 | 926 | hg19 | chr19 | 44739011 | 44739011 | Missense_Mutation | SNP | C | C | G | novel | Somatic | S143C | 0.121813 | NM_182490.3 | 706 | 620 | 86 |
| 848-PTdeep | RELB | 932 | hg19 | chr19 | 45528957 | 45528957 | Missense_Mutation | SNP | G | G | A | novel | Somatic | R283Q | 0.249292 | NM_006509.4 | 706 | 530 | 176 |
| 848-PTdeep | PPP1R13L | 935 | hg19 | chr19 | 45898771 | 45898771 | Missense_Mutation | SNP | G | G | A | novel | Somatic | P426L | 0.125 | NM_006663.4 | 997 | 872 | 125 |
| 848-PTdeep | PPP1R13L | 935 | hg19 | chr19 | 45898781 | 45898781 | Nonsense_Mutation | SNP | G | G | A | novel | Somatic | Q423Ter | 0.119464 | NM_006663.4 | 971 | 855 | 116 |
| 848-PTdeep | DHX34 | 950 | hg19 | chr19 | 47858386 | 47858386 | Missense_Mutation | SNP | C | C | T | novel | Somatic | R266W | 0.106061 | NM_014681.6 | 657 | 587 | 70 |
| 848-PTdeep | DPRX | 998 | hg19 | chr19 | 54139917 | 54139917 | Missense_Mutation | SNP | A | A | C | novel | Somatic | E84A | 0.116711 | NM_001012728.1 | 753 | 665 | 88 |
| 848-PTdeep | DEFB119 | 813 | hg19 | chr20 | 29965166 | 29965166 | Nonsense_Mutation | SNP | G | G | T | novel | Somatic | Y46Ter | 0.212121 | NM_153289.4 | 527 | 415 | 112 |
| 848-PTdeep | XKR7 | 818 | hg19 | chr20 | 30556280 | 30556280 | Missense_Mutation | SNP | C | C | G | novel | Somatic | S101W | 0.145138 | NM_001011718.2 | 689 | 589 | 100 |
| 848-PTdeep | DSCAM | 1 | hg19 | chr21 | 41668063 | 41668063 | Missense_Mutation | SNP | C | C | T | novel | Somatic | G701R | 0.366234 | NM_001389.5 | 385 | 244 | 141 |
| 848-PTdeep | CCT8L2 | 715 | hg19 | chr22 | 17072537 | 17072537 | Missense_Mutation | SNP | C | C | T | novel | Somatic | E302K | 0.032787 | NM_014406.5 | 488 | 472 | 16 |
| 848-PTdeep | PKDREJ | 940 | hg19 | chr22 | 46656184 | 46656184 | Missense_Mutation | SNP | C | C | A | novel | Somatic | L1012F | 0.191638 | NM_006071.2 | 571 | 461 | 110 |
| 848-PTdeep | SHROOM2 | 660 | hg19 | chrX | 9900238 | 9900238 | Missense_Mutation | SNP | A | A | G | novel | Somatic | E972G | 0.266968 | NM_001649.4 | 662 | 485 | 177 |
| 848-PTdeep | HCCS | 83 | hg19 | chrX | 11139860 | 11139860 | Missense_Mutation | SNP | G | G | T | novel | Somatic | R246L | 0.189895 | NM_005333.5 | 574 | 465 | 109 |
| 848-PTdeep | CYBB | 872 | hg19 | chrX | 37652977 | 37652977 | Missense_Mutation | SNP | A | A | G | novel | Somatic | N133D | 0.221172 | NM_000397.4 | 529 | 412 | 117 |
| 848-PTdeep | CASK | 112 | hg19 | chrX | 41481918 | 41481918 | Intron | SNP | C | C | A | novel | Somatic |  | 0.215426 | NM_001367721.1 | 376 | 295 | 81 |
| 848-PTdeep | ZNF157 | 945 | hg19 | chrX | 47271884 | 47271884 | Nonsense_Mutation | SNP | G | G | T | novel | Somatic | G138Ter | 0.121771 | NM_003446.3 | 542 | 476 | 66 |
| 848-PTdeep | LRCH2 | 182 | hg19 | chrX | 114347878 | 114347878 | Missense_Mutation | SNP | A | A | T | novel | Somatic | H733Q | 0.403141 | NM_020871.4 | 382 | 228 | 154 |
| 848-PTdeep | AGTR2 | 1464 | hg19 | chrX | 115303867 | 115303867 | Missense_Mutation | SNP | T | T | C | novel | Somatic | F112L | 0.237057 | NM_000686.5 | 367 | 280 | 87 |
| 848-PTdeep | GLUD2 | 1501 | hg19 | chrX | 120182895 | 120182895 | Missense_Mutation | SNP | C | C | T | novel | Somatic | R453C | 0.193252 | NM_012084.4 | 651 | 525 | 126 |
| 848-LNmet | ZBTB17 | 709 | hg19 | chr1 | 16270845 | 16270845 | Missense_Mutation | SNP | C | C | T | novel | Somatic | D441N | 0.14119 | NM_003443.2 | 857 | 736 | 121 |
| 848-LNmet | PADI1 | 89 | hg19 | chr1 | 17548884 | 17548884 | Silent | SNP | C | C | T | novel | Somatic | G64= | 0.104839 | NM_013358.3 | 620 | 555 | 65 |
| 848-LNmet | DEPDC1 | 138 | hg19 | chr1 | 68949767 | 68949767 | Missense_Mutation | SNP | C | C | T | novel | Somatic | R259K | 0.046729 | NM_001114120.3 | 320 | 305 | 15 |
| 848-LNmet | MTF2 | 162 | hg19 | chr1 | 93602520 | 93602520 | Missense_Mutation | SNP | G | G | A | novel | Somatic | R573H | 0.114537 | NM_007358.4 | 454 | 402 | 52 |
| 848-LNmet | COL11A1 | 171 | hg19 | chr1 | 103463910 | 103463910 | Missense_Mutation | SNP | C | C | T | novel | Somatic | G730R | 0.092334 | NM_080629.2 | 574 | 521 | 53 |
| 848-LNmet | KCND3 | 180 | hg19 | chr1 | 112322918 | 112322918 | Missense_Mutation | SNP | G | G | T | novel | Somatic | H464N | 0.093333 | NM_004980.4 | 225 | 204 | 21 |
| 848-LNmet | SYCP1 | 183 | hg19 | chr1 | 115469133 | 115469133 | Frame_Shift_Ins | INS | - | - | A | novel | Somatic | G629RfsTer12 | 0.027837 | NM_003176.4 | 467 | 454 | 13 |
| 848-LNmet | VANGL1 | 1471 | hg19 | chr1 | 116206634 | 116206634 | Missense_Mutation | SNP | G | G | A | novel | Somatic | R186H | 0.049896 | NM_138959.3 | 481 | 457 | 24 |
| 848-LNmet | SPAG17 | 186 | hg19 | chr1 | 118527339 | 118527339 | Missense_Mutation | SNP | A | A | T | novel | Somatic | F1912L | 0.098394 | NM_206996.4 | 496 | 447 | 49 |
| 848-LNmet | SV2A | 1728 | hg19 | chr1 | 149885223 | 149885223 | In_Frame_Del | DEL | TCA | TCA | - | novel | Somatic | D57del | 0.021097 | NM_014849.5 | 709 | 694 | 15 |
| 848-LNmet | FCRL3 | 1787 | hg19 | chr1 | 157659707 | 157659707 | Missense_Mutation | SNP | C | C | T | novel | Somatic | G564E | 0.043643 | NM_052939.4 | 525 | 502 | 23 |
| 848-LNmet | FCRL3 | 1787 | hg19 | chr1 | 157667089 | 157667089 | Missense_Mutation | SNP | A | A | G | novel | Somatic | F229L | 0.071429 | NM_052939.4 | 798 | 741 | 57 |
| 848-LNmet | OR6Y1 | 1794 | hg19 | chr1 | 158517290 | 158517290 | Missense_Mutation | SNP | C | C | T | novel | Somatic | M202I | 0.061776 | NM_001005189.1 | 517 | 485 | 32 |
| 848-LNmet | CFHR3 | 2086 | hg19 | chr1 | 196748468 | 196748468 | Missense_Mutation | SNP | C | C | T | novel | Somatic | P79S | 0.080597 | NM_021023.5 | 334 | 307 | 27 |
| 848-LNmet | LAX1 | 2139 | hg19 | chr1 | 203743576 | 203743576 | Missense_Mutation | SNP | G | G | A | novel | Somatic | E322K | 0.089692 | NM_017773.4 | 747 | 680 | 67 |
| 848-LNmet | SOX13 | 267 | hg19 | chr1 | 204093869 | 204093869 | Missense_Mutation | SNP | G | G | T | novel | Somatic | K492N | 0.194566 | NM_005686.3 | 1141 | 919 | 222 |
| 848-LNmet | NFASC | 2148 | hg19 | chr1 | 204981927 | 204981927 | Nonsense_Mutation | SNP | C | C | T | novel | Somatic | R1142Ter | 0.045673 | NM_001005388.2 | 415 | 396 | 19 |
| 848-LNmet | LPGAT1 | 275 | hg19 | chr1 | 212002509 | 212002509 | Nonsense_Mutation | SNP | G | G | A | novel | Somatic | Q44Ter | 0.067492 | NM_014873.3 | 889 | 829 | 60 |
| 848-LNmet | GUK1 | 2327 | hg19 | chr1 | 228328021 | 228328021 | Frame_Shift_Del | DEL | GGCCGGGCTGGCTGCGGCCGCCCTG | GGCCGGGCTGGCTGCGGCCGCCCTG | - | novel | Somatic | L9PfsTer14 | 0.034595 | NM_001242840.2 | 925 | 893 | 32 |
| 848-LNmet | PCNX2 | 2365 | hg19 | chr1 | 233161036 | 233161036 | Missense_Mutation | SNP | G | G | T | novel | Somatic | N1487K | 0.17176 | NM_014801.4 | 948 | 785 | 163 |
| 848-LNmet | MATN3 | 739 | hg19 | chr2 | 20212323 | 20212323 | In_Frame_Del | DEL | GCA | GCA | - | novel | Somatic | L23del | 0.027344 | NM_002381.5 | 511 | 497 | 14 |
| 848-LNmet | ADCY3 | 776 | hg19 | chr2 | 25141376 | 25141376 | Missense_Mutation | SNP | G | G | A | novel | Somatic | R161C | 0.076482 | NM_001320613.2 | 1046 | 966 | 80 |
| 848-LNmet | GAREM2 | 786 | hg19 | chr2 | 26407588 | 26407588 | Missense_Mutation | SNP | C | C | G | novel | Somatic | L291V | 0.163666 | NM_001168241.2 | 610 | 510 | 100 |
| 848-LNmet | BMP10 | 1112 | hg19 | chr2 | 69093172 | 69093172 | Missense_Mutation | SNP | C | C | G | novel | Somatic | S289T | 0.023861 | NM_014482.3 | 461 | 450 | 11 |
| 848-LNmet | AFF3 | 2 | hg19 | chr2 | 100170808 | 100170808 | Missense_Mutation | SNP | C | C | A | novel | Somatic | W1200L | 0.059487 | NM_001025108.2 | 974 | 916 | 58 |
| 848-LNmet | ST6GAL2 | 175 | hg19 | chr2 | 107460187 | 107460187 | Missense_Mutation | SNP | C | C | T | novel | Somatic | A83T | 0.19765 | NM_001322362.2 | 935 | 750 | 185 |
| 848-LNmet | CNTNAP5 | 192 | hg19 | chr2 | 125521709 | 125521709 | Nonsense_Mutation | SNP | C | C | T | novel | Somatic | R840Ter | 0.144543 | NM_001367498.1 | 339 | 290 | 49 |
| 848-LNmet | AMER3 | 1588 | hg19 | chr2 | 131519833 | 131519833 | Missense_Mutation | SNP | A | A | G | novel | Somatic | D63G | 0.130243 | NM_152698.3 | 453 | 394 | 59 |
| 848-LNmet | NEB | 218 | hg19 | chr2 | 152394755 | 152394755 | Missense_Mutation | SNP | G | G | A | novel | Somatic | R6966C | 0.111588 | NM_001271208.2 | 233 | 207 | 26 |
| 848-LNmet | TTN | 1955 | hg19 | chr2 | 179489290 | 179489290 | Missense_Mutation | SNP | A | A | G | novel | Somatic | V14906A | 0.218391 | NM_001267550.2 | 347 | 271 | 76 |
| 848-LNmet | TTN | 1955 | hg19 | chr2 | 179580313 | 179580313 | Missense_Mutation | SNP | G | G | T | novel | Somatic | H8610N | 0.236203 | NM_001267550.2 | 453 | 346 | 107 |
| 848-LNmet | NBEAL1 | 267 | hg19 | chr2 | 203922058 | 203922058 | Frame_Shift_Del | DEL | A | A | - | novel | Somatic | E136RfsTer14 | 0.02863 | NM_001114132.1 | 489 | 475 | 14 |
| 848-LNmet | ERBB4 | 34 | hg19 | chr2 | 212289014 | 212289014 | Nonsense_Mutation | SNP | C | C | T | novel | Somatic | W911Ter | 0.099448 | NM_005235.3 | 361 | 325 | 36 |
| 848-LNmet | PLCD4 | 2259 | hg19 | chr2 | 219500624 | 219500624 | Missense_Mutation | SNP | C | C | T | novel | Somatic | R668W | 0.104364 | NM_032726.4 | 527 | 472 | 55 |
| 848-LNmet | CUL3 | 2304 | hg19 | chr2 | 225339071 | 225339071 | Missense_Mutation | SNP | C | C | A | novel | Somatic | R739L | 0.077748 | NM_001257198.2 | 372 | 343 | 29 |
| 848-LNmet | KCNH8 | 91 | hg19 | chr3 | 19554522 | 19554522 | Missense_Mutation | SNP | G | G | A | novel | Somatic | E714K | 0.109023 | NM_144633.3 | 531 | 473 | 58 |
| 848-LNmet | CCR8 | 885 | hg19 | chr3 | 39374178 | 39374178 | Missense_Mutation | SNP | G | G | A | novel | Somatic | S119N | 0.11002 | NM_005201.4 | 508 | 452 | 56 |
| 848-LNmet | CSPG5 | 948 | hg19 | chr3 | 47618698 | 47618698 | Missense_Mutation | SNP | T | T | G | novel | Somatic | D273A | 0.085299 | NM_006574.4 | 551 | 504 | 47 |
| 848-LNmet | KLHDC8B | 960 | hg19 | chr3 | 49212285 | 49212285 | Missense_Mutation | SNP | G | G | A | novel | Somatic | A218T | 0.118201 | NM_173546.3 | 955 | 842 | 113 |
| 848-LNmet | ROBO2 | 18 | hg19 | chr3 | 77657038 | 77657038 | Frame_Shift_Del | DEL | C | C | - | novel | Somatic | P1082QfsTer95 | 0.026178 | NM_001290040.2 | 763 | 743 | 20 |
| 848-LNmet | EPHA3 | 158 | hg19 | chr3 | 89521723 | 89521723 | Missense_Mutation | SNP | G | G | A | novel | Somatic | G934S | 0.235405 | NM_005233.6 | 531 | 406 | 125 |
| 848-LNmet | SLC9C1 | 179 | hg19 | chr3 | 111999556 | 111999556 | Missense_Mutation | SNP | C | C | T | novel | Somatic | E55K | 0.044408 | NM_183061.3 | 608 | 581 | 27 |
| 848-LNmet | TRIM42 | 1656 | hg19 | chr3 | 140401662 | 140401662 | Missense_Mutation | SNP | G | G | A | novel | Somatic | G234R | 0.090472 | NM_152616.5 | 1039 | 945 | 94 |
| 848-LNmet | GK5 | 1667 | hg19 | chr3 | 141891222 | 141891222 | Missense_Mutation | SNP | C | C | G | novel | Somatic | R407P | 0.037333 | NM_001039547.3 | 373 | 359 | 14 |
| 848-LNmet | GK5 | 1667 | hg19 | chr3 | 141934501 | 141934501 | Missense_Mutation | SNP | C | C | T | novel | Somatic | E61K | 0.091434 | NM_001039547.3 | 1039 | 944 | 95 |
| 848-LNmet | ANKUB1 | 1725 | hg19 | chr3 | 149485876 | 149485876 | Missense_Mutation | SNP | C | C | A | novel | Somatic | Q191H | 0.068528 | NM_001144960.3 | 393 | 366 | 27 |
| 848-LNmet | KCNAB1 | 27 | hg19 | chr3 | 156232230 | 156232230 | Missense_Mutation | SNP | G | G | A | novel | Somatic | E246K | 0.094595 | NM_172160.2 | 370 | 335 | 35 |
| 848-LNmet | B3GNT5 | 247 | hg19 | chr3 | 182988544 | 182988544 | Missense_Mutation | SNP | A | A | G | novel | Somatic | M320V | 0.079128 | NM_032047.5 | 872 | 803 | 69 |
| 848-LNmet | VWA5B2 | 1988 | hg19 | chr3 | 183954152 | 183954152 | Missense_Mutation | SNP | G | G | A | novel | Somatic | E412K | 0.06383 | NM_138345.3 | 705 | 660 | 45 |
| 848-LNmet | IL1RAP | 254 | hg19 | chr3 | 190321919 | 190321919 | Missense_Mutation | SNP | C | C | A | novel | Somatic | R23S | 0.071885 | NM_001167931.2 | 626 | 581 | 45 |
| 848-LNmet | LMLN | 2093 | hg19 | chr3 | 197729927 | 197729927 | Frame_Shift_Del | DEL | G | G | - | novel | Somatic | G426AfsTer27 | 0.047945 | NM_001136049.2 | 584 | 556 | 28 |
| 848-LNmet | AFAP1 | 80 | hg19 | chr4 | 7795457 | 7795457 | Missense_Mutation | SNP | C | C | A | novel | Somatic | D455Y | 0.113208 | NM_001134647.1 | 477 | 423 | 54 |
| 848-LNmet | LIMCH1 | 112 | hg19 | chr4 | 41634839 | 41634839 | Missense_Mutation | SNP | G | G | C | novel | Somatic | E559Q | 0.113208 | NM_001330672.2 | 530 | 470 | 60 |
| 848-LNmet | PTPN13 | 156 | hg19 | chr4 | 87728733 | 87728733 | Missense_Mutation | SNP | C | C | T | novel | Somatic | L2261F | 0.033505 | NM_080685.2 | 388 | 375 | 13 |
| 848-LNmet | NAP1L5 | 1268 | hg19 | chr4 | 89618728 | 89618728 | Missense_Mutation | SNP | C | C | T | novel | Somatic | E60K | 0.065279 | NM_153757.4 | 1055 | 986 | 69 |
| 848-LNmet | POU4F2 | 1710 | hg19 | chr4 | 147560504 | 147560504 | Missense_Mutation | SNP | G | G | T | novel | Somatic | S71I | 0.047739 | NM_004575.3 | 397 | 378 | 19 |
| 848-LNmet | DDX60 | 234 | hg19 | chr4 | 169196671 | 169196671 | Intron | SNP | G | G | C | novel | Somatic |  | 0.124031 | NM_017631.6 | 258 | 226 | 32 |
| 848-LNmet | TRIML2 | 2027 | hg19 | chr4 | 189012729 | 189012729 | Missense_Mutation | SNP | C | C | T | novel | Somatic | G396D | 0.207071 | NM_001303419.1 | 394 | 312 | 82 |
| 848-LNmet | PCDHAC2 | 1655 | hg19 | chr5 | 140347516 | 140347516 | Missense_Mutation | SNP | G | G | T | novel | Somatic | V389F | 0.280072 | NM_018899.6 | 557 | 401 | 156 |
| 848-LNmet | PCDHB11 | 1657 | hg19 | chr5 | 140579789 | 140579789 | Frame_Shift_Ins | INS | - | - | T | novel | Somatic | G149WfsTer33 | 0.244211 | NM_018931.3 | 470 | 354 | 116 |
| 848-LNmet | PCDHGA8 | 1659 | hg19 | chr5 | 140773002 | 140773002 | Missense_Mutation | SNP | C | C | T | novel | Somatic | H208Y | 0.03128 | NM_032088.1 | 1055 | 1022 | 33 |
| 848-LNmet | KCTD16 | 26 | hg19 | chr5 | 143853531 | 143853531 | Frame_Shift_Del | DEL | A | A | - | novel | Somatic | A384LfsTer20 | 0.02214 | NM_020768.4 | 812 | 794 | 18 |
| 848-LNmet | NMUR2 | 217 | hg19 | chr5 | 151784001 | 151784001 | Missense_Mutation | SNP | T | T | C | novel | Somatic | Y225C | 0.121693 | NM_020167.4 | 378 | 332 | 46 |
| 848-LNmet | DRD1 | 1919 | hg19 | chr5 | 174869394 | 174869394 | Missense_Mutation | SNP | G | G | A | novel | Somatic | H237Y | 0.070229 | NM_000794.5 | 654 | 608 | 46 |
| 848-LNmet | CPLX2 | 1922 | hg19 | chr5 | 175306051 | 175306051 | Missense_Mutation | SNP | G | G | A | novel | Somatic | E58K | 0.117521 | NM_001008220.2 | 936 | 826 | 110 |
| 848-LNmet | HUS1B | 590 | hg19 | chr6 | 656542 | 656542 | Missense_Mutation | SNP | G | G | A | novel | Somatic | R135W | 0.103208 | NM_148959.3 | 716 | 642 | 74 |
| 848-LNmet | PGBD1 | 800 | hg19 | chr6 | 28269624 | 28269624 | Missense_Mutation | SNP | C | C | A | novel | Somatic | H665N | 0.040741 | NM_001184743.1 | 270 | 259 | 11 |
| 848-LNmet | C6orf89 | 866 | hg19 | chr6 | 36870003 | 36870003 | Missense_Mutation | SNP | G | G | A | novel | Somatic | A73T | 0.204301 | NM_152734.4 | 371 | 295 | 76 |
| 848-LNmet | DEFB113 | 965 | hg19 | chr6 | 49936573 | 49936573 | Missense_Mutation | SNP | C | C | G | novel | Somatic | Q22H | 0.109049 | NM_001037729.1 | 430 | 383 | 47 |
| 848-LNmet | FBXO30 | 1699 | hg19 | chr6 | 146127163 | 146127163 | Nonsense_Mutation | SNP | G | G | A | novel | Somatic | Q127Ter | 0.087814 | NM_032145.5 | 557 | 508 | 49 |
| 848-LNmet | MTRF1L | 1754 | hg19 | chr6 | 153319684 | 153319684 | Splice_Site | SNP | C | C | A | novel | Somatic |  | 0.100932 | NM_019041.7 | 641 | 576 | 65 |
| 848-LNmet | IGF2R | 28 | hg19 | chr6 | 160485853 | 160485853 | Missense_Mutation | SNP | G | G | C | novel | Somatic | E1345D | 0.054455 | NM_000876.3 | 404 | 382 | 22 |
| 848-LNmet | AGPAT4 | 227 | hg19 | chr6 | 161560504 | 161560504 | Missense_Mutation | SNP | C | C | A | novel | Somatic | R331M | 0.129902 | NM_020133.3 | 816 | 710 | 106 |
| 848-LNmet | ZNF735 | 1070 | hg19 | chr7 | 63674444 | 63674444 | Missense_Mutation | DNP | CC | CC | AA | novel | Somatic | P74N | 0.029369 | NM_001159524.1 | 679 | 659 | 20 |
| 848-LNmet | COL1A2 | 1302 | hg19 | chr7 | 94053715 | 94053715 | Missense_Mutation | SNP | C | C | T | novel | Somatic | S878L | 0.092081 | NM_000089.4 | 543 | 493 | 50 |
| 848-LNmet | SLC25A13 | 164 | hg19 | chr7 | 95818605 | 95818605 | Splice_Site | SNP | C | C | T | novel | Somatic |  | 0.077419 | NM_014251.3 | 310 | 286 | 24 |
| 848-LNmet | KMT2C | 27 | hg19 | chr7 | 151874147 | 151874147 | Frame_Shift_Ins | INS | - | - | T | novel | Somatic | E2798GfsTer11 | 0.02716 | NM_170606.3 | 403 | 392 | 11 |
| 848-LNmet | CSMD1 | 9 | hg19 | chr8 | 3081326 | 3081326 | Missense_Mutation | SNP | G | G | A | novel | Somatic | P1470L | 0.269565 | NM_033225.6 | 460 | 336 | 124 |
| 848-LNmet | MCPH1 | 9 | hg19 | chr8 | 6289099 | 6289099 | Frame_Shift_Del | DEL | A | A | - | novel | Somatic | K107NfsTer39 | 0.027174 | NM_024596.5 | 552 | 537 | 15 |
| 848-LNmet | LONRF1 | 10 | hg19 | chr8 | 12595494 | 12595494 | Intron | SNP | C | C | T | novel | Somatic |  | 0.046512 | NM_152271.5 | 387 | 369 | 18 |
| 848-LNmet | KAT6A | 112 | hg19 | chr8 | 41791261 | 41791261 | Frame_Shift_Ins | INS | - | - | CTGG | novel | Somatic | S1493PfsTer15 | 0.090336 | NM_006766.5 | 946 | 860 | 86 |
| 848-LNmet | YTHDF3 | 134 | hg19 | chr8 | 64099908 | 64099908 | Missense_Mutation | SNP | C | C | T | novel | Somatic | R446C | 0.0721 | NM_152758.6 | 637 | 591 | 46 |
| 848-LNmet | CSMD3 | 22 | hg19 | chr8 | 113599378 | 113599378 | Missense_Mutation | SNP | T | T | C | novel | Somatic | I1268V | 0.070093 | NM_198123.2 | 428 | 398 | 30 |
| 848-LNmet | DEPTOR | 188 | hg19 | chr8 | 120940713 | 120940713 | Missense_Mutation | SNP | G | G | A | novel | Somatic | A66T | 0.098039 | NM_022783.4 | 764 | 689 | 75 |
| 848-LNmet | ATAD2 | 191 | hg19 | chr8 | 124384893 | 124384893 | Frame_Shift_Del | DEL | T | T | - | novel | Somatic | E119KfsTer8 | 0.023058 | NM_014109.4 | 823 | 804 | 19 |
| 848-LNmet | PLEC | 1691 | hg19 | chr8 | 144994931 | 144994931 | Missense_Mutation | SNP | G | G | C | novel | Somatic | L3157V | 0.072479 | NM_201380.4 | 947 | 878 | 69 |
| 848-LNmet | ZNF883 | 1468 | hg19 | chr9 | 115759660 | 115759660 | Missense_Mutation | SNP | G | G | C | novel | Somatic | Q294E | 0.050251 | NM_001101338.1 | 394 | 374 | 20 |
| 848-LNmet | PAPPA | 186 | hg19 | chr9 | 118997469 | 118997469 | Missense_Mutation | SNP | C | C | T | novel | Somatic | P762L | 0.142066 | NM_002581.5 | 542 | 465 | 77 |
| 848-LNmet | NELFB | 1654 | hg19 | chr9 | 140149972 | 140149972 | Missense_Mutation | SNP | G | G | A | novel | Somatic | G52E | 0.253799 | NM_015456.5 | 658 | 491 | 167 |
| 848-LNmet | CUBN | 89 | hg19 | chr10 | 17107611 | 17107611 | Missense_Mutation | SNP | G | G | A | novel | Somatic | S1012F | 0.098039 | NM_001081.4 | 459 | 414 | 45 |
| 848-LNmet | ZNF33B | 114 | hg19 | chr10 | 43127384 | 43127384 | Missense_Mutation | SNP | G | G | T | novel | Somatic | S81R | 0.079872 | NM_006955.3 | 313 | 288 | 25 |
| 848-LNmet | KCNMA1 | 1190 | hg19 | chr10 | 78844467 | 78844467 | Missense_Mutation | SNP | G | G | A | novel | Somatic | A484V | 0.112381 | NM_001161352.2 | 521 | 462 | 59 |
| 848-LNmet | ATRNL1 | 184 | hg19 | chr10 | 116853585 | 116853585 | In_Frame_Del | DEL | GGC | GGC | - | novel | Somatic | G31del | 0.03125 | NM_207303.4 | 350 | 339 | 11 |
| 848-LNmet | USH1C | 89 | hg19 | chr11 | 17542429 | 17542429 | Missense_Mutation | SNP | G | G | A | novel | Somatic | R400C | 0.060484 | NM_153676.4 | 248 | 233 | 15 |
| 848-LNmet | OR4C15 | 1007 | hg19 | chr11 | 55322321 | 55322321 | Missense_Mutation | SNP | G | G | T | novel | Somatic | C126F | 0.095331 | NM_001001920.2 | 514 | 465 | 49 |
| 848-LNmet | VPS37C | 1049 | hg19 | chr11 | 60899539 | 60899539 | Missense_Mutation | SNP | C | C | T | novel | Somatic | G274E | 0.089918 | NM_017966.5 | 733 | 667 | 66 |
| 848-LNmet | NXF1 | 1062 | hg19 | chr11 | 62566016 | 62566016 | Missense_Mutation | SNP | G | G | A | novel | Somatic | R350C | 0.020921 | NM_006362.5 | 477 | 467 | 10 |
| 848-LNmet | ARHGAP20 | 178 | hg19 | chr11 | 110485337 | 110485337 | Missense_Mutation | SNP | G | G | A | novel | Somatic | P193L | 0.113577 | NM_020809.3 | 765 | 678 | 87 |
| 848-LNmet | CD163 | 643 | hg19 | chr12 | 7639511 | 7639511 | Nonsense_Mutation | SNP | C | C | A | novel | Somatic | E708Ter | 0.226981 | NM_004244.5 | 467 | 361 | 106 |
| 848-LNmet | KLRF1 | 661 | hg19 | chr12 | 9997090 | 9997090 | In_Frame_Ins | INS | - | - | GCA | novel | Somatic | S223dup | 0.101482 | NM_016523.3 | 877 | 788 | 89 |
| 848-LNmet | LOC255308 | Unknown | hg19 | chr12 | 10659547 | 10659547 | Missense_Mutation | SNP | G | G | A | novel | Somatic | R349Q | 0.189112 | NM_001357734.1 | 698 | 566 | 132 |
| 848-LNmet | ARID2 | 14 | hg19 | chr12 | 46245781 | 46245781 | Missense_Mutation | SNP | G | G | A | novel | Somatic | S1292N | 0.131213 | NM_152641.4 | 503 | 437 | 66 |
| 848-LNmet | SMARCC2 | 1016 | hg19 | chr12 | 56575296 | 56575296 | Missense_Mutation | SNP | G | G | C | novel | Somatic | P309R | 0.096244 | NM_001330288.2 | 426 | 385 | 41 |
| 848-LNmet | LYZ | 1117 | hg19 | chr12 | 69746053 | 69746053 | Missense_Mutation | SNP | C | C | T | novel | Somatic | R119C | 0.066667 | NM_000239.3 | 525 | 490 | 35 |
| 848-LNmet | NAV3 | 147 | hg19 | chr12 | 78400658 | 78400658 | Missense_Mutation | SNP | C | C | T | novel | Somatic | A447V | 0.102362 | NM_001024383.2 | 506 | 454 | 52 |
| 848-LNmet | SLC6A15 | 1235 | hg19 | chr12 | 85266555 | 85266555 | Missense_Mutation | SNP | C | C | T | novel | Somatic | M376I | 0.123862 | NM_182767.6 | 549 | 481 | 68 |
| 848-LNmet | ANKS1B | 167 | hg19 | chr12 | 99837530 | 99837530 | Missense_Mutation | SNP | G | G | A | novel | Somatic | S499L | 0.087302 | NM_001352186.1 | 504 | 460 | 44 |
| 848-LNmet | KLF5 | 1146 | hg19 | chr13 | 73649905 | 73649905 | Missense_Mutation | SNP | G | G | C | novel | Somatic | E419Q | 0.021258 | NM_001730.5 | 1126 | 1102 | 24 |
| 848-LNmet | FANCM | 116 | hg19 | chr14 | 45628301 | 45628301 | Missense_Mutation | SNP | G | G | A | novel | Somatic | E467K | 0.078431 | NM_020937.4 | 255 | 235 | 20 |
| 848-LNmet | C15orf39 | 2 | hg19 | chr15 | 75500089 | 75500089 | Nonsense_Mutation | SNP | C | C | A | novel | Somatic | S567Ter | 0.190635 | NM_015492.5 | 598 | 484 | 114 |
| 848-LNmet | ARPIN | 1275 | hg19 | chr15 | 90451604 | 90451604 | Missense_Mutation | SNP | T | T | C | novel | Somatic | H70R | 0.210361 | NM_182616.4 | 637 | 503 | 134 |
| 848-LNmet | ECI1 | 602 | hg19 | chr16 | 2290078 | 2290078 | Missense_Mutation | SNP | C | C | T | novel | Somatic | D271N | 0.105072 | NM_001919.4 | 1654 | 1480 | 174 |
| 848-LNmet | ABCC1 | 88 | hg19 | chr16 | 16200707 | 16200707 | Missense_Mutation | SNP | G | G | A | novel | Somatic | A950T | 0.111111 | NM_004996.4 | 603 | 536 | 67 |
| 848-LNmet | ZNF646 | 822 | hg19 | chr16 | 31090337 | 31090337 | Missense_Mutation | SNP | C | C | T | novel | Somatic | L898F | 0.093525 | NM_014699.4 | 1390 | 1260 | 130 |
| 848-LNmet | ZNF267 | 828 | hg19 | chr16 | 31926927 | 31926927 | Missense_Mutation | SNP | C | C | A | novel | Somatic | Q453K | 0.092369 | NM_003414.6 | 498 | 452 | 46 |
| 848-LNmet | IRX5 | 1004 | hg19 | chr16 | 54967457 | 54967457 | Missense_Mutation | SNP | G | G | T | novel | Somatic | R375L | 0.230014 | NM_005853.6 | 712 | 548 | 164 |
| 848-LNmet | LPCAT2 | 15 | hg19 | chr16 | 55563831 | 55563831 | Missense_Mutation | SNP | G | G | T | novel | Somatic | R205L | 0.086643 | NM_017839.5 | 553 | 505 | 48 |
| 848-LNmet | CES1 | 1011 | hg19 | chr16 | 55844469 | 55844469 | Missense_Mutation | SNP | C | C | T | novel | Somatic | M426I | 0.134615 | NM_001025195.2 | 884 | 765 | 119 |
| 848-LNmet | ZNF319 | 1027 | hg19 | chr16 | 58031041 | 58031041 | Missense_Mutation | SNP | C | C | T | novel | Somatic | E377K | 0.082718 | NM_020807.2 | 677 | 621 | 56 |
| 848-LNmet | TP53 | 642 | hg19 | chr17 | 7577067 | 7577067 | Nonsense_Mutation | SNP | T | T | A | novel | Somatic | K291Ter | 0.182628 | NM_000546.5 | 1347 | 1101 | 246 |
| 848-LNmet | PIK3R6 | 651 | hg19 | chr17 | 8726793 | 8726793 | Silent | SNP | G | G | T | novel | Somatic | R513= | 0.061086 | NM_001010855.4 | 442 | 415 | 27 |
| 848-LNmet | EVPLL | 724 | hg19 | chr17 | 18285955 | 18285955 | Splice_Region | SNP | G | G | A | novel | Somatic |  | 0.105263 | NM_001145127.2 | 285 | 255 | 30 |
| 848-LNmet | NLE1 | 840 | hg19 | chr17 | 33464561 | 33464561 | Splice_Site | SNP | C | C | T | novel | Somatic |  | 0.06986 | NM_018096.5 | 501 | 466 | 35 |
| 848-LNmet | KRT14 | 888 | hg19 | chr17 | 39742678 | 39742678 | Nonsense_Mutation | SNP | C | C | A | novel | Somatic | E137Ter | 0.264151 | NM_000526.5 | 1484 | 1092 | 392 |
| 848-LNmet | BRCA1 | 899 | hg19 | chr17 | 41276095 | 41276095 | Missense_Mutation | SNP | G | G | A | novel | Somatic | R7C | 0.022853 | NM_007294.4 | 1269 | 1240 | 29 |
| 848-LNmet | DBF4B | 911 | hg19 | chr17 | 42809600 | 42809600 | Missense_Mutation | SNP | C | C | T | novel | Somatic | R175C | 0.072359 | NM_145663.3 | 691 | 641 | 50 |
| 848-LNmet | ASXL3 | 102 | hg19 | chr18 | 31326113 | 31326113 | Missense_Mutation | SNP | G | G | A | novel | Somatic | V2101I | 0.069869 | NM_030632.3 | 458 | 426 | 32 |
| 848-LNmet | CTDP1 | 146 | hg19 | chr18 | 77513770 | 77513770 | Missense_Mutation | SNP | G | G | C | novel | Somatic | E956Q | 0.077199 | NM_004715.4 | 556 | 513 | 43 |
| 848-LNmet | TLE2 | 75 | hg19 | chr19 | 3000686 | 3000686 | Missense_Mutation | SNP | G | G | T | novel | Somatic | L695I | 0.081744 | NM_003260.5 | 734 | 674 | 60 |
| 848-LNmet | CD70 | 635 | hg19 | chr19 | 6586080 | 6586080 | Missense_Mutation | SNP | G | G | T | novel | Somatic | S178Y | 0.068996 | NM_001252.5 | 1142 | 1063 | 79 |
| 848-LNmet | SH2D3A | 636 | hg19 | chr19 | 6753509 | 6753509 | Missense_Mutation | SNP | C | C | T | novel | Somatic | D510N | 0.035619 | NM_005490.3 | 1123 | 1083 | 40 |
| 848-LNmet | ZNF557 | 79 | hg19 | chr19 | 7083388 | 7083388 | Missense_Mutation | SNP | C | C | G | novel | Somatic | S309W | 0.247956 | NM_024341.3 | 731 | 549 | 182 |
| 848-LNmet | ZNF440 | 84 | hg19 | chr19 | 11942890 | 11942890 | Missense_Mutation | SNP | G | G | A | novel | Somatic | R300H | 0.084548 | NM_152357.3 | 685 | 627 | 58 |
| 848-LNmet | RFX1 | 692 | hg19 | chr19 | 14077237 | 14077237 | Missense_Mutation | SNP | C | C | A | novel | Somatic | A653S | 0.092127 | NM_002918.5 | 596 | 541 | 55 |
| 848-LNmet | OR10H1 | 706 | hg19 | chr19 | 15918043 | 15918043 | Missense_Mutation | SNP | G | G | C | novel | Somatic | L269V | 0.073452 | NM_013940.3 | 952 | 882 | 70 |
| 848-LNmet | NPHS1 | 862 | hg19 | chr19 | 36335301 | 36335301 | Missense_Mutation | SNP | G | G | A | novel | Somatic | A664V | 0.24481 | NM_004646.3 | 1155 | 872 | 283 |
| 848-LNmet | ZNF529 | 867 | hg19 | chr19 | 37047043 | 37047043 | Missense_Mutation | SNP | A | A | G | novel | Somatic | F24S | 0.115124 | NM_020951.5 | 443 | 392 | 51 |
| 848-LNmet | ZNF585A | 872 | hg19 | chr19 | 37642547 | 37642547 | Missense_Mutation | SNP | C | C | A | novel | Somatic | G752W | 0.080183 | NM_001288800.2 | 873 | 803 | 70 |
| 848-LNmet | ZNF227 | 926 | hg19 | chr19 | 44739011 | 44739011 | Missense_Mutation | SNP | C | C | G | novel | Somatic | S143C | 0.069549 | NM_182490.3 | 531 | 494 | 37 |
| 848-LNmet | RELB | 932 | hg19 | chr19 | 45528957 | 45528957 | Missense_Mutation | SNP | G | G | A | novel | Somatic | R283Q | 0.139966 | NM_006509.4 | 593 | 510 | 83 |
| 848-LNmet | PPP1R13L | 935 | hg19 | chr19 | 45898771 | 45898771 | Missense_Mutation | SNP | G | G | A | novel | Somatic | P426L | 0.067894 | NM_006663.4 | 869 | 810 | 59 |
| 848-LNmet | PPP1R13L | 935 | hg19 | chr19 | 45898781 | 45898781 | Nonsense_Mutation | SNP | G | G | A | novel | Somatic | Q423Ter | 0.067757 | NM_006663.4 | 855 | 797 | 58 |
| 848-LNmet | DHX34 | 950 | hg19 | chr19 | 47858386 | 47858386 | Missense_Mutation | SNP | C | C | T | novel | Somatic | R266W | 0.08605 | NM_014681.6 | 766 | 700 | 66 |
| 848-LNmet | DPRX | 998 | hg19 | chr19 | 54139917 | 54139917 | Missense_Mutation | SNP | A | A | C | novel | Somatic | E84A | 0.062102 | NM_001012728.1 | 627 | 588 | 39 |
| 848-LNmet | NLRP12 | 999 | hg19 | chr19 | 54313035 | 54313035 | Missense_Mutation | SNP | G | G | C | novel | Somatic | I626M | 0.044248 | NM_144687.3 | 676 | 646 | 30 |
| 848-LNmet | DEFB119 | 813 | hg19 | chr20 | 29965166 | 29965166 | Nonsense_Mutation | SNP | G | G | T | novel | Somatic | Y46Ter | 0.082803 | NM_153289.4 | 469 | 430 | 39 |
| 848-LNmet | XKR7 | 818 | hg19 | chr20 | 30556280 | 30556280 | Missense_Mutation | SNP | C | C | G | novel | Somatic | S101W | 0.110754 | NM_001011718.2 | 623 | 554 | 69 |
| 848-LNmet | TCEA2 | 1063 | hg19 | chr20 | 62701763 | 62701763 | Missense_Mutation | SNP | C | C | T | novel | Somatic | S223L | 0.057554 | NM_003195.6 | 831 | 783 | 48 |
| 848-LNmet | DSCAM | 1 | hg19 | chr21 | 41668063 | 41668063 | Missense_Mutation | SNP | C | C | T | novel | Somatic | G701R | 0.166172 | NM_001389.5 | 337 | 281 | 56 |
| 848-LNmet | PKDREJ | 940 | hg19 | chr22 | 46656184 | 46656184 | Missense_Mutation | SNP | C | C | A | novel | Somatic | L1012F | 0.084859 | NM_006071.2 | 601 | 550 | 51 |
| 848-LNmet | SHROOM2 | 660 | hg19 | chrX | 9900238 | 9900238 | Missense_Mutation | SNP | A | A | G | novel | Somatic | E972G | 0.127753 | NM_001649.4 | 679 | 592 | 87 |
| 848-LNmet | HCCS | 83 | hg19 | chrX | 11139860 | 11139860 | Missense_Mutation | SNP | G | G | T | novel | Somatic | R246L | 0.12218 | NM_005333.5 | 532 | 467 | 65 |
| 848-LNmet | CASK | 112 | hg19 | chrX | 41481918 | 41481918 | Intron | SNP | C | C | A | novel | Somatic |  | 0.057377 | NM_001367721.1 | 366 | 345 | 21 |
| 848-LNmet | ZNF157 | 945 | hg19 | chrX | 47271884 | 47271884 | Nonsense_Mutation | SNP | G | G | T | novel | Somatic | G138Ter | 0.071048 | NM_003446.3 | 563 | 523 | 40 |
| 848-LNmet | PCDH19 | 20 | hg19 | chrX | 99663560 | 99663560 | In_Frame_Del | DEL | CAG | CAG | - | novel | Somatic | L12del | 0.022263 | NM_001184880.2 | 539 | 527 | 12 |
| 848-LNmet | LRCH2 | 182 | hg19 | chrX | 114347878 | 114347878 | Missense_Mutation | SNP | A | A | T | novel | Somatic | H733Q | 0.228288 | NM_020871.4 | 401 | 309 | 92 |
| 848-LNmet | AGTR2 | 1464 | hg19 | chrX | 115303867 | 115303867 | Missense_Mutation | SNP | T | T | C | novel | Somatic | F112L | 0.11976 | NM_000686.5 | 333 | 293 | 40 |
| 848-LNmet | GLUD2 | 1501 | hg19 | chrX | 120182895 | 120182895 | Missense_Mutation | SNP | C | C | T | novel | Somatic | R453C | 0.072886 | NM_012084.4 | 686 | 636 | 50 |
| 879-PTsup | IFI44L | 1188 | hg19 | chr1 | 79093715 | 79093715 | Missense_Mutation | SNP | G | G | C | novel | Somatic | D39H | 0.551948 | NM_006820.4 | 154 | 69 | 85 |
| 879-PTsup | SCAMP3 | 1769 | hg19 | chr1 | 155227135 | 155227135 | Missense_Mutation | SNP | G | G | T | novel | Somatic | F240L | 0.24375 | NM_005698.4 | 160 | 121 | 39 |
| 879-PTsup | MROH9 | 29 | hg19 | chr1 | 170993671 | 170993671 | Missense_Mutation | SNP | G | G | A | novel | Somatic | D682N | 0.128302 | NM_001163629.1 | 265 | 231 | 34 |
| 879-PTsup | TRMT1L | 1997 | hg19 | chr1 | 185094192 | 185094192 | Missense_Mutation | SNP | G | G | A | novel | Somatic | S548F | 0.326667 | NM_030934.5 | 300 | 202 | 98 |
| 879-PTsup | IPO9 | 265 | hg19 | chr1 | 201824313 | 201824313 | Splice_Region | SNP | A | A | G | novel | Somatic |  | 0.269231 | NM_018085.5 | 130 | 95 | 35 |
| 879-PTsup | DISP1 | 285 | hg19 | chr1 | 223178584 | 223178584 | Missense_Mutation | SNP | A | A | G | novel | Somatic | Y1282C | 0.410377 | NM_032890.5 | 212 | 125 | 87 |
| 879-PTsup | NCAPH | 1325 | hg19 | chr2 | 97019070 | 97019070 | Missense_Mutation | SNP | C | C | T | novel | Somatic | R313C | 0.709677 | NM_015341.5 | 278 | 80 | 198 |
| 879-PTsup | NCKAP5 | 0 | hg19 | chr2 | 133541865 | 133541865 | Missense_Mutation | SNP | G | G | T | novel | Somatic | A840D | 0.100402 | NM_207363.3 | 248 | 223 | 25 |
| 879-PTsup | SPOPL | 205 | hg19 | chr2 | 139316665 | 139316665 | Missense_Mutation | SNP | G | G | T | novel | Somatic | R185L | 0.648889 | NM_001001664.3 | 225 | 79 | 146 |
| 879-PTsup | UBR3 | 29 | hg19 | chr2 | 170761755 | 170761755 | Missense_Mutation | SNP | G | G | A | novel | Somatic | D533N | 0.28169 | NM_172070.4 | 284 | 204 | 80 |
| 879-PTsup | FSIP2 | 31 | hg19 | chr2 | 186670324 | 186670324 | Missense_Mutation | SNP | A | A | G | novel | Somatic | S5431G | 0.656522 | NM_173651.4 | 229 | 78 | 151 |
| 879-PTsup | XYLB | 877 | hg19 | chr3 | 38390124 | 38390124 | Splice_Site | INS | - | - | T | novel | Somatic |  | 0.396341 | NM_005108.4 | 163 | 98 | 65 |
| 879-PTsup | BST1 | 10 | hg19 | chr4 | 15717358 | 15717358 | Missense_Mutation | SNP | C | C | T | novel | Somatic | L214F | 0.234899 | NM_004334.3 | 149 | 114 | 35 |
| 879-PTsup | KLF3 | 109 | hg19 | chr4 | 38696497 | 38696497 | Missense_Mutation | SNP | C | C | T | novel | Somatic | H276Y | 0.547059 | NM_016531.6 | 170 | 77 | 93 |
| 879-PTsup | FIP1L1 | 124 | hg19 | chr4 | 54257211 | 54257211 | Missense_Mutation | SNP | A | A | T | novel | Somatic | N181Y | 0.540323 | NM_030917.4 | 123 | 56 | 67 |
| 879-PTsup | SHROOM3 | 18 | hg19 | chr4 | 77662895 | 77662895 | Missense_Mutation | SNP | G | G | A | novel | Somatic | S1190N | 0.539763 | NM_020859.4 | 586 | 267 | 319 |
| 879-PTsup | SLC6A18 | 594 | hg19 | chr5 | 1225687 | 1225687 | Missense_Mutation | SNP | C | C | T | novel | Somatic | T32I | 0.076433 | NM_182632.3 | 178 | 154 | 24 |
| 879-PTsup | TERT | 594 | hg19 | chr5 | 1282667 | 1282667 | Frame_Shift_Del | DEL | ATCAGCCA | ATCAGCCA | - | novel | Somatic | W547EfsTer79 | 0.242105 | NM_198253.3 | 283 | 214 | 69 |
| 879-PTsup | NIM1K | 114 | hg19 | chr5 | 43280665 | 43280665 | Nonsense_Mutation | SNP | C | C | G | novel | Somatic | S382Ter | 0.345455 | NM_153361.4 | 219 | 143 | 76 |
| 879-PTsup | HARS | 1653 | hg19 | chr5 | 140059383 | 140059383 | Missense_Mutation | SNP | T | T | C | novel | Somatic | Y129C | 0.428571 | NM_002109.6 | 105 | 60 | 45 |
| 879-PTsup | TIAM2 | 1771 | hg19 | chr6 | 155575684 | 155575684 | Missense_Mutation | SNP | G | G | T | novel | Somatic | R1482L | 0.369369 | NM_012454.3 | 331 | 208 | 123 |
| 879-PTsup | FNDC1 | 225 | hg19 | chr6 | 159687239 | 159687239 | Missense_Mutation | SNP | C | C | T | novel | Somatic | S1803L | 0.090476 | NM_032532.3 | 210 | 191 | 19 |
| 879-PTsup | SUN1 | 591 | hg19 | chr7 | 912913 | 912913 | Frame_Shift_Del | DEL | ATCCTGA | ATCCTGA | - | novel | Somatic | H906RfsTer67 | 0.064356 | NM_001367651.1 | 202 | 189 | 13 |
| 879-PTsup | ABCB1 | 156 | hg19 | chr7 | 87175277 | 87175277 | Missense_Mutation | SNP | C | C | T | novel | Somatic | V667I | 0.146119 | NM_001348945.1 | 437 | 373 | 64 |
| 879-PTsup | HTR5A | 1766 | hg19 | chr7 | 154862965 | 154862965 | Missense_Mutation | SNP | C | C | T | novel | Somatic | A119V | 0.205405 | NM_024012.3 | 1106 | 878 | 228 |
| 879-PTsup | DOCK5 | 776 | hg19 | chr8 | 25181459 | 25181459 | Missense_Mutation | SNP | G | G | T | novel | Somatic | V571F | 0.138298 | NM_024940.8 | 188 | 162 | 26 |
| 879-PTsup | UBR5 | 171 | hg19 | chr8 | 103287769 | 103287769 | Missense_Mutation | SNP | A | A | C | novel | Somatic | S2237A | 0.356223 | NM_015902.6 | 231 | 148 | 83 |
| 879-PTsup | CDKN2A | 752 | hg19 | chr9 | 21971186 | 21971186 | Nonsense_Mutation | SNP | G | G | A | novel | Somatic | R58Ter | 0.590747 | NM_000077.4 | 557 | 225 | 332 |
| 879-PTsup | ASTN2 | 186 | hg19 | chr9 | 119188320 | 119188320 | Missense_Mutation | SNP | C | C | A | novel | Somatic | C1277F | 0.031414 | NM_001365068.1 | 382 | 370 | 12 |
| 879-PTsup | CNTRL | 1530 | hg19 | chr9 | 123875984 | 123875984 | Frame_Shift_Ins | INS | - | - | A | novel | Somatic | I446NfsTer13 | 0.029018 | NM_007018.5 | 444 | 431 | 13 |
| 879-PTsup | LRRC8A | 1589 | hg19 | chr9 | 131669768 | 131669768 | Missense_Mutation | SNP | G | G | C | novel | Somatic | V109L | 0.227011 | NM_019594.3 | 347 | 268 | 79 |
| 879-PTsup | SNAPC4 | 1647 | hg19 | chr9 | 139273295 | 139273295 | Missense_Mutation | SNP | T | T | A | novel | Somatic | E995V | 0.07218 | NM_003086.3 | 654 | 606 | 48 |
| 879-PTsup | NOTCH1 | 1648 | hg19 | chr9 | 139412672 | 139412672 | Missense_Mutation | SNP | G | G | A | novel | Somatic | P391L | 0.261251 | NM_017617.5 | 910 | 672 | 238 |
| 879-PTsup | NOTCH1 | 1648 | hg19 | chr9 | 139412690 | 139412690 | Missense_Mutation | SNP | G | G | A | novel | Somatic | S385F | 0.524146 | NM_017617.5 | 845 | 400 | 445 |
| 879-PTsup | PTEN | 158 | hg19 | chr10 | 89717695 | 89717695 | Frame_Shift_Del | DEL | CTTTGAGTTC | CTTTGAGTTC | - | novel | Somatic | F241LfsTer12 | 0.295238 | NM_000314.8 | 105 | 74 | 31 |
| 879-PTsup | NUP98 | 76 | hg19 | chr11 | 3723825 | 3723825 | Missense_Mutation | SNP | C | C | T | novel | Somatic | R1127H | 0.199029 | NM_016320.5 | 206 | 165 | 41 |
| 879-PTsup | DNHD1 | 634 | hg19 | chr11 | 6519537 | 6519537 | Missense_Mutation | SNP | G | G | T | novel | Somatic | S31I | 0.399038 | NM_144666.2 | 204 | 121 | 83 |
| 879-PTsup | DCHS1 | 635 | hg19 | chr11 | 6661424 | 6661424 | Missense_Mutation | SNP | C | C | T | novel | Somatic | R474H | 0.197232 | NM_003737.4 | 287 | 230 | 57 |
| 879-PTsup | SBF2 | 82 | hg19 | chr11 | 9878168 | 9878168 | Missense_Mutation | SNP | C | C | T | novel | Somatic | E734K | 0.341463 | NM_030962.3 | 164 | 108 | 56 |
| 879-PTsup | HTATIP2 | 740 | hg19 | chr11 | 20385382 | 20385382 | In_Frame_Del | DEL | GCG | GCG | - | novel | Somatic | A14del | 0.023173 | NM_001098520.1 | 559 | 546 | 13 |
| 879-PTsup | OR8H3 | 1011 | hg19 | chr11 | 55890134 | 55890134 | Missense_Mutation | SNP | G | G | T | novel | Somatic | G96C | 0.089041 | NM_001005201.1 | 292 | 266 | 26 |
| 879-PTsup | OR5M11 | 1014 | hg19 | chr11 | 56310345 | 56310345 | Missense_Mutation | SNP | C | C | T | novel | Somatic | R130H | 0.352459 | NM_001005245.1 | 122 | 79 | 43 |
| 879-PTsup | OR5B12 | 1029 | hg19 | chr11 | 58207203 | 58207203 | Missense_Mutation | SNP | C | C | T | novel | Somatic | C141Y | 0.0625 | NM_001004733.2 | 87 | 69 | 18 |
| 879-PTsup | CCDC88B | 1074 | hg19 | chr11 | 64108738 | 64108738 | Splice_Region | SNP | G | G | T | novel | Somatic |  | 0.102894 | NM_032251.6 | 310 | 278 | 32 |
| 879-PTsup | ANO1 | 139 | hg19 | chr11 | 69933963 | 69933963 | Missense_Mutation | SNP | A | A | G | novel | Somatic | R72G | 0.45738 | NM_018043.6 | 480 | 260 | 220 |
| 879-PTsup | PIWIL4 | 1304 | hg19 | chr11 | 94322352 | 94322352 | Missense_Mutation | SNP | A | A | T | novel | Somatic | Q327L | 0.064151 | NM_152431.3 | 17 | 0 | 17 |
| 879-PTsup | BTG4 | 1434 | hg19 | chr11 | 111368004 | 111368004 | Missense_Mutation | SNP | C | C | T | novel | Somatic | D148N | 0.310185 | NM_001367975.1 | 216 | 149 | 67 |
| 879-PTsup | RASSF8 | 12 | hg19 | chr12 | 26217839 | 26217839 | Missense_Mutation | SNP | G | G | T | novel | Somatic | R171L | 0.335664 | NM_001164748.2 | 285 | 189 | 96 |
| 879-PTsup | KMT2D | 120 | hg19 | chr12 | 49437194 | 49437194 | Frame_Shift_Del | DEL | T | T | - | novel | Somatic | I1829LfsTer23 | 0.275641 | NM_003482.3 | 156 | 113 | 43 |
| 879-PTsup | TBC1D15 | 1136 | hg19 | chr12 | 72316848 | 72316848 | Frame_Shift_Del | DEL | ATGTTGTC | ATGTTGTC | - | novel | Somatic | V648DfsTer11 | 0.246094 | NM_022771.5 | 256 | 193 | 63 |
| 879-PTsup | OTOGL | 18 | hg19 | chr12 | 80647322 | 80647322 | Silent | SNP | T | T | A | novel | Somatic | V445= | 0.313167 | NM_173591.3 | 281 | 193 | 88 |
| 879-PTsup | TMPO | 1339 | hg19 | chr12 | 98926948 | 98926948 | Missense_Mutation | SNP | G | G | T | novel | Somatic | V305F | 0.349091 | NM_003276.2 | 273 | 177 | 96 |
| 879-PTsup | TMEM119 | 1416 | hg19 | chr12 | 108985825 | 108985825 | Missense_Mutation | SNP | A | A | G | novel | Somatic | V112A | 0.341373 | NM_181724.3 | 536 | 352 | 184 |
| 879-PTsup | ANAPC7 | 1430 | hg19 | chr12 | 110813881 | 110813881 | Missense_Mutation | SNP | T | T | C | novel | Somatic | I534V | 0.385965 | NM_016238.2 | 285 | 175 | 110 |
| 879-PTsup | SACS | 767 | hg19 | chr13 | 23909772 | 23909772 | Missense_Mutation | SNP | G | G | T | novel | Somatic | S2748Y | 0.110599 | NM_014363.6 | 217 | 193 | 24 |
| 879-PTsup | FOXO1 | 112 | hg19 | chr13 | 41240279 | 41240279 | Missense_Mutation | SNP | G | G | A | novel | Somatic | T24I | 0.283814 | NM_002015.4 | 451 | 323 | 128 |
| 879-PTsup | PCDH17 | 1029 | hg19 | chr13 | 58207323 | 58207323 | Missense_Mutation | SNP | G | G | T | novel | Somatic | A215S | 0.07173 | NM_001040429.3 | 237 | 220 | 17 |
| 879-PTsup | MYCBP2 | 147 | hg19 | chr13 | 77764414 | 77764414 | Missense_Mutation | SNP | G | G | A | novel | Somatic | A1376V | 0.251748 | NM_015057.5 | 143 | 107 | 36 |
| 879-PTsup | NIN | 121 | hg19 | chr14 | 51224291 | 51224291 | Missense_Mutation | SNP | G | G | A | novel | Somatic | R1153W | 0.140969 | NM_020921.3 | 227 | 195 | 32 |
| 879-PTsup | USP3 | 133 | hg19 | chr15 | 63880513 | 63880513 | Missense_Mutation | SNP | G | G | A | novel | Somatic | R369H | 0.216216 | NM_006537.4 | 148 | 116 | 32 |
| 879-PTsup | NTRK3 | 157 | hg19 | chr15 | 88483985 | 88483985 | Splice_Site | SNP | C | C | A | novel | Somatic |  | 0.03838 | NM_001012338.2 | 468 | 450 | 18 |
| 879-PTsup | ZNF768 | 817 | hg19 | chr16 | 30536125 | 30536125 | Missense_Mutation | SNP | T | T | C | novel | Somatic | I446V | 0.339607 | NM_024671.4 | 1162 | 765 | 397 |
| 879-PTsup | NFAT5 | 1116 | hg19 | chr16 | 69727099 | 69727099 | Missense_Mutation | SNP | C | C | G | novel | Somatic | T1124R | 0.33 | NM_138713.4 | 200 | 134 | 66 |
| 879-PTsup | TP53 | 642 | hg19 | chr17 | 7578406 | 7578406 | Missense_Mutation | SNP | C | C | T | novel | Somatic | R175H | 0.642384 | NM_000546.5 | 300 | 106 | 194 |
| 879-PTsup | CTC1 | 647 | hg19 | chr17 | 8135688 | 8135688 | Missense_Mutation | SNP | T | T | A | novel | Somatic | Q684L | 0.7 | NM_025099.6 | 109 | 32 | 77 |
| 879-PTsup | SDF2 | 790 | hg19 | chr17 | 26976064 | 26976064 | Missense_Mutation | DNP | GC | GC | AA | novel | Somatic | G193V | 0.262222 | NM_006923.4 | 219 | 160 | 59 |
| 879-PTsup | TMEM132E | 836 | hg19 | chr17 | 32965144 | 32965144 | Nonsense_Mutation | SNP | G | G | T | novel | Somatic | E1040Ter | 0.46806 | NM_001304438.2 | 859 | 456 | 403 |
| 879-PTsup | DDX42 | 16 | hg19 | chr17 | 61886282 | 61886282 | Missense_Mutation | SNP | G | G | A | novel | Somatic | G376R | 0.315927 | NM_007372.3 | 383 | 262 | 121 |
| 879-PTsup | BTBD17 | 1137 | hg19 | chr17 | 72353826 | 72353826 | Missense_Mutation | SNP | A | A | G | novel | Somatic | I136T | 0.022606 | NM_001080466.2 | 752 | 735 | 17 |
| 879-PTsup | CDK3 | 1149 | hg19 | chr17 | 73998025 | 73998025 | Missense_Mutation | SNP | A | A | G | novel | Somatic | I63V | 0.140221 | NM_001258.2 | 271 | 233 | 38 |
| 879-PTsup | NPC1 | 746 | hg19 | chr18 | 21119795 | 21119795 | Nonsense_Mutation | INS | - | - | TTAAATA | novel | Somatic | A926IfsTer2 | 0.087523 | NM_000271.5 | 537 | 490 | 47 |
| 879-PTsup | NPC1 | 746 | hg19 | chr18 | 21136524 | 21136524 | Missense_Mutation | SNP | G | G | A | novel | Somatic | R337W | 0.0448 | NM_000271.5 | 624 | 596 | 28 |
| 879-PTsup | AMH | 602 | hg19 | chr19 | 2250393 | 2250393 | Missense_Mutation | SNP | G | G | C | novel | Somatic | G157A | 0.324159 | NM_000479.5 | 654 | 442 | 212 |
| 879-PTsup | CATSPERD | 78 | hg19 | chr19 | 5754191 | 5754191 | Frame_Shift_Del | DEL | AT | AT | - | novel | Somatic | M405AfsTer109 | 0.183908 | NM_152784.4 | 174 | 142 | 32 |
| 879-PTsup | KEAP1 | 665 | hg19 | chr19 | 10610562 | 10610562 | Missense_Mutation | SNP | G | G | C | novel | Somatic | R50G | 0.15528 | NM_203500.2 | 322 | 272 | 50 |
| 879-PTsup | OR10H5 | 706 | hg19 | chr19 | 15905273 | 15905273 | Missense_Mutation | SNP | C | C | T | novel | Somatic | R139W | 0.293821 | NM_001004466.1 | 792 | 559 | 233 |
| 879-PTsup | SIN3B | 714 | hg19 | chr19 | 16942421 | 16942421 | Missense_Mutation | SNP | A | A | G | novel | Somatic | N115S | 0.372414 | NM_015260.4 | 145 | 91 | 54 |
| 879-PTsup | ZNF776 | 1029 | hg19 | chr19 | 58265844 | 58265844 | Missense_Mutation | SNP | A | A | G | novel | Somatic | Q449R | 0.17803 | NM_173632.4 | 262 | 215 | 47 |
| 879-PTsup | SNX5 | 721 | hg19 | chr20 | 17929583 | 17929583 | Missense_Mutation | SNP | T | T | A | novel | Somatic | K290M | 0.354497 | NM_014426.4 | 187 | 120 | 67 |
| 879-PTsup | CHRNA4 | 1057 | hg19 | chr20 | 61981730 | 61981730 | Missense_Mutation | SNP | G | G | A | novel | Somatic | R345C | 0.390863 | NM_000744.6 | 589 | 358 | 231 |
| 879-PTsup | GART | 851 | hg19 | chr21 | 34904736 | 34904736 | Missense_Mutation | SNP | T | T | C | novel | Somatic | K148R | 0.078652 | NM_000819.5 | 178 | 164 | 14 |
| 879-PTsup | SF3A1 | 819 | hg19 | chr22 | 30742395 | 30742395 | Missense_Mutation | SNP | T | T | C | novel | Somatic | E100G | 0.197279 | NM_005877.6 | 291 | 233 | 58 |
| 879-PTsup | APOL2 | 864 | hg19 | chr22 | 36627478 | 36627478 | Frame_Shift_Ins | INS | - | - | TAAG | novel | Somatic | F16LfsTer17 | 0.218085 | NM_145637.2 | 185 | 144 | 41 |
| 879-PTsup | SUN2 | 883 | hg19 | chr22 | 39135911 | 39135911 | Frame_Shift_Ins | INS | - | - | GG | novel | Somatic | L486PfsTer48 | 0.255708 | NM_001199579.2 | 219 | 163 | 56 |
| 879-PTsup | OPHN1 | 137 | hg19 | chrX | 67272395 | 67272395 | Missense_Mutation | SNP | G | G | A | novel | Somatic | R788W | 0.5 | NM_002547.3 | 127 | 63 | 64 |
| 879-PTsup | NAP1L3 | 1293 | hg19 | chrX | 92927304 | 92927304 | Missense_Mutation | SNP | C | C | T | novel | Somatic | E334K | 0.263158 | NM_004538.6 | 209 | 154 | 55 |
| 879-PTsup | CDR1 | 1652 | hg19 | chrX | 139866222 | 139866222 | Missense_Mutation | SNP | C | C | T | novel | Somatic | D104N | 0.726027 | NM_004065.2 | 146 | 40 | 106 |
| 879-PTdeep | TMEM54 | 839 | hg19 | chr1 | 33361163 | 33361163 | Missense_Mutation | SNP | C | C | A | novel | Somatic | R151L | 0.074689 | NM_033504.4 | 480 | 444 | 36 |
| 879-PTdeep | SNIP1 | 109 | hg19 | chr1 | 38003368 | 38003368 | In_Frame_Del | DEL | TCC | TCC | - | novel | Somatic | E392del | 0.037037 | NM_024700.4 | 297 | 286 | 11 |
| 879-PTdeep | IFI44L | 1188 | hg19 | chr1 | 79093715 | 79093715 | Missense_Mutation | SNP | G | G | C | novel | Somatic | D39H | 0.540881 | NM_006820.4 | 159 | 73 | 86 |
| 879-PTdeep | SCAMP3 | 1769 | hg19 | chr1 | 155227135 | 155227135 | Missense_Mutation | SNP | G | G | T | novel | Somatic | F240L | 0.353612 | NM_005698.4 | 263 | 170 | 93 |
| 879-PTdeep | TRMT1L | 1997 | hg19 | chr1 | 185094192 | 185094192 | Missense_Mutation | SNP | G | G | A | novel | Somatic | S548F | 0.355204 | NM_030934.5 | 441 | 284 | 157 |
| 879-PTdeep | IPO9 | 265 | hg19 | chr1 | 201824313 | 201824313 | Splice_Region | SNP | A | A | G | novel | Somatic |  | 0.331429 | NM_018085.5 | 175 | 117 | 58 |
| 879-PTdeep | DISP1 | 285 | hg19 | chr1 | 223178584 | 223178584 | Missense_Mutation | SNP | A | A | G | novel | Somatic | Y1282C | 0.346072 | NM_032890.5 | 470 | 307 | 163 |
| 879-PTdeep | NCAPH | 1325 | hg19 | chr2 | 97019070 | 97019070 | Missense_Mutation | SNP | C | C | T | novel | Somatic | R313C | 0.792627 | NM_015341.5 | 433 | 89 | 344 |
| 879-PTdeep | SPOPL | 205 | hg19 | chr2 | 139316665 | 139316665 | Missense_Mutation | SNP | G | G | T | novel | Somatic | R185L | 0.75 | NM_001001664.3 | 328 | 82 | 246 |
| 879-PTdeep | UBR3 | 29 | hg19 | chr2 | 170761755 | 170761755 | Missense_Mutation | SNP | G | G | A | novel | Somatic | D533N | 0.376682 | NM_172070.4 | 445 | 277 | 168 |
| 879-PTdeep | FSIP2 | 31 | hg19 | chr2 | 186670324 | 186670324 | Missense_Mutation | SNP | A | A | G | novel | Somatic | S5431G | 0.809211 | NM_173651.4 | 301 | 55 | 246 |
| 879-PTdeep | DNAH7 | 260 | hg19 | chr2 | 196788373 | 196788373 | Frame_Shift_Ins | INS | - | - | T | novel | Somatic | N1257KfsTer3 | 0.04119 | NM_018897.3 | 437 | 419 | 18 |
| 879-PTdeep | XYLB | 877 | hg19 | chr3 | 38390124 | 38390124 | Splice_Site | INS | - | - | T | novel | Somatic |  | 0.619835 | NM_005108.4 | 239 | 89 | 150 |
| 879-PTdeep | DZIP1L | 1636 | hg19 | chr3 | 137816584 | 137816584 | Splice_Site | DEL | CGGGCTGCTGTCTGGACTCA | CGGGCTGCTGTCTGGACTCA | - | novel | Somatic |  | 0.173121 | NM_173543.3 | 437 | 361 | 76 |
| 879-PTdeep | KLF3 | 109 | hg19 | chr4 | 38696497 | 38696497 | Missense_Mutation | SNP | C | C | T | novel | Somatic | H276Y | 0.692661 | NM_016531.6 | 217 | 66 | 151 |
| 879-PTdeep | FIP1L1 | 124 | hg19 | chr4 | 54257211 | 54257211 | Missense_Mutation | SNP | A | A | T | novel | Somatic | N181Y | 0.646667 | NM_030917.4 | 150 | 53 | 97 |
| 879-PTdeep | SHROOM3 | 18 | hg19 | chr4 | 77662895 | 77662895 | Missense_Mutation | SNP | G | G | A | novel | Somatic | S1190N | 0.63786 | NM_020859.4 | 728 | 263 | 465 |
| 879-PTdeep | FAT4 | 193 | hg19 | chr4 | 126241765 | 126241765 | Missense_Mutation | SNP | G | G | A | novel | Somatic | R1400H | 0.080247 | NM_024582.4 | 162 | 149 | 13 |
| 879-PTdeep | SMARCA5 | 210 | hg19 | chr4 | 144442610 | 144442610 | Missense_Mutation | SNP | A | A | G | novel | Somatic | Y94C | 0.085427 | NM_003601.4 | 199 | 182 | 17 |
| 879-PTdeep | TERT | 594 | hg19 | chr5 | 1282667 | 1282667 | Frame_Shift_Del | DEL | ATCAGCCA | ATCAGCCA | - | novel | Somatic | W547EfsTer79 | 0.220588 | NM_198253.3 | 609 | 474 | 135 |
| 879-PTdeep | NIM1K | 114 | hg19 | chr5 | 43280665 | 43280665 | Nonsense_Mutation | SNP | C | C | G | novel | Somatic | S382Ter | 0.191874 | NM_153361.4 | 443 | 358 | 85 |
| 879-PTdeep | HARS | 1653 | hg19 | chr5 | 140059383 | 140059383 | Missense_Mutation | SNP | T | T | C | novel | Somatic | Y129C | 0.296296 | NM_002109.6 | 215 | 151 | 64 |
| 879-PTdeep | TIAM2 | 1771 | hg19 | chr6 | 155575684 | 155575684 | Missense_Mutation | SNP | G | G | T | novel | Somatic | R1482L | 0.381356 | NM_012454.3 | 469 | 289 | 180 |
| 879-PTdeep | SUN1 | 591 | hg19 | chr7 | 912913 | 912913 | Frame_Shift_Del | DEL | ATCCTGA | ATCCTGA | - | novel | Somatic | H906RfsTer67 | 0.56962 | NM_001367651.1 | 235 | 100 | 135 |
| 879-PTdeep | UBR5 | 171 | hg19 | chr8 | 103287769 | 103287769 | Missense_Mutation | SNP | A | A | C | novel | Somatic | S2237A | 0.358166 | NM_015902.6 | 349 | 224 | 125 |
| 879-PTdeep | CDKN2A | 752 | hg19 | chr9 | 21971186 | 21971186 | Nonsense_Mutation | SNP | G | G | A | novel | Somatic | R58Ter | 0.764187 | NM_000077.4 | 792 | 186 | 606 |
| 879-PTdeep | ASTN2 | 186 | hg19 | chr9 | 119188320 | 119188320 | Missense_Mutation | SNP | C | C | A | novel | Somatic | C1277F | 0.252555 | NM_001365068.1 | 684 | 511 | 173 |
| 879-PTdeep | CNTRL | 1530 | hg19 | chr9 | 123875984 | 123875984 | Frame_Shift_Ins | INS | - | - | A | novel | Somatic | I446NfsTer13 | 0.02977 | NM_007018.5 | 725 | 703 | 22 |
| 879-PTdeep | LRRC8A | 1589 | hg19 | chr9 | 131669768 | 131669768 | Missense_Mutation | SNP | G | G | C | novel | Somatic | V109L | 0.302452 | NM_019594.3 | 732 | 510 | 222 |
| 879-PTdeep | SNAPC4 | 1647 | hg19 | chr9 | 139273295 | 139273295 | Missense_Mutation | SNP | T | T | A | novel | Somatic | E995V | 0.265182 | NM_003086.3 | 987 | 725 | 262 |
| 879-PTdeep | NOTCH1 | 1648 | hg19 | chr9 | 139412672 | 139412672 | Missense_Mutation | SNP | G | G | A | novel | Somatic | P391L | 0.282804 | NM_017617.5 | 1227 | 880 | 347 |
| 879-PTdeep | NOTCH1 | 1648 | hg19 | chr9 | 139412690 | 139412690 | Missense_Mutation | SNP | G | G | A | novel | Somatic | S385F | 0.582028 | NM_017617.5 | 1213 | 507 | 706 |
| 879-PTdeep | APBB1IP | 98 | hg19 | chr10 | 26792159 | 26792159 | Missense_Mutation | SNP | A | A | C | novel | Somatic | I163L | 0.142857 | NM_019043.4 | 637 | 546 | 91 |
| 879-PTdeep | PTEN | 158 | hg19 | chr10 | 89717695 | 89717695 | Frame_Shift_Del | DEL | CTTTGAGTTC | CTTTGAGTTC | - | novel | Somatic | F241LfsTer12 | 0.635 | NM_000314.8 | 398 | 144 | 254 |
| 879-PTdeep | NUP98 | 76 | hg19 | chr11 | 3723825 | 3723825 | Missense_Mutation | SNP | C | C | T | novel | Somatic | R1127H | 0.362069 | NM_016320.5 | 405 | 258 | 147 |
| 879-PTdeep | DNHD1 | 634 | hg19 | chr11 | 6519537 | 6519537 | Missense_Mutation | SNP | G | G | T | novel | Somatic | S31I | 0.417633 | NM_144666.2 | 428 | 248 | 180 |
| 879-PTdeep | DCHS1 | 635 | hg19 | chr11 | 6661424 | 6661424 | Missense_Mutation | SNP | C | C | T | novel | Somatic | R474H | 0.365854 | NM_003737.4 | 609 | 384 | 225 |
| 879-PTdeep | USP47 | 84 | hg19 | chr11 | 11964590 | 11964590 | Nonsense_Mutation | SNP | G | G | T | novel | Somatic | E1028Ter | 0.037523 | NM_001330208.2 | 531 | 511 | 20 |
| 879-PTdeep | OR8H3 | 1011 | hg19 | chr11 | 55890134 | 55890134 | Missense_Mutation | SNP | G | G | T | novel | Somatic | G96C | 0.4 | NM_001005201.1 | 489 | 293 | 196 |
| 879-PTdeep | OR5M11 | 1014 | hg19 | chr11 | 56310345 | 56310345 | Missense_Mutation | SNP | C | C | T | novel | Somatic | R130H | 0.371025 | NM_001005245.1 | 283 | 178 | 105 |
| 879-PTdeep | LTBP3 | 1083 | hg19 | chr11 | 65307562 | 65307562 | Missense_Mutation | SNP | A | A | G | novel | Somatic | S1103P | 0.020782 | NM_001130144.2 | 816 | 799 | 17 |
| 879-PTdeep | ANO1 | 139 | hg19 | chr11 | 69933963 | 69933963 | Missense_Mutation | SNP | A | A | G | novel | Somatic | R72G | 0.381356 | NM_018043.6 | 944 | 584 | 360 |
| 879-PTdeep | BTG4 | 1434 | hg19 | chr11 | 111368004 | 111368004 | Missense_Mutation | SNP | C | C | T | novel | Somatic | D148N | 0.379135 | NM_001367975.1 | 392 | 243 | 149 |
| 879-PTdeep | RASSF8 | 12 | hg19 | chr12 | 26217839 | 26217839 | Missense_Mutation | SNP | G | G | T | novel | Somatic | R171L | 0.562278 | NM_001164748.2 | 558 | 242 | 316 |
| 879-PTdeep | KMT2D | 120 | hg19 | chr12 | 49437194 | 49437194 | Frame_Shift_Del | DEL | T | T | - | novel | Somatic | I1829LfsTer23 | 0.337209 | NM_003482.3 | 257 | 170 | 87 |
| 879-PTdeep | SPRYD3 | 992 | hg19 | chr12 | 53459656 | 53459656 | Missense_Mutation | SNP | C | C | A | novel | Somatic | G430V | 0.060976 | NM_032840.3 | 328 | 308 | 20 |
| 879-PTdeep | TBC1D15 | 1136 | hg19 | chr12 | 72316848 | 72316848 | Frame_Shift_Del | DEL | ATGTTGTC | ATGTTGTC | - | novel | Somatic | V648DfsTer11 | 0.255274 | NM_022771.5 | 473 | 352 | 121 |
| 879-PTdeep | OTOGL | 18 | hg19 | chr12 | 80647322 | 80647322 | Silent | SNP | T | T | A | novel | Somatic | V445= | 0.395095 | NM_173591.3 | 366 | 221 | 145 |
| 879-PTdeep | TMPO | 1339 | hg19 | chr12 | 98926948 | 98926948 | Missense_Mutation | SNP | G | G | T | novel | Somatic | V305F | 0.382046 | NM_003276.2 | 476 | 293 | 183 |
| 879-PTdeep | TMEM119 | 1416 | hg19 | chr12 | 108985825 | 108985825 | Missense_Mutation | SNP | A | A | G | novel | Somatic | V112A | 0.415274 | NM_181724.3 | 837 | 489 | 348 |
| 879-PTdeep | ANAPC7 | 1430 | hg19 | chr12 | 110813881 | 110813881 | Missense_Mutation | SNP | T | T | C | novel | Somatic | I534V | 0.412393 | NM_016238.2 | 466 | 273 | 193 |
| 879-PTdeep | EP400 | 199 | hg19 | chr12 | 132471330 | 132471330 | Missense_Mutation | SNP | C | C | T | novel | Somatic | T734M | 0.394737 | NM_015409.5 | 494 | 299 | 195 |
| 879-PTdeep | FOXO1 | 112 | hg19 | chr13 | 41240279 | 41240279 | Missense_Mutation | SNP | G | G | A | novel | Somatic | T24I | 0.412409 | NM_002015.4 | 819 | 480 | 339 |
| 879-PTdeep | PCDH17 | 1029 | hg19 | chr13 | 58207323 | 58207323 | Missense_Mutation | SNP | G | G | T | novel | Somatic | A215S | 0.371869 | NM_001040429.3 | 518 | 325 | 193 |
| 879-PTdeep | MYCBP2 | 147 | hg19 | chr13 | 77764414 | 77764414 | Missense_Mutation | SNP | G | G | A | novel | Somatic | A1376V | 0.33574 | NM_015057.5 | 276 | 183 | 93 |
| 879-PTdeep | USP3 | 133 | hg19 | chr15 | 63880513 | 63880513 | Missense_Mutation | SNP | G | G | A | novel | Somatic | R369H | 0.275862 | NM_006537.4 | 232 | 168 | 64 |
| 879-PTdeep | NTRK3 | 157 | hg19 | chr15 | 88483985 | 88483985 | Splice_Site | SNP | C | C | A | novel | Somatic |  | 0.074946 | NM_001012338.2 | 931 | 861 | 70 |
| 879-PTdeep | ZNF768 | 817 | hg19 | chr16 | 30536125 | 30536125 | Missense_Mutation | SNP | T | T | C | novel | Somatic | I446V | 0.394544 | NM_024671.4 | 1499 | 906 | 593 |
| 879-PTdeep | FBXL19 | 821 | hg19 | chr16 | 30939203 | 30939203 | Frame_Shift_Ins | INS | - | - | C | novel | Somatic | E206GfsTer17 | 0.027542 | NM_001099784.2 | 463 | 450 | 13 |
| 879-PTdeep | NFAT5 | 1116 | hg19 | chr16 | 69727099 | 69727099 | Missense_Mutation | SNP | C | C | G | novel | Somatic | T1124R | 0.306233 | NM_138713.4 | 369 | 256 | 113 |
| 879-PTdeep | TP53 | 642 | hg19 | chr17 | 7578406 | 7578406 | Missense_Mutation | SNP | C | C | T | novel | Somatic | R175H | 0.759124 | NM_000546.5 | 548 | 132 | 416 |
| 879-PTdeep | CTC1 | 647 | hg19 | chr17 | 8135688 | 8135688 | Missense_Mutation | SNP | T | T | A | novel | Somatic | Q684L | 0.791165 | NM_025099.6 | 249 | 52 | 197 |
| 879-PTdeep | SDF2 | 790 | hg19 | chr17 | 26976064 | 26976064 | Missense_Mutation | DNP | GC | GC | AA | novel | Somatic | G193V | 0.237037 | NM_006923.4 | 535 | 407 | 128 |
| 879-PTdeep | TMEM132E | 836 | hg19 | chr17 | 32965144 | 32965144 | Nonsense_Mutation | SNP | G | G | T | novel | Somatic | E1040Ter | 0.597127 | NM_001304438.2 | 1461 | 588 | 873 |
| 879-PTdeep | DDX42 | 16 | hg19 | chr17 | 61886282 | 61886282 | Missense_Mutation | SNP | G | G | A | novel | Somatic | G376R | 0.270911 | NM_007372.3 | 801 | 584 | 217 |
| 879-PTdeep | CDK3 | 1149 | hg19 | chr17 | 73998025 | 73998025 | Missense_Mutation | SNP | A | A | G | novel | Somatic | I63V | 0.30384 | NM_001258.2 | 599 | 417 | 182 |
| 879-PTdeep | FGF22 | 589 | hg19 | chr19 | 643410 | 643410 | Nonsense_Mutation | SNP | C | C | T | novel | Somatic | R107Ter | 0.055807 | NM_020637.1 | 661 | 624 | 37 |
| 879-PTdeep | AMH | 602 | hg19 | chr19 | 2250393 | 2250393 | Missense_Mutation | SNP | G | G | C | novel | Somatic | G157A | 0.40587 | NM_000479.5 | 987 | 586 | 401 |
| 879-PTdeep | SIN3B | 714 | hg19 | chr19 | 16942421 | 16942421 | Missense_Mutation | SNP | A | A | G | novel | Somatic | N115S | 0.304965 | NM_015260.4 | 282 | 196 | 86 |
| 879-PTdeep | SHKBP1 | 898 | hg19 | chr19 | 41097089 | 41097089 | Missense_Mutation | SNP | G | G | A | novel | Somatic | M700I | 0.029036 | NM_138392.4 | 861 | 836 | 25 |
| 879-PTdeep | ZNF776 | 1029 | hg19 | chr19 | 58265844 | 58265844 | Missense_Mutation | SNP | A | A | G | novel | Somatic | Q449R | 0.453382 | NM_173632.4 | 545 | 297 | 248 |
| 879-PTdeep | SNX5 | 721 | hg19 | chr20 | 17929583 | 17929583 | Missense_Mutation | SNP | T | T | A | novel | Somatic | K290M | 0.338279 | NM_014426.4 | 337 | 223 | 114 |
| 879-PTdeep | CHRNA4 | 1057 | hg19 | chr20 | 61981730 | 61981730 | Missense_Mutation | SNP | G | G | A | novel | Somatic | R345C | 0.679558 | NM_000744.6 | 542 | 173 | 369 |
| 879-PTdeep | GART | 851 | hg19 | chr21 | 34904736 | 34904736 | Missense_Mutation | SNP | T | T | C | novel | Somatic | K148R | 0.408571 | NM_000819.5 | 348 | 205 | 143 |
| 879-PTdeep | SF3A1 | 819 | hg19 | chr22 | 30742395 | 30742395 | Missense_Mutation | SNP | T | T | C | novel | Somatic | E100G | 0.373984 | NM_005877.6 | 491 | 307 | 184 |
| 879-PTdeep | APOL2 | 864 | hg19 | chr22 | 36627478 | 36627478 | Frame_Shift_Ins | INS | - | - | TAAG | novel | Somatic | F16LfsTer17 | 0.254408 | NM_145637.2 | 393 | 292 | 101 |
| 879-PTdeep | SUN2 | 883 | hg19 | chr22 | 39135911 | 39135911 | Frame_Shift_Ins | INS | - | - | GG | novel | Somatic | L486PfsTer48 | 0.355102 | NM_001199579.2 | 490 | 316 | 174 |
| 879-PTdeep | NAP1L3 | 1293 | hg19 | chrX | 92927304 | 92927304 | Missense_Mutation | SNP | C | C | T | novel | Somatic | E334K | 0.866005 | NM_004538.6 | 403 | 54 | 349 |
| 879-PTdeep | CDR1 | 1652 | hg19 | chrX | 139866222 | 139866222 | Missense_Mutation | SNP | C | C | T | novel | Somatic | D104N | 0.877551 | NM_004065.2 | 294 | 36 | 258 |
| 879-LNmet | IFI44L | 1188 | hg19 | chr1 | 79093715 | 79093715 | Missense_Mutation | SNP | G | G | C | novel | Somatic | D39H | 0.164122 | NM_006820.4 | 262 | 219 | 43 |
| 879-LNmet | FLG | 1746 | hg19 | chr1 | 152277448 | 152277448 | Missense_Mutation | SNP | C | C | T | novel | Somatic | R3305H | 0.027174 | NM_002016.2 | 908 | 883 | 25 |
| 879-LNmet | SCAMP3 | 1769 | hg19 | chr1 | 155227135 | 155227135 | Missense_Mutation | SNP | G | G | T | novel | Somatic | F240L | 0.16369 | NM_005698.4 | 336 | 281 | 55 |
| 879-LNmet | GPATCH4 | 1779 | hg19 | chr1 | 156565503 | 156565503 | Frame_Shift_Ins | INS | - | - | T | novel | Somatic | K211EfsTer16 | 0.029358 | NM_015590.3 | 528 | 512 | 16 |
| 879-LNmet | TRMT1L | 1997 | hg19 | chr1 | 185094192 | 185094192 | Missense_Mutation | SNP | G | G | A | novel | Somatic | S548F | 0.140693 | NM_030934.5 | 459 | 394 | 65 |
| 879-LNmet | IPO9 | 265 | hg19 | chr1 | 201824313 | 201824313 | Splice_Region | SNP | A | A | G | novel | Somatic |  | 0.166667 | NM_018085.5 | 198 | 165 | 33 |
| 879-LNmet | DISP1 | 285 | hg19 | chr1 | 223178584 | 223178584 | Missense_Mutation | SNP | A | A | G | novel | Somatic | Y1282C | 0.138418 | NM_032890.5 | 354 | 305 | 49 |
| 879-LNmet | NCAPH | 1325 | hg19 | chr2 | 97019070 | 97019070 | Missense_Mutation | SNP | C | C | T | novel | Somatic | R313C | 0.327014 | NM_015341.5 | 421 | 283 | 138 |
| 879-LNmet | SPOPL | 205 | hg19 | chr2 | 139316665 | 139316665 | Missense_Mutation | SNP | G | G | T | novel | Somatic | R185L | 0.251445 | NM_001001664.3 | 344 | 257 | 87 |
| 879-LNmet | UBR3 | 29 | hg19 | chr2 | 170761755 | 170761755 | Missense_Mutation | SNP | G | G | A | novel | Somatic | D533N | 0.17316 | NM_172070.4 | 459 | 379 | 80 |
| 879-LNmet | FSIP2 | 31 | hg19 | chr2 | 186670324 | 186670324 | Missense_Mutation | SNP | A | A | G | novel | Somatic | S5431G | 0.27027 | NM_173651.4 | 296 | 216 | 80 |
| 879-LNmet | XYLB | 877 | hg19 | chr3 | 38390124 | 38390124 | Splice_Site | INS | - | - | T | novel | Somatic |  | 0.129199 | NM_005108.4 | 385 | 335 | 50 |
| 879-LNmet | DZIP1L | 1636 | hg19 | chr3 | 137816584 | 137816584 | Splice_Site | DEL | CGGGCTGCTGTCTGGACTCA | CGGGCTGCTGTCTGGACTCA | - | novel | Somatic |  | 0.054662 | NM_173543.3 | 309 | 292 | 17 |
| 879-LNmet | KLF3 | 109 | hg19 | chr4 | 38696497 | 38696497 | Missense_Mutation | SNP | C | C | T | novel | Somatic | H276Y | 0.233422 | NM_016531.6 | 377 | 289 | 88 |
| 879-LNmet | FIP1L1 | 124 | hg19 | chr4 | 54257211 | 54257211 | Missense_Mutation | SNP | A | A | T | novel | Somatic | N181Y | 0.176692 | NM_030917.4 | 266 | 219 | 47 |
| 879-LNmet | SHROOM3 | 18 | hg19 | chr4 | 77662895 | 77662895 | Missense_Mutation | SNP | G | G | A | novel | Somatic | S1190N | 0.210412 | NM_020859.4 | 1379 | 1088 | 291 |
| 879-LNmet | TERT | 594 | hg19 | chr5 | 1282667 | 1282667 | Frame_Shift_Del | DEL | ATCAGCCA | ATCAGCCA | - | novel | Somatic | W547EfsTer79 | 0.110879 | NM_198253.3 | 475 | 422 | 53 |
| 879-LNmet | NIM1K | 114 | hg19 | chr5 | 43280665 | 43280665 | Nonsense_Mutation | SNP | C | C | G | novel | Somatic | S382Ter | 0.101604 | NM_153361.4 | 373 | 335 | 38 |
| 879-LNmet | HARS | 1653 | hg19 | chr5 | 140059383 | 140059383 | Missense_Mutation | SNP | T | T | C | novel | Somatic | Y129C | 0.15122 | NM_002109.6 | 205 | 174 | 31 |
| 879-LNmet | TIAM2 | 1771 | hg19 | chr6 | 155575684 | 155575684 | Missense_Mutation | SNP | G | G | T | novel | Somatic | R1482L | 0.1625 | NM_012454.3 | 480 | 402 | 78 |
| 879-LNmet | SUN1 | 591 | hg19 | chr7 | 912913 | 912913 | Frame_Shift_Del | DEL | ATCCTGA | ATCCTGA | - | novel | Somatic | H906RfsTer67 | 0.140704 | NM_001367651.1 | 397 | 341 | 56 |
| 879-LNmet | CLIP2 | 143 | hg19 | chr7 | 73803561 | 73803561 | Missense_Mutation | SNP | A | A | G | novel | Somatic | I898V | 0.03902 | NM_003388.5 | 1102 | 1059 | 43 |
| 879-LNmet | UBR5 | 171 | hg19 | chr8 | 103287769 | 103287769 | Missense_Mutation | SNP | A | A | C | novel | Somatic | S2237A | 0.122302 | NM_015902.6 | 417 | 366 | 51 |
| 879-LNmet | CDKN2A | 752 | hg19 | chr9 | 21971186 | 21971186 | Nonsense_Mutation | SNP | G | G | A | novel | Somatic | R58Ter | 0.289474 | NM_000077.4 | 1216 | 864 | 352 |
| 879-LNmet | ASTN2 | 186 | hg19 | chr9 | 119188320 | 119188320 | Missense_Mutation | SNP | C | C | A | novel | Somatic | C1277F | 0.161905 | NM_001365068.1 | 524 | 439 | 85 |
| 879-LNmet | LRRC8A | 1589 | hg19 | chr9 | 131669768 | 131669768 | Missense_Mutation | SNP | G | G | C | novel | Somatic | V109L | 0.164 | NM_019594.3 | 499 | 417 | 82 |
| 879-LNmet | SNAPC4 | 1647 | hg19 | chr9 | 139273295 | 139273295 | Missense_Mutation | SNP | T | T | A | novel | Somatic | E995V | 0.133195 | NM_003086.3 | 960 | 832 | 128 |
| 879-LNmet | NOTCH1 | 1648 | hg19 | chr9 | 139412672 | 139412672 | Missense_Mutation | SNP | G | G | A | novel | Somatic | P391L | 0.165507 | NM_017617.5 | 1292 | 1078 | 214 |
| 879-LNmet | NOTCH1 | 1648 | hg19 | chr9 | 139412690 | 139412690 | Missense_Mutation | SNP | G | G | A | novel | Somatic | S385F | 0.305643 | NM_017617.5 | 1276 | 886 | 390 |
| 879-LNmet | APBB1IP | 98 | hg19 | chr10 | 26792159 | 26792159 | Missense_Mutation | SNP | A | A | C | novel | Somatic | I163L | 0.104566 | NM_019043.4 | 679 | 608 | 71 |
| 879-LNmet | PTEN | 158 | hg19 | chr10 | 89717695 | 89717695 | Frame_Shift_Del | DEL | CTTTGAGTTC | CTTTGAGTTC | - | novel | Somatic | F241LfsTer12 | 0.288462 | NM_000314.8 | 416 | 296 | 120 |
| 879-LNmet | NUP98 | 76 | hg19 | chr11 | 3723825 | 3723825 | Missense_Mutation | SNP | C | C | T | novel | Somatic | R1127H | 0.160287 | NM_016320.5 | 417 | 350 | 67 |
| 879-LNmet | DCHS1 | 635 | hg19 | chr11 | 6661424 | 6661424 | Missense_Mutation | SNP | C | C | T | novel | Somatic | R474H | 0.178571 | NM_003737.4 | 504 | 414 | 90 |
| 879-LNmet | OR8H3 | 1011 | hg19 | chr11 | 55890134 | 55890134 | Missense_Mutation | SNP | G | G | T | novel | Somatic | G96C | 0.142857 | NM_001005201.1 | 461 | 395 | 66 |
| 879-LNmet | OR5M11 | 1014 | hg19 | chr11 | 56310345 | 56310345 | Missense_Mutation | SNP | C | C | T | novel | Somatic | R130H | 0.153846 | NM_001005245.1 | 233 | 197 | 36 |
| 879-LNmet | ANO1 | 139 | hg19 | chr11 | 69933963 | 69933963 | Missense_Mutation | SNP | A | A | G | novel | Somatic | R72G | 0.226596 | NM_018043.6 | 940 | 727 | 213 |
| 879-LNmet | BTG4 | 1434 | hg19 | chr11 | 111368004 | 111368004 | Missense_Mutation | SNP | C | C | T | novel | Somatic | D148N | 0.175439 | NM_001367975.1 | 399 | 329 | 70 |
| 879-LNmet | A2M | 655 | hg19 | chr12 | 9230424 | 9230424 | Missense_Mutation | SNP | G | G | A | novel | Somatic | A1050V | 0.030303 | NM_000014.5 | 627 | 608 | 19 |
| 879-LNmet | RASSF8 | 12 | hg19 | chr12 | 26217839 | 26217839 | Missense_Mutation | SNP | G | G | T | novel | Somatic | R171L | 0.300172 | NM_001164748.2 | 581 | 406 | 175 |
| 879-LNmet | KMT2D | 120 | hg19 | chr12 | 49437194 | 49437194 | Frame_Shift_Del | DEL | T | T | - | novel | Somatic | I1829LfsTer23 | 0.1777 | NM_003482.3 | 287 | 236 | 51 |
| 879-LNmet | TBC1D15 | 1136 | hg19 | chr12 | 72316848 | 72316848 | Frame_Shift_Del | DEL | ATGTTGTC | ATGTTGTC | - | novel | Somatic | V648DfsTer11 | 0.097674 | NM_022771.5 | 430 | 388 | 42 |
| 879-LNmet | OTOGL | 18 | hg19 | chr12 | 80647322 | 80647322 | Silent | SNP | T | T | A | novel | Somatic | V445= | 0.135135 | NM_173591.3 | 407 | 352 | 55 |
| 879-LNmet | TMPO | 1339 | hg19 | chr12 | 98926948 | 98926948 | Missense_Mutation | SNP | G | G | T | novel | Somatic | V305F | 0.180915 | NM_003276.2 | 502 | 411 | 91 |
| 879-LNmet | TMEM119 | 1416 | hg19 | chr12 | 108985825 | 108985825 | Missense_Mutation | SNP | A | A | G | novel | Somatic | V112A | 0.157957 | NM_181724.3 | 842 | 709 | 133 |
| 879-LNmet | ANAPC7 | 1430 | hg19 | chr12 | 110813881 | 110813881 | Missense_Mutation | SNP | T | T | C | novel | Somatic | I534V | 0.165877 | NM_016238.2 | 422 | 352 | 70 |
| 879-LNmet | EP400 | 199 | hg19 | chr12 | 132471330 | 132471330 | Missense_Mutation | SNP | C | C | T | novel | Somatic | T734M | 0.181644 | NM_015409.5 | 523 | 428 | 95 |
| 879-LNmet | FOXO1 | 112 | hg19 | chr13 | 41240279 | 41240279 | Missense_Mutation | SNP | G | G | A | novel | Somatic | T24I | 0.207048 | NM_002015.4 | 1134 | 899 | 235 |
| 879-LNmet | PCDH17 | 1029 | hg19 | chr13 | 58207323 | 58207323 | Missense_Mutation | SNP | G | G | T | novel | Somatic | A215S | 0.228381 | NM_001040429.3 | 449 | 346 | 103 |
| 879-LNmet | MYCBP2 | 147 | hg19 | chr13 | 77764414 | 77764414 | Missense_Mutation | SNP | G | G | A | novel | Somatic | A1376V | 0.128492 | NM_015057.5 | 358 | 312 | 46 |
| 879-LNmet | USP3 | 133 | hg19 | chr15 | 63880513 | 63880513 | Missense_Mutation | SNP | G | G | A | novel | Somatic | R369H | 0.203647 | NM_006537.4 | 329 | 262 | 67 |
| 879-LNmet | NTRK3 | 157 | hg19 | chr15 | 88483985 | 88483985 | Splice_Site | SNP | C | C | A | novel | Somatic |  | 0.034307 | NM_001012338.2 | 787 | 760 | 27 |
| 879-LNmet | NPIPB11 | 809 | hg19 | chr16 | 29394452 | 29394452 | Missense_Mutation | SNP | C | C | G | novel | Somatic | D601H | 0.053508 | NM_001310137.2 | 1668 | 1578 | 90 |
| 879-LNmet | ZNF768 | 817 | hg19 | chr16 | 30536125 | 30536125 | Missense_Mutation | SNP | T | T | C | novel | Somatic | I446V | 0.185205 | NM_024671.4 | 1866 | 1518 | 348 |
| 879-LNmet | NFAT5 | 1116 | hg19 | chr16 | 69727099 | 69727099 | Missense_Mutation | SNP | C | C | G | novel | Somatic | T1124R | 0.162534 | NM_138713.4 | 363 | 304 | 59 |
| 879-LNmet | TP53 | 642 | hg19 | chr17 | 7578406 | 7578406 | Missense_Mutation | SNP | C | C | T | novel | Somatic | R175H | 0.357513 | NM_000546.5 | 577 | 370 | 207 |
| 879-LNmet | CTC1 | 647 | hg19 | chr17 | 8135688 | 8135688 | Missense_Mutation | SNP | T | T | A | novel | Somatic | Q684L | 0.348548 | NM_025099.6 | 241 | 157 | 84 |
| 879-LNmet | SDF2 | 790 | hg19 | chr17 | 26976064 | 26976064 | Missense_Mutation | DNP | GC | GC | AA | novel | Somatic | G193V | 0.124668 | NM_006923.4 | 372 | 325 | 47 |
| 879-LNmet | TMEM132E | 836 | hg19 | chr17 | 32965144 | 32965144 | Nonsense_Mutation | SNP | G | G | T | novel | Somatic | E1040Ter | 0.391493 | NM_001304438.2 | 1150 | 699 | 451 |
| 879-LNmet | DDX42 | 16 | hg19 | chr17 | 61886282 | 61886282 | Missense_Mutation | SNP | G | G | A | novel | Somatic | G376R | 0.131667 | NM_007372.3 | 600 | 521 | 79 |
| 879-LNmet | CDK3 | 1149 | hg19 | chr17 | 73998025 | 73998025 | Missense_Mutation | SNP | A | A | G | novel | Somatic | I63V | 0.149877 | NM_001258.2 | 406 | 345 | 61 |
| 879-LNmet | CDH2 | 97 | hg19 | chr18 | 25562964 | 25562964 | Missense_Mutation | SNP | C | C | T | novel | Somatic | D765N | 0.075912 | NM_001792.5 | 683 | 631 | 52 |
| 879-LNmet | AMH | 602 | hg19 | chr19 | 2250393 | 2250393 | Missense_Mutation | SNP | G | G | C | novel | Somatic | G157A | 0.191304 | NM_000479.5 | 1035 | 837 | 198 |
| 879-LNmet | SIN3B | 714 | hg19 | chr19 | 16942421 | 16942421 | Missense_Mutation | SNP | A | A | G | novel | Somatic | N115S | 0.146259 | NM_015260.4 | 294 | 251 | 43 |
| 879-LNmet | ZNF429 | 750 | hg19 | chr19 | 21720557 | 21720557 | Missense_Mutation | SNP | A | A | G | novel | Somatic | K568E | 0.024129 | NM_001001415.4 | 745 | 727 | 18 |
| 879-LNmet | ZNF776 | 1029 | hg19 | chr19 | 58265844 | 58265844 | Missense_Mutation | SNP | A | A | G | novel | Somatic | Q449R | 0.195164 | NM_173632.4 | 579 | 466 | 113 |
| 879-LNmet | SNX5 | 721 | hg19 | chr20 | 17929583 | 17929583 | Missense_Mutation | SNP | T | T | A | novel | Somatic | K290M | 0.106061 | NM_014426.4 | 330 | 295 | 35 |
| 879-LNmet | CHRNA4 | 1057 | hg19 | chr20 | 61981730 | 61981730 | Missense_Mutation | SNP | G | G | A | novel | Somatic | R345C | 0.222222 | NM_000744.6 | 915 | 711 | 204 |
| 879-LNmet | GART | 851 | hg19 | chr21 | 34904736 | 34904736 | Missense_Mutation | SNP | T | T | C | novel | Somatic | K148R | 0.117834 | NM_000819.5 | 314 | 277 | 37 |
| 879-LNmet | SF3A1 | 819 | hg19 | chr22 | 30742395 | 30742395 | Missense_Mutation | SNP | T | T | C | novel | Somatic | E100G | 0.14684 | NM_005877.6 | 537 | 458 | 79 |
| 879-LNmet | APOL2 | 864 | hg19 | chr22 | 36627478 | 36627478 | Frame_Shift_Ins | INS | - | - | TAAG | novel | Somatic | F16LfsTer17 | 0.095556 | NM_145637.2 | 449 | 406 | 43 |
| 879-LNmet | SUN2 | 883 | hg19 | chr22 | 39135911 | 39135911 | Frame_Shift_Ins | INS | - | - | GG | novel | Somatic | L486PfsTer48 | 0.193018 | NM_001199579.2 | 487 | 393 | 94 |
| 879-LNmet | NAP1L3 | 1293 | hg19 | chrX | 92927304 | 92927304 | Missense_Mutation | SNP | C | C | T | novel | Somatic | E334K | 0.521978 | NM_004538.6 | 364 | 174 | 190 |
| 879-LNmet | CDR1 | 1652 | hg19 | chrX | 139866222 | 139866222 | Missense_Mutation | SNP | C | C | T | novel | Somatic | D104N | 0.415771 | NM_004065.2 | 277 | 161 | 116 |
| 926-PTsup | SLC25A34 | 707 | hg19 | chr1 | 16065807 | 16065807 | Missense_Mutation | SNP | C | C | T | novel | Somatic | A274V | 0.272013 | NM_207348.3 | 630 | 457 | 173 |
| 926-PTsup | MST1L | 715 | hg19 | chr1 | 17087316 | 17087316 | Nonsense_Mutation | SNP | G | G | A | novel | Somatic | Q90Ter | 0.05788 | NM_001271733.1 | 1431 | 1348 | 83 |
| 926-PTsup | SPOCD1 | 831 | hg19 | chr1 | 32257833 | 32257833 | Missense_Mutation | SNP | C | C | T | novel | Somatic | R982H | 0.231068 | NM_144569.6 | 515 | 396 | 119 |
| 926-PTsup | SYDE2 | 1238 | hg19 | chr1 | 85648289 | 85648289 | Frame_Shift_Del | DEL | GATAT | GATAT | - | novel | Somatic | I678WfsTer11 | 0.166667 | NM_032184.2 | 311 | 259 | 52 |
| 926-PTsup | HFM1 | 160 | hg19 | chr1 | 91742185 | 91742185 | Missense_Mutation | SNP | C | C | A | novel | Somatic | K1196N | 0.229008 | NM_001017975.5 | 130 | 100 | 30 |
| 926-PTsup | ADAMTSL4 | 1733 | hg19 | chr1 | 150531580 | 150531580 | Missense_Mutation | SNP | G | G | A | novel | Somatic | R924H | 0.023724 | NM_001288608.1 | 2779 | 2713 | 66 |
| 926-PTsup | DENND4B | 1759 | hg19 | chr1 | 153915477 | 153915477 | In_Frame_Del | DEL | CCG | CCG | - | novel | Somatic | R149del | 0.022642 | NM_014856.3 | 792 | 774 | 18 |
| 926-PTsup | LRRC71 | 222 | hg19 | chr1 | 156897684 | 156897684 | Intron | SNP | C | C | A | novel | Somatic |  | 0.178819 | NM_144702.3 | 574 | 471 | 103 |
| 926-PTsup | NOS1AP | 1823 | hg19 | chr1 | 162324997 | 162324997 | Missense_Mutation | SNP | G | G | A | novel | Somatic | E206K | 0.125448 | NM_014697.3 | 556 | 486 | 70 |
| 926-PTsup | LHX9 | 2094 | hg19 | chr1 | 197890484 | 197890484 | Missense_Mutation | SNP | C | C | T | novel | Somatic | S143L | 0.281863 | NM_020204.3 | 407 | 292 | 115 |
| 926-PTsup | CR1 | 271 | hg19 | chr1 | 207760844 | 207760844 | Missense_Mutation | SNP | G | G | T | novel | Somatic | G1882W | 0.196078 | NM_000651.6 | 407 | 327 | 80 |
| 926-PTsup | ATAD2B | 11 | hg19 | chr2 | 24118738 | 24118738 | Nonsense_Mutation | SNP | G | G | A | novel | Somatic | R107Ter | 0.173489 | NM_017552.4 | 510 | 421 | 89 |
| 926-PTsup | ASXL2 | 97 | hg19 | chr2 | 25972944 | 25972944 | Missense_Mutation | SNP | A | A | C | novel | Somatic | V494G | 0.204036 | NM_018263.6 | 445 | 354 | 91 |
| 926-PTsup | CCT7 | 1145 | hg19 | chr2 | 73461513 | 73461513 | Splice_Site | SNP | G | G | A | novel | Somatic |  | 0.055109 | NM_006429.4 | 870 | 822 | 48 |
| 926-PTsup | IMP4 | 1585 | hg19 | chr2 | 131103006 | 131103006 | Frame_Shift_Del | DEL | T | T | - | novel | Somatic | M86Ter | 0.285285 | NM_033416.3 | 327 | 232 | 95 |
| 926-PTsup | TTN | 1955 | hg19 | chr2 | 179485306 | 179485306 | Missense_Mutation | SNP | C | C | T | novel | Somatic | M15314I | 0.080863 | NM_001267550.2 | 370 | 340 | 30 |
| 926-PTsup | CSRNP1 | 883 | hg19 | chr3 | 39184830 | 39184830 | Missense_Mutation | SNP | C | C | A | novel | Somatic | D496Y | 0.287037 | NM_033027.4 | 216 | 154 | 62 |
| 926-PTsup | LTF | 939 | hg19 | chr3 | 46490473 | 46490473 | Missense_Mutation | SNP | C | C | T | novel | Somatic | V365M | 0.212682 | NM_002343.6 | 754 | 593 | 161 |
| 926-PTsup | USP19 | 119 | hg19 | chr3 | 49152447 | 49152447 | Missense_Mutation | SNP | C | C | T | novel | Somatic | R708H | 0.259947 | NM_001351100.1 | 374 | 276 | 98 |
| 926-PTsup | DOCK3 | 15 | hg19 | chr3 | 51417604 | 51417604 | Frame_Shift_Del | DEL | C | C | - | novel | Somatic | P1852QfsTer45 | 0.02509 | NM_004947.5 | 557 | 543 | 14 |
| 926-PTsup | PXK | 128 | hg19 | chr3 | 58351613 | 58351613 | Nonsense_Mutation | SNP | C | C | T | novel | Somatic | R39Ter | 0.030445 | NM_017771.5 | 425 | 412 | 13 |
| 926-PTsup | MED12L | 3 | hg19 | chr3 | 151094952 | 151094952 | Missense_Mutation | SNP | A | A | G | novel | Somatic | I1399M | 0.115217 | NM_053002.5 | 460 | 407 | 53 |
| 926-PTsup | SLITRK3 | 1843 | hg19 | chr3 | 164907616 | 164907616 | Missense_Mutation | SNP | G | G | C | novel | Somatic | P335A | 0.087197 | NM_001318810.2 | 1443 | 1317 | 126 |
| 926-PTsup | ABCC5 | 1986 | hg19 | chr3 | 183665257 | 183665257 | Frame_Shift_Del | DEL | A | A | - | novel | Somatic | L1090CfsTer26 | 0.020619 | NM_005688.4 | 1164 | 1140 | 24 |
| 926-PTsup | SLC34A2 | 780 | hg19 | chr4 | 25673346 | 25673346 | Splice_Region | SNP | G | G | T | novel | Somatic |  | 0.200717 | NM_006424.3 | 279 | 223 | 56 |
| 926-PTsup | KIAA1109 | 190 | hg19 | chr4 | 123280852 | 123280852 | Missense_Mutation | SNP | A | A | T | novel | Somatic | T4926S | 0.27451 | NM_015312.3 | 204 | 148 | 56 |
| 926-PTsup | GRIA2 | 223 | hg19 | chr4 | 158254482 | 158254482 | Missense_Mutation | SNP | C | C | T | novel | Somatic | L378F | 0.330645 | NM_001083619.1 | 123 | 82 | 41 |
| 926-PTsup | SEMA5A | 10 | hg19 | chr5 | 9066758 | 9066758 | Missense_Mutation | SNP | C | C | T | novel | Somatic | E692K | 0.135135 | NM_003966.3 | 221 | 191 | 30 |
| 926-PTsup | MYO10 | 1 | hg19 | chr5 | 16704730 | 16704730 | Missense_Mutation | SNP | G | G | A | novel | Somatic | A745V | 0.069841 | NM_012334.3 | 627 | 583 | 44 |
| 926-PTsup | FGF10 | 923 | hg19 | chr5 | 44388621 | 44388621 | Missense_Mutation | SNP | G | G | A | novel | Somatic | S55F | 0.368996 | NM_004465.2 | 457 | 288 | 169 |
| 926-PTsup | ZSWIM6 | 16 | hg19 | chr5 | 60835626 | 60835626 | Intron | SNP | C | C | G | novel | Somatic |  | 0.35503 | NM_020928.2 | 169 | 109 | 60 |
| 926-PTsup | DHFR | 1194 | hg19 | chr5 | 79929811 | 79929811 | Missense_Mutation | SNP | C | C | T | novel | Somatic | E124K | 0.290541 | NM_000791.4 | 296 | 210 | 86 |
| 926-PTsup | MTRNR2L2 | 1194 | hg19 | chr5 | 79945871 | 79945871 | Missense_Mutation | SNP | G | G | A | novel | Somatic | S12L | 0.077419 | NM_001190470.2 | 306 | 282 | 24 |
| 926-PTsup | DMXL1 | 23 | hg19 | chr5 | 118510954 | 118510954 | Splice_Site | SNP | A | A | T | novel | Somatic |  | 0.100775 | NM_001290321.2 | 258 | 232 | 26 |
| 926-PTsup | PPP2R2B | 212 | hg19 | chr5 | 146258290 | 146258290 | In_Frame_Ins | INS | - | - | GCTGCTGCT | novel | Somatic | S17_S19dup | 0.116643 | NM_181675.3 | 1279 | 1115 | 164 |
| 926-PTsup | HMMR | 1827 | hg19 | chr5 | 162917425 | 162917425 | Frame_Shift_Ins | INS | - | - | A | novel | Somatic | Q668TfsTer3 | 0.05802 | NM_001142556.2 | 291 | 274 | 17 |
| 926-PTsup | ZSCAN12 | 801 | hg19 | chr6 | 28359193 | 28359193 | Missense_Mutation | SNP | G | G | A | novel | Somatic | H292Y | 0.26506 | NM_001368124.1 | 413 | 303 | 110 |
| 926-PTsup | TULP1 | 855 | hg19 | chr6 | 35478756 | 35478756 | Missense_Mutation | SNP | G | G | C | novel | Somatic | D127E | 0.020408 | NM_003322.6 | 580 | 568 | 12 |
| 926-PTsup | TNFAIP3 | 1639 | hg19 | chr6 | 138197132 | 138197132 | Splice_Site | SNP | G | G | C | novel | Somatic |  | 0.114155 | NM_001270508.2 | 218 | 193 | 25 |
| 926-PTsup | THBS2 | 1879 | hg19 | chr6 | 169648841 | 169648841 | Missense_Mutation | SNP | C | C | T | novel | Somatic | G94S | 0.176471 | NM_003247.3 | 508 | 418 | 90 |
| 926-PTsup | TNRC18 | 78 | hg19 | chr7 | 5348546 | 5348546 | Missense_Mutation | SNP | G | G | A | novel | Somatic | A2887V | 0.08121 | NM_001080495.2 | 1254 | 1152 | 102 |
| 926-PTsup | ADCY1 | 933 | hg19 | chr7 | 45632443 | 45632443 | Missense_Mutation | SNP | C | C | A | novel | Somatic | A242E | 0.165541 | NM_021116.4 | 591 | 493 | 98 |
| 926-PTsup | NSUN5 | 1139 | hg19 | chr7 | 72721452 | 72721452 | Missense_Mutation | SNP | A | A | G | novel | Somatic | S147P | 0.110865 | NM_148956.4 | 451 | 401 | 50 |
| 926-PTsup | FKBP6 | 142 | hg19 | chr7 | 72754687 | 72754687 | Missense_Mutation | SNP | C | C | G | novel | Somatic | H212Q | 0.238916 | NM_003602.5 | 406 | 309 | 97 |
| 926-PTsup | AKAP9 | 160 | hg19 | chr7 | 91736679 | 91736679 | Missense_Mutation | SNP | C | C | T | novel | Somatic | T3830M | 0.224687 | NM_005751.4 | 1032 | 799 | 233 |
| 926-PTsup | ATP5J2-PTCD1 | 1340 | hg19 | chr7 | 99032438 | 99032438 | Missense_Mutation | SNP | C | C | A | novel | Somatic | C192F | 0.296117 | NM_001198879.2 | 411 | 289 | 122 |
| 926-PTsup | MUC3A | 1352 | hg19 | chr7 | 100550396 | 100550396 | Missense_Mutation | SNP | G | G | C | novel | Somatic | S326T | 0.026154 | NM_005960.2 | 649 | 632 | 17 |
| 926-PTsup | CNOT7 | 715 | hg19 | chr8 | 17092298 | 17092298 | Missense_Mutation | SNP | A | A | C | novel | Somatic | F182C | 0.084142 | NM_013354.7 | 309 | 283 | 26 |
| 926-PTsup | MBOAT4 | 813 | hg19 | chr8 | 29989550 | 29989550 | Nonsense_Mutation | SNP | C | C | T | novel | Somatic | W406Ter | 0.056856 | NM_001100916.2 | 298 | 281 | 17 |
| 926-PTsup | ZFHX4 | 18 | hg19 | chr8 | 77763352 | 77763352 | Missense_Mutation | SNP | T | T | G | novel | Somatic | C1399G | 0.220751 | NM_024721.5 | 452 | 352 | 100 |
| 926-PTsup | ANGPT1 | 176 | hg19 | chr8 | 108509527 | 108509527 | Missense_Mutation | SNP | T | T | C | novel | Somatic | E87G | 0.205517 | NM_001146.5 | 724 | 575 | 149 |
| 926-PTsup | CSMD3 | 22 | hg19 | chr8 | 113353909 | 113353909 | Missense_Mutation | SNP | T | T | G | novel | Somatic | H2150P | 0.037267 | NM_198123.2 | 322 | 310 | 12 |
| 926-PTsup | GPR20 | 1671 | hg19 | chr8 | 142367126 | 142367126 | Missense_Mutation | SNP | C | C | T | novel | Somatic | V300I | 0.143378 | NM_005293.3 | 822 | 704 | 118 |
| 926-PTsup | CDKN2A | 752 | hg19 | chr9 | 21974744 | 21974744 | Frame_Shift_Del | DEL | ACCTCCTCTACCCGACCCCGGGCCGCGGCCGT | ACCTCCTCTACCCGACCCCGGGCCGCGGCCGT | - | novel | Somatic | T18AfsTer15 | 0.305062 | NM_000077.4 | 720 | 497 | 223 |
| 926-PTsup | AKNA | 1478 | hg19 | chr9 | 117130838 | 117130838 | Missense_Mutation | SNP | C | C | T | novel | Somatic | S485N | 0.061224 | NM_001317950.2 | 587 | 551 | 36 |
| 926-PTsup | NOTCH1 | 1648 | hg19 | chr9 | 139399507 | 139399507 | Missense_Mutation | SNP | C | C | A | novel | Somatic | D1546Y | 0.205558 | NM_017617.5 | 2085 | 1656 | 429 |
| 926-PTsup | BEND7 | 85 | hg19 | chr10 | 13568140 | 13568140 | Frame_Shift_Del | DEL | A | A | - | novel | Somatic | L48Ter | 0.22884 | NM_001369863.1 | 319 | 246 | 73 |
| 926-PTsup | ZNF33A | 877 | hg19 | chr10 | 38345465 | 38345465 | Missense_Mutation | SNP | G | G | A | novel | Somatic | D826N | 0.091716 | NM_001278177.2 | 336 | 305 | 31 |
| 926-PTsup | IRF7 | 589 | hg19 | chr11 | 613844 | 613844 | Missense_Mutation | SNP | G | G | A | novel | Somatic | S263F | 0.067398 | NM_001572.5 | 636 | 593 | 43 |
| 926-PTsup | OR52E4 | 630 | hg19 | chr11 | 5905892 | 5905892 | Missense_Mutation | SNP | C | C | T | novel | Somatic | R124C | 0.292135 | NM_001005165.1 | 267 | 189 | 78 |
| 926-PTsup | CKAP5 | 117 | hg19 | chr11 | 46784260 | 46784260 | Missense_Mutation | SNP | C | C | T | novel | Somatic | R1315Q | 0.081556 | NM_001008938.4 | 795 | 730 | 65 |
| 926-PTsup | OR5D14 | 1008 | hg19 | chr11 | 55563038 | 55563038 | Missense_Mutation | SNP | A | A | G | novel | Somatic | M3V | 0.144487 | NM_001004735.1 | 263 | 225 | 38 |
| 926-PTsup | P2RX3 | 1020 | hg19 | chr11 | 57116179 | 57116179 | Splice_Site | SNP | G | G | C | novel | Somatic |  | 0.047244 | NM_002559.5 | 381 | 363 | 18 |
| 926-PTsup | OR6Q1 | 1025 | hg19 | chr11 | 57798750 | 57798750 | Missense_Mutation | SNP | C | C | T | novel | Somatic | T109I | 0.218884 | NM_001005186.2 | 232 | 181 | 51 |
| 926-PTsup | EHBP1L1 | 1083 | hg19 | chr11 | 65352809 | 65352809 | Missense_Mutation | SNP | G | G | A | novel | Somatic | R1227Q | 0.052706 | NM_001099409.3 | 1421 | 1346 | 75 |
| 926-PTsup | GAL3ST3 | 1087 | hg19 | chr11 | 65810316 | 65810316 | Missense_Mutation | SNP | C | C | T | novel | Somatic | E320K | 0.3379 | NM_033036.3 | 657 | 435 | 222 |
| 926-PTsup | MYO7A | 1171 | hg19 | chr11 | 76885925 | 76885925 | Missense_Mutation | SNP | G | G | A | novel | Somatic | V687M | 0.258824 | NM_000260.4 | 425 | 315 | 110 |
| 926-PTsup | PGR | 169 | hg19 | chr11 | 100998355 | 100998355 | Frame_Shift_Del | DEL | GCGCCTTGCAGGGCGGCGGC | GCGCCTTGCAGGGCGGCGGC | - | novel | Somatic | P477GfsTer90 | 0.182812 | NM_000926.4 | 637 | 520 | 117 |
| 926-PTsup | DIXDC1 | 1438 | hg19 | chr11 | 111864390 | 111864390 | Splice_Region | SNP | C | C | A | novel | Somatic |  | 0.246429 | NM_001037954.4 | 277 | 208 | 69 |
| 926-PTsup | KDM5A | 73 | hg19 | chr12 | 416952 | 416952 | Frame_Shift_Ins | INS | - | - | T | novel | Somatic | G1200RfsTer7 | 0.024609 | NM_001042603.3 | 447 | 436 | 11 |
| 926-PTsup | WNK1 | 73 | hg19 | chr12 | 1006773 | 1006773 | Missense_Mutation | SNP | G | G | A | novel | Somatic | R2125Q | 0.230019 | NM_018979.4 | 512 | 394 | 118 |
| 926-PTsup | NAB2 | 1023 | hg19 | chr12 | 57485781 | 57485781 | Splice_Region | SNP | G | G | A | novel | Somatic | E319= | 0.249516 | NM_005967.4 | 517 | 388 | 129 |
| 926-PTsup | RFX4 | 175 | hg19 | chr12 | 107002573 | 107002573 | Splice_Site | SNP | A | A | T | novel | Somatic |  | 0.023529 | NM_001206691.2 | 762 | 744 | 18 |
| 926-PTsup | LATS2 | 93 | hg19 | chr13 | 21562482 | 21562482 | In_Frame_Ins | INS | - | - | GGGGCG | novel | Somatic | P479_A480dup | 0.307692 | NM_014572.3 | 352 | 236 | 116 |
| 926-PTsup | CCDC168 | 1373 | hg19 | chr13 | 103396628 | 103396628 | Missense_Mutation | SNP | C | C | T | novel | Somatic | R2140Q | 0.102439 | NM_001146197.3 | 205 | 184 | 21 |
| 926-PTsup | TTC6 | 109 | hg19 | chr14 | 38208351 | 38208351 | Missense_Mutation | SNP | C | C | T | novel | Somatic | S801F | 0.136778 | NM_001310135.2 | 328 | 283 | 45 |
| 926-PTsup | PYGL | 15 | hg19 | chr14 | 51375660 | 51375660 | Missense_Mutation | SNP | C | C | T | novel | Somatic | E731K | 0.056285 | NM_002863.5 | 532 | 502 | 30 |
| 926-PTsup | SYNE2 | 1078 | hg19 | chr14 | 64518939 | 64518939 | Missense_Mutation | SNP | G | G | C | novel | Somatic | V2770L | 0.042697 | NM_182914.2 | 445 | 426 | 19 |
| 926-PTsup | RIN3 | 161 | hg19 | chr14 | 93142824 | 93142824 | Missense_Mutation | SNP | G | G | T | novel | Somatic | K780N | 0.101695 | NM_024832.5 | 295 | 265 | 30 |
| 926-PTsup | CEP170B | 1388 | hg19 | chr14 | 105350384 | 105350384 | Missense_Mutation | SNP | C | C | T | novel | Somatic | T423M | 0.622754 | NM_001112726.3 | 1835 | 691 | 1144 |
| 926-PTsup | AHNAK2 | 1389 | hg19 | chr14 | 105416454 | 105416454 | Missense_Mutation | SNP | C | C | T | novel | Somatic | M1778I | 0.128556 | NM_138420.4 | 944 | 822 | 122 |
| 926-PTsup | NPAP1 | 775 | hg19 | chr15 | 24922728 | 24922728 | Missense_Mutation | SNP | G | G | C | novel | Somatic | D572H | 0.084375 | NM_018958.3 | 320 | 293 | 27 |
| 926-PTsup | AQP9 | 128 | hg19 | chr15 | 58476253 | 58476253 | Missense_Mutation | SNP | C | C | A | novel | Somatic | H269Q | 0.375622 | NM_020980.5 | 399 | 248 | 151 |
| 926-PTsup | SLX4 | 612 | hg19 | chr16 | 3640883 | 3640883 | Missense_Mutation | SNP | G | G | A | novel | Somatic | T919I | 0.298405 | NM_032444.4 | 438 | 307 | 131 |
| 926-PTsup | ABCC1 | 88 | hg19 | chr16 | 16208773 | 16208773 | Missense_Mutation | SNP | C | C | A | novel | Somatic | S1077Y | 0.258523 | NM_004996.4 | 352 | 261 | 91 |
| 926-PTsup | SEPHS2 | 817 | hg19 | chr16 | 30456729 | 30456729 | Missense_Mutation | SNP | C | C | A | novel | Somatic | G107V | 0.289412 | NM_012248.4 | 843 | 597 | 246 |
| 926-PTsup | ADCY7 | 1 | hg19 | chr16 | 50343519 | 50343519 | Missense_Mutation | SNP | G | G | A | novel | Somatic | A708T | 0.268493 | NM_001114.5 | 365 | 267 | 98 |
| 926-PTsup | PKD1L2 | 1204 | hg19 | chr16 | 81224296 | 81224296 | Missense_Mutation | SNP | A | A | T | novel | Somatic | S549T | 0.394102 | NM_001076780.2 | 373 | 226 | 147 |
| 926-PTsup | WDR81 | 597 | hg19 | chr17 | 1634452 | 1634452 | Missense_Mutation | SNP | A | A | G | novel | Somatic | I1353V | 0.346558 | NM_001163809.1 | 856 | 559 | 297 |
| 926-PTsup | TP53 | 642 | hg19 | chr17 | 7578508 | 7578508 | Missense_Mutation | SNP | C | C | T | novel | Somatic | C141Y | 0.59432 | NM_000546.5 | 493 | 200 | 293 |
| 926-PTsup | KCNJ12 | 747 | hg19 | chr17 | 21319695 | 21319695 | Missense_Mutation | SNP | G | G | C | novel | Somatic | K347N | 0.133383 | NM_021012.5 | 1342 | 1163 | 179 |
| 926-PTsup | SLFN12 | 842 | hg19 | chr17 | 33749657 | 33749657 | Missense_Mutation | SNP | T | T | C | novel | Somatic | I131V | 0.148649 | NM_018042.5 | 370 | 315 | 55 |
| 926-PTsup | TRIM37 | 127 | hg19 | chr17 | 57148333 | 57148333 | Intron | DEL | AACA | AACA | - | novel | Somatic |  | 0.257895 | NM_015294.6 | 379 | 281 | 98 |
| 926-PTsup | C17orf64 | 1031 | hg19 | chr17 | 58503528 | 58503528 | Missense_Mutation | SNP | G | G | C | novel | Somatic | E54Q | 0.113586 | NM_181707.3 | 449 | 398 | 51 |
| 926-PTsup | ARSG | 136 | hg19 | chr17 | 66303712 | 66303712 | Nonsense_Mutation | SNP | C | C | A | novel | Somatic | C26Ter | 0.336207 | NM_014960.5 | 347 | 230 | 117 |
| 926-PTsup | ABCA8 | 1095 | hg19 | chr17 | 66873703 | 66873703 | Missense_Mutation | SNP | C | C | T | novel | Somatic | V1386M | 0.534435 | NM_001288985.2 | 362 | 168 | 194 |
| 926-PTsup | CBX2 | 1178 | hg19 | chr17 | 77758744 | 77758744 | Missense_Mutation | SNP | A | A | T | novel | Somatic | E501V | 0.071618 | NM_005189.3 | 752 | 698 | 54 |
| 926-PTsup | RBBP8 | 92 | hg19 | chr18 | 20572852 | 20572852 | Frame_Shift_Ins | INS | - | - | A | novel | Somatic | H358TfsTer8 | 0.037773 | NM_002894.3 | 499 | 480 | 19 |
| 926-PTsup | ZNF521 | 94 | hg19 | chr18 | 22804421 | 22804421 | Missense_Mutation | SNP | T | T | A | novel | Somatic | Q1154L | 0.356083 | NM_015461.3 | 337 | 217 | 120 |
| 926-PTsup | CC2D1A | 86 | hg19 | chr19 | 14024281 | 14024281 | Missense_Mutation | SNP | C | C | T | novel | Somatic | P193L | 0.245283 | NM_017721.5 | 529 | 399 | 130 |
| 926-PTsup | SLC5A5 | 722 | hg19 | chr19 | 17992987 | 17992987 | Missense_Mutation | SNP | G | G | A | novel | Somatic | V401M | 0.064057 | NM_000453.3 | 280 | 262 | 18 |
| 926-PTsup | UPF1 | 729 | hg19 | chr19 | 18960988 | 18960988 | Missense_Mutation | SNP | G | G | A | novel | Somatic | R189H | 0.302817 | NM_002911.4 | 426 | 297 | 129 |
| 926-PTsup | ZNF429 | 750 | hg19 | chr19 | 21720557 | 21720557 | Missense_Mutation | SNP | A | A | G | novel | Somatic | K568E | 0.023576 | NM_001001415.4 | 508 | 496 | 12 |
| 926-PTsup | ZNF43 | 752 | hg19 | chr19 | 21992169 | 21992169 | Missense_Mutation | SNP | T | T | G | novel | Somatic | N224H | 0.035176 | NM_003423.4 | 397 | 383 | 14 |
| 926-PTsup | TGFB1 | 904 | hg19 | chr19 | 41858919 | 41858919 | In_Frame_Del | DEL | GCG | GCG | - | novel | Somatic | P10del | 0.020178 | NM_000660.7 | 1234 | 1209 | 25 |
| 926-PTsup | MEGF8 | 113 | hg19 | chr19 | 42872635 | 42872635 | Missense_Mutation | SNP | G | G | A | novel | Somatic | R2101Q | 0.130526 | NM_001271938.2 | 942 | 818 | 124 |
| 926-PTsup | PSG1 | 915 | hg19 | chr19 | 43376019 | 43376019 | Missense_Mutation | SNP | A | A | T | novel | Somatic | F203L | 0.233456 | NM_001297773.2 | 544 | 417 | 127 |
| 926-PTsup | ZNF227 | 926 | hg19 | chr19 | 44739389 | 44739389 | Missense_Mutation | SNP | C | C | T | novel | Somatic | P269L | 0.292035 | NM_182490.3 | 452 | 320 | 132 |
| 926-PTsup | FLT3LG | 966 | hg19 | chr19 | 49982165 | 49982165 | Frame_Shift_Ins | INS | - | - | C | novel | Somatic | S118QfsTer130 | 0.047619 | NM_001204502.2 | 457 | 434 | 23 |
| 926-PTsup | SMOX | 616 | hg19 | chr20 | 4164164 | 4164164 | Missense_Mutation | SNP | C | C | T | novel | Somatic | R465W | 0.240204 | NM_001270691.1 | 585 | 444 | 141 |
| 926-PTsup | MYH7B | 1 | hg19 | chr20 | 33580910 | 33580910 | Missense_Mutation | SNP | G | G | A | novel | Somatic | R772H | 0.093236 | NM_020884.5 | 546 | 495 | 51 |
| 926-PTsup | CSE1L | 118 | hg19 | chr20 | 47691910 | 47691910 | Missense_Mutation | SNP | T | T | A | novel | Somatic | F396L | 0.050633 | NM_001316.4 | 395 | 375 | 20 |
| 926-PTsup | BCAS1 | 986 | hg19 | chr20 | 52583477 | 52583477 | Missense_Mutation | SNP | T | T | C | novel | Somatic | R485G | 0.326923 | NM_001366298.1 | 520 | 350 | 170 |
| 926-PTsup | ZNF831 | 128 | hg19 | chr20 | 57768952 | 57768952 | Missense_Mutation | SNP | C | C | G | novel | Somatic | P960A | 0.190065 | NM_178457.2 | 462 | 374 | 88 |
| 926-PTsup | TAF4 | 130 | hg19 | chr20 | 60640306 | 60640306 | In_Frame_Del | DEL | GCCAGG | GCCAGG | - | novel | Somatic | P186_G187del | 0.140884 | NM_003185.3 | 724 | 622 | 102 |
| 926-PTsup | ITGB2 | 938 | hg19 | chr21 | 46326899 | 46326899 | Missense_Mutation | SNP | T | T | G | novel | Somatic | T87P | 0.55493 | NM_000211.5 | 355 | 158 | 197 |
| 926-PTsup | CCT8L2 | 715 | hg19 | chr22 | 17072543 | 17072543 | Missense_Mutation | SNP | C | C | T | novel | Somatic | D300N | 0.055375 | NM_014406.5 | 307 | 290 | 17 |
| 926-PTsup | CABIN1 | 96 | hg19 | chr22 | 24515615 | 24515615 | Frame_Shift_Del | DEL | AA | AA | - | novel | Somatic | K1528EfsTer65 | 0.081461 | NM_012295.4 | 354 | 325 | 29 |
| 926-PTsup | RASD2 | 859 | hg19 | chr22 | 35948044 | 35948044 | Nonsense_Mutation | SNP | C | C | T | novel | Somatic | Q256Ter | 0.082051 | NM_014310.4 | 779 | 715 | 64 |
| 926-PTsup | ADGRG4 | 202 | hg19 | chrX | 135432482 | 135432482 | Missense_Mutation | SNP | C | C | A | novel | Somatic | A2206E | 0.355769 | NM_153834.4 | 207 | 133 | 74 |
| 926-PTsup | PLXNA3 | 1757 | hg19 | chrX | 153700930 | 153700930 | Splice_Region | SNP | C | C | A | novel | Somatic |  | 0.370787 | NM_017514.5 | 89 | 56 | 33 |
| 926-PTdeep | SLC25A34 | 707 | hg19 | chr1 | 16065807 | 16065807 | Missense_Mutation | SNP | C | C | T | novel | Somatic | A274V | 0.078635 | NM_207348.3 | 673 | 620 | 53 |
| 926-PTdeep | SPOCD1 | 831 | hg19 | chr1 | 32257833 | 32257833 | Missense_Mutation | SNP | C | C | T | novel | Somatic | R982H | 0.102158 | NM_144569.6 | 692 | 621 | 71 |
| 926-PTdeep | HFM1 | 160 | hg19 | chr1 | 91742185 | 91742185 | Missense_Mutation | SNP | C | C | A | novel | Somatic | K1196N | 0.102041 | NM_001017975.5 | 147 | 132 | 15 |
| 926-PTdeep | ADAMTSL4 | 1733 | hg19 | chr1 | 150531580 | 150531580 | Missense_Mutation | SNP | G | G | A | novel | Somatic | R924H | 0.034105 | NM_001288608.1 | 1641 | 1585 | 56 |
| 926-PTdeep | LRRC71 | 222 | hg19 | chr1 | 156897684 | 156897684 | Intron | SNP | C | C | A | novel | Somatic |  | 0.086242 | NM_144702.3 | 487 | 445 | 42 |
| 926-PTdeep | LHX9 | 2094 | hg19 | chr1 | 197890484 | 197890484 | Missense_Mutation | SNP | C | C | T | novel | Somatic | S143L | 0.082828 | NM_020204.3 | 495 | 454 | 41 |
| 926-PTdeep | CR1 | 271 | hg19 | chr1 | 207760844 | 207760844 | Missense_Mutation | SNP | G | G | T | novel | Somatic | G1882W | 0.030848 | NM_000651.6 | 388 | 376 | 12 |
| 926-PTdeep | ATAD2B | 11 | hg19 | chr2 | 24118738 | 24118738 | Nonsense_Mutation | SNP | G | G | A | novel | Somatic | R107Ter | 0.067308 | NM_017552.4 | 415 | 387 | 28 |
| 926-PTdeep | ASXL2 | 97 | hg19 | chr2 | 25972944 | 25972944 | Missense_Mutation | SNP | A | A | C | novel | Somatic | V494G | 0.098039 | NM_018263.6 | 458 | 413 | 45 |
| 926-PTdeep | CCT7 | 1145 | hg19 | chr2 | 73461513 | 73461513 | Splice_Site | SNP | G | G | A | novel | Somatic |  | 0.080895 | NM_006429.4 | 581 | 534 | 47 |
| 926-PTdeep | IMP4 | 1585 | hg19 | chr2 | 131103006 | 131103006 | Frame_Shift_Del | DEL | T | T | - | novel | Somatic | M86Ter | 0.166234 | NM_033416.3 | 385 | 321 | 64 |
| 926-PTdeep | GRB14 | 230 | hg19 | chr2 | 165365288 | 165365288 | Frame_Shift_Del | DEL | T | T | - | novel | Somatic | K297NfsTer23 | 0.056782 | NM_004490.3 | 316 | 298 | 18 |
| 926-PTdeep | TTN | 1955 | hg19 | chr2 | 179485306 | 179485306 | Missense_Mutation | SNP | C | C | T | novel | Somatic | M15314I | 0.056962 | NM_001267550.2 | 316 | 298 | 18 |
| 926-PTdeep | CSRNP1 | 883 | hg19 | chr3 | 39184830 | 39184830 | Missense_Mutation | SNP | C | C | A | novel | Somatic | D496Y | 0.111111 | NM_033027.4 | 315 | 280 | 35 |
| 926-PTdeep | LTF | 939 | hg19 | chr3 | 46490473 | 46490473 | Missense_Mutation | SNP | C | C | T | novel | Somatic | V365M | 0.075058 | NM_002343.6 | 864 | 799 | 65 |
| 926-PTdeep | USP19 | 119 | hg19 | chr3 | 49152447 | 49152447 | Missense_Mutation | SNP | C | C | T | novel | Somatic | R708H | 0.086003 | NM_001351100.1 | 592 | 541 | 51 |
| 926-PTdeep | SLITRK3 | 1843 | hg19 | chr3 | 164907616 | 164907616 | Missense_Mutation | SNP | G | G | C | novel | Somatic | P335A | 0.026217 | NM_001318810.2 | 1066 | 1038 | 28 |
| 926-PTdeep | SEC31A | 152 | hg19 | chr4 | 83785565 | 83785565 | Frame_Shift_Del | DEL | T | T | - | novel | Somatic | I462LfsTer16 | 0.047923 | NM_001318120.1 | 313 | 298 | 15 |
| 926-PTdeep | KIAA1109 | 190 | hg19 | chr4 | 123280852 | 123280852 | Missense_Mutation | SNP | A | A | T | novel | Somatic | T4926S | 0.069672 | NM_015312.3 | 243 | 226 | 17 |
| 926-PTdeep | GRIA2 | 223 | hg19 | chr4 | 158254482 | 158254482 | Missense_Mutation | SNP | C | C | T | novel | Somatic | L378F | 0.064516 | NM_001083619.1 | 155 | 145 | 10 |
| 926-PTdeep | FGF10 | 923 | hg19 | chr5 | 44388621 | 44388621 | Missense_Mutation | SNP | G | G | A | novel | Somatic | S55F | 0.101923 | NM_004465.2 | 519 | 466 | 53 |
| 926-PTdeep | TNFAIP3 | 1639 | hg19 | chr6 | 138197132 | 138197132 | Splice_Site | SNP | G | G | C | novel | Somatic |  | 0.078947 | NM_001270508.2 | 228 | 210 | 18 |
| 926-PTdeep | THBS2 | 1879 | hg19 | chr6 | 169648841 | 169648841 | Missense_Mutation | SNP | C | C | T | novel | Somatic | G94S | 0.070912 | NM_003247.3 | 689 | 640 | 49 |
| 926-PTdeep | TNRC18 | 78 | hg19 | chr7 | 5348546 | 5348546 | Missense_Mutation | SNP | G | G | A | novel | Somatic | A2887V | 0.05814 | NM_001080495.2 | 943 | 888 | 55 |
| 926-PTdeep | ADCY1 | 933 | hg19 | chr7 | 45632443 | 45632443 | Missense_Mutation | SNP | C | C | A | novel | Somatic | A242E | 0.045273 | NM_021116.4 | 747 | 713 | 34 |
| 926-PTdeep | FKBP6 | 142 | hg19 | chr7 | 72754687 | 72754687 | Missense_Mutation | SNP | C | C | G | novel | Somatic | H212Q | 0.144105 | NM_003602.5 | 458 | 392 | 66 |
| 926-PTdeep | AKAP9 | 160 | hg19 | chr7 | 91736679 | 91736679 | Missense_Mutation | SNP | C | C | T | novel | Somatic | T3830M | 0.17094 | NM_005751.4 | 584 | 484 | 100 |
| 926-PTdeep | PEX1 | 160 | hg19 | chr7 | 92146721 | 92146721 | Frame_Shift_Del | DEL | T | T | - | novel | Somatic | I370LfsTer17 | 0.048493 | NM_000466.3 | 761 | 724 | 37 |
| 926-PTdeep | ATP5J2-PTCD1 | 1340 | hg19 | chr7 | 99032438 | 99032438 | Missense_Mutation | SNP | C | C | A | novel | Somatic | C192F | 0.078091 | NM_001198879.2 | 460 | 424 | 36 |
| 926-PTdeep | CNOT7 | 715 | hg19 | chr8 | 17092298 | 17092298 | Missense_Mutation | SNP | A | A | C | novel | Somatic | F182C | 0.054422 | NM_013354.7 | 294 | 278 | 16 |
| 926-PTdeep | MBOAT4 | 813 | hg19 | chr8 | 29989550 | 29989550 | Nonsense_Mutation | SNP | C | C | T | novel | Somatic | W406Ter | 0.046189 | NM_001100916.2 | 432 | 412 | 20 |
| 926-PTdeep | ANGPT1 | 176 | hg19 | chr8 | 108509527 | 108509527 | Missense_Mutation | SNP | T | T | C | novel | Somatic | E87G | 0.069231 | NM_001146.5 | 520 | 484 | 36 |
| 926-PTdeep | CSMD3 | 22 | hg19 | chr8 | 113304841 | 113304841 | Missense_Mutation | SNP | A | A | T | novel | Somatic | Y2905N | 0.038123 | NM_198123.2 | 341 | 328 | 13 |
| 926-PTdeep | AKNA | 1478 | hg19 | chr9 | 117130838 | 117130838 | Missense_Mutation | SNP | C | C | T | novel | Somatic | S485N | 0.053191 | NM_001317950.2 | 843 | 798 | 45 |
| 926-PTdeep | NOTCH1 | 1648 | hg19 | chr9 | 139399507 | 139399507 | Missense_Mutation | SNP | C | C | A | novel | Somatic | D1546Y | 0.072452 | NM_017617.5 | 1251 | 1160 | 91 |
| 926-PTdeep | BEND7 | 85 | hg19 | chr10 | 13568140 | 13568140 | Frame_Shift_Del | DEL | A | A | - | novel | Somatic | L48Ter | 0.062745 | NM_001369863.1 | 252 | 236 | 16 |
| 926-PTdeep | CKAP5 | 117 | hg19 | chr11 | 46784260 | 46784260 | Missense_Mutation | SNP | C | C | T | novel | Somatic | R1315Q | 0.055432 | NM_001008938.4 | 450 | 425 | 25 |
| 926-PTdeep | P2RX3 | 1020 | hg19 | chr11 | 57116179 | 57116179 | Splice_Site | SNP | G | G | C | novel | Somatic |  | 0.054054 | NM_002559.5 | 333 | 315 | 18 |
| 926-PTdeep | RARRES3 | 133 | hg19 | chr11 | 63313708 | 63313708 | Frame_Shift_Ins | INS | - | - | A | novel | Somatic | A162SfsTer55 | 0.021692 | NM_004585.5 | 459 | 449 | 10 |
| 926-PTdeep | GAL3ST3 | 1087 | hg19 | chr11 | 65810316 | 65810316 | Missense_Mutation | SNP | C | C | T | novel | Somatic | E320K | 0.115385 | NM_033036.3 | 234 | 207 | 27 |
| 926-PTdeep | MYO7A | 1171 | hg19 | chr11 | 76885925 | 76885925 | Missense_Mutation | SNP | G | G | A | novel | Somatic | V687M | 0.081996 | NM_000260.4 | 561 | 515 | 46 |
| 926-PTdeep | DIXDC1 | 1438 | hg19 | chr11 | 111864390 | 111864390 | Splice_Region | SNP | C | C | A | novel | Somatic |  | 0.063063 | NM_001037954.4 | 331 | 310 | 21 |
| 926-PTdeep | TECTA | 188 | hg19 | chr11 | 120989227 | 120989227 | Missense_Mutation | SNP | G | G | A | novel | Somatic | G335S | 0.083333 | NM_005422.2 | 480 | 440 | 40 |
| 926-PTdeep | WNK1 | 73 | hg19 | chr12 | 1006773 | 1006773 | Missense_Mutation | SNP | G | G | A | novel | Somatic | R2125Q | 0.067839 | NM_018979.4 | 398 | 371 | 27 |
| 926-PTdeep | CHD4 | 79 | hg19 | chr12 | 6711546 | 6711546 | Frame_Shift_Del | DEL | T | T | - | novel | Somatic | K73RfsTer129 | 0.020833 | NM_001273.5 | 768 | 752 | 16 |
| 926-PTdeep | NAB2 | 1023 | hg19 | chr12 | 57485781 | 57485781 | Splice_Region | SNP | G | G | A | novel | Somatic | E319= | 0.074951 | NM_005967.4 | 507 | 469 | 38 |
| 926-PTdeep | LATS2 | 93 | hg19 | chr13 | 21562482 | 21562482 | In_Frame_Ins | INS | - | - | GGGGCG | novel | Somatic | P479_A480dup | 0.320388 | NM_014572.3 | 192 | 126 | 66 |
| 926-PTdeep | CCDC168 | 1373 | hg19 | chr13 | 103396628 | 103396628 | Missense_Mutation | SNP | C | C | T | novel | Somatic | R2140Q | 0.117371 | NM_001146197.3 | 213 | 188 | 25 |
| 926-PTdeep | RIN3 | 161 | hg19 | chr14 | 93142824 | 93142824 | Missense_Mutation | SNP | G | G | T | novel | Somatic | K780N | 0.066313 | NM_024832.5 | 376 | 351 | 25 |
| 926-PTdeep | CEP170B | 1388 | hg19 | chr14 | 105350384 | 105350384 | Missense_Mutation | SNP | C | C | T | novel | Somatic | T423M | 0.196391 | NM_001112726.3 | 1437 | 1154 | 283 |
| 926-PTdeep | NPAP1 | 775 | hg19 | chr15 | 24922728 | 24922728 | Missense_Mutation | SNP | G | G | C | novel | Somatic | D572H | 0.04119 | NM_018958.3 | 436 | 418 | 18 |
| 926-PTdeep | AQP9 | 128 | hg19 | chr15 | 58476253 | 58476253 | Missense_Mutation | SNP | C | C | A | novel | Somatic | H269Q | 0.138498 | NM_020980.5 | 426 | 367 | 59 |
| 926-PTdeep | SLX4 | 612 | hg19 | chr16 | 3640883 | 3640883 | Missense_Mutation | SNP | G | G | A | novel | Somatic | T919I | 0.056088 | NM_032444.4 | 731 | 690 | 41 |
| 926-PTdeep | ABCC1 | 88 | hg19 | chr16 | 16208773 | 16208773 | Missense_Mutation | SNP | C | C | A | novel | Somatic | S1077Y | 0.054118 | NM_004996.4 | 423 | 400 | 23 |
| 926-PTdeep | SEPHS2 | 817 | hg19 | chr16 | 30456729 | 30456729 | Missense_Mutation | SNP | C | C | A | novel | Somatic | G107V | 0.072027 | NM_012248.4 | 596 | 553 | 43 |
| 926-PTdeep | ADCY7 | 1 | hg19 | chr16 | 50343519 | 50343519 | Missense_Mutation | SNP | G | G | A | novel | Somatic | A708T | 0.077855 | NM_001114.5 | 577 | 532 | 45 |
| 926-PTdeep | PKD1L2 | 1204 | hg19 | chr16 | 81224296 | 81224296 | Missense_Mutation | SNP | A | A | T | novel | Somatic | S549T | 0.093976 | NM_001076780.2 | 415 | 376 | 39 |
| 926-PTdeep | WDR81 | 597 | hg19 | chr17 | 1634452 | 1634452 | Missense_Mutation | SNP | A | A | G | novel | Somatic | I1353V | 0.092369 | NM_001163809.1 | 745 | 676 | 69 |
| 926-PTdeep | TP53 | 642 | hg19 | chr17 | 7578508 | 7578508 | Missense_Mutation | SNP | C | C | T | novel | Somatic | C141Y | 0.22114 | NM_000546.5 | 717 | 558 | 159 |
| 926-PTdeep | KCNJ12 | 747 | hg19 | chr17 | 21319695 | 21319695 | Missense_Mutation | SNP | G | G | C | novel | Somatic | K347N | 0.024976 | NM_021012.5 | 2081 | 2029 | 52 |
| 926-PTdeep | TRIM37 | 127 | hg19 | chr17 | 57148333 | 57148333 | Intron | DEL | AACA | AACA | - | novel | Somatic |  | 0.102426 | NM_015294.6 | 371 | 333 | 38 |
| 926-PTdeep | C17orf64 | 1031 | hg19 | chr17 | 58503528 | 58503528 | Missense_Mutation | SNP | G | G | C | novel | Somatic | E54Q | 0.100324 | NM_181707.3 | 617 | 555 | 62 |
| 926-PTdeep | ARSG | 136 | hg19 | chr17 | 66303712 | 66303712 | Nonsense_Mutation | SNP | C | C | A | novel | Somatic | C26Ter | 0.108571 | NM_014960.5 | 349 | 311 | 38 |
| 926-PTdeep | ABCA8 | 1095 | hg19 | chr17 | 66873703 | 66873703 | Missense_Mutation | SNP | C | C | T | novel | Somatic | V1386M | 0.200375 | NM_001288985.2 | 534 | 427 | 107 |
| 926-PTdeep | CBX2 | 1178 | hg19 | chr17 | 77758744 | 77758744 | Missense_Mutation | SNP | A | A | T | novel | Somatic | E501V | 0.057692 | NM_005189.3 | 778 | 733 | 45 |
| 926-PTdeep | RBBP8 | 92 | hg19 | chr18 | 20572852 | 20572852 | Frame_Shift_Ins | INS | - | - | A | novel | Somatic | H358TfsTer8 | 0.036329 | NM_002894.3 | 520 | 501 | 19 |
| 926-PTdeep | ZNF521 | 94 | hg19 | chr18 | 22804421 | 22804421 | Missense_Mutation | SNP | T | T | A | novel | Somatic | Q1154L | 0.151316 | NM_015461.3 | 608 | 516 | 92 |
| 926-PTdeep | CC2D1A | 86 | hg19 | chr19 | 14024281 | 14024281 | Missense_Mutation | SNP | C | C | T | novel | Somatic | P193L | 0.069767 | NM_017721.5 | 687 | 639 | 48 |
| 926-PTdeep | SLC5A5 | 722 | hg19 | chr19 | 17992987 | 17992987 | Missense_Mutation | SNP | G | G | A | novel | Somatic | V401M | 0.061404 | NM_000453.3 | 341 | 320 | 21 |
| 926-PTdeep | UPF1 | 729 | hg19 | chr19 | 18960988 | 18960988 | Missense_Mutation | SNP | G | G | A | novel | Somatic | R189H | 0.07948 | NM_002911.4 | 690 | 635 | 55 |
| 926-PTdeep | ZNF429 | 750 | hg19 | chr19 | 21720557 | 21720557 | Missense_Mutation | SNP | A | A | G | novel | Somatic | K568E | 0.022109 | NM_001001415.4 | 586 | 573 | 13 |
| 926-PTdeep | NCCRP1 | 887 | hg19 | chr19 | 39689785 | 39689785 | Intron | SNP | G | G | A | novel | Somatic |  | 0.101351 | NM_001001414.2 | 147 | 132 | 15 |
| 926-PTdeep | PSG1 | 915 | hg19 | chr19 | 43376019 | 43376019 | Missense_Mutation | SNP | A | A | T | novel | Somatic | F203L | 0.079545 | NM_001297773.2 | 792 | 729 | 63 |
| 926-PTdeep | ZNF227 | 926 | hg19 | chr19 | 44739389 | 44739389 | Missense_Mutation | SNP | C | C | T | novel | Somatic | P269L | 0.079336 | NM_182490.3 | 539 | 496 | 43 |
| 926-PTdeep | SMOX | 616 | hg19 | chr20 | 4164164 | 4164164 | Missense_Mutation | SNP | C | C | T | novel | Somatic | R465W | 0.061611 | NM_001270691.1 | 843 | 791 | 52 |
| 926-PTdeep | MYH7B | 1 | hg19 | chr20 | 33580910 | 33580910 | Missense_Mutation | SNP | G | G | A | novel | Somatic | R772H | 0.050964 | NM_020884.5 | 725 | 688 | 37 |
| 926-PTdeep | PHF20 | 105 | hg19 | chr20 | 34459686 | 34459686 | Frame_Shift_Del | DEL | A | A | - | novel | Somatic | N407TfsTer3 | 0.022779 | NM_016436.5 | 439 | 429 | 10 |
| 926-PTdeep | BCAS1 | 986 | hg19 | chr20 | 52583477 | 52583477 | Missense_Mutation | SNP | T | T | C | novel | Somatic | R485G | 0.096234 | NM_001366298.1 | 717 | 648 | 69 |
| 926-PTdeep | ZNF831 | 128 | hg19 | chr20 | 57768952 | 57768952 | Missense_Mutation | SNP | C | C | G | novel | Somatic | P960A | 0.032508 | NM_178457.2 | 646 | 625 | 21 |
| 926-PTdeep | TAF4 | 130 | hg19 | chr20 | 60640306 | 60640306 | In_Frame_Del | DEL | GCCAGG | GCCAGG | - | novel | Somatic | P186_G187del | 0.08011 | NM_003185.3 | 362 | 333 | 29 |
| 926-PTdeep | TRAPPC10 | 932 | hg19 | chr21 | 45472274 | 45472274 | Frame_Shift_Ins | INS | - | - | A | novel | Somatic | N136KfsTer18 | 0.026871 | NM_003274.5 | 521 | 507 | 14 |
| 926-PTdeep | ITGB2 | 938 | hg19 | chr21 | 46326899 | 46326899 | Missense_Mutation | SNP | T | T | G | novel | Somatic | T87P | 0.150515 | NM_000211.5 | 484 | 411 | 73 |
| 926-PTdeep | CCT8L2 | 715 | hg19 | chr22 | 17072543 | 17072543 | Missense_Mutation | SNP | C | C | T | novel | Somatic | D300N | 0.053933 | NM_014406.5 | 443 | 419 | 24 |
| 926-PTdeep | CABIN1 | 96 | hg19 | chr22 | 24515615 | 24515615 | Frame_Shift_Del | DEL | AA | AA | - | novel | Somatic | K1528EfsTer65 | 0.066802 | NM_012295.4 | 492 | 459 | 33 |
| 926-PTdeep | RASD2 | 859 | hg19 | chr22 | 35948044 | 35948044 | Nonsense_Mutation | SNP | C | C | T | novel | Somatic | Q256Ter | 0.068756 | NM_014310.4 | 989 | 921 | 68 |
| 926-LNmet | SLC25A34 | 707 | hg19 | chr1 | 16065807 | 16065807 | Missense_Mutation | SNP | C | C | T | novel | Somatic | A274V | 0.157601 | NM_207348.3 | 717 | 604 | 113 |
| 926-LNmet | MST1L | 715 | hg19 | chr1 | 17087316 | 17087316 | Nonsense_Mutation | SNP | G | G | A | novel | Somatic | Q90Ter | 0.03416 | NM_001271733.1 | 1815 | 1753 | 62 |
| 926-LNmet | PHACTR4 | 804 | hg19 | chr1 | 28800272 | 28800272 | Frame_Shift_Ins | INS | - | - | C | novel | Somatic | L357TfsTer18 | 0.028355 | NM_023923.4 | 523 | 508 | 15 |
| 926-LNmet | SPOCD1 | 831 | hg19 | chr1 | 32257833 | 32257833 | Missense_Mutation | SNP | C | C | T | novel | Somatic | R982H | 0.106707 | NM_144569.6 | 656 | 586 | 70 |
| 926-LNmet | HFM1 | 160 | hg19 | chr1 | 91742185 | 91742185 | Missense_Mutation | SNP | C | C | A | novel | Somatic | K1196N | 0.112069 | NM_001017975.5 | 116 | 103 | 13 |
| 926-LNmet | ADAMTSL4 | 1733 | hg19 | chr1 | 150531580 | 150531580 | Missense_Mutation | SNP | G | G | A | novel | Somatic | R924H | 0.055505 | NM_001288608.1 | 2216 | 2093 | 123 |
| 926-LNmet | HRNR | 1746 | hg19 | chr1 | 152195729 | 152195729 | Translation_Start_Site | DEL | TT | TT | - | novel | Somatic |  | 0.109375 | NM_001009931.2 | 74 | 46 | 28 |
| 926-LNmet | LRRC71 | 222 | hg19 | chr1 | 156897684 | 156897684 | Intron | SNP | C | C | A | novel | Somatic |  | 0.127119 | NM_144702.3 | 589 | 514 | 75 |
| 926-LNmet | LHX9 | 2094 | hg19 | chr1 | 197890484 | 197890484 | Missense_Mutation | SNP | C | C | T | novel | Somatic | S143L | 0.134884 | NM_020204.3 | 430 | 372 | 58 |
| 926-LNmet | CR1 | 271 | hg19 | chr1 | 207760844 | 207760844 | Missense_Mutation | SNP | G | G | T | novel | Somatic | G1882W | 0.159744 | NM_000651.6 | 313 | 263 | 50 |
| 926-LNmet | ATAD2B | 11 | hg19 | chr2 | 24118738 | 24118738 | Nonsense_Mutation | SNP | G | G | A | novel | Somatic | R107Ter | 0.136842 | NM_017552.4 | 377 | 325 | 52 |
| 926-LNmet | ASXL2 | 97 | hg19 | chr2 | 25972944 | 25972944 | Missense_Mutation | SNP | A | A | C | novel | Somatic | V494G | 0.133333 | NM_018263.6 | 375 | 325 | 50 |
| 926-LNmet | CCT7 | 1145 | hg19 | chr2 | 73461513 | 73461513 | Splice_Site | SNP | G | G | A | novel | Somatic |  | 0.074419 | NM_006429.4 | 644 | 596 | 48 |
| 926-LNmet | DPP10 | 22 | hg19 | chr2 | 116572388 | 116572388 | Nonsense_Mutation | SNP | C | C | T | novel | Somatic | Q591Ter | 0.022831 | NM_001321905.2 | 438 | 428 | 10 |
| 926-LNmet | IMP4 | 1585 | hg19 | chr2 | 131103006 | 131103006 | Frame_Shift_Del | DEL | T | T | - | novel | Somatic | M86Ter | 0.211196 | NM_033416.3 | 391 | 308 | 83 |
| 926-LNmet | TTN | 1955 | hg19 | chr2 | 179485306 | 179485306 | Missense_Mutation | SNP | C | C | T | novel | Somatic | M15314I | 0.118132 | NM_001267550.2 | 363 | 320 | 43 |
| 926-LNmet | FBLN2 | 10 | hg19 | chr3 | 13612616 | 13612616 | Missense_Mutation | SNP | C | C | T | novel | Somatic | P254L | 0.022788 | NM_001004019.2 | 744 | 727 | 17 |
| 926-LNmet | CSRNP1 | 883 | hg19 | chr3 | 39184830 | 39184830 | Missense_Mutation | SNP | C | C | A | novel | Somatic | D496Y | 0.148515 | NM_033027.4 | 303 | 258 | 45 |
| 926-LNmet | LTF | 939 | hg19 | chr3 | 46490473 | 46490473 | Missense_Mutation | SNP | C | C | T | novel | Somatic | V365M | 0.122867 | NM_002343.6 | 879 | 771 | 108 |
| 926-LNmet | USP19 | 119 | hg19 | chr3 | 49152447 | 49152447 | Missense_Mutation | SNP | C | C | T | novel | Somatic | R708H | 0.13913 | NM_001351100.1 | 459 | 395 | 64 |
| 926-LNmet | MORC1 | 176 | hg19 | chr3 | 108723989 | 108723989 | Missense_Mutation | SNP | C | C | A | novel | Somatic | E647D | 0.085 | NM_014429.4 | 398 | 364 | 34 |
| 926-LNmet | EPHB1 | 201 | hg19 | chr3 | 134977867 | 134977867 | Missense_Mutation | SNP | A | A | G | novel | Somatic | I954V | 0.076923 | NM_004441.5 | 299 | 276 | 23 |
| 926-LNmet | MED12L | 3 | hg19 | chr3 | 151094952 | 151094952 | Missense_Mutation | SNP | A | A | G | novel | Somatic | I1399M | 0.107246 | NM_053002.5 | 344 | 307 | 37 |
| 926-LNmet | SLITRK3 | 1843 | hg19 | chr3 | 164907616 | 164907616 | Missense_Mutation | SNP | G | G | C | novel | Somatic | P335A | 0.055556 | NM_001318810.2 | 772 | 729 | 43 |
| 926-LNmet | ABCC5 | 1986 | hg19 | chr3 | 183665257 | 183665257 | Frame_Shift_Del | DEL | A | A | - | novel | Somatic | L1090CfsTer26 | 0.020672 | NM_005688.4 | 1161 | 1137 | 24 |
| 926-LNmet | ZFYVE28 | 75 | hg19 | chr4 | 2306616 | 2306616 | Missense_Mutation | SNP | G | G | A | novel | Somatic | S484L | 0.044215 | NM_020972.3 | 1357 | 1297 | 60 |
| 926-LNmet | RASGEF1B | 1213 | hg19 | chr4 | 82363492 | 82363492 | Frame_Shift_Del | DEL | T | T | - | novel | Somatic | T323LfsTer5 | 0.053333 | NM_152545.3 | 225 | 213 | 12 |
| 926-LNmet | KIAA1109 | 190 | hg19 | chr4 | 123280852 | 123280852 | Missense_Mutation | SNP | A | A | T | novel | Somatic | T4926S | 0.13617 | NM_015312.3 | 235 | 203 | 32 |
| 926-LNmet | GRIA2 | 223 | hg19 | chr4 | 158254482 | 158254482 | Missense_Mutation | SNP | C | C | T | novel | Somatic | L378F | 0.087248 | NM_001083619.1 | 149 | 136 | 13 |
| 926-LNmet | SEMA5A | 10 | hg19 | chr5 | 9066758 | 9066758 | Missense_Mutation | SNP | C | C | T | novel | Somatic | E692K | 0.090909 | NM_003966.3 | 230 | 209 | 21 |
| 926-LNmet | FGF10 | 923 | hg19 | chr5 | 44388621 | 44388621 | Missense_Mutation | SNP | G | G | A | novel | Somatic | S55F | 0.179752 | NM_004465.2 | 483 | 396 | 87 |
| 926-LNmet | ZSWIM6 | 16 | hg19 | chr5 | 60835626 | 60835626 | Intron | SNP | C | C | G | novel | Somatic |  | 0.135714 | NM_020928.2 | 140 | 121 | 19 |
| 926-LNmet | GCNT4 | 1152 | hg19 | chr5 | 74324983 | 74324983 | Frame_Shift_Del | DEL | G | G | - | novel | Somatic | H294IfsTer14 | 0.042904 | NM_001366737.1 | 302 | 289 | 13 |
| 926-LNmet | DHFR | 1194 | hg19 | chr5 | 79929811 | 79929811 | Missense_Mutation | SNP | C | C | T | novel | Somatic | E124K | 0.119835 | NM_000791.4 | 241 | 212 | 29 |
| 926-LNmet | DMXL1 | 23 | hg19 | chr5 | 118510954 | 118510954 | Splice_Site | SNP | A | A | T | novel | Somatic |  | 0.110497 | NM_001290321.2 | 180 | 160 | 20 |
| 926-LNmet | BTN1A1 | 787 | hg19 | chr6 | 26509049 | 26509049 | Missense_Mutation | SNP | C | C | T | novel | Somatic | R410W | 0.098039 | NM_001732.3 | 408 | 368 | 40 |
| 926-LNmet | THBS2 | 1879 | hg19 | chr6 | 169648841 | 169648841 | Missense_Mutation | SNP | C | C | T | novel | Somatic | G94S | 0.13172 | NM_003247.3 | 743 | 645 | 98 |
| 926-LNmet | ADCY1 | 933 | hg19 | chr7 | 45632443 | 45632443 | Missense_Mutation | SNP | C | C | A | novel | Somatic | A242E | 0.132231 | NM_021116.4 | 603 | 523 | 80 |
| 926-LNmet | FKBP6 | 142 | hg19 | chr7 | 72754687 | 72754687 | Missense_Mutation | SNP | C | C | G | novel | Somatic | H212Q | 0.131868 | NM_003602.5 | 364 | 316 | 48 |
| 926-LNmet | AKAP9 | 160 | hg19 | chr7 | 91736679 | 91736679 | Missense_Mutation | SNP | C | C | T | novel | Somatic | T3830M | 0.217228 | NM_005751.4 | 799 | 625 | 174 |
| 926-LNmet | ATP5J2-PTCD1 | 1340 | hg19 | chr7 | 99032438 | 99032438 | Missense_Mutation | SNP | C | C | A | novel | Somatic | C192F | 0.168508 | NM_001198879.2 | 362 | 301 | 61 |
| 926-LNmet | CNOT7 | 715 | hg19 | chr8 | 17092298 | 17092298 | Missense_Mutation | SNP | A | A | C | novel | Somatic | F182C | 0.118211 | NM_013354.7 | 313 | 276 | 37 |
| 926-LNmet | MBOAT4 | 813 | hg19 | chr8 | 29989550 | 29989550 | Nonsense_Mutation | SNP | C | C | T | novel | Somatic | W406Ter | 0.109694 | NM_001100916.2 | 392 | 349 | 43 |
| 926-LNmet | HGSNAT | 913 | hg19 | chr8 | 43033289 | 43033289 | Missense_Mutation | SNP | C | C | G | novel | Somatic | F308L | 0.115869 | NM_152419.3 | 397 | 351 | 46 |
| 926-LNmet | ZFHX4 | 18 | hg19 | chr8 | 77763352 | 77763352 | Missense_Mutation | SNP | T | T | G | novel | Somatic | C1399G | 0.098881 | NM_024721.5 | 535 | 482 | 53 |
| 926-LNmet | ANGPT1 | 176 | hg19 | chr8 | 108509527 | 108509527 | Missense_Mutation | SNP | T | T | C | novel | Somatic | E87G | 0.1 | NM_001146.5 | 440 | 396 | 44 |
| 926-LNmet | CSMD3 | 22 | hg19 | chr8 | 113304841 | 113304841 | Missense_Mutation | SNP | A | A | T | novel | Somatic | Y2905N | 0.104167 | NM_198123.2 | 288 | 258 | 30 |
| 926-LNmet | VLDLR | 605 | hg19 | chr9 | 2635500 | 2635500 | Missense_Mutation | SNP | C | C | T | novel | Somatic | R44C | 0.020576 | NM_003383.5 | 484 | 474 | 10 |
| 926-LNmet | CDKN2A | 752 | hg19 | chr9 | 21974744 | 21974744 | Frame_Shift_Del | DEL | ACCTCCTCTACCCGACCCCGGGCCGCGGCCGT | ACCTCCTCTACCCGACCCCGGGCCGCGGCCGT | - | novel | Somatic | T18AfsTer15 | 0.110224 | NM_000077.4 | 620 | 551 | 69 |
| 926-LNmet | NOTCH1 | 1648 | hg19 | chr9 | 139399507 | 139399507 | Missense_Mutation | SNP | C | C | A | novel | Somatic | D1546Y | 0.121685 | NM_017617.5 | 1278 | 1122 | 156 |
| 926-LNmet | BEND7 | 85 | hg19 | chr10 | 13568140 | 13568140 | Frame_Shift_Del | DEL | A | A | - | novel | Somatic | L48Ter | 0.136364 | NM_001369863.1 | 263 | 227 | 36 |
| 926-LNmet | NEBL | 93 | hg19 | chr10 | 21098812 | 21098812 | Missense_Mutation | SNP | C | C | G | novel | Somatic | R845P | 0.106443 | NM_006393.2 | 357 | 319 | 38 |
| 926-LNmet | BMPR1A | 157 | hg19 | chr10 | 88683229 | 88683229 | Missense_Mutation | SNP | G | G | A | novel | Somatic | R480Q | 0.081967 | NM_004329.2 | 427 | 392 | 35 |
| 926-LNmet | TRPM5 | 603 | hg19 | chr11 | 2436168 | 2436168 | Missense_Mutation | SNP | C | C | T | novel | Somatic | R530H | 0.021053 | NM_014555.3 | 1137 | 1113 | 24 |
| 926-LNmet | OR52E4 | 630 | hg19 | chr11 | 5905892 | 5905892 | Missense_Mutation | SNP | C | C | T | novel | Somatic | R124C | 0.103321 | NM_001005165.1 | 271 | 243 | 28 |
| 926-LNmet | CKAP5 | 117 | hg19 | chr11 | 46784260 | 46784260 | Missense_Mutation | SNP | C | C | T | novel | Somatic | R1315Q | 0.093588 | NM_001008938.4 | 575 | 521 | 54 |
| 926-LNmet | OR5D14 | 1008 | hg19 | chr11 | 55563038 | 55563038 | Missense_Mutation | SNP | A | A | G | novel | Somatic | M3V | 0.104938 | NM_001004735.1 | 162 | 145 | 17 |
| 926-LNmet | GAL3ST3 | 1087 | hg19 | chr11 | 65810316 | 65810316 | Missense_Mutation | SNP | C | C | T | novel | Somatic | E320K | 0.239067 | NM_033036.3 | 343 | 261 | 82 |
| 926-LNmet | MYO7A | 1171 | hg19 | chr11 | 76885925 | 76885925 | Missense_Mutation | SNP | G | G | A | novel | Somatic | V687M | 0.125 | NM_000260.4 | 535 | 468 | 67 |
| 926-LNmet | PGR | 169 | hg19 | chr11 | 100998355 | 100998355 | Frame_Shift_Del | DEL | GCGCCTTGCAGGGCGGCGGC | GCGCCTTGCAGGGCGGCGGC | - | novel | Somatic | P477GfsTer90 | 0.089974 | NM_000926.4 | 389 | 354 | 35 |
| 926-LNmet | DIXDC1 | 1438 | hg19 | chr11 | 111864390 | 111864390 | Splice_Region | SNP | C | C | A | novel | Somatic |  | 0.116505 | NM_001037954.4 | 308 | 272 | 36 |
| 926-LNmet | TECTA | 188 | hg19 | chr11 | 120989227 | 120989227 | Missense_Mutation | SNP | G | G | A | novel | Somatic | G335S | 0.123256 | NM_005422.2 | 430 | 377 | 53 |
| 926-LNmet | WNK1 | 73 | hg19 | chr12 | 1006773 | 1006773 | Missense_Mutation | SNP | G | G | A | novel | Somatic | R2125Q | 0.111429 | NM_018979.4 | 349 | 310 | 39 |
| 926-LNmet | NAB2 | 1023 | hg19 | chr12 | 57485781 | 57485781 | Splice_Region | SNP | G | G | A | novel | Somatic | E319= | 0.11925 | NM_005967.4 | 587 | 517 | 70 |
| 926-LNmet | LATS2 | 93 | hg19 | chr13 | 21562482 | 21562482 | In_Frame_Ins | INS | - | - | GGGGCG | novel | Somatic | P479_A480dup | 0.330275 | NM_014572.3 | 203 | 131 | 72 |
| 926-LNmet | ZIC2 | 1352 | hg19 | chr13 | 100635045 | 100635045 | Frame_Shift_Del | DEL | T | T | - | novel | Somatic | F244SfsTer13 | 0.020149 | NM_007129.5 | 1339 | 1312 | 27 |
| 926-LNmet | CCDC168 | 1373 | hg19 | chr13 | 103396628 | 103396628 | Missense_Mutation | SNP | C | C | T | novel | Somatic | R2140Q | 0.162562 | NM_001146197.3 | 203 | 170 | 33 |
| 926-LNmet | ARHGAP5 | 833 | hg19 | chr14 | 32561340 | 32561340 | Missense_Mutation | SNP | G | G | A | novel | Somatic | E489K | 0.471186 | NM_001030055.2 | 590 | 312 | 278 |
| 926-LNmet | RIN3 | 161 | hg19 | chr14 | 93142824 | 93142824 | Missense_Mutation | SNP | G | G | T | novel | Somatic | K780N | 0.123675 | NM_024832.5 | 283 | 248 | 35 |
| 926-LNmet | CEP170B | 1388 | hg19 | chr14 | 105350384 | 105350384 | Missense_Mutation | SNP | C | C | T | novel | Somatic | T423M | 0.368241 | NM_001112726.3 | 1460 | 922 | 538 |
| 926-LNmet | NPAP1 | 775 | hg19 | chr15 | 24922728 | 24922728 | Missense_Mutation | SNP | G | G | C | novel | Somatic | D572H | 0.087087 | NM_018958.3 | 333 | 304 | 29 |
| 926-LNmet | AQP9 | 128 | hg19 | chr15 | 58476253 | 58476253 | Missense_Mutation | SNP | C | C | A | novel | Somatic | H269Q | 0.252747 | NM_020980.5 | 364 | 272 | 92 |
| 926-LNmet | CACNA1H | 594 | hg19 | chr16 | 1260976 | 1260976 | Splice_Region | SNP | G | G | A | novel | Somatic |  | 0.132042 | NM_021098.3 | 568 | 493 | 75 |
| 926-LNmet | SLX4 | 612 | hg19 | chr16 | 3640883 | 3640883 | Missense_Mutation | SNP | G | G | A | novel | Somatic | T919I | 0.155556 | NM_032444.4 | 674 | 569 | 105 |
| 926-LNmet | ABCC1 | 88 | hg19 | chr16 | 16208773 | 16208773 | Missense_Mutation | SNP | C | C | A | novel | Somatic | S1077Y | 0.155263 | NM_004996.4 | 379 | 320 | 59 |
| 926-LNmet | ZP2 | 746 | hg19 | chr16 | 21209090 | 21209090 | Frame_Shift_Del | DEL | GT | GT | - | novel | Somatic | G699CfsTer? | 0.048387 | NM_003460.2 | 309 | 294 | 15 |
| 926-LNmet | SEPHS2 | 817 | hg19 | chr16 | 30456729 | 30456729 | Missense_Mutation | SNP | C | C | A | novel | Somatic | G107V | 0.133568 | NM_012248.4 | 568 | 492 | 76 |
| 926-LNmet | ADCY7 | 1 | hg19 | chr16 | 50343519 | 50343519 | Missense_Mutation | SNP | G | G | A | novel | Somatic | A708T | 0.106796 | NM_001114.5 | 515 | 460 | 55 |
| 926-LNmet | PKD1L2 | 1204 | hg19 | chr16 | 81224296 | 81224296 | Missense_Mutation | SNP | A | A | T | novel | Somatic | S549T | 0.155963 | NM_001076780.2 | 327 | 276 | 51 |
| 926-LNmet | WDR81 | 597 | hg19 | chr17 | 1634452 | 1634452 | Missense_Mutation | SNP | A | A | G | novel | Somatic | I1353V | 0.243158 | NM_001163809.1 | 949 | 718 | 231 |
| 926-LNmet | TP53 | 642 | hg19 | chr17 | 7578508 | 7578508 | Missense_Mutation | SNP | C | C | T | novel | Somatic | C141Y | 0.367188 | NM_000546.5 | 639 | 404 | 235 |
| 926-LNmet | KCNJ12 | 747 | hg19 | chr17 | 21319695 | 21319695 | Missense_Mutation | SNP | G | G | C | novel | Somatic | K347N | 0.050653 | NM_021012.5 | 1915 | 1818 | 97 |
| 926-LNmet | COIL | 1004 | hg19 | chr17 | 55028117 | 55028117 | Frame_Shift_Ins | INS | - | - | T | novel | Somatic | N162KfsTer6 | 0.133333 | NM_004645.3 | 617 | 533 | 84 |
| 926-LNmet | TRIM37 | 127 | hg19 | chr17 | 57148333 | 57148333 | Intron | DEL | AACA | AACA | - | novel | Somatic |  | 0.196809 | NM_015294.6 | 375 | 301 | 74 |
| 926-LNmet | C17orf64 | 1031 | hg19 | chr17 | 58503528 | 58503528 | Missense_Mutation | SNP | G | G | C | novel | Somatic | E54Q | 0.223035 | NM_181707.3 | 547 | 425 | 122 |
| 926-LNmet | ARSG | 136 | hg19 | chr17 | 66303712 | 66303712 | Nonsense_Mutation | SNP | C | C | A | novel | Somatic | C26Ter | 0.239264 | NM_014960.5 | 326 | 248 | 78 |
| 926-LNmet | ABCA8 | 1095 | hg19 | chr17 | 66873703 | 66873703 | Missense_Mutation | SNP | C | C | T | novel | Somatic | V1386M | 0.359801 | NM_001288985.2 | 403 | 258 | 145 |
| 926-LNmet | ZNF521 | 94 | hg19 | chr18 | 22804421 | 22804421 | Missense_Mutation | SNP | T | T | A | novel | Somatic | Q1154L | 0.14497 | NM_015461.3 | 338 | 289 | 49 |
| 926-LNmet | CC2D1A | 86 | hg19 | chr19 | 14024281 | 14024281 | Missense_Mutation | SNP | C | C | T | novel | Somatic | P193L | 0.123611 | NM_017721.5 | 718 | 629 | 89 |
| 926-LNmet | SLC5A5 | 722 | hg19 | chr19 | 17992987 | 17992987 | Missense_Mutation | SNP | G | G | A | novel | Somatic | V401M | 0.19802 | NM_000453.3 | 303 | 243 | 60 |
| 926-LNmet | UPF1 | 729 | hg19 | chr19 | 18960988 | 18960988 | Missense_Mutation | SNP | G | G | A | novel | Somatic | R189H | 0.163424 | NM_002911.4 | 769 | 643 | 126 |
| 926-LNmet | ZNF729 | 756 | hg19 | chr19 | 22496875 | 22496875 | Missense_Mutation | SNP | C | C | T | novel | Somatic | S219L | 0.177719 | NM_001242680.2 | 377 | 310 | 67 |
| 926-LNmet | NCCRP1 | 887 | hg19 | chr19 | 39689785 | 39689785 | Intron | SNP | G | G | A | novel | Somatic |  | 0.121429 | NM_001001414.2 | 140 | 123 | 17 |
| 926-LNmet | PSG1 | 915 | hg19 | chr19 | 43376019 | 43376019 | Missense_Mutation | SNP | A | A | T | novel | Somatic | F203L | 0.122507 | NM_001297773.2 | 702 | 616 | 86 |
| 926-LNmet | ZNF227 | 926 | hg19 | chr19 | 44739389 | 44739389 | Missense_Mutation | SNP | C | C | T | novel | Somatic | P269L | 0.160271 | NM_182490.3 | 443 | 372 | 71 |
| 926-LNmet | ZSCAN1 | 1031 | hg19 | chr19 | 58565242 | 58565242 | Missense_Mutation | SNP | A | A | C | novel | Somatic | K350N | 0.130129 | NM_182572.4 | 852 | 741 | 111 |
| 926-LNmet | SMOX | 616 | hg19 | chr20 | 4164164 | 4164164 | Missense_Mutation | SNP | C | C | T | novel | Somatic | R465W | 0.152893 | NM_001270691.1 | 725 | 614 | 111 |
| 926-LNmet | MYH7B | 1 | hg19 | chr20 | 33580910 | 33580910 | Missense_Mutation | SNP | G | G | A | novel | Somatic | R772H | 0.093058 | NM_020884.5 | 677 | 614 | 63 |
| 926-LNmet | SLC35C2 | 928 | hg19 | chr20 | 44986386 | 44986386 | Silent | SNP | C | C | T | novel | Somatic | T78= | 0.080537 | NM_001281458.1 | 447 | 411 | 36 |
| 926-LNmet | BCAS1 | 986 | hg19 | chr20 | 52583477 | 52583477 | Missense_Mutation | SNP | T | T | C | novel | Somatic | R485G | 0.227444 | NM_001366298.1 | 532 | 411 | 121 |
| 926-LNmet | ZNF831 | 128 | hg19 | chr20 | 57768952 | 57768952 | Missense_Mutation | SNP | C | C | G | novel | Somatic | P960A | 0.087189 | NM_178457.2 | 562 | 513 | 49 |
| 926-LNmet | TAF4 | 130 | hg19 | chr20 | 60640306 | 60640306 | In_Frame_Del | DEL | GCCAGG | GCCAGG | - | novel | Somatic | P186_G187del | 0.121475 | NM_003185.3 | 460 | 404 | 56 |
| 926-LNmet | ITGB2 | 938 | hg19 | chr21 | 46326899 | 46326899 | Missense_Mutation | SNP | T | T | G | novel | Somatic | T87P | 0.313514 | NM_000211.5 | 552 | 378 | 174 |
| 926-LNmet | CABIN1 | 96 | hg19 | chr22 | 24515615 | 24515615 | Frame_Shift_Del | DEL | AA | AA | - | novel | Somatic | K1528EfsTer65 | 0.110672 | NM_012295.4 | 506 | 450 | 56 |
| 926-LNmet | RASD2 | 859 | hg19 | chr22 | 35948044 | 35948044 | Nonsense_Mutation | SNP | C | C | T | novel | Somatic | Q256Ter | 0.125348 | NM_014310.4 | 1077 | 942 | 135 |
| 926-LNmet | ARFGAP3 | 914 | hg19 | chr22 | 43213780 | 43213780 | Frame_Shift_Del | DEL | T | T | - | novel | Somatic | N299MfsTer22 | 0.026608 | NM_014570.5 | 451 | 439 | 12 |
| 973-PTsup | CLSTN1 | 82 | hg19 | chr1 | 9790640 | 9790640 | In_Frame_Del | DEL | CCT | CCT | - | novel | Somatic | E957del | 0.024131 | NM_001009566.3 | 1407 | 1373 | 34 |
| 973-PTsup | AADACL3 | 682 | hg19 | chr1 | 12785492 | 12785492 | Frame_Shift_Ins | INS | - | - | T | novel | Somatic | Q255SfsTer22 | 0.026854 | NM_001103170.3 | 778 | 757 | 21 |
| 973-PTsup | KLHDC7A | 728 | hg19 | chr1 | 18809264 | 18809264 | Missense_Mutation | SNP | G | G | A | novel | Somatic | E597K | 0.1678 | NM_152375.2 | 1323 | 1101 | 222 |
| 973-PTsup | PRMT6 | 1405 | hg19 | chr1 | 107599928 | 107599928 | Missense_Mutation | SNP | C | C | A | novel | Somatic | S197R | 0.111667 | NM_018137.3 | 1200 | 1066 | 134 |
| 973-PTsup | AKNAD1 | 1419 | hg19 | chr1 | 109391377 | 109391377 | Missense_Mutation | SNP | C | C | A | novel | Somatic | K414N | 0.040816 | NM_152763.5 | 781 | 749 | 32 |
| 973-PTsup | SPTA1 | 224 | hg19 | chr1 | 158614072 | 158614072 | Missense_Mutation | SNP | C | C | A | novel | Somatic | D1437Y | 0.158621 | NM_003126.4 | 866 | 728 | 138 |
| 973-PTsup | MIA3 | 285 | hg19 | chr1 | 222827625 | 222827625 | Missense_Mutation | SNP | G | G | T | novel | Somatic | M1454I | 0.138325 | NM_198551.4 | 787 | 678 | 109 |
| 973-PTsup | PGBD5 | 292 | hg19 | chr1 | 230473003 | 230473003 | Frame_Shift_Del | DEL | G | G | - | novel | Somatic | P309QfsTer7 | 0.088942 | NM_001258311.2 | 831 | 757 | 74 |
| 973-PTsup | PGBD5 | 292 | hg19 | chr1 | 230473006 | 230473006 | Missense_Mutation | SNP | C | C | T | novel | Somatic | G308D | 0.092616 | NM_001258311.2 | 797 | 723 | 74 |
| 973-PTsup | OR2L3 | 2478 | hg19 | chr1 | 248224394 | 248224394 | Missense_Mutation | SNP | A | A | C | novel | Somatic | K137N | 0.11789 | NM_001004687.1 | 1213 | 1070 | 143 |
| 973-PTsup | MATN3 | 739 | hg19 | chr2 | 20212266 | 20212266 | Missense_Mutation | SNP | C | C | G | novel | Somatic | G43R | 0.061714 | NM_002381.5 | 875 | 821 | 54 |
| 973-PTsup | SOS1 | 110 | hg19 | chr2 | 39237780 | 39237780 | Missense_Mutation | SNP | G | G | A | novel | Somatic | P819S | 0.119424 | NM_005633.3 | 695 | 612 | 83 |
| 973-PTsup | CCDC88A | 15 | hg19 | chr2 | 55549680 | 55549680 | Missense_Mutation | SNP | A | A | G | novel | Somatic | I1049T | 0.09164 | NM_001365480.1 | 622 | 565 | 57 |
| 973-PTsup | PTCD3 | 1243 | hg19 | chr2 | 86355084 | 86355084 | Missense_Mutation | SNP | C | C | A | novel | Somatic | H373N | 0.047666 | NM_017952.6 | 1004 | 956 | 48 |
| 973-PTsup | ZNF514 | 164 | hg19 | chr2 | 95818461 | 95818461 | Missense_Mutation | SNP | G | G | C | novel | Somatic | P59A | 0.138889 | NM_032788.3 | 1044 | 899 | 145 |
| 973-PTsup | MERTK | 180 | hg19 | chr2 | 112732857 | 112732857 | Intron | SNP | T | T | G | novel | Somatic |  | 0.117757 | NM_006343.3 | 535 | 472 | 63 |
| 973-PTsup | EPB41L5 | 188 | hg19 | chr2 | 120834593 | 120834593 | Silent | SNP | C | C | T | novel | Somatic | V184= | 0.113343 | NM_020909.4 | 697 | 618 | 79 |
| 973-PTsup | SCN1A | 232 | hg19 | chr2 | 166904240 | 166904240 | Missense_Mutation | SNP | C | C | T | novel | Somatic | R356K | 0.086287 | NM_006920.6 | 649 | 593 | 56 |
| 973-PTsup | LRP2 | 235 | hg19 | chr2 | 169997024 | 169997024 | Frame_Shift_Ins | INS | - | - | G | novel | Somatic | C4381MfsTer12 | 0.020988 | NM_004525.3 | 809 | 792 | 17 |
| 973-PTsup | FSIP2 | 31 | hg19 | chr2 | 186655800 | 186655800 | Missense_Mutation | SNP | C | C | T | novel | Somatic | H1313Y | 0.102913 | NM_173651.4 | 514 | 461 | 53 |
| 973-PTsup | FETUB | 2006 | hg19 | chr3 | 186362543 | 186362543 | Frame_Shift_Ins | INS | - | - | A | novel | Somatic | I147DfsTer7 | 0.026403 | NM_014375.3 | 908 | 884 | 24 |
| 973-PTsup | HMX1 | 652 | hg19 | chr4 | 8873040 | 8873040 | Missense_Mutation | SNP | C | C | T | novel | Somatic | G101S | 0.134715 | NM_018942.3 | 384 | 332 | 52 |
| 973-PTsup | GABRA2 | 117 | hg19 | chr4 | 46314555 | 46314555 | Missense_Mutation | SNP | A | A | G | novel | Somatic | L145S | 0.104361 | NM_000807.4 | 642 | 575 | 67 |
| 973-PTsup | NFXL1 | 950 | hg19 | chr4 | 47898638 | 47898638 | Missense_Mutation | SNP | C | C | G | novel | Somatic | V411L | 0.105528 | NM_001278624.2 | 595 | 532 | 63 |
| 973-PTsup | UBA6 | 138 | hg19 | chr4 | 68506947 | 68506947 | Splice_Site | SNP | C | C | G | novel | Somatic |  | 0.141002 | NM_018227.6 | 539 | 463 | 76 |
| 973-PTsup | FAT1 | 251 | hg19 | chr4 | 187541121 | 187541121 | Missense_Mutation | SNP | T | T | C | novel | Somatic | N2207D | 0.060901 | NM_005245.4 | 821 | 771 | 50 |
| 973-PTsup | THBS4 | 1190 | hg19 | chr5 | 79335969 | 79335969 | Missense_Mutation | SNP | C | C | T | novel | Somatic | P53L | 0.133554 | NM_003248.6 | 906 | 785 | 121 |
| 973-PTsup | CEP120 | 23 | hg19 | chr5 | 122726977 | 122726977 | Missense_Mutation | SNP | C | C | T | novel | Somatic | G289R | 0.160109 | NM_153223.3 | 737 | 619 | 118 |
| 973-PTsup | ETV7 | 862 | hg19 | chr6 | 36334677 | 36334677 | In_Frame_Ins | INS | - | - | CTT | novel | Somatic | K294dup | 0.120209 | NM_016135.4 | 573 | 504 | 69 |
| 973-PTsup | CASP8AP2 | 159 | hg19 | chr6 | 90576391 | 90576391 | Frame_Shift_Del | DEL | A | A | - | novel | Somatic | K1130NfsTer6 | 0.031646 | NM_012115.4 | 474 | 459 | 15 |
| 973-PTsup | SMPD2 | 1422 | hg19 | chr6 | 109763188 | 109763188 | Missense_Mutation | SNP | G | G | C | novel | Somatic | G79A | 0.108571 | NM_003080.3 | 700 | 624 | 76 |
| 973-PTsup | NPSR1 | 106 | hg19 | chr7 | 34818157 | 34818157 | Nonsense_Mutation | SNP | C | C | T | novel | Somatic | R122Ter | 0.164326 | NM_001300935.1 | 709 | 592 | 117 |
| 973-PTsup | SFRP4 | 874 | hg19 | chr7 | 37955737 | 37955737 | Missense_Mutation | SNP | C | C | T | novel | Somatic | V135M | 0.151363 | NM_003014.4 | 1394 | 1183 | 211 |
| 973-PTsup | ADCY1 | 933 | hg19 | chr7 | 45743283 | 45743283 | Missense_Mutation | SNP | G | G | A | novel | Somatic | G886S | 0.108216 | NM_021116.4 | 1497 | 1335 | 162 |
| 973-PTsup | LOC102723899 | Unknown | hg19 | chr7 | 90116642 | 90116642 | Missense_Mutation | SNP | G | G | A | novel | Somatic | C65Y | 0.072773 | NM_001365443.2 | 795 | 737 | 58 |
| 973-PTsup | LINC00998 | Unknown | hg19 | chr7 | 112757567 | 112757567 | Missense_Mutation | SNP | C | C | T | novel | Somatic | E23K | 0.052073 | NM_001352687.1 | 1037 | 983 | 54 |
| 973-PTsup | CNTNAP2 | 26 | hg19 | chr7 | 147844694 | 147844694 | Missense_Mutation | SNP | G | G | C | novel | Somatic | R889T | 0.117711 | NM_014141.6 | 925 | 816 | 109 |
| 973-PTsup | ASIC3 | 1735 | hg19 | chr7 | 150747911 | 150747911 | Missense_Mutation | SNP | G | G | C | novel | Somatic | E294Q | 0.154728 | NM_004769.4 | 1047 | 885 | 162 |
| 973-PTsup | CSMD3 | 22 | hg19 | chr8 | 113349023 | 113349023 | Missense_Mutation | SNP | T | T | A | novel | Somatic | T2293S | 0.096045 | NM_198123.2 | 707 | 639 | 68 |
| 973-PTsup | NAPRT | 1688 | hg19 | chr8 | 144657082 | 144657082 | Intron | SNP | A | A | T | novel | Somatic |  | 0.1198 | NM_145201.6 | 1202 | 1058 | 144 |
| 973-PTsup | FPGS | 1581 | hg19 | chr9 | 130566669 | 130566669 | Missense_Mutation | SNP | T | T | A | novel | Somatic | Y82N | 0.169689 | NM_004957.6 | 772 | 641 | 131 |
| 973-PTsup | FNBP1 | 199 | hg19 | chr9 | 132687243 | 132687243 | Frame_Shift_Del | DEL | T | T | - | novel | Somatic | N328IfsTer7 | 0.031634 | NM_015033.3 | 569 | 551 | 18 |
| 973-PTsup | MLLT10 | 751 | hg19 | chr10 | 21962768 | 21962768 | Missense_Mutation | SNP | C | C | G | novel | Somatic | S514C | 0.040082 | NM_001195626.3 | 973 | 934 | 39 |
| 973-PTsup | CSGALNACT2 | 918 | hg19 | chr10 | 43662493 | 43662493 | Missense_Mutation | SNP | C | C | T | novel | Somatic | P401S | 0.06089 | NM_018590.5 | 854 | 802 | 52 |
| 973-PTsup | UTF1 | 1615 | hg19 | chr10 | 135044608 | 135044608 | Missense_Mutation | SNP | G | G | A | novel | Somatic | A230T | 0.222846 | NM_003577.3 | 528 | 409 | 119 |
| 973-PTsup | PANO1 | 591 | hg19 | chr11 | 798152 | 798152 | Missense_Mutation | SNP | C | C | T | novel | Somatic | S176L | 0.175737 | NM_001293167.1 | 882 | 727 | 155 |
| 973-PTsup | TRIM66 | 81 | hg19 | chr11 | 8642828 | 8642828 | Missense_Mutation | SNP | C | C | T | novel | Somatic | G923D | 0.134052 | NM_014818.1 | 649 | 562 | 87 |
| 973-PTsup | PIK3C2A | 89 | hg19 | chr11 | 17172180 | 17172180 | Missense_Mutation | SNP | T | T | A | novel | Somatic | T398S | 0.152893 | NM_001321378.2 | 726 | 615 | 111 |
| 973-PTsup | ARFGAP2 | 14 | hg19 | chr11 | 47198361 | 47198361 | Frame_Shift_Del | DEL | TTAAAAAGAG | TTAAAAAGAG | - | novel | Somatic | T12RfsTer46 | 0.104972 | NM_032389.6 | 723 | 647 | 76 |
| 973-PTsup | OR4C5 | 954 | hg19 | chr11 | 48387342 | 48387342 | Missense_Mutation | SNP | G | G | T | novel | Somatic | L226M | 0.03962 | NM_001348223.1 | 1261 | 1211 | 50 |
| 973-PTsup | OR8K5 | 1011 | hg19 | chr11 | 55927228 | 55927228 | Missense_Mutation | SNP | C | C | A | novel | Somatic | C189F | 0.1529 | NM_001004058.2 | 569 | 482 | 87 |
| 973-PTsup | TESMIN | 1107 | hg19 | chr11 | 68512541 | 68512541 | Missense_Mutation | SNP | A | A | C | novel | Somatic | C223W | 0.33969 | NM_004923.3 | 839 | 554 | 285 |
| 973-PTsup | SHANK2 | 140 | hg19 | chr11 | 70666506 | 70666506 | Missense_Mutation | SNP | C | C | T | novel | Somatic | E437K | 0.124524 | NM_012309.4 | 2626 | 2299 | 327 |
| 973-PTsup | NADSYN1 | 140 | hg19 | chr11 | 71208650 | 71208650 | Missense_Mutation | SNP | C | C | T | novel | Somatic | P629L | 0.179448 | NM_018161.5 | 651 | 534 | 117 |
| 973-PTsup | MPZL3 | 1486 | hg19 | chr11 | 118110996 | 118110996 | Frame_Shift_Ins | INS | - | - | TTGAC | novel | Somatic | T57SfsTer12 | 0.110363 | NM_198275.3 | 739 | 657 | 82 |
| 973-PTsup | PANX3 | 1534 | hg19 | chr11 | 124487217 | 124487217 | Silent | SNP | G | G | T | novel | Somatic | P124= | 0.161765 | NM_052959.3 | 1020 | 855 | 165 |
| 973-PTsup | CD9 | 633 | hg19 | chr12 | 6341822 | 6341822 | Missense_Mutation | SNP | G | G | A | novel | Somatic | G67D | 0.098569 | NM_001769.4 | 628 | 566 | 62 |
| 973-PTsup | GSG1 | 85 | hg19 | chr12 | 13240952 | 13240952 | Missense_Mutation | SNP | C | C | T | novel | Somatic | G215R | 0.103982 | NM_001367363.2 | 1354 | 1213 | 141 |
| 973-PTsup | KIF21A | 110 | hg19 | chr12 | 39731002 | 39731002 | Missense_Mutation | SNP | G | G | A | novel | Somatic | R772C | 0.103399 | NM_001173464.2 | 706 | 633 | 73 |
| 973-PTsup | MUC19 | 13 | hg19 | chr12 | 40925831 | 40925831 | Missense_Mutation | SNP | G | G | A | novel | Somatic | G7161D | 0.109356 | NM_173600.2 | 821 | 731 | 90 |
| 973-PTsup | SCN8A | 122 | hg19 | chr12 | 52159705 | 52159705 | Missense_Mutation | SNP | T | T | C | novel | Somatic | V932A | 0.102752 | NM_014191.4 | 1090 | 978 | 112 |
| 973-PTsup | AGAP2 | 1028 | hg19 | chr12 | 58126739 | 58126739 | Missense_Mutation | SNP | G | G | A | novel | Somatic | R525W | 0.137931 | NM_001122772.2 | 811 | 699 | 112 |
| 973-PTsup | PTPRQ | 150 | hg19 | chr12 | 80943267 | 80943267 | Missense_Mutation | SNP | C | C | A | novel | Somatic | Q1195K | 0.112613 | NM_001145026.2 | 886 | 786 | 100 |
| 973-PTsup | MGAT4C | 155 | hg19 | chr12 | 86377397 | 86377397 | Missense_Mutation | SNP | C | C | G | novel | Somatic | E105Q | 0.107996 | NM_001351282.2 | 963 | 859 | 104 |
| 973-PTsup | CCDC175 | 130 | hg19 | chr14 | 59998550 | 59998550 | Missense_Mutation | SNP | C | C | T | novel | Somatic | A568T | 0.096606 | NM_001164399.2 | 1149 | 1038 | 111 |
| 973-PTsup | SYNE2 | 1078 | hg19 | chr14 | 64554398 | 64554398 | Missense_Mutation | SNP | G | G | C | novel | Somatic | E3832Q | 0.036563 | NM_182914.2 | 546 | 526 | 20 |
| 973-PTsup | MPP5 | 137 | hg19 | chr14 | 67799632 | 67799632 | Missense_Mutation | SNP | G | G | C | novel | Somatic | D663H | 0.109827 | NM_022474.4 | 692 | 616 | 76 |
| 973-PTsup | FLVCR2 | 1165 | hg19 | chr14 | 76107558 | 76107558 | Nonsense_Mutation | SNP | C | C | T | novel | Somatic | Q459Ter | 0.10114 | NM_017791.3 | 702 | 631 | 71 |
| 973-PTsup | POMT2 | 1178 | hg19 | chr14 | 77778317 | 77778317 | Missense_Mutation | SNP | A | A | C | novel | Somatic | F103C | 0.129359 | NM_013382.5 | 889 | 774 | 115 |
| 973-PTsup | SLC24A4 | 161 | hg19 | chr14 | 92958069 | 92958069 | Missense_Mutation | SNP | G | G | A | novel | Somatic | G533E | 0.161172 | NM_153646.3 | 546 | 458 | 88 |
| 973-PTsup | CDC42BPB | 171 | hg19 | chr14 | 103470339 | 103470339 | Missense_Mutation | SNP | G | G | A | novel | Somatic | R125C | 0.145363 | NM_006035.4 | 1196 | 1022 | 174 |
| 973-PTsup | DPH6 | 858 | hg19 | chr15 | 35743001 | 35743001 | Nonsense_Mutation | SNP | A | A | T | novel | Somatic | C130Ter | 0.10643 | NM_080650.4 | 451 | 403 | 48 |
| 973-PTsup | ZNF106 | 113 | hg19 | chr15 | 42744109 | 42744109 | Missense_Mutation | SNP | C | C | T | novel | Somatic | D121N | 0.150761 | NM_001366845.2 | 722 | 613 | 109 |
| 973-PTsup | FAM227B | 120 | hg19 | chr15 | 49880233 | 49880233 | Missense_Mutation | SNP | G | G | T | novel | Somatic | H129N | 0.056478 | NM_152647.3 | 602 | 568 | 34 |
| 973-PTsup | PIAS1 | 138 | hg19 | chr15 | 68438207 | 68438207 | Missense_Mutation | SNP | T | T | G | novel | Somatic | L221R | 0.043062 | NM_001320687.1 | 1254 | 1200 | 54 |
| 973-PTsup | ISLR2 | 1152 | hg19 | chr15 | 74426327 | 74426327 | Missense_Mutation | SNP | C | C | T | novel | Somatic | S411F | 0.042074 | NM_020851.3 | 1329 | 1273 | 56 |
| 973-PTsup | ODF3L1 | 1164 | hg19 | chr15 | 76019693 | 76019693 | Missense_Mutation | SNP | C | C | G | novel | Somatic | Q213E | 0.116859 | NM_175881.5 | 1641 | 1449 | 192 |
| 973-PTsup | SNRPA1 | 1361 | hg19 | chr15 | 101827865 | 101827865 | Missense_Mutation | SNP | G | G | C | novel | Somatic | L118V | 0.146985 | NM_003090.4 | 794 | 677 | 117 |
| 973-PTsup | GSPT1 | 676 | hg19 | chr16 | 11969717 | 11969717 | Missense_Mutation | SNP | C | C | G | novel | Somatic | D588H | 0.146176 | NM_002094.4 | 1031 | 880 | 151 |
| 973-PTsup | CMTR2 | 1129 | hg19 | chr16 | 71319679 | 71319679 | Missense_Mutation | SNP | G | G | C | novel | Somatic | P49A | 0.047297 | NM_018348.6 | 591 | 563 | 28 |
| 973-PTsup | TP53 | 642 | hg19 | chr17 | 7577570 | 7577570 | Missense_Mutation | SNP | C | C | T | novel | Somatic | M237I | 0.146218 | NM_000546.5 | 595 | 508 | 87 |
| 973-PTsup | MYH2 | 664 | hg19 | chr17 | 10438667 | 10438667 | Missense_Mutation | SNP | G | G | T | novel | Somatic | L664M | 0.079186 | NM_017534.6 | 442 | 407 | 35 |
| 973-PTsup | NCOR1 | 707 | hg19 | chr17 | 15984004 | 15984004 | Missense_Mutation | SNP | G | G | C | novel | Somatic | A1072G | 0.079232 | NM_006311.4 | 833 | 767 | 66 |
| 973-PTsup | SLC47A2 | 734 | hg19 | chr17 | 19617300 | 19617300 | Intron | SNP | C | C | T | novel | Somatic |  | 0.075071 | NM_152908.4 | 706 | 653 | 53 |
| 973-PTsup | NAGLU | 895 | hg19 | chr17 | 40692991 | 40692991 | Missense_Mutation | SNP | C | C | T | novel | Somatic | T263M | 0.069697 | NM_000263.4 | 660 | 614 | 46 |
| 973-PTsup | BRCA1 | 899 | hg19 | chr17 | 41243479 | 41243479 | Nonsense_Mutation | SNP | C | C | A | novel | Somatic | E1357Ter | 0.124022 | NM_007294.4 | 894 | 783 | 111 |
| 973-PTsup | AKAP1 | 125 | hg19 | chr17 | 55182908 | 55182908 | Missense_Mutation | SNP | G | G | A | novel | Somatic | R28H | 0.093537 | NM_001370427.1 | 588 | 533 | 55 |
| 973-PTsup | MBD2 | 979 | hg19 | chr18 | 51750459 | 51750459 | Frame_Shift_Del | DEL | G | G | - | novel | Somatic | G158DfsTer7 | 0.021898 | NM_003927.5 | 822 | 804 | 18 |
| 973-PTsup | FAM129C | 719 | hg19 | chr19 | 17644432 | 17644432 | Missense_Mutation | SNP | G | G | T | novel | Somatic | A148S | 0.114849 | NM_173544.4 | 862 | 763 | 99 |
| 973-PTsup | LRFN3 | 862 | hg19 | chr19 | 36431237 | 36431237 | Missense_Mutation | SNP | C | C | T | novel | Somatic | P304S | 0.144381 | NM_024509.2 | 1219 | 1043 | 176 |
| 973-PTsup | ZNF607 | 876 | hg19 | chr19 | 38202516 | 38202516 | Missense_Mutation | SNP | A | A | C | novel | Somatic | S2A | 0.550218 | NM_032689.5 | 252 | 0 | 252 |
| 973-PTsup | LIPE | 912 | hg19 | chr19 | 42906191 | 42906191 | Missense_Mutation | SNP | T | T | G | novel | Somatic | M1002L | 0.133724 | NM_005357.4 | 2048 | 1774 | 274 |
| 973-PTsup | ZNF296 | 932 | hg19 | chr19 | 45579381 | 45579381 | Missense_Mutation | SNP | G | G | C | novel | Somatic | P84R | 0.117794 | NM_145288.3 | 1995 | 1760 | 235 |
| 973-PTsup | PPP1R15A | 961 | hg19 | chr19 | 49379145 | 49379145 | Missense_Mutation | SNP | C | C | T | novel | Somatic | T647M | 0.139865 | NM_014330.3 | 1336 | 1149 | 187 |
| 973-PTsup | ZNF845 | 995 | hg19 | chr19 | 53854993 | 53854993 | Nonsense_Mutation | SNP | C | C | G | novel | Somatic | Y355Ter | 0.089567 | NM_138374.3 | 1016 | 925 | 91 |
| 973-PTsup | TNNI3 | 1009 | hg19 | chr19 | 55667987 | 55667987 | Missense_Mutation | SNP | C | C | G | novel | Somatic | R45T | 0.033283 | NM_000363.5 | 1321 | 1277 | 44 |
| 973-PTsup | CD93 | 760 | hg19 | chr20 | 23065715 | 23065715 | Missense_Mutation | SNP | C | C | T | novel | Somatic | G372D | 0.147475 | NM_012072.4 | 990 | 844 | 146 |
| 973-PTsup | MICALL1 | 877 | hg19 | chr22 | 38321978 | 38321978 | Missense_Mutation | SNP | G | G | A | novel | Somatic | G465S | 0.156125 | NM_033386.4 | 1249 | 1054 | 195 |
| 973-PTsup | SHROOM4 | 121 | hg19 | chrX | 50377243 | 50377243 | Missense_Mutation | SNP | C | C | A | novel | Somatic | L610F | 0.137931 | NM_020717.3 | 348 | 300 | 48 |
| 973-PTsup | RBMXL3 | 182 | hg19 | chrX | 114426588 | 114426588 | Missense_Mutation | SNP | G | G | A | novel | Somatic | E862K | 0.286567 | NM_001145346.1 | 670 | 478 | 192 |
| 973-PTsup | SPANXN2 | 1674 | hg19 | chrX | 142803692 | 142803692 | Frame_Shift_Del | DEL | T | T | - | novel | Somatic | N24MfsTer12 | 0.230627 | NM_001009615.2 | 539 | 414 | 125 |
| 973-PTdeep | KLHDC7A | 728 | hg19 | chr1 | 18809264 | 18809264 | Missense_Mutation | SNP | G | G | A | novel | Somatic | E597K | 0.281818 | NM_152375.2 | 880 | 632 | 248 |
| 973-PTdeep | ZMPSTE24 | 895 | hg19 | chr1 | 40756542 | 40756542 | Frame_Shift_Ins | INS | - | - | T | novel | Somatic | L362FfsTer19 | 0.032178 | NM_005857.5 | 403 | 390 | 13 |
| 973-PTdeep | PRMT6 | 1405 | hg19 | chr1 | 107599928 | 107599928 | Missense_Mutation | SNP | C | C | A | novel | Somatic | S197R | 0.216245 | NM_018137.3 | 946 | 741 | 205 |
| 973-PTdeep | SPTA1 | 224 | hg19 | chr1 | 158614072 | 158614072 | Missense_Mutation | SNP | C | C | A | novel | Somatic | D1437Y | 0.250774 | NM_003126.4 | 646 | 484 | 162 |
| 973-PTdeep | MIA3 | 285 | hg19 | chr1 | 222827625 | 222827625 | Missense_Mutation | SNP | G | G | T | novel | Somatic | M1454I | 0.260365 | NM_198551.4 | 602 | 445 | 157 |
| 973-PTdeep | PGBD5 | 292 | hg19 | chr1 | 230473003 | 230473003 | Frame_Shift_Del | DEL | G | G | - | novel | Somatic | P309QfsTer7 | 0.153355 | NM_001258311.2 | 939 | 795 | 144 |
| 973-PTdeep | PGBD5 | 292 | hg19 | chr1 | 230473006 | 230473006 | Missense_Mutation | SNP | C | C | T | novel | Somatic | G308D | 0.161184 | NM_001258311.2 | 912 | 765 | 147 |
| 973-PTdeep | OR2L3 | 2478 | hg19 | chr1 | 248224394 | 248224394 | Missense_Mutation | SNP | A | A | C | novel | Somatic | K137N | 0.146893 | NM_001004687.1 | 885 | 755 | 130 |
| 973-PTdeep | MATN3 | 739 | hg19 | chr2 | 20212266 | 20212266 | Missense_Mutation | SNP | C | C | G | novel | Somatic | G43R | 0.040062 | NM_002381.5 | 649 | 623 | 26 |
| 973-PTdeep | SOS1 | 110 | hg19 | chr2 | 39237780 | 39237780 | Missense_Mutation | SNP | G | G | A | novel | Somatic | P819S | 0.177446 | NM_005633.3 | 602 | 495 | 107 |
| 973-PTdeep | CCDC88A | 15 | hg19 | chr2 | 55549680 | 55549680 | Missense_Mutation | SNP | A | A | G | novel | Somatic | I1049T | 0.219027 | NM_001365480.1 | 452 | 353 | 99 |
| 973-PTdeep | PTCD3 | 1243 | hg19 | chr2 | 86355084 | 86355084 | Missense_Mutation | SNP | C | C | A | novel | Somatic | H373N | 0.038889 | NM_017952.6 | 719 | 691 | 28 |
| 973-PTdeep | ZNF514 | 164 | hg19 | chr2 | 95818461 | 95818461 | Missense_Mutation | SNP | G | G | C | novel | Somatic | P59A | 0.199413 | NM_032788.3 | 680 | 544 | 136 |
| 973-PTdeep | MERTK | 180 | hg19 | chr2 | 112732857 | 112732857 | Intron | SNP | T | T | G | novel | Somatic |  | 0.217742 | NM_006343.3 | 372 | 291 | 81 |
| 973-PTdeep | EPB41L5 | 188 | hg19 | chr2 | 120834593 | 120834593 | Silent | SNP | C | C | T | novel | Somatic | V184= | 0.19338 | NM_020909.4 | 573 | 462 | 111 |
| 973-PTdeep | SCN1A | 232 | hg19 | chr2 | 166904240 | 166904240 | Missense_Mutation | SNP | C | C | T | novel | Somatic | R356K | 0.171492 | NM_006920.6 | 448 | 371 | 77 |
| 973-PTdeep | LRP2 | 235 | hg19 | chr2 | 169997024 | 169997024 | Frame_Shift_Ins | INS | - | - | G | novel | Somatic | C4381MfsTer12 | 0.022567 | NM_004525.3 | 707 | 691 | 16 |
| 973-PTdeep | FSIP2 | 31 | hg19 | chr2 | 186655800 | 186655800 | Missense_Mutation | SNP | C | C | T | novel | Somatic | H1313Y | 0.179221 | NM_173651.4 | 385 | 316 | 69 |
| 973-PTdeep | SMARCAL1 | 280 | hg19 | chr2 | 217280151 | 217280151 | Missense_Mutation | SNP | G | G | A | novel | Somatic | V242I | 0.022587 | NM_014140.4 | 487 | 476 | 11 |
| 973-PTdeep | COL6A6 | 197 | hg19 | chr3 | 130361846 | 130361846 | Nonsense_Mutation | SNP | C | C | T | novel | Somatic | Q1736Ter | 0.068376 | NM_001102608.2 | 583 | 543 | 40 |
| 973-PTdeep | CPNE4 | 198 | hg19 | chr3 | 131442466 | 131442466 | Missense_Mutation | SNP | C | C | G | novel | Somatic | D80H | 0.055556 | NM_001289112.1 | 503 | 475 | 28 |
| 973-PTdeep | HMX1 | 652 | hg19 | chr4 | 8873040 | 8873040 | Missense_Mutation | SNP | C | C | T | novel | Somatic | G101S | 0.327586 | NM_018942.3 | 232 | 156 | 76 |
| 973-PTdeep | GABRA2 | 117 | hg19 | chr4 | 46314555 | 46314555 | Missense_Mutation | SNP | A | A | G | novel | Somatic | L145S | 0.1491 | NM_000807.4 | 389 | 331 | 58 |
| 973-PTdeep | NFXL1 | 950 | hg19 | chr4 | 47898638 | 47898638 | Missense_Mutation | SNP | C | C | G | novel | Somatic | V411L | 0.199063 | NM_001278624.2 | 427 | 342 | 85 |
| 973-PTdeep | FRYL | 119 | hg19 | chr4 | 48611768 | 48611768 | Missense_Mutation | SNP | T | T | C | novel | Somatic | K162E | 0.060215 | NM_015030.2 | 465 | 437 | 28 |
| 973-PTdeep | UBA6 | 138 | hg19 | chr4 | 68506947 | 68506947 | Splice_Site | SNP | C | C | G | novel | Somatic |  | 0.189125 | NM_018227.6 | 423 | 343 | 80 |
| 973-PTdeep | FAT1 | 251 | hg19 | chr4 | 187541121 | 187541121 | Missense_Mutation | SNP | T | T | C | novel | Somatic | N2207D | 0.095318 | NM_005245.4 | 598 | 541 | 57 |
| 973-PTdeep | THBS4 | 1190 | hg19 | chr5 | 79335969 | 79335969 | Missense_Mutation | SNP | C | C | T | novel | Somatic | P53L | 0.24406 | NM_003248.6 | 463 | 350 | 113 |
| 973-PTdeep | CEP120 | 23 | hg19 | chr5 | 122726977 | 122726977 | Missense_Mutation | SNP | C | C | T | novel | Somatic | G289R | 0.268627 | NM_153223.3 | 510 | 373 | 137 |
| 973-PTdeep | ITPR3 | 841 | hg19 | chr6 | 33633710 | 33633710 | Missense_Mutation | SNP | G | G | A | novel | Somatic | R503Q | 0.096774 | NM_002224.4 | 837 | 756 | 81 |
| 973-PTdeep | ETV7 | 862 | hg19 | chr6 | 36334677 | 36334677 | In_Frame_Ins | INS | - | - | CTT | novel | Somatic | K294dup | 0.226506 | NM_016135.4 | 411 | 317 | 94 |
| 973-PTdeep | SMPD2 | 1422 | hg19 | chr6 | 109763188 | 109763188 | Missense_Mutation | SNP | G | G | C | novel | Somatic | G79A | 0.242206 | NM_003080.3 | 416 | 315 | 101 |
| 973-PTdeep | STXBP5 | 213 | hg19 | chr6 | 147705853 | 147705853 | Frame_Shift_Del | DEL | ATGTTGAAATACAAAGATA | ATGTTGAAATACAAAGATA | - | novel | Somatic | M1140RfsTer30 | 0.04007 | NM_001127715.2 | 574 | 551 | 23 |
| 973-PTdeep | NPSR1 | 106 | hg19 | chr7 | 34818157 | 34818157 | Nonsense_Mutation | SNP | C | C | T | novel | Somatic | R122Ter | 0.216495 | NM_001300935.1 | 484 | 379 | 105 |
| 973-PTdeep | SFRP4 | 874 | hg19 | chr7 | 37955737 | 37955737 | Missense_Mutation | SNP | C | C | T | novel | Somatic | V135M | 0.219081 | NM_003014.4 | 1132 | 884 | 248 |
| 973-PTdeep | ADCY1 | 933 | hg19 | chr7 | 45743283 | 45743283 | Missense_Mutation | SNP | G | G | A | novel | Somatic | G886S | 0.208967 | NM_021116.4 | 1248 | 987 | 261 |
| 973-PTdeep | LINC00998 | Unknown | hg19 | chr7 | 112757567 | 112757567 | Missense_Mutation | SNP | C | C | T | novel | Somatic | E23K | 0.135103 | NM_001352687.1 | 828 | 716 | 112 |
| 973-PTdeep | CPED1 | 188 | hg19 | chr7 | 120935656 | 120935656 | Missense_Mutation | SNP | T | T | C | novel | Somatic | C1011R | 0.055468 | NM_024913.5 | 631 | 596 | 35 |
| 973-PTdeep | CNTNAP2 | 26 | hg19 | chr7 | 147844694 | 147844694 | Missense_Mutation | SNP | G | G | C | novel | Somatic | R889T | 0.185325 | NM_014141.6 | 1063 | 866 | 197 |
| 973-PTdeep | ASIC3 | 1735 | hg19 | chr7 | 150747911 | 150747911 | Missense_Mutation | SNP | G | G | C | novel | Somatic | E294Q | 0.228886 | NM_004769.4 | 815 | 628 | 187 |
| 973-PTdeep | CSMD3 | 22 | hg19 | chr8 | 113349023 | 113349023 | Missense_Mutation | SNP | T | T | A | novel | Somatic | T2293S | 0.139269 | NM_198123.2 | 438 | 377 | 61 |
| 973-PTdeep | NAPRT | 1688 | hg19 | chr8 | 144657082 | 144657082 | Intron | SNP | A | A | T | novel | Somatic |  | 0.197624 | NM_145201.6 | 925 | 742 | 183 |
| 973-PTdeep | FPGS | 1581 | hg19 | chr9 | 130566669 | 130566669 | Missense_Mutation | SNP | T | T | A | novel | Somatic | Y82N | 0.270631 | NM_004957.6 | 824 | 601 | 223 |
| 973-PTdeep | MLLT10 | 751 | hg19 | chr10 | 21962768 | 21962768 | Missense_Mutation | SNP | C | C | G | novel | Somatic | S514C | 0.039882 | NM_001195626.3 | 677 | 650 | 27 |
| 973-PTdeep | CSGALNACT2 | 918 | hg19 | chr10 | 43662493 | 43662493 | Missense_Mutation | SNP | C | C | T | novel | Somatic | P401S | 0.070312 | NM_018590.5 | 640 | 595 | 45 |
| 973-PTdeep | UTF1 | 1615 | hg19 | chr10 | 135044608 | 135044608 | Missense_Mutation | SNP | G | G | A | novel | Somatic | A230T | 0.31694 | NM_003577.3 | 360 | 244 | 116 |
| 973-PTdeep | PANO1 | 591 | hg19 | chr11 | 798152 | 798152 | Missense_Mutation | SNP | C | C | T | novel | Somatic | S176L | 0.287561 | NM_001293167.1 | 619 | 441 | 178 |
| 973-PTdeep | OR51I1 | 626 | hg19 | chr11 | 5462325 | 5462325 | Missense_Mutation | SNP | G | G | C | novel | Somatic | H140Q | 0.036446 | NM_001005288.2 | 439 | 423 | 16 |
| 973-PTdeep | TRIM66 | 81 | hg19 | chr11 | 8642828 | 8642828 | Missense_Mutation | SNP | C | C | T | novel | Somatic | G923D | 0.251799 | NM_014818.1 | 695 | 520 | 175 |
| 973-PTdeep | PIK3C2A | 89 | hg19 | chr11 | 17172180 | 17172180 | Missense_Mutation | SNP | T | T | A | novel | Somatic | T398S | 0.239819 | NM_001321378.2 | 442 | 336 | 106 |
| 973-PTdeep | ARFGAP2 | 14 | hg19 | chr11 | 47198361 | 47198361 | Frame_Shift_Del | DEL | TTAAAAAGAG | TTAAAAAGAG | - | novel | Somatic | T12RfsTer46 | 0.165247 | NM_032389.6 | 587 | 490 | 97 |
| 973-PTdeep | OR8K5 | 1011 | hg19 | chr11 | 55927228 | 55927228 | Missense_Mutation | SNP | C | C | A | novel | Somatic | C189F | 0.193732 | NM_001004058.2 | 351 | 283 | 68 |
| 973-PTdeep | TESMIN | 1107 | hg19 | chr11 | 68512541 | 68512541 | Missense_Mutation | SNP | A | A | C | novel | Somatic | C223W | 0.418543 | NM_004923.3 | 755 | 439 | 316 |
| 973-PTdeep | FGF3 | 1116 | hg19 | chr11 | 69631141 | 69631141 | Missense_Mutation | SNP | G | G | C | novel | Somatic | L91V | 0.038309 | NM_005247.4 | 1513 | 1455 | 58 |
| 973-PTdeep | SHANK2 | 140 | hg19 | chr11 | 70666506 | 70666506 | Missense_Mutation | SNP | C | C | T | novel | Somatic | E437K | 0.182559 | NM_012309.4 | 2087 | 1706 | 381 |
| 973-PTdeep | NADSYN1 | 140 | hg19 | chr11 | 71208650 | 71208650 | Missense_Mutation | SNP | C | C | T | novel | Somatic | P629L | 0.232227 | NM_018161.5 | 633 | 486 | 147 |
| 973-PTdeep | MPZL3 | 1486 | hg19 | chr11 | 118110996 | 118110996 | Frame_Shift_Ins | INS | - | - | TTGAC | novel | Somatic | T57SfsTer12 | 0.181435 | NM_198275.3 | 469 | 383 | 86 |
| 973-PTdeep | PANX3 | 1534 | hg19 | chr11 | 124487217 | 124487217 | Silent | SNP | G | G | T | novel | Somatic | P124= | 0.24644 | NM_052959.3 | 913 | 688 | 225 |
| 973-PTdeep | CD9 | 633 | hg19 | chr12 | 6341822 | 6341822 | Missense_Mutation | SNP | G | G | A | novel | Somatic | G67D | 0.153584 | NM_001769.4 | 878 | 743 | 135 |
| 973-PTdeep | GSG1 | 85 | hg19 | chr12 | 13240952 | 13240952 | Missense_Mutation | SNP | C | C | T | novel | Somatic | G215R | 0.148183 | NM_001367363.2 | 1072 | 913 | 159 |
| 973-PTdeep | KIF21A | 110 | hg19 | chr12 | 39731002 | 39731002 | Missense_Mutation | SNP | G | G | A | novel | Somatic | R772C | 0.162933 | NM_001173464.2 | 490 | 410 | 80 |
| 973-PTdeep | MUC19 | 13 | hg19 | chr12 | 40925831 | 40925831 | Missense_Mutation | SNP | G | G | A | novel | Somatic | G7161D | 0.143106 | NM_173600.2 | 573 | 491 | 82 |
| 973-PTdeep | SCN8A | 122 | hg19 | chr12 | 52159705 | 52159705 | Missense_Mutation | SNP | T | T | C | novel | Somatic | V932A | 0.164276 | NM_014191.4 | 767 | 641 | 126 |
| 973-PTdeep | AGAP2 | 1028 | hg19 | chr12 | 58126739 | 58126739 | Missense_Mutation | SNP | G | G | A | novel | Somatic | R525W | 0.206771 | NM_001122772.2 | 825 | 654 | 171 |
| 973-PTdeep | PTPRQ | 150 | hg19 | chr12 | 80943267 | 80943267 | Missense_Mutation | SNP | C | C | A | novel | Somatic | Q1195K | 0.210351 | NM_001145026.2 | 597 | 471 | 126 |
| 973-PTdeep | MGAT4C | 155 | hg19 | chr12 | 86377397 | 86377397 | Missense_Mutation | SNP | C | C | G | novel | Somatic | E105Q | 0.188602 | NM_001351282.2 | 736 | 597 | 139 |
| 973-PTdeep | UHRF1BP1L | 168 | hg19 | chr12 | 100451390 | 100451390 | Nonsense_Mutation | SNP | A | A | C | novel | Somatic | L1128Ter | 0.056338 | NM_015054.2 | 426 | 402 | 24 |
| 973-PTdeep | BICDL1 | 187 | hg19 | chr12 | 120518739 | 120518739 | Nonsense_Mutation | SNP | C | C | T | novel | Somatic | R501Ter | 0.066802 | NM_001367886.1 | 494 | 461 | 33 |
| 973-PTdeep | RFC3 | 847 | hg19 | chr13 | 34398063 | 34398063 | Frame_Shift_Del | DEL | A | A | - | novel | Somatic | I82LfsTer25 | 0.052257 | NM_002915.4 | 421 | 399 | 22 |
| 973-PTdeep | NOVA1 | 98 | hg19 | chr14 | 26917847 | 26917847 | Missense_Mutation | SNP | G | G | T | novel | Somatic | T281N | 0.067766 | NM_002515.3 | 546 | 509 | 37 |
| 973-PTdeep | CCDC175 | 130 | hg19 | chr14 | 59998550 | 59998550 | Missense_Mutation | SNP | C | C | T | novel | Somatic | A568T | 0.18125 | NM_001164399.2 | 796 | 651 | 145 |
| 973-PTdeep | MPP5 | 137 | hg19 | chr14 | 67799632 | 67799632 | Missense_Mutation | SNP | G | G | C | novel | Somatic | D663H | 0.191358 | NM_022474.4 | 486 | 393 | 93 |
| 973-PTdeep | FLVCR2 | 1165 | hg19 | chr14 | 76107558 | 76107558 | Nonsense_Mutation | SNP | C | C | T | novel | Somatic | Q459Ter | 0.175966 | NM_017791.3 | 465 | 383 | 82 |
| 973-PTdeep | POMT2 | 1178 | hg19 | chr14 | 77778317 | 77778317 | Missense_Mutation | SNP | A | A | C | novel | Somatic | F103C | 0.191199 | NM_013382.5 | 659 | 533 | 126 |
| 973-PTdeep | SLC24A4 | 161 | hg19 | chr14 | 92958069 | 92958069 | Missense_Mutation | SNP | G | G | A | novel | Somatic | G533E | 0.260317 | NM_153646.3 | 315 | 233 | 82 |
| 973-PTdeep | CDC42BPB | 171 | hg19 | chr14 | 103470339 | 103470339 | Missense_Mutation | SNP | G | G | A | novel | Somatic | R125C | 0.262418 | NM_006035.4 | 1064 | 784 | 280 |
| 973-PTdeep | DPH6 | 858 | hg19 | chr15 | 35743001 | 35743001 | Nonsense_Mutation | SNP | A | A | T | novel | Somatic | C130Ter | 0.188034 | NM_080650.4 | 351 | 285 | 66 |
| 973-PTdeep | ZNF106 | 113 | hg19 | chr15 | 42744109 | 42744109 | Missense_Mutation | SNP | C | C | T | novel | Somatic | D121N | 0.21327 | NM_001366845.2 | 422 | 332 | 90 |
| 973-PTdeep | ODF3L1 | 1164 | hg19 | chr15 | 76019693 | 76019693 | Missense_Mutation | SNP | C | C | G | novel | Somatic | Q213E | 0.204748 | NM_175881.5 | 1348 | 1072 | 276 |
| 973-PTdeep | SNRPA1 | 1361 | hg19 | chr15 | 101827865 | 101827865 | Missense_Mutation | SNP | G | G | C | novel | Somatic | L118V | 0.18231 | NM_003090.4 | 554 | 453 | 101 |
| 973-PTdeep | GSPT1 | 676 | hg19 | chr16 | 11969717 | 11969717 | Missense_Mutation | SNP | C | C | G | novel | Somatic | D588H | 0.255285 | NM_002094.4 | 615 | 458 | 157 |
| 973-PTdeep | CMTR2 | 1129 | hg19 | chr16 | 71319679 | 71319679 | Missense_Mutation | SNP | G | G | C | novel | Somatic | P49A | 0.057395 | NM_018348.6 | 451 | 425 | 26 |
| 973-PTdeep | SPG7 | 1268 | hg19 | chr16 | 89574941 | 89574941 | Missense_Mutation | SNP | G | G | A | novel | Somatic | R39K | 0.06815 | NM_003119.4 | 719 | 670 | 49 |
| 973-PTdeep | TP53 | 642 | hg19 | chr17 | 7577570 | 7577570 | Missense_Mutation | SNP | C | C | T | novel | Somatic | M237I | 0.250467 | NM_000546.5 | 534 | 400 | 134 |
| 973-PTdeep | MYH2 | 664 | hg19 | chr17 | 10438667 | 10438667 | Missense_Mutation | SNP | G | G | T | novel | Somatic | L664M | 0.237705 | NM_017534.6 | 244 | 186 | 58 |
| 973-PTdeep | NCOR1 | 707 | hg19 | chr17 | 15984004 | 15984004 | Missense_Mutation | SNP | G | G | C | novel | Somatic | A1072G | 0.117754 | NM_006311.4 | 552 | 487 | 65 |
| 973-PTdeep | SLC47A2 | 734 | hg19 | chr17 | 19617300 | 19617300 | Intron | SNP | C | C | T | novel | Somatic |  | 0.121839 | NM_152908.4 | 435 | 382 | 53 |
| 973-PTdeep | NAGLU | 895 | hg19 | chr17 | 40692991 | 40692991 | Missense_Mutation | SNP | C | C | T | novel | Somatic | T263M | 0.15619 | NM_000263.4 | 524 | 442 | 82 |
| 973-PTdeep | BRCA1 | 899 | hg19 | chr17 | 41243479 | 41243479 | Nonsense_Mutation | SNP | C | C | A | novel | Somatic | E1357Ter | 0.190259 | NM_007294.4 | 657 | 532 | 125 |
| 973-PTdeep | AKAP1 | 125 | hg19 | chr17 | 55182908 | 55182908 | Missense_Mutation | SNP | G | G | A | novel | Somatic | R28H | 0.194656 | NM_001370427.1 | 784 | 631 | 153 |
| 973-PTdeep | MPND | 618 | hg19 | chr19 | 4352936 | 4352936 | In_Frame_Del | DEL | GAG | GAG | - | novel | Somatic | E195del | 0.022398 | NM_001300862.1 | 759 | 742 | 17 |
| 973-PTdeep | TNFSF9 | 634 | hg19 | chr19 | 6534723 | 6534723 | Missense_Mutation | SNP | G | G | T | novel | Somatic | K137N | 0.063025 | NM_003811.4 | 951 | 891 | 60 |
| 973-PTdeep | FAM129C | 719 | hg19 | chr19 | 17644432 | 17644432 | Missense_Mutation | SNP | G | G | T | novel | Somatic | A148S | 0.202532 | NM_173544.4 | 552 | 440 | 112 |
| 973-PTdeep | LRFN3 | 862 | hg19 | chr19 | 36431237 | 36431237 | Missense_Mutation | SNP | C | C | T | novel | Somatic | P304S | 0.181208 | NM_024509.2 | 894 | 732 | 162 |
| 973-PTdeep | ZNF607 | 876 | hg19 | chr19 | 38202516 | 38202516 | Missense_Mutation | SNP | A | A | C | novel | Somatic | S2A | 0.656338 | NM_032689.5 | 233 | 0 | 233 |
| 973-PTdeep | LIPE | 912 | hg19 | chr19 | 42906191 | 42906191 | Missense_Mutation | SNP | T | T | G | novel | Somatic | M1002L | 0.171691 | NM_005357.4 | 1526 | 1264 | 262 |
| 973-PTdeep | ZNF296 | 932 | hg19 | chr19 | 45579381 | 45579381 | Missense_Mutation | SNP | G | G | C | novel | Somatic | P84R | 0.178481 | NM_145288.3 | 1578 | 1296 | 282 |
| 973-PTdeep | PPP1R15A | 961 | hg19 | chr19 | 49379145 | 49379145 | Missense_Mutation | SNP | C | C | T | novel | Somatic | T647M | 0.247121 | NM_014330.3 | 1129 | 850 | 279 |
| 973-PTdeep | ZNF845 | 995 | hg19 | chr19 | 53854993 | 53854993 | Nonsense_Mutation | SNP | C | C | G | novel | Somatic | Y355Ter | 0.199324 | NM_138374.3 | 592 | 474 | 118 |
| 973-PTdeep | ZNF133 | 724 | hg19 | chr20 | 18296910 | 18296910 | Missense_Mutation | SNP | G | G | T | novel | Somatic | G489V | 0.026369 | NM_001282996.3 | 986 | 960 | 26 |
| 973-PTdeep | CD93 | 760 | hg19 | chr20 | 23065715 | 23065715 | Missense_Mutation | SNP | C | C | T | novel | Somatic | G372D | 0.242967 | NM_012072.4 | 782 | 592 | 190 |
| 973-PTdeep | MICALL1 | 877 | hg19 | chr22 | 38321978 | 38321978 | Missense_Mutation | SNP | G | G | A | novel | Somatic | G465S | 0.264673 | NM_033386.4 | 902 | 663 | 239 |
| 973-PTdeep | SHROOM4 | 121 | hg19 | chrX | 50377243 | 50377243 | Missense_Mutation | SNP | C | C | A | novel | Somatic | L610F | 0.234657 | NM_020717.3 | 277 | 212 | 65 |
| 973-PTdeep | RBMXL3 | 182 | hg19 | chrX | 114426588 | 114426588 | Missense_Mutation | SNP | G | G | A | novel | Somatic | E862K | 0.409336 | NM_001145346.1 | 557 | 329 | 228 |
| 973-PTdeep | SPANXN2 | 1674 | hg19 | chrX | 142803692 | 142803692 | Frame_Shift_Del | DEL | T | T | - | novel | Somatic | N24MfsTer12 | 0.309677 | NM_001009615.2 | 308 | 212 | 96 |
| 973-LNmet | PADI2 | 89 | hg19 | chr1 | 17413091 | 17413091 | Frame_Shift_Del | DEL | T | T | - | novel | Somatic | G254AfsTer29 | 0.036779 | NM_007365.3 | 1004 | 967 | 37 |
| 973-LNmet | KLHDC7A | 728 | hg19 | chr1 | 18809264 | 18809264 | Missense_Mutation | SNP | G | G | A | novel | Somatic | E597K | 0.310345 | NM_152375.2 | 841 | 580 | 261 |
| 973-LNmet | CSMD2 | 13 | hg19 | chr1 | 34190230 | 34190230 | Missense_Mutation | SNP | T | T | C | novel | Somatic | Y924C | 0.032692 | NM_001281956.1 | 1040 | 1006 | 34 |
| 973-LNmet | FPGT-TNNI3K | 144 | hg19 | chr1 | 74957824 | 74957824 | In_Frame_Del | DEL | CTT | CTT | - | novel | Somatic | S860del | 0.024752 | NM_001112808.2 | 808 | 788 | 20 |
| 973-LNmet | PRMT6 | 1405 | hg19 | chr1 | 107599928 | 107599928 | Missense_Mutation | SNP | C | C | A | novel | Somatic | S197R | 0.197336 | NM_018137.3 | 1201 | 964 | 237 |
| 973-LNmet | SYCP1 | 183 | hg19 | chr1 | 115469133 | 115469133 | Frame_Shift_Ins | INS | - | - | A | novel | Somatic | G629RfsTer12 | 0.052897 | NM_003176.4 | 394 | 373 | 21 |
| 973-LNmet | SPTA1 | 224 | hg19 | chr1 | 158614072 | 158614072 | Missense_Mutation | SNP | C | C | A | novel | Somatic | D1437Y | 0.182553 | NM_003126.4 | 616 | 503 | 113 |
| 973-LNmet | MIA3 | 285 | hg19 | chr1 | 222827625 | 222827625 | Missense_Mutation | SNP | G | G | T | novel | Somatic | M1454I | 0.114332 | NM_198551.4 | 621 | 550 | 71 |
| 973-LNmet | PGBD5 | 292 | hg19 | chr1 | 230473003 | 230473003 | Frame_Shift_Del | DEL | G | G | - | novel | Somatic | P309QfsTer7 | 0.189605 | NM_001258311.2 | 1037 | 840 | 197 |
| 973-LNmet | PGBD5 | 292 | hg19 | chr1 | 230473006 | 230473006 | Missense_Mutation | SNP | C | C | T | novel | Somatic | G308D | 0.2002 | NM_001258311.2 | 998 | 798 | 200 |
| 973-LNmet | OR2L3 | 2478 | hg19 | chr1 | 248224394 | 248224394 | Missense_Mutation | SNP | A | A | C | novel | Somatic | K137N | 0.169944 | NM_001004687.1 | 712 | 591 | 121 |
| 973-LNmet | SOS1 | 110 | hg19 | chr2 | 39237780 | 39237780 | Missense_Mutation | SNP | G | G | A | novel | Somatic | P819S | 0.208835 | NM_005633.3 | 497 | 393 | 104 |
| 973-LNmet | CCDC88A | 15 | hg19 | chr2 | 55549680 | 55549680 | Missense_Mutation | SNP | A | A | G | novel | Somatic | I1049T | 0.210243 | NM_001365480.1 | 371 | 293 | 78 |
| 973-LNmet | ZNF514 | 164 | hg19 | chr2 | 95818461 | 95818461 | Missense_Mutation | SNP | G | G | C | novel | Somatic | P59A | 0.248062 | NM_032788.3 | 902 | 678 | 224 |
| 973-LNmet | MERTK | 180 | hg19 | chr2 | 112732857 | 112732857 | Intron | SNP | T | T | G | novel | Somatic |  | 0.233503 | NM_006343.3 | 589 | 451 | 138 |
| 973-LNmet | EPB41L5 | 188 | hg19 | chr2 | 120834593 | 120834593 | Silent | SNP | C | C | T | novel | Somatic | V184= | 0.142268 | NM_020909.4 | 485 | 416 | 69 |
| 973-LNmet | SCN1A | 232 | hg19 | chr2 | 166904240 | 166904240 | Missense_Mutation | SNP | C | C | T | novel | Somatic | R356K | 0.158774 | NM_006920.6 | 359 | 302 | 57 |
| 973-LNmet | LRP2 | 235 | hg19 | chr2 | 169997024 | 169997024 | Frame_Shift_Ins | INS | - | - | G | novel | Somatic | C4381MfsTer12 | 0.022073 | NM_004525.3 | 1040 | 1017 | 23 |
| 973-LNmet | CLK1 | 265 | hg19 | chr2 | 201726143 | 201726143 | Nonsense_Mutation | SNP | G | G | A | novel | Somatic | R112Ter | 0.043689 | NM_001162407.1 | 412 | 394 | 18 |
| 973-LNmet | MORC1 | 176 | hg19 | chr3 | 108780851 | 108780851 | Missense_Mutation | SNP | G | G | C | novel | Somatic | S317C | 0.034539 | NM_014429.4 | 608 | 587 | 21 |
| 973-LNmet | HMX1 | 652 | hg19 | chr4 | 8873040 | 8873040 | Missense_Mutation | SNP | C | C | T | novel | Somatic | G101S | 0.276423 | NM_018942.3 | 369 | 267 | 102 |
| 973-LNmet | GABRA2 | 117 | hg19 | chr4 | 46314555 | 46314555 | Missense_Mutation | SNP | A | A | G | novel | Somatic | L145S | 0.173653 | NM_000807.4 | 334 | 276 | 58 |
| 973-LNmet | NFXL1 | 950 | hg19 | chr4 | 47898638 | 47898638 | Missense_Mutation | SNP | C | C | G | novel | Somatic | V411L | 0.201681 | NM_001278624.2 | 357 | 285 | 72 |
| 973-LNmet | ENAM | 1130 | hg19 | chr4 | 71497578 | 71497578 | Nonsense_Mutation | SNP | C | C | T | novel | Somatic | R46Ter | 0.020305 | NM_031889.3 | 591 | 579 | 12 |
| 973-LNmet | THBS4 | 1190 | hg19 | chr5 | 79335969 | 79335969 | Missense_Mutation | SNP | C | C | T | novel | Somatic | P53L | 0.237826 | NM_003248.6 | 883 | 673 | 210 |
| 973-LNmet | CEP120 | 23 | hg19 | chr5 | 122726977 | 122726977 | Missense_Mutation | SNP | C | C | T | novel | Somatic | G289R | 0.287755 | NM_153223.3 | 490 | 349 | 141 |
| 973-LNmet | ETV7 | 862 | hg19 | chr6 | 36334677 | 36334677 | In_Frame_Ins | INS | - | - | CTT | novel | Somatic | K294dup | 0.234266 | NM_016135.4 | 567 | 433 | 134 |
| 973-LNmet | SMPD2 | 1422 | hg19 | chr6 | 109763188 | 109763188 | Missense_Mutation | SNP | G | G | C | novel | Somatic | G79A | 0.289076 | NM_003080.3 | 592 | 420 | 172 |
| 973-LNmet | KIAA0408 | 1559 | hg19 | chr6 | 127768806 | 127768806 | Missense_Mutation | SNP | G | G | T | novel | Somatic | Q220K | 0.043814 | NM_014702.5 | 388 | 371 | 17 |
| 973-LNmet | NPSR1 | 106 | hg19 | chr7 | 34818157 | 34818157 | Nonsense_Mutation | SNP | C | C | T | novel | Somatic | R122Ter | 0.180577 | NM_001300935.1 | 659 | 540 | 119 |
| 973-LNmet | ADCY1 | 933 | hg19 | chr7 | 45743283 | 45743283 | Missense_Mutation | SNP | G | G | A | novel | Somatic | G886S | 0.193506 | NM_021116.4 | 1509 | 1217 | 292 |
| 973-LNmet | CNTNAP2 | 26 | hg19 | chr7 | 147844694 | 147844694 | Missense_Mutation | SNP | G | G | C | novel | Somatic | R889T | 0.178039 | NM_014141.6 | 1275 | 1048 | 227 |
| 973-LNmet | ADGRA2 | 872 | hg19 | chr8 | 37699038 | 37699038 | Missense_Mutation | SNP | T | T | C | novel | Somatic | L1061P | 0.142313 | NM_032777.10 | 784 | 672 | 112 |
| 973-LNmet | CNGB3 | 156 | hg19 | chr8 | 87588235 | 87588235 | In_Frame_Del | DEL | CTT | CTT | - | novel | Somatic | K742_G743delinsR | 0.02809 | NM_019098.4 | 533 | 518 | 15 |
| 973-LNmet | CSMD3 | 22 | hg19 | chr8 | 113349023 | 113349023 | Missense_Mutation | SNP | T | T | A | novel | Somatic | T2293S | 0.202198 | NM_198123.2 | 455 | 363 | 92 |
| 973-LNmet | NAPRT | 1688 | hg19 | chr8 | 144657082 | 144657082 | Intron | SNP | A | A | T | novel | Somatic |  | 0.200477 | NM_145201.6 | 1257 | 1005 | 252 |
| 973-LNmet | PTCH1 | 1334 | hg19 | chr9 | 98279101 | 98279101 | Translation_Start_Site | SNP | A | A | G | novel | Somatic | M1? | 0.036364 | NM_001083603.2 | 385 | 371 | 14 |
| 973-LNmet | RABEPK | 1561 | hg19 | chr9 | 127969897 | 127969897 | Missense_Mutation | SNP | T | T | G | novel | Somatic | C36W | 0.033654 | NM_005833.4 | 1040 | 1005 | 35 |
| 973-LNmet | FPGS | 1581 | hg19 | chr9 | 130566669 | 130566669 | Missense_Mutation | SNP | T | T | A | novel | Somatic | Y82N | 0.282876 | NM_004957.6 | 1057 | 758 | 299 |
| 973-LNmet | ANTXRL | 948 | hg19 | chr10 | 47701156 | 47701156 | Nonsense_Mutation | SNP | C | C | T | novel | Somatic | Q578Ter | 0.125448 | NM_001278688.2 | 1115 | 975 | 140 |
| 973-LNmet | KAZALD1 | 1369 | hg19 | chr10 | 102824171 | 102824171 | Missense_Mutation | SNP | C | C | G | novel | Somatic | S222C | 0.023342 | NM_030929.5 | 814 | 795 | 19 |
| 973-LNmet | UTF1 | 1615 | hg19 | chr10 | 135044608 | 135044608 | Missense_Mutation | SNP | G | G | A | novel | Somatic | A230T | 0.323596 | NM_003577.3 | 436 | 292 | 144 |
| 973-LNmet | PANO1 | 591 | hg19 | chr11 | 798152 | 798152 | Missense_Mutation | SNP | C | C | T | novel | Somatic | S176L | 0.26383 | NM_001293167.1 | 937 | 689 | 248 |
| 973-LNmet | OR52N5 | 629 | hg19 | chr11 | 5798967 | 5798967 | Missense_Mutation | SNP | T | T | A | novel | Somatic | I300F | 0.172867 | NM_001001922.2 | 457 | 378 | 79 |
| 973-LNmet | TRIM66 | 81 | hg19 | chr11 | 8642828 | 8642828 | Missense_Mutation | SNP | C | C | T | novel | Somatic | G923D | 0.305048 | NM_014818.1 | 930 | 646 | 284 |
| 973-LNmet | PIK3C2A | 89 | hg19 | chr11 | 17172180 | 17172180 | Missense_Mutation | SNP | T | T | A | novel | Somatic | T398S | 0.342733 | NM_001321378.2 | 461 | 303 | 158 |
| 973-LNmet | SLC35C1 | 934 | hg19 | chr11 | 45832453 | 45832453 | Missense_Mutation | SNP | C | C | T | novel | Somatic | P221L | 0.029552 | NM_018389.5 | 1049 | 1018 | 31 |
| 973-LNmet | ARFGAP2 | 14 | hg19 | chr11 | 47198361 | 47198361 | Frame_Shift_Del | DEL | TTAAAAAGAG | TTAAAAAGAG | - | novel | Somatic | T12RfsTer46 | 0.184096 | NM_032389.6 | 917 | 748 | 169 |
| 973-LNmet | OR4C5 | 954 | hg19 | chr11 | 48387342 | 48387342 | Missense_Mutation | SNP | G | G | T | novel | Somatic | L226M | 0.088565 | NM_001348223.1 | 892 | 813 | 79 |
| 973-LNmet | TESMIN | 1107 | hg19 | chr11 | 68512541 | 68512541 | Missense_Mutation | SNP | A | A | C | novel | Somatic | C223W | 0.52982 | NM_004923.3 | 721 | 339 | 382 |
| 973-LNmet | SHANK2 | 140 | hg19 | chr11 | 70666506 | 70666506 | Missense_Mutation | SNP | C | C | T | novel | Somatic | E437K | 0.159277 | NM_012309.4 | 2321 | 1951 | 370 |
| 973-LNmet | NADSYN1 | 140 | hg19 | chr11 | 71208650 | 71208650 | Missense_Mutation | SNP | C | C | T | novel | Somatic | P629L | 0.284192 | NM_018161.5 | 1126 | 806 | 320 |
| 973-LNmet | ANAPC15 | 1132 | hg19 | chr11 | 71821193 | 71821193 | Missense_Mutation | SNP | G | G | C | novel | Somatic | Q87E | 0.053592 | NM_001330321.2 | 877 | 830 | 47 |
| 973-LNmet | MPZL3 | 1486 | hg19 | chr11 | 118110996 | 118110996 | Frame_Shift_Ins | INS | - | - | TTGAC | novel | Somatic | T57SfsTer12 | 0.187908 | NM_198275.3 | 609 | 494 | 115 |
| 973-LNmet | CD9 | 633 | hg19 | chr12 | 6341822 | 6341822 | Missense_Mutation | SNP | G | G | A | novel | Somatic | G67D | 0.065185 | NM_001769.4 | 1349 | 1261 | 88 |
| 973-LNmet | GSG1 | 85 | hg19 | chr12 | 13240952 | 13240952 | Missense_Mutation | SNP | C | C | T | novel | Somatic | G215R | 0.278592 | NM_001367363.2 | 1700 | 1225 | 475 |
| 973-LNmet | KIF21A | 110 | hg19 | chr12 | 39731002 | 39731002 | Missense_Mutation | SNP | G | G | A | novel | Somatic | R772C | 0.113333 | NM_001173464.2 | 450 | 399 | 51 |
| 973-LNmet | MUC19 | 13 | hg19 | chr12 | 40925831 | 40925831 | Missense_Mutation | SNP | G | G | A | novel | Somatic | G7161D | 0.282609 | NM_173600.2 | 550 | 394 | 156 |
| 973-LNmet | SCN8A | 122 | hg19 | chr12 | 52159705 | 52159705 | Missense_Mutation | SNP | T | T | C | novel | Somatic | V932A | 0.122308 | NM_014191.4 | 1300 | 1141 | 159 |
| 973-LNmet | PTPRQ | 150 | hg19 | chr12 | 80943267 | 80943267 | Missense_Mutation | SNP | C | C | A | novel | Somatic | Q1195K | 0.112713 | NM_001145026.2 | 763 | 677 | 86 |
| 973-LNmet | UHRF1BP1L | 168 | hg19 | chr12 | 100451390 | 100451390 | Nonsense_Mutation | SNP | A | A | C | novel | Somatic | L1128Ter | 0.091566 | NM_015054.2 | 415 | 377 | 38 |
| 973-LNmet | ALDH2 | 1441 | hg19 | chr12 | 112204832 | 112204832 | Missense_Mutation | SNP | C | C | T | novel | Somatic | L16F | 0.031705 | NM_000690.4 | 1167 | 1130 | 37 |
| 973-LNmet | BICDL1 | 187 | hg19 | chr12 | 120518739 | 120518739 | Nonsense_Mutation | SNP | C | C | T | novel | Somatic | R501Ter | 0.101399 | NM_001367886.1 | 856 | 769 | 87 |
| 973-LNmet | CCDC175 | 130 | hg19 | chr14 | 59998550 | 59998550 | Missense_Mutation | SNP | C | C | T | novel | Somatic | A568T | 0.155067 | NM_001164399.2 | 819 | 692 | 127 |
| 973-LNmet | MPP5 | 137 | hg19 | chr14 | 67799632 | 67799632 | Missense_Mutation | SNP | G | G | C | novel | Somatic | D663H | 0.214612 | NM_022474.4 | 438 | 344 | 94 |
| 973-LNmet | FLVCR2 | 1165 | hg19 | chr14 | 76107558 | 76107558 | Nonsense_Mutation | SNP | C | C | T | novel | Somatic | Q459Ter | 0.188103 | NM_017791.3 | 622 | 505 | 117 |
| 973-LNmet | POMT2 | 1178 | hg19 | chr14 | 77778317 | 77778317 | Missense_Mutation | SNP | A | A | C | novel | Somatic | F103C | 0.22476 | NM_013382.5 | 832 | 645 | 187 |
| 973-LNmet | SLC24A4 | 161 | hg19 | chr14 | 92958069 | 92958069 | Missense_Mutation | SNP | G | G | A | novel | Somatic | G533E | 0.251346 | NM_153646.3 | 556 | 416 | 140 |
| 973-LNmet | CDC42BPB | 171 | hg19 | chr14 | 103470339 | 103470339 | Missense_Mutation | SNP | G | G | A | novel | Somatic | R125C | 0.212073 | NM_006035.4 | 1259 | 992 | 267 |
| 973-LNmet | SPTBN5 | 906 | hg19 | chr15 | 42149632 | 42149632 | Missense_Mutation | SNP | C | C | G | novel | Somatic | E2809Q | 0.022005 | NM_016642.4 | 818 | 800 | 18 |
| 973-LNmet | ZNF106 | 113 | hg19 | chr15 | 42744109 | 42744109 | Missense_Mutation | SNP | C | C | T | novel | Somatic | D121N | 0.187146 | NM_001366845.2 | 529 | 430 | 99 |
| 973-LNmet | SNRPA1 | 1361 | hg19 | chr15 | 101827865 | 101827865 | Missense_Mutation | SNP | G | G | C | novel | Somatic | L118V | 0.241379 | NM_003090.4 | 549 | 416 | 133 |
| 973-LNmet | SCARF1 | 596 | hg19 | chr17 | 1542127 | 1542127 | In_Frame_Del | DEL | CAG | CAG | - | novel | Somatic | C445del | 0.023641 | NM_003693.4 | 846 | 826 | 20 |
| 973-LNmet | ITGAE | 76 | hg19 | chr17 | 3651273 | 3651273 | Missense_Mutation | SNP | C | C | T | novel | Somatic | V700I | 0.129765 | NM_002208.5 | 1232 | 1072 | 160 |
| 973-LNmet | CAMTA2 | 622 | hg19 | chr17 | 4875738 | 4875738 | Frame_Shift_Del | DEL | G | G | - | novel | Somatic | P866LfsTer9 | 0.020819 | NM_015099.4 | 1487 | 1456 | 31 |
| 973-LNmet | TP53 | 642 | hg19 | chr17 | 7577570 | 7577570 | Missense_Mutation | SNP | C | C | T | novel | Somatic | M237I | 0.315186 | NM_000546.5 | 698 | 478 | 220 |
| 973-LNmet | MYH2 | 664 | hg19 | chr17 | 10438667 | 10438667 | Missense_Mutation | SNP | G | G | T | novel | Somatic | L664M | 0.192771 | NM_017534.6 | 249 | 201 | 48 |
| 973-LNmet | NCOR1 | 707 | hg19 | chr17 | 15943760 | 15943760 | Missense_Mutation | SNP | G | G | T | novel | Somatic | T2243K | 0.03562 | NM_006311.4 | 758 | 731 | 27 |
| 973-LNmet | NCOR1 | 707 | hg19 | chr17 | 15984004 | 15984004 | Missense_Mutation | SNP | G | G | C | novel | Somatic | A1072G | 0.145349 | NM_006311.4 | 687 | 587 | 100 |
| 973-LNmet | SLC47A2 | 734 | hg19 | chr17 | 19617300 | 19617300 | Intron | SNP | C | C | T | novel | Somatic |  | 0.26094 | NM_152908.4 | 617 | 456 | 161 |
| 973-LNmet | STAT5B | 111 | hg19 | chr17 | 40379630 | 40379630 | Missense_Mutation | SNP | G | G | T | novel | Somatic | L68M | 0.052097 | NM_012448.4 | 1476 | 1399 | 77 |
| 973-LNmet | NAGLU | 895 | hg19 | chr17 | 40692991 | 40692991 | Missense_Mutation | SNP | C | C | T | novel | Somatic | T263M | 0.195775 | NM_000263.4 | 710 | 571 | 139 |
| 973-LNmet | HDAC5 | 906 | hg19 | chr17 | 42170732 | 42170732 | Missense_Mutation | SNP | C | C | G | novel | Somatic | R160P | 0.103896 | NM_001015053.2 | 1463 | 1311 | 152 |
| 973-LNmet | AKAP1 | 125 | hg19 | chr17 | 55182908 | 55182908 | Missense_Mutation | SNP | G | G | A | novel | Somatic | R28H | 0.218557 | NM_001370427.1 | 969 | 757 | 212 |
| 973-LNmet | POLG2 | 1061 | hg19 | chr17 | 62474003 | 62474003 | Missense_Mutation | SNP | C | C | A | novel | Somatic | M465I | 0.042705 | NM_007215.4 | 562 | 538 | 24 |
| 973-LNmet | ZCCHC2 | 1044 | hg19 | chr18 | 60232254 | 60232254 | Missense_Mutation | SNP | G | G | C | novel | Somatic | D591H | 0.053398 | NM_017742.6 | 412 | 390 | 22 |
| 973-LNmet | ZNF844 | 677 | hg19 | chr19 | 12187775 | 12187775 | Missense_Mutation | SNP | A | A | G | novel | Somatic | M614V | 0.025948 | NM_001136501.3 | 501 | 488 | 13 |
| 973-LNmet | FAM129C | 719 | hg19 | chr19 | 17644432 | 17644432 | Missense_Mutation | SNP | G | G | T | novel | Somatic | A148S | 0.155191 | NM_173544.4 | 914 | 772 | 142 |
| 973-LNmet | LRFN3 | 862 | hg19 | chr19 | 36431237 | 36431237 | Missense_Mutation | SNP | C | C | T | novel | Somatic | P304S | 0.161998 | NM_024509.2 | 821 | 688 | 133 |
| 973-LNmet | ACTN4 | 110 | hg19 | chr19 | 39191689 | 39191689 | Missense_Mutation | SNP | A | A | T | novel | Somatic | I109F | 0.114936 | NM_004924.6 | 1801 | 1594 | 207 |
| 973-LNmet | ZNF296 | 932 | hg19 | chr19 | 45579381 | 45579381 | Missense_Mutation | SNP | G | G | C | novel | Somatic | P84R | 0.162659 | NM_145288.3 | 2118 | 1773 | 345 |
| 973-LNmet | PPP1R15A | 961 | hg19 | chr19 | 49379145 | 49379145 | Missense_Mutation | SNP | C | C | T | novel | Somatic | T647M | 0.244068 | NM_014330.3 | 1468 | 1108 | 360 |
| 973-LNmet | ZNF845 | 995 | hg19 | chr19 | 53854993 | 53854993 | Nonsense_Mutation | SNP | C | C | G | novel | Somatic | Y355Ter | 0.171026 | NM_138374.3 | 497 | 412 | 85 |
| 973-LNmet | JAG1 | 666 | hg19 | chr20 | 10621458 | 10621458 | Missense_Mutation | SNP | G | G | T | novel | Somatic | Q1058K | 0.214226 | NM_000214.3 | 1195 | 939 | 256 |
| 973-LNmet | FLRT3 | 694 | hg19 | chr20 | 14306365 | 14306365 | Missense_Mutation | SNP | C | C | A | novel | Somatic | M596I | 0.028139 | NM_198391.3 | 461 | 448 | 13 |
| 973-LNmet | CD93 | 760 | hg19 | chr20 | 23065715 | 23065715 | Missense_Mutation | SNP | C | C | T | novel | Somatic | G372D | 0.33214 | NM_012072.4 | 1117 | 746 | 371 |
| 973-LNmet | SNX21 | 924 | hg19 | chr20 | 44469737 | 44469737 | Missense_Mutation | SNP | C | C | T | novel | Somatic | R303W | 0.221584 | NM_033421.4 | 871 | 678 | 193 |
| 973-LNmet | APP | 12 | hg19 | chr21 | 27354743 | 27354743 | Missense_Mutation | SNP | C | C | G | novel | Somatic | E380Q | 0.023895 | NM_000484.4 | 835 | 815 | 20 |
| 973-LNmet | ADAMTS1 | 800 | hg19 | chr21 | 28214747 | 28214747 | Missense_Mutation | SNP | G | G | A | novel | Somatic | R330W | 0.058011 | NM_006988.5 | 1084 | 1021 | 63 |
| 973-LNmet | SLC37A1 | 920 | hg19 | chr21 | 43938602 | 43938602 | Missense_Mutation | SNP | C | C | T | novel | Somatic | S13L | 0.059395 | NM_001320537.2 | 1851 | 1741 | 110 |
| 973-LNmet | MICALL1 | 877 | hg19 | chr22 | 38321978 | 38321978 | Missense_Mutation | SNP | G | G | A | novel | Somatic | G465S | 0.300868 | NM_033386.4 | 1035 | 723 | 312 |
| 973-LNmet | RBMXL3 | 182 | hg19 | chrX | 114426588 | 114426588 | Missense_Mutation | SNP | G | G | A | novel | Somatic | E862K | 0.413793 | NM_001145346.1 | 522 | 306 | 216 |
| 973-LNmet | SPANXN2 | 1674 | hg19 | chrX | 142803692 | 142803692 | Frame_Shift_Del | DEL | T | T | - | novel | Somatic | N24MfsTer12 | 0.357333 | NM_001009615.2 | 368 | 234 | 134 |
| 973-LNmet | IDH3G | 1752 | hg19 | chrX | 153052323 | 153052323 | Missense_Mutation | SNP | C | C | T | novel | Somatic | G286E | 0.056584 | NM_004135.4 | 972 | 917 | 55 |
